# Supplementary material for: A system wide approach to managing zoo collections for visitor attendance and in situ conservation
Source: Nat Commun. 2020 Feb 4;11:584. doi: 10.1038/s41467-020-14303-2 (PMC7000708; doi:10.1038/s41467-020-14303-2)
Supplement: Supplementary file 1 — Supplementary Information [file 41467_2020_14303_MOESM1_ESM.pdf]

Supplementary Information

A system wide approach to managing zoo collections for visitor attendance and *in situ* conservation

Mooney *et al*.

Contents:

Supplementary Note 1

Supplementary Figures 1 – 5

Supplementary Tables 1 - 6

References

Supplementary Notes

Supplementary Note 1: Development of *a priori* meta-model

The *a priori* meta-model shown in Supplementary Figure 1 represents the combined prior theoretical knowledge and proposed causal relationships influencing visitor attendance and *in situ* contributions at zoological institutions globally. The development of a theoretical model based on prior knowledge is a key step in Structural Equation Modelling (SEM), as it guides model specification and modification[1]. This meta-model represents general relationships between multiple variables, while omitting statistical details[1]. We consulted the literature pertaining to visitor attendance and *in situ* contributions of zoological institutions globally in order to develop the main theoretical constructs involved and their interconnections. These evidence-based relationships are highlighted in Supplementary Figure 1 with appropriate citations and are further explained below.

Evidence-Based Relationships

Leader-Williams *et al.*[2] suggest that most zoos are located in and receive more visitors in richer countries, demonstrated by a positive correlation between number of zoos and number of visitors per million people and GDP. As a result, we link GDP and 'Country' to 'Institution Attendance' in our meta-model. Davey[3] investigated trends in visitor attendance for zoos in Australia, Germany, Japan, North America, New Zealand and the UK, and how they relate to socio-economic data. It is shown that a significant positive correlation exists between institutional attendance and national population size and between institutional attendance and gross national income[3]. This study failed to find any correlation between institutional

attendance and tertiary education. As a result, we link GDP and 'National Population Size' to 'Institution Attendance' in our meta-model.

Whitworth[4] conducted an investigation into the factors determining visitor attendance to UK zoos. It is shown that visitor numbers are positively correlated with the popularity of institutional collections (in terms of species kept)[4]. It is also shown that 'rare' species are more popular than 'common' species[4]. We deduce from this that 'rare' species are more popular within a collection and that increased collection popularity results in increased visitor attendance. Therefore, we link 'Threatened Species' to 'Institution Attendance' in our meta-model. This study also highlights the importance of institutional types in determining the composition of collections, for example it is shown that safari parks, although much larger than traditional zoos, contain fewer species relative to their size. Therefore, we link 'Institution Type' to 'Species Richness' in our meta-model. Although this study found that the popularity of collections (in terms of species kept) is more important in determining visitor attendance than demographic variables, it also shows that institutions are clustered around larger cities with higher populations or areas of high tourism. Therefore, we link 'Local Population Size' to 'Institution Attendance' in our meta-model.

Dickie *et al*.[5] provide insight into how institutions link charismatic and endangered species with *in situ* conservation activities. For instance, they highlight the example of the Fossa (*Cryptoprocta ferox*) in European zoos. The Fossa is the largest endemic carnivore on Madagascar, yet is also Vulnerable according to the IUCN. The Fossa Fund, operated by Zoo Duisburg has been instrumental in using captive Fossa populations to generate funds for *in situ* conservation and research. Any zoo wishing to acquire Fossa as part of the EAZA Fossa Endangered Species Programme (EEP) must also pay a "conservation surcharge" of

approximately £1,000 to the Fossa Fund. This ensures *ex situ* institutions are also financially committed to the conservation of the species *in situ*. Similarly, the Congo Gorilla Rainforest exhibit at the Bronx Zoo has been used to generate funding for the conservation of African forest wildlife *in situ*[5]. Upon opening this exhibit in 1999, the Bronx Zoo not only imposed a special admission fee to support wildlife conservation in tropical African forests, but also allowed visitors to choose how their fees could be spent *in situ*. By 2009, $10.6 million had been raised and expended on African forest wildlife conservation from this source alone[6]. As a result of these clear examples linking large, charismatic and threatened species to *in situ* conservation activities, we link 'Threatened Species' and 'Institution Body Mass' to '*In Situ* Project Investment' in our meta-model.

When looking at the interconnections between the various concepts, Fa *et al.*[7] proved invaluable. They show that larger zoos hold proportionately larger numbers of individual animals, and that a positive correlation exists between the number of individual animals and overall institutional species richness[7]. These correlations let us link 'Institution Area' and 'Institution Species Richness' to 'Number of Animals' in our meta-model. However, they also note that although a positive correlation exists between the number of individual animals and overall institutional species richness, this rate is not consistent across taxonomic groups, and that the number of individual animals increases at a significantly higher rate for mammals[7]. As a result, we link 'Collection Taxonomy' to 'Number of Animals' in our meta-model. Furthermore, it is shown that the majority of threatened species with viable population sizes across institutions are mammalian species. This mammalian bias is a constant trend throughout this work, due to the perception that mammalian species are more attractive to the public. Therefore, we link 'Collection Taxonomy' to 'Threatened Species' in our meta-model.

This mammalian bias is elaborated upon in Frynta *et al.*[8], who explicitly investigated the influence of body size on the representation of mammals across zoological collections. They show that there is a higher probability of large and attractive mammalian families being kept in zoos[8]. Additionally, they show that once kept, these large and attractive mammalian species are presented in larger numbers and in more institutions[8]. This provides further support that taxonomic biases across institutions influence the numbers of animals kept. Based on these findings, we link 'Institution Body Mass' and 'Collection Taxonomy' to 'Number of Animals' in our meta-model.

During our review of the literature we recorded the types and number of variables measured by the various authors. As the individual studies cited in the *a priori* meta-model measured very specific relationships, we simplified their measures into broader themes. For example, Frynta *et al.*[8] show that mammalian families, once kept in zoos, are presented in larger numbers than non-mammalian families. Therefore, in the *a priori* meta-model we interpret this and broaden it to 'Collection Taxonomy' (mammalian bias in this example) being correlated with the 'Number of Animals'. However, these are general concepts used to help define the *a priori* meta-model and they do not appear in the final model. Rather than create single indicator latent variables, we place the exact variables measured into our final models. So, instead of placing 'Collection Taxonomy' in the final model as a single indicator latent variable, we used the exact measured variable i.e. "Mammal Species Richness [per institution]". In this manner we use specific relationships from the literature to define general concepts in the *a priori* meta-model, these general concepts are then represented in the final models by specific measurements once again. This explains why certain variables in the *a priori* meta-model do not appear in the final models (e.g. 'Collection Taxonomy').

Proposed Causal Hypotheses

Although we gained significant insight into various aspects of the system from our literature review, we failed to find any work integrating the various institutional characteristics and socio-economic variables into a study investigating visitor attendance and *in situ* conservation contributions. Although, considerable work has been done on the links between socio-economic variables and visitor attendance, there is a noticeable lack of research on the influence of institutional characteristics (in terms of type and number of species kept) on visitor attendance. This is surprising due to the persistent belief that large, charismatic mammals are necessary to attract visitors; while this concept was found many times in our literature review, it lacks rigorous assessment.

The modelling approach used here was semi-exploratory, similar to Grace *et al.* [9]. Therefore, we combined prior theoretical knowledge with proposed causal hypotheses, capturing all evidence-based relationships and all plausible and suspected predictors of attendance and *in situ* contributions. These plausible direct pathways have no citation in the reported *a priori* meta-model due to an absence of related studies in the literature, however their potential influence was an important consideration. For example, it is repeatedly mentioned in the literature that visitors expect to see large, charismatic mammals, so in the absence of published work to demonstrate this phenomenon, we link 'Collection Taxonomy', 'Mammal Species Richness' and 'Institution Body Mass' to 'Institution Attendance' in our meta-model.

Similarly, given that there is a higher probability of large and attractive mammals being kept in zoos and that they are presented in larger numbers once kept, we also think it is plausible that 'Institution Species Richness' and 'Number of Animals' could both influence 'Institution Attendance'. It may also be expected that 'Collection Diversity' would influence 'Institution

Attendance' as Whitworth has already shown that 'rare' and 'exotic' species are the most popular among visitors[4]. Therefore, we include various metrics (diversity and dissimilarity) among the variables we include during analyses. Finally, it is logical to assume that larger institutions will attract more visitors, so we link 'Institution Area' and 'Institution Type' to 'Institution Attendance' in our meta-model. Unfortunately, the extreme lack of published literature on '*In Situ* Project Investment' meant that the only plausible and defensible link we could add was that from 'Institution Attendance' to '*In Situ* Project Investment', as we assume 'Institution Attendance' is a good proxy for the available funds for potential *in situ* investment.

All of these relationships resulted in the generation of the *a priori* meta-model shown in Supplementary Figure 1. This hypothesised causal diagram was combined with available data to test the effects of institutional compositional characteristics and socio-economic variables on visitor attendance and *in situ* contributions. This meta-model was only the first step in the modelling procedure, guiding original model specification and modifications[1].

Code Availability

Please see the Supplementary Code for further details on how the meta-model was refined into the final models depicted in Figure 2 and Supplementary Figure 4, using the approach described in Grace *et al.* [10] and similar to that implemented in Grace *et al.* [9].

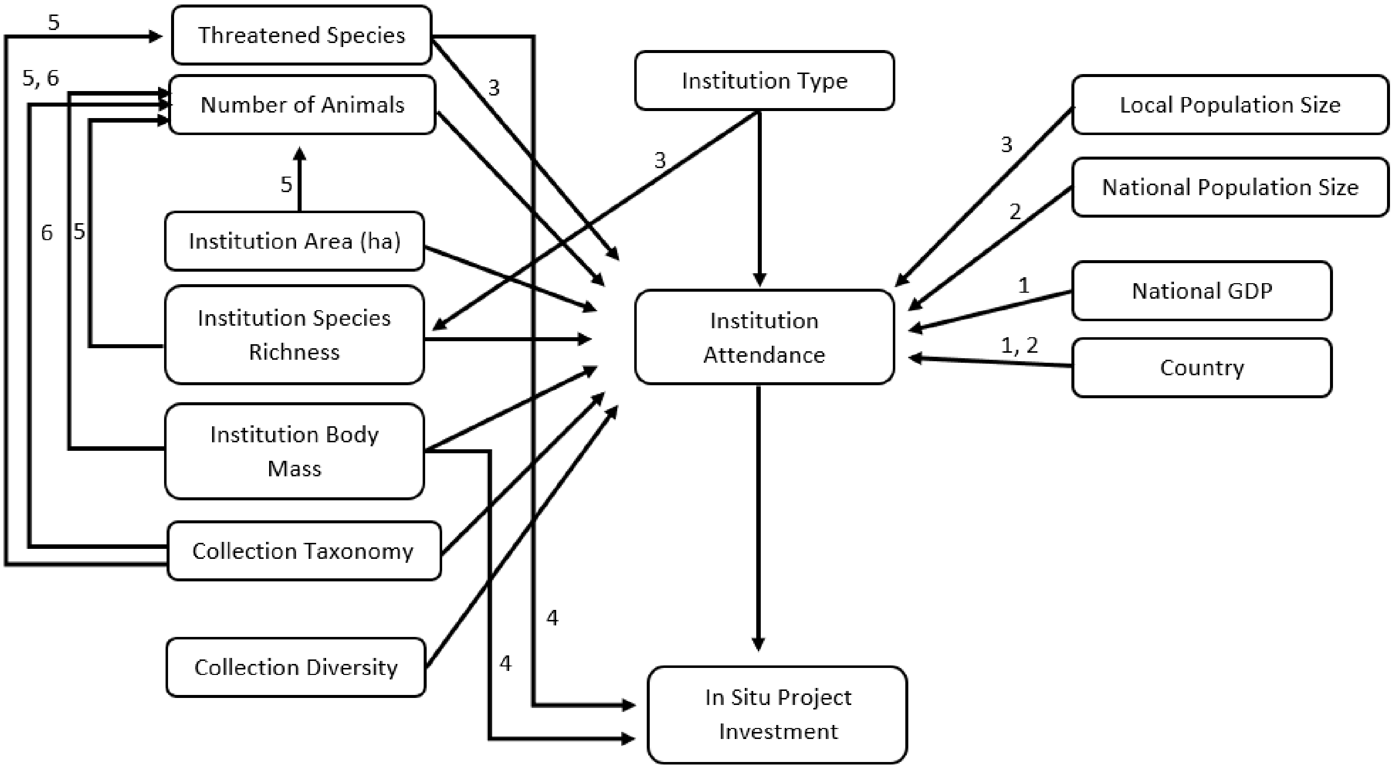

Supplementary Figure 1 | The *a priori* SEM framework meta-model, combining both theoretical knowledge and proposed causal hypotheses. Numbers indicate evidence-based relationships from the scientific literature. 1: [2], 2: [3], 3: [4], 4: [5], 5: [7], 6: [8].

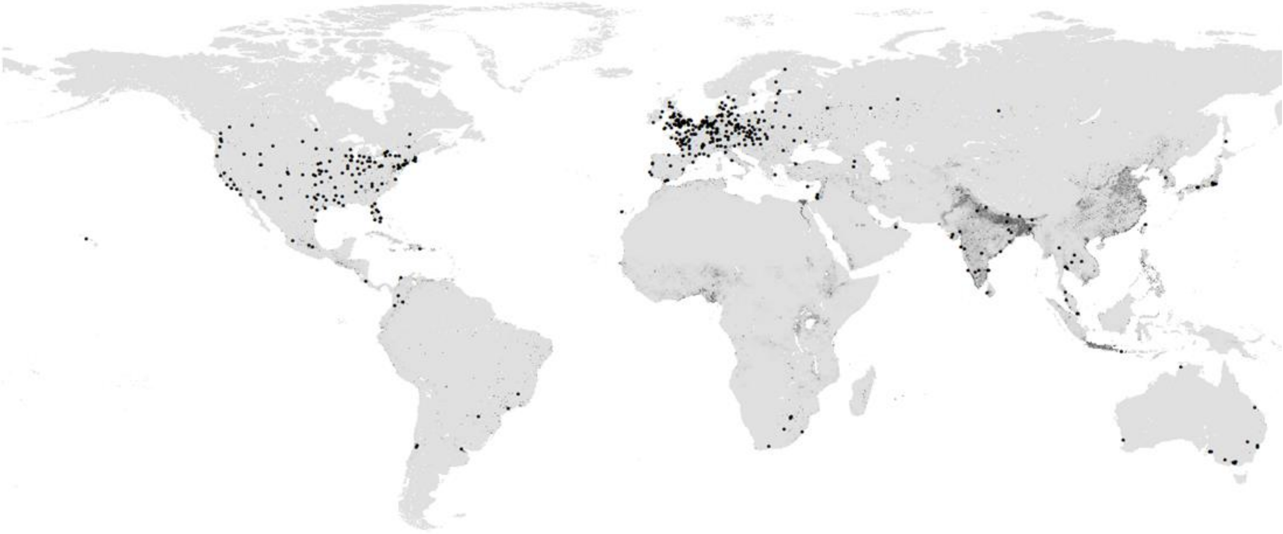

Supplementary Figure 2 | The global distribution of the Species360 and IZY member institutions used in this study (n = 458). Black dots represent individual institutions. These are presented on a global map showing population density at a resolution of 1 km (darker meaning more densely populated)[11]. As institution location could be used to identify the anonymized data, we have not provided location data for the institutions in Supplementary Data 1 and 2.

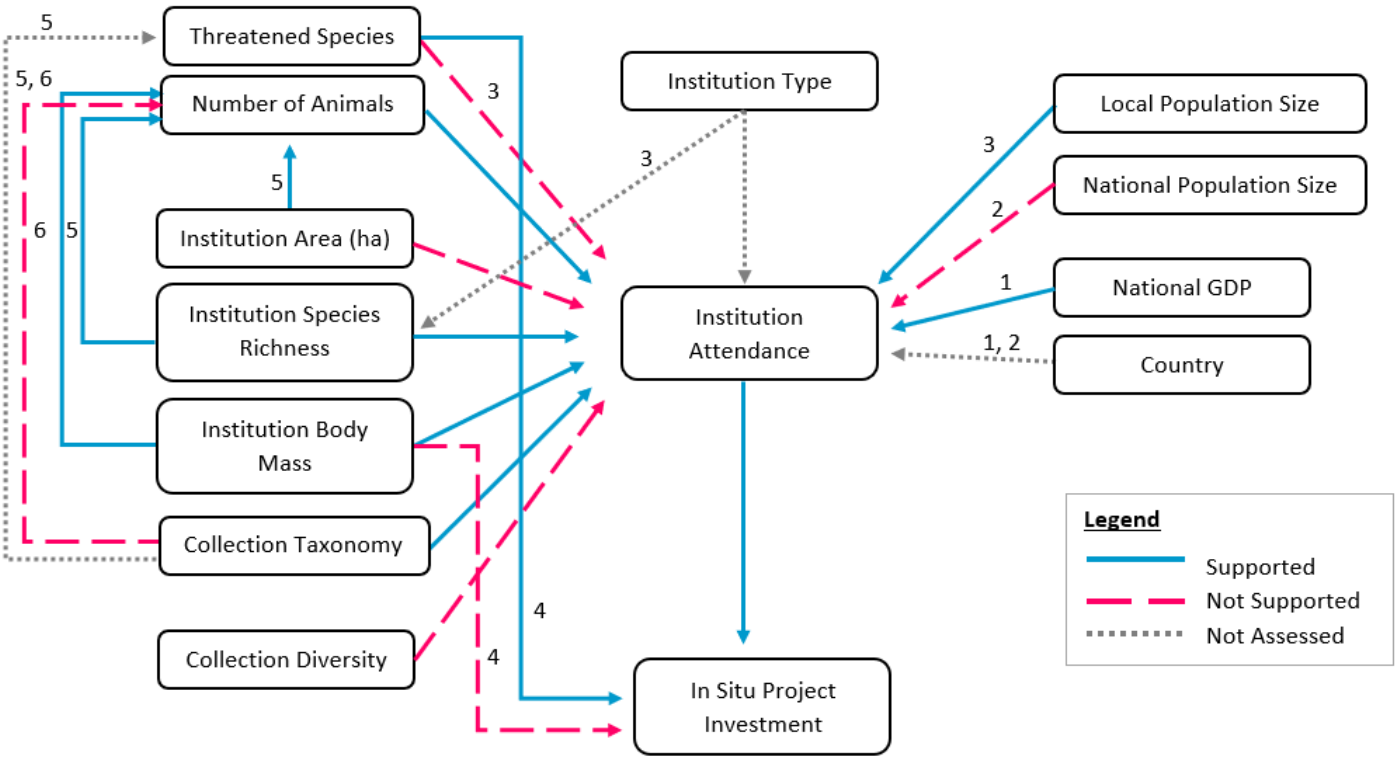

Supplementary Figure 3 | The *a priori* SEM framework meta-model updated with the results of this study. Blue lines indicate relationships supported by this study, dashed pink lines indicate relationships for which no support was found and grey dotted lines indicate relationships that were not assessed. 1: [2], 2: [3], 3: [4], 4: [5], 5: [7], 6: [8]. Source Data: supported and unsupported relationships are documented in Supplementary Code provided.

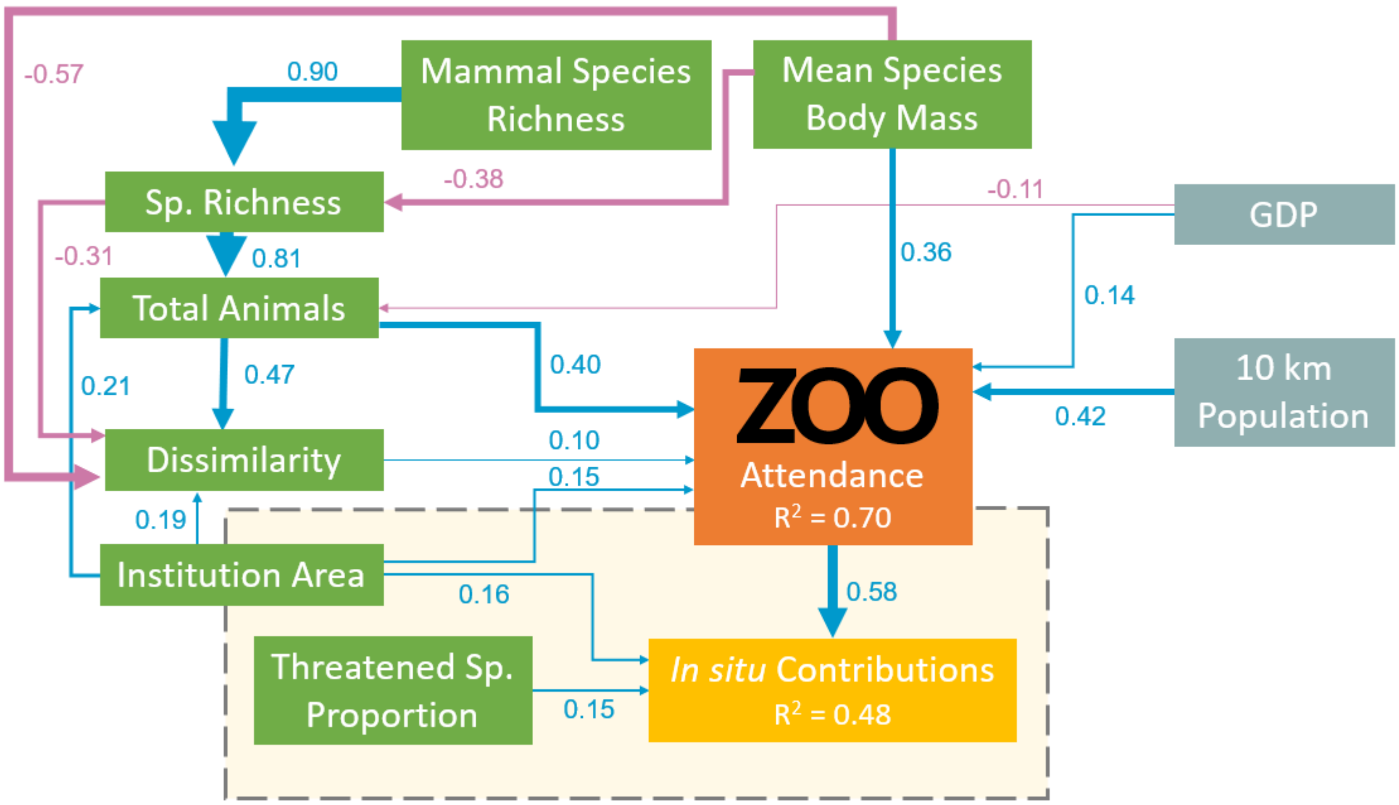

Supplementary Figure 4 | The combined Attendance (n = 458) and *In Situ* (n = 119) SEMs representing the connections between institution attendance, *in situ* contributions and various institutional and socio-economic variables. Model results presented reflect species presence-absence models. Path coefficients shown are standardised. The yellow box indicates the additional pathways included in the *In Situ* model. Effect sizes and R^2 for the attendance portion of the figure are derived from the Attendance model, with values for the pathways in the yellow box derived from the *In Situ* model. Blue arrows represent positive effects and purple arrows represent negative effects. Line width represents relative effect sizes. Grey boxes represent socio-economic variables and green boxes represent institutional variables. See Table 1 for variable descriptions, Supplementary Table 3 for test statistics and fit indices and Supplementary Table 4 for standardised path coefficients, total effect sizes, significance values and proposed interpretations of causal pathways. Source Data: Supplementary Data 1 and 2 provided.

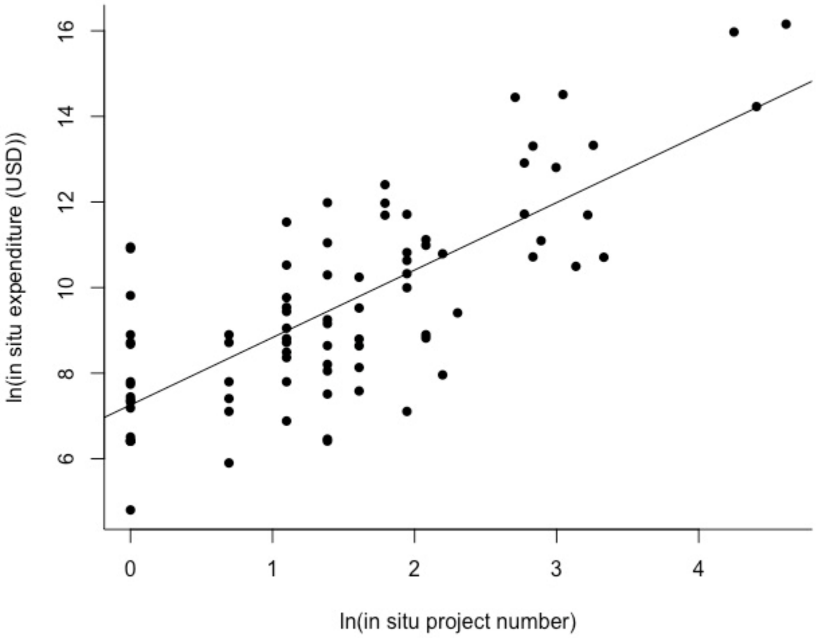

Supplementary Figure 5. The relationship between the number of *in situ* conservation projects supported and the total financial *in situ* conservation expenditure (USDollars) of 83 individual BIAZA institutions for the year 2018, both variables are natural log transformed. The significant effect of project number ($P < 0.0001$) is shown as the best fit line from a linear regression with an $R^2 = 0.56$. Source Data: Supplementary Data 3 provided.

Supplementary Table 1 | AICc values of competing Attendance and *In Situ* models, reflecting both species presence-absence and species abundance adjusted models. Shown are AICc values for the *a priori* meta-models (Model A) and the four top performing models (Models B – E), including the final models selected (Model E). See the Supplementary R Code provided for specific model details. Source Data: Supplementary Data 1 and 2 provided.

	Species Presence-Absence		Species Abundance	
	AICc	Parameters	AICc	Parameters
**Attendance Model**				
Model E (Final Model)	9589.54	43	9575.78	44
Model D	9715.77	42	9574.71	45
Model C	10244.94	50	9589.28	44
Model B	10252.14	49	9595.53	43
Model A *(a priori* meta-model)	14684.32	78	15315.69	88
***In Situ* Model**				
Model E (Final Model)	2672.51	34	2677.51	34
Model D	2671.34	35	2679.70	35
Model C	2683.82	34	2868.82	39
Model B	2686.40	35	2869.63	40
Model A *(a priori* meta-model)	3419.69	66	3447.56	67

Supplementary Table 2 | Chi-Squared Statistics and Absolute and Incremental Fit Indices for both the Attendance and *In Situ* models. Model results presented reflect abundance adjusted models. Good model fit ranges determined by[12]. Numbers in bold represent values that fall within the good model fit ranges. Adjusted Goodness of Fit Index (AGFI), Root Mean Square Residual (RMR), Standardised Root Mean Square Residual (SRMR), Root Mean Square Error of Approximation (RMSEA), Comparative Fit Index (CFI), Non-Normed Fit Index (NNFI) and Tucker Lewis Index (TLI). Source Data: Supplementary Data 1 and 2 provided.

	Absolute Fit Indices					Incremental Fit Indices		
Goodness of Fit Measures	χ2 / (df)	AGFI	RMR	SRMR	RMSEA	CFI	NNFI	TLI
Good Model Fit Ranges	< 3.0	> 0.90	< 0.08	< 0.08	< 0.06	> 0.95	> 0.90	> 0.90
Attendance Model	**1.953 (10)**	0.802	**0.053**	**0.048**	0.122	**0.966**	**0.910**	**0.910**
In Situ Model	**2.217 (21)**	0.826	**0.056**	**0.057**	0.101	**0.959**	**0.932**	**0.932**

Supplementary Table 3 | Chi-Squared Statistics and Absolute and Incremental Fit Indices for both the Attendance and *In Situ* models. Model results presented reflect species presence-absence models. Good model fit ranges determined by[12]. Numbers in bold represent values that fall within the good model fit ranges. Adjusted Goodness of Fit Index (AGFI), Root Mean Square Residual (RMR), Standardised Root Mean Square Residual (SRMR), Root Mean Square Error of Approximation (RMSEA), Comparative Fit Index (CFI), Non-Normed Fit Index (NNFI) and Tucker Lewis Index (TLI). Source Data: Supplementary Data 1 and 2 provided.

	Absolute Fit Indices					Incremental Fit Indices		
Goodness of Fit Measures	χ2 / (df)	AGFI	RMR	SRMR	RMSEA	CFI	NNFI	TLI
Good Model Fit Ranges	< 3.0	> 0.90	< 0.05	< 0.08	< 0.08	> 0.95	> 0.90	> 0.90
Attendance Model	**2.665 (11)**	0.787	0.063	**0.053**	0.122	**0.963**	**0.911**	**0.911**
In Situ Model	**2.020 (21)**	0.842	0.058	**0.059**	0.093	**0.965**	**0.942**	**0.942**

Supplementary Table 4 | Direct and total standardised effect sizes, R^2 values, standard errors, p-values and proposed interpretations for both the Attendance and *In Situ* models. Relationships are ranked according to direct effect size magnitude. Model results presented reflect species presence-absence models. Only the *in situ* component of the *In Situ* model is reported as all other pathways were analogous to the Attendance model. Source Data: Supplementary Data 1 and 2 provided.

	P-Value	Direct Effect (SE)	Total Effect	Interpretation
		**Attendance Model**		
**Attendance (R^2 = 0.702)**				
Attendance ~ 10km Population	< 0.001	0.416 (0.039)	0.416	Attendance is positively correlated with the local population size (10 km radius) surrounding an institution
Attendance ~ Total Animals	< 0.001	0.397 (0.035)	0.444	Attendance is positively correlated with total number of animals in an institution
Attendance ~ Body Mass	< 0.001	0.361 (0.048)	0.181	Attendance is positively correlated with the mean species body mass for an institution
Attendance ~ Institution Area	0.005	0.148 (0.053)	0.261	Attendance has a small, but positive correlation with institution area
Attendance ~ GDP	0.001	0.139 (0.042)	0.090	Attendance is positively correlated with national GDP
Attendance ~ Dissimilarity	0.006	0.101 (0.037)	0.101	Attendance is positively correlated with collection dissimilarity
**Total Animals (R^2 = 0.772)**				
Total Animals ~ Species Richness	< 0.001	0.812 (0.036)	0.812	The total number of animals in an institution is positively correlated with institutional species richness
Total Animals ~ Institution Area	< 0.001	0.208 (0.037)	0.208	The total number of animals in an institution is positively correlated with institutional area
Total Animals ~ GDP	0.155	-0.111 (0.078)	-0.111	The total number of animals in an institution is negatively correlated with National GDP
**Species Richness (R^2 = 0.682)**				
Species Richness ~ Mammal Species Richness	< 0.001	0.902 (0.101)	0.902	Institutional species richness is strongly positively correlated with institutional mammal species richness
Species Richness ~ Body Mass	< 0.001	-0.376 (0.057)	-0.376	Institutional species richness is negatively correlated with the mean species body mass of an institution
**Dissimilarity (R^2 = 0.297)**				
Dissimilarity ~ Total Animals	0.001	0.470 (0.140)	0.470	Collection composition dissimilarity is positively correlated with the total number of animals in an institution
Dissimilarity ~ Institution Area	0.027	0.194 (0.087)	0.292	Collection composition dissimilarity is positively correlated with the mean species body mass of an institution
Dissimilarity ~ Species Richness	0.145	-0.313 (0.215)	0.069	Collection composition dissimilarity is negatively correlated with institutional species richness
Dissimilarity ~ Body Mass	< 0.001	-0.569 (0.076)	-0.712	Collection composition dissimilarity is negatively correlated with the mean species body mass of an institution
		***In Situ* Model**		
***In Situ* Contributions (R^2 = 0.476)**				
In Situ ~ Attendance	< 0.001	0.576 (0.074)	0.576	Institutional *in situ* contributions are positively correlated with institutional attendance
In Situ ~ Institution Area	0.025	0.156 (0.070)	0.295	Institutional *in situ* contributions are positively correlated with institutional area
In Situ ~ Threatened Species Proportion	0.029	0.149 (0.068)	0.149	Institutional *in situ* contributions are positively correlated with the proportion of threatened species in an institution

Supplementary Table 5 | Residual covariances for both the Attendance (n = 458) and *In Situ* (n = 119) models. Results presented reflect species abundance models. Source Data: Supplementary Data 1 and 2 provided.

	Estimate	Standard Error	P-Value
**Attendance Model**			
Mammal Species Richness ~~ Body Mass	0.227	0.078	0.003
Mammal Species Richness ~~ 10km Population	0.284	0.066	< 0.001
Mammal Species Richness ~~ GDP	-0.060	0.064	0.349
Mammal Species Richness ~~ Institution Area	0.381	0.064	< 0.001
Body Mass ~~ 10km Population	0.021	0.064	0.742
Body Mass ~~ GDP	-0.079	0.038	0.040
Body Mass ~~ Institution Area	0.522	0.101	< 0.001
10km Population ~~ GDP	-0.028	0.056	0.613
10km Population ~~ Institution Area	-0.010	0.078	0.901
GDP ~~ Institution Area	-0.027	0.041	0.501
***In Situ* Model**			
Body Mass ~~ 10km Population	-0.209	0.093	0.025
Body Mass ~~ Institution Area	0.527	0.103	< 0.001
Body Mass ~~ Mammal Species Richness	0.219	0.093	0.018
Body Mass ~~ Threatened Species Proportion	0.092	0.091	0.312
10km Population ~~ Institution Area	-0.099	0.091	0.277
10km Population ~~ Mammal Species Richness	0.421	0.099	< 0.001
10km Population ~~ Threatened Species Proportion	0.278	0.094	0.003
Institution Area ~~ Mammal Species Richness	0.536	0.103	< 0.001
Institution Area ~~ Threatened Species Proportion	0.071	0.091	0.438
Mammal Species Richness ~~ Threatened Species Proportion	0.203	0.093	0.028

Supplementary Table 6 | Residual covariances for both the Attendance (n = 458) and *In Situ* (n = 119) models. Results presented reflect species presence-absence models. Source Data: Supplementary Data 1 and 2 provided.

	Estimate	Standard Error	P-Value
**Attendance Model**			
Institution Area ~~ Body Mass	0.570	0.136	< 0.001
Institution Area ~~ 10km Population	-0.025	0.083	0.763
Institution Area ~~ GDP	-0.020	0.074	0.788
Institution Area ~~ Mammal Species Richness	0.292	0.087	0.001
Body Mass ~~ 10km Population	0.127	0.104	0.221
Body Mass ~~ GDP	-0.145	0.063	0.021
Body Mass ~~ Mammal Species Richness	0.218	0.119	0.066
10km Population ~~ GDP	-0.119	0.071	0.094
10km Population ~~ Mammal Species Richness	0.258	0.095	0.007
GDP ~~ Mammal Species Richness	-0.057	0.093	0.540
***In Situ* Model**			
Body Mass ~~ 10km Population	-0.023	0.091	0.804
Body Mass ~~ Institution Area	0.560	0.104	< 0.001
Body Mass ~~ Mammal Species Richness	0.373	0.097	< 0.001
Body Mass ~~ Threatened Species Proportion	0.277	0.094	0.003
10km Population ~~ Institution Area	-0.099	0.091	0.277
10km Population ~~ Mammal Species Richness	0.421	0.099	< 0.001
10km Population ~~ Threatened Species Proportion	0.287	0.095	0.002
Institution Area ~~ Mammal Species Richness	0.536	0.103	< 0.001
Institution Area ~~ Threatened Species Proportion	0.189	0.093	0.041
Mammal Species Richness ~~ Threatened Species Proportion	0.322	0.096	0.001

Supplementary Information References

1. Grace, J. B., Anderson, T. M., Olff, H. & Scheiner, S. M. On the specification of structural equation models for ecological systems. *Ecol. Monogr.* **80,** 67–87 (2010).
2. Leader-Williams, N. *et al.* Beyond the ark: conservation biologists' views of the achievements of zoos in conservation. in *Zoos in the 21st century: Catalysts for conservation?* (eds. Zimmermann, A., Hatchwell, M., Dickie, L. A. & West, C.) 236–254 (Cambridge University Press, 2007).
3. Davey, G. An analysis of country, socio-economic and time factors on worldwide zoo attendance during a 40 year period. *Int. Zoo Yearb.* **41,** 217–225 (2007).
4. Whitworth, A. W. An Investigation into the Determining Factors of Zoo Visitor Attendances in UK Zoos. *PLoS One* **7,** e29839 (2012).
5. Dickie, L. A., Bonner, J. P. & West, C. D. *In situ* and *ex situ* conservation: blurring the boundaries between zoos and the wild. in *Zoos in the 21st Century: Catalysts for Conservation?* (eds. Zimmermann, A., Hatchwell, M., Dickie, L. A. & West, C.) 220–235 (Cambridge University Press, 2007).
6. Conway, W. G. Buying Time for wild animals With Zoos. *Zoo Biol.* **30,** 1–8 (2011).
7. Fa, J. E., Funk, S. M. & OConnell, D. *Zoo Conservation Biology*. (Cambridge University Press, 2011). doi:10.1017/CBO9780511993435
8. Frynta, D., Šimková, O., Lišková, S. & Landová, E. Mammalian Collection on Noah's Ark: The Effects of Beauty, Brain and Body Size. *PLoS One* **8,** e63110 (2013).
9. Grace, J. B. *et al.* Integrative modelling reveals mechanisms linking productivity and plant species richness. *Nature* **529,** 390–393 (2016).
10. Grace, J. B., Scheiner, S. M. & Schoolmaster, D. R. J. Structural equation modeling:

building and evaluating causal models. in *Ecological Statistics: Contemporary theory and application* (eds. Fox, G. A., Negrete-Yankelevich, S. & Sosa, V. J.) 168–199 (Oxford University Press, 2015).

11. Center for International Earth Science Information Network (CIESIN) - Columbia University, Gridded Population of the World, Version 4 (GPWv4): Population Count, Revision 10 (2017), (Palisades, NY: NASA Socioeconomic Data and Applications Center (SEDAC), 2017) (available at https://doi.org/10.7927/H4PG1PPM).

12. Hu, L. & Bentler, P. M. Cutoff criteria for fit indexes in covariance structure analysis: Conventional criteria versus new alternatives. *Struct. Equ. Model. A Multidiscip. J.* **6,** 1–55 (1999).
