# Supplementary material for: A system wide approach to managing zoo collections for visitor attendance and in situ conservation
Source: Nat Commun. 2020 Feb 4;11:584. doi: 10.1038/s41467-020-14303-2 (PMC7000708; doi:10.1038/s41467-020-14303-2)
Supplement: Supplementary file 3 — Description of Additional Supplementary Information Files [file 41467_2020_14303_MOESM3_ESM.docx]

**Description of Additional Supplementary Files**

**Metadata**

**File Name:** Supplementary Data 1. (separate file)

**Description:** All data required for the Attendance SEM framework (n = 458).

*Identity:* Supplementary Data 1.csv

*Format:* Microsoft Excel Comma Separated Values File (.csv)

*Header information:*

Annonymous.ID, Country, Zoo.Area.ha, Attendance, Sp.Richness, Total.Animals, Mam.Sp.Richness, Prop.Mam.Sp, Total.Mammals, Prop.Mam.Abdun, Threatened.Sp, Prop.Threat.Sp, Threatened.Abund, Prop.Threat.Abund, Total.Sp.BodyMass, Mean.Sp.BodyMass, Total.Sp.BodyMassXAbund, Mean.Sp.BodyMassXAbund, Brillouin.Index, Mean.Jaccard.Dissim.P.A., Mean.Raup.Crick, GDP(Millions)_WBank2015, Nat_Pop _WB2015, 50km_Pop, 10km_Pop

**Metadata Supplementary Table 1 | The variable names used, an explanation of their meaning and the source of the data contained in that variable for the Attendance model (n = 458).** Variables assessed, but not retained in the final models are also presented.

| **Variable name** | **Variable definition** | **Variable Source and Date** |
| --- | --- | --- |
| **Annonymous.ID** | Anonymous institution identification number |  |
| **Country** | Country of institution |  |
| **Zoo.Area.ha** | Institutional area in hectares | International Zoo Yearbook, 2016 |
| **Attendance** | Institutional attendance | International Zoo Yearbook, 2016 |
| **Sp.Richness** | Total number of species in institution | ZIMS, 2017 |
| **Total.Animals** | Total number of individual animals in institution | ZIMS, 2017 |
| **Mam.Sp.Richness** | Total number of mammalian species in institution | ZIMS, 2017 |
| **Prop.Mam.Sp** | The proportion of total species made up of mammals in institution | ZIMS, 2017 |
| **Total.Mammals** | The total number of individual mammals in institution | ZIMS, 2017 |
| **Prop.Mam.Abdun** | The proportion of total individual animals made up of mammals in institution | ZIMS, 2017 |
| **Threatened.Sp** | Number of ‘Threatened’ (IUCN ‘CR’, ‘EN’, ‘VU’ categories) species within institution | ZIMS, 2017 |
| **Prop.Threat.Sp** | The proportion of total species made up of ‘Threatened’ species in institution | ZIMS, 2017 |
| **Threatened.Abund** | Number of ‘Threatened’ individuals within institution | ZIMS, 2017 |
| **Prop.Threat.Abund** | The proportion of total individual animals made up of ‘Threatened’ species in institution | ZIMS, 2017 |
| **Total.Sp.BodyMass** | The total body mass (g) of each species within the institution | ZIMS, 2017 / Conde *et al.,* 2019 |
| **Mean.Sp.BodyMass** | The mean species body mass (g) of the institution | ZIMS, 2017 / Conde *et al.,* 2019 |
| **Total.Sp.BodyMassXAbund** | The total body mass (g) of each individual within the institution | ZIMS, 2017 / Conde *et al*., 2019 |
| **Mean.Sp.BodyMassXAbund** | The mean individual body mass (g) of the institution | ZIMS, 2017 / Conde *et al.,* 2019 |
| **Brillouin.Index** | The Brillouin index for the institution | ZIMS, 2017 |
| **Mean.Jaccard.Dissim.P.A.** | The mean Jaccard Dissimilarity Index for the institution | ZIMS, 2017 |
| **Mean. Raup.Crick** | The mean Raup–Crick Dissimilarity Index for the institution. | ZIMS, 2017 |
| **GDP(Millions)_WBank2015** | GDP in Millions (US$) for the year 2015 | World Bank, 2017 |
| **Nat_Pop _WB2015** | National Population Size for the year 2015 | World Bank, 2017 |
| **50km_Pop** | Estimated population count within a 50km radius of the institution | CIESIN, 2016 |
| **10km_Pop** | Estimated population count within a 10km radius of the institution | CIESIN, 2016 |

**File Name:** Supplementary Data 2. (separate file)

**Description:** All data required for the *In Situ* SEM framework (n = 119 subset).

*Identity:* Supplementary Data 2.csv

*Format:* Microsoft Excel Comma Separated Values File (.csv)

*Header information:*

Annonymous.ID, Country, Zoo.Area.ha, Attendance, Sp.Richness, Total.Animals, Mam.Sp.Richness, Prop.Mam.Sp, Total.Mammals, Prop.Mam.Abdun, Threatened.Sp, Prop.Threat.Sp, Threatened.Abund, Prop.Threat.Abund, Total.Sp.BodyMass, Mean.Sp.BodyMass, Total.Sp.BodyMassXAbund, Mean.Sp.BodyMassXAbund, Brillouin.Index, Mean.Jaccard.Dissim.P.A., Mean.Raup.Crick, GDP(Millions)_WBank2015, Nat_Pop _WB2015, 50km_Pop, 10km_Pop, insitu

**Metadata Supplementary Table 2 | The variable names used, an explanation of their meaning and the source of the data contained in that variable for the *In Situ* model (n = 119).** Variables assessed, but not retained in the final models are also presented.

| **Variable name** | **Variable definition** | **Variable Source and Date** |
| --- | --- | --- |
| **Annonymous.ID** | Anonymous institution identification number |  |
| **Country** | Country of institution |  |
| **Zoo.Area.ha** | Institutional area in hectares | International Zoo Yearbook, 2016 |
| **Attendance** | Institutional attendance | International Zoo Yearbook, 2016 |
| **Sp.Richness** | Total number of species in institution | ZIMS, 2017 |
| **Total.Animals** | Total number of individual animals in institution | ZIMS, 2017 |
| **Mam.Sp.Richness** | Total number of mammalian species in institution | ZIMS, 2017 |
| **Prop.Mam.Sp** | The proportion of total species made up of mammals in institution | ZIMS, 2017 |
| **Total.Mammals** | The total number of individual mammals in institution | ZIMS, 2017 |
| **Prop.Mam.Abdun** | The proportion of total individual animals made up of mammals in institution | ZIMS, 2017 |
| **Threatened.Sp** | Number of ‘Threatened’ (IUCN ‘CR’, ‘EN’, ‘VU’ categories) species within institution | ZIMS, 2017 |
| **Prop.Threat.Sp** | The proportion of total species made up of ‘Threatened’ species in institution | ZIMS, 2017 |
| **Threatened.Abund** | Number of ‘Threatened’ individuals within institution | ZIMS, 2017 |
| **Prop.Threat.Abund** | The proportion of total individual animals made up of ‘Threatened’ species in institution | ZIMS, 2017 |
| **Total.Sp.BodyMass** | The total body mass (g) of each species within the institution | ZIMS, 2017 / Conde *et al.,* 2019 |
| **Mean.Sp.BodyMass** | The mean species body mass (g) of the institution | ZIMS, 2017 / Conde *et al.,* 2019 |
| **Total.Sp.BodyMassXAbund** | The total body mass (g) of each individual within the institution | ZIMS, 2017 / Conde *et al.,* 2019 |
| **Mean.Sp.BodyMassXAbund** | The mean individual body mass (g) of the institution | ZIMS, 2017 / Conde *et al.,* 2019 |
| **Brillouin.Index** | The Brillouin index for the institution | ZIMS, 2017 |
| **Mean.Jaccard.Dissim.P.A.** | The mean Jaccard Dissimilarity Index for the institution | ZIMS, 2017 |
| **Mean. Raup.Crick** | The mean Raup–Crick Dissimilarity Index for the institution. | ZIMS, 2017 |
| **GDP(Millions)_WBank2015** | GDP in Millions (US$) for the year 2015 | World Bank, 2017 |
| **Nat_Pop _WB2015** | National Population Size for the year 2015 | World Bank, 2017 |
| **50km_Pop** | Estimated population count within a 50km radius of the institution | CIESIN, 2016 |
| **10km_Pop** | Estimated population count within a 10km radius of the institution | CIESIN, 2016 |
| **insitu** | The number of field conservation projects invested in by the institution in 2015 | AZA, 2015 |

**File Name:** Supplementary Data 3. (separate file)

**Description:** All data required for Supplementary Figure 5.

*Identity:* Supplementary Data 3.csv

*Format:* Microsoft Excel Comma Separated Values File (.csv)

*Header information:*

AnonID, in_situ_projects, in_situ_pounds, in_situ_dollars

**Metadata Supplementary Table 3 | The variable names used, an explanation of their meaning and the source of the data contained in Figure 3.**

| **Variable name** | **Variable definition** | **Variable Source and Date** |
| --- | --- | --- |
| **AnonID** | Anonymous institution identification number |  |
| **in_situ_projects** | The number of field conservation projects invested in by the institution in 2018 | BIAZA, 2019 |
| **in_situ_pounds** | The financial expenditure (in GBP) on field conservation projects by the institution in 2018 | BIAZA, 2019 |
| **in_situ_dollars** | The financial expenditure (in USD) on field conservation projects by the institution in 2018 | BIAZA, 2019 |

**File Name:** Supplementary Data 4. (separate file)

**Description:** Validation Set 1.

**File Name:** Supplementary Data 5. (separate file)

**Description:** Validation Set 2.

**File Name:** Supplementary Data 6. (separate file)

**Description:** Validation Set 3.

**File Name:** Supplementary Data 7. (separate file)

**Description:** Validation Set 4.
