# Supplementary material for: A system wide approach to managing zoo collections for visitor attendance and in situ conservation
Source: Nat Commun. 2020 Feb 4;11:584. doi: 10.1038/s41467-020-14303-2 (PMC7000708; doi:10.1038/s41467-020-14303-2)
Supplement: Supplementary file 4 — Supplementary Code [file 41467_2020_14303_MOESM4_ESM.pdf]

Zoo Attendance SEM

Andew Mooney

08/08/2019

This script describes how we developed the structural equation models in the paper Mooney *et al.,* 2019.

This is based on the approach described in (Grace *et al.,* 2015) and similar to the semi-exploratory modelling approach implemented in (Grace *et al.,* 2016). The module **Quantitative Analysis Using Structural Equation Modeling** provided by the United States Geological Survey and developed by Dr. James Grace (available at:https://www.usgs.gov/centers/wetland-and-aquatic-research-center/science/quantitative-analysis-using-structural-equation?qt-science_center_objects=0#qt-science_center_objects) was used as an introduction to SEM in ecology and as a guide for the correction implementation of SEM. Fan *et al.,* 2016 was also used as a guide to help avoid common mistakes in SEM implementation and reporting, with a particular focus on SEM in ecological studies.

Contents

Data and Packages
Attendance SEM Species Presence - Absence models
Final Attendance Model (Species Presence - Absence) Species Abundance models
Final Attendance Model (Species Abundance)
Species Abundance In Situ models (Abundance)
Final In Situ Model (Species Abundance)

Data and Packages

Contents
We use the lavaan package, lavaan.survey, and AICcmodavg packages to fit the models and determine which variables to include in the final SEM model.

```
# load the packages
library(lavaan)
```

```
## This is lavaan 0.5-23.1097

## lavaan is BETA software! Please report any bugs.
```

```
library(lavaan.survey)
```

```
## Loading required package: survey

## Loading required package: grid

## Loading required package: Matrix

## Loading required package: survival

##
## Attaching package: 'survey'

## The following object is masked from 'package:graphics':
##
##     dotchart
```

```r
library(AICcmodavg)
library(survey)

# We also load the additional function "lavaan.modavg.R" to generate a table
# of AICc model comparisons.
source("lavaan.modavg.R")
```

We then upload the data. This included the attendance model data (Supplementary Data 1, n = 458 institutions), the in situ model data (Supplementary Data 2, subset of n = 119 institutions) and four random sets of the attendance data (consisting of n = 200 each) which were used to validate the final model. See the supplementary information for definitions of each variable included in Supplementary Data 1 and 2.

```r
# Load Data S1 (attendance model data)
DataS1 <- read.csv("Supplementary Data 1.csv", header = T, sep = ",")

# reading the in situ model data (Data S2)
DataS2 <- read.csv("Supplementary Data 2.csv", header = T, sep = ",")

#read in the four validation data sets
validationset1 <- read.csv("Supplementary Data 4.csv", header = T, sep = "," )
validationset2 <- read.csv("Supplementary Data 5.csv", header = T, sep = "," )
validationset3 <- read.csv("Supplementary Data 6.csv", header = T, sep = "," )
validationset4 <- read.csv("Supplementary Data 7.csv", header = T, sep = "," )

# We then rename the attendance and in situ model data and retain the raw orginal data.
sem_attendance_data <- DataS1
sem_in_situ_data <- DataS2
```

Logging and mean centered data

Contents
Standardising the data is an important step for SEM and a requirement of using lavaan, as lavaan requires the standardization of variances to an approximately similar scale. This ensures all variables are on similar scales and hence weighted appropriately when fitting the model. Here we log each of the variables.

```r
# log transformation of the variables
sem_attendance_data$lAttendance <- log(sem_attendance_data$Attendance)
sem_attendance_data$lZoo.Area.ha <- log(sem_attendance_data$Zoo.Area.ha)
sem_attendance_data$lSp.Richness <- log(sem_attendance_data$Sp.Richness)
sem_attendance_data$lTotal.Animals <- log(sem_attendance_data$Total.Animals)
sem_attendance_data$lThreatened.Sp <- log(sem_attendance_data$Threatened.Sp)
sem_attendance_data$lProp.Threat.Sp <- log(sem_attendance_data$Prop.Threat.Sp)
sem_attendance_data$lProp.Threat.Abund <- log(sem_attendance_data$Prop.Threat.Abund)
sem_attendance_data$lMean.Sp.BodyMass <- log(sem_attendance_data$Mean.Sp.BodyMass)
sem_attendance_data$lMean.Sp.BodyMassXAbund <- log(sem_attendance_data$Mean.Sp.BodyMassXAbund)
sem_attendance_data$lBrillouin.Index <- log(sem_attendance_data$Brillouin.Index)
sem_attendance_data$lMean.Jaccard.Dissim.P.A. <- log(sem_attendance_data$Mean.Jaccard.Dissim.P.A.)
sem_attendance_data$lGDP.Millions <- log(sem_attendance_data$GDP.Millions)
sem_attendance_data$lNat_Pop._WB2015 <- log(sem_attendance_data$Nat_Pop._WB2015)
sem_attendance_data$lX50km_Pop <- log(sem_attendance_data$X50km_Pop)
sem_attendance_data$lX10km_Pop <- log(sem_attendance_data$X10km_Pop)
sem_attendance_data$lMean.Raup.Crick <- log(sem_attendance_data$Mean.Raup.Crick)

sem_in_situ_data$linsitu <- log(sem_in_situ_data$insitu)
```

```
sem_in_situ_data$lAttendance <- log(sem_in_situ_data$Attendance)
sem_in_situ_data$lZoo.Area.ha <- log(sem_in_situ_data$Zoo.Area.ha)
sem_in_situ_data$lSp.Richness <- log(sem_in_situ_data$Sp.Richness)
sem_in_situ_data$lTotal.Animals <- log(sem_in_situ_data$Total.Animals)
sem_in_situ_data$lThreatened.Sp <- log(sem_in_situ_data$Threatened.Sp)
sem_in_situ_data$lProp.Threat.Sp <- log(sem_in_situ_data$Prop.Threat.Sp)
sem_in_situ_data$lProp.Threat.Abund <- log(sem_in_situ_data$Prop.Threat.Abund)
sem_in_situ_data$lMean.Sp.BodyMass <- log(sem_in_situ_data$Mean.Sp.BodyMass)
sem_in_situ_data$lMean.Sp.BodyMassXAbund <- log(sem_in_situ_data$Mean.Sp.BodyMassXAbund)
sem_in_situ_data$lBrillouin.Index <- log(sem_in_situ_data$Brillouin.Index)
sem_in_situ_data$lMean.Jaccard.Dissim.P.A. <- log(sem_in_situ_data$Mean.Jaccard.Dissim.P.A.)
sem_in_situ_data$lGDP.Millions <- log(sem_in_situ_data$GDP.Millions)
sem_in_situ_data$lNat_Pop._WB2015 <- log(sem_in_situ_data$Nat_Pop._WB2015)
sem_in_situ_data$lX50km_Pop <- log(sem_in_situ_data$X50km_Pop)
sem_in_situ_data$lX10km_Pop <- log(sem_in_situ_data$X10km_Pop)
sem_in_situ_data$lMean.Raup.Crick <- log(sem_in_situ_data$Mean.Raup.Crick)
```

We then mean center the data and express it in units of standard deviation.

```
# Mean Centering and Standardizing
sem_attendance_data$Attendance2 <- mean_center(sem_attendance_data$lAttendance)
sem_attendance_data$Zoo.Area.ha2 <- mean_center(sem_attendance_data$lZoo.Area.ha)
sem_attendance_data$Sp.Richness2 <- mean_center(sem_attendance_data$lSp.Richness)
sem_attendance_data$Total.Animals2 <- mean_center(sem_attendance_data$lTotal.Animals)
sem_attendance_data$Mam.Sp.Richness2 <- mean_center(sem_attendance_data$Mam.Sp.Richness)
sem_attendance_data$Prop.Mam.Sp2 <- mean_center(sem_attendance_data$Prop.Mam.Sp)
sem_attendance_data$Prop.Mam.Abdun2 <- mean_center(sem_attendance_data$Prop.Mam.Abdun)
sem_attendance_data$Threatened.Sp2 <- mean_center(sem_attendance_data$lThreatened.Sp)
sem_attendance_data$Prop.Threat.Sp2 <- mean_center(sem_attendance_data$lProp.Threat.Sp)
sem_attendance_data$Prop.Threat.Abund2 <- mean_center(sem_attendance_data$lProp.Threat.Abund)
sem_attendance_data$Mean.Sp.BodyMass2 <- mean_center(sem_attendance_data$lMean.Sp.BodyMass)
sem_attendance_data$Mean.Sp.BodyMassXAbund2 <- mean_center(sem_attendance_data$lMean.Sp.BodyMassXAbund)
sem_attendance_data$Brillouin.Index2 <- mean_center(sem_attendance_data$lBrillouin.Index)
sem_attendance_data$Mean.Jaccard.Dissim.P.A.2 <- mean_center(sem_attendance_data$lMean.Jaccard.Dissim.P
sem_attendance_data$GDP.Millions2 <- mean_center(sem_attendance_data$lGDP.Millions)
sem_attendance_data$Nat_Pop._WB20152 <- mean_center(sem_attendance_data$lNat_Pop._WB2015)
sem_attendance_data$X50km_Pop2 <- mean_center(sem_attendance_data$lX50km_Pop)
sem_attendance_data$X10km_Pop2 <- mean_center(sem_attendance_data$lX10km_Pop)
sem_attendance_data$Mean.Raup.Crick2 <- mean_center(sem_attendance_data$lMean.Raup.Crick)


sem_in_situ_data$insitu2 <- mean_center(sem_in_situ_data$linsitu)
sem_in_situ_data$Attendance2 <- mean_center(sem_in_situ_data$lAttendance)
sem_in_situ_data$Zoo.Area.ha2 <- mean_center(sem_in_situ_data$lZoo.Area.ha)
sem_in_situ_data$Sp.Richness2 <- mean_center(sem_in_situ_data$lSp.Richness)
sem_in_situ_data$Total.Animals2 <- mean_center(sem_in_situ_data$lTotal.Animals)
sem_in_situ_data$Mam.Sp.Richness2 <- mean_center(sem_in_situ_data$Mam.Sp.Richness)
sem_in_situ_data$Prop.Mam.Sp2 <- mean_center(sem_in_situ_data$Prop.Mam.Sp)
sem_in_situ_data$Prop.Mam.Abdun2 <- mean_center(sem_in_situ_data$Prop.Mam.Abdun)
sem_in_situ_data$Threatened.Sp2 <- mean_center(sem_in_situ_data$lThreatened.Sp)
sem_in_situ_data$Prop.Threat.Sp2 <- mean_center(sem_in_situ_data$lProp.Threat.Sp)
sem_in_situ_data$Prop.Threat.Abund2 <- mean_center(sem_in_situ_data$lProp.Threat.Abund)
sem_in_situ_data$Mean.Sp.BodyMass2 <- mean_center(sem_in_situ_data$lMean.Sp.BodyMass)
sem_in_situ_data$Mean.Sp.BodyMassXAbund2 <- mean_center(sem_in_situ_data$lMean.Sp.BodyMassXAbund)
```

```
sem_in_situ_data$Brillouin.Index2 <- mean_center(sem_in_situ_data$lBrillouin.Index)
sem_in_situ_data$Mean.Jaccard.Dissim.P.A.2 <- mean_center(sem_in_situ_data$lMean.Jaccard.Dissim.P.A.)
sem_in_situ_data$GDP.Millions2 <- mean_center(sem_in_situ_data$lGDP.Millions)
sem_in_situ_data$Nat_Pop._WB20152 <- mean_center(sem_in_situ_data$lNat_Pop._WB2015)
sem_in_situ_data$X50km_Pop2 <- mean_center(sem_in_situ_data$lX50km_Pop)
sem_in_situ_data$X10km_Pop2 <- mean_center(sem_in_situ_data$lX10km_Pop)
sem_in_situ_data$Mean.Raup.Crick2<- mean_center(sem_in_situ_data$lMean.Raup.Crick)
```

Attendance SEM

Contents
Two distinct SEM frameworks were tested, the Attendance model and the In Situ model. The Attendance model tested the relationship between visitor attendance and all the various specified variables for 458 institutions globally. This model did not include any in situ contribution data. The In Situ model tested the relationship between visitor attendance, in situ contributions and all the various specified variables for a subset of 119 institutions in North America for which in situ contribution data was available. The results of the Attendance model were used to guide the development of the Attendance linked pathways in the In Situ model as the larger sample size of the Attendance model had higher power.

Two models were developed, one based on species presence-absence per institution and the the other which also conisdered species' population sizes per institution. This was important for calculating mean species body mass per institution, the proportion of threatened species per institution etc. First we develop the model based on species presence-absence.

Species Presence - Absence

Model 1 (a priori meta-model)

We now define our starting model for the SEM based on the a priori meta-model which is constructed based on evidence from the litrature and combined with proposed causal hypotheses. This model includes all previously identified evidence-based relationships and the numerous proposed causal pathways to visitor attendance. The development of this model can be found in the Supplementary information.

```
# Attendance SEM (Presence-Absence)

# Model 1
# Based on a priori meta-model and proposed causal pathways

mod.1 <- 'Attendance2 ~ Zoo.Area.ha2 + Sp.Richness2 + Total.Animals2
+ Mam.Sp.Richness2 + Prop.Mam.Sp2 + Prop.Threat.Sp2
+ Mean.Sp.BodyMass2 + Brillouin.Index2 + Mean.Raup.Crick2
+ X50km_Pop2 + X10km_Pop2 + GDP.Millions2 + Nat_Pop._WB20152

Total.Animals2 ~ Zoo.Area.ha2 + Sp.Richness2
Sp.Richness2 ~ Zoo.Area.ha2 + Prop.Mam.Sp2
Prop.Threat.Sp2 ~ Brillouin.Index2
Brillouin.Index2 ~ Sp.Richness2 + Total.Animals2
Mean.Raup.Crick2 ~ Sp.Richness2 + Total.Animals2'
```

We then fit the model, generate the model summary and generate a selection of absolute fit indices (e.g. Standardized Root Mean Square Residual) and incremental fit indices (e.g. Comparative Fit Index), to account

for the differential sensitivity of fit indices to data distribution, model size and sample size (Hu & Bentler, 1999). We then generate modification indices to identify pathways for inclusion, with a standard cut-off level for the chi-square test criterion of 3.84 (Burnham and Anderson, 2002).

Given the nested nature of the data (institutions within countries), we then adjust the model design to take this into considerdation. This is done using the lavaan.survey package. Once again, the model summary, fit indices and modification indices were all generated. A warning is produced by lavaan about model identifiability, most likely due to over-fitting, co-linearity and too few institutions within countries (in the nested design). We proceed with the model fitting noting this warning and as the model is refined this warning does not occur in the final set of models tested.

```
# Fit model and generate model summary
mod.1.fit <- sem(mod.1, data = sem_attendance_data, fixed.x=FALSE)
summary(mod.1.fit, rsq = TRUE)
```

```
## lavaan (0.5-23.1097) converged normally after  50 iterations
##
##   Number of observations                           458
##
##   Estimator                                         ML
##   Minimum Function Test Statistic             1225.956
##   Degrees of freedom                                41
##   P-value (Chi-square)                           0.000
##
## Parameter Estimates:
##
##   Information                                 Expected
##   Standard Errors                             Standard
##
## Regressions:
##                      Estimate  Std.Err  z-value  P(>|z|)
##   Attendance2 ~
##     Zoo.Area.ha2        0.086    0.035    2.480    0.013
##     Sp.Richness2       -0.249    0.074   -3.374    0.001
##     Total.Animals2      0.454    0.054    8.353    0.000
##     Mam.Sp.Rchnss2      0.132    0.029    4.591    0.000
##     Prop.Mam.Sp2       -0.065    0.038   -1.732    0.083
##     Prop.Thret.Sp2      0.009    0.024    0.371    0.711
##     Men.Sp.BdyMss2      0.339    0.037    9.277    0.000
##     Brilloun.Indx2      0.077    0.045    1.716    0.086
##     Mean.Rap.Crck2      0.146    0.025    5.822    0.000
##     X50km_Pop2          0.079    0.038    2.096    0.036
##     X10km_Pop2          0.397    0.041    9.624    0.000
##     GDP.Millions2       0.259    0.047    5.473    0.000
##     Nt_Pp._WB20152     -0.124    0.049   -2.523    0.012
##   Total.Animals2 ~
##     Zoo.Area.ha2        0.223    0.024    9.384    0.000
##     Sp.Richness2        0.798    0.024   33.520    0.000
##   Sp.Richness2 ~
##     Zoo.Area.ha2        0.363    0.039    9.240    0.000
##     Prop.Mam.Sp2       -0.607    0.039  -15.463    0.000
##   Prop.Threat.Sp2 ~
##     Brilloun.Indx2     -0.084    0.047   -1.795    0.073
##   Brillouin.Index2 ~
##     Sp.Richness2        1.232    0.046   26.770    0.000
```

```
##     Total.Animals2     -0.527    0.046  -11.451    0.000
##   Mean.Raup.Crick2 ~
##     Sp.Richness2       -0.135    0.083   -1.636    0.102
##     Total.Animals2      0.355    0.083    4.302    0.000
##
## Covariances:
##                        Estimate  Std.Err  z-value  P(>|z|)
##   Zoo.Area.ha2 ~~
##     Mam.Sp.Rchnss2        0.381    0.050    7.634    0.000
##     Prop.Mam.Sp2          0.312    0.049    6.389    0.000
##     Men.Sp.BdyMss2        0.534    0.053   10.096    0.000
##     X50km_Pop2            0.060    0.047    1.291    0.197
##     X10km_Pop2           -0.010    0.047   -0.208    0.835
##     GDP.Millions2        -0.027    0.047   -0.588    0.557
##     Nt_Pp._WB20152        0.061    0.047    1.310    0.190
##   Mam.Sp.Richness2 ~~
##     Prop.Mam.Sp2          0.088    0.047    1.882    0.060
##     Men.Sp.BdyMss2        0.303    0.049    6.216    0.000
##     X50km_Pop2            0.204    0.048    4.281    0.000
##     X10km_Pop2            0.284    0.048    5.852    0.000
##     GDP.Millions2        -0.060    0.047   -1.277    0.202
##     Nt_Pp._WB20152       -0.088    0.047   -1.881    0.060
##   Prop.Mam.Sp2 ~~
##     Men.Sp.BdyMss2        0.604    0.055   11.083    0.000
##     X50km_Pop2           -0.096    0.047   -2.054    0.040
##     X10km_Pop2           -0.204    0.048   -4.293    0.000
##     GDP.Millions2        -0.123    0.047   -2.615    0.009
##     Nt_Pp._WB20152       -0.115    0.047   -2.447    0.014
##   Mean.Sp.BodyMass2 ~~
##     X50km_Pop2            0.101    0.047    2.166    0.030
##     X10km_Pop2            0.126    0.047    2.683    0.007
##     GDP.Millions2        -0.082    0.047   -1.748    0.080
##     Nt_Pp._WB20152        0.006    0.047    0.124    0.901
##   X50km_Pop2 ~~
##     X10km_Pop2            0.752    0.058   12.886    0.000
##     GDP.Millions2        -0.013    0.047   -0.281    0.779
##     Nt_Pp._WB20152        0.191    0.047    4.033    0.000
##   X10km_Pop2 ~~
##     GDP.Millions2        -0.028    0.047   -0.604    0.546
##     Nt_Pp._WB20152        0.173    0.047    3.646    0.000
##   GDP.Millions2 ~~
##     Nt_Pp._WB20152        0.826    0.061   13.642    0.000
##
## Variances:
##                    Estimate  Std.Err  z-value  P(>|z|)
##    .Attendance2       0.267    0.018   15.133    0.000
##    .Total.Animals2    0.251    0.017   15.133    0.000
##    .Sp.Richness2      0.636    0.042   15.133    0.000
##    .Prop.Thret.Sp2    0.991    0.065   15.133    0.000
##    .Brilloun.Indx2    0.290    0.019   15.133    0.000
##    .Mean.Rap.Crck2    0.934    0.062   15.133    0.000
##     Zoo.Area.ha2      0.998    0.066   15.133    0.000
##     Mam.Sp.Rchnss2    0.998    0.066   15.133    0.000
##     Prop.Mam.Sp2      0.998    0.066   15.133    0.000
```

```
##     Men.Sp.BdyMss2    0.998    0.066   15.133    0.000
##     X50km_Pop2        0.998    0.066   15.133    0.000
##     X10km_Pop2        0.998    0.066   15.133    0.000
##     GDP.Millions2     0.998    0.066   15.133    0.000
##     Nt_Pp._WB20152    0.998    0.066   15.133    0.000
##
## R-Square:
##                    Estimate
##     Attendance2       0.717
##     Total.Animals2    0.748
##     Sp.Richness2      0.363
##     Prop.Thret.Sp2    0.007
##     Brilloun.Indx2    0.709
##     Mean.Rap.Crck2    0.064
```

```
# Generate fit indices
fitMeasures(mod.1.fit, c("agfi", "rmr", "srmr", "rmsea", "cfi", "nnfi", "tli"))
```

```
##  agfi   rmr  srmr rmsea   cfi  nnfi   tli
## 0.463 0.149 0.150 0.251 0.630 0.432 0.432
```

```
# Generate modification indices
mi1 <- modindices(mod.1.fit)
print(mi1[mi1$mi > 3.0,])
```

```
##                  lhs op               rhs      mi    epc sepc.lv sepc.all
## 71    Total.Animals2 ~~   Prop.Threat.Sp2  17.524  0.101   0.101    0.101
## 73    Total.Animals2 ~~  Mean.Raup.Crick2  11.525  0.191   0.191    0.192
## 74      Sp.Richness2 ~~   Prop.Threat.Sp2  27.971  0.257   0.257    0.258
## 75      Sp.Richness2 ~~  Brillouin.Index2   5.179  0.076   0.076    0.076
## 76      Sp.Richness2 ~~  Mean.Raup.Crick2  78.108 -0.531  -0.531   -0.532
## 77   Prop.Threat.Sp2 ~~  Brillouin.Index2   4.221  0.061   0.061    0.061
## 78   Prop.Threat.Sp2 ~~  Mean.Raup.Crick2  38.195 -0.278  -0.278   -0.278
## 81    Total.Animals2  ~   Prop.Threat.Sp2  17.776  0.103   0.103    0.103
## 83    Total.Animals2  ~  Mean.Raup.Crick2  11.525  0.205   0.205    0.205
## 87    Total.Animals2  ~        X50km_Pop2  34.446  0.138   0.138    0.138
## 88    Total.Animals2  ~        X10km_Pop2   7.972  0.067   0.067    0.067
## 89    Total.Animals2  ~     GDP.Millions2  30.032 -0.129  -0.129   -0.129
## 90    Total.Animals2  ~  Nat_Pop._WB20152   8.055 -0.067  -0.067   -0.067
## 91      Sp.Richness2  ~       Attendance2  61.165  0.457   0.457    0.444
## 93      Sp.Richness2  ~   Prop.Threat.Sp2  26.993  0.255   0.255    0.255
## 94      Sp.Richness2  ~  Brillouin.Index2   5.103  0.235   0.235    0.235
## 95      Sp.Richness2  ~  Mean.Raup.Crick2  77.140 -0.556  -0.556   -0.556
## 96      Sp.Richness2  ~  Mam.Sp.Richness2 349.341  0.755   0.755    0.755
## 97      Sp.Richness2  ~ Mean.Sp.BodyMass2   7.496  0.144   0.144    0.144
## 98      Sp.Richness2  ~        X50km_Pop2  13.582  0.139   0.139    0.139
## 99      Sp.Richness2  ~        X10km_Pop2  32.091  0.216   0.216    0.216
## 101     Sp.Richness2  ~  Nat_Pop._WB20152  10.411 -0.122  -0.122   -0.122
## 102  Prop.Threat.Sp2  ~       Attendance2  39.370  0.360   0.360    0.350
## 103  Prop.Threat.Sp2  ~    Total.Animals2  14.140  0.203   0.203    0.203
## 105  Prop.Threat.Sp2  ~  Mean.Raup.Crick2  24.245 -0.230  -0.230   -0.230
## 106  Prop.Threat.Sp2  ~      Zoo.Area.ha2  10.359  0.150   0.150    0.150
## 107  Prop.Threat.Sp2  ~  Mam.Sp.Richness2  21.403  0.215   0.215    0.215
## 108  Prop.Threat.Sp2  ~      Prop.Mam.Sp2  47.215  0.356   0.356    0.356
## 109  Prop.Threat.Sp2  ~ Mean.Sp.BodyMass2  47.052  0.326   0.326    0.326
## 110  Prop.Threat.Sp2  ~        X50km_Pop2  19.224  0.205   0.205    0.205
```

```
## 111   Prop.Threat.Sp2  ~        X10km_Pop2  16.562  0.190   0.190    0.190
## 114  Brillouin.Index2  ~       Attendance2   5.954 -0.090  -0.090   -0.088
## 115  Brillouin.Index2  ~   Prop.Threat.Sp2   4.221  0.062   0.062    0.062
## 119  Brillouin.Index2  ~      Prop.Mam.Sp2   9.791  0.092   0.092    0.092
## 122  Brillouin.Index2  ~        X10km_Pop2   3.677 -0.049  -0.049   -0.049
## 123  Brillouin.Index2  ~     GDP.Millions2  58.745 -0.194  -0.194   -0.194
## 124  Brillouin.Index2  ~  Nat_Pop._WB20152  51.006 -0.181  -0.181   -0.181
## 125  Mean.Raup.Crick2  ~       Attendance2  20.322 -0.304  -0.304   -0.296
## 126  Mean.Raup.Crick2  ~   Prop.Threat.Sp2  37.487 -0.277  -0.277   -0.277
## 128  Mean.Raup.Crick2  ~      Zoo.Area.ha2  11.525 -0.170  -0.170   -0.170
## 129  Mean.Raup.Crick2  ~  Mam.Sp.Richness2  18.995 -0.200  -0.200   -0.200
## 130  Mean.Raup.Crick2  ~      Prop.Mam.Sp2 106.001 -0.545  -0.545   -0.545
## 131  Mean.Raup.Crick2  ~ Mean.Sp.BodyMass2 115.574 -0.508  -0.508   -0.508
## 132  Mean.Raup.Crick2  ~        X50km_Pop2   6.052  0.112   0.112    0.112
## 133  Mean.Raup.Crick2  ~        X10km_Pop2   4.241  0.094   0.094    0.094
## 135  Mean.Raup.Crick2  ~  Nat_Pop._WB20152  10.765  0.149   0.149    0.149
## 136      Zoo.Area.ha2  ~       Attendance2   4.462 -0.354  -0.354   -0.344
## 137      Zoo.Area.ha2  ~    Total.Animals2  55.766 -0.565  -0.565   -0.565
## 138      Zoo.Area.ha2  ~      Sp.Richness2  89.386 -0.744  -0.744   -0.744
## 140      Zoo.Area.ha2  ~  Brillouin.Index2  21.333 -0.262  -0.262   -0.262
## 141      Zoo.Area.ha2  ~  Mean.Raup.Crick2   6.059  0.100   0.100    0.100
## 149  Mam.Sp.Richness2  ~       Attendance2  43.855  0.817   0.817    0.795
## 150  Mam.Sp.Richness2  ~    Total.Animals2 152.463  0.597   0.597    0.597
## 151  Mam.Sp.Richness2  ~      Sp.Richness2 267.086  0.802   0.802    0.802
## 152  Mam.Sp.Richness2  ~   Prop.Threat.Sp2   3.131  0.069   0.069    0.069
## 153  Mam.Sp.Richness2  ~  Brillouin.Index2 149.822  0.543   0.543    0.543
## 165      Prop.Mam.Sp2  ~   Prop.Threat.Sp2  17.634  0.154   0.154    0.154
## 166      Prop.Mam.Sp2  ~  Brillouin.Index2   3.285  0.092   0.092    0.092
## 167      Prop.Mam.Sp2  ~  Mean.Raup.Crick2   5.483 -0.091  -0.091   -0.091
## 175 Mean.Sp.BodyMass2  ~       Attendance2  11.172 -0.325  -0.325   -0.316
## 178 Mean.Sp.BodyMass2  ~   Prop.Threat.Sp2   3.152  0.055   0.055    0.055
## 180 Mean.Sp.BodyMass2  ~  Mean.Raup.Crick2  48.092 -0.216  -0.216   -0.216
## 188        X50km_Pop2  ~       Attendance2  23.854  0.460   0.460    0.448
## 189        X50km_Pop2  ~    Total.Animals2  14.934  0.143   0.143    0.143
## 214     GDP.Millions2  ~       Attendance2  11.864 -0.259  -0.259   -0.252
## 216     GDP.Millions2  ~      Sp.Richness2   7.200  0.080   0.080    0.080
## 219     GDP.Millions2  ~  Mean.Raup.Crick2  13.482 -0.088  -0.088   -0.088
## 229  Nat_Pop._WB20152  ~      Sp.Richness2   7.489 -0.079  -0.079   -0.079
## 230  Nat_Pop._WB20152  ~   Prop.Threat.Sp2   3.595  0.044   0.044    0.044
## 231  Nat_Pop._WB20152  ~  Brillouin.Index2   9.150 -0.079  -0.079   -0.079
## 232  Nat_Pop._WB20152  ~  Mean.Raup.Crick2  12.502  0.082   0.082    0.082
##     sepc.nox
## 71     0.101
## 73     0.192
## 74     0.258
## 75     0.076
## 76    -0.532
## 77     0.061
## 78    -0.278
## 81     0.103
## 83     0.205
## 87     0.138
## 88     0.067
## 89    -0.129
```

```
## 90    -0.067
## 91     0.444
## 93     0.255
## 94     0.235
## 95    -0.556
## 96     0.756
## 97     0.144
## 98     0.139
## 99     0.217
## 101   -0.122
## 102    0.350
## 103    0.203
## 105   -0.230
## 106    0.150
## 107    0.216
## 108    0.356
## 109    0.327
## 110    0.205
## 111    0.191
## 114   -0.088
## 115    0.062
## 119    0.092
## 122   -0.049
## 123   -0.194
## 124   -0.181
## 125   -0.296
## 126   -0.277
## 128   -0.170
## 129   -0.200
## 130   -0.545
## 131   -0.508
## 132    0.112
## 133    0.094
## 135    0.149
## 136   -0.344
## 137   -0.565
## 138   -0.744
## 140   -0.262
## 141    0.100
## 149    0.795
## 150    0.597
## 151    0.802
## 152    0.069
## 153    0.543
## 165    0.154
## 166    0.092
## 167   -0.091
## 175   -0.316
## 178    0.055
## 180   -0.216
## 188    0.448
## 189    0.143
## 214   -0.252
## 216    0.080
```

```
## 219   -0.088
## 229   -0.079
## 230    0.044
## 231   -0.079
## 232    0.082
```

```
# Adjust for the nested nature of the data (institutions within countries)
# Fit model and generate model summary
design <- svydesign(ids = ~Country, nest=TRUE, data=sem_attendance_data)
fit.adj1 <- lavaan.survey(lavaan.fit = mod.1.fit, survey.design = design)
summary(fit.adj1, rsq = TRUE)
```

```
## lavaan (0.5-23.1097) converged normally after  49 iterations
##
##   Number of observations                           458
##
##   Estimator                                         ML      Robust
##   Minimum Function Test Statistic             1225.956     268.831
##   Degrees of freedom                                41          41
##   P-value (Chi-square)                           0.000       0.000
##   Scaling correction factor                                  4.560
##     for the Satorra-Bentler correction
##
## Parameter Estimates:
##
##   Information                                 Expected
##   Standard Errors                           Robust.sem
##
## Regressions:
##                    Estimate  Std.Err  z-value  P(>|z|)
##   Attendance2 ~
##     Zoo.Area.ha2      0.086    0.044    1.962    0.050
##     Sp.Richness2     -0.249    0.077   -3.230    0.001
##     Total.Animals2    0.454    0.051    8.995    0.000
##     Mam.Sp.Rchnss2    0.132    0.026    5.183    0.000
##     Prop.Mam.Sp2     -0.065    0.035   -1.883    0.060
##     Prop.Thret.Sp2    0.009    0.024    0.371    0.710
##     Men.Sp.BdyMss2    0.339    0.029   11.577    0.000
##     Brilloun.Indx2    0.077    0.054    1.414    0.157
##     Mean.Rap.Crck2    0.146    0.027    5.343    0.000
##     X50km_Pop2        0.079    0.034    2.362    0.018
##     X10km_Pop2        0.397    0.042    9.352    0.000
##     GDP.Millions2     0.259    0.059    4.419    0.000
##     Nt_Pp._WB20152   -0.124    0.067   -1.845    0.065
##   Total.Animals2 ~
##     Zoo.Area.ha2      0.223    0.038    5.856    0.000
##     Sp.Richness2      0.798    0.048   16.678    0.000
##   Sp.Richness2 ~
##     Zoo.Area.ha2      0.363    0.080    4.545    0.000
##     Prop.Mam.Sp2     -0.607    0.062   -9.853    0.000
##   Prop.Threat.Sp2 ~
##     Brilloun.Indx2   -0.084    0.101   -0.825    0.409
##   Brillouin.Index2 ~
##     Sp.Richness2      1.232    0.068   18.134    0.000
##     Total.Animals2   -0.527    0.078   -6.780    0.000
```

```
##   Mean.Raup.Crick2 ~
##     Sp.Richness2      -0.135    0.215   -0.626    0.531
##     Total.Animals2     0.355    0.156    2.277    0.023
##
## Covariances:
##                       Estimate  Std.Err  z-value  P(>|z|)
##   Zoo.Area.ha2 ~~
##     Mam.Sp.Rchnss2       0.381    0.064    5.985    0.000
##     Prop.Mam.Sp2         0.312    0.089    3.510    0.000
##     Men.Sp.BdyMss2       0.534    0.104    5.147    0.000
##     X50km_Pop2           0.060    0.069    0.880    0.379
##     X10km_Pop2          -0.010    0.078   -0.124    0.901
##     GDP.Millions2       -0.027    0.041   -0.673    0.501
##     Nt_Pp._WB20152       0.061    0.090    0.682    0.495
##   Mam.Sp.Richness2 ~~
##     Prop.Mam.Sp2         0.088    0.061    1.435    0.151
##     Men.Sp.BdyMss2       0.303    0.081    3.730    0.000
##     X50km_Pop2           0.204    0.082    2.487    0.013
##     X10km_Pop2           0.284    0.066    4.278    0.000
##     GDP.Millions2       -0.060    0.064   -0.937    0.349
##     Nt_Pp._WB20152      -0.088    0.102   -0.863    0.388
##   Prop.Mam.Sp2 ~~
##     Men.Sp.BdyMss2       0.604    0.130    4.656    0.000
##     X50km_Pop2          -0.096    0.051   -1.888    0.059
##     X10km_Pop2          -0.204    0.055   -3.685    0.000
##     GDP.Millions2       -0.123    0.105   -1.172    0.241
##     Nt_Pp._WB20152      -0.115    0.091   -1.257    0.209
##   Mean.Sp.BodyMass2 ~~
##     X50km_Pop2           0.101    0.072    1.400    0.161
##     X10km_Pop2           0.126    0.067    1.895    0.058
##     GDP.Millions2       -0.082    0.046   -1.759    0.079
##     Nt_Pp._WB20152       0.006    0.092    0.063    0.950
##   X50km_Pop2 ~~
##     X10km_Pop2           0.752    0.126    5.982    0.000
##     GDP.Millions2       -0.013    0.100   -0.131    0.896
##     Nt_Pp._WB20152       0.191    0.177    1.083    0.279
##   X10km_Pop2 ~~
##     GDP.Millions2       -0.028    0.056   -0.505    0.613
##     Nt_Pp._WB20152       0.173    0.101    1.701    0.089
##   GDP.Millions2 ~~
##     Nt_Pp._WB20152       0.826    0.202    4.089    0.000
##
## Intercepts:
##                   Estimate  Std.Err  z-value  P(>|z|)
##    .Attendance2     -0.000    0.031   -0.000    1.000
##    .Total.Animals2  -0.000    0.104   -0.000    1.000
##    .Sp.Richness2     0.000    0.090    0.000    1.000
##    .Prop.Thret.Sp2   0.000    0.099    0.000    1.000
##    .Brilloun.Indx2  -0.000    0.106   -0.000    1.000
##    .Mean.Rap.Crck2   0.000    0.092    0.000    1.000
##     Zoo.Area.ha2     0.000    0.064    0.000    1.000
##     Mam.Sp.Rchnss2  -0.000    0.079   -0.000    1.000
##     Prop.Mam.Sp2     0.000    0.118    0.000    1.000
##     Men.Sp.BdyMss2  -0.000    0.080   -0.000    1.000
```

```
##     X50km_Pop2       -0.000    0.114   -0.000    1.000
##     X10km_Pop2        0.000    0.105    0.000    1.000
##     GDP.Millions2    -0.000    0.345   -0.000    1.000
##     Nt_Pp._WB20152    0.000    0.295    0.000    1.000
##
## Variances:
##                    Estimate  Std.Err  z-value  P(>|z|)
##    .Attendance2       0.267    0.028    9.566    0.000
##    .Total.Animals2    0.251    0.044    5.729    0.000
##    .Sp.Richness2      0.636    0.144    4.407    0.000
##    .Prop.Thret.Sp2    0.991    0.135    7.335    0.000
##    .Brilloun.Indx2    0.290    0.079    3.656    0.000
##    .Mean.Rap.Crck2    0.934    0.100    9.311    0.000
##     Zoo.Area.ha2      0.998    0.112    8.886    0.000
##     Mam.Sp.Rchnss2    0.998    0.115    8.669    0.000
##     Prop.Mam.Sp2      0.998    0.150    6.662    0.000
##     Men.Sp.BdyMss2    0.998    0.173    5.783    0.000
##     X50km_Pop2        0.998    0.131    7.623    0.000
##     X10km_Pop2        0.998    0.174    5.745    0.000
##     GDP.Millions2     0.998    0.246    4.061    0.000
##     Nt_Pp._WB20152    0.998    0.225    4.439    0.000
##
## R-Square:
##                    Estimate
##     Attendance2       0.717
##     Total.Animals2    0.748
##     Sp.Richness2      0.363
##     Prop.Thret.Sp2    0.007
##     Brilloun.Indx2    0.709
##     Mean.Rap.Crck2    0.064
```

```r
# Generate fit indices
fitMeasures(fit.adj1, c("agfi", "rmr", "srmr", "rmsea", "cfi", "nnfi", "tli"))
```

```
##  agfi   rmr  srmr rmsea   cfi  nnfi   tli
## 0.392 0.149 0.140 0.251 0.630 0.432 0.432
```

```r
# Generate modification indices
mi1adj <- modindices(fit.adj1)
print(mi1adj[mi1adj$mi > 3.0,])
```

```
##                   lhs op              rhs     mi mi.scaled    epc
## 85     Total.Animals2 ~~   Prop.Threat.Sp2 17.524     3.843  0.101
## 87     Total.Animals2 ~~  Mean.Raup.Crick2 11.524     2.527  0.191
## 88       Sp.Richness2 ~~   Prop.Threat.Sp2 27.970     6.133  0.257
## 89       Sp.Richness2 ~~  Brillouin.Index2  5.178     1.136  0.076
## 90       Sp.Richness2 ~~  Mean.Raup.Crick2 78.107    17.127 -0.531
## 91    Prop.Threat.Sp2 ~~  Brillouin.Index2  4.221     0.926  0.061
## 92    Prop.Threat.Sp2 ~~  Mean.Raup.Crick2 38.195     8.375 -0.278
## 95     Total.Animals2  ~   Prop.Threat.Sp2 17.776     3.898  0.103
## 97     Total.Animals2  ~  Mean.Raup.Crick2 11.524     2.527  0.205
## 101    Total.Animals2  ~        X50km_Pop2 34.446     7.553  0.138
## 102    Total.Animals2  ~        X10km_Pop2  7.972     1.748  0.067
## 103    Total.Animals2  ~     GDP.Millions2 30.032     6.586 -0.129
## 104    Total.Animals2  ~  Nat_Pop._WB20152  8.055     1.766 -0.067
## 105      Sp.Richness2  ~       Attendance2 61.165    13.412  0.457
```

```
## 107      Sp.Richness2  ~   Prop.Threat.Sp2  26.993     5.919  0.255
## 108      Sp.Richness2  ~  Brillouin.Index2   5.102     1.119  0.235
## 109      Sp.Richness2  ~  Mean.Raup.Crick2  77.138    16.915 -0.556
## 110      Sp.Richness2  ~  Mam.Sp.Richness2 349.340    76.604  0.755
## 111      Sp.Richness2  ~ Mean.Sp.BodyMass2   7.496     1.644  0.144
## 112      Sp.Richness2  ~        X50km_Pop2  13.582     2.978  0.139
## 113      Sp.Richness2  ~        X10km_Pop2  32.091     7.037  0.216
## 115      Sp.Richness2  ~  Nat_Pop._WB20152  10.411     2.283 -0.122
## 116   Prop.Threat.Sp2  ~       Attendance2  39.369     8.633  0.360
## 117   Prop.Threat.Sp2  ~    Total.Animals2  14.140     3.101  0.203
## 119   Prop.Threat.Sp2  ~  Mean.Raup.Crick2  24.245     5.317 -0.230
## 120   Prop.Threat.Sp2  ~       Zoo.Area.ha2  10.359     2.272  0.150
## 121   Prop.Threat.Sp2  ~  Mam.Sp.Richness2  21.403     4.693  0.215
## 122   Prop.Threat.Sp2  ~      Prop.Mam.Sp2  47.215    10.353  0.356
## 123   Prop.Threat.Sp2  ~ Mean.Sp.BodyMass2  47.052    10.318  0.326
## 124   Prop.Threat.Sp2  ~        X50km_Pop2  19.224     4.215  0.205
## 125   Prop.Threat.Sp2  ~        X10km_Pop2  16.562     3.632  0.190
## 128  Brillouin.Index2  ~       Attendance2   5.955     1.306 -0.090
## 129  Brillouin.Index2  ~   Prop.Threat.Sp2   4.221     0.926  0.062
## 133  Brillouin.Index2  ~      Prop.Mam.Sp2   9.791     2.147  0.092
## 136  Brillouin.Index2  ~        X10km_Pop2   3.677     0.806 -0.049
## 137  Brillouin.Index2  ~     GDP.Millions2  58.746    12.882 -0.194
## 138  Brillouin.Index2  ~  Nat_Pop._WB20152  51.007    11.185 -0.181
## 139  Mean.Raup.Crick2  ~       Attendance2  20.322     4.456 -0.304
## 140  Mean.Raup.Crick2  ~   Prop.Threat.Sp2  37.487     8.220 -0.277
## 142  Mean.Raup.Crick2  ~       Zoo.Area.ha2  11.525     2.527 -0.170
## 143  Mean.Raup.Crick2  ~  Mam.Sp.Richness2  18.995     4.165 -0.200
## 144  Mean.Raup.Crick2  ~      Prop.Mam.Sp2 106.002    23.244 -0.545
## 145  Mean.Raup.Crick2  ~ Mean.Sp.BodyMass2 115.574    25.343 -0.508
## 146  Mean.Raup.Crick2  ~        X50km_Pop2   6.052     1.327  0.112
## 147  Mean.Raup.Crick2  ~        X10km_Pop2   4.241     0.930  0.094
## 149  Mean.Raup.Crick2  ~  Nat_Pop._WB20152  10.765     2.361  0.149
## 150      Zoo.Area.ha2  ~       Attendance2   4.461     0.978 -0.354
## 151      Zoo.Area.ha2  ~    Total.Animals2  55.767    12.229 -0.565
## 152      Zoo.Area.ha2  ~      Sp.Richness2  89.388    19.601 -0.744
## 154      Zoo.Area.ha2  ~  Brillouin.Index2  21.334     4.678 -0.262
## 155      Zoo.Area.ha2  ~  Mean.Raup.Crick2   6.059     1.329  0.100
## 163  Mam.Sp.Richness2  ~       Attendance2  43.856     9.617  0.817
## 164  Mam.Sp.Richness2  ~    Total.Animals2 152.464    33.433  0.597
## 165  Mam.Sp.Richness2  ~      Sp.Richness2 267.085    58.567  0.802
## 166  Mam.Sp.Richness2  ~   Prop.Threat.Sp2   3.131     0.687  0.069
## 167  Mam.Sp.Richness2  ~  Brillouin.Index2 149.822    32.853  0.543
## 179      Prop.Mam.Sp2  ~   Prop.Threat.Sp2  17.634     3.867  0.154
## 180      Prop.Mam.Sp2  ~  Brillouin.Index2   3.285     0.720  0.092
## 181      Prop.Mam.Sp2  ~  Mean.Raup.Crick2   5.483     1.202 -0.091
## 189 Mean.Sp.BodyMass2  ~       Attendance2  11.172     2.450 -0.325
## 192 Mean.Sp.BodyMass2  ~   Prop.Threat.Sp2   3.152     0.691  0.055
## 194 Mean.Sp.BodyMass2  ~  Mean.Raup.Crick2  48.091    10.546 -0.216
## 202        X50km_Pop2  ~       Attendance2  23.855     5.231  0.460
## 203        X50km_Pop2  ~    Total.Animals2  14.935     3.275  0.143
## 228     GDP.Millions2  ~       Attendance2  11.865     2.602 -0.259
## 230     GDP.Millions2  ~      Sp.Richness2   7.200     1.579  0.080
## 233     GDP.Millions2  ~  Mean.Raup.Crick2  13.482     2.956 -0.088
## 243  Nat_Pop._WB20152  ~      Sp.Richness2   7.489     1.642 -0.079
```

```
## 244  Nat_Pop._WB20152  ~   Prop.Threat.Sp2   3.595     0.788  0.044
## 245  Nat_Pop._WB20152  ~  Brillouin.Index2   9.150     2.006 -0.079
## 246  Nat_Pop._WB20152  ~  Mean.Raup.Crick2  12.502     2.741  0.082
##     sepc.lv sepc.all sepc.nox
## 85    0.101    0.101    0.101
## 87    0.191    0.192    0.192
## 88    0.257    0.258    0.258
## 89    0.076    0.076    0.076
## 90   -0.531   -0.532   -0.532
## 91    0.061    0.061    0.061
## 92   -0.278   -0.278   -0.278
## 95    0.103    0.103    0.103
## 97    0.205    0.205    0.205
## 101   0.138    0.138    0.138
## 102   0.067    0.067    0.067
## 103  -0.129   -0.129   -0.129
## 104  -0.067   -0.067   -0.067
## 105   0.457    0.444    0.444
## 107   0.255    0.255    0.255
## 108   0.235    0.235    0.235
## 109  -0.556   -0.556   -0.556
## 110   0.755    0.755    0.756
## 111   0.144    0.144    0.144
## 112   0.139    0.139    0.139
## 113   0.216    0.216    0.217
## 115  -0.122   -0.122   -0.122
## 116   0.360    0.350    0.350
## 117   0.203    0.203    0.203
## 119  -0.230   -0.230   -0.230
## 120   0.150    0.150    0.150
## 121   0.215    0.215    0.216
## 122   0.356    0.356    0.356
## 123   0.326    0.326    0.327
## 124   0.205    0.205    0.205
## 125   0.190    0.190    0.191
## 128  -0.090   -0.088   -0.088
## 129   0.062    0.062    0.062
## 133   0.092    0.092    0.092
## 136  -0.049   -0.049   -0.049
## 137  -0.194   -0.194   -0.194
## 138  -0.181   -0.181   -0.181
## 139  -0.304   -0.296   -0.296
## 140  -0.277   -0.277   -0.277
## 142  -0.170   -0.170   -0.170
## 143  -0.200   -0.200   -0.200
## 144  -0.545   -0.545   -0.545
## 145  -0.508   -0.508   -0.508
## 146   0.112    0.112    0.112
## 147   0.094    0.094    0.094
## 149   0.149    0.149    0.149
## 150  -0.354   -0.344   -0.344
## 151  -0.565   -0.565   -0.565
## 152  -0.744   -0.744   -0.744
## 154  -0.262   -0.262   -0.262
```

```
## 155   0.100    0.100    0.100
## 163   0.817    0.795    0.795
## 164   0.597    0.597    0.597
## 165   0.802    0.802    0.802
## 166   0.069    0.069    0.069
## 167   0.543    0.543    0.543
## 179   0.154    0.154    0.154
## 180   0.092    0.092    0.092
## 181  -0.091   -0.091   -0.091
## 189  -0.325   -0.316   -0.316
## 192   0.055    0.055    0.055
## 194  -0.216   -0.216   -0.216
## 202   0.460    0.448    0.448
## 203   0.143    0.143    0.143
## 228  -0.259   -0.252   -0.252
## 230   0.080    0.080    0.080
## 233  -0.088   -0.088   -0.088
## 243  -0.079   -0.079   -0.079
## 244   0.044    0.044    0.044
## 245  -0.079   -0.079   -0.079
## 246   0.082    0.082    0.082
```

Model 2

Based on the modification indices generated from the first model, we can see that **Sp.Richness2** ~ **Mam.Sp.Richness2** has the highest mi value of **349.340**. This far exceeds the standard cut-off level for the chi-square test criterion of 3.84 (Burnham and Anderson, 2002). This is also one of the most intuitive relationships, as if we increase mammal species richness, then of course we are going to increase overall species richness simulataneously. As a result, we add this relationship to our model. Once again, the model summary, fit indices and modification indices were all generated for the model, adjusting for the nested nature of data.

```
# Attendance SEM (Presence-Absence)

# Model 2
# Addition of Sp.Richness2 ~ Mam.Sp.Richness2, mi = 349.342

mod.2 <- 'Attendance2 ~ Zoo.Area.ha2 + Sp.Richness2 + Total.Animals2
+ Mam.Sp.Richness2 + Prop.Mam.Sp2 + Prop.Threat.Sp2
+ Mean.Sp.BodyMass2 + Brillouin.Index2 + Mean.Raup.Crick2
+ X50km_Pop2 + X10km_Pop2 + GDP.Millions2 + Nat_Pop._WB20152

Total.Animals2 ~ Zoo.Area.ha2 + Sp.Richness2
Sp.Richness2 ~ Zoo.Area.ha2 + Prop.Mam.Sp2 + Mam.Sp.Richness2
Prop.Threat.Sp2 ~ Brillouin.Index2
Brillouin.Index2 ~ Sp.Richness2 + Total.Animals2
Mean.Raup.Crick2 ~ Sp.Richness2 + Total.Animals2'

# Fit model and generate model summary
mod.2.fit <- sem(mod.2, data = sem_attendance_data, fixed.x=FALSE)
summary(mod.2.fit, rsq = TRUE)
```

```
## lavaan (0.5-23.1097) converged normally after  54 iterations
##
##   Number of observations                           458
```

```
##
##   Estimator                                         ML
##   Minimum Function Test Statistic              567.053
##   Degrees of freedom                                40
##   P-value (Chi-square)                           0.000
##
## Parameter Estimates:
##
##   Information                                 Expected
##   Standard Errors                             Standard
##
## Regressions:
##                      Estimate  Std.Err  z-value  P(>|z|)
##   Attendance2 ~
##     Zoo.Area.ha2        0.086    0.033    2.593    0.010
##     Sp.Richness2       -0.249    0.092   -2.719    0.007
##     Total.Animals2      0.454    0.054    8.353    0.000
##     Mam.Sp.Rchnss2      0.132    0.055    2.403    0.016
##     Prop.Mam.Sp2       -0.065    0.049   -1.337    0.181
##     Prop.Thret.Sp2      0.009    0.024    0.371    0.711
##     Men.Sp.BdyMss2      0.339    0.037    9.277    0.000
##     Brilloun.Indx2      0.077    0.045    1.716    0.086
##     Mean.Rap.Crck2      0.146    0.025    5.822    0.000
##     X50km_Pop2          0.079    0.038    2.096    0.036
##     X10km_Pop2          0.397    0.041    9.624    0.000
##     GDP.Millions2       0.259    0.047    5.473    0.000
##     Nt_Pp._WB20152     -0.124    0.049   -2.523    0.012
##   Total.Animals2 ~
##     Zoo.Area.ha2        0.223    0.024    9.384    0.000
##     Sp.Richness2        0.798    0.024   33.520    0.000
##   Sp.Richness2 ~
##     Zoo.Area.ha2        0.067    0.021    3.225    0.001
##     Prop.Mam.Sp2       -0.581    0.019  -30.364    0.000
##     Mam.Sp.Rchnss2      0.755    0.020   38.373    0.000
##   Prop.Threat.Sp2 ~
##     Brilloun.Indx2     -0.084    0.047   -1.795    0.073
##   Brillouin.Index2 ~
##     Sp.Richness2        1.232    0.046   26.770    0.000
##     Total.Animals2     -0.527    0.046  -11.451    0.000
##   Mean.Raup.Crick2 ~
##     Sp.Richness2       -0.135    0.083   -1.636    0.102
##     Total.Animals2      0.355    0.083    4.302    0.000
##
## Covariances:
##                        Estimate  Std.Err  z-value  P(>|z|)
##   Zoo.Area.ha2 ~~
##     Mam.Sp.Rchnss2        0.381    0.050    7.634    0.000
##     Prop.Mam.Sp2          0.312    0.049    6.389    0.000
##     Men.Sp.BdyMss2        0.534    0.053   10.096    0.000
##     X50km_Pop2            0.060    0.047    1.291    0.197
##     X10km_Pop2           -0.010    0.047   -0.208    0.835
##     GDP.Millions2        -0.027    0.047   -0.588    0.557
##     Nt_Pp._WB20152        0.061    0.047    1.310    0.190
##   Mam.Sp.Richness2 ~~
```

```
##     Prop.Mam.Sp2         0.088    0.047    1.882    0.060
##     Men.Sp.BdyMss2       0.303    0.049    6.216    0.000
##     X50km_Pop2           0.204    0.048    4.281    0.000
##     X10km_Pop2           0.284    0.048    5.852    0.000
##     GDP.Millions2       -0.060    0.047   -1.277    0.202
##     Nt_Pp._WB20152      -0.088    0.047   -1.881    0.060
##   Prop.Mam.Sp2 ~~
##     Men.Sp.BdyMss2       0.604    0.055   11.083    0.000
##     X50km_Pop2          -0.096    0.047   -2.054    0.040
##     X10km_Pop2          -0.204    0.048   -4.293    0.000
##     GDP.Millions2       -0.123    0.047   -2.615    0.009
##     Nt_Pp._WB20152      -0.115    0.047   -2.447    0.014
##   Mean.Sp.BodyMass2 ~~
##     X50km_Pop2           0.101    0.047    2.166    0.030
##     X10km_Pop2           0.126    0.047    2.683    0.007
##     GDP.Millions2       -0.082    0.047   -1.748    0.080
##     Nt_Pp._WB20152       0.006    0.047    0.124    0.901
##   X50km_Pop2 ~~
##     X10km_Pop2           0.752    0.058   12.886    0.000
##     GDP.Millions2       -0.013    0.047   -0.281    0.779
##     Nt_Pp._WB20152       0.191    0.047    4.033    0.000
##   X10km_Pop2 ~~
##     GDP.Millions2       -0.028    0.047   -0.604    0.546
##     Nt_Pp._WB20152       0.173    0.047    3.646    0.000
##   GDP.Millions2 ~~
##     Nt_Pp._WB20152       0.826    0.061   13.642    0.000
##
## Variances:
##                    Estimate  Std.Err  z-value  P(>|z|)
##    .Attendance2       0.267    0.018   15.133    0.000
##    .Total.Animals2    0.251    0.017   15.133    0.000
##    .Sp.Richness2      0.151    0.010   15.133    0.000
##    .Prop.Thret.Sp2    0.991    0.065   15.133    0.000
##    .Brilloun.Indx2    0.290    0.019   15.133    0.000
##    .Mean.Rap.Crck2    0.934    0.062   15.133    0.000
##     Zoo.Area.ha2      0.998    0.066   15.133    0.000
##     Mam.Sp.Rchnss2    0.998    0.066   15.133    0.000
##     Prop.Mam.Sp2      0.998    0.066   15.133    0.000
##     Men.Sp.BdyMss2    0.998    0.066   15.133    0.000
##     X50km_Pop2        0.998    0.066   15.133    0.000
##     X10km_Pop2        0.998    0.066   15.133    0.000
##     GDP.Millions2     0.998    0.066   15.133    0.000
##     Nt_Pp._WB20152    0.998    0.066   15.133    0.000
##
## R-Square:
##                    Estimate
##     Attendance2       0.740
##     Total.Animals2    0.748
##     Sp.Richness2      0.849
##     Prop.Thret.Sp2    0.007
##     Brilloun.Indx2    0.709
##     Mean.Rap.Crck2    0.064
```

```r
# Generate fit indices
fitMeasures(mod.2.fit, c("agfi", "rmr", "srmr", "rmsea", "cfi", "nnfi", "tli"))
```

```
##  agfi   rmr  srmr rmsea   cfi  nnfi   tli
## 0.640 0.102 0.103 0.170 0.835 0.741 0.741
```

```r
# Generate modification indices
mi2 <- modindices(mod.2.fit)
print(mi2[mi2$mi > 3.0,])
```

```
##                   lhs op                rhs      mi    epc sepc.lv sepc.all
## 72     Total.Animals2 ~~   Prop.Threat.Sp2  17.524  0.101   0.101    0.101
## 74     Total.Animals2 ~~  Mean.Raup.Crick2  11.525  0.191   0.191    0.192
## 76       Sp.Richness2 ~~  Brillouin.Index2   8.649  0.031   0.031    0.031
## 77       Sp.Richness2 ~~  Mean.Raup.Crick2  21.687 -0.089  -0.089   -0.089
## 78    Prop.Threat.Sp2 ~~  Brillouin.Index2   4.221  0.061   0.061    0.061
## 79    Prop.Threat.Sp2 ~~  Mean.Raup.Crick2  38.195 -0.278  -0.278   -0.278
## 82     Total.Animals2  ~   Prop.Threat.Sp2  17.776  0.103   0.103    0.103
## 84     Total.Animals2  ~  Mean.Raup.Crick2  11.525  0.205   0.205    0.205
## 88     Total.Animals2  ~        X50km_Pop2  35.809  0.144   0.144    0.144
## 89     Total.Animals2  ~        X10km_Pop2   8.873  0.074   0.074    0.074
## 90     Total.Animals2  ~      GDP.Millions2  29.909 -0.128  -0.128   -0.128
## 91     Total.Animals2  ~  Nat_Pop._WB20152   8.000 -0.066  -0.066   -0.066
## 95       Sp.Richness2  ~  Brillouin.Index2   6.728  0.085   0.085    0.085
## 96       Sp.Richness2  ~  Mean.Raup.Crick2  20.798 -0.091  -0.091   -0.091
## 101      Sp.Richness2  ~  Nat_Pop._WB20152   3.059 -0.032  -0.032   -0.032
## 102   Prop.Threat.Sp2  ~       Attendance2  38.882  0.355   0.355    0.360
## 103   Prop.Threat.Sp2  ~    Total.Animals2  14.140  0.203   0.203    0.203
## 105   Prop.Threat.Sp2  ~  Mean.Raup.Crick2  24.245 -0.230  -0.230   -0.230
## 106   Prop.Threat.Sp2  ~       Zoo.Area.ha2  10.359  0.150   0.150    0.150
## 107   Prop.Threat.Sp2  ~  Mam.Sp.Richness2  30.499  0.307   0.307    0.307
## 108   Prop.Threat.Sp2  ~      Prop.Mam.Sp2  47.215  0.356   0.356    0.356
## 109   Prop.Threat.Sp2  ~ Mean.Sp.BodyMass2  45.919  0.318   0.318    0.318
## 110   Prop.Threat.Sp2  ~        X50km_Pop2  19.706  0.210   0.210    0.210
## 111   Prop.Threat.Sp2  ~        X10km_Pop2  17.702  0.204   0.204    0.204
## 114  Brillouin.Index2  ~       Attendance2   6.933 -0.105  -0.105   -0.106
## 115  Brillouin.Index2  ~   Prop.Threat.Sp2   4.221  0.062   0.062    0.062
## 119  Brillouin.Index2  ~      Prop.Mam.Sp2   9.791  0.092   0.092    0.092
## 122  Brillouin.Index2  ~        X10km_Pop2   4.078 -0.054  -0.054   -0.054
## 123  Brillouin.Index2  ~      GDP.Millions2  58.529 -0.193  -0.193   -0.193
## 124  Brillouin.Index2  ~  Nat_Pop._WB20152  50.588 -0.179  -0.179   -0.179
## 125  Mean.Raup.Crick2  ~       Attendance2  23.818 -0.357  -0.357   -0.362
## 126  Mean.Raup.Crick2  ~   Prop.Threat.Sp2  37.487 -0.277  -0.277   -0.277
## 128  Mean.Raup.Crick2  ~       Zoo.Area.ha2  11.525 -0.170  -0.170   -0.170
## 129  Mean.Raup.Crick2  ~  Mam.Sp.Richness2  40.269 -0.424  -0.424   -0.424
## 130  Mean.Raup.Crick2  ~      Prop.Mam.Sp2 106.001 -0.545  -0.545   -0.545
## 131  Mean.Raup.Crick2  ~ Mean.Sp.BodyMass2 112.474 -0.494  -0.494   -0.494
## 132  Mean.Raup.Crick2  ~        X50km_Pop2   6.300  0.116   0.116    0.116
## 133  Mean.Raup.Crick2  ~        X10km_Pop2   4.704  0.104   0.104    0.104
## 135  Mean.Raup.Crick2  ~  Nat_Pop._WB20152  10.677  0.148   0.148    0.148
## 136      Zoo.Area.ha2  ~       Attendance2   6.203  0.440   0.440    0.446
## 138      Zoo.Area.ha2  ~      Sp.Richness2   9.364  0.602   0.602    0.602
## 140      Zoo.Area.ha2  ~  Brillouin.Index2   3.470  0.121   0.121    0.121
## 141      Zoo.Area.ha2  ~  Mean.Raup.Crick2  10.010  0.124   0.124    0.124
## 149  Mam.Sp.Richness2  ~       Attendance2   6.871 -0.479  -0.479   -0.486
```

```
## 150  Mam.Sp.Richness2  ~    Total.Animals2   3.877 -0.190  -0.190   -0.190
## 151  Mam.Sp.Richness2  ~      Sp.Richness2   9.521 -0.743  -0.743   -0.743
## 152  Mam.Sp.Richness2  ~   Prop.Threat.Sp2   9.855  0.144   0.144    0.144
## 154  Mam.Sp.Richness2  ~  Mean.Raup.Crick2   9.153 -0.156  -0.156   -0.156
## 165      Prop.Mam.Sp2  ~   Prop.Threat.Sp2  17.680  0.154   0.154    0.154
## 167      Prop.Mam.Sp2  ~  Mean.Raup.Crick2   5.722 -0.092  -0.092   -0.092
## 175 Mean.Sp.BodyMass2  ~       Attendance2  13.733 -0.399  -0.399   -0.405
## 178 Mean.Sp.BodyMass2  ~   Prop.Threat.Sp2   3.159  0.055   0.055    0.055
## 180 Mean.Sp.BodyMass2  ~  Mean.Raup.Crick2  48.621 -0.219  -0.219   -0.219
## 188        X50km_Pop2  ~       Attendance2  29.322  0.566   0.566    0.574
## 189        X50km_Pop2  ~    Total.Animals2  28.216  0.269   0.269    0.269
## 214     GDP.Millions2  ~       Attendance2  14.584 -0.318  -0.318   -0.323
## 216     GDP.Millions2  ~      Sp.Richness2  30.346  0.338   0.338    0.338
## 218     GDP.Millions2  ~  Brillouin.Index2   3.057  0.062   0.062    0.062
## 219     GDP.Millions2  ~  Mean.Raup.Crick2  13.631 -0.089  -0.089   -0.089
## 228  Nat_Pop._WB20152  ~    Total.Animals2   3.776 -0.076  -0.076   -0.076
## 229  Nat_Pop._WB20152  ~      Sp.Richness2  31.565 -0.333  -0.333   -0.333
## 230  Nat_Pop._WB20152  ~   Prop.Threat.Sp2   3.603  0.044   0.044    0.044
## 231  Nat_Pop._WB20152  ~  Brillouin.Index2  15.508 -0.134  -0.134   -0.134
## 232  Nat_Pop._WB20152  ~  Mean.Raup.Crick2  12.639  0.083   0.083    0.083
##     sepc.nox
## 72     0.101
## 74     0.192
## 76     0.031
## 77    -0.089
## 78     0.061
## 79    -0.278
## 82     0.103
## 84     0.205
## 88     0.144
## 89     0.074
## 90    -0.128
## 91    -0.067
## 95     0.085
## 96    -0.091
## 101   -0.032
## 102    0.360
## 103    0.203
## 105   -0.230
## 106    0.150
## 107    0.307
## 108    0.356
## 109    0.319
## 110    0.210
## 111    0.204
## 114   -0.106
## 115    0.062
## 119    0.092
## 122   -0.054
## 123   -0.193
## 124   -0.180
## 125   -0.362
## 126   -0.277
## 128   -0.170
```

```
## 129   -0.424
## 130   -0.545
## 131   -0.494
## 132    0.116
## 133    0.104
## 135    0.148
## 136    0.446
## 138    0.602
## 140    0.121
## 141    0.124
## 149   -0.486
## 150   -0.190
## 151   -0.743
## 152    0.144
## 154   -0.156
## 165    0.154
## 167   -0.092
## 175   -0.405
## 178    0.055
## 180   -0.219
## 188    0.574
## 189    0.269
## 214   -0.323
## 216    0.338
## 218    0.062
## 219   -0.089
## 228   -0.076
## 229   -0.333
## 230    0.044
## 231   -0.134
## 232    0.083
```

```
# Adjust for the nested nature of the data (institutions within countries)
# Fit model and generate model summary
design <- svydesign(ids = ~Country, nest=TRUE, data=sem_attendance_data)
fit.adj2 <- lavaan.survey(lavaan.fit = mod.2.fit, survey.design = design)
summary(fit.adj2, rsq = TRUE)
```

```
## lavaan (0.5-23.1097) converged normally after  52 iterations
##
##   Number of observations                           458
##
##   Estimator                                         ML      Robust
##   Minimum Function Test Statistic              567.053     140.826
##   Degrees of freedom                                40          40
##   P-value (Chi-square)                           0.000       0.000
##   Scaling correction factor                                  4.027
##     for the Satorra-Bentler correction
##
## Parameter Estimates:
##
##   Information                                 Expected
##   Standard Errors                           Robust.sem
##
## Regressions:
```

```
##                       Estimate  Std.Err  z-value  P(>|z|)
##   Attendance2 ~
##     Zoo.Area.ha2         0.086    0.041    2.115    0.034
##     Sp.Richness2        -0.249    0.082   -3.045    0.002
##     Total.Animals2       0.454    0.051    8.995    0.000
##     Mam.Sp.Rchnss2       0.132    0.049    2.712    0.007
##     Prop.Mam.Sp2        -0.065    0.044   -1.475    0.140
##     Prop.Thret.Sp2       0.009    0.024    0.371    0.710
##     Men.Sp.BdyMss2       0.339    0.029   11.577    0.000
##     Brilloun.Indx2       0.077    0.054    1.414    0.157
##     Mean.Rap.Crck2       0.146    0.027    5.344    0.000
##     X50km_Pop2           0.079    0.033    2.363    0.018
##     X10km_Pop2           0.397    0.042    9.352    0.000
##     GDP.Millions2        0.259    0.059    4.419    0.000
##     Nt_Pp._WB20152      -0.124    0.067   -1.845    0.065
##   Total.Animals2 ~
##     Zoo.Area.ha2         0.223    0.038    5.856    0.000
##     Sp.Richness2         0.798    0.048   16.678    0.000
##   Sp.Richness2 ~
##     Zoo.Area.ha2         0.067    0.044    1.519    0.129
##     Prop.Mam.Sp2        -0.581    0.038  -15.231    0.000
##     Mam.Sp.Rchnss2       0.755    0.068   11.066    0.000
##   Prop.Threat.Sp2 ~
##     Brilloun.Indx2      -0.084    0.101   -0.825    0.409
##   Brillouin.Index2 ~
##     Sp.Richness2         1.232    0.068   18.134    0.000
##     Total.Animals2      -0.527    0.078   -6.780    0.000
##   Mean.Raup.Crick2 ~
##     Sp.Richness2        -0.135    0.215   -0.626    0.531
##     Total.Animals2       0.355    0.156    2.277    0.023
##
## Covariances:
##                         Estimate  Std.Err  z-value  P(>|z|)
##   Zoo.Area.ha2 ~~
##     Mam.Sp.Rchnss2         0.381    0.064    5.985    0.000
##     Prop.Mam.Sp2           0.312    0.089    3.510    0.000
##     Men.Sp.BdyMss2         0.534    0.104    5.147    0.000
##     X50km_Pop2             0.060    0.069    0.880    0.379
##     X10km_Pop2            -0.010    0.078   -0.124    0.901
##     GDP.Millions2         -0.027    0.041   -0.673    0.501
##     Nt_Pp._WB20152         0.061    0.090    0.682    0.495
##   Mam.Sp.Richness2 ~~
##     Prop.Mam.Sp2           0.088    0.061    1.435    0.151
##     Men.Sp.BdyMss2         0.303    0.081    3.730    0.000
##     X50km_Pop2             0.204    0.082    2.487    0.013
##     X10km_Pop2             0.284    0.066    4.278    0.000
##     GDP.Millions2         -0.060    0.064   -0.937    0.349
##     Nt_Pp._WB20152        -0.088    0.102   -0.863    0.388
##   Prop.Mam.Sp2 ~~
##     Men.Sp.BdyMss2         0.604    0.130    4.656    0.000
##     X50km_Pop2            -0.096    0.051   -1.888    0.059
##     X10km_Pop2            -0.204    0.055   -3.685    0.000
##     GDP.Millions2         -0.123    0.105   -1.172    0.241
##     Nt_Pp._WB20152        -0.115    0.091   -1.257    0.209
```

```
##   Mean.Sp.BodyMass2 ~~
##     X50km_Pop2          0.101    0.072    1.400    0.161
##     X10km_Pop2          0.126    0.067    1.895    0.058
##     GDP.Millions2      -0.082    0.046   -1.759    0.079
##     Nt_Pp._WB20152      0.006    0.092    0.063    0.950
##   X50km_Pop2 ~~
##     X10km_Pop2          0.752    0.126    5.982    0.000
##     GDP.Millions2      -0.013    0.100   -0.131    0.896
##     Nt_Pp._WB20152      0.191    0.177    1.083    0.279
##   X10km_Pop2 ~~
##     GDP.Millions2      -0.028    0.056   -0.505    0.613
##     Nt_Pp._WB20152      0.173    0.101    1.701    0.089
##   GDP.Millions2 ~~
##     Nt_Pp._WB20152      0.826    0.202    4.089    0.000
##
## Intercepts:
##                    Estimate  Std.Err  z-value  P(>|z|)
##    .Attendance2      -0.000    0.031   -0.000    1.000
##    .Total.Animals2   -0.000    0.104   -0.000    1.000
##    .Sp.Richness2      0.000    0.034    0.000    1.000
##    .Prop.Thret.Sp2    0.000    0.099    0.000    1.000
##    .Brilloun.Indx2   -0.000    0.106   -0.000    1.000
##    .Mean.Rap.Crck2    0.000    0.092    0.000    1.000
##     Zoo.Area.ha2      0.000    0.064    0.000    1.000
##     Mam.Sp.Rchnss2   -0.000    0.079   -0.000    1.000
##     Prop.Mam.Sp2      0.000    0.118    0.000    1.000
##     Men.Sp.BdyMss2   -0.000    0.080   -0.000    1.000
##     X50km_Pop2       -0.000    0.114   -0.000    1.000
##     X10km_Pop2        0.000    0.105    0.000    1.000
##     GDP.Millions2    -0.000    0.345   -0.000    1.000
##     Nt_Pp._WB20152    0.000    0.295    0.000    1.000
##
## Variances:
##                    Estimate  Std.Err  z-value  P(>|z|)
##    .Attendance2       0.267    0.028    9.566    0.000
##    .Total.Animals2    0.251    0.044    5.729    0.000
##    .Sp.Richness2      0.151    0.046    3.269    0.001
##    .Prop.Thret.Sp2    0.991    0.135    7.335    0.000
##    .Brilloun.Indx2    0.290    0.079    3.656    0.000
##    .Mean.Rap.Crck2    0.934    0.100    9.311    0.000
##     Zoo.Area.ha2      0.998    0.112    8.886    0.000
##     Mam.Sp.Rchnss2    0.998    0.115    8.669    0.000
##     Prop.Mam.Sp2      0.998    0.150    6.662    0.000
##     Men.Sp.BdyMss2    0.998    0.173    5.783    0.000
##     X50km_Pop2        0.998    0.131    7.623    0.000
##     X10km_Pop2        0.998    0.174    5.745    0.000
##     GDP.Millions2     0.998    0.246    4.061    0.000
##     Nt_Pp._WB20152    0.998    0.225    4.439    0.000
##
## R-Square:
##                    Estimate
##     Attendance2       0.740
##     Total.Animals2    0.748
##     Sp.Richness2      0.849
```

```
##      Prop.Thret.Sp2    0.007
##      Brilloun.Indx2    0.709
##      Mean.Rap.Crck2    0.064
```

```
# Generate fit indices
fitMeasures(fit.adj2, c("agfi", "rmr", "srmr", "rmsea", "cfi", "nnfi", "tli"))
```

```
##  agfi   rmr  srmr rmsea   cfi  nnfi   tli
## 0.592 0.102 0.096 0.170 0.835 0.741 0.741
```

```
# Generate modification indices
mi2adj <- modindices(fit.adj2)
print(mi2adj[mi2adj$mi > 3.0,])
```

```
##                 lhs op               rhs      mi mi.scaled    epc
## 86      Total.Animals2 ~~    Prop.Threat.Sp2  17.524     4.352  0.101
## 88      Total.Animals2 ~~   Mean.Raup.Crick2  11.525     2.862  0.191
## 90        Sp.Richness2 ~~   Brillouin.Index2   8.649     2.148  0.031
## 91        Sp.Richness2 ~~   Mean.Raup.Crick2  21.687     5.386 -0.089
## 92     Prop.Threat.Sp2 ~~   Brillouin.Index2   4.221     1.048  0.061
## 93     Prop.Threat.Sp2 ~~   Mean.Raup.Crick2  38.195     9.486 -0.278
## 96      Total.Animals2  ~    Prop.Threat.Sp2  17.776     4.415  0.103
## 98      Total.Animals2  ~   Mean.Raup.Crick2  11.525     2.862  0.205
## 102     Total.Animals2  ~        X50km_Pop2  35.809     8.893  0.144
## 103     Total.Animals2  ~        X10km_Pop2   8.873     2.204  0.074
## 104     Total.Animals2  ~      GDP.Millions2  29.909     7.428 -0.128
## 105     Total.Animals2  ~   Nat_Pop._WB20152   8.000     1.987 -0.066
## 109       Sp.Richness2  ~   Brillouin.Index2   6.728     1.671  0.085
## 110       Sp.Richness2  ~   Mean.Raup.Crick2  20.798     5.165 -0.091
## 115       Sp.Richness2  ~   Nat_Pop._WB20152   3.059     0.760 -0.032
## 116    Prop.Threat.Sp2  ~       Attendance2  38.883     9.656  0.355
## 117    Prop.Threat.Sp2  ~     Total.Animals2  14.140     3.512  0.203
## 119    Prop.Threat.Sp2  ~   Mean.Raup.Crick2  24.245     6.021 -0.230
## 120    Prop.Threat.Sp2  ~        Zoo.Area.ha2  10.359     2.573  0.150
## 121    Prop.Threat.Sp2  ~   Mam.Sp.Richness2  30.499     7.574  0.307
## 122    Prop.Threat.Sp2  ~       Prop.Mam.Sp2  47.215    11.726  0.356
## 123    Prop.Threat.Sp2  ~ Mean.Sp.BodyMass2  45.919    11.404  0.318
## 124    Prop.Threat.Sp2  ~        X50km_Pop2  19.706     4.894  0.210
## 125    Prop.Threat.Sp2  ~        X10km_Pop2  17.702     4.396  0.204
## 128   Brillouin.Index2  ~       Attendance2   6.933     1.722 -0.105
## 129   Brillouin.Index2  ~    Prop.Threat.Sp2   4.221     1.048  0.062
## 133   Brillouin.Index2  ~       Prop.Mam.Sp2   9.791     2.432  0.092
## 136   Brillouin.Index2  ~        X10km_Pop2   4.078     1.013 -0.054
## 137   Brillouin.Index2  ~      GDP.Millions2  58.529    14.536 -0.193
## 138   Brillouin.Index2  ~   Nat_Pop._WB20152  50.588    12.563 -0.179
## 139   Mean.Raup.Crick2  ~       Attendance2  23.818     5.915 -0.357
## 140   Mean.Raup.Crick2  ~    Prop.Threat.Sp2  37.487     9.310 -0.277
## 142   Mean.Raup.Crick2  ~        Zoo.Area.ha2  11.525     2.862 -0.170
## 143   Mean.Raup.Crick2  ~   Mam.Sp.Richness2  40.269    10.001 -0.424
## 144   Mean.Raup.Crick2  ~       Prop.Mam.Sp2 106.002    26.325 -0.545
## 145   Mean.Raup.Crick2  ~ Mean.Sp.BodyMass2 112.474    27.933 -0.494
## 146   Mean.Raup.Crick2  ~        X50km_Pop2   6.300     1.565  0.116
## 147   Mean.Raup.Crick2  ~        X10km_Pop2   4.704     1.168  0.104
## 149   Mean.Raup.Crick2  ~   Nat_Pop._WB20152  10.677     2.652  0.148
## 150       Zoo.Area.ha2  ~       Attendance2   6.203     1.541  0.440
## 152       Zoo.Area.ha2  ~       Sp.Richness2   9.363     2.325  0.602
```

```
## 154       Zoo.Area.ha2  ~  Brillouin.Index2   3.470     0.862  0.121
## 155       Zoo.Area.ha2  ~  Mean.Raup.Crick2  10.010     2.486  0.124
## 163  Mam.Sp.Richness2  ~       Attendance2   6.871     1.706 -0.479
## 164  Mam.Sp.Richness2  ~    Total.Animals2   3.877     0.963 -0.190
## 165  Mam.Sp.Richness2  ~      Sp.Richness2   9.521     2.364 -0.742
## 166  Mam.Sp.Richness2  ~   Prop.Threat.Sp2   9.855     2.447  0.144
## 168  Mam.Sp.Richness2  ~  Mean.Raup.Crick2   9.153     2.273 -0.156
## 179      Prop.Mam.Sp2  ~   Prop.Threat.Sp2  17.680     4.391  0.154
## 181      Prop.Mam.Sp2  ~  Mean.Raup.Crick2   5.722     1.421 -0.092
## 189 Mean.Sp.BodyMass2  ~       Attendance2  13.734     3.411 -0.399
## 192 Mean.Sp.BodyMass2  ~   Prop.Threat.Sp2   3.159     0.785  0.055
## 194 Mean.Sp.BodyMass2  ~  Mean.Raup.Crick2  48.621    12.075 -0.219
## 202        X50km_Pop2  ~       Attendance2  29.323     7.282  0.566
## 203        X50km_Pop2  ~    Total.Animals2  28.216     7.007  0.269
## 228     GDP.Millions2  ~       Attendance2  14.584     3.622 -0.318
## 230     GDP.Millions2  ~      Sp.Richness2  30.346     7.536  0.338
## 232     GDP.Millions2  ~  Brillouin.Index2   3.057     0.759  0.062
## 233     GDP.Millions2  ~  Mean.Raup.Crick2  13.631     3.385 -0.089
## 242  Nat_Pop._WB20152  ~    Total.Animals2   3.776     0.938 -0.076
## 243  Nat_Pop._WB20152  ~      Sp.Richness2  31.565     7.839 -0.333
## 244  Nat_Pop._WB20152  ~   Prop.Threat.Sp2   3.603     0.895  0.044
## 245  Nat_Pop._WB20152  ~  Brillouin.Index2  15.508     3.851 -0.134
## 246  Nat_Pop._WB20152  ~  Mean.Raup.Crick2  12.639     3.139  0.083
##     sepc.lv sepc.all sepc.nox
## 86    0.101    0.101    0.101
## 88    0.191    0.192    0.192
## 90    0.031    0.031    0.031
## 91   -0.089   -0.089   -0.089
## 92    0.061    0.061    0.061
## 93   -0.278   -0.278   -0.278
## 96    0.103    0.103    0.103
## 98    0.205    0.205    0.205
## 102   0.144    0.144    0.144
## 103   0.074    0.074    0.074
## 104  -0.128   -0.128   -0.128
## 105  -0.066   -0.066   -0.067
## 109   0.085    0.085    0.085
## 110  -0.091   -0.091   -0.091
## 115  -0.032   -0.032   -0.032
## 116   0.355    0.360    0.360
## 117   0.203    0.203    0.203
## 119  -0.230   -0.230   -0.230
## 120   0.150    0.150    0.150
## 121   0.307    0.307    0.307
## 122   0.356    0.356    0.356
## 123   0.318    0.318    0.319
## 124   0.210    0.210    0.210
## 125   0.204    0.204    0.204
## 128  -0.105   -0.106   -0.106
## 129   0.062    0.062    0.062
## 133   0.092    0.092    0.092
## 136  -0.054   -0.054   -0.054
## 137  -0.193   -0.193   -0.193
## 138  -0.179   -0.179   -0.180
```

```
## 139  -0.357   -0.362   -0.362
## 140  -0.277   -0.277   -0.277
## 142  -0.170   -0.170   -0.170
## 143  -0.424   -0.424   -0.424
## 144  -0.545   -0.545   -0.545
## 145  -0.494   -0.494   -0.494
## 146   0.116    0.116    0.116
## 147   0.104    0.104    0.104
## 149   0.148    0.148    0.148
## 150   0.440    0.446    0.446
## 152   0.602    0.602    0.602
## 154   0.121    0.121    0.121
## 155   0.124    0.124    0.124
## 163  -0.479   -0.486   -0.486
## 164  -0.190   -0.190   -0.190
## 165  -0.742   -0.742   -0.742
## 166   0.144    0.144    0.144
## 168  -0.156   -0.156   -0.156
## 179   0.154    0.154    0.154
## 181  -0.092   -0.092   -0.092
## 189  -0.399   -0.405   -0.405
## 192   0.055    0.055    0.055
## 194  -0.219   -0.219   -0.219
## 202   0.566    0.574    0.574
## 203   0.269    0.269    0.269
## 228  -0.318   -0.323   -0.323
## 230   0.338    0.338    0.338
## 232   0.062    0.062    0.062
## 233  -0.089   -0.089   -0.089
## 242  -0.076   -0.076   -0.076
## 243  -0.333   -0.333   -0.333
## 244   0.044    0.044    0.044
## 245  -0.134   -0.134   -0.134
## 246   0.083    0.083    0.083
```

Model 3

Based on the modification indices generated from the second model, we can see that **Mean.Raup.Crick2** ~ **Mean.Sp.BodyMass2** has the highest mi value of **112.474**. This far exceeds the standard cut-off level for the chi-square test criterion of 3.84 (Burnham and Anderson, 2002). This also makes theoretical sense, as if we increase the mean species body mass of a collection, then logically the institution can hold fewer species overall and therefore has a higher chance of having an overlapping collection composition with other institutions (Raup Crick is a community ecology dissimilarity index based on species presence/absence data). As a result, we add this relationship to our model. Once again, the model summary, fit indices and modification indices were all generated for the model, adjusting for the nested nature of data.

```
# Attendance SEM (Presence-Absence)

# Model 3
# Addition of Mean.Raup.Crick2 ~ Mean.Sp.BodyMass2, mi = 112.474

mod.3 <- 'Attendance2 ~ Zoo.Area.ha2 + Sp.Richness2 + Total.Animals2
+ Mam.Sp.Richness2 + Prop.Mam.Sp2 + Prop.Threat.Sp2
```

```r
+ Mean.Sp.BodyMass2 + Brillouin.Index2 + Mean.Raup.Crick2
+ X50km_Pop2 + X10km_Pop2 + GDP.Millions2 + Nat_Pop._WB20152

Total.Animals2 ~ Zoo.Area.ha2 + Sp.Richness2
Sp.Richness2 ~ Zoo.Area.ha2 + Prop.Mam.Sp2 + Mam.Sp.Richness2
Prop.Threat.Sp2 ~ Brillouin.Index2
Brillouin.Index2 ~ Sp.Richness2 + Total.Animals2
Mean.Raup.Crick2 ~ Sp.Richness2 + Total.Animals2 + Mean.Sp.BodyMass2'

# Fit model and generate model summary
mod.3.fit <- sem(mod.3, data = sem_attendance_data, fixed.x=FALSE)
summary(mod.3.fit, rsq = TRUE)
```

```
## lavaan (0.5-23.1097) converged normally after  55 iterations
##
##   Number of observations                           458
##
##   Estimator                                         ML
##   Minimum Function Test Statistic              437.526
##   Degrees of freedom                                39
##   P-value (Chi-square)                           0.000
##
## Parameter Estimates:
##
##   Information                                 Expected
##   Standard Errors                             Standard
##
## Regressions:
##                    Estimate  Std.Err  z-value  P(>|z|)
##   Attendance2 ~
##     Zoo.Area.ha2      0.086    0.033    2.593    0.010
##     Sp.Richness2     -0.249    0.092   -2.704    0.007
##     Total.Animals2    0.454    0.056    8.110    0.000
##     Mam.Sp.Rchnss2    0.132    0.055    2.403    0.016
##     Prop.Mam.Sp2     -0.065    0.049   -1.337    0.181
##     Prop.Thret.Sp2    0.009    0.024    0.371    0.711
##     Men.Sp.BdyMss2    0.339    0.039    8.644    0.000
##     Brilloun.Indx2    0.077    0.045    1.716    0.086
##     Mean.Rap.Crck2    0.146    0.029    5.054    0.000
##     X50km_Pop2        0.079    0.038    2.096    0.036
##     X10km_Pop2        0.397    0.041    9.624    0.000
##     GDP.Millions2     0.259    0.047    5.473    0.000
##     Nt_Pp._WB20152   -0.124    0.049   -2.523    0.012
##   Total.Animals2 ~
##     Zoo.Area.ha2      0.223    0.024    9.384    0.000
##     Sp.Richness2      0.798    0.024   33.520    0.000
##   Sp.Richness2 ~
##     Zoo.Area.ha2      0.067    0.021    3.225    0.001
##     Prop.Mam.Sp2     -0.581    0.019  -30.364    0.000
##     Mam.Sp.Rchnss2    0.755    0.020   38.373    0.000
##   Prop.Threat.Sp2 ~
##     Brilloun.Indx2   -0.084    0.047   -1.795    0.073
##   Brillouin.Index2 ~
##     Sp.Richness2      1.232    0.046   26.770    0.000
```

```
##     Total.Animals2     -0.527    0.046  -11.451    0.000
##   Mean.Raup.Crick2 ~
##     Sp.Richness2       -0.355    0.074   -4.820    0.000
##     Total.Animals2      0.558    0.074    7.594    0.000
##     Men.Sp.BdyMss2     -0.495    0.040  -12.254    0.000
##
## Covariances:
##                        Estimate  Std.Err  z-value  P(>|z|)
##   Zoo.Area.ha2 ~~
##     Mam.Sp.Rchnss2        0.381    0.050    7.634    0.000
##     Prop.Mam.Sp2          0.312    0.049    6.389    0.000
##     Men.Sp.BdyMss2        0.534    0.053   10.096    0.000
##     X50km_Pop2            0.060    0.047    1.291    0.197
##     X10km_Pop2           -0.010    0.047   -0.208    0.835
##     GDP.Millions2        -0.027    0.047   -0.588    0.557
##     Nt_Pp._WB20152        0.061    0.047    1.310    0.190
##   Mam.Sp.Richness2 ~~
##     Prop.Mam.Sp2          0.088    0.047    1.882    0.060
##     Men.Sp.BdyMss2        0.303    0.049    6.216    0.000
##     X50km_Pop2            0.204    0.048    4.281    0.000
##     X10km_Pop2            0.284    0.048    5.852    0.000
##     GDP.Millions2        -0.060    0.047   -1.276    0.202
##     Nt_Pp._WB20152       -0.088    0.047   -1.881    0.060
##   Prop.Mam.Sp2 ~~
##     Men.Sp.BdyMss2        0.604    0.055   11.083    0.000
##     X50km_Pop2           -0.096    0.047   -2.054    0.040
##     X10km_Pop2           -0.204    0.048   -4.293    0.000
##     GDP.Millions2        -0.123    0.047   -2.615    0.009
##     Nt_Pp._WB20152       -0.115    0.047   -2.447    0.014
##   Mean.Sp.BodyMass2 ~~
##     X50km_Pop2            0.101    0.047    2.166    0.030
##     X10km_Pop2            0.126    0.047    2.683    0.007
##     GDP.Millions2        -0.082    0.047   -1.748    0.080
##     Nt_Pp._WB20152        0.006    0.047    0.124    0.901
##   X50km_Pop2 ~~
##     X10km_Pop2            0.752    0.058   12.886    0.000
##     GDP.Millions2        -0.013    0.047   -0.281    0.779
##     Nt_Pp._WB20152        0.191    0.047    4.033    0.000
##   X10km_Pop2 ~~
##     GDP.Millions2        -0.028    0.047   -0.604    0.546
##     Nt_Pp._WB20152        0.173    0.047    3.646    0.000
##   GDP.Millions2 ~~
##     Nt_Pp._WB20152        0.826    0.061   13.642    0.000
##
## Variances:
##                    Estimate  Std.Err  z-value  P(>|z|)
##    .Attendance2       0.267    0.018   15.133    0.000
##    .Total.Animals2    0.251    0.017   15.133    0.000
##    .Sp.Richness2      0.151    0.010   15.133    0.000
##    .Prop.Thret.Sp2    0.991    0.065   15.133    0.000
##    .Brilloun.Indx2    0.290    0.019   15.133    0.000
##    .Mean.Rap.Crck2    0.704    0.047   15.133    0.000
##     Zoo.Area.ha2      0.998    0.066   15.133    0.000
##     Mam.Sp.Rchnss2    0.998    0.066   15.133    0.000
```

```
##     Prop.Mam.Sp2      0.998    0.066   15.133    0.000
##     Men.Sp.BdyMss2    0.998    0.066   15.133    0.000
##     X50km_Pop2        0.998    0.066   15.133    0.000
##     X10km_Pop2        0.998    0.066   15.133    0.000
##     GDP.Millions2     0.998    0.066   15.133    0.000
##     Nt_Pp._WB20152    0.998    0.066   15.133    0.000
##
## R-Square:
##                    Estimate
##     Attendance2       0.723
##     Total.Animals2    0.748
##     Sp.Richness2      0.849
##     Prop.Thret.Sp2    0.007
##     Brilloun.Indx2    0.709
##     Mean.Rap.Crck2    0.294
```

```
# Generate fit indices
fitMeasures(mod.3.fit, c("agfi", "rmr", "srmr", "rmsea", "cfi", "nnfi", "tli"))
```

```
##  agfi   rmr  srmr rmsea   cfi  nnfi   tli
## 0.708 0.083 0.084 0.149 0.876 0.799 0.799
```

```
# Generate modification indices
mi3 <- modindices(mod.3.fit)
print(mi3[mi3$mi > 3.0,])
```

```
##                 lhs op               rhs     mi    epc sepc.lv sepc.all
## 73     Total.Animals2 ~~   Prop.Threat.Sp2 17.524  0.101   0.101    0.101
## 75     Total.Animals2 ~~  Mean.Raup.Crick2  9.205 -0.175  -0.175   -0.175
## 77       Sp.Richness2 ~~  Brillouin.Index2  8.649  0.031   0.031    0.031
## 78       Sp.Richness2 ~~  Mean.Raup.Crick2 27.606 -0.087  -0.087   -0.087
## 79    Prop.Threat.Sp2 ~~  Brillouin.Index2  4.221  0.061   0.061    0.061
## 80    Prop.Threat.Sp2 ~~  Mean.Raup.Crick2 14.275 -0.147  -0.147   -0.148
## 83     Total.Animals2  ~   Prop.Threat.Sp2 17.776  0.103   0.103    0.103
## 85     Total.Animals2  ~  Mean.Raup.Crick2  3.003 -0.081  -0.081   -0.081
## 89     Total.Animals2  ~        X50km_Pop2 35.809  0.144   0.144    0.144
## 90     Total.Animals2  ~        X10km_Pop2  8.873  0.074   0.074    0.074
## 91     Total.Animals2  ~     GDP.Millions2 29.909 -0.128  -0.128   -0.128
## 92     Total.Animals2  ~  Nat_Pop._WB20152  8.000 -0.066  -0.066   -0.066
## 96       Sp.Richness2  ~  Brillouin.Index2  6.728  0.085   0.085    0.085
## 97       Sp.Richness2  ~  Mean.Raup.Crick2 17.010 -0.085  -0.085   -0.085
## 102      Sp.Richness2  ~  Nat_Pop._WB20152  3.059 -0.032  -0.032   -0.032
## 103   Prop.Threat.Sp2  ~       Attendance2 42.829  0.391   0.391    0.384
## 104   Prop.Threat.Sp2  ~    Total.Animals2 14.140  0.203   0.203    0.203
## 106   Prop.Threat.Sp2  ~  Mean.Raup.Crick2 24.260 -0.230  -0.230   -0.230
## 107   Prop.Threat.Sp2  ~      Zoo.Area.ha2 10.359  0.150   0.150    0.150
## 108   Prop.Threat.Sp2  ~  Mam.Sp.Richness2 30.499  0.307   0.307    0.307
## 109   Prop.Threat.Sp2  ~      Prop.Mam.Sp2 47.215  0.356   0.356    0.356
## 110   Prop.Threat.Sp2  ~ Mean.Sp.BodyMass2 45.919  0.318   0.318    0.318
## 111   Prop.Threat.Sp2  ~        X50km_Pop2 19.706  0.210   0.210    0.210
## 112   Prop.Threat.Sp2  ~        X10km_Pop2 17.702  0.204   0.204    0.204
## 115  Brillouin.Index2  ~       Attendance2  8.180 -0.124  -0.124   -0.122
## 116  Brillouin.Index2  ~   Prop.Threat.Sp2  4.221  0.062   0.062    0.062
## 120  Brillouin.Index2  ~      Prop.Mam.Sp2  9.791  0.092   0.092    0.092
## 123  Brillouin.Index2  ~        X10km_Pop2  4.078 -0.054  -0.054   -0.054
## 124  Brillouin.Index2  ~     GDP.Millions2 58.529 -0.193  -0.193   -0.193
```

```
## 125  Brillouin.Index2  ~  Nat_Pop._WB20152 50.588 -0.179  -0.179   -0.179
## 126  Mean.Raup.Crick2  ~       Attendance2 19.321  0.394   0.394    0.388
## 127  Mean.Raup.Crick2  ~   Prop.Threat.Sp2 14.003 -0.147  -0.147   -0.147
## 129  Mean.Raup.Crick2  ~      Zoo.Area.ha2  9.205  0.155   0.155    0.155
## 131  Mean.Raup.Crick2  ~      Prop.Mam.Sp2 28.655 -0.318  -0.318   -0.318
## 132  Mean.Raup.Crick2  ~        X50km_Pop2 14.173  0.152   0.152    0.153
## 133  Mean.Raup.Crick2  ~        X10km_Pop2 19.575  0.187   0.187    0.187
## 135  Mean.Raup.Crick2  ~  Nat_Pop._WB20152 16.563  0.160   0.160    0.160
## 136      Zoo.Area.ha2  ~       Attendance2  4.180  0.369   0.369    0.363
## 138      Zoo.Area.ha2  ~      Sp.Richness2  9.364  0.602   0.602    0.602
## 140      Zoo.Area.ha2  ~  Brillouin.Index2  3.470  0.121   0.121    0.121
## 141      Zoo.Area.ha2  ~  Mean.Raup.Crick2  7.546  0.122   0.122    0.122
## 149  Mam.Sp.Richness2  ~       Attendance2  4.255 -0.372  -0.372   -0.366
## 150  Mam.Sp.Richness2  ~    Total.Animals2  3.877 -0.190  -0.190   -0.190
## 151  Mam.Sp.Richness2  ~      Sp.Richness2  9.521 -0.742  -0.742   -0.742
## 152  Mam.Sp.Richness2  ~   Prop.Threat.Sp2  9.855  0.144   0.144    0.144
## 154  Mam.Sp.Richness2  ~  Mean.Raup.Crick2  4.940 -0.128  -0.128   -0.128
## 165      Prop.Mam.Sp2  ~   Prop.Threat.Sp2 17.680  0.154   0.154    0.154
## 167      Prop.Mam.Sp2  ~  Mean.Raup.Crick2 11.046 -0.142  -0.142   -0.142
## 178 Mean.Sp.BodyMass2  ~   Prop.Threat.Sp2  3.159  0.055   0.055    0.055
## 188        X50km_Pop2  ~       Attendance2 28.697  0.554   0.554    0.544
## 189        X50km_Pop2  ~    Total.Animals2 28.216  0.269   0.269    0.269
## 193        X50km_Pop2  ~  Mean.Raup.Crick2  3.649  0.065   0.065    0.064
## 214     GDP.Millions2  ~       Attendance2 14.273 -0.311  -0.311   -0.306
## 216     GDP.Millions2  ~      Sp.Richness2 30.345  0.338   0.338    0.338
## 218     GDP.Millions2  ~  Brillouin.Index2  3.057  0.062   0.062    0.062
## 219     GDP.Millions2  ~  Mean.Raup.Crick2 16.858 -0.111  -0.111   -0.111
## 228  Nat_Pop._WB20152  ~    Total.Animals2  3.776 -0.076  -0.076   -0.076
## 229  Nat_Pop._WB20152  ~      Sp.Richness2 31.564 -0.333  -0.333   -0.333
## 230  Nat_Pop._WB20152  ~   Prop.Threat.Sp2  3.603  0.044   0.044    0.044
## 231  Nat_Pop._WB20152  ~  Brillouin.Index2 15.508 -0.134  -0.134   -0.134
## 232  Nat_Pop._WB20152  ~  Mean.Raup.Crick2 15.632  0.103   0.103    0.103
##     sepc.nox
## 73     0.101
## 75    -0.175
## 77     0.031
## 78    -0.087
## 79     0.061
## 80    -0.148
## 83     0.103
## 85    -0.081
## 89     0.144
## 90     0.074
## 91    -0.128
## 92    -0.067
## 96     0.085
## 97    -0.085
## 102   -0.032
## 103    0.384
## 104    0.203
## 106   -0.230
## 107    0.150
## 108    0.307
## 109    0.356
```

```
## 110    0.319
## 111    0.210
## 112    0.204
## 115   -0.122
## 116    0.062
## 120    0.092
## 123   -0.054
## 124   -0.193
## 125   -0.180
## 126    0.388
## 127   -0.147
## 129    0.156
## 131   -0.319
## 132    0.153
## 133    0.187
## 135    0.160
## 136    0.363
## 138    0.602
## 140    0.121
## 141    0.122
## 149   -0.366
## 150   -0.190
## 151   -0.742
## 152    0.144
## 154   -0.128
## 165    0.154
## 167   -0.142
## 178    0.055
## 188    0.544
## 189    0.269
## 193    0.064
## 214   -0.306
## 216    0.338
## 218    0.062
## 219   -0.111
## 228   -0.076
## 229   -0.333
## 230    0.044
## 231   -0.134
## 232    0.103
```

```
# Adjust for the nested nature of the data (institutions within countries)
# Fit model and generate model summary
design <- svydesign(ids = ~Country, nest=TRUE, data=sem_attendance_data)
fit.adj3 <- lavaan.survey(lavaan.fit = mod.3.fit, survey.design = design)
summary(fit.adj3, rsq = TRUE)
```

```
## lavaan (0.5-23.1097) converged normally after  55 iterations
##
##   Number of observations                           458
##
##   Estimator                                         ML      Robust
##   Minimum Function Test Statistic              437.526     104.311
##   Degrees of freedom                                39          39
##   P-value (Chi-square)                           0.000       0.000
```

```
##   Scaling correction factor                                  4.194
##     for the Satorra-Bentler correction
##
## Parameter Estimates:
##
##   Information                                 Expected
##   Standard Errors                           Robust.sem
##
## Regressions:
##                      Estimate  Std.Err  z-value  P(>|z|)
##   Attendance2 ~
##     Zoo.Area.ha2        0.086    0.041    2.115    0.034
##     Sp.Richness2       -0.249    0.083   -3.017    0.003
##     Total.Animals2      0.454    0.055    8.313    0.000
##     Mam.Sp.Rchnss2      0.132    0.049    2.712    0.007
##     Prop.Mam.Sp2       -0.065    0.044   -1.475    0.140
##     Prop.Thret.Sp2      0.009    0.024    0.371    0.710
##     Men.Sp.BdyMss2      0.339    0.030   11.327    0.000
##     Brilloun.Indx2      0.077    0.054    1.414    0.157
##     Mean.Rap.Crck2      0.146    0.029    5.002    0.000
##     X50km_Pop2          0.079    0.033    2.363    0.018
##     X10km_Pop2          0.397    0.042    9.352    0.000
##     GDP.Millions2       0.259    0.059    4.419    0.000
##     Nt_Pp._WB20152     -0.124    0.067   -1.845    0.065
##   Total.Animals2 ~
##     Zoo.Area.ha2        0.223    0.038    5.856    0.000
##     Sp.Richness2        0.798    0.048   16.678    0.000
##   Sp.Richness2 ~
##     Zoo.Area.ha2        0.067    0.044    1.519    0.129
##     Prop.Mam.Sp2       -0.581    0.038  -15.231    0.000
##     Mam.Sp.Rchnss2      0.755    0.068   11.066    0.000
##   Prop.Threat.Sp2 ~
##     Brilloun.Indx2     -0.084    0.101   -0.825    0.409
##   Brillouin.Index2 ~
##     Sp.Richness2        1.232    0.068   18.134    0.000
##     Total.Animals2     -0.527    0.078   -6.780    0.000
##   Mean.Raup.Crick2 ~
##     Sp.Richness2       -0.355    0.228   -1.555    0.120
##     Total.Animals2      0.558    0.153    3.656    0.000
##     Men.Sp.BdyMss2     -0.495    0.052   -9.463    0.000
##
## Covariances:
##                        Estimate  Std.Err  z-value  P(>|z|)
##   Zoo.Area.ha2 ~~
##     Mam.Sp.Rchnss2        0.381    0.064    5.985    0.000
##     Prop.Mam.Sp2          0.312    0.089    3.510    0.000
##     Men.Sp.BdyMss2        0.534    0.104    5.147    0.000
##     X50km_Pop2            0.060    0.069    0.880    0.379
##     X10km_Pop2           -0.010    0.078   -0.124    0.901
##     GDP.Millions2        -0.027    0.041   -0.673    0.501
##     Nt_Pp._WB20152        0.061    0.090    0.682    0.495
##   Mam.Sp.Richness2 ~~
##     Prop.Mam.Sp2          0.088    0.061    1.435    0.151
##     Men.Sp.BdyMss2        0.303    0.081    3.730    0.000
```

```
##     X50km_Pop2           0.204    0.082    2.487    0.013
##     X10km_Pop2           0.284    0.066    4.278    0.000
##     GDP.Millions2       -0.060    0.064   -0.937    0.349
##     Nt_Pp._WB20152      -0.088    0.102   -0.863    0.388
##   Prop.Mam.Sp2 ~~
##     Men.Sp.BdyMss2       0.604    0.130    4.656    0.000
##     X50km_Pop2          -0.096    0.051   -1.888    0.059
##     X10km_Pop2          -0.204    0.055   -3.685    0.000
##     GDP.Millions2       -0.123    0.105   -1.172    0.241
##     Nt_Pp._WB20152      -0.115    0.091   -1.257    0.209
##   Mean.Sp.BodyMass2 ~~
##     X50km_Pop2           0.101    0.072    1.400    0.161
##     X10km_Pop2           0.126    0.067    1.895    0.058
##     GDP.Millions2       -0.082    0.046   -1.759    0.079
##     Nt_Pp._WB20152       0.006    0.092    0.063    0.950
##   X50km_Pop2 ~~
##     X10km_Pop2           0.752    0.126    5.982    0.000
##     GDP.Millions2       -0.013    0.100   -0.131    0.896
##     Nt_Pp._WB20152       0.191    0.177    1.083    0.279
##   X10km_Pop2 ~~
##     GDP.Millions2       -0.028    0.056   -0.505    0.613
##     Nt_Pp._WB20152       0.173    0.101    1.701    0.089
##   GDP.Millions2 ~~
##     Nt_Pp._WB20152       0.826    0.202    4.089    0.000
##
## Intercepts:
##                    Estimate  Std.Err  z-value  P(>|z|)
##    .Attendance2      -0.000    0.031   -0.000    1.000
##    .Total.Animals2   -0.000    0.104   -0.000    1.000
##    .Sp.Richness2      0.000    0.034    0.000    1.000
##    .Prop.Thret.Sp2    0.000    0.099    0.000    1.000
##    .Brilloun.Indx2   -0.000    0.106   -0.000    1.000
##    .Mean.Rap.Crck2    0.000    0.094    0.000    1.000
##     Zoo.Area.ha2      0.000    0.064    0.000    1.000
##     Mam.Sp.Rchnss2   -0.000    0.079   -0.000    1.000
##     Prop.Mam.Sp2      0.000    0.118    0.000    1.000
##     Men.Sp.BdyMss2   -0.000    0.080   -0.000    1.000
##     X50km_Pop2       -0.000    0.114   -0.000    1.000
##     X10km_Pop2        0.000    0.105    0.000    1.000
##     GDP.Millions2    -0.000    0.345   -0.000    1.000
##     Nt_Pp._WB20152    0.000    0.295    0.000    1.000
##
## Variances:
##                    Estimate  Std.Err  z-value  P(>|z|)
##    .Attendance2       0.267    0.028    9.566    0.000
##    .Total.Animals2    0.251    0.044    5.729    0.000
##    .Sp.Richness2      0.151    0.046    3.269    0.001
##    .Prop.Thret.Sp2    0.991    0.135    7.335    0.000
##    .Brilloun.Indx2    0.290    0.079    3.656    0.000
##    .Mean.Rap.Crck2    0.704    0.063   11.098    0.000
##     Zoo.Area.ha2      0.998    0.112    8.886    0.000
##     Mam.Sp.Rchnss2    0.998    0.115    8.670    0.000
##     Prop.Mam.Sp2      0.998    0.150    6.662    0.000
##     Men.Sp.BdyMss2    0.998    0.173    5.783    0.000
```

```
##     X50km_Pop2        0.998    0.131    7.623    0.000
##     X10km_Pop2        0.998    0.174    5.745    0.000
##     GDP.Millions2     0.998    0.246    4.061    0.000
##     Nt_Pp._WB20152    0.998    0.225    4.439    0.000
##
## R-Square:
##                    Estimate
##     Attendance2       0.723
##     Total.Animals2    0.748
##     Sp.Richness2      0.849
##     Prop.Thret.Sp2    0.007
##     Brilloun.Indx2    0.709
##     Mean.Rap.Crck2    0.294
```

```r
# Generate fit indices
fitMeasures(fit.adj3, c("agfi", "rmr", "srmr", "rmsea", "cfi", "nnfi", "tli"))
```

```
##  agfi   rmr  srmr rmsea   cfi  nnfi   tli
## 0.669 0.083 0.079 0.149 0.876 0.799 0.799
```

```r
# Generate modification indices
mi3adj <- modindices(fit.adj3)
print(mi3adj[mi3adj$mi > 3.0,])
```

```
##                    lhs op               rhs     mi mi.scaled    epc sepc.lv
## 87      Total.Animals2 ~~   Prop.Threat.Sp2 17.524     4.178  0.101   0.101
## 89      Total.Animals2 ~~  Mean.Raup.Crick2  9.204     2.194 -0.175  -0.175
## 91        Sp.Richness2 ~~  Brillouin.Index2  8.649     2.062  0.031   0.031
## 92        Sp.Richness2 ~~  Mean.Raup.Crick2 27.606     6.582 -0.087  -0.087
## 93     Prop.Threat.Sp2 ~~  Brillouin.Index2  4.221     1.006  0.061   0.061
## 94     Prop.Threat.Sp2 ~~  Mean.Raup.Crick2 14.275     3.403 -0.147  -0.147
## 97      Total.Animals2  ~   Prop.Threat.Sp2 17.776     4.238  0.103   0.103
## 99      Total.Animals2  ~  Mean.Raup.Crick2  3.003     0.716 -0.081  -0.081
## 103     Total.Animals2  ~        X50km_Pop2 35.809     8.537  0.144   0.144
## 104     Total.Animals2  ~        X10km_Pop2  8.873     2.115  0.074   0.074
## 105     Total.Animals2  ~     GDP.Millions2 29.909     7.131 -0.128  -0.128
## 106     Total.Animals2  ~  Nat_Pop._WB20152  8.000     1.907 -0.066  -0.066
## 110       Sp.Richness2  ~  Brillouin.Index2  6.728     1.604  0.085   0.085
## 111       Sp.Richness2  ~  Mean.Raup.Crick2 17.010     4.055 -0.085  -0.085
## 116       Sp.Richness2  ~  Nat_Pop._WB20152  3.059     0.729 -0.032  -0.032
## 117    Prop.Threat.Sp2  ~       Attendance2 42.828    10.211  0.391   0.391
## 118    Prop.Threat.Sp2  ~    Total.Animals2 14.140     3.371  0.203   0.203
## 120    Prop.Threat.Sp2  ~  Mean.Raup.Crick2 24.260     5.784 -0.230  -0.230
## 121    Prop.Threat.Sp2  ~      Zoo.Area.ha2 10.359     2.470  0.150   0.150
## 122    Prop.Threat.Sp2  ~  Mam.Sp.Richness2 30.499     7.271  0.307   0.307
## 123    Prop.Threat.Sp2  ~       Prop.Mam.Sp2 47.215    11.256  0.356   0.356
## 124    Prop.Threat.Sp2  ~ Mean.Sp.BodyMass2 45.919    10.948  0.318   0.318
## 125    Prop.Threat.Sp2  ~        X50km_Pop2 19.706     4.698  0.210   0.210
## 126    Prop.Threat.Sp2  ~        X10km_Pop2 17.702     4.220  0.204   0.204
## 129   Brillouin.Index2  ~       Attendance2  8.181     1.950 -0.124  -0.124
## 130   Brillouin.Index2  ~   Prop.Threat.Sp2  4.221     1.006  0.062   0.062
## 134   Brillouin.Index2  ~      Prop.Mam.Sp2  9.791     2.334  0.092   0.092
## 137   Brillouin.Index2  ~        X10km_Pop2  4.078     0.972 -0.054  -0.054
## 138   Brillouin.Index2  ~     GDP.Millions2 58.529    13.954 -0.193  -0.193
## 139   Brillouin.Index2  ~  Nat_Pop._WB20152 50.587    12.061 -0.179  -0.179
## 140   Mean.Raup.Crick2  ~       Attendance2 19.322     4.606  0.394   0.394
```

```
## 141  Mean.Raup.Crick2  ~   Prop.Threat.Sp2 14.003     3.339 -0.147  -0.147
## 143  Mean.Raup.Crick2  ~      Zoo.Area.ha2  9.205     2.194  0.155   0.155
## 145  Mean.Raup.Crick2  ~      Prop.Mam.Sp2 28.655     6.832 -0.318  -0.318
## 146  Mean.Raup.Crick2  ~        X50km_Pop2 14.173     3.379  0.152   0.152
## 147  Mean.Raup.Crick2  ~        X10km_Pop2 19.575     4.667  0.187   0.187
## 149  Mean.Raup.Crick2  ~  Nat_Pop._WB20152 16.563     3.949  0.160   0.160
## 150      Zoo.Area.ha2  ~       Attendance2  4.180     0.997  0.369   0.369
## 152      Zoo.Area.ha2  ~      Sp.Richness2  9.363     2.232  0.602   0.602
## 154      Zoo.Area.ha2  ~  Brillouin.Index2  3.470     0.827  0.121   0.121
## 155      Zoo.Area.ha2  ~  Mean.Raup.Crick2  7.546     1.799  0.122   0.122
## 163  Mam.Sp.Richness2  ~       Attendance2  4.255     1.014 -0.372  -0.372
## 164  Mam.Sp.Richness2  ~     Total.Animals2  3.877     0.924 -0.190  -0.190
## 165  Mam.Sp.Richness2  ~      Sp.Richness2  9.521     2.270 -0.743  -0.743
## 166  Mam.Sp.Richness2  ~   Prop.Threat.Sp2  9.855     2.349  0.144   0.144
## 168  Mam.Sp.Richness2  ~  Mean.Raup.Crick2  4.940     1.178 -0.128  -0.128
## 179      Prop.Mam.Sp2  ~   Prop.Threat.Sp2 17.680     4.215  0.154   0.154
## 181      Prop.Mam.Sp2  ~  Mean.Raup.Crick2 11.046     2.634 -0.142  -0.142
## 192 Mean.Sp.BodyMass2  ~   Prop.Threat.Sp2  3.159     0.753  0.055   0.055
## 202        X50km_Pop2  ~       Attendance2 28.696     6.842  0.554   0.554
## 203        X50km_Pop2  ~     Total.Animals2 28.216     6.727  0.269   0.269
## 207        X50km_Pop2  ~  Mean.Raup.Crick2  3.649     0.870  0.065   0.065
## 228     GDP.Millions2  ~       Attendance2 14.273     3.403 -0.311  -0.311
## 230     GDP.Millions2  ~      Sp.Richness2 30.345     7.235  0.338   0.338
## 232     GDP.Millions2  ~  Brillouin.Index2  3.057     0.729  0.062   0.062
## 233     GDP.Millions2  ~  Mean.Raup.Crick2 16.858     4.019 -0.111  -0.111
## 242  Nat_Pop._WB20152  ~     Total.Animals2  3.776     0.900 -0.076  -0.076
## 243  Nat_Pop._WB20152  ~      Sp.Richness2 31.564     7.525 -0.333  -0.333
## 244  Nat_Pop._WB20152  ~   Prop.Threat.Sp2  3.603     0.859  0.044   0.044
## 245  Nat_Pop._WB20152  ~  Brillouin.Index2 15.508     3.697 -0.134  -0.134
## 246  Nat_Pop._WB20152  ~  Mean.Raup.Crick2 15.632     3.727  0.103   0.103
##     sepc.all sepc.nox
## 87     0.101    0.101
## 89    -0.175   -0.175
## 91     0.031    0.031
## 92    -0.087   -0.087
## 93     0.061    0.061
## 94    -0.148   -0.148
## 97     0.103    0.103
## 99    -0.081   -0.081
## 103    0.144    0.144
## 104    0.074    0.074
## 105   -0.128   -0.128
## 106   -0.066   -0.067
## 110    0.085    0.085
## 111   -0.085   -0.085
## 116   -0.032   -0.032
## 117    0.384    0.384
## 118    0.203    0.203
## 120   -0.230   -0.230
## 121    0.150    0.150
## 122    0.307    0.307
## 123    0.356    0.356
## 124    0.318    0.319
## 125    0.210    0.210
```

```
## 126    0.204    0.204
## 129   -0.122   -0.122
## 130    0.062    0.062
## 134    0.092    0.092
## 137   -0.054   -0.054
## 138   -0.193   -0.193
## 139   -0.179   -0.180
## 140    0.388    0.388
## 141   -0.147   -0.147
## 143    0.155    0.156
## 145   -0.318   -0.319
## 146    0.153    0.153
## 147    0.187    0.187
## 149    0.160    0.160
## 150    0.363    0.363
## 152    0.602    0.602
## 154    0.121    0.121
## 155    0.122    0.122
## 163   -0.366   -0.366
## 164   -0.190   -0.190
## 165   -0.743   -0.743
## 166    0.144    0.144
## 168   -0.128   -0.128
## 179    0.154    0.154
## 181   -0.142   -0.142
## 192    0.055    0.055
## 202    0.544    0.544
## 203    0.269    0.269
## 207    0.064    0.064
## 228   -0.306   -0.306
## 230    0.338    0.338
## 232    0.062    0.062
## 233   -0.111   -0.111
## 242   -0.076   -0.076
## 243   -0.333   -0.333
## 244    0.044    0.044
## 245   -0.134   -0.134
## 246    0.103    0.103
```

Model 4

Based on the modification indices generated from the third model, we can see that **Brillouin.Index2** ~ **GDP.Millions2** has the highest mi value of **58.529**. This far exceeds the standard cut-off level for the chi-square test criterion of 3.84 (Burnham and Anderson, 2002). This also makes theoretical sense, as it is logical to assume that countries with a higher GDP can afford to have much more diverse zoological collections. There is likely a historical element also at play here, however this is beyond what is deducible from the data available. As a result, we add this relationship to our model. Once again, the model summary, fit indices and modification indices were all generated for the model, adjusting for the nested nature of data.

```
# Attendance SEM (Presence-Absence)

# Model 4
# Addition of Brillouin.Index2 ~ GDP.Millions2, mi = 58.529
```

```r
mod.4 <- 'Attendance2 ~ Zoo.Area.ha2 + Sp.Richness2 + Total.Animals2
+ Mam.Sp.Richness2 + Prop.Mam.Sp2 + Prop.Threat.Sp2
+ Mean.Sp.BodyMass2 + Brillouin.Index2 + Mean.Raup.Crick2
+ X50km_Pop2 + X10km_Pop2 + GDP.Millions2 + Nat_Pop._WB20152

Total.Animals2 ~ Zoo.Area.ha2 + Sp.Richness2
Sp.Richness2 ~ Zoo.Area.ha2 + Prop.Mam.Sp2 + Mam.Sp.Richness2
Prop.Threat.Sp2 ~ Brillouin.Index2
Brillouin.Index2 ~ Sp.Richness2 + Total.Animals2 + GDP.Millions2
Mean.Raup.Crick2 ~ Sp.Richness2 + Total.Animals2 + Mean.Sp.BodyMass2'

# Fit model and generate model summary
mod.4.fit <- sem(mod.4, data = sem_attendance_data, fixed.x=FALSE)
summary(mod.4.fit, rsq = TRUE)
```

```
## lavaan (0.5-23.1097) converged normally after  54 iterations
##
##   Number of observations                           458
##
##   Estimator                                         ML
##   Minimum Function Test Statistic              370.335
##   Degrees of freedom                                38
##   P-value (Chi-square)                           0.000
##
## Parameter Estimates:
##
##   Information                                 Expected
##   Standard Errors                             Standard
##
## Regressions:
##                    Estimate  Std.Err  z-value  P(>|z|)
##   Attendance2 ~
##     Zoo.Area.ha2      0.086    0.033    2.593    0.010
##     Sp.Richness2     -0.249    0.097   -2.557    0.011
##     Total.Animals2    0.454    0.059    7.706    0.000
##     Mam.Sp.Rchnss2    0.132    0.055    2.403    0.016
##     Prop.Mam.Sp2     -0.065    0.049   -1.337    0.181
##     Prop.Thret.Sp2    0.009    0.024    0.371    0.711
##     Men.Sp.BdyMss2    0.339    0.039    8.644    0.000
##     Brilloun.Indx2    0.077    0.048    1.595    0.111
##     Mean.Rap.Crck2    0.146    0.029    5.054    0.000
##     X50km_Pop2        0.079    0.038    2.096    0.036
##     X10km_Pop2        0.397    0.041    9.624    0.000
##     GDP.Millions2     0.259    0.048    5.356    0.000
##     Nt_Pp._WB20152   -0.124    0.049   -2.523    0.012
##   Total.Animals2 ~
##     Zoo.Area.ha2      0.223    0.024    9.384    0.000
##     Sp.Richness2      0.798    0.024   33.520    0.000
##   Sp.Richness2 ~
##     Zoo.Area.ha2      0.067    0.021    3.225    0.001
##     Prop.Mam.Sp2     -0.581    0.019  -30.364    0.000
##     Mam.Sp.Rchnss2    0.755    0.020   38.373    0.000
##   Prop.Threat.Sp2 ~
```

```
##     Brilloun.Indx2     -0.084    0.046   -1.831    0.067
##   Brillouin.Index2 ~
##     Sp.Richness2        1.320    0.043   30.864    0.000
##     Total.Animals2     -0.620    0.043  -14.506    0.000
##     GDP.Millions2      -0.206    0.023   -8.791    0.000
##   Mean.Raup.Crick2 ~
##     Sp.Richness2       -0.355    0.074   -4.820    0.000
##     Total.Animals2      0.558    0.074    7.594    0.000
##     Men.Sp.BdyMss2     -0.495    0.040  -12.254    0.000
##
## Covariances:
##                        Estimate  Std.Err  z-value  P(>|z|)
##   Zoo.Area.ha2 ~~
##     Mam.Sp.Rchnss2        0.381    0.050    7.634    0.000
##     Prop.Mam.Sp2          0.312    0.049    6.389    0.000
##     Men.Sp.BdyMss2        0.534    0.053   10.096    0.000
##     X50km_Pop2            0.060    0.047    1.291    0.197
##     X10km_Pop2           -0.010    0.047   -0.208    0.835
##     GDP.Millions2        -0.027    0.047   -0.588    0.557
##     Nt_Pp._WB20152        0.061    0.047    1.310    0.190
##   Mam.Sp.Richness2 ~~
##     Prop.Mam.Sp2          0.088    0.047    1.882    0.060
##     Men.Sp.BdyMss2        0.303    0.049    6.216    0.000
##     X50km_Pop2            0.204    0.048    4.281    0.000
##     X10km_Pop2            0.284    0.048    5.852    0.000
##     GDP.Millions2        -0.060    0.047   -1.277    0.202
##     Nt_Pp._WB20152       -0.088    0.047   -1.881    0.060
##   Prop.Mam.Sp2 ~~
##     Men.Sp.BdyMss2        0.604    0.055   11.083    0.000
##     X50km_Pop2           -0.096    0.047   -2.054    0.040
##     X10km_Pop2           -0.204    0.048   -4.293    0.000
##     GDP.Millions2        -0.123    0.047   -2.615    0.009
##     Nt_Pp._WB20152       -0.115    0.047   -2.447    0.014
##   Mean.Sp.BodyMass2 ~~
##     X50km_Pop2            0.101    0.047    2.166    0.030
##     X10km_Pop2            0.126    0.047    2.683    0.007
##     GDP.Millions2        -0.082    0.047   -1.748    0.080
##     Nt_Pp._WB20152        0.006    0.047    0.124    0.901
##   X50km_Pop2 ~~
##     X10km_Pop2            0.752    0.058   12.886    0.000
##     GDP.Millions2        -0.013    0.047   -0.281    0.779
##     Nt_Pp._WB20152        0.191    0.047    4.033    0.000
##   X10km_Pop2 ~~
##     GDP.Millions2        -0.028    0.047   -0.604    0.546
##     Nt_Pp._WB20152        0.173    0.047    3.646    0.000
##   GDP.Millions2 ~~
##     Nt_Pp._WB20152        0.826    0.061   13.642    0.000
##
## Variances:
##                    Estimate  Std.Err  z-value  P(>|z|)
##    .Attendance2       0.267    0.018   15.133    0.000
##    .Total.Animals2    0.251    0.017   15.133    0.000
##    .Sp.Richness2      0.151    0.010   15.133    0.000
##    .Prop.Thret.Sp2    0.991    0.065   15.133    0.000
```

```
##    .Brilloun.Indx2    0.251    0.017   15.133    0.000
##    .Mean.Rap.Crck2    0.704    0.047   15.133    0.000
##     Zoo.Area.ha2      0.998    0.066   15.133    0.000
##     Mam.Sp.Rchnss2    0.998    0.066   15.133    0.000
##     Prop.Mam.Sp2      0.998    0.066   15.133    0.000
##     Men.Sp.BdyMss2    0.998    0.066   15.133    0.000
##     X50km_Pop2        0.998    0.066   15.133    0.000
##     X10km_Pop2        0.998    0.066   15.133    0.000
##     GDP.Millions2     0.998    0.066   15.133    0.000
##     Nt_Pp._WB20152    0.998    0.066   15.133    0.000
##
## R-Square:
##                    Estimate
##     Attendance2       0.721
##     Total.Animals2    0.748
##     Sp.Richness2      0.849
##     Prop.Thret.Sp2    0.007
##     Brilloun.Indx2    0.759
##     Mean.Rap.Crck2    0.294
```

```
# Generate fit indices
fitMeasures(mod.4.fit, c("agfi", "rmr", "srmr", "rmsea", "cfi", "nnfi", "tli"))
```

```
##  agfi   rmr  srmr rmsea   cfi  nnfi   tli
## 0.732 0.082 0.082 0.138 0.896 0.828 0.828
```

```
# Generate modification indices
mi4 <- modindices(mod.4.fit)
print(mi4[mi4$mi > 3.0,])
```

```
##                  lhs op               rhs     mi    epc sepc.lv sepc.all
## 74    Total.Animals2 ~~   Prop.Threat.Sp2 17.971  0.104   0.104    0.104
## 75    Total.Animals2 ~~  Brillouin.Index2  5.591 -0.069  -0.069   -0.068
## 76    Total.Animals2 ~~  Mean.Raup.Crick2  9.204 -0.175  -0.175   -0.175
## 78      Sp.Richness2 ~~  Brillouin.Index2 12.470  0.035   0.035    0.034
## 79      Sp.Richness2 ~~  Mean.Raup.Crick2 27.606 -0.087  -0.087   -0.087
## 80   Prop.Threat.Sp2 ~~  Brillouin.Index2  7.080  0.071   0.071    0.070
## 81   Prop.Threat.Sp2 ~~  Mean.Raup.Crick2 14.275 -0.147  -0.147   -0.148
## 84    Total.Animals2  ~   Prop.Threat.Sp2 17.478  0.103   0.103    0.103
## 85    Total.Animals2  ~  Brillouin.Index2  5.096  0.184   0.184    0.188
## 86    Total.Animals2  ~  Mean.Raup.Crick2  3.003 -0.081  -0.081   -0.081
## 90    Total.Animals2  ~        X50km_Pop2 35.809  0.144   0.144    0.144
## 91    Total.Animals2  ~        X10km_Pop2  8.873  0.074   0.074    0.074
## 92    Total.Animals2  ~     GDP.Millions2 29.909 -0.128  -0.128   -0.128
## 93    Total.Animals2  ~  Nat_Pop._WB20152  8.000 -0.066  -0.066   -0.066
## 97      Sp.Richness2  ~  Brillouin.Index2  5.177  0.071   0.071    0.073
## 98      Sp.Richness2  ~  Mean.Raup.Crick2 17.010 -0.085  -0.085   -0.085
## 103     Sp.Richness2  ~  Nat_Pop._WB20152  3.059 -0.032  -0.032   -0.032
## 104  Prop.Threat.Sp2  ~       Attendance2 41.612  0.380   0.380    0.372
## 105  Prop.Threat.Sp2  ~    Total.Animals2 13.574  0.195   0.195    0.195
## 107  Prop.Threat.Sp2  ~  Mean.Raup.Crick2 24.211 -0.230  -0.230   -0.229
## 108  Prop.Threat.Sp2  ~      Zoo.Area.ha2 10.355  0.150   0.150    0.150
## 109  Prop.Threat.Sp2  ~  Mam.Sp.Richness2 30.651  0.309   0.309    0.309
## 110  Prop.Threat.Sp2  ~      Prop.Mam.Sp2 46.213  0.348   0.348    0.348
## 111  Prop.Threat.Sp2  ~ Mean.Sp.BodyMass2 45.833  0.318   0.318    0.318
## 112  Prop.Threat.Sp2  ~        X50km_Pop2 19.712  0.210   0.210    0.210
```

```
## 113   Prop.Threat.Sp2  ~        X10km_Pop2 17.755  0.204   0.204    0.204
## 117  Brillouin.Index2  ~   Prop.Threat.Sp2  7.080  0.072   0.072    0.070
## 119  Brillouin.Index2  ~      Zoo.Area.ha2  5.591  0.061   0.061    0.060
## 121  Brillouin.Index2  ~      Prop.Mam.Sp2  7.549  0.076   0.076    0.074
## 124  Brillouin.Index2  ~        X10km_Pop2  5.633 -0.059  -0.059   -0.058
## 126  Mean.Raup.Crick2  ~       Attendance2 19.786  0.403   0.403    0.396
## 127  Mean.Raup.Crick2  ~   Prop.Threat.Sp2 14.003 -0.147  -0.147   -0.147
## 129  Mean.Raup.Crick2  ~      Zoo.Area.ha2  9.205  0.155   0.155    0.155
## 131  Mean.Raup.Crick2  ~      Prop.Mam.Sp2 28.655 -0.318  -0.318   -0.318
## 132  Mean.Raup.Crick2  ~        X50km_Pop2 14.173  0.152   0.152    0.153
## 133  Mean.Raup.Crick2  ~        X10km_Pop2 19.575  0.187   0.187    0.187
## 135  Mean.Raup.Crick2  ~  Nat_Pop._WB20152 16.563  0.160   0.160    0.160
## 136      Zoo.Area.ha2  ~       Attendance2  4.816  0.400   0.400    0.392
## 138      Zoo.Area.ha2  ~      Sp.Richness2  9.363  0.602   0.602    0.602
## 140      Zoo.Area.ha2  ~  Brillouin.Index2  6.547  0.173   0.173    0.177
## 141      Zoo.Area.ha2  ~  Mean.Raup.Crick2  7.546  0.122   0.122    0.122
## 149  Mam.Sp.Richness2  ~       Attendance2  4.643 -0.394  -0.394   -0.386
## 150  Mam.Sp.Richness2  ~     Total.Animals2  3.877 -0.190  -0.190   -0.190
## 151  Mam.Sp.Richness2  ~      Sp.Richness2  9.521 -0.742  -0.742   -0.742
## 152  Mam.Sp.Richness2  ~   Prop.Threat.Sp2  9.946  0.144   0.144    0.144
## 154  Mam.Sp.Richness2  ~  Mean.Raup.Crick2  4.940 -0.128  -0.128   -0.128
## 165      Prop.Mam.Sp2  ~   Prop.Threat.Sp2 17.572  0.153   0.153    0.153
## 166      Prop.Mam.Sp2  ~  Brillouin.Index2  3.198  0.107   0.107    0.109
## 167      Prop.Mam.Sp2  ~  Mean.Raup.Crick2 11.046 -0.142  -0.142   -0.142
## 178 Mean.Sp.BodyMass2  ~   Prop.Threat.Sp2  3.159  0.055   0.055    0.055
## 188        X50km_Pop2  ~       Attendance2 29.375  0.567   0.567    0.555
## 189        X50km_Pop2  ~     Total.Animals2 28.216  0.269   0.269    0.269
## 193        X50km_Pop2  ~  Mean.Raup.Crick2  3.649  0.065   0.065    0.064
## 214      GDP.Millions2  ~       Attendance2 10.433 -0.270  -0.270   -0.265
## 216      GDP.Millions2  ~      Sp.Richness2 30.346  0.338   0.338    0.338
## 218      GDP.Millions2  ~  Brillouin.Index2 30.672  0.213   0.213    0.217
## 219      GDP.Millions2  ~  Mean.Raup.Crick2 16.858 -0.111  -0.111   -0.111
## 228  Nat_Pop._WB20152  ~     Total.Animals2  3.776 -0.076  -0.076   -0.076
## 229  Nat_Pop._WB20152  ~      Sp.Richness2 31.565 -0.333  -0.333   -0.333
## 230  Nat_Pop._WB20152  ~   Prop.Threat.Sp2  3.603  0.044   0.044    0.044
## 231  Nat_Pop._WB20152  ~  Brillouin.Index2 15.828 -0.137  -0.137   -0.139
## 232  Nat_Pop._WB20152  ~  Mean.Raup.Crick2 15.632  0.103   0.103    0.103
##     sepc.nox
## 74     0.104
## 75    -0.068
## 76    -0.175
## 78     0.034
## 79    -0.087
## 80     0.070
## 81    -0.148
## 84     0.103
## 85     0.188
## 86    -0.081
## 90     0.144
## 91     0.074
## 92    -0.128
## 93    -0.067
## 97     0.073
## 98    -0.085
```

```
## 103   -0.032
## 104    0.372
## 105    0.195
## 107   -0.229
## 108    0.150
## 109    0.309
## 110    0.349
## 111    0.318
## 112    0.210
## 113    0.204
## 117    0.070
## 119    0.060
## 121    0.075
## 124   -0.058
## 126    0.396
## 127   -0.147
## 129    0.156
## 131   -0.319
## 132    0.153
## 133    0.187
## 135    0.160
## 136    0.392
## 138    0.602
## 140    0.177
## 141    0.122
## 149   -0.386
## 150   -0.190
## 151   -0.742
## 152    0.144
## 154   -0.128
## 165    0.153
## 166    0.109
## 167   -0.142
## 178    0.055
## 188    0.555
## 189    0.269
## 193    0.064
## 214   -0.265
## 216    0.338
## 218    0.217
## 219   -0.111
## 228   -0.076
## 229   -0.333
## 230    0.044
## 231   -0.139
## 232    0.103
```

```r
# Adjust for the nested nature of the data (institutions within countries)
# Fit model and generate model summary
design <- svydesign(ids = ~Country, nest=TRUE, data=sem_attendance_data)
fit.adj4 <- lavaan.survey(lavaan.fit = mod.4.fit, survey.design = design)
summary(fit.adj4, rsq = TRUE)
```

```
## lavaan (0.5-23.1097) converged normally after  54 iterations
##
```

```
##   Number of observations                           458
##
##   Estimator                                         ML      Robust
##   Minimum Function Test Statistic              370.335      92.345
##   Degrees of freedom                                38          38
##   P-value (Chi-square)                           0.000       0.000
##   Scaling correction factor                                  4.010
##     for the Satorra-Bentler correction
##
## Parameter Estimates:
##
##   Information                                 Expected
##   Standard Errors                           Robust.sem
##
## Regressions:
##                    Estimate  Std.Err  z-value  P(>|z|)
##   Attendance2 ~
##     Zoo.Area.ha2       0.086    0.041    2.115    0.034
##     Sp.Richness2      -0.249    0.102   -2.449    0.014
##     Total.Animals2     0.454    0.062    7.316    0.000
##     Mam.Sp.Rchnss2     0.132    0.049    2.712    0.007
##     Prop.Mam.Sp2      -0.065    0.044   -1.475    0.140
##     Prop.Thret.Sp2     0.009    0.024    0.371    0.710
##     Men.Sp.BdyMss2     0.339    0.030   11.327    0.000
##     Brilloun.Indx2     0.077    0.065    1.176    0.240
##     Mean.Rap.Crck2     0.146    0.029    5.002    0.000
##     X50km_Pop2         0.079    0.033    2.363    0.018
##     X10km_Pop2         0.397    0.042    9.352    0.000
##     GDP.Millions2      0.259    0.060    4.315    0.000
##     Nt_Pp._WB20152    -0.124    0.067   -1.845    0.065
##   Total.Animals2 ~
##     Zoo.Area.ha2       0.223    0.038    5.856    0.000
##     Sp.Richness2       0.798    0.048   16.678    0.000
##   Sp.Richness2 ~
##     Zoo.Area.ha2       0.067    0.044    1.519    0.129
##     Prop.Mam.Sp2      -0.581    0.038  -15.231    0.000
##     Mam.Sp.Rchnss2     0.755    0.068   11.066    0.000
##   Prop.Threat.Sp2 ~
##     Brilloun.Indx2    -0.084    0.097   -0.859    0.390
##   Brillouin.Index2 ~
##     Sp.Richness2       1.320    0.069   19.079    0.000
##     Total.Animals2    -0.620    0.102   -6.085    0.000
##     GDP.Millions2     -0.206    0.059   -3.492    0.000
##   Mean.Raup.Crick2 ~
##     Sp.Richness2      -0.355    0.228   -1.555    0.120
##     Total.Animals2     0.558    0.153    3.656    0.000
##     Men.Sp.BdyMss2    -0.495    0.052   -9.463    0.000
##
## Covariances:
##                      Estimate  Std.Err  z-value  P(>|z|)
##   Zoo.Area.ha2 ~~
##     Mam.Sp.Rchnss2       0.381    0.064    5.985    0.000
##     Prop.Mam.Sp2         0.312    0.089    3.510    0.000
##     Men.Sp.BdyMss2       0.534    0.104    5.147    0.000
```

```
##     X50km_Pop2           0.060    0.069    0.880    0.379
##     X10km_Pop2          -0.010    0.078   -0.124    0.901
##     GDP.Millions2       -0.027    0.041   -0.673    0.501
##     Nt_Pp._WB20152       0.061    0.090    0.682    0.495
##   Mam.Sp.Richness2 ~~
##     Prop.Mam.Sp2         0.088    0.061    1.435    0.151
##     Men.Sp.BdyMss2       0.303    0.081    3.730    0.000
##     X50km_Pop2           0.204    0.082    2.487    0.013
##     X10km_Pop2           0.284    0.066    4.278    0.000
##     GDP.Millions2       -0.060    0.064   -0.937    0.349
##     Nt_Pp._WB20152      -0.088    0.102   -0.863    0.388
##   Prop.Mam.Sp2 ~~
##     Men.Sp.BdyMss2       0.604    0.130    4.656    0.000
##     X50km_Pop2          -0.096    0.051   -1.888    0.059
##     X10km_Pop2          -0.204    0.055   -3.685    0.000
##     GDP.Millions2       -0.123    0.105   -1.172    0.241
##     Nt_Pp._WB20152      -0.115    0.091   -1.257    0.209
##   Mean.Sp.BodyMass2 ~~
##     X50km_Pop2           0.101    0.072    1.400    0.161
##     X10km_Pop2           0.126    0.067    1.895    0.058
##     GDP.Millions2       -0.082    0.046   -1.759    0.079
##     Nt_Pp._WB20152       0.006    0.092    0.063    0.950
##   X50km_Pop2 ~~
##     X10km_Pop2           0.752    0.126    5.982    0.000
##     GDP.Millions2       -0.013    0.100   -0.131    0.896
##     Nt_Pp._WB20152       0.191    0.177    1.083    0.279
##   X10km_Pop2 ~~
##     GDP.Millions2       -0.028    0.056   -0.505    0.613
##     Nt_Pp._WB20152       0.173    0.101    1.701    0.089
##   GDP.Millions2 ~~
##     Nt_Pp._WB20152       0.826    0.202    4.089    0.000
##
## Intercepts:
##                    Estimate  Std.Err  z-value  P(>|z|)
##    .Attendance2      -0.000    0.031   -0.000    1.000
##    .Total.Animals2   -0.000    0.104   -0.000    1.000
##    .Sp.Richness2      0.000    0.034    0.000    1.000
##    .Prop.Thret.Sp2    0.000    0.099    0.000    1.000
##    .Brilloun.Indx2   -0.000    0.055   -0.000    1.000
##    .Mean.Rap.Crck2    0.000    0.094    0.000    1.000
##     Zoo.Area.ha2      0.000    0.064    0.000    1.000
##     Mam.Sp.Rchnss2   -0.000    0.079   -0.000    1.000
##     Prop.Mam.Sp2      0.000    0.118    0.000    1.000
##     Men.Sp.BdyMss2   -0.000    0.080   -0.000    1.000
##     X50km_Pop2       -0.000    0.114   -0.000    1.000
##     X10km_Pop2        0.000    0.105    0.000    1.000
##     GDP.Millions2    -0.000    0.345   -0.000    1.000
##     Nt_Pp._WB20152    0.000    0.295    0.000    1.000
##
## Variances:
##                    Estimate  Std.Err  z-value  P(>|z|)
##    .Attendance2       0.267    0.028    9.566    0.000
##    .Total.Animals2    0.251    0.044    5.729    0.000
##    .Sp.Richness2      0.151    0.046    3.269    0.001
```

```
##    .Prop.Thret.Sp2    0.991    0.135    7.335    0.000
##    .Brilloun.Indx2    0.251    0.053    4.691    0.000
##    .Mean.Rap.Crck2    0.704    0.063   11.098    0.000
##     Zoo.Area.ha2      0.998    0.112    8.886    0.000
##     Mam.Sp.Rchnss2    0.998    0.115    8.669    0.000
##     Prop.Mam.Sp2      0.998    0.150    6.662    0.000
##     Men.Sp.BdyMss2    0.998    0.173    5.783    0.000
##     X50km_Pop2        0.998    0.131    7.623    0.000
##     X10km_Pop2        0.998    0.174    5.745    0.000
##     GDP.Millions2     0.998    0.246    4.061    0.000
##     Nt_Pp._WB20152    0.998    0.225    4.439    0.000
##
## R-Square:
##                    Estimate
##     Attendance2       0.721
##     Total.Animals2    0.748
##     Sp.Richness2      0.849
##     Prop.Thret.Sp2    0.007
##     Brilloun.Indx2    0.759
##     Mean.Rap.Crck2    0.294
```

```r
# Generate fit indices
fitMeasures(fit.adj4, c("agfi", "rmr", "srmr", "rmsea", "cfi", "nnfi", "tli"))
```

```
##  agfi   rmr  srmr rmsea   cfi  nnfi   tli
## 0.696 0.082 0.077 0.138 0.896 0.828 0.828
```

```r
# Generate modification indices
mi4adj <- modindices(fit.adj4)
print(mi4adj[mi4adj$mi > 3.0,])
```

```
##                  lhs op              rhs     mi mi.scaled    epc sepc.lv
## 88     Total.Animals2 ~~   Prop.Threat.Sp2 17.971     4.481  0.104   0.104
## 89     Total.Animals2 ~~  Brillouin.Index2  5.591     1.394 -0.069  -0.069
## 90     Total.Animals2 ~~  Mean.Raup.Crick2  9.205     2.295 -0.175  -0.175
## 92       Sp.Richness2 ~~  Brillouin.Index2 12.470     3.109  0.035   0.035
## 93       Sp.Richness2 ~~  Mean.Raup.Crick2 27.606     6.884 -0.087  -0.087
## 94    Prop.Threat.Sp2 ~~  Brillouin.Index2  7.080     1.765  0.071   0.071
## 95    Prop.Threat.Sp2 ~~  Mean.Raup.Crick2 14.275     3.559 -0.147  -0.147
## 98     Total.Animals2  ~   Prop.Threat.Sp2 17.478     4.358  0.103   0.103
## 99     Total.Animals2  ~  Brillouin.Index2  5.096     1.271  0.184   0.184
## 100    Total.Animals2  ~  Mean.Raup.Crick2  3.003     0.749 -0.081  -0.081
## 104    Total.Animals2  ~        X50km_Pop2 35.809     8.929  0.144   0.144
## 105    Total.Animals2  ~        X10km_Pop2  8.873     2.213  0.074   0.074
## 106    Total.Animals2  ~     GDP.Millions2 29.909     7.458 -0.128  -0.128
## 107    Total.Animals2  ~  Nat_Pop._WB20152  8.000     1.995 -0.066  -0.066
## 111      Sp.Richness2  ~  Brillouin.Index2  5.177     1.291  0.071   0.071
## 112      Sp.Richness2  ~  Mean.Raup.Crick2 17.010     4.242 -0.085  -0.085
## 117      Sp.Richness2  ~  Nat_Pop._WB20152  3.059     0.763 -0.032  -0.032
## 118   Prop.Threat.Sp2  ~       Attendance2 41.612    10.376  0.380   0.380
## 119   Prop.Threat.Sp2  ~    Total.Animals2 13.574     3.385  0.195   0.195
## 121   Prop.Threat.Sp2  ~  Mean.Raup.Crick2 24.211     6.037 -0.230  -0.230
## 122   Prop.Threat.Sp2  ~      Zoo.Area.ha2 10.355     2.582  0.150   0.150
## 123   Prop.Threat.Sp2  ~  Mam.Sp.Richness2 30.651     7.643  0.309   0.309
## 124   Prop.Threat.Sp2  ~      Prop.Mam.Sp2 46.213    11.523  0.348   0.348
## 125   Prop.Threat.Sp2  ~ Mean.Sp.BodyMass2 45.833    11.429  0.318   0.318
```

```
## 126   Prop.Threat.Sp2  ~        X50km_Pop2 19.712     4.915  0.210   0.210
## 127   Prop.Threat.Sp2  ~        X10km_Pop2 17.755     4.427  0.204   0.204
## 131  Brillouin.Index2  ~   Prop.Threat.Sp2  7.080     1.765  0.072   0.072
## 133  Brillouin.Index2  ~      Zoo.Area.ha2  5.591     1.394  0.061   0.061
## 135  Brillouin.Index2  ~      Prop.Mam.Sp2  7.549     1.882  0.076   0.076
## 138  Brillouin.Index2  ~        X10km_Pop2  5.633     1.405 -0.059  -0.059
## 140  Mean.Raup.Crick2  ~       Attendance2 19.786     4.934  0.403   0.403
## 141  Mean.Raup.Crick2  ~   Prop.Threat.Sp2 14.003     3.492 -0.147  -0.147
## 143  Mean.Raup.Crick2  ~      Zoo.Area.ha2  9.205     2.295  0.155   0.155
## 145  Mean.Raup.Crick2  ~      Prop.Mam.Sp2 28.655     7.145 -0.318  -0.318
## 146  Mean.Raup.Crick2  ~        X50km_Pop2 14.173     3.534  0.152   0.152
## 147  Mean.Raup.Crick2  ~        X10km_Pop2 19.575     4.881  0.187   0.187
## 149  Mean.Raup.Crick2  ~  Nat_Pop._WB20152 16.563     4.130  0.160   0.160
## 150      Zoo.Area.ha2  ~       Attendance2  4.816     1.201  0.400   0.400
## 152      Zoo.Area.ha2  ~      Sp.Richness2  9.363     2.335  0.602   0.602
## 154      Zoo.Area.ha2  ~  Brillouin.Index2  6.547     1.633  0.173   0.173
## 155      Zoo.Area.ha2  ~  Mean.Raup.Crick2  7.546     1.882  0.122   0.122
## 163  Mam.Sp.Richness2  ~       Attendance2  4.643     1.158 -0.394  -0.394
## 164  Mam.Sp.Richness2  ~     Total.Animals2  3.877     0.967 -0.190  -0.190
## 165  Mam.Sp.Richness2  ~      Sp.Richness2  9.521     2.374 -0.742  -0.742
## 166  Mam.Sp.Richness2  ~   Prop.Threat.Sp2  9.946     2.480  0.144   0.144
## 168  Mam.Sp.Richness2  ~  Mean.Raup.Crick2  4.940     1.232 -0.128  -0.128
## 179      Prop.Mam.Sp2  ~   Prop.Threat.Sp2 17.572     4.382  0.153   0.153
## 180      Prop.Mam.Sp2  ~  Brillouin.Index2  3.198     0.798  0.107   0.107
## 181      Prop.Mam.Sp2  ~  Mean.Raup.Crick2 11.046     2.754 -0.142  -0.142
## 192 Mean.Sp.BodyMass2  ~   Prop.Threat.Sp2  3.159     0.788  0.055   0.055
## 202        X50km_Pop2  ~       Attendance2 29.374     7.325  0.567   0.567
## 203        X50km_Pop2  ~     Total.Animals2 28.216     7.036  0.269   0.269
## 207        X50km_Pop2  ~  Mean.Raup.Crick2  3.649     0.910  0.065   0.065
## 228      GDP.Millions2  ~       Attendance2 10.433     2.602 -0.270  -0.270
## 230      GDP.Millions2  ~      Sp.Richness2 30.346     7.567  0.338   0.338
## 232      GDP.Millions2  ~  Brillouin.Index2 30.672     7.648  0.213   0.213
## 233      GDP.Millions2  ~  Mean.Raup.Crick2 16.858     4.204 -0.111  -0.111
## 242  Nat_Pop._WB20152  ~     Total.Animals2  3.776     0.942 -0.076  -0.076
## 243  Nat_Pop._WB20152  ~      Sp.Richness2 31.564     7.871 -0.333  -0.333
## 244  Nat_Pop._WB20152  ~   Prop.Threat.Sp2  3.603     0.898  0.044   0.044
## 245  Nat_Pop._WB20152  ~  Brillouin.Index2 15.828     3.947 -0.137  -0.137
## 246  Nat_Pop._WB20152  ~  Mean.Raup.Crick2 15.632     3.898  0.103   0.103
##     sepc.all sepc.nox
## 88     0.104    0.104
## 89    -0.068   -0.068
## 90    -0.175   -0.175
## 92     0.034    0.034
## 93    -0.087   -0.087
## 94     0.070    0.070
## 95    -0.148   -0.148
## 98     0.103    0.103
## 99     0.188    0.188
## 100   -0.081   -0.081
## 104    0.144    0.144
## 105    0.074    0.074
## 106   -0.128   -0.128
## 107   -0.066   -0.067
## 111    0.073    0.073
```

```
## 112   -0.085   -0.085
## 117   -0.032   -0.032
## 118    0.372    0.372
## 119    0.195    0.195
## 121   -0.229   -0.229
## 122    0.150    0.150
## 123    0.309    0.309
## 124    0.348    0.349
## 125    0.318    0.318
## 126    0.210    0.210
## 127    0.204    0.204
## 131    0.070    0.070
## 133    0.060    0.060
## 135    0.074    0.075
## 138   -0.058   -0.058
## 140    0.396    0.396
## 141   -0.147   -0.147
## 143    0.155    0.156
## 145   -0.318   -0.319
## 146    0.153    0.153
## 147    0.187    0.187
## 149    0.160    0.160
## 150    0.392    0.392
## 152    0.602    0.602
## 154    0.177    0.177
## 155    0.122    0.122
## 163   -0.386   -0.386
## 164   -0.190   -0.190
## 165   -0.742   -0.742
## 166    0.144    0.144
## 168   -0.128   -0.128
## 179    0.153    0.153
## 180    0.109    0.109
## 181   -0.142   -0.142
## 192    0.055    0.055
## 202    0.555    0.555
## 203    0.269    0.269
## 207    0.064    0.064
## 228   -0.265   -0.265
## 230    0.338    0.338
## 232    0.217    0.217
## 233   -0.111   -0.111
## 242   -0.076   -0.076
## 243   -0.333   -0.333
## 244    0.044    0.044
## 245   -0.139   -0.139
## 246    0.103    0.103
```

Model 5

Based on the modification indices generated from the fourth model, we can see that **Prop.Threat.Sp2** ~ **Prop.Mam.Sp2** has the highest mi value of **46.213**. This far exceeds the standard cut-off level for the chi-square test criterion of 3.84 (Burnham and Anderson, 2002). As contemporary zoological collections are

taxonomically biased towards mammals, and particularly biased towards mammalian species which are often less threatened than their close relatives not held in zoos (Martin *et al.*, 2014), this suggestion should not come as a surprise. As a result, we add this relationship to our model. Once again, the model summary, fit indices and modification indices were all generated for the model, adjusting for the nested nature of data.

```r
# Attendance SEM (Presence-Absence)

# Model 5
# Addition of Prop.Threat.Sp2 ~ Prop.Mam.Sp2, mi = 46.213

mod.5 <- 'Attendance2 ~ Zoo.Area.ha2 + Sp.Richness2 + Total.Animals2
+ Mam.Sp.Richness2 + Prop.Mam.Sp2 + Prop.Threat.Sp2
+ Mean.Sp.BodyMass2 + Brillouin.Index2 + Mean.Raup.Crick2
+ X50km_Pop2 + X10km_Pop2 + GDP.Millions2 + Nat_Pop._WB20152

Total.Animals2 ~ Zoo.Area.ha2 + Sp.Richness2
Sp.Richness2 ~ Zoo.Area.ha2 + Prop.Mam.Sp2 + Mam.Sp.Richness2
Prop.Threat.Sp2 ~ Brillouin.Index2 + Prop.Mam.Sp2
Brillouin.Index2 ~ Sp.Richness2 + Total.Animals2 + GDP.Millions2
Mean.Raup.Crick2 ~ Sp.Richness2 + Total.Animals2 + Mean.Sp.BodyMass2'

# Fit model and generate model summary
mod.5.fit <- sem(mod.5, data = sem_attendance_data, fixed.x=FALSE)
summary(mod.5.fit, rsq = TRUE)
```

```
## lavaan (0.5-23.1097) converged normally after  59 iterations
##
##   Number of observations                           458
##
##   Estimator                                         ML
##   Minimum Function Test Statistic              324.001
##   Degrees of freedom                                37
##   P-value (Chi-square)                           0.000
##
## Parameter Estimates:
##
##   Information                                 Expected
##   Standard Errors                             Standard
##
## Regressions:
##                     Estimate  Std.Err  z-value  P(>|z|)
##   Attendance2 ~
##     Zoo.Area.ha2        0.086    0.033    2.593    0.010
##     Sp.Richness2       -0.249    0.097   -2.557    0.011
##     Total.Animals2      0.454    0.059    7.706    0.000
##     Mam.Sp.Rchnss2      0.132    0.055    2.403    0.016
##     Prop.Mam.Sp2       -0.065    0.050   -1.317    0.188
##     Prop.Thret.Sp2      0.009    0.026    0.353    0.724
##     Men.Sp.BdyMss2      0.339    0.039    8.644    0.000
##     Brilloun.Indx2      0.077    0.048    1.596    0.111
##     Mean.Rap.Crck2      0.146    0.029    5.054    0.000
##     X50km_Pop2          0.079    0.038    2.096    0.036
##     X10km_Pop2          0.397    0.041    9.624    0.000
##     GDP.Millions2       0.259    0.048    5.356    0.000
##     Nt_Pp._WB20152     -0.124    0.049   -2.523    0.012
```

```
##   Total.Animals2 ~
##     Zoo.Area.ha2        0.223    0.024    9.384    0.000
##     Sp.Richness2        0.798    0.024   33.520    0.000
##   Sp.Richness2 ~
##     Zoo.Area.ha2        0.067    0.021    3.225    0.001
##     Prop.Mam.Sp2       -0.581    0.019  -30.364    0.000
##     Mam.Sp.Rchnss2      0.755    0.020   38.373    0.000
##   Prop.Threat.Sp2 ~
##     Brilloun.Indx2      0.038    0.048    0.792    0.428
##     Prop.Mam.Sp2        0.332    0.049    6.819    0.000
##   Brillouin.Index2 ~
##     Sp.Richness2        1.320    0.043   30.864    0.000
##     Total.Animals2     -0.620    0.043  -14.506    0.000
##     GDP.Millions2      -0.206    0.023   -8.791    0.000
##   Mean.Raup.Crick2 ~
##     Sp.Richness2       -0.355    0.074   -4.820    0.000
##     Total.Animals2      0.558    0.074    7.594    0.000
##     Men.Sp.BdyMss2     -0.495    0.040  -12.254    0.000
##
## Covariances:
##                        Estimate  Std.Err  z-value  P(>|z|)
##   Zoo.Area.ha2 ~~
##     Mam.Sp.Rchnss2        0.381    0.050    7.634    0.000
##     Prop.Mam.Sp2          0.312    0.049    6.389    0.000
##     Men.Sp.BdyMss2        0.534    0.053   10.096    0.000
##     X50km_Pop2            0.060    0.047    1.291    0.197
##     X10km_Pop2           -0.010    0.047   -0.208    0.835
##     GDP.Millions2        -0.027    0.047   -0.588    0.557
##     Nt_Pp._WB20152        0.061    0.047    1.310    0.190
##   Mam.Sp.Richness2 ~~
##     Prop.Mam.Sp2          0.088    0.047    1.882    0.060
##     Men.Sp.BdyMss2        0.303    0.049    6.216    0.000
##     X50km_Pop2            0.204    0.048    4.281    0.000
##     X10km_Pop2            0.284    0.048    5.852    0.000
##     GDP.Millions2        -0.060    0.047   -1.277    0.202
##     Nt_Pp._WB20152       -0.088    0.047   -1.881    0.060
##   Prop.Mam.Sp2 ~~
##     Men.Sp.BdyMss2        0.604    0.055   11.083    0.000
##     X50km_Pop2           -0.096    0.047   -2.054    0.040
##     X10km_Pop2           -0.204    0.048   -4.293    0.000
##     GDP.Millions2        -0.123    0.047   -2.615    0.009
##     Nt_Pp._WB20152       -0.115    0.047   -2.447    0.014
##   Mean.Sp.BodyMass2 ~~
##     X50km_Pop2            0.101    0.047    2.166    0.030
##     X10km_Pop2            0.126    0.047    2.683    0.007
##     GDP.Millions2        -0.082    0.047   -1.748    0.080
##     Nt_Pp._WB20152        0.006    0.047    0.124    0.901
##   X50km_Pop2 ~~
##     X10km_Pop2            0.752    0.058   12.886    0.000
##     GDP.Millions2        -0.013    0.047   -0.281    0.779
##     Nt_Pp._WB20152        0.191    0.047    4.033    0.000
##   X10km_Pop2 ~~
##     GDP.Millions2        -0.028    0.047   -0.604    0.546
##     Nt_Pp._WB20152        0.173    0.047    3.646    0.000
```

```
##   GDP.Millions2 ~~
##     Nt_Pp._WB20152        0.826    0.061   13.642    0.000
##
## Variances:
##                    Estimate  Std.Err  z-value  P(>|z|)
##    .Attendance2       0.267    0.018   15.133    0.000
##    .Total.Animals2    0.251    0.017   15.133    0.000
##    .Sp.Richness2      0.151    0.010   15.133    0.000
##    .Prop.Thret.Sp2    0.896    0.059   15.133    0.000
##    .Brilloun.Indx2    0.251    0.017   15.133    0.000
##    .Mean.Rap.Crck2    0.704    0.047   15.133    0.000
##     Zoo.Area.ha2      0.998    0.066   15.133    0.000
##     Mam.Sp.Rchnss2    0.998    0.066   15.133    0.000
##     Prop.Mam.Sp2      0.998    0.066   15.133    0.000
##     Men.Sp.BdyMss2    0.998    0.066   15.133    0.000
##     X50km_Pop2        0.998    0.066   15.133    0.000
##     X10km_Pop2        0.998    0.066   15.133    0.000
##     GDP.Millions2     0.998    0.066   15.133    0.000
##     Nt_Pp._WB20152    0.998    0.066   15.133    0.000
##
## R-Square:
##                    Estimate
##     Attendance2       0.721
##     Total.Animals2    0.748
##     Sp.Richness2      0.849
##     Prop.Thret.Sp2    0.101
##     Brilloun.Indx2    0.759
##     Mean.Rap.Crck2    0.294
```

```r
# Generate fit indices
fitMeasures(mod.5.fit, c("agfi", "rmr", "srmr", "rmsea", "cfi", "nnfi", "tli"))
```

```
##  agfi   rmr  srmr rmsea   cfi  nnfi   tli
## 0.757 0.069 0.069 0.130 0.910 0.847 0.847
```

```r
# Generate modification indices
mi5 <- modindices(mod.5.fit)
print(mi5[mi5$mi > 3.0,])
```

```
##                   lhs op               rhs     mi    epc sepc.lv sepc.all
## 75     Total.Animals2 ~~   Prop.Threat.Sp2 29.840  0.129   0.129    0.129
## 76     Total.Animals2 ~~  Brillouin.Index2  5.591 -0.069  -0.069   -0.068
## 77     Total.Animals2 ~~  Mean.Raup.Crick2  9.205 -0.175  -0.175   -0.175
## 79       Sp.Richness2 ~~  Brillouin.Index2 12.470  0.035   0.035    0.034
## 80       Sp.Richness2 ~~  Mean.Raup.Crick2 27.606 -0.087  -0.087   -0.087
## 82    Prop.Threat.Sp2 ~~  Mean.Raup.Crick2  7.213 -0.100  -0.100   -0.100
## 85     Total.Animals2  ~   Prop.Threat.Sp2 26.465  0.130   0.130    0.130
## 86     Total.Animals2  ~  Brillouin.Index2  5.096  0.184   0.184    0.188
## 87     Total.Animals2  ~  Mean.Raup.Crick2  3.003 -0.081  -0.081   -0.081
## 91     Total.Animals2  ~        X50km_Pop2 35.809  0.144   0.144    0.144
## 92     Total.Animals2  ~        X10km_Pop2  8.873  0.074   0.074    0.074
## 93     Total.Animals2  ~     GDP.Millions2 29.909 -0.128  -0.128   -0.128
## 94     Total.Animals2  ~  Nat_Pop._WB20152  8.000 -0.066  -0.066   -0.066
## 98       Sp.Richness2  ~  Brillouin.Index2  5.177  0.071   0.071    0.073
## 99       Sp.Richness2  ~  Mean.Raup.Crick2 17.010 -0.085  -0.085   -0.085
## 104      Sp.Richness2  ~  Nat_Pop._WB20152  3.059 -0.032  -0.032   -0.032
```

```
## 105   Prop.Threat.Sp2  ~       Attendance2 40.939  0.361   0.361    0.354
## 106   Prop.Threat.Sp2  ~    Total.Animals2 27.111  0.265   0.265    0.265
## 107   Prop.Threat.Sp2  ~      Sp.Richness2 13.563  0.273   0.273    0.273
## 108   Prop.Threat.Sp2  ~  Mean.Raup.Crick2  4.101 -0.095  -0.095   -0.095
## 110   Prop.Threat.Sp2  ~  Mam.Sp.Richness2 12.250  0.204   0.204    0.204
## 111   Prop.Threat.Sp2  ~ Mean.Sp.BodyMass2 13.226  0.205   0.205    0.206
## 112   Prop.Threat.Sp2  ~        X50km_Pop2 26.650  0.232   0.232    0.232
## 113   Prop.Threat.Sp2  ~        X10km_Pop2 30.631  0.256   0.256    0.256
## 115   Prop.Threat.Sp2  ~  Nat_Pop._WB20152 10.024  0.145   0.145    0.146
## 119  Brillouin.Index2  ~      Zoo.Area.ha2  5.591  0.061   0.061    0.060
## 121  Brillouin.Index2  ~      Prop.Mam.Sp2  7.549  0.076   0.076    0.074
## 124  Brillouin.Index2  ~        X10km_Pop2  5.633 -0.059  -0.059   -0.058
## 126  Mean.Raup.Crick2  ~       Attendance2 19.817  0.404   0.404    0.396
## 127  Mean.Raup.Crick2  ~   Prop.Threat.Sp2 14.724 -0.155  -0.155   -0.155
## 129  Mean.Raup.Crick2  ~      Zoo.Area.ha2  9.204  0.155   0.155    0.155
## 131  Mean.Raup.Crick2  ~      Prop.Mam.Sp2 28.655 -0.318  -0.318   -0.318
## 132  Mean.Raup.Crick2  ~        X50km_Pop2 14.173  0.152   0.152    0.153
## 133  Mean.Raup.Crick2  ~        X10km_Pop2 19.575  0.187   0.187    0.187
## 135  Mean.Raup.Crick2  ~  Nat_Pop._WB20152 16.563  0.160   0.160    0.160
## 136      Zoo.Area.ha2  ~       Attendance2  4.853  0.402   0.402    0.394
## 138      Zoo.Area.ha2  ~      Sp.Richness2  9.364  0.602   0.602    0.602
## 140      Zoo.Area.ha2  ~  Brillouin.Index2  6.547  0.173   0.173    0.177
## 141      Zoo.Area.ha2  ~  Mean.Raup.Crick2  7.546  0.122   0.122    0.122
## 149  Mam.Sp.Richness2  ~       Attendance2  4.900 -0.405  -0.405   -0.397
## 150  Mam.Sp.Richness2  ~    Total.Animals2  3.877 -0.190  -0.190   -0.190
## 151  Mam.Sp.Richness2  ~      Sp.Richness2  9.522 -0.743  -0.743   -0.743
## 154  Mam.Sp.Richness2  ~  Mean.Raup.Crick2  4.940 -0.128  -0.128   -0.128
## 166      Prop.Mam.Sp2  ~  Brillouin.Index2  3.198  0.107   0.107    0.109
## 167      Prop.Mam.Sp2  ~  Mean.Raup.Crick2 11.046 -0.142  -0.142   -0.142
## 178 Mean.Sp.BodyMass2  ~   Prop.Threat.Sp2  3.504  0.061   0.061    0.061
## 188        X50km_Pop2  ~       Attendance2 29.403  0.567   0.567    0.556
## 189        X50km_Pop2  ~    Total.Animals2 28.216  0.269   0.269    0.269
## 193        X50km_Pop2  ~  Mean.Raup.Crick2  3.649  0.065   0.065    0.064
## 214     GDP.Millions2  ~       Attendance2 10.389 -0.270  -0.270   -0.264
## 216     GDP.Millions2  ~      Sp.Richness2 30.346  0.338   0.338    0.338
## 218     GDP.Millions2  ~  Brillouin.Index2 30.672  0.213   0.213    0.217
## 219     GDP.Millions2  ~  Mean.Raup.Crick2 16.858 -0.111  -0.111   -0.111
## 228  Nat_Pop._WB20152  ~    Total.Animals2  3.776 -0.076  -0.076   -0.076
## 229  Nat_Pop._WB20152  ~      Sp.Richness2 31.565 -0.333  -0.333   -0.333
## 230  Nat_Pop._WB20152  ~   Prop.Threat.Sp2  3.997  0.049   0.049    0.049
## 231  Nat_Pop._WB20152  ~  Brillouin.Index2 15.828 -0.137  -0.137   -0.139
## 232  Nat_Pop._WB20152  ~  Mean.Raup.Crick2 15.632  0.103   0.103    0.103
##     sepc.nox
## 75     0.129
## 76    -0.068
## 77    -0.175
## 79     0.034
## 80    -0.087
## 82    -0.100
## 85     0.130
## 86     0.188
## 87    -0.081
## 91     0.144
## 92     0.074
```

```
## 93    -0.128
## 94    -0.067
## 98     0.073
## 99    -0.085
## 104   -0.032
## 105    0.354
## 106    0.265
## 107    0.273
## 108   -0.095
## 110    0.205
## 111    0.206
## 112    0.232
## 113    0.257
## 115    0.146
## 119    0.060
## 121    0.075
## 124   -0.058
## 126    0.396
## 127   -0.155
## 129    0.156
## 131   -0.319
## 132    0.153
## 133    0.187
## 135    0.160
## 136    0.394
## 138    0.602
## 140    0.177
## 141    0.122
## 149   -0.397
## 150   -0.190
## 151   -0.743
## 154   -0.128
## 166    0.109
## 167   -0.142
## 178    0.061
## 188    0.556
## 189    0.269
## 193    0.064
## 214   -0.264
## 216    0.338
## 218    0.217
## 219   -0.111
## 228   -0.076
## 229   -0.333
## 230    0.049
## 231   -0.139
## 232    0.103
```

```
# Adjust for the nested nature of the data (institutions within countries)
# Fit model and generate model summary
design <- svydesign(ids = ~Country, nest=TRUE, data=sem_attendance_data)
fit.adj5 <- lavaan.survey(lavaan.fit = mod.5.fit, survey.design = design)
summary(fit.adj5, rsq = TRUE)
```

```
## lavaan (0.5-23.1097) converged normally after  57 iterations
```

```
##
##   Number of observations                           458
##
##   Estimator                                         ML      Robust
##   Minimum Function Test Statistic              324.001      78.487
##   Degrees of freedom                                37          37
##   P-value (Chi-square)                           0.000       0.000
##   Scaling correction factor                                  4.128
##     for the Satorra-Bentler correction
##
## Parameter Estimates:
##
##   Information                                 Expected
##   Standard Errors                           Robust.sem
##
## Regressions:
##                      Estimate  Std.Err  z-value  P(>|z|)
##   Attendance2 ~
##     Zoo.Area.ha2        0.086    0.041    2.115    0.034
##     Sp.Richness2       -0.249    0.102   -2.449    0.014
##     Total.Animals2      0.454    0.062    7.316    0.000
##     Mam.Sp.Rchnss2      0.132    0.049    2.712    0.007
##     Prop.Mam.Sp2       -0.065    0.045   -1.448    0.148
##     Prop.Thret.Sp2      0.009    0.029    0.306    0.760
##     Men.Sp.BdyMss2      0.339    0.030   11.327    0.000
##     Brilloun.Indx2      0.077    0.066    1.160    0.246
##     Mean.Rap.Crck2      0.146    0.029    5.002    0.000
##     X50km_Pop2          0.079    0.033    2.363    0.018
##     X10km_Pop2          0.397    0.042    9.352    0.000
##     GDP.Millions2       0.259    0.060    4.315    0.000
##     Nt_Pp._WB20152     -0.124    0.067   -1.845    0.065
##   Total.Animals2 ~
##     Zoo.Area.ha2        0.223    0.038    5.856    0.000
##     Sp.Richness2        0.798    0.048   16.678    0.000
##   Sp.Richness2 ~
##     Zoo.Area.ha2        0.067    0.044    1.519    0.129
##     Prop.Mam.Sp2       -0.581    0.038  -15.231    0.000
##     Mam.Sp.Rchnss2      0.755    0.068   11.066    0.000
##   Prop.Threat.Sp2 ~
##     Brilloun.Indx2      0.038    0.114    0.331    0.741
##     Prop.Mam.Sp2        0.332    0.068    4.870    0.000
##   Brillouin.Index2 ~
##     Sp.Richness2        1.320    0.069   19.079    0.000
##     Total.Animals2     -0.620    0.102   -6.085    0.000
##     GDP.Millions2      -0.206    0.059   -3.492    0.000
##   Mean.Raup.Crick2 ~
##     Sp.Richness2       -0.355    0.228   -1.555    0.120
##     Total.Animals2      0.558    0.153    3.656    0.000
##     Men.Sp.BdyMss2     -0.495    0.052   -9.463    0.000
##
## Covariances:
##                        Estimate  Std.Err  z-value  P(>|z|)
##   Zoo.Area.ha2 ~~
##     Mam.Sp.Rchnss2        0.381    0.064    5.985    0.000
```

```
##     Prop.Mam.Sp2         0.312    0.089    3.510    0.000
##     Men.Sp.BdyMss2       0.534    0.104    5.147    0.000
##     X50km_Pop2           0.060    0.069    0.880    0.379
##     X10km_Pop2          -0.010    0.078   -0.124    0.901
##     GDP.Millions2       -0.027    0.041   -0.673    0.501
##     Nt_Pp._WB20152       0.061    0.090    0.682    0.495
##   Mam.Sp.Richness2 ~~
##     Prop.Mam.Sp2         0.088    0.061    1.435    0.151
##     Men.Sp.BdyMss2       0.303    0.081    3.730    0.000
##     X50km_Pop2           0.204    0.082    2.487    0.013
##     X10km_Pop2           0.284    0.066    4.278    0.000
##     GDP.Millions2       -0.060    0.064   -0.937    0.349
##     Nt_Pp._WB20152      -0.088    0.102   -0.863    0.388
##   Prop.Mam.Sp2 ~~
##     Men.Sp.BdyMss2       0.604    0.130    4.656    0.000
##     X50km_Pop2          -0.096    0.051   -1.888    0.059
##     X10km_Pop2          -0.204    0.055   -3.685    0.000
##     GDP.Millions2       -0.123    0.105   -1.172    0.241
##     Nt_Pp._WB20152      -0.115    0.091   -1.257    0.209
##   Mean.Sp.BodyMass2 ~~
##     X50km_Pop2           0.101    0.072    1.400    0.161
##     X10km_Pop2           0.126    0.067    1.895    0.058
##     GDP.Millions2       -0.082    0.046   -1.759    0.079
##     Nt_Pp._WB20152       0.006    0.092    0.063    0.950
##   X50km_Pop2 ~~
##     X10km_Pop2           0.752    0.126    5.982    0.000
##     GDP.Millions2       -0.013    0.100   -0.131    0.896
##     Nt_Pp._WB20152       0.191    0.177    1.083    0.279
##   X10km_Pop2 ~~
##     GDP.Millions2       -0.028    0.056   -0.505    0.613
##     Nt_Pp._WB20152       0.173    0.101    1.701    0.089
##   GDP.Millions2 ~~
##     Nt_Pp._WB20152       0.826    0.202    4.089    0.000
##
## Intercepts:
##                    Estimate  Std.Err  z-value  P(>|z|)
##    .Attendance2      -0.000    0.031   -0.000    1.000
##    .Total.Animals2   -0.000    0.104   -0.000    1.000
##    .Sp.Richness2      0.000    0.034    0.000    1.000
##    .Prop.Thret.Sp2    0.000    0.083    0.000    1.000
##    .Brilloun.Indx2   -0.000    0.055   -0.000    1.000
##    .Mean.Rap.Crck2    0.000    0.094    0.000    1.000
##     Zoo.Area.ha2      0.000    0.064    0.000    1.000
##     Mam.Sp.Rchnss2   -0.000    0.079   -0.000    1.000
##     Prop.Mam.Sp2      0.000    0.118    0.000    1.000
##     Men.Sp.BdyMss2   -0.000    0.080   -0.000    1.000
##     X50km_Pop2       -0.000    0.114   -0.000    1.000
##     X10km_Pop2        0.000    0.105    0.000    1.000
##     GDP.Millions2    -0.000    0.345   -0.000    1.000
##     Nt_Pp._WB20152    0.000    0.295    0.000    1.000
##
## Variances:
##                    Estimate  Std.Err  z-value  P(>|z|)
##    .Attendance2       0.267    0.028    9.566    0.000
```

```
##    .Total.Animals2    0.251    0.044    5.729    0.000
##    .Sp.Richness2      0.151    0.046    3.269    0.001
##    .Prop.Thret.Sp2    0.896    0.122    7.319    0.000
##    .Brilloun.Indx2    0.251    0.053    4.691    0.000
##    .Mean.Rap.Crck2    0.704    0.063   11.098    0.000
##     Zoo.Area.ha2      0.998    0.112    8.886    0.000
##     Mam.Sp.Rchnss2    0.998    0.115    8.669    0.000
##     Prop.Mam.Sp2      0.998    0.150    6.662    0.000
##     Men.Sp.BdyMss2    0.998    0.173    5.783    0.000
##     X50km_Pop2        0.998    0.131    7.623    0.000
##     X10km_Pop2        0.998    0.174    5.745    0.000
##     GDP.Millions2     0.998    0.246    4.061    0.000
##     Nt_Pp._WB20152    0.998    0.225    4.439    0.000
##
## R-Square:
##                    Estimate
##     Attendance2       0.721
##     Total.Animals2    0.748
##     Sp.Richness2      0.849
##     Prop.Thret.Sp2    0.101
##     Brilloun.Indx2    0.759
##     Mean.Rap.Crck2    0.294
```

```
# Generate fit indices
fitMeasures(fit.adj5, c("agfi", "rmr", "srmr", "rmsea", "cfi", "nnfi", "tli"))
```

```
##  agfi   rmr  srmr rmsea   cfi  nnfi   tli
## 0.725 0.069 0.065 0.130 0.910 0.847 0.847
```

```
# Generate modification indices
mi5adj <- modindices(fit.adj5)
print(mi5adj[mi5adj$mi > 3.0,])
```

```
##                  lhs op              rhs     mi mi.scaled    epc sepc.lv
## 89    Total.Animals2 ~~   Prop.Threat.Sp2 29.840     7.229  0.129   0.129
## 90    Total.Animals2 ~~  Brillouin.Index2  5.591     1.354 -0.069  -0.069
## 91    Total.Animals2 ~~  Mean.Raup.Crick2  9.205     2.230 -0.175  -0.175
## 93      Sp.Richness2 ~~  Brillouin.Index2 12.470     3.021  0.035   0.035
## 94      Sp.Richness2 ~~  Mean.Raup.Crick2 27.606     6.687 -0.087  -0.087
## 96   Prop.Threat.Sp2 ~~  Mean.Raup.Crick2  7.213     1.747 -0.100  -0.100
## 99    Total.Animals2  ~   Prop.Threat.Sp2 26.465     6.411  0.130   0.130
## 100   Total.Animals2  ~  Brillouin.Index2  5.096     1.234  0.184   0.184
## 101   Total.Animals2  ~  Mean.Raup.Crick2  3.003     0.727 -0.081  -0.081
## 105   Total.Animals2  ~        X50km_Pop2 35.809     8.674  0.144   0.144
## 106   Total.Animals2  ~        X10km_Pop2  8.873     2.149  0.074   0.074
## 107   Total.Animals2  ~     GDP.Millions2 29.909     7.245 -0.128  -0.128
## 108   Total.Animals2  ~  Nat_Pop._WB20152  8.000     1.938 -0.066  -0.066
## 112     Sp.Richness2  ~  Brillouin.Index2  5.177     1.254  0.071   0.071
## 113     Sp.Richness2  ~  Mean.Raup.Crick2 17.010     4.121 -0.085  -0.085
## 118     Sp.Richness2  ~  Nat_Pop._WB20152  3.059     0.741 -0.032  -0.032
## 119  Prop.Threat.Sp2  ~       Attendance2 40.939     9.917  0.361   0.361
## 120  Prop.Threat.Sp2  ~    Total.Animals2 27.111     6.567  0.265   0.265
## 121  Prop.Threat.Sp2  ~      Sp.Richness2 13.563     3.285  0.273   0.273
## 122  Prop.Threat.Sp2  ~  Mean.Raup.Crick2  4.101     0.993 -0.095  -0.095
## 124  Prop.Threat.Sp2  ~  Mam.Sp.Richness2 12.250     2.968  0.204   0.204
## 125  Prop.Threat.Sp2  ~ Mean.Sp.BodyMass2 13.226     3.204  0.205   0.205
```

```
## 126   Prop.Threat.Sp2  ~        X50km_Pop2 26.650     6.456  0.232   0.232
## 127   Prop.Threat.Sp2  ~        X10km_Pop2 30.631     7.420  0.256   0.256
## 129   Prop.Threat.Sp2  ~  Nat_Pop._WB20152 10.024     2.428  0.145   0.145
## 133  Brillouin.Index2  ~      Zoo.Area.ha2  5.591     1.354  0.061   0.061
## 135  Brillouin.Index2  ~      Prop.Mam.Sp2  7.549     1.829  0.076   0.076
## 138  Brillouin.Index2  ~        X10km_Pop2  5.633     1.365 -0.059  -0.059
## 140  Mean.Raup.Crick2  ~       Attendance2 19.817     4.801  0.404   0.404
## 141  Mean.Raup.Crick2  ~   Prop.Threat.Sp2 14.724     3.567 -0.155  -0.155
## 143  Mean.Raup.Crick2  ~      Zoo.Area.ha2  9.205     2.230  0.155   0.155
## 145  Mean.Raup.Crick2  ~      Prop.Mam.Sp2 28.655     6.942 -0.318  -0.318
## 146  Mean.Raup.Crick2  ~        X50km_Pop2 14.173     3.433  0.152   0.152
## 147  Mean.Raup.Crick2  ~        X10km_Pop2 19.575     4.742  0.187   0.187
## 149  Mean.Raup.Crick2  ~  Nat_Pop._WB20152 16.563     4.012  0.160   0.160
## 150      Zoo.Area.ha2  ~       Attendance2  4.852     1.175  0.402   0.402
## 152      Zoo.Area.ha2  ~      Sp.Richness2  9.363     2.268  0.602   0.602
## 154      Zoo.Area.ha2  ~  Brillouin.Index2  6.547     1.586  0.173   0.173
## 155      Zoo.Area.ha2  ~  Mean.Raup.Crick2  7.546     1.828  0.122   0.122
## 163  Mam.Sp.Richness2  ~       Attendance2  4.900     1.187 -0.405  -0.405
## 164  Mam.Sp.Richness2  ~    Total.Animals2  3.877     0.939 -0.190  -0.190
## 165  Mam.Sp.Richness2  ~      Sp.Richness2  9.521     2.306 -0.742  -0.742
## 168  Mam.Sp.Richness2  ~  Mean.Raup.Crick2  4.940     1.197 -0.128  -0.128
## 180      Prop.Mam.Sp2  ~  Brillouin.Index2  3.198     0.775  0.107   0.107
## 181      Prop.Mam.Sp2  ~  Mean.Raup.Crick2 11.046     2.676 -0.142  -0.142
## 192 Mean.Sp.BodyMass2  ~   Prop.Threat.Sp2  3.504     0.849  0.061   0.061
## 202        X50km_Pop2  ~       Attendance2 29.403     7.123  0.567   0.567
## 203        X50km_Pop2  ~    Total.Animals2 28.216     6.835  0.269   0.269
## 207        X50km_Pop2  ~  Mean.Raup.Crick2  3.649     0.884  0.065   0.065
## 228     GDP.Millions2  ~       Attendance2 10.389     2.517 -0.270  -0.270
## 230     GDP.Millions2  ~      Sp.Richness2 30.346     7.351  0.338   0.338
## 232     GDP.Millions2  ~  Brillouin.Index2 30.672     7.430  0.213   0.213
## 233     GDP.Millions2  ~  Mean.Raup.Crick2 16.858     4.084 -0.111  -0.111
## 242  Nat_Pop._WB20152  ~    Total.Animals2  3.776     0.915 -0.076  -0.076
## 243  Nat_Pop._WB20152  ~      Sp.Richness2 31.565     7.646 -0.333  -0.333
## 244  Nat_Pop._WB20152  ~   Prop.Threat.Sp2  3.997     0.968  0.049   0.049
## 245  Nat_Pop._WB20152  ~  Brillouin.Index2 15.828     3.834 -0.137  -0.137
## 246  Nat_Pop._WB20152  ~  Mean.Raup.Crick2 15.632     3.787  0.103   0.103
##     sepc.all sepc.nox
## 89     0.129    0.129
## 90    -0.068   -0.068
## 91    -0.175   -0.175
## 93     0.034    0.034
## 94    -0.087   -0.087
## 96    -0.100   -0.100
## 99     0.130    0.130
## 100    0.188    0.188
## 101   -0.081   -0.081
## 105    0.144    0.144
## 106    0.074    0.074
## 107   -0.128   -0.128
## 108   -0.066   -0.067
## 112    0.073    0.073
## 113   -0.085   -0.085
## 118   -0.032   -0.032
## 119    0.354    0.354
```

```
## 120    0.265    0.265
## 121    0.273    0.273
## 122   -0.095   -0.095
## 124    0.204    0.205
## 125    0.206    0.206
## 126    0.232    0.232
## 127    0.256    0.257
## 129    0.146    0.146
## 133    0.060    0.060
## 135    0.074    0.075
## 138   -0.058   -0.058
## 140    0.396    0.396
## 141   -0.155   -0.155
## 143    0.155    0.156
## 145   -0.318   -0.319
## 146    0.153    0.153
## 147    0.187    0.187
## 149    0.160    0.160
## 150    0.394    0.394
## 152    0.602    0.602
## 154    0.177    0.177
## 155    0.122    0.122
## 163   -0.397   -0.397
## 164   -0.190   -0.190
## 165   -0.742   -0.742
## 168   -0.128   -0.128
## 180    0.109    0.109
## 181   -0.142   -0.142
## 192    0.061    0.061
## 202    0.556    0.556
## 203    0.269    0.269
## 207    0.064    0.064
## 228   -0.264   -0.264
## 230    0.338    0.338
## 232    0.217    0.217
## 233   -0.111   -0.111
## 242   -0.076   -0.076
## 243   -0.333   -0.333
## 244    0.049    0.049
## 245   -0.139   -0.139
## 246    0.103    0.103
```

Model 6

Based on the modification indices generated from the fifth model, we can see that there are several relationships with similarly high mi values, for example: ___Nat_Pop._WB20152 ~ Sp.Richness2 31.565, mi = 31.565___ **GDP.Millions2 ~ Brillouin.Index2, mi = 30.672 GDP.Millions2 ~ Sp.Richness2, mi = 30.346 Prop.Threat.Sp2 ~ X10km_Pop2, mi = 30.631** However as modification indices do not take into account whether or not relationships make theoretical sense, these were not considered for inclusion. For example, although **GDP.Millions2 ~ Brillouin.Index2** has an mi value of **30.672**, it makes no sense to suggest that the GDP of a country is in any way caused by the taxonomic diversity of individual zoological collections. Therefore, the most intuitive relationship with the highest mi value was identified as **Total.Animals2 ~ GDP.Millions2**, with a corresponding mi value of **29.909**. Although not the highest

mi value, this is the most intuitive and still exceeds the standard cut-off level for the chi-square test criterion of 3.84 (Burnham and Anderson, 2002). This is considered intuituive as it is logical to assume that countries with a higher GDP can afford to have zoological collections with many more animals. Again, the exact reasoning behind this is likely rooted in an historical context that exceeds the scope of this work. As a result, we add this relationship to our model. Once again, the model summary, fit indices and modification indices were all generated for the model, adjusting for the nested nature of data.

Similar rationale and theoretical sense were appied to all interpretations of modification indices, often resulting in the selection of suggested relationships for inclusion, despite the fact that relationships with higher mi values were observed. This is the reason the development of the a priori meta-model is key and why a deep understanding of the study system in question is needed to guide model modification an interpretation.

```
# Attendance SEM (Presence-Absence)

# Model 6
# Addition of Total.Animals2 ~ GDP.Millions2, mi = 29.909

mod.6 <- 'Attendance2 ~ Zoo.Area.ha2 + Sp.Richness2 + Total.Animals2
+ Mam.Sp.Richness2 + Prop.Mam.Sp2 + Prop.Threat.Sp2
+ Mean.Sp.BodyMass2 + Brillouin.Index2 + Mean.Raup.Crick2
+ X50km_Pop2 + X10km_Pop2 + GDP.Millions2 + Nat_Pop._WB20152

Total.Animals2 ~ Zoo.Area.ha2 + Sp.Richness2 + GDP.Millions2
Sp.Richness2 ~ Zoo.Area.ha2 + Prop.Mam.Sp2 + Mam.Sp.Richness2
Prop.Threat.Sp2 ~ Brillouin.Index2 + Prop.Mam.Sp2
Brillouin.Index2 ~ Sp.Richness2 + Total.Animals2 + GDP.Millions2
Mean.Raup.Crick2 ~ Sp.Richness2 + Total.Animals2 + Mean.Sp.BodyMass2'

# Fit model and generate model summary
mod.6.fit <- sem(mod.6, data = sem_attendance_data, fixed.x=FALSE)
summary(mod.6.fit, rsq = TRUE)
```

```
## lavaan (0.5-23.1097) converged normally after  57 iterations
##
##   Number of observations                           458
##
##   Estimator                                         ML
##   Minimum Function Test Statistic              293.003
##   Degrees of freedom                                36
##   P-value (Chi-square)                           0.000
##
## Parameter Estimates:
##
##   Information                                 Expected
##   Standard Errors                             Standard
##
## Regressions:
##                     Estimate  Std.Err  z-value  P(>|z|)
##   Attendance2 ~
##     Zoo.Area.ha2       0.086    0.033    2.590    0.010
##     Sp.Richness2      -0.249    0.098   -2.539    0.011
##     Total.Animals2     0.454    0.060    7.532    0.000
##     Mam.Sp.Rchnss2     0.132    0.055    2.403    0.016
##     Prop.Mam.Sp2      -0.065    0.050   -1.317    0.188
##     Prop.Thret.Sp2     0.009    0.026    0.353    0.724
```

```
##     Men.Sp.BdyMss2      0.339    0.039    8.644    0.000
##     Brilloun.Indx2      0.077    0.048    1.596    0.111
##     Mean.Rap.Crck2      0.146    0.029    5.054    0.000
##     X50km_Pop2          0.079    0.038    2.096    0.036
##     X10km_Pop2          0.397    0.041    9.624    0.000
##     GDP.Millions2       0.259    0.049    5.310    0.000
##     Nt_Pp._WB20152     -0.124    0.049   -2.523    0.012
##   Total.Animals2 ~
##     Zoo.Area.ha2        0.219    0.023    9.495    0.000
##     Sp.Richness2        0.805    0.023   34.969    0.000
##     GDP.Millions2      -0.129    0.023   -5.669    0.000
##   Sp.Richness2 ~
##     Zoo.Area.ha2        0.067    0.021    3.225    0.001
##     Prop.Mam.Sp2       -0.581    0.019  -30.364    0.000
##     Mam.Sp.Rchnss2      0.755    0.020   38.373    0.000
##   Prop.Threat.Sp2 ~
##     Brilloun.Indx2      0.038    0.049    0.773    0.440
##     Prop.Mam.Sp2        0.332    0.049    6.769    0.000
##   Brillouin.Index2 ~
##     Sp.Richness2        1.320    0.044   30.015    0.000
##     Total.Animals2     -0.620    0.044  -14.052    0.000
##     GDP.Millions2      -0.206    0.024   -8.518    0.000
##   Mean.Raup.Crick2 ~
##     Sp.Richness2       -0.355    0.074   -4.791    0.000
##     Total.Animals2      0.558    0.074    7.566    0.000
##     Men.Sp.BdyMss2     -0.495    0.041  -12.212    0.000
##
## Covariances:
##                        Estimate  Std.Err  z-value  P(>|z|)
##   Zoo.Area.ha2 ~~
##     Mam.Sp.Rchnss2        0.381    0.050    7.634    0.000
##     Prop.Mam.Sp2          0.312    0.049    6.389    0.000
##     Men.Sp.BdyMss2        0.534    0.053   10.096    0.000
##     X50km_Pop2            0.060    0.047    1.291    0.197
##     X10km_Pop2           -0.010    0.047   -0.208    0.835
##     GDP.Millions2        -0.027    0.047   -0.588    0.557
##     Nt_Pp._WB20152        0.061    0.047    1.310    0.190
##   Mam.Sp.Richness2 ~~
##     Prop.Mam.Sp2          0.088    0.047    1.882    0.060
##     Men.Sp.BdyMss2        0.303    0.049    6.216    0.000
##     X50km_Pop2            0.204    0.048    4.281    0.000
##     X10km_Pop2            0.284    0.048    5.852    0.000
##     GDP.Millions2        -0.060    0.047   -1.277    0.202
##     Nt_Pp._WB20152       -0.088    0.047   -1.881    0.060
##   Prop.Mam.Sp2 ~~
##     Men.Sp.BdyMss2        0.604    0.055   11.083    0.000
##     X50km_Pop2           -0.096    0.047   -2.054    0.040
##     X10km_Pop2           -0.204    0.048   -4.293    0.000
##     GDP.Millions2        -0.123    0.047   -2.615    0.009
##     Nt_Pp._WB20152       -0.115    0.047   -2.447    0.014
##   Mean.Sp.BodyMass2 ~~
##     X50km_Pop2            0.101    0.047    2.166    0.030
##     X10km_Pop2            0.126    0.047    2.683    0.007
##     GDP.Millions2        -0.082    0.047   -1.748    0.080
```

```
##     Nt_Pp._WB20152        0.006    0.047    0.124    0.901
##   X50km_Pop2 ~~
##     X10km_Pop2            0.752    0.058   12.886    0.000
##     GDP.Millions2        -0.013    0.047   -0.281    0.779
##     Nt_Pp._WB20152        0.191    0.047    4.033    0.000
##   X10km_Pop2 ~~
##     GDP.Millions2        -0.028    0.047   -0.604    0.546
##     Nt_Pp._WB20152        0.173    0.047    3.646    0.000
##   GDP.Millions2 ~~
##     Nt_Pp._WB20152        0.826    0.061   13.642    0.000
##
## Variances:
##                    Estimate  Std.Err  z-value  P(>|z|)
##    .Attendance2       0.267    0.018   15.133    0.000
##    .Total.Animals2    0.235    0.016   15.133    0.000
##    .Sp.Richness2      0.151    0.010   15.133    0.000
##    .Prop.Thret.Sp2    0.896    0.059   15.133    0.000
##    .Brilloun.Indx2    0.251    0.017   15.133    0.000
##    .Mean.Rap.Crck2    0.704    0.047   15.133    0.000
##     Zoo.Area.ha2      0.998    0.066   15.133    0.000
##     Mam.Sp.Rchnss2    0.998    0.066   15.133    0.000
##     Prop.Mam.Sp2      0.998    0.066   15.133    0.000
##     Men.Sp.BdyMss2    0.998    0.066   15.133    0.000
##     X50km_Pop2        0.998    0.066   15.133    0.000
##     X10km_Pop2        0.998    0.066   15.133    0.000
##     GDP.Millions2     0.998    0.066   15.133    0.000
##     Nt_Pp._WB20152    0.998    0.066   15.133    0.000
##
## R-Square:
##                    Estimate
##     Attendance2       0.718
##     Total.Animals2    0.766
##     Sp.Richness2      0.849
##     Prop.Thret.Sp2    0.101
##     Brilloun.Indx2    0.750
##     Mean.Rap.Crck2    0.291
```

```r
# Generate fit indices
fitMeasures(mod.6.fit, c("agfi", "rmr", "srmr", "rmsea", "cfi", "nnfi", "tli"))
```

```
##  agfi   rmr  srmr rmsea   cfi  nnfi   tli
## 0.768 0.068 0.069 0.125 0.920 0.860 0.860
```

```r
# Generate modification indices
mi6 <- modindices(mod.6.fit)
print(mi6[mi6$mi > 3.0,])
```

```
##                  lhs op               rhs     mi    epc sepc.lv sepc.all
## 76     Total.Animals2 ~~   Prop.Threat.Sp2 34.979  0.134   0.134    0.134
## 77     Total.Animals2 ~~ Brillouin.Index2  5.613 -0.066  -0.066   -0.066
## 78     Total.Animals2 ~~  Mean.Raup.Crick2  4.260 -0.096  -0.096   -0.096
## 80       Sp.Richness2 ~~ Brillouin.Index2 12.470  0.035   0.035    0.035
## 81       Sp.Richness2 ~~  Mean.Raup.Crick2 27.601 -0.087  -0.087   -0.087
## 83    Prop.Threat.Sp2 ~~  Mean.Raup.Crick2  7.213 -0.100  -0.100   -0.100
## 85     Total.Animals2  ~       Attendance2  3.967  0.085   0.085    0.083
## 86     Total.Animals2  ~   Prop.Threat.Sp2 28.764  0.131   0.131    0.130
```

```
## 87     Total.Animals2  ~  Brillouin.Index2  5.613 -0.264  -0.264   -0.264
## 92     Total.Animals2  ~        X50km_Pop2 36.696  0.141   0.141    0.140
## 93     Total.Animals2  ~        X10km_Pop2  7.848  0.068   0.068    0.067
## 94     Total.Animals2  ~  Nat_Pop._WB20152 10.481  0.132   0.132    0.132
## 98       Sp.Richness2  ~  Brillouin.Index2  6.033  0.081   0.081    0.081
## 99       Sp.Richness2  ~  Mean.Raup.Crick2 17.310 -0.085  -0.085   -0.085
## 104      Sp.Richness2  ~  Nat_Pop._WB20152  3.059 -0.032  -0.032   -0.032
## 105   Prop.Threat.Sp2  ~       Attendance2 43.084  0.379   0.379    0.370
## 106   Prop.Threat.Sp2  ~    Total.Animals2 27.892  0.272   0.272    0.273
## 107   Prop.Threat.Sp2  ~      Sp.Richness2 14.069  0.283   0.283    0.283
## 108   Prop.Threat.Sp2  ~  Mean.Raup.Crick2  4.088 -0.094  -0.094   -0.094
## 110   Prop.Threat.Sp2  ~  Mam.Sp.Richness2 12.513  0.209   0.209    0.209
## 111   Prop.Threat.Sp2  ~ Mean.Sp.BodyMass2 13.244  0.206   0.206    0.206
## 112   Prop.Threat.Sp2  ~        X50km_Pop2 26.657  0.232   0.232    0.232
## 113   Prop.Threat.Sp2  ~        X10km_Pop2 30.626  0.256   0.256    0.256
## 115   Prop.Threat.Sp2  ~  Nat_Pop._WB20152  9.734  0.141   0.141    0.141
## 119  Brillouin.Index2  ~      Zoo.Area.ha2  5.613  0.062   0.062    0.062
## 121  Brillouin.Index2  ~      Prop.Mam.Sp2  7.555  0.076   0.076    0.076
## 124  Brillouin.Index2  ~        X10km_Pop2  5.633 -0.059  -0.059   -0.059
## 126  Mean.Raup.Crick2  ~       Attendance2 19.945  0.407   0.407    0.397
## 127  Mean.Raup.Crick2  ~   Prop.Threat.Sp2 14.727 -0.155  -0.155   -0.155
## 129  Mean.Raup.Crick2  ~      Zoo.Area.ha2  9.137  0.154   0.154    0.155
## 131  Mean.Raup.Crick2  ~      Prop.Mam.Sp2 28.743 -0.319  -0.319   -0.320
## 132  Mean.Raup.Crick2  ~        X50km_Pop2 14.172  0.152   0.152    0.153
## 133  Mean.Raup.Crick2  ~        X10km_Pop2 19.556  0.187   0.187    0.187
## 135  Mean.Raup.Crick2  ~  Nat_Pop._WB20152 17.087  0.165   0.165    0.165
## 136      Zoo.Area.ha2  ~       Attendance2  5.873  0.451   0.451    0.439
## 138      Zoo.Area.ha2  ~      Sp.Richness2  9.363  0.602   0.602    0.602
## 140      Zoo.Area.ha2  ~  Brillouin.Index2  6.203  0.169   0.169    0.170
## 141      Zoo.Area.ha2  ~  Mean.Raup.Crick2  7.812  0.124   0.124    0.124
## 149  Mam.Sp.Richness2  ~       Attendance2  6.235 -0.469  -0.469   -0.457
## 150  Mam.Sp.Richness2  ~    Total.Animals2  5.172 -0.226  -0.226   -0.227
## 151  Mam.Sp.Richness2  ~      Sp.Richness2  9.521 -0.742  -0.742   -0.742
## 152  Mam.Sp.Richness2  ~   Prop.Threat.Sp2  3.019  0.088   0.088    0.088
## 154  Mam.Sp.Richness2  ~  Mean.Raup.Crick2  5.330 -0.133  -0.133   -0.133
## 166      Prop.Mam.Sp2  ~  Brillouin.Index2  3.002  0.105   0.105    0.105
## 167      Prop.Mam.Sp2  ~  Mean.Raup.Crick2 10.813 -0.141  -0.141   -0.141
## 178 Mean.Sp.BodyMass2  ~   Prop.Threat.Sp2  3.504  0.061   0.061    0.061
## 188        X50km_Pop2  ~       Attendance2 30.802  0.594   0.594    0.579
## 189        X50km_Pop2  ~    Total.Animals2 29.464  0.281   0.281    0.282
## 193        X50km_Pop2  ~  Mean.Raup.Crick2  3.672  0.065   0.065    0.065
## 202        X10km_Pop2  ~    Total.Animals2  3.040 -0.083  -0.083   -0.083
## 215     GDP.Millions2  ~    Total.Animals2  4.926  0.102   0.102    0.102
## 216     GDP.Millions2  ~      Sp.Richness2 30.346  0.338   0.338    0.338
## 218     GDP.Millions2  ~  Brillouin.Index2 18.587  0.173   0.173    0.173
## 219     GDP.Millions2  ~  Mean.Raup.Crick2 10.851 -0.091  -0.091   -0.091
## 228  Nat_Pop._WB20152  ~    Total.Animals2  3.943 -0.079  -0.079   -0.080
## 229  Nat_Pop._WB20152  ~      Sp.Richness2 31.565 -0.333  -0.333   -0.333
## 230  Nat_Pop._WB20152  ~   Prop.Threat.Sp2  3.997  0.049   0.049    0.049
## 231  Nat_Pop._WB20152  ~  Brillouin.Index2 16.094 -0.139  -0.139   -0.139
## 232  Nat_Pop._WB20152  ~  Mean.Raup.Crick2 15.732  0.104   0.104    0.103
##     sepc.nox
## 76     0.134
## 77    -0.066
```

```
## 78    -0.096
## 80     0.035
## 81    -0.087
## 83    -0.100
## 85     0.083
## 86     0.130
## 87    -0.264
## 92     0.140
## 93     0.067
## 94     0.132
## 98     0.081
## 99    -0.085
## 104   -0.032
## 105    0.370
## 106    0.273
## 107    0.283
## 108   -0.094
## 110    0.209
## 111    0.206
## 112    0.232
## 113    0.257
## 115    0.142
## 119    0.062
## 121    0.076
## 124   -0.059
## 126    0.397
## 127   -0.155
## 129    0.155
## 131   -0.320
## 132    0.153
## 133    0.188
## 135    0.165
## 136    0.439
## 138    0.602
## 140    0.170
## 141    0.124
## 149   -0.457
## 150   -0.227
## 151   -0.742
## 152    0.088
## 154   -0.133
## 166    0.105
## 167   -0.141
## 178    0.061
## 188    0.579
## 189    0.282
## 193    0.065
## 202   -0.083
## 215    0.102
## 216    0.338
## 218    0.173
## 219   -0.091
## 228   -0.080
## 229   -0.333
```

```
## 230    0.049
## 231   -0.139
## 232    0.103
```

```r
# Adjust for the nested nature of the data (institutions within countries)
# Fit model and generate model summary
design <- svydesign(ids = ~Country, nest=TRUE, data=sem_attendance_data)
fit.adj6 <- lavaan.survey(lavaan.fit = mod.6.fit, survey.design = design)
summary(fit.adj6, rsq = TRUE)
```

```
## lavaan (0.5-23.1097) converged normally after  61 iterations
##
##   Number of observations                           458
##
##   Estimator                                         ML      Robust
##   Minimum Function Test Statistic              293.003      76.426
##   Degrees of freedom                                36          36
##   P-value (Chi-square)                           0.000       0.000
##   Scaling correction factor                                  3.834
##     for the Satorra-Bentler correction
##
## Parameter Estimates:
##
##   Information                                 Expected
##   Standard Errors                           Robust.sem
##
## Regressions:
##                      Estimate  Std.Err  z-value  P(>|z|)
##   Attendance2 ~
##     Zoo.Area.ha2        0.086    0.041    2.106    0.035
##     Sp.Richness2       -0.249    0.106   -2.353    0.019
##     Total.Animals2      0.454    0.068    6.725    0.000
##     Mam.Sp.Rchnss2      0.132    0.049    2.712    0.007
##     Prop.Mam.Sp2       -0.065    0.045   -1.448    0.148
##     Prop.Thret.Sp2      0.009    0.029    0.306    0.760
##     Men.Sp.BdyMss2      0.339    0.030   11.327    0.000
##     Brilloun.Indx2      0.077    0.066    1.160    0.246
##     Mean.Rap.Crck2      0.146    0.029    5.002    0.000
##     X50km_Pop2          0.079    0.033    2.363    0.018
##     X10km_Pop2          0.397    0.042    9.352    0.000
##     GDP.Millions2       0.259    0.061    4.251    0.000
##     Nt_Pp._WB20152     -0.124    0.067   -1.845    0.065
##   Total.Animals2 ~
##     Zoo.Area.ha2        0.219    0.036    5.992    0.000
##     Sp.Richness2        0.805    0.051   15.917    0.000
##     GDP.Millions2      -0.129    0.072   -1.785    0.074
##   Sp.Richness2 ~
##     Zoo.Area.ha2        0.067    0.044    1.519    0.129
##     Prop.Mam.Sp2       -0.581    0.038  -15.231    0.000
##     Mam.Sp.Rchnss2      0.755    0.068   11.066    0.000
##   Prop.Threat.Sp2 ~
##     Brilloun.Indx2      0.038    0.120    0.315    0.753
##     Prop.Mam.Sp2        0.332    0.070    4.742    0.000
##   Brillouin.Index2 ~
##     Sp.Richness2        1.320    0.089   14.753    0.000
```

```
##     Total.Animals2     -0.620    0.127   -4.883    0.000
##     GDP.Millions2      -0.206    0.072   -2.846    0.004
##   Mean.Raup.Crick2 ~
##     Sp.Richness2       -0.355    0.230   -1.543    0.123
##     Total.Animals2      0.558    0.154    3.625    0.000
##     Men.Sp.BdyMss2     -0.495    0.052   -9.504    0.000
##
## Covariances:
##                        Estimate  Std.Err  z-value  P(>|z|)
##   Zoo.Area.ha2 ~~
##     Mam.Sp.Rchnss2        0.381    0.064    5.985    0.000
##     Prop.Mam.Sp2          0.312    0.089    3.510    0.000
##     Men.Sp.BdyMss2        0.534    0.104    5.147    0.000
##     X50km_Pop2            0.060    0.069    0.880    0.379
##     X10km_Pop2           -0.010    0.078   -0.124    0.901
##     GDP.Millions2        -0.027    0.041   -0.673    0.501
##     Nt_Pp._WB20152        0.061    0.090    0.682    0.495
##   Mam.Sp.Richness2 ~~
##     Prop.Mam.Sp2          0.088    0.061    1.435    0.151
##     Men.Sp.BdyMss2        0.303    0.081    3.730    0.000
##     X50km_Pop2            0.204    0.082    2.487    0.013
##     X10km_Pop2            0.284    0.066    4.278    0.000
##     GDP.Millions2        -0.060    0.064   -0.937    0.349
##     Nt_Pp._WB20152       -0.088    0.102   -0.863    0.388
##   Prop.Mam.Sp2 ~~
##     Men.Sp.BdyMss2        0.604    0.130    4.656    0.000
##     X50km_Pop2           -0.096    0.051   -1.888    0.059
##     X10km_Pop2           -0.204    0.055   -3.685    0.000
##     GDP.Millions2        -0.123    0.105   -1.172    0.241
##     Nt_Pp._WB20152       -0.115    0.091   -1.257    0.209
##   Mean.Sp.BodyMass2 ~~
##     X50km_Pop2            0.101    0.072    1.400    0.161
##     X10km_Pop2            0.126    0.067    1.895    0.058
##     GDP.Millions2        -0.082    0.046   -1.759    0.079
##     Nt_Pp._WB20152        0.006    0.092    0.063    0.950
##   X50km_Pop2 ~~
##     X10km_Pop2            0.752    0.126    5.982    0.000
##     GDP.Millions2        -0.013    0.100   -0.131    0.896
##     Nt_Pp._WB20152        0.191    0.177    1.083    0.279
##   X10km_Pop2 ~~
##     GDP.Millions2        -0.028    0.056   -0.505    0.613
##     Nt_Pp._WB20152        0.173    0.101    1.701    0.089
##   GDP.Millions2 ~~
##     Nt_Pp._WB20152        0.826    0.202    4.089    0.000
##
## Intercepts:
##                    Estimate  Std.Err  z-value  P(>|z|)
##    .Attendance2      -0.000    0.031   -0.000    1.000
##    .Total.Animals2   -0.000    0.070   -0.000    1.000
##    .Sp.Richness2      0.000    0.034    0.000    1.000
##    .Prop.Thret.Sp2    0.000    0.083    0.000    1.000
##    .Brilloun.Indx2   -0.000    0.055   -0.000    1.000
##    .Mean.Rap.Crck2    0.000    0.094    0.000    1.000
##     Zoo.Area.ha2      0.000    0.064    0.000    1.000
```

```
##     Mam.Sp.Rchnss2   -0.000    0.079   -0.000    1.000
##     Prop.Mam.Sp2      0.000    0.118    0.000    1.000
##     Men.Sp.BdyMss2   -0.000    0.080   -0.000    1.000
##     X50km_Pop2       -0.000    0.114   -0.000    1.000
##     X10km_Pop2        0.000    0.105    0.000    1.000
##     GDP.Millions2    -0.000    0.345   -0.000    1.000
##     Nt_Pp._WB20152    0.000    0.295    0.000    1.000
##
## Variances:
##                    Estimate  Std.Err  z-value  P(>|z|)
##    .Attendance2       0.267    0.028    9.566    0.000
##    .Total.Animals2    0.235    0.038    6.139    0.000
##    .Sp.Richness2      0.151    0.046    3.269    0.001
##    .Prop.Thret.Sp2    0.896    0.122    7.319    0.000
##    .Brilloun.Indx2    0.251    0.053    4.691    0.000
##    .Mean.Rap.Crck2    0.704    0.063   11.098    0.000
##     Zoo.Area.ha2      0.998    0.112    8.886    0.000
##     Mam.Sp.Rchnss2    0.998    0.115    8.669    0.000
##     Prop.Mam.Sp2      0.998    0.150    6.662    0.000
##     Men.Sp.BdyMss2    0.998    0.173    5.783    0.000
##     X50km_Pop2        0.998    0.131    7.623    0.000
##     X10km_Pop2        0.998    0.174    5.745    0.000
##     GDP.Millions2     0.998    0.246    4.061    0.000
##     Nt_Pp._WB20152    0.998    0.225    4.439    0.000
##
## R-Square:
##                    Estimate
##     Attendance2       0.718
##     Total.Animals2    0.766
##     Sp.Richness2      0.849
##     Prop.Thret.Sp2    0.101
##     Brilloun.Indx2    0.750
##     Mean.Rap.Crck2    0.291
```

```
# Generate fit indices
fitMeasures(fit.adj6, c("agfi", "rmr", "srmr", "rmsea", "cfi", "nnfi", "tli"))
```

```
##  agfi   rmr  srmr rmsea   cfi  nnfi   tli
## 0.737 0.068 0.064 0.125 0.920 0.860 0.860
```

```
# Generate modification indices
mi6adj <- modindices(fit.adj6)
print(mi6adj[mi6adj$mi > 3.0,])
```

```
##                lhs op              rhs     mi mi.scaled    epc sepc.lv
## 90    Total.Animals2 ~~   Prop.Threat.Sp2 34.979     9.124  0.134   0.134
## 91    Total.Animals2 ~~  Brillouin.Index2  5.613     1.464 -0.066  -0.066
## 92    Total.Animals2 ~~  Mean.Raup.Crick2  4.260     1.111 -0.096  -0.096
## 94      Sp.Richness2 ~~  Brillouin.Index2 12.470     3.253  0.035   0.035
## 95      Sp.Richness2 ~~  Mean.Raup.Crick2 27.601     7.199 -0.087  -0.087
## 97   Prop.Threat.Sp2 ~~  Mean.Raup.Crick2  7.213     1.881 -0.100  -0.100
## 99    Total.Animals2  ~       Attendance2  3.967     1.035  0.085   0.085
## 100   Total.Animals2  ~   Prop.Threat.Sp2 28.764     7.503  0.131   0.131
## 101   Total.Animals2  ~  Brillouin.Index2  5.614     1.464 -0.264  -0.264
## 106   Total.Animals2  ~        X50km_Pop2 36.696     9.572  0.141   0.141
## 107   Total.Animals2  ~        X10km_Pop2  7.848     2.047  0.068   0.068
```

```
## 108    Total.Animals2  ~  Nat_Pop._WB20152 10.481     2.734  0.132   0.132
## 112      Sp.Richness2  ~  Brillouin.Index2  6.033     1.574  0.081   0.081
## 113      Sp.Richness2  ~  Mean.Raup.Crick2 17.310     4.515 -0.085  -0.085
## 118      Sp.Richness2  ~  Nat_Pop._WB20152  3.059     0.798 -0.032  -0.032
## 119   Prop.Threat.Sp2  ~       Attendance2 43.084    11.238  0.379   0.379
## 120   Prop.Threat.Sp2  ~    Total.Animals2 27.892     7.275  0.272   0.272
## 121   Prop.Threat.Sp2  ~      Sp.Richness2 14.069     3.670  0.283   0.283
## 122   Prop.Threat.Sp2  ~  Mean.Raup.Crick2  4.088     1.066 -0.094  -0.094
## 124   Prop.Threat.Sp2  ~  Mam.Sp.Richness2 12.513     3.264  0.209   0.209
## 125   Prop.Threat.Sp2  ~ Mean.Sp.BodyMass2 13.244     3.454  0.206   0.206
## 126   Prop.Threat.Sp2  ~        X50km_Pop2 26.657     6.953  0.232   0.232
## 127   Prop.Threat.Sp2  ~        X10km_Pop2 30.626     7.988  0.256   0.256
## 129   Prop.Threat.Sp2  ~  Nat_Pop._WB20152  9.734     2.539  0.141   0.141
## 133  Brillouin.Index2  ~      Zoo.Area.ha2  5.613     1.464  0.062   0.062
## 135  Brillouin.Index2  ~      Prop.Mam.Sp2  7.555     1.971  0.076   0.076
## 138  Brillouin.Index2  ~        X10km_Pop2  5.633     1.469 -0.059  -0.059
## 140  Mean.Raup.Crick2  ~       Attendance2 19.945     5.202  0.407   0.407
## 141  Mean.Raup.Crick2  ~   Prop.Threat.Sp2 14.727     3.841 -0.155  -0.155
## 143  Mean.Raup.Crick2  ~      Zoo.Area.ha2  9.137     2.383  0.154   0.154
## 145  Mean.Raup.Crick2  ~      Prop.Mam.Sp2 28.743     7.497 -0.319  -0.319
## 146  Mean.Raup.Crick2  ~        X50km_Pop2 14.172     3.696  0.152   0.152
## 147  Mean.Raup.Crick2  ~        X10km_Pop2 19.556     5.101  0.187   0.187
## 149  Mean.Raup.Crick2  ~  Nat_Pop._WB20152 17.087     4.457  0.165   0.165
## 150      Zoo.Area.ha2  ~       Attendance2  5.873     1.532  0.451   0.451
## 152      Zoo.Area.ha2  ~      Sp.Richness2  9.363     2.442  0.602   0.602
## 154      Zoo.Area.ha2  ~  Brillouin.Index2  6.203     1.618  0.169   0.169
## 155      Zoo.Area.ha2  ~  Mean.Raup.Crick2  7.812     2.038  0.124   0.124
## 163  Mam.Sp.Richness2  ~       Attendance2  6.235     1.626 -0.469  -0.469
## 164  Mam.Sp.Richness2  ~    Total.Animals2  5.172     1.349 -0.226  -0.226
## 165  Mam.Sp.Richness2  ~      Sp.Richness2  9.521     2.483 -0.742  -0.742
## 166  Mam.Sp.Richness2  ~   Prop.Threat.Sp2  3.019     0.787  0.088   0.088
## 168  Mam.Sp.Richness2  ~  Mean.Raup.Crick2  5.330     1.390 -0.133  -0.133
## 180      Prop.Mam.Sp2  ~  Brillouin.Index2  3.002     0.783  0.105   0.105
## 181      Prop.Mam.Sp2  ~  Mean.Raup.Crick2 10.813     2.820 -0.141  -0.141
## 192 Mean.Sp.BodyMass2  ~   Prop.Threat.Sp2  3.504     0.914  0.061   0.061
## 202        X50km_Pop2  ~       Attendance2 30.802     8.034  0.594   0.594
## 203        X50km_Pop2  ~    Total.Animals2 29.464     7.685  0.281   0.281
## 207        X50km_Pop2  ~  Mean.Raup.Crick2  3.672     0.958  0.065   0.065
## 216        X10km_Pop2  ~    Total.Animals2  3.040     0.793 -0.083  -0.083
## 229     GDP.Millions2  ~    Total.Animals2  4.926     1.285  0.102   0.102
## 230     GDP.Millions2  ~      Sp.Richness2 30.346     7.915  0.338   0.338
## 232     GDP.Millions2  ~  Brillouin.Index2 18.587     4.848  0.173   0.173
## 233     GDP.Millions2  ~  Mean.Raup.Crick2 10.852     2.830 -0.091  -0.091
## 242  Nat_Pop._WB20152  ~    Total.Animals2  3.943     1.028 -0.079  -0.079
## 243  Nat_Pop._WB20152  ~      Sp.Richness2 31.565     8.233 -0.333  -0.333
## 244  Nat_Pop._WB20152  ~   Prop.Threat.Sp2  3.997     1.042  0.049   0.049
## 245  Nat_Pop._WB20152  ~  Brillouin.Index2 16.094     4.198 -0.139  -0.139
## 246  Nat_Pop._WB20152  ~  Mean.Raup.Crick2 15.732     4.104  0.104   0.104
##     sepc.all sepc.nox
## 90     0.134    0.134
## 91    -0.066   -0.066
## 92    -0.096   -0.096
## 94     0.035    0.035
## 95    -0.087   -0.087
```

```
## 97    -0.100   -0.100
## 99     0.083    0.083
## 100    0.130    0.130
## 101   -0.264   -0.264
## 106    0.140    0.140
## 107    0.067    0.067
## 108    0.132    0.132
## 112    0.081    0.081
## 113   -0.085   -0.085
## 118   -0.032   -0.032
## 119    0.370    0.370
## 120    0.273    0.273
## 121    0.283    0.283
## 122   -0.094   -0.094
## 124    0.209    0.209
## 125    0.206    0.206
## 126    0.232    0.232
## 127    0.256    0.257
## 129    0.141    0.142
## 133    0.062    0.062
## 135    0.076    0.076
## 138   -0.059   -0.059
## 140    0.397    0.397
## 141   -0.155   -0.155
## 143    0.155    0.155
## 145   -0.320   -0.320
## 146    0.153    0.153
## 147    0.187    0.188
## 149    0.165    0.165
## 150    0.439    0.439
## 152    0.602    0.602
## 154    0.170    0.170
## 155    0.124    0.124
## 163   -0.457   -0.457
## 164   -0.227   -0.227
## 165   -0.742   -0.742
## 166    0.088    0.088
## 168   -0.133   -0.133
## 180    0.105    0.105
## 181   -0.141   -0.141
## 192    0.061    0.061
## 202    0.579    0.579
## 203    0.282    0.282
## 207    0.065    0.065
## 216   -0.083   -0.083
## 229    0.102    0.102
## 230    0.338    0.338
## 232    0.173    0.173
## 233   -0.091   -0.091
## 242   -0.080   -0.080
## 243   -0.333   -0.333
## 244    0.049    0.049
## 245   -0.139   -0.139
## 246    0.103    0.103
```

Model 7

Based on the modification indices generated from the sixth model, there was no clear rationale for the inclusion of any particular relationship. We opted to include the relationship **Sp.Richness2 ~ Mean.Sp.BodyMass2**, as there is clear evidence within the literature that zoological institutions have a limited carrying capacity and that large vertebrates, by their very nature, require ever larger enclosure sizes. Therefore it is deducible that institutions with larger vertebrates will be able to hold fewer species. This semi-exploratory modelling approach is similar to that implemented in Grace *et al.,* 2016. As a result, we add this relationship to our model. Once again, the model summary, fit indices and modification indices were all generated for the model, adjusting for the nested nature of data.

```
# Attendance SEM (Presence-Absence)

# Model 7
# Addition of Sp.Richness2 ~ Mean.Sp.BodyMass2

mod.7 <- 'Attendance2 ~ Zoo.Area.ha2 + Sp.Richness2 + Total.Animals2
+ Mam.Sp.Richness2 + Prop.Mam.Sp2 + Prop.Threat.Sp2
+ Mean.Sp.BodyMass2 + Brillouin.Index2 + Mean.Raup.Crick2
+ X50km_Pop2 + X10km_Pop2 + GDP.Millions2 + Nat_Pop._WB20152

Total.Animals2 ~ Zoo.Area.ha2 + Sp.Richness2 + GDP.Millions2
Sp.Richness2 ~ Zoo.Area.ha2 + Prop.Mam.Sp2 + Mam.Sp.Richness2 + Mean.Sp.BodyMass2
Prop.Threat.Sp2 ~ Brillouin.Index2 + Prop.Mam.Sp2
Brillouin.Index2 ~ Sp.Richness2 + Total.Animals2 + GDP.Millions2
Mean.Raup.Crick2 ~ Sp.Richness2 + Total.Animals2 + Mean.Sp.BodyMass2'

# Fit model and generate model summary
mod.7.fit <- sem(mod.7, data = sem_attendance_data, fixed.x=FALSE)
summary(mod.7.fit, rsq = TRUE)
```

```
## lavaan (0.5-23.1097) converged normally after  55 iterations
##
##   Number of observations                           458
##
##   Estimator                                         ML
##   Minimum Function Test Statistic              291.771
##   Degrees of freedom                                35
##   P-value (Chi-square)                           0.000
##
## Parameter Estimates:
##
##   Information                                 Expected
##   Standard Errors                             Standard
##
## Regressions:
##                      Estimate  Std.Err  z-value  P(>|z|)
##   Attendance2 ~
##     Zoo.Area.ha2        0.086    0.033    2.584    0.010
##     Sp.Richness2       -0.249    0.098   -2.538    0.011
##     Total.Animals2      0.454    0.060    7.532    0.000
##     Mam.Sp.Rchnss2      0.132    0.055    2.391    0.017
##     Prop.Mam.Sp2       -0.065    0.049   -1.333    0.182
##     Prop.Thret.Sp2      0.009    0.026    0.353    0.724
##     Men.Sp.BdyMss2      0.339    0.039    8.635    0.000
```

```
##     Brilloun.Indx2      0.077    0.048    1.596    0.111
##     Mean.Rap.Crck2      0.146    0.029    5.054    0.000
##     X50km_Pop2          0.079    0.038    2.096    0.036
##     X10km_Pop2          0.397    0.041    9.624    0.000
##     GDP.Millions2       0.259    0.049    5.310    0.000
##     Nt_Pp._WB20152     -0.124    0.049   -2.523    0.012
##   Total.Animals2 ~
##     Zoo.Area.ha2        0.219    0.023    9.495    0.000
##     Sp.Richness2        0.805    0.023   34.969    0.000
##     GDP.Millions2      -0.129    0.023   -5.669    0.000
##   Sp.Richness2 ~
##     Zoo.Area.ha2        0.076    0.022    3.408    0.001
##     Prop.Mam.Sp2       -0.567    0.023  -24.670    0.000
##     Mam.Sp.Rchnss2      0.759    0.020   38.026    0.000
##     Men.Sp.BdyMss2     -0.029    0.026   -1.111    0.267
##   Prop.Threat.Sp2 ~
##     Brilloun.Indx2      0.038    0.049    0.773    0.440
##     Prop.Mam.Sp2        0.332    0.049    6.769    0.000
##   Brillouin.Index2 ~
##     Sp.Richness2        1.320    0.044   30.015    0.000
##     Total.Animals2     -0.620    0.044  -14.052    0.000
##     GDP.Millions2      -0.206    0.024   -8.518    0.000
##   Mean.Raup.Crick2 ~
##     Sp.Richness2       -0.355    0.074   -4.780    0.000
##     Total.Animals2      0.558    0.074    7.563    0.000
##     Men.Sp.BdyMss2     -0.495    0.041  -12.192    0.000
##
## Covariances:
##                      Estimate  Std.Err  z-value  P(>|z|)
##   Zoo.Area.ha2 ~~
##     Mam.Sp.Rchnss2        0.381    0.050    7.634    0.000
##     Prop.Mam.Sp2          0.312    0.049    6.389    0.000
##     Men.Sp.BdyMss2        0.534    0.053   10.096    0.000
##     X50km_Pop2            0.060    0.047    1.291    0.197
##     X10km_Pop2           -0.010    0.047   -0.208    0.835
##     GDP.Millions2        -0.027    0.047   -0.588    0.557
##     Nt_Pp._WB20152        0.061    0.047    1.310    0.190
##   Mam.Sp.Richness2 ~~
##     Prop.Mam.Sp2          0.088    0.047    1.882    0.060
##     Men.Sp.BdyMss2        0.303    0.049    6.216    0.000
##     X50km_Pop2            0.204    0.048    4.281    0.000
##     X10km_Pop2            0.284    0.048    5.852    0.000
##     GDP.Millions2        -0.060    0.047   -1.277    0.202
##     Nt_Pp._WB20152       -0.088    0.047   -1.881    0.060
##   Prop.Mam.Sp2 ~~
##     Men.Sp.BdyMss2        0.604    0.055   11.083    0.000
##     X50km_Pop2           -0.096    0.047   -2.054    0.040
##     X10km_Pop2           -0.204    0.048   -4.293    0.000
##     GDP.Millions2        -0.123    0.047   -2.615    0.009
##     Nt_Pp._WB20152       -0.115    0.047   -2.447    0.014
##   Mean.Sp.BodyMass2 ~~
##     X50km_Pop2            0.101    0.047    2.166    0.030
##     X10km_Pop2            0.126    0.047    2.683    0.007
##     GDP.Millions2        -0.082    0.047   -1.748    0.080
```

```
##     Nt_Pp._WB20152        0.006    0.047    0.124    0.901
##   X50km_Pop2 ~~
##     X10km_Pop2            0.752    0.058   12.886    0.000
##     GDP.Millions2        -0.013    0.047   -0.281    0.779
##     Nt_Pp._WB20152        0.191    0.047    4.033    0.000
##   X10km_Pop2 ~~
##     GDP.Millions2        -0.028    0.047   -0.604    0.546
##     Nt_Pp._WB20152        0.173    0.047    3.646    0.000
##   GDP.Millions2 ~~
##     Nt_Pp._WB20152        0.826    0.061   13.642    0.000
##
## Variances:
##                    Estimate  Std.Err  z-value  P(>|z|)
##    .Attendance2       0.267    0.018   15.133    0.000
##    .Total.Animals2    0.235    0.016   15.133    0.000
##    .Sp.Richness2      0.150    0.010   15.133    0.000
##    .Prop.Thret.Sp2    0.896    0.059   15.133    0.000
##    .Brilloun.Indx2    0.251    0.017   15.133    0.000
##    .Mean.Rap.Crck2    0.704    0.047   15.133    0.000
##     Zoo.Area.ha2      0.998    0.066   15.133    0.000
##     Mam.Sp.Rchnss2    0.998    0.066   15.133    0.000
##     Prop.Mam.Sp2      0.998    0.066   15.133    0.000
##     Men.Sp.BdyMss2    0.998    0.066   15.133    0.000
##     X50km_Pop2        0.998    0.066   15.133    0.000
##     X10km_Pop2        0.998    0.066   15.133    0.000
##     GDP.Millions2     0.998    0.066   15.133    0.000
##     Nt_Pp._WB20152    0.998    0.066   15.133    0.000
##
## R-Square:
##                    Estimate
##     Attendance2       0.717
##     Total.Animals2    0.766
##     Sp.Richness2      0.849
##     Prop.Thret.Sp2    0.101
##     Brilloun.Indx2    0.750
##     Mean.Rap.Crck2    0.292
```

```r
# Generate fit indices
fitMeasures(mod.7.fit, c("agfi", "rmr", "srmr", "rmsea", "cfi", "nnfi", "tli"))
```

```
##  agfi   rmr  srmr rmsea   cfi  nnfi   tli
## 0.763 0.069 0.069 0.127 0.920 0.856 0.856
```

```r
# Generate modification indices
mi7 <- modindices(mod.7.fit)
print(mi7[mi7$mi > 3.0,])
```

```
##                   lhs op               rhs     mi    epc sepc.lv sepc.all
## 77     Total.Animals2 ~~   Prop.Threat.Sp2 34.979  0.134   0.134    0.134
## 78     Total.Animals2 ~~  Brillouin.Index2  5.613 -0.066  -0.066   -0.066
## 79     Total.Animals2 ~~  Mean.Raup.Crick2  4.273 -0.096  -0.096   -0.096
## 81       Sp.Richness2 ~~  Brillouin.Index2 11.965  0.034   0.034    0.034
## 82       Sp.Richness2 ~~  Mean.Raup.Crick2 26.814 -0.086  -0.086   -0.086
## 84    Prop.Threat.Sp2 ~~  Mean.Raup.Crick2  7.213 -0.100  -0.100   -0.100
## 86     Total.Animals2  ~       Attendance2  3.928  0.085   0.085    0.082
## 87     Total.Animals2  ~   Prop.Threat.Sp2 28.764  0.131   0.131    0.130
```

```
## 88     Total.Animals2  ~  Brillouin.Index2  5.613 -0.264  -0.264   -0.264
## 93     Total.Animals2  ~        X50km_Pop2 36.649  0.140   0.140    0.140
## 94     Total.Animals2  ~        X10km_Pop2  7.814  0.067   0.067    0.067
## 95     Total.Animals2  ~  Nat_Pop._WB20152 10.485  0.132   0.132    0.132
## 99       Sp.Richness2  ~  Brillouin.Index2  5.706  0.078   0.078    0.079
## 100      Sp.Richness2  ~  Mean.Raup.Crick2 24.113 -0.109  -0.109   -0.109
## 105   Prop.Threat.Sp2  ~       Attendance2 43.011  0.379   0.379    0.369
## 106   Prop.Threat.Sp2  ~    Total.Animals2 27.892  0.272   0.272    0.273
## 107   Prop.Threat.Sp2  ~      Sp.Richness2 14.069  0.283   0.283    0.283
## 108   Prop.Threat.Sp2  ~  Mean.Raup.Crick2  4.078 -0.094  -0.094   -0.094
## 110   Prop.Threat.Sp2  ~  Mam.Sp.Richness2 12.514  0.209   0.209    0.209
## 111   Prop.Threat.Sp2  ~ Mean.Sp.BodyMass2 13.170  0.205   0.205    0.205
## 112   Prop.Threat.Sp2  ~        X50km_Pop2 26.636  0.232   0.232    0.232
## 113   Prop.Threat.Sp2  ~        X10km_Pop2 30.556  0.256   0.256    0.256
## 115   Prop.Threat.Sp2  ~  Nat_Pop._WB20152  9.739  0.141   0.141    0.141
## 119  Brillouin.Index2  ~      Zoo.Area.ha2  5.613  0.062   0.062    0.062
## 121  Brillouin.Index2  ~      Prop.Mam.Sp2  7.555  0.076   0.076    0.076
## 124  Brillouin.Index2  ~        X10km_Pop2  5.610 -0.059  -0.059   -0.059
## 126  Mean.Raup.Crick2  ~       Attendance2 19.913  0.406   0.406    0.396
## 127  Mean.Raup.Crick2  ~   Prop.Threat.Sp2 14.716 -0.155  -0.155   -0.155
## 129  Mean.Raup.Crick2  ~      Zoo.Area.ha2  9.180  0.155   0.155    0.155
## 131  Mean.Raup.Crick2  ~      Prop.Mam.Sp2 28.295 -0.314  -0.314   -0.315
## 132  Mean.Raup.Crick2  ~        X50km_Pop2 14.164  0.152   0.152    0.153
## 133  Mean.Raup.Crick2  ~        X10km_Pop2 19.507  0.187   0.187    0.187
## 135  Mean.Raup.Crick2  ~  Nat_Pop._WB20152 17.086  0.165   0.165    0.165
## 136      Zoo.Area.ha2  ~       Attendance2  5.230  0.429   0.429    0.418
## 138      Zoo.Area.ha2  ~      Sp.Richness2 12.212  1.093   1.093    1.093
## 140      Zoo.Area.ha2  ~  Brillouin.Index2  5.303  0.161   0.161    0.161
## 141      Zoo.Area.ha2  ~  Mean.Raup.Crick2  7.714  0.124   0.124    0.123
## 149  Mam.Sp.Richness2  ~       Attendance2  6.635 -0.486  -0.486   -0.473
## 150  Mam.Sp.Richness2  ~    Total.Animals2  5.978 -0.246  -0.246   -0.247
## 151  Mam.Sp.Richness2  ~      Sp.Richness2 14.531 -0.995  -0.995   -0.995
## 152  Mam.Sp.Richness2  ~   Prop.Threat.Sp2  3.025  0.088   0.088    0.088
## 154  Mam.Sp.Richness2  ~  Mean.Raup.Crick2  5.425 -0.135  -0.135   -0.135
## 162      Prop.Mam.Sp2  ~       Attendance2  3.329 -0.254  -0.254   -0.248
## 167      Prop.Mam.Sp2  ~  Mean.Raup.Crick2 10.881 -0.141  -0.141   -0.141
## 178 Mean.Sp.BodyMass2  ~   Prop.Threat.Sp2  3.556  0.061   0.061    0.061
## 188        X50km_Pop2  ~       Attendance2 30.807  0.594   0.594    0.578
## 189        X50km_Pop2  ~    Total.Animals2 29.487  0.282   0.282    0.282
## 193        X50km_Pop2  ~  Mean.Raup.Crick2  3.672  0.065   0.065    0.065
## 202        X10km_Pop2  ~    Total.Animals2  3.042 -0.083  -0.083   -0.083
## 215     GDP.Millions2  ~    Total.Animals2  4.930  0.102   0.102    0.102
## 216     GDP.Millions2  ~      Sp.Richness2 30.428  0.339   0.339    0.339
## 218     GDP.Millions2  ~  Brillouin.Index2 18.601  0.173   0.173    0.173
## 219     GDP.Millions2  ~  Mean.Raup.Crick2 10.852 -0.091  -0.091   -0.091
## 228  Nat_Pop._WB20152  ~    Total.Animals2  3.946 -0.079  -0.079   -0.080
## 229  Nat_Pop._WB20152  ~      Sp.Richness2 31.650 -0.334  -0.334   -0.334
## 230  Nat_Pop._WB20152  ~   Prop.Threat.Sp2  3.997  0.049   0.049    0.049
## 231  Nat_Pop._WB20152  ~  Brillouin.Index2 16.104 -0.139  -0.139   -0.139
## 232  Nat_Pop._WB20152  ~  Mean.Raup.Crick2 15.733  0.104   0.104    0.103
##     sepc.nox
## 77     0.134
## 78    -0.066
## 79    -0.096
```

```
## 81      0.034
## 82     -0.086
## 84     -0.100
## 86      0.082
## 87      0.130
## 88     -0.264
## 93      0.140
## 94      0.067
## 95      0.132
## 99      0.079
## 100    -0.109
## 105     0.369
## 106     0.273
## 107     0.283
## 108    -0.094
## 110     0.209
## 111     0.205
## 112     0.232
## 113     0.256
## 115     0.142
## 119     0.062
## 121     0.076
## 124    -0.059
## 126     0.396
## 127    -0.155
## 129     0.155
## 131    -0.315
## 132     0.153
## 133     0.187
## 135     0.165
## 136     0.418
## 138     1.093
## 140     0.161
## 141     0.123
## 149    -0.473
## 150    -0.247
## 151    -0.995
## 152     0.088
## 154    -0.135
## 162    -0.248
## 167    -0.141
## 178     0.061
## 188     0.578
## 189     0.282
## 193     0.065
## 202    -0.083
## 215     0.102
## 216     0.339
## 218     0.173
## 219    -0.091
## 228    -0.080
## 229    -0.334
## 230     0.049
## 231    -0.139
```

```
## 232    0.103
```

```r
# Adjust for the nested nature of the data (institutions within countries)
# Fit model and generate model summary
design <- svydesign(ids = ~Country, nest=TRUE, data=sem_attendance_data)
fit.adj7 <- lavaan.survey(lavaan.fit = mod.7.fit, survey.design = design)
summary(fit.adj7, rsq = TRUE)
```

```
## lavaan (0.5-23.1097) converged normally after  54 iterations
##
##   Number of observations                           458
##
##   Estimator                                         ML      Robust
##   Minimum Function Test Statistic              291.771      77.106
##   Degrees of freedom                                35          35
##   P-value (Chi-square)                           0.000       0.000
##   Scaling correction factor                                  3.784
##     for the Satorra-Bentler correction
##
## Parameter Estimates:
##
##   Information                                 Expected
##   Standard Errors                           Robust.sem
##
## Regressions:
##                        Estimate  Std.Err  z-value  P(>|z|)
##   Attendance2 ~
##     Zoo.Area.ha2          0.086    0.041    2.103    0.035
##     Sp.Richness2         -0.249    0.106   -2.345    0.019
##     Total.Animals2        0.454    0.068    6.725    0.000
##     Mam.Sp.Rchnss2        0.132    0.049    2.692    0.007
##     Prop.Mam.Sp2         -0.065    0.044   -1.475    0.140
##     Prop.Thret.Sp2        0.009    0.029    0.306    0.760
##     Men.Sp.BdyMss2        0.339    0.030   11.362    0.000
##     Brilloun.Indx2        0.077    0.066    1.160    0.246
##     Mean.Rap.Crck2        0.146    0.029    5.002    0.000
##     X50km_Pop2            0.079    0.033    2.363    0.018
##     X10km_Pop2            0.397    0.042    9.352    0.000
##     GDP.Millions2         0.259    0.061    4.251    0.000
##     Nt_Pp._WB20152       -0.124    0.067   -1.845    0.065
##   Total.Animals2 ~
##     Zoo.Area.ha2          0.219    0.036    5.992    0.000
##     Sp.Richness2          0.805    0.051   15.916    0.000
##     GDP.Millions2        -0.129    0.072   -1.785    0.074
##   Sp.Richness2 ~
##     Zoo.Area.ha2          0.076    0.037    2.083    0.037
##     Prop.Mam.Sp2         -0.567    0.048  -11.722    0.000
##     Mam.Sp.Rchnss2        0.759    0.072   10.553    0.000
##     Men.Sp.BdyMss2       -0.029    0.050   -0.576    0.564
##   Prop.Threat.Sp2 ~
##     Brilloun.Indx2        0.038    0.120    0.315    0.753
##     Prop.Mam.Sp2          0.332    0.070    4.742    0.000
##   Brillouin.Index2 ~
##     Sp.Richness2          1.320    0.089   14.752    0.000
##     Total.Animals2       -0.620    0.127   -4.883    0.000
```

```
##     GDP.Millions2      -0.206    0.072   -2.846    0.004
##   Mean.Raup.Crick2 ~
##     Sp.Richness2       -0.355    0.231   -1.540    0.124
##     Total.Animals2      0.558    0.155    3.610    0.000
##     Men.Sp.BdyMss2     -0.495    0.052   -9.568    0.000
##
## Covariances:
##                        Estimate  Std.Err  z-value  P(>|z|)
##   Zoo.Area.ha2 ~~
##     Mam.Sp.Rchnss2        0.381    0.064    5.985    0.000
##     Prop.Mam.Sp2          0.312    0.089    3.510    0.000
##     Men.Sp.BdyMss2        0.534    0.104    5.147    0.000
##     X50km_Pop2            0.060    0.069    0.880    0.379
##     X10km_Pop2           -0.010    0.078   -0.124    0.901
##     GDP.Millions2        -0.027    0.041   -0.673    0.501
##     Nt_Pp._WB20152        0.061    0.090    0.682    0.495
##   Mam.Sp.Richness2 ~~
##     Prop.Mam.Sp2          0.088    0.061    1.435    0.151
##     Men.Sp.BdyMss2        0.303    0.081    3.730    0.000
##     X50km_Pop2            0.204    0.082    2.487    0.013
##     X10km_Pop2            0.284    0.066    4.278    0.000
##     GDP.Millions2        -0.060    0.064   -0.937    0.349
##     Nt_Pp._WB20152       -0.088    0.102   -0.863    0.388
##   Prop.Mam.Sp2 ~~
##     Men.Sp.BdyMss2        0.604    0.130    4.656    0.000
##     X50km_Pop2           -0.096    0.051   -1.888    0.059
##     X10km_Pop2           -0.204    0.055   -3.685    0.000
##     GDP.Millions2        -0.123    0.105   -1.172    0.241
##     Nt_Pp._WB20152       -0.115    0.091   -1.257    0.209
##   Mean.Sp.BodyMass2 ~~
##     X50km_Pop2            0.101    0.072    1.400    0.161
##     X10km_Pop2            0.126    0.067    1.895    0.058
##     GDP.Millions2        -0.082    0.046   -1.759    0.079
##     Nt_Pp._WB20152        0.006    0.092    0.063    0.950
##   X50km_Pop2 ~~
##     X10km_Pop2            0.752    0.126    5.982    0.000
##     GDP.Millions2        -0.013    0.100   -0.131    0.896
##     Nt_Pp._WB20152        0.191    0.177    1.083    0.279
##   X10km_Pop2 ~~
##     GDP.Millions2        -0.028    0.056   -0.505    0.613
##     Nt_Pp._WB20152        0.173    0.101    1.701    0.089
##   GDP.Millions2 ~~
##     Nt_Pp._WB20152        0.826    0.202    4.089    0.000
##
## Intercepts:
##                    Estimate  Std.Err  z-value  P(>|z|)
##    .Attendance2      -0.000    0.031   -0.000    1.000
##    .Total.Animals2   -0.000    0.070   -0.000    1.000
##    .Sp.Richness2      0.000    0.034    0.000    1.000
##    .Prop.Thret.Sp2    0.000    0.083    0.000    1.000
##    .Brilloun.Indx2   -0.000    0.055   -0.000    1.000
##    .Mean.Rap.Crck2    0.000    0.094    0.000    1.000
##     Zoo.Area.ha2      0.000    0.064    0.000    1.000
##     Mam.Sp.Rchnss2   -0.000    0.079   -0.000    1.000
```

```
##     Prop.Mam.Sp2      0.000    0.118    0.000    1.000
##     Men.Sp.BdyMss2   -0.000    0.080   -0.000    1.000
##     X50km_Pop2       -0.000    0.114   -0.000    1.000
##     X10km_Pop2        0.000    0.105    0.000    1.000
##     GDP.Millions2    -0.000    0.345   -0.000    1.000
##     Nt_Pp._WB20152    0.000    0.295    0.000    1.000
##
## Variances:
##                    Estimate  Std.Err  z-value  P(>|z|)
##    .Attendance2       0.267    0.028    9.566    0.000
##    .Total.Animals2    0.235    0.038    6.139    0.000
##    .Sp.Richness2      0.150    0.045    3.344    0.001
##    .Prop.Thret.Sp2    0.896    0.122    7.319    0.000
##    .Brilloun.Indx2    0.251    0.053    4.691    0.000
##    .Mean.Rap.Crck2    0.704    0.063   11.098    0.000
##     Zoo.Area.ha2      0.998    0.112    8.886    0.000
##     Mam.Sp.Rchnss2    0.998    0.115    8.669    0.000
##     Prop.Mam.Sp2      0.998    0.150    6.662    0.000
##     Men.Sp.BdyMss2    0.998    0.173    5.783    0.000
##     X50km_Pop2        0.998    0.131    7.623    0.000
##     X10km_Pop2        0.998    0.174    5.745    0.000
##     GDP.Millions2     0.998    0.246    4.061    0.000
##     Nt_Pp._WB20152    0.998    0.225    4.439    0.000
##
## R-Square:
##                    Estimate
##     Attendance2       0.717
##     Total.Animals2    0.766
##     Sp.Richness2      0.849
##     Prop.Thret.Sp2    0.101
##     Brilloun.Indx2    0.750
##     Mean.Rap.Crck2    0.292
```

```r
# Generate fit indices
fitMeasures(fit.adj7, c("agfi", "rmr", "srmr", "rmsea", "cfi", "nnfi", "tli"))
```

```
##  agfi   rmr  srmr rmsea   cfi  nnfi   tli
## 0.731 0.069 0.065 0.127 0.920 0.856 0.856
```

```r
# Generate modification indices
mi7adj <- modindices(fit.adj7)
print(mi7adj[mi7adj$mi > 3.0,])
```

```
##                lhs op              rhs     mi mi.scaled    epc sepc.lv
## 91     Total.Animals2 ~~   Prop.Threat.Sp2 34.979     9.244  0.134   0.134
## 92     Total.Animals2 ~~  Brillouin.Index2  5.613     1.483 -0.066  -0.066
## 93     Total.Animals2 ~~  Mean.Raup.Crick2  4.273     1.129 -0.096  -0.096
## 95       Sp.Richness2 ~~  Brillouin.Index2 11.965     3.162  0.034   0.034
## 96       Sp.Richness2 ~~  Mean.Raup.Crick2 26.814     7.086 -0.086  -0.086
## 98    Prop.Threat.Sp2 ~~  Mean.Raup.Crick2  7.213     1.906 -0.100  -0.100
## 100    Total.Animals2  ~       Attendance2  3.928     1.038  0.085   0.085
## 101    Total.Animals2  ~   Prop.Threat.Sp2 28.764     7.602  0.131   0.131
## 102    Total.Animals2  ~  Brillouin.Index2  5.613     1.483 -0.264  -0.264
## 107    Total.Animals2  ~        X50km_Pop2 36.649     9.685  0.140   0.140
## 108    Total.Animals2  ~        X10km_Pop2  7.814     2.065  0.067   0.067
## 109    Total.Animals2  ~  Nat_Pop._WB20152 10.485     2.771  0.132   0.132
```

```
## 113      Sp.Richness2  ~  Brillouin.Index2  5.707     1.508  0.078   0.078
## 114      Sp.Richness2  ~  Mean.Raup.Crick2 24.113     6.372 -0.109  -0.109
## 119   Prop.Threat.Sp2  ~       Attendance2 43.012    11.367  0.379   0.379
## 120   Prop.Threat.Sp2  ~     Total.Animals2 27.892     7.371  0.272   0.272
## 121   Prop.Threat.Sp2  ~      Sp.Richness2 14.069     3.718  0.283   0.283
## 122   Prop.Threat.Sp2  ~  Mean.Raup.Crick2  4.078     1.078 -0.094  -0.094
## 124   Prop.Threat.Sp2  ~  Mam.Sp.Richness2 12.514     3.307  0.209   0.209
## 125   Prop.Threat.Sp2  ~ Mean.Sp.BodyMass2 13.170     3.480  0.205   0.205
## 126   Prop.Threat.Sp2  ~       X50km_Pop2 26.636     7.039  0.232   0.232
## 127   Prop.Threat.Sp2  ~       X10km_Pop2 30.556     8.075  0.256   0.256
## 129   Prop.Threat.Sp2  ~  Nat_Pop._WB20152  9.739     2.574  0.141   0.141
## 133  Brillouin.Index2  ~       Zoo.Area.ha2  5.613     1.483  0.062   0.062
## 135  Brillouin.Index2  ~      Prop.Mam.Sp2  7.555     1.996  0.076   0.076
## 138  Brillouin.Index2  ~       X10km_Pop2  5.610     1.482 -0.059  -0.059
## 140  Mean.Raup.Crick2  ~       Attendance2 19.913     5.262  0.406   0.406
## 141  Mean.Raup.Crick2  ~   Prop.Threat.Sp2 14.716     3.889 -0.155  -0.155
## 143  Mean.Raup.Crick2  ~      Zoo.Area.ha2  9.180     2.426  0.155   0.155
## 145  Mean.Raup.Crick2  ~      Prop.Mam.Sp2 28.295     7.477 -0.314  -0.314
## 146  Mean.Raup.Crick2  ~       X50km_Pop2 14.164     3.743  0.152   0.152
## 147  Mean.Raup.Crick2  ~       X10km_Pop2 19.507     5.155  0.187   0.187
## 149  Mean.Raup.Crick2  ~  Nat_Pop._WB20152 17.086     4.515  0.165   0.165
## 150      Zoo.Area.ha2  ~       Attendance2  5.230     1.382  0.429   0.429
## 152      Zoo.Area.ha2  ~      Sp.Richness2 12.211     3.227  1.093   1.093
## 154      Zoo.Area.ha2  ~  Brillouin.Index2  5.303     1.401  0.161   0.161
## 155      Zoo.Area.ha2  ~  Mean.Raup.Crick2  7.714     2.039  0.124   0.124
## 163  Mam.Sp.Richness2  ~       Attendance2  6.634     1.753 -0.486  -0.486
## 164  Mam.Sp.Richness2  ~     Total.Animals2  5.978     1.580 -0.246  -0.246
## 165  Mam.Sp.Richness2  ~      Sp.Richness2 14.530     3.840 -0.995  -0.995
## 166  Mam.Sp.Richness2  ~   Prop.Threat.Sp2  3.025     0.799  0.088   0.088
## 168  Mam.Sp.Richness2  ~  Mean.Raup.Crick2  5.425     1.434 -0.135  -0.135
## 176      Prop.Mam.Sp2  ~       Attendance2  3.329     0.880 -0.254  -0.254
## 181      Prop.Mam.Sp2  ~  Mean.Raup.Crick2 10.881     2.876 -0.141  -0.141
## 192 Mean.Sp.BodyMass2  ~   Prop.Threat.Sp2  3.556     0.940  0.061   0.061
## 202        X50km_Pop2  ~       Attendance2 30.807     8.141  0.594   0.594
## 203        X50km_Pop2  ~     Total.Animals2 29.487     7.793  0.282   0.282
## 207        X50km_Pop2  ~  Mean.Raup.Crick2  3.672     0.970  0.065   0.065
## 216        X10km_Pop2  ~     Total.Animals2  3.042     0.804 -0.083  -0.083
## 229     GDP.Millions2  ~     Total.Animals2  4.930     1.303  0.102   0.102
## 230     GDP.Millions2  ~      Sp.Richness2 30.428     8.041  0.339   0.339
## 232     GDP.Millions2  ~  Brillouin.Index2 18.601     4.916  0.173   0.173
## 233     GDP.Millions2  ~  Mean.Raup.Crick2 10.852     2.868 -0.091  -0.091
## 242  Nat_Pop._WB20152  ~     Total.Animals2  3.946     1.043 -0.079  -0.079
## 243  Nat_Pop._WB20152  ~      Sp.Richness2 31.650     8.364 -0.334  -0.334
## 244  Nat_Pop._WB20152  ~   Prop.Threat.Sp2  3.997     1.056  0.049   0.049
## 245  Nat_Pop._WB20152  ~  Brillouin.Index2 16.104     4.256 -0.139  -0.139
## 246  Nat_Pop._WB20152  ~  Mean.Raup.Crick2 15.732     4.158  0.104   0.104
##     sepc.all sepc.nox
## 91     0.134    0.134
## 92    -0.066   -0.066
## 93    -0.096   -0.096
## 95     0.034    0.034
## 96    -0.086   -0.086
## 98    -0.100   -0.100
## 100    0.082    0.082
```

```
## 101    0.130    0.130
## 102   -0.264   -0.264
## 107    0.140    0.140
## 108    0.067    0.067
## 109    0.132    0.132
## 113    0.079    0.079
## 114   -0.109   -0.109
## 119    0.369    0.369
## 120    0.273    0.273
## 121    0.283    0.283
## 122   -0.094   -0.094
## 124    0.209    0.209
## 125    0.205    0.205
## 126    0.232    0.232
## 127    0.256    0.256
## 129    0.141    0.142
## 133    0.062    0.062
## 135    0.076    0.076
## 138   -0.059   -0.059
## 140    0.396    0.396
## 141   -0.155   -0.155
## 143    0.155    0.155
## 145   -0.315   -0.315
## 146    0.153    0.153
## 147    0.187    0.187
## 149    0.165    0.165
## 150    0.418    0.418
## 152    1.093    1.093
## 154    0.161    0.161
## 155    0.123    0.123
## 163   -0.473   -0.473
## 164   -0.247   -0.247
## 165   -0.995   -0.995
## 166    0.088    0.088
## 168   -0.135   -0.135
## 176   -0.248   -0.248
## 181   -0.141   -0.141
## 192    0.061    0.061
## 202    0.578    0.578
## 203    0.282    0.282
## 207    0.065    0.065
## 216   -0.083   -0.083
## 229    0.102    0.102
## 230    0.339    0.339
## 232    0.173    0.173
## 233   -0.091   -0.091
## 242   -0.080   -0.080
## 243   -0.334   -0.334
## 244    0.049    0.049
## 245   -0.139   -0.139
## 246    0.103    0.103
```

Model 8

Based on the modification indices generated from the seventh model, the most intuitive relationship with the highest mi value was identified as **Mean.Raup.Crick2 ~ Prop.Threat.Sp2**, with a corresponding mi value of **14.716**. Although not the highest mi value, this is the most intuitive and still exceeds the standard cut-off level for the chi-square test criterion of 3.84 (Burnham and Anderson, 2002). This is considered intuituive as it is logical to assume that instiutions with a higher proportion of threatened species will be dissimilar to other institutions, as traditionally species in zoos are often less threatened than their close relatives not held in zoos and are found in large numbers (Frynta *et al., 2013*; Martin *et al.,* 2014). Therefore threatened species are likely to be less common and found in fewer numbers, making collections housing them more dissimilar to other institutions. As a result, we add this relationship to our model. Once again, the model summary, fit indices and modification indices were all generated for the model, adjusting for the nested nature of data.

```
# Attendance SEM (Presence-Absence)

# Model 8
# Addition of Mean.Raup.Crick2 ~ Prop.Threat.Sp2

mod.8 <- 'Attendance2 ~ Zoo.Area.ha2 + Sp.Richness2 + Total.Animals2
+ Mam.Sp.Richness2 + Prop.Mam.Sp2 + Prop.Threat.Sp2
+ Mean.Sp.BodyMass2 + Brillouin.Index2 + Mean.Raup.Crick2
+ X50km_Pop2 + X10km_Pop2 + GDP.Millions2 + Nat_Pop._WB20152

Total.Animals2 ~ Zoo.Area.ha2 + Sp.Richness2 + GDP.Millions2
Sp.Richness2 ~ Zoo.Area.ha2 + Prop.Mam.Sp2 + Mam.Sp.Richness2 + Mean.Sp.BodyMass2
Prop.Threat.Sp2 ~ Brillouin.Index2 + Prop.Mam.Sp2
Brillouin.Index2 ~ Sp.Richness2 + Total.Animals2 + GDP.Millions2
Mean.Raup.Crick2 ~ Sp.Richness2 + Total.Animals2 + Mean.Sp.BodyMass2 + Prop.Threat.Sp2'

# Fit model and generate model summary
mod.8.fit <- sem(mod.8, data = sem_attendance_data, fixed.x=FALSE)
summary(mod.8.fit, rsq = TRUE)
```

```
## lavaan (0.5-23.1097) converged normally after  55 iterations
##
##   Number of observations                           458
##
##   Estimator                                         ML
##   Minimum Function Test Statistic              275.287
##   Degrees of freedom                                34
##   P-value (Chi-square)                           0.000
##
## Parameter Estimates:
##
##   Information                                 Expected
##   Standard Errors                             Standard
##
## Regressions:
##                    Estimate  Std.Err  z-value  P(>|z|)
##   Attendance2 ~
##     Zoo.Area.ha2      0.086    0.033    2.584    0.010
##     Sp.Richness2     -0.249    0.098   -2.533    0.011
##     Total.Animals2    0.454    0.061    7.461    0.000
##     Mam.Sp.Rchnss2    0.132    0.055    2.391    0.017
```

```
##     Prop.Mam.Sp2       -0.065    0.049   -1.333    0.182
##     Prop.Thret.Sp2      0.009    0.026    0.346    0.729
##     Men.Sp.BdyMss2      0.339    0.039    8.723    0.000
##     Brilloun.Indx2      0.077    0.048    1.596    0.111
##     Mean.Rap.Crck2      0.146    0.029    4.964    0.000
##     X50km_Pop2          0.079    0.038    2.096    0.036
##     X10km_Pop2          0.397    0.041    9.624    0.000
##     GDP.Millions2       0.259    0.049    5.310    0.000
##     Nt_Pp._WB20152     -0.124    0.049   -2.523    0.012
##   Total.Animals2 ~
##     Zoo.Area.ha2        0.219    0.023    9.495    0.000
##     Sp.Richness2        0.805    0.023   34.969    0.000
##     GDP.Millions2      -0.129    0.023   -5.669    0.000
##   Sp.Richness2 ~
##     Zoo.Area.ha2        0.076    0.022    3.408    0.001
##     Prop.Mam.Sp2       -0.567    0.023  -24.670    0.000
##     Mam.Sp.Rchnss2      0.759    0.020   38.026    0.000
##     Men.Sp.BdyMss2     -0.029    0.026   -1.111    0.267
##   Prop.Threat.Sp2 ~
##     Brilloun.Indx2      0.038    0.049    0.773    0.440
##     Prop.Mam.Sp2        0.332    0.049    6.769    0.000
##   Brillouin.Index2 ~
##     Sp.Richness2        1.320    0.044   30.015    0.000
##     Total.Animals2     -0.620    0.044  -14.052    0.000
##     GDP.Millions2      -0.206    0.024   -8.518    0.000
##   Mean.Raup.Crick2 ~
##     Sp.Richness2       -0.407    0.073   -5.560    0.000
##     Total.Animals2      0.618    0.073    8.526    0.000
##     Men.Sp.BdyMss2     -0.448    0.041  -11.046    0.000
##     Prop.Thret.Sp2     -0.170    0.040   -4.297    0.000
##
## Covariances:
##                        Estimate  Std.Err  z-value  P(>|z|)
##   Zoo.Area.ha2 ~~
##     Mam.Sp.Rchnss2        0.381    0.050    7.634    0.000
##     Prop.Mam.Sp2          0.312    0.049    6.389    0.000
##     Men.Sp.BdyMss2        0.534    0.053   10.096    0.000
##     X50km_Pop2            0.060    0.047    1.291    0.197
##     X10km_Pop2           -0.010    0.047   -0.208    0.835
##     GDP.Millions2        -0.027    0.047   -0.588    0.557
##     Nt_Pp._WB20152        0.061    0.047    1.310    0.190
##   Mam.Sp.Richness2 ~~
##     Prop.Mam.Sp2          0.088    0.047    1.882    0.060
##     Men.Sp.BdyMss2        0.303    0.049    6.216    0.000
##     X50km_Pop2            0.204    0.048    4.281    0.000
##     X10km_Pop2            0.284    0.048    5.852    0.000
##     GDP.Millions2        -0.060    0.047   -1.277    0.202
##     Nt_Pp._WB20152       -0.088    0.047   -1.881    0.060
##   Prop.Mam.Sp2 ~~
##     Men.Sp.BdyMss2        0.604    0.055   11.083    0.000
##     X50km_Pop2           -0.096    0.047   -2.054    0.040
##     X10km_Pop2           -0.204    0.048   -4.293    0.000
##     GDP.Millions2        -0.123    0.047   -2.615    0.009
##     Nt_Pp._WB20152       -0.115    0.047   -2.447    0.014
```

```
##   Mean.Sp.BodyMass2 ~~
##     X50km_Pop2           0.101    0.047    2.166    0.030
##     X10km_Pop2           0.126    0.047    2.683    0.007
##     GDP.Millions2       -0.082    0.047   -1.748    0.080
##     Nt_Pp._WB20152       0.006    0.047    0.124    0.901
##   X50km_Pop2 ~~
##     X10km_Pop2           0.752    0.058   12.886    0.000
##     GDP.Millions2       -0.013    0.047   -0.281    0.779
##     Nt_Pp._WB20152       0.191    0.047    4.033    0.000
##   X10km_Pop2 ~~
##     GDP.Millions2       -0.028    0.047   -0.604    0.546
##     Nt_Pp._WB20152       0.173    0.047    3.646    0.000
##   GDP.Millions2 ~~
##     Nt_Pp._WB20152       0.826    0.061   13.642    0.000
##
## Variances:
##                    Estimate  Std.Err  z-value  P(>|z|)
##    .Attendance2       0.267    0.018   15.133    0.000
##    .Total.Animals2    0.235    0.016   15.133    0.000
##    .Sp.Richness2      0.150    0.010   15.133    0.000
##    .Prop.Thret.Sp2    0.896    0.059   15.133    0.000
##    .Brilloun.Indx2    0.251    0.017   15.133    0.000
##    .Mean.Rap.Crck2    0.679    0.045   15.133    0.000
##     Zoo.Area.ha2      0.998    0.066   15.133    0.000
##     Mam.Sp.Rchnss2    0.998    0.066   15.133    0.000
##     Prop.Mam.Sp2      0.998    0.066   15.133    0.000
##     Men.Sp.BdyMss2    0.998    0.066   15.133    0.000
##     X50km_Pop2        0.998    0.066   15.133    0.000
##     X10km_Pop2        0.998    0.066   15.133    0.000
##     GDP.Millions2     0.998    0.066   15.133    0.000
##     Nt_Pp._WB20152    0.998    0.066   15.133    0.000
##
## R-Square:
##                    Estimate
##     Attendance2       0.720
##     Total.Animals2    0.766
##     Sp.Richness2      0.849
##     Prop.Thret.Sp2    0.101
##     Brilloun.Indx2    0.750
##     Mean.Rap.Crck2    0.322
```

```r
# Generate fit indices
fitMeasures(mod.8.fit, c("agfi", "rmr", "srmr", "rmsea", "cfi", "nnfi", "tli"))
```

```
##  agfi   rmr  srmr rmsea   cfi  nnfi   tli
## 0.760 0.066 0.067 0.124 0.925 0.860 0.860
```

```r
# Generate modification indices
mi8 <- modindices(mod.8.fit)
print(mi8[mi8$mi > 3.0,])
```

```
##                  lhs op              rhs     mi    epc sepc.lv sepc.all
## 78     Total.Animals2 ~~   Prop.Threat.Sp2 34.979  0.134   0.134    0.134
## 79     Total.Animals2 ~~  Brillouin.Index2  5.613 -0.066  -0.066   -0.066
## 82       Sp.Richness2 ~~  Brillouin.Index2 11.965  0.034   0.034    0.034
## 83       Sp.Richness2 ~~  Mean.Raup.Crick2 25.675 -0.082  -0.082   -0.082
```

```
## 85    Prop.Threat.Sp2 ~~  Mean.Raup.Crick2 22.929  0.764   0.764    0.764
## 87     Total.Animals2  ~       Attendance2  3.216  0.076   0.076    0.074
## 88     Total.Animals2  ~   Prop.Threat.Sp2 28.764  0.131   0.131    0.130
## 89     Total.Animals2  ~  Brillouin.Index2  5.614 -0.264  -0.264   -0.264
## 90     Total.Animals2  ~  Mean.Raup.Crick2  5.050 -0.095  -0.095   -0.095
## 94     Total.Animals2  ~       X50km_Pop2 36.649  0.140   0.140    0.140
## 95     Total.Animals2  ~       X10km_Pop2  7.814  0.067   0.067    0.067
## 96     Total.Animals2  ~  Nat_Pop._WB20152 10.485  0.132   0.132    0.132
## 100       Sp.Richness2  ~  Brillouin.Index2  5.707  0.078   0.078    0.079
## 101       Sp.Richness2  ~  Mean.Raup.Crick2 22.733 -0.105  -0.105   -0.105
## 106    Prop.Threat.Sp2  ~       Attendance2 52.511  0.420   0.420    0.411
## 107    Prop.Threat.Sp2  ~    Total.Animals2 27.892  0.272   0.272    0.273
## 108    Prop.Threat.Sp2  ~      Sp.Richness2 14.069  0.283   0.283    0.283
## 109    Prop.Threat.Sp2  ~  Mean.Raup.Crick2 10.329  0.298   0.298    0.299
## 111    Prop.Threat.Sp2  ~  Mam.Sp.Richness2 12.514  0.209   0.209    0.209
## 112    Prop.Threat.Sp2  ~ Mean.Sp.BodyMass2 13.170  0.205   0.205    0.205
## 113    Prop.Threat.Sp2  ~       X50km_Pop2 26.636  0.232   0.232    0.232
## 114    Prop.Threat.Sp2  ~       X10km_Pop2 30.556  0.256   0.256    0.256
## 116    Prop.Threat.Sp2  ~  Nat_Pop._WB20152  9.739  0.141   0.141    0.141
## 120  Brillouin.Index2  ~      Zoo.Area.ha2  5.613  0.062   0.062    0.062
## 122  Brillouin.Index2  ~      Prop.Mam.Sp2  7.555  0.076   0.076    0.076
## 125  Brillouin.Index2  ~       X10km_Pop2  5.610 -0.059  -0.059   -0.059
## 127  Mean.Raup.Crick2  ~       Attendance2 27.465  0.468   0.468    0.457
## 129  Mean.Raup.Crick2  ~      Zoo.Area.ha2  6.928  0.132   0.132    0.132
## 131  Mean.Raup.Crick2  ~      Prop.Mam.Sp2 22.676 -0.284  -0.284   -0.283
## 132  Mean.Raup.Crick2  ~       X50km_Pop2 19.180  0.174   0.174    0.174
## 133  Mean.Raup.Crick2  ~       X10km_Pop2 25.871  0.211   0.211    0.211
## 135  Mean.Raup.Crick2  ~  Nat_Pop._WB20152 22.005  0.184   0.184    0.184
## 136       Zoo.Area.ha2  ~       Attendance2  4.440  0.393   0.393    0.384
## 138       Zoo.Area.ha2  ~      Sp.Richness2 12.212  1.093   1.093    1.093
## 140       Zoo.Area.ha2  ~  Brillouin.Index2  5.303  0.161   0.161    0.161
## 141       Zoo.Area.ha2  ~  Mean.Raup.Crick2  6.183  0.110   0.110    0.110
## 149  Mam.Sp.Richness2  ~       Attendance2  6.094 -0.461  -0.461   -0.451
## 150  Mam.Sp.Richness2  ~    Total.Animals2  5.978 -0.246  -0.246   -0.247
## 151  Mam.Sp.Richness2  ~      Sp.Richness2 14.531 -0.995  -0.995   -0.995
## 152  Mam.Sp.Richness2  ~   Prop.Threat.Sp2  3.025  0.088   0.088    0.088
## 154  Mam.Sp.Richness2  ~  Mean.Raup.Crick2  4.580 -0.123  -0.123   -0.123
## 167       Prop.Mam.Sp2  ~  Mean.Raup.Crick2  5.383 -0.101  -0.101   -0.101
## 178 Mean.Sp.BodyMass2  ~   Prop.Threat.Sp2  3.556  0.061   0.061    0.061
## 188         X50km_Pop2  ~       Attendance2 30.164  0.582   0.582    0.569
## 189         X50km_Pop2  ~    Total.Animals2 29.487  0.282   0.282    0.282
## 193         X50km_Pop2  ~  Mean.Raup.Crick2  3.587  0.063   0.063    0.064
## 202         X10km_Pop2  ~    Total.Animals2  3.042 -0.083  -0.083   -0.083
## 215      GDP.Millions2  ~    Total.Animals2  4.930  0.102   0.102    0.102
## 216      GDP.Millions2  ~      Sp.Richness2 30.428  0.339   0.339    0.339
## 218      GDP.Millions2  ~  Brillouin.Index2 18.601  0.173   0.173    0.173
## 219      GDP.Millions2  ~  Mean.Raup.Crick2 10.096 -0.087  -0.087   -0.087
## 228  Nat_Pop._WB20152  ~    Total.Animals2  3.946 -0.079  -0.079   -0.080
## 229  Nat_Pop._WB20152  ~      Sp.Richness2 31.650 -0.334  -0.334   -0.334
## 230  Nat_Pop._WB20152  ~   Prop.Threat.Sp2  3.997  0.049   0.049    0.049
## 231  Nat_Pop._WB20152  ~  Brillouin.Index2 16.104 -0.139  -0.139   -0.139
## 232  Nat_Pop._WB20152  ~  Mean.Raup.Crick2 15.367  0.101   0.101    0.101
##     sepc.nox
## 78     0.134
```

```
## 79    -0.066
## 82     0.034
## 83    -0.082
## 85     0.764
## 87     0.074
## 88     0.130
## 89    -0.264
## 90    -0.095
## 94     0.140
## 95     0.067
## 96     0.132
## 100    0.079
## 101   -0.105
## 106    0.411
## 107    0.273
## 108    0.283
## 109    0.299
## 111    0.209
## 112    0.205
## 113    0.232
## 114    0.256
## 116    0.142
## 120    0.062
## 122    0.076
## 125   -0.059
## 127    0.457
## 129    0.132
## 131   -0.283
## 132    0.174
## 133    0.211
## 135    0.184
## 136    0.384
## 138    1.093
## 140    0.161
## 141    0.110
## 149   -0.451
## 150   -0.247
## 151   -0.995
## 152    0.088
## 154   -0.123
## 167   -0.101
## 178    0.061
## 188    0.569
## 189    0.282
## 193    0.064
## 202   -0.083
## 215    0.102
## 216    0.339
## 218    0.173
## 219   -0.087
## 228   -0.080
## 229   -0.334
## 230    0.049
## 231   -0.139
```

```
## 232    0.101
```

```
# Adjust for the nested nature of the data (institutions within countries)
# Fit model and generate model summary
design <- svydesign(ids = ~Country, nest=TRUE, data=sem_attendance_data)
fit.adj8 <- lavaan.survey(lavaan.fit = mod.8.fit, survey.design = design)
summary(fit.adj8, rsq = TRUE)
```

```
## lavaan (0.5-23.1097) converged normally after  55 iterations
##
##   Number of observations                           458
##
##   Estimator                                         ML      Robust
##   Minimum Function Test Statistic              275.287      70.561
##   Degrees of freedom                                34          34
##   P-value (Chi-square)                           0.000       0.000
##   Scaling correction factor                                  3.901
##     for the Satorra-Bentler correction
##
## Parameter Estimates:
##
##   Information                                 Expected
##   Standard Errors                           Robust.sem
##
## Regressions:
##                      Estimate  Std.Err  z-value  P(>|z|)
##   Attendance2 ~
##     Zoo.Area.ha2        0.086    0.041    2.103    0.035
##     Sp.Richness2       -0.249    0.108   -2.317    0.021
##     Total.Animals2      0.454    0.069    6.543    0.000
##     Mam.Sp.Rchnss2      0.132    0.049    2.692    0.007
##     Prop.Mam.Sp2       -0.065    0.044   -1.475    0.140
##     Prop.Thret.Sp2      0.009    0.030    0.299    0.765
##     Men.Sp.BdyMss2      0.339    0.029   11.775    0.000
##     Brilloun.Indx2      0.077    0.066    1.160    0.246
##     Mean.Rap.Crck2      0.146    0.030    4.808    0.000
##     X50km_Pop2          0.079    0.033    2.363    0.018
##     X10km_Pop2          0.397    0.042    9.352    0.000
##     GDP.Millions2       0.259    0.061    4.251    0.000
##     Nt_Pp._WB20152     -0.124    0.067   -1.845    0.065
##   Total.Animals2 ~
##     Zoo.Area.ha2        0.219    0.036    5.992    0.000
##     Sp.Richness2        0.805    0.051   15.916    0.000
##     GDP.Millions2      -0.129    0.072   -1.785    0.074
##   Sp.Richness2 ~
##     Zoo.Area.ha2        0.076    0.037    2.083    0.037
##     Prop.Mam.Sp2       -0.567    0.048  -11.722    0.000
##     Mam.Sp.Rchnss2      0.759    0.072   10.553    0.000
##     Men.Sp.BdyMss2     -0.029    0.050   -0.576    0.564
##   Prop.Threat.Sp2 ~
##     Brilloun.Indx2      0.038    0.120    0.315    0.753
##     Prop.Mam.Sp2        0.332    0.070    4.742    0.000
##   Brillouin.Index2 ~
##     Sp.Richness2        1.320    0.089   14.752    0.000
##     Total.Animals2     -0.620    0.127   -4.883    0.000
```

```
##     GDP.Millions2      -0.206    0.072   -2.846    0.004
##   Mean.Raup.Crick2 ~
##     Sp.Richness2       -0.407    0.238   -1.712    0.087
##     Total.Animals2      0.618    0.155    3.990    0.000
##     Men.Sp.BdyMss2     -0.448    0.045   -9.925    0.000
##     Prop.Thret.Sp2     -0.170    0.059   -2.874    0.004
##
## Covariances:
##                        Estimate  Std.Err  z-value  P(>|z|)
##   Zoo.Area.ha2 ~~
##     Mam.Sp.Rchnss2        0.381    0.064    5.985    0.000
##     Prop.Mam.Sp2          0.312    0.089    3.510    0.000
##     Men.Sp.BdyMss2        0.534    0.104    5.147    0.000
##     X50km_Pop2            0.060    0.069    0.880    0.379
##     X10km_Pop2           -0.010    0.078   -0.124    0.901
##     GDP.Millions2        -0.027    0.041   -0.673    0.501
##     Nt_Pp._WB20152        0.061    0.090    0.682    0.495
##   Mam.Sp.Richness2 ~~
##     Prop.Mam.Sp2          0.088    0.061    1.435    0.151
##     Men.Sp.BdyMss2        0.303    0.081    3.730    0.000
##     X50km_Pop2            0.204    0.082    2.487    0.013
##     X10km_Pop2            0.284    0.066    4.278    0.000
##     GDP.Millions2        -0.060    0.064   -0.937    0.349
##     Nt_Pp._WB20152       -0.088    0.102   -0.863    0.388
##   Prop.Mam.Sp2 ~~
##     Men.Sp.BdyMss2        0.604    0.130    4.656    0.000
##     X50km_Pop2           -0.096    0.051   -1.888    0.059
##     X10km_Pop2           -0.204    0.055   -3.685    0.000
##     GDP.Millions2        -0.123    0.105   -1.172    0.241
##     Nt_Pp._WB20152       -0.115    0.091   -1.257    0.209
##   Mean.Sp.BodyMass2 ~~
##     X50km_Pop2            0.101    0.072    1.400    0.161
##     X10km_Pop2            0.126    0.067    1.895    0.058
##     GDP.Millions2        -0.082    0.046   -1.759    0.079
##     Nt_Pp._WB20152        0.006    0.092    0.063    0.950
##   X50km_Pop2 ~~
##     X10km_Pop2            0.752    0.126    5.982    0.000
##     GDP.Millions2        -0.013    0.100   -0.131    0.896
##     Nt_Pp._WB20152        0.191    0.177    1.083    0.279
##   X10km_Pop2 ~~
##     GDP.Millions2        -0.028    0.056   -0.505    0.613
##     Nt_Pp._WB20152        0.173    0.101    1.701    0.089
##   GDP.Millions2 ~~
##     Nt_Pp._WB20152        0.826    0.202    4.089    0.000
##
## Intercepts:
##                    Estimate  Std.Err  z-value  P(>|z|)
##    .Attendance2      -0.000    0.031   -0.000    1.000
##    .Total.Animals2   -0.000    0.070   -0.000    1.000
##    .Sp.Richness2      0.000    0.034    0.000    1.000
##    .Prop.Thret.Sp2    0.000    0.083    0.000    1.000
##    .Brilloun.Indx2   -0.000    0.055   -0.000    1.000
##    .Mean.Rap.Crck2    0.000    0.089    0.000    1.000
##     Zoo.Area.ha2      0.000    0.064    0.000    1.000
```

```
##     Mam.Sp.Rchnss2   -0.000    0.079   -0.000    1.000
##     Prop.Mam.Sp2      0.000    0.118    0.000    1.000
##     Men.Sp.BdyMss2   -0.000    0.080   -0.000    1.000
##     X50km_Pop2       -0.000    0.114   -0.000    1.000
##     X10km_Pop2        0.000    0.105    0.000    1.000
##     GDP.Millions2    -0.000    0.345   -0.000    1.000
##     Nt_Pp._WB20152    0.000    0.295    0.000    1.000
##
## Variances:
##                    Estimate  Std.Err  z-value  P(>|z|)
##    .Attendance2       0.267    0.028    9.566    0.000
##    .Total.Animals2    0.235    0.038    6.139    0.000
##    .Sp.Richness2      0.150    0.045    3.344    0.001
##    .Prop.Thret.Sp2    0.896    0.122    7.319    0.000
##    .Brilloun.Indx2    0.251    0.053    4.691    0.000
##    .Mean.Rap.Crck2    0.679    0.074    9.218    0.000
##     Zoo.Area.ha2      0.998    0.112    8.886    0.000
##     Mam.Sp.Rchnss2    0.998    0.115    8.669    0.000
##     Prop.Mam.Sp2      0.998    0.150    6.662    0.000
##     Men.Sp.BdyMss2    0.998    0.173    5.783    0.000
##     X50km_Pop2        0.998    0.131    7.623    0.000
##     X10km_Pop2        0.998    0.174    5.745    0.000
##     GDP.Millions2     0.998    0.246    4.061    0.000
##     Nt_Pp._WB20152    0.998    0.225    4.439    0.000
##
## R-Square:
##                    Estimate
##     Attendance2       0.720
##     Total.Animals2    0.766
##     Sp.Richness2      0.849
##     Prop.Thret.Sp2    0.101
##     Brilloun.Indx2    0.750
##     Mean.Rap.Crck2    0.322
```

```r
# Generate fit indices
fitMeasures(fit.adj8, c("agfi", "rmr", "srmr", "rmsea", "cfi", "nnfi", "tli"))
```

```
##  agfi   rmr  srmr rmsea   cfi  nnfi   tli
## 0.728 0.066 0.063 0.124 0.925 0.860 0.860
```

```r
# Generate modification indices
mi8adj <- modindices(fit.adj8)
print(mi8adj[mi8adj$mi > 3.0,])
```

```
##                  lhs op              rhs     mi mi.scaled    epc sepc.lv
## 92     Total.Animals2 ~~   Prop.Threat.Sp2 34.979     8.966  0.134   0.134
## 93     Total.Animals2 ~~ Brillouin.Index2  5.613     1.439 -0.066  -0.066
## 96       Sp.Richness2 ~~ Brillouin.Index2 11.965     3.067  0.034   0.034
## 97       Sp.Richness2 ~~ Mean.Raup.Crick2 25.675     6.581 -0.082  -0.082
## 99     Prop.Threat.Sp2 ~~ Mean.Raup.Crick2 22.929     5.877  0.764   0.764
## 101    Total.Animals2  ~       Attendance2  3.216     0.824  0.076   0.076
## 102    Total.Animals2  ~   Prop.Threat.Sp2 28.764     7.373  0.131   0.131
## 103    Total.Animals2  ~ Brillouin.Index2  5.613     1.439 -0.264  -0.264
## 104    Total.Animals2  ~ Mean.Raup.Crick2  5.050     1.294 -0.095  -0.095
## 108    Total.Animals2  ~        X50km_Pop2 36.649     9.394  0.140   0.140
## 109    Total.Animals2  ~        X10km_Pop2  7.814     2.003  0.067   0.067
```

```
## 110     Total.Animals2  ~  Nat_Pop._WB20152 10.485     2.687  0.132   0.132
## 114       Sp.Richness2  ~  Brillouin.Index2  5.706     1.463  0.078   0.078
## 115       Sp.Richness2  ~  Mean.Raup.Crick2 22.733     5.827 -0.105  -0.105
## 120    Prop.Threat.Sp2  ~      Attendance2 52.511    13.460  0.420   0.420
## 121    Prop.Threat.Sp2  ~    Total.Animals2 27.892     7.149  0.272   0.272
## 122    Prop.Threat.Sp2  ~      Sp.Richness2 14.069     3.606  0.283   0.283
## 123    Prop.Threat.Sp2  ~  Mean.Raup.Crick2 10.329     2.647  0.298   0.298
## 125    Prop.Threat.Sp2  ~  Mam.Sp.Richness2 12.514     3.207  0.209   0.209
## 126    Prop.Threat.Sp2  ~ Mean.Sp.BodyMass2 13.170     3.376  0.205   0.205
## 127    Prop.Threat.Sp2  ~        X50km_Pop2 26.636     6.827  0.232   0.232
## 128    Prop.Threat.Sp2  ~        X10km_Pop2 30.556     7.832  0.256   0.256
## 130    Prop.Threat.Sp2  ~  Nat_Pop._WB20152  9.739     2.496  0.141   0.141
## 134  Brillouin.Index2  ~       Zoo.Area.ha2  5.613     1.439  0.062   0.062
## 136  Brillouin.Index2  ~       Prop.Mam.Sp2  7.555     1.936  0.076   0.076
## 139  Brillouin.Index2  ~        X10km_Pop2  5.610     1.438 -0.059  -0.059
## 141  Mean.Raup.Crick2  ~       Attendance2 27.466     7.040  0.468   0.468
## 143  Mean.Raup.Crick2  ~       Zoo.Area.ha2  6.928     1.776  0.132   0.132
## 145  Mean.Raup.Crick2  ~       Prop.Mam.Sp2 22.676     5.812 -0.284  -0.284
## 146  Mean.Raup.Crick2  ~        X50km_Pop2 19.180     4.916  0.174   0.174
## 147  Mean.Raup.Crick2  ~        X10km_Pop2 25.871     6.631  0.211   0.211
## 149  Mean.Raup.Crick2  ~  Nat_Pop._WB20152 22.005     5.640  0.184   0.184
## 150       Zoo.Area.ha2  ~       Attendance2  4.440     1.138  0.393   0.393
## 152       Zoo.Area.ha2  ~       Sp.Richness2 12.211     3.130  1.093   1.093
## 154       Zoo.Area.ha2  ~  Brillouin.Index2  5.303     1.359  0.161   0.161
## 155       Zoo.Area.ha2  ~  Mean.Raup.Crick2  6.183     1.585  0.110   0.110
## 163  Mam.Sp.Richness2  ~       Attendance2  6.094     1.562 -0.461  -0.461
## 164  Mam.Sp.Richness2  ~    Total.Animals2  5.978     1.532 -0.246  -0.246
## 165  Mam.Sp.Richness2  ~      Sp.Richness2 14.531     3.724 -0.995  -0.995
## 166  Mam.Sp.Richness2  ~   Prop.Threat.Sp2  3.025     0.775  0.088   0.088
## 168  Mam.Sp.Richness2  ~  Mean.Raup.Crick2  4.580     1.174 -0.123  -0.123
## 181       Prop.Mam.Sp2  ~  Mean.Raup.Crick2  5.383     1.380 -0.101  -0.101
## 192 Mean.Sp.BodyMass2  ~   Prop.Threat.Sp2  3.556     0.911  0.061   0.061
## 202         X50km_Pop2  ~       Attendance2 30.164     7.732  0.582   0.582
## 203         X50km_Pop2  ~    Total.Animals2 29.487     7.558  0.282   0.282
## 207         X50km_Pop2  ~  Mean.Raup.Crick2  3.587     0.919  0.063   0.063
## 216         X10km_Pop2  ~    Total.Animals2  3.042     0.780 -0.083  -0.083
## 229      GDP.Millions2  ~    Total.Animals2  4.930     1.264  0.102   0.102
## 230      GDP.Millions2  ~      Sp.Richness2 30.427     7.799  0.339   0.339
## 232      GDP.Millions2  ~  Brillouin.Index2 18.601     4.768  0.173   0.173
## 233      GDP.Millions2  ~  Mean.Raup.Crick2 10.096     2.588 -0.087  -0.087
## 242  Nat_Pop._WB20152  ~    Total.Animals2  3.946     1.011 -0.079  -0.079
## 243  Nat_Pop._WB20152  ~      Sp.Richness2 31.649     8.112 -0.334  -0.334
## 244  Nat_Pop._WB20152  ~   Prop.Threat.Sp2  3.997     1.024  0.049   0.049
## 245  Nat_Pop._WB20152  ~  Brillouin.Index2 16.104     4.128 -0.139  -0.139
## 246  Nat_Pop._WB20152  ~  Mean.Raup.Crick2 15.367     3.939  0.101   0.101
##     sepc.all sepc.nox
## 92     0.134    0.134
## 93    -0.066   -0.066
## 96     0.034    0.034
## 97    -0.082   -0.082
## 99     0.765    0.765
## 101    0.074    0.074
## 102    0.130    0.130
## 103   -0.264   -0.264
```

```
## 104   -0.095   -0.095
## 108    0.140    0.140
## 109    0.067    0.067
## 110    0.132    0.132
## 114    0.079    0.079
## 115   -0.105   -0.105
## 120    0.411    0.411
## 121    0.273    0.273
## 122    0.283    0.283
## 123    0.299    0.299
## 125    0.209    0.209
## 126    0.205    0.205
## 127    0.232    0.232
## 128    0.256    0.256
## 130    0.141    0.142
## 134    0.062    0.062
## 136    0.076    0.076
## 139   -0.059   -0.059
## 141    0.457    0.457
## 143    0.132    0.132
## 145   -0.283   -0.283
## 146    0.174    0.174
## 147    0.211    0.211
## 149    0.184    0.184
## 150    0.384    0.384
## 152    1.093    1.093
## 154    0.161    0.161
## 155    0.110    0.110
## 163   -0.451   -0.451
## 164   -0.247   -0.247
## 165   -0.995   -0.995
## 166    0.088    0.088
## 168   -0.123   -0.123
## 181   -0.101   -0.101
## 192    0.061    0.061
## 202    0.569    0.569
## 203    0.282    0.282
## 207    0.064    0.064
## 216   -0.083   -0.083
## 229    0.102    0.102
## 230    0.339    0.339
## 232    0.173    0.173
## 233   -0.087   -0.087
## 242   -0.080   -0.080
## 243   -0.334   -0.334
## 244    0.049    0.049
## 245   -0.139   -0.139
## 246    0.101    0.101
```

Model Comparisons 1

At this stage we compare the eight models generated using AICc values. Overall model selection from the pool of competing models is achieved using AICc values, with a threshold of more than 2 AICc units lower

than nearest competing model being considered sufficient for model selection.

```
# Model Comparisons using AICc

# Comparing models with and without adjustment for nested nature of data
# library(AICcmodavg)
# source("lavaan.modavg.R")

aictab.lavaan(list(mod.1.fit, mod.2.fit, mod.3.fit, mod.4.fit, mod.5.fit, mod.6.fit, mod.7.fit,
                   mod.8.fit, fit.adj1, fit.adj2, fit.adj3, fit.adj4, fit.adj5, fit.adj6, fit.adj7, fit
              c("mod.1", "mod.2", "mod.3", "mod.4", "mod.5", "mod.6", "mod.7", "mod.8",
                "mod.1.nested", "mod.2.nested", "mod.3.nested", "mod.4.nested", "mod.5.nested", "mod.6.
```

```
##
## Model selection based on AICc:
##
##               K     AICc Delta_AICc AICcWt Cum.Wt       LL
## mod.8        71 13721.39       0.00      1      1 -6787.66
## mod.6        69 13734.55      13.17      0      1 -6796.52
## mod.7        70 13735.59      14.20      0      1 -6795.90
## mod.8.nested 85 13749.39      28.00      0      1 -6787.66
## mod.6.nested 83 13762.55      41.17      0      1 -6796.52
## mod.5        68 13763.29      41.91      0      1 -6812.02
## mod.7.nested 84 13763.59      42.20      0      1 -6795.90
## mod.5.nested 82 13791.29      69.91      0      1 -6812.02
## mod.4        67 13807.38      85.99      0      1 -6835.18
## mod.4.nested 81 13835.38     113.99      0      1 -6835.18
## mod.3        66 13872.33     150.94      0      1 -6868.78
## mod.3.nested 80 13900.33     178.94      0      1 -6868.78
## mod.2        65 13999.63     278.24      0      1 -6933.54
## mod.2.nested 79 14027.63     306.24      0      1 -6933.54
## mod.1        64 14656.32     934.93      0      1 -7263.00
## mod.1.nested 78 14684.32     962.93      0      1 -7263.00
```

Baed on these results we can see that model 8 is the superior model (lowest AICc values), for both models with and without adjustment for nested nature of data.

At this stage no other addition is conceptually appealling. Now we use p-values to identify potentially unsupported pathways, with a threshold of 0.05. Highest p-values are considered first for removal.

Model 9

Based on the results generated from the nested eight model, the highest p-value relationships to be considered for removal are **Attendance2** ~ **Prop.Thret.Sp2** with a p-value of **0.765** and **Prop.Threat.Sp2** ~ **Brilloun.Indx2** with a p-value of **0.753**. Therefore we decide to remove these pathways (and subsequently Prop.Thret.Sp2 from the model). Once again, the model summary, fit indices and modification indices were all generated for the model, adjusting for the nested nature of data.

```
# Attendance SEM (Presence-Absence)

# Model 9
# Removal of Attendance2 ~ Prop.Thret.Sp2, p = 0.765
# And subsequently Prop.Thret.Sp2 pathways

mod.9 <- 'Attendance2 ~ Zoo.Area.ha2 + Sp.Richness2 + Total.Animals2
```

```
+ Mam.Sp.Richness2 + Prop.Mam.Sp2
+ Mean.Sp.BodyMass2 + Brillouin.Index2 + Mean.Raup.Crick2
+ X50km_Pop2 + X10km_Pop2 + GDP.Millions2 + Nat_Pop._WB20152

Total.Animals2 ~ Zoo.Area.ha2 + Sp.Richness2 + GDP.Millions2
Sp.Richness2 ~ Zoo.Area.ha2 + Prop.Mam.Sp2 + Mam.Sp.Richness2 + Mean.Sp.BodyMass2
Brillouin.Index2 ~ Sp.Richness2 + Total.Animals2 + GDP.Millions2
Mean.Raup.Crick2 ~ Sp.Richness2 + Total.Animals2 + Mean.Sp.BodyMass2'

# Fit model and generate model summary
mod.9.fit <- sem(mod.9, data = sem_attendance_data, fixed.x=FALSE)
summary(mod.9.fit, rsq = TRUE)
```

```
## lavaan (0.5-23.1097) converged normally after  60 iterations
##
##   Number of observations                           458
##
##   Estimator                                         ML
##   Minimum Function Test Statistic              202.658
##   Degrees of freedom                                25
##   P-value (Chi-square)                           0.000
##
## Parameter Estimates:
##
##   Information                                 Expected
##   Standard Errors                             Standard
##
## Regressions:
##                    Estimate  Std.Err  z-value  P(>|z|)
##   Attendance2 ~
##     Zoo.Area.ha2      0.085    0.033    2.547    0.011
##     Sp.Richness2     -0.254    0.098   -2.586    0.010
##     Total.Animals2    0.460    0.060    7.620    0.000
##     Mam.Sp.Rchnss2    0.133    0.055    2.403    0.016
##     Prop.Mam.Sp2     -0.063    0.048   -1.303    0.193
##     Men.Sp.BdyMss2    0.340    0.039    8.639    0.000
##     Brilloun.Indx2    0.078    0.048    1.619    0.105
##     Mean.Rap.Crck2    0.144    0.029    4.985    0.000
##     X50km_Pop2        0.079    0.038    2.099    0.036
##     X10km_Pop2        0.399    0.041    9.654    0.000
##     GDP.Millions2     0.259    0.049    5.298    0.000
##     Nt_Pp._WB20152   -0.121    0.049   -2.479    0.013
##   Total.Animals2 ~
##     Zoo.Area.ha2      0.219    0.023    9.495    0.000
##     Sp.Richness2      0.805    0.023   34.969    0.000
##     GDP.Millions2    -0.129    0.023   -5.669    0.000
##   Sp.Richness2 ~
##     Zoo.Area.ha2      0.076    0.022    3.408    0.001
##     Prop.Mam.Sp2     -0.567    0.023  -24.670    0.000
##     Mam.Sp.Rchnss2    0.759    0.020   38.026    0.000
##     Men.Sp.BdyMss2   -0.029    0.026   -1.111    0.267
##   Brillouin.Index2 ~
##     Sp.Richness2      1.320    0.044   30.015    0.000
##     Total.Animals2   -0.620    0.044  -14.052    0.000
```

```
##     GDP.Millions2      -0.206    0.024   -8.518    0.000
##   Mean.Raup.Crick2 ~
##     Sp.Richness2       -0.355    0.074   -4.780    0.000
##     Total.Animals2      0.558    0.074    7.563    0.000
##     Men.Sp.BdyMss2     -0.495    0.041  -12.192    0.000
##
## Covariances:
##                       Estimate  Std.Err  z-value  P(>|z|)
##   Zoo.Area.ha2 ~~
##     Mam.Sp.Rchnss2        0.381    0.050    7.634    0.000
##     Prop.Mam.Sp2          0.312    0.049    6.389    0.000
##     Men.Sp.BdyMss2        0.534    0.053   10.096    0.000
##     X50km_Pop2            0.060    0.047    1.291    0.197
##     X10km_Pop2           -0.010    0.047   -0.208    0.835
##     GDP.Millions2        -0.027    0.047   -0.588    0.557
##     Nt_Pp._WB20152        0.061    0.047    1.310    0.190
##   Mam.Sp.Richness2 ~~
##     Prop.Mam.Sp2          0.088    0.047    1.882    0.060
##     Men.Sp.BdyMss2        0.303    0.049    6.216    0.000
##     X50km_Pop2            0.204    0.048    4.281    0.000
##     X10km_Pop2            0.284    0.048    5.852    0.000
##     GDP.Millions2        -0.060    0.047   -1.277    0.202
##     Nt_Pp._WB20152       -0.088    0.047   -1.881    0.060
##   Prop.Mam.Sp2 ~~
##     Men.Sp.BdyMss2        0.604    0.055   11.083    0.000
##     X50km_Pop2           -0.096    0.047   -2.054    0.040
##     X10km_Pop2           -0.204    0.048   -4.293    0.000
##     GDP.Millions2        -0.123    0.047   -2.615    0.009
##     Nt_Pp._WB20152       -0.115    0.047   -2.447    0.014
##   Mean.Sp.BodyMass2 ~~
##     X50km_Pop2            0.101    0.047    2.166    0.030
##     X10km_Pop2            0.126    0.047    2.683    0.007
##     GDP.Millions2        -0.082    0.047   -1.748    0.080
##     Nt_Pp._WB20152        0.006    0.047    0.124    0.901
##   X50km_Pop2 ~~
##     X10km_Pop2            0.752    0.058   12.886    0.000
##     GDP.Millions2        -0.013    0.047   -0.281    0.779
##     Nt_Pp._WB20152        0.191    0.047    4.033    0.000
##   X10km_Pop2 ~~
##     GDP.Millions2        -0.028    0.047   -0.604    0.546
##     Nt_Pp._WB20152        0.173    0.047    3.646    0.000
##   GDP.Millions2 ~~
##     Nt_Pp._WB20152        0.826    0.061   13.642    0.000
##
## Variances:
##                   Estimate  Std.Err  z-value  P(>|z|)
##    .Attendance2       0.267    0.018   15.133    0.000
##    .Total.Animals2    0.235    0.016   15.133    0.000
##    .Sp.Richness2      0.150    0.010   15.133    0.000
##    .Brilloun.Indx2    0.251    0.017   15.133    0.000
##    .Mean.Rap.Crck2    0.704    0.047   15.133    0.000
##     Zoo.Area.ha2      0.998    0.066   15.133    0.000
##     Mam.Sp.Rchnss2    0.998    0.066   15.133    0.000
##     Prop.Mam.Sp2      0.998    0.066   15.133    0.000
```

```
##     Men.Sp.BdyMss2    0.998    0.066   15.133    0.000
##     X50km_Pop2        0.998    0.066   15.133    0.000
##     X10km_Pop2        0.998    0.066   15.133    0.000
##     GDP.Millions2     0.998    0.066   15.133    0.000
##     Nt_Pp._WB20152    0.998    0.066   15.133    0.000
##
## R-Square:
##                    Estimate
##     Attendance2       0.718
##     Total.Animals2    0.766
##     Sp.Richness2      0.849
##     Brilloun.Indx2    0.750
##     Mean.Rap.Crck2    0.292
```

```
# Generate fit indices
fitMeasures(mod.9.fit, c("agfi", "rmr", "srmr", "rmsea", "cfi", "nnfi", "tli"))
```

```
##  agfi   rmr  srmr rmsea   cfi  nnfi   tli
## 0.778 0.050 0.050 0.125 0.942 0.885 0.885
```

```
# Generate modification indices
mi9 <- modindices(mod.9.fit)
print(mi9[mi9$mi > 3.0,])
```

```
##                  lhs op              rhs     mi    epc sepc.lv sepc.all
## 72    Total.Animals2 ~~ Brillouin.Index2  5.613 -0.066  -0.066   -0.066
## 73    Total.Animals2 ~~ Mean.Raup.Crick2  4.273 -0.096  -0.096   -0.096
## 74      Sp.Richness2 ~~ Brillouin.Index2 11.965  0.034   0.034    0.034
## 75      Sp.Richness2 ~~ Mean.Raup.Crick2 26.814 -0.086  -0.086   -0.086
## 77    Total.Animals2  ~      Attendance2  3.615  0.081   0.081    0.079
## 78    Total.Animals2  ~ Brillouin.Index2  5.613 -0.264  -0.264   -0.264
## 83    Total.Animals2  ~       X50km_Pop2 36.649  0.140   0.140    0.140
## 84    Total.Animals2  ~       X10km_Pop2  7.814  0.067   0.067    0.067
## 85    Total.Animals2  ~ Nat_Pop._WB20152 10.485  0.132   0.132    0.132
## 88      Sp.Richness2  ~ Brillouin.Index2  5.707  0.078   0.078    0.079
## 89      Sp.Richness2  ~ Mean.Raup.Crick2 24.113 -0.109  -0.109   -0.109
## 96  Brillouin.Index2  ~     Zoo.Area.ha2  5.613  0.062   0.062    0.062
## 98  Brillouin.Index2  ~     Prop.Mam.Sp2  7.555  0.076   0.076    0.076
## 101 Brillouin.Index2  ~       X10km_Pop2  5.610 -0.059  -0.059   -0.059
## 103 Mean.Raup.Crick2  ~      Attendance2 20.482  0.410   0.410    0.401
## 105 Mean.Raup.Crick2  ~     Zoo.Area.ha2  9.180  0.155   0.155    0.155
## 107 Mean.Raup.Crick2  ~     Prop.Mam.Sp2 28.295 -0.314  -0.314   -0.315
## 108 Mean.Raup.Crick2  ~       X50km_Pop2 14.164  0.152   0.152    0.153
## 109 Mean.Raup.Crick2  ~       X10km_Pop2 19.507  0.187   0.187    0.187
## 111 Mean.Raup.Crick2  ~ Nat_Pop._WB20152 17.086  0.165   0.165    0.165
## 112     Zoo.Area.ha2  ~      Attendance2  5.395  0.436   0.436    0.426
## 114     Zoo.Area.ha2  ~     Sp.Richness2 12.212  1.093   1.093    1.093
## 115     Zoo.Area.ha2  ~ Brillouin.Index2  5.303  0.161   0.161    0.161
## 116     Zoo.Area.ha2  ~ Mean.Raup.Crick2  7.714  0.124   0.124    0.123
## 124 Mam.Sp.Richness2  ~      Attendance2  6.891 -0.494  -0.494   -0.482
## 125 Mam.Sp.Richness2  ~   Total.Animals2  5.978 -0.246  -0.246   -0.247
## 126 Mam.Sp.Richness2  ~     Sp.Richness2 14.531 -0.995  -0.995   -0.995
## 128 Mam.Sp.Richness2  ~ Mean.Raup.Crick2  5.425 -0.135  -0.135   -0.135
## 136     Prop.Mam.Sp2  ~      Attendance2  3.190 -0.248  -0.248   -0.242
## 140     Prop.Mam.Sp2  ~ Mean.Raup.Crick2 10.882 -0.141  -0.141   -0.141
## 160       X50km_Pop2  ~      Attendance2 30.582  0.591   0.591    0.576
```

```
## 161       X50km_Pop2  ~   Total.Animals2 29.487  0.282   0.282    0.282
## 164       X50km_Pop2  ~ Mean.Raup.Crick2  3.672  0.065   0.065    0.065
## 172       X10km_Pop2  ~      Attendance2  3.167 -0.174  -0.174   -0.170
## 173       X10km_Pop2  ~   Total.Animals2  3.042 -0.083  -0.083   -0.083
## 185    GDP.Millions2  ~   Total.Animals2  4.930  0.102   0.102    0.102
## 186    GDP.Millions2  ~     Sp.Richness2 30.428  0.339   0.339    0.339
## 187    GDP.Millions2  ~ Brillouin.Index2 18.601  0.173   0.173    0.173
## 188    GDP.Millions2  ~ Mean.Raup.Crick2 10.852 -0.091  -0.091   -0.091
## 197 Nat_Pop._WB20152  ~   Total.Animals2  3.946 -0.079  -0.079   -0.080
## 198 Nat_Pop._WB20152  ~     Sp.Richness2 31.650 -0.334  -0.334   -0.334
## 199 Nat_Pop._WB20152  ~ Brillouin.Index2 16.104 -0.139  -0.139   -0.139
## 200 Nat_Pop._WB20152  ~ Mean.Raup.Crick2 15.732  0.104   0.104    0.103
##     sepc.nox
## 72    -0.066
## 73    -0.096
## 74     0.034
## 75    -0.086
## 77     0.079
## 78    -0.264
## 83     0.140
## 84     0.067
## 85     0.132
## 88     0.079
## 89    -0.109
## 96     0.062
## 98     0.076
## 101   -0.059
## 103    0.401
## 105    0.155
## 107   -0.315
## 108    0.153
## 109    0.187
## 111    0.165
## 112    0.426
## 114    1.093
## 115    0.161
## 116    0.123
## 124   -0.482
## 125   -0.247
## 126   -0.995
## 128   -0.135
## 136   -0.242
## 140   -0.141
## 160    0.576
## 161    0.282
## 164    0.065
## 172   -0.170
## 173   -0.083
## 185    0.102
## 186    0.339
## 187    0.173
## 188   -0.091
## 197   -0.080
## 198   -0.334
```

```
## 199   -0.139
## 200    0.103
```

```r
# Adjust for the nested nature of the data (institutions within countries)
# Fit model and generate model summary
design <- svydesign(ids = ~Country, nest=TRUE, data=sem_attendance_data)
fit.adj9 <- lavaan.survey(lavaan.fit = mod.9.fit, survey.design = design)
summary(fit.adj9, rsq = TRUE)
```

```
## lavaan (0.5-23.1097) converged normally after  53 iterations
##
##   Number of observations                           458
##
##   Estimator                                         ML      Robust
##   Minimum Function Test Statistic              202.658      49.545
##   Degrees of freedom                                25          25
##   P-value (Chi-square)                           0.000       0.002
##   Scaling correction factor                                  4.090
##     for the Satorra-Bentler correction
##
## Parameter Estimates:
##
##   Information                                 Expected
##   Standard Errors                           Robust.sem
##
## Regressions:
##                        Estimate  Std.Err  z-value  P(>|z|)
##   Attendance2 ~
##     Zoo.Area.ha2          0.085    0.041    2.074    0.038
##     Sp.Richness2         -0.254    0.106   -2.398    0.016
##     Total.Animals2        0.460    0.067    6.891    0.000
##     Mam.Sp.Rchnss2        0.133    0.049    2.718    0.007
##     Prop.Mam.Sp2         -0.063    0.043   -1.451    0.147
##     Men.Sp.BdyMss2        0.340    0.030   11.330    0.000
##     Brilloun.Indx2        0.078    0.066    1.189    0.235
##     Mean.Rap.Crck2        0.144    0.029    4.963    0.000
##     X50km_Pop2            0.079    0.033    2.403    0.016
##     X10km_Pop2            0.399    0.042    9.438    0.000
##     GDP.Millions2         0.259    0.061    4.240    0.000
##     Nt_Pp._WB20152       -0.121    0.067   -1.811    0.070
##   Total.Animals2 ~
##     Zoo.Area.ha2          0.219    0.036    5.992    0.000
##     Sp.Richness2          0.805    0.051   15.916    0.000
##     GDP.Millions2        -0.129    0.072   -1.785    0.074
##   Sp.Richness2 ~
##     Zoo.Area.ha2          0.076    0.037    2.083    0.037
##     Prop.Mam.Sp2         -0.567    0.048  -11.722    0.000
##     Mam.Sp.Rchnss2        0.759    0.072   10.553    0.000
##     Men.Sp.BdyMss2       -0.029    0.050   -0.576    0.564
##   Brillouin.Index2 ~
##     Sp.Richness2          1.320    0.089   14.752    0.000
##     Total.Animals2       -0.620    0.127   -4.883    0.000
##     GDP.Millions2        -0.206    0.072   -2.846    0.004
##   Mean.Raup.Crick2 ~
##     Sp.Richness2         -0.355    0.231   -1.540    0.124
```

```
##     Total.Animals2      0.558    0.155    3.610    0.000
##     Men.Sp.BdyMss2     -0.495    0.052   -9.568    0.000
##
## Covariances:
##                        Estimate  Std.Err  z-value  P(>|z|)
##   Zoo.Area.ha2 ~~
##     Mam.Sp.Rchnss2        0.381    0.064    5.985    0.000
##     Prop.Mam.Sp2          0.312    0.089    3.510    0.000
##     Men.Sp.BdyMss2        0.534    0.104    5.147    0.000
##     X50km_Pop2            0.060    0.069    0.880    0.379
##     X10km_Pop2           -0.010    0.078   -0.124    0.901
##     GDP.Millions2        -0.027    0.041   -0.673    0.501
##     Nt_Pp._WB20152        0.061    0.090    0.682    0.495
##   Mam.Sp.Richness2 ~~
##     Prop.Mam.Sp2          0.088    0.061    1.435    0.151
##     Men.Sp.BdyMss2        0.303    0.081    3.730    0.000
##     X50km_Pop2            0.204    0.082    2.487    0.013
##     X10km_Pop2            0.284    0.066    4.278    0.000
##     GDP.Millions2        -0.060    0.064   -0.937    0.349
##     Nt_Pp._WB20152       -0.088    0.102   -0.863    0.388
##   Prop.Mam.Sp2 ~~
##     Men.Sp.BdyMss2        0.604    0.130    4.656    0.000
##     X50km_Pop2           -0.096    0.051   -1.888    0.059
##     X10km_Pop2           -0.204    0.055   -3.685    0.000
##     GDP.Millions2        -0.123    0.105   -1.172    0.241
##     Nt_Pp._WB20152       -0.115    0.091   -1.257    0.209
##   Mean.Sp.BodyMass2 ~~
##     X50km_Pop2            0.101    0.072    1.400    0.161
##     X10km_Pop2            0.126    0.067    1.895    0.058
##     GDP.Millions2        -0.082    0.046   -1.759    0.079
##     Nt_Pp._WB20152        0.006    0.092    0.063    0.950
##   X50km_Pop2 ~~
##     X10km_Pop2            0.752    0.126    5.982    0.000
##     GDP.Millions2        -0.013    0.100   -0.131    0.896
##     Nt_Pp._WB20152        0.191    0.177    1.083    0.279
##   X10km_Pop2 ~~
##     GDP.Millions2        -0.028    0.056   -0.505    0.613
##     Nt_Pp._WB20152        0.173    0.101    1.701    0.089
##   GDP.Millions2 ~~
##     Nt_Pp._WB20152        0.826    0.202    4.089    0.000
##
## Intercepts:
##                    Estimate  Std.Err  z-value  P(>|z|)
##    .Attendance2      -0.000    0.030   -0.000    1.000
##    .Total.Animals2   -0.000    0.070   -0.000    1.000
##    .Sp.Richness2      0.000    0.034    0.000    1.000
##    .Brilloun.Indx2   -0.000    0.055   -0.000    1.000
##    .Mean.Rap.Crck2    0.000    0.094    0.000    1.000
##     Zoo.Area.ha2      0.000    0.064    0.000    1.000
##     Mam.Sp.Rchnss2   -0.000    0.079   -0.000    1.000
##     Prop.Mam.Sp2      0.000    0.118    0.000    1.000
##     Men.Sp.BdyMss2   -0.000    0.080   -0.000    1.000
##     X50km_Pop2       -0.000    0.114   -0.000    1.000
##     X10km_Pop2        0.000    0.105    0.000    1.000
```

```
##     GDP.Millions2    -0.000    0.345   -0.000    1.000
##     Nt_Pp._WB20152    0.000    0.295    0.000    1.000
##
## Variances:
##                    Estimate  Std.Err  z-value  P(>|z|)
##    .Attendance2       0.267    0.028    9.565    0.000
##    .Total.Animals2    0.235    0.038    6.139    0.000
##    .Sp.Richness2      0.150    0.045    3.344    0.001
##    .Brilloun.Indx2    0.251    0.053    4.691    0.000
##    .Mean.Rap.Crck2    0.704    0.063   11.098    0.000
##     Zoo.Area.ha2      0.998    0.112    8.886    0.000
##     Mam.Sp.Rchnss2    0.998    0.115    8.669    0.000
##     Prop.Mam.Sp2      0.998    0.150    6.662    0.000
##     Men.Sp.BdyMss2    0.998    0.173    5.783    0.000
##     X50km_Pop2        0.998    0.131    7.623    0.000
##     X10km_Pop2        0.998    0.174    5.745    0.000
##     GDP.Millions2     0.998    0.246    4.061    0.000
##     Nt_Pp._WB20152    0.998    0.225    4.439    0.000
##
## R-Square:
##                    Estimate
##     Attendance2       0.718
##     Total.Animals2    0.766
##     Sp.Richness2      0.849
##     Brilloun.Indx2    0.750
##     Mean.Rap.Crck2    0.292
```

```
# Generate fit indices
fitMeasures(fit.adj9, c("agfi", "rmr", "srmr", "rmsea", "cfi", "nnfi", "tli"))
```

```
##  agfi   rmr  srmr rmsea   cfi  nnfi   tli
## 0.746 0.050 0.047 0.125 0.942 0.885 0.885
```

```
# Generate modification indices
mi9adj <- modindices(fit.adj9)
print(mi9adj[mi9adj$mi > 3.0,])
```

```
##                   lhs op               rhs     mi mi.scaled    epc sepc.lv
## 85    Total.Animals2 ~~ Brillouin.Index2  5.613     1.372 -0.066  -0.066
## 86    Total.Animals2 ~~ Mean.Raup.Crick2  4.273     1.045 -0.096  -0.096
## 87      Sp.Richness2 ~~ Brillouin.Index2 11.965     2.925  0.034   0.034
## 88      Sp.Richness2 ~~ Mean.Raup.Crick2 26.814     6.555 -0.086  -0.086
## 90    Total.Animals2  ~      Attendance2  3.615     0.884  0.081   0.081
## 91    Total.Animals2  ~ Brillouin.Index2  5.613     1.372 -0.264  -0.264
## 96    Total.Animals2  ~       X50km_Pop2 36.649     8.960  0.140   0.140
## 97    Total.Animals2  ~       X10km_Pop2  7.814     1.910  0.067   0.067
## 98    Total.Animals2  ~ Nat_Pop._WB20152 10.485     2.563  0.132   0.132
## 101     Sp.Richness2  ~ Brillouin.Index2  5.706     1.395  0.078   0.078
## 102     Sp.Richness2  ~ Mean.Raup.Crick2 24.113     5.895 -0.109  -0.109
## 109 Brillouin.Index2  ~     Zoo.Area.ha2  5.613     1.372  0.062   0.062
## 111 Brillouin.Index2  ~     Prop.Mam.Sp2  7.555     1.847  0.076   0.076
## 114 Brillouin.Index2  ~       X10km_Pop2  5.610     1.371 -0.059  -0.059
## 116 Mean.Raup.Crick2  ~      Attendance2 20.482     5.007  0.410   0.410
## 118 Mean.Raup.Crick2  ~     Zoo.Area.ha2  9.180     2.244  0.155   0.155
## 120 Mean.Raup.Crick2  ~     Prop.Mam.Sp2 28.295     6.917 -0.314  -0.314
## 121 Mean.Raup.Crick2  ~       X50km_Pop2 14.164     3.463  0.152   0.152
```

```
## 122 Mean.Raup.Crick2  ~       X10km_Pop2 19.507     4.769  0.187   0.187
## 124 Mean.Raup.Crick2  ~ Nat_Pop._WB20152 17.086     4.177  0.165   0.165
## 125     Zoo.Area.ha2  ~      Attendance2  5.395     1.319  0.436   0.436
## 127     Zoo.Area.ha2  ~     Sp.Richness2 12.212     2.985  1.093   1.093
## 128     Zoo.Area.ha2  ~ Brillouin.Index2  5.303     1.296  0.161   0.161
## 129     Zoo.Area.ha2  ~ Mean.Raup.Crick2  7.714     1.886  0.124   0.124
## 137 Mam.Sp.Richness2  ~      Attendance2  6.892     1.685 -0.494  -0.494
## 138 Mam.Sp.Richness2  ~   Total.Animals2  5.978     1.461 -0.246  -0.246
## 139 Mam.Sp.Richness2  ~     Sp.Richness2 14.531     3.552 -0.995  -0.995
## 141 Mam.Sp.Richness2  ~ Mean.Raup.Crick2  5.425     1.326 -0.135  -0.135
## 149     Prop.Mam.Sp2  ~      Attendance2  3.190     0.780 -0.248  -0.248
## 153     Prop.Mam.Sp2  ~ Mean.Raup.Crick2 10.881     2.660 -0.141  -0.141
## 173       X50km_Pop2  ~      Attendance2 30.582     7.477  0.591   0.591
## 174       X50km_Pop2  ~   Total.Animals2 29.487     7.209  0.282   0.282
## 177       X50km_Pop2  ~ Mean.Raup.Crick2  3.672     0.898  0.065   0.065
## 185       X10km_Pop2  ~      Attendance2  3.167     0.774 -0.174  -0.174
## 186       X10km_Pop2  ~   Total.Animals2  3.042     0.744 -0.083  -0.083
## 198    GDP.Millions2  ~   Total.Animals2  4.930     1.205  0.102   0.102
## 199    GDP.Millions2  ~     Sp.Richness2 30.428     7.439  0.339   0.339
## 200    GDP.Millions2  ~ Brillouin.Index2 18.601     4.548  0.173   0.173
## 201    GDP.Millions2  ~ Mean.Raup.Crick2 10.852     2.653 -0.091  -0.091
## 210 Nat_Pop._WB20152  ~   Total.Animals2  3.946     0.965 -0.079  -0.079
## 211 Nat_Pop._WB20152  ~     Sp.Richness2 31.650     7.738 -0.334  -0.334
## 212 Nat_Pop._WB20152  ~ Brillouin.Index2 16.104     3.937 -0.139  -0.139
## 213 Nat_Pop._WB20152  ~ Mean.Raup.Crick2 15.733     3.846  0.104   0.104
##     sepc.all sepc.nox
## 85    -0.066   -0.066
## 86    -0.096   -0.096
## 87     0.034    0.034
## 88    -0.086   -0.086
## 90     0.079    0.079
## 91    -0.264   -0.264
## 96     0.140    0.140
## 97     0.067    0.067
## 98     0.132    0.132
## 101    0.079    0.079
## 102   -0.109   -0.109
## 109    0.062    0.062
## 111    0.076    0.076
## 114   -0.059   -0.059
## 116    0.401    0.401
## 118    0.155    0.155
## 120   -0.315   -0.315
## 121    0.153    0.153
## 122    0.187    0.187
## 124    0.165    0.165
## 125    0.426    0.426
## 127    1.093    1.093
## 128    0.161    0.161
## 129    0.123    0.123
## 137   -0.482   -0.482
## 138   -0.247   -0.247
## 139   -0.995   -0.995
## 141   -0.135   -0.135
```

```
## 149   -0.242   -0.242
## 153   -0.141   -0.141
## 173    0.576    0.576
## 174    0.282    0.282
## 177    0.065    0.065
## 185   -0.170   -0.170
## 186   -0.083   -0.083
## 198    0.102    0.102
## 199    0.339    0.339
## 200    0.173    0.173
## 201   -0.091   -0.091
## 210   -0.080   -0.080
## 211   -0.334   -0.334
## 212   -0.139   -0.139
## 213    0.103    0.103
```

Model 10

Based on the results generated from the nested ninth model, the highest p-value relationship to be considered for removal is **Sp.Richness2** ~ **Men.Sp.BdyMss2** with a p-value of **0.564**. Therefore we decide to remove this pathway. Once again, the model summary, fit indices and modification indices were all generated for the model, adjusting for the nested nature of data.

```r
# Attendance SEM (Presence-Absence)

# Model 10
# Removal of Sp.Richness2 ~ Men.Sp.BdyMss2, p = 0.564

mod.10 <- 'Attendance2 ~ Zoo.Area.ha2 + Sp.Richness2 + Total.Animals2
+ Mam.Sp.Richness2 + Prop.Mam.Sp2
+ Mean.Sp.BodyMass2 + Brillouin.Index2 + Mean.Raup.Crick2
+ X50km_Pop2 + X10km_Pop2 + GDP.Millions2 + Nat_Pop._WB20152

Total.Animals2 ~ Zoo.Area.ha2 + Sp.Richness2 + GDP.Millions2
Sp.Richness2 ~ Zoo.Area.ha2 + Prop.Mam.Sp2 + Mam.Sp.Richness2
Brillouin.Index2 ~ Sp.Richness2 + Total.Animals2 + GDP.Millions2
Mean.Raup.Crick2 ~ Sp.Richness2 + Total.Animals2 + Mean.Sp.BodyMass2'

# Fit model and generate model summary
mod.10.fit <- sem(mod.10, data = sem_attendance_data, fixed.x=FALSE)
summary(mod.10.fit, rsq = TRUE)
```

```
## lavaan (0.5-23.1097) converged normally after  64 iterations
##
##   Number of observations                           458
##
##   Estimator                                         ML
##   Minimum Function Test Statistic              203.890
##   Degrees of freedom                                26
##   P-value (Chi-square)                           0.000
##
## Parameter Estimates:
##
##   Information                                 Expected
```

```
##   Standard Errors                             Standard
##
## Regressions:
##                      Estimate  Std.Err  z-value  P(>|z|)
##   Attendance2 ~
##     Zoo.Area.ha2        0.085    0.033    2.553    0.011
##     Sp.Richness2       -0.254    0.098   -2.588    0.010
##     Total.Animals2      0.460    0.060    7.620    0.000
##     Mam.Sp.Rchnss2      0.133    0.055    2.415    0.016
##     Prop.Mam.Sp2       -0.063    0.049   -1.286    0.198
##     Men.Sp.BdyMss2      0.340    0.039    8.648    0.000
##     Brilloun.Indx2      0.078    0.048    1.619    0.105
##     Mean.Rap.Crck2      0.144    0.029    4.985    0.000
##     X50km_Pop2          0.079    0.038    2.099    0.036
##     X10km_Pop2          0.399    0.041    9.654    0.000
##     GDP.Millions2       0.259    0.049    5.298    0.000
##     Nt_Pp._WB20152     -0.121    0.049   -2.479    0.013
##   Total.Animals2 ~
##     Zoo.Area.ha2        0.219    0.023    9.495    0.000
##     Sp.Richness2        0.805    0.023   34.969    0.000
##     GDP.Millions2      -0.129    0.023   -5.669    0.000
##   Sp.Richness2 ~
##     Zoo.Area.ha2        0.067    0.021    3.225    0.001
##     Prop.Mam.Sp2       -0.581    0.019  -30.364    0.000
##     Mam.Sp.Rchnss2      0.755    0.020   38.373    0.000
##   Brillouin.Index2 ~
##     Sp.Richness2        1.320    0.044   30.015    0.000
##     Total.Animals2     -0.620    0.044  -14.052    0.000
##     GDP.Millions2      -0.206    0.024   -8.518    0.000
##   Mean.Raup.Crick2 ~
##     Sp.Richness2       -0.355    0.074   -4.791    0.000
##     Total.Animals2      0.558    0.074    7.566    0.000
##     Men.Sp.BdyMss2     -0.495    0.041  -12.212    0.000
##
## Covariances:
##                        Estimate  Std.Err  z-value  P(>|z|)
##   Zoo.Area.ha2 ~~
##     Mam.Sp.Rchnss2        0.381    0.050    7.634    0.000
##     Prop.Mam.Sp2          0.312    0.049    6.389    0.000
##     Men.Sp.BdyMss2        0.534    0.053   10.096    0.000
##     X50km_Pop2            0.060    0.047    1.291    0.197
##     X10km_Pop2           -0.010    0.047   -0.208    0.835
##     GDP.Millions2        -0.027    0.047   -0.588    0.557
##     Nt_Pp._WB20152        0.061    0.047    1.310    0.190
##   Mam.Sp.Richness2 ~~
##     Prop.Mam.Sp2          0.088    0.047    1.882    0.060
##     Men.Sp.BdyMss2        0.303    0.049    6.216    0.000
##     X50km_Pop2            0.204    0.048    4.281    0.000
##     X10km_Pop2            0.284    0.048    5.852    0.000
##     GDP.Millions2        -0.060    0.047   -1.277    0.202
##     Nt_Pp._WB20152       -0.088    0.047   -1.881    0.060
##   Prop.Mam.Sp2 ~~
##     Men.Sp.BdyMss2        0.604    0.055   11.083    0.000
##     X50km_Pop2           -0.096    0.047   -2.054    0.040
```

```
##     X10km_Pop2          -0.204    0.048   -4.293    0.000
##     GDP.Millions2       -0.123    0.047   -2.615    0.009
##     Nt_Pp._WB20152      -0.115    0.047   -2.447    0.014
##   Mean.Sp.BodyMass2 ~~
##     X50km_Pop2           0.101    0.047    2.166    0.030
##     X10km_Pop2           0.126    0.047    2.683    0.007
##     GDP.Millions2       -0.082    0.047   -1.748    0.080
##     Nt_Pp._WB20152       0.006    0.047    0.124    0.901
##   X50km_Pop2 ~~
##     X10km_Pop2           0.752    0.058   12.886    0.000
##     GDP.Millions2       -0.013    0.047   -0.281    0.779
##     Nt_Pp._WB20152       0.191    0.047    4.033    0.000
##   X10km_Pop2 ~~
##     GDP.Millions2       -0.028    0.047   -0.604    0.546
##     Nt_Pp._WB20152       0.173    0.047    3.646    0.000
##   GDP.Millions2 ~~
##     Nt_Pp._WB20152       0.826    0.061   13.642    0.000
##
## Variances:
##                    Estimate  Std.Err  z-value  P(>|z|)
##    .Attendance2       0.267    0.018   15.133    0.000
##    .Total.Animals2    0.235    0.016   15.133    0.000
##    .Sp.Richness2      0.151    0.010   15.133    0.000
##    .Brilloun.Indx2    0.251    0.017   15.133    0.000
##    .Mean.Rap.Crck2    0.704    0.047   15.133    0.000
##     Zoo.Area.ha2      0.998    0.066   15.133    0.000
##     Mam.Sp.Rchnss2    0.998    0.066   15.133    0.000
##     Prop.Mam.Sp2      0.998    0.066   15.133    0.000
##     Men.Sp.BdyMss2    0.998    0.066   15.133    0.000
##     X50km_Pop2        0.998    0.066   15.133    0.000
##     X10km_Pop2        0.998    0.066   15.133    0.000
##     GDP.Millions2     0.998    0.066   15.133    0.000
##     Nt_Pp._WB20152    0.998    0.066   15.133    0.000
##
## R-Square:
##                    Estimate
##     Attendance2       0.719
##     Total.Animals2    0.766
##     Sp.Richness2      0.849
##     Brilloun.Indx2    0.750
##     Mean.Rap.Crck2    0.291
```

```r
# Generate fit indices
fitMeasures(mod.10.fit, c("agfi", "rmr", "srmr", "rmsea", "cfi", "nnfi", "tli"))
```

```
##  agfi   rmr  srmr rmsea   cfi  nnfi   tli
## 0.784 0.050 0.050 0.122 0.942 0.889 0.889
```

```r
# Generate modification indices
mi10 <- modindices(mod.10.fit)
print(mi10[mi10$mi > 3.0,])
```

```
##                  lhs op               rhs     mi    epc sepc.lv sepc.all
## 71    Total.Animals2 ~~ Brillouin.Index2  5.613 -0.066  -0.066   -0.066
## 72    Total.Animals2 ~~ Mean.Raup.Crick2  4.260 -0.096  -0.096   -0.096
## 73      Sp.Richness2 ~~ Brillouin.Index2 12.470  0.035   0.035    0.035
```

```
## 74      Sp.Richness2 ~~ Mean.Raup.Crick2 27.601 -0.087  -0.087   -0.087
## 76    Total.Animals2  ~      Attendance2  3.651  0.082   0.082    0.079
## 77    Total.Animals2  ~ Brillouin.Index2  5.613 -0.264  -0.264   -0.264
## 82    Total.Animals2  ~       X50km_Pop2 36.696  0.141   0.141    0.140
## 83    Total.Animals2  ~       X10km_Pop2  7.848  0.068   0.068    0.067
## 84    Total.Animals2  ~ Nat_Pop._WB20152 10.481  0.132   0.132    0.132
## 87      Sp.Richness2  ~ Brillouin.Index2  6.033  0.081   0.081    0.081
## 88      Sp.Richness2  ~ Mean.Raup.Crick2 17.310 -0.085  -0.085   -0.085
## 93      Sp.Richness2  ~ Nat_Pop._WB20152  3.059 -0.032  -0.032   -0.032
## 96  Brillouin.Index2  ~     Zoo.Area.ha2  5.613  0.062   0.062    0.062
## 98  Brillouin.Index2  ~     Prop.Mam.Sp2  7.555  0.076   0.076    0.076
## 101 Brillouin.Index2  ~       X10km_Pop2  5.633 -0.059  -0.059   -0.059
## 103 Mean.Raup.Crick2  ~      Attendance2 20.515  0.411   0.411    0.402
## 105 Mean.Raup.Crick2  ~     Zoo.Area.ha2  9.137  0.154   0.154    0.155
## 107 Mean.Raup.Crick2  ~     Prop.Mam.Sp2 28.743 -0.319  -0.319   -0.320
## 108 Mean.Raup.Crick2  ~       X50km_Pop2 14.172  0.152   0.152    0.153
## 109 Mean.Raup.Crick2  ~       X10km_Pop2 19.556  0.187   0.187    0.187
## 111 Mean.Raup.Crick2  ~ Nat_Pop._WB20152 17.087  0.165   0.165    0.165
## 112     Zoo.Area.ha2  ~      Attendance2  6.046  0.458   0.458    0.447
## 114     Zoo.Area.ha2  ~     Sp.Richness2  9.363  0.602   0.602    0.602
## 115     Zoo.Area.ha2  ~ Brillouin.Index2  6.203  0.169   0.169    0.170
## 116     Zoo.Area.ha2  ~ Mean.Raup.Crick2  7.812  0.124   0.124    0.124
## 124 Mam.Sp.Richness2  ~      Attendance2  6.485 -0.477  -0.477   -0.465
## 125 Mam.Sp.Richness2  ~    Total.Animals2  5.172 -0.226  -0.226   -0.227
## 126 Mam.Sp.Richness2  ~     Sp.Richness2  9.521 -0.742  -0.742   -0.742
## 128 Mam.Sp.Richness2  ~ Mean.Raup.Crick2  5.331 -0.133  -0.133   -0.133
## 139     Prop.Mam.Sp2  ~ Brillouin.Index2  3.002  0.105   0.105    0.105
## 140     Prop.Mam.Sp2  ~ Mean.Raup.Crick2 10.813 -0.141  -0.141   -0.141
## 160       X50km_Pop2  ~      Attendance2 30.576  0.590   0.590    0.577
## 161       X50km_Pop2  ~    Total.Animals2 29.464  0.281   0.281    0.282
## 164       X50km_Pop2  ~ Mean.Raup.Crick2  3.672  0.065   0.065    0.065
## 172       X10km_Pop2  ~      Attendance2  3.167 -0.174  -0.174   -0.170
## 173       X10km_Pop2  ~    Total.Animals2  3.040 -0.083  -0.083   -0.083
## 185    GDP.Millions2  ~    Total.Animals2  4.925  0.102   0.102    0.102
## 186    GDP.Millions2  ~     Sp.Richness2 30.346  0.338   0.338    0.338
## 187    GDP.Millions2  ~ Brillouin.Index2 18.587  0.173   0.173    0.173
## 188    GDP.Millions2  ~ Mean.Raup.Crick2 10.852 -0.091  -0.091   -0.091
## 197 Nat_Pop._WB20152  ~    Total.Animals2  3.943 -0.079  -0.079   -0.080
## 198 Nat_Pop._WB20152  ~     Sp.Richness2 31.564 -0.333  -0.333   -0.333
## 199 Nat_Pop._WB20152  ~ Brillouin.Index2 16.094 -0.139  -0.139   -0.139
## 200 Nat_Pop._WB20152  ~ Mean.Raup.Crick2 15.732  0.104   0.104    0.103
##     sepc.nox
## 71    -0.066
## 72    -0.096
## 73     0.035
## 74    -0.087
## 76     0.079
## 77    -0.264
## 82     0.140
## 83     0.067
## 84     0.132
## 87     0.081
## 88    -0.085
## 93    -0.032
```

```
## 96     0.062
## 98     0.076
## 101   -0.059
## 103    0.402
## 105    0.155
## 107   -0.320
## 108    0.153
## 109    0.188
## 111    0.165
## 112    0.447
## 114    0.602
## 115    0.170
## 116    0.124
## 124   -0.465
## 125   -0.227
## 126   -0.742
## 128   -0.133
## 139    0.105
## 140   -0.141
## 160    0.577
## 161    0.282
## 164    0.065
## 172   -0.170
## 173   -0.083
## 185    0.102
## 186    0.338
## 187    0.173
## 188   -0.091
## 197   -0.080
## 198   -0.333
## 199   -0.139
## 200    0.103
```

```
# Adjust for the nested nature of the data (institutions within countries)
# Fit model and generate model summary
design <- svydesign(ids = ~Country, nest=TRUE, data=sem_attendance_data)
fit.adj10 <- lavaan.survey(lavaan.fit = mod.10.fit, survey.design = design)
summary(fit.adj10, rsq = TRUE)
```

```
## lavaan (0.5-23.1097) converged normally after  63 iterations
##
##   Number of observations                           458
##
##   Estimator                                         ML      Robust
##   Minimum Function Test Statistic              203.890      49.197
##   Degrees of freedom                                26          26
##   P-value (Chi-square)                           0.000       0.004
##   Scaling correction factor                                  4.144
##     for the Satorra-Bentler correction
##
## Parameter Estimates:
##
##   Information                                 Expected
##   Standard Errors                           Robust.sem
##
```

```
## Regressions:
##                    Estimate  Std.Err  z-value  P(>|z|)
##   Attendance2 ~
##     Zoo.Area.ha2       0.085    0.041    2.078    0.038
##     Sp.Richness2      -0.254    0.106   -2.406    0.016
##     Total.Animals2     0.460    0.067    6.892    0.000
##     Mam.Sp.Rchnss2     0.133    0.049    2.737    0.006
##     Prop.Mam.Sp2      -0.063    0.044   -1.422    0.155
##     Men.Sp.BdyMss2     0.340    0.030   11.293    0.000
##     Brilloun.Indx2     0.078    0.066    1.189    0.235
##     Mean.Rap.Crck2     0.144    0.029    4.963    0.000
##     X50km_Pop2         0.079    0.033    2.403    0.016
##     X10km_Pop2         0.399    0.042    9.438    0.000
##     GDP.Millions2      0.259    0.061    4.240    0.000
##     Nt_Pp._WB20152    -0.121    0.067   -1.811    0.070
##   Total.Animals2 ~
##     Zoo.Area.ha2       0.219    0.036    5.992    0.000
##     Sp.Richness2       0.805    0.051   15.917    0.000
##     GDP.Millions2     -0.129    0.072   -1.785    0.074
##   Sp.Richness2 ~
##     Zoo.Area.ha2       0.067    0.044    1.519    0.129
##     Prop.Mam.Sp2      -0.581    0.038  -15.231    0.000
##     Mam.Sp.Rchnss2     0.755    0.068   11.066    0.000
##   Brillouin.Index2 ~
##     Sp.Richness2       1.320    0.089   14.753    0.000
##     Total.Animals2    -0.620    0.127   -4.883    0.000
##     GDP.Millions2     -0.206    0.072   -2.846    0.004
##   Mean.Raup.Crick2 ~
##     Sp.Richness2      -0.355    0.230   -1.543    0.123
##     Total.Animals2     0.558    0.154    3.625    0.000
##     Men.Sp.BdyMss2    -0.495    0.052   -9.504    0.000
##
## Covariances:
##                      Estimate  Std.Err  z-value  P(>|z|)
##   Zoo.Area.ha2 ~~
##     Mam.Sp.Rchnss2       0.381    0.064    5.985    0.000
##     Prop.Mam.Sp2         0.312    0.089    3.510    0.000
##     Men.Sp.BdyMss2       0.534    0.104    5.147    0.000
##     X50km_Pop2           0.060    0.069    0.880    0.379
##     X10km_Pop2          -0.010    0.078   -0.124    0.901
##     GDP.Millions2       -0.027    0.041   -0.673    0.501
##     Nt_Pp._WB20152       0.061    0.090    0.682    0.495
##   Mam.Sp.Richness2 ~~
##     Prop.Mam.Sp2         0.088    0.061    1.435    0.151
##     Men.Sp.BdyMss2       0.303    0.081    3.730    0.000
##     X50km_Pop2           0.204    0.082    2.487    0.013
##     X10km_Pop2           0.284    0.066    4.278    0.000
##     GDP.Millions2       -0.060    0.064   -0.937    0.349
##     Nt_Pp._WB20152      -0.088    0.102   -0.863    0.388
##   Prop.Mam.Sp2 ~~
##     Men.Sp.BdyMss2       0.604    0.130    4.656    0.000
##     X50km_Pop2          -0.096    0.051   -1.888    0.059
##     X10km_Pop2          -0.204    0.055   -3.685    0.000
##     GDP.Millions2       -0.123    0.105   -1.172    0.241
```

```
##     Nt_Pp._WB20152       -0.115    0.091   -1.257    0.209
##   Mean.Sp.BodyMass2 ~~
##     X50km_Pop2            0.101    0.072    1.400    0.161
##     X10km_Pop2            0.126    0.067    1.895    0.058
##     GDP.Millions2        -0.082    0.046   -1.759    0.079
##     Nt_Pp._WB20152        0.006    0.092    0.063    0.950
##   X50km_Pop2 ~~
##     X10km_Pop2            0.752    0.126    5.982    0.000
##     GDP.Millions2        -0.013    0.100   -0.131    0.896
##     Nt_Pp._WB20152        0.191    0.177    1.083    0.279
##   X10km_Pop2 ~~
##     GDP.Millions2        -0.028    0.056   -0.505    0.613
##     Nt_Pp._WB20152        0.173    0.101    1.701    0.089
##   GDP.Millions2 ~~
##     Nt_Pp._WB20152        0.826    0.202    4.089    0.000
##
## Intercepts:
##                    Estimate  Std.Err  z-value  P(>|z|)
##    .Attendance2      -0.000    0.030   -0.000    1.000
##    .Total.Animals2   -0.000    0.070   -0.000    1.000
##    .Sp.Richness2      0.000    0.034    0.000    1.000
##    .Brilloun.Indx2   -0.000    0.055   -0.000    1.000
##    .Mean.Rap.Crck2    0.000    0.094    0.000    1.000
##     Zoo.Area.ha2      0.000    0.064    0.000    1.000
##     Mam.Sp.Rchnss2   -0.000    0.079   -0.000    1.000
##     Prop.Mam.Sp2      0.000    0.118    0.000    1.000
##     Men.Sp.BdyMss2   -0.000    0.080   -0.000    1.000
##     X50km_Pop2       -0.000    0.114   -0.000    1.000
##     X10km_Pop2        0.000    0.105    0.000    1.000
##     GDP.Millions2    -0.000    0.345   -0.000    1.000
##     Nt_Pp._WB20152    0.000    0.295    0.000    1.000
##
## Variances:
##                    Estimate  Std.Err  z-value  P(>|z|)
##    .Attendance2       0.267    0.028    9.565    0.000
##    .Total.Animals2    0.235    0.038    6.139    0.000
##    .Sp.Richness2      0.151    0.046    3.269    0.001
##    .Brilloun.Indx2    0.251    0.053    4.691    0.000
##    .Mean.Rap.Crck2    0.704    0.063   11.098    0.000
##     Zoo.Area.ha2      0.998    0.112    8.886    0.000
##     Mam.Sp.Rchnss2    0.998    0.115    8.669    0.000
##     Prop.Mam.Sp2      0.998    0.150    6.662    0.000
##     Men.Sp.BdyMss2    0.998    0.173    5.783    0.000
##     X50km_Pop2        0.998    0.131    7.623    0.000
##     X10km_Pop2        0.998    0.174    5.745    0.000
##     GDP.Millions2     0.998    0.246    4.061    0.000
##     Nt_Pp._WB20152    0.998    0.225    4.439    0.000
##
## R-Square:
##                    Estimate
##     Attendance2       0.719
##     Total.Animals2    0.766
##     Sp.Richness2      0.849
##     Brilloun.Indx2    0.750
```

```
##     Mean.Rap.Crck2    0.291
```

```r
# Generate fit indices
fitMeasures(fit.adj10, c("agfi", "rmr", "srmr", "rmsea", "cfi", "nnfi", "tli"))
```

```
##  agfi   rmr  srmr rmsea   cfi  nnfi   tli
## 0.753 0.050 0.047 0.122 0.942 0.889 0.889
```

```r
# Generate modification indices
mi10adj <- modindices(fit.adj10)
print(mi10adj[mi10adj$mi > 3.0,])
```

```
##                  lhs op             rhs     mi mi.scaled    epc sepc.lv
## 84    Total.Animals2 ~~ Brillouin.Index2  5.614     1.355 -0.066  -0.066
## 85    Total.Animals2 ~~ Mean.Raup.Crick2  4.259     1.028 -0.096  -0.096
## 86      Sp.Richness2 ~~ Brillouin.Index2 12.470     3.009  0.035   0.035
## 87      Sp.Richness2 ~~ Mean.Raup.Crick2 27.601     6.660 -0.087  -0.087
## 89    Total.Animals2  ~      Attendance2  3.651     0.881  0.082   0.082
## 90    Total.Animals2  ~ Brillouin.Index2  5.614     1.355 -0.264  -0.264
## 95    Total.Animals2  ~       X50km_Pop2 36.696     8.854  0.141   0.141
## 96    Total.Animals2  ~       X10km_Pop2  7.848     1.894  0.068   0.068
## 97    Total.Animals2  ~ Nat_Pop._WB20152 10.481     2.529  0.132   0.132
## 100     Sp.Richness2  ~ Brillouin.Index2  6.033     1.456  0.081   0.081
## 101     Sp.Richness2  ~ Mean.Raup.Crick2 17.310     4.177 -0.085  -0.085
## 106     Sp.Richness2  ~ Nat_Pop._WB20152  3.059     0.738 -0.032  -0.032
## 109 Brillouin.Index2  ~      Zoo.Area.ha2  5.613     1.354  0.062   0.062
## 111 Brillouin.Index2  ~      Prop.Mam.Sp2  7.555     1.823  0.076   0.076
## 114 Brillouin.Index2  ~       X10km_Pop2  5.633     1.359 -0.059  -0.059
## 116 Mean.Raup.Crick2  ~      Attendance2 20.515     4.950  0.411   0.411
## 118 Mean.Raup.Crick2  ~      Zoo.Area.ha2  9.137     2.205  0.154   0.154
## 120 Mean.Raup.Crick2  ~      Prop.Mam.Sp2 28.744     6.936 -0.319  -0.319
## 121 Mean.Raup.Crick2  ~       X50km_Pop2 14.172     3.420  0.152   0.152
## 122 Mean.Raup.Crick2  ~       X10km_Pop2 19.556     4.719  0.187   0.187
## 124 Mean.Raup.Crick2  ~ Nat_Pop._WB20152 17.087     4.123  0.165   0.165
## 125     Zoo.Area.ha2  ~      Attendance2  6.045     1.459  0.458   0.458
## 127     Zoo.Area.ha2  ~     Sp.Richness2  9.363     2.259  0.602   0.602
## 128     Zoo.Area.ha2  ~ Brillouin.Index2  6.203     1.497  0.169   0.169
## 129     Zoo.Area.ha2  ~ Mean.Raup.Crick2  7.812     1.885  0.124   0.124
## 137 Mam.Sp.Richness2  ~      Attendance2  6.484     1.565 -0.477  -0.477
## 138 Mam.Sp.Richness2  ~   Total.Animals2  5.172     1.248 -0.226  -0.226
## 139 Mam.Sp.Richness2  ~     Sp.Richness2  9.520     2.297 -0.742  -0.742
## 141 Mam.Sp.Richness2  ~ Mean.Raup.Crick2  5.330     1.286 -0.133  -0.133
## 152     Prop.Mam.Sp2  ~ Brillouin.Index2  3.002     0.724  0.105   0.105
## 153     Prop.Mam.Sp2  ~ Mean.Raup.Crick2 10.813     2.609 -0.141  -0.141
## 173       X50km_Pop2  ~      Attendance2 30.576     7.378  0.590   0.590
## 174       X50km_Pop2  ~   Total.Animals2 29.464     7.109  0.281   0.281
## 177       X50km_Pop2  ~ Mean.Raup.Crick2  3.672     0.886  0.065   0.065
## 185       X10km_Pop2  ~      Attendance2  3.167     0.764 -0.174  -0.174
## 186       X10km_Pop2  ~   Total.Animals2  3.040     0.733 -0.083  -0.083
## 198     GDP.Millions2  ~   Total.Animals2  4.925     1.188  0.102   0.102
## 199     GDP.Millions2  ~     Sp.Richness2 30.345     7.322  0.338   0.338
## 200     GDP.Millions2  ~ Brillouin.Index2 18.586     4.485  0.173   0.173
## 201     GDP.Millions2  ~ Mean.Raup.Crick2 10.852     2.618 -0.091  -0.091
## 210 Nat_Pop._WB20152  ~   Total.Animals2  3.943     0.951 -0.079  -0.079
## 211 Nat_Pop._WB20152  ~     Sp.Richness2 31.563     7.616 -0.333  -0.333
## 212 Nat_Pop._WB20152  ~ Brillouin.Index2 16.094     3.883 -0.139  -0.139
```

```
## 213 Nat_Pop._WB20152  ~ Mean.Raup.Crick2 15.733     3.796  0.104   0.104
##     sepc.all sepc.nox
## 84    -0.066   -0.066
## 85    -0.096   -0.096
## 86     0.035    0.035
## 87    -0.087   -0.087
## 89     0.079    0.079
## 90    -0.264   -0.264
## 95     0.140    0.140
## 96     0.067    0.067
## 97     0.132    0.132
## 100    0.081    0.081
## 101   -0.085   -0.085
## 106   -0.032   -0.032
## 109    0.062    0.062
## 111    0.076    0.076
## 114   -0.059   -0.059
## 116    0.402    0.402
## 118    0.155    0.155
## 120   -0.320   -0.320
## 121    0.153    0.153
## 122    0.187    0.188
## 124    0.165    0.165
## 125    0.447    0.447
## 127    0.602    0.602
## 128    0.170    0.170
## 129    0.124    0.124
## 137   -0.465   -0.465
## 138   -0.227   -0.227
## 139   -0.742   -0.742
## 141   -0.133   -0.133
## 152    0.105    0.105
## 153   -0.141   -0.141
## 173    0.577    0.577
## 174    0.282    0.282
## 177    0.065    0.065
## 185   -0.170   -0.170
## 186   -0.083   -0.083
## 198    0.102    0.102
## 199    0.338    0.338
## 200    0.173    0.173
## 201   -0.091   -0.091
## 210   -0.080   -0.080
## 211   -0.333   -0.333
## 212   -0.139   -0.139
## 213    0.103    0.103
```

```r
# Test whether removal of pathway was justified
anova(fit.adj9, fit.adj10)
```

```
## Scaled Chi Square Difference Test (method = "satorra.bentler.2001")
##
##            Df   AIC   BIC  Chisq Chisq diff Df diff Pr(>Chisq)
## fit.adj9   25 12501 12827 202.66
## fit.adj10  26 12500 12822 203.89    0.22428       1     0.6358
```

```r
# no significant difference, so link was not needed.
```

Model 11

Based on the results generated from the nested tenth model, the highest p-value relationship to be considered for removal is **Attendance2** ~ **Brilloun.Indx2** with a p-value of **0.235**. Therefore we decide to remove this pathway. Once again, the model summary, fit indices and modification indices were all generated for the model, adjusting for the nested nature of data.

```r
# Attendance SEM (Presence-Absence)

# Model 11
# Removal of Attendance2 ~ Brilloun.Indx2, p = 0.235

mod.11 <- 'Attendance2 ~ Zoo.Area.ha2 + Sp.Richness2 + Total.Animals2
+ Mam.Sp.Richness2 + Prop.Mam.Sp2
+ Mean.Sp.BodyMass2 + Mean.Raup.Crick2
+ X50km_Pop2 + X10km_Pop2 + GDP.Millions2 + Nat_Pop._WB20152

Total.Animals2 ~ Zoo.Area.ha2 + Sp.Richness2 + GDP.Millions2
Sp.Richness2 ~ Zoo.Area.ha2 + Prop.Mam.Sp2 + Mam.Sp.Richness2
Brillouin.Index2 ~ Sp.Richness2 + Total.Animals2 + GDP.Millions2
Mean.Raup.Crick2 ~ Sp.Richness2 + Total.Animals2 + Mean.Sp.BodyMass2'

# Fit model and generate model summary
mod.11.fit <- sem(mod.11, data = sem_attendance_data, fixed.x=FALSE)
summary(mod.11.fit, rsq = TRUE)
```

```
## lavaan (0.5-23.1097) converged normally after  57 iterations
##
##   Number of observations                           458
##
##   Estimator                                         ML
##   Minimum Function Test Statistic              203.890
##   Degrees of freedom                                26
##   P-value (Chi-square)                           0.000
##
## Parameter Estimates:
##
##   Information                                 Expected
##   Standard Errors                             Standard
##
## Regressions:
##                    Estimate  Std.Err  z-value  P(>|z|)
##   Attendance2 ~
##     Zoo.Area.ha2      0.085    0.033    2.553    0.011
##     Sp.Richness2     -0.151    0.075   -2.018    0.044
##     Total.Animals2    0.411    0.053    7.834    0.000
##     Mam.Sp.Rchnss2    0.133    0.055    2.415    0.016
##     Prop.Mam.Sp2     -0.063    0.049   -1.286    0.198
##     Men.Sp.BdyMss2    0.340    0.039    8.648    0.000
##     Mean.Rap.Crck2    0.144    0.029    4.985    0.000
##     X50km_Pop2        0.079    0.038    2.099    0.036
```

```
##     X10km_Pop2          0.399    0.041    9.654    0.000
##     GDP.Millions2       0.242    0.048    5.070    0.000
##     Nt_Pp._WB20152     -0.121    0.049   -2.479    0.013
##   Total.Animals2 ~
##     Zoo.Area.ha2        0.219    0.023    9.495    0.000
##     Sp.Richness2        0.805    0.023   34.969    0.000
##     GDP.Millions2      -0.129    0.023   -5.669    0.000
##   Sp.Richness2 ~
##     Zoo.Area.ha2        0.067    0.021    3.225    0.001
##     Prop.Mam.Sp2       -0.581    0.019  -30.364    0.000
##     Mam.Sp.Rchnss2      0.755    0.020   38.373    0.000
##   Brillouin.Index2 ~
##     Sp.Richness2        1.320    0.044   30.015    0.000
##     Total.Animals2     -0.620    0.044  -14.052    0.000
##     GDP.Millions2      -0.206    0.024   -8.518    0.000
##   Mean.Raup.Crick2 ~
##     Sp.Richness2       -0.355    0.074   -4.791    0.000
##     Total.Animals2      0.558    0.074    7.566    0.000
##     Men.Sp.BdyMss2     -0.495    0.041  -12.212    0.000
##
## Covariances:
##                        Estimate  Std.Err  z-value  P(>|z|)
##  .Attendance2 ~~
##    .Brilloun.Indx2        0.020    0.012    1.610    0.107
##   Zoo.Area.ha2 ~~
##     Mam.Sp.Rchnss2        0.381    0.050    7.634    0.000
##     Prop.Mam.Sp2          0.312    0.049    6.389    0.000
##     Men.Sp.BdyMss2        0.534    0.053   10.096    0.000
##     X50km_Pop2            0.060    0.047    1.291    0.197
##     X10km_Pop2           -0.010    0.047   -0.208    0.835
##     GDP.Millions2        -0.027    0.047   -0.588    0.557
##     Nt_Pp._WB20152        0.061    0.047    1.310    0.190
##   Mam.Sp.Richness2 ~~
##     Prop.Mam.Sp2          0.088    0.047    1.882    0.060
##     Men.Sp.BdyMss2        0.303    0.049    6.216    0.000
##     X50km_Pop2            0.204    0.048    4.281    0.000
##     X10km_Pop2            0.284    0.048    5.852    0.000
##     GDP.Millions2        -0.060    0.047   -1.277    0.202
##     Nt_Pp._WB20152       -0.088    0.047   -1.881    0.060
##   Prop.Mam.Sp2 ~~
##     Men.Sp.BdyMss2        0.604    0.055   11.083    0.000
##     X50km_Pop2           -0.096    0.047   -2.054    0.040
##     X10km_Pop2           -0.204    0.048   -4.293    0.000
##     GDP.Millions2        -0.123    0.047   -2.615    0.009
##     Nt_Pp._WB20152       -0.115    0.047   -2.447    0.014
##   Mean.Sp.BodyMass2 ~~
##     X50km_Pop2            0.101    0.047    2.166    0.030
##     X10km_Pop2            0.126    0.047    2.683    0.007
##     GDP.Millions2        -0.082    0.047   -1.748    0.080
##     Nt_Pp._WB20152        0.006    0.047    0.124    0.901
##   X50km_Pop2 ~~
##     X10km_Pop2            0.752    0.058   12.886    0.000
##     GDP.Millions2        -0.013    0.047   -0.281    0.779
##     Nt_Pp._WB20152        0.191    0.047    4.033    0.000
```

```
##   X10km_Pop2 ~~
##     GDP.Millions2        -0.028    0.047   -0.604    0.546
##     Nt_Pp._WB20152        0.173    0.047    3.646    0.000
##   GDP.Millions2 ~~
##     Nt_Pp._WB20152        0.826    0.061   13.642    0.000
##
## Variances:
##                    Estimate  Std.Err  z-value  P(>|z|)
##    .Attendance2       0.269    0.018   15.133    0.000
##    .Total.Animals2    0.235    0.016   15.133    0.000
##    .Sp.Richness2      0.151    0.010   15.133    0.000
##    .Brilloun.Indx2    0.251    0.017   15.133    0.000
##    .Mean.Rap.Crck2    0.704    0.047   15.133    0.000
##     Zoo.Area.ha2      0.998    0.066   15.133    0.000
##     Mam.Sp.Rchnss2    0.998    0.066   15.133    0.000
##     Prop.Mam.Sp2      0.998    0.066   15.133    0.000
##     Men.Sp.BdyMss2    0.998    0.066   15.133    0.000
##     X50km_Pop2        0.998    0.066   15.133    0.000
##     X10km_Pop2        0.998    0.066   15.133    0.000
##     GDP.Millions2     0.998    0.066   15.133    0.000
##     Nt_Pp._WB20152    0.998    0.066   15.133    0.000
##
## R-Square:
##                    Estimate
##     Attendance2       0.717
##     Total.Animals2    0.766
##     Sp.Richness2      0.849
##     Brilloun.Indx2    0.750
##     Mean.Rap.Crck2    0.291
```

```
# Generate fit indices
fitMeasures(mod.11.fit, c("agfi", "rmr", "srmr", "rmsea", "cfi", "nnfi", "tli"))
```

```
##  agfi   rmr  srmr rmsea   cfi  nnfi   tli
## 0.784 0.050 0.050 0.122 0.942 0.889 0.889
```

```
# Generate modification indices
mi11 <- modindices(mod.11.fit)
print(mi11[mi11$mi > 3.0,])
```

```
##                  lhs op              rhs     mi    epc sepc.lv sepc.all
## 70    Total.Animals2 ~~ Brillouin.Index2  5.613 -0.066  -0.066   -0.066
## 71    Total.Animals2 ~~ Mean.Raup.Crick2  4.260 -0.096  -0.096   -0.096
## 72      Sp.Richness2 ~~ Brillouin.Index2 12.470  0.035   0.035    0.035
## 73      Sp.Richness2 ~~ Mean.Raup.Crick2 27.601 -0.087  -0.087   -0.087
## 76    Total.Animals2  ~      Attendance2  3.651  0.082   0.082    0.079
## 77    Total.Animals2  ~ Brillouin.Index2  5.613 -0.264  -0.264   -0.264
## 82    Total.Animals2  ~       X50km_Pop2 36.696  0.141   0.141    0.140
## 83    Total.Animals2  ~       X10km_Pop2  7.848  0.068   0.068    0.067
## 84    Total.Animals2  ~ Nat_Pop._WB20152 10.481  0.132   0.132    0.132
## 87      Sp.Richness2  ~ Brillouin.Index2  6.033  0.081   0.081    0.081
## 88      Sp.Richness2  ~ Mean.Raup.Crick2 17.310 -0.085  -0.085   -0.085
## 93      Sp.Richness2  ~ Nat_Pop._WB20152  3.059 -0.032  -0.032   -0.032
## 96  Brillouin.Index2  ~     Zoo.Area.ha2  5.613  0.062   0.062    0.062
## 98  Brillouin.Index2  ~     Prop.Mam.Sp2  7.555  0.076   0.076    0.076
## 101 Brillouin.Index2  ~       X10km_Pop2  5.633 -0.059  -0.059   -0.059
```

```
## 103 Mean.Raup.Crick2  ~      Attendance2 20.515  0.411   0.411    0.402
## 105 Mean.Raup.Crick2  ~     Zoo.Area.ha2  9.137  0.154   0.154    0.155
## 107 Mean.Raup.Crick2  ~     Prop.Mam.Sp2 28.743 -0.319  -0.319   -0.320
## 108 Mean.Raup.Crick2  ~       X50km_Pop2 14.172  0.152   0.152    0.153
## 109 Mean.Raup.Crick2  ~       X10km_Pop2 19.556  0.187   0.187    0.187
## 111 Mean.Raup.Crick2  ~ Nat_Pop._WB20152 17.087  0.165   0.165    0.165
## 112     Zoo.Area.ha2  ~      Attendance2  6.046  0.458   0.458    0.447
## 114     Zoo.Area.ha2  ~     Sp.Richness2  9.363  0.602   0.602    0.602
## 115     Zoo.Area.ha2  ~ Brillouin.Index2  6.203  0.169   0.169    0.170
## 116     Zoo.Area.ha2  ~ Mean.Raup.Crick2  7.812  0.124   0.124    0.124
## 124 Mam.Sp.Richness2  ~      Attendance2  6.484 -0.477  -0.477   -0.465
## 125 Mam.Sp.Richness2  ~   Total.Animals2  5.172 -0.226  -0.226   -0.227
## 126 Mam.Sp.Richness2  ~     Sp.Richness2  9.521 -0.742  -0.742   -0.742
## 128 Mam.Sp.Richness2  ~ Mean.Raup.Crick2  5.330 -0.133  -0.133   -0.133
## 139     Prop.Mam.Sp2  ~ Brillouin.Index2  3.002  0.105   0.105    0.105
## 140     Prop.Mam.Sp2  ~ Mean.Raup.Crick2 10.813 -0.141  -0.141   -0.141
## 160       X50km_Pop2  ~      Attendance2 30.577  0.590   0.590    0.577
## 161       X50km_Pop2  ~   Total.Animals2 29.464  0.281   0.281    0.282
## 164       X50km_Pop2  ~ Mean.Raup.Crick2  3.672  0.065   0.065    0.065
## 172       X10km_Pop2  ~      Attendance2  3.167 -0.174  -0.174   -0.170
## 173       X10km_Pop2  ~   Total.Animals2  3.040 -0.083  -0.083   -0.083
## 185    GDP.Millions2  ~   Total.Animals2  4.926  0.102   0.102    0.102
## 186    GDP.Millions2  ~     Sp.Richness2 30.346  0.338   0.338    0.338
## 187    GDP.Millions2  ~ Brillouin.Index2 18.587  0.173   0.173    0.173
## 188    GDP.Millions2  ~ Mean.Raup.Crick2 10.851 -0.091  -0.091   -0.091
## 197 Nat_Pop._WB20152  ~   Total.Animals2  3.943 -0.079  -0.079   -0.080
## 198 Nat_Pop._WB20152  ~     Sp.Richness2 31.565 -0.333  -0.333   -0.333
## 199 Nat_Pop._WB20152  ~ Brillouin.Index2 16.094 -0.139  -0.139   -0.139
## 200 Nat_Pop._WB20152  ~ Mean.Raup.Crick2 15.732  0.104   0.104    0.103
##     sepc.nox
## 70    -0.066
## 71    -0.096
## 72     0.035
## 73    -0.087
## 76     0.079
## 77    -0.264
## 82     0.140
## 83     0.067
## 84     0.132
## 87     0.081
## 88    -0.085
## 93    -0.032
## 96     0.062
## 98     0.076
## 101   -0.059
## 103    0.402
## 105    0.155
## 107   -0.320
## 108    0.153
## 109    0.188
## 111    0.165
## 112    0.447
## 114    0.602
## 115    0.170
```

```
## 116    0.124
## 124   -0.465
## 125   -0.227
## 126   -0.742
## 128   -0.133
## 139    0.105
## 140   -0.141
## 160    0.577
## 161    0.282
## 164    0.065
## 172   -0.170
## 173   -0.083
## 185    0.102
## 186    0.338
## 187    0.173
## 188   -0.091
## 197   -0.080
## 198   -0.333
## 199   -0.139
## 200    0.103
```

```
# Adjust for the nested nature of the data (institutions within countries)
# Fit model and generate model summary
design <- svydesign(ids = ~Country, nest=TRUE, data=sem_attendance_data)
fit.adj11 <- lavaan.survey(lavaan.fit = mod.11.fit, survey.design = design)
summary(fit.adj11, rsq = TRUE)
```

```
## lavaan (0.5-23.1097) converged normally after  55 iterations
##
##   Number of observations                           458
##
##   Estimator                                         ML      Robust
##   Minimum Function Test Statistic              203.890      49.197
##   Degrees of freedom                                26          26
##   P-value (Chi-square)                           0.000       0.004
##   Scaling correction factor                                  4.144
##     for the Satorra-Bentler correction
##
## Parameter Estimates:
##
##   Information                                 Expected
##   Standard Errors                           Robust.sem
##
## Regressions:
##                      Estimate  Std.Err  z-value  P(>|z|)
##   Attendance2 ~
##     Zoo.Area.ha2        0.085    0.041    2.078    0.038
##     Sp.Richness2       -0.151    0.065   -2.311    0.021
##     Total.Animals2      0.411    0.060    6.799    0.000
##     Mam.Sp.Rchnss2      0.133    0.049    2.737    0.006
##     Prop.Mam.Sp2       -0.063    0.044   -1.422    0.155
##     Men.Sp.BdyMss2      0.340    0.030   11.293    0.000
##     Mean.Rap.Crck2      0.144    0.029    4.963    0.000
##     X50km_Pop2          0.079    0.033    2.403    0.016
##     X10km_Pop2          0.399    0.042    9.438    0.000
```

```
##     GDP.Millions2       0.242    0.059    4.096    0.000
##     Nt_Pp._WB20152     -0.121    0.067   -1.811    0.070
##   Total.Animals2 ~
##     Zoo.Area.ha2        0.219    0.036    5.992    0.000
##     Sp.Richness2        0.805    0.051   15.917    0.000
##     GDP.Millions2      -0.129    0.072   -1.785    0.074
##   Sp.Richness2 ~
##     Zoo.Area.ha2        0.067    0.044    1.519    0.129
##     Prop.Mam.Sp2       -0.581    0.038  -15.231    0.000
##     Mam.Sp.Rchnss2      0.755    0.068   11.066    0.000
##   Brillouin.Index2 ~
##     Sp.Richness2        1.320    0.089   14.753    0.000
##     Total.Animals2     -0.620    0.127   -4.883    0.000
##     GDP.Millions2      -0.206    0.072   -2.846    0.004
##   Mean.Raup.Crick2 ~
##     Sp.Richness2       -0.355    0.230   -1.543    0.123
##     Total.Animals2      0.558    0.154    3.625    0.000
##     Men.Sp.BdyMss2     -0.495    0.052   -9.504    0.000
##
## Covariances:
##                     Estimate  Std.Err  z-value  P(>|z|)
##  .Attendance2 ~~
##    .Brilloun.Indx2       0.020    0.018    1.082    0.279
##   Zoo.Area.ha2 ~~
##     Mam.Sp.Rchnss2       0.381    0.064    5.985    0.000
##     Prop.Mam.Sp2         0.312    0.089    3.510    0.000
##     Men.Sp.BdyMss2       0.534    0.104    5.147    0.000
##     X50km_Pop2           0.060    0.069    0.880    0.379
##     X10km_Pop2          -0.010    0.078   -0.124    0.901
##     GDP.Millions2       -0.027    0.041   -0.673    0.501
##     Nt_Pp._WB20152       0.061    0.090    0.682    0.495
##   Mam.Sp.Richness2 ~~
##     Prop.Mam.Sp2         0.088    0.061    1.435    0.151
##     Men.Sp.BdyMss2       0.303    0.081    3.730    0.000
##     X50km_Pop2           0.204    0.082    2.487    0.013
##     X10km_Pop2           0.284    0.066    4.278    0.000
##     GDP.Millions2       -0.060    0.064   -0.937    0.349
##     Nt_Pp._WB20152      -0.088    0.102   -0.863    0.388
##   Prop.Mam.Sp2 ~~
##     Men.Sp.BdyMss2       0.604    0.130    4.656    0.000
##     X50km_Pop2          -0.096    0.051   -1.888    0.059
##     X10km_Pop2          -0.204    0.055   -3.685    0.000
##     GDP.Millions2       -0.123    0.105   -1.172    0.241
##     Nt_Pp._WB20152      -0.115    0.091   -1.257    0.209
##   Mean.Sp.BodyMass2 ~~
##     X50km_Pop2           0.101    0.072    1.400    0.161
##     X10km_Pop2           0.126    0.067    1.895    0.058
##     GDP.Millions2       -0.082    0.046   -1.759    0.079
##     Nt_Pp._WB20152       0.006    0.092    0.063    0.950
##   X50km_Pop2 ~~
##     X10km_Pop2           0.752    0.126    5.982    0.000
##     GDP.Millions2       -0.013    0.100   -0.131    0.896
##     Nt_Pp._WB20152       0.191    0.177    1.083    0.279
##   X10km_Pop2 ~~
```

```
##     GDP.Millions2        -0.028    0.056   -0.505    0.613
##     Nt_Pp._WB20152        0.173    0.101    1.701    0.089
##   GDP.Millions2 ~~
##     Nt_Pp._WB20152        0.826    0.202    4.089    0.000
##
## Intercepts:
##                    Estimate  Std.Err  z-value  P(>|z|)
##    .Attendance2      -0.000    0.029   -0.000    1.000
##    .Total.Animals2   -0.000    0.070   -0.000    1.000
##    .Sp.Richness2      0.000    0.034    0.000    1.000
##    .Brilloun.Indx2   -0.000    0.055   -0.000    1.000
##    .Mean.Rap.Crck2    0.000    0.094    0.000    1.000
##     Zoo.Area.ha2      0.000    0.064    0.000    1.000
##     Mam.Sp.Rchnss2   -0.000    0.079   -0.000    1.000
##     Prop.Mam.Sp2      0.000    0.118    0.000    1.000
##     Men.Sp.BdyMss2   -0.000    0.080   -0.000    1.000
##     X50km_Pop2       -0.000    0.114   -0.000    1.000
##     X10km_Pop2        0.000    0.105    0.000    1.000
##     GDP.Millions2    -0.000    0.345   -0.000    1.000
##     Nt_Pp._WB20152    0.000    0.295    0.000    1.000
##
## Variances:
##                    Estimate  Std.Err  z-value  P(>|z|)
##    .Attendance2       0.269    0.027   10.083    0.000
##    .Total.Animals2    0.235    0.038    6.139    0.000
##    .Sp.Richness2      0.151    0.046    3.269    0.001
##    .Brilloun.Indx2    0.251    0.053    4.691    0.000
##    .Mean.Rap.Crck2    0.704    0.063   11.098    0.000
##     Zoo.Area.ha2      0.998    0.112    8.886    0.000
##     Mam.Sp.Rchnss2    0.998    0.115    8.670    0.000
##     Prop.Mam.Sp2      0.998    0.150    6.662    0.000
##     Men.Sp.BdyMss2    0.998    0.173    5.783    0.000
##     X50km_Pop2        0.998    0.131    7.623    0.000
##     X10km_Pop2        0.998    0.174    5.745    0.000
##     GDP.Millions2     0.998    0.246    4.061    0.000
##     Nt_Pp._WB20152    0.998    0.225    4.439    0.000
##
## R-Square:
##                    Estimate
##     Attendance2       0.717
##     Total.Animals2    0.766
##     Sp.Richness2      0.849
##     Brilloun.Indx2    0.750
##     Mean.Rap.Crck2    0.291
```

```r
# Generate fit indices
fitMeasures(fit.adj11, c("agfi", "rmr", "srmr", "rmsea", "cfi", "nnfi", "tli"))
```

```
##  agfi   rmr  srmr rmsea   cfi  nnfi   tli
## 0.753 0.050 0.047 0.122 0.942 0.889 0.889
```

```r
# Generate modification indices
mi11adj <- modindices(fit.adj11)
print(mi11adj[mi11adj$mi > 3.0,])
```

```
##                    lhs op              rhs     mi mi.scaled    epc sepc.lv
```

```
## 83    Total.Animals2 ~~ Brillouin.Index2  5.613     1.354 -0.066  -0.066
## 84    Total.Animals2 ~~ Mean.Raup.Crick2  4.260     1.028 -0.096  -0.096
## 85      Sp.Richness2 ~~ Brillouin.Index2 12.470     3.009  0.035   0.035
## 86      Sp.Richness2 ~~ Mean.Raup.Crick2 27.601     6.660 -0.087  -0.087
## 89    Total.Animals2  ~      Attendance2  3.651     0.881  0.082   0.082
## 90    Total.Animals2  ~ Brillouin.Index2  5.613     1.354 -0.264  -0.264
## 95    Total.Animals2  ~       X50km_Pop2 36.696     8.854  0.141   0.141
## 96    Total.Animals2  ~       X10km_Pop2  7.848     1.894  0.068   0.068
## 97    Total.Animals2  ~ Nat_Pop._WB20152 10.481     2.529  0.132   0.132
## 100     Sp.Richness2  ~ Brillouin.Index2  6.033     1.456  0.081   0.081
## 101     Sp.Richness2  ~ Mean.Raup.Crick2 17.310     4.177 -0.085  -0.085
## 106     Sp.Richness2  ~ Nat_Pop._WB20152  3.059     0.738 -0.032  -0.032
## 109 Brillouin.Index2  ~     Zoo.Area.ha2  5.613     1.354  0.062   0.062
## 111 Brillouin.Index2  ~     Prop.Mam.Sp2  7.555     1.823  0.076   0.076
## 114 Brillouin.Index2  ~       X10km_Pop2  5.633     1.359 -0.059  -0.059
## 116 Mean.Raup.Crick2  ~      Attendance2 20.515     4.950  0.411   0.411
## 118 Mean.Raup.Crick2  ~     Zoo.Area.ha2  9.137     2.205  0.154   0.154
## 120 Mean.Raup.Crick2  ~     Prop.Mam.Sp2 28.743     6.935 -0.319  -0.319
## 121 Mean.Raup.Crick2  ~       X50km_Pop2 14.172     3.419  0.152   0.152
## 122 Mean.Raup.Crick2  ~       X10km_Pop2 19.556     4.719  0.187   0.187
## 124 Mean.Raup.Crick2  ~ Nat_Pop._WB20152 17.087     4.123  0.165   0.165
## 125     Zoo.Area.ha2  ~      Attendance2  6.046     1.459  0.458   0.458
## 127     Zoo.Area.ha2  ~     Sp.Richness2  9.363     2.259  0.602   0.602
## 128     Zoo.Area.ha2  ~ Brillouin.Index2  6.203     1.497  0.169   0.169
## 129     Zoo.Area.ha2  ~ Mean.Raup.Crick2  7.812     1.885  0.124   0.124
## 137 Mam.Sp.Richness2  ~      Attendance2  6.484     1.565 -0.477  -0.477
## 138 Mam.Sp.Richness2  ~   Total.Animals2  5.172     1.248 -0.226  -0.226
## 139 Mam.Sp.Richness2  ~     Sp.Richness2  9.521     2.297 -0.743  -0.743
## 141 Mam.Sp.Richness2  ~ Mean.Raup.Crick2  5.330     1.286 -0.133  -0.133
## 152     Prop.Mam.Sp2  ~ Brillouin.Index2  3.002     0.724  0.105   0.105
## 153     Prop.Mam.Sp2  ~ Mean.Raup.Crick2 10.813     2.609 -0.141  -0.141
## 173       X50km_Pop2  ~      Attendance2 30.576     7.378  0.590   0.590
## 174       X50km_Pop2  ~   Total.Animals2 29.464     7.109  0.281   0.281
## 177       X50km_Pop2  ~ Mean.Raup.Crick2  3.672     0.886  0.065   0.065
## 185       X10km_Pop2  ~      Attendance2  3.167     0.764 -0.174  -0.174
## 186       X10km_Pop2  ~   Total.Animals2  3.040     0.733 -0.083  -0.083
## 198    GDP.Millions2  ~   Total.Animals2  4.925     1.188  0.102   0.102
## 199    GDP.Millions2  ~     Sp.Richness2 30.346     7.322  0.338   0.338
## 200    GDP.Millions2  ~ Brillouin.Index2 18.587     4.485  0.173   0.173
## 201    GDP.Millions2  ~ Mean.Raup.Crick2 10.851     2.618 -0.091  -0.091
## 210 Nat_Pop._WB20152  ~   Total.Animals2  3.943     0.951 -0.079  -0.079
## 211 Nat_Pop._WB20152  ~     Sp.Richness2 31.564     7.616 -0.333  -0.333
## 212 Nat_Pop._WB20152  ~ Brillouin.Index2 16.094     3.883 -0.139  -0.139
## 213 Nat_Pop._WB20152  ~ Mean.Raup.Crick2 15.732     3.796  0.104   0.104
##     sepc.all sepc.nox
## 83    -0.066   -0.066
## 84    -0.096   -0.096
## 85     0.035    0.035
## 86    -0.087   -0.087
## 89     0.079    0.079
## 90    -0.264   -0.264
## 95     0.140    0.140
## 96     0.067    0.067
## 97     0.132    0.132
```

```
## 100    0.081    0.081
## 101   -0.085   -0.085
## 106   -0.032   -0.032
## 109    0.062    0.062
## 111    0.076    0.076
## 114   -0.059   -0.059
## 116    0.402    0.402
## 118    0.155    0.155
## 120   -0.320   -0.320
## 121    0.153    0.153
## 122    0.187    0.188
## 124    0.165    0.165
## 125    0.447    0.447
## 127    0.602    0.602
## 128    0.170    0.170
## 129    0.124    0.124
## 137   -0.465   -0.465
## 138   -0.227   -0.227
## 139   -0.743   -0.743
## 141   -0.133   -0.133
## 152    0.105    0.105
## 153   -0.141   -0.141
## 173    0.577    0.577
## 174    0.282    0.282
## 177    0.065    0.065
## 185   -0.170   -0.170
## 186   -0.083   -0.083
## 198    0.102    0.102
## 199    0.338    0.338
## 200    0.173    0.173
## 201   -0.091   -0.091
## 210   -0.080   -0.080
## 211   -0.333   -0.333
## 212   -0.139   -0.139
## 213    0.103    0.103
```

```
# Test whether removal of pathway was justified
anova(fit.adj10, fit.adj11)
```

```
## Scaled Chi Square Difference Test (method = "satorra.bentler.2001")
##
##           Df   AIC   BIC  Chisq Chisq diff Df diff Pr(>Chisq)
## fit.adj10 26 12500 12822 203.89
## fit.adj11 26 12500 12822 203.89          0       0          1
```

```
# no significant difference, so link was not needed.
```

Model 12

Based on the results generated from the nested eleventh model, the highest p-value relationship to be considered for removal is **Attendance2 ~ Prop.Mam.Sp2** with a p-value of **0.155**. Therefore we decide to remove this pathway. Once again, the model summary, fit indices and modification indices were all generated for the model, adjusting for the nested nature of data.

```r
# Attendance SEM (Presence-Absence)

# Model 12
# Removal of Attendance2 ~ Prop.Mam.Sp2, p = 0.155

mod.12 <- 'Attendance2 ~ Zoo.Area.ha2 + Sp.Richness2 + Total.Animals2
+ Mam.Sp.Richness2 + Mean.Sp.BodyMass2 + Mean.Raup.Crick2
+ X50km_Pop2 + X10km_Pop2 + GDP.Millions2 + Nat_Pop._WB20152

Total.Animals2 ~ Zoo.Area.ha2 + Sp.Richness2 + GDP.Millions2
Sp.Richness2 ~ Zoo.Area.ha2 + Prop.Mam.Sp2 + Mam.Sp.Richness2
Brillouin.Index2 ~ Sp.Richness2 + Total.Animals2 + GDP.Millions2
Mean.Raup.Crick2 ~ Sp.Richness2 + Total.Animals2 + Mean.Sp.BodyMass2'

# Fit model and generate model summary
mod.12.fit <- sem(mod.12, data = sem_attendance_data, fixed.x=FALSE)
summary(mod.12.fit, rsq = TRUE)
```

```
## lavaan (0.5-23.1097) converged normally after  55 iterations
##
##   Number of observations                           458
##
##   Estimator                                         ML
##   Minimum Function Test Statistic              205.381
##   Degrees of freedom                                27
##   P-value (Chi-square)                           0.000
##
## Parameter Estimates:
##
##   Information                                 Expected
##   Standard Errors                             Standard
##
## Regressions:
##                    Estimate  Std.Err  z-value  P(>|z|)
##   Attendance2 ~
##     Zoo.Area.ha2      0.079    0.033    2.374    0.018
##     Sp.Richness2     -0.086    0.059   -1.458    0.145
##     Total.Animals2    0.407    0.053    7.739    0.000
##     Mam.Sp.Rchnss2    0.089    0.043    2.059    0.039
##     Men.Sp.BdyMss2    0.328    0.036    9.009    0.000
##     Mean.Rap.Crck2    0.153    0.029    5.305    0.000
##     X50km_Pop2        0.074    0.038    1.966    0.049
##     X10km_Pop2        0.406    0.040   10.020    0.000
##     GDP.Millions2     0.234    0.048    4.896    0.000
##     Nt_Pp._WB20152   -0.111    0.049   -2.264    0.024
##   Total.Animals2 ~
##     Zoo.Area.ha2      0.219    0.023    9.495    0.000
##     Sp.Richness2      0.805    0.023   34.969    0.000
##     GDP.Millions2    -0.129    0.023   -5.669    0.000
##   Sp.Richness2 ~
##     Zoo.Area.ha2      0.067    0.021    3.225    0.001
##     Prop.Mam.Sp2     -0.581    0.019  -30.364    0.000
##     Mam.Sp.Rchnss2    0.755    0.020   38.373    0.000
##   Brillouin.Index2 ~
```

```
##     Sp.Richness2        1.320    0.044   30.015    0.000
##     Total.Animals2     -0.620    0.044  -14.052    0.000
##     GDP.Millions2      -0.206    0.024   -8.518    0.000
##   Mean.Raup.Crick2 ~
##     Sp.Richness2       -0.355    0.074   -4.791    0.000
##     Total.Animals2      0.558    0.074    7.566    0.000
##     Men.Sp.BdyMss2     -0.495    0.041  -12.212    0.000
##
## Covariances:
##                        Estimate  Std.Err  z-value  P(>|z|)
##  .Attendance2 ~~
##    .Brilloun.Indx2        0.017    0.012    1.422    0.155
##   Zoo.Area.ha2 ~~
##     Mam.Sp.Rchnss2        0.381    0.050    7.634    0.000
##     Men.Sp.BdyMss2        0.534    0.053   10.096    0.000
##     X50km_Pop2            0.060    0.047    1.291    0.197
##     X10km_Pop2           -0.010    0.047   -0.208    0.835
##     GDP.Millions2        -0.027    0.047   -0.588    0.557
##     Nt_Pp._WB20152        0.061    0.047    1.310    0.190
##     Prop.Mam.Sp2          0.312    0.049    6.389    0.000
##   Mam.Sp.Richness2 ~~
##     Men.Sp.BdyMss2        0.303    0.049    6.216    0.000
##     X50km_Pop2            0.204    0.048    4.281    0.000
##     X10km_Pop2            0.284    0.048    5.852    0.000
##     GDP.Millions2        -0.060    0.047   -1.277    0.202
##     Nt_Pp._WB20152       -0.088    0.047   -1.881    0.060
##     Prop.Mam.Sp2          0.088    0.047    1.882    0.060
##   Mean.Sp.BodyMass2 ~~
##     X50km_Pop2            0.101    0.047    2.166    0.030
##     X10km_Pop2            0.126    0.047    2.683    0.007
##     GDP.Millions2        -0.082    0.047   -1.748    0.080
##     Nt_Pp._WB20152        0.006    0.047    0.124    0.901
##     Prop.Mam.Sp2          0.604    0.055   11.083    0.000
##   X50km_Pop2 ~~
##     X10km_Pop2            0.752    0.058   12.886    0.000
##     GDP.Millions2        -0.013    0.047   -0.281    0.779
##     Nt_Pp._WB20152        0.191    0.047    4.033    0.000
##     Prop.Mam.Sp2         -0.096    0.047   -2.054    0.040
##   X10km_Pop2 ~~
##     GDP.Millions2        -0.028    0.047   -0.604    0.546
##     Nt_Pp._WB20152        0.173    0.047    3.646    0.000
##     Prop.Mam.Sp2         -0.204    0.048   -4.293    0.000
##   GDP.Millions2 ~~
##     Nt_Pp._WB20152        0.826    0.061   13.642    0.000
##     Prop.Mam.Sp2         -0.123    0.047   -2.615    0.009
##   Nat_Pop._WB20152 ~~
##     Prop.Mam.Sp2         -0.115    0.047   -2.447    0.014
##
## Variances:
##                    Estimate  Std.Err  z-value  P(>|z|)
##    .Attendance2       0.269    0.018   15.133    0.000
##    .Total.Animals2    0.235    0.016   15.133    0.000
##    .Sp.Richness2      0.151    0.010   15.133    0.000
##    .Brilloun.Indx2    0.251    0.017   15.133    0.000
```

```
##    .Mean.Rap.Crck2    0.704    0.047   15.133    0.000
##     Zoo.Area.ha2      0.998    0.066   15.133    0.000
##     Mam.Sp.Rchnss2    0.998    0.066   15.133    0.000
##     Men.Sp.BdyMss2    0.998    0.066   15.133    0.000
##     X50km_Pop2        0.998    0.066   15.133    0.000
##     X10km_Pop2        0.998    0.066   15.133    0.000
##     GDP.Millions2     0.998    0.066   15.133    0.000
##     Nt_Pp._WB20152    0.998    0.066   15.133    0.000
##     Prop.Mam.Sp2      0.998    0.066   15.133    0.000
##
## R-Square:
##                    Estimate
##     Attendance2       0.717
##     Total.Animals2    0.766
##     Sp.Richness2      0.849
##     Brilloun.Indx2    0.750
##     Mean.Rap.Crck2    0.291
```

```
# Generate fit indices
fitMeasures(mod.12.fit, c("agfi", "rmr", "srmr", "rmsea", "cfi", "nnfi", "tli"))
```

```
##  agfi   rmr  srmr rmsea   cfi  nnfi   tli
## 0.790 0.050 0.050 0.120 0.942 0.893 0.893
```

```
# Generate modification indices
mi12 <- modindices(mod.12.fit)
print(mi12[mi12$mi > 3.0,])
```

```
##                  lhs op               rhs     mi    epc sepc.lv sepc.all
## 69    Total.Animals2 ~~ Brillouin.Index2  5.613 -0.066  -0.066   -0.066
## 70    Total.Animals2 ~~ Mean.Raup.Crick2  4.260 -0.096  -0.096   -0.096
## 71      Sp.Richness2 ~~ Brillouin.Index2 12.875  0.035   0.035    0.035
## 72      Sp.Richness2 ~~ Mean.Raup.Crick2 27.601 -0.087  -0.087   -0.087
## 76    Total.Animals2  ~      Attendance2  3.623  0.082   0.082    0.079
## 77    Total.Animals2  ~ Brillouin.Index2  5.613 -0.264  -0.264   -0.264
## 81    Total.Animals2  ~       X50km_Pop2 36.696  0.141   0.141    0.140
## 82    Total.Animals2  ~       X10km_Pop2  7.848  0.068   0.068    0.067
## 83    Total.Animals2  ~ Nat_Pop._WB20152 10.481  0.132   0.132    0.132
## 87      Sp.Richness2  ~ Brillouin.Index2  6.033  0.081   0.081    0.081
## 88      Sp.Richness2  ~ Mean.Raup.Crick2 17.310 -0.085  -0.085   -0.085
## 93      Sp.Richness2  ~ Nat_Pop._WB20152  3.059 -0.032  -0.032   -0.032
## 96  Brillouin.Index2  ~     Zoo.Area.ha2  5.613  0.062   0.062    0.062
## 100 Brillouin.Index2  ~       X10km_Pop2  5.633 -0.059  -0.059   -0.059
## 102 Brillouin.Index2  ~     Prop.Mam.Sp2  7.793  0.077   0.077    0.077
## 103 Mean.Raup.Crick2  ~      Attendance2 17.519  0.384   0.384    0.376
## 105 Mean.Raup.Crick2  ~     Zoo.Area.ha2  9.137  0.154   0.154    0.155
## 107 Mean.Raup.Crick2  ~       X50km_Pop2 14.172  0.152   0.152    0.153
## 108 Mean.Raup.Crick2  ~       X10km_Pop2 19.556  0.187   0.187    0.187
## 110 Mean.Raup.Crick2  ~ Nat_Pop._WB20152 17.087  0.165   0.165    0.165
## 111 Mean.Raup.Crick2  ~     Prop.Mam.Sp2 28.743 -0.319  -0.319   -0.320
## 112     Zoo.Area.ha2  ~      Attendance2  6.317  0.452   0.452    0.441
## 114     Zoo.Area.ha2  ~     Sp.Richness2  9.363  0.602   0.602    0.602
## 115     Zoo.Area.ha2  ~ Brillouin.Index2  6.203  0.169   0.169    0.170
## 116     Zoo.Area.ha2  ~ Mean.Raup.Crick2  7.812  0.124   0.124    0.124
## 124 Mam.Sp.Richness2  ~      Attendance2  7.485 -0.501  -0.501   -0.489
## 125 Mam.Sp.Richness2  ~   Total.Animals2  5.172 -0.226  -0.226   -0.227
```

```
## 126 Mam.Sp.Richness2  ~     Sp.Richness2  9.521 -0.742  -0.742   -0.742
## 128 Mam.Sp.Richness2  ~ Mean.Raup.Crick2  5.330 -0.133  -0.133   -0.133
## 148       X50km_Pop2  ~      Attendance2 30.406  0.562   0.562    0.548
## 149       X50km_Pop2  ~   Total.Animals2 29.464  0.281   0.281    0.282
## 152       X50km_Pop2  ~ Mean.Raup.Crick2  3.672  0.065   0.065    0.065
## 160       X10km_Pop2  ~      Attendance2  3.571 -0.167  -0.167   -0.163
## 161       X10km_Pop2  ~   Total.Animals2  3.040 -0.083  -0.083   -0.083
## 173    GDP.Millions2  ~   Total.Animals2  4.926  0.102   0.102    0.102
## 174    GDP.Millions2  ~     Sp.Richness2 30.346  0.338   0.338    0.338
## 175    GDP.Millions2  ~ Brillouin.Index2 18.587  0.173   0.173    0.173
## 176    GDP.Millions2  ~ Mean.Raup.Crick2 10.852 -0.091  -0.091   -0.091
## 185 Nat_Pop._WB20152  ~   Total.Animals2  3.943 -0.079  -0.079   -0.080
## 186 Nat_Pop._WB20152  ~     Sp.Richness2 31.565 -0.333  -0.333   -0.333
## 187 Nat_Pop._WB20152  ~ Brillouin.Index2 16.094 -0.139  -0.139   -0.139
## 188 Nat_Pop._WB20152  ~ Mean.Raup.Crick2 15.732  0.104   0.104    0.103
## 196     Prop.Mam.Sp2  ~      Attendance2  3.593 -0.150  -0.150   -0.147
## 199     Prop.Mam.Sp2  ~ Brillouin.Index2  3.002  0.105   0.105    0.105
## 200     Prop.Mam.Sp2  ~ Mean.Raup.Crick2 10.813 -0.141  -0.141   -0.141
##     sepc.nox
## 69    -0.066
## 70    -0.096
## 71     0.035
## 72    -0.087
## 76     0.079
## 77    -0.264
## 81     0.140
## 82     0.067
## 83     0.132
## 87     0.081
## 88    -0.085
## 93    -0.032
## 96     0.062
## 100   -0.059
## 102    0.077
## 103    0.376
## 105    0.155
## 107    0.153
## 108    0.188
## 110    0.165
## 111   -0.320
## 112    0.441
## 114    0.602
## 115    0.170
## 116    0.124
## 124   -0.489
## 125   -0.227
## 126   -0.742
## 128   -0.133
## 148    0.548
## 149    0.282
## 152    0.065
## 160   -0.163
## 161   -0.083
## 173    0.102
```

```
## 174    0.338
## 175    0.173
## 176   -0.091
## 185   -0.080
## 186   -0.333
## 187   -0.139
## 188    0.103
## 196   -0.147
## 199    0.105
## 200   -0.141
```

```r
# Adjust for the nested nature of the data (institutions within countries)
# Fit model and generate model summary
design <- svydesign(ids = ~Country, nest=TRUE, data=sem_attendance_data)
fit.adj12 <- lavaan.survey(lavaan.fit = mod.12.fit, survey.design = design)
summary(fit.adj12, rsq = TRUE)
```

```
## lavaan (0.5-23.1097) converged normally after  55 iterations
##
##   Number of observations                           458
##
##   Estimator                                         ML      Robust
##   Minimum Function Test Statistic              205.381      51.080
##   Degrees of freedom                                27          27
##   P-value (Chi-square)                           0.000       0.003
##   Scaling correction factor                                  4.021
##     for the Satorra-Bentler correction
##
## Parameter Estimates:
##
##   Information                                 Expected
##   Standard Errors                           Robust.sem
##
## Regressions:
##                    Estimate  Std.Err  z-value  P(>|z|)
##   Attendance2 ~
##     Zoo.Area.ha2      0.079    0.041    1.939    0.053
##     Sp.Richness2     -0.086    0.063   -1.374    0.169
##     Total.Animals2    0.407    0.061    6.707    0.000
##     Mam.Sp.Rchnss2    0.089    0.039    2.264    0.024
##     Men.Sp.BdyMss2    0.328    0.026   12.438    0.000
##     Mean.Rap.Crck2    0.153    0.029    5.202    0.000
##     X50km_Pop2        0.074    0.033    2.248    0.025
##     X10km_Pop2        0.406    0.041   10.000    0.000
##     GDP.Millions2     0.234    0.061    3.838    0.000
##     Nt_Pp._WB20152   -0.111    0.070   -1.595    0.111
##   Total.Animals2 ~
##     Zoo.Area.ha2      0.219    0.036    5.992    0.000
##     Sp.Richness2      0.805    0.051   15.917    0.000
##     GDP.Millions2    -0.129    0.072   -1.785    0.074
##   Sp.Richness2 ~
##     Zoo.Area.ha2      0.067    0.044    1.519    0.129
##     Prop.Mam.Sp2     -0.581    0.038  -15.231    0.000
##     Mam.Sp.Rchnss2    0.755    0.068   11.066    0.000
##   Brillouin.Index2 ~
```

```
##     Sp.Richness2        1.320    0.089   14.753    0.000
##     Total.Animals2     -0.620    0.127   -4.883    0.000
##     GDP.Millions2      -0.206    0.072   -2.846    0.004
##   Mean.Raup.Crick2 ~
##     Sp.Richness2       -0.355    0.230   -1.543    0.123
##     Total.Animals2      0.558    0.154    3.625    0.000
##     Men.Sp.BdyMss2     -0.495    0.052   -9.504    0.000
##
## Covariances:
##                        Estimate  Std.Err  z-value  P(>|z|)
##  .Attendance2 ~~
##    .Brilloun.Indx2        0.017    0.018    0.952    0.341
##   Zoo.Area.ha2 ~~
##     Mam.Sp.Rchnss2        0.381    0.064    5.985    0.000
##     Men.Sp.BdyMss2        0.534    0.104    5.147    0.000
##     X50km_Pop2            0.060    0.069    0.880    0.379
##     X10km_Pop2           -0.010    0.078   -0.124    0.901
##     GDP.Millions2        -0.027    0.041   -0.673    0.501
##     Nt_Pp._WB20152        0.061    0.090    0.682    0.495
##     Prop.Mam.Sp2          0.312    0.089    3.510    0.000
##   Mam.Sp.Richness2 ~~
##     Men.Sp.BdyMss2        0.303    0.081    3.730    0.000
##     X50km_Pop2            0.204    0.082    2.487    0.013
##     X10km_Pop2            0.284    0.066    4.278    0.000
##     GDP.Millions2        -0.060    0.064   -0.937    0.349
##     Nt_Pp._WB20152       -0.088    0.102   -0.863    0.388
##     Prop.Mam.Sp2          0.088    0.061    1.435    0.151
##   Mean.Sp.BodyMass2 ~~
##     X50km_Pop2            0.101    0.072    1.400    0.161
##     X10km_Pop2            0.126    0.067    1.895    0.058
##     GDP.Millions2        -0.082    0.046   -1.759    0.079
##     Nt_Pp._WB20152        0.006    0.092    0.063    0.950
##     Prop.Mam.Sp2          0.604    0.130    4.656    0.000
##   X50km_Pop2 ~~
##     X10km_Pop2            0.752    0.126    5.982    0.000
##     GDP.Millions2        -0.013    0.100   -0.131    0.896
##     Nt_Pp._WB20152        0.191    0.177    1.083    0.279
##     Prop.Mam.Sp2         -0.096    0.051   -1.888    0.059
##   X10km_Pop2 ~~
##     GDP.Millions2        -0.028    0.056   -0.505    0.613
##     Nt_Pp._WB20152        0.173    0.101    1.701    0.089
##     Prop.Mam.Sp2         -0.204    0.055   -3.685    0.000
##   GDP.Millions2 ~~
##     Nt_Pp._WB20152        0.826    0.202    4.089    0.000
##     Prop.Mam.Sp2         -0.123    0.105   -1.172    0.241
##   Nat_Pop._WB20152 ~~
##     Prop.Mam.Sp2         -0.115    0.091   -1.257    0.209
##
## Intercepts:
##                    Estimate  Std.Err  z-value  P(>|z|)
##    .Attendance2      -0.000    0.030   -0.000    1.000
##    .Total.Animals2   -0.000    0.070   -0.000    1.000
##    .Sp.Richness2      0.000    0.034    0.000    1.000
##    .Brilloun.Indx2   -0.000    0.055   -0.000    1.000
```

```
##     .Mean.Rap.Crck2    0.000    0.094    0.000    1.000
##      Zoo.Area.ha2      0.000    0.064    0.000    1.000
##      Mam.Sp.Rchnss2   -0.000    0.079   -0.000    1.000
##      Men.Sp.BdyMss2   -0.000    0.080   -0.000    1.000
##      X50km_Pop2       -0.000    0.114   -0.000    1.000
##      X10km_Pop2        0.000    0.105    0.000    1.000
##      GDP.Millions2    -0.000    0.345   -0.000    1.000
##      Nt_Pp._WB20152    0.000    0.295    0.000    1.000
##      Prop.Mam.Sp2      0.000    0.118    0.000    1.000
##
## Variances:
##                    Estimate  Std.Err  z-value  P(>|z|)
##     .Attendance2       0.269    0.027    9.996    0.000
##     .Total.Animals2    0.235    0.038    6.139    0.000
##     .Sp.Richness2      0.151    0.046    3.269    0.001
##     .Brilloun.Indx2    0.251    0.053    4.691    0.000
##     .Mean.Rap.Crck2    0.704    0.063   11.098    0.000
##      Zoo.Area.ha2      0.998    0.112    8.886    0.000
##      Mam.Sp.Rchnss2    0.998    0.115    8.669    0.000
##      Men.Sp.BdyMss2    0.998    0.173    5.783    0.000
##      X50km_Pop2        0.998    0.131    7.623    0.000
##      X10km_Pop2        0.998    0.174    5.745    0.000
##      GDP.Millions2     0.998    0.246    4.061    0.000
##      Nt_Pp._WB20152    0.998    0.225    4.439    0.000
##      Prop.Mam.Sp2      0.998    0.150    6.662    0.000
##
## R-Square:
##                    Estimate
##      Attendance2       0.717
##      Total.Animals2    0.766
##      Sp.Richness2      0.849
##      Brilloun.Indx2    0.750
##      Mean.Rap.Crck2    0.291
```

```
# Generate fit indices
fitMeasures(fit.adj12, c("agfi", "rmr", "srmr", "rmsea", "cfi", "nnfi", "tli"))
```

```
##  agfi   rmr  srmr rmsea   cfi  nnfi   tli
## 0.761 0.050 0.047 0.120 0.942 0.893 0.893
```

```
# Generate modification indices
mi12adj <- modindices(fit.adj12)
print(mi12adj[mi12adj$mi > 3.0,])
```

```
##                 lhs op              rhs     mi mi.scaled    epc sepc.lv
## 82    Total.Animals2 ~~ Brillouin.Index2  5.613     1.396 -0.066  -0.066
## 83    Total.Animals2 ~~ Mean.Raup.Crick2  4.260     1.059 -0.096  -0.096
## 84      Sp.Richness2 ~~ Brillouin.Index2 12.875     3.202  0.035   0.035
## 85      Sp.Richness2 ~~ Mean.Raup.Crick2 27.601     6.865 -0.087  -0.087
## 89    Total.Animals2  ~      Attendance2  3.623     0.901  0.082   0.082
## 90    Total.Animals2  ~ Brillouin.Index2  5.613     1.396 -0.264  -0.264
## 94    Total.Animals2  ~       X50km_Pop2 36.696     9.127  0.141   0.141
## 95    Total.Animals2  ~       X10km_Pop2  7.848     1.952  0.068   0.068
## 96    Total.Animals2  ~ Nat_Pop._WB20152 10.481     2.607  0.132   0.132
## 100     Sp.Richness2  ~ Brillouin.Index2  6.034     1.501  0.081   0.081
## 101     Sp.Richness2  ~ Mean.Raup.Crick2 17.310     4.305 -0.085  -0.085
```

```
## 106      Sp.Richness2  ~ Nat_Pop._WB20152  3.059     0.761 -0.032  -0.032
## 109 Brillouin.Index2  ~     Zoo.Area.ha2  5.613     1.396  0.062   0.062
## 113 Brillouin.Index2  ~       X10km_Pop2  5.633     1.401 -0.059  -0.059
## 115 Brillouin.Index2  ~     Prop.Mam.Sp2  7.793     1.938  0.077   0.077
## 116 Mean.Raup.Crick2  ~      Attendance2 17.519     4.357  0.384   0.384
## 118 Mean.Raup.Crick2  ~     Zoo.Area.ha2  9.137     2.272  0.154   0.154
## 120 Mean.Raup.Crick2  ~       X50km_Pop2 14.172     3.525  0.152   0.152
## 121 Mean.Raup.Crick2  ~       X10km_Pop2 19.556     4.864  0.187   0.187
## 123 Mean.Raup.Crick2  ~ Nat_Pop._WB20152 17.087     4.250  0.165   0.165
## 124 Mean.Raup.Crick2  ~     Prop.Mam.Sp2 28.743     7.149 -0.319  -0.319
## 125     Zoo.Area.ha2  ~      Attendance2  6.317     1.571  0.452   0.452
## 127     Zoo.Area.ha2  ~     Sp.Richness2  9.363     2.329  0.602   0.602
## 128     Zoo.Area.ha2  ~ Brillouin.Index2  6.203     1.543  0.169   0.169
## 129     Zoo.Area.ha2  ~ Mean.Raup.Crick2  7.812     1.943  0.124   0.124
## 137 Mam.Sp.Richness2  ~      Attendance2  7.485     1.862 -0.501  -0.501
## 138 Mam.Sp.Richness2  ~    Total.Animals2  5.172     1.286 -0.226  -0.226
## 139 Mam.Sp.Richness2  ~     Sp.Richness2  9.521     2.368 -0.742  -0.742
## 141 Mam.Sp.Richness2  ~ Mean.Raup.Crick2  5.330     1.326 -0.133  -0.133
## 161       X50km_Pop2  ~      Attendance2 30.406     7.562  0.562   0.562
## 162       X50km_Pop2  ~    Total.Animals2 29.464     7.328  0.281   0.281
## 165       X50km_Pop2  ~ Mean.Raup.Crick2  3.672     0.913  0.065   0.065
## 173       X10km_Pop2  ~      Attendance2  3.571     0.888 -0.167  -0.167
## 174       X10km_Pop2  ~    Total.Animals2  3.040     0.756 -0.083  -0.083
## 186     GDP.Millions2  ~    Total.Animals2  4.926     1.225  0.102   0.102
## 187     GDP.Millions2  ~     Sp.Richness2 30.346     7.547  0.338   0.338
## 188     GDP.Millions2  ~ Brillouin.Index2 18.587     4.623  0.173   0.173
## 189     GDP.Millions2  ~ Mean.Raup.Crick2 10.851     2.699 -0.091  -0.091
## 198 Nat_Pop._WB20152  ~    Total.Animals2  3.943     0.981 -0.079  -0.079
## 199 Nat_Pop._WB20152  ~     Sp.Richness2 31.565     7.850 -0.333  -0.333
## 200 Nat_Pop._WB20152  ~ Brillouin.Index2 16.094     4.003 -0.139  -0.139
## 201 Nat_Pop._WB20152  ~ Mean.Raup.Crick2 15.732     3.913  0.104   0.104
## 209     Prop.Mam.Sp2  ~      Attendance2  3.593     0.894 -0.150  -0.150
## 212     Prop.Mam.Sp2  ~ Brillouin.Index2  3.002     0.747  0.105   0.105
## 213     Prop.Mam.Sp2  ~ Mean.Raup.Crick2 10.813     2.689 -0.141  -0.141
##     sepc.all sepc.nox
## 82    -0.066   -0.066
## 83    -0.096   -0.096
## 84     0.035    0.035
## 85    -0.087   -0.087
## 89     0.079    0.079
## 90    -0.264   -0.264
## 94     0.140    0.140
## 95     0.067    0.067
## 96     0.132    0.132
## 100    0.081    0.081
## 101   -0.085   -0.085
## 106   -0.032   -0.032
## 109    0.062    0.062
## 113   -0.059   -0.059
## 115    0.077    0.077
## 116    0.376    0.376
## 118    0.155    0.155
## 120    0.153    0.153
## 121    0.187    0.188
```

```
## 123    0.165    0.165
## 124   -0.320   -0.320
## 125    0.441    0.441
## 127    0.602    0.602
## 128    0.170    0.170
## 129    0.124    0.124
## 137   -0.489   -0.489
## 138   -0.227   -0.227
## 139   -0.742   -0.742
## 141   -0.133   -0.133
## 161    0.548    0.548
## 162    0.282    0.282
## 165    0.065    0.065
## 173   -0.163   -0.163
## 174   -0.083   -0.083
## 186    0.102    0.102
## 187    0.338    0.338
## 188    0.173    0.173
## 189   -0.091   -0.091
## 198   -0.080   -0.080
## 199   -0.333   -0.333
## 200   -0.139   -0.139
## 201    0.103    0.103
## 209   -0.147   -0.147
## 212    0.105    0.105
## 213   -0.141   -0.141
```

```
# Test whether removal of pathway was justified
anova(fit.adj11, fit.adj12)
```

```
## Scaled Chi Square Difference Test (method = "satorra.bentler.2001")
##
##           Df   AIC   BIC  Chisq Chisq diff Df diff Pr(>Chisq)
## fit.adj11 26 12500 12822 203.89
## fit.adj12 27 12499 12817 205.38      1.848       1      0.174
```

```
# no significant difference, so link was not needed.
```

Model 13

Based on the results generated from the nested twelfth model, the highest p-value relationship to be considered for removal is **Attendance2** ~ **Sp.Richness2** with a p-value of **0.169**. Therefore we decide to remove this pathway. Once again, the model summary, fit indices and modification indices were all generated for the model, adjusting for the nested nature of data.

```
# Attendance SEM (Presence-Absence)

# Model 13
# Removal of Attendance2 ~ Sp.Richness2, p = 0.169

mod.13 <- 'Attendance2 ~ Zoo.Area.ha2 + Total.Animals2
+ Mam.Sp.Richness2 + Mean.Sp.BodyMass2 + Mean.Raup.Crick2
+ X50km_Pop2 + X10km_Pop2 + GDP.Millions2 + Nat_Pop._WB20152

Total.Animals2 ~ Zoo.Area.ha2 + Sp.Richness2 + GDP.Millions2
```

```r
Sp.Richness2 ~ Zoo.Area.ha2 + Prop.Mam.Sp2 + Mam.Sp.Richness2
Brillouin.Index2 ~ Sp.Richness2 + Total.Animals2 + GDP.Millions2
Mean.Raup.Crick2 ~ Sp.Richness2 + Total.Animals2 + Mean.Sp.BodyMass2'

# Fit model and generate model summary
mod.13.fit <- sem(mod.13, data = sem_attendance_data, fixed.x=FALSE)
summary(mod.13.fit, rsq = TRUE)
```

```
## lavaan (0.5-23.1097) converged normally after  48 iterations
##
##   Number of observations                           458
##
##   Estimator                                         ML
##   Minimum Function Test Statistic              207.279
##   Degrees of freedom                                28
##   P-value (Chi-square)                           0.000
##
## Parameter Estimates:
##
##   Information                                 Expected
##   Standard Errors                             Standard
##
## Regressions:
##                         Estimate  Std.Err  z-value  P(>|z|)
##   Attendance2 ~
##     Zoo.Area.ha2           0.084    0.033    2.547    0.011
##     Total.Animals2         0.353    0.038    9.410    0.000
##     Mam.Sp.Rchnss2         0.056    0.037    1.521    0.128
##     Men.Sp.BdyMss2         0.348    0.034   10.329    0.000
##     Mean.Rap.Crck2         0.158    0.028    5.545    0.000
##     X50km_Pop2             0.087    0.038    2.299    0.021
##     X10km_Pop2             0.389    0.040    9.727    0.000
##     GDP.Millions2          0.217    0.048    4.550    0.000
##     Nt_Pp._WB20152        -0.102    0.049   -2.074    0.038
##   Total.Animals2 ~
##     Zoo.Area.ha2           0.219    0.023    9.495    0.000
##     Sp.Richness2           0.805    0.023   34.969    0.000
##     GDP.Millions2         -0.129    0.023   -5.669    0.000
##   Sp.Richness2 ~
##     Zoo.Area.ha2           0.067    0.021    3.225    0.001
##     Prop.Mam.Sp2          -0.581    0.019  -30.364    0.000
##     Mam.Sp.Rchnss2         0.755    0.020   38.373    0.000
##   Brillouin.Index2 ~
##     Sp.Richness2           1.323    0.044   30.110    0.000
##     Total.Animals2        -0.623    0.044  -14.115    0.000
##     GDP.Millions2         -0.206    0.024   -8.533    0.000
##   Mean.Raup.Crick2 ~
##     Sp.Richness2          -0.355    0.074   -4.791    0.000
##     Total.Animals2         0.558    0.074    7.566    0.000
##     Men.Sp.BdyMss2        -0.495    0.041  -12.212    0.000
##
## Covariances:
##                           Estimate  Std.Err  z-value  P(>|z|)
##  .Attendance2 ~~
```

```
##    .Brilloun.Indx2        0.016    0.012    1.312    0.190
##   Zoo.Area.ha2 ~~
##     Mam.Sp.Rchnss2        0.381    0.050    7.634    0.000
##     Men.Sp.BdyMss2        0.534    0.053   10.096    0.000
##     X50km_Pop2            0.060    0.047    1.291    0.197
##     X10km_Pop2           -0.010    0.047   -0.208    0.835
##     GDP.Millions2        -0.027    0.047   -0.588    0.557
##     Nt_Pp._WB20152        0.061    0.047    1.310    0.190
##     Prop.Mam.Sp2          0.312    0.049    6.389    0.000
##   Mam.Sp.Richness2 ~~
##     Men.Sp.BdyMss2        0.303    0.049    6.216    0.000
##     X50km_Pop2            0.204    0.048    4.281    0.000
##     X10km_Pop2            0.284    0.048    5.852    0.000
##     GDP.Millions2        -0.060    0.047   -1.277    0.202
##     Nt_Pp._WB20152       -0.088    0.047   -1.881    0.060
##     Prop.Mam.Sp2          0.088    0.047    1.882    0.060
##   Mean.Sp.BodyMass2 ~~
##     X50km_Pop2            0.101    0.047    2.166    0.030
##     X10km_Pop2            0.126    0.047    2.683    0.007
##     GDP.Millions2        -0.082    0.047   -1.748    0.080
##     Nt_Pp._WB20152        0.006    0.047    0.124    0.901
##     Prop.Mam.Sp2          0.604    0.055   11.083    0.000
##   X50km_Pop2 ~~
##     X10km_Pop2            0.752    0.058   12.886    0.000
##     GDP.Millions2        -0.013    0.047   -0.281    0.779
##     Nt_Pp._WB20152        0.191    0.047    4.033    0.000
##     Prop.Mam.Sp2         -0.096    0.047   -2.054    0.040
##   X10km_Pop2 ~~
##     GDP.Millions2        -0.028    0.047   -0.604    0.546
##     Nt_Pp._WB20152        0.173    0.047    3.646    0.000
##     Prop.Mam.Sp2         -0.204    0.048   -4.293    0.000
##   GDP.Millions2 ~~
##     Nt_Pp._WB20152        0.826    0.061   13.642    0.000
##     Prop.Mam.Sp2         -0.123    0.047   -2.615    0.009
##   Nat_Pop._WB20152 ~~
##     Prop.Mam.Sp2         -0.115    0.047   -2.447    0.014
##
## Variances:
##                    Estimate  Std.Err  z-value  P(>|z|)
##    .Attendance2       0.270    0.018   15.133    0.000
##    .Total.Animals2    0.235    0.016   15.133    0.000
##    .Sp.Richness2      0.151    0.010   15.133    0.000
##    .Brilloun.Indx2    0.251    0.017   15.133    0.000
##    .Mean.Rap.Crck2    0.704    0.047   15.133    0.000
##     Zoo.Area.ha2      0.998    0.066   15.133    0.000
##     Mam.Sp.Rchnss2    0.998    0.066   15.133    0.000
##     Men.Sp.BdyMss2    0.998    0.066   15.133    0.000
##     X50km_Pop2        0.998    0.066   15.133    0.000
##     X10km_Pop2        0.998    0.066   15.133    0.000
##     GDP.Millions2     0.998    0.066   15.133    0.000
##     Nt_Pp._WB20152    0.998    0.066   15.133    0.000
##     Prop.Mam.Sp2      0.998    0.066   15.133    0.000
##
## R-Square:
```

```
##                     Estimate
##     Attendance2        0.716
##     Total.Animals2     0.766
##     Sp.Richness2       0.849
##     Brilloun.Indx2     0.750
##     Mean.Rap.Crck2     0.291
```

```r
# Generate fit indices
fitMeasures(mod.13.fit, c("agfi", "rmr", "srmr", "rmsea", "cfi", "nnfi", "tli"))
```

```
##  agfi   rmr  srmr rmsea   cfi  nnfi   tli
## 0.796 0.050 0.050 0.118 0.942 0.896 0.896
```

```r
# Generate modification indices
mi13 <- modindices(mod.13.fit)
print(mi13[mi13$mi > 3.0,])
```

```
##                  lhs op              rhs     mi    epc sepc.lv sepc.all
## 68    Total.Animals2 ~~ Brillouin.Index2  5.692 -0.067  -0.067   -0.066
## 69    Total.Animals2 ~~ Mean.Raup.Crick2  4.260 -0.096  -0.096   -0.096
## 70      Sp.Richness2 ~~ Brillouin.Index2 13.001  0.036   0.036    0.035
## 71      Sp.Richness2 ~~ Mean.Raup.Crick2 27.601 -0.087  -0.087   -0.087
## 76    Total.Animals2  ~      Attendance2  5.858  0.086   0.086    0.084
## 77    Total.Animals2  ~ Brillouin.Index2  5.030 -0.249  -0.249   -0.249
## 81    Total.Animals2  ~       X50km_Pop2 36.696  0.141   0.141    0.140
## 82    Total.Animals2  ~       X10km_Pop2  7.848  0.068   0.068    0.067
## 83    Total.Animals2  ~ Nat_Pop._WB20152 10.481  0.132   0.132    0.132
## 87      Sp.Richness2  ~ Brillouin.Index2  5.958  0.080   0.080    0.080
## 88      Sp.Richness2  ~ Mean.Raup.Crick2 17.310 -0.085  -0.085   -0.085
## 93      Sp.Richness2  ~ Nat_Pop._WB20152  3.059 -0.032  -0.032   -0.032
## 96  Brillouin.Index2  ~      Zoo.Area.ha2  5.692  0.062   0.062    0.062
## 100 Brillouin.Index2  ~       X10km_Pop2  5.665 -0.059  -0.059   -0.059
## 102 Brillouin.Index2  ~      Prop.Mam.Sp2  7.701  0.077   0.077    0.076
## 103 Mean.Raup.Crick2  ~      Attendance2 15.737  0.363   0.363    0.355
## 105 Mean.Raup.Crick2  ~      Zoo.Area.ha2  9.137  0.154   0.154    0.155
## 107 Mean.Raup.Crick2  ~       X50km_Pop2 14.172  0.152   0.152    0.153
## 108 Mean.Raup.Crick2  ~       X10km_Pop2 19.556  0.187   0.187    0.187
## 110 Mean.Raup.Crick2  ~ Nat_Pop._WB20152 17.087  0.165   0.165    0.165
## 111 Mean.Raup.Crick2  ~      Prop.Mam.Sp2 28.743 -0.319  -0.319   -0.320
## 112     Zoo.Area.ha2  ~      Attendance2  8.212  0.532   0.532    0.519
## 114     Zoo.Area.ha2  ~     Sp.Richness2  9.363  0.602   0.602    0.602
## 115     Zoo.Area.ha2  ~ Brillouin.Index2  6.264  0.170   0.170    0.171
## 116     Zoo.Area.ha2  ~ Mean.Raup.Crick2  7.812  0.124   0.124    0.124
## 124 Mam.Sp.Richness2  ~      Attendance2  8.265 -0.561  -0.561   -0.548
## 125 Mam.Sp.Richness2  ~   Total.Animals2  5.172 -0.226  -0.226   -0.227
## 126 Mam.Sp.Richness2  ~     Sp.Richness2  9.521 -0.742  -0.742   -0.742
## 128 Mam.Sp.Richness2  ~ Mean.Raup.Crick2  5.330 -0.133  -0.133   -0.133
## 148       X50km_Pop2  ~      Attendance2 25.964  0.537   0.537    0.524
## 149       X50km_Pop2  ~   Total.Animals2 29.464  0.281   0.281    0.282
## 152       X50km_Pop2  ~ Mean.Raup.Crick2  3.672  0.065   0.065    0.065
## 161       X10km_Pop2  ~   Total.Animals2  3.040 -0.083  -0.083   -0.083
## 173     GDP.Millions2  ~   Total.Animals2  4.926  0.102   0.102    0.102
## 174     GDP.Millions2  ~     Sp.Richness2 30.346  0.338   0.338    0.338
## 175     GDP.Millions2  ~ Brillouin.Index2 18.558  0.173   0.173    0.173
## 176     GDP.Millions2  ~ Mean.Raup.Crick2 10.852 -0.091  -0.091   -0.091
## 185 Nat_Pop._WB20152  ~   Total.Animals2  3.943 -0.079  -0.079   -0.080
```

```
## 186 Nat_Pop._WB20152  ~     Sp.Richness2 31.564 -0.333  -0.333   -0.333
## 187 Nat_Pop._WB20152  ~ Brillouin.Index2 16.062 -0.139  -0.139   -0.139
## 188 Nat_Pop._WB20152  ~ Mean.Raup.Crick2 15.732  0.104   0.104    0.103
## 199     Prop.Mam.Sp2  ~ Brillouin.Index2  3.043  0.105   0.105    0.106
## 200     Prop.Mam.Sp2  ~ Mean.Raup.Crick2 10.813 -0.141  -0.141   -0.141
##     sepc.nox
## 68    -0.066
## 69    -0.096
## 70     0.035
## 71    -0.087
## 76     0.084
## 77    -0.249
## 81     0.140
## 82     0.067
## 83     0.132
## 87     0.080
## 88    -0.085
## 93    -0.032
## 96     0.062
## 100   -0.059
## 102    0.077
## 103    0.355
## 105    0.155
## 107    0.153
## 108    0.188
## 110    0.165
## 111   -0.320
## 112    0.519
## 114    0.602
## 115    0.171
## 116    0.124
## 124   -0.548
## 125   -0.227
## 126   -0.742
## 128   -0.133
## 148    0.524
## 149    0.282
## 152    0.065
## 161   -0.083
## 173    0.102
## 174    0.338
## 175    0.173
## 176   -0.091
## 185   -0.080
## 186   -0.333
## 187   -0.139
## 188    0.103
## 199    0.106
## 200   -0.141
```

```r
# Adjust for the nested nature of the data (institutions within countries)
# Fit model and generate model summary
design <- svydesign(ids = ~Country, nest=TRUE, data=sem_attendance_data)
fit.adj13 <- lavaan.survey(lavaan.fit = mod.13.fit, survey.design = design)
```

```r
summary(fit.adj13, rsq = TRUE)
```

```
## lavaan (0.5-23.1097) converged normally after  51 iterations
##
##   Number of observations                           458
##
##   Estimator                                         ML      Robust
##   Minimum Function Test Statistic              207.279      52.844
##   Degrees of freedom                                28          28
##   P-value (Chi-square)                           0.000       0.003
##   Scaling correction factor                                  3.922
##     for the Satorra-Bentler correction
##
## Parameter Estimates:
##
##   Information                                 Expected
##   Standard Errors                           Robust.sem
##
## Regressions:
##                      Estimate  Std.Err  z-value  P(>|z|)
##   Attendance2 ~
##     Zoo.Area.ha2        0.084    0.038    2.194    0.028
##     Total.Animals2      0.353    0.037    9.571    0.000
##     Mam.Sp.Rchnss2      0.056    0.034    1.647    0.100
##     Men.Sp.BdyMss2      0.348    0.028   12.560    0.000
##     Mean.Rap.Crck2      0.158    0.028    5.711    0.000
##     X50km_Pop2          0.087    0.034    2.570    0.010
##     X10km_Pop2          0.389    0.040    9.665    0.000
##     GDP.Millions2       0.217    0.065    3.343    0.001
##     Nt_Pp._WB20152     -0.102    0.076   -1.334    0.182
##   Total.Animals2 ~
##     Zoo.Area.ha2        0.219    0.036    5.992    0.000
##     Sp.Richness2        0.805    0.051   15.917    0.000
##     GDP.Millions2      -0.129    0.072   -1.785    0.074
##   Sp.Richness2 ~
##     Zoo.Area.ha2        0.067    0.044    1.519    0.129
##     Prop.Mam.Sp2       -0.581    0.038  -15.231    0.000
##     Mam.Sp.Rchnss2      0.755    0.068   11.066    0.000
##   Brillouin.Index2 ~
##     Sp.Richness2        1.323    0.089   14.903    0.000
##     Total.Animals2     -0.623    0.126   -4.927    0.000
##     GDP.Millions2      -0.206    0.072   -2.848    0.004
##   Mean.Raup.Crick2 ~
##     Sp.Richness2       -0.355    0.230   -1.543    0.123
##     Total.Animals2      0.558    0.154    3.625    0.000
##     Men.Sp.BdyMss2     -0.495    0.052   -9.504    0.000
##
## Covariances:
##                        Estimate  Std.Err  z-value  P(>|z|)
##  .Attendance2 ~~
##    .Brilloun.Indx2        0.016    0.018    0.910    0.363
##   Zoo.Area.ha2 ~~
##     Mam.Sp.Rchnss2        0.381    0.064    5.985    0.000
##     Men.Sp.BdyMss2        0.534    0.104    5.147    0.000
```

```
##     X50km_Pop2          0.060    0.069    0.880    0.379
##     X10km_Pop2         -0.010    0.078   -0.124    0.901
##     GDP.Millions2      -0.027    0.041   -0.673    0.501
##     Nt_Pp._WB20152      0.061    0.090    0.682    0.495
##     Prop.Mam.Sp2        0.312    0.089    3.510    0.000
##   Mam.Sp.Richness2 ~~
##     Men.Sp.BdyMss2      0.303    0.081    3.730    0.000
##     X50km_Pop2          0.204    0.082    2.487    0.013
##     X10km_Pop2          0.284    0.066    4.278    0.000
##     GDP.Millions2      -0.060    0.064   -0.937    0.349
##     Nt_Pp._WB20152     -0.088    0.102   -0.863    0.388
##     Prop.Mam.Sp2        0.088    0.061    1.435    0.151
##   Mean.Sp.BodyMass2 ~~
##     X50km_Pop2          0.101    0.072    1.400    0.161
##     X10km_Pop2          0.126    0.067    1.895    0.058
##     GDP.Millions2      -0.082    0.046   -1.759    0.079
##     Nt_Pp._WB20152      0.006    0.092    0.063    0.950
##     Prop.Mam.Sp2        0.604    0.130    4.656    0.000
##   X50km_Pop2 ~~
##     X10km_Pop2          0.752    0.126    5.982    0.000
##     GDP.Millions2      -0.013    0.100   -0.131    0.896
##     Nt_Pp._WB20152      0.191    0.177    1.083    0.279
##     Prop.Mam.Sp2       -0.096    0.051   -1.888    0.059
##   X10km_Pop2 ~~
##     GDP.Millions2      -0.028    0.056   -0.505    0.613
##     Nt_Pp._WB20152      0.173    0.101    1.701    0.089
##     Prop.Mam.Sp2       -0.204    0.055   -3.685    0.000
##   GDP.Millions2 ~~
##     Nt_Pp._WB20152      0.826    0.202    4.089    0.000
##     Prop.Mam.Sp2       -0.123    0.105   -1.172    0.241
##   Nat_Pop._WB20152 ~~
##     Prop.Mam.Sp2       -0.115    0.091   -1.257    0.209
##
## Intercepts:
##                    Estimate  Std.Err  z-value  P(>|z|)
##    .Attendance2      -0.000    0.030   -0.000    1.000
##    .Total.Animals2   -0.000    0.070   -0.000    1.000
##    .Sp.Richness2      0.000    0.034    0.000    1.000
##    .Brilloun.Indx2   -0.000    0.055   -0.000    1.000
##    .Mean.Rap.Crck2    0.000    0.094    0.000    1.000
##     Zoo.Area.ha2      0.000    0.064    0.000    1.000
##     Mam.Sp.Rchnss2   -0.000    0.079   -0.000    1.000
##     Men.Sp.BdyMss2   -0.000    0.080   -0.000    1.000
##     X50km_Pop2       -0.000    0.114   -0.000    1.000
##     X10km_Pop2        0.000    0.105    0.000    1.000
##     GDP.Millions2    -0.000    0.345   -0.000    1.000
##     Nt_Pp._WB20152    0.000    0.295    0.000    1.000
##     Prop.Mam.Sp2      0.000    0.118    0.000    1.000
##
## Variances:
##                    Estimate  Std.Err  z-value  P(>|z|)
##    .Attendance2       0.270    0.028    9.694    0.000
##    .Total.Animals2    0.235    0.038    6.139    0.000
##    .Sp.Richness2      0.151    0.046    3.269    0.001
```

```
##    .Brilloun.Indx2    0.251    0.053    4.702    0.000
##    .Mean.Rap.Crck2    0.704    0.063   11.098    0.000
##     Zoo.Area.ha2      0.998    0.112    8.886    0.000
##     Mam.Sp.Rchnss2    0.998    0.115    8.669    0.000
##     Men.Sp.BdyMss2    0.998    0.173    5.783    0.000
##     X50km_Pop2        0.998    0.131    7.623    0.000
##     X10km_Pop2        0.998    0.174    5.745    0.000
##     GDP.Millions2     0.998    0.246    4.061    0.000
##     Nt_Pp._WB20152    0.998    0.225    4.439    0.000
##     Prop.Mam.Sp2      0.998    0.150    6.662    0.000
##
## R-Square:
##                    Estimate
##     Attendance2       0.716
##     Total.Animals2    0.766
##     Sp.Richness2      0.849
##     Brilloun.Indx2    0.750
##     Mean.Rap.Crck2    0.291
```

```r
# Generate fit indices
fitMeasures(fit.adj13, c("agfi", "rmr", "srmr", "rmsea", "cfi", "nnfi", "tli"))
```

```
##  agfi   rmr  srmr rmsea   cfi  nnfi   tli
## 0.767 0.050 0.047 0.118 0.942 0.896 0.896
```

```r
# Generate modification indices
mi13adj <- modindices(fit.adj13)
print(mi13adj[mi13adj$mi > 3.0,])
```

```
##                  lhs op              rhs     mi mi.scaled    epc sepc.lv
## 81    Total.Animals2 ~~ Brillouin.Index2  5.692     1.451 -0.067  -0.067
## 82    Total.Animals2 ~~ Mean.Raup.Crick2  4.260     1.086 -0.096  -0.096
## 83      Sp.Richness2 ~~ Brillouin.Index2 13.001     3.315  0.036   0.036
## 84      Sp.Richness2 ~~ Mean.Raup.Crick2 27.601     7.037 -0.087  -0.087
## 89    Total.Animals2  ~      Attendance2  5.858     1.493  0.086   0.086
## 90    Total.Animals2  ~ Brillouin.Index2  5.029     1.282 -0.249  -0.249
## 94    Total.Animals2  ~       X50km_Pop2 36.696     9.355  0.141   0.141
## 95    Total.Animals2  ~       X10km_Pop2  7.848     2.001  0.068   0.068
## 96    Total.Animals2  ~ Nat_Pop._WB20152 10.481     2.672  0.132   0.132
## 100     Sp.Richness2  ~ Brillouin.Index2  5.958     1.519  0.080   0.080
## 101     Sp.Richness2  ~ Mean.Raup.Crick2 17.310     4.413 -0.085  -0.085
## 106     Sp.Richness2  ~ Nat_Pop._WB20152  3.059     0.780 -0.032  -0.032
## 109 Brillouin.Index2  ~     Zoo.Area.ha2  5.692     1.451  0.062   0.062
## 113 Brillouin.Index2  ~       X10km_Pop2  5.665     1.444 -0.059  -0.059
## 115 Brillouin.Index2  ~     Prop.Mam.Sp2  7.702     1.963  0.077   0.077
## 116 Mean.Raup.Crick2  ~      Attendance2 15.737     4.012  0.363   0.363
## 118 Mean.Raup.Crick2  ~     Zoo.Area.ha2  9.137     2.329  0.154   0.154
## 120 Mean.Raup.Crick2  ~       X50km_Pop2 14.172     3.613  0.152   0.152
## 121 Mean.Raup.Crick2  ~       X10km_Pop2 19.556     4.986  0.187   0.187
## 123 Mean.Raup.Crick2  ~ Nat_Pop._WB20152 17.087     4.356  0.165   0.165
## 124 Mean.Raup.Crick2  ~     Prop.Mam.Sp2 28.743     7.328 -0.319  -0.319
## 125     Zoo.Area.ha2  ~      Attendance2  8.212     2.094  0.532   0.532
## 127     Zoo.Area.ha2  ~     Sp.Richness2  9.363     2.387  0.602   0.602
## 128     Zoo.Area.ha2  ~ Brillouin.Index2  6.265     1.597  0.170   0.170
## 129     Zoo.Area.ha2  ~ Mean.Raup.Crick2  7.812     1.992  0.124   0.124
## 137 Mam.Sp.Richness2  ~      Attendance2  8.265     2.107 -0.561  -0.561
```

```
## 138 Mam.Sp.Richness2  ~   Total.Animals2  5.172     1.319 -0.226  -0.226
## 139 Mam.Sp.Richness2  ~     Sp.Richness2  9.521     2.427 -0.742  -0.742
## 141 Mam.Sp.Richness2  ~ Mean.Raup.Crick2  5.330     1.359 -0.133  -0.133
## 161       X50km_Pop2  ~      Attendance2 25.964     6.619  0.537   0.537
## 162       X50km_Pop2  ~   Total.Animals2 29.464     7.512  0.281   0.281
## 165       X50km_Pop2  ~ Mean.Raup.Crick2  3.672     0.936  0.065   0.065
## 174       X10km_Pop2  ~   Total.Animals2  3.040     0.775 -0.083  -0.083
## 186    GDP.Millions2  ~   Total.Animals2  4.926     1.256  0.102   0.102
## 187    GDP.Millions2  ~     Sp.Richness2 30.346     7.737  0.338   0.338
## 188    GDP.Millions2  ~ Brillouin.Index2 18.559     4.731  0.173   0.173
## 189    GDP.Millions2  ~ Mean.Raup.Crick2 10.851     2.767 -0.091  -0.091
## 198 Nat_Pop._WB20152  ~   Total.Animals2  3.943     1.005 -0.079  -0.079
## 199 Nat_Pop._WB20152  ~     Sp.Richness2 31.565     8.047 -0.333  -0.333
## 200 Nat_Pop._WB20152  ~ Brillouin.Index2 16.062     4.095 -0.139  -0.139
## 201 Nat_Pop._WB20152  ~ Mean.Raup.Crick2 15.732     4.011  0.104   0.104
## 212      Prop.Mam.Sp2  ~ Brillouin.Index2  3.043     0.776  0.105   0.105
## 213      Prop.Mam.Sp2  ~ Mean.Raup.Crick2 10.813     2.757 -0.141  -0.141
##     sepc.all sepc.nox
## 81    -0.066   -0.066
## 82    -0.096   -0.096
## 83     0.035    0.035
## 84    -0.087   -0.087
## 89     0.084    0.084
## 90    -0.249   -0.249
## 94     0.140    0.140
## 95     0.067    0.067
## 96     0.132    0.132
## 100    0.080    0.080
## 101   -0.085   -0.085
## 106   -0.032   -0.032
## 109    0.062    0.062
## 113   -0.059   -0.059
## 115    0.076    0.077
## 116    0.355    0.355
## 118    0.155    0.155
## 120    0.153    0.153
## 121    0.187    0.188
## 123    0.165    0.165
## 124   -0.320   -0.320
## 125    0.519    0.519
## 127    0.602    0.602
## 128    0.171    0.171
## 129    0.124    0.124
## 137   -0.548   -0.548
## 138   -0.227   -0.227
## 139   -0.742   -0.742
## 141   -0.133   -0.133
## 161    0.524    0.524
## 162    0.282    0.282
## 165    0.065    0.065
## 174   -0.083   -0.083
## 186    0.102    0.102
## 187    0.338    0.338
## 188    0.173    0.173
```

```
## 189   -0.091   -0.091
## 198   -0.080   -0.080
## 199   -0.333   -0.333
## 200   -0.139   -0.139
## 201    0.103    0.103
## 212    0.106    0.106
## 213   -0.141   -0.141
```

```
# Test whether removal of pathway was justified
anova(fit.adj12, fit.adj13)
```

```
## Scaled Chi Square Difference Test (method = "satorra.bentler.2001")
##
##           Df   AIC   BIC  Chisq Chisq diff Df diff Pr(>Chisq)
## fit.adj12 27 12499 12817 205.38
## fit.adj13 28 12499 12813 207.28     1.4962       1     0.2213
```

```
# no significant difference, so link was not needed.
```

Model 14

Based on the results generated from the nested thirteenth model, the highest p-value relationship to be considered for removal is **Sp.Richness2 ~ Zoo.Area.ha2** with a p-value of **0.129**. Therefore we decide to remove this pathway. Once again, the model summary, fit indices and modification indices were all generated for the model, adjusting for the nested nature of data.

```
# Attendance SEM (Presence-Absence)

# Model 14
# Removal of Sp.Richness2 ~ Zoo.Area.ha2, p = 0.129

mod.14 <- 'Attendance2 ~ Zoo.Area.ha2 + Total.Animals2
+ Mam.Sp.Richness2 + Mean.Sp.BodyMass2 + Mean.Raup.Crick2
+ X50km_Pop2 + X10km_Pop2 + GDP.Millions2 + Nat_Pop._WB20152

Total.Animals2 ~ Zoo.Area.ha2 + Sp.Richness2 + GDP.Millions2
Sp.Richness2 ~ Prop.Mam.Sp2 + Mam.Sp.Richness2
Brillouin.Index2 ~ Sp.Richness2 + Total.Animals2 + GDP.Millions2
Mean.Raup.Crick2 ~ Sp.Richness2 + Total.Animals2 + Mean.Sp.BodyMass2'

# Fit model and generate model summary
mod.14.fit <- sem(mod.14, data = sem_attendance_data, fixed.x=FALSE)
summary(mod.14.fit, rsq = TRUE)
```

```
## lavaan (0.5-23.1097) converged normally after  49 iterations
##
##   Number of observations                           458
##
##   Estimator                                         ML
##   Minimum Function Test Statistic              217.562
##   Degrees of freedom                                29
##   P-value (Chi-square)                           0.000
##
## Parameter Estimates:
##
```

```
##   Information                                Expected
##   Standard Errors                            Standard
##
## Regressions:
##                     Estimate  Std.Err  z-value  P(>|z|)
##   Attendance2 ~
##     Zoo.Area.ha2       0.084    0.032    2.590    0.010
##     Total.Animals2     0.353    0.038    9.354    0.000
##     Mam.Sp.Rchnss2     0.056    0.037    1.500    0.134
##     Men.Sp.BdyMss2     0.348    0.034   10.355    0.000
##     Mean.Rap.Crck2     0.158    0.028    5.544    0.000
##     X50km_Pop2         0.087    0.038    2.300    0.021
##     X10km_Pop2         0.389    0.040    9.734    0.000
##     GDP.Millions2      0.217    0.048    4.550    0.000
##     Nt_Pp._WB20152    -0.102    0.049   -2.074    0.038
##   Total.Animals2 ~
##     Zoo.Area.ha2       0.219    0.023    9.570    0.000
##     Sp.Richness2       0.805    0.023   35.245    0.000
##     GDP.Millions2     -0.129    0.023   -5.670    0.000
##   Sp.Richness2 ~
##     Prop.Mam.Sp2      -0.563    0.018  -30.494    0.000
##     Mam.Sp.Rchnss2     0.779    0.018   42.208    0.000
##   Brillouin.Index2 ~
##     Sp.Richness2       1.323    0.043   30.429    0.000
##     Total.Animals2    -0.623    0.044  -14.133    0.000
##     GDP.Millions2     -0.206    0.024   -8.535    0.000
##   Mean.Raup.Crick2 ~
##     Sp.Richness2      -0.355    0.074   -4.829    0.000
##     Total.Animals2     0.558    0.074    7.575    0.000
##     Men.Sp.BdyMss2    -0.495    0.041  -12.194    0.000
##
## Covariances:
##                       Estimate  Std.Err  z-value  P(>|z|)
##  .Attendance2 ~~
##    .Brilloun.Indx2       0.016    0.012    1.312    0.190
##   Zoo.Area.ha2 ~~
##     Mam.Sp.Rchnss2       0.381    0.050    7.634    0.000
##     Men.Sp.BdyMss2       0.534    0.053   10.096    0.000
##     X50km_Pop2           0.060    0.047    1.291    0.197
##     X10km_Pop2          -0.010    0.047   -0.208    0.835
##     GDP.Millions2       -0.027    0.047   -0.588    0.557
##     Nt_Pp._WB20152       0.061    0.047    1.310    0.190
##     Prop.Mam.Sp2         0.312    0.049    6.389    0.000
##   Mam.Sp.Richness2 ~~
##     Men.Sp.BdyMss2       0.303    0.049    6.216    0.000
##     X50km_Pop2           0.204    0.048    4.281    0.000
##     X10km_Pop2           0.284    0.048    5.852    0.000
##     GDP.Millions2       -0.060    0.047   -1.277    0.202
##     Nt_Pp._WB20152      -0.088    0.047   -1.881    0.060
##     Prop.Mam.Sp2         0.088    0.047    1.882    0.060
##   Mean.Sp.BodyMass2 ~~
##     X50km_Pop2           0.101    0.047    2.166    0.030
##     X10km_Pop2           0.126    0.047    2.683    0.007
##     GDP.Millions2       -0.082    0.047   -1.748    0.080
```

```
##     Nt_Pp._WB20152        0.006    0.047    0.124    0.901
##     Prop.Mam.Sp2          0.604    0.055   11.083    0.000
##   X50km_Pop2 ~~
##     X10km_Pop2            0.752    0.058   12.886    0.000
##     GDP.Millions2        -0.013    0.047   -0.281    0.779
##     Nt_Pp._WB20152        0.191    0.047    4.033    0.000
##     Prop.Mam.Sp2         -0.096    0.047   -2.054    0.040
##   X10km_Pop2 ~~
##     GDP.Millions2        -0.028    0.047   -0.604    0.546
##     Nt_Pp._WB20152        0.173    0.047    3.646    0.000
##     Prop.Mam.Sp2         -0.204    0.048   -4.293    0.000
##   GDP.Millions2 ~~
##     Nt_Pp._WB20152        0.826    0.061   13.642    0.000
##     Prop.Mam.Sp2         -0.123    0.047   -2.615    0.009
##   Nat_Pop._WB20152 ~~
##     Prop.Mam.Sp2         -0.115    0.047   -2.447    0.014
##
## Variances:
##                    Estimate  Std.Err  z-value  P(>|z|)
##    .Attendance2       0.270    0.018   15.133    0.000
##    .Total.Animals2    0.235    0.016   15.133    0.000
##    .Sp.Richness2      0.154    0.010   15.133    0.000
##    .Brilloun.Indx2    0.251    0.017   15.133    0.000
##    .Mean.Rap.Crck2    0.704    0.047   15.133    0.000
##     Zoo.Area.ha2      0.998    0.066   15.133    0.000
##     Mam.Sp.Rchnss2    0.998    0.066   15.133    0.000
##     Men.Sp.BdyMss2    0.998    0.066   15.133    0.000
##     X50km_Pop2        0.998    0.066   15.133    0.000
##     X10km_Pop2        0.998    0.066   15.133    0.000
##     GDP.Millions2     0.998    0.066   15.133    0.000
##     Nt_Pp._WB20152    0.998    0.066   15.133    0.000
##     Prop.Mam.Sp2      0.998    0.066   15.133    0.000
##
## R-Square:
##                    Estimate
##     Attendance2       0.714
##     Total.Animals2    0.762
##     Sp.Richness2      0.845
##     Brilloun.Indx2    0.753
##     Mean.Rap.Crck2    0.291
```

```
# Generate fit indices
fitMeasures(mod.14.fit, c("agfi", "rmr", "srmr", "rmsea", "cfi", "nnfi", "tli"))
```

```
##  agfi   rmr  srmr rmsea   cfi  nnfi   tli
## 0.799 0.051 0.052 0.119 0.939 0.894 0.894
```

```
# Generate modification indices
mi14 <- modindices(mod.14.fit)
print(mi14[mi14$mi > 3.0,])
```

```
##                 lhs op              rhs     mi    epc sepc.lv sepc.all
## 67    Total.Animals2 ~~ Brillouin.Index2  5.618 -0.066  -0.066   -0.066
## 68    Total.Animals2 ~~ Mean.Raup.Crick2  4.206 -0.094  -0.094   -0.095
## 69      Sp.Richness2 ~~ Brillouin.Index2 14.829  0.038   0.038    0.038
## 70      Sp.Richness2 ~~ Mean.Raup.Crick2 20.844 -0.077  -0.077   -0.077
```

```
## 75    Total.Animals2  ~      Attendance2  5.899  0.087   0.087    0.085
## 76    Total.Animals2  ~ Brillouin.Index2  4.966 -0.246  -0.246   -0.250
## 80    Total.Animals2  ~       X50km_Pop2 36.704  0.141   0.141    0.142
## 81    Total.Animals2  ~       X10km_Pop2  7.850  0.068   0.068    0.068
## 82    Total.Animals2  ~ Nat_Pop._WB20152 10.486  0.132   0.132    0.133
## 86      Sp.Richness2  ~ Brillouin.Index2  3.381  0.060   0.060    0.060
## 87      Sp.Richness2  ~ Mean.Raup.Crick2 13.478 -0.076  -0.076   -0.076
## 88      Sp.Richness2  ~     Zoo.Area.ha2 10.169  0.067   0.067    0.067
## 96  Brillouin.Index2  ~     Zoo.Area.ha2  5.618  0.061   0.061    0.061
## 100 Brillouin.Index2  ~       X10km_Pop2  5.677 -0.059  -0.059   -0.059
## 102 Brillouin.Index2  ~     Prop.Mam.Sp2  7.668  0.076   0.076    0.076
## 103 Mean.Raup.Crick2  ~      Attendance2 15.708  0.362   0.362    0.353
## 105 Mean.Raup.Crick2  ~     Zoo.Area.ha2  8.934  0.151   0.151    0.151
## 107 Mean.Raup.Crick2  ~       X50km_Pop2 14.178  0.153   0.153    0.153
## 108 Mean.Raup.Crick2  ~       X10km_Pop2 19.643  0.188   0.188    0.188
## 110 Mean.Raup.Crick2  ~ Nat_Pop._WB20152 17.081  0.165   0.165    0.165
## 111 Mean.Raup.Crick2  ~     Prop.Mam.Sp2 28.146 -0.312  -0.312   -0.313
## 112     Zoo.Area.ha2  ~      Attendance2 16.211  0.660   0.660    0.642
## 113     Zoo.Area.ha2  ~   Total.Animals2  8.898  0.247   0.247    0.245
## 114     Zoo.Area.ha2  ~     Sp.Richness2 17.992  0.391   0.391    0.391
## 115     Zoo.Area.ha2  ~ Brillouin.Index2 13.742  0.222   0.222    0.224
## 116     Zoo.Area.ha2  ~ Mean.Raup.Crick2  8.530  0.130   0.130    0.130
## 124 Mam.Sp.Richness2  ~      Attendance2 12.233 -0.680  -0.680   -0.661
## 125 Mam.Sp.Richness2  ~   Total.Animals2  9.446 -0.299  -0.299   -0.297
## 126 Mam.Sp.Richness2  ~     Sp.Richness2 19.172 -0.809  -0.809   -0.809
## 128 Mam.Sp.Richness2  ~ Mean.Raup.Crick2  5.859 -0.143  -0.143   -0.142
## 148       X50km_Pop2  ~      Attendance2 25.848  0.534   0.534    0.520
## 149       X50km_Pop2  ~   Total.Animals2 29.269  0.280   0.280    0.278
## 152       X50km_Pop2  ~ Mean.Raup.Crick2  3.672  0.065   0.065    0.065
## 161       X10km_Pop2  ~   Total.Animals2  3.019 -0.082  -0.082   -0.082
## 173    GDP.Millions2  ~   Total.Animals2  4.885  0.101   0.101    0.100
## 174    GDP.Millions2  ~     Sp.Richness2 29.673  0.331   0.331    0.331
## 175    GDP.Millions2  ~ Brillouin.Index2 18.438  0.171   0.171    0.173
## 176    GDP.Millions2  ~ Mean.Raup.Crick2 10.851 -0.091  -0.091   -0.091
## 185 Nat_Pop._WB20152  ~   Total.Animals2  3.917 -0.079  -0.079   -0.078
## 186 Nat_Pop._WB20152  ~     Sp.Richness2 30.865 -0.326  -0.326   -0.326
## 187 Nat_Pop._WB20152  ~ Brillouin.Index2 15.979 -0.138  -0.138   -0.139
## 188 Nat_Pop._WB20152  ~ Mean.Raup.Crick2 15.732  0.104   0.104    0.103
## 200     Prop.Mam.Sp2  ~ Mean.Raup.Crick2 10.883 -0.141  -0.141   -0.140
##     sepc.nox
## 67    -0.066
## 68    -0.095
## 69     0.038
## 70    -0.077
## 75     0.085
## 76    -0.250
## 80     0.142
## 81     0.068
## 82     0.133
## 86     0.060
## 87    -0.076
## 88     0.067
## 96     0.061
## 100   -0.059
```

```
## 102    0.076
## 103    0.353
## 105    0.151
## 107    0.153
## 108    0.188
## 110    0.165
## 111   -0.314
## 112    0.642
## 113    0.245
## 114    0.391
## 115    0.224
## 116    0.130
## 124   -0.661
## 125   -0.297
## 126   -0.809
## 128   -0.142
## 148    0.520
## 149    0.278
## 152    0.065
## 161   -0.082
## 173    0.100
## 174    0.331
## 175    0.173
## 176   -0.091
## 185   -0.078
## 186   -0.326
## 187   -0.139
## 188    0.103
## 200   -0.140
```

```
# Adjust for the nested nature of the data (institutions within countries)
# Fit model and generate model summary
design <- svydesign(ids = ~Country, nest=TRUE, data=sem_attendance_data)
fit.adj14 <- lavaan.survey(lavaan.fit = mod.14.fit, survey.design = design)
summary(fit.adj14, rsq = TRUE)
```

```
## lavaan (0.5-23.1097) converged normally after  51 iterations
##
##   Number of observations                           458
##
##   Estimator                                         ML      Robust
##   Minimum Function Test Statistic              217.562      59.253
##   Degrees of freedom                                29          29
##   P-value (Chi-square)                           0.000       0.001
##   Scaling correction factor                                  3.672
##     for the Satorra-Bentler correction
##
## Parameter Estimates:
##
##   Information                                 Expected
##   Standard Errors                           Robust.sem
##
## Regressions:
##                      Estimate  Std.Err  z-value  P(>|z|)
##   Attendance2 ~
```

```
##     Zoo.Area.ha2        0.084    0.037    2.280    0.023
##     Total.Animals2      0.353    0.037    9.642    0.000
##     Mam.Sp.Rchnss2      0.056    0.035    1.615    0.106
##     Men.Sp.BdyMss2      0.348    0.028   12.614    0.000
##     Mean.Rap.Crck2      0.158    0.028    5.712    0.000
##     X50km_Pop2          0.087    0.034    2.570    0.010
##     X10km_Pop2          0.389    0.040    9.652    0.000
##     GDP.Millions2       0.217    0.065    3.344    0.001
##     Nt_Pp._WB20152     -0.102    0.076   -1.334    0.182
##   Total.Animals2 ~
##     Zoo.Area.ha2        0.219    0.034    6.384    0.000
##     Sp.Richness2        0.805    0.049   16.513    0.000
##     GDP.Millions2      -0.129    0.072   -1.786    0.074
##   Sp.Richness2 ~
##     Prop.Mam.Sp2       -0.563    0.041  -13.858    0.000
##     Mam.Sp.Rchnss2      0.779    0.057   13.593    0.000
##   Brillouin.Index2 ~
##     Sp.Richness2        1.323    0.087   15.119    0.000
##     Total.Animals2     -0.623    0.127   -4.909    0.000
##     GDP.Millions2      -0.206    0.073   -2.846    0.004
##   Mean.Raup.Crick2 ~
##     Sp.Richness2       -0.355    0.225   -1.576    0.115
##     Total.Animals2      0.558    0.151    3.706    0.000
##     Men.Sp.BdyMss2     -0.495    0.052   -9.545    0.000
##
## Covariances:
##                        Estimate  Std.Err  z-value  P(>|z|)
##  .Attendance2 ~~
##    .Brilloun.Indx2        0.016    0.018    0.910    0.363
##   Zoo.Area.ha2 ~~
##     Mam.Sp.Rchnss2        0.381    0.064    5.985    0.000
##     Men.Sp.BdyMss2        0.534    0.104    5.147    0.000
##     X50km_Pop2            0.060    0.069    0.880    0.379
##     X10km_Pop2           -0.010    0.078   -0.124    0.901
##     GDP.Millions2        -0.027    0.041   -0.673    0.501
##     Nt_Pp._WB20152        0.061    0.090    0.682    0.495
##     Prop.Mam.Sp2          0.312    0.089    3.510    0.000
##   Mam.Sp.Richness2 ~~
##     Men.Sp.BdyMss2        0.303    0.081    3.730    0.000
##     X50km_Pop2            0.204    0.082    2.487    0.013
##     X10km_Pop2            0.284    0.066    4.278    0.000
##     GDP.Millions2        -0.060    0.064   -0.937    0.349
##     Nt_Pp._WB20152       -0.088    0.102   -0.863    0.388
##     Prop.Mam.Sp2          0.088    0.061    1.435    0.151
##   Mean.Sp.BodyMass2 ~~
##     X50km_Pop2            0.101    0.072    1.400    0.161
##     X10km_Pop2            0.126    0.067    1.895    0.058
##     GDP.Millions2        -0.082    0.046   -1.759    0.079
##     Nt_Pp._WB20152        0.006    0.092    0.063    0.950
##     Prop.Mam.Sp2          0.604    0.130    4.656    0.000
##   X50km_Pop2 ~~
##     X10km_Pop2            0.752    0.126    5.982    0.000
##     GDP.Millions2        -0.013    0.100   -0.131    0.896
##     Nt_Pp._WB20152        0.191    0.177    1.083    0.279
```

```
##     Prop.Mam.Sp2        -0.096    0.051   -1.888    0.059
##   X10km_Pop2 ~~
##     GDP.Millions2       -0.028    0.056   -0.505    0.613
##     Nt_Pp._WB20152       0.173    0.101    1.701    0.089
##     Prop.Mam.Sp2        -0.204    0.055   -3.685    0.000
##   GDP.Millions2 ~~
##     Nt_Pp._WB20152       0.826    0.202    4.089    0.000
##     Prop.Mam.Sp2        -0.123    0.105   -1.172    0.241
##   Nat_Pop._WB20152 ~~
##     Prop.Mam.Sp2        -0.115    0.091   -1.257    0.209
##
## Intercepts:
##                    Estimate  Std.Err  z-value  P(>|z|)
##    .Attendance2      -0.000    0.030   -0.000    1.000
##    .Total.Animals2   -0.000    0.070   -0.000    1.000
##    .Sp.Richness2      0.000    0.032    0.000    1.000
##    .Brilloun.Indx2   -0.000    0.055   -0.000    1.000
##    .Mean.Rap.Crck2    0.000    0.094    0.000    1.000
##     Zoo.Area.ha2      0.000    0.064    0.000    1.000
##     Mam.Sp.Rchnss2   -0.000    0.079   -0.000    1.000
##     Men.Sp.BdyMss2   -0.000    0.080   -0.000    1.000
##     X50km_Pop2       -0.000    0.114   -0.000    1.000
##     X10km_Pop2        0.000    0.105    0.000    1.000
##     GDP.Millions2    -0.000    0.345   -0.000    1.000
##     Nt_Pp._WB20152    0.000    0.295    0.000    1.000
##     Prop.Mam.Sp2      0.000    0.118    0.000    1.000
##
## Variances:
##                    Estimate  Std.Err  z-value  P(>|z|)
##    .Attendance2       0.270    0.028    9.694    0.000
##    .Total.Animals2    0.235    0.038    6.139    0.000
##    .Sp.Richness2      0.154    0.044    3.506    0.000
##    .Brilloun.Indx2    0.251    0.053    4.702    0.000
##    .Mean.Rap.Crck2    0.704    0.063   11.098    0.000
##     Zoo.Area.ha2      0.998    0.112    8.886    0.000
##     Mam.Sp.Rchnss2    0.998    0.115    8.669    0.000
##     Men.Sp.BdyMss2    0.998    0.173    5.783    0.000
##     X50km_Pop2        0.998    0.131    7.623    0.000
##     X10km_Pop2        0.998    0.174    5.745    0.000
##     GDP.Millions2     0.998    0.246    4.061    0.000
##     Nt_Pp._WB20152    0.998    0.225    4.439    0.000
##     Prop.Mam.Sp2      0.998    0.150    6.662    0.000
##
## R-Square:
##                    Estimate
##     Attendance2       0.714
##     Total.Animals2    0.762
##     Sp.Richness2      0.845
##     Brilloun.Indx2    0.753
##     Mean.Rap.Crck2    0.291
```

```
# Generate fit indices
fitMeasures(fit.adj14, c("agfi", "rmr", "srmr", "rmsea", "cfi", "nnfi", "tli"))
```

```
##  agfi   rmr  srmr rmsea   cfi  nnfi   tli
```

```
## 0.770 0.051 0.048 0.119 0.939 0.894 0.894
```

```r
# Generate modification indices
mi14adj <- modindices(fit.adj14)
print(mi14adj[mi14adj$mi > 3.0,])
```

```
##                  lhs op              rhs     mi mi.scaled    epc sepc.lv
## 80    Total.Animals2 ~~ Brillouin.Index2  5.618     1.530 -0.066  -0.066
## 81    Total.Animals2 ~~ Mean.Raup.Crick2  4.206     1.145 -0.094  -0.094
## 82      Sp.Richness2 ~~ Brillouin.Index2 14.829     4.039  0.038   0.038
## 83      Sp.Richness2 ~~ Mean.Raup.Crick2 20.844     5.677 -0.077  -0.077
## 88    Total.Animals2  ~      Attendance2  5.899     1.607  0.087   0.087
## 89    Total.Animals2  ~ Brillouin.Index2  4.966     1.353 -0.246  -0.246
## 93    Total.Animals2  ~       X50km_Pop2 36.704     9.996  0.141   0.141
## 94    Total.Animals2  ~       X10km_Pop2  7.850     2.138  0.068   0.068
## 95    Total.Animals2  ~ Nat_Pop._WB20152 10.486     2.856  0.132   0.132
## 99      Sp.Richness2  ~ Brillouin.Index2  3.381     0.921  0.060   0.060
## 100     Sp.Richness2  ~ Mean.Raup.Crick2 13.478     3.671 -0.076  -0.076
## 101     Sp.Richness2  ~      Zoo.Area.ha2 10.169     2.769  0.067   0.067
## 109 Brillouin.Index2  ~      Zoo.Area.ha2  5.618     1.530  0.061   0.061
## 113 Brillouin.Index2  ~       X10km_Pop2  5.677     1.546 -0.059  -0.059
## 115 Brillouin.Index2  ~      Prop.Mam.Sp2  7.668     2.088  0.076   0.076
## 116 Mean.Raup.Crick2  ~      Attendance2 15.708     4.278  0.362   0.362
## 118 Mean.Raup.Crick2  ~      Zoo.Area.ha2  8.934     2.433  0.151   0.151
## 120 Mean.Raup.Crick2  ~       X50km_Pop2 14.178     3.861  0.153   0.153
## 121 Mean.Raup.Crick2  ~       X10km_Pop2 19.643     5.350  0.188   0.188
## 123 Mean.Raup.Crick2  ~ Nat_Pop._WB20152 17.081     4.652  0.165   0.165
## 124 Mean.Raup.Crick2  ~      Prop.Mam.Sp2 28.146     7.666 -0.312  -0.312
## 125     Zoo.Area.ha2  ~      Attendance2 16.210     4.415  0.660   0.660
## 126     Zoo.Area.ha2  ~   Total.Animals2  8.897     2.423  0.247   0.247
## 127     Zoo.Area.ha2  ~     Sp.Richness2 17.991     4.900  0.391   0.391
## 128     Zoo.Area.ha2  ~ Brillouin.Index2 13.742     3.743  0.222   0.222
## 129     Zoo.Area.ha2  ~ Mean.Raup.Crick2  8.530     2.323  0.130   0.130
## 137 Mam.Sp.Richness2  ~      Attendance2 12.234     3.332 -0.680  -0.680
## 138 Mam.Sp.Richness2  ~   Total.Animals2  9.446     2.573 -0.299  -0.299
## 139 Mam.Sp.Richness2  ~     Sp.Richness2 19.172     5.222 -0.809  -0.809
## 141 Mam.Sp.Richness2  ~ Mean.Raup.Crick2  5.859     1.596 -0.143  -0.143
## 161       X50km_Pop2  ~      Attendance2 25.848     7.040  0.534   0.534
## 162       X50km_Pop2  ~   Total.Animals2 29.269     7.971  0.280   0.280
## 165       X50km_Pop2  ~ Mean.Raup.Crick2  3.672     1.000  0.065   0.065
## 174       X10km_Pop2  ~   Total.Animals2  3.020     0.822 -0.082  -0.082
## 186     GDP.Millions2  ~   Total.Animals2  4.885     1.331  0.101   0.101
## 187     GDP.Millions2  ~     Sp.Richness2 29.672     8.081  0.331   0.331
## 188     GDP.Millions2  ~ Brillouin.Index2 18.437     5.021  0.171   0.171
## 189     GDP.Millions2  ~ Mean.Raup.Crick2 10.851     2.955 -0.091  -0.091
## 198 Nat_Pop._WB20152  ~   Total.Animals2  3.917     1.067 -0.079  -0.079
## 199 Nat_Pop._WB20152  ~     Sp.Richness2 30.864     8.406 -0.326  -0.326
## 200 Nat_Pop._WB20152  ~ Brillouin.Index2 15.978     4.352 -0.138  -0.138
## 201 Nat_Pop._WB20152  ~ Mean.Raup.Crick2 15.732     4.285  0.104   0.104
## 213      Prop.Mam.Sp2  ~ Mean.Raup.Crick2 10.883     2.964 -0.141  -0.141
##     sepc.all sepc.nox
## 80    -0.066   -0.066
## 81    -0.095   -0.095
## 82     0.038    0.038
## 83    -0.077   -0.077
```

```
## 88      0.085    0.085
## 89     -0.250   -0.250
## 93      0.142    0.142
## 94      0.068    0.068
## 95      0.133    0.133
## 99      0.060    0.060
## 100    -0.076   -0.076
## 101     0.067    0.067
## 109     0.061    0.061
## 113    -0.059   -0.059
## 115     0.076    0.076
## 116     0.353    0.353
## 118     0.151    0.151
## 120     0.153    0.153
## 121     0.188    0.188
## 123     0.165    0.165
## 124    -0.313   -0.314
## 125     0.642    0.642
## 126     0.245    0.245
## 127     0.391    0.391
## 128     0.224    0.224
## 129     0.130    0.130
## 137    -0.661   -0.661
## 138    -0.297   -0.297
## 139    -0.809   -0.809
## 141    -0.142   -0.142
## 161     0.520    0.520
## 162     0.278    0.278
## 165     0.065    0.065
## 174    -0.082   -0.082
## 186     0.100    0.100
## 187     0.331    0.331
## 188     0.173    0.173
## 189    -0.091   -0.091
## 198    -0.078   -0.078
## 199    -0.326   -0.326
## 200    -0.139   -0.139
## 201     0.103    0.103
## 213    -0.140   -0.140
```

Model 15

Based on the results generated from the nested fourteenth model, the highest p-value relationship to be considered for removal is **Mean.Raup.Crick2 ~ Sp.Richness2** with a p-value of **0.115**. Therefore we decide to remove this pathway. Once again, the model summary, fit indices and modification indices were all generated for the model, adjusting for the nested nature of data.

```
# Attendance SEM (Presence-Absence)

# Model 15
# Removal of Mean.Raup.Crick2 ~ Sp.Richness2 , p = 0.115

mod.15 <- 'Attendance2 ~ Zoo.Area.ha2 + Total.Animals2
```

```
+ Mam.Sp.Richness2 + Mean.Sp.BodyMass2 + Mean.Raup.Crick2
+ X50km_Pop2 + X10km_Pop2 + GDP.Millions2 + Nat_Pop._WB20152

Total.Animals2 ~ Zoo.Area.ha2 + Sp.Richness2 + GDP.Millions2
Sp.Richness2 ~ Prop.Mam.Sp2 + Mam.Sp.Richness2
Brillouin.Index2 ~ Sp.Richness2 + Total.Animals2 + GDP.Millions2
Mean.Raup.Crick2 ~ Total.Animals2 + Mean.Sp.BodyMass2'

# Fit model and generate model summary
mod.15.fit <- sem(mod.15, data = sem_attendance_data, fixed.x=FALSE)
summary(mod.15.fit, rsq = TRUE)
```

```
## lavaan (0.5-23.1097) converged normally after  41 iterations
##
##   Number of observations                           458
##
##   Estimator                                         ML
##   Minimum Function Test Statistic              240.130
##   Degrees of freedom                                30
##   P-value (Chi-square)                           0.000
##
## Parameter Estimates:
##
##   Information                                 Expected
##   Standard Errors                             Standard
##
## Regressions:
##                      Estimate  Std.Err  z-value  P(>|z|)
##   Attendance2 ~
##     Zoo.Area.ha2        0.084    0.032    2.592    0.010
##     Total.Animals2      0.353    0.037    9.507    0.000
##     Mam.Sp.Rchnss2      0.056    0.037    1.510    0.131
##     Men.Sp.BdyMss2      0.348    0.034   10.296    0.000
##     Mean.Rap.Crck2      0.158    0.028    5.597    0.000
##     X50km_Pop2          0.087    0.038    2.300    0.021
##     X10km_Pop2          0.389    0.040    9.738    0.000
##     GDP.Millions2       0.217    0.048    4.551    0.000
##     Nt_Pp._WB20152     -0.102    0.049   -2.074    0.038
##   Total.Animals2 ~
##     Zoo.Area.ha2        0.219    0.023    9.570    0.000
##     Sp.Richness2        0.805    0.023   35.245    0.000
##     GDP.Millions2      -0.129    0.023   -5.670    0.000
##   Sp.Richness2 ~
##     Prop.Mam.Sp2       -0.563    0.018  -30.494    0.000
##     Mam.Sp.Rchnss2      0.779    0.018   42.208    0.000
##   Brillouin.Index2 ~
##     Sp.Richness2        1.323    0.043   30.430    0.000
##     Total.Animals2     -0.623    0.044  -14.133    0.000
##     GDP.Millions2      -0.206    0.024   -8.535    0.000
##   Mean.Raup.Crick2 ~
##     Total.Animals2      0.259    0.041    6.400    0.000
##     Men.Sp.BdyMss2     -0.448    0.040  -11.127    0.000
##
## Covariances:
```

```
##                        Estimate  Std.Err  z-value  P(>|z|)
##   .Attendance2 ~~
##    .Brilloun.Indx2        0.016    0.012    1.312    0.190
##   Zoo.Area.ha2 ~~
##     Mam.Sp.Rchnss2        0.381    0.050    7.634    0.000
##     Men.Sp.BdyMss2        0.534    0.053   10.096    0.000
##     X50km_Pop2            0.060    0.047    1.291    0.197
##     X10km_Pop2           -0.010    0.047   -0.208    0.835
##     GDP.Millions2        -0.027    0.047   -0.588    0.557
##     Nt_Pp._WB20152        0.061    0.047    1.310    0.190
##     Prop.Mam.Sp2          0.312    0.049    6.389    0.000
##   Mam.Sp.Richness2 ~~
##     Men.Sp.BdyMss2        0.303    0.049    6.216    0.000
##     X50km_Pop2            0.204    0.048    4.281    0.000
##     X10km_Pop2            0.284    0.048    5.852    0.000
##     GDP.Millions2        -0.060    0.047   -1.277    0.202
##     Nt_Pp._WB20152       -0.088    0.047   -1.881    0.060
##     Prop.Mam.Sp2          0.088    0.047    1.882    0.060
##   Mean.Sp.BodyMass2 ~~
##     X50km_Pop2            0.101    0.047    2.166    0.030
##     X10km_Pop2            0.126    0.047    2.683    0.007
##     GDP.Millions2        -0.082    0.047   -1.748    0.080
##     Nt_Pp._WB20152        0.006    0.047    0.124    0.901
##     Prop.Mam.Sp2          0.604    0.055   11.083    0.000
##   X50km_Pop2 ~~
##     X10km_Pop2            0.752    0.058   12.886    0.000
##     GDP.Millions2        -0.013    0.047   -0.281    0.779
##     Nt_Pp._WB20152        0.191    0.047    4.033    0.000
##     Prop.Mam.Sp2         -0.096    0.047   -2.054    0.040
##   X10km_Pop2 ~~
##     GDP.Millions2        -0.028    0.047   -0.604    0.546
##     Nt_Pp._WB20152        0.173    0.047    3.646    0.000
##     Prop.Mam.Sp2         -0.204    0.048   -4.293    0.000
##   GDP.Millions2 ~~
##     Nt_Pp._WB20152        0.826    0.061   13.642    0.000
##     Prop.Mam.Sp2         -0.123    0.047   -2.615    0.009
##   Nat_Pop._WB20152 ~~
##     Prop.Mam.Sp2         -0.115    0.047   -2.447    0.014
##
## Variances:
##                    Estimate  Std.Err  z-value  P(>|z|)
##    .Attendance2       0.270    0.018   15.133    0.000
##    .Total.Animals2    0.235    0.016   15.133    0.000
##    .Sp.Richness2      0.154    0.010   15.133    0.000
##    .Brilloun.Indx2    0.251    0.017   15.133    0.000
##    .Mean.Rap.Crck2    0.739    0.049   15.133    0.000
##     Zoo.Area.ha2      0.998    0.066   15.133    0.000
##     Mam.Sp.Rchnss2    0.998    0.066   15.133    0.000
##     Men.Sp.BdyMss2    0.998    0.066   15.133    0.000
##     X50km_Pop2        0.998    0.066   15.133    0.000
##     X10km_Pop2        0.998    0.066   15.133    0.000
##     GDP.Millions2     0.998    0.066   15.133    0.000
##     Nt_Pp._WB20152    0.998    0.066   15.133    0.000
##     Prop.Mam.Sp2      0.998    0.066   15.133    0.000
```

```
##
## R-Square:
##                    Estimate
##     Attendance2       0.716
##     Total.Animals2    0.762
##     Sp.Richness2      0.845
##     Brilloun.Indx2    0.753
##     Mean.Rap.Crck2    0.257
```

```r
# Generate fit indices
fitMeasures(mod.15.fit, c("agfi", "rmr", "srmr", "rmsea", "cfi", "nnfi", "tli"))
```

```
##  agfi   rmr  srmr rmsea   cfi  nnfi   tli
## 0.781 0.051 0.051 0.124 0.932 0.886 0.886
```

```r
# Generate modification indices
mi15 <- modindices(mod.15.fit)
print(mi15[mi15$mi > 3.0,])
```

```
##                   lhs op              rhs     mi    epc sepc.lv sepc.all
## 66    Total.Animals2 ~~ Brillouin.Index2  5.618 -0.066  -0.066   -0.066
## 67    Total.Animals2 ~~ Mean.Raup.Crick2 10.289  0.072   0.072    0.072
## 68      Sp.Richness2 ~~ Brillouin.Index2 14.829  0.038   0.038    0.038
## 69      Sp.Richness2 ~~ Mean.Raup.Crick2 27.799 -0.088  -0.088   -0.088
## 74    Total.Animals2  ~      Attendance2  9.795  0.110   0.110    0.108
## 75    Total.Animals2  ~ Brillouin.Index2  4.965 -0.246  -0.246   -0.250
## 76    Total.Animals2  ~ Mean.Raup.Crick2  9.331  0.083   0.083    0.083
## 79    Total.Animals2  ~       X50km_Pop2 36.704  0.141   0.141    0.142
## 80    Total.Animals2  ~       X10km_Pop2  7.850  0.068   0.068    0.068
## 81    Total.Animals2  ~ Nat_Pop._WB20152 10.486  0.132   0.132    0.133
## 85      Sp.Richness2  ~ Brillouin.Index2  3.381  0.060   0.060    0.060
## 86      Sp.Richness2  ~ Mean.Raup.Crick2 22.606 -0.099  -0.099   -0.099
## 87      Sp.Richness2  ~      Zoo.Area.ha2 10.169  0.067   0.067    0.067
## 95  Brillouin.Index2  ~      Zoo.Area.ha2  5.618  0.061   0.061    0.061
## 99  Brillouin.Index2  ~       X10km_Pop2  5.677 -0.059  -0.059   -0.059
## 101 Brillouin.Index2  ~      Prop.Mam.Sp2  7.668  0.076   0.076    0.076
## 102 Mean.Raup.Crick2  ~      Attendance2  8.705  0.268   0.268    0.262
## 103 Mean.Raup.Crick2  ~     Sp.Richness2 21.846 -0.352  -0.352   -0.353
## 104 Mean.Raup.Crick2  ~ Brillouin.Index2 16.526 -0.191  -0.191   -0.193
## 105 Mean.Raup.Crick2  ~      Zoo.Area.ha2 12.524  0.180   0.180    0.180
## 106 Mean.Raup.Crick2  ~ Mam.Sp.Richness2 11.750 -0.203  -0.203   -0.204
## 107 Mean.Raup.Crick2  ~       X50km_Pop2 16.155  0.165   0.165    0.166
## 108 Mean.Raup.Crick2  ~       X10km_Pop2 12.518  0.149   0.149    0.149
## 110 Mean.Raup.Crick2  ~ Nat_Pop._WB20152 13.450  0.148   0.148    0.148
## 111 Mean.Raup.Crick2  ~      Prop.Mam.Sp2 10.037 -0.177  -0.177   -0.178
## 112     Zoo.Area.ha2  ~      Attendance2 22.932  0.783   0.783    0.765
## 113     Zoo.Area.ha2  ~   Total.Animals2  8.897  0.247   0.247    0.245
## 114     Zoo.Area.ha2  ~     Sp.Richness2 17.992  0.391   0.391    0.391
## 115     Zoo.Area.ha2  ~ Brillouin.Index2 13.742  0.222   0.222    0.224
## 116     Zoo.Area.ha2  ~ Mean.Raup.Crick2 16.392  0.171   0.171    0.171
## 124 Mam.Sp.Richness2  ~      Attendance2 23.391 -0.957  -0.957   -0.935
## 125 Mam.Sp.Richness2  ~   Total.Animals2  9.446 -0.299  -0.299   -0.297
## 126 Mam.Sp.Richness2  ~     Sp.Richness2 19.172 -0.809  -0.809   -0.809
## 128 Mam.Sp.Richness2  ~ Mean.Raup.Crick2 18.929 -0.231  -0.231   -0.231
## 148       X50km_Pop2  ~      Attendance2 28.148  0.582   0.582    0.568
## 149       X50km_Pop2  ~   Total.Animals2 29.269  0.280   0.280    0.278
```

```
## 152       X50km_Pop2  ~ Mean.Raup.Crick2  3.751  0.066   0.066    0.066
## 161       X10km_Pop2  ~   Total.Animals2  3.020 -0.082  -0.082   -0.082
## 173    GDP.Millions2  ~   Total.Animals2  4.885  0.101   0.101    0.100
## 174    GDP.Millions2  ~     Sp.Richness2 29.672  0.331   0.331    0.331
## 175    GDP.Millions2  ~ Brillouin.Index2 18.438  0.171   0.171    0.173
## 176    GDP.Millions2  ~ Mean.Raup.Crick2 14.170 -0.103  -0.103   -0.103
## 185 Nat_Pop._WB20152  ~   Total.Animals2  3.917 -0.079  -0.079   -0.078
## 186 Nat_Pop._WB20152  ~     Sp.Richness2 30.864 -0.326  -0.326   -0.326
## 187 Nat_Pop._WB20152  ~ Brillouin.Index2 15.979 -0.138  -0.138   -0.139
## 188 Nat_Pop._WB20152  ~ Mean.Raup.Crick2 16.072  0.106   0.106    0.106
## 200     Prop.Mam.Sp2  ~ Mean.Raup.Crick2  3.984 -0.083  -0.083   -0.083
##     sepc.nox
## 66    -0.066
## 67     0.072
## 68     0.038
## 69    -0.088
## 74     0.108
## 75    -0.250
## 76     0.083
## 79     0.142
## 80     0.068
## 81     0.133
## 85     0.060
## 86    -0.099
## 87     0.067
## 95     0.061
## 99    -0.059
## 101    0.076
## 102    0.262
## 103   -0.353
## 104   -0.193
## 105    0.181
## 106   -0.204
## 107    0.166
## 108    0.149
## 110    0.149
## 111   -0.178
## 112    0.765
## 113    0.245
## 114    0.391
## 115    0.224
## 116    0.171
## 124   -0.935
## 125   -0.297
## 126   -0.809
## 128   -0.231
## 148    0.568
## 149    0.278
## 152    0.066
## 161   -0.082
## 173    0.100
## 174    0.331
## 175    0.173
## 176   -0.103
```

```
## 185   -0.078
## 186   -0.326
## 187   -0.139
## 188    0.106
## 200   -0.083
```

```
# Adjust for the nested nature of the data (institutions within countries)
# Fit model and generate model summary
design <- svydesign(ids = ~Country, nest=TRUE, data=sem_attendance_data)
fit.adj15 <- lavaan.survey(lavaan.fit = mod.15.fit, survey.design = design)
summary(fit.adj15, rsq = TRUE)
```

```
## lavaan (0.5-23.1097) converged normally after  45 iterations
##
##   Number of observations                           458
##
##   Estimator                                         ML      Robust
##   Minimum Function Test Statistic              240.130      53.004
##   Degrees of freedom                                30          30
##   P-value (Chi-square)                           0.000       0.006
##   Scaling correction factor                                  4.530
##     for the Satorra-Bentler correction
##
## Parameter Estimates:
##
##   Information                                 Expected
##   Standard Errors                           Robust.sem
##
## Regressions:
##                      Estimate  Std.Err  z-value  P(>|z|)
##   Attendance2 ~
##     Zoo.Area.ha2        0.084    0.037    2.279    0.023
##     Total.Animals2      0.353    0.035    9.972    0.000
##     Mam.Sp.Rchnss2      0.056    0.034    1.650    0.099
##     Men.Sp.BdyMss2      0.348    0.028   12.568    0.000
##     Mean.Rap.Crck2      0.158    0.028    5.703    0.000
##     X50km_Pop2          0.087    0.034    2.563    0.010
##     X10km_Pop2          0.389    0.041    9.600    0.000
##     GDP.Millions2       0.217    0.065    3.326    0.001
##     Nt_Pp._WB20152     -0.102    0.076   -1.335    0.182
##   Total.Animals2 ~
##     Zoo.Area.ha2        0.219    0.034    6.384    0.000
##     Sp.Richness2        0.805    0.049   16.513    0.000
##     GDP.Millions2      -0.129    0.072   -1.786    0.074
##   Sp.Richness2 ~
##     Prop.Mam.Sp2       -0.563    0.041  -13.858    0.000
##     Mam.Sp.Rchnss2      0.779    0.057   13.593    0.000
##   Brillouin.Index2 ~
##     Sp.Richness2        1.323    0.088   15.114    0.000
##     Total.Animals2     -0.623    0.127   -4.909    0.000
##     GDP.Millions2      -0.206    0.073   -2.846    0.004
##   Mean.Raup.Crick2 ~
##     Total.Animals2      0.259    0.062    4.216    0.000
##     Men.Sp.BdyMss2     -0.448    0.082   -5.495    0.000
##
```

```
## Covariances:
##                        Estimate  Std.Err  z-value  P(>|z|)
##  .Attendance2 ~~
##    .Brilloun.Indx2        0.016    0.018    0.910    0.363
##   Zoo.Area.ha2 ~~
##     Mam.Sp.Rchnss2        0.381    0.064    5.985    0.000
##     Men.Sp.BdyMss2        0.534    0.104    5.147    0.000
##     X50km_Pop2            0.060    0.069    0.880    0.379
##     X10km_Pop2           -0.010    0.078   -0.124    0.901
##     GDP.Millions2        -0.027    0.041   -0.673    0.501
##     Nt_Pp._WB20152        0.061    0.090    0.682    0.495
##     Prop.Mam.Sp2          0.312    0.089    3.510    0.000
##   Mam.Sp.Richness2 ~~
##     Men.Sp.BdyMss2        0.303    0.081    3.730    0.000
##     X50km_Pop2            0.204    0.082    2.487    0.013
##     X10km_Pop2            0.284    0.066    4.278    0.000
##     GDP.Millions2        -0.060    0.064   -0.937    0.349
##     Nt_Pp._WB20152       -0.088    0.102   -0.863    0.388
##     Prop.Mam.Sp2          0.088    0.061    1.435    0.151
##   Mean.Sp.BodyMass2 ~~
##     X50km_Pop2            0.101    0.072    1.400    0.161
##     X10km_Pop2            0.126    0.067    1.895    0.058
##     GDP.Millions2        -0.082    0.046   -1.759    0.079
##     Nt_Pp._WB20152        0.006    0.092    0.063    0.950
##     Prop.Mam.Sp2          0.604    0.130    4.656    0.000
##   X50km_Pop2 ~~
##     X10km_Pop2            0.752    0.126    5.982    0.000
##     GDP.Millions2        -0.013    0.100   -0.131    0.896
##     Nt_Pp._WB20152        0.191    0.177    1.083    0.279
##     Prop.Mam.Sp2         -0.096    0.051   -1.888    0.059
##   X10km_Pop2 ~~
##     GDP.Millions2        -0.028    0.056   -0.505    0.613
##     Nt_Pp._WB20152        0.173    0.101    1.701    0.089
##     Prop.Mam.Sp2         -0.204    0.055   -3.685    0.000
##   GDP.Millions2 ~~
##     Nt_Pp._WB20152        0.826    0.202    4.089    0.000
##     Prop.Mam.Sp2         -0.123    0.105   -1.172    0.241
##   Nat_Pop._WB20152 ~~
##     Prop.Mam.Sp2         -0.115    0.091   -1.257    0.209
##
## Intercepts:
##                    Estimate  Std.Err  z-value  P(>|z|)
##    .Attendance2      -0.000    0.030   -0.000    1.000
##    .Total.Animals2   -0.000    0.070   -0.000    1.000
##    .Sp.Richness2      0.000    0.032    0.000    1.000
##    .Brilloun.Indx2   -0.000    0.055   -0.000    1.000
##    .Mean.Rap.Crck2   -0.000    0.098   -0.000    1.000
##     Zoo.Area.ha2      0.000    0.064    0.000    1.000
##     Mam.Sp.Rchnss2   -0.000    0.079   -0.000    1.000
##     Men.Sp.BdyMss2   -0.000    0.080   -0.000    1.000
##     X50km_Pop2       -0.000    0.114   -0.000    1.000
##     X10km_Pop2        0.000    0.105    0.000    1.000
##     GDP.Millions2    -0.000    0.345   -0.000    1.000
##     Nt_Pp._WB20152    0.000    0.295    0.000    1.000
```

```
##     Prop.Mam.Sp2      0.000    0.118    0.000    1.000
##
## Variances:
##                    Estimate  Std.Err  z-value  P(>|z|)
##    .Attendance2       0.270    0.028    9.694    0.000
##    .Total.Animals2    0.235    0.038    6.139    0.000
##    .Sp.Richness2      0.154    0.044    3.506    0.000
##    .Brilloun.Indx2    0.251    0.053    4.702    0.000
##    .Mean.Rap.Crck2    0.739    0.109    6.767    0.000
##     Zoo.Area.ha2      0.998    0.112    8.886    0.000
##     Mam.Sp.Rchnss2    0.998    0.115    8.669    0.000
##     Men.Sp.BdyMss2    0.998    0.173    5.783    0.000
##     X50km_Pop2        0.998    0.131    7.623    0.000
##     X10km_Pop2        0.998    0.174    5.745    0.000
##     GDP.Millions2     0.998    0.246    4.061    0.000
##     Nt_Pp._WB20152    0.998    0.225    4.439    0.000
##     Prop.Mam.Sp2      0.998    0.150    6.662    0.000
##
## R-Square:
##                    Estimate
##     Attendance2       0.716
##     Total.Animals2    0.762
##     Sp.Richness2      0.845
##     Brilloun.Indx2    0.753
##     Mean.Rap.Crck2    0.257
```

```
# Generate fit indices
fitMeasures(fit.adj15, c("agfi", "rmr", "srmr", "rmsea", "cfi", "nnfi", "tli"))
```

```
##  agfi   rmr  srmr rmsea   cfi  nnfi   tli
## 0.750 0.051 0.048 0.124 0.932 0.886 0.886
```

```
# Generate modification indices
mi15adj <- modindices(fit.adj15)
print(mi15adj[mi15adj$mi > 3.0,])
```

```
##                  lhs op              rhs     mi mi.scaled    epc sepc.lv
## 79    Total.Animals2 ~~ Brillouin.Index2  5.618     1.240 -0.066  -0.066
## 80    Total.Animals2 ~~ Mean.Raup.Crick2 10.289     2.271  0.072   0.072
## 81      Sp.Richness2 ~~ Brillouin.Index2 14.829     3.273  0.038   0.038
## 82      Sp.Richness2 ~~ Mean.Raup.Crick2 27.799     6.136 -0.088  -0.088
## 87    Total.Animals2  ~      Attendance2  9.795     2.162  0.110   0.110
## 88    Total.Animals2  ~ Brillouin.Index2  4.965     1.096 -0.246  -0.246
## 89    Total.Animals2  ~ Mean.Raup.Crick2  9.331     2.060  0.083   0.083
## 92    Total.Animals2  ~       X50km_Pop2 36.704     8.102  0.141   0.141
## 93    Total.Animals2  ~       X10km_Pop2  7.850     1.733  0.068   0.068
## 94    Total.Animals2  ~ Nat_Pop._WB20152 10.486     2.315  0.132   0.132
## 98      Sp.Richness2  ~ Brillouin.Index2  3.381     0.746  0.060   0.060
## 99      Sp.Richness2  ~ Mean.Raup.Crick2 22.606     4.990 -0.099  -0.099
## 100     Sp.Richness2  ~     Zoo.Area.ha2 10.169     2.245  0.067   0.067
## 108 Brillouin.Index2  ~     Zoo.Area.ha2  5.618     1.240  0.061   0.061
## 112 Brillouin.Index2  ~       X10km_Pop2  5.677     1.253 -0.059  -0.059
## 114 Brillouin.Index2  ~     Prop.Mam.Sp2  7.667     1.692  0.076   0.076
## 115 Mean.Raup.Crick2  ~      Attendance2  8.705     1.921  0.268   0.268
## 116 Mean.Raup.Crick2  ~     Sp.Richness2 21.846     4.822 -0.352  -0.352
## 117 Mean.Raup.Crick2  ~ Brillouin.Index2 16.525     3.648 -0.191  -0.191
```

```
## 118 Mean.Raup.Crick2  ~     Zoo.Area.ha2 12.524     2.764  0.180   0.180
## 119 Mean.Raup.Crick2  ~ Mam.Sp.Richness2 11.750     2.594 -0.203  -0.203
## 120 Mean.Raup.Crick2  ~       X50km_Pop2 16.155     3.566  0.165   0.165
## 121 Mean.Raup.Crick2  ~       X10km_Pop2 12.518     2.763  0.149   0.149
## 123 Mean.Raup.Crick2  ~ Nat_Pop._WB20152 13.450     2.969  0.148   0.148
## 124 Mean.Raup.Crick2  ~     Prop.Mam.Sp2 10.037     2.215 -0.177  -0.177
## 125     Zoo.Area.ha2  ~      Attendance2 22.932     5.062  0.783   0.783
## 126     Zoo.Area.ha2  ~   Total.Animals2  8.897     1.964  0.247   0.247
## 127     Zoo.Area.ha2  ~     Sp.Richness2 17.991     3.971  0.391   0.391
## 128     Zoo.Area.ha2  ~ Brillouin.Index2 13.742     3.033  0.222   0.222
## 129     Zoo.Area.ha2  ~ Mean.Raup.Crick2 16.392     3.618  0.171   0.171
## 137 Mam.Sp.Richness2  ~      Attendance2 23.392     5.163 -0.957  -0.957
## 138 Mam.Sp.Richness2  ~   Total.Animals2  9.446     2.085 -0.299  -0.299
## 139 Mam.Sp.Richness2  ~     Sp.Richness2 19.172     4.232 -0.809  -0.809
## 141 Mam.Sp.Richness2  ~ Mean.Raup.Crick2 18.929     4.178 -0.231  -0.231
## 161       X50km_Pop2  ~      Attendance2 28.148     6.213  0.582   0.582
## 162       X50km_Pop2  ~   Total.Animals2 29.269     6.460  0.280   0.280
## 165       X50km_Pop2  ~ Mean.Raup.Crick2  3.751     0.828  0.066   0.066
## 174       X10km_Pop2  ~   Total.Animals2  3.020     0.667 -0.082  -0.082
## 186    GDP.Millions2  ~   Total.Animals2  4.885     1.078  0.101   0.101
## 187    GDP.Millions2  ~     Sp.Richness2 29.672     6.550  0.331   0.331
## 188    GDP.Millions2  ~ Brillouin.Index2 18.438     4.070  0.171   0.171
## 189    GDP.Millions2  ~ Mean.Raup.Crick2 14.170     3.128 -0.103  -0.103
## 198 Nat_Pop._WB20152  ~   Total.Animals2  3.917     0.865 -0.079  -0.079
## 199 Nat_Pop._WB20152  ~     Sp.Richness2 30.864     6.813 -0.326  -0.326
## 200 Nat_Pop._WB20152  ~ Brillouin.Index2 15.979     3.527 -0.138  -0.138
## 201 Nat_Pop._WB20152  ~ Mean.Raup.Crick2 16.072     3.548  0.106   0.106
## 213     Prop.Mam.Sp2  ~ Mean.Raup.Crick2  3.984     0.879 -0.083  -0.083
##     sepc.all sepc.nox
## 79    -0.066   -0.066
## 80     0.072    0.072
## 81     0.038    0.038
## 82    -0.088   -0.088
## 87     0.108    0.108
## 88    -0.250   -0.250
## 89     0.083    0.083
## 92     0.142    0.142
## 93     0.068    0.068
## 94     0.133    0.133
## 98     0.060    0.060
## 99    -0.099   -0.099
## 100    0.067    0.067
## 108    0.061    0.061
## 112   -0.059   -0.059
## 114    0.076    0.076
## 115    0.262    0.262
## 116   -0.353   -0.353
## 117   -0.193   -0.193
## 118    0.180    0.181
## 119   -0.204   -0.204
## 120    0.166    0.166
## 121    0.149    0.149
## 123    0.148    0.149
## 124   -0.178   -0.178
```

```
## 125    0.765    0.765
## 126    0.245    0.245
## 127    0.391    0.391
## 128    0.224    0.224
## 129    0.171    0.171
## 137   -0.935   -0.935
## 138   -0.297   -0.297
## 139   -0.809   -0.809
## 141   -0.231   -0.231
## 161    0.568    0.568
## 162    0.278    0.278
## 165    0.066    0.066
## 174   -0.082   -0.082
## 186    0.100    0.100
## 187    0.331    0.331
## 188    0.173    0.173
## 189   -0.103   -0.103
## 198   -0.078   -0.078
## 199   -0.326   -0.326
## 200   -0.139   -0.139
## 201    0.106    0.106
## 213   -0.083   -0.083
```

Model 16

Based on the results generated from the nested fifteenth model, the highest p-value relationship to be considered for removal is ___Attendance2 ~ Nt_Pp._WB20152___ with a p-value of **0.182**. Therefore we decide to remove this pathway. Once again, the model summary, fit indices and modification indices were all generated for the model, adjusting for the nested nature of data.

```
# Attendance SEM (Presence-Absence)

# Model 16
# Removal of Attendance2 ~ Nt_Pp._WB20152, p = 0.182

mod.16 <- 'Attendance2 ~ Zoo.Area.ha2 + Total.Animals2
+ Mam.Sp.Richness2 + Mean.Sp.BodyMass2 + Mean.Raup.Crick2
+ X50km_Pop2 + X10km_Pop2 + GDP.Millions2

Total.Animals2 ~ Zoo.Area.ha2 + Sp.Richness2 + GDP.Millions2
Sp.Richness2 ~ Prop.Mam.Sp2 + Mam.Sp.Richness2
Brillouin.Index2 ~ Sp.Richness2 + Total.Animals2 + GDP.Millions2
Mean.Raup.Crick2 ~ Total.Animals2 + Mean.Sp.BodyMass2'

# Fit model and generate model summary
mod.16.fit <- sem(mod.16, data = sem_attendance_data, fixed.x=FALSE)
summary(mod.16.fit, rsq = TRUE)
```

```
## lavaan (0.5-23.1097) converged normally after  33 iterations
##
##   Number of observations                           458
##
##   Estimator                                         ML
##   Minimum Function Test Statistic              192.979
```

```
##   Degrees of freedom                                26
##   P-value (Chi-square)                           0.000
##
## Parameter Estimates:
##
##   Information                                 Expected
##   Standard Errors                             Standard
##
## Regressions:
##                       Estimate  Std.Err  z-value  P(>|z|)
##   Attendance2 ~
##     Zoo.Area.ha2         0.073    0.032    2.268    0.023
##     Total.Animals2       0.361    0.037    9.684    0.000
##     Mam.Sp.Rchnss2       0.067    0.036    1.843    0.065
##     Men.Sp.BdyMss2       0.340    0.034   10.020    0.000
##     Mean.Rap.Crck2       0.144    0.028    5.096    0.000
##     X50km_Pop2           0.076    0.038    2.035    0.042
##     X10km_Pop2           0.374    0.039    9.521    0.000
##     GDP.Millions2        0.133    0.025    5.348    0.000
##   Total.Animals2 ~
##     Zoo.Area.ha2         0.219    0.023    9.570    0.000
##     Sp.Richness2         0.805    0.023   35.245    0.000
##     GDP.Millions2       -0.129    0.023   -5.670    0.000
##   Sp.Richness2 ~
##     Prop.Mam.Sp2        -0.563    0.018  -30.494    0.000
##     Mam.Sp.Rchnss2       0.779    0.018   42.208    0.000
##   Brillouin.Index2 ~
##     Sp.Richness2         1.322    0.043   30.425    0.000
##     Total.Animals2      -0.622    0.044  -14.127    0.000
##     GDP.Millions2       -0.206    0.024   -8.533    0.000
##   Mean.Raup.Crick2 ~
##     Total.Animals2       0.259    0.041    6.400    0.000
##     Men.Sp.BdyMss2      -0.448    0.040  -11.127    0.000
##
## Covariances:
##                         Estimate  Std.Err  z-value  P(>|z|)
##  .Attendance2 ~~
##    .Brilloun.Indx2         0.017    0.012    1.394    0.163
##   Zoo.Area.ha2 ~~
##     Mam.Sp.Rchnss2         0.381    0.050    7.634    0.000
##     Men.Sp.BdyMss2         0.534    0.053   10.096    0.000
##     X50km_Pop2             0.060    0.047    1.291    0.197
##     X10km_Pop2            -0.010    0.047   -0.208    0.835
##     GDP.Millions2         -0.027    0.047   -0.588    0.557
##     Prop.Mam.Sp2           0.312    0.049    6.389    0.000
##   Mam.Sp.Richness2 ~~
##     Men.Sp.BdyMss2         0.303    0.049    6.216    0.000
##     X50km_Pop2             0.204    0.048    4.281    0.000
##     X10km_Pop2             0.284    0.048    5.852    0.000
##     GDP.Millions2         -0.060    0.047   -1.277    0.202
##     Prop.Mam.Sp2           0.088    0.047    1.882    0.060
##   Mean.Sp.BodyMass2 ~~
##     X50km_Pop2             0.101    0.047    2.166    0.030
##     X10km_Pop2             0.126    0.047    2.683    0.007
```

```
##     GDP.Millions2        -0.082    0.047   -1.748    0.080
##     Prop.Mam.Sp2          0.604    0.055   11.083    0.000
##   X50km_Pop2 ~~
##     X10km_Pop2            0.752    0.058   12.886    0.000
##     GDP.Millions2        -0.013    0.047   -0.281    0.779
##     Prop.Mam.Sp2         -0.096    0.047   -2.054    0.040
##   X10km_Pop2 ~~
##     GDP.Millions2        -0.028    0.047   -0.604    0.546
##     Prop.Mam.Sp2         -0.204    0.048   -4.293    0.000
##   GDP.Millions2 ~~
##     Prop.Mam.Sp2         -0.123    0.047   -2.615    0.009
##
## Variances:
##                    Estimate  Std.Err  z-value  P(>|z|)
##    .Attendance2       0.273    0.018   15.133    0.000
##    .Total.Animals2    0.235    0.016   15.133    0.000
##    .Sp.Richness2      0.154    0.010   15.133    0.000
##    .Brilloun.Indx2    0.251    0.017   15.133    0.000
##    .Mean.Rap.Crck2    0.739    0.049   15.133    0.000
##     Zoo.Area.ha2      0.998    0.066   15.133    0.000
##     Mam.Sp.Rchnss2    0.998    0.066   15.133    0.000
##     Men.Sp.BdyMss2    0.998    0.066   15.133    0.000
##     X50km_Pop2        0.998    0.066   15.133    0.000
##     X10km_Pop2        0.998    0.066   15.133    0.000
##     GDP.Millions2     0.998    0.066   15.133    0.000
##     Prop.Mam.Sp2      0.998    0.066   15.133    0.000
##
## R-Square:
##                    Estimate
##     Attendance2       0.714
##     Total.Animals2    0.762
##     Sp.Richness2      0.845
##     Brilloun.Indx2    0.753
##     Mean.Rap.Crck2    0.257
```

```r
# Generate fit indices
fitMeasures(mod.16.fit, c("agfi", "rmr", "srmr", "rmsea", "cfi", "nnfi", "tli"))
```

```
##  agfi   rmr  srmr rmsea   cfi  nnfi   tli
## 0.818 0.052 0.052 0.118 0.945 0.905 0.905
```

```r
# Generate modification indices
mi16 <- modindices(mod.16.fit)
print(mi16[mi16$mi > 3.0,])
```

```
##                 lhs op              rhs     mi    epc sepc.lv sepc.all
## 57    Total.Animals2 ~~ Brillouin.Index2  5.606 -0.066  -0.066   -0.066
## 58    Total.Animals2 ~~ Mean.Raup.Crick2 10.289  0.072   0.072    0.072
## 59      Sp.Richness2 ~~ Brillouin.Index2 14.694  0.038   0.038    0.038
## 60      Sp.Richness2 ~~ Mean.Raup.Crick2 27.799 -0.088  -0.088   -0.088
## 65    Total.Animals2  ~      Attendance2  9.397  0.108   0.108    0.106
## 66    Total.Animals2  ~ Brillouin.Index2  5.036 -0.247  -0.247   -0.251
## 67    Total.Animals2  ~ Mean.Raup.Crick2  9.331  0.083   0.083    0.083
## 70    Total.Animals2  ~       X50km_Pop2 36.704  0.141   0.141    0.142
## 71    Total.Animals2  ~       X10km_Pop2  7.850  0.068   0.068    0.068
## 75      Sp.Richness2  ~ Brillouin.Index2  3.389  0.060   0.060    0.060
```

```
## 76      Sp.Richness2  ~ Mean.Raup.Crick2 22.606 -0.099  -0.099   -0.099
## 77      Sp.Richness2  ~     Zoo.Area.ha2 10.169  0.067   0.067    0.067
## 84  Brillouin.Index2  ~     Zoo.Area.ha2  5.606  0.061   0.061    0.061
## 88  Brillouin.Index2  ~       X10km_Pop2  5.673 -0.059  -0.059   -0.059
## 89  Brillouin.Index2  ~     Prop.Mam.Sp2  7.623  0.076   0.076    0.075
## 90  Mean.Raup.Crick2  ~      Attendance2 12.432  0.321   0.321    0.314
## 91  Mean.Raup.Crick2  ~     Sp.Richness2 21.846 -0.352  -0.352   -0.353
## 92  Mean.Raup.Crick2  ~ Brillouin.Index2 16.532 -0.191  -0.191   -0.193
## 93  Mean.Raup.Crick2  ~     Zoo.Area.ha2 12.524  0.180   0.180    0.180
## 94  Mean.Raup.Crick2  ~ Mam.Sp.Richness2 11.750 -0.203  -0.203   -0.204
## 95  Mean.Raup.Crick2  ~       X50km_Pop2 16.155  0.165   0.165    0.166
## 96  Mean.Raup.Crick2  ~       X10km_Pop2 12.518  0.149   0.149    0.149
## 98  Mean.Raup.Crick2  ~     Prop.Mam.Sp2 10.037 -0.177  -0.177   -0.178
## 99      Zoo.Area.ha2  ~      Attendance2 24.534  0.853   0.853    0.834
## 100     Zoo.Area.ha2  ~   Total.Animals2  6.487  0.219   0.219    0.217
## 101     Zoo.Area.ha2  ~     Sp.Richness2 10.153  0.300   0.300    0.300
## 102     Zoo.Area.ha2  ~ Brillouin.Index2  8.331  0.178   0.178    0.179
## 103     Zoo.Area.ha2  ~ Mean.Raup.Crick2 24.815  0.216   0.216    0.215
## 110 Mam.Sp.Richness2  ~      Attendance2 27.371 -1.141  -1.141   -1.115
## 111 Mam.Sp.Richness2  ~   Total.Animals2  6.212 -0.264  -0.264   -0.262
## 112 Mam.Sp.Richness2  ~     Sp.Richness2  3.181 -0.384  -0.384   -0.384
## 114 Mam.Sp.Richness2  ~ Mean.Raup.Crick2 35.932 -0.337  -0.337   -0.337
## 132       X50km_Pop2  ~      Attendance2 29.808  0.611   0.611    0.597
## 133       X50km_Pop2  ~   Total.Animals2 26.927  0.271   0.271    0.269
## 136       X50km_Pop2  ~ Mean.Raup.Crick2  6.276  0.087   0.087    0.087
## 144       X10km_Pop2  ~   Total.Animals2  4.593 -0.103  -0.103   -0.102
## 169     Prop.Mam.Sp2  ~ Mean.Raup.Crick2  4.449 -0.088  -0.088   -0.088
##     sepc.nox
## 57    -0.066
## 58     0.072
## 59     0.038
## 60    -0.088
## 65     0.106
## 66    -0.251
## 67     0.083
## 70     0.142
## 71     0.068
## 75     0.060
## 76    -0.099
## 77     0.067
## 84     0.061
## 88    -0.059
## 89     0.076
## 90     0.314
## 91    -0.353
## 92    -0.193
## 93     0.181
## 94    -0.204
## 95     0.166
## 96     0.149
## 98    -0.178
## 99     0.834
## 100    0.217
## 101    0.300
```

```
## 102    0.179
## 103    0.215
## 110   -1.115
## 111   -0.262
## 112   -0.384
## 114   -0.337
## 132    0.597
## 133    0.269
## 136    0.087
## 144   -0.102
## 169   -0.088
```

```r
# Adjust for the nested nature of the data (institutions within countries)
# Fit model and generate model summary
design <- svydesign(ids = ~Country, nest=TRUE, data=sem_attendance_data)
fit.adj16 <- lavaan.survey(lavaan.fit = mod.16.fit, survey.design = design)
summary(fit.adj16, rsq = TRUE)
```

```
## lavaan (0.5-23.1097) converged normally after  33 iterations
##
##   Number of observations                           458
##
##   Estimator                                         ML      Robust
##   Minimum Function Test Statistic              192.979      65.825
##   Degrees of freedom                                26          26
##   P-value (Chi-square)                           0.000       0.000
##   Scaling correction factor                                  2.932
##     for the Satorra-Bentler correction
##
## Parameter Estimates:
##
##   Information                                 Expected
##   Standard Errors                           Robust.sem
##
## Regressions:
##                      Estimate  Std.Err  z-value  P(>|z|)
##   Attendance2 ~
##     Zoo.Area.ha2        0.073    0.036    2.032    0.042
##     Total.Animals2      0.361    0.035   10.286    0.000
##     Mam.Sp.Rchnss2      0.067    0.029    2.303    0.021
##     Men.Sp.BdyMss2      0.340    0.028   12.193    0.000
##     Mean.Rap.Crck2      0.144    0.026    5.467    0.000
##     X50km_Pop2          0.076    0.035    2.155    0.031
##     X10km_Pop2          0.374    0.043    8.791    0.000
##     GDP.Millions2       0.133    0.025    5.397    0.000
##   Total.Animals2 ~
##     Zoo.Area.ha2        0.219    0.034    6.384    0.000
##     Sp.Richness2        0.805    0.049   16.513    0.000
##     GDP.Millions2      -0.129    0.072   -1.786    0.074
##   Sp.Richness2 ~
##     Prop.Mam.Sp2       -0.563    0.041  -13.858    0.000
##     Mam.Sp.Rchnss2      0.779    0.057   13.593    0.000
##   Brillouin.Index2 ~
##     Sp.Richness2        1.322    0.087   15.125    0.000
##     Total.Animals2     -0.622    0.127   -4.906    0.000
```

```
##     GDP.Millions2      -0.206    0.072   -2.847    0.004
##   Mean.Raup.Crick2 ~
##     Total.Animals2      0.259    0.062    4.216    0.000
##     Men.Sp.BdyMss2     -0.448    0.082   -5.495    0.000
##
## Covariances:
##                        Estimate  Std.Err  z-value  P(>|z|)
##  .Attendance2 ~~
##    .Brilloun.Indx2        0.017    0.017    0.978    0.328
##   Zoo.Area.ha2 ~~
##     Mam.Sp.Rchnss2        0.381    0.064    5.985    0.000
##     Men.Sp.BdyMss2        0.534    0.104    5.147    0.000
##     X50km_Pop2            0.060    0.069    0.880    0.379
##     X10km_Pop2           -0.010    0.078   -0.124    0.901
##     GDP.Millions2        -0.027    0.041   -0.673    0.501
##     Prop.Mam.Sp2          0.312    0.089    3.510    0.000
##   Mam.Sp.Richness2 ~~
##     Men.Sp.BdyMss2        0.303    0.081    3.730    0.000
##     X50km_Pop2            0.204    0.082    2.487    0.013
##     X10km_Pop2            0.284    0.066    4.278    0.000
##     GDP.Millions2        -0.060    0.064   -0.937    0.349
##     Prop.Mam.Sp2          0.088    0.061    1.435    0.151
##   Mean.Sp.BodyMass2 ~~
##     X50km_Pop2            0.101    0.072    1.400    0.161
##     X10km_Pop2            0.126    0.067    1.895    0.058
##     GDP.Millions2        -0.082    0.046   -1.759    0.079
##     Prop.Mam.Sp2          0.604    0.130    4.656    0.000
##   X50km_Pop2 ~~
##     X10km_Pop2            0.752    0.126    5.982    0.000
##     GDP.Millions2        -0.013    0.100   -0.131    0.896
##     Prop.Mam.Sp2         -0.096    0.051   -1.888    0.059
##   X10km_Pop2 ~~
##     GDP.Millions2        -0.028    0.056   -0.505    0.613
##     Prop.Mam.Sp2         -0.204    0.055   -3.685    0.000
##   GDP.Millions2 ~~
##     Prop.Mam.Sp2         -0.123    0.105   -1.172    0.241
##
## Intercepts:
##                    Estimate  Std.Err  z-value  P(>|z|)
##    .Attendance2      -0.000    0.030   -0.000    1.000
##    .Total.Animals2   -0.000    0.070   -0.000    1.000
##    .Sp.Richness2      0.000    0.032    0.000    1.000
##    .Brilloun.Indx2   -0.000    0.055   -0.000    1.000
##    .Mean.Rap.Crck2   -0.000    0.098   -0.000    1.000
##     Zoo.Area.ha2      0.000    0.064    0.000    1.000
##     Mam.Sp.Rchnss2   -0.000    0.079   -0.000    1.000
##     Men.Sp.BdyMss2   -0.000    0.080   -0.000    1.000
##     X50km_Pop2       -0.000    0.114   -0.000    1.000
##     X10km_Pop2        0.000    0.105    0.000    1.000
##     GDP.Millions2    -0.000    0.345   -0.000    1.000
##     Prop.Mam.Sp2      0.000    0.118    0.000    1.000
##
## Variances:
##                    Estimate  Std.Err  z-value  P(>|z|)
```

```
##    .Attendance2       0.273    0.029    9.569    0.000
##    .Total.Animals2    0.235    0.038    6.139    0.000
##    .Sp.Richness2      0.154    0.044    3.506    0.000
##    .Brilloun.Indx2    0.251    0.053    4.701    0.000
##    .Mean.Rap.Crck2    0.739    0.109    6.767    0.000
##     Zoo.Area.ha2      0.998    0.112    8.886    0.000
##     Mam.Sp.Rchnss2    0.998    0.115    8.669    0.000
##     Men.Sp.BdyMss2    0.998    0.173    5.783    0.000
##     X50km_Pop2        0.998    0.131    7.623    0.000
##     X10km_Pop2        0.998    0.174    5.745    0.000
##     GDP.Millions2     0.998    0.246    4.061    0.000
##     Prop.Mam.Sp2      0.998    0.150    6.662    0.000
##
## R-Square:
##                    Estimate
##     Attendance2       0.714
##     Total.Animals2    0.762
##     Sp.Richness2      0.845
##     Brilloun.Indx2    0.753
##     Mean.Rap.Crck2    0.257
```

```
# Generate fit indices
fitMeasures(fit.adj16, c("agfi", "rmr", "srmr", "rmsea", "cfi", "nnfi", "tli"))
```

```
##  agfi   rmr  srmr rmsea   cfi  nnfi   tli
## 0.790 0.052 0.048 0.118 0.945 0.905 0.905
```

```
# Generate modification indices
mi16adj <- modindices(fit.adj16)
print(mi16adj[mi16adj$mi > 3.0,])
```

```
##                   lhs op              rhs     mi mi.scaled    epc sepc.lv
## 69    Total.Animals2 ~~ Brillouin.Index2  5.606     1.912 -0.066  -0.066
## 70    Total.Animals2 ~~ Mean.Raup.Crick2 10.289     3.510  0.072   0.072
## 71      Sp.Richness2 ~~ Brillouin.Index2 14.694     5.012  0.038   0.038
## 72      Sp.Richness2 ~~ Mean.Raup.Crick2 27.799     9.482 -0.088  -0.088
## 77    Total.Animals2  ~      Attendance2  9.397     3.205  0.108   0.108
## 78    Total.Animals2  ~ Brillouin.Index2  5.036     1.718 -0.247  -0.247
## 79    Total.Animals2  ~ Mean.Raup.Crick2  9.331     3.183  0.083   0.083
## 82    Total.Animals2  ~       X50km_Pop2 36.704    12.520  0.141   0.141
## 83    Total.Animals2  ~       X10km_Pop2  7.850     2.677  0.068   0.068
## 87      Sp.Richness2  ~ Brillouin.Index2  3.389     1.156  0.060   0.060
## 88      Sp.Richness2  ~ Mean.Raup.Crick2 22.606     7.711 -0.099  -0.099
## 89      Sp.Richness2  ~     Zoo.Area.ha2 10.169     3.468  0.067   0.067
## 96  Brillouin.Index2  ~     Zoo.Area.ha2  5.606     1.912  0.061   0.061
## 100 Brillouin.Index2  ~       X10km_Pop2  5.673     1.935 -0.059  -0.059
## 101 Brillouin.Index2  ~     Prop.Mam.Sp2  7.623     2.600  0.076   0.076
## 102 Mean.Raup.Crick2  ~      Attendance2 12.432     4.241  0.321   0.321
## 103 Mean.Raup.Crick2  ~     Sp.Richness2 21.846     7.452 -0.352  -0.352
## 104 Mean.Raup.Crick2  ~ Brillouin.Index2 16.532     5.639 -0.191  -0.191
## 105 Mean.Raup.Crick2  ~     Zoo.Area.ha2 12.524     4.272  0.180   0.180
## 106 Mean.Raup.Crick2  ~ Mam.Sp.Richness2 11.750     4.008 -0.203  -0.203
## 107 Mean.Raup.Crick2  ~       X50km_Pop2 16.155     5.511  0.165   0.165
## 108 Mean.Raup.Crick2  ~       X10km_Pop2 12.518     4.270  0.149   0.149
## 110 Mean.Raup.Crick2  ~     Prop.Mam.Sp2 10.037     3.423 -0.177  -0.177
## 111     Zoo.Area.ha2  ~      Attendance2 24.533     8.368  0.853   0.853
```

```
## 112     Zoo.Area.ha2  ~   Total.Animals2  6.487     2.213  0.219   0.219
## 113     Zoo.Area.ha2  ~     Sp.Richness2 10.153     3.463  0.300   0.300
## 114     Zoo.Area.ha2  ~ Brillouin.Index2  8.331     2.842  0.178   0.178
## 115     Zoo.Area.ha2  ~ Mean.Raup.Crick2 24.815     8.464  0.216   0.216
## 122 Mam.Sp.Richness2  ~      Attendance2 27.371     9.336 -1.141  -1.141
## 123 Mam.Sp.Richness2  ~   Total.Animals2  6.212     2.119 -0.264  -0.264
## 124 Mam.Sp.Richness2  ~     Sp.Richness2  3.181     1.085 -0.384  -0.384
## 126 Mam.Sp.Richness2  ~ Mean.Raup.Crick2 35.932    12.256 -0.337  -0.337
## 144       X50km_Pop2  ~      Attendance2 29.808    10.168  0.611   0.611
## 145       X50km_Pop2  ~   Total.Animals2 26.927     9.185  0.271   0.271
## 148       X50km_Pop2  ~ Mean.Raup.Crick2  6.276     2.141  0.087   0.087
## 156       X10km_Pop2  ~   Total.Animals2  4.593     1.567 -0.103  -0.103
## 181     Prop.Mam.Sp2  ~ Mean.Raup.Crick2  4.449     1.517 -0.088  -0.088
##     sepc.all sepc.nox
## 69    -0.066   -0.066
## 70     0.072    0.072
## 71     0.038    0.038
## 72    -0.088   -0.088
## 77     0.106    0.106
## 78    -0.251   -0.251
## 79     0.083    0.083
## 82     0.142    0.142
## 83     0.068    0.068
## 87     0.060    0.060
## 88    -0.099   -0.099
## 89     0.067    0.067
## 96     0.061    0.061
## 100   -0.059   -0.059
## 101    0.075    0.076
## 102    0.314    0.314
## 103   -0.353   -0.353
## 104   -0.193   -0.193
## 105    0.180    0.181
## 106   -0.204   -0.204
## 107    0.166    0.166
## 108    0.149    0.149
## 110   -0.178   -0.178
## 111    0.834    0.834
## 112    0.217    0.217
## 113    0.300    0.300
## 114    0.179    0.179
## 115    0.215    0.215
## 122   -1.115   -1.115
## 123   -0.262   -0.262
## 124   -0.384   -0.384
## 126   -0.337   -0.337
## 144    0.597    0.597
## 145    0.269    0.269
## 148    0.087    0.087
## 156   -0.102   -0.102
## 181   -0.088   -0.088
```

Model 17

Based on the results generated from the nested sixteenth model, the highest p-value relationship to be considered for removal is **Total.Animals2 ~ GDP.Millions2** with a p-value of **0.074**. Therefore we decide to remove this pathway. Once again, the model summary, fit indices and modification indices were all generated for the model, adjusting for the nested nature of data.

```
# Attendance SEM (Presence-Absence)

# Model 17
# Removal of Total.Animals2 ~ GDP.Millions2, p = 0.074

mod.17 <- 'Attendance2 ~ Zoo.Area.ha2 + Total.Animals2
+ Mam.Sp.Richness2 + Mean.Sp.BodyMass2 + Mean.Raup.Crick2
+ X50km_Pop2 + X10km_Pop2 + GDP.Millions2

Total.Animals2 ~ Zoo.Area.ha2 + Sp.Richness2
Sp.Richness2 ~ Prop.Mam.Sp2 + Mam.Sp.Richness2
Brillouin.Index2 ~ Sp.Richness2 + Total.Animals2 + GDP.Millions2
Mean.Raup.Crick2 ~ Total.Animals2 + Mean.Sp.BodyMass2'

# Fit model and generate model summary
mod.17.fit <- sem(mod.17, data = sem_attendance_data, fixed.x=FALSE)
summary(mod.17.fit, rsq = TRUE)
```

```
## lavaan (0.5-23.1097) converged normally after  32 iterations
##
##   Number of observations                           458
##
##   Estimator                                         ML
##   Minimum Function Test Statistic              223.977
##   Degrees of freedom                                27
##   P-value (Chi-square)                           0.000
##
## Parameter Estimates:
##
##   Information                                 Expected
##   Standard Errors                             Standard
##
## Regressions:
##                    Estimate  Std.Err  z-value  P(>|z|)
##   Attendance2 ~
##     Zoo.Area.ha2      0.073    0.032    2.267    0.023
##     Total.Animals2    0.361    0.037    9.816    0.000
##     Mam.Sp.Rchnss2    0.067    0.036    1.860    0.063
##     Men.Sp.BdyMss2    0.340    0.034   10.044    0.000
##     Mean.Rap.Crck2    0.144    0.028    5.096    0.000
##     X50km_Pop2        0.076    0.038    2.035    0.042
##     X10km_Pop2        0.374    0.039    9.526    0.000
##     GDP.Millions2     0.133    0.025    5.391    0.000
##   Total.Animals2 ~
##     Zoo.Area.ha2      0.223    0.024    9.458    0.000
##     Sp.Richness2      0.798    0.024   33.781    0.000
##   Sp.Richness2 ~
##     Prop.Mam.Sp2     -0.563    0.018  -30.494    0.000
```

```
##     Mam.Sp.Rchnss2      0.779    0.018   42.208    0.000
##   Brillouin.Index2 ~
##     Sp.Richness2        1.322    0.042   31.285    0.000
##     Total.Animals2     -0.622    0.043  -14.583    0.000
##     GDP.Millions2      -0.206    0.023   -8.804    0.000
##   Mean.Raup.Crick2 ~
##     Total.Animals2      0.259    0.041    6.383    0.000
##     Men.Sp.BdyMss2     -0.448    0.040  -11.130    0.000
##
## Covariances:
##                        Estimate  Std.Err  z-value  P(>|z|)
##  .Attendance2 ~~
##    .Brilloun.Indx2        0.017    0.012    1.394    0.163
##   Zoo.Area.ha2 ~~
##     Mam.Sp.Rchnss2        0.381    0.050    7.634    0.000
##     Men.Sp.BdyMss2        0.534    0.053   10.096    0.000
##     X50km_Pop2            0.060    0.047    1.291    0.197
##     X10km_Pop2           -0.010    0.047   -0.208    0.835
##     GDP.Millions2        -0.027    0.047   -0.588    0.557
##     Prop.Mam.Sp2          0.312    0.049    6.389    0.000
##   Mam.Sp.Richness2 ~~
##     Men.Sp.BdyMss2        0.303    0.049    6.216    0.000
##     X50km_Pop2            0.204    0.048    4.281    0.000
##     X10km_Pop2            0.284    0.048    5.852    0.000
##     GDP.Millions2        -0.060    0.047   -1.277    0.202
##     Prop.Mam.Sp2          0.088    0.047    1.882    0.060
##   Mean.Sp.BodyMass2 ~~
##     X50km_Pop2            0.101    0.047    2.166    0.030
##     X10km_Pop2            0.126    0.047    2.683    0.007
##     GDP.Millions2        -0.082    0.047   -1.748    0.080
##     Prop.Mam.Sp2          0.604    0.055   11.083    0.000
##   X50km_Pop2 ~~
##     X10km_Pop2            0.752    0.058   12.886    0.000
##     GDP.Millions2        -0.013    0.047   -0.281    0.779
##     Prop.Mam.Sp2         -0.096    0.047   -2.054    0.040
##   X10km_Pop2 ~~
##     GDP.Millions2        -0.028    0.047   -0.604    0.546
##     Prop.Mam.Sp2         -0.204    0.048   -4.293    0.000
##   GDP.Millions2 ~~
##     Prop.Mam.Sp2         -0.123    0.047   -2.615    0.009
##
## Variances:
##                    Estimate  Std.Err  z-value  P(>|z|)
##    .Attendance2       0.273    0.018   15.133    0.000
##    .Total.Animals2    0.251    0.017   15.133    0.000
##    .Sp.Richness2      0.154    0.010   15.133    0.000
##    .Brilloun.Indx2    0.251    0.017   15.133    0.000
##    .Mean.Rap.Crck2    0.739    0.049   15.133    0.000
##     Zoo.Area.ha2      0.998    0.066   15.133    0.000
##     Mam.Sp.Rchnss2    0.998    0.066   15.133    0.000
##     Men.Sp.BdyMss2    0.998    0.066   15.133    0.000
##     X50km_Pop2        0.998    0.066   15.133    0.000
##     X10km_Pop2        0.998    0.066   15.133    0.000
##     GDP.Millions2     0.998    0.066   15.133    0.000
```

```
##     Prop.Mam.Sp2      0.998    0.066   15.133    0.000
##
## R-Square:
##                    Estimate
##     Attendance2       0.716
##     Total.Animals2    0.744
##     Sp.Richness2      0.845
##     Brilloun.Indx2    0.762
##     Mean.Rap.Crck2    0.258
```

```
# Generate fit indices
fitMeasures(mod.17.fit, c("agfi", "rmr", "srmr", "rmsea", "cfi", "nnfi", "tli"))
```

```
##  agfi   rmr  srmr rmsea   cfi  nnfi   tli
## 0.801 0.054 0.054 0.126 0.935 0.892 0.892
```

```
# Generate modification indices
mi17 <- modindices(mod.17.fit)
print(mi17[mi17$mi > 3.0,])
```

```
##                  lhs op              rhs     mi    epc sepc.lv sepc.all
## 56    Total.Animals2 ~~ Brillouin.Index2  5.584 -0.069  -0.069   -0.068
## 57    Total.Animals2 ~~ Mean.Raup.Crick2  9.956  0.074   0.074    0.075
## 58      Sp.Richness2 ~~ Brillouin.Index2 14.694  0.038   0.038    0.037
## 59      Sp.Richness2 ~~ Mean.Raup.Crick2 27.760 -0.088  -0.088   -0.088
## 64    Total.Animals2  ~      Attendance2  4.732  0.079   0.079    0.078
## 65    Total.Animals2  ~ Brillouin.Index2  5.366  0.188   0.188    0.194
## 66    Total.Animals2  ~ Mean.Raup.Crick2  7.996  0.080   0.080    0.081
## 69    Total.Animals2  ~       X50km_Pop2 35.817  0.144   0.144    0.145
## 70    Total.Animals2  ~       X10km_Pop2  8.876  0.074   0.074    0.075
## 71    Total.Animals2  ~     GDP.Millions2 29.903 -0.128  -0.128   -0.129
## 76      Sp.Richness2  ~ Mean.Raup.Crick2 22.422 -0.098  -0.098   -0.098
## 77      Sp.Richness2  ~      Zoo.Area.ha2 10.169  0.067   0.067    0.067
## 84  Brillouin.Index2  ~      Zoo.Area.ha2  5.584  0.061   0.061    0.059
## 88  Brillouin.Index2  ~       X10km_Pop2  5.673 -0.059  -0.059   -0.058
## 89  Brillouin.Index2  ~      Prop.Mam.Sp2  7.618  0.076   0.076    0.074
## 90  Mean.Raup.Crick2  ~      Attendance2 12.777  0.330   0.330    0.324
## 91  Mean.Raup.Crick2  ~      Sp.Richness2 21.560 -0.348  -0.348   -0.348
## 92  Mean.Raup.Crick2  ~ Brillouin.Index2 15.162 -0.175  -0.175   -0.180
## 93  Mean.Raup.Crick2  ~      Zoo.Area.ha2 12.585  0.181   0.181    0.181
## 94  Mean.Raup.Crick2  ~ Mam.Sp.Richness2 11.509 -0.199  -0.199   -0.199
## 95  Mean.Raup.Crick2  ~       X50km_Pop2 16.145  0.165   0.165    0.165
## 96  Mean.Raup.Crick2  ~       X10km_Pop2 12.485  0.149   0.149    0.149
## 98  Mean.Raup.Crick2  ~      Prop.Mam.Sp2 10.137 -0.179  -0.179   -0.179
## 99      Zoo.Area.ha2  ~      Attendance2 22.735  0.815   0.815    0.800
## 100     Zoo.Area.ha2  ~   Total.Animals2  5.439  0.198   0.198    0.196
## 101     Zoo.Area.ha2  ~      Sp.Richness2 10.153  0.300   0.300    0.300
## 102     Zoo.Area.ha2  ~ Brillouin.Index2  8.723  0.181   0.181    0.186
## 103     Zoo.Area.ha2  ~ Mean.Raup.Crick2 24.599  0.215   0.215    0.215
## 110 Mam.Sp.Richness2  ~      Attendance2 24.137 -1.050  -1.050   -1.031
## 111 Mam.Sp.Richness2  ~   Total.Animals2  4.672 -0.223  -0.223   -0.221
## 112 Mam.Sp.Richness2  ~      Sp.Richness2  3.181 -0.384  -0.384   -0.384
## 114 Mam.Sp.Richness2  ~ Mean.Raup.Crick2 35.260 -0.333  -0.333   -0.333
## 132       X50km_Pop2  ~      Attendance2 28.865  0.592   0.592    0.581
## 133       X50km_Pop2  ~   Total.Animals2 25.797  0.259   0.259    0.257
## 136       X50km_Pop2  ~ Mean.Raup.Crick2  6.268  0.086   0.086    0.086
```

```
## 144       X10km_Pop2  ~   Total.Animals2  4.400 -0.099  -0.099   -0.098
## 154    GDP.Millions2  ~      Attendance2 14.519 -0.650  -0.650   -0.638
## 155    GDP.Millions2  ~   Total.Animals2 16.231 -0.315  -0.315   -0.312
## 157    GDP.Millions2  ~ Brillouin.Index2 23.998  0.497   0.497    0.510
## 169     Prop.Mam.Sp2  ~ Mean.Raup.Crick2  4.520 -0.089  -0.089   -0.089
##     sepc.nox
## 56    -0.068
## 57     0.075
## 58     0.037
## 59    -0.088
## 64     0.078
## 65     0.194
## 66     0.081
## 69     0.145
## 70     0.075
## 71    -0.130
## 76    -0.098
## 77     0.067
## 84     0.059
## 88    -0.058
## 89     0.074
## 90     0.324
## 91    -0.348
## 92    -0.180
## 93     0.181
## 94    -0.200
## 95     0.165
## 96     0.149
## 98    -0.179
## 99     0.800
## 100    0.196
## 101    0.300
## 102    0.186
## 103    0.215
## 110   -1.031
## 111   -0.221
## 112   -0.384
## 114   -0.333
## 132    0.581
## 133    0.257
## 136    0.086
## 144   -0.098
## 154   -0.638
## 155   -0.312
## 157    0.510
## 169   -0.089
```

```
# Adjust for the nested nature of the data (institutions within countries)
# Fit model and generate model summary
design <- svydesign(ids = ~Country, nest=TRUE, data=sem_attendance_data)
fit.adj17 <- lavaan.survey(lavaan.fit = mod.17.fit, survey.design = design)
summary(fit.adj17, rsq = TRUE)
```

```
## lavaan (0.5-23.1097) converged normally after  34 iterations
##
```

```
##   Number of observations                           458
##
##   Estimator                                         ML      Robust
##   Minimum Function Test Statistic              223.977      65.772
##   Degrees of freedom                                27          27
##   P-value (Chi-square)                           0.000       0.000
##   Scaling correction factor                                  3.405
##     for the Satorra-Bentler correction
##
## Parameter Estimates:
##
##   Information                                 Expected
##   Standard Errors                           Robust.sem
##
## Regressions:
##                      Estimate  Std.Err  z-value  P(>|z|)
##   Attendance2 ~
##     Zoo.Area.ha2        0.073    0.036    2.027    0.043
##     Total.Animals2      0.361    0.034   10.737    0.000
##     Mam.Sp.Rchnss2      0.067    0.029    2.303    0.021
##     Men.Sp.BdyMss2      0.340    0.027   12.438    0.000
##     Mean.Rap.Crck2      0.144    0.026    5.467    0.000
##     X50km_Pop2          0.076    0.035    2.155    0.031
##     X10km_Pop2          0.374    0.042    8.832    0.000
##     GDP.Millions2       0.133    0.024    5.535    0.000
##   Total.Animals2 ~
##     Zoo.Area.ha2        0.223    0.036    6.235    0.000
##     Sp.Richness2        0.798    0.046   17.394    0.000
##   Sp.Richness2 ~
##     Prop.Mam.Sp2       -0.563    0.041  -13.858    0.000
##     Mam.Sp.Rchnss2      0.779    0.057   13.593    0.000
##   Brillouin.Index2 ~
##     Sp.Richness2        1.322    0.068   19.555    0.000
##     Total.Animals2     -0.622    0.102   -6.105    0.000
##     GDP.Millions2      -0.206    0.059   -3.485    0.000
##   Mean.Raup.Crick2 ~
##     Total.Animals2      0.259    0.062    4.214    0.000
##     Men.Sp.BdyMss2     -0.448    0.081   -5.512    0.000
##
## Covariances:
##                        Estimate  Std.Err  z-value  P(>|z|)
##  .Attendance2 ~~
##    .Brilloun.Indx2        0.017    0.017    0.978    0.328
##   Zoo.Area.ha2 ~~
##     Mam.Sp.Rchnss2        0.381    0.064    5.985    0.000
##     Men.Sp.BdyMss2        0.534    0.104    5.147    0.000
##     X50km_Pop2            0.060    0.069    0.880    0.379
##     X10km_Pop2           -0.010    0.078   -0.124    0.901
##     GDP.Millions2        -0.027    0.041   -0.673    0.501
##     Prop.Mam.Sp2          0.312    0.089    3.510    0.000
##   Mam.Sp.Richness2 ~~
##     Men.Sp.BdyMss2        0.303    0.081    3.730    0.000
##     X50km_Pop2            0.204    0.082    2.487    0.013
##     X10km_Pop2            0.284    0.066    4.278    0.000
```

```
##     GDP.Millions2        -0.060    0.064   -0.937    0.349
##     Prop.Mam.Sp2          0.088    0.061    1.435    0.151
##   Mean.Sp.BodyMass2 ~~
##     X50km_Pop2            0.101    0.072    1.400    0.161
##     X10km_Pop2            0.126    0.067    1.895    0.058
##     GDP.Millions2        -0.082    0.046   -1.759    0.079
##     Prop.Mam.Sp2          0.604    0.130    4.656    0.000
##   X50km_Pop2 ~~
##     X10km_Pop2            0.752    0.126    5.982    0.000
##     GDP.Millions2        -0.013    0.100   -0.131    0.896
##     Prop.Mam.Sp2         -0.096    0.051   -1.888    0.059
##   X10km_Pop2 ~~
##     GDP.Millions2        -0.028    0.056   -0.505    0.613
##     Prop.Mam.Sp2         -0.204    0.055   -3.685    0.000
##   GDP.Millions2 ~~
##     Prop.Mam.Sp2         -0.123    0.105   -1.172    0.241
##
## Intercepts:
##                    Estimate  Std.Err  z-value  P(>|z|)
##    .Attendance2      -0.000    0.030   -0.000    1.000
##    .Total.Animals2   -0.000    0.104   -0.000    1.000
##    .Sp.Richness2      0.000    0.032    0.000    1.000
##    .Brilloun.Indx2   -0.000    0.055   -0.000    1.000
##    .Mean.Rap.Crck2   -0.000    0.098   -0.000    1.000
##     Zoo.Area.ha2      0.000    0.064    0.000    1.000
##     Mam.Sp.Rchnss2   -0.000    0.079   -0.000    1.000
##     Men.Sp.BdyMss2   -0.000    0.080   -0.000    1.000
##     X50km_Pop2       -0.000    0.114   -0.000    1.000
##     X10km_Pop2        0.000    0.105    0.000    1.000
##     GDP.Millions2    -0.000    0.345   -0.000    1.000
##     Prop.Mam.Sp2      0.000    0.118    0.000    1.000
##
## Variances:
##                    Estimate  Std.Err  z-value  P(>|z|)
##    .Attendance2       0.273    0.029    9.569    0.000
##    .Total.Animals2    0.251    0.044    5.729    0.000
##    .Sp.Richness2      0.154    0.044    3.506    0.000
##    .Brilloun.Indx2    0.251    0.053    4.701    0.000
##    .Mean.Rap.Crck2    0.739    0.109    6.767    0.000
##     Zoo.Area.ha2      0.998    0.112    8.886    0.000
##     Mam.Sp.Rchnss2    0.998    0.115    8.669    0.000
##     Men.Sp.BdyMss2    0.998    0.173    5.783    0.000
##     X50km_Pop2        0.998    0.131    7.623    0.000
##     X10km_Pop2        0.998    0.174    5.745    0.000
##     GDP.Millions2     0.998    0.246    4.061    0.000
##     Prop.Mam.Sp2      0.998    0.150    6.662    0.000
##
## R-Square:
##                    Estimate
##     Attendance2       0.716
##     Total.Animals2    0.744
##     Sp.Richness2      0.845
##     Brilloun.Indx2    0.762
##     Mean.Rap.Crck2    0.258
```

```r
# Generate fit indices
fitMeasures(fit.adj17, c("agfi", "rmr", "srmr", "rmsea", "cfi", "nnfi", "tli"))
```

```
##  agfi   rmr  srmr rmsea   cfi  nnfi   tli
## 0.770 0.054 0.051 0.126 0.935 0.892 0.892
```

```r
# Generate modification indices
mi17adj <- modindices(fit.adj17)
print(mi17adj[mi17adj$mi > 3.0,])
```

```
##                  lhs op              rhs     mi mi.scaled    epc sepc.lv
## 68    Total.Animals2 ~~ Brillouin.Index2  5.584     1.640 -0.069  -0.069
## 69    Total.Animals2 ~~ Mean.Raup.Crick2  9.956     2.924  0.074   0.074
## 70      Sp.Richness2 ~~ Brillouin.Index2 14.694     4.315  0.038   0.038
## 71      Sp.Richness2 ~~ Mean.Raup.Crick2 27.760     8.152 -0.088  -0.088
## 76    Total.Animals2  ~      Attendance2  4.732     1.389  0.079   0.079
## 77    Total.Animals2  ~ Brillouin.Index2  5.366     1.576  0.188   0.188
## 78    Total.Animals2  ~ Mean.Raup.Crick2  7.996     2.348  0.080   0.080
## 81    Total.Animals2  ~       X50km_Pop2 35.817    10.518  0.144   0.144
## 82    Total.Animals2  ~       X10km_Pop2  8.876     2.606  0.074   0.074
## 83    Total.Animals2  ~    GDP.Millions2 29.903     8.781 -0.128  -0.128
## 88      Sp.Richness2  ~ Mean.Raup.Crick2 22.422     6.584 -0.098  -0.098
## 89      Sp.Richness2  ~      Zoo.Area.ha2 10.169     2.986  0.067   0.067
## 96  Brillouin.Index2  ~      Zoo.Area.ha2  5.584     1.640  0.061   0.061
## 100 Brillouin.Index2  ~       X10km_Pop2  5.673     1.666 -0.059  -0.059
## 101 Brillouin.Index2  ~      Prop.Mam.Sp2  7.618     2.237  0.076   0.076
## 102 Mean.Raup.Crick2  ~      Attendance2 12.777     3.752  0.330   0.330
## 103 Mean.Raup.Crick2  ~     Sp.Richness2 21.560     6.331 -0.348  -0.348
## 104 Mean.Raup.Crick2  ~ Brillouin.Index2 15.162     4.452 -0.175  -0.175
## 105 Mean.Raup.Crick2  ~      Zoo.Area.ha2 12.585     3.696  0.181   0.181
## 106 Mean.Raup.Crick2  ~ Mam.Sp.Richness2 11.509     3.380 -0.199  -0.199
## 107 Mean.Raup.Crick2  ~       X50km_Pop2 16.145     4.741  0.165   0.165
## 108 Mean.Raup.Crick2  ~       X10km_Pop2 12.485     3.666  0.149   0.149
## 110 Mean.Raup.Crick2  ~      Prop.Mam.Sp2 10.137     2.977 -0.179  -0.179
## 111      Zoo.Area.ha2  ~      Attendance2 22.735     6.676  0.815   0.815
## 112      Zoo.Area.ha2  ~    Total.Animals2  5.439     1.597  0.198   0.198
## 113      Zoo.Area.ha2  ~     Sp.Richness2 10.153     2.981  0.300   0.300
## 114      Zoo.Area.ha2  ~ Brillouin.Index2  8.723     2.562  0.181   0.181
## 115      Zoo.Area.ha2  ~ Mean.Raup.Crick2 24.599     7.224  0.215   0.215
## 122 Mam.Sp.Richness2  ~      Attendance2 24.136     7.088 -1.050  -1.050
## 123 Mam.Sp.Richness2  ~    Total.Animals2  4.672     1.372 -0.223  -0.223
## 124 Mam.Sp.Richness2  ~     Sp.Richness2  3.181     0.934 -0.384  -0.384
## 126 Mam.Sp.Richness2  ~ Mean.Raup.Crick2 35.260    10.354 -0.333  -0.333
## 144       X50km_Pop2  ~      Attendance2 28.865     8.476  0.592   0.592
## 145       X50km_Pop2  ~    Total.Animals2 25.797     7.575  0.259   0.259
## 148       X50km_Pop2  ~ Mean.Raup.Crick2  6.268     1.841  0.086   0.086
## 156       X10km_Pop2  ~    Total.Animals2  4.400     1.292 -0.099  -0.099
## 166    GDP.Millions2  ~      Attendance2 14.519     4.263 -0.650  -0.650
## 167    GDP.Millions2  ~    Total.Animals2 16.231     4.766 -0.315  -0.315
## 169    GDP.Millions2  ~ Brillouin.Index2 23.999     7.047  0.497   0.497
## 181      Prop.Mam.Sp2  ~ Mean.Raup.Crick2  4.520     1.327 -0.089  -0.089
##     sepc.all sepc.nox
## 68    -0.068   -0.068
## 69     0.075    0.075
## 70     0.037    0.037
```

```
## 71    -0.088   -0.088
## 76     0.078    0.078
## 77     0.194    0.194
## 78     0.081    0.081
## 81     0.145    0.145
## 82     0.075    0.075
## 83    -0.129   -0.130
## 88    -0.098   -0.098
## 89     0.067    0.067
## 96     0.059    0.059
## 100   -0.058   -0.058
## 101    0.074    0.074
## 102    0.324    0.324
## 103   -0.348   -0.348
## 104   -0.180   -0.180
## 105    0.181    0.181
## 106   -0.199   -0.200
## 107    0.165    0.165
## 108    0.149    0.149
## 110   -0.179   -0.179
## 111    0.800    0.800
## 112    0.196    0.196
## 113    0.300    0.300
## 114    0.186    0.186
## 115    0.215    0.215
## 122   -1.031   -1.031
## 123   -0.221   -0.221
## 124   -0.384   -0.384
## 126   -0.333   -0.333
## 144    0.581    0.581
## 145    0.257    0.257
## 148    0.086    0.086
## 156   -0.098   -0.098
## 166   -0.638   -0.638
## 167   -0.312   -0.312
## 169    0.510    0.510
## 181   -0.089   -0.089
```

Model 18

Based on the results generated from the nested seventeenth model, we see that **Attendance2 ~ X50km_Pop2** has a p-value of **0.031**, considering a lot of this data is captured in **Attendance2 ~ X10km_Pop2**, we we decide to remove this pathway. Once again, the model summary, fit indices and modification indices were all generated for the model, adjusting for the nested nature of data.

```
# Attendance SEM (Presence-Absence)

# Model 18
# Removal of Attendance2 ~ X50km_Pop2, p = 0.031

mod.18 <- 'Attendance2 ~ Zoo.Area.ha2 + Total.Animals2
+ Mam.Sp.Richness2 + Mean.Sp.BodyMass2 + Mean.Raup.Crick2
+ X10km_Pop2 + GDP.Millions2
```

```
Total.Animals2 ~ Zoo.Area.ha2 + Sp.Richness2
Sp.Richness2 ~ Prop.Mam.Sp2 + Mam.Sp.Richness2
Brillouin.Index2 ~ Sp.Richness2 + Total.Animals2 + GDP.Millions2
Mean.Raup.Crick2 ~ Total.Animals2 + Mean.Sp.BodyMass2'

# Fit model and generate model summary
mod.18.fit <- sem(mod.18, data = sem_attendance_data, fixed.x=FALSE)
summary(mod.18.fit, rsq = TRUE)
```

```
## lavaan (0.5-23.1097) converged normally after  33 iterations
##
##   Number of observations                           458
##
##   Estimator                                         ML
##   Minimum Function Test Statistic              179.753
##   Degrees of freedom                                23
##   P-value (Chi-square)                           0.000
##
## Parameter Estimates:
##
##   Information                                 Expected
##   Standard Errors                             Standard
##
## Regressions:
##                      Estimate  Std.Err  z-value  P(>|z|)
##   Attendance2 ~
##     Zoo.Area.ha2        0.075    0.032    2.363    0.018
##     Total.Animals2      0.371    0.037   10.058    0.000
##     Mam.Sp.Rchnss2      0.058    0.036    1.611    0.107
##     Men.Sp.BdyMss2      0.343    0.034   10.118    0.000
##     Mean.Rap.Crck2      0.149    0.028    5.237    0.000
##     X10km_Pop2          0.431    0.026   16.329    0.000
##     GDP.Millions2       0.134    0.025    5.420    0.000
##   Total.Animals2 ~
##     Zoo.Area.ha2        0.223    0.024    9.458    0.000
##     Sp.Richness2        0.798    0.024   33.781    0.000
##   Sp.Richness2 ~
##     Prop.Mam.Sp2       -0.563    0.018  -30.494    0.000
##     Mam.Sp.Rchnss2      0.779    0.018   42.208    0.000
##   Brillouin.Index2 ~
##     Sp.Richness2        1.324    0.042   31.337    0.000
##     Total.Animals2     -0.624    0.043  -14.620    0.000
##     GDP.Millions2      -0.207    0.023   -8.813    0.000
##   Mean.Raup.Crick2 ~
##     Total.Animals2      0.259    0.041    6.383    0.000
##     Men.Sp.BdyMss2     -0.448    0.040  -11.130    0.000
##
## Covariances:
##                        Estimate  Std.Err  z-value  P(>|z|)
##  .Attendance2 ~~
##    .Brilloun.Indx2        0.020    0.012    1.613    0.107
##   Zoo.Area.ha2 ~~
##     Mam.Sp.Rchnss2        0.381    0.050    7.634    0.000
```

```
##     Men.Sp.BdyMss2        0.534    0.053   10.096    0.000
##     X10km_Pop2           -0.010    0.047   -0.208    0.835
##     GDP.Millions2        -0.027    0.047   -0.588    0.557
##     Prop.Mam.Sp2          0.312    0.049    6.389    0.000
##   Mam.Sp.Richness2 ~~
##     Men.Sp.BdyMss2        0.303    0.049    6.216    0.000
##     X10km_Pop2            0.284    0.048    5.852    0.000
##     GDP.Millions2        -0.060    0.047   -1.277    0.202
##     Prop.Mam.Sp2          0.088    0.047    1.882    0.060
##   Mean.Sp.BodyMass2 ~~
##     X10km_Pop2            0.126    0.047    2.683    0.007
##     GDP.Millions2        -0.082    0.047   -1.748    0.080
##     Prop.Mam.Sp2          0.604    0.055   11.083    0.000
##   X10km_Pop2 ~~
##     GDP.Millions2        -0.028    0.047   -0.604    0.546
##     Prop.Mam.Sp2         -0.204    0.048   -4.293    0.000
##   GDP.Millions2 ~~
##     Prop.Mam.Sp2         -0.123    0.047   -2.615    0.009
##
## Variances:
##                    Estimate  Std.Err  z-value  P(>|z|)
##    .Attendance2       0.276    0.018   15.133    0.000
##    .Total.Animals2    0.251    0.017   15.133    0.000
##    .Sp.Richness2      0.154    0.010   15.133    0.000
##    .Brilloun.Indx2    0.251    0.017   15.133    0.000
##    .Mean.Rap.Crck2    0.739    0.049   15.133    0.000
##     Zoo.Area.ha2      0.998    0.066   15.133    0.000
##     Mam.Sp.Rchnss2    0.998    0.066   15.133    0.000
##     Men.Sp.BdyMss2    0.998    0.066   15.133    0.000
##     X10km_Pop2        0.998    0.066   15.133    0.000
##     GDP.Millions2     0.998    0.066   15.133    0.000
##     Prop.Mam.Sp2      0.998    0.066   15.133    0.000
##
## R-Square:
##                    Estimate
##     Attendance2       0.715
##     Total.Animals2    0.744
##     Sp.Richness2      0.845
##     Brilloun.Indx2    0.762
##     Mean.Rap.Crck2    0.258
```

```
# Generate fit indices
fitMeasures(mod.18.fit, c("agfi", "rmr", "srmr", "rmsea", "cfi", "nnfi", "tli"))
```

```
##  agfi   rmr  srmr rmsea   cfi  nnfi   tli
## 0.824 0.049 0.049 0.122 0.948 0.909 0.909
```

```
# Generate modification indices
mi18 <- modindices(mod.18.fit)
print(mi18[mi18$mi > 3.0,])
```

```
##                 lhs op               rhs     mi    epc sepc.lv sepc.all
## 48   Total.Animals2 ~~ Brillouin.Index2  5.630 -0.069  -0.069   -0.068
## 49   Total.Animals2 ~~ Mean.Raup.Crick2  9.956  0.074   0.074    0.075
## 50     Sp.Richness2 ~~ Brillouin.Index2 14.774  0.038   0.038    0.037
## 51     Sp.Richness2 ~~ Mean.Raup.Crick2 27.759 -0.088  -0.088   -0.088
```

```
## 56    Total.Animals2  ~      Attendance2  3.968  0.072   0.072    0.072
## 57    Total.Animals2  ~ Brillouin.Index2  5.587  0.191   0.191    0.198
## 58    Total.Animals2  ~ Mean.Raup.Crick2  7.996  0.080   0.080    0.081
## 61    Total.Animals2  ~       X10km_Pop2  8.876  0.074   0.074    0.075
## 62    Total.Animals2  ~     GDP.Millions2 29.903 -0.128  -0.128   -0.129
## 67      Sp.Richness2  ~ Mean.Raup.Crick2 22.422 -0.098  -0.098   -0.098
## 68      Sp.Richness2  ~      Zoo.Area.ha2 10.169  0.067   0.067    0.067
## 74  Brillouin.Index2  ~      Zoo.Area.ha2  5.630  0.061   0.061    0.060
## 77  Brillouin.Index2  ~       X10km_Pop2  5.691 -0.059  -0.059   -0.058
## 78  Brillouin.Index2  ~      Prop.Mam.Sp2  7.557  0.076   0.076    0.074
## 79  Mean.Raup.Crick2  ~       Attendance2 11.727  0.321   0.321    0.316
## 80  Mean.Raup.Crick2  ~      Sp.Richness2 21.560 -0.348  -0.348   -0.348
## 81  Mean.Raup.Crick2  ~ Brillouin.Index2 15.137 -0.175  -0.175   -0.180
## 82  Mean.Raup.Crick2  ~      Zoo.Area.ha2 12.585  0.181   0.181    0.181
## 83  Mean.Raup.Crick2  ~ Mam.Sp.Richness2 11.509 -0.199  -0.199   -0.199
## 84  Mean.Raup.Crick2  ~       X10km_Pop2 12.485  0.149   0.149    0.149
## 86  Mean.Raup.Crick2  ~      Prop.Mam.Sp2 10.137 -0.179  -0.179   -0.179
## 87      Zoo.Area.ha2  ~       Attendance2 34.157  0.985   0.985    0.970
## 88      Zoo.Area.ha2  ~    Total.Animals2 10.968  0.286   0.286    0.283
## 89      Zoo.Area.ha2  ~      Sp.Richness2 10.722  0.311   0.311    0.311
## 90      Zoo.Area.ha2  ~ Brillouin.Index2  8.230  0.177   0.177    0.182
## 91      Zoo.Area.ha2  ~ Mean.Raup.Crick2 28.663  0.234   0.234    0.234
## 97  Mam.Sp.Richness2  ~       Attendance2 28.440 -1.107  -1.107   -1.091
## 98  Mam.Sp.Richness2  ~    Total.Animals2  6.733 -0.268  -0.268   -0.266
## 99  Mam.Sp.Richness2  ~      Sp.Richness2  3.410 -0.400  -0.400   -0.400
## 101 Mam.Sp.Richness2  ~ Mean.Raup.Crick2 37.454 -0.344  -0.344   -0.344
## 117        X10km_Pop2  ~       Attendance2 12.986  0.469   0.469    0.462
## 118        X10km_Pop2  ~    Total.Animals2  6.086  0.173   0.173    0.171
## 120        X10km_Pop2  ~ Brillouin.Index2  5.318 -0.142  -0.142   -0.146
## 121        X10km_Pop2  ~ Mean.Raup.Crick2 19.900  0.211   0.211    0.211
## 127     GDP.Millions2  ~       Attendance2 13.086 -0.600  -0.600   -0.591
## 128     GDP.Millions2  ~    Total.Animals2 15.369 -0.306  -0.306   -0.304
## 130     GDP.Millions2  ~ Brillouin.Index2 23.795  0.494   0.494    0.508
## 141      Prop.Mam.Sp2  ~ Mean.Raup.Crick2  3.335 -0.077  -0.077   -0.077
##     sepc.nox
## 48    -0.068
## 49     0.075
## 50     0.037
## 51    -0.088
## 56     0.072
## 57     0.198
## 58     0.081
## 61     0.075
## 62    -0.130
## 67    -0.098
## 68     0.067
## 74     0.060
## 77    -0.058
## 78     0.074
## 79     0.316
## 80    -0.348
## 81    -0.180
## 82     0.181
## 83    -0.200
```

```
## 84     0.149
## 86    -0.179
## 87     0.970
## 88     0.283
## 89     0.311
## 90     0.182
## 91     0.234
## 97    -1.091
## 98    -0.266
## 99    -0.400
## 101   -0.344
## 117    0.462
## 118    0.171
## 120   -0.146
## 121    0.211
## 127   -0.591
## 128   -0.304
## 130    0.508
## 141   -0.077
```

```
# Adjust for the nested nature of the data (institutions within countries)
# Fit model and generate model summary
design <- svydesign(ids = ~Country, nest=TRUE, data=sem_attendance_data)
fit.adj18 <- lavaan.survey(lavaan.fit = mod.18.fit, survey.design = design)
summary(fit.adj18, rsq = TRUE)
```

```
## lavaan (0.5-23.1097) converged normally after  33 iterations
##
##   Number of observations                           458
##
##   Estimator                                         ML      Robust
##   Minimum Function Test Statistic              179.753      53.012
##   Degrees of freedom                                23          23
##   P-value (Chi-square)                           0.000       0.000
##   Scaling correction factor                                  3.391
##     for the Satorra-Bentler correction
##
## Parameter Estimates:
##
##   Information                                 Expected
##   Standard Errors                           Robust.sem
##
## Regressions:
##                      Estimate  Std.Err  z-value  P(>|z|)
##   Attendance2 ~
##     Zoo.Area.ha2        0.075    0.036    2.106    0.035
##     Total.Animals2      0.371    0.034   10.883    0.000
##     Mam.Sp.Rchnss2      0.058    0.030    1.947    0.052
##     Men.Sp.BdyMss2      0.343    0.028   12.442    0.000
##     Mean.Rap.Crck2      0.149    0.027    5.430    0.000
##     X10km_Pop2          0.431    0.034   12.558    0.000
##     GDP.Millions2       0.134    0.027    5.024    0.000
##   Total.Animals2 ~
##     Zoo.Area.ha2        0.223    0.036    6.235    0.000
##     Sp.Richness2        0.798    0.046   17.394    0.000
```

```
##   Sp.Richness2 ~
##     Prop.Mam.Sp2       -0.563    0.041  -13.858    0.000
##     Mam.Sp.Rchnss2      0.779    0.057   13.593    0.000
##   Brillouin.Index2 ~
##     Sp.Richness2        1.324    0.068   19.512    0.000
##     Total.Animals2     -0.624    0.102   -6.107    0.000
##     GDP.Millions2      -0.207    0.059   -3.481    0.001
##   Mean.Raup.Crick2 ~
##     Total.Animals2      0.259    0.062    4.214    0.000
##     Men.Sp.BdyMss2     -0.448    0.081   -5.512    0.000
##
## Covariances:
##                        Estimate  Std.Err  z-value  P(>|z|)
##  .Attendance2 ~~
##    .Brilloun.Indx2        0.020    0.017    1.183    0.237
##   Zoo.Area.ha2 ~~
##     Mam.Sp.Rchnss2        0.381    0.064    5.985    0.000
##     Men.Sp.BdyMss2        0.534    0.104    5.147    0.000
##     X10km_Pop2           -0.010    0.078   -0.124    0.901
##     GDP.Millions2        -0.027    0.041   -0.673    0.501
##     Prop.Mam.Sp2          0.312    0.089    3.510    0.000
##   Mam.Sp.Richness2 ~~
##     Men.Sp.BdyMss2        0.303    0.081    3.730    0.000
##     X10km_Pop2            0.284    0.066    4.278    0.000
##     GDP.Millions2        -0.060    0.064   -0.937    0.349
##     Prop.Mam.Sp2          0.088    0.061    1.435    0.151
##   Mean.Sp.BodyMass2 ~~
##     X10km_Pop2            0.126    0.067    1.895    0.058
##     GDP.Millions2        -0.082    0.046   -1.759    0.079
##     Prop.Mam.Sp2          0.604    0.130    4.656    0.000
##   X10km_Pop2 ~~
##     GDP.Millions2        -0.028    0.056   -0.505    0.613
##     Prop.Mam.Sp2         -0.204    0.055   -3.685    0.000
##   GDP.Millions2 ~~
##     Prop.Mam.Sp2         -0.123    0.105   -1.172    0.241
##
## Intercepts:
##                    Estimate  Std.Err  z-value  P(>|z|)
##    .Attendance2      -0.000    0.030   -0.000    1.000
##    .Total.Animals2   -0.000    0.104   -0.000    1.000
##    .Sp.Richness2      0.000    0.032    0.000    1.000
##    .Brilloun.Indx2   -0.000    0.055   -0.000    1.000
##    .Mean.Rap.Crck2   -0.000    0.098   -0.000    1.000
##     Zoo.Area.ha2      0.000    0.064    0.000    1.000
##     Mam.Sp.Rchnss2   -0.000    0.079   -0.000    1.000
##     Men.Sp.BdyMss2   -0.000    0.080   -0.000    1.000
##     X10km_Pop2        0.000    0.105    0.000    1.000
##     GDP.Millions2    -0.000    0.345   -0.000    1.000
##     Prop.Mam.Sp2      0.000    0.118    0.000    1.000
##
## Variances:
##                    Estimate  Std.Err  z-value  P(>|z|)
##    .Attendance2       0.276    0.028    9.883    0.000
##    .Total.Animals2    0.251    0.044    5.729    0.000
```

```
##    .Sp.Richness2      0.154    0.044    3.506    0.000
##    .Brilloun.Indx2    0.251    0.053    4.707    0.000
##    .Mean.Rap.Crck2    0.739    0.109    6.767    0.000
##     Zoo.Area.ha2      0.998    0.112    8.886    0.000
##     Mam.Sp.Rchnss2    0.998    0.115    8.669    0.000
##     Men.Sp.BdyMss2    0.998    0.173    5.783    0.000
##     X10km_Pop2        0.998    0.174    5.745    0.000
##     GDP.Millions2     0.998    0.246    4.061    0.000
##     Prop.Mam.Sp2      0.998    0.150    6.662    0.000
##
## R-Square:
##                    Estimate
##     Attendance2       0.715
##     Total.Animals2    0.744
##     Sp.Richness2      0.845
##     Brilloun.Indx2    0.762
##     Mean.Rap.Crck2    0.258
```

```
# Generate fit indices
fitMeasures(fit.adj18, c("agfi", "rmr", "srmr", "rmsea", "cfi", "nnfi", "tli"))
```

```
##  agfi   rmr  srmr rmsea   cfi  nnfi   tli
## 0.795 0.049 0.046 0.122 0.948 0.909 0.909
```

```
# Generate modification indices
mi18adj <- modindices(fit.adj18)
print(mi18adj[mi18adj$mi > 3.0,])
```

```
##                  lhs op              rhs     mi mi.scaled    epc sepc.lv
## 59    Total.Animals2 ~~ Brillouin.Index2  5.630     1.660 -0.069  -0.069
## 60    Total.Animals2 ~~ Mean.Raup.Crick2  9.956     2.936  0.074   0.074
## 61      Sp.Richness2 ~~ Brillouin.Index2 14.774     4.357  0.038   0.038
## 62      Sp.Richness2 ~~ Mean.Raup.Crick2 27.759     8.187 -0.088  -0.088
## 67    Total.Animals2  ~      Attendance2  3.968     1.170  0.072   0.072
## 68    Total.Animals2  ~ Brillouin.Index2  5.587     1.648  0.191   0.191
## 69    Total.Animals2  ~ Mean.Raup.Crick2  7.996     2.358  0.080   0.080
## 72    Total.Animals2  ~       X10km_Pop2  8.876     2.618  0.074   0.074
## 73    Total.Animals2  ~    GDP.Millions2 29.903     8.819 -0.128  -0.128
## 78      Sp.Richness2  ~ Mean.Raup.Crick2 22.422     6.613 -0.098  -0.098
## 79      Sp.Richness2  ~     Zoo.Area.ha2 10.169     2.999  0.067   0.067
## 85  Brillouin.Index2  ~     Zoo.Area.ha2  5.630     1.660  0.061   0.061
## 88  Brillouin.Index2  ~       X10km_Pop2  5.691     1.678 -0.059  -0.059
## 89  Brillouin.Index2  ~     Prop.Mam.Sp2  7.557     2.229  0.076   0.076
## 90  Mean.Raup.Crick2  ~      Attendance2 11.727     3.459  0.321   0.321
## 91  Mean.Raup.Crick2  ~     Sp.Richness2 21.560     6.358 -0.348  -0.348
## 92  Mean.Raup.Crick2  ~ Brillouin.Index2 15.137     4.464 -0.175  -0.175
## 93  Mean.Raup.Crick2  ~     Zoo.Area.ha2 12.585     3.712  0.181   0.181
## 94  Mean.Raup.Crick2  ~ Mam.Sp.Richness2 11.509     3.394 -0.199  -0.199
## 95  Mean.Raup.Crick2  ~       X10km_Pop2 12.485     3.682  0.149   0.149
## 97  Mean.Raup.Crick2  ~     Prop.Mam.Sp2 10.137     2.990 -0.179  -0.179
## 98      Zoo.Area.ha2  ~      Attendance2 34.157    10.073  0.985   0.985
## 99      Zoo.Area.ha2  ~   Total.Animals2 10.968     3.235  0.286   0.286
## 100     Zoo.Area.ha2  ~     Sp.Richness2 10.722     3.162  0.311   0.311
## 101     Zoo.Area.ha2  ~ Brillouin.Index2  8.230     2.427  0.177   0.177
## 102     Zoo.Area.ha2  ~ Mean.Raup.Crick2 28.663     8.453  0.234   0.234
## 108 Mam.Sp.Richness2  ~      Attendance2 28.440     8.387 -1.107  -1.107
```

```
## 109 Mam.Sp.Richness2  ~   Total.Animals2  6.733     1.986 -0.268  -0.268
## 110 Mam.Sp.Richness2  ~     Sp.Richness2  3.410     1.006 -0.400  -0.400
## 112 Mam.Sp.Richness2  ~ Mean.Raup.Crick2 37.454    11.046 -0.344  -0.344
## 128       X10km_Pop2  ~      Attendance2 12.986     3.830  0.469   0.469
## 129       X10km_Pop2  ~   Total.Animals2  6.086     1.795  0.173   0.173
## 131       X10km_Pop2  ~ Brillouin.Index2  5.318     1.568 -0.142  -0.142
## 132       X10km_Pop2  ~ Mean.Raup.Crick2 19.900     5.869  0.211   0.211
## 138    GDP.Millions2  ~      Attendance2 13.086     3.859 -0.600  -0.600
## 139    GDP.Millions2  ~   Total.Animals2 15.369     4.533 -0.306  -0.306
## 141    GDP.Millions2  ~ Brillouin.Index2 23.795     7.017  0.494   0.494
## 152     Prop.Mam.Sp2  ~ Mean.Raup.Crick2  3.335     0.984 -0.077  -0.077
##     sepc.all sepc.nox
## 59    -0.068   -0.068
## 60     0.075    0.075
## 61     0.037    0.037
## 62    -0.088   -0.088
## 67     0.072    0.072
## 68     0.198    0.198
## 69     0.081    0.081
## 72     0.075    0.075
## 73    -0.129   -0.130
## 78    -0.098   -0.098
## 79     0.067    0.067
## 85     0.060    0.060
## 88    -0.058   -0.058
## 89     0.074    0.074
## 90     0.316    0.316
## 91    -0.348   -0.348
## 92    -0.180   -0.180
## 93     0.181    0.181
## 94    -0.199   -0.200
## 95     0.149    0.149
## 97    -0.179   -0.179
## 98     0.970    0.970
## 99     0.283    0.283
## 100    0.311    0.311
## 101    0.182    0.182
## 102    0.234    0.234
## 108   -1.091   -1.091
## 109   -0.266   -0.266
## 110   -0.400   -0.400
## 112   -0.344   -0.344
## 128    0.462    0.462
## 129    0.171    0.171
## 131   -0.146   -0.146
## 132    0.211    0.211
## 138   -0.591   -0.591
## 139   -0.304   -0.304
## 141    0.508    0.508
## 152   -0.077   -0.077
```

Model 19

Based on the results generated from the nested eighteenth model, the highest p-value relationship to be considered for removal is **Attendance2 ~ Mam.Sp.Rchnss2** with a p-value of **0.052**. Therefore we decide to remove this pathway. Once again, the model summary, fit indices and modification indices were all generated for the model, adjusting for the nested nature of data.

```
# Attendance SEM (Presence-Absence)

# Model 19
# Removal of Attendance2 ~ Mam.Sp.Rchnss2, p = 0.052

mod.19 <- 'Attendance2 ~ Zoo.Area.ha2 + Total.Animals2
+ Mean.Sp.BodyMass2 + Mean.Raup.Crick2
+ X10km_Pop2 + GDP.Millions2

Total.Animals2 ~ Zoo.Area.ha2 + Sp.Richness2
Sp.Richness2 ~ Prop.Mam.Sp2 + Mam.Sp.Richness2
Brillouin.Index2 ~ Sp.Richness2 + Total.Animals2 + GDP.Millions2
Mean.Raup.Crick2 ~ Total.Animals2 + Mean.Sp.BodyMass2'

# Fit model and generate model summary
mod.19.fit <- sem(mod.19, data = sem_attendance_data, fixed.x=FALSE)
summary(mod.19.fit, rsq = TRUE)
```

```
## lavaan (0.5-23.1097) converged normally after  40 iterations
##
##   Number of observations                           458
##
##   Estimator                                         ML
##   Minimum Function Test Statistic              182.403
##   Degrees of freedom                                24
##   P-value (Chi-square)                           0.000
##
## Parameter Estimates:
##
##   Information                                 Expected
##   Standard Errors                             Standard
##
## Regressions:
##                     Estimate  Std.Err  z-value  P(>|z|)
##   Attendance2 ~
##     Zoo.Area.ha2       0.078    0.032    2.450    0.014
##     Total.Animals2     0.409    0.029   14.052    0.000
##     Men.Sp.BdyMss2     0.354    0.033   10.754    0.000
##     Mean.Rap.Crck2     0.140    0.029    4.922    0.000
##     X10km_Pop2         0.435    0.026   16.557    0.000
##     GDP.Millions2      0.135    0.025    5.463    0.000
##   Total.Animals2 ~
##     Zoo.Area.ha2       0.223    0.024    9.458    0.000
##     Sp.Richness2       0.798    0.024   33.781    0.000
##   Sp.Richness2 ~
##     Prop.Mam.Sp2      -0.563    0.018  -30.494    0.000
##     Mam.Sp.Rchnss2     0.779    0.018   42.208    0.000
##   Brillouin.Index2 ~
```

```
##     Sp.Richness2        1.321    0.042   31.289    0.000
##     Total.Animals2     -0.621    0.043  -14.568    0.000
##     GDP.Millions2      -0.206    0.023   -8.796    0.000
##   Mean.Raup.Crick2 ~
##     Total.Animals2      0.259    0.041    6.383    0.000
##     Men.Sp.BdyMss2     -0.448    0.040  -11.130    0.000
##
## Covariances:
##                        Estimate  Std.Err  z-value  P(>|z|)
##  .Attendance2 ~~
##    .Brilloun.Indx2        0.019    0.012    1.572    0.116
##   Zoo.Area.ha2 ~~
##     Men.Sp.BdyMss2        0.534    0.053   10.096    0.000
##     X10km_Pop2           -0.010    0.047   -0.208    0.835
##     GDP.Millions2        -0.027    0.047   -0.588    0.557
##     Prop.Mam.Sp2          0.312    0.049    6.389    0.000
##     Mam.Sp.Rchnss2        0.381    0.050    7.634    0.000
##   Mean.Sp.BodyMass2 ~~
##     X10km_Pop2            0.126    0.047    2.683    0.007
##     GDP.Millions2        -0.082    0.047   -1.748    0.080
##     Prop.Mam.Sp2          0.604    0.055   11.083    0.000
##     Mam.Sp.Rchnss2        0.303    0.049    6.216    0.000
##   X10km_Pop2 ~~
##     GDP.Millions2        -0.028    0.047   -0.604    0.546
##     Prop.Mam.Sp2         -0.204    0.048   -4.293    0.000
##     Mam.Sp.Rchnss2        0.284    0.048    5.852    0.000
##   GDP.Millions2 ~~
##     Prop.Mam.Sp2         -0.123    0.047   -2.615    0.009
##     Mam.Sp.Rchnss2       -0.060    0.047   -1.277    0.202
##   Prop.Mam.Sp2 ~~
##     Mam.Sp.Rchnss2        0.088    0.047    1.882    0.060
##
## Variances:
##                    Estimate  Std.Err  z-value  P(>|z|)
##    .Attendance2       0.277    0.018   15.133    0.000
##    .Total.Animals2    0.251    0.017   15.133    0.000
##    .Sp.Richness2      0.154    0.010   15.133    0.000
##    .Brilloun.Indx2    0.251    0.017   15.133    0.000
##    .Mean.Rap.Crck2    0.739    0.049   15.133    0.000
##     Zoo.Area.ha2      0.998    0.066   15.133    0.000
##     Men.Sp.BdyMss2    0.998    0.066   15.133    0.000
##     X10km_Pop2        0.998    0.066   15.133    0.000
##     GDP.Millions2     0.998    0.066   15.133    0.000
##     Prop.Mam.Sp2      0.998    0.066   15.133    0.000
##     Mam.Sp.Rchnss2    0.998    0.066   15.133    0.000
##
## R-Square:
##                    Estimate
##     Attendance2       0.713
##     Total.Animals2    0.744
##     Sp.Richness2      0.845
##     Brilloun.Indx2    0.762
##     Mean.Rap.Crck2    0.258
```

```r
# Generate fit indices
fitMeasures(mod.19.fit, c("agfi", "rmr", "srmr", "rmsea", "cfi", "nnfi", "tli"))
```

```
##  agfi   rmr  srmr rmsea   cfi  nnfi   tli
## 0.829 0.049 0.049 0.120 0.947 0.912 0.912
```

```r
# Generate modification indices
mi19 <- modindices(mod.19.fit)
print(mi19[mi19$mi > 3.0,])
```

```
##                  lhs op              rhs     mi    epc sepc.lv sepc.all
## 44       Attendance2 ~~     Sp.Richness2  3.391 -0.019  -0.019   -0.019
## 47    Total.Animals2 ~~ Brillouin.Index2  5.545 -0.068  -0.068   -0.067
## 48    Total.Animals2 ~~ Mean.Raup.Crick2  9.956  0.074   0.074    0.075
## 49      Sp.Richness2 ~~ Brillouin.Index2 15.197  0.039   0.039    0.038
## 50      Sp.Richness2 ~~ Mean.Raup.Crick2 27.759 -0.088  -0.088   -0.088
## 57    Total.Animals2  ~ Brillouin.Index2  5.159  0.184   0.184    0.190
## 58    Total.Animals2  ~ Mean.Raup.Crick2  7.996  0.080   0.080    0.081
## 60    Total.Animals2  ~       X10km_Pop2  8.876  0.074   0.074    0.075
## 61    Total.Animals2  ~    GDP.Millions2 29.903 -0.128  -0.128   -0.129
## 67      Sp.Richness2  ~ Mean.Raup.Crick2 22.422 -0.098  -0.098   -0.098
## 68      Sp.Richness2  ~     Zoo.Area.ha2 10.169  0.067   0.067    0.067
## 74  Brillouin.Index2  ~     Zoo.Area.ha2  5.545  0.061   0.061    0.059
## 76  Brillouin.Index2  ~       X10km_Pop2  5.656 -0.059  -0.059   -0.058
## 77  Brillouin.Index2  ~     Prop.Mam.Sp2  7.247  0.074   0.074    0.072
## 79  Mean.Raup.Crick2  ~      Attendance2 14.464  0.357   0.357    0.351
## 80  Mean.Raup.Crick2  ~     Sp.Richness2 21.560 -0.348  -0.348   -0.348
## 81  Mean.Raup.Crick2  ~ Brillouin.Index2 15.182 -0.176  -0.176   -0.180
## 82  Mean.Raup.Crick2  ~     Zoo.Area.ha2 12.585  0.181   0.181    0.181
## 83  Mean.Raup.Crick2  ~       X10km_Pop2 12.485  0.149   0.149    0.149
## 85  Mean.Raup.Crick2  ~     Prop.Mam.Sp2 10.137 -0.179  -0.179   -0.179
## 86  Mean.Raup.Crick2  ~ Mam.Sp.Richness2 11.509 -0.199  -0.199   -0.199
## 87      Zoo.Area.ha2  ~      Attendance2 18.338  0.617   0.617    0.606
## 88      Zoo.Area.ha2  ~   Total.Animals2 10.968  0.286   0.286    0.283
## 89      Zoo.Area.ha2  ~     Sp.Richness2 10.722  0.311   0.311    0.311
## 90      Zoo.Area.ha2  ~ Brillouin.Index2  8.151  0.177   0.177    0.181
## 91      Zoo.Area.ha2  ~ Mean.Raup.Crick2 28.663  0.234   0.234    0.234
## 107       X10km_Pop2  ~      Attendance2  9.985  0.388   0.388    0.382
## 108       X10km_Pop2  ~   Total.Animals2  6.086  0.173   0.173    0.171
## 110       X10km_Pop2  ~ Brillouin.Index2  5.330 -0.142  -0.142   -0.146
## 111       X10km_Pop2  ~ Mean.Raup.Crick2 19.900  0.211   0.211    0.211
## 117    GDP.Millions2  ~      Attendance2 12.176 -0.545  -0.545   -0.536
## 118    GDP.Millions2  ~   Total.Animals2 15.369 -0.306  -0.306   -0.304
## 120    GDP.Millions2  ~ Brillouin.Index2 23.739  0.495   0.495    0.508
## 131     Prop.Mam.Sp2  ~ Mean.Raup.Crick2  3.335 -0.077  -0.077   -0.077
## 138 Mam.Sp.Richness2  ~   Total.Animals2  6.733 -0.268  -0.268   -0.266
## 139 Mam.Sp.Richness2  ~     Sp.Richness2  3.410 -0.400  -0.400   -0.400
## 141 Mam.Sp.Richness2  ~ Mean.Raup.Crick2 37.454 -0.344  -0.344   -0.344
##     sepc.nox
## 44    -0.019
## 47    -0.067
## 48     0.075
## 49     0.038
## 50    -0.088
## 57     0.190
```

```
## 58     0.081
## 60     0.075
## 61    -0.130
## 67    -0.098
## 68     0.067
## 74     0.059
## 76    -0.058
## 77     0.072
## 79     0.351
## 80    -0.348
## 81    -0.180
## 82     0.181
## 83     0.149
## 85    -0.179
## 86    -0.200
## 87     0.606
## 88     0.283
## 89     0.311
## 90     0.181
## 91     0.234
## 107    0.382
## 108    0.171
## 110   -0.146
## 111    0.211
## 117   -0.536
## 118   -0.304
## 120    0.508
## 131   -0.077
## 138   -0.266
## 139   -0.400
## 141   -0.344
```

```
# Adjust for the nested nature of the data (institutions within countries)
# Fit model and generate model summary
design <- svydesign(ids = ~Country, nest=TRUE, data=sem_attendance_data)
fit.adj19 <- lavaan.survey(lavaan.fit = mod.19.fit, survey.design = design)
summary(fit.adj19, rsq = TRUE)
```

```
## lavaan (0.5-23.1097) converged normally after  39 iterations
##
##   Number of observations                           458
##
##   Estimator                                         ML      Robust
##   Minimum Function Test Statistic              182.403      55.549
##   Degrees of freedom                                24          24
##   P-value (Chi-square)                           0.000       0.000
##   Scaling correction factor                                  3.284
##     for the Satorra-Bentler correction
##
## Parameter Estimates:
##
##   Information                                 Expected
##   Standard Errors                           Robust.sem
##
## Regressions:
```

```
##                     Estimate  Std.Err  z-value  P(>|z|)
##   Attendance2 ~
##     Zoo.Area.ha2       0.078    0.037    2.099    0.036
##     Total.Animals2     0.409    0.026   16.002    0.000
##     Men.Sp.BdyMss2     0.354    0.029   12.024    0.000
##     Mean.Rap.Crck2     0.140    0.027    5.157    0.000
##     X10km_Pop2         0.435    0.034   12.832    0.000
##     GDP.Millions2      0.135    0.026    5.256    0.000
##   Total.Animals2 ~
##     Zoo.Area.ha2       0.223    0.036    6.235    0.000
##     Sp.Richness2       0.798    0.046   17.394    0.000
##   Sp.Richness2 ~
##     Prop.Mam.Sp2      -0.563    0.041  -13.858    0.000
##     Mam.Sp.Rchnss2     0.779    0.057   13.593    0.000
##   Brillouin.Index2 ~
##     Sp.Richness2       1.321    0.068   19.524    0.000
##     Total.Animals2    -0.621    0.102   -6.086    0.000
##     GDP.Millions2     -0.206    0.059   -3.489    0.000
##   Mean.Raup.Crick2 ~
##     Total.Animals2     0.259    0.062    4.214    0.000
##     Men.Sp.BdyMss2    -0.448    0.081   -5.512    0.000
##
## Covariances:
##                       Estimate  Std.Err  z-value  P(>|z|)
##  .Attendance2 ~~
##    .Brilloun.Indx2       0.019    0.017    1.129    0.259
##   Zoo.Area.ha2 ~~
##     Men.Sp.BdyMss2       0.534    0.104    5.147    0.000
##     X10km_Pop2          -0.010    0.078   -0.124    0.901
##     GDP.Millions2       -0.027    0.041   -0.673    0.501
##     Prop.Mam.Sp2         0.312    0.089    3.510    0.000
##     Mam.Sp.Rchnss2       0.381    0.064    5.985    0.000
##   Mean.Sp.BodyMass2 ~~
##     X10km_Pop2           0.126    0.067    1.895    0.058
##     GDP.Millions2       -0.082    0.046   -1.759    0.079
##     Prop.Mam.Sp2         0.604    0.130    4.656    0.000
##     Mam.Sp.Rchnss2       0.303    0.081    3.730    0.000
##   X10km_Pop2 ~~
##     GDP.Millions2       -0.028    0.056   -0.505    0.613
##     Prop.Mam.Sp2        -0.204    0.055   -3.685    0.000
##     Mam.Sp.Rchnss2       0.284    0.066    4.278    0.000
##   GDP.Millions2 ~~
##     Prop.Mam.Sp2        -0.123    0.105   -1.172    0.241
##     Mam.Sp.Rchnss2      -0.060    0.064   -0.937    0.349
##   Prop.Mam.Sp2 ~~
##     Mam.Sp.Rchnss2       0.088    0.061    1.435    0.151
##
## Intercepts:
##                    Estimate  Std.Err  z-value  P(>|z|)
##    .Attendance2      -0.000    0.029   -0.000    1.000
##    .Total.Animals2   -0.000    0.104   -0.000    1.000
##    .Sp.Richness2      0.000    0.032    0.000    1.000
##    .Brilloun.Indx2   -0.000    0.055   -0.000    1.000
##    .Mean.Rap.Crck2   -0.000    0.098   -0.000    1.000
```

```
##     Zoo.Area.ha2      0.000    0.064    0.000    1.000
##     Men.Sp.BdyMss2   -0.000    0.080   -0.000    1.000
##     X10km_Pop2        0.000    0.105    0.000    1.000
##     GDP.Millions2    -0.000    0.345   -0.000    1.000
##     Prop.Mam.Sp2      0.000    0.118    0.000    1.000
##     Mam.Sp.Rchnss2   -0.000    0.079   -0.000    1.000
##
## Variances:
##                    Estimate  Std.Err  z-value  P(>|z|)
##    .Attendance2       0.277    0.028   10.043    0.000
##    .Total.Animals2    0.251    0.044    5.729    0.000
##    .Sp.Richness2      0.154    0.044    3.506    0.000
##    .Brilloun.Indx2    0.251    0.053    4.695    0.000
##    .Mean.Rap.Crck2    0.739    0.109    6.767    0.000
##     Zoo.Area.ha2      0.998    0.112    8.886    0.000
##     Men.Sp.BdyMss2    0.998    0.173    5.783    0.000
##     X10km_Pop2        0.998    0.174    5.745    0.000
##     GDP.Millions2     0.998    0.246    4.061    0.000
##     Prop.Mam.Sp2      0.998    0.150    6.662    0.000
##     Mam.Sp.Rchnss2    0.998    0.115    8.669    0.000
##
## R-Square:
##                    Estimate
##     Attendance2       0.713
##     Total.Animals2    0.744
##     Sp.Richness2      0.845
##     Brilloun.Indx2    0.762
##     Mean.Rap.Crck2    0.258
```

```
# Generate fit indices
fitMeasures(fit.adj19, c("agfi", "rmr", "srmr", "rmsea", "cfi", "nnfi", "tli"))
```

```
##  agfi   rmr  srmr rmsea   cfi  nnfi   tli
## 0.800 0.049 0.045 0.120 0.947 0.912 0.912
```

```
# Generate modification indices
mi19adj <- modindices(fit.adj19)
print(mi19adj[mi19adj$mi > 3.0,])
```

```
##                  lhs op              rhs     mi mi.scaled    epc sepc.lv
## 55       Attendance2 ~~     Sp.Richness2  3.391     1.033 -0.019  -0.019
## 58    Total.Animals2 ~~ Brillouin.Index2  5.545     1.689 -0.068  -0.068
## 59    Total.Animals2 ~~ Mean.Raup.Crick2  9.956     3.032  0.074   0.074
## 60      Sp.Richness2 ~~ Brillouin.Index2 15.198     4.628  0.039   0.039
## 61      Sp.Richness2 ~~ Mean.Raup.Crick2 27.759     8.454 -0.088  -0.088
## 68    Total.Animals2  ~ Brillouin.Index2  5.159     1.571  0.184   0.184
## 69    Total.Animals2  ~ Mean.Raup.Crick2  7.996     2.435  0.080   0.080
## 71    Total.Animals2  ~       X10km_Pop2  8.876     2.703  0.074   0.074
## 72    Total.Animals2  ~    GDP.Millions2 29.903     9.107 -0.128  -0.128
## 78      Sp.Richness2  ~ Mean.Raup.Crick2 22.422     6.829 -0.098  -0.098
## 79      Sp.Richness2  ~     Zoo.Area.ha2 10.169     3.097  0.067   0.067
## 85  Brillouin.Index2  ~     Zoo.Area.ha2  5.545     1.689  0.061   0.061
## 87  Brillouin.Index2  ~       X10km_Pop2  5.656     1.723 -0.059  -0.059
## 88  Brillouin.Index2  ~     Prop.Mam.Sp2  7.247     2.207  0.074   0.074
## 90  Mean.Raup.Crick2  ~      Attendance2 14.465     4.405  0.357   0.357
## 91  Mean.Raup.Crick2  ~     Sp.Richness2 21.560     6.566 -0.348  -0.348
```

```
## 92  Mean.Raup.Crick2  ~ Brillouin.Index2 15.182     4.624 -0.176  -0.176
## 93  Mean.Raup.Crick2  ~     Zoo.Area.ha2 12.585     3.833  0.181   0.181
## 94  Mean.Raup.Crick2  ~       X10km_Pop2 12.485     3.802  0.149   0.149
## 96  Mean.Raup.Crick2  ~     Prop.Mam.Sp2 10.137     3.087 -0.179  -0.179
## 97  Mean.Raup.Crick2  ~ Mam.Sp.Richness2 11.509     3.505 -0.199  -0.199
## 98      Zoo.Area.ha2  ~      Attendance2 18.338     5.585  0.617   0.617
## 99      Zoo.Area.ha2  ~   Total.Animals2 10.968     3.340  0.286   0.286
## 100     Zoo.Area.ha2  ~     Sp.Richness2 10.722     3.265  0.311   0.311
## 101     Zoo.Area.ha2  ~ Brillouin.Index2  8.151     2.482  0.177   0.177
## 102     Zoo.Area.ha2  ~ Mean.Raup.Crick2 28.663     8.729  0.234   0.234
## 118       X10km_Pop2  ~      Attendance2  9.985     3.041  0.388   0.388
## 119       X10km_Pop2  ~   Total.Animals2  6.086     1.853  0.173   0.173
## 121       X10km_Pop2  ~ Brillouin.Index2  5.330     1.623 -0.142  -0.142
## 122       X10km_Pop2  ~ Mean.Raup.Crick2 19.900     6.060  0.211   0.211
## 128    GDP.Millions2  ~      Attendance2 12.177     3.708 -0.545  -0.545
## 129    GDP.Millions2  ~   Total.Animals2 15.369     4.681 -0.306  -0.306
## 131    GDP.Millions2  ~ Brillouin.Index2 23.739     7.230  0.495   0.495
## 142     Prop.Mam.Sp2  ~ Mean.Raup.Crick2  3.335     1.016 -0.077  -0.077
## 149 Mam.Sp.Richness2  ~   Total.Animals2  6.733     2.051 -0.268  -0.268
## 150 Mam.Sp.Richness2  ~     Sp.Richness2  3.410     1.038 -0.400  -0.400
## 152 Mam.Sp.Richness2  ~ Mean.Raup.Crick2 37.454    11.406 -0.344  -0.344
##     sepc.all sepc.nox
## 55    -0.019   -0.019
## 58    -0.067   -0.067
## 59     0.075    0.075
## 60     0.038    0.038
## 61    -0.088   -0.088
## 68     0.190    0.190
## 69     0.081    0.081
## 71     0.075    0.075
## 72    -0.129   -0.130
## 78    -0.098   -0.098
## 79     0.067    0.067
## 85     0.059    0.059
## 87    -0.058   -0.058
## 88     0.072    0.072
## 90     0.351    0.351
## 91    -0.348   -0.348
## 92    -0.180   -0.180
## 93     0.181    0.181
## 94     0.149    0.149
## 96    -0.179   -0.179
## 97    -0.199   -0.200
## 98     0.606    0.606
## 99     0.283    0.283
## 100    0.311    0.311
## 101    0.181    0.181
## 102    0.234    0.234
## 118    0.382    0.382
## 119    0.171    0.171
## 121   -0.146   -0.146
## 122    0.211    0.211
## 128   -0.536   -0.536
## 129   -0.304   -0.304
```

```
## 131    0.508    0.508
## 142   -0.077   -0.077
## 149   -0.266   -0.266
## 150   -0.400   -0.400
## 152   -0.344   -0.344
```

Model 20

Based on the fact that Brillouin Index is no longer contributing to visitor attendance, it was decided to remove the from the analysis completely in a semi-exploratory manner. Therefore we decide to remove the three pathways associated with this parameter. Once again, the model summary, fit indices and modification indices were all generated for the model, adjusting for the nested nature of data.

```
# Attendance SEM (Presence-Absence)

# Model 20
# Removal of Brillouin.Index2 ~ Sp.Richness2 + Total.Animals2 + GDP.Millions2

mod.20 <- 'Attendance2 ~ Zoo.Area.ha2 + Total.Animals2
+ Mean.Sp.BodyMass2 + Mean.Raup.Crick2
+ X10km_Pop2 + GDP.Millions2

Total.Animals2 ~ Zoo.Area.ha2 + Sp.Richness2
Sp.Richness2 ~ Prop.Mam.Sp2 + Mam.Sp.Richness2
Mean.Raup.Crick2 ~ Total.Animals2 + Mean.Sp.BodyMass2'

# Fit model and generate model summary
mod.20.fit <- sem(mod.20, data = sem_attendance_data, fixed.x=FALSE)
summary(mod.20.fit, rsq = TRUE)
```

```
## lavaan (0.5-23.1097) converged normally after  28 iterations
##
##   Number of observations                           458
##
##   Estimator                                         ML
##   Minimum Function Test Statistic              157.850
##   Degrees of freedom                                18
##   P-value (Chi-square)                           0.000
##
## Parameter Estimates:
##
##   Information                                 Expected
##   Standard Errors                             Standard
##
## Regressions:
##                    Estimate  Std.Err  z-value  P(>|z|)
##   Attendance2 ~
##     Zoo.Area.ha2      0.082    0.032    2.570    0.010
##     Total.Animals2    0.409    0.029   14.043    0.000
##     Men.Sp.BdyMss2    0.353    0.033   10.711    0.000
##     Mean.Rap.Crck2    0.139    0.029    4.876    0.000
##     X10km_Pop2        0.431    0.026   16.370    0.000
##     GDP.Millions2     0.135    0.025    5.462    0.000
##   Total.Animals2 ~
```

```
##     Zoo.Area.ha2        0.223    0.024    9.458    0.000
##     Sp.Richness2        0.798    0.024   33.781    0.000
##   Sp.Richness2 ~
##     Prop.Mam.Sp2       -0.563    0.018  -30.494    0.000
##     Mam.Sp.Rchnss2      0.779    0.018   42.208    0.000
##   Mean.Raup.Crick2 ~
##     Total.Animals2      0.259    0.041    6.383    0.000
##     Men.Sp.BdyMss2     -0.448    0.040  -11.130    0.000
##
## Covariances:
##                       Estimate  Std.Err  z-value  P(>|z|)
##   Zoo.Area.ha2 ~~
##     Men.Sp.BdyMss2        0.534    0.053   10.096    0.000
##     X10km_Pop2           -0.010    0.047   -0.208    0.835
##     GDP.Millions2        -0.027    0.047   -0.588    0.557
##     Prop.Mam.Sp2          0.312    0.049    6.389    0.000
##     Mam.Sp.Rchnss2        0.381    0.050    7.634    0.000
##   Mean.Sp.BodyMass2 ~~
##     X10km_Pop2            0.126    0.047    2.683    0.007
##     GDP.Millions2        -0.082    0.047   -1.748    0.080
##     Prop.Mam.Sp2          0.604    0.055   11.083    0.000
##     Mam.Sp.Rchnss2        0.303    0.049    6.216    0.000
##   X10km_Pop2 ~~
##     GDP.Millions2        -0.028    0.047   -0.604    0.546
##     Prop.Mam.Sp2         -0.204    0.048   -4.293    0.000
##     Mam.Sp.Rchnss2        0.284    0.048    5.852    0.000
##   GDP.Millions2 ~~
##     Prop.Mam.Sp2         -0.123    0.047   -2.615    0.009
##     Mam.Sp.Rchnss2       -0.060    0.047   -1.277    0.202
##   Prop.Mam.Sp2 ~~
##     Mam.Sp.Rchnss2        0.088    0.047    1.882    0.060
##
## Variances:
##                     Estimate  Std.Err  z-value  P(>|z|)
##    .Attendance2        0.277    0.018   15.133    0.000
##    .Total.Animals2     0.251    0.017   15.133    0.000
##    .Sp.Richness2       0.154    0.010   15.133    0.000
##    .Mean.Rap.Crck2     0.739    0.049   15.133    0.000
##     Zoo.Area.ha2       0.998    0.066   15.133    0.000
##     Men.Sp.BdyMss2     0.998    0.066   15.133    0.000
##     X10km_Pop2         0.998    0.066   15.133    0.000
##     GDP.Millions2      0.998    0.066   15.133    0.000
##     Prop.Mam.Sp2       0.998    0.066   15.133    0.000
##     Mam.Sp.Rchnss2     0.998    0.066   15.133    0.000
##
## R-Square:
##                     Estimate
##     Attendance2        0.712
##     Total.Animals2     0.744
##     Sp.Richness2       0.845
##     Mean.Rap.Crck2     0.258
```

```r
# Generate fit indices
fitMeasures(mod.20.fit, c("agfi", "rmr", "srmr", "rmsea", "cfi", "nnfi", "tli"))
```

```
##  agfi   rmr  srmr rmsea   cfi  nnfi   tli
## 0.822 0.043 0.043 0.130 0.940 0.900 0.900
```

```r
# Generate modification indices
mi20 <- modindices(mod.20.fit)
print(mi20[mi20$mi > 3.0,])
```

```
##                  lhs op              rhs     mi    epc sepc.lv sepc.all
## 42    Total.Animals2 ~~ Mean.Raup.Crick2  9.956  0.074   0.074    0.075
## 43      Sp.Richness2 ~~ Mean.Raup.Crick2 27.760 -0.088  -0.088   -0.088
## 48    Total.Animals2  ~ Mean.Raup.Crick2  7.996  0.080   0.080    0.081
## 50    Total.Animals2  ~       X10km_Pop2  8.876  0.074   0.074    0.075
## 51    Total.Animals2  ~    GDP.Millions2 29.903 -0.128  -0.128   -0.129
## 56      Sp.Richness2  ~ Mean.Raup.Crick2 22.422 -0.098  -0.098   -0.098
## 57      Sp.Richness2  ~      Zoo.Area.ha2 10.169  0.067   0.067    0.067
## 61  Mean.Raup.Crick2  ~      Attendance2 15.151  0.370   0.370    0.364
## 62  Mean.Raup.Crick2  ~     Sp.Richness2 21.560 -0.348  -0.348   -0.348
## 63  Mean.Raup.Crick2  ~      Zoo.Area.ha2 12.585  0.181   0.181    0.181
## 64  Mean.Raup.Crick2  ~       X10km_Pop2 12.485  0.149   0.149    0.149
## 66  Mean.Raup.Crick2  ~      Prop.Mam.Sp2 10.137 -0.179  -0.179   -0.179
## 67  Mean.Raup.Crick2  ~ Mam.Sp.Richness2 11.509 -0.199  -0.199   -0.199
## 68      Zoo.Area.ha2  ~      Attendance2 16.535  0.593   0.593    0.582
## 69      Zoo.Area.ha2  ~   Total.Animals2 10.968  0.286   0.286    0.283
## 70      Zoo.Area.ha2  ~     Sp.Richness2 10.722  0.311   0.311    0.311
## 71      Zoo.Area.ha2  ~ Mean.Raup.Crick2 28.663  0.234   0.234    0.234
## 86        X10km_Pop2  ~      Attendance2 11.547  0.420   0.420    0.413
## 87        X10km_Pop2  ~   Total.Animals2  6.086  0.173   0.173    0.171
## 89        X10km_Pop2  ~ Mean.Raup.Crick2 19.900  0.211   0.211    0.211
## 95     GDP.Millions2  ~      Attendance2 12.199 -0.547  -0.547   -0.537
## 96     GDP.Millions2  ~   Total.Animals2 15.369 -0.306  -0.306   -0.304
## 107     Prop.Mam.Sp2  ~ Mean.Raup.Crick2  3.335 -0.077  -0.077   -0.077
## 114 Mam.Sp.Richness2  ~   Total.Animals2  6.733 -0.268  -0.268   -0.266
## 115 Mam.Sp.Richness2  ~     Sp.Richness2  3.410 -0.400  -0.400   -0.400
## 116 Mam.Sp.Richness2  ~ Mean.Raup.Crick2 37.454 -0.344  -0.344   -0.344
##     sepc.nox
## 42     0.075
## 43    -0.088
## 48     0.081
## 50     0.075
## 51    -0.130
## 56    -0.098
## 57     0.067
## 61     0.364
## 62    -0.348
## 63     0.181
## 64     0.149
## 66    -0.179
## 67    -0.200
## 68     0.582
## 69     0.283
## 70     0.311
## 71     0.234
## 86     0.413
## 87     0.171
## 89     0.211
```

```
## 95    -0.537
## 96    -0.304
## 107   -0.077
## 114   -0.266
## 115   -0.400
## 116   -0.344
```

```
# Adjust for the nested nature of the data (institutions within countries)
# Fit model and generate model summary
design <- svydesign(ids = ~Country, nest=TRUE, data=sem_attendance_data)
fit.adj20 <- lavaan.survey(lavaan.fit = mod.20.fit, survey.design = design)
summary(fit.adj20, rsq = TRUE)
```

```
## lavaan (0.5-23.1097) converged normally after  28 iterations
##
##   Number of observations                           458
##
##   Estimator                                         ML      Robust
##   Minimum Function Test Statistic              157.850      39.123
##   Degrees of freedom                                18          18
##   P-value (Chi-square)                           0.000       0.003
##   Scaling correction factor                                  4.035
##     for the Satorra-Bentler correction
##
## Parameter Estimates:
##
##   Information                                 Expected
##   Standard Errors                           Robust.sem
##
## Regressions:
##                        Estimate  Std.Err  z-value  P(>|z|)
##   Attendance2 ~
##     Zoo.Area.ha2          0.082    0.038    2.161    0.031
##     Total.Animals2        0.409    0.026   15.858    0.000
##     Men.Sp.BdyMss2        0.353    0.030   11.790    0.000
##     Mean.Rap.Crck2        0.139    0.028    5.042    0.000
##     X10km_Pop2            0.431    0.033   13.120    0.000
##     GDP.Millions2         0.135    0.026    5.274    0.000
##   Total.Animals2 ~
##     Zoo.Area.ha2          0.223    0.036    6.235    0.000
##     Sp.Richness2          0.798    0.046   17.394    0.000
##   Sp.Richness2 ~
##     Prop.Mam.Sp2         -0.563    0.041  -13.858    0.000
##     Mam.Sp.Rchnss2        0.779    0.057   13.593    0.000
##   Mean.Raup.Crick2 ~
##     Total.Animals2        0.259    0.062    4.214    0.000
##     Men.Sp.BdyMss2       -0.448    0.081   -5.512    0.000
##
## Covariances:
##                          Estimate  Std.Err  z-value  P(>|z|)
##   Zoo.Area.ha2 ~~
##     Men.Sp.BdyMss2          0.534    0.104    5.147    0.000
##     X10km_Pop2             -0.010    0.078   -0.124    0.901
##     GDP.Millions2          -0.027    0.041   -0.673    0.501
##     Prop.Mam.Sp2            0.312    0.089    3.510    0.000
```

```
##     Mam.Sp.Rchnss2        0.381    0.064    5.985    0.000
##   Mean.Sp.BodyMass2 ~~
##     X10km_Pop2            0.126    0.067    1.895    0.058
##     GDP.Millions2        -0.082    0.046   -1.759    0.079
##     Prop.Mam.Sp2          0.604    0.130    4.656    0.000
##     Mam.Sp.Rchnss2        0.303    0.081    3.730    0.000
##   X10km_Pop2 ~~
##     GDP.Millions2        -0.028    0.056   -0.505    0.613
##     Prop.Mam.Sp2         -0.204    0.055   -3.685    0.000
##     Mam.Sp.Rchnss2        0.284    0.066    4.278    0.000
##   GDP.Millions2 ~~
##     Prop.Mam.Sp2         -0.123    0.105   -1.172    0.241
##     Mam.Sp.Rchnss2       -0.060    0.064   -0.937    0.349
##   Prop.Mam.Sp2 ~~
##     Mam.Sp.Rchnss2        0.088    0.061    1.435    0.151
##
## Intercepts:
##                    Estimate  Std.Err  z-value  P(>|z|)
##    .Attendance2      -0.000    0.029   -0.000    1.000
##    .Total.Animals2   -0.000    0.104   -0.000    1.000
##    .Sp.Richness2      0.000    0.032    0.000    1.000
##    .Mean.Rap.Crck2   -0.000    0.098   -0.000    1.000
##     Zoo.Area.ha2      0.000    0.064    0.000    1.000
##     Men.Sp.BdyMss2   -0.000    0.080   -0.000    1.000
##     X10km_Pop2        0.000    0.105    0.000    1.000
##     GDP.Millions2    -0.000    0.345   -0.000    1.000
##     Prop.Mam.Sp2      0.000    0.118    0.000    1.000
##     Mam.Sp.Rchnss2   -0.000    0.079   -0.000    1.000
##
## Variances:
##                    Estimate  Std.Err  z-value  P(>|z|)
##    .Attendance2       0.277    0.028   10.008    0.000
##    .Total.Animals2    0.251    0.044    5.729    0.000
##    .Sp.Richness2      0.154    0.044    3.506    0.000
##    .Mean.Rap.Crck2    0.739    0.109    6.767    0.000
##     Zoo.Area.ha2      0.998    0.112    8.886    0.000
##     Men.Sp.BdyMss2    0.998    0.173    5.783    0.000
##     X10km_Pop2        0.998    0.174    5.745    0.000
##     GDP.Millions2     0.998    0.246    4.061    0.000
##     Prop.Mam.Sp2      0.998    0.150    6.662    0.000
##     Mam.Sp.Rchnss2    0.998    0.115    8.669    0.000
##
## R-Square:
##                    Estimate
##     Attendance2       0.712
##     Total.Animals2    0.744
##     Sp.Richness2      0.845
##     Mean.Rap.Crck2    0.258
```

```r
# Generate fit indices
fitMeasures(fit.adj20, c("agfi", "rmr", "srmr", "rmsea", "cfi", "nnfi", "tli"))
```

```
##  agfi   rmr  srmr rmsea   cfi  nnfi   tli
## 0.790 0.043 0.039 0.130 0.940 0.900 0.900
```

```r
# Generate modification indices
mi20adj <- modindices(fit.adj20)
print(mi20adj[mi20adj$mi > 3.0,])
```

```
##                  lhs op              rhs     mi mi.scaled    epc sepc.lv
## 52     Total.Animals2 ~~ Mean.Raup.Crick2  9.956     2.468  0.074   0.074
## 53       Sp.Richness2 ~~ Mean.Raup.Crick2 27.760     6.880 -0.088  -0.088
## 58     Total.Animals2  ~ Mean.Raup.Crick2  7.996     1.982  0.080   0.080
## 60     Total.Animals2  ~       X10km_Pop2  8.876     2.200  0.074   0.074
## 61     Total.Animals2  ~    GDP.Millions2 29.903     7.411 -0.128  -0.128
## 66       Sp.Richness2  ~ Mean.Raup.Crick2 22.422     5.557 -0.098  -0.098
## 67       Sp.Richness2  ~      Zoo.Area.ha2 10.169     2.520  0.067   0.067
## 71   Mean.Raup.Crick2  ~      Attendance2 15.151     3.755  0.370   0.370
## 72   Mean.Raup.Crick2  ~      Sp.Richness2 21.560     5.344 -0.348  -0.348
## 73   Mean.Raup.Crick2  ~      Zoo.Area.ha2 12.585     3.119  0.181   0.181
## 74   Mean.Raup.Crick2  ~       X10km_Pop2 12.485     3.094  0.149   0.149
## 76   Mean.Raup.Crick2  ~      Prop.Mam.Sp2 10.137     2.512 -0.179  -0.179
## 77   Mean.Raup.Crick2  ~ Mam.Sp.Richness2 11.509     2.853 -0.199  -0.199
## 78       Zoo.Area.ha2  ~      Attendance2 16.535     4.098  0.593   0.593
## 79       Zoo.Area.ha2  ~    Total.Animals2 10.968     2.718  0.286   0.286
## 80       Zoo.Area.ha2  ~      Sp.Richness2 10.722     2.657  0.311   0.311
## 81       Zoo.Area.ha2  ~ Mean.Raup.Crick2 28.663     7.104  0.234   0.234
## 96         X10km_Pop2  ~      Attendance2 11.547     2.862  0.420   0.420
## 97         X10km_Pop2  ~    Total.Animals2  6.086     1.508  0.173   0.173
## 99         X10km_Pop2  ~ Mean.Raup.Crick2 19.900     4.932  0.211   0.211
## 105     GDP.Millions2  ~      Attendance2 12.199     3.023 -0.547  -0.547
## 106     GDP.Millions2  ~    Total.Animals2 15.369     3.809 -0.306  -0.306
## 117      Prop.Mam.Sp2  ~ Mean.Raup.Crick2  3.335     0.827 -0.077  -0.077
## 124 Mam.Sp.Richness2  ~    Total.Animals2  6.733     1.669 -0.268  -0.268
## 125 Mam.Sp.Richness2  ~      Sp.Richness2  3.410     0.845 -0.400  -0.400
## 126 Mam.Sp.Richness2  ~ Mean.Raup.Crick2 37.454     9.283 -0.344  -0.344
##     sepc.all sepc.nox
## 52     0.075    0.075
## 53    -0.088   -0.088
## 58     0.081    0.081
## 60     0.075    0.075
## 61    -0.129   -0.130
## 66    -0.098   -0.098
## 67     0.067    0.067
## 71     0.364    0.364
## 72    -0.348   -0.348
## 73     0.181    0.181
## 74     0.149    0.149
## 76    -0.179   -0.179
## 77    -0.199   -0.200
## 78     0.582    0.582
## 79     0.283    0.283
## 80     0.311    0.311
## 81     0.234    0.234
## 96     0.413    0.413
## 97     0.171    0.171
## 99     0.211    0.211
## 105   -0.537   -0.537
## 106   -0.304   -0.304
```

```
## 117   -0.077   -0.077
## 124   -0.266   -0.266
## 125   -0.400   -0.400
## 126   -0.344   -0.344
```

Model Comparisons 2

At this stage we again compare the models generated using AICc values. Overall model selection from the pool of competing models is achieved using AICc values, with a threshold of more than 2 AICc units lower than nearest competing model being considered sufficient for model selection. Based on these results it is clear that model 20 is superior and the removal of the Brillouin Index was justified.

```r
# Model Comparisons using AICc

# Comparing models with and without adjustment for nested nature of data
# library(AICcmodavg)
# source("lavaan.modavg.R")

aictab.lavaan(list(mod.1.fit, mod.2.fit, mod.3.fit, mod.4.fit, mod.5.fit, mod.6.fit, mod.7.fit, mod.8.fi
              c("mod.1", "mod.2", "mod.3", "mod.4", "mod.5", "mod.6", "mod.7", "mod.8", "mod.9", "mod.1(
```

```
##
## Model selection based on AICc:
##
##                 K     AICc Delta_AICc AICcWt Cum.Wt       LL
## mod20          37 10281.46       0.00      1      1 -5103.38
## mod.20.nested 47 10301.46      20.00      0      1 -5103.38
## mod.18         43 10955.33     673.87      0      1 -5433.97
## mod19          42 10955.82     674.36      0      1 -5435.30
## mod.18.nested 54 10977.33     695.87      0      1 -5433.97
## mod.19.nested 53 10977.82     696.36      0      1 -5435.30
## mod.17.nested 63 11890.76    1609.30      0      1 -5881.60
## mod.16.nested 64 11861.94    1580.48      0      1 -5866.10
## mod.15.nested 74 12530.10    2248.64      0      1 -6190.09
## mod.14.nested 75 12509.73    2228.27      0      1 -6178.80
## mod.13.nested 76 12501.65    2220.19      0      1 -6173.66
## mod.12.nested 77 12501.97    2220.51      0      1 -6172.71
## mod.11.nested 78 12502.71    2221.25      0      1 -6171.97
## mod.10.nested 78 12502.71    2221.25      0      1 -6171.97
## mod.9.nested  79 12503.71    2222.25      0      1 -6171.35
## mod.8.nested  85 13749.39    3467.92      0      1 -6787.66
## mod.7.nested  84 13763.59    3482.13      0      1 -6795.90
## mod.6.nested  83 13762.55    3481.09      0      1 -6796.52
## mod.5.nested  82 13791.29    3509.83      0      1 -6812.02
## mod.4.nested  81 13835.38    3553.92      0      1 -6835.18
## mod.3.nested  80 13900.33    3618.87      0      1 -6868.78
## mod.2.nested  79 14027.63    3746.17      0      1 -6933.54
## mod.1.nested  78 14684.32    4402.85      0      1 -7263.00
## mod.17         51 11866.76    1585.30      0      1 -5881.60
## mod.16         52 11837.94    1556.48      0      1 -5866.10
## mod.15         61 12504.10    2222.64      0      1 -6190.09
## mod.14         62 12483.73    2202.27      0      1 -6178.80
## mod.13         63 12475.65    2194.19      0      1 -6173.66
## mod.12         64 12475.97    2194.51      0      1 -6172.71
```

```
## mod.11        65 12476.71    2195.25      0      1 -6171.97
## mod.10        65 12476.71    2195.25      0      1 -6171.97
## mod.9         66 12477.71    2196.25      0      1 -6171.35
## mod.8         71 13721.39    3439.92      0      1 -6787.66
## mod.7         70 13735.59    3454.13      0      1 -6795.90
## mod.6         69 13734.55    3453.09      0      1 -6796.52
## mod.5         68 13763.29    3481.83      0      1 -6812.02
## mod.4         67 13807.38    3525.92      0      1 -6835.18
## mod.3         66 13872.33    3590.87      0      1 -6868.78
## mod.2         65 13999.63    3718.17      0      1 -6933.54
## mod.1         64 14656.32    4374.85      0      1 -7263.00
```

```
# Model 20 superior
```

At this point we turn to the modification indices once again and see if any new suggested links have been generated.

Model 21

Based on the modification indices generated from the nested twentieth model, we can see that **Total.Animals2 ~ GDP.Millions2** has an mi value of **29.903** which exceeds the standard cut-off level for the chi-square test criterion of 3.84 (Burnham and Anderson, 2002). Although only the second highest mi value, this is by far the most intuitive of the two, as it is logical to assume that countries with higher GDP values can afford to have more individual animals within a collection or conversely that high GDP will lead to lower number of animals due to increased enclosure sizes and welfare standards in more developed nations. As a result, we add this relationship to our model. Once again, the model summary, fit indices and modification indices were all generated for the model, adjusting for the nested nature of data.

```
# Attendance SEM (Presence-Absence)

# Model 21
# Addition of Total.Animals2 ~ GDP.Millions2, mi = 29.903

mod.21 <- 'Attendance2 ~ Zoo.Area.ha2 + Total.Animals2
+ Mean.Sp.BodyMass2 + Mean.Raup.Crick2
+ X10km_Pop2 + GDP.Millions2

Total.Animals2 ~ Zoo.Area.ha2 + Sp.Richness2 + GDP.Millions2
Sp.Richness2 ~ Prop.Mam.Sp2 + Mam.Sp.Richness2
Mean.Raup.Crick2 ~ Total.Animals2 + Mean.Sp.BodyMass2'

# Fit model and generate model summary
mod.21.fit <- sem(mod.21, data = sem_attendance_data, fixed.x=FALSE)
summary(mod.21.fit, rsq = TRUE)
```

```
## lavaan (0.5-23.1097) converged normally after  28 iterations
##
##   Number of observations                           458
##
##   Estimator                                         ML
##   Minimum Function Test Statistic              126.852
##   Degrees of freedom                                17
##   P-value (Chi-square)                           0.000
##
## Parameter Estimates:
```

```
##
##   Information                                 Expected
##   Standard Errors                             Standard
##
## Regressions:
##                      Estimate  Std.Err  z-value  P(>|z|)
##   Attendance2 ~
##     Zoo.Area.ha2        0.082    0.032    2.571    0.010
##     Total.Animals2      0.409    0.029   13.980    0.000
##     Men.Sp.BdyMss2      0.353    0.033   10.705    0.000
##     Mean.Rap.Crck2      0.139    0.029    4.876    0.000
##     X10km_Pop2          0.431    0.026   16.347    0.000
##     GDP.Millions2       0.135    0.025    5.415    0.000
##   Total.Animals2 ~
##     Zoo.Area.ha2        0.219    0.023    9.570    0.000
##     Sp.Richness2        0.805    0.023   35.245    0.000
##     GDP.Millions2      -0.129    0.023   -5.670    0.000
##   Sp.Richness2 ~
##     Prop.Mam.Sp2       -0.563    0.018  -30.494    0.000
##     Mam.Sp.Rchnss2      0.779    0.018   42.208    0.000
##   Mean.Raup.Crick2 ~
##     Total.Animals2      0.259    0.041    6.400    0.000
##     Men.Sp.BdyMss2     -0.448    0.040  -11.127    0.000
##
## Covariances:
##                        Estimate  Std.Err  z-value  P(>|z|)
##   Zoo.Area.ha2 ~~
##     Men.Sp.BdyMss2        0.534    0.053   10.096    0.000
##     X10km_Pop2           -0.010    0.047   -0.208    0.835
##     GDP.Millions2        -0.027    0.047   -0.588    0.557
##     Prop.Mam.Sp2          0.312    0.049    6.389    0.000
##     Mam.Sp.Rchnss2        0.381    0.050    7.634    0.000
##   Mean.Sp.BodyMass2 ~~
##     X10km_Pop2            0.126    0.047    2.683    0.007
##     GDP.Millions2        -0.082    0.047   -1.748    0.080
##     Prop.Mam.Sp2          0.604    0.055   11.083    0.000
##     Mam.Sp.Rchnss2        0.303    0.049    6.216    0.000
##   X10km_Pop2 ~~
##     GDP.Millions2        -0.028    0.047   -0.604    0.546
##     Prop.Mam.Sp2         -0.204    0.048   -4.293    0.000
##     Mam.Sp.Rchnss2        0.284    0.048    5.852    0.000
##   GDP.Millions2 ~~
##     Prop.Mam.Sp2         -0.123    0.047   -2.615    0.009
##     Mam.Sp.Rchnss2       -0.060    0.047   -1.277    0.202
##   Prop.Mam.Sp2 ~~
##     Mam.Sp.Rchnss2        0.088    0.047    1.882    0.060
##
## Variances:
##                    Estimate  Std.Err  z-value  P(>|z|)
##    .Attendance2       0.277    0.018   15.133    0.000
##    .Total.Animals2    0.235    0.016   15.133    0.000
##    .Sp.Richness2      0.154    0.010   15.133    0.000
##    .Mean.Rap.Crck2    0.739    0.049   15.133    0.000
##     Zoo.Area.ha2      0.998    0.066   15.133    0.000
```

```
##     Men.Sp.BdyMss2    0.998    0.066   15.133    0.000
##     X10km_Pop2        0.998    0.066   15.133    0.000
##     GDP.Millions2     0.998    0.066   15.133    0.000
##     Prop.Mam.Sp2      0.998    0.066   15.133    0.000
##     Mam.Sp.Rchnss2    0.998    0.066   15.133    0.000
##
## R-Square:
##                    Estimate
##     Attendance2       0.709
##     Total.Animals2    0.762
##     Sp.Richness2      0.845
##     Mean.Rap.Crck2    0.257
```

```
# Generate fit indices
fitMeasures(mod.21.fit, c("agfi", "rmr", "srmr", "rmsea", "cfi", "nnfi", "tli"))
```

```
##  agfi   rmr  srmr rmsea   cfi  nnfi   tli
## 0.848 0.040 0.040 0.119 0.953 0.917 0.917
```

```
# Generate modification indices
mi21 <- modindices(mod.21.fit)
print(mi21[mi21$mi > 3.0,])
```

```
##                  lhs op              rhs     mi    epc sepc.lv sepc.all
## 43    Total.Animals2 ~~ Mean.Raup.Crick2 10.289  0.072   0.072    0.072
## 44      Sp.Richness2 ~~ Mean.Raup.Crick2 27.799 -0.088  -0.088   -0.088
## 48    Total.Animals2  ~      Attendance2  4.746  0.072   0.072    0.071
## 49    Total.Animals2  ~ Mean.Raup.Crick2  9.331  0.083   0.083    0.083
## 51    Total.Animals2  ~       X10km_Pop2  7.850  0.068   0.068    0.068
## 56      Sp.Richness2  ~ Mean.Raup.Crick2 22.606 -0.099  -0.099   -0.099
## 57      Sp.Richness2  ~     Zoo.Area.ha2 10.169  0.067   0.067    0.067
## 61  Mean.Raup.Crick2  ~      Attendance2 14.802  0.362   0.362    0.354
## 62  Mean.Raup.Crick2  ~     Sp.Richness2 21.846 -0.352  -0.352   -0.353
## 63  Mean.Raup.Crick2  ~     Zoo.Area.ha2 12.524  0.180   0.180    0.180
## 64  Mean.Raup.Crick2  ~       X10km_Pop2 12.518  0.149   0.149    0.149
## 66  Mean.Raup.Crick2  ~     Prop.Mam.Sp2 10.037 -0.177  -0.177   -0.178
## 67  Mean.Raup.Crick2  ~ Mam.Sp.Richness2 11.750 -0.203  -0.203   -0.204
## 68      Zoo.Area.ha2  ~      Attendance2 18.021  0.624   0.624    0.610
## 69      Zoo.Area.ha2  ~   Total.Animals2 12.532  0.309   0.309    0.307
## 70      Zoo.Area.ha2  ~     Sp.Richness2 10.722  0.311   0.311    0.311
## 71      Zoo.Area.ha2  ~ Mean.Raup.Crick2 28.895  0.235   0.235    0.235
## 86        X10km_Pop2  ~      Attendance2 11.934  0.435   0.435    0.425
## 87        X10km_Pop2  ~   Total.Animals2  6.353  0.180   0.180    0.179
## 89        X10km_Pop2  ~ Mean.Raup.Crick2 19.926  0.212   0.212    0.211
## 107     Prop.Mam.Sp2  ~ Mean.Raup.Crick2  3.271 -0.076  -0.076   -0.076
## 114 Mam.Sp.Richness2  ~   Total.Animals2  8.637 -0.312  -0.312   -0.310
## 115 Mam.Sp.Richness2  ~     Sp.Richness2  3.410 -0.400  -0.400   -0.400
## 116 Mam.Sp.Richness2  ~ Mean.Raup.Crick2 38.155 -0.348  -0.348   -0.348
##     sepc.nox
## 43     0.072
## 44    -0.088
## 48     0.071
## 49     0.083
## 51     0.068
## 56    -0.099
## 57     0.067
```

```
## 61     0.354
## 62    -0.353
## 63     0.181
## 64     0.149
## 66    -0.178
## 67    -0.204
## 68     0.610
## 69     0.307
## 70     0.311
## 71     0.235
## 86     0.425
## 87     0.179
## 89     0.211
## 107   -0.076
## 114   -0.310
## 115   -0.400
## 116   -0.348
```

```
# Adjust for the nested nature of the data (institutions within countries)
# Fit model and generate model summary
design <- svydesign(ids = ~Country, nest=TRUE, data=sem_attendance_data)
fit.adj21 <- lavaan.survey(lavaan.fit = mod.21.fit, survey.design = design)
summary(fit.adj21, rsq = TRUE)
```

```
## lavaan (0.5-23.1097) converged normally after  28 iterations
##
##   Number of observations                           458
##
##   Estimator                                         ML      Robust
##   Minimum Function Test Statistic              126.852      36.675
##   Degrees of freedom                                17          17
##   P-value (Chi-square)                           0.000       0.004
##   Scaling correction factor                                  3.459
##     for the Satorra-Bentler correction
##
## Parameter Estimates:
##
##   Information                                 Expected
##   Standard Errors                           Robust.sem
##
## Regressions:
##                        Estimate  Std.Err  z-value  P(>|z|)
##   Attendance2 ~
##     Zoo.Area.ha2          0.082    0.038    2.140    0.032
##     Total.Animals2        0.409    0.027   14.993    0.000
##     Men.Sp.BdyMss2        0.353    0.030   11.632    0.000
##     Mean.Rap.Crck2        0.139    0.028    5.042    0.000
##     X10km_Pop2            0.431    0.033   12.953    0.000
##     GDP.Millions2         0.135    0.027    5.066    0.000
##   Total.Animals2 ~
##     Zoo.Area.ha2          0.219    0.034    6.384    0.000
##     Sp.Richness2          0.805    0.049   16.513    0.000
##     GDP.Millions2        -0.129    0.072   -1.786    0.074
##   Sp.Richness2 ~
##     Prop.Mam.Sp2         -0.563    0.041  -13.858    0.000
```

```
##     Mam.Sp.Rchnss2      0.779    0.057   13.593    0.000
##   Mean.Raup.Crick2 ~
##     Total.Animals2      0.259    0.062    4.216    0.000
##     Men.Sp.BdyMss2     -0.448    0.082   -5.495    0.000
##
## Covariances:
##                        Estimate  Std.Err  z-value  P(>|z|)
##   Zoo.Area.ha2 ~~
##     Men.Sp.BdyMss2        0.534    0.104    5.147    0.000
##     X10km_Pop2           -0.010    0.078   -0.124    0.901
##     GDP.Millions2        -0.027    0.041   -0.673    0.501
##     Prop.Mam.Sp2          0.312    0.089    3.510    0.000
##     Mam.Sp.Rchnss2        0.381    0.064    5.985    0.000
##   Mean.Sp.BodyMass2 ~~
##     X10km_Pop2            0.126    0.067    1.895    0.058
##     GDP.Millions2        -0.082    0.046   -1.759    0.079
##     Prop.Mam.Sp2          0.604    0.130    4.656    0.000
##     Mam.Sp.Rchnss2        0.303    0.081    3.730    0.000
##   X10km_Pop2 ~~
##     GDP.Millions2        -0.028    0.056   -0.505    0.613
##     Prop.Mam.Sp2         -0.204    0.055   -3.685    0.000
##     Mam.Sp.Rchnss2        0.284    0.066    4.278    0.000
##   GDP.Millions2 ~~
##     Prop.Mam.Sp2         -0.123    0.105   -1.172    0.241
##     Mam.Sp.Rchnss2       -0.060    0.064   -0.937    0.349
##   Prop.Mam.Sp2 ~~
##     Mam.Sp.Rchnss2        0.088    0.061    1.435    0.151
##
## Intercepts:
##                    Estimate  Std.Err  z-value  P(>|z|)
##    .Attendance2      -0.000    0.029   -0.000    1.000
##    .Total.Animals2   -0.000    0.070   -0.000    1.000
##    .Sp.Richness2      0.000    0.032    0.000    1.000
##    .Mean.Rap.Crck2   -0.000    0.098   -0.000    1.000
##     Zoo.Area.ha2      0.000    0.064    0.000    1.000
##     Men.Sp.BdyMss2   -0.000    0.080   -0.000    1.000
##     X10km_Pop2        0.000    0.105    0.000    1.000
##     GDP.Millions2    -0.000    0.345   -0.000    1.000
##     Prop.Mam.Sp2      0.000    0.118    0.000    1.000
##     Mam.Sp.Rchnss2   -0.000    0.079   -0.000    1.000
##
## Variances:
##                    Estimate  Std.Err  z-value  P(>|z|)
##    .Attendance2       0.277    0.028   10.008    0.000
##    .Total.Animals2    0.235    0.038    6.139    0.000
##    .Sp.Richness2      0.154    0.044    3.506    0.000
##    .Mean.Rap.Crck2    0.739    0.109    6.767    0.000
##     Zoo.Area.ha2      0.998    0.112    8.886    0.000
##     Men.Sp.BdyMss2    0.998    0.173    5.783    0.000
##     X10km_Pop2        0.998    0.174    5.745    0.000
##     GDP.Millions2     0.998    0.246    4.061    0.000
##     Prop.Mam.Sp2      0.998    0.150    6.662    0.000
##     Mam.Sp.Rchnss2    0.998    0.115    8.669    0.000
##
```

```
## R-Square:
##                    Estimate
##     Attendance2       0.709
##     Total.Animals2    0.762
##     Sp.Richness2      0.845
##     Mean.Rap.Crck2    0.257
```

```r
# Generate fit indices
fitMeasures(fit.adj21, c("agfi", "rmr", "srmr", "rmsea", "cfi", "nnfi", "tli"))
```

```
##  agfi   rmr  srmr rmsea   cfi  nnfi   tli
## 0.820 0.040 0.037 0.119 0.953 0.917 0.917
```

```r
# Generate modification indices
mi21adj <- modindices(fit.adj21)
print(mi21adj[mi21adj$mi > 3.0,])
```

```
##                  lhs op               rhs     mi mi.scaled    epc sepc.lv
## 53    Total.Animals2 ~~ Mean.Raup.Crick2 10.289     2.975  0.072   0.072
## 54      Sp.Richness2 ~~ Mean.Raup.Crick2 27.799     8.037 -0.088  -0.088
## 58    Total.Animals2  ~       Attendance2  4.746     1.372  0.072   0.072
## 59    Total.Animals2  ~ Mean.Raup.Crick2  9.331     2.698  0.083   0.083
## 61    Total.Animals2  ~        X10km_Pop2  7.850     2.269  0.068   0.068
## 66      Sp.Richness2  ~ Mean.Raup.Crick2 22.606     6.536 -0.099  -0.099
## 67      Sp.Richness2  ~      Zoo.Area.ha2 10.169     2.940  0.067   0.067
## 71  Mean.Raup.Crick2  ~       Attendance2 14.802     4.280  0.362   0.362
## 72  Mean.Raup.Crick2  ~      Sp.Richness2 21.846     6.316 -0.352  -0.352
## 73  Mean.Raup.Crick2  ~      Zoo.Area.ha2 12.524     3.621  0.180   0.180
## 74  Mean.Raup.Crick2  ~        X10km_Pop2 12.518     3.619  0.149   0.149
## 76  Mean.Raup.Crick2  ~      Prop.Mam.Sp2 10.037     2.902 -0.177  -0.177
## 77  Mean.Raup.Crick2  ~ Mam.Sp.Richness2 11.750     3.397 -0.203  -0.203
## 78      Zoo.Area.ha2  ~       Attendance2 18.021     5.210  0.624   0.624
## 79      Zoo.Area.ha2  ~    Total.Animals2 12.532     3.623  0.309   0.309
## 80      Zoo.Area.ha2  ~      Sp.Richness2 10.722     3.100  0.311   0.311
## 81      Zoo.Area.ha2  ~ Mean.Raup.Crick2 28.895     8.354  0.235   0.235
## 96        X10km_Pop2  ~       Attendance2 11.934     3.450  0.435   0.435
## 97        X10km_Pop2  ~    Total.Animals2  6.353     1.837  0.180   0.180
## 99        X10km_Pop2  ~ Mean.Raup.Crick2 19.926     5.761  0.212   0.212
## 117     Prop.Mam.Sp2  ~ Mean.Raup.Crick2  3.271     0.946 -0.076  -0.076
## 124 Mam.Sp.Richness2  ~    Total.Animals2  8.637     2.497 -0.312  -0.312
## 125 Mam.Sp.Richness2  ~      Sp.Richness2  3.410     0.986 -0.400  -0.400
## 126 Mam.Sp.Richness2  ~ Mean.Raup.Crick2 38.155    11.031 -0.348  -0.348
##     sepc.all sepc.nox
## 53     0.072    0.072
## 54    -0.088   -0.088
## 58     0.071    0.071
## 59     0.083    0.083
## 61     0.068    0.068
## 66    -0.099   -0.099
## 67     0.067    0.067
## 71     0.354    0.354
## 72    -0.353   -0.353
## 73     0.180    0.181
## 74     0.149    0.149
## 76    -0.178   -0.178
## 77    -0.204   -0.204
```

```
## 78      0.610    0.610
## 79      0.307    0.307
## 80      0.311    0.311
## 81      0.235    0.235
## 96      0.425    0.425
## 97      0.179    0.179
## 99      0.211    0.211
## 117   -0.076   -0.076
## 124   -0.310   -0.310
## 125   -0.400   -0.400
## 126   -0.348   -0.348
```

Model 22

Based on the modification indices generated from the nested twenty first model, we can see that **Mean.Raup.Crick2** ~ **Sp.Richness2** has an mi value of **21.846** which exceeds the standard cut-off level for the chi-square test criterion of 3.84 (Burnham and Anderson, 2002). Although only the second highest mi value, this is by far the most intuitive of the two, as it is logical to assume that insistutions with higher species richness have a greater probability of being dissimilar to other institutional collections. As a result, we add this relationship to our model. Once again, the model summary, fit indices and modification indices were all generated for the model, adjusting for the nested nature of data.

```
# Attendance SEM (Presence-Absence)

# Model 22
# Addition of Mean.Raup.Crick2 ~ Sp.Richness2 21.846, mi = 21.846

mod.22 <- 'Attendance2 ~ Zoo.Area.ha2 + Total.Animals2
+ Mean.Sp.BodyMass2 + Mean.Raup.Crick2
+ X10km_Pop2 + GDP.Millions2

Total.Animals2 ~ Zoo.Area.ha2 + Sp.Richness2 + GDP.Millions2
Sp.Richness2 ~ Prop.Mam.Sp2 + Mam.Sp.Richness2
Mean.Raup.Crick2 ~ Total.Animals2 + Mean.Sp.BodyMass2 + Sp.Richness2'

# Fit model and generate model summary
mod.22.fit <- sem(mod.22, data = sem_attendance_data, fixed.x=FALSE)
summary(mod.22.fit, rsq = TRUE)
```

```
## lavaan (0.5-23.1097) converged normally after  31 iterations
##
##   Number of observations                           458
##
##   Estimator                                         ML
##   Minimum Function Test Statistic              104.283
##   Degrees of freedom                                16
##   P-value (Chi-square)                           0.000
##
## Parameter Estimates:
##
##   Information                                 Expected
##   Standard Errors                             Standard
##
## Regressions:
```

```
##                       Estimate  Std.Err  z-value  P(>|z|)
##   Attendance2 ~
##     Zoo.Area.ha2         0.082    0.032    2.570    0.010
##     Total.Animals2       0.409    0.029   13.996    0.000
##     Men.Sp.BdyMss2       0.353    0.033   10.650    0.000
##     Mean.Rap.Crck2       0.139    0.029    4.863    0.000
##     X10km_Pop2           0.431    0.026   16.330    0.000
##     GDP.Millions2        0.135    0.025    5.410    0.000
##   Total.Animals2 ~
##     Zoo.Area.ha2         0.219    0.023    9.570    0.000
##     Sp.Richness2         0.805    0.023   35.245    0.000
##     GDP.Millions2       -0.129    0.023   -5.670    0.000
##   Sp.Richness2 ~
##     Prop.Mam.Sp2        -0.563    0.018  -30.494    0.000
##     Mam.Sp.Rchnss2       0.779    0.018   42.208    0.000
##   Mean.Raup.Crick2 ~
##     Total.Animals2       0.558    0.074    7.575    0.000
##     Men.Sp.BdyMss2      -0.495    0.041  -12.194    0.000
##     Sp.Richness2        -0.355    0.074   -4.829    0.000
##
## Covariances:
##                         Estimate  Std.Err  z-value  P(>|z|)
##   Zoo.Area.ha2 ~~
##     Men.Sp.BdyMss2         0.534    0.053   10.096    0.000
##     X10km_Pop2            -0.010    0.047   -0.208    0.835
##     GDP.Millions2         -0.027    0.047   -0.588    0.557
##     Prop.Mam.Sp2           0.312    0.049    6.389    0.000
##     Mam.Sp.Rchnss2         0.381    0.050    7.634    0.000
##   Mean.Sp.BodyMass2 ~~
##     X10km_Pop2             0.126    0.047    2.683    0.007
##     GDP.Millions2         -0.082    0.047   -1.748    0.080
##     Prop.Mam.Sp2           0.604    0.055   11.083    0.000
##     Mam.Sp.Rchnss2         0.303    0.049    6.216    0.000
##   X10km_Pop2 ~~
##     GDP.Millions2         -0.028    0.047   -0.604    0.546
##     Prop.Mam.Sp2          -0.204    0.048   -4.293    0.000
##     Mam.Sp.Rchnss2         0.284    0.048    5.852    0.000
##   GDP.Millions2 ~~
##     Prop.Mam.Sp2          -0.123    0.047   -2.615    0.009
##     Mam.Sp.Rchnss2        -0.060    0.047   -1.277    0.202
##   Prop.Mam.Sp2 ~~
##     Mam.Sp.Rchnss2         0.088    0.047    1.882    0.060
##
## Variances:
##                     Estimate  Std.Err  z-value  P(>|z|)
##    .Attendance2        0.277    0.018   15.133    0.000
##    .Total.Animals2     0.235    0.016   15.133    0.000
##    .Sp.Richness2       0.154    0.010   15.133    0.000
##    .Mean.Rap.Crck2     0.704    0.047   15.133    0.000
##     Zoo.Area.ha2       0.998    0.066   15.133    0.000
##     Men.Sp.BdyMss2     0.998    0.066   15.133    0.000
##     X10km_Pop2         0.998    0.066   15.133    0.000
##     GDP.Millions2      0.998    0.066   15.133    0.000
##     Prop.Mam.Sp2       0.998    0.066   15.133    0.000
```

```
##     Mam.Sp.Rchnss2    0.998    0.066   15.133    0.000
##
## R-Square:
##                    Estimate
##     Attendance2       0.707
##     Total.Animals2    0.762
##     Sp.Richness2      0.845
##     Mean.Rap.Crck2    0.291
```

```r
# Generate fit indices
fitMeasures(mod.22.fit, c("agfi", "rmr", "srmr", "rmsea", "cfi", "nnfi", "tli"))
```

```
##  agfi   rmr  srmr rmsea   cfi  nnfi   tli
## 0.869 0.042 0.042 0.110 0.962 0.929 0.929
```

```r
# Generate modification indices
mi22 <- modindices(mod.22.fit)
print(mi22[mi22$mi > 3.0,])
```

```
##                  lhs op               rhs     mi    epc sepc.lv sepc.all
## 44    Total.Animals2 ~~ Mean.Raup.Crick2  4.206 -0.094  -0.094   -0.095
## 45      Sp.Richness2 ~~ Mean.Raup.Crick2 20.844 -0.077  -0.077   -0.077
## 52    Total.Animals2  ~       X10km_Pop2  7.850  0.068   0.068    0.068
## 57      Sp.Richness2  ~ Mean.Raup.Crick2 13.478 -0.076  -0.076   -0.076
## 58      Sp.Richness2  ~     Zoo.Area.ha2 10.169  0.067   0.067    0.067
## 62  Mean.Raup.Crick2  ~      Attendance2 22.802  0.440   0.440    0.430
## 63  Mean.Raup.Crick2  ~     Zoo.Area.ha2  8.934  0.151   0.151    0.151
## 64  Mean.Raup.Crick2  ~       X10km_Pop2 19.643  0.188   0.188    0.188
## 66  Mean.Raup.Crick2  ~     Prop.Mam.Sp2 28.146 -0.312  -0.312   -0.313
## 68      Zoo.Area.ha2  ~      Attendance2 13.287  0.538   0.538    0.524
## 69      Zoo.Area.ha2  ~   Total.Animals2 12.532  0.309   0.309    0.307
## 70      Zoo.Area.ha2  ~     Sp.Richness2 10.722  0.311   0.311    0.311
## 71      Zoo.Area.ha2  ~ Mean.Raup.Crick2 18.401  0.198   0.198    0.197
## 86        X10km_Pop2  ~      Attendance2 11.287  0.411   0.411    0.400
## 87        X10km_Pop2  ~   Total.Animals2  6.353  0.180   0.180    0.179
## 89        X10km_Pop2  ~ Mean.Raup.Crick2 19.503  0.207   0.207    0.207
## 107     Prop.Mam.Sp2  ~ Mean.Raup.Crick2  9.734 -0.134  -0.134   -0.134
## 114 Mam.Sp.Richness2  ~   Total.Animals2  8.637 -0.312  -0.312   -0.310
## 115 Mam.Sp.Richness2  ~     Sp.Richness2  3.410 -0.400  -0.400   -0.400
## 116 Mam.Sp.Richness2  ~ Mean.Raup.Crick2 19.116 -0.278  -0.278   -0.277
##     sepc.nox
## 44    -0.095
## 45    -0.077
## 52     0.068
## 57    -0.076
## 58     0.067
## 62     0.430
## 63     0.151
## 64     0.188
## 66    -0.314
## 68     0.524
## 69     0.307
## 70     0.311
## 71     0.197
## 86     0.400
## 87     0.179
```

```
## 89     0.207
## 107   -0.134
## 114   -0.310
## 115   -0.400
## 116   -0.277
```

```
# Adjust for the nested nature of the data (institutions within countries)
# Fit model and generate model summary
design <- svydesign(ids = ~Country, nest=TRUE, data=sem_attendance_data)
fit.adj22 <- lavaan.survey(lavaan.fit = mod.22.fit, survey.design = design)
summary(fit.adj22, rsq = TRUE)
```

```
## lavaan (0.5-23.1097) converged normally after  31 iterations
##
##   Number of observations                           458
##
##   Estimator                                         ML      Robust
##   Minimum Function Test Statistic              104.283      41.161
##   Degrees of freedom                                16          16
##   P-value (Chi-square)                           0.000       0.001
##   Scaling correction factor                                  2.534
##     for the Satorra-Bentler correction
##
## Parameter Estimates:
##
##   Information                                 Expected
##   Standard Errors                           Robust.sem
##
## Regressions:
##                        Estimate  Std.Err  z-value  P(>|z|)
##   Attendance2 ~
##     Zoo.Area.ha2          0.082    0.038    2.147    0.032
##     Total.Animals2        0.409    0.027   15.138    0.000
##     Men.Sp.BdyMss2        0.353    0.030   11.610    0.000
##     Mean.Rap.Crck2        0.139    0.027    5.131    0.000
##     X10km_Pop2            0.431    0.033   12.912    0.000
##     GDP.Millions2         0.135    0.026    5.140    0.000
##   Total.Animals2 ~
##     Zoo.Area.ha2          0.219    0.034    6.384    0.000
##     Sp.Richness2          0.805    0.049   16.513    0.000
##     GDP.Millions2        -0.129    0.072   -1.786    0.074
##   Sp.Richness2 ~
##     Prop.Mam.Sp2         -0.563    0.041  -13.858    0.000
##     Mam.Sp.Rchnss2        0.779    0.057   13.593    0.000
##   Mean.Raup.Crick2 ~
##     Total.Animals2        0.558    0.151    3.706    0.000
##     Men.Sp.BdyMss2       -0.495    0.052   -9.545    0.000
##     Sp.Richness2         -0.355    0.225   -1.576    0.115
##
## Covariances:
##                          Estimate  Std.Err  z-value  P(>|z|)
##   Zoo.Area.ha2 ~~
##     Men.Sp.BdyMss2          0.534    0.104    5.147    0.000
##     X10km_Pop2             -0.010    0.078   -0.124    0.901
##     GDP.Millions2          -0.027    0.041   -0.673    0.501
```

```
##     Prop.Mam.Sp2          0.312    0.089    3.510    0.000
##     Mam.Sp.Rchnss2        0.381    0.064    5.985    0.000
##   Mean.Sp.BodyMass2 ~~
##     X10km_Pop2            0.126    0.067    1.895    0.058
##     GDP.Millions2        -0.082    0.046   -1.759    0.079
##     Prop.Mam.Sp2          0.604    0.130    4.656    0.000
##     Mam.Sp.Rchnss2        0.303    0.081    3.730    0.000
##   X10km_Pop2 ~~
##     GDP.Millions2        -0.028    0.056   -0.505    0.613
##     Prop.Mam.Sp2         -0.204    0.055   -3.685    0.000
##     Mam.Sp.Rchnss2        0.284    0.066    4.278    0.000
##   GDP.Millions2 ~~
##     Prop.Mam.Sp2         -0.123    0.105   -1.172    0.241
##     Mam.Sp.Rchnss2       -0.060    0.064   -0.937    0.349
##   Prop.Mam.Sp2 ~~
##     Mam.Sp.Rchnss2        0.088    0.061    1.435    0.151
##
## Intercepts:
##                    Estimate  Std.Err  z-value  P(>|z|)
##    .Attendance2      -0.000    0.029   -0.000    1.000
##    .Total.Animals2   -0.000    0.070   -0.000    1.000
##    .Sp.Richness2      0.000    0.032    0.000    1.000
##    .Mean.Rap.Crck2    0.000    0.094    0.000    1.000
##     Zoo.Area.ha2      0.000    0.064    0.000    1.000
##     Men.Sp.BdyMss2   -0.000    0.080   -0.000    1.000
##     X10km_Pop2        0.000    0.105    0.000    1.000
##     GDP.Millions2    -0.000    0.345   -0.000    1.000
##     Prop.Mam.Sp2      0.000    0.118    0.000    1.000
##     Mam.Sp.Rchnss2   -0.000    0.079   -0.000    1.000
##
## Variances:
##                    Estimate  Std.Err  z-value  P(>|z|)
##    .Attendance2       0.277    0.028   10.008    0.000
##    .Total.Animals2    0.235    0.038    6.139    0.000
##    .Sp.Richness2      0.154    0.044    3.506    0.000
##    .Mean.Rap.Crck2    0.704    0.063   11.098    0.000
##     Zoo.Area.ha2      0.998    0.112    8.886    0.000
##     Men.Sp.BdyMss2    0.998    0.173    5.783    0.000
##     X10km_Pop2        0.998    0.174    5.745    0.000
##     GDP.Millions2     0.998    0.246    4.061    0.000
##     Prop.Mam.Sp2      0.998    0.150    6.662    0.000
##     Mam.Sp.Rchnss2    0.998    0.115    8.669    0.000
##
## R-Square:
##                    Estimate
##     Attendance2       0.707
##     Total.Animals2    0.762
##     Sp.Richness2      0.845
##     Mean.Rap.Crck2    0.291
```

```r
# Generate fit indices
fitMeasures(fit.adj22, c("agfi", "rmr", "srmr", "rmsea", "cfi", "nnfi", "tli"))
```

```
##  agfi   rmr  srmr rmsea   cfi  nnfi   tli
## 0.845 0.042 0.039 0.110 0.962 0.929 0.929
```

```r
# Generate modification indices
mi22adj <- modindices(fit.adj22)
print(mi22adj[mi22adj$mi > 3.0,])
```

```
##                  lhs op             rhs     mi mi.scaled    epc sepc.lv
## 54    Total.Animals2 ~~ Mean.Raup.Crick2  4.206     1.660 -0.094  -0.094
## 55      Sp.Richness2 ~~ Mean.Raup.Crick2 20.844     8.227 -0.077  -0.077
## 62    Total.Animals2  ~       X10km_Pop2  7.850     3.098  0.068   0.068
## 67      Sp.Richness2  ~ Mean.Raup.Crick2 13.478     5.320 -0.076  -0.076
## 68      Sp.Richness2  ~     Zoo.Area.ha2 10.169     4.014  0.067   0.067
## 72  Mean.Raup.Crick2  ~      Attendance2 22.802     9.000  0.440   0.440
## 73  Mean.Raup.Crick2  ~     Zoo.Area.ha2  8.934     3.526  0.151   0.151
## 74  Mean.Raup.Crick2  ~       X10km_Pop2 19.643     7.753  0.188   0.188
## 76  Mean.Raup.Crick2  ~     Prop.Mam.Sp2 28.146    11.109 -0.312  -0.312
## 78      Zoo.Area.ha2  ~      Attendance2 13.287     5.244  0.538   0.538
## 79      Zoo.Area.ha2  ~   Total.Animals2 12.532     4.946  0.309   0.309
## 80      Zoo.Area.ha2  ~     Sp.Richness2 10.722     4.232  0.311   0.311
## 81      Zoo.Area.ha2  ~ Mean.Raup.Crick2 18.401     7.263  0.198   0.198
## 96        X10km_Pop2  ~      Attendance2 11.287     4.455  0.411   0.411
## 97        X10km_Pop2  ~   Total.Animals2  6.353     2.507  0.180   0.180
## 99        X10km_Pop2  ~ Mean.Raup.Crick2 19.503     7.698  0.207   0.207
## 117     Prop.Mam.Sp2  ~ Mean.Raup.Crick2  9.734     3.842 -0.134  -0.134
## 124 Mam.Sp.Richness2  ~   Total.Animals2  8.637     3.409 -0.312  -0.312
## 125 Mam.Sp.Richness2  ~     Sp.Richness2  3.410     1.346 -0.400  -0.400
## 126 Mam.Sp.Richness2  ~ Mean.Raup.Crick2 19.116     7.545 -0.278  -0.278
##     sepc.all sepc.nox
## 54    -0.095   -0.095
## 55    -0.077   -0.077
## 62     0.068    0.068
## 67    -0.076   -0.076
## 68     0.067    0.067
## 72     0.430    0.430
## 73     0.151    0.151
## 74     0.188    0.188
## 76    -0.313   -0.314
## 78     0.524    0.524
## 79     0.307    0.307
## 80     0.311    0.311
## 81     0.197    0.197
## 96     0.400    0.400
## 97     0.179    0.179
## 99     0.207    0.207
## 117   -0.134   -0.134
## 124   -0.310   -0.310
## 125   -0.400   -0.400
## 126   -0.277   -0.277
```

Model 23

Based on the modification indices generated from the nested twenty second model, we can see that **Mean.Raup.Crick2 ~ Zoo.Area.ha2** has an mi value of **8.934** which exceeds the standard cut-off level for the chi-square test criterion of 3.84 (Burnham and Anderson, 2002). Although not the highest mi value, this is by far the most conceptually appealling, as it is logical to assume that insistutions with more space

have the potential to house more animals and therefore have a greater probability of being dissimilar to other institutional collections. As a result, we add this relationship to our model. Once again, the model summary, fit indices and modification indices were all generated for the model, adjusting for the nested nature of data.

```r
# Attendance SEM (Presence-Absence)

# Model 23
# Addition of Mean.Raup.Crick2 ~ Zoo.Area.ha2, mi = 8.934

mod.23 <- 'Attendance2 ~ Zoo.Area.ha2 + Total.Animals2
+ Mean.Sp.BodyMass2 + Mean.Raup.Crick2
+ X10km_Pop2 + GDP.Millions2

Total.Animals2 ~ Zoo.Area.ha2 + Sp.Richness2 + GDP.Millions2
Sp.Richness2 ~ Prop.Mam.Sp2 + Mam.Sp.Richness2
Mean.Raup.Crick2 ~ Total.Animals2 + Mean.Sp.BodyMass2 + Sp.Richness2 + Zoo.Area.ha2'

# Fit model and generate model summary
mod.23.fit <- sem(mod.23, data = sem_attendance_data, fixed.x=FALSE)
summary(mod.23.fit, rsq = TRUE)
```

```
## lavaan (0.5-23.1097) converged normally after  35 iterations
##
##   Number of observations                           458
##
##   Estimator                                         ML
##   Minimum Function Test Statistic               94.939
##   Degrees of freedom                                15
##   P-value (Chi-square)                           0.000
##
## Parameter Estimates:
##
##   Information                                 Expected
##   Standard Errors                             Standard
##
## Regressions:
##                    Estimate  Std.Err  z-value  P(>|z|)
##   Attendance2 ~
##     Zoo.Area.ha2      0.082    0.032    2.534    0.011
##     Total.Animals2    0.409    0.029   14.153    0.000
##     Men.Sp.BdyMss2    0.353    0.034   10.314    0.000
##     Mean.Rap.Crck2    0.139    0.029    4.797    0.000
##     X10km_Pop2        0.431    0.026   16.333    0.000
##     GDP.Millions2     0.135    0.025    5.410    0.000
##   Total.Animals2 ~
##     Zoo.Area.ha2      0.219    0.023    9.570    0.000
##     Sp.Richness2      0.805    0.023   35.245    0.000
##     GDP.Millions2    -0.129    0.023   -5.670    0.000
##   Sp.Richness2 ~
##     Prop.Mam.Sp2     -0.563    0.018  -30.494    0.000
##     Mam.Sp.Rchnss2    0.779    0.018   42.208    0.000
##   Mean.Raup.Crick2 ~
##     Total.Animals2    0.477    0.077    6.164    0.000
##     Men.Sp.BdyMss2   -0.573    0.047  -12.193    0.000
##     Sp.Richness2     -0.322    0.074   -4.357    0.000
```

```
##     Zoo.Area.ha2        0.156    0.050    3.126    0.002
##
## Covariances:
##                        Estimate  Std.Err  z-value  P(>|z|)
##   Zoo.Area.ha2 ~~
##     Men.Sp.BdyMss2        0.534    0.053   10.096    0.000
##     X10km_Pop2           -0.010    0.047   -0.208    0.835
##     GDP.Millions2        -0.027    0.047   -0.588    0.557
##     Prop.Mam.Sp2          0.312    0.049    6.389    0.000
##     Mam.Sp.Rchnss2        0.381    0.050    7.634    0.000
##   Mean.Sp.BodyMass2 ~~
##     X10km_Pop2            0.126    0.047    2.683    0.007
##     GDP.Millions2        -0.082    0.047   -1.748    0.080
##     Prop.Mam.Sp2          0.604    0.055   11.083    0.000
##     Mam.Sp.Rchnss2        0.303    0.049    6.216    0.000
##   X10km_Pop2 ~~
##     GDP.Millions2        -0.028    0.047   -0.604    0.546
##     Prop.Mam.Sp2         -0.204    0.048   -4.293    0.000
##     Mam.Sp.Rchnss2        0.284    0.048    5.852    0.000
##   GDP.Millions2 ~~
##     Prop.Mam.Sp2         -0.123    0.047   -2.615    0.009
##     Mam.Sp.Rchnss2       -0.060    0.047   -1.277    0.202
##   Prop.Mam.Sp2 ~~
##     Mam.Sp.Rchnss2        0.088    0.047    1.882    0.060
##
## Variances:
##                    Estimate  Std.Err  z-value  P(>|z|)
##    .Attendance2       0.277    0.018   15.133    0.000
##    .Total.Animals2    0.235    0.016   15.133    0.000
##    .Sp.Richness2      0.154    0.010   15.133    0.000
##    .Mean.Rap.Crck2    0.690    0.046   15.133    0.000
##     Zoo.Area.ha2      0.998    0.066   15.133    0.000
##     Men.Sp.BdyMss2    0.998    0.066   15.133    0.000
##     X10km_Pop2        0.998    0.066   15.133    0.000
##     GDP.Millions2     0.998    0.066   15.133    0.000
##     Prop.Mam.Sp2      0.998    0.066   15.133    0.000
##     Mam.Sp.Rchnss2    0.998    0.066   15.133    0.000
##
## R-Square:
##                    Estimate
##     Attendance2       0.707
##     Total.Animals2    0.762
##     Sp.Richness2      0.845
##     Mean.Rap.Crck2    0.305
```

```
# Generate fit indices
fitMeasures(mod.23.fit, c("agfi", "rmr", "srmr", "rmsea", "cfi", "nnfi", "tli"))
```

```
##  agfi   rmr  srmr rmsea   cfi  nnfi   tli
## 0.864 0.043 0.043 0.108 0.966 0.932 0.932
```

```
# Generate modification indices
mi23 <- modindices(mod.23.fit)
print(mi23[mi23$mi > 3.0,])
```

```
##                    lhs op              rhs     mi    epc sepc.lv sepc.all
```

```
## 46       Sp.Richness2 ~~ Mean.Raup.Crick2 22.710 -0.079  -0.079   -0.080
## 50     Total.Animals2  ~      Attendance2  3.130  0.060   0.060    0.059
## 53     Total.Animals2  ~       X10km_Pop2  7.850  0.068   0.068    0.068
## 58       Sp.Richness2  ~ Mean.Raup.Crick2 11.211 -0.069  -0.069   -0.069
## 59       Sp.Richness2  ~     Zoo.Area.ha2 10.169  0.067   0.067    0.067
## 63   Mean.Raup.Crick2  ~      Attendance2 25.092  0.459   0.459    0.448
## 64   Mean.Raup.Crick2  ~       X10km_Pop2 28.117  0.226   0.226    0.226
## 66   Mean.Raup.Crick2  ~     Prop.Mam.Sp2 34.668 -0.345  -0.345   -0.346
## 68       Zoo.Area.ha2  ~      Attendance2  6.838  0.431   0.431    0.420
## 69       Zoo.Area.ha2  ~   Total.Animals2 12.532  0.309   0.309    0.307
## 70       Zoo.Area.ha2  ~     Sp.Richness2 10.722  0.311   0.311    0.311
## 71       Zoo.Area.ha2  ~ Mean.Raup.Crick2 19.438  0.654   0.654    0.652
## 80  Mean.Sp.BodyMass2  ~ Mean.Raup.Crick2  4.482  0.121   0.121    0.120
## 86         X10km_Pop2  ~      Attendance2 11.615  0.423   0.423    0.412
## 87         X10km_Pop2  ~   Total.Animals2  6.353  0.180   0.180    0.179
## 89         X10km_Pop2  ~ Mean.Raup.Crick2 20.413  0.217   0.217    0.216
## 107      Prop.Mam.Sp2  ~ Mean.Raup.Crick2 12.754 -0.158  -0.158   -0.157
## 114  Mam.Sp.Richness2  ~   Total.Animals2  8.637 -0.312  -0.312   -0.310
## 115  Mam.Sp.Richness2  ~     Sp.Richness2  3.410 -0.400  -0.400   -0.400
## 116  Mam.Sp.Richness2  ~ Mean.Raup.Crick2 14.360 -0.251  -0.251   -0.250
##     sepc.nox
## 46    -0.080
## 50     0.059
## 53     0.068
## 58    -0.069
## 59     0.067
## 63     0.448
## 64     0.227
## 66    -0.346
## 68     0.420
## 69     0.307
## 70     0.311
## 71     0.652
## 80     0.120
## 86     0.412
## 87     0.179
## 89     0.216
## 107   -0.157
## 114   -0.310
## 115   -0.400
## 116   -0.250
```

```
# Adjust for the nested nature of the data (institutions within countries)
# Fit model and generate model summary
design <- svydesign(ids = ~Country, nest=TRUE, data=sem_attendance_data)
fit.adj23 <- lavaan.survey(lavaan.fit = mod.23.fit, survey.design = design)
summary(fit.adj23, rsq = TRUE)
```

```
## lavaan (0.5-23.1097) converged normally after  35 iterations
##
##   Number of observations                           458
##
##   Estimator                                         ML      Robust
##   Minimum Function Test Statistic               94.939      36.548
##   Degrees of freedom                                15          15
```

```
##   P-value (Chi-square)                           0.000       0.001
##   Scaling correction factor                                 2.598
##     for the Satorra-Bentler correction
##
## Parameter Estimates:
##
##   Information                                 Expected
##   Standard Errors                           Robust.sem
##
## Regressions:
##                      Estimate  Std.Err  z-value  P(>|z|)
##   Attendance2 ~
##     Zoo.Area.ha2        0.082    0.039    2.111    0.035
##     Total.Animals2      0.409    0.026   15.721    0.000
##     Men.Sp.BdyMss2      0.353    0.031   11.226    0.000
##     Mean.Rap.Crck2      0.139    0.028    5.005    0.000
##     X10km_Pop2          0.431    0.033   12.909    0.000
##     GDP.Millions2       0.135    0.026    5.130    0.000
##   Total.Animals2 ~
##     Zoo.Area.ha2        0.219    0.034    6.384    0.000
##     Sp.Richness2        0.805    0.049   16.513    0.000
##     GDP.Millions2      -0.129    0.072   -1.786    0.074
##   Sp.Richness2 ~
##     Prop.Mam.Sp2       -0.563    0.041  -13.858    0.000
##     Mam.Sp.Rchnss2      0.779    0.057   13.593    0.000
##   Mean.Raup.Crick2 ~
##     Total.Animals2      0.477    0.149    3.212    0.001
##     Men.Sp.BdyMss2     -0.573    0.056  -10.304    0.000
##     Sp.Richness2       -0.322    0.217   -1.485    0.138
##     Zoo.Area.ha2        0.156    0.049    3.200    0.001
##
## Covariances:
##                        Estimate  Std.Err  z-value  P(>|z|)
##   Zoo.Area.ha2 ~~
##     Men.Sp.BdyMss2        0.534    0.104    5.147    0.000
##     X10km_Pop2           -0.010    0.078   -0.124    0.901
##     GDP.Millions2        -0.027    0.041   -0.673    0.501
##     Prop.Mam.Sp2          0.312    0.089    3.510    0.000
##     Mam.Sp.Rchnss2        0.381    0.064    5.985    0.000
##   Mean.Sp.BodyMass2 ~~
##     X10km_Pop2            0.126    0.067    1.895    0.058
##     GDP.Millions2        -0.082    0.046   -1.759    0.079
##     Prop.Mam.Sp2          0.604    0.130    4.656    0.000
##     Mam.Sp.Rchnss2        0.303    0.081    3.730    0.000
##   X10km_Pop2 ~~
##     GDP.Millions2        -0.028    0.056   -0.505    0.613
##     Prop.Mam.Sp2         -0.204    0.055   -3.685    0.000
##     Mam.Sp.Rchnss2        0.284    0.066    4.278    0.000
##   GDP.Millions2 ~~
##     Prop.Mam.Sp2         -0.123    0.105   -1.172    0.241
##     Mam.Sp.Rchnss2       -0.060    0.064   -0.937    0.349
##   Prop.Mam.Sp2 ~~
##     Mam.Sp.Rchnss2        0.088    0.061    1.435    0.151
##
```

```
## Intercepts:
##                    Estimate  Std.Err  z-value  P(>|z|)
##    .Attendance2      -0.000    0.029   -0.000    1.000
##    .Total.Animals2   -0.000    0.070   -0.000    1.000
##    .Sp.Richness2      0.000    0.032    0.000    1.000
##    .Mean.Rap.Crck2    0.000    0.089    0.000    1.000
##     Zoo.Area.ha2      0.000    0.064    0.000    1.000
##     Men.Sp.BdyMss2   -0.000    0.080   -0.000    1.000
##     X10km_Pop2        0.000    0.105    0.000    1.000
##     GDP.Millions2    -0.000    0.345   -0.000    1.000
##     Prop.Mam.Sp2      0.000    0.118    0.000    1.000
##     Mam.Sp.Rchnss2   -0.000    0.079   -0.000    1.000
##
## Variances:
##                    Estimate  Std.Err  z-value  P(>|z|)
##    .Attendance2       0.277    0.028   10.008    0.000
##    .Total.Animals2    0.235    0.038    6.139    0.000
##    .Sp.Richness2      0.154    0.044    3.506    0.000
##    .Mean.Rap.Crck2    0.690    0.061   11.233    0.000
##     Zoo.Area.ha2      0.998    0.112    8.886    0.000
##     Men.Sp.BdyMss2    0.998    0.173    5.783    0.000
##     X10km_Pop2        0.998    0.174    5.745    0.000
##     GDP.Millions2     0.998    0.246    4.061    0.000
##     Prop.Mam.Sp2      0.998    0.150    6.662    0.000
##     Mam.Sp.Rchnss2    0.998    0.115    8.669    0.000
##
## R-Square:
##                    Estimate
##     Attendance2       0.707
##     Total.Animals2    0.762
##     Sp.Richness2      0.845
##     Mean.Rap.Crck2    0.305
```

```r
# Generate fit indices
fitMeasures(fit.adj23, c("agfi", "rmr", "srmr", "rmsea", "cfi", "nnfi", "tli"))
```

```
##  agfi   rmr  srmr rmsea   cfi  nnfi   tli
## 0.839 0.043 0.039 0.108 0.966 0.932 0.932
```

```r
# Generate modification indices
mi23adj <- modindices(fit.adj23)
print(mi23adj[mi23adj$mi > 3.0,])
```

```
##                   lhs op               rhs     mi mi.scaled    epc sepc.lv
## 56       Sp.Richness2 ~~ Mean.Raup.Crick2 22.710     8.742 -0.079  -0.079
## 60     Total.Animals2  ~       Attendance2  3.130     1.205  0.060   0.060
## 63     Total.Animals2  ~        X10km_Pop2  7.850     3.022  0.068   0.068
## 68       Sp.Richness2  ~ Mean.Raup.Crick2 11.211     4.316 -0.069  -0.069
## 69       Sp.Richness2  ~      Zoo.Area.ha2 10.169     3.915  0.067   0.067
## 73   Mean.Raup.Crick2  ~       Attendance2 25.092     9.659  0.459   0.459
## 74   Mean.Raup.Crick2  ~        X10km_Pop2 28.118    10.824  0.226   0.226
## 76   Mean.Raup.Crick2  ~      Prop.Mam.Sp2 34.668    13.346 -0.345  -0.345
## 78       Zoo.Area.ha2  ~       Attendance2  6.838     2.632  0.431   0.431
## 79       Zoo.Area.ha2  ~    Total.Animals2 12.532     4.824  0.309   0.309
## 80       Zoo.Area.ha2  ~      Sp.Richness2 10.722     4.128  0.311   0.311
## 81       Zoo.Area.ha2  ~ Mean.Raup.Crick2 19.438     7.483  0.654   0.654
```

```
## 90  Mean.Sp.BodyMass2  ~ Mean.Raup.Crick2  4.482     1.725  0.121   0.121
## 96          X10km_Pop2  ~      Attendance2 11.615     4.471  0.423   0.423
## 97          X10km_Pop2  ~   Total.Animals2  6.353     2.446  0.180   0.180
## 99          X10km_Pop2  ~ Mean.Raup.Crick2 20.413     7.858  0.217   0.217
## 117      Prop.Mam.Sp2  ~ Mean.Raup.Crick2 12.754     4.910 -0.158  -0.158
## 124  Mam.Sp.Richness2  ~   Total.Animals2  8.637     3.325 -0.312  -0.312
## 125  Mam.Sp.Richness2  ~     Sp.Richness2  3.410     1.313 -0.400  -0.400
## 126  Mam.Sp.Richness2  ~ Mean.Raup.Crick2 14.360     5.528 -0.251  -0.251
##     sepc.all sepc.nox
## 56    -0.080   -0.080
## 60     0.059    0.059
## 63     0.068    0.068
## 68    -0.069   -0.069
## 69     0.067    0.067
## 73     0.448    0.448
## 74     0.226    0.227
## 76    -0.346   -0.346
## 78     0.420    0.420
## 79     0.307    0.307
## 80     0.311    0.311
## 81     0.652    0.652
## 90     0.120    0.120
## 96     0.412    0.412
## 97     0.179    0.179
## 99     0.216    0.216
## 117   -0.157   -0.157
## 124   -0.310   -0.310
## 125   -0.400   -0.400
## 126   -0.250   -0.250
```

Model 24

At this point we decided to remove **Sp.Richness2 ~ Prop.Mam.Sp2** from the analysis in a semi-exploratory fashion, as although it is statistically significant, the relationship fails to add much more information than that provided by the existing **Sp.Richness2 ~ Mam.Sp.Richness2** relationship. As a result, we remove this relationship from our model. Once again, the model summary, fit indices and modification indices were all generated for the model, adjusting for the nested nature of data.

```
# Attendance SEM (Presence-Absence)

# Model 24
# Removal of Sp.Richness2 ~ Prop.Mam.Sp2

mod.24 <- 'Attendance2 ~ Zoo.Area.ha2 + Total.Animals2
+ Mean.Sp.BodyMass2 + Mean.Raup.Crick2
+ X10km_Pop2 + GDP.Millions2

Total.Animals2 ~ Zoo.Area.ha2 + Sp.Richness2 + GDP.Millions2
Sp.Richness2 ~Mam.Sp.Richness2
Mean.Raup.Crick2 ~ Total.Animals2 + Mean.Sp.BodyMass2 + Sp.Richness2 + Zoo.Area.ha2'

# Fit model and generate model summary
mod.24.fit <- sem(mod.24, data = sem_attendance_data, fixed.x=FALSE)
```

```r
summary(mod.24.fit, rsq = TRUE)
```

```
## lavaan (0.5-23.1097) converged normally after  26 iterations
##
##   Number of observations                           458
##
##   Estimator                                         ML
##   Minimum Function Test Statistic              216.505
##   Degrees of freedom                                12
##   P-value (Chi-square)                           0.000
##
## Parameter Estimates:
##
##   Information                                 Expected
##   Standard Errors                             Standard
##
## Regressions:
##                      Estimate  Std.Err  z-value  P(>|z|)
##   Attendance2 ~
##     Zoo.Area.ha2        0.082    0.032    2.570    0.010
##     Total.Animals2      0.409    0.028   14.572    0.000
##     Men.Sp.BdyMss2      0.353    0.034   10.368    0.000
##     Mean.Rap.Crck2      0.139    0.029    4.799    0.000
##     X10km_Pop2          0.431    0.025   17.023    0.000
##     GDP.Millions2       0.135    0.025    5.384    0.000
##   Total.Animals2 ~
##     Zoo.Area.ha2        0.219    0.024    9.263    0.000
##     Sp.Richness2        0.805    0.024   34.092    0.000
##     GDP.Millions2      -0.129    0.023   -5.668    0.000
##   Sp.Richness2 ~
##     Mam.Sp.Rchnss2      0.729    0.032   22.790    0.000
##   Mean.Raup.Crick2 ~
##     Total.Animals2      0.477    0.077    6.164    0.000
##     Men.Sp.BdyMss2     -0.573    0.046  -12.399    0.000
##     Sp.Richness2       -0.322    0.075   -4.316    0.000
##     Zoo.Area.ha2        0.156    0.050    3.138    0.002
##
## Covariances:
##                        Estimate  Std.Err  z-value  P(>|z|)
##   Zoo.Area.ha2 ~~
##     Men.Sp.BdyMss2        0.534    0.053   10.096    0.000
##     X10km_Pop2           -0.010    0.047   -0.208    0.835
##     GDP.Millions2        -0.027    0.047   -0.588    0.557
##     Mam.Sp.Rchnss2        0.381    0.050    7.634    0.000
##   Mean.Sp.BodyMass2 ~~
##     X10km_Pop2            0.126    0.047    2.683    0.007
##     GDP.Millions2        -0.082    0.047   -1.748    0.080
##     Mam.Sp.Rchnss2        0.303    0.049    6.216    0.000
##   X10km_Pop2 ~~
##     GDP.Millions2        -0.028    0.047   -0.604    0.546
##     Mam.Sp.Rchnss2        0.284    0.048    5.852    0.000
##   GDP.Millions2 ~~
##     Mam.Sp.Rchnss2       -0.060    0.047   -1.277    0.202
##
```

```
## Variances:
##                    Estimate  Std.Err  z-value  P(>|z|)
##    .Attendance2       0.277    0.018   15.133    0.000
##    .Total.Animals2    0.235    0.016   15.133    0.000
##    .Sp.Richness2      0.468    0.031   15.133    0.000
##    .Mean.Rap.Crck2    0.690    0.046   15.133    0.000
##     Zoo.Area.ha2      0.998    0.066   15.133    0.000
##     Men.Sp.BdyMss2    0.998    0.066   15.133    0.000
##     X10km_Pop2        0.998    0.066   15.133    0.000
##     GDP.Millions2     0.998    0.066   15.133    0.000
##     Mam.Sp.Rchnss2    0.998    0.066   15.133    0.000
##
## R-Square:
##                    Estimate
##     Attendance2       0.719
##     Total.Animals2    0.777
##     Sp.Richness2      0.531
##     Mean.Rap.Crck2    0.293
```

```
# Generate fit indices
fitMeasures(mod.24.fit, c("agfi", "rmr", "srmr", "rmsea", "cfi", "nnfi", "tli"))
```

```
##  agfi   rmr  srmr rmsea   cfi  nnfi   tli
## 0.689 0.093 0.093 0.193 0.895 0.774 0.774
```

```
# Generate modification indices
mi24 <- modindices(mod.24.fit)
print(mi24[mi24$mi > 3.0,])
```

```
##                  lhs op              rhs      mi    epc sepc.lv sepc.all
## 35       Attendance2 ~~      Sp.Richness2   3.007 -0.037  -0.037   -0.037
## 42    Total.Animals2  ~       Attendance2   3.117  0.060   0.060    0.058
## 45    Total.Animals2  ~        X10km_Pop2   7.281  0.063   0.063    0.061
## 47      Sp.Richness2  ~       Attendance2   8.065 -0.126  -0.126   -0.126
## 49      Sp.Richness2  ~  Mean.Raup.Crick2  50.761  0.296   0.296    0.292
## 50      Sp.Richness2  ~      Zoo.Area.ha2  12.709 -0.123  -0.123   -0.123
## 51      Sp.Richness2  ~ Mean.Sp.BodyMass2 111.948 -0.355  -0.355   -0.355
## 52      Sp.Richness2  ~        X10km_Pop2  15.333  0.131   0.131    0.131
## 53      Sp.Richness2  ~     GDP.Millions2   8.442  0.093   0.093    0.093
## 54  Mean.Raup.Crick2  ~       Attendance2  22.546  0.413   0.413    0.415
## 55  Mean.Raup.Crick2  ~        X10km_Pop2  25.052  0.201   0.201    0.204
## 58      Zoo.Area.ha2  ~       Attendance2   5.697  0.300   0.300    0.298
## 59      Zoo.Area.ha2  ~    Total.Animals2   8.687  0.180   0.180    0.185
## 60      Zoo.Area.ha2  ~      Sp.Richness2   5.839  0.132   0.132    0.132
## 61      Zoo.Area.ha2  ~  Mean.Raup.Crick2  23.735  0.711   0.711    0.703
## 66 Mean.Sp.BodyMass2  ~       Attendance2  66.302 -0.959  -0.959   -0.954
## 67 Mean.Sp.BodyMass2  ~    Total.Animals2  68.288 -0.436  -0.436   -0.448
## 68 Mean.Sp.BodyMass2  ~      Sp.Richness2 108.673 -0.590  -0.590   -0.590
## 69 Mean.Sp.BodyMass2  ~  Mean.Raup.Crick2  14.532 -0.540  -0.540   -0.534
## 74        X10km_Pop2  ~       Attendance2  36.930  0.725   0.725    0.721
## 75        X10km_Pop2  ~    Total.Animals2  31.778  0.338   0.338    0.348
## 76        X10km_Pop2  ~      Sp.Richness2  22.652  0.306   0.306    0.306
## 77        X10km_Pop2  ~  Mean.Raup.Crick2  38.558  0.317   0.317    0.313
## 82     GDP.Millions2  ~       Attendance2   4.852  0.397   0.397    0.394
## 83     GDP.Millions2  ~    Total.Animals2   4.453  0.178   0.178    0.183
## 84     GDP.Millions2  ~      Sp.Richness2   4.696  0.147   0.147    0.147
```

```
## 92  Mam.Sp.Richness2  ~      Sp.Richness2   5.979  0.301   0.301    0.301
## 93  Mam.Sp.Richness2  ~  Mean.Raup.Crick2  11.622 -0.216  -0.216   -0.214
##    sepc.nox
## 35   -0.037
## 42    0.058
## 45    0.061
## 47   -0.126
## 49    0.292
## 50   -0.124
## 51   -0.356
## 52    0.131
## 53    0.093
## 54    0.415
## 55    0.204
## 58    0.298
## 59    0.185
## 60    0.132
## 61    0.703
## 66   -0.954
## 67   -0.448
## 68   -0.590
## 69   -0.534
## 74    0.721
## 75    0.348
## 76    0.306
## 77    0.313
## 82    0.394
## 83    0.183
## 84    0.147
## 92    0.301
## 93   -0.214
```

```
# Adjust for the nested nature of the data (institutions within countries)
# Fit model and generate model summary
design <- svydesign(ids = ~Country, nest=TRUE, data=sem_attendance_data)
fit.adj24 <- lavaan.survey(lavaan.fit = mod.24.fit, survey.design = design)
summary(fit.adj24, rsq = TRUE)
```

```
## lavaan (0.5-23.1097) converged normally after  26 iterations
##
##   Number of observations                           458
##
##   Estimator                                         ML      Robust
##   Minimum Function Test Statistic              216.505      68.863
##   Degrees of freedom                                12          12
##   P-value (Chi-square)                           0.000       0.000
##   Scaling correction factor                                  3.144
##     for the Satorra-Bentler correction
##
## Parameter Estimates:
##
##   Information                                 Expected
##   Standard Errors                           Robust.sem
##
## Regressions:
```

```
##                      Estimate  Std.Err  z-value  P(>|z|)
##   Attendance2 ~
##     Zoo.Area.ha2        0.082    0.039    2.090    0.037
##     Total.Animals2      0.409    0.026   15.490    0.000
##     Men.Sp.BdyMss2      0.353    0.031   11.231    0.000
##     Mean.Rap.Crck2      0.139    0.027    5.088    0.000
##     X10km_Pop2          0.431    0.031   13.796    0.000
##     GDP.Millions2       0.135    0.026    5.154    0.000
##   Total.Animals2 ~
##     Zoo.Area.ha2        0.219    0.043    5.132    0.000
##     Sp.Richness2        0.805    0.055   14.683    0.000
##     GDP.Millions2      -0.129    0.072   -1.796    0.072
##   Sp.Richness2 ~
##     Mam.Sp.Rchnss2      0.729    0.064   11.465    0.000
##   Mean.Raup.Crick2 ~
##     Total.Animals2      0.477    0.151    3.167    0.002
##     Men.Sp.BdyMss2     -0.573    0.075   -7.607    0.000
##     Sp.Richness2       -0.322    0.240   -1.345    0.179
##     Zoo.Area.ha2        0.156    0.048    3.226    0.001
##
## Covariances:
##                        Estimate  Std.Err  z-value  P(>|z|)
##   Zoo.Area.ha2 ~~
##     Men.Sp.BdyMss2        0.534    0.104    5.147    0.000
##     X10km_Pop2           -0.010    0.078   -0.124    0.901
##     GDP.Millions2        -0.027    0.041   -0.673    0.501
##     Mam.Sp.Rchnss2        0.381    0.064    5.985    0.000
##   Mean.Sp.BodyMass2 ~~
##     X10km_Pop2            0.126    0.067    1.895    0.058
##     GDP.Millions2        -0.082    0.046   -1.759    0.079
##     Mam.Sp.Rchnss2        0.303    0.081    3.730    0.000
##   X10km_Pop2 ~~
##     GDP.Millions2        -0.028    0.056   -0.505    0.613
##     Mam.Sp.Rchnss2        0.284    0.066    4.278    0.000
##   GDP.Millions2 ~~
##     Mam.Sp.Rchnss2       -0.060    0.064   -0.937    0.349
##
## Intercepts:
##                    Estimate  Std.Err  z-value  P(>|z|)
##    .Attendance2      -0.000    0.029   -0.000    1.000
##    .Total.Animals2   -0.000    0.070   -0.000    1.000
##    .Sp.Richness2      0.000    0.088    0.000    1.000
##    .Mean.Rap.Crck2    0.000    0.089    0.000    1.000
##     Zoo.Area.ha2      0.000    0.064    0.000    1.000
##     Men.Sp.BdyMss2   -0.000    0.080   -0.000    1.000
##     X10km_Pop2        0.000    0.105    0.000    1.000
##     GDP.Millions2    -0.000    0.345   -0.000    1.000
##     Mam.Sp.Rchnss2   -0.000    0.079   -0.000    1.000
##
## Variances:
##                    Estimate  Std.Err  z-value  P(>|z|)
##    .Attendance2       0.277    0.028   10.008    0.000
##    .Total.Animals2    0.235    0.038    6.139    0.000
##    .Sp.Richness2      0.468    0.066    7.056    0.000
```

```
##    .Mean.Rap.Crck2    0.690    0.061   11.232    0.000
##     Zoo.Area.ha2      0.998    0.112    8.886    0.000
##     Men.Sp.BdyMss2    0.998    0.173    5.783    0.000
##     X10km_Pop2        0.998    0.174    5.745    0.000
##     GDP.Millions2     0.998    0.246    4.061    0.000
##     Mam.Sp.Rchnss2    0.998    0.115    8.669    0.000
##
## R-Square:
##                    Estimate
##     Attendance2       0.719
##     Total.Animals2    0.777
##     Sp.Richness2      0.531
##     Mean.Rap.Crck2    0.293
```

```
# Generate fit indices
fitMeasures(fit.adj24, c("agfi", "rmr", "srmr", "rmsea", "cfi", "nnfi", "tli"))
```

```
##  agfi   rmr  srmr rmsea   cfi  nnfi   tli
## 0.626 0.093 0.085 0.193 0.895 0.774 0.774
```

```
# Generate modification indices
mi24adj <- modindices(fit.adj24)
print(mi24adj[mi24adj$mi > 3.0,])
```

```
##                    lhs op              rhs      mi mi.scaled    epc
## 44         Attendance2 ~~     Sp.Richness2   3.007     0.956 -0.037
## 51      Total.Animals2  ~      Attendance2   3.117     0.992  0.060
## 54      Total.Animals2  ~       X10km_Pop2   7.281     2.316  0.063
## 56        Sp.Richness2  ~      Attendance2   8.065     2.565 -0.126
## 58        Sp.Richness2  ~  Mean.Raup.Crick2  50.761    16.145  0.296
## 59        Sp.Richness2  ~     Zoo.Area.ha2  12.709     4.042 -0.123
## 60        Sp.Richness2  ~ Mean.Sp.BodyMass2 111.948    35.607 -0.355
## 61        Sp.Richness2  ~       X10km_Pop2  15.333     4.877  0.131
## 62        Sp.Richness2  ~    GDP.Millions2   8.442     2.685  0.093
## 63    Mean.Raup.Crick2  ~      Attendance2  22.546     7.171  0.413
## 64    Mean.Raup.Crick2  ~       X10km_Pop2  25.052     7.968  0.201
## 67        Zoo.Area.ha2  ~      Attendance2   5.697     1.812  0.300
## 68        Zoo.Area.ha2  ~   Total.Animals2   8.687     2.763  0.180
## 69        Zoo.Area.ha2  ~     Sp.Richness2   5.839     1.857  0.132
## 70        Zoo.Area.ha2  ~  Mean.Raup.Crick2  23.735     7.549  0.711
## 75   Mean.Sp.BodyMass2  ~      Attendance2  66.302    21.088 -0.959
## 76   Mean.Sp.BodyMass2  ~   Total.Animals2  68.288    21.720 -0.436
## 77   Mean.Sp.BodyMass2  ~     Sp.Richness2 108.673    34.565 -0.590
## 78   Mean.Sp.BodyMass2  ~  Mean.Raup.Crick2  14.532     4.622 -0.540
## 83          X10km_Pop2  ~      Attendance2  36.930    11.746  0.725
## 84          X10km_Pop2  ~   Total.Animals2  31.778    10.108  0.338
## 85          X10km_Pop2  ~     Sp.Richness2  22.652     7.205  0.306
## 86          X10km_Pop2  ~  Mean.Raup.Crick2  38.558    12.264  0.317
## 91       GDP.Millions2  ~      Attendance2   4.852     1.543  0.397
## 92       GDP.Millions2  ~   Total.Animals2   4.453     1.416  0.178
## 93       GDP.Millions2  ~     Sp.Richness2   4.696     1.493  0.147
## 101   Mam.Sp.Richness2  ~     Sp.Richness2   5.979     1.902  0.301
## 102   Mam.Sp.Richness2  ~  Mean.Raup.Crick2  11.622     3.697 -0.216
##     sepc.lv sepc.all sepc.nox
## 44   -0.037   -0.037   -0.037
## 51    0.060    0.058    0.058
```

```
## 54    0.063    0.061    0.061
## 56   -0.126   -0.126   -0.126
## 58    0.296    0.292    0.292
## 59   -0.123   -0.123   -0.124
## 60   -0.355   -0.355   -0.356
## 61    0.131    0.131    0.131
## 62    0.093    0.093    0.093
## 63    0.413    0.415    0.415
## 64    0.201    0.204    0.204
## 67    0.300    0.298    0.298
## 68    0.180    0.185    0.185
## 69    0.132    0.132    0.132
## 70    0.711    0.703    0.703
## 75   -0.959   -0.954   -0.954
## 76   -0.436   -0.448   -0.448
## 77   -0.590   -0.590   -0.590
## 78   -0.540   -0.534   -0.534
## 83    0.725    0.721    0.721
## 84    0.338    0.348    0.348
## 85    0.306    0.306    0.306
## 86    0.317    0.313    0.313
## 91    0.397    0.394    0.394
## 92    0.178    0.183    0.183
## 93    0.147    0.147    0.147
## 101   0.301    0.301    0.301
## 102  -0.216   -0.214   -0.214
```

Model 25

Based on the modification indices generated from the nested twenty fourth model, we can see that a new relationship has been suggested **Sp.Richness2 ~ Mean.Sp.BodyMass2**, which has the highest mi value of **111.948**,exceeding the standard cut-off level for the chi-square test criterion of 3.84 (Burnham and Anderson, 2002). This relationship is to be expected, as if institutions have larger animals, then owing to their limited carrying capacity and space availability, then they will be able to house fewer species. As a result, we add this relationship to our model. Once again, the model summary, fit indices and modification indices were all generated for the model, adjusting for the nested nature of data.

```
# Attendance SEM (Presence-Absence)

# Model 25
# Addition of Sp.Richness2 ~ Mean.Sp.BodyMass2, mi = 11.948

mod.25 <- 'Attendance2 ~ Zoo.Area.ha2 + Total.Animals2
+ Mean.Sp.BodyMass2 + Mean.Raup.Crick2
+ X10km_Pop2 + GDP.Millions2

Total.Animals2 ~ Zoo.Area.ha2 + Sp.Richness2 + GDP.Millions2
Sp.Richness2 ~Mam.Sp.Richness2 + Mean.Sp.BodyMass2
Mean.Raup.Crick2 ~ Total.Animals2 + Mean.Sp.BodyMass2 + Sp.Richness2 + Zoo.Area.ha2'

# Fit model and generate model summary
mod.25.fit <- sem(mod.25, data = sem_attendance_data, fixed.x=FALSE)
summary(mod.25.fit, rsq = TRUE)
```

```
## lavaan (0.5-23.1097) converged normally after  32 iterations
##
##   Number of observations                           458
##
##   Estimator                                         ML
##   Minimum Function Test Statistic               88.137
##   Degrees of freedom                                11
##   P-value (Chi-square)                           0.000
##
## Parameter Estimates:
##
##   Information                                 Expected
##   Standard Errors                             Standard
##
## Regressions:
##                      Estimate  Std.Err  z-value  P(>|z|)
##   Attendance2 ~
##     Zoo.Area.ha2        0.082    0.032    2.550    0.011
##     Total.Animals2      0.409    0.028   14.658    0.000
##     Men.Sp.BdyMss2      0.353    0.034   10.339    0.000
##     Mean.Rap.Crck2      0.139    0.029    4.799    0.000
##     X10km_Pop2          0.431    0.025   16.943    0.000
##     GDP.Millions2       0.135    0.025    5.382    0.000
##   Total.Animals2 ~
##     Zoo.Area.ha2        0.219    0.023    9.561    0.000
##     Sp.Richness2        0.805    0.023   35.215    0.000
##     GDP.Millions2      -0.129    0.023   -5.671    0.000
##   Sp.Richness2 ~
##     Mam.Sp.Rchnss2      0.837    0.029   28.676    0.000
##     Men.Sp.BdyMss2     -0.355    0.029  -12.172    0.000
##   Mean.Raup.Crick2 ~
##     Total.Animals2      0.477    0.077    6.164    0.000
##     Men.Sp.BdyMss2     -0.573    0.047  -12.187    0.000
##     Sp.Richness2       -0.322    0.074   -4.333    0.000
##     Zoo.Area.ha2        0.156    0.050    3.124    0.002
##
## Covariances:
##                        Estimate  Std.Err  z-value  P(>|z|)
##   Zoo.Area.ha2 ~~
##     Men.Sp.BdyMss2        0.534    0.053   10.096    0.000
##     X10km_Pop2           -0.010    0.047   -0.208    0.835
##     GDP.Millions2        -0.027    0.047   -0.588    0.557
##     Mam.Sp.Rchnss2        0.381    0.050    7.634    0.000
##   Mean.Sp.BodyMass2 ~~
##     X10km_Pop2            0.126    0.047    2.683    0.007
##     GDP.Millions2        -0.082    0.047   -1.748    0.080
##     Mam.Sp.Rchnss2        0.303    0.049    6.216    0.000
##   X10km_Pop2 ~~
##     GDP.Millions2        -0.028    0.047   -0.604    0.546
##     Mam.Sp.Rchnss2        0.284    0.048    5.852    0.000
##   GDP.Millions2 ~~
##     Mam.Sp.Rchnss2       -0.060    0.047   -1.277    0.202
##
## Variances:
```

```
##                    Estimate  Std.Err  z-value  P(>|z|)
##    .Attendance2        0.277    0.018   15.133    0.000
##    .Total.Animals2     0.235    0.016   15.133    0.000
##    .Sp.Richness2       0.353    0.023   15.133    0.000
##    .Mean.Rap.Crck2     0.690    0.046   15.133    0.000
##     Zoo.Area.ha2       0.998    0.066   15.133    0.000
##     Men.Sp.BdyMss2     0.998    0.066   15.133    0.000
##     X10km_Pop2         0.998    0.066   15.133    0.000
##     GDP.Millions2      0.998    0.066   15.133    0.000
##     Mam.Sp.Rchnss2     0.998    0.066   15.133    0.000
##
## R-Square:
##                    Estimate
##     Attendance2        0.693
##     Total.Animals2     0.765
##     Sp.Richness2       0.646
##     Mean.Rap.Crck2     0.305
```

```
# Generate fit indices
fitMeasures(mod.25.fit, c("agfi", "rmr", "srmr", "rmsea", "cfi", "nnfi", "tli"))
```

```
##  agfi   rmr  srmr rmsea   cfi  nnfi   tli
## 0.836 0.058 0.058 0.124 0.961 0.907 0.907
```

```
# Generate modification indices
mi25 <- modindices(mod.25.fit)
print(mi25[mi25$mi > 3.0,])
```

```
##                  lhs op             rhs     mi    epc sepc.lv sepc.all
## 36       Attendance2 ~~     Sp.Richness2  4.358 -0.036  -0.036   -0.038
## 43    Total.Animals2  ~      Attendance2  3.033  0.058   0.058    0.055
## 46    Total.Animals2  ~       X10km_Pop2  7.211  0.062   0.062    0.062
## 48      Sp.Richness2  ~      Attendance2  9.432  0.132   0.132    0.125
## 51      Sp.Richness2  ~     Zoo.Area.ha2  3.704  0.066   0.066    0.066
## 52      Sp.Richness2  ~       X10km_Pop2 25.438  0.146   0.146    0.146
## 53      Sp.Richness2  ~    GDP.Millions2  6.409  0.071   0.071    0.071
## 54  Mean.Raup.Crick2  ~      Attendance2 22.769  0.417   0.417    0.398
## 55  Mean.Raup.Crick2  ~       X10km_Pop2 25.373  0.204   0.204    0.204
## 58      Zoo.Area.ha2  ~      Attendance2  6.734  0.355   0.355    0.337
## 59      Zoo.Area.ha2  ~   Total.Animals2 10.477  0.217   0.217    0.217
## 60      Zoo.Area.ha2  ~     Sp.Richness2  7.729  0.175   0.175    0.175
## 61      Zoo.Area.ha2  ~ Mean.Raup.Crick2 25.670  0.769   0.769    0.767
## 66 Mean.Sp.BodyMass2  ~      Attendance2  5.054 -0.357  -0.357   -0.339
## 67 Mean.Sp.BodyMass2  ~   Total.Animals2  3.638 -0.142  -0.142   -0.142
## 68 Mean.Sp.BodyMass2  ~     Sp.Richness2  8.060 -0.376  -0.376   -0.376
## 69 Mean.Sp.BodyMass2  ~ Mean.Raup.Crick2  6.328 -0.376  -0.376   -0.375
## 74        X10km_Pop2  ~      Attendance2 41.678  0.818   0.818    0.778
## 75        X10km_Pop2  ~   Total.Animals2 36.854  0.392   0.392    0.392
## 76        X10km_Pop2  ~     Sp.Richness2 29.981  0.405   0.405    0.405
## 77        X10km_Pop2  ~ Mean.Raup.Crick2 38.581  0.317   0.317    0.316
## 82     GDP.Millions2  ~      Attendance2  6.033  0.493   0.493    0.469
## 83     GDP.Millions2  ~   Total.Animals2  5.877  0.234   0.234    0.234
## 84     GDP.Millions2  ~     Sp.Richness2  6.215  0.195   0.195    0.195
## 91  Mam.Sp.Richness2  ~   Total.Animals2 23.138 -0.519  -0.519   -0.519
## 92  Mam.Sp.Richness2  ~     Sp.Richness2 26.006 -0.881  -0.881   -0.881
## 93  Mam.Sp.Richness2  ~ Mean.Raup.Crick2 16.662 -0.293  -0.293   -0.293
```

```
##    sepc.nox
## 36   -0.038
## 43    0.055
## 46    0.062
## 48    0.125
## 51    0.066
## 52    0.147
## 53    0.071
## 54    0.398
## 55    0.205
## 58    0.337
## 59    0.217
## 60    0.175
## 61    0.767
## 66   -0.339
## 67   -0.142
## 68   -0.376
## 69   -0.375
## 74    0.778
## 75    0.392
## 76    0.405
## 77    0.316
## 82    0.469
## 83    0.234
## 84    0.195
## 91   -0.519
## 92   -0.881
## 93   -0.293
```

```
# Adjust for the nested nature of the data (institutions within countries)
# Fit model and generate model summary
design <- svydesign(ids = ~Country, nest=TRUE, data=sem_attendance_data)
fit.adj25 <- lavaan.survey(lavaan.fit = mod.25.fit, survey.design = design)
summary(fit.adj25, rsq = TRUE)
```

```
## lavaan (0.5-23.1097) converged normally after  32 iterations
##
##   Number of observations                           458
##
##   Estimator                                         ML      Robust
##   Minimum Function Test Statistic               88.137      34.901
##   Degrees of freedom                                11          11
##   P-value (Chi-square)                           0.000       0.000
##   Scaling correction factor                                  2.525
##     for the Satorra-Bentler correction
##
## Parameter Estimates:
##
##   Information                                 Expected
##   Standard Errors                           Robust.sem
##
## Regressions:
##                    Estimate  Std.Err  z-value  P(>|z|)
##   Attendance2 ~
##     Zoo.Area.ha2      0.082    0.039    2.121    0.034
```

```
##     Total.Animals2      0.409    0.025   16.423    0.000
##     Men.Sp.BdyMss2      0.353    0.031   11.362    0.000
##     Mean.Rap.Crck2      0.139    0.028    5.014    0.000
##     X10km_Pop2          0.431    0.032   13.549    0.000
##     GDP.Millions2       0.135    0.027    5.067    0.000
##   Total.Animals2 ~
##     Zoo.Area.ha2        0.219    0.035    6.305    0.000
##     Sp.Richness2        0.805    0.048   16.640    0.000
##     GDP.Millions2      -0.129    0.072   -1.796    0.072
##   Sp.Richness2 ~
##     Mam.Sp.Rchnss2      0.837    0.064   13.051    0.000
##     Men.Sp.BdyMss2     -0.355    0.043   -8.283    0.000
##   Mean.Raup.Crick2 ~
##     Total.Animals2      0.477    0.151    3.168    0.002
##     Men.Sp.BdyMss2     -0.573    0.055  -10.334    0.000
##     Sp.Richness2       -0.322    0.220   -1.463    0.143
##     Zoo.Area.ha2        0.156    0.049    3.196    0.001
##
## Covariances:
##                        Estimate  Std.Err  z-value  P(>|z|)
##   Zoo.Area.ha2 ~~
##     Men.Sp.BdyMss2        0.534    0.104    5.147    0.000
##     X10km_Pop2           -0.010    0.078   -0.124    0.901
##     GDP.Millions2        -0.027    0.041   -0.673    0.501
##     Mam.Sp.Rchnss2        0.381    0.064    5.985    0.000
##   Mean.Sp.BodyMass2 ~~
##     X10km_Pop2            0.126    0.067    1.895    0.058
##     GDP.Millions2        -0.082    0.046   -1.759    0.079
##     Mam.Sp.Rchnss2        0.303    0.081    3.730    0.000
##   X10km_Pop2 ~~
##     GDP.Millions2        -0.028    0.056   -0.505    0.613
##     Mam.Sp.Rchnss2        0.284    0.066    4.278    0.000
##   GDP.Millions2 ~~
##     Mam.Sp.Rchnss2       -0.060    0.064   -0.937    0.349
##
## Intercepts:
##                    Estimate  Std.Err  z-value  P(>|z|)
##    .Attendance2      -0.000    0.029   -0.000    1.000
##    .Total.Animals2   -0.000    0.070   -0.000    1.000
##    .Sp.Richness2      0.000    0.070    0.000    1.000
##    .Mean.Rap.Crck2    0.000    0.089    0.000    1.000
##     Zoo.Area.ha2      0.000    0.064    0.000    1.000
##     Men.Sp.BdyMss2   -0.000    0.080   -0.000    1.000
##     X10km_Pop2        0.000    0.105    0.000    1.000
##     GDP.Millions2    -0.000    0.345   -0.000    1.000
##     Mam.Sp.Rchnss2   -0.000    0.079   -0.000    1.000
##
## Variances:
##                    Estimate  Std.Err  z-value  P(>|z|)
##    .Attendance2       0.277    0.028   10.008    0.000
##    .Total.Animals2    0.235    0.038    6.139    0.000
##    .Sp.Richness2      0.353    0.036    9.948    0.000
##    .Mean.Rap.Crck2    0.690    0.061   11.233    0.000
##     Zoo.Area.ha2      0.998    0.112    8.886    0.000
```

```
##     Men.Sp.BdyMss2    0.998    0.173    5.783    0.000
##     X10km_Pop2        0.998    0.174    5.745    0.000
##     GDP.Millions2     0.998    0.246    4.061    0.000
##     Mam.Sp.Rchnss2    0.998    0.115    8.669    0.000
##
## R-Square:
##                    Estimate
##     Attendance2       0.693
##     Total.Animals2    0.765
##     Sp.Richness2      0.646
##     Mean.Rap.Crck2    0.305
```

```
# Generate fit indices
fitMeasures(fit.adj25, c("agfi", "rmr", "srmr", "rmsea", "cfi", "nnfi", "tli"))
```

```
##  agfi   rmr  srmr rmsea   cfi  nnfi   tli
## 0.803 0.058 0.053 0.124 0.961 0.907 0.907
```

```
# Generate modification indices
mi25adj <- modindices(fit.adj25)
print(mi25adj[mi25adj$mi > 3.0,])
```

```
##                   lhs op             rhs     mi mi.scaled    epc sepc.lv
## 45        Attendance2 ~~    Sp.Richness2  4.358     1.726 -0.036  -0.036
## 52     Total.Animals2  ~     Attendance2  3.033     1.201  0.058   0.058
## 55     Total.Animals2  ~      X10km_Pop2  7.211     2.856  0.062   0.062
## 57       Sp.Richness2  ~     Attendance2  9.432     3.735  0.132   0.132
## 60       Sp.Richness2  ~     Zoo.Area.ha2  3.704     1.467  0.066   0.066
## 61       Sp.Richness2  ~      X10km_Pop2 25.438    10.073  0.146   0.146
## 62       Sp.Richness2  ~   GDP.Millions2  6.409     2.538  0.071   0.071
## 63   Mean.Raup.Crick2  ~     Attendance2 22.769     9.016  0.417   0.417
## 64   Mean.Raup.Crick2  ~      X10km_Pop2 25.373    10.047  0.204   0.204
## 67       Zoo.Area.ha2  ~     Attendance2  6.734     2.666  0.355   0.355
## 68       Zoo.Area.ha2  ~  Total.Animals2 10.477     4.149  0.217   0.217
## 69       Zoo.Area.ha2  ~    Sp.Richness2  7.729     3.060  0.175   0.175
## 70       Zoo.Area.ha2  ~ Mean.Raup.Crick2 25.670    10.165  0.769   0.769
## 75  Mean.Sp.BodyMass2  ~     Attendance2  5.054     2.001 -0.357  -0.357
## 76  Mean.Sp.BodyMass2  ~  Total.Animals2  3.638     1.441 -0.142  -0.142
## 77  Mean.Sp.BodyMass2  ~    Sp.Richness2  8.060     3.192 -0.376  -0.376
## 78  Mean.Sp.BodyMass2  ~ Mean.Raup.Crick2  6.328     2.506 -0.376  -0.376
## 83         X10km_Pop2  ~     Attendance2 41.678    16.504  0.818   0.818
## 84         X10km_Pop2  ~  Total.Animals2 36.854    14.594  0.392   0.392
## 85         X10km_Pop2  ~    Sp.Richness2 29.981    11.872  0.405   0.405
## 86         X10km_Pop2  ~ Mean.Raup.Crick2 38.581    15.278  0.317   0.317
## 91      GDP.Millions2  ~     Attendance2  6.033     2.389  0.493   0.493
## 92      GDP.Millions2  ~  Total.Animals2  5.877     2.327  0.234   0.234
## 93      GDP.Millions2  ~    Sp.Richness2  6.215     2.461  0.195   0.195
## 100  Mam.Sp.Richness2  ~  Total.Animals2 23.138     9.163 -0.519  -0.519
## 101  Mam.Sp.Richness2  ~    Sp.Richness2 26.006    10.298 -0.881  -0.881
## 102  Mam.Sp.Richness2  ~ Mean.Raup.Crick2 16.662     6.598 -0.293  -0.293
##     sepc.all sepc.nox
## 45    -0.038   -0.038
## 52     0.055    0.055
## 55     0.062    0.062
## 57     0.125    0.125
## 60     0.066    0.066
```

```
## 61      0.146    0.147
## 62      0.071    0.071
## 63      0.398    0.398
## 64      0.204    0.205
## 67      0.337    0.337
## 68      0.217    0.217
## 69      0.175    0.175
## 70      0.767    0.767
## 75     -0.339   -0.339
## 76     -0.142   -0.142
## 77     -0.376   -0.376
## 78     -0.375   -0.375
## 83      0.778    0.778
## 84      0.392    0.392
## 85      0.405    0.405
## 86      0.316    0.316
## 91      0.469    0.469
## 92      0.234    0.234
## 93      0.195    0.195
## 100    -0.519   -0.519
## 101    -0.881   -0.881
## 102    -0.293   -0.293
```

Model Comparisons 3

At this stage no more addition seems conceptually appealling, so we again compare the models generated using AICc values. Overall model selection from the pool of competing models is achieved using AICc values, with a threshold of more than 2 AICc units lower than nearest competing model being considered sufficient for model selection. Based on these results it is clear that model 25 is superior.

```
# Model Comparisons using AICc

# Comparing models with and without adjustment for nested nature of data
# library(AICcmodavg)
# source("lavaan.modavg.R")

aictab.lavaan(list(mod.1.fit, mod.2.fit, mod.3.fit, mod.4.fit, mod.5.fit, mod.6.fit, mod.7.fit, mod.8.fi
              c("mod.1", "mod.2", "mod.3", "mod.4", "mod.5", "mod.6", "mod.7", "mod.8", "mod.9", "mod.1
```

```
##
## Model selection based on AICc:
##
##                K     AICc Delta_AICc AICcWt Cum.Wt       LL
## mod25         34  9571.54       0.00      1      1 -4751.23
## mod.25.nested 43  9589.54      18.00      0      1 -4751.23
## mod24         33  9697.77     126.23      0      1 -4815.41
## mod.24.nested 42  9715.77     144.23      0      1 -4815.41
## mod23         40 10224.94     653.40      0      1 -5071.93
## mod22         39 10232.14     660.60      0      1 -5076.60
## mod.23.nested 50 10244.94     673.40      0      1 -5071.93
## mod.22.nested 49 10252.14     680.60      0      1 -5076.60
## mod21         38 10252.58     681.04      0      1 -5087.88
## mod.21.nested 48 10272.58     701.04      0      1 -5087.88
## mod20         37 10281.46     709.92      0      1 -5103.38
```

```
## mod.20.nested 47 10301.46     729.92       0       1 -5103.38
## mod.18        43 10955.33    1383.79       0       1 -5433.97
## mod19         42 10955.82    1384.28       0       1 -5435.30
## mod.18.nested 54 10977.33    1405.79       0       1 -5433.97
## mod.19.nested 53 10977.82    1406.28       0       1 -5435.30
## mod.17.nested 63 11890.76    2319.22       0       1 -5881.60
## mod.16.nested 64 11861.94    2290.40       0       1 -5866.10
## mod.15.nested 74 12530.10    2958.56       0       1 -6190.09
## mod.14.nested 75 12509.73    2938.19       0       1 -6178.80
## mod.13.nested 76 12501.65    2930.11       0       1 -6173.66
## mod.12.nested 77 12501.97    2930.43       0       1 -6172.71
## mod.11.nested 78 12502.71    2931.17       0       1 -6171.97
## mod.10.nested 78 12502.71    2931.17       0       1 -6171.97
## mod.9.nested  79 12503.71    2932.18       0       1 -6171.35
## mod.8.nested  85 13749.39    4177.85       0       1 -6787.66
## mod.7.nested  84 13763.59    4192.05       0       1 -6795.90
## mod.6.nested  83 13762.55    4191.01       0       1 -6796.52
## mod.5.nested  82 13791.29    4219.75       0       1 -6812.02
## mod.4.nested  81 13835.38    4263.84       0       1 -6835.18
## mod.3.nested  80 13900.33    4328.79       0       1 -6868.78
## mod.2.nested  79 14027.63    4456.09       0       1 -6933.54
## mod.1.nested  78 14684.32    5112.78       0       1 -7263.00
## mod.17        51 11866.76    2295.22       0       1 -5881.60
## mod.16        52 11837.94    2266.40       0       1 -5866.10
## mod.15        61 12504.10    2932.56       0       1 -6190.09
## mod.14        62 12483.73    2912.19       0       1 -6178.80
## mod.13        63 12475.65    2904.11       0       1 -6173.66
## mod.12        64 12475.97    2904.43       0       1 -6172.71
## mod.11        65 12476.71    2905.17       0       1 -6171.97
## mod.10        65 12476.71    2905.17       0       1 -6171.97
## mod.9         66 12477.71    2906.18       0       1 -6171.35
## mod.8         71 13721.39    4149.85       0       1 -6787.66
## mod.7         70 13735.59    4164.05       0       1 -6795.90
## mod.6         69 13734.55    4163.01       0       1 -6796.52
## mod.5         68 13763.29    4191.75       0       1 -6812.02
## mod.4         67 13807.38    4235.84       0       1 -6835.18
## mod.3         66 13872.33    4300.79       0       1 -6868.78
## mod.2         65 13999.63    4428.09       0       1 -6933.54
## mod.1         64 14656.32    5084.78       0       1 -7263.00
```

Now we look to the three remaining non-significant relationships found in Model 25 and investigate whether each one is justified in its retention.

Model 26

Removal of non-significant relationship **Attendance2** ~ **Zoo.Area.ha2** and testing whether this is justified.

```
# Attendance SEM (Presence-Absence)

# Model 26
# Removal of Attendance2 ~ Zoo.Area.ha2 from model 25, p =

mod.26 <- 'Attendance2 ~ Total.Animals2
+ Mean.Sp.BodyMass2 + Mean.Raup.Crick2
```

```r
+ X10km_Pop2 + GDP.Millions2

Total.Animals2 ~ Zoo.Area.ha2 + Sp.Richness2 + GDP.Millions2
Sp.Richness2 ~Mam.Sp.Richness2 + Mean.Sp.BodyMass2
Mean.Raup.Crick2 ~ Total.Animals2 + Mean.Sp.BodyMass2 + Sp.Richness2 + Zoo.Area.ha2'

# Fit model and generate model summary
mod.26.fit <- sem(mod.26, data = sem_attendance_data, fixed.x=FALSE)
summary(mod.26.fit, rsq = TRUE)
```

```
## lavaan (0.5-23.1097) converged normally after  32 iterations
##
##   Number of observations                           458
##
##   Estimator                                         ML
##   Minimum Function Test Statistic               93.965
##   Degrees of freedom                                12
##   P-value (Chi-square)                           0.000
##
## Parameter Estimates:
##
##   Information                                 Expected
##   Standard Errors                             Standard
##
## Regressions:
##                     Estimate  Std.Err  z-value  P(>|z|)
##   Attendance2 ~
##     Total.Animals2     0.440    0.026   16.623    0.000
##     Men.Sp.BdyMss2     0.406    0.028   14.437    0.000
##     Mean.Rap.Crck2     0.156    0.029    5.404    0.000
##     X10km_Pop2         0.411    0.025   16.250    0.000
##     GDP.Millions2      0.139    0.025    5.543    0.000
##   Total.Animals2 ~
##     Zoo.Area.ha2       0.219    0.023    9.561    0.000
##     Sp.Richness2       0.805    0.023   35.215    0.000
##     GDP.Millions2     -0.129    0.023   -5.671    0.000
##   Sp.Richness2 ~
##     Mam.Sp.Rchnss2     0.837    0.029   28.676    0.000
##     Men.Sp.BdyMss2    -0.355    0.029  -12.172    0.000
##   Mean.Raup.Crick2 ~
##     Total.Animals2     0.477    0.077    6.164    0.000
##     Men.Sp.BdyMss2    -0.573    0.047  -12.187    0.000
##     Sp.Richness2      -0.322    0.074   -4.333    0.000
##     Zoo.Area.ha2       0.156    0.050    3.124    0.002
##
## Covariances:
##                       Estimate  Std.Err  z-value  P(>|z|)
##   Mean.Sp.BodyMass2 ~~
##     X10km_Pop2           0.126    0.047    2.683    0.007
##     GDP.Millions2       -0.082    0.047   -1.748    0.080
##     Zoo.Area.ha2         0.534    0.053   10.096    0.000
##     Mam.Sp.Rchnss2       0.303    0.049    6.216    0.000
##   X10km_Pop2 ~~
##     GDP.Millions2       -0.028    0.047   -0.604    0.546
```

```
##     Zoo.Area.ha2         -0.010    0.047   -0.208    0.835
##     Mam.Sp.Rchnss2        0.284    0.048    5.852    0.000
##   GDP.Millions2 ~~
##     Zoo.Area.ha2         -0.027    0.047   -0.588    0.557
##     Mam.Sp.Rchnss2       -0.060    0.047   -1.277    0.202
##   Zoo.Area.ha2 ~~
##     Mam.Sp.Rchnss2        0.381    0.050    7.634    0.000
##
## Variances:
##                    Estimate  Std.Err  z-value  P(>|z|)
##    .Attendance2       0.281    0.019   15.133    0.000
##    .Total.Animals2    0.235    0.016   15.133    0.000
##    .Sp.Richness2      0.353    0.023   15.133    0.000
##    .Mean.Rap.Crck2    0.690    0.046   15.133    0.000
##     Men.Sp.BdyMss2    0.998    0.066   15.133    0.000
##     X10km_Pop2        0.998    0.066   15.133    0.000
##     GDP.Millions2     0.998    0.066   15.133    0.000
##     Zoo.Area.ha2      0.998    0.066   15.133    0.000
##     Mam.Sp.Rchnss2    0.998    0.066   15.133    0.000
##
## R-Square:
##                    Estimate
##     Attendance2       0.689
##     Total.Animals2    0.765
##     Sp.Richness2      0.646
##     Mean.Rap.Crck2    0.305
```

```
# Generate fit indices
fitMeasures(mod.26.fit, c("agfi", "rmr", "srmr", "rmsea", "cfi", "nnfi", "tli"))
```

```
##  agfi   rmr  srmr rmsea   cfi  nnfi   tli
## 0.842 0.059 0.059 0.122 0.958 0.909 0.909
```

```
# Generate modification indices
mi26 <- modindices(mod.26.fit)
print(mi26[mi26$mi > 3.0,])
```

```
##                   lhs op               rhs     mi    epc sepc.lv sepc.all
## 35        Attendance2 ~~     Sp.Richness2  5.500 -0.040  -0.040   -0.042
## 41        Attendance2  ~     Zoo.Area.ha2  5.224  0.074   0.074    0.078
## 42        Attendance2  ~ Mam.Sp.Richness2  3.118  0.066   0.066    0.070
## 46     Total.Animals2  ~       X10km_Pop2  7.211  0.062   0.062    0.062
## 48       Sp.Richness2  ~      Attendance2  6.629  0.111   0.111    0.105
## 51       Sp.Richness2  ~       X10km_Pop2 25.438  0.146   0.146    0.146
## 52       Sp.Richness2  ~    GDP.Millions2  6.409  0.071   0.071    0.071
## 53       Sp.Richness2  ~     Zoo.Area.ha2  3.704  0.066   0.066    0.066
## 54   Mean.Raup.Crick2  ~      Attendance2 17.547  0.372   0.372    0.354
## 55   Mean.Raup.Crick2  ~       X10km_Pop2 25.373  0.204   0.204    0.204
## 58 Mean.Sp.BodyMass2  ~      Attendance2 10.188 -0.341  -0.341   -0.324
## 59 Mean.Sp.BodyMass2  ~    Total.Animals2  3.638 -0.142  -0.142   -0.142
## 60 Mean.Sp.BodyMass2  ~     Sp.Richness2  8.060 -0.376  -0.376   -0.376
## 61 Mean.Sp.BodyMass2  ~ Mean.Raup.Crick2  6.328 -0.376  -0.376   -0.375
## 66         X10km_Pop2  ~      Attendance2 47.999  0.799   0.799    0.760
## 67         X10km_Pop2  ~    Total.Animals2 36.854  0.392   0.392    0.392
## 68         X10km_Pop2  ~     Sp.Richness2 29.981  0.405   0.405    0.405
## 69         X10km_Pop2  ~ Mean.Raup.Crick2 38.581  0.317   0.317    0.316
```

```
## 74     GDP.Millions2  ~      Attendance2  5.308  0.426   0.426    0.405
## 75     GDP.Millions2  ~   Total.Animals2  5.877  0.234   0.234    0.234
## 76     GDP.Millions2  ~     Sp.Richness2  6.215  0.195   0.195    0.195
## 82      Zoo.Area.ha2  ~      Attendance2 11.219  0.217   0.217    0.207
## 83      Zoo.Area.ha2  ~   Total.Animals2 10.477  0.217   0.217    0.217
## 84      Zoo.Area.ha2  ~     Sp.Richness2  7.729  0.175   0.175    0.175
## 85      Zoo.Area.ha2  ~ Mean.Raup.Crick2 25.670  0.769   0.769    0.767
## 90  Mam.Sp.Richness2  ~      Attendance2  3.041 -0.153  -0.153   -0.145
## 91  Mam.Sp.Richness2  ~   Total.Animals2 23.139 -0.519  -0.519   -0.519
## 92  Mam.Sp.Richness2  ~     Sp.Richness2 26.007 -0.881  -0.881   -0.881
## 93  Mam.Sp.Richness2  ~ Mean.Raup.Crick2 16.662 -0.293  -0.293   -0.293
##    sepc.nox
## 35   -0.042
## 41    0.078
## 42    0.070
## 46    0.062
## 48    0.105
## 51    0.147
## 52    0.071
## 53    0.066
## 54    0.354
## 55    0.205
## 58   -0.324
## 59   -0.142
## 60   -0.376
## 61   -0.375
## 66    0.760
## 67    0.392
## 68    0.405
## 69    0.316
## 74    0.405
## 75    0.234
## 76    0.195
## 82    0.207
## 83    0.217
## 84    0.175
## 85    0.767
## 90   -0.145
## 91   -0.519
## 92   -0.881
## 93   -0.293
```

```
# Adjust for the nested nature of the data (institutions within countries)
# Fit model and generate model summary
design <- svydesign(ids = ~Country, nest=TRUE, data=sem_attendance_data)
fit.adj26 <- lavaan.survey(lavaan.fit = mod.26.fit, survey.design = design)
summary(fit.adj26, rsq = TRUE)
```

```
## lavaan (0.5-23.1097) converged normally after  32 iterations
##
##   Number of observations                           458
##
##   Estimator                                         ML      Robust
##   Minimum Function Test Statistic               93.965      38.865
##   Degrees of freedom                                12          12
```

```
##   P-value (Chi-square)                          0.000       0.000
##   Scaling correction factor                                 2.418
##     for the Satorra-Bentler correction
##
## Parameter Estimates:
##
##   Information                                 Expected
##   Standard Errors                           Robust.sem
##
## Regressions:
##                      Estimate  Std.Err  z-value  P(>|z|)
##   Attendance2 ~
##     Total.Animals2      0.440    0.023   19.384    0.000
##     Men.Sp.BdyMss2      0.406    0.028   14.681    0.000
##     Mean.Rap.Crck2      0.156    0.028    5.592    0.000
##     X10km_Pop2          0.411    0.032   12.765    0.000
##     GDP.Millions2       0.139    0.025    5.469    0.000
##   Total.Animals2 ~
##     Zoo.Area.ha2        0.219    0.035    6.305    0.000
##     Sp.Richness2        0.805    0.048   16.640    0.000
##     GDP.Millions2      -0.129    0.072   -1.796    0.072
##   Sp.Richness2 ~
##     Mam.Sp.Rchnss2      0.837    0.064   13.051    0.000
##     Men.Sp.BdyMss2     -0.355    0.043   -8.283    0.000
##   Mean.Raup.Crick2 ~
##     Total.Animals2      0.477    0.151    3.168    0.002
##     Men.Sp.BdyMss2     -0.573    0.055  -10.334    0.000
##     Sp.Richness2       -0.322    0.220   -1.463    0.143
##     Zoo.Area.ha2        0.156    0.049    3.196    0.001
##
## Covariances:
##                        Estimate  Std.Err  z-value  P(>|z|)
##   Mean.Sp.BodyMass2 ~~
##     X10km_Pop2            0.126    0.067    1.895    0.058
##     GDP.Millions2        -0.082    0.046   -1.759    0.079
##     Zoo.Area.ha2          0.534    0.104    5.147    0.000
##     Mam.Sp.Rchnss2        0.303    0.081    3.730    0.000
##   X10km_Pop2 ~~
##     GDP.Millions2        -0.028    0.056   -0.505    0.613
##     Zoo.Area.ha2         -0.010    0.078   -0.124    0.901
##     Mam.Sp.Rchnss2        0.284    0.066    4.278    0.000
##   GDP.Millions2 ~~
##     Zoo.Area.ha2         -0.027    0.041   -0.673    0.501
##     Mam.Sp.Rchnss2       -0.060    0.064   -0.937    0.349
##   Zoo.Area.ha2 ~~
##     Mam.Sp.Rchnss2        0.381    0.064    5.985    0.000
##
## Intercepts:
##                    Estimate  Std.Err  z-value  P(>|z|)
##    .Attendance2      -0.000    0.029   -0.000    1.000
##    .Total.Animals2   -0.000    0.070   -0.000    1.000
##    .Sp.Richness2      0.000    0.070    0.000    1.000
##    .Mean.Rap.Crck2    0.000    0.089    0.000    1.000
##     Men.Sp.BdyMss2   -0.000    0.080   -0.000    1.000
```

```
##     X10km_Pop2        0.000    0.105    0.000    1.000
##     GDP.Millions2    -0.000    0.345   -0.000    1.000
##     Zoo.Area.ha2      0.000    0.064    0.000    1.000
##     Mam.Sp.Rchnss2   -0.000    0.079   -0.000    1.000
##
## Variances:
##                    Estimate  Std.Err  z-value  P(>|z|)
##    .Attendance2       0.281    0.026   10.698    0.000
##    .Total.Animals2    0.235    0.038    6.139    0.000
##    .Sp.Richness2      0.353    0.036    9.948    0.000
##    .Mean.Rap.Crck2    0.690    0.061   11.232    0.000
##     Men.Sp.BdyMss2    0.998    0.173    5.783    0.000
##     X10km_Pop2        0.998    0.174    5.745    0.000
##     GDP.Millions2     0.998    0.246    4.061    0.000
##     Zoo.Area.ha2      0.998    0.112    8.886    0.000
##     Mam.Sp.Rchnss2    0.998    0.115    8.669    0.000
##
## R-Square:
##                    Estimate
##     Attendance2       0.689
##     Total.Animals2    0.765
##     Sp.Richness2      0.646
##     Mean.Rap.Crck2    0.305
```

```
# Generate fit indices
fitMeasures(fit.adj26, c("agfi", "rmr", "srmr", "rmsea", "cfi", "nnfi", "tli"))
```

```
##  agfi   rmr  srmr rmsea   cfi  nnfi   tli
## 0.811 0.059 0.054 0.122 0.958 0.909 0.909
```

```
# Generate modification indices
mi26adj <- modindices(fit.adj26)
print(mi26adj[mi26adj$mi > 3.0,])
```

```
##                   lhs op             rhs     mi mi.scaled    epc sepc.lv
## 44        Attendance2 ~~     Sp.Richness2  5.500     2.275 -0.040  -0.040
## 50        Attendance2  ~     Zoo.Area.ha2  5.224     2.161  0.074   0.074
## 51        Attendance2  ~ Mam.Sp.Richness2  3.118     1.290  0.066   0.066
## 55     Total.Animals2  ~       X10km_Pop2  7.211     2.983  0.062   0.062
## 57       Sp.Richness2  ~      Attendance2  6.629     2.742  0.111   0.111
## 60       Sp.Richness2  ~       X10km_Pop2 25.438    10.522  0.146   0.146
## 61       Sp.Richness2  ~    GDP.Millions2  6.409     2.651  0.071   0.071
## 62       Sp.Richness2  ~     Zoo.Area.ha2  3.704     1.532  0.066   0.066
## 63   Mean.Raup.Crick2  ~      Attendance2 17.547     7.258  0.372   0.372
## 64   Mean.Raup.Crick2  ~       X10km_Pop2 25.373    10.495  0.204   0.204
## 67  Mean.Sp.BodyMass2  ~      Attendance2 10.188     4.214 -0.341  -0.341
## 68  Mean.Sp.BodyMass2  ~   Total.Animals2  3.638     1.505 -0.142  -0.142
## 69  Mean.Sp.BodyMass2  ~     Sp.Richness2  8.060     3.334 -0.376  -0.376
## 70  Mean.Sp.BodyMass2  ~ Mean.Raup.Crick2  6.328     2.617 -0.376  -0.376
## 75         X10km_Pop2  ~      Attendance2 47.999    19.853  0.799   0.799
## 76         X10km_Pop2  ~   Total.Animals2 36.854    15.243  0.392   0.392
## 77         X10km_Pop2  ~     Sp.Richness2 29.981    12.400  0.405   0.405
## 78         X10km_Pop2  ~ Mean.Raup.Crick2 38.581    15.958  0.317   0.317
## 83      GDP.Millions2  ~      Attendance2  5.308     2.196  0.426   0.426
## 84      GDP.Millions2  ~   Total.Animals2  5.877     2.431  0.234   0.234
## 85      GDP.Millions2  ~     Sp.Richness2  6.215     2.570  0.195   0.195
```

```
## 91       Zoo.Area.ha2  ~      Attendance2 11.219     4.640  0.217   0.217
## 92       Zoo.Area.ha2  ~   Total.Animals2 10.477     4.334  0.217   0.217
## 93       Zoo.Area.ha2  ~     Sp.Richness2  7.729     3.197  0.175   0.175
## 94       Zoo.Area.ha2  ~ Mean.Raup.Crick2 25.670    10.617  0.769   0.769
## 99   Mam.Sp.Richness2  ~      Attendance2  3.041     1.258 -0.153  -0.153
## 100  Mam.Sp.Richness2  ~   Total.Animals2 23.139     9.570 -0.519  -0.519
## 101  Mam.Sp.Richness2  ~     Sp.Richness2 26.007    10.757 -0.881  -0.881
## 102  Mam.Sp.Richness2  ~ Mean.Raup.Crick2 16.662     6.892 -0.293  -0.293
##     sepc.all sepc.nox
## 44    -0.042   -0.042
## 50     0.078    0.078
## 51     0.070    0.070
## 55     0.062    0.062
## 57     0.105    0.105
## 60     0.146    0.147
## 61     0.071    0.071
## 62     0.066    0.066
## 63     0.354    0.354
## 64     0.204    0.205
## 67    -0.324   -0.324
## 68    -0.142   -0.142
## 69    -0.376   -0.376
## 70    -0.375   -0.375
## 75     0.760    0.760
## 76     0.392    0.392
## 77     0.405    0.405
## 78     0.316    0.316
## 83     0.405    0.405
## 84     0.234    0.234
## 85     0.195    0.195
## 91     0.207    0.207
## 92     0.217    0.217
## 93     0.175    0.175
## 94     0.767    0.767
## 99    -0.145   -0.145
## 100   -0.519   -0.519
## 101   -0.881   -0.881
## 102   -0.293   -0.293
```

```r
# Test whether removal of pathway was justified
anova(fit.adj25, fit.adj26)
```

```
## Scaled Chi Square Difference Test (method = "satorra.bentler.2001")
##
##           Df    AIC    BIC  Chisq Chisq diff Df diff Pr(>Chisq)
## fit.adj25 11 9588.5 9765.9 88.137
## fit.adj26 12 9592.3 9765.6 93.965     4.7227       1    0.02977 *
## ---
## Signif. codes:  0 '***' 0.001 '**' 0.01 '*' 0.05 '.' 0.1 ' ' 1
```

Although the p-value is non-significant, the results from the anova suggest that the link **Attendance2** ~ **Zoo.Area.ha2** should be retained.

Model 27

Removal of non-significant relationship **Total.Animals2 ~ GDP.Millions2** and testing whether this is justified.

```
# Attendance SEM (Presence-Absence)

# Model 27
# Removal of Total.Animals2 ~ GDP.Millions2 from model 25, p = 0.072

mod.27 <- 'Attendance2 ~ Zoo.Area.ha2 + Total.Animals2
+ Mean.Sp.BodyMass2 + Mean.Raup.Crick2
+ X10km_Pop2 + GDP.Millions2

Total.Animals2 ~ Zoo.Area.ha2 + Sp.Richness2
Sp.Richness2 ~Mam.Sp.Richness2 + Mean.Sp.BodyMass2
Mean.Raup.Crick2 ~ Total.Animals2 + Mean.Sp.BodyMass2 + Sp.Richness2 + Zoo.Area.ha2'

# Fit model and generate model summary
mod.27.fit <- sem(mod.27, data = sem_attendance_data, fixed.x=FALSE)
summary(mod.27.fit, rsq = TRUE)
```

```
## lavaan (0.5-23.1097) converged normally after  28 iterations
##
##   Number of observations                           458
##
##   Estimator                                         ML
##   Minimum Function Test Statistic              119.135
##   Degrees of freedom                                12
##   P-value (Chi-square)                           0.000
##
## Parameter Estimates:
##
##   Information                                 Expected
##   Standard Errors                             Standard
##
## Regressions:
##                      Estimate  Std.Err  z-value  P(>|z|)
##   Attendance2 ~
##     Zoo.Area.ha2        0.082    0.032    2.550    0.011
##     Total.Animals2      0.409    0.028   14.698    0.000
##     Men.Sp.BdyMss2      0.353    0.034   10.353    0.000
##     Mean.Rap.Crck2      0.139    0.029    4.805    0.000
##     X10km_Pop2          0.431    0.025   16.951    0.000
##     GDP.Millions2       0.135    0.025    5.461    0.000
##   Total.Animals2 ~
##     Zoo.Area.ha2        0.223    0.024    9.448    0.000
##     Sp.Richness2        0.798    0.024   33.746    0.000
##   Sp.Richness2 ~
##     Mam.Sp.Rchnss2      0.837    0.029   28.676    0.000
##     Men.Sp.BdyMss2     -0.355    0.029  -12.172    0.000
##   Mean.Raup.Crick2 ~
##     Total.Animals2      0.477    0.077    6.165    0.000
##     Men.Sp.BdyMss2     -0.573    0.047  -12.190    0.000
##     Sp.Richness2       -0.322    0.074   -4.378    0.000
```

```
##     Zoo.Area.ha2        0.156    0.050    3.110    0.002
##
## Covariances:
##                       Estimate  Std.Err  z-value  P(>|z|)
##   Zoo.Area.ha2 ~~
##     Men.Sp.BdyMss2        0.534    0.053   10.096    0.000
##     X10km_Pop2           -0.010    0.047   -0.208    0.835
##     GDP.Millions2        -0.027    0.047   -0.588    0.557
##     Mam.Sp.Rchnss2        0.381    0.050    7.634    0.000
##   Mean.Sp.BodyMass2 ~~
##     X10km_Pop2            0.126    0.047    2.683    0.007
##     GDP.Millions2        -0.082    0.047   -1.748    0.080
##     Mam.Sp.Rchnss2        0.303    0.049    6.216    0.000
##   X10km_Pop2 ~~
##     GDP.Millions2        -0.028    0.047   -0.604    0.546
##     Mam.Sp.Rchnss2        0.284    0.048    5.852    0.000
##   GDP.Millions2 ~~
##     Mam.Sp.Rchnss2       -0.060    0.047   -1.277    0.202
##
## Variances:
##                   Estimate  Std.Err  z-value  P(>|z|)
##    .Attendance2       0.277    0.018   15.133    0.000
##    .Total.Animals2    0.251    0.017   15.133    0.000
##    .Sp.Richness2      0.353    0.023   15.133    0.000
##    .Mean.Rap.Crck2    0.690    0.046   15.133    0.000
##     Zoo.Area.ha2      0.998    0.066   15.133    0.000
##     Men.Sp.BdyMss2    0.998    0.066   15.133    0.000
##     X10km_Pop2        0.998    0.066   15.133    0.000
##     GDP.Millions2     0.998    0.066   15.133    0.000
##     Mam.Sp.Rchnss2    0.998    0.066   15.133    0.000
##
## R-Square:
##                   Estimate
##     Attendance2       0.697
##     Total.Animals2    0.744
##     Sp.Richness2      0.646
##     Mean.Rap.Crck2    0.308
```

```r
# Generate fit indices
fitMeasures(mod.27.fit, c("agfi", "rmr", "srmr", "rmsea", "cfi", "nnfi", "tli"))
```

```
##  agfi   rmr  srmr rmsea   cfi  nnfi   tli
## 0.803 0.059 0.059 0.140 0.945 0.881 0.881
```

```r
# Generate modification indices
mi27 <- modindices(mod.27.fit)
print(mi27[mi27$mi > 3.0,])
```

```
##                 lhs op              rhs     mi    epc sepc.lv sepc.all
## 35       Attendance2 ~~     Sp.Richness2  4.318 -0.036  -0.036   -0.038
## 45    Total.Animals2  ~       X10km_Pop2  8.162  0.068   0.068    0.069
## 46    Total.Animals2  ~    GDP.Millions2 29.891 -0.128  -0.128   -0.129
## 48      Sp.Richness2  ~      Attendance2  9.455  0.129   0.129    0.124
## 50      Sp.Richness2  ~ Mean.Raup.Crick2  3.017  0.067   0.067    0.067
## 51      Sp.Richness2  ~     Zoo.Area.ha2  3.704  0.066   0.066    0.066
## 52      Sp.Richness2  ~       X10km_Pop2 25.438  0.146   0.146    0.146
```

```
## 53      Sp.Richness2  ~    GDP.Millions2  6.409  0.071   0.071    0.071
## 54  Mean.Raup.Crick2  ~      Attendance2 22.571  0.413   0.413    0.396
## 55  Mean.Raup.Crick2  ~       X10km_Pop2 25.373  0.204   0.204    0.204
## 58      Zoo.Area.ha2  ~      Attendance2  5.955  0.332   0.332    0.317
## 59      Zoo.Area.ha2  ~   Total.Animals2  9.497  0.206   0.206    0.204
## 60      Zoo.Area.ha2  ~     Sp.Richness2  7.729  0.175   0.175    0.175
## 61      Zoo.Area.ha2  ~ Mean.Raup.Crick2 23.617  0.730   0.730    0.730
## 66 Mean.Sp.BodyMass2  ~      Attendance2  5.177 -0.352  -0.352   -0.336
## 67 Mean.Sp.BodyMass2  ~   Total.Animals2  3.780 -0.141  -0.141   -0.140
## 68 Mean.Sp.BodyMass2  ~     Sp.Richness2  8.060 -0.376  -0.376   -0.376
## 69 Mean.Sp.BodyMass2  ~ Mean.Raup.Crick2  6.405 -0.369  -0.369   -0.368
## 74        X10km_Pop2  ~      Attendance2 40.617  0.797   0.797    0.762
## 75        X10km_Pop2  ~   Total.Animals2 35.895  0.382   0.382    0.379
## 76        X10km_Pop2  ~     Sp.Richness2 29.981  0.405   0.405    0.405
## 77        X10km_Pop2  ~ Mean.Raup.Crick2 38.395  0.315   0.315    0.315
## 82     GDP.Millions2  ~      Attendance2  4.883 -0.310  -0.310   -0.296
## 83     GDP.Millions2  ~   Total.Animals2  5.190 -0.153  -0.153   -0.152
## 84     GDP.Millions2  ~     Sp.Richness2  6.215  0.195   0.195    0.195
## 91  Mam.Sp.Richness2  ~   Total.Animals2 19.443 -0.465  -0.465   -0.461
## 92  Mam.Sp.Richness2  ~     Sp.Richness2 26.007 -0.881  -0.881   -0.881
## 93  Mam.Sp.Richness2  ~ Mean.Raup.Crick2 15.818 -0.285  -0.285   -0.285
##    sepc.nox
## 35   -0.038
## 45    0.069
## 46   -0.129
## 48    0.124
## 50    0.067
## 51    0.066
## 52    0.147
## 53    0.071
## 54    0.396
## 55    0.204
## 58    0.317
## 59    0.204
## 60    0.175
## 61    0.730
## 66   -0.336
## 67   -0.140
## 68   -0.376
## 69   -0.368
## 74    0.762
## 75    0.379
## 76    0.405
## 77    0.315
## 82   -0.296
## 83   -0.152
## 84    0.195
## 91   -0.461
## 92   -0.881
## 93   -0.285
```

```
# Adjust for the nested nature of the data (institutions within countries)
# Fit model and generate model summary
design <- svydesign(ids = ~Country, nest=TRUE, data=sem_attendance_data)
```

```r
fit.adj27 <- lavaan.survey(lavaan.fit = mod.27.fit, survey.design = design)
summary(fit.adj27, rsq = TRUE)
```

```
## lavaan (0.5-23.1097) converged normally after  28 iterations
##
##   Number of observations                           458
##
##   Estimator                                         ML      Robust
##   Minimum Function Test Statistic              119.135      33.318
##   Degrees of freedom                                12          12
##   P-value (Chi-square)                           0.000       0.001
##   Scaling correction factor                                  3.576
##     for the Satorra-Bentler correction
##
## Parameter Estimates:
##
##   Information                                 Expected
##   Standard Errors                           Robust.sem
##
## Regressions:
##                      Estimate  Std.Err  z-value  P(>|z|)
##   Attendance2 ~
##     Zoo.Area.ha2        0.082    0.038    2.136    0.033
##     Total.Animals2      0.409    0.024   17.215    0.000
##     Men.Sp.BdyMss2      0.353    0.031   11.570    0.000
##     Mean.Rap.Crck2      0.139    0.028    4.946    0.000
##     X10km_Pop2          0.431    0.032   13.635    0.000
##     GDP.Millions2       0.135    0.026    5.243    0.000
##   Total.Animals2 ~
##     Zoo.Area.ha2        0.223    0.036    6.176    0.000
##     Sp.Richness2        0.798    0.046   17.286    0.000
##   Sp.Richness2 ~
##     Mam.Sp.Rchnss2      0.837    0.064   13.051    0.000
##     Men.Sp.BdyMss2     -0.355    0.043   -8.283    0.000
##   Mean.Raup.Crick2 ~
##     Total.Animals2      0.477    0.148    3.228    0.001
##     Men.Sp.BdyMss2     -0.573    0.056  -10.193    0.000
##     Sp.Richness2       -0.322    0.216   -1.493    0.136
##     Zoo.Area.ha2        0.156    0.049    3.165    0.002
##
## Covariances:
##                        Estimate  Std.Err  z-value  P(>|z|)
##   Zoo.Area.ha2 ~~
##     Men.Sp.BdyMss2        0.534    0.104    5.147    0.000
##     X10km_Pop2           -0.010    0.078   -0.124    0.901
##     GDP.Millions2        -0.027    0.041   -0.673    0.501
##     Mam.Sp.Rchnss2        0.381    0.064    5.985    0.000
##   Mean.Sp.BodyMass2 ~~
##     X10km_Pop2            0.126    0.067    1.895    0.058
##     GDP.Millions2        -0.082    0.046   -1.759    0.079
##     Mam.Sp.Rchnss2        0.303    0.081    3.730    0.000
##   X10km_Pop2 ~~
##     GDP.Millions2        -0.028    0.056   -0.505    0.613
##     Mam.Sp.Rchnss2        0.284    0.066    4.278    0.000
```

```
##   GDP.Millions2 ~~
##     Mam.Sp.Rchnss2       -0.060    0.064   -0.937    0.349
##
## Intercepts:
##                    Estimate  Std.Err  z-value  P(>|z|)
##    .Attendance2      -0.000    0.029   -0.000    1.000
##    .Total.Animals2   -0.000    0.104   -0.000    1.000
##    .Sp.Richness2      0.000    0.070    0.000    1.000
##    .Mean.Rap.Crck2    0.000    0.089    0.000    1.000
##     Zoo.Area.ha2      0.000    0.064    0.000    1.000
##     Men.Sp.BdyMss2   -0.000    0.080   -0.000    1.000
##     X10km_Pop2        0.000    0.105    0.000    1.000
##     GDP.Millions2    -0.000    0.345   -0.000    1.000
##     Mam.Sp.Rchnss2   -0.000    0.079   -0.000    1.000
##
## Variances:
##                    Estimate  Std.Err  z-value  P(>|z|)
##    .Attendance2       0.277    0.028   10.008    0.000
##    .Total.Animals2    0.251    0.044    5.729    0.000
##    .Sp.Richness2      0.353    0.036    9.948    0.000
##    .Mean.Rap.Crck2    0.690    0.061   11.233    0.000
##     Zoo.Area.ha2      0.998    0.112    8.886    0.000
##     Men.Sp.BdyMss2    0.998    0.173    5.783    0.000
##     X10km_Pop2        0.998    0.174    5.745    0.000
##     GDP.Millions2     0.998    0.246    4.061    0.000
##     Mam.Sp.Rchnss2    0.998    0.115    8.669    0.000
##
## R-Square:
##                    Estimate
##     Attendance2       0.697
##     Total.Animals2    0.744
##     Sp.Richness2      0.646
##     Mean.Rap.Crck2    0.308
```

```
# Generate fit indices
fitMeasures(fit.adj27, c("agfi", "rmr", "srmr", "rmsea", "cfi", "nnfi", "tli"))
```

```
##  agfi   rmr  srmr rmsea   cfi  nnfi   tli
## 0.763 0.059 0.054 0.140 0.945 0.881 0.881
```

```
# Generate modification indices
mi27adj <- modindices(fit.adj27)
print(mi27adj[mi27adj$mi > 3.0,])
```

```
##                  lhs op              rhs     mi mi.scaled    epc sepc.lv
## 44       Attendance2 ~~     Sp.Richness2  4.318     1.208 -0.036  -0.036
## 54     Total.Animals2  ~       X10km_Pop2  8.162     2.283  0.068   0.068
## 55     Total.Animals2  ~    GDP.Millions2 29.891     8.360 -0.128  -0.128
## 57       Sp.Richness2  ~      Attendance2  9.455     2.644  0.129   0.129
## 59       Sp.Richness2  ~ Mean.Raup.Crick2  3.017     0.844  0.067   0.067
## 60       Sp.Richness2  ~     Zoo.Area.ha2  3.704     1.036  0.066   0.066
## 61       Sp.Richness2  ~       X10km_Pop2 25.438     7.114  0.146   0.146
## 62       Sp.Richness2  ~    GDP.Millions2  6.409     1.792  0.071   0.071
## 63   Mean.Raup.Crick2  ~      Attendance2 22.571     6.313  0.413   0.413
## 64   Mean.Raup.Crick2  ~       X10km_Pop2 25.373     7.096  0.204   0.204
## 67       Zoo.Area.ha2  ~      Attendance2  5.955     1.665  0.332   0.332
```

```
## 68       Zoo.Area.ha2  ~   Total.Animals2  9.497     2.656  0.206   0.206
## 69       Zoo.Area.ha2  ~     Sp.Richness2  7.729     2.161  0.175   0.175
## 70       Zoo.Area.ha2  ~ Mean.Raup.Crick2 23.617     6.605  0.730   0.730
## 75  Mean.Sp.BodyMass2  ~      Attendance2  5.177     1.448 -0.352  -0.352
## 76  Mean.Sp.BodyMass2  ~   Total.Animals2  3.780     1.057 -0.141  -0.141
## 77  Mean.Sp.BodyMass2  ~     Sp.Richness2  8.060     2.254 -0.376  -0.376
## 78  Mean.Sp.BodyMass2  ~ Mean.Raup.Crick2  6.405     1.791 -0.369  -0.369
## 83         X10km_Pop2  ~      Attendance2 40.617    11.359  0.797   0.797
## 84         X10km_Pop2  ~   Total.Animals2 35.895    10.039  0.382   0.382
## 85         X10km_Pop2  ~     Sp.Richness2 29.981     8.385  0.405   0.405
## 86         X10km_Pop2  ~ Mean.Raup.Crick2 38.395    10.738  0.315   0.315
## 91      GDP.Millions2  ~      Attendance2  4.883     1.366 -0.310  -0.310
## 92      GDP.Millions2  ~   Total.Animals2  5.190     1.451 -0.153  -0.153
## 93      GDP.Millions2  ~     Sp.Richness2  6.215     1.738  0.195   0.195
## 100  Mam.Sp.Richness2  ~   Total.Animals2 19.443     5.438 -0.465  -0.465
## 101  Mam.Sp.Richness2  ~     Sp.Richness2 26.007     7.273 -0.881  -0.881
## 102  Mam.Sp.Richness2  ~ Mean.Raup.Crick2 15.818     4.424 -0.285  -0.285
##     sepc.all sepc.nox
## 44    -0.038   -0.038
## 54     0.069    0.069
## 55    -0.129   -0.129
## 57     0.124    0.124
## 59     0.067    0.067
## 60     0.066    0.066
## 61     0.146    0.147
## 62     0.071    0.071
## 63     0.396    0.396
## 64     0.204    0.204
## 67     0.317    0.317
## 68     0.204    0.204
## 69     0.175    0.175
## 70     0.730    0.730
## 75    -0.336   -0.336
## 76    -0.140   -0.140
## 77    -0.376   -0.376
## 78    -0.368   -0.368
## 83     0.762    0.762
## 84     0.379    0.379
## 85     0.405    0.405
## 86     0.315    0.315
## 91    -0.296   -0.296
## 92    -0.152   -0.152
## 93     0.195    0.195
## 100   -0.461   -0.461
## 101   -0.881   -0.881
## 102   -0.285   -0.285
```

```
# Test whether removal of pathway was justified
anova(fit.adj25, fit.adj27)
```

```
## Scaled Chi Square Difference Test (method = "satorra.bentler.2001")
##
##           Df    AIC    BIC   Chisq Chisq diff Df diff Pr(>Chisq)
## fit.adj25 11 9588.5 9765.9  88.137
## fit.adj27 12 9617.5 9790.8 119.135     2.0489       1     0.1523
```

Although the p-value anova appear non-significant, looking at the various absolute and incremental fit indices suggests that the link **Total.Animals2 ~ GDP.Millions2** should be retained, as it improves model-data fit.

Model 28

Removal of non-significant relationship **Mean.Raup.Crick2 ~ Sp.Richness2** and testing whether this is justified.

```
# Attendance SEM (Presence-Absence)

# Model 28
# Removal of Mean.Raup.Crick2 ~ Sp.Richness2 from model 25, p = 0.143

mod.28 <- 'Attendance2 ~ Zoo.Area.ha2 + Total.Animals2
+ Mean.Sp.BodyMass2 + Mean.Raup.Crick2
+ X10km_Pop2 + GDP.Millions2

Total.Animals2 ~ Zoo.Area.ha2 + Sp.Richness2 + GDP.Millions2
Sp.Richness2 ~Mam.Sp.Richness2 + Mean.Sp.BodyMass2
Mean.Raup.Crick2 ~ Total.Animals2 + Mean.Sp.BodyMass2 + Zoo.Area.ha2'

# Fit model and generate model summary
mod.28.fit <- sem(mod.28, data = sem_attendance_data, fixed.x=FALSE)
summary(mod.28.fit, rsq = TRUE)
```

```
## lavaan (0.5-23.1097) converged normally after  26 iterations
##
##   Number of observations                           458
##
##   Estimator                                         ML
##   Minimum Function Test Statistic              106.767
##   Degrees of freedom                                12
##   P-value (Chi-square)                           0.000
##
## Parameter Estimates:
##
##   Information                                 Expected
##   Standard Errors                             Standard
##
## Regressions:
##                    Estimate  Std.Err  z-value  P(>|z|)
##   Attendance2 ~
##     Zoo.Area.ha2      0.082    0.032    2.551    0.011
##     Total.Animals2    0.409    0.028   14.652    0.000
##     Men.Sp.BdyMss2    0.353    0.034   10.341    0.000
##     Mean.Rap.Crck2    0.139    0.029    4.806    0.000
##     X10km_Pop2        0.431    0.025   16.948    0.000
##     GDP.Millions2     0.135    0.025    5.386    0.000
##   Total.Animals2 ~
##     Zoo.Area.ha2      0.219    0.023    9.561    0.000
##     Sp.Richness2      0.805    0.023   35.215    0.000
##     GDP.Millions2    -0.129    0.023   -5.671    0.000
##   Sp.Richness2 ~
##     Mam.Sp.Rchnss2    0.837    0.029   28.676    0.000
```

```
##     Men.Sp.BdyMss2     -0.355    0.029  -12.172    0.000
##   Mean.Raup.Crick2 ~
##     Total.Animals2      0.195    0.043    4.588    0.000
##     Men.Sp.BdyMss2     -0.546    0.048  -11.489    0.000
##     Zoo.Area.ha2        0.188    0.050    3.747    0.000
##
## Covariances:
##                    Estimate  Std.Err  z-value  P(>|z|)
##   Zoo.Area.ha2 ~~
##     Men.Sp.BdyMss2        0.534    0.053   10.096    0.000
##     X10km_Pop2           -0.010    0.047   -0.208    0.835
##     GDP.Millions2        -0.027    0.047   -0.588    0.557
##     Mam.Sp.Rchnss2        0.381    0.050    7.634    0.000
##   Mean.Sp.BodyMass2 ~~
##     X10km_Pop2            0.126    0.047    2.683    0.007
##     GDP.Millions2        -0.082    0.047   -1.748    0.080
##     Mam.Sp.Rchnss2        0.303    0.049    6.216    0.000
##   X10km_Pop2 ~~
##     GDP.Millions2        -0.028    0.047   -0.604    0.546
##     Mam.Sp.Rchnss2        0.284    0.048    5.852    0.000
##   GDP.Millions2 ~~
##     Mam.Sp.Rchnss2       -0.060    0.047   -1.277    0.202
##
## Variances:
##                    Estimate  Std.Err  z-value  P(>|z|)
##    .Attendance2       0.277    0.018   15.133    0.000
##    .Total.Animals2    0.235    0.016   15.133    0.000
##    .Sp.Richness2      0.353    0.023   15.133    0.000
##    .Mean.Rap.Crck2    0.718    0.047   15.133    0.000
##     Zoo.Area.ha2      0.998    0.066   15.133    0.000
##     Men.Sp.BdyMss2    0.998    0.066   15.133    0.000
##     X10km_Pop2        0.998    0.066   15.133    0.000
##     GDP.Millions2     0.998    0.066   15.133    0.000
##     Mam.Sp.Rchnss2    0.998    0.066   15.133    0.000
##
## R-Square:
##                    Estimate
##     Attendance2       0.694
##     Total.Animals2    0.765
##     Sp.Richness2      0.646
##     Mean.Rap.Crck2    0.277
```

```r
# Generate fit indices
fitMeasures(mod.28.fit, c("agfi", "rmr", "srmr", "rmsea", "cfi", "nnfi", "tli"))
```

```
##  agfi   rmr  srmr rmsea   cfi  nnfi   tli
## 0.818 0.058 0.058 0.131 0.952 0.895 0.895
```

```r
# Generate modification indices
mi28 <- modindices(mod.28.fit)
print(mi27[mi28$mi > 3.0,])
```

```
##                 lhs op            rhs    mi    epc sepc.lv sepc.all
## 35      Attendance2 ~~   Sp.Richness2 4.318 -0.036  -0.036   -0.038
## 37   Total.Animals2 ~~   Sp.Richness2 0.339  0.010   0.010    0.010
## 42   Total.Animals2  ~    Attendance2 0.732  0.029   0.029    0.028
```

```
## 43    Total.Animals2  ~ Mean.Raup.Crick2  0.000  0.001   0.001    0.001
## 45    Total.Animals2  ~       X10km_Pop2  8.162  0.068   0.068    0.069
## 47    Total.Animals2  ~ Mam.Sp.Richness2  0.292 -0.020  -0.020   -0.021
## 50      Sp.Richness2  ~ Mean.Raup.Crick2  3.017  0.067   0.067    0.067
## 51      Sp.Richness2  ~      Zoo.Area.ha2  3.704  0.066   0.066    0.066
## 52      Sp.Richness2  ~       X10km_Pop2 25.438  0.146   0.146    0.146
## 53      Sp.Richness2  ~    GDP.Millions2  6.409  0.071   0.071    0.071
## 54  Mean.Raup.Crick2  ~      Attendance2 22.571  0.413   0.413    0.396
## 55  Mean.Raup.Crick2  ~       X10km_Pop2 25.373  0.204   0.204    0.204
## 57  Mean.Raup.Crick2  ~ Mam.Sp.Richness2  1.210 -0.076  -0.076   -0.076
## 58      Zoo.Area.ha2  ~      Attendance2  5.955  0.332   0.332    0.317
## 59      Zoo.Area.ha2  ~   Total.Animals2  9.497  0.206   0.206    0.204
## 60      Zoo.Area.ha2  ~     Sp.Richness2  7.729  0.175   0.175    0.175
## 61      Zoo.Area.ha2  ~ Mean.Raup.Crick2 23.617  0.730   0.730    0.730
## 66 Mean.Sp.BodyMass2  ~      Attendance2  5.177 -0.352  -0.352   -0.336
## 67 Mean.Sp.BodyMass2  ~   Total.Animals2  3.780 -0.141  -0.141   -0.140
## 68 Mean.Sp.BodyMass2  ~     Sp.Richness2  8.060 -0.376  -0.376   -0.376
## 69 Mean.Sp.BodyMass2  ~ Mean.Raup.Crick2  6.405 -0.369  -0.369   -0.368
## 74        X10km_Pop2  ~      Attendance2 40.617  0.797   0.797    0.762
## 75        X10km_Pop2  ~   Total.Animals2 35.895  0.382   0.382    0.379
## 76        X10km_Pop2  ~     Sp.Richness2 29.981  0.405   0.405    0.405
## 77        X10km_Pop2  ~ Mean.Raup.Crick2 38.395  0.315   0.315    0.315
## 82     GDP.Millions2  ~      Attendance2  4.883 -0.310  -0.310   -0.296
## 83     GDP.Millions2  ~   Total.Animals2  5.190 -0.153  -0.153   -0.152
## 84     GDP.Millions2  ~     Sp.Richness2  6.215  0.195   0.195    0.195
## 91  Mam.Sp.Richness2  ~   Total.Animals2 19.443 -0.465  -0.465   -0.461
## 92  Mam.Sp.Richness2  ~     Sp.Richness2 26.007 -0.881  -0.881   -0.881
## 93  Mam.Sp.Richness2  ~ Mean.Raup.Crick2 15.818 -0.285  -0.285   -0.285
##    sepc.nox
## 35   -0.038
## 37    0.010
## 42    0.028
## 43    0.001
## 45    0.069
## 47   -0.021
## 50    0.067
## 51    0.066
## 52    0.147
## 53    0.071
## 54    0.396
## 55    0.204
## 57   -0.076
## 58    0.317
## 59    0.204
## 60    0.175
## 61    0.730
## 66   -0.336
## 67   -0.140
## 68   -0.376
## 69   -0.368
## 74    0.762
## 75    0.379
## 76    0.405
## 77    0.315
```

```
## 82   -0.296
## 83   -0.152
## 84    0.195
## 91   -0.461
## 92   -0.881
## 93   -0.285
```

```r
# Adjust for the nested nature of the data (institutions within countries)
# Fit model and generate model summary
design <- svydesign(ids = ~Country, nest=TRUE, data=sem_attendance_data)
fit.adj28 <- lavaan.survey(lavaan.fit = mod.28.fit, survey.design = design)
summary(fit.adj28, rsq = TRUE)
```

```
## lavaan (0.5-23.1097) converged normally after  26 iterations
##
##   Number of observations                           458
##
##   Estimator                                         ML      Robust
##   Minimum Function Test Statistic              106.767      30.814
##   Degrees of freedom                                12          12
##   P-value (Chi-square)                           0.000       0.002
##   Scaling correction factor                                  3.465
##     for the Satorra-Bentler correction
##
## Parameter Estimates:
##
##   Information                                 Expected
##   Standard Errors                           Robust.sem
##
## Regressions:
##                    Estimate  Std.Err  z-value  P(>|z|)
##   Attendance2 ~
##     Zoo.Area.ha2      0.082    0.039    2.126    0.034
##     Total.Animals2    0.409    0.025   16.334    0.000
##     Men.Sp.BdyMss2    0.353    0.031   11.383    0.000
##     Mean.Rap.Crck2    0.139    0.028    4.965    0.000
##     X10km_Pop2        0.431    0.032   13.565    0.000
##     GDP.Millions2     0.135    0.027    5.010    0.000
##   Total.Animals2 ~
##     Zoo.Area.ha2      0.219    0.035    6.305    0.000
##     Sp.Richness2      0.805    0.048   16.640    0.000
##     GDP.Millions2    -0.129    0.072   -1.796    0.072
##   Sp.Richness2 ~
##     Mam.Sp.Rchnss2    0.837    0.064   13.051    0.000
##     Men.Sp.BdyMss2   -0.355    0.043   -8.283    0.000
##   Mean.Raup.Crick2 ~
##     Total.Animals2    0.195    0.068    2.859    0.004
##     Men.Sp.BdyMss2   -0.546    0.073   -7.501    0.000
##     Zoo.Area.ha2      0.188    0.062    3.028    0.002
##
## Covariances:
##                      Estimate  Std.Err  z-value  P(>|z|)
##   Zoo.Area.ha2 ~~
##     Men.Sp.BdyMss2      0.534    0.104    5.147    0.000
##     X10km_Pop2         -0.010    0.078   -0.124    0.901
```

```
##     GDP.Millions2        -0.027    0.041   -0.673    0.501
##     Mam.Sp.Rchnss2        0.381    0.064    5.985    0.000
##   Mean.Sp.BodyMass2 ~~
##     X10km_Pop2            0.126    0.067    1.895    0.058
##     GDP.Millions2        -0.082    0.046   -1.759    0.079
##     Mam.Sp.Rchnss2        0.303    0.081    3.730    0.000
##   X10km_Pop2 ~~
##     GDP.Millions2        -0.028    0.056   -0.505    0.613
##     Mam.Sp.Rchnss2        0.284    0.066    4.278    0.000
##   GDP.Millions2 ~~
##     Mam.Sp.Rchnss2       -0.060    0.064   -0.937    0.349
##
## Intercepts:
##                    Estimate  Std.Err  z-value  P(>|z|)
##    .Attendance2      -0.000    0.029   -0.000    1.000
##    .Total.Animals2   -0.000    0.070   -0.000    1.000
##    .Sp.Richness2      0.000    0.070    0.000    1.000
##    .Mean.Rap.Crck2   -0.000    0.092   -0.000    1.000
##     Zoo.Area.ha2      0.000    0.064    0.000    1.000
##     Men.Sp.BdyMss2   -0.000    0.080   -0.000    1.000
##     X10km_Pop2        0.000    0.105    0.000    1.000
##     GDP.Millions2    -0.000    0.345   -0.000    1.000
##     Mam.Sp.Rchnss2   -0.000    0.079   -0.000    1.000
##
## Variances:
##                    Estimate  Std.Err  z-value  P(>|z|)
##    .Attendance2       0.277    0.028   10.008    0.000
##    .Total.Animals2    0.235    0.038    6.139    0.000
##    .Sp.Richness2      0.353    0.036    9.948    0.000
##    .Mean.Rap.Crck2    0.718    0.099    7.260    0.000
##     Zoo.Area.ha2      0.998    0.112    8.886    0.000
##     Men.Sp.BdyMss2    0.998    0.173    5.783    0.000
##     X10km_Pop2        0.998    0.174    5.745    0.000
##     GDP.Millions2     0.998    0.246    4.061    0.000
##     Mam.Sp.Rchnss2    0.998    0.115    8.669    0.000
##
## R-Square:
##                    Estimate
##     Attendance2       0.694
##     Total.Animals2    0.765
##     Sp.Richness2      0.646
##     Mean.Rap.Crck2    0.277
```

```
# Generate fit indices
fitMeasures(fit.adj28, c("agfi", "rmr", "srmr", "rmsea", "cfi", "nnfi", "tli"))
```

```
##  agfi   rmr  srmr rmsea   cfi  nnfi   tli
## 0.782 0.058 0.053 0.131 0.952 0.895 0.895
```

```
# Generate modification indices
mi28adj <- modindices(fit.adj28)
print(mi28adj[mi28adj$mi > 3.0,])
```

```
##                lhs op              rhs     mi mi.scaled    epc sepc.lv
## 44        Attendance2 ~~     Sp.Richness2  4.332     1.250 -0.036  -0.036
## 47     Total.Animals2 ~~ Mean.Raup.Crick2 17.644     5.092  0.094   0.094
```

```
## 51     Total.Animals2  ~      Attendance2  5.284     1.525  0.075   0.075
## 52     Total.Animals2  ~ Mean.Raup.Crick2 14.283     4.122  0.100   0.100
## 54     Total.Animals2  ~       X10km_Pop2  7.211     2.081  0.062   0.062
## 56       Sp.Richness2  ~      Attendance2  7.942     2.292  0.121   0.121
## 59       Sp.Richness2  ~     Zoo.Area.ha2  3.704     1.069  0.066   0.066
## 60       Sp.Richness2  ~       X10km_Pop2 25.438     7.342  0.146   0.146
## 61       Sp.Richness2  ~    GDP.Millions2  6.409     1.850  0.071   0.071
## 62   Mean.Raup.Crick2  ~      Attendance2 16.510     4.765  0.366   0.366
## 63   Mean.Raup.Crick2  ~     Sp.Richness2 18.494     5.338 -0.326  -0.326
## 64   Mean.Raup.Crick2  ~       X10km_Pop2 19.860     5.732  0.183   0.183
## 66   Mean.Raup.Crick2  ~ Mam.Sp.Richness2 11.875     3.427 -0.200  -0.200
## 67       Zoo.Area.ha2  ~      Attendance2  8.061     2.326  0.386   0.386
## 68       Zoo.Area.ha2  ~   Total.Animals2 10.477     3.024  0.217   0.217
## 69       Zoo.Area.ha2  ~     Sp.Richness2  7.729     2.231  0.175   0.175
## 70       Zoo.Area.ha2  ~ Mean.Raup.Crick2 47.679    13.761  1.017   1.017
## 75  Mean.Sp.BodyMass2  ~      Attendance2  5.332     1.539 -0.389  -0.389
## 76  Mean.Sp.BodyMass2  ~   Total.Animals2  3.638     1.050 -0.142  -0.142
## 77  Mean.Sp.BodyMass2  ~     Sp.Richness2  8.060     2.326 -0.376  -0.376
## 78  Mean.Sp.BodyMass2  ~ Mean.Raup.Crick2  8.979     2.592 -0.671  -0.671
## 83         X10km_Pop2  ~      Attendance2 43.285    12.492  0.849   0.849
## 84         X10km_Pop2  ~   Total.Animals2 36.854    10.636  0.392   0.392
## 85         X10km_Pop2  ~     Sp.Richness2 29.981     8.653  0.405   0.405
## 86         X10km_Pop2  ~ Mean.Raup.Crick2 39.030    11.264  0.321   0.321
## 91      GDP.Millions2  ~      Attendance2  3.666     1.058  0.370   0.370
## 92      GDP.Millions2  ~   Total.Animals2  5.877     1.696  0.234   0.234
## 93      GDP.Millions2  ~     Sp.Richness2  6.215     1.794  0.195   0.195
## 100  Mam.Sp.Richness2  ~   Total.Animals2 23.138     6.678 -0.519  -0.519
## 101  Mam.Sp.Richness2  ~     Sp.Richness2 26.006     7.506 -0.881  -0.881
## 102  Mam.Sp.Richness2  ~ Mean.Raup.Crick2 33.111     9.556 -0.355  -0.355
##     sepc.all sepc.nox
## 44    -0.038   -0.038
## 47     0.095    0.095
## 51     0.072    0.072
## 52     0.100    0.100
## 54     0.062    0.062
## 56     0.115    0.115
## 59     0.066    0.066
## 60     0.146    0.147
## 61     0.071    0.071
## 62     0.350    0.350
## 63    -0.327   -0.327
## 64     0.183    0.183
## 66    -0.201   -0.201
## 67     0.368    0.368
## 68     0.217    0.217
## 69     0.175    0.175
## 70     1.015    1.015
## 75    -0.371   -0.371
## 76    -0.142   -0.142
## 77    -0.376   -0.376
## 78    -0.669   -0.669
## 83     0.810    0.810
## 84     0.392    0.392
## 85     0.405    0.405
```

```
## 86     0.320    0.320
## 91     0.353    0.353
## 92     0.234    0.234
## 93     0.195    0.195
## 100   -0.519   -0.519
## 101   -0.881   -0.881
## 102   -0.354   -0.354
```

```
# Test whether removal of pathway was justified
anova(fit.adj25, fit.adj28)
```

```
## Scaled Chi Square Difference Test (method = "satorra.bentler.2001")
##
##           Df    AIC    BIC   Chisq Chisq diff Df diff Pr(>Chisq)
## fit.adj25 11 9588.5 9765.9  88.137
## fit.adj28 12 9605.1 9778.4 106.767       1.35       1     0.2453
```

Although the p-value anova appear non-significant, looking at the various absolute and incremental fit indices suggests that the link **Mean.Raup.Crick2 ~ Sp.Richness2** should be retained, as it improves model-data fit.

Final Attendance Model (Species Presence - Absence)

Contents
Based on the results, we believe model 25 is an accurate representation of the system. Therefore we present the nested version of model 25 as our final Attendance Model based on species presence-absence data.

```
# Chosen Attendance SEM (Presence-Absence)
# Model 25

mod.25.final <- 'Attendance2 ~ Zoo.Area.ha2 + Total.Animals2
+ Mean.Sp.BodyMass2 + Mean.Raup.Crick2
+ X10km_Pop2 + GDP.Millions2

Total.Animals2 ~ Zoo.Area.ha2 + Sp.Richness2 + GDP.Millions2
Sp.Richness2 ~ Mam.Sp.Richness2 + Mean.Sp.BodyMass2
Mean.Raup.Crick2 ~ Total.Animals2 + Mean.Sp.BodyMass2 + Sp.Richness2 + Zoo.Area.ha2'

# Fit model and generate model summary
mod.25.final.fit <- sem(mod.25.final, data = sem_attendance_data, fixed.x=FALSE)
summary(mod.25.final.fit, rsq = TRUE)
```

```
## lavaan (0.5-23.1097) converged normally after  32 iterations
##
##   Number of observations                           458
##
##   Estimator                                         ML
##   Minimum Function Test Statistic               88.137
##   Degrees of freedom                                11
##   P-value (Chi-square)                           0.000
##
## Parameter Estimates:
##
##   Information                                 Expected
```

```
##   Standard Errors                              Standard
##
## Regressions:
##                      Estimate  Std.Err  z-value  P(>|z|)
##   Attendance2 ~
##     Zoo.Area.ha2        0.082    0.032    2.550    0.011
##     Total.Animals2      0.409    0.028   14.658    0.000
##     Men.Sp.BdyMss2      0.353    0.034   10.339    0.000
##     Mean.Rap.Crck2      0.139    0.029    4.799    0.000
##     X10km_Pop2          0.431    0.025   16.943    0.000
##     GDP.Millions2       0.135    0.025    5.382    0.000
##   Total.Animals2 ~
##     Zoo.Area.ha2        0.219    0.023    9.561    0.000
##     Sp.Richness2        0.805    0.023   35.215    0.000
##     GDP.Millions2      -0.129    0.023   -5.671    0.000
##   Sp.Richness2 ~
##     Mam.Sp.Rchnss2      0.837    0.029   28.676    0.000
##     Men.Sp.BdyMss2     -0.355    0.029  -12.172    0.000
##   Mean.Raup.Crick2 ~
##     Total.Animals2      0.477    0.077    6.164    0.000
##     Men.Sp.BdyMss2     -0.573    0.047  -12.187    0.000
##     Sp.Richness2       -0.322    0.074   -4.333    0.000
##     Zoo.Area.ha2        0.156    0.050    3.124    0.002
##
## Covariances:
##                        Estimate  Std.Err  z-value  P(>|z|)
##   Zoo.Area.ha2 ~~
##     Men.Sp.BdyMss2        0.534    0.053   10.096    0.000
##     X10km_Pop2           -0.010    0.047   -0.208    0.835
##     GDP.Millions2        -0.027    0.047   -0.588    0.557
##     Mam.Sp.Rchnss2        0.381    0.050    7.634    0.000
##   Mean.Sp.BodyMass2 ~~
##     X10km_Pop2            0.126    0.047    2.683    0.007
##     GDP.Millions2        -0.082    0.047   -1.748    0.080
##     Mam.Sp.Rchnss2        0.303    0.049    6.216    0.000
##   X10km_Pop2 ~~
##     GDP.Millions2        -0.028    0.047   -0.604    0.546
##     Mam.Sp.Rchnss2        0.284    0.048    5.852    0.000
##   GDP.Millions2 ~~
##     Mam.Sp.Rchnss2       -0.060    0.047   -1.277    0.202
##
## Variances:
##                    Estimate  Std.Err  z-value  P(>|z|)
##    .Attendance2       0.277    0.018   15.133    0.000
##    .Total.Animals2    0.235    0.016   15.133    0.000
##    .Sp.Richness2      0.353    0.023   15.133    0.000
##    .Mean.Rap.Crck2    0.690    0.046   15.133    0.000
##     Zoo.Area.ha2      0.998    0.066   15.133    0.000
##     Men.Sp.BdyMss2    0.998    0.066   15.133    0.000
##     X10km_Pop2        0.998    0.066   15.133    0.000
##     GDP.Millions2     0.998    0.066   15.133    0.000
##     Mam.Sp.Rchnss2    0.998    0.066   15.133    0.000
##
## R-Square:
```

```
##                     Estimate
##     Attendance2        0.693
##     Total.Animals2     0.765
##     Sp.Richness2       0.646
##     Mean.Rap.Crck2     0.305
```

```
# Adjust for the nested nature of the data (institutions within countries)
# Fit model and generate model summary
design <- svydesign(ids = ~Country, nest=TRUE, data=sem_attendance_data)
fit.adj25.final <- lavaan.survey(lavaan.fit = mod.25.final.fit, survey.design = design)
summary(fit.adj25.final, rsq = TRUE)
```

```
## lavaan (0.5-23.1097) converged normally after  32 iterations
##
##   Number of observations                           458
##
##   Estimator                                         ML      Robust
##   Minimum Function Test Statistic               88.137      34.901
##   Degrees of freedom                                11          11
##   P-value (Chi-square)                           0.000       0.000
##   Scaling correction factor                                  2.525
##     for the Satorra-Bentler correction
##
## Parameter Estimates:
##
##   Information                                 Expected
##   Standard Errors                           Robust.sem
##
## Regressions:
##                      Estimate  Std.Err  z-value  P(>|z|)
##   Attendance2 ~
##     Zoo.Area.ha2        0.082    0.039    2.121    0.034
##     Total.Animals2      0.409    0.025   16.423    0.000
##     Men.Sp.BdyMss2      0.353    0.031   11.362    0.000
##     Mean.Rap.Crck2      0.139    0.028    5.014    0.000
##     X10km_Pop2          0.431    0.032   13.549    0.000
##     GDP.Millions2       0.135    0.027    5.067    0.000
##   Total.Animals2 ~
##     Zoo.Area.ha2        0.219    0.035    6.305    0.000
##     Sp.Richness2        0.805    0.048   16.640    0.000
##     GDP.Millions2      -0.129    0.072   -1.796    0.072
##   Sp.Richness2 ~
##     Mam.Sp.Rchnss2      0.837    0.064   13.051    0.000
##     Men.Sp.BdyMss2     -0.355    0.043   -8.283    0.000
##   Mean.Raup.Crick2 ~
##     Total.Animals2      0.477    0.151    3.168    0.002
##     Men.Sp.BdyMss2     -0.573    0.055  -10.334    0.000
##     Sp.Richness2       -0.322    0.220   -1.463    0.143
##     Zoo.Area.ha2        0.156    0.049    3.196    0.001
##
## Covariances:
##                        Estimate  Std.Err  z-value  P(>|z|)
##   Zoo.Area.ha2 ~~
##     Men.Sp.BdyMss2        0.534    0.104    5.147    0.000
##     X10km_Pop2           -0.010    0.078   -0.124    0.901
```

```
##     GDP.Millions2        -0.027    0.041   -0.673    0.501
##     Mam.Sp.Rchnss2        0.381    0.064    5.985    0.000
##   Mean.Sp.BodyMass2 ~~
##     X10km_Pop2            0.126    0.067    1.895    0.058
##     GDP.Millions2        -0.082    0.046   -1.759    0.079
##     Mam.Sp.Rchnss2        0.303    0.081    3.730    0.000
##   X10km_Pop2 ~~
##     GDP.Millions2        -0.028    0.056   -0.505    0.613
##     Mam.Sp.Rchnss2        0.284    0.066    4.278    0.000
##   GDP.Millions2 ~~
##     Mam.Sp.Rchnss2       -0.060    0.064   -0.937    0.349
##
## Intercepts:
##                    Estimate  Std.Err  z-value  P(>|z|)
##    .Attendance2      -0.000    0.029   -0.000    1.000
##    .Total.Animals2   -0.000    0.070   -0.000    1.000
##    .Sp.Richness2      0.000    0.070    0.000    1.000
##    .Mean.Rap.Crck2    0.000    0.089    0.000    1.000
##     Zoo.Area.ha2      0.000    0.064    0.000    1.000
##     Men.Sp.BdyMss2   -0.000    0.080   -0.000    1.000
##     X10km_Pop2        0.000    0.105    0.000    1.000
##     GDP.Millions2    -0.000    0.345   -0.000    1.000
##     Mam.Sp.Rchnss2   -0.000    0.079   -0.000    1.000
##
## Variances:
##                    Estimate  Std.Err  z-value  P(>|z|)
##    .Attendance2       0.277    0.028   10.008    0.000
##    .Total.Animals2    0.235    0.038    6.139    0.000
##    .Sp.Richness2      0.353    0.036    9.948    0.000
##    .Mean.Rap.Crck2    0.690    0.061   11.233    0.000
##     Zoo.Area.ha2      0.998    0.112    8.886    0.000
##     Men.Sp.BdyMss2    0.998    0.173    5.783    0.000
##     X10km_Pop2        0.998    0.174    5.745    0.000
##     GDP.Millions2     0.998    0.246    4.061    0.000
##     Mam.Sp.Rchnss2    0.998    0.115    8.669    0.000
##
## R-Square:
##                    Estimate
##     Attendance2       0.693
##     Total.Animals2    0.765
##     Sp.Richness2      0.646
##     Mean.Rap.Crck2    0.305
```

```
# Generate fit indices
fitMeasures(fit.adj25.final, c("agfi", "rmr", "srmr", "rmsea", "cfi", "nnfi", "tli"))
```

```
##  agfi   rmr  srmr rmsea   cfi  nnfi   tli
## 0.803 0.058 0.053 0.124 0.961 0.907 0.907
```

Tests of Mediation

It is clear from Model 25 that several pathways are mediated in the system. It is necessary to evaluate whether this mediation is appropriate by comparing models with complete, partial and no mediation. Here we show tests of mediation for four of these relationships.

Attendance2 ~ Zoo.Area.ha2

Attendance2 ~ Zoo.Area.ha2 is mediated by: **Total.Animals2 ~ Zoo.Area.ha2**. Below we compare models with complete (full) mediation, partial mediation (i.e. model 25) and no mediation. Results from this output suggest that partial mediation (model 25) is the superior model.

```
# Complete Mediation Model

FULL1 <- 'Attendance2 ~ Total.Animals2
+ Mean.Sp.BodyMass2 + Mean.Raup.Crick2 + X10km_Pop2 + GDP.Millions2

Total.Animals2 ~ Zoo.Area.ha2 + Sp.Richness2 + GDP.Millions2
Sp.Richness2 ~ Mam.Sp.Richness2 + Mean.Sp.BodyMass2
Mean.Raup.Crick2 ~ Sp.Richness2 + Total.Animals2 + Mean.Sp.BodyMass2'

FULL1.fit <- sem(FULL1, data = sem_attendance_data, fixed.x=FALSE)
design <- svydesign(ids = ~Country, nest=TRUE, data=sem_attendance_data)
FULL1.fitadj <- lavaan.survey(lavaan.fit = FULL1.fit, survey.design = design)

# Partial Mediation Model (MOdel 25)

PARTIAL1.MOD25 <- 'Attendance2 ~ Zoo.Area.ha2 + Total.Animals2
+ Mean.Sp.BodyMass2 + Mean.Raup.Crick2 + X10km_Pop2 + GDP.Millions2

Total.Animals2 ~ Zoo.Area.ha2 + Sp.Richness2 + GDP.Millions2
Sp.Richness2 ~ Mam.Sp.Richness2 + Mean.Sp.BodyMass2
Mean.Raup.Crick2 ~ Sp.Richness2 + Total.Animals2 + Mean.Sp.BodyMass2'

PARTIAL1.MOD25.fit <- sem(PARTIAL1.MOD25, data = sem_attendance_data, fixed.x=FALSE)
design <- svydesign(ids = ~Country, nest=TRUE, data=sem_attendance_data)
PARTIAL1.MOD25.fitadj <- lavaan.survey(lavaan.fit = PARTIAL1.MOD25.fit, survey.design = design)

# No Mediation Model

NONE1 <- 'Attendance2 ~ Zoo.Area.ha2 + Total.Animals2
+ Mean.Sp.BodyMass2 + Mean.Raup.Crick2 + X10km_Pop2 + GDP.Millions2

Total.Animals2 ~ Sp.Richness2 + GDP.Millions2
Sp.Richness2 ~ Mam.Sp.Richness2 + Mean.Sp.BodyMass2
Mean.Raup.Crick2 ~ Sp.Richness2 + Total.Animals2 + Mean.Sp.BodyMass2'

NONE1.fit <- sem(NONE1, data = sem_attendance_data, fixed.x=FALSE)
design <- svydesign(ids = ~Country, nest=TRUE, data=sem_attendance_data)
NONE1.fitadj <- lavaan.survey(lavaan.fit = NONE1.fit, survey.design = design)

aictab.lavaan(list(FULL1.fit, PARTIAL1.MOD25.fit, NONE1.fit,
                   FULL1.fitadj, PARTIAL1.MOD25.fitadj, NONE1.fitadj),
              c("full mediation", "partial mediation", "no mediation", "full mediation (nested)", "parti
```

```
##
## Model selection based on AICc:
##
##                              K    AICc Delta_AICc AICcWt Cum.Wt       LL
## partial mediation           33 9578.75       0.00   0.86   0.86 -4755.90
## full mediation              32 9582.45       3.70   0.14   1.00 -4758.81
```

```
## partial mediation (nested) 42 9596.75      18.00   0.00   1.00 -4755.90
## full mediation (nested)    41 9600.45      21.70   0.00   1.00 -4758.81
## no mediation               32 9658.90      80.15   0.00   1.00 -4797.04
## no mediation (nested)      41 9676.90      98.15   0.00   1.00 -4797.04
```

Attendance2 ~ Total.Animals2

Attendance2 ~ Total.Animals2 is mediated by **Mean.Raup.Crick2 ~ Total.Animals2**. Below we compare models with complete (full) mediation, partial mediation (i.e. model 25) and no mediation. Results from this output suggest that partial mediation (model 25) is the superior model.

```
# Complete Mediation Model

FULL2 <- 'Attendance2 ~ Zoo.Area.ha2
+ Mean.Sp.BodyMass2 + Mean.Raup.Crick2 + X10km_Pop2 + GDP.Millions2

Total.Animals2 ~ Zoo.Area.ha2 + Sp.Richness2 + GDP.Millions2
Sp.Richness2 ~ Mam.Sp.Richness2 + Mean.Sp.BodyMass2
Mean.Raup.Crick2 ~ Sp.Richness2 + Total.Animals2 + Mean.Sp.BodyMass2'

FULL2.fit <- sem(FULL2, data = sem_attendance_data, fixed.x=FALSE)
design <- svydesign(ids = ~Country, nest=TRUE, data=sem_attendance_data)
FULL2.fitadj <- lavaan.survey(lavaan.fit = FULL2.fit, survey.design = design)

# Partial Mediation Model (Model 25)

PARTIAL2.MOD25 <- 'Attendance2 ~ Zoo.Area.ha2 + Total.Animals2
+ Mean.Sp.BodyMass2 + Mean.Raup.Crick2 + X10km_Pop2 + GDP.Millions2

Total.Animals2 ~ Zoo.Area.ha2 + Sp.Richness2 + GDP.Millions2
Sp.Richness2 ~ Mam.Sp.Richness2 + Mean.Sp.BodyMass2
Mean.Raup.Crick2 ~ Sp.Richness2 + Total.Animals2 + Mean.Sp.BodyMass2'

PARTIAL2.MOD25.fit <- sem(PARTIAL2.MOD25, data = sem_attendance_data, fixed.x=FALSE)
design <- svydesign(ids = ~Country, nest=TRUE, data=sem_attendance_data)
PARTIAL2.MOD25.fitadj <- lavaan.survey(lavaan.fit = PARTIAL2.MOD25.fit, survey.design = design)

# No Mediation Model

NONE2 <- 'Attendance2 ~ Zoo.Area.ha2 + Total.Animals2
+ Mean.Sp.BodyMass2 + Mean.Raup.Crick2 + X10km_Pop2 + GDP.Millions2

Total.Animals2 ~ Zoo.Area.ha2 + Sp.Richness2 + GDP.Millions2
Sp.Richness2 ~ Mam.Sp.Richness2 + Mean.Sp.BodyMass2
Mean.Raup.Crick2 ~ Sp.Richness2 + Mean.Sp.BodyMass2'

NONE2.fit <- sem(NONE2, data = sem_attendance_data, fixed.x=FALSE)
design <- svydesign(ids = ~Country, nest=TRUE, data=sem_attendance_data)
NONE2.fitadj <- lavaan.survey(lavaan.fit = NONE2.fit, survey.design = design)

aictab.lavaan(list(FULL2.fit, PARTIAL2.MOD25.fit, NONE2.fit,
                   FULL2.fitadj, PARTIAL2.MOD25.fitadj, NONE2.fitadj),
              c("full mediation", "partial mediation", "no mediation", "full mediation (nested)", "part
```

```
##
## Model selection based on AICc:
##
##                              K    AICc Delta_AICc AICcWt Cum.Wt       LL
## partial mediation           33 9578.75       0.00      1      1 -4755.90
## partial mediation (nested) 42 9596.75      18.00      0      1 -4755.90
## no mediation                32 9630.92      52.17      0      1 -4783.05
## no mediation (nested)       41 9648.92      70.17      0      1 -4783.05
## full mediation              32 9735.60     156.85      0      1 -4835.39
## full mediation (nested)     41 9753.60     174.85      0      1 -4835.39
```

Attendance2 ~ Mean.Sp.BodyMass2

Attendance2 ~ Mean.Sp.BodyMass2 is mediated by **Mean.Raup.Crick2 ~ Mean.Sp.BodyMass2**. Below we compare models with complete (full) mediation, partial mediation (i.e. model 25) and no mediation. Results from this output suggest that partial mediation (model 25) is the superior model.

```
# Complete Mediation Model

FULL3 <- 'Attendance2 ~ Zoo.Area.ha2 + Total.Animals2
+ Mean.Raup.Crick2 + X10km_Pop2 + GDP.Millions2

Total.Animals2 ~ Zoo.Area.ha2 + Sp.Richness2 + GDP.Millions2
Sp.Richness2 ~ Mam.Sp.Richness2 + Mean.Sp.BodyMass2
Mean.Raup.Crick2 ~ Sp.Richness2 + Total.Animals2 + Mean.Sp.BodyMass2'

FULL3.fit <- sem(FULL3, data = sem_attendance_data, fixed.x=FALSE)
design <- svydesign(ids = ~Country, nest=TRUE, data=sem_attendance_data)
FULL3.fitadj <- lavaan.survey(lavaan.fit = FULL3.fit, survey.design = design)

# Partial Mediation Model

PARTIAL3.MOD25 <- 'Attendance2 ~ Zoo.Area.ha2 + Total.Animals2
+ Mean.Sp.BodyMass2 + Mean.Raup.Crick2 + X10km_Pop2 + GDP.Millions2

Total.Animals2 ~ Zoo.Area.ha2 + Sp.Richness2 + GDP.Millions2
Sp.Richness2 ~ Mam.Sp.Richness2 + Mean.Sp.BodyMass2
Mean.Raup.Crick2 ~ Sp.Richness2 + Total.Animals2 + Mean.Sp.BodyMass2'

PARTIAL3.MOD25.fit <- sem(PARTIAL3.MOD25, data = sem_attendance_data, fixed.x=FALSE)
design <- svydesign(ids = ~Country, nest=TRUE, data=sem_attendance_data)
PARTIAL3.MOD25.fitadj <- lavaan.survey(lavaan.fit = PARTIAL3.MOD25.fit, survey.design = design)

# No Mediation Model

NONE3 <- 'Attendance2 ~ Zoo.Area.ha2 + Total.Animals2
+ Mean.Sp.BodyMass2 + Mean.Raup.Crick2 + X10km_Pop2 + GDP.Millions2

Total.Animals2 ~ Zoo.Area.ha2 + Sp.Richness2 + GDP.Millions2
Sp.Richness2 ~ Mam.Sp.Richness2 + Mean.Sp.BodyMass2
Mean.Raup.Crick2 ~ Sp.Richness2 + Total.Animals2'

NONE3.fit <- sem(NONE3, data = sem_attendance_data, fixed.x=FALSE)
design <- svydesign(ids = ~Country, nest=TRUE, data=sem_attendance_data)
```

```
NONE3.fitadj <- lavaan.survey(lavaan.fit = NONE3.fit, survey.design = design)

aictab.lavaan(list(FULL3.fit, PARTIAL3.MOD25.fit, NONE3.fit,
                   FULL3.fitadj, PARTIAL3.MOD25.fitadj, NONE3.fitadj),
              c("full mediation", "partial mediation", "no mediation", "full mediation (nested)", "part
```

```
##
## Model selection based on AICc:
##
##                              K    AICc Delta_AICc AICcWt Cum.Wt       LL
## partial mediation           33 9578.75       0.00      1      1 -4755.90
## partial mediation (nested) 42 9596.75      18.00      0      1 -4755.90
## full mediation              32 9665.11      86.36      0      1 -4800.15
## full mediation (nested)    41 9683.11     104.36      0      1 -4800.15
## no mediation                32 9706.14     127.40      0      1 -4820.66
## no mediation (nested)      41 9724.14     145.40      0      1 -4820.66
```

Attendance2 ~ GDP.Millions2

Attendance2 ~ GDP.Millions2 is mediated by **Total.Animals2 ~ GDP.Millions2**. Below we compare models with complete (full) mediation, partial mediation (i.e. model 25) and no mediation. Results from this output suggest that partial mediation (model 25) is the superior model.

```
# Complete Mediation Model

FULL4 <- 'Attendance2 ~ Zoo.Area.ha2 + Total.Animals2
+ Mean.Sp.BodyMass2 + Mean.Raup.Crick2 + X10km_Pop2

Total.Animals2 ~ Zoo.Area.ha2 + Sp.Richness2 + GDP.Millions2
Sp.Richness2 ~ Mam.Sp.Richness2 + Mean.Sp.BodyMass2
Mean.Raup.Crick2 ~ Sp.Richness2 + Total.Animals2 + Mean.Sp.BodyMass2'

FULL4.fit <- sem(FULL4, data = sem_attendance_data, fixed.x=FALSE)
design <- svydesign(ids = ~Country, nest=TRUE, data=sem_attendance_data)
FULL4.fitadj <- lavaan.survey(lavaan.fit = FULL4.fit, survey.design = design)

# Partial Mediation Model (Model 25)

PARTIAL4.MOD25 <- 'Attendance2 ~ Zoo.Area.ha2 + Total.Animals2
+ Mean.Sp.BodyMass2 + Mean.Raup.Crick2 + X10km_Pop2 + GDP.Millions2

Total.Animals2 ~ Zoo.Area.ha2 + Sp.Richness2 + GDP.Millions2
Sp.Richness2 ~ Mam.Sp.Richness2 + Mean.Sp.BodyMass2
Mean.Raup.Crick2 ~ Sp.Richness2 + Total.Animals2 + Mean.Sp.BodyMass2'

PARTIAL4.MOD25.fit <- sem(PARTIAL4.MOD25, data = sem_attendance_data, fixed.x=FALSE)
design <- svydesign(ids = ~Country, nest=TRUE, data=sem_attendance_data)
PARTIAL4.MOD25.fitadj <- lavaan.survey(lavaan.fit = PARTIAL4.MOD25.fit, survey.design = design)

# No Mediation Model

NONE4 <- 'Attendance2 ~ Zoo.Area.ha2 + Total.Animals2
+ Mean.Sp.BodyMass2 + Mean.Raup.Crick2 + X10km_Pop2 + GDP.Millions2
```

```
Total.Animals2 ~ Zoo.Area.ha2 + Sp.Richness2
Sp.Richness2 ~ Mam.Sp.Richness2 + Mean.Sp.BodyMass2
Mean.Raup.Crick2 ~ Sp.Richness2 + Total.Animals2 + Mean.Sp.BodyMass2'

NONE4.fit <- sem(NONE4, data = sem_attendance_data, fixed.x=FALSE)
design <- svydesign(ids = ~Country, nest=TRUE, data=sem_attendance_data)
NONE4.fitadj <- lavaan.survey(lavaan.fit = NONE4.fit, survey.design = design)

aictab.lavaan(list(FULL4.fit, PARTIAL4.MOD25.fit, NONE4.fit,
                   FULL4.fitadj, PARTIAL4.MOD25.fitadj, NONE4.fitadj),
              c("full mediation", "partial mediation", "no mediation", "full mediation (nested)", "parti
```

```
##
## Model selection based on AICc:
##
##                              K    AICc Delta_AICc AICcWt Cum.Wt       LL
## partial mediation           33 9578.75       0.00      1      1 -4755.90
## partial mediation (nested) 42 9596.75      18.00      0      1 -4755.90
## full mediation              32 9605.14      26.40      0      1 -4770.16
## no mediation                32 9607.62      28.87      0      1 -4771.40
## full mediation (nested)    41 9623.14      44.40      0      1 -4770.16
## no mediation (nested)      41 9625.62      46.87      0      1 -4771.40
```

Validation

Model validation is a process to provide more evidence for the selected model (Fan *et al.*, 2016), yet it is often overlooked or not reported in SEM studies. It is achieved by testing the model with two or more random datasets from the same sample to ensure paramater estimates are similar when a model is based on different datasets from the same population. Here we validate our model using four random subsets (n = 200 each time) of the existing dataset.

Validation Set 1

Parameter estimates relatively constant (some p-value differences).

```
# Chosen Attendance SEM (Presence-Absence)
# Model 25

mod.25.final <- 'Attendance2 ~ Zoo.Area.ha2 + Total.Animals2
+ Mean.Sp.BodyMass2 + Mean.Raup.Crick2
+ X10km_Pop2 + GDP.Millions2

Total.Animals2 ~ Zoo.Area.ha2 + Sp.Richness2 + GDP.Millions2
Sp.Richness2 ~ Mam.Sp.Richness2 + Mean.Sp.BodyMass2
Mean.Raup.Crick2 ~ Total.Animals2 + Mean.Sp.BodyMass2 + Sp.Richness2 + Zoo.Area.ha2'

# Fit model and generate model summary
mod.25.final.fit <- sem(mod.25, data = validationset1, fixed.x=FALSE)
summary(mod.25.final.fit, rsq = TRUE)
```

```
## lavaan (0.5-23.1097) converged normally after  29 iterations
##
```

```
##   Number of observations                           200
##
##   Estimator                                         ML
##   Minimum Function Test Statistic               53.484
##   Degrees of freedom                                11
##   P-value (Chi-square)                           0.000
##
## Parameter Estimates:
##
##   Information                                 Expected
##   Standard Errors                             Standard
##
## Regressions:
##                      Estimate  Std.Err  z-value  P(>|z|)
##   Attendance2 ~
##     Zoo.Area.ha2        0.008    0.057    0.147    0.883
##     Total.Animals2      0.454    0.045   10.053    0.000
##     Men.Sp.BdyMss2      0.393    0.056    7.041    0.000
##     Mean.Rap.Crck2      0.115    0.045    2.555    0.011
##     X10km_Pop2          0.364    0.037    9.809    0.000
##     GDP.Millions2       0.117    0.039    2.964    0.003
##   Total.Animals2 ~
##     Zoo.Area.ha2        0.306    0.039    7.821    0.000
##     Sp.Richness2        0.775    0.039   19.905    0.000
##     GDP.Millions2      -0.104    0.038   -2.759    0.006
##   Sp.Richness2 ~
##     Mam.Sp.Rchnss2      0.824    0.039   21.393    0.000
##     Men.Sp.BdyMss2     -0.300    0.038   -7.888    0.000
##   Mean.Raup.Crick2 ~
##     Total.Animals2      0.349    0.111    3.132    0.002
##     Men.Sp.BdyMss2     -0.645    0.075   -8.576    0.000
##     Sp.Richness2       -0.168    0.108   -1.553    0.120
##     Zoo.Area.ha2        0.274    0.086    3.166    0.002
##
## Covariances:
##                        Estimate  Std.Err  z-value  P(>|z|)
##   Zoo.Area.ha2 ~~
##     Men.Sp.BdyMss2        0.538    0.075    7.128    0.000
##     X10km_Pop2           -0.052    0.069   -0.761    0.447
##     GDP.Millions2        -0.077    0.065   -1.191    0.234
##     Mam.Sp.Rchnss2        0.332    0.069    4.849    0.000
##   Mean.Sp.BodyMass2 ~~
##     X10km_Pop2            0.029    0.072    0.404    0.686
##     GDP.Millions2        -0.013    0.068   -0.193    0.847
##     Mam.Sp.Rchnss2        0.214    0.069    3.087    0.002
##   X10km_Pop2 ~~
##     GDP.Millions2         0.038    0.071    0.540    0.589
##     Mam.Sp.Rchnss2        0.285    0.074    3.849    0.000
##   GDP.Millions2 ~~
##     Mam.Sp.Rchnss2       -0.099    0.067   -1.483    0.138
##
## Variances:
##                    Estimate  Std.Err  z-value  P(>|z|)
##    .Attendance2       0.280    0.028   10.000    0.000
```

```
##    .Total.Animals2    0.263    0.026   10.000    0.000
##    .Sp.Richness2      0.266    0.027   10.000    0.000
##    .Mean.Rap.Crck2    0.679    0.068   10.000    0.000
##     Zoo.Area.ha2      0.879    0.088   10.000    0.000
##     Men.Sp.BdyMss2    0.967    0.097   10.000    0.000
##     X10km_Pop2        1.075    0.107   10.000    0.000
##     GDP.Millions2     0.943    0.094   10.000    0.000
##     Mam.Sp.Rchnss2    0.944    0.094   10.000    0.000
##
## R-Square:
##                    Estimate
##     Attendance2       0.674
##     Total.Animals2    0.726
##     Sp.Richness2      0.701
##     Mean.Rap.Crck2    0.330
```

```
# Adjust for the nested nature of the data (institutions within countries)
# Fit model and generate model summary
design <- svydesign(ids = ~Country, nest=TRUE, data=validationset1)
fit.adj25.final <- lavaan.survey(lavaan.fit = mod.25.final.fit, survey.design = design)
summary(fit.adj25.final, rsq = TRUE)
```

```
## lavaan (0.5-23.1097) converged normally after  28 iterations
##
##   Number of observations                           200
##
##   Estimator                                         ML      Robust
##   Minimum Function Test Statistic               53.484      32.510
##   Degrees of freedom                                11          11
##   P-value (Chi-square)                           0.000       0.001
##   Scaling correction factor                                  1.645
##     for the Satorra-Bentler correction
##
## Parameter Estimates:
##
##   Information                                 Expected
##   Standard Errors                           Robust.sem
##
## Regressions:
##                      Estimate  Std.Err  z-value  P(>|z|)
##   Attendance2 ~
##     Zoo.Area.ha2        0.008    0.053    0.158    0.875
##     Total.Animals2      0.454    0.046    9.951    0.000
##     Men.Sp.BdyMss2      0.393    0.059    6.658    0.000
##     Mean.Rap.Crck2      0.115    0.042    2.748    0.006
##     X10km_Pop2          0.364    0.047    7.716    0.000
##     GDP.Millions2       0.117    0.043    2.679    0.007
##   Total.Animals2 ~
##     Zoo.Area.ha2        0.306    0.070    4.372    0.000
##     Sp.Richness2        0.775    0.078    9.918    0.000
##     GDP.Millions2      -0.104    0.078   -1.322    0.186
##   Sp.Richness2 ~
##     Mam.Sp.Rchnss2      0.824    0.076   10.784    0.000
##     Men.Sp.BdyMss2     -0.300    0.054   -5.534    0.000
##   Mean.Raup.Crick2 ~
```

```
##     Total.Animals2      0.349    0.179    1.950    0.051
##     Men.Sp.BdyMss2     -0.645    0.064  -10.037    0.000
##     Sp.Richness2       -0.168    0.225   -0.747    0.455
##     Zoo.Area.ha2        0.274    0.093    2.936    0.003
##
## Covariances:
##                        Estimate  Std.Err  z-value  P(>|z|)
##   Zoo.Area.ha2 ~~
##     Men.Sp.BdyMss2        0.538    0.112    4.809    0.000
##     X10km_Pop2           -0.052    0.089   -0.588    0.556
##     GDP.Millions2        -0.077    0.058   -1.334    0.182
##     Mam.Sp.Rchnss2        0.332    0.086    3.867    0.000
##   Mean.Sp.BodyMass2 ~~
##     X10km_Pop2            0.029    0.098    0.297    0.767
##     GDP.Millions2        -0.013    0.064   -0.205    0.838
##     Mam.Sp.Rchnss2        0.214    0.092    2.311    0.021
##   X10km_Pop2 ~~
##     GDP.Millions2         0.038    0.078    0.491    0.624
##     Mam.Sp.Rchnss2        0.285    0.092    3.112    0.002
##   GDP.Millions2 ~~
##     Mam.Sp.Rchnss2       -0.099    0.083   -1.197    0.231
##
## Intercepts:
##                    Estimate  Std.Err  z-value  P(>|z|)
##    .Attendance2       0.012    0.043    0.272    0.786
##    .Total.Animals2    0.036    0.075    0.484    0.629
##    .Sp.Richness2      0.024    0.068    0.357    0.721
##    .Mean.Rap.Crck2    0.014    0.091    0.159    0.874
##     Zoo.Area.ha2      0.014    0.080    0.177    0.859
##     Men.Sp.BdyMss2    0.025    0.089    0.284    0.776
##     X10km_Pop2        0.035    0.107    0.333    0.739
##     GDP.Millions2     0.059    0.360    0.163    0.870
##     Mam.Sp.Rchnss2    0.050    0.079    0.638    0.524
##
## Variances:
##                    Estimate  Std.Err  z-value  P(>|z|)
##    .Attendance2       0.280    0.034    8.223    0.000
##    .Total.Animals2    0.263    0.036    7.415    0.000
##    .Sp.Richness2      0.266    0.029    9.259    0.000
##    .Mean.Rap.Crck2    0.679    0.059   11.455    0.000
##     Zoo.Area.ha2      0.879    0.107    8.254    0.000
##     Men.Sp.BdyMss2    0.967    0.173    5.581    0.000
##     X10km_Pop2        1.075    0.246    4.366    0.000
##     GDP.Millions2     0.943    0.243    3.880    0.000
##     Mam.Sp.Rchnss2    0.944    0.159    5.919    0.000
##
## R-Square:
##                    Estimate
##     Attendance2       0.674
##     Total.Animals2    0.726
##     Sp.Richness2      0.701
##     Mean.Rap.Crck2    0.330
```

```r
# Generate fit indices
fitMeasures(fit.adj25.final, c("agfi", "rmr", "srmr", "rmsea", "cfi", "nnfi", "tli"))
```

```
##  agfi   rmr  srmr rmsea   cfi  nnfi   tli
## 0.734 0.063 0.058 0.139 0.950 0.881 0.881
```

Validation Set 2

Parameter estimates relatively constant (some p-value differences).

```r
# Chosen Attendance SEM (Presence-Absence)
# Model 25

mod.25.final <- 'Attendance2 ~ Zoo.Area.ha2 + Total.Animals2
+ Mean.Sp.BodyMass2 + Mean.Raup.Crick2
+ X10km_Pop2 + GDP.Millions2

Total.Animals2 ~ Zoo.Area.ha2 + Sp.Richness2 + GDP.Millions2
Sp.Richness2 ~ Mam.Sp.Richness2 + Mean.Sp.BodyMass2
Mean.Raup.Crick2 ~ Total.Animals2 + Mean.Sp.BodyMass2 + Sp.Richness2 + Zoo.Area.ha2'

# Fit model and generate model summary
mod.25.final.fit <- sem(mod.25, data = validationset2, fixed.x=FALSE)
summary(mod.25.final.fit, rsq = TRUE)
```

```
## lavaan (0.5-23.1097) converged normally after  32 iterations
##
##   Number of observations                           200
##
##   Estimator                                         ML
##   Minimum Function Test Statistic               55.249
##   Degrees of freedom                                11
##   P-value (Chi-square)                           0.000
##
## Parameter Estimates:
##
##   Information                                 Expected
##   Standard Errors                             Standard
##
## Regressions:
##                    Estimate  Std.Err  z-value  P(>|z|)
##   Attendance2 ~
##     Zoo.Area.ha2      0.171    0.044    3.881    0.000
##     Total.Animals2    0.339    0.041    8.363    0.000
##     Men.Sp.BdyMss2    0.303    0.047    6.491    0.000
##     Mean.Rap.Crck2    0.181    0.041    4.444    0.000
##     X10km_Pop2        0.480    0.038   12.635    0.000
##     GDP.Millions2     0.126    0.035    3.599    0.000
##   Total.Animals2 ~
##     Zoo.Area.ha2      0.175    0.031    5.568    0.000
##     Sp.Richness2      0.802    0.030   26.303    0.000
##     GDP.Millions2    -0.128    0.030   -4.325    0.000
##   Sp.Richness2 ~
##     Mam.Sp.Rchnss2    0.856    0.047   18.377    0.000
```

```
##     Men.Sp.BdyMss2     -0.364    0.047   -7.799    0.000
##   Mean.Raup.Crick2 ~
##     Total.Animals2      0.614    0.131    4.682    0.000
##     Men.Sp.BdyMss2     -0.483    0.068   -7.118    0.000
##     Sp.Richness2       -0.435    0.121   -3.598    0.000
##     Zoo.Area.ha2        0.107    0.073    1.477    0.140
##
## Covariances:
##                        Estimate  Std.Err  z-value  P(>|z|)
##   Zoo.Area.ha2 ~~
##     Men.Sp.BdyMss2        0.459    0.078    5.913    0.000
##     X10km_Pop2            0.048    0.070    0.689    0.491
##     GDP.Millions2         0.005    0.073    0.072    0.943
##     Mam.Sp.Rchnss2        0.409    0.076    5.354    0.000
##   Mean.Sp.BodyMass2 ~~
##     X10km_Pop2            0.280    0.073    3.821    0.000
##     GDP.Millions2        -0.168    0.075   -2.247    0.025
##     Mam.Sp.Rchnss2        0.343    0.076    4.546    0.000
##   X10km_Pop2 ~~
##     GDP.Millions2        -0.086    0.073   -1.182    0.237
##     Mam.Sp.Rchnss2        0.301    0.074    4.078    0.000
##   GDP.Millions2 ~~
##     Mam.Sp.Rchnss2       -0.014    0.074   -0.191    0.848
##
## Variances:
##                    Estimate  Std.Err  z-value  P(>|z|)
##    .Attendance2       0.250    0.025   10.000    0.000
##    .Total.Animals2    0.188    0.019   10.000    0.000
##    .Sp.Richness2      0.389    0.039   10.000    0.000
##    .Mean.Rap.Crck2    0.707    0.071   10.000    0.000
##     Zoo.Area.ha2      0.987    0.099   10.000    0.000
##     Men.Sp.BdyMss2    1.009    0.101   10.000    0.000
##     X10km_Pop2        0.985    0.098   10.000    0.000
##     GDP.Millions2     1.074    0.107   10.000    0.000
##     Mam.Sp.Rchnss2    1.013    0.101   10.000    0.000
##
## R-Square:
##                    Estimate
##     Attendance2       0.729
##     Total.Animals2    0.802
##     Sp.Richness2      0.630
##     Mean.Rap.Crck2    0.270
```

```r
# Adjust for the nested nature of the data (institutions within countries)
# Fit model and generate model summary
design <- svydesign(ids = ~Country, nest=TRUE, data=validationset2)
fit.adj25.final <- lavaan.survey(lavaan.fit = mod.25.final.fit, survey.design = design)
summary(fit.adj25.final, rsq = TRUE)
```

```
## lavaan (0.5-23.1097) converged normally after  33 iterations
##
##   Number of observations                           200
##
##   Estimator                                         ML      Robust
##   Minimum Function Test Statistic               55.249      30.741
```

```
##   Degrees of freedom                                 11          11
##   P-value (Chi-square)                            0.000       0.001
##   Scaling correction factor                                   1.797
##     for the Satorra-Bentler correction
##
## Parameter Estimates:
##
##   Information                                 Expected
##   Standard Errors                           Robust.sem
##
## Regressions:
##                    Estimate  Std.Err  z-value  P(>|z|)
##   Attendance2 ~
##     Zoo.Area.ha2      0.171    0.033    5.170    0.000
##     Total.Animals2    0.339    0.041    8.251    0.000
##     Men.Sp.BdyMss2    0.303    0.034    9.009    0.000
##     Mean.Rap.Crck2    0.181    0.032    5.696    0.000
##     X10km_Pop2        0.480    0.044   10.956    0.000
##     GDP.Millions2     0.126    0.024    5.208    0.000
##   Total.Animals2 ~
##     Zoo.Area.ha2      0.175    0.037    4.729    0.000
##     Sp.Richness2      0.802    0.051   15.648    0.000
##     GDP.Millions2    -0.128    0.067   -1.926    0.054
##   Sp.Richness2 ~
##     Mam.Sp.Rchnss2    0.856    0.069   12.340    0.000
##     Men.Sp.BdyMss2   -0.364    0.068   -5.311    0.000
##   Mean.Raup.Crick2 ~
##     Total.Animals2    0.614    0.164    3.743    0.000
##     Men.Sp.BdyMss2   -0.483    0.067   -7.233    0.000
##     Sp.Richness2     -0.435    0.225   -1.929    0.054
##     Zoo.Area.ha2      0.107    0.070    1.538    0.124
##
## Covariances:
##                      Estimate  Std.Err  z-value  P(>|z|)
##   Zoo.Area.ha2 ~~
##     Men.Sp.BdyMss2      0.459    0.112    4.095    0.000
##     X10km_Pop2          0.048    0.076    0.636    0.525
##     GDP.Millions2       0.005    0.049    0.106    0.916
##     Mam.Sp.Rchnss2      0.409    0.079    5.182    0.000
##   Mean.Sp.BodyMass2 ~~
##     X10km_Pop2          0.280    0.108    2.580    0.010
##     GDP.Millions2      -0.168    0.057   -2.925    0.003
##     Mam.Sp.Rchnss2      0.343    0.110    3.112    0.002
##   X10km_Pop2 ~~
##     GDP.Millions2      -0.086    0.073   -1.189    0.235
##     Mam.Sp.Rchnss2      0.301    0.091    3.310    0.001
##   GDP.Millions2 ~~
##     Mam.Sp.Rchnss2     -0.014    0.077   -0.183    0.855
##
## Intercepts:
##                  Estimate  Std.Err  z-value  P(>|z|)
##    .Attendance2    -0.028    0.034   -0.826    0.409
##    .Total.Animals2 -0.028    0.068   -0.413    0.680
##    .Sp.Richness2   -0.012    0.073   -0.168    0.866
```

```
##    .Mean.Rap.Crck2    0.017    0.111    0.156    0.876
##     Zoo.Area.ha2     -0.028    0.083   -0.331    0.741
##     Men.Sp.BdyMss2   -0.040    0.103   -0.387    0.699
##     X10km_Pop2       -0.013    0.129   -0.104    0.917
##     GDP.Millions2    -0.044    0.340   -0.131    0.896
##     Mam.Sp.Rchnss2   -0.018    0.101   -0.175    0.861
##
## Variances:
##                    Estimate  Std.Err  z-value  P(>|z|)
##    .Attendance2       0.250    0.030    8.218    0.000
##    .Total.Animals2    0.188    0.044    4.312    0.000
##    .Sp.Richness2      0.389    0.039   10.085    0.000
##    .Mean.Rap.Crck2    0.707    0.080    8.793    0.000
##     Zoo.Area.ha2      0.987    0.126    7.849    0.000
##     Men.Sp.BdyMss2    1.009    0.185    5.445    0.000
##     X10km_Pop2        0.985    0.160    6.171    0.000
##     GDP.Millions2     1.074    0.270    3.975    0.000
##     Mam.Sp.Rchnss2    1.013    0.121    8.400    0.000
##
## R-Square:
##                    Estimate
##     Attendance2       0.729
##     Total.Animals2    0.802
##     Sp.Richness2      0.630
##     Mean.Rap.Crck2    0.270
```

```
# Generate fit indices
fitMeasures(fit.adj25.final, c("agfi", "rmr", "srmr", "rmsea", "cfi", "nnfi", "tli"))
```

```
##  agfi   rmr  srmr rmsea   cfi  nnfi   tli
## 0.731 0.066 0.060 0.142 0.951 0.885 0.885
```

Validation Set 3

Parameter estimates relatively constant (some p-value differences).

```
# Chosen Attendance SEM (Presence-Absence)
# Model 25

mod.25.final <- 'Attendance2 ~ Zoo.Area.ha2 + Total.Animals2
+ Mean.Sp.BodyMass2 + Mean.Raup.Crick2
+ X10km_Pop2 + GDP.Millions2

Total.Animals2 ~ Zoo.Area.ha2 + Sp.Richness2 + GDP.Millions2
Sp.Richness2 ~ Mam.Sp.Richness2 + Mean.Sp.BodyMass2
Mean.Raup.Crick2 ~ Total.Animals2 + Mean.Sp.BodyMass2 + Sp.Richness2 + Zoo.Area.ha2'

# Fit model and generate model summary
mod.25.final.fit <- sem(mod.25, data = validationset3, fixed.x=FALSE)
summary(mod.25.final.fit, rsq = TRUE)
```

```
## lavaan (0.5-23.1097) converged normally after  34 iterations
##
##   Number of observations                           200
##
```

```
##   Estimator                                         ML
##   Minimum Function Test Statistic               45.405
##   Degrees of freedom                                11
##   P-value (Chi-square)                           0.000
##
## Parameter Estimates:
##
##   Information                                 Expected
##   Standard Errors                             Standard
##
## Regressions:
##                      Estimate  Std.Err  z-value  P(>|z|)
##   Attendance2 ~
##     Zoo.Area.ha2        0.048    0.048    1.001    0.317
##     Total.Animals2      0.436    0.043   10.092    0.000
##     Men.Sp.BdyMss2      0.327    0.050    6.481    0.000
##     Mean.Rap.Crck2      0.145    0.043    3.348    0.001
##     X10km_Pop2          0.415    0.039   10.760    0.000
##     GDP.Millions2       0.138    0.036    3.815    0.000
##   Total.Animals2 ~
##     Zoo.Area.ha2        0.231    0.034    6.724    0.000
##     Sp.Richness2        0.811    0.037   22.075    0.000
##     GDP.Millions2      -0.130    0.033   -3.907    0.000
##   Sp.Richness2 ~
##     Mam.Sp.Rchnss2      0.817    0.044   18.616    0.000
##     Men.Sp.BdyMss2     -0.393    0.043   -9.055    0.000
##   Mean.Raup.Crick2 ~
##     Total.Animals2      0.471    0.117    4.014    0.000
##     Men.Sp.BdyMss2     -0.580    0.070   -8.238    0.000
##     Sp.Richness2       -0.343    0.115   -2.983    0.003
##     Zoo.Area.ha2        0.164    0.076    2.167    0.030
##
## Covariances:
##                        Estimate  Std.Err  z-value  P(>|z|)
##   Zoo.Area.ha2 ~~
##     Men.Sp.BdyMss2        0.538    0.081    6.636    0.000
##     X10km_Pop2           -0.016    0.070   -0.234    0.815
##     GDP.Millions2        -0.048    0.073   -0.651    0.515
##     Mam.Sp.Rchnss2        0.441    0.077    5.703    0.000
##   Mean.Sp.BodyMass2 ~~
##     X10km_Pop2            0.092    0.070    1.325    0.185
##     GDP.Millions2        -0.056    0.073   -0.760    0.447
##     Mam.Sp.Rchnss2        0.391    0.076    5.161    0.000
##   X10km_Pop2 ~~
##     GDP.Millions2         0.016    0.071    0.222    0.825
##     Mam.Sp.Rchnss2        0.278    0.071    3.889    0.000
##   GDP.Millions2 ~~
##     Mam.Sp.Rchnss2       -0.049    0.072   -0.677    0.499
##
## Variances:
##                    Estimate  Std.Err  z-value  P(>|z|)
##    .Attendance2       0.269    0.027   10.000    0.000
##    .Total.Animals2    0.233    0.023   10.000    0.000
##    .Sp.Richness2      0.322    0.032   10.000    0.000
```

```
##    .Mean.Rap.Crck2    0.689    0.069   10.000    0.000
##     Zoo.Area.ha2      1.016    0.102   10.000    0.000
##     Men.Sp.BdyMss2    1.007    0.101   10.000    0.000
##     X10km_Pop2        0.958    0.096   10.000    0.000
##     GDP.Millions2     1.058    0.106   10.000    0.000
##     Mam.Sp.Rchnss2    0.986    0.099   10.000    0.000
##
## R-Square:
##                    Estimate
##     Attendance2       0.683
##     Total.Animals2    0.755
##     Sp.Richness2      0.637
##     Mean.Rap.Crck2    0.307
```

```
# Adjust for the nested nature of the data (institutions within countries)
# Fit model and generate model summary
design <- svydesign(ids = ~Country, nest=TRUE, data=validationset3)
fit.adj25.final <- lavaan.survey(lavaan.fit = mod.25.final.fit, survey.design = design)
summary(fit.adj25.final, rsq = TRUE)
```

```
## lavaan (0.5-23.1097) converged normally after  34 iterations
##
##   Number of observations                           200
##
##   Estimator                                         ML      Robust
##   Minimum Function Test Statistic               45.405      24.386
##   Degrees of freedom                                11          11
##   P-value (Chi-square)                           0.000       0.011
##   Scaling correction factor                                  1.862
##     for the Satorra-Bentler correction
##
## Parameter Estimates:
##
##   Information                                 Expected
##   Standard Errors                           Robust.sem
##
## Regressions:
##                      Estimate  Std.Err  z-value  P(>|z|)
##   Attendance2 ~
##     Zoo.Area.ha2        0.048    0.050    0.961    0.337
##     Total.Animals2      0.436    0.043   10.171    0.000
##     Men.Sp.BdyMss2      0.327    0.055    5.949    0.000
##     Mean.Rap.Crck2      0.145    0.043    3.351    0.001
##     X10km_Pop2          0.415    0.057    7.297    0.000
##     GDP.Millions2       0.138    0.032    4.328    0.000
##   Total.Animals2 ~
##     Zoo.Area.ha2        0.231    0.051    4.510    0.000
##     Sp.Richness2        0.811    0.098    8.243    0.000
##     GDP.Millions2      -0.130    0.074   -1.756    0.079
##   Sp.Richness2 ~
##     Mam.Sp.Rchnss2      0.817    0.069   11.760    0.000
##     Men.Sp.BdyMss2     -0.393    0.062   -6.319    0.000
##   Mean.Raup.Crick2 ~
##     Total.Animals2      0.471    0.201    2.345    0.019
##     Men.Sp.BdyMss2     -0.580    0.069   -8.429    0.000
```

```
##     Sp.Richness2       -0.343    0.259   -1.328    0.184
##     Zoo.Area.ha2        0.164    0.055    2.987    0.003
##
## Covariances:
##                        Estimate  Std.Err  z-value  P(>|z|)
##   Zoo.Area.ha2 ~~
##     Men.Sp.BdyMss2        0.538    0.096    5.589    0.000
##     X10km_Pop2           -0.016    0.116   -0.141    0.888
##     GDP.Millions2        -0.048    0.062   -0.773    0.439
##     Mam.Sp.Rchnss2        0.441    0.078    5.680    0.000
##   Mean.Sp.BodyMass2 ~~
##     X10km_Pop2            0.092    0.095    0.974    0.330
##     GDP.Millions2        -0.056    0.071   -0.777    0.437
##     Mam.Sp.Rchnss2        0.391    0.088    4.436    0.000
##   X10km_Pop2 ~~
##     GDP.Millions2         0.016    0.071    0.224    0.823
##     Mam.Sp.Rchnss2        0.278    0.080    3.484    0.000
##   GDP.Millions2 ~~
##     Mam.Sp.Rchnss2       -0.049    0.072   -0.675    0.499
##
## Intercepts:
##                    Estimate  Std.Err  z-value  P(>|z|)
##    .Attendance2       0.001    0.040    0.023    0.982
##    .Total.Animals2    0.005    0.083    0.063    0.950
##    .Sp.Richness2     -0.007    0.078   -0.095    0.924
##    .Mean.Rap.Crck2    0.082    0.111    0.736    0.461
##     Zoo.Area.ha2     -0.076    0.079   -0.969    0.333
##     Men.Sp.BdyMss2   -0.034    0.093   -0.369    0.712
##     X10km_Pop2        0.028    0.103    0.268    0.789
##     GDP.Millions2     0.013    0.363    0.036    0.971
##     Mam.Sp.Rchnss2   -0.032    0.086   -0.373    0.709
##
## Variances:
##                    Estimate  Std.Err  z-value  P(>|z|)
##    .Attendance2       0.269    0.037    7.209    0.000
##    .Total.Animals2    0.233    0.061    3.843    0.000
##    .Sp.Richness2      0.322    0.040    8.112    0.000
##    .Mean.Rap.Crck2    0.689    0.074    9.317    0.000
##     Zoo.Area.ha2      1.016    0.150    6.798    0.000
##     Men.Sp.BdyMss2    1.007    0.176    5.734    0.000
##     X10km_Pop2        0.958    0.202    4.733    0.000
##     GDP.Millions2     1.058    0.254    4.172    0.000
##     Mam.Sp.Rchnss2    0.986    0.103    9.607    0.000
##
## R-Square:
##                    Estimate
##     Attendance2       0.683
##     Total.Animals2    0.755
##     Sp.Richness2      0.637
##     Mean.Rap.Crck2    0.307
```

```
# Generate fit indices
fitMeasures(fit.adj25.final, c("agfi", "rmr", "srmr", "rmsea", "cfi", "nnfi", "tli"))
```

```
##  agfi   rmr  srmr rmsea   cfi  nnfi   tli
```

```
## 0.763 0.060 0.057 0.125 0.959 0.902 0.902
```

Validation Set 4

Parameter estimates relatively constant (some p-value differences).

```r
# Chosen Attendance SEM (Presence-Absence)
# Model 25

mod.25.final <- 'Attendance2 ~ Zoo.Area.ha2 + Total.Animals2
+ Mean.Sp.BodyMass2 + Mean.Raup.Crick2
+ X10km_Pop2 + GDP.Millions2

Total.Animals2 ~ Zoo.Area.ha2 + Sp.Richness2 + GDP.Millions2
Sp.Richness2 ~ Mam.Sp.Richness2 + Mean.Sp.BodyMass2
Mean.Raup.Crick2 ~ Total.Animals2 + Mean.Sp.BodyMass2 + Sp.Richness2 + Zoo.Area.ha2'

# Fit model and generate model summary
mod.25.final.fit <- sem(mod.25, data = validationset4, fixed.x=FALSE)
summary(mod.25.final.fit, rsq = TRUE)
```

```
## lavaan (0.5-23.1097) converged normally after  31 iterations
##
##   Number of observations                           200
##
##   Estimator                                         ML
##   Minimum Function Test Statistic               43.646
##   Degrees of freedom                                11
##   P-value (Chi-square)                           0.000
##
## Parameter Estimates:
##
##   Information                                 Expected
##   Standard Errors                             Standard
##
## Regressions:
##                      Estimate  Std.Err  z-value  P(>|z|)
##   Attendance2 ~
##     Zoo.Area.ha2        0.148    0.050    2.983    0.003
##     Total.Animals2      0.397    0.040    9.862    0.000
##     Men.Sp.BdyMss2      0.361    0.052    6.956    0.000
##     Mean.Rap.Crck2      0.101    0.043    2.331    0.020
##     X10km_Pop2          0.416    0.036   11.408    0.000
##     GDP.Millions2       0.139    0.039    3.567    0.000
##   Total.Animals2 ~
##     Zoo.Area.ha2        0.208    0.035    5.920    0.000
##     Sp.Richness2        0.812    0.033   24.808    0.000
##     GDP.Millions2      -0.111    0.035   -3.155    0.002
##   Sp.Richness2 ~
##     Mam.Sp.Rchnss2      0.902    0.045   20.177    0.000
##     Men.Sp.BdyMss2     -0.376    0.042   -8.873    0.000
##   Mean.Raup.Crick2 ~
##     Total.Animals2      0.470    0.120    3.919    0.000
##     Men.Sp.BdyMss2     -0.569    0.073   -7.773    0.000
```

```
##     Sp.Richness2       -0.313    0.114   -2.743    0.006
##     Zoo.Area.ha2        0.194    0.078    2.480    0.013
##
## Covariances:
##                        Estimate  Std.Err  z-value  P(>|z|)
##   Zoo.Area.ha2 ~~
##     Men.Sp.BdyMss2        0.570    0.083    6.868    0.000
##     X10km_Pop2           -0.025    0.075   -0.331    0.741
##     GDP.Millions2        -0.020    0.070   -0.287    0.774
##     Mam.Sp.Rchnss2        0.292    0.072    4.070    0.000
##   Mean.Sp.BodyMass2 ~~
##     X10km_Pop2            0.127    0.078    1.623    0.105
##     GDP.Millions2        -0.145    0.073   -1.989    0.047
##     Mam.Sp.Rchnss2        0.218    0.073    2.998    0.003
##   X10km_Pop2 ~~
##     GDP.Millions2        -0.119    0.075   -1.583    0.113
##     Mam.Sp.Rchnss2        0.258    0.076    3.403    0.001
##   GDP.Millions2 ~~
##     Mam.Sp.Rchnss2       -0.057    0.068   -0.829    0.407
##
## Variances:
##                    Estimate  Std.Err  z-value  P(>|z|)
##    .Attendance2       0.283    0.028   10.000    0.000
##    .Total.Animals2    0.243    0.024   10.000    0.000
##    .Sp.Richness2      0.362    0.036   10.000    0.000
##    .Mean.Rap.Crck2    0.734    0.073   10.000    0.000
##     Zoo.Area.ha2      0.992    0.099   10.000    0.000
##     Men.Sp.BdyMss2    1.060    0.106   10.000    0.000
##     X10km_Pop2        1.140    0.114   10.000    0.000
##     GDP.Millions2     0.980    0.098   10.000    0.000
##     Mam.Sp.Rchnss2    0.952    0.095   10.000    0.000
##
## R-Square:
##                    Estimate
##     Attendance2       0.702
##     Total.Animals2    0.772
##     Sp.Richness2      0.682
##     Mean.Rap.Crck2    0.297
```

```
# Adjust for the nested nature of the data (institutions within countries)
# Fit model and generate model summary
design <- svydesign(ids = ~Country, nest=TRUE, data=validationset4)
fit.adj25.final <- lavaan.survey(lavaan.fit = mod.25.final.fit, survey.design = design)
summary(fit.adj25.final, rsq = TRUE)
```

```
## lavaan (0.5-23.1097) converged normally after  32 iterations
##
##   Number of observations                           200
##
##   Estimator                                         ML      Robust
##   Minimum Function Test Statistic               43.646      29.310
##   Degrees of freedom                                11          11
##   P-value (Chi-square)                           0.000       0.002
##   Scaling correction factor                                  1.489
##     for the Satorra-Bentler correction
```

```
##
## Parameter Estimates:
##
##   Information                                 Expected
##   Standard Errors                           Robust.sem
##
## Regressions:
##                      Estimate  Std.Err  z-value  P(>|z|)
##   Attendance2 ~
##     Zoo.Area.ha2        0.148    0.053    2.812    0.005
##     Total.Animals2      0.397    0.035   11.226    0.000
##     Men.Sp.BdyMss2      0.361    0.048    7.535    0.000
##     Mean.Rap.Crck2      0.101    0.037    2.731    0.006
##     X10km_Pop2          0.416    0.039   10.623    0.000
##     GDP.Millions2       0.139    0.042    3.345    0.001
##   Total.Animals2 ~
##     Zoo.Area.ha2        0.208    0.037    5.550    0.000
##     Sp.Richness2        0.812    0.036   22.558    0.000
##     GDP.Millions2      -0.111    0.078   -1.421    0.155
##   Sp.Richness2 ~
##     Mam.Sp.Rchnss2      0.902    0.101    8.971    0.000
##     Men.Sp.BdyMss2     -0.376    0.057   -6.572    0.000
##   Mean.Raup.Crick2 ~
##     Total.Animals2      0.470    0.140    3.356    0.001
##     Men.Sp.BdyMss2     -0.569    0.076   -7.471    0.000
##     Sp.Richness2       -0.313    0.215   -1.456    0.145
##     Zoo.Area.ha2        0.194    0.087    2.217    0.027
##
## Covariances:
##                        Estimate  Std.Err  z-value  P(>|z|)
##   Zoo.Area.ha2 ~~
##     Men.Sp.BdyMss2        0.570    0.136    4.203    0.000
##     X10km_Pop2           -0.025    0.083   -0.301    0.763
##     GDP.Millions2        -0.020    0.074   -0.269    0.788
##     Mam.Sp.Rchnss2        0.292    0.087    3.343    0.001
##   Mean.Sp.BodyMass2 ~~
##     X10km_Pop2            0.127    0.104    1.223    0.221
##     GDP.Millions2        -0.145    0.063   -2.302    0.021
##     Mam.Sp.Rchnss2        0.218    0.119    1.839    0.066
##   X10km_Pop2 ~~
##     GDP.Millions2        -0.119    0.071   -1.673    0.094
##     Mam.Sp.Rchnss2        0.258    0.095    2.716    0.007
##   GDP.Millions2 ~~
##     Mam.Sp.Rchnss2       -0.057    0.093   -0.612    0.540
##
## Intercepts:
##                    Estimate  Std.Err  z-value  P(>|z|)
##    .Attendance2      -0.030    0.036   -0.836    0.403
##    .Total.Animals2    0.007    0.073    0.091    0.927
##    .Sp.Richness2      0.030    0.060    0.508    0.611
##    .Mean.Rap.Crck2   -0.041    0.099   -0.415    0.678
##     Zoo.Area.ha2      0.067    0.083    0.813    0.417
##     Men.Sp.BdyMss2    0.021    0.110    0.190    0.849
##     X10km_Pop2       -0.008    0.133   -0.059    0.953
```

```
##     GDP.Millions2    -0.027    0.338   -0.079    0.937
##     Mam.Sp.Rchnss2    0.013    0.099    0.130    0.896
##
## Variances:
##                    Estimate  Std.Err  z-value  P(>|z|)
##    .Attendance2       0.283    0.039    7.219    0.000
##    .Total.Animals2    0.243    0.035    6.884    0.000
##    .Sp.Richness2      0.362    0.049    7.393    0.000
##    .Mean.Rap.Crck2    0.734    0.095    7.703    0.000
##     Zoo.Area.ha2      0.992    0.107    9.300    0.000
##     Men.Sp.BdyMss2    1.060    0.207    5.115    0.000
##     X10km_Pop2        1.140    0.196    5.819    0.000
##     GDP.Millions2     0.980    0.264    3.708    0.000
##     Mam.Sp.Rchnss2    0.952    0.192    4.963    0.000
##
## R-Square:
##                    Estimate
##     Attendance2       0.702
##     Total.Animals2    0.772
##     Sp.Richness2      0.682
##     Mean.Rap.Crck2    0.297
```

```
# Generate fit indices
fitMeasures(fit.adj25.final, c("agfi", "rmr", "srmr", "rmsea", "cfi", "nnfi", "tli"))
```

```
##  agfi   rmr  srmr rmsea   cfi  nnfi   tli
## 0.787 0.063 0.053 0.122 0.963 0.911 0.911
```

Across all the validation models while we see some differences regarding p-values, as would be expected we see qualitatively similar parameter estimates across the four validation sets.

Attendance SEM

Contents

Two models were developed, one based on species presence-absence per institution and the the other which also conisdered species' population sizes per institution. This was important for calculating mean species body mass per institution, the proportion of threatened species per institution etc. Here we develop the model based on species abundance. Species abundance was incorporated into the following metrics: Mean species body mass per institution, the proportion of mammals per institution and the proportion of threatened species per institution.

Species Abundance

Model 1 (a priori meta-model)

We now define our starting model for the SEM based on the a priori meta-model which is constructed based on evidence from the litrature and combined with proposed causal hypotheses. This model includes all previously identified evidence-based relationships and the numerous proposed causal pathways to visitor attendance. The development of this model can be found in the Supplementary information.

```
# Attendance SEM (Species Abundance)
```

```r
# Model 1
# Based on a priori meta-model and proposed causal pathways

mod.1A <- 'Attendance2 ~ Zoo.Area.ha2 + Sp.Richness2 + Total.Animals2
+ Mam.Sp.Richness2 + Prop.Mam.Abdun2 + Prop.Threat.Abund2
+ Mean.Sp.BodyMassXAbund2 + Brillouin.Index2 + Mean.Raup.Crick2
+ X50km_Pop2 + X10km_Pop2 + GDP.Millions2 + Nat_Pop._WB20152

Total.Animals2 ~ Zoo.Area.ha2 + Sp.Richness2
Sp.Richness2 ~ Zoo.Area.ha2 + Prop.Mam.Sp2
Prop.Threat.Abund2 ~ Brillouin.Index2
Brillouin.Index2 ~ Sp.Richness2 + Total.Animals2
Mean.Raup.Crick2 ~ Sp.Richness2 + Total.Animals2'
```

We then fit the model, generate the model summary and generate a selection of absolute fit indices (e.g. Standardized Root Mean Square Residual) and incremental fit indices (e.g. Comparative Fit Index), to account for the differential sensitivity of fit indices to data distribution, model size and sample size (Hu & Bentler, 1999). We then generate modification indices to identify suspected pathways for inclusion, with a standard cut-off level for the chi-square test criterion of 3.84 (Burnham and Anderson, 2002).

Given the nested nature of the data (institutions within countries), we then adjust the model design to take this into considerdation. This is done using the lavaan.survey package. Once again, the model summary, fit indices and modification indices were all generated.

```r
# Fit model and generate model summary
mod.1A.fit <- sem(mod.1A, data = sem_attendance_data, fixed.x=FALSE)
summary(mod.1A.fit, rsq = TRUE)
```

```
## lavaan (0.5-23.1097) converged normally after  61 iterations
##
##   Number of observations                           458
##
##   Estimator                                         ML
##   Minimum Function Test Statistic             1213.624
##   Degrees of freedom                                47
##   P-value (Chi-square)                           0.000
##
## Parameter Estimates:
##
##   Information                                 Expected
##   Standard Errors                             Standard
##
## Regressions:
##                    Estimate  Std.Err  z-value  P(>|z|)
##   Attendance2 ~
##     Zoo.Area.ha2      0.082    0.035    2.342    0.019
##     Sp.Richness2     -0.280    0.074   -3.799    0.000
##     Total.Animals2    0.548    0.055   10.026    0.000
##     Mam.Sp.Rchnss2    0.146    0.029    5.058    0.000
##     Prop.Mam.Abdn2   -0.100    0.035   -2.836    0.005
##     Prp.Thrt.Abnd2    0.017    0.024    0.700    0.484
##     Mn.Sp.BdyMsXA2    0.324    0.034    9.401    0.000
##     Brilloun.Indx2    0.025    0.045    0.563    0.573
##     Mean.Rap.Crck2    0.116    0.025    4.612    0.000
##     X50km_Pop2        0.073    0.038    1.912    0.056
```

```
##     X10km_Pop2           0.417    0.041   10.146    0.000
##     GDP.Millions2        0.263    0.048    5.532    0.000
##     Nt_Pp._WB20152      -0.128    0.049   -2.603    0.009
##   Total.Animals2 ~
##     Zoo.Area.ha2         0.223    0.024    9.384    0.000
##     Sp.Richness2         0.798    0.024   33.520    0.000
##   Sp.Richness2 ~
##     Zoo.Area.ha2         0.363    0.039    9.240    0.000
##     Prop.Mam.Sp2        -0.607    0.039  -15.463    0.000
##   Prop.Threat.Abund2 ~
##     Brilloun.Indx2       0.107    0.046    2.311    0.021
##   Brillouin.Index2 ~
##     Sp.Richness2         1.232    0.046   26.770    0.000
##     Total.Animals2      -0.527    0.046  -11.451    0.000
##   Mean.Raup.Crick2 ~
##     Sp.Richness2        -0.135    0.083   -1.636    0.102
##     Total.Animals2       0.355    0.083    4.302    0.000
##
## Covariances:
##                           Estimate  Std.Err  z-value  P(>|z|)
##   Zoo.Area.ha2 ~~
##     Mam.Sp.Rchnss2           0.381    0.050    7.634    0.000
##     Prop.Mam.Abdn2           0.352    0.049    7.121    0.000
##     Mn.Sp.BdyMsXA2           0.522    0.053    9.923    0.000
##     X50km_Pop2               0.060    0.047    1.291    0.197
##     X10km_Pop2              -0.010    0.047   -0.208    0.835
##     GDP.Millions2           -0.027    0.047   -0.588    0.557
##     Nt_Pp._WB20152           0.061    0.047    1.310    0.190
##     Prop.Mam.Sp2             0.312    0.049    6.389    0.000
##   Mam.Sp.Richness2 ~~
##     Prop.Mam.Abdn2           0.058    0.047    1.237    0.216
##     Mn.Sp.BdyMsXA2           0.227    0.048    4.750    0.000
##     X50km_Pop2               0.204    0.048    4.281    0.000
##     X10km_Pop2               0.284    0.048    5.852    0.000
##     GDP.Millions2           -0.060    0.047   -1.277    0.202
##     Nt_Pp._WB20152          -0.088    0.047   -1.881    0.060
##     Prop.Mam.Sp2             0.088    0.047    1.882    0.060
##   Prop.Mam.Abdun2 ~~
##     Mn.Sp.BdyMsXA2           0.592    0.054   10.922    0.000
##     X50km_Pop2              -0.123    0.047   -2.622    0.009
##     X10km_Pop2              -0.248    0.048   -5.165    0.000
##     GDP.Millions2           -0.115    0.047   -2.458    0.014
##     Nt_Pp._WB20152          -0.111    0.047   -2.362    0.018
##     Prop.Mam.Sp2             0.878    0.062   14.135    0.000
##   Mean.Sp.BodyMassXAbund2 ~~
##     X50km_Pop2               0.013    0.047    0.275    0.784
##     X10km_Pop2               0.021    0.047    0.455    0.649
##     GDP.Millions2           -0.079    0.047   -1.682    0.093
##     Nt_Pp._WB20152          -0.004    0.047   -0.079    0.937
##     Prop.Mam.Sp2             0.628    0.055   11.405    0.000
##   X50km_Pop2 ~~
##     X10km_Pop2               0.752    0.058   12.886    0.000
##     GDP.Millions2           -0.013    0.047   -0.281    0.779
##     Nt_Pp._WB20152           0.191    0.047    4.033    0.000
```

```
##     Prop.Mam.Sp2              -0.096    0.047   -2.054    0.040
##   X10km_Pop2 ~~
##     GDP.Millions2             -0.028    0.047   -0.604    0.546
##     Nt_Pp._WB20152             0.173    0.047    3.646    0.000
##     Prop.Mam.Sp2              -0.204    0.048   -4.293    0.000
##   GDP.Millions2 ~~
##     Nt_Pp._WB20152             0.826    0.061   13.642    0.000
##     Prop.Mam.Sp2              -0.123    0.047   -2.615    0.009
##   Nat_Pop._WB20152 ~~
##     Prop.Mam.Sp2              -0.115    0.047   -2.447    0.014
##
## Variances:
##                    Estimate  Std.Err  z-value  P(>|z|)
##    .Attendance2       0.270    0.018   15.133    0.000
##    .Total.Animals2    0.251    0.017   15.133    0.000
##    .Sp.Richness2      0.636    0.042   15.133    0.000
##    .Prp.Thrt.Abnd2    0.986    0.065   15.133    0.000
##    .Brilloun.Indx2    0.290    0.019   15.133    0.000
##    .Mean.Rap.Crck2    0.934    0.062   15.133    0.000
##     Zoo.Area.ha2      0.998    0.066   15.133    0.000
##     Mam.Sp.Rchnss2    0.998    0.066   15.133    0.000
##     Prop.Mam.Abdn2    0.998    0.066   15.133    0.000
##     Mn.Sp.BdyMsXA2    0.998    0.066   15.133    0.000
##     X50km_Pop2        0.998    0.066   15.133    0.000
##     X10km_Pop2        0.998    0.066   15.133    0.000
##     GDP.Millions2     0.998    0.066   15.133    0.000
##     Nt_Pp._WB20152    0.998    0.066   15.133    0.000
##     Prop.Mam.Sp2      0.998    0.066   15.133    0.000
##
## R-Square:
##                    Estimate
##     Attendance2       0.718
##     Total.Animals2    0.748
##     Sp.Richness2      0.363
##     Prp.Thrt.Abnd2    0.012
##     Brilloun.Indx2    0.709
##     Mean.Rap.Crck2    0.064
```

```r
# Generate fit indices
fitMeasures(mod.1A.fit, c("agfi", "rmr", "srmr", "rmsea", "cfi", "nnfi", "tli"))
```

```
##  agfi   rmr  srmr rmsea   cfi  nnfi   tli
## 0.525 0.137 0.137 0.233 0.633 0.462 0.462
```

```r
# Generate modification indices
mi1A <- modindices(mod.1A.fit)
print(mi1A[mi1A$mi > 3.0,])
```

```
##                     lhs op                     rhs     mi    epc
## 82        Total.Animals2 ~~         Mean.Raup.Crick2 11.524  0.191
## 84          Sp.Richness2 ~~        Brillouin.Index2  5.179  0.076
## 85          Sp.Richness2 ~~         Mean.Raup.Crick2 78.109 -0.531
## 86      Prop.Threat.Abund2 ~~      Brillouin.Index2 13.812  0.110
## 93        Total.Animals2  ~         Mean.Raup.Crick2 11.524  0.205
## 95        Total.Animals2  ~          Prop.Mam.Abdun2  7.409 -0.079
## 96        Total.Animals2  ~ Mean.Sp.BodyMassXAbund2 24.991 -0.146
```

```
## 97           Total.Animals2  ~              X50km_Pop2  34.446  0.138
## 98           Total.Animals2  ~              X10km_Pop2   7.972  0.067
## 99           Total.Animals2  ~            GDP.Millions2  30.032 -0.129
## 100          Total.Animals2  ~        Nat_Pop._WB20152   8.055 -0.067
## 102            Sp.Richness2  ~             Attendance2  57.923  0.433
## 105            Sp.Richness2  ~        Brillouin.Index2   5.103  0.235
## 106            Sp.Richness2  ~        Mean.Raup.Crick2  77.140 -0.556
## 107            Sp.Richness2  ~        Mam.Sp.Richness2 349.341  0.755
## 108            Sp.Richness2  ~         Prop.Mam.Abdun2   6.273 -0.199
## 110            Sp.Richness2  ~              X50km_Pop2  13.582  0.139
## 111            Sp.Richness2  ~              X10km_Pop2  32.091  0.216
## 113            Sp.Richness2  ~        Nat_Pop._WB20152  10.411 -0.122
## 114      Prop.Threat.Abund2  ~             Attendance2   5.928  0.138
## 115      Prop.Threat.Abund2  ~          Total.Animals2   5.628 -0.128
## 116      Prop.Threat.Abund2  ~            Sp.Richness2  13.491 -0.279
## 120      Prop.Threat.Abund2  ~         Prop.Mam.Abdun2   6.180  0.124
## 121      Prop.Threat.Abund2  ~ Mean.Sp.BodyMassXAbund2  14.929  0.184
## 122      Prop.Threat.Abund2  ~              X50km_Pop2  11.699  0.159
## 123      Prop.Threat.Abund2  ~              X10km_Pop2   7.106  0.124
## 125      Prop.Threat.Abund2  ~        Nat_Pop._WB20152   7.100  0.124
## 126      Prop.Threat.Abund2  ~            Prop.Mam.Sp2  19.197  0.226
## 128        Brillouin.Index2  ~      Prop.Threat.Abund2  13.812  0.112
## 132        Brillouin.Index2  ~         Prop.Mam.Abdun2  13.234  0.102
## 133        Brillouin.Index2  ~ Mean.Sp.BodyMassXAbund2  10.854  0.087
## 135        Brillouin.Index2  ~              X10km_Pop2   3.677 -0.049
## 136        Brillouin.Index2  ~            GDP.Millions2  58.745 -0.194
## 137        Brillouin.Index2  ~        Nat_Pop._WB20152  51.007 -0.181
## 138        Brillouin.Index2  ~            Prop.Mam.Sp2   9.791  0.092
## 139        Mean.Raup.Crick2  ~             Attendance2  13.736 -0.258
## 142        Mean.Raup.Crick2  ~             Zoo.Area.ha2  11.525 -0.170
## 143        Mean.Raup.Crick2  ~        Mam.Sp.Richness2  18.995 -0.200
## 144        Mean.Raup.Crick2  ~         Prop.Mam.Abdun2  63.958 -0.403
## 145        Mean.Raup.Crick2  ~ Mean.Sp.BodyMassXAbund2  97.877 -0.468
## 146        Mean.Raup.Crick2  ~              X50km_Pop2   6.052  0.112
## 147        Mean.Raup.Crick2  ~              X10km_Pop2   4.241  0.094
## 149        Mean.Raup.Crick2  ~        Nat_Pop._WB20152  10.765  0.149
## 150        Mean.Raup.Crick2  ~            Prop.Mam.Sp2 106.002 -0.545
## 152            Zoo.Area.ha2  ~          Total.Animals2  15.539 -0.290
## 153            Zoo.Area.ha2  ~            Sp.Richness2  71.869 -0.649
## 155            Zoo.Area.ha2  ~        Brillouin.Index2  40.396 -0.354
## 156            Zoo.Area.ha2  ~        Mean.Raup.Crick2   4.521  0.085
## 165        Mam.Sp.Richness2  ~             Attendance2  31.541  0.630
## 166        Mam.Sp.Richness2  ~          Total.Animals2 149.499  0.591
## 167        Mam.Sp.Richness2  ~            Sp.Richness2 266.550  0.802
## 169        Mam.Sp.Richness2  ~        Brillouin.Index2 150.820  0.545
## 179         Prop.Mam.Abdun2  ~             Attendance2   7.091 -0.110
## 180         Prop.Mam.Abdun2  ~          Total.Animals2   6.123 -0.066
## 182         Prop.Mam.Abdun2  ~      Prop.Threat.Abund2   3.466 -0.040
## 193 Mean.Sp.BodyMassXAbund2  ~             Attendance2  41.403 -0.534
## 194 Mean.Sp.BodyMassXAbund2  ~          Total.Animals2  17.953 -0.165
## 196 Mean.Sp.BodyMassXAbund2  ~      Prop.Threat.Abund2   3.293  0.057
## 197 Mean.Sp.BodyMassXAbund2  ~        Brillouin.Index2   3.983  0.071
## 198 Mean.Sp.BodyMassXAbund2  ~        Mean.Raup.Crick2  43.558 -0.210
## 207              X50km_Pop2  ~             Attendance2  20.513  0.387
```

```
## 208             X50km_Pop2  ~         Total.Animals2  11.340  0.124
## 210             X50km_Pop2  ~     Prop.Threat.Abund2   3.347  0.055
## 235           GDP.Millions2  ~            Attendance2  11.654 -0.234
## 237           GDP.Millions2  ~           Sp.Richness2   6.935  0.079
## 240           GDP.Millions2  ~       Mean.Raup.Crick2  13.833 -0.090
## 251        Nat_Pop._WB20152  ~           Sp.Richness2   7.182 -0.077
## 253        Nat_Pop._WB20152  ~       Brillouin.Index2   9.904 -0.082
## 254        Nat_Pop._WB20152  ~       Mean.Raup.Crick2  12.931  0.084
## 263            Prop.Mam.Sp2  ~            Attendance2   8.382  0.103
## 264            Prop.Mam.Sp2  ~         Total.Animals2  15.567  0.112
## 266            Prop.Mam.Sp2  ~     Prop.Threat.Abund2  10.618  0.070
## 268            Prop.Mam.Sp2  ~       Mean.Raup.Crick2   4.260 -0.046
##     sepc.lv sepc.all sepc.nox
## 82    0.191    0.192    0.192
## 84    0.076    0.076    0.076
## 85   -0.531   -0.532   -0.532
## 86    0.110    0.111    0.111
## 93    0.205    0.205    0.205
## 95   -0.079   -0.079   -0.079
## 96   -0.146   -0.146   -0.146
## 97    0.138    0.138    0.138
## 98    0.067    0.067    0.067
## 99   -0.129   -0.129   -0.129
## 100  -0.067   -0.067   -0.067
## 102   0.433    0.424    0.424
## 105   0.235    0.235    0.235
## 106  -0.556   -0.556   -0.556
## 107   0.755    0.755    0.756
## 108  -0.199   -0.199   -0.200
## 110   0.139    0.139    0.139
## 111   0.216    0.216    0.217
## 113  -0.122   -0.122   -0.122
## 114   0.138    0.135    0.135
## 115  -0.128   -0.128   -0.128
## 116  -0.279   -0.279   -0.279
## 120   0.124    0.124    0.125
## 121   0.184    0.184    0.184
## 122   0.159    0.159    0.159
## 123   0.124    0.124    0.125
## 125   0.124    0.124    0.124
## 126   0.226    0.226    0.227
## 128   0.112    0.112    0.112
## 132   0.102    0.102    0.102
## 133   0.087    0.087    0.087
## 135  -0.049   -0.049   -0.049
## 136  -0.194   -0.194   -0.194
## 137  -0.181   -0.181   -0.181
## 138   0.092    0.092    0.092
## 139  -0.258   -0.253   -0.253
## 142  -0.170   -0.170   -0.170
## 143  -0.200   -0.200   -0.200
## 144  -0.403   -0.403   -0.403
## 145  -0.468   -0.468   -0.469
## 146   0.112    0.112    0.112
```

```
## 147   0.094    0.094    0.094
## 149   0.149    0.149    0.149
## 150  -0.545   -0.545   -0.545
## 152  -0.290   -0.290   -0.290
## 153  -0.649   -0.649   -0.649
## 155  -0.354   -0.354   -0.354
## 156   0.085    0.085    0.085
## 165   0.630    0.617    0.617
## 166   0.591    0.591    0.591
## 167   0.802    0.802    0.802
## 169   0.545    0.545    0.545
## 179  -0.110   -0.107   -0.107
## 180  -0.066   -0.066   -0.066
## 182  -0.040   -0.040   -0.040
## 193  -0.534   -0.523   -0.523
## 194  -0.165   -0.165   -0.165
## 196   0.057    0.057    0.057
## 197   0.071    0.071    0.071
## 198  -0.210   -0.210   -0.210
## 207   0.387    0.379    0.379
## 208   0.124    0.124    0.124
## 210   0.055    0.055    0.055
## 235  -0.234   -0.229   -0.229
## 237   0.079    0.079    0.079
## 240  -0.090   -0.090   -0.090
## 251  -0.077   -0.077   -0.077
## 253  -0.082   -0.082   -0.082
## 254   0.084    0.084    0.084
## 263   0.103    0.101    0.101
## 264   0.112    0.112    0.112
## 266   0.070    0.070    0.070
## 268  -0.046   -0.046   -0.046
```

```r
# Adjust for the nested nature of the data (institutions within countries)
# Fit model and generate model summary
design <- svydesign(ids = ~Country, nest=TRUE, data=sem_attendance_data)
fit.adj1A <- lavaan.survey(lavaan.fit = mod.1A.fit, survey.design = design)
summary(fit.adj1A, rsq = TRUE)
```

```
## lavaan (0.5-23.1097) converged normally after  62 iterations
##
##   Number of observations                           458
##
##   Estimator                                         ML      Robust
##   Minimum Function Test Statistic             1213.624     296.332
##   Degrees of freedom                                47          47
##   P-value (Chi-square)                           0.000       0.000
##   Scaling correction factor                                  4.095
##     for the Satorra-Bentler correction
##
## Parameter Estimates:
##
##   Information                                 Expected
##   Standard Errors                           Robust.sem
##
```

```
## Regressions:
##                    Estimate  Std.Err  z-value  P(>|z|)
##   Attendance2 ~
##     Zoo.Area.ha2      0.082    0.042    1.955    0.051
##     Sp.Richness2     -0.280    0.077   -3.612    0.000
##     Total.Animals2    0.548    0.051   10.737    0.000
##     Mam.Sp.Rchnss2    0.146    0.027    5.470    0.000
##     Prop.Mam.Abdn2   -0.100    0.027   -3.635    0.000
##     Prp.Thrt.Abnd2    0.017    0.028    0.615    0.538
##     Mn.Sp.BdyMsXA2    0.324    0.024   13.362    0.000
##     Brilloun.Indx2    0.025    0.058    0.441    0.659
##     Mean.Rap.Crck2    0.116    0.029    3.997    0.000
##     X50km_Pop2        0.073    0.032    2.261    0.024
##     X10km_Pop2        0.417    0.043    9.707    0.000
##     GDP.Millions2     0.263    0.056    4.711    0.000
##     Nt_Pp._WB20152   -0.128    0.063   -2.038    0.042
##   Total.Animals2 ~
##     Zoo.Area.ha2      0.223    0.038    5.856    0.000
##     Sp.Richness2      0.798    0.048   16.678    0.000
##   Sp.Richness2 ~
##     Zoo.Area.ha2      0.363    0.080    4.545    0.000
##     Prop.Mam.Sp2     -0.607    0.062   -9.853    0.000
##   Prop.Threat.Abund2 ~
##     Brilloun.Indx2    0.107    0.132    0.810    0.418
##   Brillouin.Index2 ~
##     Sp.Richness2      1.232    0.068   18.134    0.000
##     Total.Animals2   -0.527    0.078   -6.780    0.000
##   Mean.Raup.Crick2 ~
##     Sp.Richness2     -0.135    0.215   -0.626    0.531
##     Total.Animals2    0.355    0.156    2.277    0.023
##
## Covariances:
##                          Estimate  Std.Err  z-value  P(>|z|)
##   Zoo.Area.ha2 ~~
##     Mam.Sp.Rchnss2          0.381    0.064    5.985    0.000
##     Prop.Mam.Abdn2          0.352    0.092    3.822    0.000
##     Mn.Sp.BdyMsXA2          0.522    0.101    5.177    0.000
##     X50km_Pop2              0.060    0.069    0.880    0.379
##     X10km_Pop2             -0.010    0.078   -0.124    0.901
##     GDP.Millions2          -0.027    0.041   -0.673    0.501
##     Nt_Pp._WB20152          0.061    0.090    0.682    0.495
##     Prop.Mam.Sp2            0.312    0.089    3.510    0.000
##   Mam.Sp.Richness2 ~~
##     Prop.Mam.Abdn2          0.058    0.049    1.172    0.241
##     Mn.Sp.BdyMsXA2          0.227    0.078    2.921    0.003
##     X50km_Pop2              0.204    0.082    2.487    0.013
##     X10km_Pop2              0.284    0.066    4.278    0.000
##     GDP.Millions2          -0.060    0.064   -0.937    0.349
##     Nt_Pp._WB20152         -0.088    0.102   -0.863    0.388
##     Prop.Mam.Sp2            0.088    0.061    1.435    0.151
##   Prop.Mam.Abdun2 ~~
##     Mn.Sp.BdyMsXA2          0.592    0.109    5.445    0.000
##     X50km_Pop2             -0.123    0.054   -2.263    0.024
##     X10km_Pop2             -0.248    0.060   -4.154    0.000
```

```
##     GDP.Millions2            -0.115    0.101   -1.140    0.254
##     Nt_Pp._WB20152           -0.111    0.088   -1.264    0.206
##     Prop.Mam.Sp2              0.878    0.128    6.865    0.000
##   Mean.Sp.BodyMassXAbund2 ~~
##     X50km_Pop2                0.013    0.074    0.174    0.862
##     X10km_Pop2                0.021    0.064    0.329    0.742
##     GDP.Millions2            -0.079    0.038   -2.055    0.040
##     Nt_Pp._WB20152           -0.004    0.084   -0.044    0.965
##     Prop.Mam.Sp2              0.628    0.126    5.002    0.000
##   X50km_Pop2 ~~
##     X10km_Pop2                0.752    0.126    5.982    0.000
##     GDP.Millions2            -0.013    0.100   -0.131    0.896
##     Nt_Pp._WB20152            0.191    0.177    1.083    0.279
##     Prop.Mam.Sp2             -0.096    0.051   -1.888    0.059
##   X10km_Pop2 ~~
##     GDP.Millions2            -0.028    0.056   -0.505    0.613
##     Nt_Pp._WB20152            0.173    0.101    1.701    0.089
##     Prop.Mam.Sp2             -0.204    0.055   -3.685    0.000
##   GDP.Millions2 ~~
##     Nt_Pp._WB20152            0.826    0.202    4.089    0.000
##     Prop.Mam.Sp2             -0.123    0.105   -1.172    0.241
##   Nat_Pop._WB20152 ~~
##     Prop.Mam.Sp2             -0.115    0.091   -1.257    0.209
##
## Intercepts:
##                    Estimate  Std.Err  z-value  P(>|z|)
##    .Attendance2      -0.000    0.030   -0.000    1.000
##    .Total.Animals2   -0.000    0.104   -0.000    1.000
##    .Sp.Richness2      0.000    0.090    0.000    1.000
##    .Prp.Thrt.Abnd2   -0.000    0.077   -0.000    1.000
##    .Brilloun.Indx2   -0.000    0.106   -0.000    1.000
##    .Mean.Rap.Crck2    0.000    0.092    0.000    1.000
##     Zoo.Area.ha2      0.000    0.064    0.000    1.000
##     Mam.Sp.Rchnss2   -0.000    0.079   -0.000    1.000
##     Prop.Mam.Abdn2    0.000    0.113    0.000    1.000
##     Mn.Sp.BdyMsXA2    0.000    0.071    0.000    1.000
##     X50km_Pop2       -0.000    0.114   -0.000    1.000
##     X10km_Pop2        0.000    0.105    0.000    1.000
##     GDP.Millions2    -0.000    0.345   -0.000    1.000
##     Nt_Pp._WB20152    0.000    0.295    0.000    1.000
##     Prop.Mam.Sp2      0.000    0.118    0.000    1.000
##
## Variances:
##                    Estimate  Std.Err  z-value  P(>|z|)
##    .Attendance2       0.270    0.028    9.568    0.000
##    .Total.Animals2    0.251    0.044    5.729    0.000
##    .Sp.Richness2      0.636    0.144    4.407    0.000
##    .Prp.Thrt.Abnd2    0.986    0.133    7.389    0.000
##    .Brilloun.Indx2    0.290    0.079    3.656    0.000
##    .Mean.Rap.Crck2    0.934    0.100    9.311    0.000
##     Zoo.Area.ha2      0.998    0.112    8.886    0.000
##     Mam.Sp.Rchnss2    0.998    0.115    8.669    0.000
##     Prop.Mam.Abdn2    0.998    0.121    8.221    0.000
##     Mn.Sp.BdyMsXA2    0.998    0.160    6.219    0.000
```

```
##     X50km_Pop2        0.998    0.131    7.623    0.000
##     X10km_Pop2        0.998    0.174    5.745    0.000
##     GDP.Millions2     0.998    0.246    4.061    0.000
##     Nt_Pp._WB20152    0.998    0.225    4.439    0.000
##     Prop.Mam.Sp2      0.998    0.150    6.662    0.000
##
## R-Square:
##                    Estimate
##     Attendance2       0.718
##     Total.Animals2    0.748
##     Sp.Richness2      0.363
##     Prp.Thrt.Abnd2    0.012
##     Brilloun.Indx2    0.709
##     Mean.Rap.Crck2    0.064
```

```r
# Generate fit indices
fitMeasures(fit.adj1A, c("agfi", "rmr", "srmr", "rmsea", "cfi", "nnfi", "tli"))
```

```
##  agfi   rmr  srmr rmsea   cfi  nnfi   tli
## 0.466 0.137 0.129 0.233 0.633 0.462 0.462
```

```r
# Generate modification indices
mi1adjA <- modindices(fit.adj1A)
print(mi1adjA[mi1adjA$mi > 3.0,])
```

```
##                       lhs op                    rhs      mi mi.scaled
## 97         Total.Animals2 ~~        Mean.Raup.Crick2  11.525     2.814
## 99           Sp.Richness2 ~~       Brillouin.Index2   5.179     1.264
## 100          Sp.Richness2 ~~        Mean.Raup.Crick2  78.108    19.072
## 101      Prop.Threat.Abund2 ~~     Brillouin.Index2  13.812     3.373
## 108        Total.Animals2  ~        Mean.Raup.Crick2  11.525     2.814
## 110        Total.Animals2  ~         Prop.Mam.Abdun2   7.409     1.809
## 111        Total.Animals2  ~ Mean.Sp.BodyMassXAbund2  24.990     6.102
## 112        Total.Animals2  ~              X50km_Pop2  34.446     8.411
## 113        Total.Animals2  ~              X10km_Pop2   7.972     1.946
## 114        Total.Animals2  ~           GDP.Millions2  30.032     7.333
## 115        Total.Animals2  ~        Nat_Pop._WB20152   8.055     1.967
## 117          Sp.Richness2  ~             Attendance2  57.923    14.143
## 120          Sp.Richness2  ~        Brillouin.Index2   5.103     1.246
## 121          Sp.Richness2  ~        Mean.Raup.Crick2  77.140    18.835
## 122          Sp.Richness2  ~        Mam.Sp.Richness2 349.341    85.299
## 123          Sp.Richness2  ~         Prop.Mam.Abdun2   6.273     1.532
## 125          Sp.Richness2  ~              X50km_Pop2  13.582     3.316
## 126          Sp.Richness2  ~              X10km_Pop2  32.091     7.836
## 128          Sp.Richness2  ~        Nat_Pop._WB20152  10.411     2.542
## 129      Prop.Threat.Abund2  ~             Attendance2   5.929     1.448
## 130      Prop.Threat.Abund2  ~          Total.Animals2   5.628     1.374
## 131      Prop.Threat.Abund2  ~            Sp.Richness2  13.491     3.294
## 135      Prop.Threat.Abund2  ~         Prop.Mam.Abdun2   6.180     1.509
## 136      Prop.Threat.Abund2  ~ Mean.Sp.BodyMassXAbund2  14.929     3.645
## 137      Prop.Threat.Abund2  ~              X50km_Pop2  11.699     2.857
## 138      Prop.Threat.Abund2  ~              X10km_Pop2   7.106     1.735
## 140      Prop.Threat.Abund2  ~        Nat_Pop._WB20152   7.100     1.734
## 141      Prop.Threat.Abund2  ~            Prop.Mam.Sp2  19.197     4.687
## 143        Brillouin.Index2  ~      Prop.Threat.Abund2  13.812     3.373
## 147        Brillouin.Index2  ~         Prop.Mam.Abdun2  13.234     3.231
```

```
## 148        Brillouin.Index2  ~ Mean.Sp.BodyMassXAbund2  10.854     2.650
## 150        Brillouin.Index2  ~             X10km_Pop2   3.677     0.898
## 151        Brillouin.Index2  ~           GDP.Millions2  58.745    14.344
## 152        Brillouin.Index2  ~       Nat_Pop._WB20152  51.006    12.454
## 153        Brillouin.Index2  ~           Prop.Mam.Sp2   9.791     2.391
## 154        Mean.Raup.Crick2  ~            Attendance2  13.735     3.354
## 157        Mean.Raup.Crick2  ~            Zoo.Area.ha2  11.525     2.814
## 158        Mean.Raup.Crick2  ~       Mam.Sp.Richness2  18.995     4.638
## 159        Mean.Raup.Crick2  ~        Prop.Mam.Abdun2  63.958    15.617
## 160        Mean.Raup.Crick2  ~ Mean.Sp.BodyMassXAbund2  97.877    23.899
## 161        Mean.Raup.Crick2  ~             X50km_Pop2   6.052     1.478
## 162        Mean.Raup.Crick2  ~             X10km_Pop2   4.241     1.036
## 164        Mean.Raup.Crick2  ~       Nat_Pop._WB20152  10.765     2.629
## 165        Mean.Raup.Crick2  ~           Prop.Mam.Sp2 106.001    25.883
## 167            Zoo.Area.ha2  ~          Total.Animals2  15.539     3.794
## 168            Zoo.Area.ha2  ~           Sp.Richness2  71.870    17.548
## 170            Zoo.Area.ha2  ~       Brillouin.Index2  40.397     9.864
## 171            Zoo.Area.ha2  ~       Mean.Raup.Crick2   4.521     1.104
## 180        Mam.Sp.Richness2  ~            Attendance2  31.541     7.701
## 181        Mam.Sp.Richness2  ~          Total.Animals2 149.498    36.503
## 182        Mam.Sp.Richness2  ~           Sp.Richness2 266.550    65.084
## 184        Mam.Sp.Richness2  ~       Brillouin.Index2 150.819    36.826
## 194         Prop.Mam.Abdun2  ~            Attendance2   7.092     1.732
## 195         Prop.Mam.Abdun2  ~          Total.Animals2   6.123     1.495
## 197         Prop.Mam.Abdun2  ~      Prop.Threat.Abund2   3.466     0.846
## 208 Mean.Sp.BodyMassXAbund2  ~            Attendance2  41.403    10.109
## 209 Mean.Sp.BodyMassXAbund2  ~          Total.Animals2  17.953     4.384
## 211 Mean.Sp.BodyMassXAbund2  ~      Prop.Threat.Abund2   3.293     0.804
## 212 Mean.Sp.BodyMassXAbund2  ~       Brillouin.Index2   3.983     0.973
## 213 Mean.Sp.BodyMassXAbund2  ~       Mean.Raup.Crick2  43.558    10.636
## 222              X50km_Pop2  ~            Attendance2  20.513     5.009
## 223              X50km_Pop2  ~          Total.Animals2  11.339     2.769
## 225              X50km_Pop2  ~      Prop.Threat.Abund2   3.347     0.817
## 250           GDP.Millions2  ~            Attendance2  11.654     2.846
## 252           GDP.Millions2  ~           Sp.Richness2   6.935     1.693
## 255           GDP.Millions2  ~       Mean.Raup.Crick2  13.833     3.378
## 266        Nat_Pop._WB20152  ~           Sp.Richness2   7.182     1.754
## 268        Nat_Pop._WB20152  ~       Brillouin.Index2   9.904     2.418
## 269        Nat_Pop._WB20152  ~       Mean.Raup.Crick2  12.931     3.157
## 278            Prop.Mam.Sp2  ~            Attendance2   8.382     2.047
## 279            Prop.Mam.Sp2  ~          Total.Animals2  15.567     3.801
## 281            Prop.Mam.Sp2  ~      Prop.Threat.Abund2  10.618     2.593
## 283            Prop.Mam.Sp2  ~       Mean.Raup.Crick2   4.260     1.040
##        epc sepc.lv sepc.all sepc.nox
## 97   0.191   0.191    0.192    0.192
## 99   0.076   0.076    0.076    0.076
## 100 -0.531  -0.531   -0.532   -0.532
## 101  0.110   0.110    0.111    0.111
## 108  0.205   0.205    0.205    0.205
## 110 -0.079  -0.079   -0.079   -0.079
## 111 -0.146  -0.146   -0.146   -0.146
## 112  0.138   0.138    0.138    0.138
## 113  0.067   0.067    0.067    0.067
## 114 -0.129  -0.129   -0.129   -0.129
```

```
## 115 -0.067  -0.067   -0.067   -0.067
## 117  0.433   0.433    0.424    0.424
## 120  0.235   0.235    0.235    0.235
## 121 -0.556  -0.556   -0.556   -0.556
## 122  0.755   0.755    0.755    0.756
## 123 -0.199  -0.199   -0.199   -0.200
## 125  0.139   0.139    0.139    0.139
## 126  0.216   0.216    0.216    0.217
## 128 -0.122  -0.122   -0.122   -0.122
## 129  0.138   0.138    0.135    0.135
## 130 -0.128  -0.128   -0.128   -0.128
## 131 -0.279  -0.279   -0.279   -0.279
## 135  0.124   0.124    0.124    0.125
## 136  0.184   0.184    0.184    0.184
## 137  0.159   0.159    0.159    0.159
## 138  0.124   0.124    0.124    0.125
## 140  0.124   0.124    0.124    0.124
## 141  0.226   0.226    0.226    0.227
## 143  0.112   0.112    0.112    0.112
## 147  0.102   0.102    0.102    0.102
## 148  0.087   0.087    0.087    0.087
## 150 -0.049  -0.049   -0.049   -0.049
## 151 -0.194  -0.194   -0.194   -0.194
## 152 -0.181  -0.181   -0.181   -0.181
## 153  0.092   0.092    0.092    0.092
## 154 -0.258  -0.258   -0.253   -0.253
## 157 -0.170  -0.170   -0.170   -0.170
## 158 -0.200  -0.200   -0.200   -0.200
## 159 -0.403  -0.403   -0.403   -0.403
## 160 -0.468  -0.468   -0.468   -0.469
## 161  0.112   0.112    0.112    0.112
## 162  0.094   0.094    0.094    0.094
## 164  0.149   0.149    0.149    0.149
## 165 -0.545  -0.545   -0.545   -0.545
## 167 -0.290  -0.290   -0.290   -0.290
## 168 -0.649  -0.649   -0.649   -0.649
## 170 -0.354  -0.354   -0.354   -0.354
## 171  0.085   0.085    0.085    0.085
## 180  0.630   0.630    0.617    0.617
## 181  0.591   0.591    0.591    0.591
## 182  0.802   0.802    0.802    0.802
## 184  0.545   0.545    0.545    0.545
## 194 -0.110  -0.110   -0.107   -0.107
## 195 -0.066  -0.066   -0.066   -0.066
## 197 -0.040  -0.040   -0.040   -0.040
## 208 -0.534  -0.534   -0.523   -0.523
## 209 -0.165  -0.165   -0.165   -0.165
## 211  0.057   0.057    0.057    0.057
## 212  0.071   0.071    0.071    0.071
## 213 -0.210  -0.210   -0.210   -0.210
## 222  0.387   0.387    0.379    0.379
## 223  0.124   0.124    0.124    0.124
## 225  0.055   0.055    0.055    0.055
## 250 -0.234  -0.234   -0.229   -0.229
```

```
## 252  0.079   0.079    0.079    0.079
## 255 -0.090  -0.090   -0.090   -0.090
## 266 -0.077  -0.077   -0.077   -0.077
## 268 -0.082  -0.082   -0.082   -0.082
## 269  0.084   0.084    0.084    0.084
## 278  0.103   0.103    0.101    0.101
## 279  0.112   0.112    0.112    0.112
## 281  0.070   0.070    0.070    0.070
## 283 -0.046  -0.046   -0.046   -0.046
```

Model 2

Based on the modification indices generated from the first model, we can see that **Sp.Richness2** ~ **Mam.Sp.Richness2** has the highest mi value of **349.340**. This far exceeds the standard cut-off level for the chi-square test criterion of 3.84 (Burnham and Anderson, 2002). This is also one of the most intuitive relationships, as if we increase mammal species richness, then of course we are going to increase overall species richness simulataneously. As a result, we add this relationship to our model. Once again, the model summary, fit indices and modification indices were all generated for the model, adjusting for the nested nature of data.

```r
# Attendance SEM (Species Abundance)

# Model 2
# Addition of Sp.Richness2 ~ Mam.Sp.Richness2, mi = 349.342

mod.2A <- 'Attendance2 ~ Zoo.Area.ha2 + Sp.Richness2 + Total.Animals2
+ Mam.Sp.Richness2 + Prop.Mam.Abdun2 + Prop.Threat.Abund2
+ Mean.Sp.BodyMassXAbund2 + Brillouin.Index2 + Mean.Raup.Crick2
+ X50km_Pop2 + X10km_Pop2 + GDP.Millions2 + Nat_Pop._WB20152

Total.Animals2 ~ Zoo.Area.ha2 + Sp.Richness2
Sp.Richness2 ~ Zoo.Area.ha2 + Prop.Mam.Sp2 + Mam.Sp.Richness2
Prop.Threat.Abund2 ~ Brillouin.Index2
Brillouin.Index2 ~ Sp.Richness2 + Total.Animals2
Mean.Raup.Crick2 ~ Sp.Richness2 + Total.Animals2'

# Fit model and generate model summary
mod.2A.fit <- sem(mod.2A, data = sem_attendance_data, fixed.x=FALSE)
summary(mod.2A.fit, rsq = TRUE)
```

```
## lavaan (0.5-23.1097) converged normally after  63 iterations
##
##   Number of observations                           458
##
##   Estimator                                         ML
##   Minimum Function Test Statistic              554.721
##   Degrees of freedom                                46
##   P-value (Chi-square)                           0.000
##
## Parameter Estimates:
##
##   Information                                 Expected
##   Standard Errors                             Standard
##
## Regressions:
```

```
##                       Estimate  Std.Err  z-value  P(>|z|)
##   Attendance2 ~
##     Zoo.Area.ha2         0.082    0.034    2.450    0.014
##     Sp.Richness2        -0.280    0.085   -3.277    0.001
##     Total.Animals2       0.548    0.055   10.026    0.000
##     Mam.Sp.Rchnss2       0.146    0.048    3.058    0.002
##     Prop.Mam.Abdn2      -0.100    0.040   -2.495    0.013
##     Prp.Thrt.Abnd2       0.017    0.024    0.700    0.484
##     Mn.Sp.BdyMsXA2       0.324    0.035    9.301    0.000
##     Brilloun.Indx2       0.025    0.045    0.563    0.573
##     Mean.Rap.Crck2       0.116    0.025    4.612    0.000
##     X50km_Pop2           0.073    0.038    1.911    0.056
##     X10km_Pop2           0.417    0.041   10.141    0.000
##     GDP.Millions2        0.263    0.048    5.532    0.000
##     Nt_Pp._WB20152      -0.128    0.049   -2.603    0.009
##   Total.Animals2 ~
##     Zoo.Area.ha2         0.223    0.024    9.384    0.000
##     Sp.Richness2         0.798    0.024   33.520    0.000
##   Sp.Richness2 ~
##     Zoo.Area.ha2         0.067    0.021    3.225    0.001
##     Prop.Mam.Sp2        -0.581    0.019  -30.364    0.000
##     Mam.Sp.Rchnss2       0.755    0.020   38.373    0.000
##   Prop.Threat.Abund2 ~
##     Brilloun.Indx2       0.107    0.046    2.311    0.021
##   Brillouin.Index2 ~
##     Sp.Richness2         1.232    0.046   26.770    0.000
##     Total.Animals2      -0.527    0.046  -11.451    0.000
##   Mean.Raup.Crick2 ~
##     Sp.Richness2        -0.135    0.083   -1.636    0.102
##     Total.Animals2       0.355    0.083    4.302    0.000
##
## Covariances:
##                             Estimate  Std.Err  z-value  P(>|z|)
##   Zoo.Area.ha2 ~~
##     Mam.Sp.Rchnss2             0.381    0.050    7.634    0.000
##     Prop.Mam.Abdn2             0.352    0.049    7.121    0.000
##     Mn.Sp.BdyMsXA2             0.522    0.053    9.923    0.000
##     X50km_Pop2                 0.060    0.047    1.291    0.197
##     X10km_Pop2                -0.010    0.047   -0.208    0.835
##     GDP.Millions2             -0.027    0.047   -0.588    0.557
##     Nt_Pp._WB20152             0.061    0.047    1.310    0.190
##     Prop.Mam.Sp2               0.312    0.049    6.389    0.000
##   Mam.Sp.Richness2 ~~
##     Prop.Mam.Abdn2             0.058    0.047    1.237    0.216
##     Mn.Sp.BdyMsXA2             0.227    0.048    4.750    0.000
##     X50km_Pop2                 0.204    0.048    4.281    0.000
##     X10km_Pop2                 0.284    0.048    5.852    0.000
##     GDP.Millions2             -0.060    0.047   -1.277    0.202
##     Nt_Pp._WB20152            -0.088    0.047   -1.881    0.060
##     Prop.Mam.Sp2               0.088    0.047    1.882    0.060
##   Prop.Mam.Abdun2 ~~
##     Mn.Sp.BdyMsXA2             0.592    0.054   10.922    0.000
##     X50km_Pop2                -0.123    0.047   -2.622    0.009
##     X10km_Pop2                -0.248    0.048   -5.165    0.000
```

```
##     GDP.Millions2              -0.115    0.047   -2.458    0.014
##     Nt_Pp._WB20152             -0.111    0.047   -2.362    0.018
##     Prop.Mam.Sp2                0.878    0.062   14.135    0.000
##   Mean.Sp.BodyMassXAbund2 ~~
##     X50km_Pop2                  0.013    0.047    0.275    0.784
##     X10km_Pop2                  0.021    0.047    0.455    0.649
##     GDP.Millions2              -0.079    0.047   -1.682    0.093
##     Nt_Pp._WB20152             -0.004    0.047   -0.079    0.937
##     Prop.Mam.Sp2                0.628    0.055   11.405    0.000
##   X50km_Pop2 ~~
##     X10km_Pop2                  0.752    0.058   12.886    0.000
##     GDP.Millions2              -0.013    0.047   -0.281    0.779
##     Nt_Pp._WB20152              0.191    0.047    4.033    0.000
##     Prop.Mam.Sp2               -0.096    0.047   -2.054    0.040
##   X10km_Pop2 ~~
##     GDP.Millions2              -0.028    0.047   -0.604    0.546
##     Nt_Pp._WB20152              0.173    0.047    3.646    0.000
##     Prop.Mam.Sp2               -0.204    0.048   -4.293    0.000
##   GDP.Millions2 ~~
##     Nt_Pp._WB20152              0.826    0.061   13.642    0.000
##     Prop.Mam.Sp2               -0.123    0.047   -2.615    0.009
##   Nat_Pop._WB20152 ~~
##     Prop.Mam.Sp2               -0.115    0.047   -2.447    0.014
##
## Variances:
##                    Estimate  Std.Err  z-value  P(>|z|)
##    .Attendance2       0.270    0.018   15.133    0.000
##    .Total.Animals2    0.251    0.017   15.133    0.000
##    .Sp.Richness2      0.151    0.010   15.133    0.000
##    .Prp.Thrt.Abnd2    0.986    0.065   15.133    0.000
##    .Brilloun.Indx2    0.290    0.019   15.133    0.000
##    .Mean.Rap.Crck2    0.934    0.062   15.133    0.000
##     Zoo.Area.ha2      0.998    0.066   15.133    0.000
##     Mam.Sp.Rchnss2    0.998    0.066   15.133    0.000
##     Prop.Mam.Abdn2    0.998    0.066   15.133    0.000
##     Mn.Sp.BdyMsXA2    0.998    0.066   15.133    0.000
##     X50km_Pop2        0.998    0.066   15.133    0.000
##     X10km_Pop2        0.998    0.066   15.133    0.000
##     GDP.Millions2     0.998    0.066   15.133    0.000
##     Nt_Pp._WB20152    0.998    0.066   15.133    0.000
##     Prop.Mam.Sp2      0.998    0.066   15.133    0.000
##
## R-Square:
##                    Estimate
##     Attendance2       0.740
##     Total.Animals2    0.748
##     Sp.Richness2      0.849
##     Prp.Thrt.Abnd2    0.012
##     Brilloun.Indx2    0.709
##     Mean.Rap.Crck2    0.064
```

```r
# Generate fit indices
fitMeasures(mod.2A.fit, c("agfi", "rmr", "srmr", "rmsea", "cfi", "nnfi", "tli"))
```

```
##  agfi   rmr  srmr rmsea   cfi  nnfi   tli
```

```
## 0.689 0.090 0.090 0.155 0.840 0.760 0.760
```

```r
# Generate modification indices
mi2A <- modindices(mod.2A.fit)
print(mi2A[mi2A$mi > 3.0,])
```

```
##                        lhs op                     rhs      mi    epc
## 83           Total.Animals2 ~~        Mean.Raup.Crick2  11.525  0.191
## 85            Sp.Richness2 ~~        Brillouin.Index2   8.649  0.031
## 86            Sp.Richness2 ~~        Mean.Raup.Crick2  21.687 -0.089
## 87       Prop.Threat.Abund2 ~~       Brillouin.Index2  13.812  0.110
## 94           Total.Animals2  ~        Mean.Raup.Crick2  11.525  0.205
## 96           Total.Animals2  ~         Prop.Mam.Abdun2   7.869 -0.084
## 97           Total.Animals2  ~ Mean.Sp.BodyMassXAbund2  24.303 -0.142
## 98           Total.Animals2  ~             X50km_Pop2  35.809  0.144
## 99           Total.Animals2  ~             X10km_Pop2   8.873  0.074
## 100          Total.Animals2  ~           GDP.Millions2  29.909 -0.128
## 101          Total.Animals2  ~        Nat_Pop._WB20152   8.000 -0.066
## 106           Sp.Richness2  ~        Brillouin.Index2   6.727  0.085
## 107           Sp.Richness2  ~        Mean.Raup.Crick2  20.798 -0.091
## 109           Sp.Richness2  ~ Mean.Sp.BodyMassXAbund2   9.674 -0.081
## 113           Sp.Richness2  ~        Nat_Pop._WB20152   3.059 -0.032
## 114      Prop.Threat.Abund2  ~            Attendance2   5.798  0.135
## 115      Prop.Threat.Abund2  ~          Total.Animals2   5.628 -0.128
## 116      Prop.Threat.Abund2  ~            Sp.Richness2  13.491 -0.279
## 120      Prop.Threat.Abund2  ~         Prop.Mam.Abdun2   6.355  0.128
## 121      Prop.Threat.Abund2  ~ Mean.Sp.BodyMassXAbund2  14.759  0.182
## 122      Prop.Threat.Abund2  ~             X50km_Pop2  11.993  0.163
## 123      Prop.Threat.Abund2  ~             X10km_Pop2   7.595  0.133
## 125      Prop.Threat.Abund2  ~        Nat_Pop._WB20152   7.068  0.124
## 126      Prop.Threat.Abund2  ~            Prop.Mam.Sp2  19.197  0.226
## 127        Brillouin.Index2  ~            Attendance2   3.315 -0.076
## 128        Brillouin.Index2  ~      Prop.Threat.Abund2  13.812  0.112
## 132        Brillouin.Index2  ~         Prop.Mam.Abdun2  13.803  0.107
## 133        Brillouin.Index2  ~ Mean.Sp.BodyMassXAbund2  10.704  0.086
## 135        Brillouin.Index2  ~             X10km_Pop2   4.078 -0.054
## 136        Brillouin.Index2  ~           GDP.Millions2  58.529 -0.193
## 137        Brillouin.Index2  ~        Nat_Pop._WB20152  50.588 -0.179
## 138        Brillouin.Index2  ~            Prop.Mam.Sp2   9.791  0.092
## 139        Mean.Raup.Crick2  ~            Attendance2  16.366 -0.307
## 142        Mean.Raup.Crick2  ~            Zoo.Area.ha2  11.525 -0.170
## 143        Mean.Raup.Crick2  ~        Mam.Sp.Richness2  40.269 -0.424
## 144        Mean.Raup.Crick2  ~         Prop.Mam.Abdun2  66.708 -0.420
## 145        Mean.Raup.Crick2  ~ Mean.Sp.BodyMassXAbund2  96.529 -0.462
## 146        Mean.Raup.Crick2  ~             X50km_Pop2   6.300  0.116
## 147        Mean.Raup.Crick2  ~             X10km_Pop2   4.704  0.104
## 149        Mean.Raup.Crick2  ~        Nat_Pop._WB20152  10.677  0.148
## 150        Mean.Raup.Crick2  ~            Prop.Mam.Sp2 106.002 -0.545
## 151            Zoo.Area.ha2  ~            Attendance2  28.193  0.848
## 152            Zoo.Area.ha2  ~          Total.Animals2  31.172  0.569
## 153            Zoo.Area.ha2  ~            Sp.Richness2  22.368  0.894
## 156            Zoo.Area.ha2  ~        Mean.Raup.Crick2   8.032  0.110
## 165        Mam.Sp.Richness2  ~            Attendance2   8.657 -0.477
## 166        Mam.Sp.Richness2  ~          Total.Animals2   4.984 -0.216
## 167        Mam.Sp.Richness2  ~            Sp.Richness2  10.502 -0.783
```

```
## 170        Mam.Sp.Richness2  ~        Mean.Raup.Crick2  12.796 -0.185
## 179          Prop.Mam.Abdun2  ~             Attendance2   8.839 -0.137
## 180          Prop.Mam.Abdun2  ~          Total.Animals2  11.568 -0.124
## 182          Prop.Mam.Abdun2  ~      Prop.Threat.Abund2   3.479 -0.040
## 183          Prop.Mam.Abdun2  ~        Brillouin.Index2   4.471  0.067
## 193 Mean.Sp.BodyMassXAbund2  ~             Attendance2  49.773 -0.642
## 194 Mean.Sp.BodyMassXAbund2  ~          Total.Animals2  33.919 -0.311
## 195 Mean.Sp.BodyMassXAbund2  ~            Sp.Richness2   7.245 -0.218
## 196 Mean.Sp.BodyMassXAbund2  ~      Prop.Threat.Abund2   3.305  0.058
## 197 Mean.Sp.BodyMassXAbund2  ~        Brillouin.Index2   6.752  0.121
## 198 Mean.Sp.BodyMassXAbund2  ~        Mean.Raup.Crick2  44.038 -0.212
## 207              X50km_Pop2  ~             Attendance2  24.319  0.459
## 208              X50km_Pop2  ~          Total.Animals2  21.424  0.234
## 210              X50km_Pop2  ~      Prop.Threat.Abund2   3.359  0.055
## 235           GDP.Millions2  ~             Attendance2  13.810 -0.277
## 237           GDP.Millions2  ~            Sp.Richness2  29.232  0.332
## 239           GDP.Millions2  ~        Brillouin.Index2   3.643  0.067
## 240           GDP.Millions2  ~        Mean.Raup.Crick2  13.985 -0.091
## 251        Nat_Pop._WB20152  ~            Sp.Richness2  30.273 -0.326
## 253        Nat_Pop._WB20152  ~        Brillouin.Index2  16.787 -0.139
## 254        Nat_Pop._WB20152  ~        Mean.Raup.Crick2  13.073  0.085
## 263             Prop.Mam.Sp2  ~             Attendance2   9.878  0.125
## 264             Prop.Mam.Sp2  ~          Total.Animals2  23.928  0.185
## 266             Prop.Mam.Sp2  ~      Prop.Threat.Abund2  10.458  0.070
## 267             Prop.Mam.Sp2  ~        Brillouin.Index2   5.246 -0.075
## 268             Prop.Mam.Sp2  ~        Mean.Raup.Crick2   4.433 -0.047
##     sepc.lv sepc.all sepc.nox
## 83    0.191    0.192    0.192
## 85    0.031    0.031    0.031
## 86   -0.089   -0.089   -0.089
## 87    0.110    0.111    0.111
## 94    0.205    0.205    0.205
## 96   -0.084   -0.084   -0.084
## 97   -0.142   -0.142   -0.142
## 98    0.144    0.144    0.144
## 99    0.074    0.074    0.074
## 100  -0.128   -0.128   -0.128
## 101  -0.066   -0.066   -0.067
## 106   0.085    0.085    0.085
## 107  -0.091   -0.091   -0.091
## 109  -0.081   -0.081   -0.081
## 113  -0.032   -0.032   -0.032
## 114   0.135    0.137    0.137
## 115  -0.128   -0.128   -0.128
## 116  -0.279   -0.279   -0.279
## 120   0.128    0.128    0.128
## 121   0.182    0.182    0.182
## 122   0.163    0.163    0.163
## 123   0.133    0.133    0.133
## 125   0.124    0.124    0.124
## 126   0.226    0.226    0.227
## 127  -0.076   -0.077   -0.077
## 128   0.112    0.112    0.112
## 132   0.107    0.107    0.107
```

```
## 133   0.086    0.086    0.086
## 135  -0.054   -0.054   -0.054
## 136  -0.193   -0.193   -0.193
## 137  -0.179   -0.179   -0.180
## 138   0.092    0.092    0.092
## 139  -0.307   -0.314   -0.314
## 142  -0.170   -0.170   -0.170
## 143  -0.424   -0.424   -0.424
## 144  -0.420   -0.420   -0.421
## 145  -0.462   -0.462   -0.463
## 146   0.116    0.116    0.116
## 147   0.104    0.104    0.104
## 149   0.148    0.148    0.148
## 150  -0.545   -0.545   -0.545
## 151   0.848    0.865    0.865
## 152   0.569    0.569    0.569
## 153   0.894    0.894    0.894
## 156   0.110    0.110    0.110
## 165  -0.477   -0.487   -0.487
## 166  -0.216   -0.216   -0.216
## 167  -0.783   -0.783   -0.783
## 170  -0.185   -0.185   -0.185
## 179  -0.137   -0.139   -0.139
## 180  -0.124   -0.124   -0.124
## 182  -0.040   -0.040   -0.040
## 183   0.067    0.067    0.067
## 193  -0.642   -0.655   -0.655
## 194  -0.311   -0.311   -0.311
## 195  -0.218   -0.218   -0.218
## 196   0.058    0.058    0.058
## 197   0.121    0.121    0.121
## 198  -0.212   -0.212   -0.212
## 207   0.459    0.468    0.468
## 208   0.234    0.234    0.234
## 210   0.055    0.055    0.055
## 235  -0.277   -0.283   -0.283
## 237   0.332    0.332    0.332
## 239   0.067    0.067    0.067
## 240  -0.091   -0.091   -0.091
## 251  -0.326   -0.326   -0.326
## 253  -0.139   -0.139   -0.139
## 254   0.085    0.085    0.085
## 263   0.125    0.127    0.127
## 264   0.185    0.185    0.185
## 266   0.070    0.070    0.070
## 267  -0.075   -0.075   -0.075
## 268  -0.047   -0.047   -0.047
```

```r
# Adjust for the nested nature of the data (institutions within countries)
# Fit model and generate model summary
design <- svydesign(ids = ~Country, nest=TRUE, data=sem_attendance_data)
fit.adj2A <- lavaan.survey(lavaan.fit = mod.2A.fit, survey.design = design)
summary(fit.adj2A, rsq = TRUE)
```

```
## lavaan (0.5-23.1097) converged normally after  68 iterations
```

```
##
##   Number of observations                           458
##
##   Estimator                                         ML      Robust
##   Minimum Function Test Statistic              554.721     152.432
##   Degrees of freedom                                46          46
##   P-value (Chi-square)                           0.000       0.000
##   Scaling correction factor                                  3.639
##     for the Satorra-Bentler correction
##
## Parameter Estimates:
##
##   Information                                 Expected
##   Standard Errors                           Robust.sem
##
## Regressions:
##                        Estimate  Std.Err  z-value  P(>|z|)
##   Attendance2 ~
##     Zoo.Area.ha2          0.082    0.039    2.104    0.035
##     Sp.Richness2         -0.280    0.075   -3.714    0.000
##     Total.Animals2        0.548    0.051   10.737    0.000
##     Mam.Sp.Rchnss2        0.146    0.042    3.447    0.001
##     Prop.Mam.Abdn2       -0.100    0.030   -3.308    0.001
##     Prp.Thrt.Abnd2        0.017    0.028    0.615    0.538
##     Mn.Sp.BdyMsXA2        0.324    0.024   13.526    0.000
##     Brilloun.Indx2        0.025    0.058    0.441    0.659
##     Mean.Rap.Crck2        0.116    0.029    3.997    0.000
##     X50km_Pop2            0.073    0.032    2.257    0.024
##     X10km_Pop2            0.417    0.043    9.718    0.000
##     GDP.Millions2         0.263    0.056    4.704    0.000
##     Nt_Pp._WB20152       -0.128    0.063   -2.039    0.041
##   Total.Animals2 ~
##     Zoo.Area.ha2          0.223    0.038    5.856    0.000
##     Sp.Richness2          0.798    0.048   16.678    0.000
##   Sp.Richness2 ~
##     Zoo.Area.ha2          0.067    0.044    1.519    0.129
##     Prop.Mam.Sp2         -0.581    0.038  -15.231    0.000
##     Mam.Sp.Rchnss2        0.755    0.068   11.066    0.000
##   Prop.Threat.Abund2 ~
##     Brilloun.Indx2        0.107    0.132    0.810    0.418
##   Brillouin.Index2 ~
##     Sp.Richness2          1.232    0.068   18.134    0.000
##     Total.Animals2       -0.527    0.078   -6.780    0.000
##   Mean.Raup.Crick2 ~
##     Sp.Richness2         -0.135    0.215   -0.626    0.531
##     Total.Animals2        0.355    0.156    2.277    0.023
##
## Covariances:
##                              Estimate  Std.Err  z-value  P(>|z|)
##   Zoo.Area.ha2 ~~
##     Mam.Sp.Rchnss2              0.381    0.064    5.985    0.000
##     Prop.Mam.Abdn2              0.352    0.092    3.822    0.000
##     Mn.Sp.BdyMsXA2              0.522    0.101    5.177    0.000
##     X50km_Pop2                  0.060    0.069    0.880    0.379
```

```
##     X10km_Pop2              -0.010    0.078   -0.124    0.901
##     GDP.Millions2           -0.027    0.041   -0.673    0.501
##     Nt_Pp._WB20152           0.061    0.090    0.682    0.495
##     Prop.Mam.Sp2             0.312    0.089    3.510    0.000
##   Mam.Sp.Richness2 ~~
##     Prop.Mam.Abdn2           0.058    0.049    1.172    0.241
##     Mn.Sp.BdyMsXA2           0.227    0.078    2.921    0.003
##     X50km_Pop2               0.204    0.082    2.487    0.013
##     X10km_Pop2               0.284    0.066    4.278    0.000
##     GDP.Millions2           -0.060    0.064   -0.937    0.349
##     Nt_Pp._WB20152          -0.088    0.102   -0.863    0.388
##     Prop.Mam.Sp2             0.088    0.061    1.435    0.151
##   Prop.Mam.Abdun2 ~~
##     Mn.Sp.BdyMsXA2           0.592    0.109    5.445    0.000
##     X50km_Pop2              -0.123    0.054   -2.263    0.024
##     X10km_Pop2              -0.248    0.060   -4.154    0.000
##     GDP.Millions2           -0.115    0.101   -1.140    0.254
##     Nt_Pp._WB20152          -0.111    0.088   -1.264    0.206
##     Prop.Mam.Sp2             0.878    0.128    6.865    0.000
##   Mean.Sp.BodyMassXAbund2 ~~
##     X50km_Pop2               0.013    0.074    0.174    0.862
##     X10km_Pop2               0.021    0.064    0.329    0.742
##     GDP.Millions2           -0.079    0.038   -2.055    0.040
##     Nt_Pp._WB20152          -0.004    0.084   -0.044    0.965
##     Prop.Mam.Sp2             0.628    0.126    5.002    0.000
##   X50km_Pop2 ~~
##     X10km_Pop2               0.752    0.126    5.982    0.000
##     GDP.Millions2           -0.013    0.100   -0.131    0.896
##     Nt_Pp._WB20152           0.191    0.177    1.083    0.279
##     Prop.Mam.Sp2            -0.096    0.051   -1.888    0.059
##   X10km_Pop2 ~~
##     GDP.Millions2           -0.028    0.056   -0.505    0.613
##     Nt_Pp._WB20152           0.173    0.101    1.701    0.089
##     Prop.Mam.Sp2            -0.204    0.055   -3.685    0.000
##   GDP.Millions2 ~~
##     Nt_Pp._WB20152           0.826    0.202    4.089    0.000
##     Prop.Mam.Sp2            -0.123    0.105   -1.172    0.241
##   Nat_Pop._WB20152 ~~
##     Prop.Mam.Sp2            -0.115    0.091   -1.257    0.209
##
## Intercepts:
##                    Estimate  Std.Err  z-value  P(>|z|)
##    .Attendance2      -0.000    0.030   -0.000    1.000
##    .Total.Animals2   -0.000    0.104   -0.000    1.000
##    .Sp.Richness2      0.000    0.034    0.000    1.000
##    .Prp.Thrt.Abnd2   -0.000    0.077   -0.000    1.000
##    .Brilloun.Indx2   -0.000    0.106   -0.000    1.000
##    .Mean.Rap.Crck2    0.000    0.092    0.000    1.000
##     Zoo.Area.ha2      0.000    0.064    0.000    1.000
##     Mam.Sp.Rchnss2   -0.000    0.079   -0.000    1.000
##     Prop.Mam.Abdn2    0.000    0.113    0.000    1.000
##     Mn.Sp.BdyMsXA2    0.000    0.071    0.000    1.000
##     X50km_Pop2       -0.000    0.114   -0.000    1.000
##     X10km_Pop2        0.000    0.105    0.000    1.000
```

```
##     GDP.Millions2    -0.000    0.345   -0.000    1.000
##     Nt_Pp._WB20152    0.000    0.295    0.000    1.000
##     Prop.Mam.Sp2      0.000    0.118    0.000    1.000
##
## Variances:
##                    Estimate  Std.Err  z-value  P(>|z|)
##    .Attendance2       0.270    0.028    9.568    0.000
##    .Total.Animals2    0.251    0.044    5.729    0.000
##    .Sp.Richness2      0.151    0.046    3.269    0.001
##    .Prp.Thrt.Abnd2    0.986    0.133    7.389    0.000
##    .Brilloun.Indx2    0.290    0.079    3.656    0.000
##    .Mean.Rap.Crck2    0.934    0.100    9.311    0.000
##     Zoo.Area.ha2      0.998    0.112    8.886    0.000
##     Mam.Sp.Rchnss2    0.998    0.115    8.669    0.000
##     Prop.Mam.Abdn2    0.998    0.121    8.221    0.000
##     Mn.Sp.BdyMsXA2    0.998    0.160    6.219    0.000
##     X50km_Pop2        0.998    0.131    7.623    0.000
##     X10km_Pop2        0.998    0.174    5.745    0.000
##     GDP.Millions2     0.998    0.246    4.061    0.000
##     Nt_Pp._WB20152    0.998    0.225    4.439    0.000
##     Prop.Mam.Sp2      0.998    0.150    6.662    0.000
##
## R-Square:
##                    Estimate
##     Attendance2       0.740
##     Total.Animals2    0.748
##     Sp.Richness2      0.849
##     Prp.Thrt.Abnd2    0.012
##     Brilloun.Indx2    0.709
##     Mean.Rap.Crck2    0.064
```

```
# Generate fit indices
fitMeasures(fit.adj2A, c("agfi", "rmr", "srmr", "rmsea", "cfi", "nnfi", "tli"))
```

```
##  agfi   rmr  srmr rmsea   cfi  nnfi   tli
## 0.650 0.090 0.085 0.155 0.840 0.760 0.760
```

```
# Generate modification indices
mi2adjA <- modindices(fit.adj2A)
print(mi2adjA[mi2adjA$mi > 3.0,])
```

```
##                      lhs op                     rhs     mi mi.scaled
## 98        Total.Animals2 ~~       Mean.Raup.Crick2 11.525     3.167
## 100         Sp.Richness2 ~~      Brillouin.Index2  8.649     2.377
## 101         Sp.Richness2 ~~       Mean.Raup.Crick2 21.687     5.959
## 102     Prop.Threat.Abund2 ~~    Brillouin.Index2 13.812     3.795
## 109       Total.Animals2  ~       Mean.Raup.Crick2 11.525     3.167
## 111       Total.Animals2  ~        Prop.Mam.Abdun2  7.869     2.162
## 112       Total.Animals2  ~ Mean.Sp.BodyMassXAbund2 24.303     6.678
## 113       Total.Animals2  ~             X50km_Pop2 35.809     9.840
## 114       Total.Animals2  ~             X10km_Pop2  8.873     2.438
## 115       Total.Animals2  ~          GDP.Millions2 29.909     8.219
## 116       Total.Animals2  ~        Nat_Pop._WB20152  8.000     2.198
## 121         Sp.Richness2  ~       Brillouin.Index2  6.728     1.849
## 122         Sp.Richness2  ~       Mean.Raup.Crick2 20.798     5.715
## 124         Sp.Richness2  ~ Mean.Sp.BodyMassXAbund2  9.674     2.658
```

```
## 128            Sp.Richness2  ~        Nat_Pop._WB20152   3.059      0.841
## 129      Prop.Threat.Abund2  ~            Attendance2   5.798      1.593
## 130      Prop.Threat.Abund2  ~          Total.Animals2   5.628      1.547
## 131      Prop.Threat.Abund2  ~            Sp.Richness2  13.491      3.707
## 135      Prop.Threat.Abund2  ~          Prop.Mam.Abdun2   6.355      1.746
## 136      Prop.Threat.Abund2  ~ Mean.Sp.BodyMassXAbund2  14.759      4.056
## 137      Prop.Threat.Abund2  ~             X50km_Pop2  11.993      3.296
## 138      Prop.Threat.Abund2  ~             X10km_Pop2   7.595      2.087
## 140      Prop.Threat.Abund2  ~        Nat_Pop._WB20152   7.068      1.942
## 141      Prop.Threat.Abund2  ~            Prop.Mam.Sp2  19.197      5.275
## 142        Brillouin.Index2  ~            Attendance2   3.315      0.911
## 143        Brillouin.Index2  ~      Prop.Threat.Abund2  13.812      3.795
## 147        Brillouin.Index2  ~          Prop.Mam.Abdun2  13.803      3.793
## 148        Brillouin.Index2  ~ Mean.Sp.BodyMassXAbund2  10.704      2.941
## 150        Brillouin.Index2  ~             X10km_Pop2   4.078      1.121
## 151        Brillouin.Index2  ~           GDP.Millions2  58.529     16.083
## 152        Brillouin.Index2  ~        Nat_Pop._WB20152  50.588     13.901
## 153        Brillouin.Index2  ~            Prop.Mam.Sp2   9.791      2.690
## 154        Mean.Raup.Crick2  ~            Attendance2  16.367      4.497
## 157        Mean.Raup.Crick2  ~            Zoo.Area.ha2  11.525      3.167
## 158        Mean.Raup.Crick2  ~        Mam.Sp.Richness2  40.269     11.066
## 159        Mean.Raup.Crick2  ~          Prop.Mam.Abdun2  66.708     18.331
## 160        Mean.Raup.Crick2  ~ Mean.Sp.BodyMassXAbund2  96.529     26.525
## 161        Mean.Raup.Crick2  ~             X50km_Pop2   6.300      1.731
## 162        Mean.Raup.Crick2  ~             X10km_Pop2   4.704      1.292
## 164        Mean.Raup.Crick2  ~        Nat_Pop._WB20152  10.677      2.934
## 165        Mean.Raup.Crick2  ~            Prop.Mam.Sp2 106.001     29.128
## 166            Zoo.Area.ha2  ~            Attendance2  28.193      7.747
## 167            Zoo.Area.ha2  ~          Total.Animals2  31.171      8.566
## 168            Zoo.Area.ha2  ~            Sp.Richness2  22.368      6.146
## 171            Zoo.Area.ha2  ~        Mean.Raup.Crick2   8.032      2.207
## 180        Mam.Sp.Richness2  ~            Attendance2   8.657      2.379
## 181        Mam.Sp.Richness2  ~          Total.Animals2   4.984      1.369
## 182        Mam.Sp.Richness2  ~            Sp.Richness2  10.502      2.886
## 185        Mam.Sp.Richness2  ~        Mean.Raup.Crick2  12.796      3.516
## 194         Prop.Mam.Abdun2  ~            Attendance2   8.839      2.429
## 195         Prop.Mam.Abdun2  ~          Total.Animals2  11.568      3.179
## 197         Prop.Mam.Abdun2  ~      Prop.Threat.Abund2   3.479      0.956
## 198         Prop.Mam.Abdun2  ~        Brillouin.Index2   4.471      1.229
## 208 Mean.Sp.BodyMassXAbund2  ~            Attendance2  49.773     13.677
## 209 Mean.Sp.BodyMassXAbund2  ~          Total.Animals2  33.919      9.321
## 210 Mean.Sp.BodyMassXAbund2  ~            Sp.Richness2   7.245      1.991
## 211 Mean.Sp.BodyMassXAbund2  ~      Prop.Threat.Abund2   3.305      0.908
## 212 Mean.Sp.BodyMassXAbund2  ~        Brillouin.Index2   6.752      1.855
## 213 Mean.Sp.BodyMassXAbund2  ~        Mean.Raup.Crick2  44.037     12.101
## 222              X50km_Pop2  ~            Attendance2  24.320      6.683
## 223              X50km_Pop2  ~          Total.Animals2  21.424      5.887
## 225              X50km_Pop2  ~      Prop.Threat.Abund2   3.360      0.923
## 250           GDP.Millions2  ~            Attendance2  13.810      3.795
## 252           GDP.Millions2  ~            Sp.Richness2  29.233      8.033
## 254           GDP.Millions2  ~        Brillouin.Index2   3.643      1.001
## 255           GDP.Millions2  ~        Mean.Raup.Crick2  13.985      3.843
## 266        Nat_Pop._WB20152  ~            Sp.Richness2  30.274      8.319
## 268        Nat_Pop._WB20152  ~        Brillouin.Index2  16.787      4.613
```

```
## 269         Nat_Pop._WB20152  ~        Mean.Raup.Crick2  13.073      3.592
## 278             Prop.Mam.Sp2  ~            Attendance2   9.878      2.714
## 279             Prop.Mam.Sp2  ~          Total.Animals2  23.928      6.575
## 281             Prop.Mam.Sp2  ~      Prop.Threat.Abund2  10.458      2.874
## 282             Prop.Mam.Sp2  ~        Brillouin.Index2   5.246      1.442
## 283             Prop.Mam.Sp2  ~        Mean.Raup.Crick2   4.433      1.218
##         epc sepc.lv sepc.all sepc.nox
## 98   0.191   0.191    0.192    0.192
## 100  0.031   0.031    0.031    0.031
## 101 -0.089  -0.089   -0.089   -0.089
## 102  0.110   0.110    0.111    0.111
## 109  0.205   0.205    0.205    0.205
## 111 -0.084  -0.084   -0.084   -0.084
## 112 -0.142  -0.142   -0.142   -0.142
## 113  0.144   0.144    0.144    0.144
## 114  0.074   0.074    0.074    0.074
## 115 -0.128  -0.128   -0.128   -0.128
## 116 -0.066  -0.066   -0.066   -0.067
## 121  0.085   0.085    0.085    0.085
## 122 -0.091  -0.091   -0.091   -0.091
## 124 -0.081  -0.081   -0.081   -0.081
## 128 -0.032  -0.032   -0.032   -0.032
## 129  0.135   0.135    0.137    0.137
## 130 -0.128  -0.128   -0.128   -0.128
## 131 -0.279  -0.279   -0.279   -0.279
## 135  0.128   0.128    0.128    0.128
## 136  0.182   0.182    0.182    0.182
## 137  0.163   0.163    0.163    0.163
## 138  0.133   0.133    0.133    0.133
## 140  0.124   0.124    0.124    0.124
## 141  0.226   0.226    0.226    0.227
## 142 -0.076  -0.076   -0.077   -0.077
## 143  0.112   0.112    0.112    0.112
## 147  0.107   0.107    0.107    0.107
## 148  0.086   0.086    0.086    0.086
## 150 -0.054  -0.054   -0.054   -0.054
## 151 -0.193  -0.193   -0.193   -0.193
## 152 -0.179  -0.179   -0.179   -0.180
## 153  0.092   0.092    0.092    0.092
## 154 -0.307  -0.307   -0.314   -0.314
## 157 -0.170  -0.170   -0.170   -0.170
## 158 -0.424  -0.424   -0.424   -0.424
## 159 -0.420  -0.420   -0.420   -0.421
## 160 -0.462  -0.462   -0.462   -0.463
## 161  0.116   0.116    0.116    0.116
## 162  0.104   0.104    0.104    0.104
## 164  0.148   0.148    0.148    0.148
## 165 -0.545  -0.545   -0.545   -0.545
## 166  0.848   0.848    0.865    0.865
## 167  0.569   0.569    0.569    0.569
## 168  0.894   0.894    0.894    0.894
## 171  0.110   0.110    0.110    0.110
## 180 -0.477  -0.477   -0.487   -0.487
## 181 -0.216  -0.216   -0.216   -0.216
```

```
## 182 -0.783  -0.783   -0.783   -0.783
## 185 -0.185  -0.185   -0.185   -0.185
## 194 -0.137  -0.137   -0.139   -0.139
## 195 -0.124  -0.124   -0.124   -0.124
## 197 -0.040  -0.040   -0.040   -0.040
## 198  0.067   0.067    0.067    0.067
## 208 -0.642  -0.642   -0.655   -0.655
## 209 -0.311  -0.311   -0.311   -0.311
## 210 -0.218  -0.218   -0.218   -0.218
## 211  0.058   0.058    0.058    0.058
## 212  0.121   0.121    0.121    0.121
## 213 -0.212  -0.212   -0.212   -0.212
## 222  0.459   0.459    0.468    0.468
## 223  0.234   0.234    0.234    0.234
## 225  0.055   0.055    0.055    0.055
## 250 -0.277  -0.277   -0.283   -0.283
## 252  0.332   0.332    0.332    0.332
## 254  0.067   0.067    0.067    0.067
## 255 -0.091  -0.091   -0.091   -0.091
## 266 -0.326  -0.326   -0.326   -0.326
## 268 -0.139  -0.139   -0.139   -0.139
## 269  0.085   0.085    0.085    0.085
## 278  0.125   0.125    0.127    0.127
## 279  0.185   0.185    0.185    0.185
## 281  0.070   0.070    0.070    0.070
## 282 -0.075  -0.075   -0.075   -0.075
## 283 -0.047  -0.047   -0.047   -0.047
```

Model 3

Based on the modification indices generated from the second model, we can see that **Mean.Raup.Crick2** ~ **Mean.Sp.BodyMassXAbund2** has an mi value of **96.529**. This is the second highest mi value and far exceeds the standard cut-off level for the chi-square test criterion of 3.84 (Burnham and Anderson, 2002). This is also one of the most intuitive relationships, as if we increase the body mass of the animals in a collection, then ultimately the collection will be able to house fewer of them (due to the increased space requirements). This reduction in available spaces will mean it is more likely that the collection composition will overlap with other institutions. As a result, we add this relationship to our model. Once again, the model summary, fit indices and modification indices were all generated for the model, adjusting for the nested nature of data.

```
# Attendance SEM (Species Abundance)

# Model 3
# Addition of Mean.Raup.Crick2 ~ Mean.Sp.BodyMassXAbund2, mi = 96.529

mod.3A <- 'Attendance2 ~ Zoo.Area.ha2 + Sp.Richness2 + Total.Animals2
+ Mam.Sp.Richness2 + Prop.Mam.Abdun2 + Prop.Threat.Abund2
+ Mean.Sp.BodyMassXAbund2 + Brillouin.Index2 + Mean.Raup.Crick2
+ X50km_Pop2 + X10km_Pop2 + GDP.Millions2 + Nat_Pop._WB20152

Total.Animals2 ~ Zoo.Area.ha2 + Sp.Richness2
Sp.Richness2 ~ Zoo.Area.ha2 + Prop.Mam.Sp2 + Mam.Sp.Richness2
Prop.Threat.Abund2 ~ Brillouin.Index2
```

```r
Brillouin.Index2 ~ Sp.Richness2 + Total.Animals2
Mean.Raup.Crick2 ~ Sp.Richness2 + Total.Animals2 + Mean.Sp.BodyMassXAbund2'

# Fit model and generate model summary
mod.3A.fit <- sem(mod.3A, data = sem_attendance_data, fixed.x=FALSE)
summary(mod.3A.fit, rsq = TRUE)
```

```
## lavaan (0.5-23.1097) converged normally after  64 iterations
##
##   Number of observations                           458
##
##   Estimator                                         ML
##   Minimum Function Test Statistic              450.550
##   Degrees of freedom                                45
##   P-value (Chi-square)                           0.000
##
## Parameter Estimates:
##
##   Information                                 Expected
##   Standard Errors                             Standard
##
## Regressions:
##                           Estimate  Std.Err  z-value  P(>|z|)
##   Attendance2 ~
##     Zoo.Area.ha2            0.082    0.034    2.450    0.014
##     Sp.Richness2           -0.280    0.086   -3.268    0.001
##     Total.Animals2          0.548    0.055    9.947    0.000
##     Mam.Sp.Rchnss2          0.146    0.048    3.058    0.002
##     Prop.Mam.Abdn2         -0.100    0.040   -2.495    0.013
##     Prp.Thrt.Abnd2          0.017    0.024    0.700    0.484
##     Mn.Sp.BdyMsXA2          0.324    0.037    8.749    0.000
##     Brilloun.Indx2          0.025    0.045    0.563    0.573
##     Mean.Rap.Crck2          0.116    0.028    4.117    0.000
##     X50km_Pop2              0.073    0.038    1.911    0.056
##     X10km_Pop2              0.417    0.041   10.141    0.000
##     GDP.Millions2           0.263    0.048    5.532    0.000
##     Nt_Pp._WB20152         -0.128    0.049   -2.603    0.009
##   Total.Animals2 ~
##     Zoo.Area.ha2            0.223    0.024    9.384    0.000
##     Sp.Richness2            0.798    0.024   33.520    0.000
##   Sp.Richness2 ~
##     Zoo.Area.ha2            0.067    0.021    3.225    0.001
##     Prop.Mam.Sp2           -0.581    0.019  -30.364    0.000
##     Mam.Sp.Rchnss2          0.755    0.020   38.373    0.000
##   Prop.Threat.Abund2 ~
##     Brilloun.Indx2          0.107    0.046    2.311    0.021
##   Brillouin.Index2 ~
##     Sp.Richness2            1.232    0.046   26.770    0.000
##     Total.Animals2         -0.527    0.046  -11.451    0.000
##   Mean.Raup.Crick2 ~
##     Sp.Richness2           -0.262    0.077   -3.414    0.001
##     Total.Animals2          0.400    0.076    5.294    0.000
##     Mn.Sp.BdyMsXA2         -0.446    0.042  -10.626    0.000
##
```

```
## Covariances:
##                              Estimate  Std.Err  z-value  P(>|z|)
##   Zoo.Area.ha2 ~~
##     Mam.Sp.Rchnss2              0.381    0.050    7.634    0.000
##     Prop.Mam.Abdn2              0.352    0.049    7.121    0.000
##     Mn.Sp.BdyMsXA2              0.522    0.053    9.923    0.000
##     X50km_Pop2                  0.060    0.047    1.291    0.197
##     X10km_Pop2                 -0.010    0.047   -0.208    0.835
##     GDP.Millions2              -0.027    0.047   -0.588    0.557
##     Nt_Pp._WB20152              0.061    0.047    1.310    0.190
##     Prop.Mam.Sp2                0.312    0.049    6.389    0.000
##   Mam.Sp.Richness2 ~~
##     Prop.Mam.Abdn2              0.058    0.047    1.237    0.216
##     Mn.Sp.BdyMsXA2              0.227    0.048    4.750    0.000
##     X50km_Pop2                  0.204    0.048    4.281    0.000
##     X10km_Pop2                  0.284    0.048    5.852    0.000
##     GDP.Millions2              -0.060    0.047   -1.277    0.202
##     Nt_Pp._WB20152             -0.088    0.047   -1.881    0.060
##     Prop.Mam.Sp2                0.088    0.047    1.882    0.060
##   Prop.Mam.Abdun2 ~~
##     Mn.Sp.BdyMsXA2              0.592    0.054   10.922    0.000
##     X50km_Pop2                 -0.123    0.047   -2.622    0.009
##     X10km_Pop2                 -0.248    0.048   -5.165    0.000
##     GDP.Millions2              -0.115    0.047   -2.458    0.014
##     Nt_Pp._WB20152             -0.111    0.047   -2.362    0.018
##     Prop.Mam.Sp2                0.878    0.062   14.135    0.000
##   Mean.Sp.BodyMassXAbund2 ~~
##     X50km_Pop2                  0.013    0.047    0.275    0.784
##     X10km_Pop2                  0.021    0.047    0.455    0.649
##     GDP.Millions2              -0.079    0.047   -1.682    0.093
##     Nt_Pp._WB20152             -0.004    0.047   -0.079    0.937
##     Prop.Mam.Sp2                0.628    0.055   11.405    0.000
##   X50km_Pop2 ~~
##     X10km_Pop2                  0.752    0.058   12.886    0.000
##     GDP.Millions2              -0.013    0.047   -0.281    0.779
##     Nt_Pp._WB20152              0.191    0.047    4.033    0.000
##     Prop.Mam.Sp2               -0.096    0.047   -2.054    0.040
##   X10km_Pop2 ~~
##     GDP.Millions2              -0.028    0.047   -0.604    0.546
##     Nt_Pp._WB20152              0.173    0.047    3.646    0.000
##     Prop.Mam.Sp2               -0.204    0.048   -4.293    0.000
##   GDP.Millions2 ~~
##     Nt_Pp._WB20152              0.826    0.061   13.642    0.000
##     Prop.Mam.Sp2               -0.123    0.047   -2.615    0.009
##   Nat_Pop._WB20152 ~~
##     Prop.Mam.Sp2               -0.115    0.047   -2.447    0.014
##
## Variances:
##                    Estimate  Std.Err  z-value  P(>|z|)
##    .Attendance2       0.270    0.018   15.133    0.000
##    .Total.Animals2    0.251    0.017   15.133    0.000
##    .Sp.Richness2      0.151    0.010   15.133    0.000
##    .Prp.Thrt.Abnd2    0.986    0.065   15.133    0.000
##    .Brilloun.Indx2    0.290    0.019   15.133    0.000
```

```
##    .Mean.Rap.Crck2    0.744    0.049   15.133    0.000
##     Zoo.Area.ha2      0.998    0.066   15.133    0.000
##     Mam.Sp.Rchnss2    0.998    0.066   15.133    0.000
##     Prop.Mam.Abdn2    0.998    0.066   15.133    0.000
##     Mn.Sp.BdyMsXA2    0.998    0.066   15.133    0.000
##     X50km_Pop2        0.998    0.066   15.133    0.000
##     X10km_Pop2        0.998    0.066   15.133    0.000
##     GDP.Millions2     0.998    0.066   15.133    0.000
##     Nt_Pp._WB20152    0.998    0.066   15.133    0.000
##     Prop.Mam.Sp2      0.998    0.066   15.133    0.000
##
## R-Square:
##                    Estimate
##     Attendance2       0.728
##     Total.Animals2    0.748
##     Sp.Richness2      0.849
##     Prp.Thrt.Abnd2    0.012
##     Brilloun.Indx2    0.709
##     Mean.Rap.Crck2    0.227
```

```
# Generate fit indices
fitMeasures(mod.3A.fit, c("agfi", "rmr", "srmr", "rmsea", "cfi", "nnfi", "tli"))
```

```
##  agfi   rmr  srmr rmsea   cfi  nnfi   tli
## 0.724 0.063 0.063 0.140 0.873 0.805 0.805
```

```
# Generate modification indices
mi3A <- modindices(mod.3A.fit)
print(mi3A[mi3A$mi > 3.0,])
```

```
##                    lhs op                     rhs     mi    epc
## 84          Total.Animals2 ~~        Mean.Raup.Crick2 10.638 -0.194
## 86            Sp.Richness2 ~~        Brillouin.Index2  8.649  0.031
## 87            Sp.Richness2 ~~        Mean.Raup.Crick2 30.292 -0.094
## 88       Prop.Threat.Abund2 ~~       Brillouin.Index2 13.812  0.110
## 95          Total.Animals2  ~        Mean.Raup.Crick2  3.175  0.090
## 97          Total.Animals2  ~          Prop.Mam.Abdun2  7.869 -0.084
## 98          Total.Animals2  ~ Mean.Sp.BodyMassXAbund2 24.303 -0.142
## 99          Total.Animals2  ~              X50km_Pop2 35.809  0.144
## 100         Total.Animals2  ~              X10km_Pop2  8.873  0.074
## 101         Total.Animals2  ~           GDP.Millions2 29.909 -0.128
## 102         Total.Animals2  ~        Nat_Pop._WB20152  8.000 -0.066
## 107           Sp.Richness2  ~        Brillouin.Index2  6.727  0.085
## 108           Sp.Richness2  ~        Mean.Raup.Crick2 15.050 -0.081
## 110           Sp.Richness2  ~ Mean.Sp.BodyMassXAbund2  9.674 -0.081
## 114           Sp.Richness2  ~        Nat_Pop._WB20152  3.059 -0.032
## 115     Prop.Threat.Abund2  ~             Attendance2  6.215  0.144
## 116     Prop.Threat.Abund2  ~          Total.Animals2  5.628 -0.128
## 117     Prop.Threat.Abund2  ~            Sp.Richness2 13.491 -0.279
## 121     Prop.Threat.Abund2  ~          Prop.Mam.Abdun2  6.355  0.128
## 122     Prop.Threat.Abund2  ~ Mean.Sp.BodyMassXAbund2 14.759  0.182
## 123     Prop.Threat.Abund2  ~              X50km_Pop2 11.993  0.163
## 124     Prop.Threat.Abund2  ~              X10km_Pop2  7.595  0.133
## 126     Prop.Threat.Abund2  ~        Nat_Pop._WB20152  7.068  0.124
## 127     Prop.Threat.Abund2  ~            Prop.Mam.Sp2 19.197  0.226
## 128       Brillouin.Index2  ~             Attendance2  3.685 -0.084
```

```
## 129        Brillouin.Index2  ~      Prop.Threat.Abund2 13.812  0.112
## 133        Brillouin.Index2  ~         Prop.Mam.Abdun2 13.803  0.107
## 134        Brillouin.Index2  ~ Mean.Sp.BodyMassXAbund2 10.704  0.086
## 136        Brillouin.Index2  ~             X10km_Pop2  4.078 -0.054
## 137        Brillouin.Index2  ~          GDP.Millions2 58.529 -0.193
## 138        Brillouin.Index2  ~        Nat_Pop._WB20152 50.588 -0.179
## 139        Brillouin.Index2  ~            Prop.Mam.Sp2  9.791  0.092
## 140        Mean.Raup.Crick2  ~             Attendance2 11.294  0.291
## 143        Mean.Raup.Crick2  ~            Zoo.Area.ha2 10.638  0.172
## 145        Mean.Raup.Crick2  ~         Prop.Mam.Abdun2 12.228 -0.196
## 146        Mean.Raup.Crick2  ~              X50km_Pop2 10.789  0.136
## 147        Mean.Raup.Crick2  ~              X10km_Pop2 11.524  0.146
## 149        Mean.Raup.Crick2  ~        Nat_Pop._WB20152 13.100  0.146
## 150        Mean.Raup.Crick2  ~            Prop.Mam.Sp2 37.344 -0.370
## 151            Zoo.Area.ha2  ~             Attendance2 28.893  0.875
## 152            Zoo.Area.ha2  ~          Total.Animals2 31.172  0.569
## 153            Zoo.Area.ha2  ~            Sp.Richness2 22.368  0.894
## 156            Zoo.Area.ha2  ~        Mean.Raup.Crick2  9.532  0.134
## 165        Mam.Sp.Richness2  ~             Attendance2  5.629 -0.388
## 166        Mam.Sp.Richness2  ~          Total.Animals2  4.983 -0.216
## 167        Mam.Sp.Richness2  ~            Sp.Richness2 10.502 -0.783
## 170        Mam.Sp.Richness2  ~        Mean.Raup.Crick2  4.862 -0.128
## 179         Prop.Mam.Abdun2  ~             Attendance2  8.906 -0.138
## 180         Prop.Mam.Abdun2  ~          Total.Animals2 11.568 -0.124
## 182         Prop.Mam.Abdun2  ~      Prop.Threat.Abund2  3.479 -0.040
## 183         Prop.Mam.Abdun2  ~        Brillouin.Index2  4.471  0.067
## 193 Mean.Sp.BodyMassXAbund2  ~             Attendance2 25.409 -0.472
## 194 Mean.Sp.BodyMassXAbund2  ~          Total.Animals2 33.919 -0.311
## 195 Mean.Sp.BodyMassXAbund2  ~            Sp.Richness2  7.245 -0.218
## 196 Mean.Sp.BodyMassXAbund2  ~      Prop.Threat.Abund2  3.305  0.058
## 197 Mean.Sp.BodyMassXAbund2  ~        Brillouin.Index2  6.752  0.121
## 207              X50km_Pop2  ~             Attendance2 24.713  0.466
## 208              X50km_Pop2  ~          Total.Animals2 21.424  0.234
## 210              X50km_Pop2  ~      Prop.Threat.Abund2  3.359  0.055
## 212              X50km_Pop2  ~        Mean.Raup.Crick2  3.049  0.059
## 235           GDP.Millions2  ~             Attendance2 14.035 -0.282
## 237           GDP.Millions2  ~            Sp.Richness2 29.232  0.332
## 239           GDP.Millions2  ~        Brillouin.Index2  3.643  0.067
## 240           GDP.Millions2  ~        Mean.Raup.Crick2 17.268 -0.112
## 251        Nat_Pop._WB20152  ~            Sp.Richness2 30.273 -0.326
## 253        Nat_Pop._WB20152  ~        Brillouin.Index2 16.787 -0.139
## 254        Nat_Pop._WB20152  ~        Mean.Raup.Crick2 16.142  0.104
## 263            Prop.Mam.Sp2  ~             Attendance2  9.242  0.121
## 264            Prop.Mam.Sp2  ~          Total.Animals2 23.928  0.185
## 266            Prop.Mam.Sp2  ~      Prop.Threat.Abund2 10.458  0.070
## 267            Prop.Mam.Sp2  ~        Brillouin.Index2  5.246 -0.075
## 268            Prop.Mam.Sp2  ~        Mean.Raup.Crick2  8.609 -0.072
##     sepc.lv sepc.all sepc.nox
## 84   -0.194   -0.198   -0.198
## 86    0.031    0.031    0.031
## 87   -0.094   -0.096   -0.096
## 88    0.110    0.111    0.111
## 95    0.090    0.088    0.088
## 97   -0.084   -0.084   -0.084
```

```
## 98   -0.142   -0.142   -0.142
## 99    0.144    0.144    0.144
## 100   0.074    0.074    0.074
## 101  -0.128   -0.128   -0.128
## 102  -0.066   -0.066   -0.067
## 107   0.085    0.085    0.085
## 108  -0.081   -0.079   -0.079
## 110  -0.081   -0.081   -0.081
## 114  -0.032   -0.032   -0.032
## 115   0.144    0.144    0.144
## 116  -0.128   -0.128   -0.128
## 117  -0.279   -0.279   -0.279
## 121   0.128    0.128    0.128
## 122   0.182    0.182    0.182
## 123   0.163    0.163    0.163
## 124   0.133    0.133    0.133
## 126   0.124    0.124    0.124
## 127   0.226    0.226    0.227
## 128  -0.084   -0.084   -0.084
## 129   0.112    0.112    0.112
## 133   0.107    0.107    0.107
## 134   0.086    0.086    0.086
## 136  -0.054   -0.054   -0.054
## 137  -0.193   -0.193   -0.193
## 138  -0.179   -0.179   -0.180
## 139   0.092    0.092    0.092
## 140   0.291    0.295    0.295
## 143   0.172    0.176    0.176
## 145  -0.196   -0.199   -0.200
## 146   0.136    0.138    0.138
## 147   0.146    0.149    0.149
## 149   0.146    0.149    0.149
## 150  -0.370   -0.377   -0.378
## 151   0.875    0.873    0.873
## 152   0.569    0.569    0.569
## 153   0.894    0.894    0.894
## 156   0.134    0.132    0.132
## 165  -0.388   -0.387   -0.387
## 166  -0.216   -0.216   -0.216
## 167  -0.783   -0.783   -0.783
## 170  -0.128   -0.125   -0.125
## 179  -0.138   -0.137   -0.137
## 180  -0.124   -0.124   -0.124
## 182  -0.040   -0.040   -0.040
## 183   0.067    0.067    0.067
## 193  -0.472   -0.471   -0.471
## 194  -0.311   -0.311   -0.311
## 195  -0.218   -0.218   -0.218
## 196   0.058    0.058    0.058
## 197   0.121    0.121    0.121
## 207   0.466    0.465    0.465
## 208   0.234    0.234    0.234
## 210   0.055    0.055    0.055
## 212   0.059    0.058    0.058
```

```
## 235  -0.282   -0.281   -0.281
## 237   0.332    0.332    0.332
## 239   0.067    0.067    0.067
## 240  -0.112   -0.110   -0.110
## 251  -0.326   -0.326   -0.326
## 253  -0.139   -0.139   -0.139
## 254   0.104    0.103    0.103
## 263   0.121    0.121    0.121
## 264   0.185    0.185    0.185
## 266   0.070    0.070    0.070
## 267  -0.075   -0.075   -0.075
## 268  -0.072   -0.071   -0.071
```

```r
# Adjust for the nested nature of the data (institutions within countries)
# Fit model and generate model summary
design <- svydesign(ids = ~Country, nest=TRUE, data=sem_attendance_data)
fit.adj3A <- lavaan.survey(lavaan.fit = mod.3A.fit, survey.design = design)
summary(fit.adj3A, rsq = TRUE)
```

```
## lavaan (0.5-23.1097) converged normally after  64 iterations
##
##   Number of observations                           458
##
##   Estimator                                         ML      Robust
##   Minimum Function Test Statistic              450.550     117.401
##   Degrees of freedom                                45          45
##   P-value (Chi-square)                           0.000       0.000
##   Scaling correction factor                                  3.838
##     for the Satorra-Bentler correction
##
## Parameter Estimates:
##
##   Information                                 Expected
##   Standard Errors                           Robust.sem
##
## Regressions:
##                      Estimate  Std.Err  z-value  P(>|z|)
##   Attendance2 ~
##     Zoo.Area.ha2        0.082    0.039    2.104    0.035
##     Sp.Richness2       -0.280    0.076   -3.692    0.000
##     Total.Animals2      0.548    0.053   10.432    0.000
##     Mam.Sp.Rchnss2      0.146    0.042    3.447    0.001
##     Prop.Mam.Abdn2     -0.100    0.030   -3.308    0.001
##     Prp.Thrt.Abnd2      0.017    0.028    0.615    0.538
##     Mn.Sp.BdyMsXA2      0.324    0.027   12.021    0.000
##     Brilloun.Indx2      0.025    0.058    0.441    0.659
##     Mean.Rap.Crck2      0.116    0.031    3.713    0.000
##     X50km_Pop2          0.073    0.032    2.257    0.024
##     X10km_Pop2          0.417    0.043    9.718    0.000
##     GDP.Millions2       0.263    0.056    4.704    0.000
##     Nt_Pp._WB20152     -0.128    0.063   -2.039    0.041
##   Total.Animals2 ~
##     Zoo.Area.ha2        0.223    0.038    5.856    0.000
##     Sp.Richness2        0.798    0.048   16.678    0.000
##   Sp.Richness2 ~
```

```
##     Zoo.Area.ha2          0.067    0.044    1.519    0.129
##     Prop.Mam.Sp2         -0.581    0.038  -15.231    0.000
##     Mam.Sp.Rchnss2        0.755    0.068   11.066    0.000
##   Prop.Threat.Abund2 ~
##     Brilloun.Indx2        0.107    0.132    0.810    0.418
##   Brillouin.Index2 ~
##     Sp.Richness2          1.232    0.068   18.134    0.000
##     Total.Animals2       -0.527    0.078   -6.780    0.000
##   Mean.Raup.Crick2 ~
##     Sp.Richness2         -0.262    0.238   -1.099    0.272
##     Total.Animals2        0.400    0.164    2.446    0.014
##     Mn.Sp.BdyMsXA2       -0.446    0.058   -7.716    0.000
##
## Covariances:
##                            Estimate  Std.Err  z-value  P(>|z|)
##   Zoo.Area.ha2 ~~
##     Mam.Sp.Rchnss2            0.381    0.064    5.985    0.000
##     Prop.Mam.Abdn2            0.352    0.092    3.822    0.000
##     Mn.Sp.BdyMsXA2            0.522    0.101    5.177    0.000
##     X50km_Pop2                0.060    0.069    0.880    0.379
##     X10km_Pop2               -0.010    0.078   -0.124    0.901
##     GDP.Millions2            -0.027    0.041   -0.673    0.501
##     Nt_Pp._WB20152            0.061    0.090    0.682    0.495
##     Prop.Mam.Sp2              0.312    0.089    3.510    0.000
##   Mam.Sp.Richness2 ~~
##     Prop.Mam.Abdn2            0.058    0.049    1.172    0.241
##     Mn.Sp.BdyMsXA2            0.227    0.078    2.921    0.003
##     X50km_Pop2                0.204    0.082    2.487    0.013
##     X10km_Pop2                0.284    0.066    4.278    0.000
##     GDP.Millions2            -0.060    0.064   -0.937    0.349
##     Nt_Pp._WB20152           -0.088    0.102   -0.863    0.388
##     Prop.Mam.Sp2              0.088    0.061    1.435    0.151
##   Prop.Mam.Abdun2 ~~
##     Mn.Sp.BdyMsXA2            0.592    0.109    5.445    0.000
##     X50km_Pop2               -0.123    0.054   -2.263    0.024
##     X10km_Pop2               -0.248    0.060   -4.154    0.000
##     GDP.Millions2            -0.115    0.101   -1.140    0.254
##     Nt_Pp._WB20152           -0.111    0.088   -1.264    0.206
##     Prop.Mam.Sp2              0.878    0.128    6.865    0.000
##   Mean.Sp.BodyMassXAbund2 ~~
##     X50km_Pop2                0.013    0.074    0.174    0.862
##     X10km_Pop2                0.021    0.064    0.329    0.742
##     GDP.Millions2            -0.079    0.038   -2.055    0.040
##     Nt_Pp._WB20152           -0.004    0.084   -0.044    0.965
##     Prop.Mam.Sp2              0.628    0.126    5.002    0.000
##   X50km_Pop2 ~~
##     X10km_Pop2                0.752    0.126    5.982    0.000
##     GDP.Millions2            -0.013    0.100   -0.131    0.896
##     Nt_Pp._WB20152            0.191    0.177    1.083    0.279
##     Prop.Mam.Sp2             -0.096    0.051   -1.888    0.059
##   X10km_Pop2 ~~
##     GDP.Millions2            -0.028    0.056   -0.505    0.613
##     Nt_Pp._WB20152            0.173    0.101    1.701    0.089
##     Prop.Mam.Sp2             -0.204    0.055   -3.685    0.000
```

```
##   GDP.Millions2 ~~
##     Nt_Pp._WB20152              0.826    0.202    4.089    0.000
##     Prop.Mam.Sp2               -0.123    0.105   -1.172    0.241
##   Nat_Pop._WB20152 ~~
##     Prop.Mam.Sp2               -0.115    0.091   -1.257    0.209
##
## Intercepts:
##                    Estimate  Std.Err  z-value  P(>|z|)
##    .Attendance2      -0.000    0.030   -0.000    1.000
##    .Total.Animals2   -0.000    0.104   -0.000    1.000
##    .Sp.Richness2      0.000    0.034    0.000    1.000
##    .Prp.Thrt.Abnd2   -0.000    0.077   -0.000    1.000
##    .Brilloun.Indx2   -0.000    0.106   -0.000    1.000
##    .Mean.Rap.Crck2    0.000    0.094    0.000    1.000
##     Zoo.Area.ha2      0.000    0.064    0.000    1.000
##     Mam.Sp.Rchnss2   -0.000    0.079   -0.000    1.000
##     Prop.Mam.Abdn2    0.000    0.113    0.000    1.000
##     Mn.Sp.BdyMsXA2    0.000    0.071    0.000    1.000
##     X50km_Pop2       -0.000    0.114   -0.000    1.000
##     X10km_Pop2        0.000    0.105    0.000    1.000
##     GDP.Millions2    -0.000    0.345   -0.000    1.000
##     Nt_Pp._WB20152    0.000    0.295    0.000    1.000
##     Prop.Mam.Sp2      0.000    0.118    0.000    1.000
##
## Variances:
##                    Estimate  Std.Err  z-value  P(>|z|)
##    .Attendance2       0.270    0.028    9.568    0.000
##    .Total.Animals2    0.251    0.044    5.729    0.000
##    .Sp.Richness2      0.151    0.046    3.269    0.001
##    .Prp.Thrt.Abnd2    0.986    0.133    7.389    0.000
##    .Brilloun.Indx2    0.290    0.079    3.656    0.000
##    .Mean.Rap.Crck2    0.744    0.068   10.938    0.000
##     Zoo.Area.ha2      0.998    0.112    8.886    0.000
##     Mam.Sp.Rchnss2    0.998    0.115    8.669    0.000
##     Prop.Mam.Abdn2    0.998    0.121    8.221    0.000
##     Mn.Sp.BdyMsXA2    0.998    0.160    6.219    0.000
##     X50km_Pop2        0.998    0.131    7.623    0.000
##     X10km_Pop2        0.998    0.174    5.745    0.000
##     GDP.Millions2     0.998    0.246    4.061    0.000
##     Nt_Pp._WB20152    0.998    0.225    4.439    0.000
##     Prop.Mam.Sp2      0.998    0.150    6.662    0.000
##
## R-Square:
##                    Estimate
##     Attendance2       0.728
##     Total.Animals2    0.748
##     Sp.Richness2      0.849
##     Prp.Thrt.Abnd2    0.012
##     Brilloun.Indx2    0.709
##     Mean.Rap.Crck2    0.227
```

```r
# Generate fit indices
fitMeasures(fit.adj3A, c("agfi", "rmr", "srmr", "rmsea", "cfi", "nnfi", "tli"))
```

```
##  agfi   rmr  srmr rmsea   cfi  nnfi   tli
```

```
## 0.690 0.063 0.060 0.140 0.873 0.805 0.805
```

```r
# Generate modification indices
mi3adjA <- modindices(fit.adj3A)
print(mi3adjA[mi3adjA$mi > 3.0,])
```

```
##                         lhs op                      rhs     mi mi.scaled
## 99            Total.Animals2 ~~         Mean.Raup.Crick2 10.639     2.772
## 101             Sp.Richness2 ~~        Brillouin.Index2  8.649     2.254
## 102             Sp.Richness2 ~~        Mean.Raup.Crick2 30.292     7.893
## 103       Prop.Threat.Abund2 ~~        Brillouin.Index2 13.812     3.599
## 110           Total.Animals2  ~        Mean.Raup.Crick2  3.175     0.827
## 112           Total.Animals2  ~          Prop.Mam.Abdun2  7.869     2.050
## 113           Total.Animals2  ~ Mean.Sp.BodyMassXAbund2 24.303     6.333
## 114           Total.Animals2  ~              X50km_Pop2 35.809     9.331
## 115           Total.Animals2  ~              X10km_Pop2  8.873     2.312
## 116           Total.Animals2  ~            GDP.Millions2 29.909     7.793
## 117           Total.Animals2  ~        Nat_Pop._WB20152  8.000     2.085
## 122             Sp.Richness2  ~        Brillouin.Index2  6.728     1.753
## 123             Sp.Richness2  ~        Mean.Raup.Crick2 15.051     3.922
## 125             Sp.Richness2  ~ Mean.Sp.BodyMassXAbund2  9.674     2.521
## 129             Sp.Richness2  ~        Nat_Pop._WB20152  3.059     0.797
## 130       Prop.Threat.Abund2  ~             Attendance2  6.215     1.619
## 131       Prop.Threat.Abund2  ~          Total.Animals2  5.628     1.467
## 132       Prop.Threat.Abund2  ~            Sp.Richness2 13.491     3.515
## 136       Prop.Threat.Abund2  ~          Prop.Mam.Abdun2  6.355     1.656
## 137       Prop.Threat.Abund2  ~ Mean.Sp.BodyMassXAbund2 14.759     3.846
## 138       Prop.Threat.Abund2  ~              X50km_Pop2 11.993     3.125
## 139       Prop.Threat.Abund2  ~              X10km_Pop2  7.595     1.979
## 141       Prop.Threat.Abund2  ~        Nat_Pop._WB20152  7.068     1.842
## 142       Prop.Threat.Abund2  ~            Prop.Mam.Sp2 19.197     5.002
## 143         Brillouin.Index2  ~             Attendance2  3.685     0.960
## 144         Brillouin.Index2  ~      Prop.Threat.Abund2 13.812     3.599
## 148         Brillouin.Index2  ~          Prop.Mam.Abdun2 13.803     3.597
## 149         Brillouin.Index2  ~ Mean.Sp.BodyMassXAbund2 10.704     2.789
## 151         Brillouin.Index2  ~              X10km_Pop2  4.078     1.063
## 152         Brillouin.Index2  ~            GDP.Millions2 58.529    15.251
## 153         Brillouin.Index2  ~        Nat_Pop._WB20152 50.588    13.182
## 154         Brillouin.Index2  ~            Prop.Mam.Sp2  9.791     2.551
## 155         Mean.Raup.Crick2  ~             Attendance2 11.294     2.943
## 158         Mean.Raup.Crick2  ~             Zoo.Area.ha2 10.638     2.772
## 160         Mean.Raup.Crick2  ~          Prop.Mam.Abdun2 12.228     3.186
## 161         Mean.Raup.Crick2  ~              X50km_Pop2 10.789     2.811
## 162         Mean.Raup.Crick2  ~              X10km_Pop2 11.524     3.003
## 164         Mean.Raup.Crick2  ~        Nat_Pop._WB20152 13.100     3.413
## 165         Mean.Raup.Crick2  ~            Prop.Mam.Sp2 37.344     9.731
## 166             Zoo.Area.ha2  ~             Attendance2 28.893     7.529
## 167             Zoo.Area.ha2  ~          Total.Animals2 31.171     8.122
## 168             Zoo.Area.ha2  ~            Sp.Richness2 22.366     5.828
## 171             Zoo.Area.ha2  ~        Mean.Raup.Crick2  9.532     2.484
## 180         Mam.Sp.Richness2  ~             Attendance2  5.629     1.467
## 181         Mam.Sp.Richness2  ~          Total.Animals2  4.983     1.298
## 182         Mam.Sp.Richness2  ~            Sp.Richness2 10.501     2.736
## 185         Mam.Sp.Richness2  ~        Mean.Raup.Crick2  4.862     1.267
## 194          Prop.Mam.Abdun2  ~             Attendance2  8.905     2.321
```

```
## 195          Prop.Mam.Abdun2  ~         Total.Animals2 11.568     3.014
## 197          Prop.Mam.Abdun2  ~      Prop.Threat.Abund2  3.479     0.906
## 198          Prop.Mam.Abdun2  ~       Brillouin.Index2  4.471     1.165
## 208 Mean.Sp.BodyMassXAbund2  ~           Attendance2 25.408     6.621
## 209 Mean.Sp.BodyMassXAbund2  ~         Total.Animals2 33.919     8.838
## 210 Mean.Sp.BodyMassXAbund2  ~           Sp.Richness2  7.245     1.888
## 211 Mean.Sp.BodyMassXAbund2  ~      Prop.Threat.Abund2  3.305     0.861
## 212 Mean.Sp.BodyMassXAbund2  ~       Brillouin.Index2  6.752     1.759
## 222              X50km_Pop2  ~           Attendance2 24.713     6.440
## 223              X50km_Pop2  ~         Total.Animals2 21.424     5.583
## 225              X50km_Pop2  ~      Prop.Threat.Abund2  3.359     0.875
## 227              X50km_Pop2  ~       Mean.Raup.Crick2  3.049     0.795
## 250            GDP.Millions2  ~           Attendance2 14.035     3.657
## 252            GDP.Millions2  ~           Sp.Richness2 29.232     7.617
## 254            GDP.Millions2  ~       Brillouin.Index2  3.642     0.949
## 255            GDP.Millions2  ~       Mean.Raup.Crick2 17.267     4.499
## 266         Nat_Pop._WB20152  ~           Sp.Richness2 30.273     7.888
## 268         Nat_Pop._WB20152  ~       Brillouin.Index2 16.786     4.374
## 269         Nat_Pop._WB20152  ~       Mean.Raup.Crick2 16.142     4.206
## 278             Prop.Mam.Sp2  ~           Attendance2  9.241     2.408
## 279             Prop.Mam.Sp2  ~         Total.Animals2 23.927     6.235
## 281             Prop.Mam.Sp2  ~      Prop.Threat.Abund2 10.458     2.725
## 282             Prop.Mam.Sp2  ~       Brillouin.Index2  5.246     1.367
## 283             Prop.Mam.Sp2  ~       Mean.Raup.Crick2  8.609     2.243
##        epc sepc.lv sepc.all sepc.nox
## 99  -0.194  -0.194   -0.198   -0.198
## 101  0.031   0.031    0.031    0.031
## 102 -0.094  -0.094   -0.096   -0.096
## 103  0.110   0.110    0.111    0.111
## 110  0.090   0.090    0.088    0.088
## 112 -0.084  -0.084   -0.084   -0.084
## 113 -0.142  -0.142   -0.142   -0.142
## 114  0.144   0.144    0.144    0.144
## 115  0.074   0.074    0.074    0.074
## 116 -0.128  -0.128   -0.128   -0.128
## 117 -0.066  -0.066   -0.066   -0.067
## 122  0.085   0.085    0.085    0.085
## 123 -0.081  -0.081   -0.079   -0.079
## 125 -0.081  -0.081   -0.081   -0.081
## 129 -0.032  -0.032   -0.032   -0.032
## 130  0.144   0.144    0.144    0.144
## 131 -0.128  -0.128   -0.128   -0.128
## 132 -0.279  -0.279   -0.279   -0.279
## 136  0.128   0.128    0.128    0.128
## 137  0.182   0.182    0.182    0.182
## 138  0.163   0.163    0.163    0.163
## 139  0.133   0.133    0.133    0.133
## 141  0.124   0.124    0.124    0.124
## 142  0.226   0.226    0.226    0.227
## 143 -0.084  -0.084   -0.084   -0.084
## 144  0.112   0.112    0.112    0.112
## 148  0.107   0.107    0.107    0.107
## 149  0.086   0.086    0.086    0.086
## 151 -0.054  -0.054   -0.054   -0.054
```

```
## 152 -0.193  -0.193   -0.193   -0.193
## 153 -0.179  -0.179   -0.179   -0.180
## 154  0.092   0.092    0.092    0.092
## 155  0.291   0.291    0.295    0.295
## 158  0.172   0.172    0.176    0.176
## 160 -0.196  -0.196   -0.199   -0.200
## 161  0.136   0.136    0.138    0.138
## 162  0.146   0.146    0.149    0.149
## 164  0.146   0.146    0.149    0.149
## 165 -0.370  -0.370   -0.377   -0.378
## 166  0.875   0.875    0.873    0.873
## 167  0.569   0.569    0.569    0.569
## 168  0.894   0.894    0.894    0.894
## 171  0.134   0.134    0.132    0.132
## 180 -0.388  -0.388   -0.387   -0.387
## 181 -0.216  -0.216   -0.216   -0.216
## 182 -0.783  -0.783   -0.783   -0.783
## 185 -0.128  -0.128   -0.125   -0.125
## 194 -0.138  -0.138   -0.137   -0.137
## 195 -0.124  -0.124   -0.124   -0.124
## 197 -0.040  -0.040   -0.040   -0.040
## 198  0.067   0.067    0.067    0.067
## 208 -0.472  -0.472   -0.471   -0.471
## 209 -0.311  -0.311   -0.311   -0.311
## 210 -0.218  -0.218   -0.218   -0.218
## 211  0.058   0.058    0.058    0.058
## 212  0.121   0.121    0.121    0.121
## 222  0.466   0.466    0.465    0.465
## 223  0.234   0.234    0.234    0.234
## 225  0.055   0.055    0.055    0.055
## 227  0.059   0.059    0.058    0.058
## 250 -0.282  -0.282   -0.281   -0.281
## 252  0.332   0.332    0.332    0.332
## 254  0.067   0.067    0.067    0.067
## 255 -0.112  -0.112   -0.110   -0.110
## 266 -0.326  -0.326   -0.326   -0.326
## 268 -0.139  -0.139   -0.139   -0.139
## 269  0.104   0.104    0.103    0.103
## 278  0.121   0.121    0.121    0.121
## 279  0.185   0.185    0.185    0.185
## 281  0.070   0.070    0.070    0.070
## 282 -0.075  -0.075   -0.075   -0.075
## 283 -0.072  -0.072   -0.071   -0.071
```

Model 4

Based on the modification indices generated from the third model, we can see that **Brillouin.Index2** ~ **GDP.Millions2** has an mi value of **58.529**. This is the highest mi value and far exceeds the standard cut-off level for the chi-square test criterion of 3.84 (Burnham and Anderson, 2002). This also makes theoretical sense, as it is logical to assume that countries with a higher GDP can afford to have much more diverse zoological collections. There is likely a historical element also at play here, however this is beyond what is deducible from the data available. As a result, we add this relationship to our model. Once again, the model summary, fit indices and modification indices were all generated for the model, adjusting for the nested

nature of data..

```
# Attendance SEM (Species Abundance)

# Model 4
# Addition of Brillouin.Index2 ~ GDP.Millions2, mi = 58.529

mod.4A <- 'Attendance2 ~ Zoo.Area.ha2 + Sp.Richness2 + Total.Animals2
+ Mam.Sp.Richness2 + Prop.Mam.Abdun2 + Prop.Threat.Abund2
+ Mean.Sp.BodyMassXAbund2 + Brillouin.Index2 + Mean.Raup.Crick2
+ X50km_Pop2 + X10km_Pop2 + GDP.Millions2 + Nat_Pop._WB20152

Total.Animals2 ~ Zoo.Area.ha2 + Sp.Richness2
Sp.Richness2 ~ Zoo.Area.ha2 + Prop.Mam.Sp2 + Mam.Sp.Richness2
Prop.Threat.Abund2 ~ Brillouin.Index2
Brillouin.Index2 ~ Sp.Richness2 + Total.Animals2 + GDP.Millions2
Mean.Raup.Crick2 ~ Sp.Richness2 + Total.Animals2 + Mean.Sp.BodyMassXAbund2'

# Fit model and generate model summary
mod.4A.fit <- sem(mod.4A, data = sem_attendance_data, fixed.x=FALSE)
summary(mod.4A.fit, rsq = TRUE)
```

```
## lavaan (0.5-23.1097) converged normally after  64 iterations
##
##   Number of observations                           458
##
##   Estimator                                         ML
##   Minimum Function Test Statistic              383.360
##   Degrees of freedom                                44
##   P-value (Chi-square)                           0.000
##
## Parameter Estimates:
##
##   Information                                 Expected
##   Standard Errors                             Standard
##
## Regressions:
##                        Estimate  Std.Err  z-value  P(>|z|)
##   Attendance2 ~
##     Zoo.Area.ha2          0.082    0.034    2.450    0.014
##     Sp.Richness2         -0.280    0.091   -3.062    0.002
##     Total.Animals2        0.548    0.058    9.432    0.000
##     Mam.Sp.Rchnss2        0.146    0.048    3.058    0.002
##     Prop.Mam.Abdn2       -0.100    0.040   -2.495    0.013
##     Prp.Thrt.Abnd2        0.017    0.024    0.700    0.484
##     Mn.Sp.BdyMsXA2        0.324    0.037    8.749    0.000
##     Brilloun.Indx2        0.025    0.049    0.524    0.601
##     Mean.Rap.Crck2        0.116    0.028    4.117    0.000
##     X50km_Pop2            0.073    0.038    1.911    0.056
##     X10km_Pop2            0.417    0.041   10.141    0.000
##     GDP.Millions2         0.263    0.049    5.414    0.000
##     Nt_Pp._WB20152       -0.128    0.049   -2.603    0.009
##   Total.Animals2 ~
##     Zoo.Area.ha2          0.223    0.024    9.384    0.000
##     Sp.Richness2          0.798    0.024   33.520    0.000
```

```
##   Sp.Richness2 ~
##     Zoo.Area.ha2          0.067    0.021    3.225    0.001
##     Prop.Mam.Sp2         -0.581    0.019  -30.364    0.000
##     Mam.Sp.Rchnss2        0.755    0.020   38.373    0.000
##   Prop.Threat.Abund2 ~
##     Brilloun.Indx2        0.107    0.046    2.358    0.018
##   Brillouin.Index2 ~
##     Sp.Richness2          1.320    0.043   30.864    0.000
##     Total.Animals2       -0.620    0.043  -14.507    0.000
##     GDP.Millions2        -0.206    0.023   -8.791    0.000
##   Mean.Raup.Crick2 ~
##     Sp.Richness2         -0.262    0.077   -3.414    0.001
##     Total.Animals2        0.400    0.076    5.294    0.000
##     Mn.Sp.BdyMsXA2       -0.446    0.042  -10.626    0.000
##
## Covariances:
##                            Estimate  Std.Err  z-value  P(>|z|)
##   Zoo.Area.ha2 ~~
##     Mam.Sp.Rchnss2            0.381    0.050    7.634    0.000
##     Prop.Mam.Abdn2            0.352    0.049    7.121    0.000
##     Mn.Sp.BdyMsXA2            0.522    0.053    9.923    0.000
##     X50km_Pop2                0.060    0.047    1.291    0.197
##     X10km_Pop2               -0.010    0.047   -0.208    0.835
##     GDP.Millions2            -0.027    0.047   -0.588    0.557
##     Nt_Pp._WB20152            0.061    0.047    1.310    0.190
##     Prop.Mam.Sp2              0.312    0.049    6.389    0.000
##   Mam.Sp.Richness2 ~~
##     Prop.Mam.Abdn2            0.058    0.047    1.237    0.216
##     Mn.Sp.BdyMsXA2            0.227    0.048    4.750    0.000
##     X50km_Pop2                0.204    0.048    4.281    0.000
##     X10km_Pop2                0.284    0.048    5.852    0.000
##     GDP.Millions2            -0.060    0.047   -1.277    0.202
##     Nt_Pp._WB20152           -0.088    0.047   -1.881    0.060
##     Prop.Mam.Sp2              0.088    0.047    1.882    0.060
##   Prop.Mam.Abdun2 ~~
##     Mn.Sp.BdyMsXA2            0.592    0.054   10.922    0.000
##     X50km_Pop2               -0.123    0.047   -2.622    0.009
##     X10km_Pop2               -0.248    0.048   -5.165    0.000
##     GDP.Millions2            -0.115    0.047   -2.458    0.014
##     Nt_Pp._WB20152           -0.111    0.047   -2.362    0.018
##     Prop.Mam.Sp2              0.878    0.062   14.135    0.000
##   Mean.Sp.BodyMassXAbund2 ~~
##     X50km_Pop2                0.013    0.047    0.275    0.784
##     X10km_Pop2                0.021    0.047    0.455    0.649
##     GDP.Millions2            -0.079    0.047   -1.682    0.093
##     Nt_Pp._WB20152           -0.004    0.047   -0.079    0.937
##     Prop.Mam.Sp2              0.628    0.055   11.405    0.000
##   X50km_Pop2 ~~
##     X10km_Pop2                0.752    0.058   12.886    0.000
##     GDP.Millions2            -0.013    0.047   -0.281    0.779
##     Nt_Pp._WB20152            0.191    0.047    4.033    0.000
##     Prop.Mam.Sp2             -0.096    0.047   -2.054    0.040
##   X10km_Pop2 ~~
##     GDP.Millions2            -0.028    0.047   -0.604    0.546
```

```
##     Nt_Pp._WB20152              0.173    0.047    3.646    0.000
##     Prop.Mam.Sp2               -0.204    0.048   -4.293    0.000
##   GDP.Millions2 ~~
##     Nt_Pp._WB20152              0.826    0.061   13.642    0.000
##     Prop.Mam.Sp2               -0.123    0.047   -2.615    0.009
##   Nat_Pop._WB20152 ~~
##     Prop.Mam.Sp2               -0.115    0.047   -2.447    0.014
##
## Variances:
##                    Estimate  Std.Err  z-value  P(>|z|)
##    .Attendance2       0.270    0.018   15.133    0.000
##    .Total.Animals2    0.251    0.017   15.133    0.000
##    .Sp.Richness2      0.151    0.010   15.133    0.000
##    .Prp.Thrt.Abnd2    0.986    0.065   15.133    0.000
##    .Brilloun.Indx2    0.251    0.017   15.133    0.000
##    .Mean.Rap.Crck2    0.744    0.049   15.133    0.000
##     Zoo.Area.ha2      0.998    0.066   15.133    0.000
##     Mam.Sp.Rchnss2    0.998    0.066   15.133    0.000
##     Prop.Mam.Abdn2    0.998    0.066   15.133    0.000
##     Mn.Sp.BdyMsXA2    0.998    0.066   15.133    0.000
##     X50km_Pop2        0.998    0.066   15.133    0.000
##     X10km_Pop2        0.998    0.066   15.133    0.000
##     GDP.Millions2     0.998    0.066   15.133    0.000
##     Nt_Pp._WB20152    0.998    0.066   15.133    0.000
##     Prop.Mam.Sp2      0.998    0.066   15.133    0.000
##
## R-Square:
##                    Estimate
##     Attendance2       0.727
##     Total.Animals2    0.748
##     Sp.Richness2      0.849
##     Prp.Thrt.Abnd2    0.012
##     Brilloun.Indx2    0.759
##     Mean.Rap.Crck2    0.227
```

```
# Generate fit indices
fitMeasures(mod.4A.fit, c("agfi", "rmr", "srmr", "rmsea", "cfi", "nnfi", "tli"))
```

```
##  agfi   rmr  srmr rmsea   cfi  nnfi   tli
## 0.746 0.062 0.062 0.130 0.893 0.833 0.833
```

```
# Generate modification indices
mi4A <- modindices(mod.4A.fit)
print(mi4A[mi4A$mi > 3.0,])
```

```
##                         lhs op                     rhs     mi    epc
## 84           Total.Animals2 ~~        Brillouin.Index2  5.591 -0.069
## 85           Total.Animals2 ~~        Mean.Raup.Crick2 10.639 -0.194
## 87             Sp.Richness2 ~~        Brillouin.Index2 12.470  0.035
## 88             Sp.Richness2 ~~        Mean.Raup.Crick2 30.292 -0.094
## 89       Prop.Threat.Abund2 ~~        Brillouin.Index2 21.809  0.125
## 95           Total.Animals2  ~        Brillouin.Index2  5.096  0.184
## 96           Total.Animals2  ~        Mean.Raup.Crick2  3.175  0.090
## 98           Total.Animals2  ~          Prop.Mam.Abdun2  7.869 -0.084
## 99           Total.Animals2  ~ Mean.Sp.BodyMassXAbund2 24.303 -0.142
## 100          Total.Animals2  ~              X50km_Pop2 35.809  0.144
```

```
## 101          Total.Animals2  ~              X10km_Pop2  8.873  0.074
## 102          Total.Animals2  ~          GDP.Millions2 29.909 -0.128
## 103          Total.Animals2  ~        Nat_Pop._WB20152  8.000 -0.066
## 108            Sp.Richness2  ~        Brillouin.Index2  5.177  0.071
## 109            Sp.Richness2  ~        Mean.Raup.Crick2 15.050 -0.081
## 111            Sp.Richness2  ~ Mean.Sp.BodyMassXAbund2  9.674 -0.081
## 115            Sp.Richness2  ~        Nat_Pop._WB20152  3.059 -0.032
## 116      Prop.Threat.Abund2  ~             Attendance2  6.016  0.140
## 117      Prop.Threat.Abund2  ~          Total.Animals2  5.403 -0.122
## 118      Prop.Threat.Abund2  ~            Sp.Richness2 12.906 -0.267
## 122      Prop.Threat.Abund2  ~          Prop.Mam.Abdun2  6.249  0.126
## 123      Prop.Threat.Abund2  ~ Mean.Sp.BodyMassXAbund2 14.720  0.181
## 124      Prop.Threat.Abund2  ~              X50km_Pop2 11.997  0.163
## 125      Prop.Threat.Abund2  ~              X10km_Pop2  7.617  0.133
## 127      Prop.Threat.Abund2  ~        Nat_Pop._WB20152  7.283  0.127
## 128      Prop.Threat.Abund2  ~            Prop.Mam.Sp2 18.789  0.222
## 130        Brillouin.Index2  ~      Prop.Threat.Abund2 21.809  0.126
## 132        Brillouin.Index2  ~            Zoo.Area.ha2  5.591  0.061
## 134        Brillouin.Index2  ~          Prop.Mam.Abdun2 10.810  0.088
## 135        Brillouin.Index2  ~ Mean.Sp.BodyMassXAbund2  9.147  0.074
## 137        Brillouin.Index2  ~              X10km_Pop2  5.633 -0.059
## 139        Brillouin.Index2  ~            Prop.Mam.Sp2  7.549  0.076
## 140        Mean.Raup.Crick2  ~             Attendance2 11.377  0.293
## 143        Mean.Raup.Crick2  ~            Zoo.Area.ha2 10.638  0.172
## 145        Mean.Raup.Crick2  ~          Prop.Mam.Abdun2 12.228 -0.196
## 146        Mean.Raup.Crick2  ~              X50km_Pop2 10.789  0.136
## 147        Mean.Raup.Crick2  ~              X10km_Pop2 11.524  0.146
## 149        Mean.Raup.Crick2  ~        Nat_Pop._WB20152 13.100  0.146
## 150        Mean.Raup.Crick2  ~            Prop.Mam.Sp2 37.344 -0.370
## 151            Zoo.Area.ha2  ~             Attendance2 29.525  0.887
## 152            Zoo.Area.ha2  ~          Total.Animals2 31.171  0.569
## 153            Zoo.Area.ha2  ~            Sp.Richness2 22.367  0.894
## 156            Zoo.Area.ha2  ~        Mean.Raup.Crick2  9.532  0.134
## 165        Mam.Sp.Richness2  ~             Attendance2  5.775 -0.394
## 166        Mam.Sp.Richness2  ~          Total.Animals2  4.983 -0.216
## 167        Mam.Sp.Richness2  ~            Sp.Richness2 10.501 -0.783
## 170        Mam.Sp.Richness2  ~        Mean.Raup.Crick2  4.862 -0.128
## 179         Prop.Mam.Abdun2  ~             Attendance2  8.937 -0.138
## 180         Prop.Mam.Abdun2  ~          Total.Animals2 11.568 -0.124
## 182         Prop.Mam.Abdun2  ~      Prop.Threat.Abund2  3.479 -0.040
## 183         Prop.Mam.Abdun2  ~        Brillouin.Index2  4.564  0.068
## 193 Mean.Sp.BodyMassXAbund2  ~             Attendance2 25.578 -0.475
## 194 Mean.Sp.BodyMassXAbund2  ~          Total.Animals2 33.919 -0.311
## 195 Mean.Sp.BodyMassXAbund2  ~            Sp.Richness2  7.245 -0.218
## 196 Mean.Sp.BodyMassXAbund2  ~      Prop.Threat.Abund2  3.306  0.058
## 197 Mean.Sp.BodyMassXAbund2  ~        Brillouin.Index2  6.891  0.123
## 207              X50km_Pop2  ~             Attendance2 24.898  0.470
## 208              X50km_Pop2  ~          Total.Animals2 21.424  0.234
## 210              X50km_Pop2  ~      Prop.Threat.Abund2  3.360  0.055
## 212              X50km_Pop2  ~        Mean.Raup.Crick2  3.049  0.059
## 235           GDP.Millions2  ~             Attendance2 12.724 -0.269
## 237           GDP.Millions2  ~            Sp.Richness2 29.233  0.332
## 239           GDP.Millions2  ~        Brillouin.Index2 32.618  0.219
## 240           GDP.Millions2  ~        Mean.Raup.Crick2 17.267 -0.112
```

```
## 251        Nat_Pop._WB20152  ~           Sp.Richness2 30.274 -0.326
## 253        Nat_Pop._WB20152  ~       Brillouin.Index2 17.134 -0.142
## 254        Nat_Pop._WB20152  ~       Mean.Raup.Crick2 16.142  0.104
## 263            Prop.Mam.Sp2  ~            Attendance2  9.289  0.122
## 264            Prop.Mam.Sp2  ~         Total.Animals2 23.928  0.185
## 266            Prop.Mam.Sp2  ~     Prop.Threat.Abund2 10.511  0.070
## 267            Prop.Mam.Sp2  ~       Brillouin.Index2  4.820 -0.073
## 268            Prop.Mam.Sp2  ~       Mean.Raup.Crick2  8.609 -0.072
##     sepc.lv sepc.all sepc.nox
## 84   -0.069   -0.068   -0.068
## 85   -0.194   -0.198   -0.198
## 87    0.035    0.034    0.034
## 88   -0.094   -0.096   -0.096
## 89    0.125    0.122    0.122
## 95    0.184    0.188    0.188
## 96    0.090    0.088    0.088
## 98   -0.084   -0.084   -0.084
## 99   -0.142   -0.142   -0.142
## 100   0.144    0.144    0.144
## 101   0.074    0.074    0.074
## 102  -0.128   -0.128   -0.128
## 103  -0.066   -0.066   -0.067
## 108   0.071    0.073    0.073
## 109  -0.081   -0.079   -0.079
## 111  -0.081   -0.081   -0.081
## 115  -0.032   -0.032   -0.032
## 116   0.140    0.139    0.139
## 117  -0.122   -0.122   -0.122
## 118  -0.267   -0.267   -0.267
## 122   0.126    0.126    0.126
## 123   0.181    0.181    0.181
## 124   0.163    0.163    0.163
## 125   0.133    0.133    0.134
## 127   0.127    0.127    0.127
## 128   0.222    0.221    0.222
## 130   0.126    0.124    0.124
## 132   0.061    0.060    0.060
## 134   0.088    0.086    0.087
## 135   0.074    0.072    0.073
## 137  -0.059   -0.058   -0.058
## 139   0.076    0.074    0.075
## 140   0.293    0.297    0.297
## 143   0.172    0.176    0.176
## 145  -0.196   -0.199   -0.200
## 146   0.136    0.138    0.138
## 147   0.146    0.149    0.149
## 149   0.146    0.149    0.149
## 150  -0.370   -0.377   -0.378
## 151   0.887    0.884    0.884
## 152   0.569    0.569    0.569
## 153   0.894    0.894    0.894
## 156   0.134    0.132    0.132
## 165  -0.394   -0.393   -0.393
## 166  -0.216   -0.216   -0.216
```

```
## 167  -0.783   -0.783   -0.783
## 170  -0.128   -0.125   -0.125
## 179  -0.138   -0.138   -0.138
## 180  -0.124   -0.124   -0.124
## 182  -0.040   -0.040   -0.040
## 183   0.068    0.070    0.070
## 193  -0.475   -0.473   -0.473
## 194  -0.311   -0.311   -0.311
## 195  -0.218   -0.218   -0.218
## 196   0.058    0.058    0.058
## 197   0.123    0.126    0.126
## 207   0.470    0.468    0.468
## 208   0.234    0.234    0.234
## 210   0.055    0.055    0.055
## 212   0.059    0.058    0.058
## 235  -0.269   -0.268   -0.268
## 237   0.332    0.332    0.332
## 239   0.219    0.224    0.224
## 240  -0.112   -0.110   -0.110
## 251  -0.326   -0.326   -0.326
## 253  -0.142   -0.145   -0.145
## 254   0.104    0.103    0.103
## 263   0.122    0.121    0.121
## 264   0.185    0.185    0.185
## 266   0.070    0.070    0.070
## 267  -0.073   -0.074   -0.074
## 268  -0.072   -0.071   -0.071
```

```r
# Adjust for the nested nature of the data (institutions within countries)
# Fit model and generate model summary
design <- svydesign(ids = ~Country, nest=TRUE, data=sem_attendance_data)
fit.adj4A <- lavaan.survey(lavaan.fit = mod.4A.fit, survey.design = design)
summary(fit.adj4A, rsq = TRUE)
```

```
## lavaan (0.5-23.1097) converged normally after  68 iterations
##
##   Number of observations                           458
##
##   Estimator                                         ML      Robust
##   Minimum Function Test Statistic              383.360     104.290
##   Degrees of freedom                                44          44
##   P-value (Chi-square)                           0.000       0.000
##   Scaling correction factor                                  3.676
##     for the Satorra-Bentler correction
##
## Parameter Estimates:
##
##   Information                                 Expected
##   Standard Errors                           Robust.sem
##
## Regressions:
##                    Estimate  Std.Err  z-value  P(>|z|)
##   Attendance2 ~
##     Zoo.Area.ha2      0.082    0.039    2.104    0.035
##     Sp.Richness2     -0.280    0.097   -2.893    0.004
```

```
##     Total.Animals2        0.548    0.062    8.891    0.000
##     Mam.Sp.Rchnss2        0.146    0.042    3.447    0.001
##     Prop.Mam.Abdn2       -0.100    0.030   -3.308    0.001
##     Prp.Thrt.Abnd2        0.017    0.028    0.615    0.538
##     Mn.Sp.BdyMsXA2        0.324    0.027   12.021    0.000
##     Brilloun.Indx2        0.025    0.068    0.372    0.710
##     Mean.Rap.Crck2        0.116    0.031    3.713    0.000
##     X50km_Pop2            0.073    0.032    2.257    0.024
##     X10km_Pop2            0.417    0.043    9.718    0.000
##     GDP.Millions2         0.263    0.057    4.631    0.000
##     Nt_Pp._WB20152       -0.128    0.063   -2.039    0.041
##   Total.Animals2 ~
##     Zoo.Area.ha2          0.223    0.038    5.856    0.000
##     Sp.Richness2          0.798    0.048   16.678    0.000
##   Sp.Richness2 ~
##     Zoo.Area.ha2          0.067    0.044    1.519    0.129
##     Prop.Mam.Sp2         -0.581    0.038  -15.231    0.000
##     Mam.Sp.Rchnss2        0.755    0.068   11.066    0.000
##   Prop.Threat.Abund2 ~
##     Brilloun.Indx2        0.107    0.127    0.844    0.399
##   Brillouin.Index2 ~
##     Sp.Richness2          1.320    0.069   19.079    0.000
##     Total.Animals2       -0.620    0.102   -6.085    0.000
##     GDP.Millions2        -0.206    0.059   -3.492    0.000
##   Mean.Raup.Crick2 ~
##     Sp.Richness2         -0.262    0.238   -1.099    0.272
##     Total.Animals2        0.400    0.164    2.446    0.014
##     Mn.Sp.BdyMsXA2       -0.446    0.058   -7.716    0.000
##
## Covariances:
##                            Estimate  Std.Err  z-value  P(>|z|)
##   Zoo.Area.ha2 ~~
##     Mam.Sp.Rchnss2            0.381    0.064    5.985    0.000
##     Prop.Mam.Abdn2            0.352    0.092    3.822    0.000
##     Mn.Sp.BdyMsXA2            0.522    0.101    5.177    0.000
##     X50km_Pop2                0.060    0.069    0.880    0.379
##     X10km_Pop2               -0.010    0.078   -0.124    0.901
##     GDP.Millions2            -0.027    0.041   -0.673    0.501
##     Nt_Pp._WB20152            0.061    0.090    0.682    0.495
##     Prop.Mam.Sp2              0.312    0.089    3.510    0.000
##   Mam.Sp.Richness2 ~~
##     Prop.Mam.Abdn2            0.058    0.049    1.172    0.241
##     Mn.Sp.BdyMsXA2            0.227    0.078    2.921    0.003
##     X50km_Pop2                0.204    0.082    2.487    0.013
##     X10km_Pop2                0.284    0.066    4.278    0.000
##     GDP.Millions2            -0.060    0.064   -0.937    0.349
##     Nt_Pp._WB20152           -0.088    0.102   -0.863    0.388
##     Prop.Mam.Sp2              0.088    0.061    1.435    0.151
##   Prop.Mam.Abdun2 ~~
##     Mn.Sp.BdyMsXA2            0.592    0.109    5.445    0.000
##     X50km_Pop2               -0.123    0.054   -2.263    0.024
##     X10km_Pop2               -0.248    0.060   -4.154    0.000
##     GDP.Millions2            -0.115    0.101   -1.140    0.254
##     Nt_Pp._WB20152           -0.111    0.088   -1.264    0.206
```

```
##     Prop.Mam.Sp2              0.878    0.128    6.865    0.000
##   Mean.Sp.BodyMassXAbund2 ~~
##     X50km_Pop2                0.013    0.074    0.174    0.862
##     X10km_Pop2                0.021    0.064    0.329    0.742
##     GDP.Millions2            -0.079    0.038   -2.055    0.040
##     Nt_Pp._WB20152           -0.004    0.084   -0.044    0.965
##     Prop.Mam.Sp2              0.628    0.126    5.002    0.000
##   X50km_Pop2 ~~
##     X10km_Pop2                0.752    0.126    5.982    0.000
##     GDP.Millions2            -0.013    0.100   -0.131    0.896
##     Nt_Pp._WB20152            0.191    0.177    1.083    0.279
##     Prop.Mam.Sp2             -0.096    0.051   -1.888    0.059
##   X10km_Pop2 ~~
##     GDP.Millions2            -0.028    0.056   -0.505    0.613
##     Nt_Pp._WB20152            0.173    0.101    1.701    0.089
##     Prop.Mam.Sp2             -0.204    0.055   -3.685    0.000
##   GDP.Millions2 ~~
##     Nt_Pp._WB20152            0.826    0.202    4.089    0.000
##     Prop.Mam.Sp2             -0.123    0.105   -1.172    0.241
##   Nat_Pop._WB20152 ~~
##     Prop.Mam.Sp2             -0.115    0.091   -1.257    0.209
##
## Intercepts:
##                    Estimate  Std.Err  z-value  P(>|z|)
##    .Attendance2      -0.000    0.030   -0.000    1.000
##    .Total.Animals2   -0.000    0.104   -0.000    1.000
##    .Sp.Richness2      0.000    0.034    0.000    1.000
##    .Prp.Thrt.Abnd2   -0.000    0.077   -0.000    1.000
##    .Brilloun.Indx2   -0.000    0.055   -0.000    1.000
##    .Mean.Rap.Crck2    0.000    0.094    0.000    1.000
##     Zoo.Area.ha2      0.000    0.064    0.000    1.000
##     Mam.Sp.Rchnss2   -0.000    0.079   -0.000    1.000
##     Prop.Mam.Abdn2    0.000    0.113    0.000    1.000
##     Mn.Sp.BdyMsXA2    0.000    0.071    0.000    1.000
##     X50km_Pop2       -0.000    0.114   -0.000    1.000
##     X10km_Pop2        0.000    0.105    0.000    1.000
##     GDP.Millions2    -0.000    0.345   -0.000    1.000
##     Nt_Pp._WB20152    0.000    0.295    0.000    1.000
##     Prop.Mam.Sp2      0.000    0.118    0.000    1.000
##
## Variances:
##                    Estimate  Std.Err  z-value  P(>|z|)
##    .Attendance2       0.270    0.028    9.568    0.000
##    .Total.Animals2    0.251    0.044    5.729    0.000
##    .Sp.Richness2      0.151    0.046    3.269    0.001
##    .Prp.Thrt.Abnd2    0.986    0.133    7.389    0.000
##    .Brilloun.Indx2    0.251    0.053    4.691    0.000
##    .Mean.Rap.Crck2    0.744    0.068   10.938    0.000
##     Zoo.Area.ha2      0.998    0.112    8.886    0.000
##     Mam.Sp.Rchnss2    0.998    0.115    8.669    0.000
##     Prop.Mam.Abdn2    0.998    0.121    8.221    0.000
##     Mn.Sp.BdyMsXA2    0.998    0.160    6.219    0.000
##     X50km_Pop2        0.998    0.131    7.623    0.000
##     X10km_Pop2        0.998    0.174    5.745    0.000
```

```
##     GDP.Millions2     0.998    0.246    4.061    0.000
##     Nt_Pp._WB20152    0.998    0.225    4.439    0.000
##     Prop.Mam.Sp2      0.998    0.150    6.662    0.000
##
## R-Square:
##                    Estimate
##     Attendance2       0.727
##     Total.Animals2    0.748
##     Sp.Richness2      0.849
##     Prp.Thrt.Abnd2    0.012
##     Brilloun.Indx2    0.759
##     Mean.Rap.Crck2    0.227
```

```r
# Generate fit indices
fitMeasures(fit.adj4A, c("agfi", "rmr", "srmr", "rmsea", "cfi", "nnfi", "tli"))
```

```
##  agfi   rmr  srmr rmsea   cfi  nnfi   tli
## 0.714 0.062 0.058 0.130 0.893 0.833 0.833
```

```r
# Generate modification indices
mi4adjA <- modindices(fit.adj4A)
print(mi4adjA[mi4adjA$mi > 3.0,])
```

```
##                      lhs op                     rhs     mi mi.scaled
## 99        Total.Animals2 ~~       Brillouin.Index2  5.591     1.521
## 100       Total.Animals2 ~~       Mean.Raup.Crick2 10.638     2.894
## 102         Sp.Richness2 ~~       Brillouin.Index2 12.470     3.392
## 103         Sp.Richness2 ~~       Mean.Raup.Crick2 30.292     8.241
## 104     Prop.Threat.Abund2 ~~       Brillouin.Index2 21.810     5.933
## 110       Total.Animals2  ~       Brillouin.Index2  5.096     1.386
## 111       Total.Animals2  ~       Mean.Raup.Crick2  3.175     0.864
## 113       Total.Animals2  ~        Prop.Mam.Abdun2  7.869     2.141
## 114       Total.Animals2  ~ Mean.Sp.BodyMassXAbund2 24.303     6.611
## 115       Total.Animals2  ~             X50km_Pop2 35.809     9.742
## 116       Total.Animals2  ~             X10km_Pop2  8.873     2.414
## 117       Total.Animals2  ~          GDP.Millions2 29.909     8.136
## 118       Total.Animals2  ~       Nat_Pop._WB20152  8.000     2.176
## 123         Sp.Richness2  ~       Brillouin.Index2  5.177     1.408
## 124         Sp.Richness2  ~       Mean.Raup.Crick2 15.050     4.094
## 126         Sp.Richness2  ~ Mean.Sp.BodyMassXAbund2  9.674     2.632
## 130         Sp.Richness2  ~       Nat_Pop._WB20152  3.059     0.832
## 131   Prop.Threat.Abund2  ~            Attendance2  6.016     1.637
## 132   Prop.Threat.Abund2  ~         Total.Animals2  5.403     1.470
## 133   Prop.Threat.Abund2  ~           Sp.Richness2 12.906     3.511
## 137   Prop.Threat.Abund2  ~        Prop.Mam.Abdun2  6.249     1.700
## 138   Prop.Threat.Abund2  ~ Mean.Sp.BodyMassXAbund2 14.720     4.004
## 139   Prop.Threat.Abund2  ~             X50km_Pop2 11.997     3.264
## 140   Prop.Threat.Abund2  ~             X10km_Pop2  7.617     2.072
## 142   Prop.Threat.Abund2  ~       Nat_Pop._WB20152  7.283     1.981
## 143   Prop.Threat.Abund2  ~           Prop.Mam.Sp2 18.789     5.112
## 145     Brillouin.Index2  ~     Prop.Threat.Abund2 21.810     5.933
## 147     Brillouin.Index2  ~            Zoo.Area.ha2  5.591     1.521
## 149     Brillouin.Index2  ~        Prop.Mam.Abdun2 10.810     2.941
## 150     Brillouin.Index2  ~ Mean.Sp.BodyMassXAbund2  9.147     2.488
## 152     Brillouin.Index2  ~             X10km_Pop2  5.633     1.532
## 154     Brillouin.Index2  ~           Prop.Mam.Sp2  7.549     2.054
```

```
## 155        Mean.Raup.Crick2  ~            Attendance2 11.377     3.095
## 158        Mean.Raup.Crick2  ~            Zoo.Area.ha2 10.639     2.894
## 160        Mean.Raup.Crick2  ~        Prop.Mam.Abdun2 12.229     3.327
## 161        Mean.Raup.Crick2  ~            X50km_Pop2 10.789     2.935
## 162        Mean.Raup.Crick2  ~            X10km_Pop2 11.524     3.135
## 164        Mean.Raup.Crick2  ~       Nat_Pop._WB20152 13.100     3.564
## 165        Mean.Raup.Crick2  ~           Prop.Mam.Sp2 37.344    10.159
## 166            Zoo.Area.ha2  ~            Attendance2 29.525     8.032
## 167            Zoo.Area.ha2  ~         Total.Animals2 31.171     8.480
## 168            Zoo.Area.ha2  ~            Sp.Richness2 22.367     6.085
## 171            Zoo.Area.ha2  ~       Mean.Raup.Crick2  9.532     2.593
## 180        Mam.Sp.Richness2  ~            Attendance2  5.775     1.571
## 181        Mam.Sp.Richness2  ~         Total.Animals2  4.983     1.356
## 182        Mam.Sp.Richness2  ~            Sp.Richness2 10.501     2.857
## 185        Mam.Sp.Richness2  ~       Mean.Raup.Crick2  4.862     1.323
## 194         Prop.Mam.Abdun2  ~            Attendance2  8.937     2.431
## 195         Prop.Mam.Abdun2  ~         Total.Animals2 11.568     3.147
## 197         Prop.Mam.Abdun2  ~      Prop.Threat.Abund2  3.479     0.946
## 198         Prop.Mam.Abdun2  ~       Brillouin.Index2  4.564     1.242
## 208 Mean.Sp.BodyMassXAbund2  ~            Attendance2 25.578     6.958
## 209 Mean.Sp.BodyMassXAbund2  ~         Total.Animals2 33.919     9.227
## 210 Mean.Sp.BodyMassXAbund2  ~            Sp.Richness2  7.245     1.971
## 211 Mean.Sp.BodyMassXAbund2  ~      Prop.Threat.Abund2  3.306     0.899
## 212 Mean.Sp.BodyMassXAbund2  ~       Brillouin.Index2  6.891     1.875
## 222              X50km_Pop2  ~            Attendance2 24.898     6.773
## 223              X50km_Pop2  ~         Total.Animals2 21.424     5.828
## 225              X50km_Pop2  ~      Prop.Threat.Abund2  3.360     0.914
## 227              X50km_Pop2  ~       Mean.Raup.Crick2  3.049     0.829
## 250           GDP.Millions2  ~            Attendance2 12.724     3.461
## 252           GDP.Millions2  ~            Sp.Richness2 29.233     7.953
## 254           GDP.Millions2  ~       Brillouin.Index2 32.618     8.874
## 255           GDP.Millions2  ~       Mean.Raup.Crick2 17.267     4.697
## 266        Nat_Pop._WB20152  ~            Sp.Richness2 30.274     8.236
## 268        Nat_Pop._WB20152  ~       Brillouin.Index2 17.134     4.661
## 269        Nat_Pop._WB20152  ~       Mean.Raup.Crick2 16.142     4.391
## 278            Prop.Mam.Sp2  ~            Attendance2  9.289     2.527
## 279            Prop.Mam.Sp2  ~         Total.Animals2 23.928     6.509
## 281            Prop.Mam.Sp2  ~      Prop.Threat.Abund2 10.511     2.860
## 282            Prop.Mam.Sp2  ~       Brillouin.Index2  4.820     1.311
## 283            Prop.Mam.Sp2  ~       Mean.Raup.Crick2  8.609     2.342
##        epc sepc.lv sepc.all sepc.nox
## 99  -0.069  -0.069   -0.068   -0.068
## 100 -0.194  -0.194   -0.198   -0.198
## 102  0.035   0.035    0.034    0.034
## 103 -0.094  -0.094   -0.096   -0.096
## 104  0.125   0.125    0.122    0.122
## 110  0.184   0.184    0.188    0.188
## 111  0.090   0.090    0.088    0.088
## 113 -0.084  -0.084   -0.084   -0.084
## 114 -0.142  -0.142   -0.142   -0.142
## 115  0.144   0.144    0.144    0.144
## 116  0.074   0.074    0.074    0.074
## 117 -0.128  -0.128   -0.128   -0.128
## 118 -0.066  -0.066   -0.066   -0.067
```

```
## 123  0.071   0.071    0.073    0.073
## 124 -0.081  -0.081   -0.079   -0.079
## 126 -0.081  -0.081   -0.081   -0.081
## 130 -0.032  -0.032   -0.032   -0.032
## 131  0.140   0.140    0.139    0.139
## 132 -0.122  -0.122   -0.122   -0.122
## 133 -0.267  -0.267   -0.267   -0.267
## 137  0.126   0.126    0.126    0.126
## 138  0.181   0.181    0.181    0.181
## 139  0.163   0.163    0.163    0.163
## 140  0.133   0.133    0.133    0.134
## 142  0.127   0.127    0.127    0.127
## 143  0.222   0.222    0.221    0.222
## 145  0.126   0.126    0.124    0.124
## 147  0.061   0.061    0.060    0.060
## 149  0.088   0.088    0.086    0.087
## 150  0.074   0.074    0.072    0.073
## 152 -0.059  -0.059   -0.058   -0.058
## 154  0.076   0.076    0.074    0.075
## 155  0.293   0.293    0.297    0.297
## 158  0.172   0.172    0.176    0.176
## 160 -0.196  -0.196   -0.199   -0.200
## 161  0.136   0.136    0.138    0.138
## 162  0.146   0.146    0.149    0.149
## 164  0.146   0.146    0.149    0.149
## 165 -0.370  -0.370   -0.377   -0.378
## 166  0.887   0.887    0.884    0.884
## 167  0.569   0.569    0.569    0.569
## 168  0.894   0.894    0.894    0.894
## 171  0.134   0.134    0.132    0.132
## 180 -0.394  -0.394   -0.393   -0.393
## 181 -0.216  -0.216   -0.216   -0.216
## 182 -0.783  -0.783   -0.783   -0.783
## 185 -0.128  -0.128   -0.125   -0.125
## 194 -0.138  -0.138   -0.138   -0.138
## 195 -0.124  -0.124   -0.124   -0.124
## 197 -0.040  -0.040   -0.040   -0.040
## 198  0.068   0.068    0.070    0.070
## 208 -0.475  -0.475   -0.473   -0.473
## 209 -0.311  -0.311   -0.311   -0.311
## 210 -0.218  -0.218   -0.218   -0.218
## 211  0.058   0.058    0.058    0.058
## 212  0.123   0.123    0.126    0.126
## 222  0.470   0.470    0.468    0.468
## 223  0.234   0.234    0.234    0.234
## 225  0.055   0.055    0.055    0.055
## 227  0.059   0.059    0.058    0.058
## 250 -0.269  -0.269   -0.268   -0.268
## 252  0.332   0.332    0.332    0.332
## 254  0.219   0.219    0.224    0.224
## 255 -0.112  -0.112   -0.110   -0.110
## 266 -0.326  -0.326   -0.326   -0.326
## 268 -0.142  -0.142   -0.145   -0.145
## 269  0.104   0.104    0.103    0.103
```

```
## 278  0.122   0.122    0.121    0.121
## 279  0.185   0.185    0.185    0.185
## 281  0.070   0.070    0.070    0.070
## 282 -0.073  -0.073   -0.074   -0.074
## 283 -0.072  -0.072   -0.071   -0.071
```

Model 5

Based on the modification indices generated from the fourth model, we can see that **Mean.Raup.Crick2** ~ **Prop.Mam.Sp2** has an mi value of **37.344**. This is the highest mi value and far exceeds the standard cut-off level for the chi-square test criterion of 3.84 (Burnham and Anderson, 2002). This also makes theoretical sense, as contemporary zoological collections are taxonomically biased towards mammals, therefore if an institution has a high proportion of mammals it is more likely to have more overlap with other collections. As a result, we add this relationship to our model. Once again, the model summary, fit indices and modification indices were all generated for the model, adjusting for the nested nature of data.

```
# Attendance SEM (Species Abundance)

# Model 5
# Addition of Mean.Raup.Crick2 ~ Prop.Mam.Sp2, mi = 37.344

mod.5A <- 'Attendance2 ~ Zoo.Area.ha2 + Sp.Richness2 + Total.Animals2
+ Mam.Sp.Richness2 + Prop.Mam.Abdun2 + Prop.Threat.Abund2
+ Mean.Sp.BodyMassXAbund2 + Brillouin.Index2 + Mean.Raup.Crick2
+ X50km_Pop2 + X10km_Pop2 + GDP.Millions2 + Nat_Pop._WB20152

Total.Animals2 ~ Zoo.Area.ha2 + Sp.Richness2
Sp.Richness2 ~ Zoo.Area.ha2 + Prop.Mam.Sp2 + Mam.Sp.Richness2
Prop.Threat.Abund2 ~ Brillouin.Index2
Brillouin.Index2 ~ Sp.Richness2 + Total.Animals2 + GDP.Millions2
Mean.Raup.Crick2 ~ Sp.Richness2 + Total.Animals2 + Mean.Sp.BodyMassXAbund2 + Prop.Mam.Sp2'

# Fit model and generate model summary
mod.5A.fit <- sem(mod.5A, data = sem_attendance_data, fixed.x=FALSE)
summary(mod.5A.fit, rsq = TRUE)
```

```
## lavaan (0.5-23.1097) converged normally after  63 iterations
##
##   Number of observations                           458
##
##   Estimator                                         ML
##   Minimum Function Test Statistic              344.874
##   Degrees of freedom                                43
##   P-value (Chi-square)                           0.000
##
## Parameter Estimates:
##
##   Information                                 Expected
##   Standard Errors                             Standard
##
## Regressions:
##                    Estimate  Std.Err  z-value  P(>|z|)
##   Attendance2 ~
##     Zoo.Area.ha2      0.082    0.034    2.450    0.014
```

```
##     Sp.Richness2        -0.280    0.091   -3.061    0.002
##     Total.Animals2       0.548    0.059    9.343    0.000
##     Mam.Sp.Rchnss2       0.146    0.048    3.044    0.002
##     Prop.Mam.Abdn2      -0.100    0.040   -2.471    0.013
##     Prp.Thrt.Abnd2       0.017    0.024    0.700    0.484
##     Mn.Sp.BdyMsXA2       0.324    0.036    9.028    0.000
##     Brilloun.Indx2       0.025    0.049    0.524    0.601
##     Mean.Rap.Crck2       0.116    0.029    4.001    0.000
##     X50km_Pop2           0.073    0.038    1.911    0.056
##     X10km_Pop2           0.417    0.041   10.140    0.000
##     GDP.Millions2        0.263    0.049    5.414    0.000
##     Nt_Pp._WB20152      -0.128    0.049   -2.603    0.009
##   Total.Animals2 ~
##     Zoo.Area.ha2         0.223    0.024    9.384    0.000
##     Sp.Richness2         0.798    0.024   33.520    0.000
##   Sp.Richness2 ~
##     Zoo.Area.ha2         0.067    0.021    3.225    0.001
##     Prop.Mam.Sp2        -0.581    0.019  -30.364    0.000
##     Mam.Sp.Rchnss2       0.755    0.020   38.373    0.000
##   Prop.Threat.Abund2 ~
##     Brilloun.Indx2       0.107    0.046    2.358    0.018
##   Brillouin.Index2 ~
##     Sp.Richness2         1.320    0.043   30.864    0.000
##     Total.Animals2      -0.620    0.043  -14.507    0.000
##     GDP.Millions2       -0.206    0.023   -8.791    0.000
##   Mean.Raup.Crick2 ~
##     Sp.Richness2        -0.467    0.078   -5.977    0.000
##     Total.Animals2       0.478    0.073    6.581    0.000
##     Mn.Sp.BdyMsXA2      -0.246    0.052   -4.760    0.000
##     Prop.Mam.Sp2        -0.366    0.058   -6.300    0.000
##
## Covariances:
##                            Estimate  Std.Err  z-value  P(>|z|)
##   Zoo.Area.ha2 ~~
##     Mam.Sp.Rchnss2            0.381    0.050    7.634    0.000
##     Prop.Mam.Abdn2            0.352    0.049    7.121    0.000
##     Mn.Sp.BdyMsXA2            0.522    0.053    9.923    0.000
##     X50km_Pop2                0.060    0.047    1.291    0.197
##     X10km_Pop2               -0.010    0.047   -0.208    0.835
##     GDP.Millions2            -0.027    0.047   -0.588    0.557
##     Nt_Pp._WB20152            0.061    0.047    1.310    0.190
##     Prop.Mam.Sp2              0.312    0.049    6.389    0.000
##   Mam.Sp.Richness2 ~~
##     Prop.Mam.Abdn2            0.058    0.047    1.237    0.216
##     Mn.Sp.BdyMsXA2            0.227    0.048    4.750    0.000
##     X50km_Pop2                0.204    0.048    4.281    0.000
##     X10km_Pop2                0.284    0.048    5.852    0.000
##     GDP.Millions2            -0.060    0.047   -1.277    0.202
##     Nt_Pp._WB20152           -0.088    0.047   -1.881    0.060
##     Prop.Mam.Sp2              0.088    0.047    1.882    0.060
##   Prop.Mam.Abdun2 ~~
##     Mn.Sp.BdyMsXA2            0.592    0.054   10.922    0.000
##     X50km_Pop2               -0.123    0.047   -2.622    0.009
##     X10km_Pop2               -0.248    0.048   -5.165    0.000
```

```
##     GDP.Millions2              -0.115    0.047   -2.458    0.014
##     Nt_Pp._WB20152             -0.111    0.047   -2.362    0.018
##     Prop.Mam.Sp2                0.878    0.062   14.135    0.000
##   Mean.Sp.BodyMassXAbund2 ~~
##     X50km_Pop2                  0.013    0.047    0.275    0.784
##     X10km_Pop2                  0.021    0.047    0.455    0.649
##     GDP.Millions2              -0.079    0.047   -1.682    0.093
##     Nt_Pp._WB20152             -0.004    0.047   -0.079    0.937
##     Prop.Mam.Sp2                0.628    0.055   11.405    0.000
##   X50km_Pop2 ~~
##     X10km_Pop2                  0.752    0.058   12.886    0.000
##     GDP.Millions2              -0.013    0.047   -0.281    0.779
##     Nt_Pp._WB20152              0.191    0.047    4.033    0.000
##     Prop.Mam.Sp2               -0.096    0.047   -2.054    0.040
##   X10km_Pop2 ~~
##     GDP.Millions2              -0.028    0.047   -0.604    0.546
##     Nt_Pp._WB20152              0.173    0.047    3.646    0.000
##     Prop.Mam.Sp2               -0.204    0.048   -4.293    0.000
##   GDP.Millions2 ~~
##     Nt_Pp._WB20152              0.826    0.061   13.642    0.000
##     Prop.Mam.Sp2               -0.123    0.047   -2.615    0.009
##   Nat_Pop._WB20152 ~~
##     Prop.Mam.Sp2               -0.115    0.047   -2.447    0.014
##
## Variances:
##                    Estimate  Std.Err  z-value  P(>|z|)
##    .Attendance2       0.270    0.018   15.133    0.000
##    .Total.Animals2    0.251    0.017   15.133    0.000
##    .Sp.Richness2      0.151    0.010   15.133    0.000
##    .Prp.Thrt.Abnd2    0.986    0.065   15.133    0.000
##    .Brilloun.Indx2    0.251    0.017   15.133    0.000
##    .Mean.Rap.Crck2    0.684    0.045   15.133    0.000
##     Zoo.Area.ha2      0.998    0.066   15.133    0.000
##     Mam.Sp.Rchnss2    0.998    0.066   15.133    0.000
##     Prop.Mam.Abdn2    0.998    0.066   15.133    0.000
##     Mn.Sp.BdyMsXA2    0.998    0.066   15.133    0.000
##     X50km_Pop2        0.998    0.066   15.133    0.000
##     X10km_Pop2        0.998    0.066   15.133    0.000
##     GDP.Millions2     0.998    0.066   15.133    0.000
##     Nt_Pp._WB20152    0.998    0.066   15.133    0.000
##     Prop.Mam.Sp2      0.998    0.066   15.133    0.000
##
## R-Square:
##                    Estimate
##     Attendance2       0.729
##     Total.Animals2    0.748
##     Sp.Richness2      0.849
##     Prp.Thrt.Abnd2    0.012
##     Brilloun.Indx2    0.759
##     Mean.Rap.Crck2    0.298
```

```
# Generate fit indices
fitMeasures(mod.5A.fit, c("agfi", "rmr", "srmr", "rmsea", "cfi", "nnfi", "tli"))
```

```
##  agfi   rmr  srmr rmsea   cfi  nnfi   tli
```

```
## 0.768 0.057 0.058 0.124 0.905 0.848 0.848
```

```r
# Generate modification indices
mi5A <- modindices(mod.5A.fit)
print(mi5A[mi5A$mi > 3.0,])
```

```
##                        lhs op                    rhs     mi    epc
## 85           Total.Animals2 ~~        Brillouin.Index2  5.591 -0.069
## 86           Total.Animals2 ~~        Mean.Raup.Crick2 16.277 -0.233
## 88             Sp.Richness2 ~~        Brillouin.Index2 12.470  0.035
## 89             Sp.Richness2 ~~        Mean.Raup.Crick2 13.892 -0.063
## 90        Prop.Threat.Abund2 ~~        Brillouin.Index2 21.809  0.125
## 96           Total.Animals2  ~        Brillouin.Index2  5.096  0.184
## 99           Total.Animals2  ~         Prop.Mam.Abdun2  7.869 -0.084
## 100          Total.Animals2  ~ Mean.Sp.BodyMassXAbund2 24.303 -0.142
## 101          Total.Animals2  ~             X50km_Pop2 35.809  0.144
## 102          Total.Animals2  ~             X10km_Pop2  8.873  0.074
## 103          Total.Animals2  ~          GDP.Millions2 29.909 -0.128
## 104          Total.Animals2  ~        Nat_Pop._WB20152  8.000 -0.066
## 109            Sp.Richness2  ~        Brillouin.Index2  5.177  0.071
## 110            Sp.Richness2  ~        Mean.Raup.Crick2  7.711 -0.064
## 112            Sp.Richness2  ~ Mean.Sp.BodyMassXAbund2  9.674 -0.081
## 116            Sp.Richness2  ~        Nat_Pop._WB20152  3.059 -0.032
## 117       Prop.Threat.Abund2  ~            Attendance2  5.960  0.138
## 118       Prop.Threat.Abund2  ~          Total.Animals2  5.403 -0.122
## 119       Prop.Threat.Abund2  ~            Sp.Richness2 12.906 -0.267
## 123       Prop.Threat.Abund2  ~         Prop.Mam.Abdun2  6.249  0.126
## 124       Prop.Threat.Abund2  ~ Mean.Sp.BodyMassXAbund2 14.720  0.181
## 125       Prop.Threat.Abund2  ~             X50km_Pop2 11.997  0.163
## 126       Prop.Threat.Abund2  ~             X10km_Pop2  7.617  0.133
## 128       Prop.Threat.Abund2  ~        Nat_Pop._WB20152  7.283  0.127
## 129       Prop.Threat.Abund2  ~            Prop.Mam.Sp2 18.789  0.222
## 131         Brillouin.Index2  ~      Prop.Threat.Abund2 21.809  0.126
## 133         Brillouin.Index2  ~            Zoo.Area.ha2  5.591  0.061
## 135         Brillouin.Index2  ~         Prop.Mam.Abdun2 10.810  0.088
## 136         Brillouin.Index2  ~ Mean.Sp.BodyMassXAbund2  9.147  0.074
## 138         Brillouin.Index2  ~             X10km_Pop2  5.633 -0.059
## 140         Brillouin.Index2  ~            Prop.Mam.Sp2  7.549  0.076
## 141         Mean.Raup.Crick2  ~            Attendance2  8.767  0.244
## 144         Mean.Raup.Crick2  ~            Zoo.Area.ha2 16.276  0.207
## 145         Mean.Raup.Crick2  ~        Mam.Sp.Richness2 10.165  0.274
## 146         Mean.Raup.Crick2  ~         Prop.Mam.Abdun2  3.605  0.156
## 147         Mean.Raup.Crick2  ~             X50km_Pop2  8.649  0.117
## 148         Mean.Raup.Crick2  ~             X10km_Pop2  6.465  0.106
## 150         Mean.Raup.Crick2  ~        Nat_Pop._WB20152  7.559  0.108
## 151             Zoo.Area.ha2  ~            Attendance2 28.790  0.876
## 152             Zoo.Area.ha2  ~          Total.Animals2 31.171  0.569
## 153             Zoo.Area.ha2  ~            Sp.Richness2 22.367  0.894
## 156             Zoo.Area.ha2  ~        Mean.Raup.Crick2  9.238  0.137
## 166         Mam.Sp.Richness2  ~          Total.Animals2  4.983 -0.216
## 167         Mam.Sp.Richness2  ~            Sp.Richness2 10.502 -0.783
## 179          Prop.Mam.Abdun2  ~            Attendance2  9.027 -0.140
## 180          Prop.Mam.Abdun2  ~          Total.Animals2 11.568 -0.124
## 182          Prop.Mam.Abdun2  ~      Prop.Threat.Abund2  3.479 -0.040
## 183          Prop.Mam.Abdun2  ~        Brillouin.Index2  4.564  0.068
```

```
## 193 Mean.Sp.BodyMassXAbund2  ~           Attendance2 37.356 -0.579
## 194 Mean.Sp.BodyMassXAbund2  ~         Total.Animals2 33.919 -0.311
## 195 Mean.Sp.BodyMassXAbund2  ~           Sp.Richness2  7.245 -0.218
## 196 Mean.Sp.BodyMassXAbund2  ~      Prop.Threat.Abund2  3.306  0.058
## 197 Mean.Sp.BodyMassXAbund2  ~       Brillouin.Index2  6.891  0.123
## 198 Mean.Sp.BodyMassXAbund2  ~       Mean.Raup.Crick2 47.533 -0.501
## 207              X50km_Pop2  ~           Attendance2 24.673  0.465
## 208              X50km_Pop2  ~         Total.Animals2 21.424  0.234
## 210              X50km_Pop2  ~      Prop.Threat.Abund2  3.360  0.055
## 212              X50km_Pop2  ~       Mean.Raup.Crick2  3.223  0.062
## 235           GDP.Millions2  ~           Attendance2 12.607 -0.267
## 237           GDP.Millions2  ~           Sp.Richness2 29.232  0.332
## 239           GDP.Millions2  ~       Brillouin.Index2 32.618  0.219
## 240           GDP.Millions2  ~       Mean.Raup.Crick2 18.250 -0.118
## 251        Nat_Pop._WB20152  ~           Sp.Richness2 30.273 -0.326
## 253        Nat_Pop._WB20152  ~       Brillouin.Index2 17.134 -0.142
## 254        Nat_Pop._WB20152  ~       Mean.Raup.Crick2 17.060  0.110
## 263             Prop.Mam.Sp2  ~           Attendance2 13.604  0.149
## 264             Prop.Mam.Sp2  ~         Total.Animals2 23.928  0.185
## 266             Prop.Mam.Sp2  ~      Prop.Threat.Abund2 10.511  0.070
## 267             Prop.Mam.Sp2  ~       Brillouin.Index2  4.820 -0.073
##     sepc.lv sepc.all sepc.nox
## 85   -0.069   -0.068   -0.068
## 86   -0.233   -0.236   -0.236
## 88    0.035    0.034    0.034
## 89   -0.063   -0.064   -0.064
## 90    0.125    0.122    0.122
## 96    0.184    0.188    0.188
## 99   -0.084   -0.084   -0.084
## 100  -0.142   -0.142   -0.142
## 101   0.144    0.144    0.144
## 102   0.074    0.074    0.074
## 103  -0.128   -0.128   -0.128
## 104  -0.066   -0.066   -0.067
## 109   0.071    0.073    0.073
## 110  -0.064   -0.063   -0.063
## 112  -0.081   -0.081   -0.081
## 116  -0.032   -0.032   -0.032
## 117   0.138    0.138    0.138
## 118  -0.122   -0.122   -0.122
## 119  -0.267   -0.267   -0.267
## 123   0.126    0.126    0.126
## 124   0.181    0.181    0.181
## 125   0.163    0.163    0.163
## 126   0.133    0.133    0.134
## 128   0.127    0.127    0.127
## 129   0.222    0.221    0.222
## 131   0.126    0.124    0.124
## 133   0.061    0.060    0.060
## 135   0.088    0.086    0.087
## 136   0.074    0.072    0.073
## 138  -0.059   -0.058   -0.058
## 140   0.076    0.074    0.075
## 141   0.244    0.246    0.246
```

```
## 144   0.207    0.209    0.210
## 145   0.274    0.277    0.278
## 146   0.156    0.158    0.158
## 147   0.117    0.118    0.118
## 148   0.106    0.107    0.107
## 150   0.108    0.109    0.109
## 151   0.876    0.875    0.875
## 152   0.569    0.569    0.569
## 153   0.894    0.894    0.894
## 156   0.137    0.135    0.135
## 166  -0.216   -0.216   -0.216
## 167  -0.783   -0.783   -0.783
## 179  -0.140   -0.139   -0.139
## 180  -0.124   -0.124   -0.124
## 182  -0.040   -0.040   -0.040
## 183   0.068    0.070    0.070
## 193  -0.579   -0.579   -0.579
## 194  -0.311   -0.311   -0.311
## 195  -0.218   -0.218   -0.218
## 196   0.058    0.058    0.058
## 197   0.123    0.126    0.126
## 198  -0.501   -0.495   -0.495
## 207   0.465    0.465    0.465
## 208   0.234    0.234    0.234
## 210   0.055    0.055    0.055
## 212   0.062    0.061    0.061
## 235  -0.267   -0.267   -0.267
## 237   0.332    0.332    0.332
## 239   0.219    0.224    0.224
## 240  -0.118   -0.117   -0.117
## 251  -0.326   -0.326   -0.326
## 253  -0.142   -0.145   -0.145
## 254   0.110    0.109    0.109
## 263   0.149    0.149    0.149
## 264   0.185    0.185    0.185
## 266   0.070    0.070    0.070
## 267  -0.073   -0.074   -0.074
```

```
# Adjust for the nested nature of the data (institutions within countries)
# Fit model and generate model summary
design <- svydesign(ids = ~Country, nest=TRUE, data=sem_attendance_data)
fit.adj5A <- lavaan.survey(lavaan.fit = mod.5A.fit, survey.design = design)
summary(fit.adj5A, rsq = TRUE)
```

```
## lavaan (0.5-23.1097) converged normally after  66 iterations
##
##   Number of observations                           458
##
##   Estimator                                         ML      Robust
##   Minimum Function Test Statistic              344.874      98.813
##   Degrees of freedom                                43          43
##   P-value (Chi-square)                           0.000       0.000
##   Scaling correction factor                                  3.490
##     for the Satorra-Bentler correction
##
```

```
## Parameter Estimates:
##
##   Information                                 Expected
##   Standard Errors                           Robust.sem
##
## Regressions:
##                        Estimate  Std.Err  z-value  P(>|z|)
##   Attendance2 ~
##     Zoo.Area.ha2          0.082    0.039    2.105    0.035
##     Sp.Richness2         -0.280    0.096   -2.907    0.004
##     Total.Animals2        0.548    0.063    8.730    0.000
##     Mam.Sp.Rchnss2        0.146    0.042    3.478    0.001
##     Prop.Mam.Abdn2       -0.100    0.032   -3.119    0.002
##     Prp.Thrt.Abnd2        0.017    0.028    0.615    0.538
##     Mn.Sp.BdyMsXA2        0.324    0.025   12.913    0.000
##     Brilloun.Indx2        0.025    0.068    0.372    0.710
##     Mean.Rap.Crck2        0.116    0.034    3.439    0.001
##     X50km_Pop2            0.073    0.032    2.250    0.024
##     X10km_Pop2            0.417    0.043    9.679    0.000
##     GDP.Millions2         0.263    0.057    4.629    0.000
##     Nt_Pp._WB20152       -0.128    0.063   -2.039    0.041
##   Total.Animals2 ~
##     Zoo.Area.ha2          0.223    0.038    5.856    0.000
##     Sp.Richness2          0.798    0.048   16.678    0.000
##   Sp.Richness2 ~
##     Zoo.Area.ha2          0.067    0.044    1.519    0.129
##     Prop.Mam.Sp2         -0.581    0.038  -15.231    0.000
##     Mam.Sp.Rchnss2        0.755    0.068   11.066    0.000
##   Prop.Threat.Abund2 ~
##     Brilloun.Indx2        0.107    0.127    0.844    0.399
##   Brillouin.Index2 ~
##     Sp.Richness2          1.320    0.069   19.079    0.000
##     Total.Animals2       -0.620    0.102   -6.085    0.000
##     GDP.Millions2        -0.206    0.059   -3.492    0.000
##   Mean.Raup.Crick2 ~
##     Sp.Richness2         -0.467    0.187   -2.496    0.013
##     Total.Animals2        0.478    0.115    4.141    0.000
##     Mn.Sp.BdyMsXA2       -0.246    0.081   -3.024    0.002
##     Prop.Mam.Sp2         -0.366    0.078   -4.699    0.000
##
## Covariances:
##                              Estimate  Std.Err  z-value  P(>|z|)
##   Zoo.Area.ha2 ~~
##     Mam.Sp.Rchnss2              0.381    0.064    5.985    0.000
##     Prop.Mam.Abdn2              0.352    0.092    3.822    0.000
##     Mn.Sp.BdyMsXA2              0.522    0.101    5.177    0.000
##     X50km_Pop2                  0.060    0.069    0.880    0.379
##     X10km_Pop2                 -0.010    0.078   -0.124    0.901
##     GDP.Millions2              -0.027    0.041   -0.673    0.501
##     Nt_Pp._WB20152              0.061    0.090    0.682    0.495
##     Prop.Mam.Sp2                0.312    0.089    3.510    0.000
##   Mam.Sp.Richness2 ~~
##     Prop.Mam.Abdn2              0.058    0.049    1.172    0.241
##     Mn.Sp.BdyMsXA2              0.227    0.078    2.921    0.003
```

```
##     X50km_Pop2                0.204    0.082    2.487    0.013
##     X10km_Pop2                0.284    0.066    4.278    0.000
##     GDP.Millions2            -0.060    0.064   -0.937    0.349
##     Nt_Pp._WB20152           -0.088    0.102   -0.863    0.388
##     Prop.Mam.Sp2              0.088    0.061    1.435    0.151
##   Prop.Mam.Abdun2 ~~
##     Mn.Sp.BdyMsXA2            0.592    0.109    5.445    0.000
##     X50km_Pop2               -0.123    0.054   -2.263    0.024
##     X10km_Pop2               -0.248    0.060   -4.154    0.000
##     GDP.Millions2            -0.115    0.101   -1.140    0.254
##     Nt_Pp._WB20152           -0.111    0.088   -1.264    0.206
##     Prop.Mam.Sp2              0.878    0.128    6.865    0.000
##   Mean.Sp.BodyMassXAbund2 ~~
##     X50km_Pop2                0.013    0.074    0.174    0.862
##     X10km_Pop2                0.021    0.064    0.329    0.742
##     GDP.Millions2            -0.079    0.038   -2.055    0.040
##     Nt_Pp._WB20152           -0.004    0.084   -0.044    0.965
##     Prop.Mam.Sp2              0.628    0.126    5.002    0.000
##   X50km_Pop2 ~~
##     X10km_Pop2                0.752    0.126    5.982    0.000
##     GDP.Millions2            -0.013    0.100   -0.131    0.896
##     Nt_Pp._WB20152            0.191    0.177    1.083    0.279
##     Prop.Mam.Sp2             -0.096    0.051   -1.888    0.059
##   X10km_Pop2 ~~
##     GDP.Millions2            -0.028    0.056   -0.505    0.613
##     Nt_Pp._WB20152            0.173    0.101    1.701    0.089
##     Prop.Mam.Sp2             -0.204    0.055   -3.685    0.000
##   GDP.Millions2 ~~
##     Nt_Pp._WB20152            0.826    0.202    4.089    0.000
##     Prop.Mam.Sp2             -0.123    0.105   -1.172    0.241
##   Nat_Pop._WB20152 ~~
##     Prop.Mam.Sp2             -0.115    0.091   -1.257    0.209
##
## Intercepts:
##                    Estimate  Std.Err  z-value  P(>|z|)
##    .Attendance2      -0.000    0.030   -0.000    1.000
##    .Total.Animals2   -0.000    0.104   -0.000    1.000
##    .Sp.Richness2      0.000    0.034    0.000    1.000
##    .Prp.Thrt.Abnd2   -0.000    0.077   -0.000    1.000
##    .Brilloun.Indx2   -0.000    0.055   -0.000    1.000
##    .Mean.Rap.Crck2    0.000    0.076    0.000    1.000
##     Zoo.Area.ha2      0.000    0.064    0.000    1.000
##     Mam.Sp.Rchnss2   -0.000    0.079   -0.000    1.000
##     Prop.Mam.Abdn2    0.000    0.113    0.000    1.000
##     Mn.Sp.BdyMsXA2    0.000    0.071    0.000    1.000
##     X50km_Pop2       -0.000    0.114   -0.000    1.000
##     X10km_Pop2        0.000    0.105    0.000    1.000
##     GDP.Millions2    -0.000    0.345   -0.000    1.000
##     Nt_Pp._WB20152    0.000    0.295    0.000    1.000
##     Prop.Mam.Sp2      0.000    0.118    0.000    1.000
##
## Variances:
##                    Estimate  Std.Err  z-value  P(>|z|)
##    .Attendance2       0.270    0.028    9.568    0.000
```

```
##    .Total.Animals2    0.251    0.044    5.729    0.000
##    .Sp.Richness2      0.151    0.046    3.269    0.001
##    .Prp.Thrt.Abnd2    0.986    0.133    7.389    0.000
##    .Brilloun.Indx2    0.251    0.053    4.691    0.000
##    .Mean.Rap.Crck2    0.684    0.059   11.527    0.000
##     Zoo.Area.ha2      0.998    0.112    8.886    0.000
##     Mam.Sp.Rchnss2    0.998    0.115    8.669    0.000
##     Prop.Mam.Abdn2    0.998    0.121    8.221    0.000
##     Mn.Sp.BdyMsXA2    0.998    0.160    6.219    0.000
##     X50km_Pop2        0.998    0.131    7.623    0.000
##     X10km_Pop2        0.998    0.174    5.745    0.000
##     GDP.Millions2     0.998    0.246    4.061    0.000
##     Nt_Pp._WB20152    0.998    0.225    4.439    0.000
##     Prop.Mam.Sp2      0.998    0.150    6.662    0.000
##
## R-Square:
##                    Estimate
##     Attendance2       0.729
##     Total.Animals2    0.748
##     Sp.Richness2      0.849
##     Prp.Thrt.Abnd2    0.012
##     Brilloun.Indx2    0.759
##     Mean.Rap.Crck2    0.298
```

```
# Generate fit indices
fitMeasures(fit.adj5A, c("agfi", "rmr", "srmr", "rmsea", "cfi", "nnfi", "tli"))
```

```
##  agfi   rmr  srmr rmsea   cfi  nnfi   tli
## 0.739 0.057 0.054 0.124 0.905 0.848 0.848
```

```
# Generate modification indices
mi5adjA <- modindices(fit.adj5A)
print(mi5adjA[mi5adjA$mi > 3.0,])
```

```
##                         lhs op                    rhs     mi mi.scaled
## 100          Total.Animals2 ~~       Brillouin.Index2  5.591     1.602
## 101          Total.Animals2 ~~       Mean.Raup.Crick2 16.276     4.663
## 103            Sp.Richness2 ~~       Brillouin.Index2 12.470     3.573
## 104            Sp.Richness2 ~~       Mean.Raup.Crick2 13.892     3.980
## 105        Prop.Threat.Abund2 ~~       Brillouin.Index2 21.810     6.249
## 111          Total.Animals2  ~       Brillouin.Index2  5.096     1.460
## 114          Total.Animals2  ~         Prop.Mam.Abdun2  7.869     2.255
## 115          Total.Animals2  ~ Mean.Sp.BodyMassXAbund2 24.303     6.963
## 116          Total.Animals2  ~              X50km_Pop2 35.809    10.260
## 117          Total.Animals2  ~              X10km_Pop2  8.873     2.542
## 118          Total.Animals2  ~           GDP.Millions2 29.909     8.569
## 119          Total.Animals2  ~        Nat_Pop._WB20152  8.000     2.292
## 124            Sp.Richness2  ~       Brillouin.Index2  5.177     1.483
## 125            Sp.Richness2  ~       Mean.Raup.Crick2  7.711     2.209
## 127            Sp.Richness2  ~ Mean.Sp.BodyMassXAbund2  9.674     2.772
## 131            Sp.Richness2  ~        Nat_Pop._WB20152  3.059     0.877
## 132        Prop.Threat.Abund2  ~            Attendance2  5.960     1.708
## 133        Prop.Threat.Abund2  ~         Total.Animals2  5.403     1.548
## 134        Prop.Threat.Abund2  ~           Sp.Richness2 12.906     3.698
## 138        Prop.Threat.Abund2  ~         Prop.Mam.Abdun2  6.249     1.790
## 139        Prop.Threat.Abund2  ~ Mean.Sp.BodyMassXAbund2 14.720     4.217
```

```
## 140      Prop.Threat.Abund2  ~             X50km_Pop2 11.997     3.437
## 141      Prop.Threat.Abund2  ~             X10km_Pop2  7.617     2.182
## 143      Prop.Threat.Abund2  ~        Nat_Pop._WB20152  7.283     2.087
## 144      Prop.Threat.Abund2  ~           Prop.Mam.Sp2 18.789     5.384
## 146        Brillouin.Index2  ~      Prop.Threat.Abund2 21.810     6.249
## 148        Brillouin.Index2  ~           Zoo.Area.ha2  5.591     1.602
## 150        Brillouin.Index2  ~         Prop.Mam.Abdun2 10.810     3.097
## 151        Brillouin.Index2  ~ Mean.Sp.BodyMassXAbund2  9.147     2.621
## 153        Brillouin.Index2  ~             X10km_Pop2  5.633     1.614
## 155        Brillouin.Index2  ~           Prop.Mam.Sp2  7.549     2.163
## 156        Mean.Raup.Crick2  ~            Attendance2  8.767     2.512
## 159        Mean.Raup.Crick2  ~           Zoo.Area.ha2 16.276     4.663
## 160        Mean.Raup.Crick2  ~        Mam.Sp.Richness2 10.165     2.912
## 161        Mean.Raup.Crick2  ~         Prop.Mam.Abdun2  3.605     1.033
## 162        Mean.Raup.Crick2  ~             X50km_Pop2  8.649     2.478
## 163        Mean.Raup.Crick2  ~             X10km_Pop2  6.465     1.852
## 165        Mean.Raup.Crick2  ~        Nat_Pop._WB20152  7.559     2.166
## 166            Zoo.Area.ha2  ~            Attendance2 28.789     8.249
## 167            Zoo.Area.ha2  ~          Total.Animals2 31.171     8.931
## 168            Zoo.Area.ha2  ~            Sp.Richness2 22.367     6.409
## 171            Zoo.Area.ha2  ~        Mean.Raup.Crick2  9.238     2.647
## 181        Mam.Sp.Richness2  ~          Total.Animals2  4.983     1.428
## 182        Mam.Sp.Richness2  ~            Sp.Richness2 10.501     3.009
## 194         Prop.Mam.Abdun2  ~            Attendance2  9.027     2.586
## 195         Prop.Mam.Abdun2  ~          Total.Animals2 11.568     3.315
## 197         Prop.Mam.Abdun2  ~      Prop.Threat.Abund2  3.479     0.997
## 198         Prop.Mam.Abdun2  ~        Brillouin.Index2  4.564     1.308
## 208 Mean.Sp.BodyMassXAbund2  ~            Attendance2 37.355    10.703
## 209 Mean.Sp.BodyMassXAbund2  ~          Total.Animals2 33.919     9.718
## 210 Mean.Sp.BodyMassXAbund2  ~            Sp.Richness2  7.245     2.076
## 211 Mean.Sp.BodyMassXAbund2  ~      Prop.Threat.Abund2  3.306     0.947
## 212 Mean.Sp.BodyMassXAbund2  ~        Brillouin.Index2  6.891     1.974
## 213 Mean.Sp.BodyMassXAbund2  ~        Mean.Raup.Crick2 47.533    13.619
## 222              X50km_Pop2  ~            Attendance2 24.673     7.069
## 223              X50km_Pop2  ~          Total.Animals2 21.424     6.138
## 225              X50km_Pop2  ~      Prop.Threat.Abund2  3.360     0.963
## 227              X50km_Pop2  ~        Mean.Raup.Crick2  3.223     0.923
## 250           GDP.Millions2  ~            Attendance2 12.607     3.612
## 252           GDP.Millions2  ~            Sp.Richness2 29.233     8.376
## 254           GDP.Millions2  ~        Brillouin.Index2 32.618     9.346
## 255           GDP.Millions2  ~        Mean.Raup.Crick2 18.249     5.229
## 266        Nat_Pop._WB20152  ~            Sp.Richness2 30.274     8.674
## 268        Nat_Pop._WB20152  ~        Brillouin.Index2 17.134     4.909
## 269        Nat_Pop._WB20152  ~        Mean.Raup.Crick2 17.060     4.888
## 278            Prop.Mam.Sp2  ~            Attendance2 13.604     3.898
## 279            Prop.Mam.Sp2  ~          Total.Animals2 23.928     6.856
## 281            Prop.Mam.Sp2  ~      Prop.Threat.Abund2 10.511     3.012
## 282            Prop.Mam.Sp2  ~        Brillouin.Index2  4.820     1.381
##        epc sepc.lv sepc.all sepc.nox
## 100 -0.069  -0.069   -0.068   -0.068
## 101 -0.233  -0.233   -0.236   -0.236
## 103  0.035   0.035    0.034    0.034
## 104 -0.063  -0.063   -0.064   -0.064
## 105  0.125   0.125    0.122    0.122
```

```
## 111  0.184   0.184    0.188    0.188
## 114 -0.084  -0.084   -0.084   -0.084
## 115 -0.142  -0.142   -0.142   -0.142
## 116  0.144   0.144    0.144    0.144
## 117  0.074   0.074    0.074    0.074
## 118 -0.128  -0.128   -0.128   -0.128
## 119 -0.066  -0.066   -0.066   -0.067
## 124  0.071   0.071    0.073    0.073
## 125 -0.064  -0.064   -0.063   -0.063
## 127 -0.081  -0.081   -0.081   -0.081
## 131 -0.032  -0.032   -0.032   -0.032
## 132  0.138   0.138    0.138    0.138
## 133 -0.122  -0.122   -0.122   -0.122
## 134 -0.267  -0.267   -0.267   -0.267
## 138  0.126   0.126    0.126    0.126
## 139  0.181   0.181    0.181    0.181
## 140  0.163   0.163    0.163    0.163
## 141  0.133   0.133    0.133    0.134
## 143  0.127   0.127    0.127    0.127
## 144  0.222   0.222    0.221    0.222
## 146  0.126   0.126    0.124    0.124
## 148  0.061   0.061    0.060    0.060
## 150  0.088   0.088    0.086    0.087
## 151  0.074   0.074    0.072    0.073
## 153 -0.059  -0.059   -0.058   -0.058
## 155  0.076   0.076    0.074    0.075
## 156  0.244   0.244    0.246    0.246
## 159  0.207   0.207    0.209    0.210
## 160  0.274   0.274    0.277    0.278
## 161  0.156   0.156    0.158    0.158
## 162  0.117   0.117    0.118    0.118
## 163  0.106   0.106    0.107    0.107
## 165  0.108   0.108    0.109    0.109
## 166  0.876   0.876    0.875    0.875
## 167  0.569   0.569    0.569    0.569
## 168  0.894   0.894    0.894    0.894
## 171  0.137   0.137    0.135    0.135
## 181 -0.216  -0.216   -0.216   -0.216
## 182 -0.783  -0.783   -0.783   -0.783
## 194 -0.140  -0.140   -0.139   -0.139
## 195 -0.124  -0.124   -0.124   -0.124
## 197 -0.040  -0.040   -0.040   -0.040
## 198  0.068   0.068    0.070    0.070
## 208 -0.579  -0.579   -0.579   -0.579
## 209 -0.311  -0.311   -0.311   -0.311
## 210 -0.218  -0.218   -0.218   -0.218
## 211  0.058   0.058    0.058    0.058
## 212  0.123   0.123    0.126    0.126
## 213 -0.501  -0.501   -0.495   -0.495
## 222  0.465   0.465    0.465    0.465
## 223  0.234   0.234    0.234    0.234
## 225  0.055   0.055    0.055    0.055
## 227  0.062   0.062    0.061    0.061
## 250 -0.267  -0.267   -0.267   -0.267
```

```
## 252  0.332   0.332    0.332    0.332
## 254  0.219   0.219    0.224    0.224
## 255 -0.118  -0.118   -0.117   -0.117
## 266 -0.326  -0.326   -0.326   -0.326
## 268 -0.142  -0.142   -0.145   -0.145
## 269  0.110   0.110    0.109    0.109
## 278  0.149   0.149    0.149    0.149
## 279  0.185   0.185    0.185    0.185
## 281  0.070   0.070    0.070    0.070
## 282 -0.073  -0.073   -0.074   -0.074
```

Model 6

Based on the modification indices generated from the fifth model, we can see that **Total.Animals2** ~ **Mean.Sp.BodyMassXAbund2** has an mi value of **24.303**. This is one of the highest mi values and far exceeds the standard cut-off level for the chi-square test criterion of 3.84 (Burnham and Anderson, 2002). This is also a conceptually appealling addition, as it is logical to assume that if we increase the body mass of the species within a collection, then owing to their increased space requirements, the total number of animals capabale of being maintained would be reduced. As a result, we add this relationship to our model. Once again, the model summary, fit indices and modification indices were all generated for the model, adjusting for the nested nature of data.

```
# Attendance SEM (Species Abundance)

# Model 6
# Addition of Total.Animals2 ~ Mean.Sp.BodyMassXAbund2, mi = 24.303

mod.6A <- 'Attendance2 ~ Zoo.Area.ha2 + Sp.Richness2 + Total.Animals2
+ Mam.Sp.Richness2 + Prop.Mam.Abdun2 + Prop.Threat.Abund2
+ Mean.Sp.BodyMassXAbund2 + Brillouin.Index2 + Mean.Raup.Crick2
+ X50km_Pop2 + X10km_Pop2 + GDP.Millions2 + Nat_Pop._WB20152

Total.Animals2 ~ Zoo.Area.ha2 + Sp.Richness2 + Mean.Sp.BodyMassXAbund2
Sp.Richness2 ~ Zoo.Area.ha2 + Prop.Mam.Sp2 + Mam.Sp.Richness2
Prop.Threat.Abund2 ~ Brillouin.Index2
Brillouin.Index2 ~ Sp.Richness2 + Total.Animals2 + GDP.Millions2
Mean.Raup.Crick2 ~ Sp.Richness2 + Total.Animals2 + Mean.Sp.BodyMassXAbund2 + Prop.Mam.Sp2'

# Fit model and generate model summary
mod.6A.fit <- sem(mod.6A, data = sem_attendance_data, fixed.x=FALSE)
summary(mod.6A.fit, rsq = TRUE)
```

```
## lavaan (0.5-23.1097) converged normally after  66 iterations
##
##   Number of observations                           458
##
##   Estimator                                         ML
##   Minimum Function Test Statistic              319.027
##   Degrees of freedom                                42
##   P-value (Chi-square)                           0.000
##
## Parameter Estimates:
##
##   Information                                 Expected
```

```
##   Standard Errors                             Standard
##
## Regressions:
##                        Estimate  Std.Err  z-value  P(>|z|)
##   Attendance2 ~
##     Zoo.Area.ha2          0.082    0.035    2.331    0.020
##     Sp.Richness2         -0.280    0.091   -3.076    0.002
##     Total.Animals2        0.548    0.060    9.163    0.000
##     Mam.Sp.Rchnss2        0.146    0.048    3.044    0.002
##     Prop.Mam.Abdn2       -0.100    0.040   -2.471    0.013
##     Prp.Thrt.Abnd2        0.017    0.024    0.700    0.484
##     Mn.Sp.BdyMsXA2        0.324    0.037    8.845    0.000
##     Brilloun.Indx2        0.025    0.049    0.524    0.601
##     Mean.Rap.Crck2        0.116    0.029    4.001    0.000
##     X50km_Pop2            0.073    0.038    1.911    0.056
##     X10km_Pop2            0.417    0.041   10.140    0.000
##     GDP.Millions2         0.263    0.049    5.414    0.000
##     Nt_Pp._WB20152       -0.128    0.049   -2.603    0.009
##   Total.Animals2 ~
##     Zoo.Area.ha2          0.308    0.028   10.962    0.000
##     Sp.Richness2          0.754    0.024   31.108    0.000
##     Mn.Sp.BdyMsXA2       -0.147    0.028   -5.244    0.000
##   Sp.Richness2 ~
##     Zoo.Area.ha2          0.067    0.021    3.225    0.001
##     Prop.Mam.Sp2         -0.581    0.019  -30.364    0.000
##     Mam.Sp.Rchnss2        0.755    0.020   38.373    0.000
##   Prop.Threat.Abund2 ~
##     Brilloun.Indx2        0.107    0.045    2.367    0.018
##   Brillouin.Index2 ~
##     Sp.Richness2          1.320    0.043   31.053    0.000
##     Total.Animals2       -0.620    0.043  -14.530    0.000
##     GDP.Millions2        -0.206    0.023   -8.791    0.000
##   Mean.Raup.Crick2 ~
##     Sp.Richness2         -0.467    0.076   -6.147    0.000
##     Total.Animals2        0.478    0.071    6.753    0.000
##     Mn.Sp.BdyMsXA2       -0.246    0.051   -4.809    0.000
##     Prop.Mam.Sp2         -0.366    0.058   -6.292    0.000
##
## Covariances:
##                               Estimate  Std.Err  z-value  P(>|z|)
##   Zoo.Area.ha2 ~~
##     Mam.Sp.Rchnss2               0.381    0.050    7.634    0.000
##     Prop.Mam.Abdn2               0.352    0.049    7.121    0.000
##     Mn.Sp.BdyMsXA2               0.522    0.053    9.923    0.000
##     X50km_Pop2                   0.060    0.047    1.291    0.197
##     X10km_Pop2                  -0.010    0.047   -0.208    0.835
##     GDP.Millions2               -0.027    0.047   -0.588    0.557
##     Nt_Pp._WB20152               0.061    0.047    1.310    0.190
##     Prop.Mam.Sp2                 0.312    0.049    6.389    0.000
##   Mam.Sp.Richness2 ~~
##     Prop.Mam.Abdn2               0.058    0.047    1.237    0.216
##     Mn.Sp.BdyMsXA2               0.227    0.048    4.750    0.000
##     X50km_Pop2                   0.204    0.048    4.281    0.000
##     X10km_Pop2                   0.284    0.048    5.852    0.000
```

```
##     GDP.Millions2             -0.060    0.047   -1.276    0.202
##     Nt_Pp._WB20152            -0.088    0.047   -1.881    0.060
##     Prop.Mam.Sp2               0.088    0.047    1.882    0.060
##   Prop.Mam.Abdun2 ~~
##     Mn.Sp.BdyMsXA2             0.592    0.054   10.922    0.000
##     X50km_Pop2                -0.123    0.047   -2.622    0.009
##     X10km_Pop2                -0.248    0.048   -5.165    0.000
##     GDP.Millions2             -0.115    0.047   -2.458    0.014
##     Nt_Pp._WB20152            -0.111    0.047   -2.362    0.018
##     Prop.Mam.Sp2               0.878    0.062   14.135    0.000
##   Mean.Sp.BodyMassXAbund2 ~~
##     X50km_Pop2                 0.013    0.047    0.275    0.784
##     X10km_Pop2                 0.021    0.047    0.455    0.649
##     GDP.Millions2             -0.079    0.047   -1.682    0.093
##     Nt_Pp._WB20152            -0.004    0.047   -0.079    0.937
##     Prop.Mam.Sp2               0.628    0.055   11.405    0.000
##   X50km_Pop2 ~~
##     X10km_Pop2                 0.752    0.058   12.886    0.000
##     GDP.Millions2             -0.013    0.047   -0.281    0.779
##     Nt_Pp._WB20152             0.191    0.047    4.033    0.000
##     Prop.Mam.Sp2              -0.096    0.047   -2.054    0.040
##   X10km_Pop2 ~~
##     GDP.Millions2             -0.028    0.047   -0.604    0.546
##     Nt_Pp._WB20152             0.173    0.047    3.646    0.000
##     Prop.Mam.Sp2              -0.204    0.048   -4.293    0.000
##   GDP.Millions2 ~~
##     Nt_Pp._WB20152             0.826    0.061   13.642    0.000
##     Prop.Mam.Sp2              -0.123    0.047   -2.615    0.009
##   Nat_Pop._WB20152 ~~
##     Prop.Mam.Sp2              -0.115    0.047   -2.447    0.014
##
## Variances:
##                    Estimate  Std.Err  z-value  P(>|z|)
##    .Attendance2       0.270    0.018   15.133    0.000
##    .Total.Animals2    0.237    0.016   15.133    0.000
##    .Sp.Richness2      0.151    0.010   15.133    0.000
##    .Prp.Thrt.Abnd2    0.986    0.065   15.133    0.000
##    .Brilloun.Indx2    0.251    0.017   15.133    0.000
##    .Mean.Rap.Crck2    0.684    0.045   15.133    0.000
##     Zoo.Area.ha2      0.998    0.066   15.133    0.000
##     Mam.Sp.Rchnss2    0.998    0.066   15.133    0.000
##     Prop.Mam.Abdn2    0.998    0.066   15.133    0.000
##     Mn.Sp.BdyMsXA2    0.998    0.066   15.133    0.000
##     X50km_Pop2        0.998    0.066   15.133    0.000
##     X10km_Pop2        0.998    0.066   15.133    0.000
##     GDP.Millions2     0.998    0.066   15.133    0.000
##     Nt_Pp._WB20152    0.998    0.066   15.133    0.000
##     Prop.Mam.Sp2      0.998    0.066   15.133    0.000
##
## R-Square:
##                    Estimate
##     Attendance2       0.717
##     Total.Animals2    0.760
##     Sp.Richness2      0.849
```

```
##     Prp.Thrt.Abnd2    0.012
##     Brilloun.Indx2    0.760
##     Mean.Rap.Crck2    0.325
```

```r
# Generate fit indices
fitMeasures(mod.6A.fit, c("agfi", "rmr", "srmr", "rmsea", "cfi", "nnfi", "tli"))
```

```
##  agfi   rmr  srmr rmsea   cfi  nnfi   tli 
## 0.776 0.056 0.056 0.120 0.913 0.857 0.857
```

```r
# Generate modification indices
mi6A <- modindices(mod.6A.fit)
print(mi6A[mi6A$mi > 3.0,])
```

```
##                     lhs op                    rhs     mi    epc
## 87          Total.Animals2 ~~        Mean.Raup.Crick2 18.130 -0.178
## 89            Sp.Richness2 ~~        Brillouin.Index2 12.502  0.035
## 90            Sp.Richness2 ~~        Mean.Raup.Crick2 13.936 -0.063
## 91       Prop.Threat.Abund2 ~~        Brillouin.Index2 21.756  0.124
## 95          Total.Animals2  ~           Attendance2  3.148  0.086
## 97          Total.Animals2  ~        Brillouin.Index2 10.235  0.237
## 98          Total.Animals2  ~        Mean.Raup.Crick2 23.757 -0.250
## 99          Total.Animals2  ~        Mam.Sp.Richness2  3.597  0.074
## 101         Total.Animals2  ~             X50km_Pop2 41.510  0.151
## 102         Total.Animals2  ~             X10km_Pop2 15.682  0.097
## 103         Total.Animals2  ~           GDP.Millions2 35.428 -0.136
## 104         Total.Animals2  ~        Nat_Pop._WB20152 10.342 -0.074
## 105         Total.Animals2  ~            Prop.Mam.Sp2  5.715  0.083
## 109           Sp.Richness2  ~        Brillouin.Index2  3.580  0.059
## 110           Sp.Richness2  ~        Mean.Raup.Crick2  6.385 -0.058
## 112           Sp.Richness2  ~ Mean.Sp.BodyMassXAbund2  9.674 -0.081
## 116           Sp.Richness2  ~        Nat_Pop._WB20152  3.059 -0.032
## 117     Prop.Threat.Abund2  ~           Attendance2  6.432  0.149
## 118     Prop.Threat.Abund2  ~          Total.Animals2  5.427 -0.123
## 119     Prop.Threat.Abund2  ~            Sp.Richness2 12.929 -0.267
## 123     Prop.Threat.Abund2  ~         Prop.Mam.Abdun2  6.119  0.123
## 124     Prop.Threat.Abund2  ~ Mean.Sp.BodyMassXAbund2 14.448  0.178
## 125     Prop.Threat.Abund2  ~             X50km_Pop2 12.010  0.163
## 126     Prop.Threat.Abund2  ~             X10km_Pop2  7.664  0.134
## 128     Prop.Threat.Abund2  ~        Nat_Pop._WB20152  7.290  0.127
## 129     Prop.Threat.Abund2  ~            Prop.Mam.Sp2 18.280  0.216
## 131       Brillouin.Index2  ~      Prop.Threat.Abund2 21.755  0.126
## 133       Brillouin.Index2  ~            Zoo.Area.ha2  5.600  0.062
## 135       Brillouin.Index2  ~         Prop.Mam.Abdun2 10.534  0.086
## 136       Brillouin.Index2  ~ Mean.Sp.BodyMassXAbund2  8.695  0.070
## 138       Brillouin.Index2  ~             X10km_Pop2  5.649 -0.059
## 140       Brillouin.Index2  ~            Prop.Mam.Sp2  7.344  0.074
## 141       Mean.Raup.Crick2  ~           Attendance2  8.767  0.244
## 144       Mean.Raup.Crick2  ~            Zoo.Area.ha2 18.130  0.230
## 145       Mean.Raup.Crick2  ~        Mam.Sp.Richness2 10.166  0.274
## 146       Mean.Raup.Crick2  ~         Prop.Mam.Abdun2  3.614  0.156
## 147       Mean.Raup.Crick2  ~             X50km_Pop2  8.649  0.117
## 148       Mean.Raup.Crick2  ~             X10km_Pop2  6.474  0.106
## 150       Mean.Raup.Crick2  ~        Nat_Pop._WB20152  7.567  0.108
## 151           Zoo.Area.ha2  ~           Attendance2  6.650  0.607
## 152           Zoo.Area.ha2  ~          Total.Animals2  7.577  0.434
```

```
## 153           Zoo.Area.ha2  ~          Sp.Richness2 22.366  0.894
## 155           Zoo.Area.ha2  ~      Brillouin.Index2  4.268  0.150
## 156           Zoo.Area.ha2  ~      Mean.Raup.Crick2  5.626  0.113
## 167       Mam.Sp.Richness2  ~          Sp.Richness2 10.501 -0.783
## 179          Prop.Mam.Abdun2  ~           Attendance2  9.286 -0.144
## 180          Prop.Mam.Abdun2  ~        Total.Animals2 12.428 -0.133
## 182          Prop.Mam.Abdun2  ~     Prop.Threat.Abund2  3.479 -0.040
## 183          Prop.Mam.Abdun2  ~      Brillouin.Index2  4.548  0.068
## 193 Mean.Sp.BodyMassXAbund2  ~           Attendance2 12.893 -0.516
## 194 Mean.Sp.BodyMassXAbund2  ~        Total.Animals2 10.777 -0.259
## 195 Mean.Sp.BodyMassXAbund2  ~          Sp.Richness2  7.245 -0.218
## 198 Mean.Sp.BodyMassXAbund2  ~      Mean.Raup.Crick2 28.087 -0.448
## 207             X50km_Pop2  ~           Attendance2 26.223  0.495
## 208             X50km_Pop2  ~        Total.Animals2 23.017  0.251
## 210             X50km_Pop2  ~     Prop.Threat.Abund2  3.360  0.055
## 212             X50km_Pop2  ~      Mean.Raup.Crick2  3.234  0.062
## 235           GDP.Millions2  ~           Attendance2 13.403 -0.284
## 237           GDP.Millions2  ~          Sp.Richness2 29.232  0.332
## 239           GDP.Millions2  ~      Brillouin.Index2 32.484  0.219
## 240           GDP.Millions2  ~      Mean.Raup.Crick2 18.312 -0.119
## 251       Nat_Pop._WB20152  ~          Sp.Richness2 30.273 -0.326
## 253       Nat_Pop._WB20152  ~      Brillouin.Index2 17.074 -0.142
## 254       Nat_Pop._WB20152  ~      Mean.Raup.Crick2 17.119  0.111
## 263            Prop.Mam.Sp2  ~           Attendance2 11.903  0.141
## 264            Prop.Mam.Sp2  ~        Total.Animals2 21.355  0.182
## 266            Prop.Mam.Sp2  ~     Prop.Threat.Abund2 10.650  0.071
## 267            Prop.Mam.Sp2  ~      Brillouin.Index2  3.830 -0.065
##     sepc.lv sepc.all sepc.nox
## 87   -0.178   -0.178   -0.178
## 89    0.035    0.034    0.034
## 90   -0.063   -0.063   -0.063
## 91    0.124    0.122    0.122
## 95    0.086    0.085    0.085
## 97    0.237    0.244    0.244
## 98   -0.250   -0.253   -0.253
## 99    0.074    0.074    0.074
## 101   0.151    0.151    0.151
## 102   0.097    0.097    0.098
## 103  -0.136   -0.137   -0.137
## 104  -0.074   -0.074   -0.074
## 105   0.083    0.083    0.083
## 109   0.059    0.061    0.061
## 110  -0.058   -0.058   -0.058
## 112  -0.081   -0.081   -0.081
## 116  -0.032   -0.032   -0.032
## 117   0.149    0.146    0.146
## 118  -0.123   -0.122   -0.122
## 119  -0.267   -0.267   -0.267
## 123   0.123    0.123    0.123
## 124   0.178    0.178    0.178
## 125   0.163    0.163    0.164
## 126   0.134    0.134    0.134
## 128   0.127    0.127    0.127
## 129   0.216    0.215    0.216
```

```
## 131   0.126    0.123    0.123
## 133   0.062    0.060    0.060
## 135   0.086    0.084    0.084
## 136   0.070    0.069    0.069
## 138  -0.059   -0.058   -0.058
## 140   0.074    0.072    0.072
## 141   0.244    0.237    0.237
## 144   0.230    0.229    0.229
## 145   0.274    0.272    0.272
## 146   0.156    0.155    0.155
## 147   0.117    0.116    0.116
## 148   0.106    0.105    0.105
## 150   0.108    0.107    0.107
## 151   0.607    0.594    0.594
## 152   0.434    0.432    0.432
## 153   0.894    0.894    0.894
## 155   0.150    0.154    0.154
## 156   0.113    0.114    0.114
## 167  -0.783   -0.783   -0.783
## 179  -0.144   -0.140   -0.140
## 180  -0.133   -0.132   -0.132
## 182  -0.040   -0.040   -0.040
## 183   0.068    0.070    0.070
## 193  -0.516   -0.505   -0.505
## 194  -0.259   -0.257   -0.257
## 195  -0.218   -0.218   -0.218
## 198  -0.448   -0.452   -0.452
## 207   0.495    0.484    0.484
## 208   0.251    0.250    0.250
## 210   0.055    0.055    0.055
## 212   0.062    0.063    0.063
## 235  -0.284   -0.278   -0.278
## 237   0.332    0.332    0.332
## 239   0.219    0.224    0.224
## 240  -0.119   -0.119   -0.119
## 251  -0.326   -0.326   -0.326
## 253  -0.142   -0.145   -0.145
## 254   0.111    0.112    0.112
## 263   0.141    0.138    0.138
## 264   0.182    0.181    0.181
## 266   0.071    0.071    0.071
## 267  -0.065   -0.066   -0.066
```

```
# Adjust for the nested nature of the data (institutions within countries)
# Fit model and generate model summary
design <- svydesign(ids = ~Country, nest=TRUE, data=sem_attendance_data)
fit.adj6A <- lavaan.survey(lavaan.fit = mod.6A.fit, survey.design = design)
summary(fit.adj6A, rsq = TRUE)
```

```
## lavaan (0.5-23.1097) converged normally after  66 iterations
##
##   Number of observations                           458
##
##   Estimator                                         ML      Robust
##   Minimum Function Test Statistic              319.027      90.781
```

```
##   Degrees of freedom                                42          42
##   P-value (Chi-square)                           0.000       0.000
##   Scaling correction factor                                  3.514
##     for the Satorra-Bentler correction
##
## Parameter Estimates:
##
##   Information                                 Expected
##   Standard Errors                           Robust.sem
##
## Regressions:
##                        Estimate  Std.Err  z-value  P(>|z|)
##   Attendance2 ~
##     Zoo.Area.ha2          0.082    0.042    1.963    0.050
##     Sp.Richness2         -0.280    0.097   -2.874    0.004
##     Total.Animals2        0.548    0.066    8.247    0.000
##     Mam.Sp.Rchnss2        0.146    0.042    3.478    0.001
##     Prop.Mam.Abdn2       -0.100    0.032   -3.119    0.002
##     Prp.Thrt.Abnd2        0.017    0.028    0.615    0.538
##     Mn.Sp.BdyMsXA2        0.324    0.025   12.838    0.000
##     Brilloun.Indx2        0.025    0.068    0.372    0.710
##     Mean.Rap.Crck2        0.116    0.034    3.439    0.001
##     X50km_Pop2            0.073    0.032    2.250    0.024
##     X10km_Pop2            0.417    0.043    9.680    0.000
##     GDP.Millions2         0.263    0.057    4.629    0.000
##     Nt_Pp._WB20152       -0.128    0.063   -2.039    0.041
##   Total.Animals2 ~
##     Zoo.Area.ha2          0.308    0.045    6.866    0.000
##     Sp.Richness2          0.754    0.048   15.667    0.000
##     Mn.Sp.BdyMsXA2       -0.147    0.033   -4.400    0.000
##   Sp.Richness2 ~
##     Zoo.Area.ha2          0.067    0.044    1.519    0.129
##     Prop.Mam.Sp2         -0.581    0.038  -15.231    0.000
##     Mam.Sp.Rchnss2        0.755    0.068   11.066    0.000
##   Prop.Threat.Abund2 ~
##     Brilloun.Indx2        0.107    0.126    0.850    0.395
##   Brillouin.Index2 ~
##     Sp.Richness2          1.320    0.068   19.461    0.000
##     Total.Animals2       -0.620    0.101   -6.143    0.000
##     GDP.Millions2        -0.206    0.058   -3.524    0.000
##   Mean.Raup.Crick2 ~
##     Sp.Richness2         -0.467    0.185   -2.527    0.012
##     Total.Animals2        0.478    0.114    4.201    0.000
##     Mn.Sp.BdyMsXA2       -0.246    0.084   -2.943    0.003
##     Prop.Mam.Sp2         -0.366    0.079   -4.643    0.000
##
## Covariances:
##                              Estimate  Std.Err  z-value  P(>|z|)
##   Zoo.Area.ha2 ~~
##     Mam.Sp.Rchnss2              0.381    0.064    5.985    0.000
##     Prop.Mam.Abdn2              0.352    0.092    3.822    0.000
##     Mn.Sp.BdyMsXA2              0.522    0.101    5.177    0.000
##     X50km_Pop2                  0.060    0.069    0.880    0.379
##     X10km_Pop2                 -0.010    0.078   -0.124    0.901
```

```
##     GDP.Millions2            -0.027    0.041   -0.673    0.501
##     Nt_Pp._WB20152            0.061    0.090    0.682    0.495
##     Prop.Mam.Sp2              0.312    0.089    3.510    0.000
##   Mam.Sp.Richness2 ~~
##     Prop.Mam.Abdn2            0.058    0.049    1.172    0.241
##     Mn.Sp.BdyMsXA2            0.227    0.078    2.921    0.003
##     X50km_Pop2                0.204    0.082    2.487    0.013
##     X10km_Pop2                0.284    0.066    4.278    0.000
##     GDP.Millions2            -0.060    0.064   -0.937    0.349
##     Nt_Pp._WB20152           -0.088    0.102   -0.863    0.388
##     Prop.Mam.Sp2              0.088    0.061    1.435    0.151
##   Prop.Mam.Abdun2 ~~
##     Mn.Sp.BdyMsXA2            0.592    0.109    5.445    0.000
##     X50km_Pop2               -0.123    0.054   -2.263    0.024
##     X10km_Pop2               -0.248    0.060   -4.154    0.000
##     GDP.Millions2            -0.115    0.101   -1.140    0.254
##     Nt_Pp._WB20152           -0.111    0.088   -1.264    0.206
##     Prop.Mam.Sp2              0.878    0.128    6.865    0.000
##   Mean.Sp.BodyMassXAbund2 ~~
##     X50km_Pop2                0.013    0.074    0.174    0.862
##     X10km_Pop2                0.021    0.064    0.329    0.742
##     GDP.Millions2            -0.079    0.038   -2.055    0.040
##     Nt_Pp._WB20152           -0.004    0.084   -0.044    0.965
##     Prop.Mam.Sp2              0.628    0.126    5.002    0.000
##   X50km_Pop2 ~~
##     X10km_Pop2                0.752    0.126    5.982    0.000
##     GDP.Millions2            -0.013    0.100   -0.131    0.896
##     Nt_Pp._WB20152            0.191    0.177    1.083    0.279
##     Prop.Mam.Sp2             -0.096    0.051   -1.888    0.059
##   X10km_Pop2 ~~
##     GDP.Millions2            -0.028    0.056   -0.505    0.613
##     Nt_Pp._WB20152            0.173    0.101    1.701    0.089
##     Prop.Mam.Sp2             -0.204    0.055   -3.685    0.000
##   GDP.Millions2 ~~
##     Nt_Pp._WB20152            0.826    0.202    4.089    0.000
##     Prop.Mam.Sp2             -0.123    0.105   -1.172    0.241
##   Nat_Pop._WB20152 ~~
##     Prop.Mam.Sp2             -0.115    0.091   -1.257    0.209
##
## Intercepts:
##                    Estimate  Std.Err  z-value  P(>|z|)
##    .Attendance2      -0.000    0.030   -0.000    1.000
##    .Total.Animals2   -0.000    0.103   -0.000    1.000
##    .Sp.Richness2      0.000    0.034    0.000    1.000
##    .Prp.Thrt.Abnd2   -0.000    0.077   -0.000    1.000
##    .Brilloun.Indx2   -0.000    0.055   -0.000    1.000
##    .Mean.Rap.Crck2    0.000    0.076    0.000    1.000
##     Zoo.Area.ha2      0.000    0.064    0.000    1.000
##     Mam.Sp.Rchnss2   -0.000    0.079   -0.000    1.000
##     Prop.Mam.Abdn2    0.000    0.113    0.000    1.000
##     Mn.Sp.BdyMsXA2    0.000    0.071    0.000    1.000
##     X50km_Pop2       -0.000    0.114   -0.000    1.000
##     X10km_Pop2        0.000    0.105    0.000    1.000
##     GDP.Millions2    -0.000    0.345   -0.000    1.000
```

```
##     Nt_Pp._WB20152    0.000    0.295    0.000    1.000
##     Prop.Mam.Sp2      0.000    0.118    0.000    1.000
##
## Variances:
##                    Estimate  Std.Err  z-value  P(>|z|)
##    .Attendance2       0.270    0.028    9.568    0.000
##    .Total.Animals2    0.237    0.041    5.722    0.000
##    .Sp.Richness2      0.151    0.046    3.269    0.001
##    .Prp.Thrt.Abnd2    0.986    0.133    7.389    0.000
##    .Brilloun.Indx2    0.251    0.053    4.691    0.000
##    .Mean.Rap.Crck2    0.684    0.059   11.527    0.000
##     Zoo.Area.ha2      0.998    0.112    8.886    0.000
##     Mam.Sp.Rchnss2    0.998    0.115    8.669    0.000
##     Prop.Mam.Abdn2    0.998    0.121    8.221    0.000
##     Mn.Sp.BdyMsXA2    0.998    0.160    6.219    0.000
##     X50km_Pop2        0.998    0.131    7.623    0.000
##     X10km_Pop2        0.998    0.174    5.745    0.000
##     GDP.Millions2     0.998    0.246    4.061    0.000
##     Nt_Pp._WB20152    0.998    0.225    4.439    0.000
##     Prop.Mam.Sp2      0.998    0.150    6.662    0.000
##
## R-Square:
##                    Estimate
##     Attendance2       0.717
##     Total.Animals2    0.760
##     Sp.Richness2      0.849
##     Prp.Thrt.Abnd2    0.012
##     Brilloun.Indx2    0.760
##     Mean.Rap.Crck2    0.325
```

```
# Generate fit indices
fitMeasures(fit.adj6A, c("agfi", "rmr", "srmr", "rmsea", "cfi", "nnfi", "tli"))
```

```
##  agfi   rmr  srmr rmsea   cfi  nnfi   tli
## 0.748 0.056 0.053 0.120 0.913 0.857 0.857
```

```
# Generate modification indices
mi6adjA <- modindices(fit.adj6A)
print(mi6adjA[mi6adjA$mi > 3.0,])
```

```
##                         lhs op                 rhs     mi mi.scaled
## 102          Total.Animals2 ~~    Mean.Raup.Crick2 18.130     5.159
## 104            Sp.Richness2 ~~   Brillouin.Index2 12.502     3.558
## 105            Sp.Richness2 ~~    Mean.Raup.Crick2 13.936     3.966
## 106      Prop.Threat.Abund2 ~~   Brillouin.Index2 21.755     6.191
## 110          Total.Animals2  ~         Attendance2  3.149     0.896
## 112          Total.Animals2  ~   Brillouin.Index2 10.235     2.913
## 113          Total.Animals2  ~    Mean.Raup.Crick2 23.757     6.760
## 114          Total.Animals2  ~    Mam.Sp.Richness2  3.597     1.024
## 116          Total.Animals2  ~          X50km_Pop2 41.510    11.812
## 117          Total.Animals2  ~          X10km_Pop2 15.682     4.463
## 118          Total.Animals2  ~       GDP.Millions2 35.428    10.081
## 119          Total.Animals2  ~     Nat_Pop._WB20152 10.342     2.943
## 120          Total.Animals2  ~        Prop.Mam.Sp2  5.715     1.626
## 124            Sp.Richness2  ~   Brillouin.Index2  3.580     1.019
## 125            Sp.Richness2  ~    Mean.Raup.Crick2  6.385     1.817
```

```
## 127            Sp.Richness2  ~ Mean.Sp.BodyMassXAbund2  9.674     2.753
## 131            Sp.Richness2  ~        Nat_Pop._WB20152  3.059     0.871
## 132      Prop.Threat.Abund2  ~             Attendance2  6.432     1.830
## 133      Prop.Threat.Abund2  ~          Total.Animals2  5.427     1.544
## 134      Prop.Threat.Abund2  ~            Sp.Richness2 12.929     3.679
## 138      Prop.Threat.Abund2  ~          Prop.Mam.Abdun2  6.119     1.741
## 139      Prop.Threat.Abund2  ~ Mean.Sp.BodyMassXAbund2 14.448     4.111
## 140      Prop.Threat.Abund2  ~              X50km_Pop2 12.010     3.417
## 141      Prop.Threat.Abund2  ~              X10km_Pop2  7.664     2.181
## 143      Prop.Threat.Abund2  ~        Nat_Pop._WB20152  7.290     2.074
## 144      Prop.Threat.Abund2  ~            Prop.Mam.Sp2 18.280     5.202
## 146        Brillouin.Index2  ~      Prop.Threat.Abund2 21.755     6.191
## 148        Brillouin.Index2  ~            Zoo.Area.ha2  5.600     1.594
## 150        Brillouin.Index2  ~          Prop.Mam.Abdun2 10.534     2.998
## 151        Brillouin.Index2  ~ Mean.Sp.BodyMassXAbund2  8.695     2.474
## 153        Brillouin.Index2  ~              X10km_Pop2  5.649     1.607
## 155        Brillouin.Index2  ~            Prop.Mam.Sp2  7.343     2.090
## 156        Mean.Raup.Crick2  ~             Attendance2  8.767     2.495
## 159        Mean.Raup.Crick2  ~            Zoo.Area.ha2 18.130     5.159
## 160        Mean.Raup.Crick2  ~        Mam.Sp.Richness2 10.166     2.893
## 161        Mean.Raup.Crick2  ~          Prop.Mam.Abdun2  3.614     1.028
## 162        Mean.Raup.Crick2  ~              X50km_Pop2  8.649     2.461
## 163        Mean.Raup.Crick2  ~              X10km_Pop2  6.474     1.842
## 165        Mean.Raup.Crick2  ~        Nat_Pop._WB20152  7.567     2.153
## 166            Zoo.Area.ha2  ~             Attendance2  6.650     1.892
## 167            Zoo.Area.ha2  ~          Total.Animals2  7.578     2.156
## 168            Zoo.Area.ha2  ~            Sp.Richness2 22.368     6.365
## 170            Zoo.Area.ha2  ~        Brillouin.Index2  4.269     1.215
## 171            Zoo.Area.ha2  ~        Mean.Raup.Crick2  5.626     1.601
## 182        Mam.Sp.Richness2  ~            Sp.Richness2 10.501     2.988
## 194         Prop.Mam.Abdun2  ~             Attendance2  9.286     2.643
## 195         Prop.Mam.Abdun2  ~          Total.Animals2 12.428     3.537
## 197         Prop.Mam.Abdun2  ~      Prop.Threat.Abund2  3.479     0.990
## 198         Prop.Mam.Abdun2  ~        Brillouin.Index2  4.548     1.294
## 208 Mean.Sp.BodyMassXAbund2  ~             Attendance2 12.893     3.669
## 209 Mean.Sp.BodyMassXAbund2  ~          Total.Animals2 10.777     3.067
## 210 Mean.Sp.BodyMassXAbund2  ~            Sp.Richness2  7.245     2.062
## 213 Mean.Sp.BodyMassXAbund2  ~        Mean.Raup.Crick2 28.087     7.992
## 222              X50km_Pop2  ~             Attendance2 26.222     7.462
## 223              X50km_Pop2  ~          Total.Animals2 23.017     6.550
## 225              X50km_Pop2  ~      Prop.Threat.Abund2  3.360     0.956
## 227              X50km_Pop2  ~        Mean.Raup.Crick2  3.234     0.920
## 250            GDP.Millions2  ~             Attendance2 13.403     3.814
## 252            GDP.Millions2  ~            Sp.Richness2 29.232     8.318
## 254            GDP.Millions2  ~        Brillouin.Index2 32.484     9.244
## 255            GDP.Millions2  ~        Mean.Raup.Crick2 18.312     5.211
## 266        Nat_Pop._WB20152  ~            Sp.Richness2 30.273     8.614
## 268        Nat_Pop._WB20152  ~        Brillouin.Index2 17.074     4.858
## 269        Nat_Pop._WB20152  ~        Mean.Raup.Crick2 17.119     4.871
## 278            Prop.Mam.Sp2  ~             Attendance2 11.903     3.387
## 279            Prop.Mam.Sp2  ~          Total.Animals2 21.355     6.077
## 281            Prop.Mam.Sp2  ~      Prop.Threat.Abund2 10.650     3.030
## 282            Prop.Mam.Sp2  ~        Brillouin.Index2  3.830     1.090
##        epc sepc.lv sepc.all sepc.nox
```

```
## 102 -0.178  -0.178   -0.178   -0.178
## 104  0.035   0.035    0.034    0.034
## 105 -0.063  -0.063   -0.063   -0.063
## 106  0.124   0.124    0.122    0.122
## 110  0.086   0.086    0.085    0.085
## 112  0.237   0.237    0.244    0.244
## 113 -0.250  -0.250   -0.253   -0.253
## 114  0.074   0.074    0.074    0.074
## 116  0.151   0.151    0.151    0.151
## 117  0.097   0.097    0.097    0.098
## 118 -0.136  -0.136   -0.137   -0.137
## 119 -0.074  -0.074   -0.074   -0.074
## 120  0.083   0.083    0.083    0.083
## 124  0.059   0.059    0.061    0.061
## 125 -0.058  -0.058   -0.058   -0.058
## 127 -0.081  -0.081   -0.081   -0.081
## 131 -0.032  -0.032   -0.032   -0.032
## 132  0.149   0.149    0.146    0.146
## 133 -0.123  -0.123   -0.122   -0.122
## 134 -0.267  -0.267   -0.267   -0.267
## 138  0.123   0.123    0.123    0.123
## 139  0.178   0.178    0.178    0.178
## 140  0.163   0.163    0.163    0.164
## 141  0.134   0.134    0.134    0.134
## 143  0.127   0.127    0.127    0.127
## 144  0.216   0.216    0.215    0.216
## 146  0.126   0.126    0.123    0.123
## 148  0.062   0.062    0.060    0.060
## 150  0.086   0.086    0.084    0.084
## 151  0.070   0.070    0.069    0.069
## 153 -0.059  -0.059   -0.058   -0.058
## 155  0.074   0.074    0.072    0.072
## 156  0.244   0.244    0.237    0.237
## 159  0.230   0.230    0.229    0.229
## 160  0.274   0.274    0.272    0.272
## 161  0.156   0.156    0.155    0.155
## 162  0.117   0.117    0.116    0.116
## 163  0.106   0.106    0.105    0.105
## 165  0.108   0.108    0.107    0.107
## 166  0.607   0.607    0.594    0.594
## 167  0.434   0.434    0.432    0.432
## 168  0.894   0.894    0.894    0.894
## 170  0.150   0.150    0.154    0.154
## 171  0.113   0.113    0.114    0.114
## 182 -0.783  -0.783   -0.783   -0.783
## 194 -0.144  -0.144   -0.140   -0.140
## 195 -0.133  -0.133   -0.132   -0.132
## 197 -0.040  -0.040   -0.040   -0.040
## 198  0.068   0.068    0.070    0.070
## 208 -0.516  -0.516   -0.505   -0.505
## 209 -0.259  -0.259   -0.257   -0.257
## 210 -0.218  -0.218   -0.218   -0.218
## 213 -0.448  -0.448   -0.452   -0.452
## 222  0.495   0.495    0.484    0.484
```

```
## 223  0.251   0.251    0.250    0.250
## 225  0.055   0.055    0.055    0.055
## 227  0.062   0.062    0.063    0.063
## 250 -0.284  -0.284   -0.278   -0.278
## 252  0.332   0.332    0.332    0.332
## 254  0.219   0.219    0.224    0.224
## 255 -0.119  -0.119   -0.119   -0.119
## 266 -0.326  -0.326   -0.326   -0.326
## 268 -0.142  -0.142   -0.145   -0.145
## 269  0.111   0.111    0.112    0.112
## 278  0.141   0.141    0.138    0.138
## 279  0.182   0.182    0.181    0.181
## 281  0.071   0.071    0.071    0.071
## 282 -0.065  -0.065   -0.066   -0.066
```

Model 7

Based on the modification indices generated from the sixth model, we can see that **Total.Animals2 ~ GDP.Millions2** has an mi value of **35.428**. This is one of the highest mi values and far exceeds the standard cut-off level for the chi-square test criterion of 3.84 (Burnham and Anderson, 2002). Although not the highest mi value, this is the most intuitive and still exceeds the standard cut-off level for the chi-square test criterion of 3.84 (Burnham and Anderson, 2002). This is considered intuituive as it is logical to assume that countries with a higher GDP can afford to have zoological collections with many more animals. Again, the exact reasoning behind this is likely rooted in an historical context that exceeds the scope of this work. As a result, we add this relationship to our model. Once again, the model summary, fit indices and modification indices were all generated for the model, adjusting for the nested nature of data.

```
# Attendance SEM (Species Abundance)

# Model 7
# Addition of Total.Animals2 ~ GDP.Millions2, mi = 35.428

mod.7A <- 'Attendance2 ~ Zoo.Area.ha2 + Sp.Richness2 + Total.Animals2
+ Mam.Sp.Richness2 + Prop.Mam.Abdun2 + Prop.Threat.Abund2
+ Mean.Sp.BodyMassXAbund2 + Brillouin.Index2 + Mean.Raup.Crick2
+ X50km_Pop2 + X10km_Pop2 + GDP.Millions2 + Nat_Pop._WB20152

Total.Animals2 ~ Zoo.Area.ha2 + Sp.Richness2 + Mean.Sp.BodyMassXAbund2 + GDP.Millions2
Sp.Richness2 ~ Zoo.Area.ha2 + Prop.Mam.Sp2 + Mam.Sp.Richness2
Prop.Threat.Abund2 ~ Brillouin.Index2
Brillouin.Index2 ~ Sp.Richness2 + Total.Animals2 + GDP.Millions2
Mean.Raup.Crick2 ~ Sp.Richness2 + Total.Animals2 + Mean.Sp.BodyMassXAbund2 + Prop.Mam.Sp2'

# Fit model and generate model summary
mod.7A.fit <- sem(mod.7A, data = sem_attendance_data, fixed.x=FALSE)
summary(mod.7A.fit, rsq = TRUE)
```

```
## lavaan (0.5-23.1097) converged normally after  67 iterations
##
##   Number of observations                           458
##
##   Estimator                                         ML
##   Minimum Function Test Statistic              282.120
##   Degrees of freedom                                41
```

```
##   P-value (Chi-square)                           0.000
##
## Parameter Estimates:
##
##   Information                                 Expected
##   Standard Errors                             Standard
##
## Regressions:
##                          Estimate  Std.Err  z-value  P(>|z|)
##   Attendance2 ~
##     Zoo.Area.ha2            0.082    0.036    2.312    0.021
##     Sp.Richness2           -0.280    0.092   -3.051    0.002
##     Total.Animals2          0.548    0.062    8.908    0.000
##     Mam.Sp.Rchnss2          0.146    0.048    3.044    0.002
##     Prop.Mam.Abdn2         -0.100    0.040   -2.471    0.013
##     Prp.Thrt.Abnd2          0.017    0.024    0.700    0.484
##     Mn.Sp.BdyMsXA2          0.324    0.037    8.803    0.000
##     Brilloun.Indx2          0.025    0.049    0.524    0.601
##     Mean.Rap.Crck2          0.116    0.029    4.001    0.000
##     X50km_Pop2              0.073    0.038    1.911    0.056
##     X10km_Pop2              0.417    0.041   10.140    0.000
##     GDP.Millions2           0.263    0.049    5.358    0.000
##     Nt_Pp._WB20152         -0.128    0.049   -2.603    0.009
##   Total.Animals2 ~
##     Zoo.Area.ha2            0.309    0.027   11.444    0.000
##     Sp.Richness2            0.759    0.023   32.580    0.000
##     Mn.Sp.BdyMsXA2         -0.157    0.027   -5.828    0.000
##     GDP.Millions2          -0.136    0.022   -6.202    0.000
##   Sp.Richness2 ~
##     Zoo.Area.ha2            0.067    0.021    3.225    0.001
##     Prop.Mam.Sp2           -0.581    0.019  -30.364    0.000
##     Mam.Sp.Rchnss2          0.755    0.020   38.373    0.000
##   Prop.Threat.Abund2 ~
##     Brilloun.Indx2          0.107    0.046    2.324    0.020
##   Brillouin.Index2 ~
##     Sp.Richness2            1.320    0.044   30.207    0.000
##     Total.Animals2         -0.620    0.044  -14.076    0.000
##     GDP.Millions2          -0.206    0.024   -8.524    0.000
##   Mean.Raup.Crick2 ~
##     Sp.Richness2           -0.467    0.077   -6.082    0.000
##     Total.Animals2          0.478    0.071    6.732    0.000
##     Mn.Sp.BdyMsXA2         -0.246    0.051   -4.808    0.000
##     Prop.Mam.Sp2           -0.366    0.058   -6.278    0.000
##
## Covariances:
##                                Estimate  Std.Err  z-value  P(>|z|)
##   Zoo.Area.ha2 ~~
##     Mam.Sp.Rchnss2                0.381    0.050    7.634    0.000
##     Prop.Mam.Abdn2                0.352    0.049    7.121    0.000
##     Mn.Sp.BdyMsXA2                0.522    0.053    9.923    0.000
##     X50km_Pop2                    0.060    0.047    1.291    0.197
##     X10km_Pop2                   -0.010    0.047   -0.208    0.835
##     GDP.Millions2                -0.027    0.047   -0.588    0.557
##     Nt_Pp._WB20152                0.061    0.047    1.310    0.190
```

```
##     Prop.Mam.Sp2              0.312    0.049    6.389    0.000
##   Mam.Sp.Richness2 ~~
##     Prop.Mam.Abdn2            0.058    0.047    1.237    0.216
##     Mn.Sp.BdyMsXA2            0.227    0.048    4.750    0.000
##     X50km_Pop2                0.204    0.048    4.281    0.000
##     X10km_Pop2                0.284    0.048    5.852    0.000
##     GDP.Millions2            -0.060    0.047   -1.277    0.202
##     Nt_Pp._WB20152           -0.088    0.047   -1.881    0.060
##     Prop.Mam.Sp2              0.088    0.047    1.882    0.060
##   Prop.Mam.Abdun2 ~~
##     Mn.Sp.BdyMsXA2            0.592    0.054   10.922    0.000
##     X50km_Pop2               -0.123    0.047   -2.622    0.009
##     X10km_Pop2               -0.248    0.048   -5.165    0.000
##     GDP.Millions2            -0.115    0.047   -2.458    0.014
##     Nt_Pp._WB20152           -0.111    0.047   -2.362    0.018
##     Prop.Mam.Sp2              0.878    0.062   14.135    0.000
##   Mean.Sp.BodyMassXAbund2 ~~
##     X50km_Pop2                0.013    0.047    0.275    0.784
##     X10km_Pop2                0.021    0.047    0.455    0.649
##     GDP.Millions2            -0.079    0.047   -1.682    0.093
##     Nt_Pp._WB20152           -0.004    0.047   -0.079    0.937
##     Prop.Mam.Sp2              0.628    0.055   11.405    0.000
##   X50km_Pop2 ~~
##     X10km_Pop2                0.752    0.058   12.886    0.000
##     GDP.Millions2            -0.013    0.047   -0.281    0.779
##     Nt_Pp._WB20152            0.191    0.047    4.033    0.000
##     Prop.Mam.Sp2             -0.096    0.047   -2.054    0.040
##   X10km_Pop2 ~~
##     GDP.Millions2            -0.028    0.047   -0.604    0.546
##     Nt_Pp._WB20152            0.173    0.047    3.646    0.000
##     Prop.Mam.Sp2             -0.204    0.048   -4.293    0.000
##   GDP.Millions2 ~~
##     Nt_Pp._WB20152            0.826    0.061   13.642    0.000
##     Prop.Mam.Sp2             -0.123    0.047   -2.615    0.009
##   Nat_Pop._WB20152 ~~
##     Prop.Mam.Sp2             -0.115    0.047   -2.447    0.014
##
## Variances:
##                    Estimate  Std.Err  z-value  P(>|z|)
##    .Attendance2       0.270    0.018   15.133    0.000
##    .Total.Animals2    0.219    0.014   15.133    0.000
##    .Sp.Richness2      0.151    0.010   15.133    0.000
##    .Prp.Thrt.Abnd2    0.986    0.065   15.133    0.000
##    .Brilloun.Indx2    0.251    0.017   15.133    0.000
##    .Mean.Rap.Crck2    0.684    0.045   15.133    0.000
##     Zoo.Area.ha2      0.998    0.066   15.133    0.000
##     Mam.Sp.Rchnss2    0.998    0.066   15.133    0.000
##     Prop.Mam.Abdn2    0.998    0.066   15.133    0.000
##     Mn.Sp.BdyMsXA2    0.998    0.066   15.133    0.000
##     X50km_Pop2        0.998    0.066   15.133    0.000
##     X10km_Pop2        0.998    0.066   15.133    0.000
##     GDP.Millions2     0.998    0.066   15.133    0.000
##     Nt_Pp._WB20152    0.998    0.066   15.133    0.000
##     Prop.Mam.Sp2      0.998    0.066   15.133    0.000
```

```
##
## R-Square:
##                    Estimate
##     Attendance2       0.711
##     Total.Animals2    0.780
##     Sp.Richness2      0.849
##     Prp.Thrt.Abnd2    0.012
##     Brilloun.Indx2    0.752
##     Mean.Rap.Crck2    0.323
```

```
# Generate fit indices
fitMeasures(mod.7A.fit, c("agfi", "rmr", "srmr", "rmsea", "cfi", "nnfi", "tli"))
```

```
##  agfi   rmr  srmr rmsea   cfi  nnfi   tli
## 0.792 0.054 0.054 0.113 0.924 0.872 0.872
```

```
# Generate modification indices
mi7A <- modindices(mod.7A.fit)
print(mi7A[mi7A$mi > 3.0,])
```

```
##                       lhs op                    rhs     mi    epc
## 88         Total.Animals2 ~~        Mean.Raup.Crick2 14.977 -0.137
## 90           Sp.Richness2 ~~        Brillouin.Index2 12.507  0.035
## 91           Sp.Richness2 ~~        Mean.Raup.Crick2 13.951 -0.063
## 92       Prop.Threat.Abund2 ~~      Brillouin.Index2 22.014  0.126
## 96         Total.Animals2  ~             Attendance2 14.245  0.184
## 99         Total.Animals2  ~        Mean.Raup.Crick2 18.614 -0.194
## 102        Total.Animals2  ~             X50km_Pop2 43.332  0.148
## 103        Total.Animals2  ~             X10km_Pop2 15.014  0.091
## 104        Total.Animals2  ~        Nat_Pop._WB20152 10.674  0.129
## 105        Total.Animals2  ~            Prop.Mam.Sp2  3.693  0.064
## 109          Sp.Richness2  ~        Brillouin.Index2  4.137  0.067
## 110          Sp.Richness2  ~        Mean.Raup.Crick2  6.484 -0.058
## 112          Sp.Richness2  ~ Mean.Sp.BodyMassXAbund2  9.674 -0.081
## 116          Sp.Richness2  ~        Nat_Pop._WB20152  3.059 -0.032
## 117    Prop.Threat.Abund2  ~             Attendance2  6.854  0.159
## 118    Prop.Threat.Abund2  ~          Total.Animals2  5.660 -0.128
## 119    Prop.Threat.Abund2  ~            Sp.Richness2 13.620 -0.282
## 123    Prop.Threat.Abund2  ~          Prop.Mam.Abdun2  6.178  0.124
## 124    Prop.Threat.Abund2  ~ Mean.Sp.BodyMassXAbund2 14.457  0.178
## 125    Prop.Threat.Abund2  ~              X50km_Pop2 12.016  0.163
## 126    Prop.Threat.Abund2  ~              X10km_Pop2  7.675  0.134
## 128    Prop.Threat.Abund2  ~        Nat_Pop._WB20152  7.152  0.125
## 129    Prop.Threat.Abund2  ~            Prop.Mam.Sp2 18.492  0.218
## 131      Brillouin.Index2  ~      Prop.Threat.Abund2 22.014  0.128
## 133      Brillouin.Index2  ~             Zoo.Area.ha2  5.621  0.062
## 135      Brillouin.Index2  ~          Prop.Mam.Abdun2 10.518  0.086
## 136      Brillouin.Index2  ~ Mean.Sp.BodyMassXAbund2  8.684  0.070
## 138      Brillouin.Index2  ~              X10km_Pop2  5.652 -0.059
## 140      Brillouin.Index2  ~            Prop.Mam.Sp2  7.333  0.074
## 141      Mean.Raup.Crick2  ~             Attendance2  8.802  0.245
## 144      Mean.Raup.Crick2  ~             Zoo.Area.ha2 18.033  0.229
## 145      Mean.Raup.Crick2  ~        Mam.Sp.Richness2 10.168  0.274
## 146      Mean.Raup.Crick2  ~          Prop.Mam.Abdun2  3.617  0.156
## 147      Mean.Raup.Crick2  ~              X50km_Pop2  8.649  0.117
## 148      Mean.Raup.Crick2  ~              X10km_Pop2  6.466  0.106
```

```
## 150        Mean.Raup.Crick2  ~        Nat_Pop._WB20152  7.741  0.111
## 151            Zoo.Area.ha2  ~             Attendance2  6.508  0.612
## 152            Zoo.Area.ha2  ~          Total.Animals2  7.437  0.440
## 153            Zoo.Area.ha2  ~            Sp.Richness2 22.367  0.894
## 155            Zoo.Area.ha2  ~        Brillouin.Index2  4.448  0.155
## 156            Zoo.Area.ha2  ~        Mean.Raup.Crick2  5.584  0.113
## 167        Mam.Sp.Richness2  ~            Sp.Richness2 10.501 -0.783
## 179         Prop.Mam.Abdun2  ~             Attendance2  9.573 -0.148
## 180         Prop.Mam.Abdun2  ~          Total.Animals2 13.134 -0.141
## 182         Prop.Mam.Abdun2  ~      Prop.Threat.Abund2  3.479 -0.040
## 183         Prop.Mam.Abdun2  ~        Brillouin.Index2  4.628  0.069
## 193 Mean.Sp.BodyMassXAbund2  ~             Attendance2 10.664 -0.480
## 194 Mean.Sp.BodyMassXAbund2  ~          Total.Animals2  8.775 -0.237
## 195 Mean.Sp.BodyMassXAbund2  ~            Sp.Richness2  7.245 -0.218
## 198 Mean.Sp.BodyMassXAbund2  ~        Mean.Raup.Crick2 26.364 -0.436
## 207              X50km_Pop2  ~             Attendance2 28.056  0.529
## 208              X50km_Pop2  ~          Total.Animals2 24.323  0.266
## 210              X50km_Pop2  ~      Prop.Threat.Abund2  3.360  0.055
## 212              X50km_Pop2  ~        Mean.Raup.Crick2  3.252  0.063
## 236            GDP.Millions2  ~          Total.Animals2  4.674  0.103
## 237            GDP.Millions2  ~            Sp.Richness2 29.233  0.332
## 239            GDP.Millions2  ~        Brillouin.Index2 19.260  0.175
## 240            GDP.Millions2  ~        Mean.Raup.Crick2 12.402 -0.100
## 250        Nat_Pop._WB20152  ~          Total.Animals2  3.151 -0.074
## 251        Nat_Pop._WB20152  ~            Sp.Richness2 30.274 -0.326
## 253        Nat_Pop._WB20152  ~        Brillouin.Index2 17.374 -0.144
## 254        Nat_Pop._WB20152  ~        Mean.Raup.Crick2 17.218  0.111
## 263             Prop.Mam.Sp2  ~             Attendance2 12.370  0.145
## 264             Prop.Mam.Sp2  ~          Total.Animals2 23.101  0.195
## 266             Prop.Mam.Sp2  ~      Prop.Threat.Abund2 10.655  0.071
## 267             Prop.Mam.Sp2  ~        Brillouin.Index2  3.998 -0.067
##     sepc.lv sepc.all sepc.nox
## 88   -0.137   -0.137   -0.137
## 90    0.035    0.035    0.035
## 91   -0.063   -0.063   -0.063
## 92    0.126    0.125    0.125
## 96    0.184    0.178    0.178
## 99   -0.194   -0.196   -0.196
## 102   0.148    0.148    0.148
## 103   0.091    0.091    0.092
## 104   0.129    0.129    0.129
## 105   0.064    0.064    0.065
## 109   0.067    0.068    0.068
## 110  -0.058   -0.059   -0.059
## 112  -0.081   -0.081   -0.081
## 116  -0.032   -0.032   -0.032
## 117   0.159    0.154    0.154
## 118  -0.128   -0.128   -0.128
## 119  -0.282   -0.282   -0.282
## 123   0.124    0.124    0.124
## 124   0.178    0.178    0.178
## 125   0.163    0.163    0.164
## 126   0.134    0.134    0.135
## 128   0.125    0.125    0.125
```

```
## 129   0.218    0.218    0.218
## 131   0.128    0.127    0.127
## 133   0.062    0.061    0.061
## 135   0.086    0.085    0.085
## 136   0.070    0.070    0.070
## 138  -0.059   -0.059   -0.059
## 140   0.074    0.073    0.073
## 141   0.245    0.236    0.236
## 144   0.229    0.228    0.228
## 145   0.274    0.273    0.273
## 146   0.156    0.155    0.156
## 147   0.117    0.116    0.116
## 148   0.106    0.105    0.105
## 150   0.111    0.110    0.110
## 151   0.612    0.592    0.592
## 152   0.440    0.439    0.439
## 153   0.894    0.894    0.894
## 155   0.155    0.156    0.156
## 156   0.113    0.113    0.113
## 167  -0.783   -0.783   -0.783
## 179  -0.148   -0.143   -0.143
## 180  -0.141   -0.140   -0.140
## 182  -0.040   -0.040   -0.040
## 183   0.069    0.070    0.070
## 193  -0.480   -0.464   -0.464
## 194  -0.237   -0.237   -0.237
## 195  -0.218   -0.218   -0.218
## 198  -0.436   -0.439   -0.439
## 207   0.529    0.512    0.512
## 208   0.266    0.265    0.265
## 210   0.055    0.055    0.055
## 212   0.063    0.063    0.063
## 236   0.103    0.103    0.103
## 237   0.332    0.332    0.332
## 239   0.175    0.176    0.176
## 240  -0.100   -0.100   -0.100
## 250  -0.074   -0.074   -0.074
## 251  -0.326   -0.326   -0.326
## 253  -0.144   -0.145   -0.145
## 254   0.111    0.112    0.112
## 263   0.145    0.140    0.140
## 264   0.195    0.194    0.194
## 266   0.071    0.071    0.071
## 267  -0.067   -0.067   -0.067
```

```r
# Adjust for the nested nature of the data (institutions within countries)
# Fit model and generate model summary
design <- svydesign(ids = ~Country, nest=TRUE, data=sem_attendance_data)
fit.adj7A <- lavaan.survey(lavaan.fit = mod.7A.fit, survey.design = design)
summary(fit.adj7A, rsq = TRUE)
```

```
## lavaan (0.5-23.1097) converged normally after  63 iterations
##
##   Number of observations                           458
##
```

```
##   Estimator                                         ML      Robust
##   Minimum Function Test Statistic              282.120      86.866
##   Degrees of freedom                                41          41
##   P-value (Chi-square)                           0.000       0.000
##   Scaling correction factor                                  3.248
##     for the Satorra-Bentler correction
##
## Parameter Estimates:
##
##   Information                                 Expected
##   Standard Errors                           Robust.sem
##
## Regressions:
##                        Estimate  Std.Err  z-value  P(>|z|)
##   Attendance2 ~
##     Zoo.Area.ha2          0.082    0.043    1.919    0.055
##     Sp.Richness2         -0.280    0.103   -2.704    0.007
##     Total.Animals2        0.548    0.074    7.365    0.000
##     Mam.Sp.Rchnss2        0.146    0.042    3.478    0.001
##     Prop.Mam.Abdn2       -0.100    0.032   -3.119    0.002
##     Prp.Thrt.Abnd2        0.017    0.028    0.615    0.538
##     Mn.Sp.BdyMsXA2        0.324    0.025   12.783    0.000
##     Brilloun.Indx2        0.025    0.068    0.372    0.710
##     Mean.Rap.Crck2        0.116    0.034    3.439    0.001
##     X50km_Pop2            0.073    0.032    2.250    0.024
##     X10km_Pop2            0.417    0.043    9.679    0.000
##     GDP.Millions2         0.263    0.059    4.467    0.000
##     Nt_Pp._WB20152       -0.128    0.063   -2.039    0.041
##   Total.Animals2 ~
##     Zoo.Area.ha2          0.309    0.042    7.287    0.000
##     Sp.Richness2          0.759    0.049   15.353    0.000
##     Mn.Sp.BdyMsXA2       -0.157    0.034   -4.588    0.000
##     GDP.Millions2        -0.136    0.069   -1.983    0.047
##   Sp.Richness2 ~
##     Zoo.Area.ha2          0.067    0.044    1.519    0.129
##     Prop.Mam.Sp2         -0.581    0.038  -15.231    0.000
##     Mam.Sp.Rchnss2        0.755    0.068   11.066    0.000
##   Prop.Threat.Abund2 ~
##     Brilloun.Indx2        0.107    0.131    0.820    0.412
##   Brillouin.Index2 ~
##     Sp.Richness2          1.320    0.088   14.917    0.000
##     Total.Animals2       -0.620    0.127   -4.891    0.000
##     GDP.Millions2        -0.206    0.072   -2.853    0.004
##   Mean.Raup.Crick2 ~
##     Sp.Richness2         -0.467    0.188   -2.482    0.013
##     Total.Animals2        0.478    0.116    4.127    0.000
##     Mn.Sp.BdyMsXA2       -0.246    0.084   -2.924    0.003
##     Prop.Mam.Sp2         -0.366    0.080   -4.580    0.000
##
## Covariances:
##                              Estimate  Std.Err  z-value  P(>|z|)
##   Zoo.Area.ha2 ~~
##     Mam.Sp.Rchnss2              0.381    0.064    5.985    0.000
##     Prop.Mam.Abdn2              0.352    0.092    3.822    0.000
```

```
##     Mn.Sp.BdyMsXA2             0.522    0.101    5.177    0.000
##     X50km_Pop2                 0.060    0.069    0.880    0.379
##     X10km_Pop2                -0.010    0.078   -0.124    0.901
##     GDP.Millions2             -0.027    0.041   -0.673    0.501
##     Nt_Pp._WB20152             0.061    0.090    0.682    0.495
##     Prop.Mam.Sp2               0.312    0.089    3.510    0.000
##   Mam.Sp.Richness2 ~~
##     Prop.Mam.Abdn2             0.058    0.049    1.172    0.241
##     Mn.Sp.BdyMsXA2             0.227    0.078    2.921    0.003
##     X50km_Pop2                 0.204    0.082    2.487    0.013
##     X10km_Pop2                 0.284    0.066    4.278    0.000
##     GDP.Millions2             -0.060    0.064   -0.937    0.349
##     Nt_Pp._WB20152            -0.088    0.102   -0.863    0.388
##     Prop.Mam.Sp2               0.088    0.061    1.435    0.151
##   Prop.Mam.Abdun2 ~~
##     Mn.Sp.BdyMsXA2             0.592    0.109    5.445    0.000
##     X50km_Pop2                -0.123    0.054   -2.263    0.024
##     X10km_Pop2                -0.248    0.060   -4.154    0.000
##     GDP.Millions2             -0.115    0.101   -1.140    0.254
##     Nt_Pp._WB20152            -0.111    0.088   -1.264    0.206
##     Prop.Mam.Sp2               0.878    0.128    6.865    0.000
##   Mean.Sp.BodyMassXAbund2 ~~
##     X50km_Pop2                 0.013    0.074    0.174    0.862
##     X10km_Pop2                 0.021    0.064    0.329    0.742
##     GDP.Millions2             -0.079    0.038   -2.055    0.040
##     Nt_Pp._WB20152            -0.004    0.084   -0.044    0.965
##     Prop.Mam.Sp2               0.628    0.126    5.002    0.000
##   X50km_Pop2 ~~
##     X10km_Pop2                 0.752    0.126    5.982    0.000
##     GDP.Millions2             -0.013    0.100   -0.131    0.896
##     Nt_Pp._WB20152             0.191    0.177    1.083    0.279
##     Prop.Mam.Sp2              -0.096    0.051   -1.888    0.059
##   X10km_Pop2 ~~
##     GDP.Millions2             -0.028    0.056   -0.505    0.613
##     Nt_Pp._WB20152             0.173    0.101    1.701    0.089
##     Prop.Mam.Sp2              -0.204    0.055   -3.685    0.000
##   GDP.Millions2 ~~
##     Nt_Pp._WB20152             0.826    0.202    4.089    0.000
##     Prop.Mam.Sp2              -0.123    0.105   -1.172    0.241
##   Nat_Pop._WB20152 ~~
##     Prop.Mam.Sp2              -0.115    0.091   -1.257    0.209
##
## Intercepts:
##                    Estimate  Std.Err  z-value  P(>|z|)
##    .Attendance2      -0.000    0.030   -0.000    1.000
##    .Total.Animals2   -0.000    0.067   -0.000    1.000
##    .Sp.Richness2      0.000    0.034    0.000    1.000
##    .Prp.Thrt.Abnd2   -0.000    0.077   -0.000    1.000
##    .Brilloun.Indx2   -0.000    0.055   -0.000    1.000
##    .Mean.Rap.Crck2    0.000    0.076    0.000    1.000
##     Zoo.Area.ha2      0.000    0.064    0.000    1.000
##     Mam.Sp.Rchnss2   -0.000    0.079   -0.000    1.000
##     Prop.Mam.Abdn2    0.000    0.113    0.000    1.000
##     Mn.Sp.BdyMsXA2    0.000    0.071    0.000    1.000
```

```
##     X50km_Pop2       -0.000    0.114   -0.000    1.000
##     X10km_Pop2        0.000    0.105    0.000    1.000
##     GDP.Millions2    -0.000    0.345   -0.000    1.000
##     Nt_Pp._WB20152    0.000    0.295    0.000    1.000
##     Prop.Mam.Sp2      0.000    0.118    0.000    1.000
##
## Variances:
##                    Estimate  Std.Err  z-value  P(>|z|)
##    .Attendance2       0.270    0.028    9.568    0.000
##    .Total.Animals2    0.219    0.037    5.986    0.000
##    .Sp.Richness2      0.151    0.046    3.269    0.001
##    .Prp.Thrt.Abnd2    0.986    0.133    7.389    0.000
##    .Brilloun.Indx2    0.251    0.053    4.691    0.000
##    .Mean.Rap.Crck2    0.684    0.059   11.527    0.000
##     Zoo.Area.ha2      0.998    0.112    8.886    0.000
##     Mam.Sp.Rchnss2    0.998    0.115    8.669    0.000
##     Prop.Mam.Abdn2    0.998    0.121    8.221    0.000
##     Mn.Sp.BdyMsXA2    0.998    0.160    6.219    0.000
##     X50km_Pop2        0.998    0.131    7.623    0.000
##     X10km_Pop2        0.998    0.174    5.745    0.000
##     GDP.Millions2     0.998    0.246    4.061    0.000
##     Nt_Pp._WB20152    0.998    0.225    4.439    0.000
##     Prop.Mam.Sp2      0.998    0.150    6.662    0.000
##
## R-Square:
##                    Estimate
##     Attendance2       0.711
##     Total.Animals2    0.780
##     Sp.Richness2      0.849
##     Prp.Thrt.Abnd2    0.012
##     Brilloun.Indx2    0.752
##     Mean.Rap.Crck2    0.323
```

```
# Generate fit indices
fitMeasures(fit.adj7A, c("agfi", "rmr", "srmr", "rmsea", "cfi", "nnfi", "tli"))
```

```
##  agfi   rmr  srmr rmsea   cfi  nnfi   tli
## 0.766 0.054 0.051 0.113 0.924 0.872 0.872
```

```
# Generate modification indices
mi7adjA <- modindices(fit.adj7A)
print(mi7adjA[mi7adjA$mi > 3.0,])
```

```
##                    lhs op                rhs     mi mi.scaled
## 103         Total.Animals2 ~~       Mean.Raup.Crick2 14.977     4.611
## 105           Sp.Richness2 ~~       Brillouin.Index2 12.507     3.851
## 106           Sp.Richness2 ~~       Mean.Raup.Crick2 13.950     4.295
## 107     Prop.Threat.Abund2 ~~       Brillouin.Index2 22.014     6.778
## 111         Total.Animals2  ~            Attendance2 14.245     4.386
## 114         Total.Animals2  ~       Mean.Raup.Crick2 18.614     5.731
## 117         Total.Animals2  ~             X50km_Pop2 43.332    13.342
## 118         Total.Animals2  ~             X10km_Pop2 15.014     4.623
## 119         Total.Animals2  ~       Nat_Pop._WB20152 10.674     3.287
## 120         Total.Animals2  ~           Prop.Mam.Sp2  3.693     1.137
## 124           Sp.Richness2  ~       Brillouin.Index2  4.137     1.274
## 125           Sp.Richness2  ~       Mean.Raup.Crick2  6.484     1.996
```

```
## 127           Sp.Richness2  ~ Mean.Sp.BodyMassXAbund2  9.674     2.979
## 131           Sp.Richness2  ~       Nat_Pop._WB20152  3.059     0.942
## 132      Prop.Threat.Abund2  ~            Attendance2  6.854     2.110
## 133      Prop.Threat.Abund2  ~          Total.Animals2  5.660     1.743
## 134      Prop.Threat.Abund2  ~           Sp.Richness2 13.620     4.194
## 138      Prop.Threat.Abund2  ~         Prop.Mam.Abdun2  6.178     1.902
## 139      Prop.Threat.Abund2  ~ Mean.Sp.BodyMassXAbund2 14.457     4.451
## 140      Prop.Threat.Abund2  ~             X50km_Pop2 12.016     3.700
## 141      Prop.Threat.Abund2  ~             X10km_Pop2  7.675     2.363
## 143      Prop.Threat.Abund2  ~       Nat_Pop._WB20152  7.152     2.202
## 144      Prop.Threat.Abund2  ~           Prop.Mam.Sp2 18.492     5.694
## 146        Brillouin.Index2  ~      Prop.Threat.Abund2 22.014     6.778
## 148        Brillouin.Index2  ~            Zoo.Area.ha2  5.621     1.731
## 150        Brillouin.Index2  ~         Prop.Mam.Abdun2 10.518     3.239
## 151        Brillouin.Index2  ~ Mean.Sp.BodyMassXAbund2  8.684     2.674
## 153        Brillouin.Index2  ~             X10km_Pop2  5.652     1.740
## 155        Brillouin.Index2  ~           Prop.Mam.Sp2  7.333     2.258
## 156        Mean.Raup.Crick2  ~            Attendance2  8.803     2.710
## 159        Mean.Raup.Crick2  ~            Zoo.Area.ha2 18.033     5.552
## 160        Mean.Raup.Crick2  ~       Mam.Sp.Richness2 10.168     3.131
## 161        Mean.Raup.Crick2  ~         Prop.Mam.Abdun2  3.616     1.114
## 162        Mean.Raup.Crick2  ~             X50km_Pop2  8.649     2.663
## 163        Mean.Raup.Crick2  ~             X10km_Pop2  6.466     1.991
## 165        Mean.Raup.Crick2  ~       Nat_Pop._WB20152  7.741     2.383
## 166            Zoo.Area.ha2  ~            Attendance2  6.508     2.004
## 167            Zoo.Area.ha2  ~          Total.Animals2  7.437     2.290
## 168            Zoo.Area.ha2  ~           Sp.Richness2 22.367     6.887
## 170            Zoo.Area.ha2  ~        Brillouin.Index2  4.448     1.369
## 171            Zoo.Area.ha2  ~        Mean.Raup.Crick2  5.584     1.719
## 182        Mam.Sp.Richness2  ~           Sp.Richness2 10.501     3.233
## 194         Prop.Mam.Abdun2  ~            Attendance2  9.573     2.948
## 195         Prop.Mam.Abdun2  ~          Total.Animals2 13.134     4.044
## 197         Prop.Mam.Abdun2  ~      Prop.Threat.Abund2  3.479     1.071
## 198         Prop.Mam.Abdun2  ~        Brillouin.Index2  4.628     1.425
## 208 Mean.Sp.BodyMassXAbund2  ~            Attendance2 10.664     3.284
## 209 Mean.Sp.BodyMassXAbund2  ~          Total.Animals2  8.775     2.702
## 210 Mean.Sp.BodyMassXAbund2  ~           Sp.Richness2  7.245     2.231
## 213 Mean.Sp.BodyMassXAbund2  ~        Mean.Raup.Crick2 26.364     8.118
## 222              X50km_Pop2  ~            Attendance2 28.056     8.639
## 223              X50km_Pop2  ~          Total.Animals2 24.323     7.489
## 225              X50km_Pop2  ~      Prop.Threat.Abund2  3.360     1.035
## 227              X50km_Pop2  ~        Mean.Raup.Crick2  3.252     1.001
## 251           GDP.Millions2  ~          Total.Animals2  4.674     1.439
## 252           GDP.Millions2  ~           Sp.Richness2 29.233     9.001
## 254           GDP.Millions2  ~        Brillouin.Index2 19.260     5.930
## 255           GDP.Millions2  ~        Mean.Raup.Crick2 12.402     3.819
## 265        Nat_Pop._WB20152  ~          Total.Animals2  3.151     0.970
## 266        Nat_Pop._WB20152  ~           Sp.Richness2 30.274     9.321
## 268        Nat_Pop._WB20152  ~        Brillouin.Index2 17.374     5.349
## 269        Nat_Pop._WB20152  ~        Mean.Raup.Crick2 17.218     5.301
## 278            Prop.Mam.Sp2  ~            Attendance2 12.370     3.809
## 279            Prop.Mam.Sp2  ~          Total.Animals2 23.101     7.113
## 281            Prop.Mam.Sp2  ~      Prop.Threat.Abund2 10.655     3.281
## 282            Prop.Mam.Sp2  ~        Brillouin.Index2  3.998     1.231
```

```
##        epc sepc.lv sepc.all sepc.nox
## 103 -0.137  -0.137   -0.137   -0.137
## 105  0.035   0.035    0.035    0.035
## 106 -0.063  -0.063   -0.063   -0.063
## 107  0.126   0.126    0.125    0.125
## 111  0.184   0.184    0.178    0.178
## 114 -0.194  -0.194   -0.196   -0.196
## 117  0.148   0.148    0.148    0.148
## 118  0.091   0.091    0.091    0.092
## 119  0.129   0.129    0.129    0.129
## 120  0.064   0.064    0.064    0.065
## 124  0.067   0.067    0.068    0.068
## 125 -0.058  -0.058   -0.059   -0.059
## 127 -0.081  -0.081   -0.081   -0.081
## 131 -0.032  -0.032   -0.032   -0.032
## 132  0.159   0.159    0.154    0.154
## 133 -0.128  -0.128   -0.128   -0.128
## 134 -0.282  -0.282   -0.282   -0.282
## 138  0.124   0.124    0.124    0.124
## 139  0.178   0.178    0.178    0.178
## 140  0.163   0.163    0.163    0.164
## 141  0.134   0.134    0.134    0.135
## 143  0.125   0.125    0.125    0.125
## 144  0.218   0.218    0.218    0.218
## 146  0.128   0.128    0.127    0.127
## 148  0.062   0.062    0.061    0.061
## 150  0.086   0.086    0.085    0.085
## 151  0.070   0.070    0.070    0.070
## 153 -0.059  -0.059   -0.059   -0.059
## 155  0.074   0.074    0.073    0.073
## 156  0.245   0.245    0.236    0.236
## 159  0.229   0.229    0.228    0.228
## 160  0.274   0.274    0.273    0.273
## 161  0.156   0.156    0.155    0.156
## 162  0.117   0.117    0.116    0.116
## 163  0.106   0.106    0.105    0.105
## 165  0.111   0.111    0.110    0.110
## 166  0.612   0.612    0.592    0.592
## 167  0.440   0.440    0.439    0.439
## 168  0.894   0.894    0.894    0.894
## 170  0.155   0.155    0.156    0.156
## 171  0.113   0.113    0.113    0.113
## 182 -0.783  -0.783   -0.783   -0.783
## 194 -0.148  -0.148   -0.143   -0.143
## 195 -0.141  -0.141   -0.140   -0.140
## 197 -0.040  -0.040   -0.040   -0.040
## 198  0.069   0.069    0.070    0.070
## 208 -0.480  -0.480   -0.464   -0.464
## 209 -0.237  -0.237   -0.237   -0.237
## 210 -0.218  -0.218   -0.218   -0.218
## 213 -0.436  -0.436   -0.439   -0.439
## 222  0.529   0.529    0.512    0.512
## 223  0.266   0.266    0.265    0.265
## 225  0.055   0.055    0.055    0.055
```

```
## 227  0.063   0.063    0.063    0.063
## 251  0.103   0.103    0.103    0.103
## 252  0.332   0.332    0.332    0.332
## 254  0.175   0.175    0.176    0.176
## 255 -0.100  -0.100   -0.100   -0.100
## 265 -0.074  -0.074   -0.074   -0.074
## 266 -0.326  -0.326   -0.326   -0.326
## 268 -0.144  -0.144   -0.145   -0.145
## 269  0.111   0.111    0.112    0.112
## 278  0.145   0.145    0.140    0.140
## 279  0.195   0.195    0.194    0.194
## 281  0.071   0.071    0.071    0.071
## 282 -0.067  -0.067   -0.067   -0.067
```

Model Comparisons 4

At this stage we compare the models generated using AICc values. Overall model selection from the pool of competing models is achieved using AICc values, with a threshold of more than 2 AICc units lower than nearest competing model being considered sufficient for model selection.

```
# Model Comparisons using AICc

# Comparing models with and without adjustment for nested nature of data
# library(AICcmodavg)
# source("lavaan.modavg.R")

aictab.lavaan(list(mod.1A.fit, mod.2A.fit, mod.3A.fit, mod.4A.fit, mod.5A.fit, mod.6A.fit, mod.7A.fit, m
              c("mod.1A", "mod.2A", "mod.3A", "mod.4A", "mod.5A", "mod.6A", "mod.7A","mod.1A.nested", "m
```

```
##
## Model selection based on AICc:
##
##                K     AICc Delta_AICc AICcWt Cum.Wt       LL
## mod.7A        79 14367.64       0.00      1      1 -7102.93
## mod.7A.nested 94 14397.64      30.00      0      1 -7102.93
## mod.6A        78 14402.28      34.64      0      1 -7121.38
## mod.5A        77 14425.87      58.23      0      1 -7134.31
## mod.6A.nested 93 14432.28      64.64      0      1 -7121.38
## mod.5A.nested 92 14455.87      88.23      0      1 -7134.31
## mod.4A        76 14462.11      94.46      0      1 -7153.55
## mod.4A.nested 91 14492.11     124.46      0      1 -7153.55
## mod.3A        75 14527.06     159.42      0      1 -7187.14
## mod.3A.nested 90 14557.06     189.42      0      1 -7187.14
## mod.2A        74 14629.00     261.36      0      1 -7239.23
## mod.2A.nested 89 14659.00     291.36      0      1 -7239.23
## mod.1A        73 15285.69     918.05      0      1 -7568.68
## mod.1A.nested 88 15315.69     948.05      0      1 -7568.68
```

Based on these results we can see that model 7 is the superior model (lowest AICc values), for both models with and without adjustment for nested nature of data.

At this stage no other addition is conceptually appealling. Now we use p-values to identify potentially unsupported pathways, with a threshold of 0.05. Highest p-values are considered first for removal.

Model 8

Based on the results generated from the nested seventh model, the highest p-value relationship to be considered for removal is **Attendance2 ~ Brilloun.Indx2** with a p-value of **0.710**. Therefore we decide to remove this pathway. Based on the Presence-Absence model results and the fact that Brillouin Index is no longer contributing to visitor attendance, it was decided to remove the variable from the analysis completely in a semi-exploratory manner (similar to the Presence-Absence model). Therefore we decide to remove the pathways associated with this variable. Once again, the model summary, fit indices and modification indices were all generated for the model, adjusting for the nested nature of data.

```
# Attendance SEM (Species Abundance)

# Model 8
# Removal of Attendance2 ~ Brilloun.Indx2, p = 0.710 (and subsequently Brilloun.Indx2 completely)

mod.8A <- 'Attendance2 ~ Zoo.Area.ha2 + Sp.Richness2 + Total.Animals2
+ Mam.Sp.Richness2 + Prop.Mam.Abdun2 + Prop.Threat.Abund2
+ Mean.Sp.BodyMassXAbund2 + Mean.Raup.Crick2
+ X50km_Pop2 + X10km_Pop2 + GDP.Millions2 + Nat_Pop._WB20152

Total.Animals2 ~ Zoo.Area.ha2 + Sp.Richness2 + Mean.Sp.BodyMassXAbund2 + GDP.Millions2
Sp.Richness2 ~ Zoo.Area.ha2 + Prop.Mam.Sp2 + Mam.Sp.Richness2
Mean.Raup.Crick2 ~ Sp.Richness2 + Total.Animals2 + Mean.Sp.BodyMassXAbund2 + Prop.Mam.Sp2'

# Fit model and generate model summary
mod.8A.fit <- sem(mod.8A, data = sem_attendance_data, fixed.x=FALSE)
summary(mod.8A.fit, rsq = TRUE)
```

```
## lavaan (0.5-23.1097) converged normally after  64 iterations
##
##   Number of observations                           458
##
##   Estimator                                         ML
##   Minimum Function Test Statistic              180.796
##   Degrees of freedom                                23
##   P-value (Chi-square)                           0.000
##
## Parameter Estimates:
##
##   Information                                 Expected
##   Standard Errors                             Standard
##
## Regressions:
##                      Estimate  Std.Err  z-value  P(>|z|)
##   Attendance2 ~
##     Zoo.Area.ha2        0.082    0.036    2.298    0.022
##     Sp.Richness2       -0.241    0.066   -3.664    0.000
##     Total.Animals2      0.533    0.054    9.914    0.000
##     Mam.Sp.Rchnss2      0.142    0.048    2.947    0.003
##     Prop.Mam.Abdn2     -0.097    0.040   -2.406    0.016
##     Prp.Thrt.Abnd2      0.020    0.025    0.808    0.419
##     Mn.Sp.BdyMsXA2      0.326    0.037    8.810    0.000
##     Mean.Rap.Crck2      0.117    0.029    4.027    0.000
##     X50km_Pop2          0.075    0.038    1.949    0.051
##     X10km_Pop2          0.413    0.041   10.062    0.000
```

```
##     GDP.Millions2       0.258    0.048    5.366    0.000
##     Nt_Pp._WB20152     -0.129    0.049   -2.616    0.009
##   Total.Animals2 ~
##     Zoo.Area.ha2        0.309    0.027   11.444    0.000
##     Sp.Richness2        0.759    0.023   32.580    0.000
##     Mn.Sp.BdyMsXA2     -0.157    0.027   -5.828    0.000
##     GDP.Millions2      -0.136    0.022   -6.202    0.000
##   Sp.Richness2 ~
##     Zoo.Area.ha2        0.067    0.021    3.225    0.001
##     Prop.Mam.Sp2       -0.581    0.019  -30.364    0.000
##     Mam.Sp.Rchnss2      0.755    0.020   38.373    0.000
##   Mean.Raup.Crick2 ~
##     Sp.Richness2       -0.467    0.077   -6.082    0.000
##     Total.Animals2      0.478    0.071    6.732    0.000
##     Mn.Sp.BdyMsXA2     -0.246    0.051   -4.808    0.000
##     Prop.Mam.Sp2       -0.366    0.058   -6.278    0.000
##
## Covariances:
##                           Estimate  Std.Err  z-value  P(>|z|)
##   Zoo.Area.ha2 ~~
##     Mam.Sp.Rchnss2           0.381    0.050    7.634    0.000
##     Prop.Mam.Abdn2           0.352    0.049    7.121    0.000
##     Prp.Thrt.Abnd2           0.054    0.047    1.156    0.248
##     Mn.Sp.BdyMsXA2           0.522    0.053    9.923    0.000
##     X50km_Pop2               0.060    0.047    1.291    0.197
##     X10km_Pop2              -0.010    0.047   -0.208    0.835
##     GDP.Millions2           -0.027    0.047   -0.588    0.557
##     Nt_Pp._WB20152           0.061    0.047    1.310    0.190
##     Prop.Mam.Sp2             0.312    0.049    6.389    0.000
##   Mam.Sp.Richness2 ~~
##     Prop.Mam.Abdn2           0.058    0.047    1.237    0.216
##     Prp.Thrt.Abnd2           0.091    0.047    1.954    0.051
##     Mn.Sp.BdyMsXA2           0.227    0.048    4.750    0.000
##     X50km_Pop2               0.204    0.048    4.281    0.000
##     X10km_Pop2               0.284    0.048    5.852    0.000
##     GDP.Millions2           -0.060    0.047   -1.277    0.202
##     Nt_Pp._WB20152          -0.088    0.047   -1.881    0.060
##     Prop.Mam.Sp2             0.088    0.047    1.882    0.060
##   Prop.Mam.Abdun2 ~~
##     Prp.Thrt.Abnd2           0.075    0.047    1.608    0.108
##     Mn.Sp.BdyMsXA2           0.592    0.054   10.922    0.000
##     X50km_Pop2              -0.123    0.047   -2.622    0.009
##     X10km_Pop2              -0.248    0.048   -5.165    0.000
##     GDP.Millions2           -0.115    0.047   -2.458    0.014
##     Nt_Pp._WB20152          -0.111    0.047   -2.362    0.018
##     Prop.Mam.Sp2             0.878    0.062   14.135    0.000
##   Prop.Threat.Abund2 ~~
##     Mn.Sp.BdyMsXA2           0.165    0.047    3.486    0.000
##     X50km_Pop2               0.168    0.047    3.547    0.000
##     X10km_Pop2               0.143    0.047    3.029    0.002
##     GDP.Millions2            0.069    0.047    1.468    0.142
##     Nt_Pp._WB20152           0.105    0.047    2.233    0.026
##     Prop.Mam.Sp2             0.143    0.047    3.046    0.002
##   Mean.Sp.BodyMassXAbund2 ~~
```

```
##     X50km_Pop2                 0.013    0.047    0.275    0.784
##     X10km_Pop2                 0.021    0.047    0.455    0.649
##     GDP.Millions2             -0.079    0.047   -1.682    0.093
##     Nt_Pp._WB20152            -0.004    0.047   -0.079    0.937
##     Prop.Mam.Sp2               0.628    0.055   11.405    0.000
##   X50km_Pop2 ~~
##     X10km_Pop2                 0.752    0.058   12.886    0.000
##     GDP.Millions2             -0.013    0.047   -0.281    0.779
##     Nt_Pp._WB20152             0.191    0.047    4.033    0.000
##     Prop.Mam.Sp2              -0.096    0.047   -2.054    0.040
##   X10km_Pop2 ~~
##     GDP.Millions2             -0.028    0.047   -0.604    0.546
##     Nt_Pp._WB20152             0.173    0.047    3.646    0.000
##     Prop.Mam.Sp2              -0.204    0.048   -4.293    0.000
##   GDP.Millions2 ~~
##     Nt_Pp._WB20152             0.826    0.061   13.642    0.000
##     Prop.Mam.Sp2              -0.123    0.047   -2.615    0.009
##   Nat_Pop._WB20152 ~~
##     Prop.Mam.Sp2              -0.115    0.047   -2.447    0.014
##
## Variances:
##                    Estimate  Std.Err  z-value  P(>|z|)
##    .Attendance2       0.270    0.018   15.133    0.000
##    .Total.Animals2    0.219    0.014   15.133    0.000
##    .Sp.Richness2      0.151    0.010   15.133    0.000
##    .Mean.Rap.Crck2    0.684    0.045   15.133    0.000
##     Zoo.Area.ha2      0.998    0.066   15.133    0.000
##     Mam.Sp.Rchnss2    0.998    0.066   15.133    0.000
##     Prop.Mam.Abdn2    0.998    0.066   15.133    0.000
##     Prp.Thrt.Abnd2    0.998    0.066   15.133    0.000
##     Mn.Sp.BdyMsXA2    0.998    0.066   15.133    0.000
##     X50km_Pop2        0.998    0.066   15.133    0.000
##     X10km_Pop2        0.998    0.066   15.133    0.000
##     GDP.Millions2     0.998    0.066   15.133    0.000
##     Nt_Pp._WB20152    0.998    0.066   15.133    0.000
##     Prop.Mam.Sp2      0.998    0.066   15.133    0.000
##
## R-Square:
##                    Estimate
##     Attendance2       0.712
##     Total.Animals2    0.780
##     Sp.Richness2      0.849
##     Mean.Rap.Crck2    0.323
```

```
# Generate fit indices
fitMeasures(mod.8A.fit, c("agfi", "rmr", "srmr", "rmsea", "cfi", "nnfi", "tli"))
```

```
##  agfi   rmr  srmr rmsea   cfi  nnfi   tli
## 0.771 0.037 0.037 0.122 0.936 0.872 0.872
```

```
# Generate modification indices
mi8A <- modindices(mod.8A.fit)
print(mi8A[mi8A$mi > 3.0,])
```

```
##                      lhs op                  rhs     mi    epc
## 87           Total.Animals2 ~~        Mean.Raup.Crick2 14.977 -0.137
```

```
## 88            Sp.Richness2 ~~        Mean.Raup.Crick2 13.951 -0.063
## 90           Total.Animals2  ~             Attendance2 13.939  0.181
## 91           Total.Animals2  ~        Mean.Raup.Crick2 18.614 -0.194
## 95           Total.Animals2  ~              X50km_Pop2 43.332  0.148
## 96           Total.Animals2  ~              X10km_Pop2 15.014  0.091
## 97           Total.Animals2  ~        Nat_Pop._WB20152 10.674  0.129
## 98           Total.Animals2  ~            Prop.Mam.Sp2  3.693  0.064
## 101            Sp.Richness2  ~        Mean.Raup.Crick2  6.484 -0.058
## 104            Sp.Richness2  ~ Mean.Sp.BodyMassXAbund2  9.674 -0.081
## 108            Sp.Richness2  ~        Nat_Pop._WB20152  3.059 -0.032
## 109        Mean.Raup.Crick2  ~             Attendance2  8.581  0.241
## 110        Mean.Raup.Crick2  ~            Zoo.Area.ha2 18.033  0.229
## 111        Mean.Raup.Crick2  ~        Mam.Sp.Richness2 10.168  0.274
## 112        Mean.Raup.Crick2  ~         Prop.Mam.Abdun2  3.616  0.156
## 114        Mean.Raup.Crick2  ~              X50km_Pop2  8.649  0.117
## 115        Mean.Raup.Crick2  ~              X10km_Pop2  6.466  0.106
## 117        Mean.Raup.Crick2  ~        Nat_Pop._WB20152  7.741  0.111
## 118            Zoo.Area.ha2  ~             Attendance2  6.244  0.605
## 119            Zoo.Area.ha2  ~           Total.Animals2  6.234  0.400
## 120            Zoo.Area.ha2  ~            Sp.Richness2 21.923  0.882
## 121            Zoo.Area.ha2  ~        Mean.Raup.Crick2  5.729  0.114
## 133        Mam.Sp.Richness2  ~            Sp.Richness2 10.251 -0.771
## 144         Prop.Mam.Abdun2  ~             Attendance2  9.922 -0.149
## 145         Prop.Mam.Abdun2  ~           Total.Animals2 14.290 -0.146
## 157      Prop.Threat.Abund2  ~             Attendance2  3.167 -0.263
## 158      Prop.Threat.Abund2  ~           Total.Animals2  3.585 -0.153
## 170 Mean.Sp.BodyMassXAbund2  ~             Attendance2 11.374 -0.500
## 171 Mean.Sp.BodyMassXAbund2  ~           Total.Animals2  7.321 -0.215
## 172 Mean.Sp.BodyMassXAbund2  ~            Sp.Richness2  7.054 -0.215
## 173 Mean.Sp.BodyMassXAbund2  ~        Mean.Raup.Crick2 26.629 -0.430
## 183              X50km_Pop2  ~             Attendance2 27.365  0.521
## 184              X50km_Pop2  ~           Total.Animals2 25.811  0.273
## 186              X50km_Pop2  ~        Mean.Raup.Crick2  3.023  0.060
## 210            GDP.Millions2  ~           Total.Animals2  4.946  0.106
## 211            GDP.Millions2  ~            Sp.Richness2 29.299  0.332
## 212            GDP.Millions2  ~        Mean.Raup.Crick2 12.530 -0.100
## 224        Nat_Pop._WB20152  ~            Sp.Richness2 30.182 -0.326
## 225        Nat_Pop._WB20152  ~        Mean.Raup.Crick2 17.081  0.111
## 235            Prop.Mam.Sp2  ~             Attendance2 12.625  0.145
## 236            Prop.Mam.Sp2  ~           Total.Animals2 25.206  0.201
##     sepc.lv sepc.all sepc.nox
## 87   -0.137   -0.137   -0.137
## 88   -0.063   -0.063   -0.063
## 90    0.181    0.176    0.176
## 91   -0.194   -0.196   -0.196
## 95    0.148    0.148    0.148
## 96    0.091    0.091    0.092
## 97    0.129    0.129    0.129
## 98    0.064    0.064    0.065
## 101  -0.058   -0.059   -0.059
## 104  -0.081   -0.081   -0.081
## 108  -0.032   -0.032   -0.032
## 109   0.241    0.232    0.232
## 110   0.229    0.228    0.228
```

```
## 111   0.274    0.273    0.273
## 112   0.156    0.155    0.156
## 114   0.117    0.116    0.116
## 115   0.106    0.105    0.105
## 117   0.111    0.110    0.110
## 118   0.605    0.586    0.586
## 119   0.400    0.399    0.399
## 120   0.882    0.882    0.882
## 121   0.114    0.115    0.115
## 133  -0.771   -0.771   -0.771
## 144  -0.149   -0.145   -0.145
## 145  -0.146   -0.146   -0.146
## 157  -0.263   -0.255   -0.255
## 158  -0.153   -0.153   -0.153
## 170  -0.500   -0.485   -0.485
## 171  -0.215   -0.215   -0.215
## 172  -0.215   -0.215   -0.215
## 173  -0.430   -0.432   -0.432
## 183   0.521    0.505    0.505
## 184   0.273    0.272    0.272
## 186   0.060    0.061    0.061
## 210   0.106    0.106    0.106
## 211   0.332    0.332    0.332
## 212  -0.100   -0.101   -0.101
## 224  -0.326   -0.326   -0.326
## 225   0.111    0.112    0.112
## 235   0.145    0.141    0.141
## 236   0.201    0.201    0.201
```

```r
# Adjust for the nested nature of the data (institutions within countries)
# Fit model and generate model summary
design <- svydesign(ids = ~Country, nest=TRUE, data=sem_attendance_data)
fit.adj8A <- lavaan.survey(lavaan.fit = mod.8A.fit, survey.design = design)
summary(fit.adj8A, rsq = TRUE)
```

```
## lavaan (0.5-23.1097) converged normally after  63 iterations
##
##   Number of observations                           458
##
##   Estimator                                         ML      Robust
##   Minimum Function Test Statistic              180.796      42.683
##   Degrees of freedom                                23          23
##   P-value (Chi-square)                           0.000       0.008
##   Scaling correction factor                                  4.236
##     for the Satorra-Bentler correction
##
## Parameter Estimates:
##
##   Information                                 Expected
##   Standard Errors                           Robust.sem
##
## Regressions:
##                        Estimate  Std.Err  z-value  P(>|z|)
##   Attendance2 ~
##     Zoo.Area.ha2          0.082    0.044    1.869    0.062
```

```
##     Sp.Richness2       -0.241    0.071   -3.402    0.001
##     Total.Animals2      0.533    0.064    8.358    0.000
##     Mam.Sp.Rchnss2      0.142    0.043    3.295    0.001
##     Prop.Mam.Abdn2     -0.097    0.032   -3.041    0.002
##     Prp.Thrt.Abnd2      0.020    0.028    0.726    0.468
##     Mn.Sp.BdyMsXA2      0.326    0.026   12.650    0.000
##     Mean.Rap.Crck2      0.117    0.034    3.443    0.001
##     X50km_Pop2          0.075    0.030    2.460    0.014
##     X10km_Pop2          0.413    0.043    9.700    0.000
##     GDP.Millions2       0.258    0.058    4.468    0.000
##     Nt_Pp._WB20152     -0.129    0.064   -2.013    0.044
##   Total.Animals2 ~
##     Zoo.Area.ha2        0.309    0.042    7.287    0.000
##     Sp.Richness2        0.759    0.049   15.353    0.000
##     Mn.Sp.BdyMsXA2     -0.157    0.034   -4.588    0.000
##     GDP.Millions2      -0.136    0.069   -1.983    0.047
##   Sp.Richness2 ~
##     Zoo.Area.ha2        0.067    0.044    1.519    0.129
##     Prop.Mam.Sp2       -0.581    0.038  -15.231    0.000
##     Mam.Sp.Rchnss2      0.755    0.068   11.066    0.000
##   Mean.Raup.Crick2 ~
##     Sp.Richness2       -0.467    0.188   -2.482    0.013
##     Total.Animals2      0.478    0.116    4.127    0.000
##     Mn.Sp.BdyMsXA2     -0.246    0.084   -2.924    0.003
##     Prop.Mam.Sp2       -0.366    0.080   -4.580    0.000
##
## Covariances:
##                          Estimate  Std.Err  z-value  P(>|z|)
##   Zoo.Area.ha2 ~~
##     Mam.Sp.Rchnss2          0.381    0.064    5.985    0.000
##     Prop.Mam.Abdn2          0.352    0.092    3.822    0.000
##     Prp.Thrt.Abnd2          0.054    0.064    0.843    0.399
##     Mn.Sp.BdyMsXA2          0.522    0.101    5.177    0.000
##     X50km_Pop2              0.060    0.069    0.880    0.379
##     X10km_Pop2             -0.010    0.078   -0.124    0.901
##     GDP.Millions2          -0.027    0.041   -0.673    0.501
##     Nt_Pp._WB20152          0.061    0.090    0.682    0.495
##     Prop.Mam.Sp2            0.312    0.089    3.510    0.000
##   Mam.Sp.Richness2 ~~
##     Prop.Mam.Abdn2          0.058    0.049    1.172    0.241
##     Prp.Thrt.Abnd2          0.091    0.050    1.844    0.065
##     Mn.Sp.BdyMsXA2          0.227    0.078    2.921    0.003
##     X50km_Pop2              0.204    0.082    2.487    0.013
##     X10km_Pop2              0.284    0.066    4.278    0.000
##     GDP.Millions2          -0.060    0.064   -0.937    0.349
##     Nt_Pp._WB20152         -0.088    0.102   -0.863    0.388
##     Prop.Mam.Sp2            0.088    0.061    1.435    0.151
##   Prop.Mam.Abdun2 ~~
##     Prp.Thrt.Abnd2          0.075    0.081    0.931    0.352
##     Mn.Sp.BdyMsXA2          0.592    0.109    5.445    0.000
##     X50km_Pop2             -0.123    0.054   -2.263    0.024
##     X10km_Pop2             -0.248    0.060   -4.154    0.000
##     GDP.Millions2          -0.115    0.101   -1.140    0.254
##     Nt_Pp._WB20152         -0.111    0.088   -1.264    0.206
```

```
##     Prop.Mam.Sp2               0.878    0.128    6.865    0.000
##   Prop.Threat.Abund2 ~~
##     Mn.Sp.BdyMsXA2             0.165    0.087    1.886    0.059
##     X50km_Pop2                 0.168    0.076    2.218    0.027
##     X10km_Pop2                 0.143    0.056    2.562    0.010
##     GDP.Millions2              0.069    0.051    1.341    0.180
##     Nt_Pp._WB20152             0.105    0.065    1.606    0.108
##     Prop.Mam.Sp2               0.143    0.093    1.548    0.122
##   Mean.Sp.BodyMassXAbund2 ~~
##     X50km_Pop2                 0.013    0.074    0.174    0.862
##     X10km_Pop2                 0.021    0.064    0.329    0.742
##     GDP.Millions2             -0.079    0.038   -2.055    0.040
##     Nt_Pp._WB20152            -0.004    0.084   -0.044    0.965
##     Prop.Mam.Sp2               0.628    0.126    5.002    0.000
##   X50km_Pop2 ~~
##     X10km_Pop2                 0.752    0.126    5.982    0.000
##     GDP.Millions2             -0.013    0.100   -0.131    0.896
##     Nt_Pp._WB20152             0.191    0.177    1.083    0.279
##     Prop.Mam.Sp2              -0.096    0.051   -1.888    0.059
##   X10km_Pop2 ~~
##     GDP.Millions2             -0.028    0.056   -0.505    0.613
##     Nt_Pp._WB20152             0.173    0.101    1.701    0.089
##     Prop.Mam.Sp2              -0.204    0.055   -3.685    0.000
##   GDP.Millions2 ~~
##     Nt_Pp._WB20152             0.826    0.202    4.089    0.000
##     Prop.Mam.Sp2              -0.123    0.105   -1.172    0.241
##   Nat_Pop._WB20152 ~~
##     Prop.Mam.Sp2              -0.115    0.091   -1.257    0.209
##
## Intercepts:
##                    Estimate  Std.Err  z-value  P(>|z|)
##    .Attendance2      -0.000    0.030   -0.000    1.000
##    .Total.Animals2   -0.000    0.067   -0.000    1.000
##    .Sp.Richness2      0.000    0.034    0.000    1.000
##    .Mean.Rap.Crck2    0.000    0.076    0.000    1.000
##     Zoo.Area.ha2      0.000    0.064    0.000    1.000
##     Mam.Sp.Rchnss2   -0.000    0.079   -0.000    1.000
##     Prop.Mam.Abdn2    0.000    0.113    0.000    1.000
##     Prp.Thrt.Abnd2   -0.000    0.073   -0.000    1.000
##     Mn.Sp.BdyMsXA2    0.000    0.071    0.000    1.000
##     X50km_Pop2       -0.000    0.114   -0.000    1.000
##     X10km_Pop2        0.000    0.105    0.000    1.000
##     GDP.Millions2    -0.000    0.345   -0.000    1.000
##     Nt_Pp._WB20152    0.000    0.295    0.000    1.000
##     Prop.Mam.Sp2      0.000    0.118    0.000    1.000
##
## Variances:
##                    Estimate  Std.Err  z-value  P(>|z|)
##    .Attendance2       0.270    0.028    9.658    0.000
##    .Total.Animals2    0.219    0.037    5.986    0.000
##    .Sp.Richness2      0.151    0.046    3.269    0.001
##    .Mean.Rap.Crck2    0.684    0.059   11.527    0.000
##     Zoo.Area.ha2      0.998    0.112    8.886    0.000
##     Mam.Sp.Rchnss2    0.998    0.115    8.669    0.000
```

```
##     Prop.Mam.Abdn2    0.998    0.121    8.221    0.000
##     Prp.Thrt.Abnd2    0.998    0.125    7.980    0.000
##     Mn.Sp.BdyMsXA2    0.998    0.160    6.219    0.000
##     X50km_Pop2        0.998    0.131    7.623    0.000
##     X10km_Pop2        0.998    0.174    5.745    0.000
##     GDP.Millions2     0.998    0.246    4.061    0.000
##     Nt_Pp._WB20152    0.998    0.225    4.439    0.000
##     Prop.Mam.Sp2      0.998    0.150    6.662    0.000
##
## R-Square:
##                    Estimate
##     Attendance2       0.712
##     Total.Animals2    0.780
##     Sp.Richness2      0.849
##     Mean.Rap.Crck2    0.323
```

```
# Generate fit indices
fitMeasures(fit.adj8A, c("agfi", "rmr", "srmr", "rmsea", "cfi", "nnfi", "tli"))
```

```
##  agfi   rmr  srmr rmsea   cfi  nnfi   tli
## 0.741 0.037 0.035 0.122 0.936 0.872 0.872
```

```
# Generate modification indices
mi8adjA <- modindices(fit.adj8A)
print(mi8adjA[mi8adjA$mi > 3.0,])
```

```
##                           lhs op                     rhs     mi mi.scaled
## 101            Total.Animals2 ~~        Mean.Raup.Crick2 14.977     3.536
## 102              Sp.Richness2 ~~        Mean.Raup.Crick2 13.951     3.293
## 104            Total.Animals2  ~             Attendance2 13.939     3.291
## 105            Total.Animals2  ~        Mean.Raup.Crick2 18.614     4.394
## 109            Total.Animals2  ~              X50km_Pop2 43.332    10.230
## 110            Total.Animals2  ~              X10km_Pop2 15.014     3.544
## 111            Total.Animals2  ~        Nat_Pop._WB20152 10.675     2.520
## 112            Total.Animals2  ~            Prop.Mam.Sp2  3.693     0.872
## 115              Sp.Richness2  ~        Mean.Raup.Crick2  6.484     1.531
## 118              Sp.Richness2  ~ Mean.Sp.BodyMassXAbund2  9.674     2.284
## 122              Sp.Richness2  ~        Nat_Pop._WB20152  3.059     0.722
## 123          Mean.Raup.Crick2  ~             Attendance2  8.581     2.026
## 124          Mean.Raup.Crick2  ~            Zoo.Area.ha2 18.033     4.257
## 125          Mean.Raup.Crick2  ~        Mam.Sp.Richness2 10.168     2.400
## 126          Mean.Raup.Crick2  ~          Prop.Mam.Abdun2  3.616     0.854
## 128          Mean.Raup.Crick2  ~              X50km_Pop2  8.649     2.042
## 129          Mean.Raup.Crick2  ~              X10km_Pop2  6.466     1.527
## 131          Mean.Raup.Crick2  ~        Nat_Pop._WB20152  7.741     1.827
## 132              Zoo.Area.ha2  ~             Attendance2  6.244     1.474
## 133              Zoo.Area.ha2  ~          Total.Animals2  6.234     1.472
## 134              Zoo.Area.ha2  ~            Sp.Richness2 21.924     5.176
## 135              Zoo.Area.ha2  ~        Mean.Raup.Crick2  5.729     1.353
## 147          Mam.Sp.Richness2  ~            Sp.Richness2 10.251     2.420
## 158           Prop.Mam.Abdun2  ~             Attendance2  9.922     2.342
## 159           Prop.Mam.Abdun2  ~          Total.Animals2 14.291     3.374
## 171         Prop.Threat.Abund2  ~             Attendance2  3.167     0.748
## 172         Prop.Threat.Abund2  ~          Total.Animals2  3.585     0.846
## 184 Mean.Sp.BodyMassXAbund2  ~             Attendance2 11.374     2.685
## 185 Mean.Sp.BodyMassXAbund2  ~          Total.Animals2  7.321     1.728
```

```
## 186 Mean.Sp.BodyMassXAbund2  ~          Sp.Richness2  7.054     1.665
## 187 Mean.Sp.BodyMassXAbund2  ~       Mean.Raup.Crick2 26.629     6.287
## 197             X50km_Pop2  ~          Attendance2 27.365     6.460
## 198             X50km_Pop2  ~        Total.Animals2 25.811     6.094
## 200             X50km_Pop2  ~       Mean.Raup.Crick2  3.023     0.714
## 224           GDP.Millions2  ~        Total.Animals2  4.946     1.168
## 225           GDP.Millions2  ~          Sp.Richness2 29.300     6.917
## 226           GDP.Millions2  ~       Mean.Raup.Crick2 12.530     2.958
## 238        Nat_Pop._WB20152  ~          Sp.Richness2 30.182     7.125
## 239        Nat_Pop._WB20152  ~       Mean.Raup.Crick2 17.082     4.033
## 249             Prop.Mam.Sp2  ~          Attendance2 12.625     2.981
## 250             Prop.Mam.Sp2  ~        Total.Animals2 25.206     5.951
## 251             Prop.Mam.Sp2  ~          Sp.Richness2  3.000     0.708
##        epc sepc.lv sepc.all sepc.nox
## 101 -0.137  -0.137   -0.137   -0.137
## 102 -0.063  -0.063   -0.063   -0.063
## 104  0.181   0.181    0.176    0.176
## 105 -0.194  -0.194   -0.196   -0.196
## 109  0.148   0.148    0.148    0.148
## 110  0.091   0.091    0.091    0.092
## 111  0.129   0.129    0.129    0.129
## 112  0.064   0.064    0.064    0.065
## 115 -0.058  -0.058   -0.059   -0.059
## 118 -0.081  -0.081   -0.081   -0.081
## 122 -0.032  -0.032   -0.032   -0.032
## 123  0.241   0.241    0.232    0.232
## 124  0.229   0.229    0.228    0.228
## 125  0.274   0.274    0.273    0.273
## 126  0.156   0.156    0.155    0.156
## 128  0.117   0.117    0.116    0.116
## 129  0.106   0.106    0.105    0.105
## 131  0.111   0.111    0.110    0.110
## 132  0.605   0.605    0.586    0.586
## 133  0.400   0.400    0.399    0.399
## 134  0.882   0.882    0.882    0.882
## 135  0.114   0.114    0.115    0.115
## 147 -0.771  -0.771   -0.771   -0.771
## 158 -0.149  -0.149   -0.145   -0.145
## 159 -0.146  -0.146   -0.146   -0.146
## 171 -0.263  -0.263   -0.255   -0.255
## 172 -0.153  -0.153   -0.153   -0.153
## 184 -0.500  -0.500   -0.485   -0.485
## 185 -0.215  -0.215   -0.215   -0.215
## 186 -0.215  -0.215   -0.215   -0.215
## 187 -0.430  -0.430   -0.432   -0.432
## 197  0.521   0.521    0.505    0.505
## 198  0.273   0.273    0.272    0.272
## 200  0.060   0.060    0.061    0.061
## 224  0.106   0.106    0.106    0.106
## 225  0.332   0.332    0.332    0.332
## 226 -0.100  -0.100   -0.101   -0.101
## 238 -0.326  -0.326   -0.326   -0.326
## 239  0.111   0.111    0.112    0.112
## 249  0.145   0.145    0.141    0.141
```

```
## 250  0.201   0.201    0.201    0.201
## 251  0.105   0.105    0.105    0.105
```

Model 9

Based on the results generated from the nested eight model, the highest p-value relationship to be considered for removal is **Attendance2 ~ Prp.Thrt.Abnd2** with a p-value of **0.468**. Therefore we decide to remove this pathway. As this is the only link involving Prp.Thrt.Abnd2, this also means Prp.Thrt.Abnd2 is subsequently removed from this model.Once again, the model summary, fit indices and modification indices were all generated for the model, adjusting for the nested nature of data.

```
# Attendance SEM (Species Abundance)

# Model 9
# Removal of Attendance2 ~ Prp.Thrt.Abnd2, p = 0.468 (and subsequently Prp.Thrt.Abnd2 completely)

mod.9A <- 'Attendance2 ~ Zoo.Area.ha2 + Sp.Richness2 + Total.Animals2
+ Mam.Sp.Richness2 + Prop.Mam.Abdun2
+ Mean.Sp.BodyMassXAbund2 + Mean.Raup.Crick2
+ X50km_Pop2 + X10km_Pop2 + GDP.Millions2 + Nat_Pop._WB20152

Total.Animals2 ~ Zoo.Area.ha2 + Sp.Richness2 + Mean.Sp.BodyMassXAbund2 + GDP.Millions2
Sp.Richness2 ~ Zoo.Area.ha2 + Prop.Mam.Sp2 + Mam.Sp.Richness2
Mean.Raup.Crick2 ~ Sp.Richness2 + Total.Animals2 + Mean.Sp.BodyMassXAbund2 + Prop.Mam.Sp2'

# Fit model and generate model summary
mod.9A.fit <- sem(mod.9A, data = sem_attendance_data, fixed.x=FALSE)
summary(mod.9A.fit, rsq = TRUE)
```

```
## lavaan (0.5-23.1097) converged normally after  63 iterations
##
##   Number of observations                           458
##
##   Estimator                                         ML
##   Minimum Function Test Statistic              175.120
##   Degrees of freedom                                20
##   P-value (Chi-square)                           0.000
##
## Parameter Estimates:
##
##   Information                                 Expected
##   Standard Errors                             Standard
##
## Regressions:
##                    Estimate  Std.Err  z-value  P(>|z|)
##   Attendance2 ~
##     Zoo.Area.ha2      0.082    0.036    2.299    0.022
##     Sp.Richness2     -0.242    0.066   -3.686    0.000
##     Total.Animals2    0.529    0.054    9.839    0.000
##     Mam.Sp.Rchnss2    0.146    0.048    3.036    0.002
##     Prop.Mam.Abdn2   -0.098    0.040   -2.436    0.015
##     Mn.Sp.BdyMsXA2    0.329    0.037    8.928    0.000
##     Mean.Rap.Crck2    0.117    0.029    4.035    0.000
##     X50km_Pop2        0.078    0.038    2.051    0.040
```

```
##     X10km_Pop2          0.414    0.041   10.062    0.000
##     GDP.Millions2       0.260    0.048    5.391    0.000
##     Nt_Pp._WB20152     -0.129    0.049   -2.611    0.009
##   Total.Animals2 ~
##     Zoo.Area.ha2        0.309    0.027   11.444    0.000
##     Sp.Richness2        0.759    0.023   32.580    0.000
##     Mn.Sp.BdyMsXA2     -0.157    0.027   -5.828    0.000
##     GDP.Millions2      -0.136    0.022   -6.202    0.000
##   Sp.Richness2 ~
##     Zoo.Area.ha2        0.067    0.021    3.225    0.001
##     Prop.Mam.Sp2       -0.581    0.019  -30.364    0.000
##     Mam.Sp.Rchnss2      0.755    0.020   38.373    0.000
##   Mean.Raup.Crick2 ~
##     Sp.Richness2       -0.467    0.077   -6.082    0.000
##     Total.Animals2      0.478    0.071    6.732    0.000
##     Mn.Sp.BdyMsXA2     -0.246    0.051   -4.808    0.000
##     Prop.Mam.Sp2       -0.366    0.058   -6.278    0.000
##
## Covariances:
##                             Estimate  Std.Err  z-value  P(>|z|)
##   Zoo.Area.ha2 ~~
##     Mam.Sp.Rchnss2             0.381    0.050    7.634    0.000
##     Prop.Mam.Abdn2             0.352    0.049    7.121    0.000
##     Mn.Sp.BdyMsXA2             0.522    0.053    9.923    0.000
##     X50km_Pop2                 0.060    0.047    1.291    0.197
##     X10km_Pop2                -0.010    0.047   -0.208    0.835
##     GDP.Millions2             -0.027    0.047   -0.588    0.557
##     Nt_Pp._WB20152             0.061    0.047    1.310    0.190
##     Prop.Mam.Sp2               0.312    0.049    6.389    0.000
##   Mam.Sp.Richness2 ~~
##     Prop.Mam.Abdn2             0.058    0.047    1.237    0.216
##     Mn.Sp.BdyMsXA2             0.227    0.048    4.750    0.000
##     X50km_Pop2                 0.204    0.048    4.281    0.000
##     X10km_Pop2                 0.284    0.048    5.852    0.000
##     GDP.Millions2             -0.060    0.047   -1.277    0.202
##     Nt_Pp._WB20152            -0.088    0.047   -1.881    0.060
##     Prop.Mam.Sp2               0.088    0.047    1.882    0.060
##   Prop.Mam.Abdun2 ~~
##     Mn.Sp.BdyMsXA2             0.592    0.054   10.922    0.000
##     X50km_Pop2                -0.123    0.047   -2.622    0.009
##     X10km_Pop2                -0.248    0.048   -5.165    0.000
##     GDP.Millions2             -0.115    0.047   -2.458    0.014
##     Nt_Pp._WB20152            -0.111    0.047   -2.362    0.018
##     Prop.Mam.Sp2               0.878    0.062   14.135    0.000
##   Mean.Sp.BodyMassXAbund2 ~~
##     X50km_Pop2                 0.013    0.047    0.275    0.784
##     X10km_Pop2                 0.021    0.047    0.455    0.649
##     GDP.Millions2             -0.079    0.047   -1.682    0.093
##     Nt_Pp._WB20152            -0.004    0.047   -0.079    0.937
##     Prop.Mam.Sp2               0.628    0.055   11.405    0.000
##   X50km_Pop2 ~~
##     X10km_Pop2                 0.752    0.058   12.886    0.000
##     GDP.Millions2             -0.013    0.047   -0.281    0.779
##     Nt_Pp._WB20152             0.191    0.047    4.033    0.000
```

```
##     Prop.Mam.Sp2              -0.096    0.047   -2.054    0.040
##   X10km_Pop2 ~~
##     GDP.Millions2             -0.028    0.047   -0.604    0.546
##     Nt_Pp._WB20152             0.173    0.047    3.646    0.000
##     Prop.Mam.Sp2              -0.204    0.048   -4.293    0.000
##   GDP.Millions2 ~~
##     Nt_Pp._WB20152             0.826    0.061   13.642    0.000
##     Prop.Mam.Sp2              -0.123    0.047   -2.615    0.009
##   Nat_Pop._WB20152 ~~
##     Prop.Mam.Sp2              -0.115    0.047   -2.447    0.014
##
## Variances:
##                    Estimate  Std.Err  z-value  P(>|z|)
##    .Attendance2       0.271    0.018   15.133    0.000
##    .Total.Animals2    0.219    0.014   15.133    0.000
##    .Sp.Richness2      0.151    0.010   15.133    0.000
##    .Mean.Rap.Crck2    0.684    0.045   15.133    0.000
##     Zoo.Area.ha2      0.998    0.066   15.133    0.000
##     Mam.Sp.Rchnss2    0.998    0.066   15.133    0.000
##     Prop.Mam.Abdn2    0.998    0.066   15.133    0.000
##     Mn.Sp.BdyMsXA2    0.998    0.066   15.133    0.000
##     X50km_Pop2        0.998    0.066   15.133    0.000
##     X10km_Pop2        0.998    0.066   15.133    0.000
##     GDP.Millions2     0.998    0.066   15.133    0.000
##     Nt_Pp._WB20152    0.998    0.066   15.133    0.000
##     Prop.Mam.Sp2      0.998    0.066   15.133    0.000
##
## R-Square:
##                    Estimate
##     Attendance2       0.711
##     Total.Animals2    0.780
##     Sp.Richness2      0.849
##     Mean.Rap.Crck2    0.323
```

```r
# Generate fit indices
fitMeasures(mod.9A.fit, c("agfi", "rmr", "srmr", "rmsea", "cfi", "nnfi", "tli"))
```

```
##  agfi   rmr  srmr rmsea   cfi  nnfi   tli
## 0.763 0.039 0.039 0.130 0.937 0.868 0.868
```

```r
# Generate modification indices
mi9A <- modindices(mod.9A.fit)
print(mi9A[mi9A$mi > 3.0,])
```

```
##                       lhs op                  rhs     mi    epc
## 76         Total.Animals2 ~~       Mean.Raup.Crick2 14.977 -0.137
## 77           Sp.Richness2 ~~       Mean.Raup.Crick2 13.951 -0.063
## 79         Total.Animals2  ~            Attendance2 14.520  0.185
## 80         Total.Animals2  ~       Mean.Raup.Crick2 18.614 -0.194
## 83         Total.Animals2  ~             X50km_Pop2 43.332  0.148
## 84         Total.Animals2  ~             X10km_Pop2 15.014  0.091
## 85         Total.Animals2  ~       Nat_Pop._WB20152 10.674  0.129
## 86         Total.Animals2  ~           Prop.Mam.Sp2  3.693  0.064
## 89           Sp.Richness2  ~       Mean.Raup.Crick2  6.484 -0.058
## 91           Sp.Richness2  ~ Mean.Sp.BodyMassXAbund2  9.674 -0.081
## 95           Sp.Richness2  ~       Nat_Pop._WB20152  3.059 -0.032
```

```
## 96         Mean.Raup.Crick2  ~             Attendance2  8.518  0.240
## 97         Mean.Raup.Crick2  ~            Zoo.Area.ha2 18.033  0.229
## 98         Mean.Raup.Crick2  ~        Mam.Sp.Richness2 10.168  0.274
## 99         Mean.Raup.Crick2  ~         Prop.Mam.Abdun2  3.617  0.156
## 100        Mean.Raup.Crick2  ~             X50km_Pop2  8.649  0.117
## 101        Mean.Raup.Crick2  ~             X10km_Pop2  6.466  0.106
## 103        Mean.Raup.Crick2  ~        Nat_Pop._WB20152  7.741  0.111
## 104            Zoo.Area.ha2  ~             Attendance2  7.209  0.649
## 105            Zoo.Area.ha2  ~          Total.Animals2  7.437  0.440
## 106            Zoo.Area.ha2  ~            Sp.Richness2 22.368  0.894
## 107            Zoo.Area.ha2  ~        Mean.Raup.Crick2  5.584  0.113
## 118        Mam.Sp.Richness2  ~            Sp.Richness2 10.502 -0.783
## 128         Prop.Mam.Abdun2  ~             Attendance2  9.783 -0.150
## 129         Prop.Mam.Abdun2  ~          Total.Animals2 13.134 -0.141
## 140 Mean.Sp.BodyMassXAbund2  ~             Attendance2 13.127 -0.536
## 141 Mean.Sp.BodyMassXAbund2  ~          Total.Animals2  8.775 -0.237
## 142 Mean.Sp.BodyMassXAbund2  ~            Sp.Richness2  7.245 -0.218
## 143 Mean.Sp.BodyMassXAbund2  ~        Mean.Raup.Crick2 26.364 -0.436
## 152              X50km_Pop2  ~             Attendance2 25.746  0.509
## 153              X50km_Pop2  ~          Total.Animals2 24.323  0.266
## 155              X50km_Pop2  ~        Mean.Raup.Crick2  3.252  0.063
## 177           GDP.Millions2  ~          Total.Animals2  4.674  0.103
## 178           GDP.Millions2  ~            Sp.Richness2 29.233  0.332
## 179           GDP.Millions2  ~        Mean.Raup.Crick2 12.402 -0.100
## 189        Nat_Pop._WB20152  ~          Total.Animals2  3.151 -0.074
## 190        Nat_Pop._WB20152  ~            Sp.Richness2 30.274 -0.326
## 191        Nat_Pop._WB20152  ~        Mean.Raup.Crick2 17.218  0.111
## 200            Prop.Mam.Sp2  ~             Attendance2 12.413  0.145
## 201            Prop.Mam.Sp2  ~          Total.Animals2 23.101  0.195
##     sepc.lv sepc.all sepc.nox
## 76   -0.137   -0.137   -0.137
## 77   -0.063   -0.063   -0.063
## 79    0.185    0.180    0.180
## 80   -0.194   -0.196   -0.196
## 83    0.148    0.148    0.148
## 84    0.091    0.091    0.092
## 85    0.129    0.129    0.129
## 86    0.064    0.064    0.065
## 89   -0.058   -0.059   -0.059
## 91   -0.081   -0.081   -0.081
## 95   -0.032   -0.032   -0.032
## 96    0.240    0.231    0.231
## 97    0.229    0.228    0.228
## 98    0.274    0.273    0.273
## 99    0.156    0.155    0.156
## 100   0.117    0.116    0.116
## 101   0.106    0.105    0.105
## 103   0.111    0.110    0.110
## 104   0.649    0.629    0.629
## 105   0.440    0.439    0.439
## 106   0.894    0.894    0.894
## 107   0.113    0.113    0.113
## 118  -0.783   -0.783   -0.783
## 128  -0.150   -0.145   -0.145
```

```
## 129  -0.141   -0.140   -0.140
## 140  -0.536   -0.519   -0.519
## 141  -0.237   -0.237   -0.237
## 142  -0.218   -0.218   -0.218
## 143  -0.436   -0.439   -0.439
## 152   0.509    0.494    0.494
## 153   0.266    0.265    0.265
## 155   0.063    0.063    0.063
## 177   0.103    0.103    0.103
## 178   0.332    0.332    0.332
## 179  -0.100   -0.100   -0.100
## 189  -0.074   -0.074   -0.074
## 190  -0.326   -0.326   -0.326
## 191   0.111    0.112    0.112
## 200   0.145    0.141    0.141
## 201   0.195    0.194    0.194
```

```
# Adjust for the nested nature of the data (institutions within countries)
# Fit model and generate model summary
design <- svydesign(ids = ~Country, nest=TRUE, data=sem_attendance_data)
fit.adj9A <- lavaan.survey(lavaan.fit = mod.9A.fit, survey.design = design)
summary(fit.adj9A, rsq = TRUE)
```

```
## lavaan (0.5-23.1097) converged normally after  63 iterations
##
##   Number of observations                           458
##
##   Estimator                                         ML      Robust
##   Minimum Function Test Statistic              175.120      38.034
##   Degrees of freedom                                20          20
##   P-value (Chi-square)                           0.000       0.009
##   Scaling correction factor                                  4.604
##     for the Satorra-Bentler correction
##
## Parameter Estimates:
##
##   Information                                 Expected
##   Standard Errors                           Robust.sem
##
## Regressions:
##                      Estimate  Std.Err  z-value  P(>|z|)
##   Attendance2 ~
##     Zoo.Area.ha2        0.082    0.043    1.914    0.056
##     Sp.Richness2       -0.242    0.071   -3.395    0.001
##     Total.Animals2      0.529    0.064    8.307    0.000
##     Mam.Sp.Rchnss2      0.146    0.042    3.466    0.001
##     Prop.Mam.Abdn2     -0.098    0.032   -3.051    0.002
##     Mn.Sp.BdyMsXA2      0.329    0.025   13.111    0.000
##     Mean.Rap.Crck2      0.117    0.034    3.467    0.001
##     X50km_Pop2          0.078    0.031    2.527    0.012
##     X10km_Pop2          0.414    0.042    9.852    0.000
##     GDP.Millions2       0.260    0.058    4.465    0.000
##     Nt_Pp._WB20152     -0.129    0.063   -2.031    0.042
##   Total.Animals2 ~
##     Zoo.Area.ha2        0.309    0.042    7.287    0.000
```

```
##     Sp.Richness2        0.759    0.049   15.353    0.000
##     Mn.Sp.BdyMsXA2     -0.157    0.034   -4.588    0.000
##     GDP.Millions2      -0.136    0.069   -1.983    0.047
##   Sp.Richness2 ~
##     Zoo.Area.ha2        0.067    0.044    1.519    0.129
##     Prop.Mam.Sp2       -0.581    0.038  -15.231    0.000
##     Mam.Sp.Rchnss2      0.755    0.068   11.066    0.000
##   Mean.Raup.Crick2 ~
##     Sp.Richness2       -0.467    0.188   -2.482    0.013
##     Total.Animals2      0.478    0.116    4.127    0.000
##     Mn.Sp.BdyMsXA2     -0.246    0.084   -2.924    0.003
##     Prop.Mam.Sp2       -0.366    0.080   -4.580    0.000
##
## Covariances:
##                            Estimate  Std.Err  z-value  P(>|z|)
##   Zoo.Area.ha2 ~~
##     Mam.Sp.Rchnss2            0.381    0.064    5.985    0.000
##     Prop.Mam.Abdn2            0.352    0.092    3.822    0.000
##     Mn.Sp.BdyMsXA2            0.522    0.101    5.177    0.000
##     X50km_Pop2                0.060    0.069    0.880    0.379
##     X10km_Pop2               -0.010    0.078   -0.124    0.901
##     GDP.Millions2            -0.027    0.041   -0.673    0.501
##     Nt_Pp._WB20152            0.061    0.090    0.682    0.495
##     Prop.Mam.Sp2              0.312    0.089    3.510    0.000
##   Mam.Sp.Richness2 ~~
##     Prop.Mam.Abdn2            0.058    0.049    1.172    0.241
##     Mn.Sp.BdyMsXA2            0.227    0.078    2.921    0.003
##     X50km_Pop2                0.204    0.082    2.487    0.013
##     X10km_Pop2                0.284    0.066    4.278    0.000
##     GDP.Millions2            -0.060    0.064   -0.937    0.349
##     Nt_Pp._WB20152           -0.088    0.102   -0.863    0.388
##     Prop.Mam.Sp2              0.088    0.061    1.435    0.151
##   Prop.Mam.Abdun2 ~~
##     Mn.Sp.BdyMsXA2            0.592    0.109    5.445    0.000
##     X50km_Pop2               -0.123    0.054   -2.263    0.024
##     X10km_Pop2               -0.248    0.060   -4.154    0.000
##     GDP.Millions2            -0.115    0.101   -1.140    0.254
##     Nt_Pp._WB20152           -0.111    0.088   -1.264    0.206
##     Prop.Mam.Sp2              0.878    0.128    6.865    0.000
##   Mean.Sp.BodyMassXAbund2 ~~
##     X50km_Pop2                0.013    0.074    0.174    0.862
##     X10km_Pop2                0.021    0.064    0.329    0.742
##     GDP.Millions2            -0.079    0.038   -2.055    0.040
##     Nt_Pp._WB20152           -0.004    0.084   -0.044    0.965
##     Prop.Mam.Sp2              0.628    0.126    5.002    0.000
##   X50km_Pop2 ~~
##     X10km_Pop2                0.752    0.126    5.982    0.000
##     GDP.Millions2            -0.013    0.100   -0.131    0.896
##     Nt_Pp._WB20152            0.191    0.177    1.083    0.279
##     Prop.Mam.Sp2             -0.096    0.051   -1.888    0.059
##   X10km_Pop2 ~~
##     GDP.Millions2            -0.028    0.056   -0.505    0.613
##     Nt_Pp._WB20152            0.173    0.101    1.701    0.089
##     Prop.Mam.Sp2             -0.204    0.055   -3.685    0.000
```

```
##   GDP.Millions2 ~~
##     Nt_Pp._WB20152             0.826    0.202    4.089    0.000
##     Prop.Mam.Sp2              -0.123    0.105   -1.172    0.241
##   Nat_Pop._WB20152 ~~
##     Prop.Mam.Sp2              -0.115    0.091   -1.257    0.209
##
## Intercepts:
##                    Estimate  Std.Err  z-value  P(>|z|)
##    .Attendance2      -0.000    0.029   -0.000    1.000
##    .Total.Animals2   -0.000    0.067   -0.000    1.000
##    .Sp.Richness2      0.000    0.034    0.000    1.000
##    .Mean.Rap.Crck2    0.000    0.076    0.000    1.000
##     Zoo.Area.ha2      0.000    0.064    0.000    1.000
##     Mam.Sp.Rchnss2   -0.000    0.079   -0.000    1.000
##     Prop.Mam.Abdn2    0.000    0.113    0.000    1.000
##     Mn.Sp.BdyMsXA2    0.000    0.071    0.000    1.000
##     X50km_Pop2       -0.000    0.114   -0.000    1.000
##     X10km_Pop2        0.000    0.105    0.000    1.000
##     GDP.Millions2    -0.000    0.345   -0.000    1.000
##     Nt_Pp._WB20152    0.000    0.295    0.000    1.000
##     Prop.Mam.Sp2      0.000    0.118    0.000    1.000
##
## Variances:
##                    Estimate  Std.Err  z-value  P(>|z|)
##    .Attendance2       0.271    0.028    9.724    0.000
##    .Total.Animals2    0.219    0.037    5.986    0.000
##    .Sp.Richness2      0.151    0.046    3.269    0.001
##    .Mean.Rap.Crck2    0.684    0.059   11.527    0.000
##     Zoo.Area.ha2      0.998    0.112    8.886    0.000
##     Mam.Sp.Rchnss2    0.998    0.115    8.669    0.000
##     Prop.Mam.Abdn2    0.998    0.121    8.221    0.000
##     Mn.Sp.BdyMsXA2    0.998    0.160    6.219    0.000
##     X50km_Pop2        0.998    0.131    7.623    0.000
##     X10km_Pop2        0.998    0.174    5.745    0.000
##     GDP.Millions2     0.998    0.246    4.061    0.000
##     Nt_Pp._WB20152    0.998    0.225    4.439    0.000
##     Prop.Mam.Sp2      0.998    0.150    6.662    0.000
##
## R-Square:
##                    Estimate
##     Attendance2       0.711
##     Total.Animals2    0.780
##     Sp.Richness2      0.849
##     Mean.Rap.Crck2    0.323
```

```r
# Generate fit indices
fitMeasures(fit.adj9A, c("agfi", "rmr", "srmr", "rmsea", "cfi", "nnfi", "tli"))
```

```
##  agfi   rmr  srmr rmsea   cfi  nnfi   tli
## 0.729 0.039 0.037 0.130 0.937 0.868 0.868
```

```r
# Generate modification indices
mi9adjA <- modindices(fit.adj9A)
print(mi9adjA[mi9adjA$mi > 3.0,])
```

```
##                          lhs op                       rhs     mi mi.scaled
```

```
## 89          Total.Animals2 ~~        Mean.Raup.Crick2 14.977     3.253
## 90            Sp.Richness2 ~~        Mean.Raup.Crick2 13.950     3.030
## 92          Total.Animals2  ~             Attendance2 14.520     3.153
## 93          Total.Animals2  ~        Mean.Raup.Crick2 18.614     4.043
## 96          Total.Animals2  ~             X50km_Pop2 43.332     9.411
## 97          Total.Animals2  ~             X10km_Pop2 15.014     3.261
## 98          Total.Animals2  ~        Nat_Pop._WB20152 10.674     2.318
## 99          Total.Animals2  ~            Prop.Mam.Sp2  3.693     0.802
## 102           Sp.Richness2  ~        Mean.Raup.Crick2  6.484     1.408
## 104           Sp.Richness2  ~ Mean.Sp.BodyMassXAbund2  9.674     2.101
## 108           Sp.Richness2  ~        Nat_Pop._WB20152  3.059     0.664
## 109       Mean.Raup.Crick2  ~             Attendance2  8.518     1.850
## 110       Mean.Raup.Crick2  ~            Zoo.Area.ha2 18.033     3.916
## 111       Mean.Raup.Crick2  ~        Mam.Sp.Richness2 10.168     2.208
## 112       Mean.Raup.Crick2  ~          Prop.Mam.Abdun2  3.616     0.785
## 113       Mean.Raup.Crick2  ~              X50km_Pop2  8.649     1.878
## 114       Mean.Raup.Crick2  ~              X10km_Pop2  6.466     1.404
## 116       Mean.Raup.Crick2  ~        Nat_Pop._WB20152  7.741     1.681
## 117           Zoo.Area.ha2  ~             Attendance2  7.209     1.566
## 118           Zoo.Area.ha2  ~          Total.Animals2  7.438     1.615
## 119           Zoo.Area.ha2  ~            Sp.Richness2 22.368     4.858
## 120           Zoo.Area.ha2  ~        Mean.Raup.Crick2  5.584     1.213
## 131       Mam.Sp.Richness2  ~            Sp.Richness2 10.502     2.281
## 141        Prop.Mam.Abdun2  ~             Attendance2  9.783     2.125
## 142        Prop.Mam.Abdun2  ~          Total.Animals2 13.134     2.852
## 153 Mean.Sp.BodyMassXAbund2  ~             Attendance2 13.127     2.851
## 154 Mean.Sp.BodyMassXAbund2  ~          Total.Animals2  8.775     1.906
## 155 Mean.Sp.BodyMassXAbund2  ~            Sp.Richness2  7.245     1.573
## 156 Mean.Sp.BodyMassXAbund2  ~        Mean.Raup.Crick2 26.364     5.726
## 165             X50km_Pop2  ~             Attendance2 25.746     5.592
## 166             X50km_Pop2  ~          Total.Animals2 24.323     5.283
## 168             X50km_Pop2  ~        Mean.Raup.Crick2  3.252     0.706
## 190          GDP.Millions2  ~          Total.Animals2  4.674     1.015
## 191          GDP.Millions2  ~            Sp.Richness2 29.233     6.349
## 192          GDP.Millions2  ~        Mean.Raup.Crick2 12.402     2.693
## 202       Nat_Pop._WB20152  ~          Total.Animals2  3.151     0.684
## 203       Nat_Pop._WB20152  ~            Sp.Richness2 30.274     6.575
## 204       Nat_Pop._WB20152  ~        Mean.Raup.Crick2 17.218     3.739
## 213           Prop.Mam.Sp2  ~             Attendance2 12.413     2.696
## 214           Prop.Mam.Sp2  ~          Total.Animals2 23.101     5.017
##        epc sepc.lv sepc.all sepc.nox
## 89  -0.137  -0.137   -0.137   -0.137
## 90  -0.063  -0.063   -0.063   -0.063
## 92   0.185   0.185    0.180    0.180
## 93  -0.194  -0.194   -0.196   -0.196
## 96   0.148   0.148    0.148    0.148
## 97   0.091   0.091    0.091    0.092
## 98   0.129   0.129    0.129    0.129
## 99   0.064   0.064    0.064    0.065
## 102 -0.058  -0.058   -0.059   -0.059
## 104 -0.081  -0.081   -0.081   -0.081
## 108 -0.032  -0.032   -0.032   -0.032
## 109  0.240   0.240    0.231    0.231
## 110  0.229   0.229    0.228    0.228
```

```
## 111  0.274   0.274    0.273    0.273
## 112  0.156   0.156    0.155    0.156
## 113  0.117   0.117    0.116    0.116
## 114  0.106   0.106    0.105    0.105
## 116  0.111   0.111    0.110    0.110
## 117  0.649   0.649    0.629    0.629
## 118  0.440   0.440    0.439    0.439
## 119  0.894   0.894    0.894    0.894
## 120  0.113   0.113    0.113    0.113
## 131 -0.783  -0.783   -0.783   -0.783
## 141 -0.150  -0.150   -0.145   -0.145
## 142 -0.141  -0.141   -0.140   -0.140
## 153 -0.536  -0.536   -0.519   -0.519
## 154 -0.237  -0.237   -0.237   -0.237
## 155 -0.218  -0.218   -0.218   -0.218
## 156 -0.436  -0.436   -0.439   -0.439
## 165  0.509   0.509    0.494    0.494
## 166  0.266   0.266    0.265    0.265
## 168  0.063   0.063    0.063    0.063
## 190  0.103   0.103    0.103    0.103
## 191  0.332   0.332    0.332    0.332
## 192 -0.100  -0.100   -0.100   -0.100
## 202 -0.074  -0.074   -0.074   -0.074
## 203 -0.326  -0.326   -0.326   -0.326
## 204  0.111   0.111    0.112    0.112
## 213  0.145   0.145    0.141    0.141
## 214  0.195   0.195    0.194    0.194
```

Model 10

Based on the results generated from the nested ninth model, the highest p-value relationship to be considered for removal is **Sp.Richness2 ~ Zoo.Area.ha2** with a p-value of **0.129**. Therefore we decide to remove this pathway. Once again, the model summary, fit indices and modification indices were all generated for the model, adjusting for the nested nature of data.

```
# Attendance SEM (Species Abundance)

# Model 10
# Removal of Sp.Richness2 ~ Zoo.Area.ha2, p = 0.129

mod.10A <- 'Attendance2 ~ Zoo.Area.ha2 + Sp.Richness2 + Total.Animals2
+ Mam.Sp.Richness2 + Prop.Mam.Abdun2
+ Mean.Sp.BodyMassXAbund2 + Mean.Raup.Crick2
+ X50km_Pop2 + X10km_Pop2 + GDP.Millions2 + Nat_Pop._WB20152

Total.Animals2 ~ Zoo.Area.ha2 + Sp.Richness2 + Mean.Sp.BodyMassXAbund2 + GDP.Millions2
Sp.Richness2 ~ Prop.Mam.Sp2 + Mam.Sp.Richness2
Mean.Raup.Crick2 ~ Sp.Richness2 + Total.Animals2 + Mean.Sp.BodyMassXAbund2 + Prop.Mam.Sp2'

# Fit model and generate model summary
mod.10A.fit <- sem(mod.10A, data = sem_attendance_data, fixed.x=FALSE)
summary(mod.10A.fit, rsq = TRUE)
```

```
## lavaan (0.5-23.1097) converged normally after  65 iterations
```

```
##
##   Number of observations                           458
##
##   Estimator                                         ML
##   Minimum Function Test Statistic              185.403
##   Degrees of freedom                                21
##   P-value (Chi-square)                           0.000
##
## Parameter Estimates:
##
##   Information                                 Expected
##   Standard Errors                             Standard
##
## Regressions:
##                      Estimate  Std.Err  z-value  P(>|z|)
##   Attendance2 ~
##     Zoo.Area.ha2        0.082    0.035    2.326    0.020
##     Sp.Richness2       -0.242    0.066   -3.680    0.000
##     Total.Animals2      0.529    0.054    9.839    0.000
##     Mam.Sp.Rchnss2      0.146    0.049    2.970    0.003
##     Prop.Mam.Abdn2     -0.098    0.040   -2.459    0.014
##     Mn.Sp.BdyMsXA2      0.329    0.037    8.934    0.000
##     Mean.Rap.Crck2      0.117    0.029    4.036    0.000
##     X50km_Pop2          0.078    0.038    2.051    0.040
##     X10km_Pop2          0.414    0.041   10.062    0.000
##     GDP.Millions2       0.260    0.048    5.391    0.000
##     Nt_Pp._WB20152     -0.129    0.049   -2.611    0.009
##   Total.Animals2 ~
##     Zoo.Area.ha2        0.309    0.027   11.617    0.000
##     Sp.Richness2        0.759    0.023   32.975    0.000
##     Mn.Sp.BdyMsXA2     -0.157    0.027   -5.852    0.000
##     GDP.Millions2      -0.136    0.022   -6.202    0.000
##   Sp.Richness2 ~
##     Prop.Mam.Sp2       -0.563    0.018  -30.494    0.000
##     Mam.Sp.Rchnss2      0.779    0.018   42.208    0.000
##   Mean.Raup.Crick2 ~
##     Sp.Richness2       -0.467    0.075   -6.198    0.000
##     Total.Animals2      0.478    0.071    6.762    0.000
##     Mn.Sp.BdyMsXA2     -0.246    0.051   -4.837    0.000
##     Prop.Mam.Sp2       -0.366    0.058   -6.343    0.000
##
## Covariances:
##                              Estimate  Std.Err  z-value  P(>|z|)
##   Zoo.Area.ha2 ~~
##     Mam.Sp.Rchnss2              0.381    0.050    7.634    0.000
##     Prop.Mam.Abdn2              0.352    0.049    7.121    0.000
##     Mn.Sp.BdyMsXA2              0.522    0.053    9.923    0.000
##     X50km_Pop2                  0.060    0.047    1.291    0.197
##     X10km_Pop2                 -0.010    0.047   -0.208    0.835
##     GDP.Millions2              -0.027    0.047   -0.588    0.557
##     Nt_Pp._WB20152              0.061    0.047    1.310    0.190
##     Prop.Mam.Sp2                0.312    0.049    6.389    0.000
##   Mam.Sp.Richness2 ~~
##     Prop.Mam.Abdn2              0.058    0.047    1.237    0.216
```

```
##     Mn.Sp.BdyMsXA2            0.227    0.048    4.750    0.000
##     X50km_Pop2                0.204    0.048    4.281    0.000
##     X10km_Pop2                0.284    0.048    5.852    0.000
##     GDP.Millions2            -0.060    0.047   -1.277    0.202
##     Nt_Pp._WB20152           -0.088    0.047   -1.881    0.060
##     Prop.Mam.Sp2              0.088    0.047    1.882    0.060
##   Prop.Mam.Abdun2 ~~
##     Mn.Sp.BdyMsXA2            0.592    0.054   10.922    0.000
##     X50km_Pop2               -0.123    0.047   -2.622    0.009
##     X10km_Pop2               -0.248    0.048   -5.165    0.000
##     GDP.Millions2            -0.115    0.047   -2.458    0.014
##     Nt_Pp._WB20152           -0.111    0.047   -2.362    0.018
##     Prop.Mam.Sp2              0.878    0.062   14.135    0.000
##   Mean.Sp.BodyMassXAbund2 ~~
##     X50km_Pop2                0.013    0.047    0.275    0.784
##     X10km_Pop2                0.021    0.047    0.455    0.649
##     GDP.Millions2            -0.079    0.047   -1.682    0.093
##     Nt_Pp._WB20152           -0.004    0.047   -0.079    0.937
##     Prop.Mam.Sp2              0.628    0.055   11.405    0.000
##   X50km_Pop2 ~~
##     X10km_Pop2                0.752    0.058   12.886    0.000
##     GDP.Millions2            -0.013    0.047   -0.281    0.779
##     Nt_Pp._WB20152            0.191    0.047    4.033    0.000
##     Prop.Mam.Sp2             -0.096    0.047   -2.054    0.040
##   X10km_Pop2 ~~
##     GDP.Millions2            -0.028    0.047   -0.604    0.546
##     Nt_Pp._WB20152            0.173    0.047    3.646    0.000
##     Prop.Mam.Sp2             -0.204    0.048   -4.293    0.000
##   GDP.Millions2 ~~
##     Nt_Pp._WB20152            0.826    0.061   13.642    0.000
##     Prop.Mam.Sp2             -0.123    0.047   -2.615    0.009
##   Nat_Pop._WB20152 ~~
##     Prop.Mam.Sp2             -0.115    0.047   -2.447    0.014
##
## Variances:
##                    Estimate  Std.Err  z-value  P(>|z|)
##    .Attendance2       0.271    0.018   15.133    0.000
##    .Total.Animals2    0.219    0.014   15.133    0.000
##    .Sp.Richness2      0.154    0.010   15.133    0.000
##    .Mean.Rap.Crck2    0.684    0.045   15.133    0.000
##     Zoo.Area.ha2      0.998    0.066   15.133    0.000
##     Mam.Sp.Rchnss2    0.998    0.066   15.133    0.000
##     Prop.Mam.Abdn2    0.998    0.066   15.133    0.000
##     Mn.Sp.BdyMsXA2    0.998    0.066   15.133    0.000
##     X50km_Pop2        0.998    0.066   15.133    0.000
##     X10km_Pop2        0.998    0.066   15.133    0.000
##     GDP.Millions2     0.998    0.066   15.133    0.000
##     Nt_Pp._WB20152    0.998    0.066   15.133    0.000
##     Prop.Mam.Sp2      0.998    0.066   15.133    0.000
##
## R-Square:
##                    Estimate
##     Attendance2       0.710
##     Total.Animals2    0.775
```

```
##     Sp.Richness2      0.845
##     Mean.Rap.Crck2    0.323
```

```r
# Generate fit indices
fitMeasures(mod.10A.fit, c("agfi", "rmr", "srmr", "rmsea", "cfi", "nnfi", "tli"))
```

```
##  agfi   rmr  srmr rmsea   cfi  nnfi   tli
## 0.766 0.040 0.040 0.131 0.933 0.867 0.867
```

```r
# Generate modification indices
mi10A <- modindices(mod.10A.fit)
print(mi10A[mi10A$mi > 3.0,])
```

```
##                          lhs op              rhs     mi    epc sepc.lv
## 75            Total.Animals2 ~~ Mean.Raup.Crick2 14.603 -0.134  -0.134
## 76              Sp.Richness2 ~~ Mean.Raup.Crick2 10.304 -0.055  -0.055
## 78            Total.Animals2  ~      Attendance2 14.492  0.185   0.185
## 79            Total.Animals2  ~ Mean.Raup.Crick2 18.135 -0.189  -0.189
## 82            Total.Animals2  ~       X50km_Pop2 43.331  0.148   0.148
## 83            Total.Animals2  ~       X10km_Pop2 14.994  0.091   0.091
## 84            Total.Animals2  ~ Nat_Pop._WB20152 10.679  0.129   0.129
## 85            Total.Animals2  ~     Prop.Mam.Sp2  3.589  0.062   0.062
## 87              Sp.Richness2  ~   Total.Animals2  4.049  0.072   0.072
## 88              Sp.Richness2  ~ Mean.Raup.Crick2  3.898 -0.046  -0.046
## 89              Sp.Richness2  ~     Zoo.Area.ha2 10.169  0.067   0.067
## 96          Mean.Raup.Crick2  ~      Attendance2  8.498  0.240   0.240
## 97          Mean.Raup.Crick2  ~     Zoo.Area.ha2 17.464  0.222   0.222
## 98          Mean.Raup.Crick2  ~ Mam.Sp.Richness2 10.304  0.278   0.278
## 99          Mean.Raup.Crick2  ~  Prop.Mam.Abdun2  3.621  0.156   0.156
## 100         Mean.Raup.Crick2  ~       X50km_Pop2  8.651  0.117   0.117
## 101         Mean.Raup.Crick2  ~       X10km_Pop2  6.491  0.106   0.106
## 103         Mean.Raup.Crick2  ~ Nat_Pop._WB20152  7.752  0.111   0.111
## 104             Zoo.Area.ha2  ~      Attendance2 12.756  0.818   0.818
## 105             Zoo.Area.ha2  ~   Total.Animals2 17.595  0.440   0.440
## 106             Zoo.Area.ha2  ~     Sp.Richness2 25.670  0.463   0.463
## 107             Zoo.Area.ha2  ~ Mean.Raup.Crick2  4.898  0.106   0.106
## 117         Mam.Sp.Richness2  ~   Total.Animals2  3.415 -0.192  -0.192
## 118         Mam.Sp.Richness2  ~     Sp.Richness2 20.237 -0.833  -0.833
## 128          Prop.Mam.Abdun2  ~      Attendance2  9.637 -0.148  -0.148
## 129          Prop.Mam.Abdun2  ~   Total.Animals2 13.050 -0.140  -0.140
## 140 Mean.Sp.BodyMassXAbund2  ~      Attendance2 12.839 -0.524  -0.524
## 141 Mean.Sp.BodyMassXAbund2  ~   Total.Animals2  8.595 -0.233  -0.233
## 142 Mean.Sp.BodyMassXAbund2  ~     Sp.Richness2  7.084 -0.213  -0.213
## 143 Mean.Sp.BodyMassXAbund2  ~ Mean.Raup.Crick2 25.521 -0.422  -0.422
## 152               X50km_Pop2  ~      Attendance2 25.720  0.509   0.509
## 153               X50km_Pop2  ~   Total.Animals2 24.167  0.264   0.264
## 155               X50km_Pop2  ~ Mean.Raup.Crick2  3.252  0.063   0.063
## 177            GDP.Millions2  ~   Total.Animals2  4.637  0.103   0.103
## 178            GDP.Millions2  ~     Sp.Richness2 28.584  0.324   0.324
## 179            GDP.Millions2  ~ Mean.Raup.Crick2 12.399 -0.099  -0.099
## 189         Nat_Pop._WB20152  ~   Total.Animals2  3.131 -0.073  -0.073
## 190         Nat_Pop._WB20152  ~     Sp.Richness2 29.602 -0.319  -0.319
## 191         Nat_Pop._WB20152  ~ Mean.Raup.Crick2 17.217  0.111   0.111
## 200             Prop.Mam.Sp2  ~      Attendance2 11.805  0.140   0.140
## 201             Prop.Mam.Sp2  ~   Total.Animals2 20.277  0.181   0.181
##     sepc.all sepc.nox
```

```
## 75    -0.135   -0.135
## 76    -0.055   -0.055
## 78     0.181    0.181
## 79    -0.193   -0.193
## 82     0.150    0.150
## 83     0.092    0.092
## 84     0.131    0.131
## 85     0.063    0.063
## 87     0.072    0.072
## 88    -0.046   -0.046
## 89     0.067    0.067
## 96     0.230    0.230
## 97     0.221    0.221
## 98     0.276    0.277
## 99     0.155    0.156
## 100    0.116    0.116
## 101    0.105    0.106
## 103    0.110    0.110
## 104    0.792    0.792
## 105    0.434    0.434
## 106    0.463    0.463
## 107    0.106    0.106
## 117   -0.190   -0.190
## 118   -0.833   -0.833
## 128   -0.143   -0.143
## 129   -0.138   -0.138
## 140   -0.507   -0.507
## 141   -0.230   -0.230
## 142   -0.213   -0.213
## 143   -0.425   -0.425
## 152    0.492    0.492
## 153    0.261    0.261
## 155    0.063    0.063
## 177    0.101    0.101
## 178    0.324    0.324
## 179   -0.100   -0.100
## 189   -0.073   -0.073
## 190   -0.319   -0.319
## 191    0.112    0.112
## 200    0.136    0.136
## 201    0.179    0.179
```

```
# Adjust for the nested nature of the data (institutions within countries)
# Fit model and generate model summary
design <- svydesign(ids = ~Country, nest=TRUE, data=sem_attendance_data)
fit.adj10A <- lavaan.survey(lavaan.fit = mod.10A.fit, survey.design = design)
summary(fit.adj10A, rsq = TRUE)
```

```
## lavaan (0.5-23.1097) converged normally after  65 iterations
##
##   Number of observations                           458
##
##   Estimator                                         ML      Robust
##   Minimum Function Test Statistic              185.403      43.649
##   Degrees of freedom                                21          21
```

```
##   P-value (Chi-square)                           0.000       0.003
##   Scaling correction factor                                 4.248
##     for the Satorra-Bentler correction
##
## Parameter Estimates:
##
##   Information                                  Expected
##   Standard Errors                            Robust.sem
##
## Regressions:
##                      Estimate  Std.Err  z-value  P(>|z|)
##   Attendance2 ~
##     Zoo.Area.ha2        0.082    0.043    1.920    0.055
##     Sp.Richness2       -0.242    0.074   -3.259    0.001
##     Total.Animals2      0.529    0.064    8.316    0.000
##     Mam.Sp.Rchnss2      0.146    0.045    3.245    0.001
##     Prop.Mam.Abdn2     -0.098    0.032   -3.058    0.002
##     Mn.Sp.BdyMsXA2      0.329    0.025   13.068    0.000
##     Mean.Rap.Crck2      0.117    0.034    3.470    0.001
##     X50km_Pop2          0.078    0.031    2.528    0.011
##     X10km_Pop2          0.414    0.042    9.851    0.000
##     GDP.Millions2       0.260    0.058    4.465    0.000
##     Nt_Pp._WB20152     -0.129    0.063   -2.031    0.042
##   Total.Animals2 ~
##     Zoo.Area.ha2        0.309    0.040    7.761    0.000
##     Sp.Richness2        0.759    0.047   16.029    0.000
##     Mn.Sp.BdyMsXA2     -0.157    0.034   -4.634    0.000
##     GDP.Millions2      -0.136    0.069   -1.983    0.047
##   Sp.Richness2 ~
##     Prop.Mam.Sp2       -0.563    0.041  -13.858    0.000
##     Mam.Sp.Rchnss2      0.779    0.057   13.593    0.000
##   Mean.Raup.Crick2 ~
##     Sp.Richness2       -0.467    0.180   -2.591    0.010
##     Total.Animals2      0.478    0.111    4.298    0.000
##     Mn.Sp.BdyMsXA2     -0.246    0.082   -3.001    0.003
##     Prop.Mam.Sp2       -0.366    0.077   -4.757    0.000
##
## Covariances:
##                              Estimate  Std.Err  z-value  P(>|z|)
##   Zoo.Area.ha2 ~~
##     Mam.Sp.Rchnss2              0.381    0.064    5.985    0.000
##     Prop.Mam.Abdn2              0.352    0.092    3.822    0.000
##     Mn.Sp.BdyMsXA2              0.522    0.101    5.177    0.000
##     X50km_Pop2                  0.060    0.069    0.880    0.379
##     X10km_Pop2                 -0.010    0.078   -0.124    0.901
##     GDP.Millions2              -0.027    0.041   -0.673    0.501
##     Nt_Pp._WB20152              0.061    0.090    0.682    0.495
##     Prop.Mam.Sp2                0.312    0.089    3.510    0.000
##   Mam.Sp.Richness2 ~~
##     Prop.Mam.Abdn2              0.058    0.049    1.172    0.241
##     Mn.Sp.BdyMsXA2              0.227    0.078    2.921    0.003
##     X50km_Pop2                  0.204    0.082    2.487    0.013
##     X10km_Pop2                  0.284    0.066    4.278    0.000
##     GDP.Millions2              -0.060    0.064   -0.937    0.349
```

```
##     Nt_Pp._WB20152            -0.088    0.102   -0.863    0.388
##     Prop.Mam.Sp2               0.088    0.061    1.435    0.151
##   Prop.Mam.Abdun2 ~~
##     Mn.Sp.BdyMsXA2             0.592    0.109    5.445    0.000
##     X50km_Pop2                -0.123    0.054   -2.263    0.024
##     X10km_Pop2                -0.248    0.060   -4.154    0.000
##     GDP.Millions2             -0.115    0.101   -1.140    0.254
##     Nt_Pp._WB20152            -0.111    0.088   -1.264    0.206
##     Prop.Mam.Sp2               0.878    0.128    6.865    0.000
##   Mean.Sp.BodyMassXAbund2 ~~
##     X50km_Pop2                 0.013    0.074    0.174    0.862
##     X10km_Pop2                 0.021    0.064    0.329    0.742
##     GDP.Millions2             -0.079    0.038   -2.055    0.040
##     Nt_Pp._WB20152            -0.004    0.084   -0.044    0.965
##     Prop.Mam.Sp2               0.628    0.126    5.002    0.000
##   X50km_Pop2 ~~
##     X10km_Pop2                 0.752    0.126    5.982    0.000
##     GDP.Millions2             -0.013    0.100   -0.131    0.896
##     Nt_Pp._WB20152             0.191    0.177    1.083    0.279
##     Prop.Mam.Sp2              -0.096    0.051   -1.888    0.059
##   X10km_Pop2 ~~
##     GDP.Millions2             -0.028    0.056   -0.505    0.613
##     Nt_Pp._WB20152             0.173    0.101    1.701    0.089
##     Prop.Mam.Sp2              -0.204    0.055   -3.685    0.000
##   GDP.Millions2 ~~
##     Nt_Pp._WB20152             0.826    0.202    4.089    0.000
##     Prop.Mam.Sp2              -0.123    0.105   -1.172    0.241
##   Nat_Pop._WB20152 ~~
##     Prop.Mam.Sp2              -0.115    0.091   -1.257    0.209
##
## Intercepts:
##                    Estimate  Std.Err  z-value  P(>|z|)
##    .Attendance2      -0.000    0.029   -0.000    1.000
##    .Total.Animals2   -0.000    0.067   -0.000    1.000
##    .Sp.Richness2      0.000    0.032    0.000    1.000
##    .Mean.Rap.Crck2    0.000    0.076    0.000    1.000
##     Zoo.Area.ha2      0.000    0.064    0.000    1.000
##     Mam.Sp.Rchnss2   -0.000    0.079   -0.000    1.000
##     Prop.Mam.Abdn2    0.000    0.113    0.000    1.000
##     Mn.Sp.BdyMsXA2    0.000    0.071    0.000    1.000
##     X50km_Pop2       -0.000    0.114   -0.000    1.000
##     X10km_Pop2        0.000    0.105    0.000    1.000
##     GDP.Millions2    -0.000    0.345   -0.000    1.000
##     Nt_Pp._WB20152    0.000    0.295    0.000    1.000
##     Prop.Mam.Sp2      0.000    0.118    0.000    1.000
##
## Variances:
##                    Estimate  Std.Err  z-value  P(>|z|)
##    .Attendance2       0.271    0.028    9.724    0.000
##    .Total.Animals2    0.219    0.037    5.986    0.000
##    .Sp.Richness2      0.154    0.044    3.506    0.000
##    .Mean.Rap.Crck2    0.684    0.059   11.527    0.000
##     Zoo.Area.ha2      0.998    0.112    8.886    0.000
##     Mam.Sp.Rchnss2    0.998    0.115    8.669    0.000
```

```
##     Prop.Mam.Abdn2    0.998    0.121    8.221    0.000
##     Mn.Sp.BdyMsXA2    0.998    0.160    6.219    0.000
##     X50km_Pop2        0.998    0.131    7.623    0.000
##     X10km_Pop2        0.998    0.174    5.745    0.000
##     GDP.Millions2     0.998    0.246    4.061    0.000
##     Nt_Pp._WB20152    0.998    0.225    4.439    0.000
##     Prop.Mam.Sp2      0.998    0.150    6.662    0.000
##
## R-Square:
##                    Estimate
##     Attendance2       0.710
##     Total.Animals2    0.775
##     Sp.Richness2      0.845
##     Mean.Rap.Crck2    0.323
```

```
# Generate fit indices
fitMeasures(fit.adj10A, c("agfi", "rmr", "srmr", "rmsea", "cfi", "nnfi", "tli"))
```

```
##  agfi   rmr  srmr rmsea   cfi  nnfi   tli
## 0.733 0.040 0.037 0.131 0.933 0.867 0.867
```

```
# Generate modification indices
mi10adjA <- modindices(fit.adj10A)
print(mi10adjA[mi10adjA$mi > 3.0,])
```

```
##                          lhs op              rhs     mi mi.scaled    epc
## 88            Total.Animals2 ~~ Mean.Raup.Crick2 14.603     3.438 -0.134
## 89              Sp.Richness2 ~~ Mean.Raup.Crick2 10.304     2.426 -0.055
## 91            Total.Animals2  ~      Attendance2 14.492     3.412  0.185
## 92            Total.Animals2  ~ Mean.Raup.Crick2 18.135     4.269 -0.189
## 95            Total.Animals2  ~       X50km_Pop2 43.331    10.201  0.148
## 96            Total.Animals2  ~       X10km_Pop2 14.994     3.530  0.091
## 97            Total.Animals2  ~ Nat_Pop._WB20152 10.679     2.514  0.129
## 98            Total.Animals2  ~     Prop.Mam.Sp2  3.589     0.845  0.062
## 100             Sp.Richness2  ~   Total.Animals2  4.049     0.953  0.072
## 101             Sp.Richness2  ~ Mean.Raup.Crick2  3.898     0.918 -0.046
## 102             Sp.Richness2  ~     Zoo.Area.ha2 10.169     2.394  0.067
## 109         Mean.Raup.Crick2  ~      Attendance2  8.499     2.001  0.240
## 110         Mean.Raup.Crick2  ~     Zoo.Area.ha2 17.464     4.111  0.222
## 111         Mean.Raup.Crick2  ~ Mam.Sp.Richness2 10.304     2.426  0.278
## 112         Mean.Raup.Crick2  ~  Prop.Mam.Abdun2  3.621     0.852  0.156
## 113         Mean.Raup.Crick2  ~       X50km_Pop2  8.651     2.037  0.117
## 114         Mean.Raup.Crick2  ~       X10km_Pop2  6.491     1.528  0.106
## 116         Mean.Raup.Crick2  ~ Nat_Pop._WB20152  7.752     1.825  0.111
## 117             Zoo.Area.ha2  ~      Attendance2 12.756     3.003  0.818
## 118             Zoo.Area.ha2  ~   Total.Animals2 17.595     4.142  0.440
## 119             Zoo.Area.ha2  ~     Sp.Richness2 25.670     6.043  0.463
## 120             Zoo.Area.ha2  ~ Mean.Raup.Crick2  4.898     1.153  0.106
## 130         Mam.Sp.Richness2  ~   Total.Animals2  3.415     0.804 -0.192
## 131         Mam.Sp.Richness2  ~     Sp.Richness2 20.237     4.764 -0.833
## 141          Prop.Mam.Abdun2  ~      Attendance2  9.637     2.269 -0.148
## 142          Prop.Mam.Abdun2  ~   Total.Animals2 13.050     3.072 -0.140
## 153 Mean.Sp.BodyMassXAbund2  ~      Attendance2 12.839     3.023 -0.524
## 154 Mean.Sp.BodyMassXAbund2  ~   Total.Animals2  8.595     2.023 -0.233
## 155 Mean.Sp.BodyMassXAbund2  ~     Sp.Richness2  7.084     1.668 -0.213
## 156 Mean.Sp.BodyMassXAbund2  ~ Mean.Raup.Crick2 25.521     6.008 -0.422
```

```
## 165             X50km_Pop2  ~      Attendance2 25.720     6.055  0.509
## 166             X50km_Pop2  ~   Total.Animals2 24.167     5.690  0.264
## 168             X50km_Pop2  ~ Mean.Raup.Crick2  3.252     0.766  0.063
## 190           GDP.Millions2  ~   Total.Animals2  4.637     1.092  0.103
## 191           GDP.Millions2  ~     Sp.Richness2 28.584     6.729  0.324
## 192           GDP.Millions2  ~ Mean.Raup.Crick2 12.399     2.919 -0.099
## 202        Nat_Pop._WB20152  ~   Total.Animals2  3.131     0.737 -0.073
## 203        Nat_Pop._WB20152  ~     Sp.Richness2 29.602     6.969 -0.319
## 204        Nat_Pop._WB20152  ~ Mean.Raup.Crick2 17.217     4.053  0.111
## 213            Prop.Mam.Sp2  ~      Attendance2 11.805     2.779  0.140
## 214            Prop.Mam.Sp2  ~   Total.Animals2 20.276     4.774  0.181
##     sepc.lv sepc.all sepc.nox
## 88   -0.134   -0.135   -0.135
## 89   -0.055   -0.055   -0.055
## 91    0.185    0.181    0.181
## 92   -0.189   -0.193   -0.193
## 95    0.148    0.150    0.150
## 96    0.091    0.092    0.092
## 97    0.129    0.131    0.131
## 98    0.062    0.063    0.063
## 100   0.072    0.072    0.072
## 101  -0.046   -0.046   -0.046
## 102   0.067    0.067    0.067
## 109   0.240    0.230    0.230
## 110   0.222    0.221    0.221
## 111   0.278    0.276    0.277
## 112   0.156    0.155    0.156
## 113   0.117    0.116    0.116
## 114   0.106    0.105    0.106
## 116   0.111    0.110    0.110
## 117   0.818    0.792    0.792
## 118   0.440    0.434    0.434
## 119   0.463    0.463    0.463
## 120   0.106    0.106    0.106
## 130  -0.192   -0.190   -0.190
## 131  -0.833   -0.833   -0.833
## 141  -0.148   -0.143   -0.143
## 142  -0.140   -0.138   -0.138
## 153  -0.524   -0.507   -0.507
## 154  -0.233   -0.230   -0.230
## 155  -0.213   -0.213   -0.213
## 156  -0.422   -0.425   -0.425
## 165   0.509    0.492    0.492
## 166   0.264    0.261    0.261
## 168   0.063    0.063    0.063
## 190   0.103    0.101    0.101
## 191   0.324    0.324    0.324
## 192  -0.099   -0.100   -0.100
## 202  -0.073   -0.073   -0.073
## 203  -0.319   -0.319   -0.319
## 204   0.111    0.112    0.112
## 213   0.140    0.136    0.136
## 214   0.181    0.179    0.179
```

Model 11

Based on the results generated from the nested tenth model, the highest p-value relationship to be considered for removal is **Attendance2 ~ Zoo.Area.ha2** with a p-value of **0.055**. Therefore we decide to remove this pathway. Once again, the model summary, fit indices and modification indices were all generated for the model, adjusting for the nested nature of data.

```
# Attendance SEM (Species Abundance)

# Model 11
# Removal of Attendance2 ~ Zoo.Area.ha2, p = 0.055

mod.11A <- 'Attendance2 ~ Sp.Richness2 + Total.Animals2
+ Mam.Sp.Richness2 + Prop.Mam.Abdun2
+ Mean.Sp.BodyMassXAbund2 + Mean.Raup.Crick2
+ X50km_Pop2 + X10km_Pop2 + GDP.Millions2 + Nat_Pop._WB20152

Total.Animals2 ~ Zoo.Area.ha2 + Sp.Richness2 + Mean.Sp.BodyMassXAbund2 + GDP.Millions2
Sp.Richness2 ~ Prop.Mam.Sp2 + Mam.Sp.Richness2
Mean.Raup.Crick2 ~ Sp.Richness2 + Total.Animals2 + Mean.Sp.BodyMassXAbund2 + Prop.Mam.Sp2'

# Fit model and generate model summary
mod.11A.fit <- sem(mod.11A, data = sem_attendance_data, fixed.x=FALSE)
summary(mod.11A.fit, rsq = TRUE)
```

```
## lavaan (0.5-23.1097) converged normally after  56 iterations
##
##   Number of observations                           458
##
##   Estimator                                         ML
##   Minimum Function Test Statistic              190.188
##   Degrees of freedom                                22
##   P-value (Chi-square)                           0.000
##
## Parameter Estimates:
##
##   Information                                 Expected
##   Standard Errors                             Standard
##
## Regressions:
##                    Estimate  Std.Err  z-value  P(>|z|)
##   Attendance2 ~
##     Sp.Richness2     -0.245    0.065   -3.799    0.000
##     Total.Animals2    0.581    0.049   11.935    0.000
##     Mam.Sp.Rchnss2    0.141    0.049    2.878    0.004
##     Prop.Mam.Abdn2   -0.077    0.040   -1.908    0.056
##     Mn.Sp.BdyMsXA2    0.372    0.034   11.067    0.000
##     Mean.Rap.Crck2    0.130    0.029    4.477    0.000
##     X50km_Pop2        0.074    0.038    1.929    0.054
##     X10km_Pop2        0.401    0.041    9.857    0.000
##     GDP.Millions2     0.253    0.048    5.238    0.000
##     Nt_Pp._WB20152   -0.111    0.049   -2.275    0.023
##   Total.Animals2 ~
##     Zoo.Area.ha2      0.309    0.027   11.617    0.000
##     Sp.Richness2      0.759    0.023   32.975    0.000
```

```
##     Mn.Sp.BdyMsXA2     -0.157    0.027   -5.852    0.000
##     GDP.Millions2      -0.136    0.022   -6.202    0.000
##   Sp.Richness2 ~
##     Prop.Mam.Sp2       -0.563    0.018  -30.494    0.000
##     Mam.Sp.Rchnss2      0.779    0.018   42.208    0.000
##   Mean.Raup.Crick2 ~
##     Sp.Richness2       -0.467    0.075   -6.198    0.000
##     Total.Animals2      0.478    0.071    6.762    0.000
##     Mn.Sp.BdyMsXA2     -0.246    0.051   -4.837    0.000
##     Prop.Mam.Sp2       -0.366    0.058   -6.343    0.000
##
## Covariances:
##                          Estimate  Std.Err  z-value  P(>|z|)
##   Mam.Sp.Richness2 ~~
##     Prop.Mam.Abdn2          0.058    0.047    1.237    0.216
##     Mn.Sp.BdyMsXA2          0.227    0.048    4.750    0.000
##     X50km_Pop2              0.204    0.048    4.281    0.000
##     X10km_Pop2              0.284    0.048    5.852    0.000
##     GDP.Millions2          -0.060    0.047   -1.277    0.202
##     Nt_Pp._WB20152         -0.088    0.047   -1.881    0.060
##     Zoo.Area.ha2            0.381    0.050    7.634    0.000
##     Prop.Mam.Sp2            0.088    0.047    1.882    0.060
##   Prop.Mam.Abdun2 ~~
##     Mn.Sp.BdyMsXA2          0.592    0.054   10.922    0.000
##     X50km_Pop2             -0.123    0.047   -2.622    0.009
##     X10km_Pop2             -0.248    0.048   -5.165    0.000
##     GDP.Millions2          -0.115    0.047   -2.458    0.014
##     Nt_Pp._WB20152         -0.111    0.047   -2.362    0.018
##     Zoo.Area.ha2            0.352    0.049    7.121    0.000
##     Prop.Mam.Sp2            0.878    0.062   14.135    0.000
##   Mean.Sp.BodyMassXAbund2 ~~
##     X50km_Pop2              0.013    0.047    0.275    0.784
##     X10km_Pop2              0.021    0.047    0.455    0.649
##     GDP.Millions2          -0.079    0.047   -1.682    0.093
##     Nt_Pp._WB20152         -0.004    0.047   -0.079    0.937
##     Zoo.Area.ha2            0.522    0.053    9.923    0.000
##     Prop.Mam.Sp2            0.628    0.055   11.405    0.000
##   X50km_Pop2 ~~
##     X10km_Pop2              0.752    0.058   12.886    0.000
##     GDP.Millions2          -0.013    0.047   -0.281    0.779
##     Nt_Pp._WB20152          0.191    0.047    4.033    0.000
##     Zoo.Area.ha2            0.060    0.047    1.291    0.197
##     Prop.Mam.Sp2           -0.096    0.047   -2.054    0.040
##   X10km_Pop2 ~~
##     GDP.Millions2          -0.028    0.047   -0.604    0.546
##     Nt_Pp._WB20152          0.173    0.047    3.646    0.000
##     Zoo.Area.ha2           -0.010    0.047   -0.208    0.835
##     Prop.Mam.Sp2           -0.204    0.048   -4.293    0.000
##   GDP.Millions2 ~~
##     Nt_Pp._WB20152          0.826    0.061   13.642    0.000
##     Zoo.Area.ha2           -0.027    0.047   -0.588    0.557
##     Prop.Mam.Sp2           -0.123    0.047   -2.615    0.009
##   Nat_Pop._WB20152 ~~
##     Zoo.Area.ha2            0.061    0.047    1.310    0.190
```

```
##     Prop.Mam.Sp2               -0.115    0.047   -2.447    0.014
##   Zoo.Area.ha2 ~~
##     Prop.Mam.Sp2                0.312    0.049    6.389    0.000
##
## Variances:
##                    Estimate  Std.Err  z-value  P(>|z|)
##    .Attendance2       0.274    0.018   15.133    0.000
##    .Total.Animals2    0.219    0.014   15.133    0.000
##    .Sp.Richness2      0.154    0.010   15.133    0.000
##    .Mean.Rap.Crck2    0.684    0.045   15.133    0.000
##     Mam.Sp.Rchnss2    0.998    0.066   15.133    0.000
##     Prop.Mam.Abdn2    0.998    0.066   15.133    0.000
##     Mn.Sp.BdyMsXA2    0.998    0.066   15.133    0.000
##     X50km_Pop2        0.998    0.066   15.133    0.000
##     X10km_Pop2        0.998    0.066   15.133    0.000
##     GDP.Millions2     0.998    0.066   15.133    0.000
##     Nt_Pp._WB20152    0.998    0.066   15.133    0.000
##     Zoo.Area.ha2      0.998    0.066   15.133    0.000
##     Prop.Mam.Sp2      0.998    0.066   15.133    0.000
##
## R-Square:
##                    Estimate
##     Attendance2       0.707
##     Total.Animals2    0.775
##     Sp.Richness2      0.845
##     Mean.Rap.Crck2    0.323
```

```
# Generate fit indices
fitMeasures(mod.11A.fit, c("agfi", "rmr", "srmr", "rmsea", "cfi", "nnfi", "tli"))
```

```
##  agfi   rmr  srmr rmsea   cfi  nnfi   tli
## 0.771 0.040 0.040 0.129 0.932 0.870 0.870
```

```
# Generate modification indices
mi11A <- modindices(mod.11A.fit)
print(mi11A[mi11A$mi > 3.0,])
```

```
##                          lhs op             rhs     mi    epc  sepc.lv
## 70              Attendance2 ~~  Total.Animals2  4.234 -0.052   -0.052
## 74           Total.Animals2 ~~ Mean.Raup.Crick2 14.603 -0.134   -0.134
## 75             Sp.Richness2 ~~ Mean.Raup.Crick2 10.304 -0.055   -0.055
## 76              Attendance2  ~     Zoo.Area.ha2  4.234  0.073    0.073
## 78           Total.Animals2  ~      Attendance2  5.195  0.101    0.101
## 79           Total.Animals2  ~ Mean.Raup.Crick2 18.135 -0.189   -0.189
## 82           Total.Animals2  ~       X50km_Pop2 43.331  0.148    0.148
## 83           Total.Animals2  ~       X10km_Pop2 14.994  0.091    0.091
## 84           Total.Animals2  ~ Nat_Pop._WB20152 10.679  0.129    0.129
## 85           Total.Animals2  ~     Prop.Mam.Sp2  3.589  0.062    0.062
## 87             Sp.Richness2  ~   Total.Animals2  4.049  0.072    0.072
## 88             Sp.Richness2  ~ Mean.Raup.Crick2  3.898 -0.046   -0.046
## 95             Sp.Richness2  ~     Zoo.Area.ha2 10.169  0.067    0.067
## 96         Mean.Raup.Crick2  ~      Attendance2  6.141  0.209    0.209
## 97         Mean.Raup.Crick2  ~ Mam.Sp.Richness2 10.304  0.278    0.278
## 98         Mean.Raup.Crick2  ~  Prop.Mam.Abdun2  3.621  0.156    0.156
## 99         Mean.Raup.Crick2  ~       X50km_Pop2  8.651  0.117    0.117
## 100        Mean.Raup.Crick2  ~       X10km_Pop2  6.491  0.106    0.106
```

```
## 102        Mean.Raup.Crick2  ~ Nat_Pop._WB20152  7.752  0.111   0.111
## 103        Mean.Raup.Crick2  ~     Zoo.Area.ha2 17.464  0.222   0.222
## 105        Mam.Sp.Richness2  ~   Total.Animals2  3.415 -0.192  -0.192
## 106        Mam.Sp.Richness2  ~     Sp.Richness2 20.237 -0.833  -0.833
## 116         Prop.Mam.Abdun2  ~      Attendance2 11.573 -0.154  -0.154
## 117         Prop.Mam.Abdun2  ~   Total.Animals2 13.050 -0.140  -0.140
## 128 Mean.Sp.BodyMassXAbund2  ~      Attendance2 18.237 -0.471  -0.471
## 129 Mean.Sp.BodyMassXAbund2  ~   Total.Animals2  8.595 -0.233  -0.233
## 130 Mean.Sp.BodyMassXAbund2  ~     Sp.Richness2  7.084 -0.213  -0.213
## 131 Mean.Sp.BodyMassXAbund2  ~ Mean.Raup.Crick2 25.520 -0.422  -0.422
## 140              X50km_Pop2  ~      Attendance2 22.322  0.424   0.424
## 141              X50km_Pop2  ~   Total.Animals2 24.167  0.264   0.264
## 143              X50km_Pop2  ~ Mean.Raup.Crick2  3.252  0.063   0.063
## 165           GDP.Millions2  ~   Total.Animals2  4.637  0.103   0.103
## 166           GDP.Millions2  ~     Sp.Richness2 28.584  0.324   0.324
## 167           GDP.Millions2  ~ Mean.Raup.Crick2 12.399 -0.099  -0.099
## 177        Nat_Pop._WB20152  ~   Total.Animals2  3.131 -0.073  -0.073
## 178        Nat_Pop._WB20152  ~     Sp.Richness2 29.602 -0.319  -0.319
## 179        Nat_Pop._WB20152  ~ Mean.Raup.Crick2 17.217  0.111   0.111
## 188            Zoo.Area.ha2  ~      Attendance2 10.635  0.237   0.237
## 189            Zoo.Area.ha2  ~   Total.Animals2 17.595  0.440   0.440
## 190            Zoo.Area.ha2  ~     Sp.Richness2 25.670  0.463   0.463
## 191            Zoo.Area.ha2  ~ Mean.Raup.Crick2  4.898  0.106   0.106
## 200            Prop.Mam.Sp2  ~      Attendance2 14.103  0.148   0.148
## 201            Prop.Mam.Sp2  ~   Total.Animals2 20.276  0.181   0.181
##     sepc.all sepc.nox
## 70    -0.054   -0.054
## 74    -0.135   -0.135
## 75    -0.055   -0.055
## 76     0.076    0.076
## 78     0.098    0.098
## 79    -0.193   -0.193
## 82     0.150    0.150
## 83     0.092    0.092
## 84     0.131    0.131
## 85     0.063    0.063
## 87     0.072    0.072
## 88    -0.046   -0.046
## 95     0.067    0.067
## 96     0.201    0.201
## 97     0.276    0.277
## 98     0.155    0.156
## 99     0.116    0.116
## 100    0.105    0.106
## 102    0.110    0.110
## 103    0.221    0.221
## 105   -0.190   -0.190
## 106   -0.833   -0.833
## 116   -0.149   -0.149
## 117   -0.138   -0.138
## 128   -0.456   -0.456
## 129   -0.230   -0.230
## 130   -0.213   -0.213
## 131   -0.425   -0.425
```

```
## 140    0.410    0.410
## 141    0.261    0.261
## 143    0.063    0.063
## 165    0.101    0.101
## 166    0.324    0.324
## 167   -0.100   -0.100
## 177   -0.073   -0.073
## 178   -0.319   -0.319
## 179    0.112    0.112
## 188    0.229    0.229
## 189    0.434    0.434
## 190    0.463    0.463
## 191    0.106    0.106
## 200    0.143    0.143
## 201    0.179    0.179
```

```
# Adjust for the nested nature of the data (institutions within countries)
# Fit model and generate model summary
design <- svydesign(ids = ~Country, nest=TRUE, data=sem_attendance_data)
fit.adj11A <- lavaan.survey(lavaan.fit = mod.11A.fit, survey.design = design)
summary(fit.adj11A, rsq = TRUE)
```

```
## lavaan (0.5-23.1097) converged normally after  63 iterations
##
##   Number of observations                           458
##
##   Estimator                                         ML      Robust
##   Minimum Function Test Statistic              190.188      46.262
##   Degrees of freedom                                22          22
##   P-value (Chi-square)                           0.000       0.002
##   Scaling correction factor                                  4.111
##     for the Satorra-Bentler correction
##
## Parameter Estimates:
##
##   Information                                 Expected
##   Standard Errors                           Robust.sem
##
## Regressions:
##                      Estimate  Std.Err  z-value  P(>|z|)
##   Attendance2 ~
##     Sp.Richness2       -0.245    0.065   -3.760    0.000
##     Total.Animals2      0.581    0.051   11.493    0.000
##     Mam.Sp.Rchnss2      0.141    0.042    3.371    0.001
##     Prop.Mam.Abdn2     -0.077    0.030   -2.526    0.012
##     Mn.Sp.BdyMsXA2      0.372    0.029   12.969    0.000
##     Mean.Rap.Crck2      0.130    0.034    3.834    0.000
##     X50km_Pop2          0.074    0.032    2.283    0.022
##     X10km_Pop2          0.401    0.043    9.339    0.000
##     GDP.Millions2       0.253    0.058    4.349    0.000
##     Nt_Pp._WB20152     -0.111    0.063   -1.767    0.077
##   Total.Animals2 ~
##     Zoo.Area.ha2        0.309    0.040    7.761    0.000
##     Sp.Richness2        0.759    0.047   16.029    0.000
##     Mn.Sp.BdyMsXA2     -0.157    0.034   -4.634    0.000
```

```
##     GDP.Millions2      -0.136    0.069   -1.983    0.047
##   Sp.Richness2 ~
##     Prop.Mam.Sp2       -0.563    0.041  -13.858    0.000
##     Mam.Sp.Rchnss2      0.779    0.057   13.593    0.000
##   Mean.Raup.Crick2 ~
##     Sp.Richness2       -0.467    0.180   -2.591    0.010
##     Total.Animals2      0.478    0.111    4.298    0.000
##     Mn.Sp.BdyMsXA2     -0.246    0.082   -3.001    0.003
##     Prop.Mam.Sp2       -0.366    0.077   -4.757    0.000
##
## Covariances:
##                           Estimate  Std.Err  z-value  P(>|z|)
##   Mam.Sp.Richness2 ~~
##     Prop.Mam.Abdn2           0.058    0.049    1.172    0.241
##     Mn.Sp.BdyMsXA2           0.227    0.078    2.921    0.003
##     X50km_Pop2               0.204    0.082    2.487    0.013
##     X10km_Pop2               0.284    0.066    4.278    0.000
##     GDP.Millions2           -0.060    0.064   -0.937    0.349
##     Nt_Pp._WB20152          -0.088    0.102   -0.863    0.388
##     Zoo.Area.ha2             0.381    0.064    5.985    0.000
##     Prop.Mam.Sp2             0.088    0.061    1.435    0.151
##   Prop.Mam.Abdun2 ~~
##     Mn.Sp.BdyMsXA2           0.592    0.109    5.445    0.000
##     X50km_Pop2              -0.123    0.054   -2.263    0.024
##     X10km_Pop2              -0.248    0.060   -4.154    0.000
##     GDP.Millions2           -0.115    0.101   -1.140    0.254
##     Nt_Pp._WB20152          -0.111    0.088   -1.264    0.206
##     Zoo.Area.ha2             0.352    0.092    3.822    0.000
##     Prop.Mam.Sp2             0.878    0.128    6.865    0.000
##   Mean.Sp.BodyMassXAbund2 ~~
##     X50km_Pop2               0.013    0.074    0.174    0.862
##     X10km_Pop2               0.021    0.064    0.329    0.742
##     GDP.Millions2           -0.079    0.038   -2.055    0.040
##     Nt_Pp._WB20152          -0.004    0.084   -0.044    0.965
##     Zoo.Area.ha2             0.522    0.101    5.177    0.000
##     Prop.Mam.Sp2             0.628    0.126    5.002    0.000
##   X50km_Pop2 ~~
##     X10km_Pop2               0.752    0.126    5.982    0.000
##     GDP.Millions2           -0.013    0.100   -0.131    0.896
##     Nt_Pp._WB20152           0.191    0.177    1.083    0.279
##     Zoo.Area.ha2             0.060    0.069    0.880    0.379
##     Prop.Mam.Sp2            -0.096    0.051   -1.888    0.059
##   X10km_Pop2 ~~
##     GDP.Millions2           -0.028    0.056   -0.505    0.613
##     Nt_Pp._WB20152           0.173    0.101    1.701    0.089
##     Zoo.Area.ha2            -0.010    0.078   -0.124    0.901
##     Prop.Mam.Sp2            -0.204    0.055   -3.685    0.000
##   GDP.Millions2 ~~
##     Nt_Pp._WB20152           0.826    0.202    4.089    0.000
##     Zoo.Area.ha2            -0.027    0.041   -0.673    0.501
##     Prop.Mam.Sp2            -0.123    0.105   -1.172    0.241
##   Nat_Pop._WB20152 ~~
##     Zoo.Area.ha2             0.061    0.090    0.682    0.495
##     Prop.Mam.Sp2            -0.115    0.091   -1.257    0.209
```

```
##   Zoo.Area.ha2 ~~
##     Prop.Mam.Sp2              0.312    0.089    3.510    0.000
##
## Intercepts:
##                    Estimate  Std.Err  z-value  P(>|z|)
##    .Attendance2      -0.000    0.030   -0.000    1.000
##    .Total.Animals2   -0.000    0.067   -0.000    1.000
##    .Sp.Richness2      0.000    0.032    0.000    1.000
##    .Mean.Rap.Crck2    0.000    0.076    0.000    1.000
##     Mam.Sp.Rchnss2   -0.000    0.079   -0.000    1.000
##     Prop.Mam.Abdn2    0.000    0.113    0.000    1.000
##     Mn.Sp.BdyMsXA2    0.000    0.071    0.000    1.000
##     X50km_Pop2       -0.000    0.114   -0.000    1.000
##     X10km_Pop2        0.000    0.105    0.000    1.000
##     GDP.Millions2    -0.000    0.345   -0.000    1.000
##     Nt_Pp._WB20152    0.000    0.295    0.000    1.000
##     Zoo.Area.ha2      0.000    0.064    0.000    1.000
##     Prop.Mam.Sp2      0.000    0.118    0.000    1.000
##
## Variances:
##                    Estimate  Std.Err  z-value  P(>|z|)
##    .Attendance2       0.274    0.027   10.234    0.000
##    .Total.Animals2    0.219    0.037    5.986    0.000
##    .Sp.Richness2      0.154    0.044    3.506    0.000
##    .Mean.Rap.Crck2    0.684    0.059   11.527    0.000
##     Mam.Sp.Rchnss2    0.998    0.115    8.669    0.000
##     Prop.Mam.Abdn2    0.998    0.121    8.221    0.000
##     Mn.Sp.BdyMsXA2    0.998    0.160    6.219    0.000
##     X50km_Pop2        0.998    0.131    7.623    0.000
##     X10km_Pop2        0.998    0.174    5.745    0.000
##     GDP.Millions2     0.998    0.246    4.061    0.000
##     Nt_Pp._WB20152    0.998    0.225    4.439    0.000
##     Zoo.Area.ha2      0.998    0.112    8.886    0.000
##     Prop.Mam.Sp2      0.998    0.150    6.662    0.000
##
## R-Square:
##                    Estimate
##     Attendance2       0.707
##     Total.Animals2    0.775
##     Sp.Richness2      0.845
##     Mean.Rap.Crck2    0.323
```

```
# Generate fit indices
fitMeasures(fit.adj11A, c("agfi", "rmr", "srmr", "rmsea", "cfi", "nnfi", "tli"))
```

```
##  agfi   rmr  srmr rmsea   cfi  nnfi   tli
## 0.738 0.040 0.038 0.129 0.932 0.870 0.870
```

```
# Generate modification indices
mi11adjA <- modindices(fit.adj11A)
print(mi11adjA[mi11adjA$mi > 3.0,])
```

```
##                        lhs op              rhs     mi mi.scaled    epc
## 83             Attendance2 ~~   Total.Animals2  4.233     1.030 -0.052
## 87          Total.Animals2 ~~ Mean.Raup.Crick2 14.603     3.552 -0.134
## 88            Sp.Richness2 ~~ Mean.Raup.Crick2 10.304     2.506 -0.055
```

```
## 89             Attendance2  ~     Zoo.Area.ha2  4.234     1.030  0.073
## 91          Total.Animals2  ~      Attendance2  5.195     1.264  0.101
## 92          Total.Animals2  ~ Mean.Raup.Crick2 18.135     4.411 -0.189
## 95          Total.Animals2  ~       X50km_Pop2 43.331    10.540  0.148
## 96          Total.Animals2  ~       X10km_Pop2 14.994     3.647  0.091
## 97          Total.Animals2  ~ Nat_Pop._WB20152 10.679     2.598  0.129
## 98          Total.Animals2  ~     Prop.Mam.Sp2  3.589     0.873  0.062
## 100           Sp.Richness2  ~   Total.Animals2  4.049     0.985  0.072
## 101           Sp.Richness2  ~ Mean.Raup.Crick2  3.898     0.948 -0.046
## 108           Sp.Richness2  ~     Zoo.Area.ha2 10.169     2.473  0.067
## 109       Mean.Raup.Crick2  ~      Attendance2  6.142     1.494  0.210
## 110       Mean.Raup.Crick2  ~ Mam.Sp.Richness2 10.304     2.506  0.278
## 111       Mean.Raup.Crick2  ~  Prop.Mam.Abdun2  3.621     0.881  0.156
## 112       Mean.Raup.Crick2  ~       X50km_Pop2  8.651     2.104  0.117
## 113       Mean.Raup.Crick2  ~       X10km_Pop2  6.491     1.579  0.106
## 115       Mean.Raup.Crick2  ~ Nat_Pop._WB20152  7.752     1.886  0.111
## 116       Mean.Raup.Crick2  ~     Zoo.Area.ha2 17.464     4.248  0.222
## 118       Mam.Sp.Richness2  ~   Total.Animals2  3.415     0.831 -0.192
## 119       Mam.Sp.Richness2  ~     Sp.Richness2 20.237     4.922 -0.833
## 129        Prop.Mam.Abdun2  ~      Attendance2 11.573     2.815 -0.154
## 130        Prop.Mam.Abdun2  ~   Total.Animals2 13.050     3.174 -0.140
## 141 Mean.Sp.BodyMassXAbund2  ~      Attendance2 18.237     4.436 -0.471
## 142 Mean.Sp.BodyMassXAbund2  ~   Total.Animals2  8.595     2.091 -0.233
## 143 Mean.Sp.BodyMassXAbund2  ~     Sp.Richness2  7.084     1.723 -0.213
## 144 Mean.Sp.BodyMassXAbund2  ~ Mean.Raup.Crick2 25.520     6.208 -0.422
## 153             X50km_Pop2  ~      Attendance2 22.323     5.430  0.424
## 154             X50km_Pop2  ~   Total.Animals2 24.167     5.879  0.264
## 156             X50km_Pop2  ~ Mean.Raup.Crick2  3.252     0.791  0.063
## 178           GDP.Millions2  ~   Total.Animals2  4.637     1.128  0.103
## 179           GDP.Millions2  ~     Sp.Richness2 28.583     6.953  0.324
## 180           GDP.Millions2  ~ Mean.Raup.Crick2 12.399     3.016 -0.099
## 190       Nat_Pop._WB20152  ~   Total.Animals2  3.131     0.761 -0.073
## 191       Nat_Pop._WB20152  ~     Sp.Richness2 29.601     7.200 -0.319
## 192       Nat_Pop._WB20152  ~ Mean.Raup.Crick2 17.217     4.188  0.111
## 201           Zoo.Area.ha2  ~      Attendance2 10.635     2.587  0.237
## 202           Zoo.Area.ha2  ~   Total.Animals2 17.595     4.280  0.440
## 203           Zoo.Area.ha2  ~     Sp.Richness2 25.670     6.244  0.463
## 204           Zoo.Area.ha2  ~ Mean.Raup.Crick2  4.898     1.191  0.106
## 213           Prop.Mam.Sp2  ~      Attendance2 14.103     3.430  0.148
## 214           Prop.Mam.Sp2  ~   Total.Animals2 20.276     4.932  0.181
##     sepc.lv sepc.all sepc.nox
## 83   -0.052   -0.054   -0.054
## 87   -0.134   -0.135   -0.135
## 88   -0.055   -0.055   -0.055
## 89    0.073    0.076    0.076
## 91    0.101    0.098    0.098
## 92   -0.189   -0.193   -0.193
## 95    0.148    0.150    0.150
## 96    0.091    0.092    0.092
## 97    0.129    0.131    0.131
## 98    0.062    0.063    0.063
## 100   0.072    0.072    0.072
## 101  -0.046   -0.046   -0.046
## 108   0.067    0.067    0.067
```

```
## 109   0.210    0.201    0.201
## 110   0.278    0.276    0.277
## 111   0.156    0.155    0.156
## 112   0.117    0.116    0.116
## 113   0.106    0.105    0.106
## 115   0.111    0.110    0.110
## 116   0.222    0.221    0.221
## 118  -0.192   -0.190   -0.190
## 119  -0.833   -0.833   -0.833
## 129  -0.154   -0.149   -0.149
## 130  -0.140   -0.138   -0.138
## 141  -0.471   -0.456   -0.456
## 142  -0.233   -0.230   -0.230
## 143  -0.213   -0.213   -0.213
## 144  -0.422   -0.425   -0.425
## 153   0.424    0.410    0.410
## 154   0.264    0.261    0.261
## 156   0.063    0.063    0.063
## 178   0.103    0.101    0.101
## 179   0.324    0.324    0.324
## 180  -0.099   -0.100   -0.100
## 190  -0.073   -0.073   -0.073
## 191  -0.319   -0.319   -0.319
## 192   0.111    0.112    0.112
## 201   0.237    0.229    0.229
## 202   0.440    0.434    0.434
## 203   0.463    0.463    0.463
## 204   0.106    0.106    0.106
## 213   0.148    0.143    0.143
## 214   0.181    0.179    0.179
```

Model 12

Based on the results generated from the nested eleventh model, the highest p-value relationship to be considered for removal is ___Attendance2 ~ Nt_Pp._WB20152___ with a p-value of **0.077**. Therefore we decide to remove this pathway. Once again, the model summary, fit indices and modification indices were all generated for the model, adjusting for the nested nature of data.

```
# Attendance SEM (Species Abundance)

# Model 12
# Removal of Attendance2 ~ Nt_Pp._WB20152, p = 0.077

mod.12A <- 'Attendance2 ~ Sp.Richness2 + Total.Animals2
+ Mam.Sp.Richness2 + Prop.Mam.Abdun2
+ Mean.Sp.BodyMassXAbund2 + Mean.Raup.Crick2
+ X50km_Pop2 + X10km_Pop2 + GDP.Millions2

Total.Animals2 ~ Zoo.Area.ha2 + Sp.Richness2 + Mean.Sp.BodyMassXAbund2 + GDP.Millions2
Sp.Richness2 ~ Prop.Mam.Sp2 + Mam.Sp.Richness2
Mean.Raup.Crick2 ~ Sp.Richness2 + Total.Animals2 + Mean.Sp.BodyMassXAbund2 + Prop.Mam.Sp2'

# Fit model and generate model summary
```

```r
mod.12A.fit <- sem(mod.12A, data = sem_attendance_data, fixed.x=FALSE)
summary(mod.12A.fit, rsq = TRUE)
```

```
## lavaan (0.5-23.1097) converged normally after  49 iterations
##
##   Number of observations                           458
##
##   Estimator                                         ML
##   Minimum Function Test Statistic              144.618
##   Degrees of freedom                                19
##   P-value (Chi-square)                           0.000
##
## Parameter Estimates:
##
##   Information                                 Expected
##   Standard Errors                             Standard
##
## Regressions:
##                        Estimate  Std.Err  z-value  P(>|z|)
##   Attendance2 ~
##     Sp.Richness2         -0.217    0.065   -3.352    0.001
##     Total.Animals2        0.569    0.049   11.676    0.000
##     Mam.Sp.Rchnss2        0.140    0.049    2.888    0.004
##     Prop.Mam.Abdn2       -0.069    0.040   -1.715    0.086
##     Mn.Sp.BdyMsXA2        0.359    0.033   10.723    0.000
##     Mean.Rap.Crck2        0.116    0.029    3.961    0.000
##     X50km_Pop2            0.065    0.038    1.714    0.086
##     X10km_Pop2            0.385    0.040    9.539    0.000
##     GDP.Millions2         0.158    0.026    6.183    0.000
##   Total.Animals2 ~
##     Zoo.Area.ha2          0.309    0.027   11.617    0.000
##     Sp.Richness2          0.759    0.023   32.975    0.000
##     Mn.Sp.BdyMsXA2       -0.157    0.027   -5.852    0.000
##     GDP.Millions2        -0.136    0.022   -6.202    0.000
##   Sp.Richness2 ~
##     Prop.Mam.Sp2         -0.563    0.018  -30.494    0.000
##     Mam.Sp.Rchnss2        0.779    0.018   42.208    0.000
##   Mean.Raup.Crick2 ~
##     Sp.Richness2         -0.467    0.075   -6.198    0.000
##     Total.Animals2        0.478    0.071    6.762    0.000
##     Mn.Sp.BdyMsXA2       -0.246    0.051   -4.837    0.000
##     Prop.Mam.Sp2         -0.366    0.058   -6.343    0.000
##
## Covariances:
##                                Estimate  Std.Err  z-value  P(>|z|)
##   Mam.Sp.Richness2 ~~
##     Prop.Mam.Abdn2                0.058    0.047    1.237    0.216
##     Mn.Sp.BdyMsXA2                0.227    0.048    4.750    0.000
##     X50km_Pop2                    0.204    0.048    4.281    0.000
##     X10km_Pop2                    0.284    0.048    5.852    0.000
##     GDP.Millions2                -0.060    0.047   -1.277    0.202
##     Zoo.Area.ha2                  0.381    0.050    7.634    0.000
##     Prop.Mam.Sp2                  0.088    0.047    1.882    0.060
##   Prop.Mam.Abdun2 ~~
```

```
##     Mn.Sp.BdyMsXA2             0.592    0.054   10.922    0.000
##     X50km_Pop2                -0.123    0.047   -2.622    0.009
##     X10km_Pop2                -0.248    0.048   -5.165    0.000
##     GDP.Millions2             -0.115    0.047   -2.458    0.014
##     Zoo.Area.ha2               0.352    0.049    7.121    0.000
##     Prop.Mam.Sp2               0.878    0.062   14.135    0.000
##   Mean.Sp.BodyMassXAbund2 ~~
##     X50km_Pop2                 0.013    0.047    0.275    0.784
##     X10km_Pop2                 0.021    0.047    0.455    0.649
##     GDP.Millions2             -0.079    0.047   -1.682    0.093
##     Zoo.Area.ha2               0.522    0.053    9.923    0.000
##     Prop.Mam.Sp2               0.628    0.055   11.405    0.000
##   X50km_Pop2 ~~
##     X10km_Pop2                 0.752    0.058   12.886    0.000
##     GDP.Millions2             -0.013    0.047   -0.281    0.779
##     Zoo.Area.ha2               0.060    0.047    1.291    0.197
##     Prop.Mam.Sp2              -0.096    0.047   -2.054    0.040
##   X10km_Pop2 ~~
##     GDP.Millions2             -0.028    0.047   -0.604    0.546
##     Zoo.Area.ha2              -0.010    0.047   -0.208    0.835
##     Prop.Mam.Sp2              -0.204    0.048   -4.293    0.000
##   GDP.Millions2 ~~
##     Zoo.Area.ha2              -0.027    0.047   -0.588    0.557
##     Prop.Mam.Sp2              -0.123    0.047   -2.615    0.009
##   Zoo.Area.ha2 ~~
##     Prop.Mam.Sp2               0.312    0.049    6.389    0.000
##
## Variances:
##                    Estimate  Std.Err  z-value  P(>|z|)
##    .Attendance2       0.276    0.018   15.133    0.000
##    .Total.Animals2    0.219    0.014   15.133    0.000
##    .Sp.Richness2      0.154    0.010   15.133    0.000
##    .Mean.Rap.Crck2    0.684    0.045   15.133    0.000
##     Mam.Sp.Rchnss2    0.998    0.066   15.133    0.000
##     Prop.Mam.Abdn2    0.998    0.066   15.133    0.000
##     Mn.Sp.BdyMsXA2    0.998    0.066   15.133    0.000
##     X50km_Pop2        0.998    0.066   15.133    0.000
##     X10km_Pop2        0.998    0.066   15.133    0.000
##     GDP.Millions2     0.998    0.066   15.133    0.000
##     Zoo.Area.ha2      0.998    0.066   15.133    0.000
##     Prop.Mam.Sp2      0.998    0.066   15.133    0.000
##
## R-Square:
##                    Estimate
##     Attendance2       0.704
##     Total.Animals2    0.775
##     Sp.Richness2      0.845
##     Mean.Rap.Crck2    0.323
```

```
# Generate fit indices
fitMeasures(mod.12A.fit, c("agfi", "rmr", "srmr", "rmsea", "cfi", "nnfi", "tli"))
```

```
##  agfi   rmr  srmr rmsea   cfi  nnfi   tli
## 0.816 0.041 0.041 0.120 0.948 0.896 0.896
```

```r
# Generate modification indices
mi12A <- modindices(mod.12A.fit)
print(mi12A[mi12A$mi > 3.0,])
```

```
##                          lhs op              rhs     mi    epc sepc.lv
## 64            Total.Animals2 ~~ Mean.Raup.Crick2 14.603 -0.134  -0.134
## 65              Sp.Richness2 ~~ Mean.Raup.Crick2 10.304 -0.055  -0.055
## 68            Total.Animals2  ~      Attendance2  7.082  0.118   0.118
## 69            Total.Animals2  ~ Mean.Raup.Crick2 18.135 -0.189  -0.189
## 72            Total.Animals2  ~       X50km_Pop2 43.331  0.148   0.148
## 73            Total.Animals2  ~       X10km_Pop2 14.994  0.091   0.091
## 74            Total.Animals2  ~     Prop.Mam.Sp2  3.589  0.062   0.062
## 76              Sp.Richness2  ~   Total.Animals2  4.049  0.072   0.072
## 77              Sp.Richness2  ~ Mean.Raup.Crick2  3.898 -0.046  -0.046
## 83              Sp.Richness2  ~     Zoo.Area.ha2 10.169  0.067   0.067
## 84          Mean.Raup.Crick2  ~      Attendance2  9.383  0.261   0.261
## 85          Mean.Raup.Crick2  ~ Mam.Sp.Richness2 10.304  0.278   0.278
## 86          Mean.Raup.Crick2  ~   Prop.Mam.Abdun2  3.621  0.156   0.156
## 87          Mean.Raup.Crick2  ~       X50km_Pop2  8.651  0.117   0.117
## 88          Mean.Raup.Crick2  ~       X10km_Pop2  6.491  0.106   0.106
## 90          Mean.Raup.Crick2  ~     Zoo.Area.ha2 17.464  0.222   0.222
## 91          Mam.Sp.Richness2  ~      Attendance2  3.723 -0.290  -0.290
## 93          Mam.Sp.Richness2  ~     Sp.Richness2  4.015 -0.432  -0.432
## 102          Prop.Mam.Abdun2  ~      Attendance2 12.726 -0.163  -0.163
## 103          Prop.Mam.Abdun2  ~   Total.Animals2 12.861 -0.139  -0.139
## 113 Mean.Sp.BodyMassXAbund2  ~      Attendance2 16.460 -0.442  -0.442
## 114 Mean.Sp.BodyMassXAbund2  ~   Total.Animals2  9.515 -0.245  -0.245
## 115 Mean.Sp.BodyMassXAbund2  ~     Sp.Richness2  8.886 -0.239  -0.239
## 116 Mean.Sp.BodyMassXAbund2  ~ Mean.Raup.Crick2 20.660 -0.383  -0.383
## 124               X50km_Pop2  ~      Attendance2 23.457  0.449   0.449
## 125               X50km_Pop2  ~   Total.Animals2 22.245  0.256   0.256
## 127               X50km_Pop2  ~ Mean.Raup.Crick2  5.766  0.084   0.084
## 157             Zoo.Area.ha2  ~      Attendance2 10.025  0.236   0.236
## 158             Zoo.Area.ha2  ~   Total.Animals2 14.664  0.424   0.424
## 159             Zoo.Area.ha2  ~     Sp.Richness2 16.564  0.379   0.379
## 160             Zoo.Area.ha2  ~ Mean.Raup.Crick2 10.470  0.159   0.159
## 168             Prop.Mam.Sp2  ~      Attendance2 15.256  0.155   0.155
## 169             Prop.Mam.Sp2  ~   Total.Animals2 20.415  0.181   0.181
##     sepc.all sepc.nox
## 64    -0.135   -0.135
## 65    -0.055   -0.055
## 68     0.115    0.115
## 69    -0.193   -0.193
## 72     0.150    0.150
## 73     0.092    0.092
## 74     0.063    0.063
## 76     0.072    0.072
## 77    -0.046   -0.046
## 83     0.067    0.067
## 84     0.251    0.251
## 85     0.276    0.277
## 86     0.155    0.156
## 87     0.116    0.116
## 88     0.105    0.106
```

```
## 90     0.221    0.221
## 91    -0.281   -0.281
## 93    -0.432   -0.432
## 102   -0.158   -0.158
## 103   -0.137   -0.137
## 113   -0.428   -0.428
## 114   -0.243   -0.243
## 115   -0.239   -0.239
## 116   -0.386   -0.386
## 124    0.434    0.434
## 125    0.253    0.253
## 127    0.085    0.085
## 157    0.229    0.229
## 158    0.419    0.419
## 159    0.379    0.379
## 160    0.160    0.160
## 168    0.150    0.150
## 169    0.179    0.179
```

```
# Adjust for the nested nature of the data (institutions within countries)
# Fit model and generate model summary
design <- svydesign(ids = ~Country, nest=TRUE, data=sem_attendance_data)
fit.adj12A <- lavaan.survey(lavaan.fit = mod.12A.fit, survey.design = design)
summary(fit.adj12A, rsq = TRUE)
```

```
## lavaan (0.5-23.1097) converged normally after  49 iterations
##
##   Number of observations                           458
##
##   Estimator                                         ML      Robust
##   Minimum Function Test Statistic              144.618      57.337
##   Degrees of freedom                                19          19
##   P-value (Chi-square)                           0.000       0.000
##   Scaling correction factor                                  2.522
##     for the Satorra-Bentler correction
##
## Parameter Estimates:
##
##   Information                                 Expected
##   Standard Errors                           Robust.sem
##
## Regressions:
##                    Estimate  Std.Err  z-value  P(>|z|)
##   Attendance2 ~
##     Sp.Richness2     -0.217    0.078   -2.802    0.005
##     Total.Animals2    0.569    0.052   10.928    0.000
##     Mam.Sp.Rchnss2    0.140    0.043    3.232    0.001
##     Prop.Mam.Abdn2   -0.069    0.031   -2.233    0.026
##     Mn.Sp.BdyMsXA2    0.359    0.029   12.496    0.000
##     Mean.Rap.Crck2    0.116    0.034    3.367    0.001
##     X50km_Pop2        0.065    0.035    1.836    0.066
##     X10km_Pop2        0.385    0.045    8.649    0.000
##     GDP.Millions2     0.158    0.027    5.877    0.000
##   Total.Animals2 ~
##     Zoo.Area.ha2      0.309    0.040    7.761    0.000
```

```
##     Sp.Richness2        0.759    0.047   16.029    0.000
##     Mn.Sp.BdyMsXA2     -0.157    0.034   -4.634    0.000
##     GDP.Millions2      -0.136    0.069   -1.983    0.047
##   Sp.Richness2 ~
##     Prop.Mam.Sp2       -0.563    0.041  -13.858    0.000
##     Mam.Sp.Rchnss2      0.779    0.057   13.593    0.000
##   Mean.Raup.Crick2 ~
##     Sp.Richness2       -0.467    0.180   -2.591    0.010
##     Total.Animals2      0.478    0.111    4.298    0.000
##     Mn.Sp.BdyMsXA2     -0.246    0.082   -3.001    0.003
##     Prop.Mam.Sp2       -0.366    0.077   -4.757    0.000
##
## Covariances:
##                             Estimate  Std.Err  z-value  P(>|z|)
##   Mam.Sp.Richness2 ~~
##     Prop.Mam.Abdn2             0.058    0.049    1.172    0.241
##     Mn.Sp.BdyMsXA2             0.227    0.078    2.921    0.003
##     X50km_Pop2                 0.204    0.082    2.487    0.013
##     X10km_Pop2                 0.284    0.066    4.278    0.000
##     GDP.Millions2             -0.060    0.064   -0.937    0.349
##     Zoo.Area.ha2               0.381    0.064    5.985    0.000
##     Prop.Mam.Sp2               0.088    0.061    1.435    0.151
##   Prop.Mam.Abdun2 ~~
##     Mn.Sp.BdyMsXA2             0.592    0.109    5.445    0.000
##     X50km_Pop2                -0.123    0.054   -2.263    0.024
##     X10km_Pop2                -0.248    0.060   -4.154    0.000
##     GDP.Millions2             -0.115    0.101   -1.140    0.254
##     Zoo.Area.ha2               0.352    0.092    3.822    0.000
##     Prop.Mam.Sp2               0.878    0.128    6.865    0.000
##   Mean.Sp.BodyMassXAbund2 ~~
##     X50km_Pop2                 0.013    0.074    0.174    0.862
##     X10km_Pop2                 0.021    0.064    0.329    0.742
##     GDP.Millions2             -0.079    0.038   -2.055    0.040
##     Zoo.Area.ha2               0.522    0.101    5.177    0.000
##     Prop.Mam.Sp2               0.628    0.126    5.002    0.000
##   X50km_Pop2 ~~
##     X10km_Pop2                 0.752    0.126    5.982    0.000
##     GDP.Millions2             -0.013    0.100   -0.131    0.896
##     Zoo.Area.ha2               0.060    0.069    0.880    0.379
##     Prop.Mam.Sp2              -0.096    0.051   -1.888    0.059
##   X10km_Pop2 ~~
##     GDP.Millions2             -0.028    0.056   -0.505    0.613
##     Zoo.Area.ha2              -0.010    0.078   -0.124    0.901
##     Prop.Mam.Sp2              -0.204    0.055   -3.685    0.000
##   GDP.Millions2 ~~
##     Zoo.Area.ha2              -0.027    0.041   -0.673    0.501
##     Prop.Mam.Sp2              -0.123    0.105   -1.172    0.241
##   Zoo.Area.ha2 ~~
##     Prop.Mam.Sp2               0.312    0.089    3.510    0.000
##
## Intercepts:
##                    Estimate  Std.Err  z-value  P(>|z|)
##    .Attendance2      -0.000    0.030   -0.000    1.000
##    .Total.Animals2   -0.000    0.067   -0.000    1.000
```

```
##    .Sp.Richness2      0.000    0.032    0.000    1.000
##    .Mean.Rap.Crck2    0.000    0.076    0.000    1.000
##     Mam.Sp.Rchnss2   -0.000    0.079   -0.000    1.000
##     Prop.Mam.Abdn2    0.000    0.113    0.000    1.000
##     Mn.Sp.BdyMsXA2    0.000    0.071    0.000    1.000
##     X50km_Pop2       -0.000    0.114   -0.000    1.000
##     X10km_Pop2        0.000    0.105    0.000    1.000
##     GDP.Millions2    -0.000    0.345   -0.000    1.000
##     Zoo.Area.ha2      0.000    0.064    0.000    1.000
##     Prop.Mam.Sp2      0.000    0.118    0.000    1.000
##
## Variances:
##                    Estimate  Std.Err  z-value  P(>|z|)
##    .Attendance2       0.276    0.028    9.964    0.000
##    .Total.Animals2    0.219    0.037    5.986    0.000
##    .Sp.Richness2      0.154    0.044    3.506    0.000
##    .Mean.Rap.Crck2    0.684    0.059   11.527    0.000
##     Mam.Sp.Rchnss2    0.998    0.115    8.669    0.000
##     Prop.Mam.Abdn2    0.998    0.121    8.221    0.000
##     Mn.Sp.BdyMsXA2    0.998    0.160    6.219    0.000
##     X50km_Pop2        0.998    0.131    7.623    0.000
##     X10km_Pop2        0.998    0.174    5.745    0.000
##     GDP.Millions2     0.998    0.246    4.061    0.000
##     Zoo.Area.ha2      0.998    0.112    8.886    0.000
##     Prop.Mam.Sp2      0.998    0.150    6.662    0.000
##
## R-Square:
##                    Estimate
##     Attendance2       0.704
##     Total.Animals2    0.775
##     Sp.Richness2      0.845
##     Mean.Rap.Crck2    0.323
```

```
# Generate fit indices
fitMeasures(fit.adj12A, c("agfi", "rmr", "srmr", "rmsea", "cfi", "nnfi", "tli"))
```

```
##  agfi   rmr  srmr rmsea   cfi  nnfi   tli
## 0.788 0.041 0.038 0.120 0.948 0.896 0.896
```

```
# Generate modification indices
mi12adjA <- modindices(fit.adj12A)
print(mi12adjA[mi12adjA$mi > 3.0,])
```

```
##                  lhs op               rhs     mi mi.scaled    epc
## 76        Total.Animals2 ~~ Mean.Raup.Crick2 14.603     5.790 -0.134
## 77          Sp.Richness2 ~~ Mean.Raup.Crick2 10.304     4.085 -0.055
## 80        Total.Animals2  ~       Attendance2  7.082     2.808  0.118
## 81        Total.Animals2  ~ Mean.Raup.Crick2 18.135     7.190 -0.189
## 84        Total.Animals2  ~        X50km_Pop2 43.331    17.180  0.148
## 85        Total.Animals2  ~        X10km_Pop2 14.994     5.945  0.091
## 86        Total.Animals2  ~      Prop.Mam.Sp2  3.589     1.423  0.062
## 88          Sp.Richness2  ~    Total.Animals2  4.049     1.605  0.072
## 89          Sp.Richness2  ~ Mean.Raup.Crick2  3.898     1.545 -0.046
## 95          Sp.Richness2  ~      Zoo.Area.ha2 10.169     4.032  0.067
## 96      Mean.Raup.Crick2  ~       Attendance2  9.383     3.720  0.261
## 97      Mean.Raup.Crick2  ~ Mam.Sp.Richness2 10.304     4.085  0.278
```

```
## 98          Mean.Raup.Crick2  ~  Prop.Mam.Abdun2  3.621     1.436  0.156
## 99          Mean.Raup.Crick2  ~       X50km_Pop2  8.651     3.430  0.117
## 100         Mean.Raup.Crick2  ~       X10km_Pop2  6.491     2.574  0.106
## 102         Mean.Raup.Crick2  ~     Zoo.Area.ha2 17.464     6.924  0.222
## 103         Mam.Sp.Richness2  ~      Attendance2  3.723     1.476 -0.290
## 105         Mam.Sp.Richness2  ~     Sp.Richness2  4.015     1.592 -0.432
## 114           Prop.Mam.Abdun2  ~      Attendance2 12.726     5.046 -0.163
## 115           Prop.Mam.Abdun2  ~   Total.Animals2 12.861     5.099 -0.139
## 125 Mean.Sp.BodyMassXAbund2  ~      Attendance2 16.460     6.526 -0.442
## 126 Mean.Sp.BodyMassXAbund2  ~   Total.Animals2  9.515     3.772 -0.245
## 127 Mean.Sp.BodyMassXAbund2  ~     Sp.Richness2  8.886     3.523 -0.239
## 128 Mean.Sp.BodyMassXAbund2  ~ Mean.Raup.Crick2 20.660     8.191 -0.383
## 136                X50km_Pop2  ~      Attendance2 23.457     9.300  0.449
## 137                X50km_Pop2  ~   Total.Animals2 22.245     8.819  0.256
## 139                X50km_Pop2  ~ Mean.Raup.Crick2  5.766     2.286  0.084
## 169              Zoo.Area.ha2  ~      Attendance2 10.025     3.975  0.236
## 170              Zoo.Area.ha2  ~   Total.Animals2 14.664     5.814  0.424
## 171              Zoo.Area.ha2  ~     Sp.Richness2 16.564     6.567  0.379
## 172              Zoo.Area.ha2  ~ Mean.Raup.Crick2 10.470     4.151  0.159
## 180              Prop.Mam.Sp2  ~      Attendance2 15.256     6.049  0.155
## 181              Prop.Mam.Sp2  ~   Total.Animals2 20.415     8.094  0.181
##     sepc.lv sepc.all sepc.nox
## 76   -0.134   -0.135   -0.135
## 77   -0.055   -0.055   -0.055
## 80    0.118    0.115    0.115
## 81   -0.189   -0.193   -0.193
## 84    0.148    0.150    0.150
## 85    0.091    0.092    0.092
## 86    0.062    0.063    0.063
## 88    0.072    0.072    0.072
## 89   -0.046   -0.046   -0.046
## 95    0.067    0.067    0.067
## 96    0.261    0.251    0.251
## 97    0.278    0.276    0.277
## 98    0.156    0.155    0.156
## 99    0.117    0.116    0.116
## 100   0.106    0.105    0.106
## 102   0.222    0.221    0.221
## 103  -0.290   -0.281   -0.281
## 105  -0.432   -0.432   -0.432
## 114  -0.163   -0.158   -0.158
## 115  -0.139   -0.137   -0.137
## 125  -0.442   -0.428   -0.428
## 126  -0.245   -0.243   -0.243
## 127  -0.239   -0.239   -0.239
## 128  -0.383   -0.386   -0.386
## 136   0.449    0.434    0.434
## 137   0.256    0.253    0.253
## 139   0.084    0.085    0.085
## 169   0.236    0.229    0.229
## 170   0.424    0.419    0.419
## 171   0.379    0.379    0.379
## 172   0.159    0.160    0.160
## 180   0.155    0.150    0.150
```

```
## 181   0.181    0.179    0.179
```

Model 13

Based on the results generated from the nested twelfth model, the highest p-value relationship to be considered for removal is **Attendance2** ~ **X50km_Pop2** with a p-value of **0.066**. Therefore we decide to remove this pathway. Once again, the model summary, fit indices and modification indices were all generated for the model, adjusting for the nested nature of data.

```
# Attendance SEM (Species Abundance)

# Model 13
# Removal of Attendance2 ~ X50km_Pop2, p = 0.066

mod.13A <- 'Attendance2 ~ Sp.Richness2 + Total.Animals2
+ Mam.Sp.Richness2 + Prop.Mam.Abdun2
+ Mean.Sp.BodyMassXAbund2 + Mean.Raup.Crick2
+ X10km_Pop2 + GDP.Millions2

Total.Animals2 ~ Zoo.Area.ha2 + Sp.Richness2 + Mean.Sp.BodyMassXAbund2 + GDP.Millions2
Sp.Richness2 ~ Prop.Mam.Sp2 + Mam.Sp.Richness2
Mean.Raup.Crick2 ~ Sp.Richness2 + Total.Animals2 + Mean.Sp.BodyMassXAbund2 + Prop.Mam.Sp2'

# Fit model and generate model summary
mod.13A.fit <- sem(mod.13A, data = sem_attendance_data, fixed.x=FALSE)
summary(mod.13A.fit, rsq = TRUE)
```

```
## lavaan (0.5-23.1097) converged normally after  44 iterations
##
##   Number of observations                           458
##
##   Estimator                                         ML
##   Minimum Function Test Statistic              107.467
##   Degrees of freedom                                16
##   P-value (Chi-square)                           0.000
##
## Parameter Estimates:
##
##   Information                                 Expected
##   Standard Errors                             Standard
##
## Regressions:
##                     Estimate  Std.Err  z-value  P(>|z|)
##   Attendance2 ~
##     Sp.Richness2      -0.234    0.065   -3.599    0.000
##     Total.Animals2     0.592    0.049   12.124    0.000
##     Mam.Sp.Rchnss2     0.137    0.049    2.816    0.005
##     Prop.Mam.Abdn2    -0.061    0.040   -1.523    0.128
##     Mn.Sp.BdyMsXA2     0.356    0.033   10.654    0.000
##     Mean.Rap.Crck2     0.120    0.029    4.072    0.000
##     X10km_Pop2         0.434    0.027   15.992    0.000
##     GDP.Millions2      0.162    0.026    6.327    0.000
##   Total.Animals2 ~
##     Zoo.Area.ha2       0.309    0.027   11.617    0.000
```

```
##     Sp.Richness2        0.759    0.023   32.975    0.000
##     Mn.Sp.BdyMsXA2     -0.157    0.027   -5.852    0.000
##     GDP.Millions2      -0.136    0.022   -6.202    0.000
##   Sp.Richness2 ~
##     Prop.Mam.Sp2       -0.563    0.018  -30.494    0.000
##     Mam.Sp.Rchnss2      0.779    0.018   42.208    0.000
##   Mean.Raup.Crick2 ~
##     Sp.Richness2       -0.467    0.075   -6.198    0.000
##     Total.Animals2      0.478    0.071    6.762    0.000
##     Mn.Sp.BdyMsXA2     -0.246    0.051   -4.837    0.000
##     Prop.Mam.Sp2       -0.366    0.058   -6.343    0.000
##
## Covariances:
##                           Estimate  Std.Err  z-value  P(>|z|)
##   Mam.Sp.Richness2 ~~
##     Prop.Mam.Abdn2           0.058    0.047    1.237    0.216
##     Mn.Sp.BdyMsXA2           0.227    0.048    4.750    0.000
##     X10km_Pop2               0.284    0.048    5.852    0.000
##     GDP.Millions2           -0.060    0.047   -1.277    0.202
##     Zoo.Area.ha2             0.381    0.050    7.634    0.000
##     Prop.Mam.Sp2             0.088    0.047    1.882    0.060
##   Prop.Mam.Abdun2 ~~
##     Mn.Sp.BdyMsXA2           0.592    0.054   10.922    0.000
##     X10km_Pop2              -0.248    0.048   -5.165    0.000
##     GDP.Millions2           -0.115    0.047   -2.458    0.014
##     Zoo.Area.ha2             0.352    0.049    7.121    0.000
##     Prop.Mam.Sp2             0.878    0.062   14.135    0.000
##   Mean.Sp.BodyMassXAbund2 ~~
##     X10km_Pop2               0.021    0.047    0.455    0.649
##     GDP.Millions2           -0.079    0.047   -1.682    0.093
##     Zoo.Area.ha2             0.522    0.053    9.923    0.000
##     Prop.Mam.Sp2             0.628    0.055   11.405    0.000
##   X10km_Pop2 ~~
##     GDP.Millions2           -0.028    0.047   -0.604    0.546
##     Zoo.Area.ha2            -0.010    0.047   -0.208    0.835
##     Prop.Mam.Sp2            -0.204    0.048   -4.293    0.000
##   GDP.Millions2 ~~
##     Zoo.Area.ha2            -0.027    0.047   -0.588    0.557
##     Prop.Mam.Sp2            -0.123    0.047   -2.615    0.009
##   Zoo.Area.ha2 ~~
##     Prop.Mam.Sp2             0.312    0.049    6.389    0.000
##
## Variances:
##                   Estimate  Std.Err  z-value  P(>|z|)
##    .Attendance2       0.278    0.018   15.133    0.000
##    .Total.Animals2    0.219    0.014   15.133    0.000
##    .Sp.Richness2      0.154    0.010   15.133    0.000
##    .Mean.Rap.Crck2    0.684    0.045   15.133    0.000
##     Mam.Sp.Rchnss2    0.998    0.066   15.133    0.000
##     Prop.Mam.Abdn2    0.998    0.066   15.133    0.000
##     Mn.Sp.BdyMsXA2    0.998    0.066   15.133    0.000
##     X10km_Pop2        0.998    0.066   15.133    0.000
##     GDP.Millions2     0.998    0.066   15.133    0.000
##     Zoo.Area.ha2      0.998    0.066   15.133    0.000
```

```
##     Prop.Mam.Sp2      0.998    0.066   15.133    0.000
##
## R-Square:
##                    Estimate
##     Attendance2       0.704
##     Total.Animals2    0.775
##     Sp.Richness2      0.845
##     Mean.Rap.Crck2    0.323
```

```r
# Generate fit indices
fitMeasures(mod.13A.fit, c("agfi", "rmr", "srmr", "rmsea", "cfi", "nnfi", "tli"))
```

```
##  agfi   rmr  srmr rmsea   cfi  nnfi   tli
## 0.846 0.031 0.031 0.112 0.962 0.918 0.918
```

```r
# Generate modification indices
mi13A <- modindices(mod.13A.fit)
print(mi13A[mi13A$mi > 3.0,])
```

```
##                         lhs op              rhs     mi    epc sepc.lv
## 55           Total.Animals2 ~~ Mean.Raup.Crick2 14.603 -0.134  -0.134
## 56             Sp.Richness2 ~~ Mean.Raup.Crick2 10.304 -0.055  -0.055
## 59           Total.Animals2  ~      Attendance2  4.763  0.097   0.097
## 60           Total.Animals2  ~ Mean.Raup.Crick2 18.135 -0.189  -0.189
## 63           Total.Animals2  ~       X10km_Pop2 14.994  0.091   0.091
## 64           Total.Animals2  ~     Prop.Mam.Sp2  3.589  0.062   0.062
## 66             Sp.Richness2  ~   Total.Animals2  4.049  0.072   0.072
## 67             Sp.Richness2  ~ Mean.Raup.Crick2  3.898 -0.046  -0.046
## 72             Sp.Richness2  ~     Zoo.Area.ha2 10.169  0.067   0.067
## 73         Mean.Raup.Crick2  ~      Attendance2  8.674  0.253   0.253
## 74         Mean.Raup.Crick2  ~ Mam.Sp.Richness2 10.304  0.278   0.278
## 75         Mean.Raup.Crick2  ~  Prop.Mam.Abdun2  3.621  0.156   0.156
## 76         Mean.Raup.Crick2  ~       X10km_Pop2  6.491  0.106   0.106
## 78         Mean.Raup.Crick2  ~     Zoo.Area.ha2 17.464  0.222   0.222
## 79         Mam.Sp.Richness2  ~      Attendance2  5.059 -0.333  -0.333
## 81         Mam.Sp.Richness2  ~     Sp.Richness2  4.166 -0.443  -0.443
## 89          Prop.Mam.Abdun2  ~      Attendance2 12.017 -0.155  -0.155
## 90          Prop.Mam.Abdun2  ~   Total.Animals2 11.654 -0.132  -0.132
## 99  Mean.Sp.BodyMassXAbund2  ~      Attendance2 24.181 -0.544  -0.544
## 100 Mean.Sp.BodyMassXAbund2  ~   Total.Animals2 16.344 -0.328  -0.328
## 101 Mean.Sp.BodyMassXAbund2  ~     Sp.Richness2  9.183 -0.246  -0.246
## 102 Mean.Sp.BodyMassXAbund2  ~ Mean.Raup.Crick2 30.852 -0.493  -0.493
## 109              X10km_Pop2  ~      Attendance2 20.445  0.572   0.572
## 110              X10km_Pop2  ~   Total.Animals2  9.871  0.239   0.239
## 112              X10km_Pop2  ~ Mean.Raup.Crick2 13.435  0.180   0.180
## 129            Zoo.Area.ha2  ~      Attendance2 13.165  0.274   0.274
## 130            Zoo.Area.ha2  ~   Total.Animals2 25.369  0.572   0.572
## 131            Zoo.Area.ha2  ~     Sp.Richness2 17.061  0.388   0.388
## 132            Zoo.Area.ha2  ~ Mean.Raup.Crick2 13.364  0.182   0.182
## 139            Prop.Mam.Sp2  ~      Attendance2 16.976  0.161   0.161
## 140            Prop.Mam.Sp2  ~   Total.Animals2 22.851  0.192   0.192
##     sepc.all sepc.nox
## 55    -0.135   -0.135
## 56    -0.055   -0.055
## 59     0.095    0.095
## 60    -0.193   -0.193
```

```
## 63     0.092    0.092
## 64     0.063    0.063
## 66     0.072    0.072
## 67    -0.046   -0.046
## 72     0.067    0.067
## 73     0.244    0.244
## 74     0.276    0.277
## 75     0.155    0.156
## 76     0.105    0.106
## 78     0.221    0.221
## 79    -0.323   -0.323
## 81    -0.443   -0.443
## 89    -0.151   -0.151
## 90    -0.131   -0.131
## 99    -0.528   -0.528
## 100   -0.324   -0.324
## 101   -0.246   -0.246
## 102   -0.496   -0.496
## 109    0.554    0.554
## 110    0.236    0.236
## 112    0.181    0.181
## 129    0.265    0.265
## 130    0.565    0.565
## 131    0.388    0.388
## 132    0.183    0.183
## 139    0.157    0.157
## 140    0.190    0.190
```

```
# Adjust for the nested nature of the data (institutions within countries)
# Fit model and generate model summary
design <- svydesign(ids = ~Country, nest=TRUE, data=sem_attendance_data)
fit.adj13A <- lavaan.survey(lavaan.fit = mod.13A.fit, survey.design = design)
summary(fit.adj13A, rsq = TRUE)
```

```
## lavaan (0.5-23.1097) converged normally after  43 iterations
##
##   Number of observations                           458
##
##   Estimator                                         ML      Robust
##   Minimum Function Test Statistic              107.467      44.628
##   Degrees of freedom                                16          16
##   P-value (Chi-square)                           0.000       0.000
##   Scaling correction factor                                  2.408
##     for the Satorra-Bentler correction
##
## Parameter Estimates:
##
##   Information                                 Expected
##   Standard Errors                           Robust.sem
##
## Regressions:
##                        Estimate  Std.Err  z-value  P(>|z|)
##   Attendance2 ~
##     Sp.Richness2         -0.234    0.072   -3.263    0.001
##     Total.Animals2        0.592    0.049   12.002    0.000
```

```
##     Mam.Sp.Rchnss2      0.137    0.043    3.195    0.001
##     Prop.Mam.Abdn2     -0.061    0.031   -2.002    0.045
##     Mn.Sp.BdyMsXA2      0.356    0.027   13.020    0.000
##     Mean.Rap.Crck2      0.120    0.036    3.351    0.001
##     X10km_Pop2          0.434    0.034   12.857    0.000
##     GDP.Millions2       0.162    0.028    5.862    0.000
##   Total.Animals2 ~
##     Zoo.Area.ha2        0.309    0.040    7.761    0.000
##     Sp.Richness2        0.759    0.047   16.029    0.000
##     Mn.Sp.BdyMsXA2     -0.157    0.034   -4.634    0.000
##     GDP.Millions2      -0.136    0.069   -1.983    0.047
##   Sp.Richness2 ~
##     Prop.Mam.Sp2       -0.563    0.041  -13.858    0.000
##     Mam.Sp.Rchnss2      0.779    0.057   13.593    0.000
##   Mean.Raup.Crick2 ~
##     Sp.Richness2       -0.467    0.180   -2.591    0.010
##     Total.Animals2      0.478    0.111    4.298    0.000
##     Mn.Sp.BdyMsXA2     -0.246    0.082   -3.001    0.003
##     Prop.Mam.Sp2       -0.366    0.077   -4.757    0.000
##
## Covariances:
##                                Estimate  Std.Err  z-value  P(>|z|)
##   Mam.Sp.Richness2 ~~
##     Prop.Mam.Abdn2                0.058    0.049    1.172    0.241
##     Mn.Sp.BdyMsXA2                0.227    0.078    2.921    0.003
##     X10km_Pop2                    0.284    0.066    4.278    0.000
##     GDP.Millions2                -0.060    0.064   -0.937    0.349
##     Zoo.Area.ha2                  0.381    0.064    5.985    0.000
##     Prop.Mam.Sp2                  0.088    0.061    1.435    0.151
##   Prop.Mam.Abdun2 ~~
##     Mn.Sp.BdyMsXA2                0.592    0.109    5.445    0.000
##     X10km_Pop2                   -0.248    0.060   -4.154    0.000
##     GDP.Millions2                -0.115    0.101   -1.140    0.254
##     Zoo.Area.ha2                  0.352    0.092    3.822    0.000
##     Prop.Mam.Sp2                  0.878    0.128    6.865    0.000
##   Mean.Sp.BodyMassXAbund2 ~~
##     X10km_Pop2                    0.021    0.064    0.329    0.742
##     GDP.Millions2                -0.079    0.038   -2.055    0.040
##     Zoo.Area.ha2                  0.522    0.101    5.177    0.000
##     Prop.Mam.Sp2                  0.628    0.126    5.002    0.000
##   X10km_Pop2 ~~
##     GDP.Millions2                -0.028    0.056   -0.505    0.613
##     Zoo.Area.ha2                 -0.010    0.078   -0.124    0.901
##     Prop.Mam.Sp2                 -0.204    0.055   -3.685    0.000
##   GDP.Millions2 ~~
##     Zoo.Area.ha2                 -0.027    0.041   -0.673    0.501
##     Prop.Mam.Sp2                 -0.123    0.105   -1.172    0.241
##   Zoo.Area.ha2 ~~
##     Prop.Mam.Sp2                  0.312    0.089    3.510    0.000
##
## Intercepts:
##                    Estimate  Std.Err  z-value  P(>|z|)
##    .Attendance2      -0.000    0.029   -0.000    1.000
##    .Total.Animals2   -0.000    0.067   -0.000    1.000
```

```
##    .Sp.Richness2      0.000    0.032    0.000    1.000
##    .Mean.Rap.Crck2    0.000    0.076    0.000    1.000
##     Mam.Sp.Rchnss2   -0.000    0.079   -0.000    1.000
##     Prop.Mam.Abdn2    0.000    0.113    0.000    1.000
##     Mn.Sp.BdyMsXA2    0.000    0.071    0.000    1.000
##     X10km_Pop2        0.000    0.105    0.000    1.000
##     GDP.Millions2    -0.000    0.345   -0.000    1.000
##     Zoo.Area.ha2      0.000    0.064    0.000    1.000
##     Prop.Mam.Sp2      0.000    0.118    0.000    1.000
##
## Variances:
##                    Estimate  Std.Err  z-value  P(>|z|)
##    .Attendance2       0.278    0.027   10.191    0.000
##    .Total.Animals2    0.219    0.037    5.986    0.000
##    .Sp.Richness2      0.154    0.044    3.506    0.000
##    .Mean.Rap.Crck2    0.684    0.059   11.527    0.000
##     Mam.Sp.Rchnss2    0.998    0.115    8.669    0.000
##     Prop.Mam.Abdn2    0.998    0.121    8.221    0.000
##     Mn.Sp.BdyMsXA2    0.998    0.160    6.219    0.000
##     X10km_Pop2        0.998    0.174    5.745    0.000
##     GDP.Millions2     0.998    0.246    4.061    0.000
##     Zoo.Area.ha2      0.998    0.112    8.886    0.000
##     Prop.Mam.Sp2      0.998    0.150    6.662    0.000
##
## R-Square:
##                    Estimate
##     Attendance2       0.704
##     Total.Animals2    0.775
##     Sp.Richness2      0.845
##     Mean.Rap.Crck2    0.323
```

```
# Generate fit indices
fitMeasures(fit.adj13A, c("agfi", "rmr", "srmr", "rmsea", "cfi", "nnfi", "tli"))
```

```
##  agfi   rmr  srmr rmsea   cfi  nnfi   tli
## 0.821 0.031 0.029 0.112 0.962 0.918 0.918
```

```
# Generate modification indices
mi13adjA <- modindices(fit.adj13A)
print(mi13adjA[mi13adjA$mi > 3.0,])
```

```
##                         lhs op             rhs     mi mi.scaled    epc
## 66           Total.Animals2 ~~ Mean.Raup.Crick2 14.603     6.064 -0.134
## 67             Sp.Richness2 ~~ Mean.Raup.Crick2 10.304     4.279 -0.055
## 70           Total.Animals2  ~      Attendance2  4.763     1.978  0.097
## 71           Total.Animals2  ~ Mean.Raup.Crick2 18.135     7.531 -0.189
## 74           Total.Animals2  ~       X10km_Pop2 14.994     6.226  0.091
## 75           Total.Animals2  ~     Prop.Mam.Sp2  3.589     1.490  0.062
## 77             Sp.Richness2  ~   Total.Animals2  4.049     1.681  0.072
## 78             Sp.Richness2  ~ Mean.Raup.Crick2  3.898     1.619 -0.046
## 83             Sp.Richness2  ~     Zoo.Area.ha2 10.169     4.223  0.067
## 84         Mean.Raup.Crick2  ~      Attendance2  8.674     3.602  0.253
## 85         Mean.Raup.Crick2  ~ Mam.Sp.Richness2 10.304     4.279  0.278
## 86         Mean.Raup.Crick2  ~  Prop.Mam.Abdun2  3.621     1.504  0.156
## 87         Mean.Raup.Crick2  ~       X10km_Pop2  6.491     2.696  0.106
## 89         Mean.Raup.Crick2  ~     Zoo.Area.ha2 17.464     7.252  0.222
```

```
## 90         Mam.Sp.Richness2  ~      Attendance2  5.059     2.101 -0.333
## 92         Mam.Sp.Richness2  ~     Sp.Richness2  4.166     1.730 -0.443
## 100         Prop.Mam.Abdun2  ~      Attendance2 12.017     4.990 -0.155
## 101         Prop.Mam.Abdun2  ~   Total.Animals2 11.654     4.840 -0.132
## 110 Mean.Sp.BodyMassXAbund2  ~      Attendance2 24.181    10.042 -0.544
## 111 Mean.Sp.BodyMassXAbund2  ~   Total.Animals2 16.344     6.787 -0.328
## 112 Mean.Sp.BodyMassXAbund2  ~     Sp.Richness2  9.183     3.814 -0.246
## 113 Mean.Sp.BodyMassXAbund2  ~ Mean.Raup.Crick2 30.852    12.812 -0.493
## 120              X10km_Pop2  ~      Attendance2 20.445     8.490  0.572
## 121              X10km_Pop2  ~   Total.Animals2  9.871     4.099  0.239
## 123              X10km_Pop2  ~ Mean.Raup.Crick2 13.435     5.579  0.180
## 140            Zoo.Area.ha2  ~      Attendance2 13.165     5.467  0.274
## 141            Zoo.Area.ha2  ~   Total.Animals2 25.369    10.535  0.572
## 142            Zoo.Area.ha2  ~     Sp.Richness2 17.062     7.085  0.388
## 143            Zoo.Area.ha2  ~ Mean.Raup.Crick2 13.364     5.550  0.182
## 150            Prop.Mam.Sp2  ~      Attendance2 16.976     7.050  0.161
## 151            Prop.Mam.Sp2  ~   Total.Animals2 22.850     9.489  0.192
##     sepc.lv sepc.all sepc.nox
## 66   -0.134   -0.135   -0.135
## 67   -0.055   -0.055   -0.055
## 70    0.097    0.095    0.095
## 71   -0.189   -0.193   -0.193
## 74    0.091    0.092    0.092
## 75    0.062    0.063    0.063
## 77    0.072    0.072    0.072
## 78   -0.046   -0.046   -0.046
## 83    0.067    0.067    0.067
## 84    0.253    0.244    0.244
## 85    0.278    0.276    0.277
## 86    0.156    0.155    0.156
## 87    0.106    0.105    0.106
## 89    0.222    0.221    0.221
## 90   -0.333   -0.323   -0.323
## 92   -0.443   -0.443   -0.443
## 100  -0.155   -0.151   -0.151
## 101  -0.132   -0.131   -0.131
## 110  -0.544   -0.528   -0.528
## 111  -0.328   -0.324   -0.324
## 112  -0.246   -0.246   -0.246
## 113  -0.493   -0.496   -0.496
## 120   0.572    0.554    0.554
## 121   0.239    0.236    0.236
## 123   0.180    0.181    0.181
## 140   0.274    0.265    0.265
## 141   0.572    0.565    0.565
## 142   0.388    0.388    0.388
## 143   0.182    0.183    0.183
## 150   0.161    0.157    0.157
## 151   0.192    0.190    0.190
```

Model 14

At this stage it was decided to remove the link **Attendance2 ~ Prop.Mam.Abdn2** from the analysis, as it adds little extra information than that provided by **Mam.Sp.Richness2**. Once again, the model summary, fit indices and modification indices were all generated for the model, adjusting for the nested nature of data.

```
# Attendance SEM (Species Abundance)

# Model 14
# Removal of Attendance2 ~ Prop.Mam.Abdun2

mod.14A <- 'Attendance2 ~ Sp.Richness2 + Total.Animals2
+ Mam.Sp.Richness2 + Mean.Sp.BodyMassXAbund2 + Mean.Raup.Crick2
+ X10km_Pop2 + GDP.Millions2

Total.Animals2 ~ Zoo.Area.ha2 + Sp.Richness2 + Mean.Sp.BodyMassXAbund2 + GDP.Millions2
Sp.Richness2 ~ Prop.Mam.Sp2 + Mam.Sp.Richness2
Mean.Raup.Crick2 ~ Sp.Richness2 + Total.Animals2 + Mean.Sp.BodyMassXAbund2 + Prop.Mam.Sp2'

# Fit model and generate model summary
mod.14A.fit <- sem(mod.14A, data = sem_attendance_data, fixed.x=FALSE)
summary(mod.14A.fit, rsq = TRUE)
```

```
## lavaan (0.5-23.1097) converged normally after  37 iterations
##
##   Number of observations                           458
##
##   Estimator                                         ML
##   Minimum Function Test Statistic               86.147
##   Degrees of freedom                                13
##   P-value (Chi-square)                           0.000
##
## Parameter Estimates:
##
##   Information                                 Expected
##   Standard Errors                             Standard
##
## Regressions:
##                         Estimate  Std.Err  z-value  P(>|z|)
##   Attendance2 ~
##     Sp.Richness2          -0.184    0.056   -3.271    0.001
##     Total.Animals2         0.587    0.049   12.042    0.000
##     Mam.Sp.Rchnss2         0.102    0.044    2.337    0.019
##     Mn.Sp.BdyMsXA2         0.340    0.031   10.802    0.000
##     Mean.Rap.Crck2         0.125    0.029    4.282    0.000
##     X10km_Pop2             0.444    0.026   16.805    0.000
##     GDP.Millions2          0.163    0.026    6.360    0.000
##   Total.Animals2 ~
##     Zoo.Area.ha2           0.309    0.027   11.617    0.000
##     Sp.Richness2           0.759    0.023   32.975    0.000
##     Mn.Sp.BdyMsXA2        -0.157    0.027   -5.852    0.000
##     GDP.Millions2         -0.136    0.022   -6.202    0.000
##   Sp.Richness2 ~
##     Prop.Mam.Sp2          -0.563    0.018  -30.494    0.000
##     Mam.Sp.Rchnss2         0.779    0.018   42.208    0.000
```

```
##   Mean.Raup.Crick2 ~
##     Sp.Richness2       -0.467    0.075   -6.198    0.000
##     Total.Animals2      0.478    0.071    6.762    0.000
##     Mn.Sp.BdyMsXA2     -0.246    0.051   -4.837    0.000
##     Prop.Mam.Sp2       -0.366    0.058   -6.343    0.000
##
## Covariances:
##                               Estimate  Std.Err  z-value  P(>|z|)
##   Mam.Sp.Richness2 ~~
##     Mn.Sp.BdyMsXA2               0.227    0.048    4.750    0.000
##     X10km_Pop2                   0.284    0.048    5.852    0.000
##     GDP.Millions2               -0.060    0.047   -1.277    0.202
##     Zoo.Area.ha2                 0.381    0.050    7.634    0.000
##     Prop.Mam.Sp2                 0.088    0.047    1.882    0.060
##   Mean.Sp.BodyMassXAbund2 ~~
##     X10km_Pop2                   0.021    0.047    0.455    0.649
##     GDP.Millions2               -0.079    0.047   -1.682    0.093
##     Zoo.Area.ha2                 0.522    0.053    9.923    0.000
##     Prop.Mam.Sp2                 0.628    0.055   11.405    0.000
##   X10km_Pop2 ~~
##     GDP.Millions2               -0.028    0.047   -0.604    0.546
##     Zoo.Area.ha2                -0.010    0.047   -0.208    0.835
##     Prop.Mam.Sp2                -0.204    0.048   -4.293    0.000
##   GDP.Millions2 ~~
##     Zoo.Area.ha2                -0.027    0.047   -0.588    0.557
##     Prop.Mam.Sp2                -0.123    0.047   -2.615    0.009
##   Zoo.Area.ha2 ~~
##     Prop.Mam.Sp2                 0.312    0.049    6.389    0.000
##
## Variances:
##                    Estimate  Std.Err  z-value  P(>|z|)
##    .Attendance2       0.279    0.018   15.133    0.000
##    .Total.Animals2    0.219    0.014   15.133    0.000
##    .Sp.Richness2      0.154    0.010   15.133    0.000
##    .Mean.Rap.Crck2    0.684    0.045   15.133    0.000
##     Mam.Sp.Rchnss2    0.998    0.066   15.133    0.000
##     Mn.Sp.BdyMsXA2    0.998    0.066   15.133    0.000
##     X10km_Pop2        0.998    0.066   15.133    0.000
##     GDP.Millions2     0.998    0.066   15.133    0.000
##     Zoo.Area.ha2      0.998    0.066   15.133    0.000
##     Prop.Mam.Sp2      0.998    0.066   15.133    0.000
##
## R-Square:
##                    Estimate
##     Attendance2       0.702
##     Total.Animals2    0.775
##     Sp.Richness2      0.845
##     Mean.Rap.Crck2    0.323
```

```
# Generate fit indices
fitMeasures(mod.14A.fit, c("agfi", "rmr", "srmr", "rmsea", "cfi", "nnfi", "tli"))
```

```
##  agfi   rmr  srmr rmsea   cfi  nnfi   tli
## 0.864 0.034 0.034 0.111 0.969 0.929 0.929
```

```r
# Generate modification indices
mi14A <- modindices(mod.14A.fit)
print(mi14A[mi14A$mi > 3.0,])
```

```
##                         lhs op              rhs     mi    epc sepc.lv
## 47            Total.Animals2 ~~ Mean.Raup.Crick2 14.603 -0.134  -0.134
## 48              Sp.Richness2 ~~ Mean.Raup.Crick2 10.304 -0.055  -0.055
## 51            Total.Animals2  ~      Attendance2  4.986  0.099   0.099
## 52            Total.Animals2  ~ Mean.Raup.Crick2 18.135 -0.189  -0.189
## 54            Total.Animals2  ~       X10km_Pop2 14.994  0.091   0.091
## 55            Total.Animals2  ~     Prop.Mam.Sp2  3.589  0.062   0.062
## 57              Sp.Richness2  ~   Total.Animals2  4.049  0.072   0.072
## 58              Sp.Richness2  ~ Mean.Raup.Crick2  3.898 -0.046  -0.046
## 62              Sp.Richness2  ~     Zoo.Area.ha2 10.169  0.067   0.067
## 63          Mean.Raup.Crick2  ~      Attendance2  7.319  0.229   0.229
## 64          Mean.Raup.Crick2  ~ Mam.Sp.Richness2 10.304  0.278   0.278
## 65          Mean.Raup.Crick2  ~       X10km_Pop2  6.491  0.106   0.106
## 67          Mean.Raup.Crick2  ~     Zoo.Area.ha2 17.464  0.222   0.222
## 70          Mam.Sp.Richness2  ~     Sp.Richness2  3.963 -0.437  -0.437
## 77  Mean.Sp.BodyMassXAbund2  ~      Attendance2 14.662 -0.356  -0.356
## 78  Mean.Sp.BodyMassXAbund2  ~   Total.Animals2 19.303 -0.358  -0.358
## 79  Mean.Sp.BodyMassXAbund2  ~     Sp.Richness2  9.429 -0.249  -0.249
## 80  Mean.Sp.BodyMassXAbund2  ~ Mean.Raup.Crick2 30.161 -0.494  -0.494
## 86                X10km_Pop2  ~      Attendance2 21.862  0.564   0.564
## 87                X10km_Pop2  ~   Total.Animals2 13.438  0.281   0.281
## 89                X10km_Pop2  ~ Mean.Raup.Crick2 12.838  0.178   0.178
## 96            GDP.Millions2  ~   Total.Animals2  3.612  0.290   0.290
## 104             Zoo.Area.ha2  ~      Attendance2  8.819  0.226   0.226
## 105             Zoo.Area.ha2  ~   Total.Animals2 18.439  0.505   0.505
## 106             Zoo.Area.ha2  ~     Sp.Richness2 16.851  0.391   0.391
## 107             Zoo.Area.ha2  ~ Mean.Raup.Crick2 14.929  0.195   0.195
## 113             Prop.Mam.Sp2  ~      Attendance2  6.144  0.189   0.189
## 114             Prop.Mam.Sp2  ~   Total.Animals2 12.448  0.262   0.262
## 116             Prop.Mam.Sp2  ~ Mean.Raup.Crick2 16.032  0.437   0.437
##     sepc.all sepc.nox
## 47    -0.135   -0.135
## 48    -0.055   -0.055
## 51     0.098    0.098
## 52    -0.193   -0.193
## 54     0.092    0.092
## 55     0.063    0.063
## 57     0.072    0.072
## 58    -0.046   -0.046
## 62     0.067    0.067
## 63     0.221    0.221
## 64     0.276    0.277
## 65     0.105    0.106
## 67     0.221    0.221
## 70    -0.437   -0.437
## 77    -0.346   -0.346
## 78    -0.353   -0.353
## 79    -0.249   -0.249
## 80    -0.497   -0.497
## 86     0.547    0.547
```

```
## 87     0.278    0.278
## 89     0.179    0.179
## 96     0.286    0.286
## 104    0.219    0.219
## 105    0.499    0.499
## 106    0.391    0.391
## 107    0.196    0.196
## 113    0.184    0.184
## 114    0.259    0.259
## 116    0.440    0.440
```

```
# Adjust for the nested nature of the data (institutions within countries)
# Fit model and generate model summary
design <- svydesign(ids = ~Country, nest=TRUE, data=sem_attendance_data)
fit.adj14A <- lavaan.survey(lavaan.fit = mod.14A.fit, survey.design = design)
summary(fit.adj14A, rsq = TRUE)
```

```
## lavaan (0.5-23.1097) converged normally after  37 iterations
##
##   Number of observations                           458
##
##   Estimator                                         ML      Robust
##   Minimum Function Test Statistic               86.147      32.059
##   Degrees of freedom                                13          13
##   P-value (Chi-square)                           0.000       0.002
##   Scaling correction factor                                  2.687
##     for the Satorra-Bentler correction
##
## Parameter Estimates:
##
##   Information                                 Expected
##   Standard Errors                           Robust.sem
##
## Regressions:
##                      Estimate  Std.Err  z-value  P(>|z|)
##   Attendance2 ~
##     Sp.Richness2       -0.184    0.060   -3.089    0.002
##     Total.Animals2      0.587    0.048   12.158    0.000
##     Mam.Sp.Rchnss2      0.102    0.040    2.544    0.011
##     Mn.Sp.BdyMsXA2      0.340    0.028   11.999    0.000
##     Mean.Rap.Crck2      0.125    0.035    3.600    0.000
##     X10km_Pop2          0.444    0.033   13.310    0.000
##     GDP.Millions2       0.163    0.028    5.922    0.000
##   Total.Animals2 ~
##     Zoo.Area.ha2        0.309    0.040    7.761    0.000
##     Sp.Richness2        0.759    0.047   16.029    0.000
##     Mn.Sp.BdyMsXA2     -0.157    0.034   -4.634    0.000
##     GDP.Millions2      -0.136    0.069   -1.983    0.047
##   Sp.Richness2 ~
##     Prop.Mam.Sp2       -0.563    0.041  -13.858    0.000
##     Mam.Sp.Rchnss2      0.779    0.057   13.593    0.000
##   Mean.Raup.Crick2 ~
##     Sp.Richness2       -0.467    0.180   -2.591    0.010
##     Total.Animals2      0.478    0.111    4.298    0.000
##     Mn.Sp.BdyMsXA2     -0.246    0.082   -3.001    0.003
```

```
##     Prop.Mam.Sp2       -0.366    0.077   -4.757    0.000
##
## Covariances:
##                             Estimate  Std.Err  z-value  P(>|z|)
##   Mam.Sp.Richness2 ~~
##     Mn.Sp.BdyMsXA2             0.227    0.078    2.921    0.003
##     X10km_Pop2                 0.284    0.066    4.278    0.000
##     GDP.Millions2             -0.060    0.064   -0.937    0.349
##     Zoo.Area.ha2               0.381    0.064    5.985    0.000
##     Prop.Mam.Sp2               0.088    0.061    1.435    0.151
##   Mean.Sp.BodyMassXAbund2 ~~
##     X10km_Pop2                 0.021    0.064    0.329    0.742
##     GDP.Millions2             -0.079    0.038   -2.055    0.040
##     Zoo.Area.ha2               0.522    0.101    5.177    0.000
##     Prop.Mam.Sp2               0.628    0.126    5.002    0.000
##   X10km_Pop2 ~~
##     GDP.Millions2             -0.028    0.056   -0.505    0.613
##     Zoo.Area.ha2              -0.010    0.078   -0.124    0.901
##     Prop.Mam.Sp2              -0.204    0.055   -3.685    0.000
##   GDP.Millions2 ~~
##     Zoo.Area.ha2              -0.027    0.041   -0.673    0.501
##     Prop.Mam.Sp2              -0.123    0.105   -1.172    0.241
##   Zoo.Area.ha2 ~~
##     Prop.Mam.Sp2               0.312    0.089    3.510    0.000
##
## Intercepts:
##                    Estimate  Std.Err  z-value  P(>|z|)
##    .Attendance2      -0.000    0.029   -0.000    1.000
##    .Total.Animals2   -0.000    0.067   -0.000    1.000
##    .Sp.Richness2     -0.000    0.032   -0.000    1.000
##    .Mean.Rap.Crck2    0.000    0.076    0.000    1.000
##     Mam.Sp.Rchnss2   -0.000    0.079   -0.000    1.000
##     Mn.Sp.BdyMsXA2    0.000    0.071    0.000    1.000
##     X10km_Pop2        0.000    0.105    0.000    1.000
##     GDP.Millions2    -0.000    0.345   -0.000    1.000
##     Zoo.Area.ha2      0.000    0.064    0.000    1.000
##     Prop.Mam.Sp2      0.000    0.118    0.000    1.000
##
## Variances:
##                    Estimate  Std.Err  z-value  P(>|z|)
##    .Attendance2       0.279    0.028   10.018    0.000
##    .Total.Animals2    0.219    0.037    5.986    0.000
##    .Sp.Richness2      0.154    0.044    3.506    0.000
##    .Mean.Rap.Crck2    0.684    0.059   11.527    0.000
##     Mam.Sp.Rchnss2    0.998    0.115    8.669    0.000
##     Mn.Sp.BdyMsXA2    0.998    0.160    6.219    0.000
##     X10km_Pop2        0.998    0.174    5.745    0.000
##     GDP.Millions2     0.998    0.246    4.061    0.000
##     Zoo.Area.ha2      0.998    0.112    8.886    0.000
##     Prop.Mam.Sp2      0.998    0.150    6.662    0.000
##
## R-Square:
##                    Estimate
##     Attendance2       0.702
```

```
##     Total.Animals2    0.775
##     Sp.Richness2      0.845
##     Mean.Rap.Crck2    0.323
```

```
# Generate fit indices
fitMeasures(fit.adj14A, c("agfi", "rmr", "srmr", "rmsea", "cfi", "nnfi", "tli"))
```

```
##  agfi   rmr  srmr rmsea   cfi  nnfi   tli
## 0.840 0.034 0.031 0.111 0.969 0.929 0.929
```

```
# Generate modification indices
mi14adjA <- modindices(fit.adj14A)
print(mi14adjA[mi14adjA$mi > 3.0,])
```

```
##                         lhs op              rhs     mi mi.scaled    epc
## 57           Total.Animals2 ~~ Mean.Raup.Crick2 14.603     5.434 -0.134
## 58             Sp.Richness2 ~~ Mean.Raup.Crick2 10.304     3.835 -0.055
## 61           Total.Animals2  ~      Attendance2  4.985     1.855  0.099
## 62           Total.Animals2  ~ Mean.Raup.Crick2 18.135     6.749 -0.189
## 64           Total.Animals2  ~       X10km_Pop2 14.994     5.580  0.091
## 65           Total.Animals2  ~     Prop.Mam.Sp2  3.589     1.336  0.062
## 67             Sp.Richness2  ~   Total.Animals2  4.049     1.507  0.072
## 68             Sp.Richness2  ~ Mean.Raup.Crick2  3.898     1.451 -0.046
## 72             Sp.Richness2  ~     Zoo.Area.ha2 10.169     3.784  0.067
## 73         Mean.Raup.Crick2  ~      Attendance2  7.319     2.724  0.229
## 74         Mean.Raup.Crick2  ~ Mam.Sp.Richness2 10.304     3.835  0.278
## 75         Mean.Raup.Crick2  ~       X10km_Pop2  6.491     2.416  0.106
## 77         Mean.Raup.Crick2  ~     Zoo.Area.ha2 17.464     6.499  0.222
## 80         Mam.Sp.Richness2  ~     Sp.Richness2  3.963     1.475 -0.437
## 87  Mean.Sp.BodyMassXAbund2  ~      Attendance2 14.662     5.456 -0.356
## 88  Mean.Sp.BodyMassXAbund2  ~   Total.Animals2 19.303     7.183 -0.358
## 89  Mean.Sp.BodyMassXAbund2  ~     Sp.Richness2  9.429     3.509 -0.249
## 90  Mean.Sp.BodyMassXAbund2  ~ Mean.Raup.Crick2 30.161    11.224 -0.494
## 96               X10km_Pop2  ~      Attendance2 21.862     8.136  0.564
## 97               X10km_Pop2  ~   Total.Animals2 13.438     5.001  0.281
## 99               X10km_Pop2  ~ Mean.Raup.Crick2 12.838     4.778  0.178
## 106            GDP.Millions2  ~   Total.Animals2  3.612     1.344  0.290
## 114             Zoo.Area.ha2  ~      Attendance2  8.819     3.282  0.226
## 115             Zoo.Area.ha2  ~   Total.Animals2 18.439     6.862  0.505
## 116             Zoo.Area.ha2  ~     Sp.Richness2 16.851     6.271  0.391
## 117             Zoo.Area.ha2  ~ Mean.Raup.Crick2 14.929     5.556  0.195
## 123             Prop.Mam.Sp2  ~      Attendance2  6.144     2.286  0.189
## 124             Prop.Mam.Sp2  ~   Total.Animals2 12.448     4.632  0.262
## 126             Prop.Mam.Sp2  ~ Mean.Raup.Crick2 16.033     5.966  0.437
##     sepc.lv sepc.all sepc.nox
## 57   -0.134   -0.135   -0.135
## 58   -0.055   -0.055   -0.055
## 61    0.099    0.098    0.098
## 62   -0.189   -0.193   -0.193
## 64    0.091    0.092    0.092
## 65    0.062    0.063    0.063
## 67    0.072    0.072    0.072
## 68   -0.046   -0.046   -0.046
## 72    0.067    0.067    0.067
## 73    0.229    0.221    0.221
## 74    0.278    0.276    0.277
```

```
## 75    0.106    0.105    0.106
## 77    0.222    0.221    0.221
## 80   -0.437   -0.437   -0.437
## 87   -0.356   -0.346   -0.346
## 88   -0.358   -0.353   -0.353
## 89   -0.249   -0.249   -0.249
## 90   -0.494   -0.497   -0.497
## 96    0.564    0.547    0.547
## 97    0.281    0.278    0.278
## 99    0.178    0.179    0.179
## 106   0.290    0.286    0.286
## 114   0.226    0.219    0.219
## 115   0.505    0.499    0.499
## 116   0.391    0.391    0.391
## 117   0.195    0.196    0.196
## 123   0.189    0.184    0.184
## 124   0.262    0.259    0.259
## 126   0.437    0.440    0.440
```

Model Comparisons 5

At this stage we compare the models generated using AICc values. Overall model selection from the pool of competing models is achieved using AICc values, with a threshold of more than 2 AICc units lower than nearest competing model being considered sufficient for model selection.

```
# Model Comparisons using AICc

# Comparing models with and without adjustment for nested nature of data
# library(AICcmodavg)
# source("lavaan.modavg.R")

aictab.lavaan(list(mod.1A.fit, mod.2A.fit, mod.3A.fit, mod.4A.fit, mod.5A.fit, mod.6A.fit, mod.7A.fit, m
              c("mod.1A", "mod.2A", "mod.3A", "mod.4A", "mod.5A", "mod.6A", "mod.7A", "mod.8A",
                "mod.9A", "mod.10A", "mod.11A", "mod.12A", "mod.13A", "mod.14A", "mod.1A.nested",
                "mod.2A.nested", "mod.3A.nested", "mod.4A.nested", "mod.5A.nested", "mod.6A.nested", "mo
```

```
##
## Model selection based on AICc:
##
##                 K     AICc Delta_AICc AICcWt Cum.Wt       LL
## mod.14A        42 10222.15       0.00      1      1 -5068.38
## mod.14A.nested 52 10242.15      20.00      0      1 -5068.38
## mod.13A        50 10823.47     601.32      0      1 -5360.95
## mod.13A.nested 61 10845.47     623.32      0      1 -5360.95
## mod.12A.nested 71 11759.11    1536.96      0      1 -5807.69
## mod.11A.nested 82 12428.03    2205.88      0      1 -6131.05
## mod.10A.nested 83 12425.44    2203.29      0      1 -6128.66
## mod.9A.nested  84 12417.36    2195.21      0      1 -6123.52
## mod.8A.nested  96 13700.23    3478.09      0      1 -6752.85
## mod.7A.nested  94 14397.64    4175.49      0      1 -7102.93
## mod.6A.nested  93 14432.28    4210.13      0      1 -7121.38
## mod.5A.nested  92 14455.87    4233.72      0      1 -7134.31
## mod.4A.nested  91 14492.11    4269.96      0      1 -7153.55
## mod.3A.nested  90 14557.06    4334.91      0      1 -7187.14
```

```
## mod.2A.nested  89 14659.00    4436.85      0      1 -7239.23
## mod.1A.nested  88 15315.69    5093.54      0      1 -7568.68
## mod.12A        59 11735.11    1512.96      0      1 -5807.69
## mod.11A        69 12402.03    2179.88      0      1 -6131.05
## mod.10A        70 12399.44    2177.29      0      1 -6128.66
## mod.9A         71 12391.36    2169.21      0      1 -6123.52
## mod.8A         82 13672.23    3450.09      0      1 -6752.85
## mod.7A         79 14367.64    4145.49      0      1 -7102.93
## mod.6A         78 14402.28    4180.13      0      1 -7121.38
## mod.5A         77 14425.87    4203.72      0      1 -7134.31
## mod.4A         76 14462.11    4239.96      0      1 -7153.55
## mod.3A         75 14527.06    4304.91      0      1 -7187.14
## mod.2A         74 14629.00    4406.85      0      1 -7239.23
## mod.1A         73 15285.69    5063.54      0      1 -7568.68
```

Based on these results we can see that model 14 is the superior model (lowest AICc values), for both models with and without adjustment for nested nature of data.

Model 15

At this stage it was decided to remove the link **Sp.Richness2** ~ **Prop.Mam.Sp2** from the analysis, as it adds little extra information than that provided by **Mam.Sp.Richness2**. Once again, the model summary, fit indices and modification indices were all generated for the model, adjusting for the nested nature of data.

```
# Attendance SEM (Species Abundance)

# Model 15
# Removal of Sp.Richness2 ~ Prop.Mam.Sp2

mod.15A <- 'Attendance2 ~ Sp.Richness2 + Total.Animals2
+ Mam.Sp.Richness2 + Mean.Sp.BodyMassXAbund2 + Mean.Raup.Crick2
+ X10km_Pop2 + GDP.Millions2

Total.Animals2 ~ Zoo.Area.ha2 + Sp.Richness2 + Mean.Sp.BodyMassXAbund2 + GDP.Millions2
Sp.Richness2 ~ Mam.Sp.Richness2
Mean.Raup.Crick2 ~ Sp.Richness2 + Total.Animals2 + Mean.Sp.BodyMassXAbund2'

# Fit model and generate model summary
mod.15A.fit <- sem(mod.15A, data = sem_attendance_data, fixed.x=FALSE)
summary(mod.15A.fit, rsq = TRUE)
```

```
## lavaan (0.5-23.1097) converged normally after  30 iterations
##
##   Number of observations                           458
##
##   Estimator                                         ML
##   Minimum Function Test Statistic              250.898
##   Degrees of freedom                                11
##   P-value (Chi-square)                           0.000
##
## Parameter Estimates:
##
##   Information                                 Expected
##   Standard Errors                             Standard
```

```
##
## Regressions:
##                    Estimate  Std.Err  z-value  P(>|z|)
##   Attendance2 ~
##     Sp.Richness2      -0.184    0.051   -3.599    0.000
##     Total.Animals2     0.587    0.048   12.213    0.000
##     Mam.Sp.Rchnss2     0.102    0.038    2.709    0.007
##     Mn.Sp.BdyMsXA2     0.340    0.029   11.907    0.000
##     Mean.Rap.Crck2     0.125    0.029    4.357    0.000
##     X10km_Pop2         0.444    0.026   17.182    0.000
##     GDP.Millions2      0.163    0.026    6.380    0.000
##   Total.Animals2 ~
##     Zoo.Area.ha2       0.309    0.026   11.697    0.000
##     Sp.Richness2       0.759    0.023   33.251    0.000
##     Mn.Sp.BdyMsXA2    -0.157    0.026   -6.101    0.000
##     GDP.Millions2     -0.136    0.022   -6.199    0.000
##   Sp.Richness2 ~
##     Mam.Sp.Rchnss2     0.729    0.032   22.790    0.000
##   Mean.Raup.Crick2 ~
##     Sp.Richness2      -0.262    0.073   -3.582    0.000
##     Total.Animals2     0.400    0.073    5.452    0.000
##     Mn.Sp.BdyMsXA2    -0.446    0.041  -10.900    0.000
##
## Covariances:
##                            Estimate  Std.Err  z-value  P(>|z|)
##   Mam.Sp.Richness2 ~~
##     Mn.Sp.BdyMsXA2             0.227    0.048    4.750    0.000
##     X10km_Pop2                 0.284    0.048    5.852    0.000
##     GDP.Millions2             -0.060    0.047   -1.277    0.202
##     Zoo.Area.ha2               0.381    0.050    7.634    0.000
##   Mean.Sp.BodyMassXAbund2 ~~
##     X10km_Pop2                 0.021    0.047    0.455    0.649
##     GDP.Millions2             -0.079    0.047   -1.682    0.093
##     Zoo.Area.ha2               0.522    0.053    9.923    0.000
##   X10km_Pop2 ~~
##     GDP.Millions2             -0.028    0.047   -0.604    0.546
##     Zoo.Area.ha2              -0.010    0.047   -0.208    0.835
##   GDP.Millions2 ~~
##     Zoo.Area.ha2              -0.027    0.047   -0.588    0.557
##
## Variances:
##                    Estimate  Std.Err  z-value  P(>|z|)
##    .Attendance2       0.279    0.018   15.133    0.000
##    .Total.Animals2    0.219    0.014   15.133    0.000
##    .Sp.Richness2      0.468    0.031   15.133    0.000
##    .Mean.Rap.Crck2    0.744    0.049   15.133    0.000
##     Mam.Sp.Rchnss2    0.998    0.066   15.133    0.000
##     Mn.Sp.BdyMsXA2    0.998    0.066   15.133    0.000
##     X10km_Pop2        0.998    0.066   15.133    0.000
##     GDP.Millions2     0.998    0.066   15.133    0.000
##     Zoo.Area.ha2      0.998    0.066   15.133    0.000
##
## R-Square:
##                    Estimate
```

```
##     Attendance2       0.706
##     Total.Animals2    0.776
##     Sp.Richness2      0.531
##     Mean.Rap.Crck2    0.244
```

```r
# Generate fit indices
fitMeasures(mod.15A.fit, c("agfi", "rmr", "srmr", "rmsea", "cfi", "nnfi", "tli"))
```

```
##  agfi   rmr  srmr rmsea   cfi  nnfi   tli
## 0.626 0.094 0.095 0.218 0.879 0.714 0.714
```

```r
# Generate modification indices
mi15A <- modindices(mod.15A.fit)
print(mi15A[mi15A$mi > 3.0,])
```

```
##                       lhs op                   rhs      mi    epc
## 39         Total.Animals2 ~~       Mean.Raup.Crick2   8.543 -0.106
## 42         Total.Animals2  ~            Attendance2   5.296  0.097
## 43         Total.Animals2  ~       Mean.Raup.Crick2   8.543 -0.142
## 45         Total.Animals2  ~             X10km_Pop2  13.645  0.083
## 46           Sp.Richness2  ~            Attendance2   3.275 -0.105
## 48           Sp.Richness2  ~       Mean.Raup.Crick2  55.507  0.307
## 49           Sp.Richness2  ~ Mean.Sp.BodyMassXAbund2 137.040 -0.385
## 50           Sp.Richness2  ~             X10km_Pop2  15.334  0.131
## 51           Sp.Richness2  ~          GDP.Millions2   8.442  0.093
## 52           Sp.Richness2  ~           Zoo.Area.ha2  12.709 -0.123
## 53       Mean.Raup.Crick2  ~            Attendance2   8.687  0.250
## 55       Mean.Raup.Crick2  ~             X10km_Pop2  10.624  0.134
## 57       Mean.Raup.Crick2  ~           Zoo.Area.ha2  11.424  0.185
## 61       Mam.Sp.Richness2  ~       Mean.Raup.Crick2  14.834 -0.236
## 66 Mean.Sp.BodyMassXAbund2  ~            Attendance2  74.334 -1.160
## 67 Mean.Sp.BodyMassXAbund2  ~         Total.Animals2 129.532 -0.869
## 68 Mean.Sp.BodyMassXAbund2  ~           Sp.Richness2 127.666 -0.655
## 69 Mean.Sp.BodyMassXAbund2  ~       Mean.Raup.Crick2  18.690 -0.447
## 74             X10km_Pop2  ~            Attendance2  36.668  0.725
## 75             X10km_Pop2  ~         Total.Animals2  26.436  0.326
## 76             X10km_Pop2  ~           Sp.Richness2  13.943  0.242
## 77             X10km_Pop2  ~       Mean.Raup.Crick2  23.002  0.241
## 82          GDP.Millions2  ~            Attendance2   3.949  0.438
## 83          GDP.Millions2  ~         Total.Animals2   4.667  0.193
## 84          GDP.Millions2  ~           Sp.Richness2   4.370  0.142
## 90           Zoo.Area.ha2  ~            Attendance2   9.316  0.221
## 91           Zoo.Area.ha2  ~         Total.Animals2   8.445  0.204
## 92           Zoo.Area.ha2  ~           Sp.Richness2   7.856  0.153
## 93           Zoo.Area.ha2  ~       Mean.Raup.Crick2  19.427  0.214
##    sepc.lv sepc.all sepc.nox
## 39  -0.106   -0.108   -0.108
## 42   0.097    0.096    0.096
## 43  -0.142   -0.142   -0.142
## 45   0.083    0.084    0.084
## 46  -0.105   -0.103   -0.103
## 48   0.307    0.305    0.305
## 49  -0.385   -0.385   -0.385
## 50   0.131    0.131    0.131
## 51   0.093    0.093    0.093
## 52  -0.123   -0.123   -0.124
```

```
## 53   0.250    0.246    0.246
## 55   0.134    0.135    0.136
## 57   0.185    0.186    0.187
## 61  -0.236   -0.234   -0.234
## 66  -1.160   -1.133   -1.133
## 67  -0.869   -0.861   -0.861
## 68  -0.655   -0.655   -0.655
## 69  -0.447   -0.444   -0.444
## 74   0.725    0.708    0.708
## 75   0.326    0.323    0.323
## 76   0.242    0.242    0.242
## 77   0.241    0.239    0.239
## 82   0.438    0.428    0.428
## 83   0.193    0.192    0.192
## 84   0.142    0.142    0.142
## 90   0.221    0.216    0.216
## 91   0.204    0.202    0.202
## 92   0.153    0.153    0.153
## 93   0.214    0.212    0.212
```

```
# Adjust for the nested nature of the data (institutions within countries)
# Fit model and generate model summary
design <- svydesign(ids = ~Country, nest=TRUE, data=sem_attendance_data)
fit.adj15A <- lavaan.survey(lavaan.fit = mod.15A.fit, survey.design = design)
summary(fit.adj15A, rsq = TRUE)
```

```
## lavaan (0.5-23.1097) converged normally after  30 iterations
##
##   Number of observations                           458
##
##   Estimator                                         ML      Robust
##   Minimum Function Test Statistic              250.898      76.682
##   Degrees of freedom                                11          11
##   P-value (Chi-square)                           0.000       0.000
##   Scaling correction factor                                  3.272
##     for the Satorra-Bentler correction
##
## Parameter Estimates:
##
##   Information                                 Expected
##   Standard Errors                           Robust.sem
##
## Regressions:
##                      Estimate  Std.Err  z-value  P(>|z|)
##   Attendance2 ~
##     Sp.Richness2       -0.184    0.060   -3.076    0.002
##     Total.Animals2      0.587    0.046   12.883    0.000
##     Mam.Sp.Rchnss2      0.102    0.039    2.609    0.009
##     Mn.Sp.BdyMsXA2      0.340    0.027   12.544    0.000
##     Mean.Rap.Crck2      0.125    0.033    3.786    0.000
##     X10km_Pop2          0.444    0.034   13.075    0.000
##     GDP.Millions2       0.163    0.027    6.120    0.000
##   Total.Animals2 ~
##     Zoo.Area.ha2        0.309    0.040    7.759    0.000
##     Sp.Richness2        0.759    0.053   14.226    0.000
```

```
##     Mn.Sp.BdyMsXA2     -0.157    0.037   -4.283    0.000
##     GDP.Millions2      -0.136    0.069   -1.988    0.047
##   Sp.Richness2 ~
##     Mam.Sp.Rchnss2      0.729    0.064   11.465    0.000
##   Mean.Raup.Crick2 ~
##     Sp.Richness2       -0.262    0.243   -1.076    0.282
##     Total.Animals2      0.400    0.159    2.514    0.012
##     Mn.Sp.BdyMsXA2     -0.446    0.082   -5.446    0.000
##
## Covariances:
##                             Estimate  Std.Err  z-value  P(>|z|)
##   Mam.Sp.Richness2 ~~
##     Mn.Sp.BdyMsXA2             0.227    0.078    2.921    0.003
##     X10km_Pop2                 0.284    0.066    4.278    0.000
##     GDP.Millions2             -0.060    0.064   -0.937    0.349
##     Zoo.Area.ha2               0.381    0.064    5.985    0.000
##   Mean.Sp.BodyMassXAbund2 ~~
##     X10km_Pop2                 0.021    0.064    0.329    0.742
##     GDP.Millions2             -0.079    0.038   -2.055    0.040
##     Zoo.Area.ha2               0.522    0.101    5.177    0.000
##   X10km_Pop2 ~~
##     GDP.Millions2             -0.028    0.056   -0.505    0.613
##     Zoo.Area.ha2              -0.010    0.078   -0.124    0.901
##   GDP.Millions2 ~~
##     Zoo.Area.ha2              -0.027    0.041   -0.673    0.501
##
## Intercepts:
##                    Estimate  Std.Err  z-value  P(>|z|)
##    .Attendance2      -0.000    0.029   -0.000    1.000
##    .Total.Animals2   -0.000    0.067   -0.000    1.000
##    .Sp.Richness2      0.000    0.088    0.000    1.000
##    .Mean.Rap.Crck2    0.000    0.094    0.000    1.000
##     Mam.Sp.Rchnss2   -0.000    0.079   -0.000    1.000
##     Mn.Sp.BdyMsXA2    0.000    0.071    0.000    1.000
##     X10km_Pop2        0.000    0.105    0.000    1.000
##     GDP.Millions2    -0.000    0.345   -0.000    1.000
##     Zoo.Area.ha2      0.000    0.064    0.000    1.000
##
## Variances:
##                    Estimate  Std.Err  z-value  P(>|z|)
##    .Attendance2       0.279    0.028   10.018    0.000
##    .Total.Animals2    0.219    0.037    5.986    0.000
##    .Sp.Richness2      0.468    0.066    7.056    0.000
##    .Mean.Rap.Crck2    0.744    0.068   10.938    0.000
##     Mam.Sp.Rchnss2    0.998    0.115    8.669    0.000
##     Mn.Sp.BdyMsXA2    0.998    0.160    6.219    0.000
##     X10km_Pop2        0.998    0.174    5.745    0.000
##     GDP.Millions2     0.998    0.246    4.061    0.000
##     Zoo.Area.ha2      0.998    0.112    8.886    0.000
##
## R-Square:
##                    Estimate
##     Attendance2       0.706
##     Total.Animals2    0.776
```

```
##     Sp.Richness2      0.531
##     Mean.Rap.Crck2    0.244
```

```r
# Generate fit indices
fitMeasures(fit.adj15A, c("agfi", "rmr", "srmr", "rmsea", "cfi", "nnfi", "tli"))
```

```
##  agfi   rmr  srmr rmsea   cfi  nnfi   tli
## 0.551 0.094 0.086 0.218 0.879 0.714 0.714
```

```r
# Generate modification indices
mi15adjA <- modindices(fit.adj15A)
print(mi15adjA[mi15adjA$mi > 3.0,])
```

```
##                          lhs op                      rhs      mi mi.scaled
## 48           Total.Animals2 ~~        Mean.Raup.Crick2   8.543     2.611
## 51           Total.Animals2  ~             Attendance2   5.296     1.619
## 52           Total.Animals2  ~        Mean.Raup.Crick2   8.543     2.611
## 54           Total.Animals2  ~              X10km_Pop2  13.645     4.170
## 55             Sp.Richness2  ~             Attendance2   3.275     1.001
## 57             Sp.Richness2  ~        Mean.Raup.Crick2  55.507    16.965
## 58             Sp.Richness2  ~ Mean.Sp.BodyMassXAbund2 137.040    41.883
## 59             Sp.Richness2  ~              X10km_Pop2  15.334     4.686
## 60             Sp.Richness2  ~            GDP.Millions2   8.442     2.580
## 61             Sp.Richness2  ~             Zoo.Area.ha2  12.709     3.884
## 62         Mean.Raup.Crick2  ~             Attendance2   8.687     2.655
## 64         Mean.Raup.Crick2  ~              X10km_Pop2  10.624     3.247
## 66         Mean.Raup.Crick2  ~             Zoo.Area.ha2  11.424     3.491
## 70         Mam.Sp.Richness2  ~        Mean.Raup.Crick2  14.834     4.534
## 75  Mean.Sp.BodyMassXAbund2  ~             Attendance2  74.334    22.719
## 76  Mean.Sp.BodyMassXAbund2  ~          Total.Animals2 129.532    39.589
## 77  Mean.Sp.BodyMassXAbund2  ~            Sp.Richness2 127.666    39.018
## 78  Mean.Sp.BodyMassXAbund2  ~        Mean.Raup.Crick2  18.690     5.712
## 83               X10km_Pop2  ~             Attendance2  36.668    11.207
## 84               X10km_Pop2  ~          Total.Animals2  26.436     8.080
## 85               X10km_Pop2  ~            Sp.Richness2  13.943     4.262
## 86               X10km_Pop2  ~        Mean.Raup.Crick2  23.002     7.030
## 91            GDP.Millions2  ~             Attendance2   3.949     1.207
## 92            GDP.Millions2  ~          Total.Animals2   4.667     1.426
## 93            GDP.Millions2  ~            Sp.Richness2   4.370     1.336
## 99             Zoo.Area.ha2  ~             Attendance2   9.316     2.847
## 100            Zoo.Area.ha2  ~          Total.Animals2   8.445     2.581
## 101            Zoo.Area.ha2  ~            Sp.Richness2   7.856     2.401
## 102            Zoo.Area.ha2  ~        Mean.Raup.Crick2  19.427     5.938
##        epc sepc.lv sepc.all sepc.nox
## 48  -0.106  -0.106   -0.108   -0.108
## 51   0.097   0.097    0.096    0.096
## 52  -0.142  -0.142   -0.142   -0.142
## 54   0.083   0.083    0.084    0.084
## 55  -0.105  -0.105   -0.103   -0.103
## 57   0.307   0.307    0.305    0.305
## 58  -0.385  -0.385   -0.385   -0.385
## 59   0.131   0.131    0.131    0.131
## 60   0.093   0.093    0.093    0.093
## 61  -0.123  -0.123   -0.123   -0.124
## 62   0.250   0.250    0.246    0.246
## 64   0.134   0.134    0.135    0.136
```

```
## 66   0.185   0.185    0.186    0.187
## 70  -0.236  -0.236   -0.234   -0.234
## 75  -1.160  -1.160   -1.133   -1.133
## 76  -0.869  -0.869   -0.861   -0.861
## 77  -0.655  -0.655   -0.655   -0.655
## 78  -0.447  -0.447   -0.444   -0.444
## 83   0.725   0.725    0.708    0.708
## 84   0.326   0.326    0.323    0.323
## 85   0.242   0.242    0.242    0.242
## 86   0.241   0.241    0.239    0.239
## 91   0.438   0.438    0.428    0.428
## 92   0.193   0.193    0.192    0.192
## 93   0.142   0.142    0.142    0.142
## 99   0.221   0.221    0.216    0.216
## 100  0.204   0.204    0.202    0.202
## 101  0.153   0.153    0.153    0.153
## 102  0.214   0.214    0.212    0.212
```

Model 16

Based on the modification indices generated from the fifteenth model, we can see that **Sp.Richness2** ~ **Mean.Sp.BodyMassXAbund2** has the highest mi value of **137.040**. This far exceeds the standard cut-off level for the chi-square test criterion of 3.84 (Burnham and Anderson, 2002). This is also one of the most intuitive relationships within the system. There is clear evidence within the literature that zoological institutions have a limited carrying capacity and that large vertebrates, by their very nature, require ever larger enclosure sizes. Therefore it is deducible that institutions with larger vertebrates will be able to hold fewer species. This semi-exploratory modelling approach is similar to that implemented in Grace *et al.*, 2016. As a result, we add this relationship to our model. Once again, the model summary, fit indices and modification indices were all generated for the model, adjusting for the nested nature of data.

```
# Attendance SEM (Species Abundance)

# Model 16
# Addition of Sp.Richness2 ~ Mean.Sp.BodyMassXAbund2, mi = 137.040

mod.16A <- 'Attendance2 ~ Sp.Richness2 + Total.Animals2
+ Mam.Sp.Richness2 + Mean.Sp.BodyMassXAbund2 + Mean.Raup.Crick2
+ X10km_Pop2 + GDP.Millions2

Total.Animals2 ~ Zoo.Area.ha2 + Sp.Richness2 + Mean.Sp.BodyMassXAbund2 + GDP.Millions2
Sp.Richness2 ~ Mam.Sp.Richness2 + Mean.Sp.BodyMassXAbund2
Mean.Raup.Crick2 ~ Sp.Richness2 + Total.Animals2 + Mean.Sp.BodyMassXAbund2'

# Fit model and generate model summary
mod.16A.fit <- sem(mod.16A, data = sem_attendance_data, fixed.x=FALSE)
summary(mod.16A.fit, rsq = TRUE)
```

```
## lavaan (0.5-23.1097) converged normally after  30 iterations
##
##   Number of observations                           458
##
##   Estimator                                         ML
##   Minimum Function Test Statistic               88.056
##   Degrees of freedom                                10
```

```
##   P-value (Chi-square)                          0.000
##
## Parameter Estimates:
##
##   Information                                Expected
##   Standard Errors                            Standard
##
## Regressions:
##                      Estimate  Std.Err  z-value  P(>|z|)
##   Attendance2 ~
##     Sp.Richness2       -0.184    0.056   -3.268    0.001
##     Total.Animals2      0.587    0.048   12.213    0.000
##     Mam.Sp.Rchnss2      0.102    0.044    2.301    0.021
##     Mn.Sp.BdyMsXA2      0.340    0.033   10.291    0.000
##     Mean.Rap.Crck2      0.125    0.029    4.357    0.000
##     X10km_Pop2          0.444    0.026   17.182    0.000
##     GDP.Millions2       0.163    0.026    6.380    0.000
##   Total.Animals2 ~
##     Zoo.Area.ha2        0.309    0.027   11.609    0.000
##     Sp.Richness2        0.759    0.023   32.794    0.000
##     Mn.Sp.BdyMsXA2     -0.157    0.027   -5.811    0.000
##     GDP.Millions2      -0.136    0.022   -6.197    0.000
##   Sp.Richness2 ~
##     Mam.Sp.Rchnss2      0.817    0.027   29.692    0.000
##     Mn.Sp.BdyMsXA2     -0.385    0.027  -13.984    0.000
##   Mean.Raup.Crick2 ~
##     Sp.Richness2       -0.262    0.074   -3.545    0.000
##     Total.Animals2      0.400    0.074    5.442    0.000
##     Mn.Sp.BdyMsXA2     -0.446    0.041  -10.818    0.000
##
## Covariances:
##                              Estimate  Std.Err  z-value  P(>|z|)
##   Mam.Sp.Richness2 ~~
##     Mn.Sp.BdyMsXA2              0.227    0.048    4.750    0.000
##     X10km_Pop2                  0.284    0.048    5.852    0.000
##     GDP.Millions2              -0.060    0.047   -1.277    0.202
##     Zoo.Area.ha2                0.381    0.050    7.634    0.000
##   Mean.Sp.BodyMassXAbund2 ~~
##     X10km_Pop2                  0.021    0.047    0.455    0.649
##     GDP.Millions2              -0.079    0.047   -1.682    0.093
##     Zoo.Area.ha2                0.522    0.053    9.923    0.000
##   X10km_Pop2 ~~
##     GDP.Millions2              -0.028    0.047   -0.604    0.546
##     Zoo.Area.ha2               -0.010    0.047   -0.208    0.835
##   GDP.Millions2 ~~
##     Zoo.Area.ha2               -0.027    0.047   -0.588    0.557
##
## Variances:
##                    Estimate  Std.Err  z-value  P(>|z|)
##    .Attendance2       0.279    0.018   15.133    0.000
##    .Total.Animals2    0.219    0.014   15.133    0.000
##    .Sp.Richness2      0.328    0.022   15.133    0.000
##    .Mean.Rap.Crck2    0.744    0.049   15.133    0.000
##     Mam.Sp.Rchnss2    0.998    0.066   15.133    0.000
```

```
##     Mn.Sp.BdyMsXA2    0.998    0.066   15.133    0.000
##     X10km_Pop2        0.998    0.066   15.133    0.000
##     GDP.Millions2     0.998    0.066   15.133    0.000
##     Zoo.Area.ha2      0.998    0.066   15.133    0.000
##
## R-Square:
##                    Estimate
##     Attendance2       0.691
##     Total.Animals2    0.777
##     Sp.Richness2      0.672
##     Mean.Rap.Crck2    0.254
```

```
# Generate fit indices
fitMeasures(mod.16A.fit, c("agfi", "rmr", "srmr", "rmsea", "cfi", "nnfi", "tli"))
```

```
##  agfi   rmr  srmr rmsea   cfi  nnfi   tli
## 0.829 0.053 0.053 0.131 0.961 0.898 0.898
```

```
# Generate modification indices
mi16A <- modindices(mod.16A.fit)
print(mi16A[mi16A$mi > 3.0,])
```

```
##                        lhs op              rhs     mi    epc sepc.lv
## 40          Total.Animals2 ~~ Mean.Raup.Crick2  8.628 -0.107  -0.107
## 43          Total.Animals2  ~      Attendance2  5.451  0.100   0.100
## 44          Total.Animals2  ~ Mean.Raup.Crick2  8.628 -0.143  -0.143
## 46          Total.Animals2  ~       X10km_Pop2 13.844  0.084   0.084
## 47            Sp.Richness2  ~      Attendance2 18.267  0.225   0.225
## 50            Sp.Richness2  ~       X10km_Pop2 16.251  0.113   0.113
## 51            Sp.Richness2  ~    GDP.Millions2  6.442  0.068   0.068
## 52            Sp.Richness2  ~     Zoo.Area.ha2  8.322  0.096   0.096
## 53        Mean.Raup.Crick2  ~      Attendance2  8.973  0.258   0.258
## 55        Mean.Raup.Crick2  ~       X10km_Pop2 10.759  0.136   0.136
## 57        Mean.Raup.Crick2  ~     Zoo.Area.ha2 11.553  0.187   0.187
## 58        Mam.Sp.Richness2  ~      Attendance2 12.324 -0.533  -0.533
## 59        Mam.Sp.Richness2  ~   Total.Animals2  9.187 -0.345  -0.345
## 60        Mam.Sp.Richness2  ~     Sp.Richness2 24.069 -0.807  -0.807
## 61        Mam.Sp.Richness2  ~ Mean.Raup.Crick2 19.683 -0.305  -0.305
## 66 Mean.Sp.BodyMassXAbund2  ~      Attendance2  7.823 -0.456  -0.456
## 67 Mean.Sp.BodyMassXAbund2  ~   Total.Animals2  8.228 -0.534  -0.534
## 68 Mean.Sp.BodyMassXAbund2  ~     Sp.Richness2  7.091 -0.377  -0.377
## 69 Mean.Sp.BodyMassXAbund2  ~ Mean.Raup.Crick2 12.931 -0.376  -0.376
## 74              X10km_Pop2  ~      Attendance2 39.533  0.782   0.782
## 75              X10km_Pop2  ~   Total.Animals2 31.658  0.391   0.391
## 76              X10km_Pop2  ~     Sp.Richness2 19.897  0.345   0.345
## 77              X10km_Pop2  ~ Mean.Raup.Crick2 23.010  0.241   0.241
## 82           GDP.Millions2  ~      Attendance2  5.093  0.565   0.565
## 83           GDP.Millions2  ~   Total.Animals2  6.660  0.276   0.276
## 84           GDP.Millions2  ~     Sp.Richness2  6.237  0.203   0.203
## 90            Zoo.Area.ha2  ~      Attendance2  9.724  0.231   0.231
## 91            Zoo.Area.ha2  ~   Total.Animals2 11.974  0.289   0.289
## 92            Zoo.Area.ha2  ~     Sp.Richness2 11.211  0.219   0.219
## 93            Zoo.Area.ha2  ~ Mean.Raup.Crick2 19.470  0.214   0.214
##    sepc.all sepc.nox
## 40   -0.108   -0.108
## 43    0.096    0.096
```

```
## 44   -0.144   -0.144
## 46    0.085    0.085
## 47    0.214    0.214
## 50    0.113    0.113
## 51    0.068    0.068
## 52    0.096    0.096
## 53    0.245    0.245
## 55    0.136    0.136
## 57    0.187    0.187
## 58   -0.507   -0.507
## 59   -0.342   -0.342
## 60   -0.807   -0.807
## 61   -0.305   -0.305
## 66   -0.434   -0.434
## 67   -0.530   -0.530
## 68   -0.377   -0.377
## 69   -0.376   -0.376
## 74    0.744    0.744
## 75    0.388    0.388
## 76    0.345    0.345
## 77    0.241    0.241
## 82    0.538    0.538
## 83    0.274    0.274
## 84    0.203    0.203
## 90    0.219    0.219
## 91    0.287    0.287
## 92    0.219    0.219
## 93    0.214    0.214
```

```r
# Adjust for the nested nature of the data (institutions within countries)
# Fit model and generate model summary
design <- svydesign(ids = ~Country, nest=TRUE, data=sem_attendance_data)
fit.adj16A <- lavaan.survey(lavaan.fit = mod.16A.fit, survey.design = design)
summary(fit.adj16A, rsq = TRUE)
```

```
## lavaan (0.5-23.1097) converged normally after  30 iterations
##
##   Number of observations                           458
##
##   Estimator                                         ML      Robust
##   Minimum Function Test Statistic               88.056      30.782
##   Degrees of freedom                                10          10
##   P-value (Chi-square)                           0.000       0.001
##   Scaling correction factor                                  2.861
##     for the Satorra-Bentler correction
##
## Parameter Estimates:
##
##   Information                                 Expected
##   Standard Errors                           Robust.sem
##
## Regressions:
##                    Estimate  Std.Err  z-value  P(>|z|)
##   Attendance2 ~
##     Sp.Richness2     -0.184    0.063   -2.945    0.003
```

```
##     Total.Animals2      0.587    0.046   12.883    0.000
##     Mam.Sp.Rchnss2      0.102    0.043    2.354    0.019
##     Mn.Sp.BdyMsXA2      0.340    0.030   11.353    0.000
##     Mean.Rap.Crck2      0.125    0.033    3.786    0.000
##     X10km_Pop2          0.444    0.034   13.075    0.000
##     GDP.Millions2       0.163    0.027    6.120    0.000
##   Total.Animals2 ~
##     Zoo.Area.ha2        0.309    0.040    7.641    0.000
##     Sp.Richness2        0.759    0.047   16.099    0.000
##     Mn.Sp.BdyMsXA2     -0.157    0.035   -4.513    0.000
##     GDP.Millions2      -0.136    0.069   -1.987    0.047
##   Sp.Richness2 ~
##     Mam.Sp.Rchnss2      0.817    0.060   13.721    0.000
##     Mn.Sp.BdyMsXA2     -0.385    0.043   -8.893    0.000
##   Mean.Raup.Crick2 ~
##     Sp.Richness2       -0.262    0.230   -1.138    0.255
##     Total.Animals2      0.400    0.159    2.515    0.012
##     Mn.Sp.BdyMsXA2     -0.446    0.059   -7.595    0.000
##
## Covariances:
##                             Estimate  Std.Err  z-value  P(>|z|)
##   Mam.Sp.Richness2 ~~
##     Mn.Sp.BdyMsXA2             0.227    0.078    2.921    0.003
##     X10km_Pop2                 0.284    0.066    4.278    0.000
##     GDP.Millions2             -0.060    0.064   -0.937    0.349
##     Zoo.Area.ha2               0.381    0.064    5.985    0.000
##   Mean.Sp.BodyMassXAbund2 ~~
##     X10km_Pop2                 0.021    0.064    0.329    0.742
##     GDP.Millions2             -0.079    0.038   -2.055    0.040
##     Zoo.Area.ha2               0.522    0.101    5.177    0.000
##   X10km_Pop2 ~~
##     GDP.Millions2             -0.028    0.056   -0.505    0.613
##     Zoo.Area.ha2              -0.010    0.078   -0.124    0.901
##   GDP.Millions2 ~~
##     Zoo.Area.ha2              -0.027    0.041   -0.673    0.501
##
## Intercepts:
##                    Estimate  Std.Err  z-value  P(>|z|)
##    .Attendance2      -0.000    0.029   -0.000    1.000
##    .Total.Animals2   -0.000    0.067   -0.000    1.000
##    .Sp.Richness2      0.000    0.073    0.000    1.000
##    .Mean.Rap.Crck2    0.000    0.094    0.000    1.000
##     Mam.Sp.Rchnss2   -0.000    0.079   -0.000    1.000
##     Mn.Sp.BdyMsXA2    0.000    0.071    0.000    1.000
##     X10km_Pop2        0.000    0.105    0.000    1.000
##     GDP.Millions2    -0.000    0.345   -0.000    1.000
##     Zoo.Area.ha2      0.000    0.064    0.000    1.000
##
## Variances:
##                    Estimate  Std.Err  z-value  P(>|z|)
##    .Attendance2       0.279    0.028   10.018    0.000
##    .Total.Animals2    0.219    0.037    5.986    0.000
##    .Sp.Richness2      0.328    0.033   10.049    0.000
##    .Mean.Rap.Crck2    0.744    0.068   10.938    0.000
```

```
##     Mam.Sp.Rchnss2    0.998    0.115    8.669    0.000
##     Mn.Sp.BdyMsXA2    0.998    0.160    6.219    0.000
##     X10km_Pop2        0.998    0.174    5.745    0.000
##     GDP.Millions2     0.998    0.246    4.061    0.000
##     Zoo.Area.ha2      0.998    0.112    8.886    0.000
##
## R-Square:
##                    Estimate
##     Attendance2       0.691
##     Total.Animals2    0.777
##     Sp.Richness2      0.672
##     Mean.Rap.Crck2    0.254
```

```
# Generate fit indices
fitMeasures(fit.adj16A, c("agfi", "rmr", "srmr", "rmsea", "cfi", "nnfi", "tli"))
```

```
##  agfi   rmr  srmr rmsea   cfi  nnfi   tli
## 0.795 0.053 0.048 0.131 0.961 0.898 0.898
```

```
# Generate modification indices
mi16adjA <- modindices(fit.adj16A)
print(mi16adjA[mi16adjA$mi > 3.0,])
```

```
##                          lhs op              rhs     mi mi.scaled    epc
## 49            Total.Animals2 ~~ Mean.Raup.Crick2  8.628     3.016 -0.107
## 52            Total.Animals2  ~      Attendance2  5.451     1.905  0.100
## 53            Total.Animals2  ~ Mean.Raup.Crick2  8.628     3.016 -0.143
## 55            Total.Animals2  ~       X10km_Pop2 13.844     4.840  0.084
## 56              Sp.Richness2  ~      Attendance2 18.267     6.386  0.225
## 59              Sp.Richness2  ~       X10km_Pop2 16.251     5.681  0.113
## 60              Sp.Richness2  ~    GDP.Millions2  6.442     2.252  0.068
## 61              Sp.Richness2  ~     Zoo.Area.ha2  8.322     2.909  0.096
## 62          Mean.Raup.Crick2  ~      Attendance2  8.973     3.137  0.258
## 64          Mean.Raup.Crick2  ~       X10km_Pop2 10.759     3.761  0.136
## 66          Mean.Raup.Crick2  ~     Zoo.Area.ha2 11.553     4.039  0.187
## 67          Mam.Sp.Richness2  ~      Attendance2 12.324     4.308 -0.533
## 68          Mam.Sp.Richness2  ~   Total.Animals2  9.187     3.212 -0.345
## 69          Mam.Sp.Richness2  ~     Sp.Richness2 24.069     8.414 -0.807
## 70          Mam.Sp.Richness2  ~ Mean.Raup.Crick2 19.683     6.881 -0.305
## 75  Mean.Sp.BodyMassXAbund2  ~      Attendance2  7.823     2.735 -0.456
## 76  Mean.Sp.BodyMassXAbund2  ~   Total.Animals2  8.228     2.876 -0.534
## 77  Mean.Sp.BodyMassXAbund2  ~     Sp.Richness2  7.091     2.479 -0.377
## 78  Mean.Sp.BodyMassXAbund2  ~ Mean.Raup.Crick2 12.931     4.520 -0.376
## 83                X10km_Pop2  ~      Attendance2 39.533    13.820  0.782
## 84                X10km_Pop2  ~   Total.Animals2 31.658    11.067  0.391
## 85                X10km_Pop2  ~     Sp.Richness2 19.897     6.956  0.345
## 86                X10km_Pop2  ~ Mean.Raup.Crick2 23.010     8.044  0.241
## 91             GDP.Millions2  ~      Attendance2  5.093     1.780  0.565
## 92             GDP.Millions2  ~   Total.Animals2  6.660     2.328  0.276
## 93             GDP.Millions2  ~     Sp.Richness2  6.237     2.180  0.203
## 99              Zoo.Area.ha2  ~      Attendance2  9.724     3.399  0.231
## 100             Zoo.Area.ha2  ~   Total.Animals2 11.974     4.186  0.289
## 101             Zoo.Area.ha2  ~     Sp.Richness2 11.211     3.919  0.219
## 102             Zoo.Area.ha2  ~ Mean.Raup.Crick2 19.470     6.806  0.214
##     sepc.lv sepc.all sepc.nox
## 49   -0.107   -0.108   -0.108
```

```
## 52    0.100    0.096    0.096
## 53   -0.143   -0.144   -0.144
## 55    0.084    0.085    0.085
## 56    0.225    0.214    0.214
## 59    0.113    0.113    0.113
## 60    0.068    0.068    0.068
## 61    0.096    0.096    0.096
## 62    0.258    0.245    0.245
## 64    0.136    0.136    0.136
## 66    0.187    0.187    0.187
## 67   -0.533   -0.507   -0.507
## 68   -0.345   -0.342   -0.342
## 69   -0.807   -0.807   -0.807
## 70   -0.305   -0.305   -0.305
## 75   -0.456   -0.434   -0.434
## 76   -0.534   -0.530   -0.530
## 77   -0.377   -0.377   -0.377
## 78   -0.376   -0.376   -0.376
## 83    0.782    0.744    0.744
## 84    0.391    0.388    0.388
## 85    0.345    0.345    0.345
## 86    0.241    0.241    0.241
## 91    0.565    0.538    0.538
## 92    0.276    0.274    0.274
## 93    0.203    0.203    0.203
## 99    0.231    0.219    0.219
## 100   0.289    0.287    0.287
## 101   0.219    0.219    0.219
## 102   0.214    0.214    0.214
```

Model 17

Based on the results generated from the nested sixteenth model, the highest p-value relationship to be considered for removal is **Mean.Raup.Crick2 ~ Sp.Richness2** with a p-value of **0.255**. As a result, we remove this relationship from our model. Once again, the model summary, fit indices and modification indices were all generated for the model, adjusting for the nested nature of data.

```
# Attendance SEM (Species Abundance)

# Model 17
# Removal of Mean.Raup.Crick2 ~ Sp.Richness2, p = 0.255

mod.17A <- 'Attendance2 ~ Sp.Richness2 + Total.Animals2
+ Mam.Sp.Richness2 + Mean.Sp.BodyMassXAbund2 + Mean.Raup.Crick2
+ X10km_Pop2 + GDP.Millions2

Total.Animals2 ~ Zoo.Area.ha2 + Sp.Richness2 + Mean.Sp.BodyMassXAbund2 + GDP.Millions2
Sp.Richness2 ~ Mam.Sp.Richness2 + Mean.Sp.BodyMassXAbund2
Mean.Raup.Crick2 ~ Total.Animals2 + Mean.Sp.BodyMassXAbund2'

# Fit model and generate model summary
mod.17A.fit <- sem(mod.17A, data = sem_attendance_data, fixed.x=FALSE)
summary(mod.17A.fit, rsq = TRUE)
```

```
## lavaan (0.5-23.1097) converged normally after  27 iterations
##
##   Number of observations                           458
##
##   Estimator                                         ML
##   Minimum Function Test Statistic              100.196
##   Degrees of freedom                                11
##   P-value (Chi-square)                           0.000
##
## Parameter Estimates:
##
##   Information                                 Expected
##   Standard Errors                             Standard
##
## Regressions:
##                      Estimate  Std.Err  z-value  P(>|z|)
##   Attendance2 ~
##     Sp.Richness2       -0.184    0.056   -3.297    0.001
##     Total.Animals2      0.587    0.047   12.499    0.000
##     Mam.Sp.Rchnss2      0.102    0.044    2.301    0.021
##     Mn.Sp.BdyMsXA2      0.340    0.033   10.387    0.000
##     Mean.Rap.Crck2      0.125    0.028    4.415    0.000
##     X10km_Pop2          0.444    0.026   17.182    0.000
##     GDP.Millions2       0.163    0.026    6.380    0.000
##   Total.Animals2 ~
##     Zoo.Area.ha2        0.309    0.027   11.609    0.000
##     Sp.Richness2        0.759    0.023   32.795    0.000
##     Mn.Sp.BdyMsXA2     -0.157    0.027   -5.812    0.000
##     GDP.Millions2      -0.136    0.022   -6.197    0.000
##   Sp.Richness2 ~
##     Mam.Sp.Rchnss2      0.817    0.027   29.692    0.000
##     Mn.Sp.BdyMsXA2     -0.385    0.027  -13.984    0.000
##   Mean.Raup.Crick2 ~
##     Total.Animals2      0.185    0.042    4.439    0.000
##     Mn.Sp.BdyMsXA2     -0.423    0.041  -10.255    0.000
##
## Covariances:
##                              Estimate  Std.Err  z-value  P(>|z|)
##   Mam.Sp.Richness2 ~~
##     Mn.Sp.BdyMsXA2              0.227    0.048    4.750    0.000
##     X10km_Pop2                  0.284    0.048    5.852    0.000
##     GDP.Millions2              -0.060    0.047   -1.277    0.202
##     Zoo.Area.ha2                0.381    0.050    7.634    0.000
##   Mean.Sp.BodyMassXAbund2 ~~
##     X10km_Pop2                  0.021    0.047    0.455    0.649
##     GDP.Millions2              -0.079    0.047   -1.682    0.093
##     Zoo.Area.ha2                0.522    0.053    9.923    0.000
##   X10km_Pop2 ~~
##     GDP.Millions2              -0.028    0.047   -0.604    0.546
##     Zoo.Area.ha2               -0.010    0.047   -0.208    0.835
##   GDP.Millions2 ~~
##     Zoo.Area.ha2               -0.027    0.047   -0.588    0.557
##
## Variances:
```

```
##                     Estimate  Std.Err  z-value  P(>|z|)
##    .Attendance2        0.279    0.018   15.133    0.000
##    .Total.Animals2     0.219    0.014   15.133    0.000
##    .Sp.Richness2       0.328    0.022   15.133    0.000
##    .Mean.Rap.Crck2     0.764    0.050   15.133    0.000
##     Mam.Sp.Rchnss2     0.998    0.066   15.133    0.000
##     Mn.Sp.BdyMsXA2     0.998    0.066   15.133    0.000
##     X10km_Pop2         0.998    0.066   15.133    0.000
##     GDP.Millions2      0.998    0.066   15.133    0.000
##     Zoo.Area.ha2       0.998    0.066   15.133    0.000
##
## R-Square:
##                     Estimate
##     Attendance2        0.691
##     Total.Animals2     0.777
##     Sp.Richness2       0.672
##     Mean.Rap.Crck2     0.234
```

```
# Generate fit indices
fitMeasures(mod.17A.fit, c("agfi", "rmr", "srmr", "rmsea", "cfi", "nnfi", "tli"))
```

```
##  agfi   rmr  srmr rmsea   cfi  nnfi   tli
## 0.826 0.054 0.055 0.133 0.955 0.894 0.894
```

```
# Generate modification indices
mi17A <- modindices(mod.17A.fit)
print(mi17A[mi17A$mi > 3.0,])
```

```
##                        lhs op              rhs     mi    epc sepc.lv
## 42          Total.Animals2  ~      Attendance2  8.005  0.120   0.120
## 45          Total.Animals2  ~       X10km_Pop2 13.844  0.084   0.084
## 46            Sp.Richness2  ~      Attendance2 16.053  0.212   0.212
## 49            Sp.Richness2  ~       X10km_Pop2 16.251  0.113   0.113
## 50            Sp.Richness2  ~    GDP.Millions2  6.442  0.068   0.068
## 51            Sp.Richness2  ~     Zoo.Area.ha2  8.322  0.096   0.096
## 52        Mean.Raup.Crick2  ~      Attendance2  7.593  0.240   0.240
## 53        Mean.Raup.Crick2  ~     Sp.Richness2 11.732 -0.256  -0.256
## 54        Mean.Raup.Crick2  ~ Mam.Sp.Richness2  8.804 -0.175  -0.175
## 55        Mean.Raup.Crick2  ~       X10km_Pop2  7.814  0.116   0.116
## 57        Mean.Raup.Crick2  ~     Zoo.Area.ha2 15.367  0.212   0.212
## 58        Mam.Sp.Richness2  ~      Attendance2 17.614 -0.636  -0.636
## 59        Mam.Sp.Richness2  ~   Total.Animals2  9.188 -0.345  -0.345
## 60        Mam.Sp.Richness2  ~     Sp.Richness2 24.069 -0.807  -0.807
## 61        Mam.Sp.Richness2  ~ Mean.Raup.Crick2 32.065 -0.317  -0.317
## 66 Mean.Sp.BodyMassXAbund2  ~      Attendance2  8.735 -0.479  -0.479
## 67 Mean.Sp.BodyMassXAbund2  ~   Total.Animals2  8.228 -0.534  -0.534
## 68 Mean.Sp.BodyMassXAbund2  ~     Sp.Richness2  7.091 -0.377  -0.377
## 69 Mean.Sp.BodyMassXAbund2  ~ Mean.Raup.Crick2 18.357 -0.426  -0.426
## 74              X10km_Pop2  ~      Attendance2 41.103  0.813   0.813
## 75              X10km_Pop2  ~   Total.Animals2 31.658  0.391   0.391
## 76              X10km_Pop2  ~     Sp.Richness2 19.897  0.345   0.345
## 77              X10km_Pop2  ~ Mean.Raup.Crick2 23.063  0.241   0.241
## 82           GDP.Millions2  ~      Attendance2  3.104  0.423   0.423
## 83           GDP.Millions2  ~   Total.Animals2  6.660  0.276   0.276
## 84           GDP.Millions2  ~     Sp.Richness2  6.237  0.203   0.203
## 90            Zoo.Area.ha2  ~      Attendance2 11.394  0.248   0.248
```

```
## 91            Zoo.Area.ha2  ~   Total.Animals2 11.974  0.289   0.289
## 92            Zoo.Area.ha2  ~     Sp.Richness2 11.210  0.219   0.219
## 93            Zoo.Area.ha2  ~ Mean.Raup.Crick2 28.590  0.235   0.235
##    sepc.all sepc.nox
## 42    0.115    0.115
## 45    0.085    0.085
## 46    0.202    0.202
## 49    0.113    0.113
## 50    0.068    0.068
## 51    0.096    0.096
## 52    0.228    0.228
## 53   -0.256   -0.256
## 54   -0.176   -0.176
## 55    0.116    0.116
## 57    0.212    0.212
## 58   -0.606   -0.606
## 59   -0.342   -0.342
## 60   -0.807   -0.807
## 61   -0.317   -0.317
## 66   -0.456   -0.456
## 67   -0.530   -0.530
## 68   -0.377   -0.377
## 69   -0.426   -0.426
## 74    0.774    0.774
## 75    0.388    0.388
## 76    0.345    0.345
## 77    0.241    0.241
## 82    0.403    0.403
## 83    0.274    0.274
## 84    0.203    0.203
## 90    0.236    0.236
## 91    0.287    0.287
## 92    0.219    0.219
## 93    0.235    0.235
```

```r
# Adjust for the nested nature of the data (institutions within countries)
# Fit model and generate model summary
design <- svydesign(ids = ~Country, nest=TRUE, data=sem_attendance_data)
fit.adj17A <- lavaan.survey(lavaan.fit = mod.17A.fit, survey.design = design)
summary(fit.adj17A, rsq = TRUE)
```

```
## lavaan (0.5-23.1097) converged normally after  27 iterations
##
##   Number of observations                           458
##
##   Estimator                                         ML      Robust
##   Minimum Function Test Statistic              100.196      25.365
##   Degrees of freedom                                11          11
##   P-value (Chi-square)                           0.000       0.008
##   Scaling correction factor                                  3.950
##     for the Satorra-Bentler correction
##
## Parameter Estimates:
##
##   Information                                 Expected
```

```
##   Standard Errors                           Robust.sem
##
## Regressions:
##                    Estimate  Std.Err  z-value  P(>|z|)
##   Attendance2 ~
##     Sp.Richness2     -0.184    0.059   -3.121    0.002
##     Total.Animals2    0.587    0.040   14.535    0.000
##     Mam.Sp.Rchnss2    0.102    0.043    2.354    0.019
##     Mn.Sp.BdyMsXA2    0.340    0.029   11.776    0.000
##     Mean.Rap.Crck2    0.125    0.031    4.041    0.000
##     X10km_Pop2        0.444    0.034   13.075    0.000
##     GDP.Millions2     0.163    0.027    6.120    0.000
##   Total.Animals2 ~
##     Zoo.Area.ha2      0.309    0.040    7.641    0.000
##     Sp.Richness2      0.759    0.047   16.099    0.000
##     Mn.Sp.BdyMsXA2   -0.157    0.035   -4.514    0.000
##     GDP.Millions2    -0.136    0.069   -1.987    0.047
##   Sp.Richness2 ~
##     Mam.Sp.Rchnss2    0.817    0.060   13.721    0.000
##     Mn.Sp.BdyMsXA2   -0.385    0.043   -8.893    0.000
##   Mean.Raup.Crick2 ~
##     Total.Animals2    0.185    0.052    3.571    0.000
##     Mn.Sp.BdyMsXA2   -0.423    0.075   -5.642    0.000
##
## Covariances:
##                            Estimate  Std.Err  z-value  P(>|z|)
##   Mam.Sp.Richness2 ~~
##     Mn.Sp.BdyMsXA2            0.227    0.078    2.921    0.003
##     X10km_Pop2                0.284    0.066    4.278    0.000
##     GDP.Millions2            -0.060    0.064   -0.937    0.349
##     Zoo.Area.ha2              0.381    0.064    5.985    0.000
##   Mean.Sp.BodyMassXAbund2 ~~
##     X10km_Pop2                0.021    0.064    0.329    0.742
##     GDP.Millions2            -0.079    0.038   -2.055    0.040
##     Zoo.Area.ha2              0.522    0.101    5.177    0.000
##   X10km_Pop2 ~~
##     GDP.Millions2            -0.028    0.056   -0.505    0.613
##     Zoo.Area.ha2             -0.010    0.078   -0.124    0.901
##   GDP.Millions2 ~~
##     Zoo.Area.ha2             -0.027    0.041   -0.673    0.501
##
## Intercepts:
##                  Estimate  Std.Err  z-value  P(>|z|)
##    .Attendance2    -0.000    0.029   -0.000    1.000
##    .Total.Animals2 -0.000    0.067   -0.000    1.000
##    .Sp.Richness2    0.000    0.073    0.000    1.000
##    .Mean.Rap.Crck2  0.000    0.097    0.000    1.000
##     Mam.Sp.Rchnss2 -0.000    0.079   -0.000    1.000
##     Mn.Sp.BdyMsXA2  0.000    0.071    0.000    1.000
##     X10km_Pop2      0.000    0.105    0.000    1.000
##     GDP.Millions2  -0.000    0.345   -0.000    1.000
##     Zoo.Area.ha2    0.000    0.064    0.000    1.000
##
## Variances:
```

```
##                    Estimate  Std.Err  z-value  P(>|z|)
##    .Attendance2       0.279    0.028   10.018    0.000
##    .Total.Animals2    0.219    0.037    5.986    0.000
##    .Sp.Richness2      0.328    0.033   10.049    0.000
##    .Mean.Rap.Crck2    0.764    0.102    7.516    0.000
##     Mam.Sp.Rchnss2    0.998    0.115    8.669    0.000
##     Mn.Sp.BdyMsXA2    0.998    0.160    6.219    0.000
##     X10km_Pop2        0.998    0.174    5.745    0.000
##     GDP.Millions2     0.998    0.246    4.061    0.000
##     Zoo.Area.ha2      0.998    0.112    8.886    0.000
##
## R-Square:
##                    Estimate
##     Attendance2       0.691
##     Total.Animals2    0.777
##     Sp.Richness2      0.672
##     Mean.Rap.Crck2    0.234
```

```
# Generate fit indices
fitMeasures(fit.adj17A, c("agfi", "rmr", "srmr", "rmsea", "cfi", "nnfi", "tli"))
```

```
##  agfi   rmr  srmr rmsea   cfi  nnfi   tli
## 0.791 0.054 0.050 0.133 0.955 0.894 0.894
```

```
# Generate modification indices
mi17adjA <- modindices(fit.adj17A)
print(mi17adjA[mi17adjA$mi > 3.0,])
```

```
##                          lhs op              rhs     mi mi.scaled    epc
## 51            Total.Animals2  ~      Attendance2  8.005     2.026  0.120
## 54            Total.Animals2  ~       X10km_Pop2 13.844     3.505  0.084
## 55              Sp.Richness2  ~      Attendance2 16.053     4.064  0.212
## 58              Sp.Richness2  ~       X10km_Pop2 16.251     4.114  0.113
## 59              Sp.Richness2  ~    GDP.Millions2  6.442     1.631  0.068
## 60              Sp.Richness2  ~     Zoo.Area.ha2  8.322     2.107  0.096
## 61          Mean.Raup.Crick2  ~      Attendance2  7.593     1.922  0.240
## 62          Mean.Raup.Crick2  ~     Sp.Richness2 11.732     2.970 -0.256
## 63          Mean.Raup.Crick2  ~ Mam.Sp.Richness2  8.804     2.229 -0.175
## 64          Mean.Raup.Crick2  ~       X10km_Pop2  7.814     1.978  0.116
## 66          Mean.Raup.Crick2  ~     Zoo.Area.ha2 15.367     3.890  0.212
## 67          Mam.Sp.Richness2  ~      Attendance2 17.614     4.459 -0.636
## 68          Mam.Sp.Richness2  ~   Total.Animals2  9.188     2.326 -0.345
## 69          Mam.Sp.Richness2  ~     Sp.Richness2 24.069     6.093 -0.807
## 70          Mam.Sp.Richness2  ~ Mean.Raup.Crick2 32.065     8.117 -0.317
## 75  Mean.Sp.BodyMassXAbund2  ~      Attendance2  8.735     2.211 -0.479
## 76  Mean.Sp.BodyMassXAbund2  ~   Total.Animals2  8.228     2.083 -0.534
## 77  Mean.Sp.BodyMassXAbund2  ~     Sp.Richness2  7.091     1.795 -0.377
## 78  Mean.Sp.BodyMassXAbund2  ~ Mean.Raup.Crick2 18.357     4.647 -0.426
## 83                X10km_Pop2  ~      Attendance2 41.103    10.405  0.813
## 84                X10km_Pop2  ~   Total.Animals2 31.658     8.014  0.391
## 85                X10km_Pop2  ~     Sp.Richness2 19.897     5.037  0.345
## 86                X10km_Pop2  ~ Mean.Raup.Crick2 23.063     5.839  0.241
## 91             GDP.Millions2  ~      Attendance2  3.104     0.786  0.423
## 92             GDP.Millions2  ~   Total.Animals2  6.660     1.686  0.276
## 93             GDP.Millions2  ~     Sp.Richness2  6.237     1.579  0.203
## 99              Zoo.Area.ha2  ~      Attendance2 11.394     2.884  0.248
```

```
## 100            Zoo.Area.ha2  ~   Total.Animals2 11.974     3.031  0.289
## 101            Zoo.Area.ha2  ~     Sp.Richness2 11.210     2.838  0.219
## 102            Zoo.Area.ha2  ~ Mean.Raup.Crick2 28.590     7.238  0.235
##     sepc.lv sepc.all sepc.nox
## 51    0.120    0.115    0.115
## 54    0.084    0.085    0.085
## 55    0.212    0.202    0.202
## 58    0.113    0.113    0.113
## 59    0.068    0.068    0.068
## 60    0.096    0.096    0.096
## 61    0.240    0.228    0.228
## 62   -0.256   -0.256   -0.256
## 63   -0.175   -0.176   -0.176
## 64    0.116    0.116    0.116
## 66    0.212    0.212    0.212
## 67   -0.636   -0.606   -0.606
## 68   -0.345   -0.342   -0.342
## 69   -0.807   -0.807   -0.807
## 70   -0.317   -0.317   -0.317
## 75   -0.479   -0.456   -0.456
## 76   -0.534   -0.530   -0.530
## 77   -0.377   -0.377   -0.377
## 78   -0.426   -0.426   -0.426
## 83    0.813    0.774    0.774
## 84    0.391    0.388    0.388
## 85    0.345    0.345    0.345
## 86    0.241    0.241    0.241
## 91    0.423    0.403    0.403
## 92    0.276    0.274    0.274
## 93    0.203    0.203    0.203
## 99    0.248    0.236    0.236
## 100   0.289    0.287    0.287
## 101   0.219    0.219    0.219
## 102   0.235    0.235    0.235
```

Model 18

Based on the modification indices generated from the nested seventeenth model, we can see that **Sp.Richness2 ~ Zoo.Area.ha2** has an mi value of **8.322**. This is one of the highest mi values and far exceeds the standard cut-off level for the chi-square test criterion of 3.84 (Burnham and Anderson, 2002). Although not the highest mi value, this is the most intuitive and still exceeds the standard cut-off level for the chi-square test criterion of 3.84 (Burnham and Anderson, 2002). This is considered intuituive as it is logical to assume that the size of an institution will influence how many species it can maintain. As a result, we add this relationship to our model. Once again, the model summary, fit indices and modification indices were all generated for the model, adjusting for the nested nature of data.

```
# Attendance SEM (Species Abundance)

# Model 18
# Addition of Sp.Richness2 ~ Zoo.Area.ha2, mi = 8.322

mod.18A <- 'Attendance2 ~ Sp.Richness2 + Total.Animals2
+ Mam.Sp.Richness2 + Mean.Sp.BodyMassXAbund2 + Mean.Raup.Crick2
```

```
+ X10km_Pop2 + GDP.Millions2

Total.Animals2 ~ Zoo.Area.ha2 + Sp.Richness2 + Mean.Sp.BodyMassXAbund2 + GDP.Millions2
Sp.Richness2 ~ Mam.Sp.Richness2 + Mean.Sp.BodyMassXAbund2 + Zoo.Area.ha2
Mean.Raup.Crick2 ~ Total.Animals2 + Mean.Sp.BodyMassXAbund2'

# Fit model and generate model summary
mod.18A.fit <- sem(mod.18A, data = sem_attendance_data, fixed.x=FALSE)
summary(mod.18A.fit, rsq = TRUE)
```

```
## lavaan (0.5-23.1097) converged normally after  29 iterations
##
##   Number of observations                           458
##
##   Estimator                                         ML
##   Minimum Function Test Statistic               91.797
##   Degrees of freedom                                10
##   P-value (Chi-square)                           0.000
##
## Parameter Estimates:
##
##   Information                                 Expected
##   Standard Errors                             Standard
##
## Regressions:
##                      Estimate  Std.Err  z-value  P(>|z|)
##   Attendance2 ~
##     Sp.Richness2       -0.184    0.058   -3.194    0.001
##     Total.Animals2      0.587    0.047   12.474    0.000
##     Mam.Sp.Rchnss2      0.102    0.044    2.304    0.021
##     Mn.Sp.BdyMsXA2      0.340    0.033   10.387    0.000
##     Mean.Rap.Crck2      0.125    0.028    4.415    0.000
##     X10km_Pop2          0.444    0.026   17.181    0.000
##     GDP.Millions2       0.163    0.026    6.379    0.000
##   Total.Animals2 ~
##     Zoo.Area.ha2        0.309    0.027   11.332    0.000
##     Sp.Richness2        0.759    0.024   32.013    0.000
##     Mn.Sp.BdyMsXA2     -0.157    0.027   -5.724    0.000
##     GDP.Millions2      -0.136    0.022   -6.197    0.000
##   Sp.Richness2 ~
##     Mam.Sp.Rchnss2      0.790    0.029   27.503    0.000
##     Mn.Sp.BdyMsXA2     -0.429    0.031  -13.754    0.000
##     Zoo.Area.ha2        0.096    0.033    2.911    0.004
##   Mean.Raup.Crick2 ~
##     Total.Animals2      0.185    0.041    4.505    0.000
##     Mn.Sp.BdyMsXA2     -0.423    0.041  -10.258    0.000
##
## Covariances:
##                              Estimate  Std.Err  z-value  P(>|z|)
##   Mam.Sp.Richness2 ~~
##     Mn.Sp.BdyMsXA2              0.227    0.048    4.750    0.000
##     X10km_Pop2                  0.284    0.048    5.852    0.000
##     GDP.Millions2              -0.060    0.047   -1.277    0.202
##     Zoo.Area.ha2                0.381    0.050    7.634    0.000
```

```
##   Mean.Sp.BodyMassXAbund2 ~~
##     X10km_Pop2                 0.021    0.047    0.455    0.649
##     GDP.Millions2             -0.079    0.047   -1.682    0.093
##     Zoo.Area.ha2               0.522    0.053    9.923    0.000
##   X10km_Pop2 ~~
##     GDP.Millions2             -0.028    0.047   -0.604    0.546
##     Zoo.Area.ha2              -0.010    0.047   -0.208    0.835
##   GDP.Millions2 ~~
##     Zoo.Area.ha2              -0.027    0.047   -0.588    0.557
##
## Variances:
##                    Estimate  Std.Err  z-value  P(>|z|)
##    .Attendance2       0.279    0.018   15.133    0.000
##    .Total.Animals2    0.219    0.014   15.133    0.000
##    .Sp.Richness2      0.322    0.021   15.133    0.000
##    .Mean.Rap.Crck2    0.764    0.050   15.133    0.000
##     Mam.Sp.Rchnss2    0.998    0.066   15.133    0.000
##     Mn.Sp.BdyMsXA2    0.998    0.066   15.133    0.000
##     X10km_Pop2        0.998    0.066   15.133    0.000
##     GDP.Millions2     0.998    0.066   15.133    0.000
##     Zoo.Area.ha2      0.998    0.066   15.133    0.000
##
## R-Square:
##                    Estimate
##     Attendance2       0.693
##     Total.Animals2    0.783
##     Sp.Richness2      0.678
##     Mean.Rap.Crck2    0.235
```

```
# Generate fit indices
fitMeasures(mod.18A.fit, c("agfi", "rmr", "srmr", "rmsea", "cfi", "nnfi", "tli"))
```

```
##  agfi   rmr  srmr rmsea   cfi  nnfi   tli
## 0.823 0.053 0.053 0.134 0.959 0.893 0.893
```

```
# Generate modification indices
mi18A <- modindices(mod.18A.fit)
print(mi18A[mi18A$mi > 3.0,])
```

```
##                        lhs op               rhs     mi    epc sepc.lv
## 43          Total.Animals2  ~       Attendance2  8.010  0.120   0.120
## 46          Total.Animals2  ~        X10km_Pop2 13.828  0.084   0.084
## 47            Sp.Richness2  ~       Attendance2 10.494  0.172   0.172
## 48            Sp.Richness2  ~    Total.Animals2  4.203 -0.138  -0.138
## 50            Sp.Richness2  ~        X10km_Pop2 20.019  0.125   0.125
## 51            Sp.Richness2  ~     GDP.Millions2  6.105  0.066   0.066
## 52          Mean.Raup.Crick2  ~       Attendance2  7.558  0.238   0.238
## 53          Mean.Raup.Crick2  ~      Sp.Richness2 12.210 -0.267  -0.267
## 54          Mean.Raup.Crick2  ~ Mam.Sp.Richness2  8.560 -0.171  -0.171
## 55          Mean.Raup.Crick2  ~        X10km_Pop2  7.788  0.116   0.116
## 57          Mean.Raup.Crick2  ~      Zoo.Area.ha2 16.351  0.225   0.225
## 58          Mam.Sp.Richness2  ~       Attendance2 13.872 -0.570  -0.570
## 59          Mam.Sp.Richness2  ~    Total.Animals2  4.458 -0.256  -0.256
## 60          Mam.Sp.Richness2  ~      Sp.Richness2 16.544 -0.935  -0.935
## 61          Mam.Sp.Richness2  ~ Mean.Raup.Crick2 29.845 -0.300  -0.300
## 66 Mean.Sp.BodyMassXAbund2  ~       Attendance2  4.725 -0.374  -0.374
```

```
## 69 Mean.Sp.BodyMassXAbund2  ~ Mean.Raup.Crick2 17.351 -0.431  -0.431
## 74              X10km_Pop2  ~      Attendance2 41.275  0.816   0.816
## 75              X10km_Pop2  ~   Total.Animals2 31.926  0.394   0.394
## 76              X10km_Pop2  ~     Sp.Richness2 20.265  0.352   0.352
## 77              X10km_Pop2  ~ Mean.Raup.Crick2 23.067  0.241   0.241
## 82           GDP.Millions2  ~      Attendance2  3.143  0.428   0.428
## 83           GDP.Millions2  ~   Total.Animals2  6.783  0.281   0.281
## 84           GDP.Millions2  ~     Sp.Richness2  6.352  0.206   0.206
## 90             Zoo.Area.ha2  ~      Attendance2  6.903  0.204   0.204
## 91             Zoo.Area.ha2  ~   Total.Animals2  6.285  0.769   0.769
## 92             Zoo.Area.ha2  ~     Sp.Richness2 14.271  1.911   1.911
## 93             Zoo.Area.ha2  ~ Mean.Raup.Crick2 26.878  0.233   0.233
##    sepc.all sepc.nox
## 43    0.113    0.113
## 46    0.083    0.084
## 47    0.165    0.165
## 48   -0.139   -0.139
## 50    0.125    0.125
## 51    0.066    0.066
## 52    0.228    0.228
## 53   -0.266   -0.266
## 54   -0.171   -0.171
## 55    0.116    0.116
## 57    0.225    0.226
## 58   -0.544   -0.544
## 59   -0.258   -0.258
## 60   -0.935   -0.935
## 61   -0.300   -0.300
## 66   -0.357   -0.357
## 69   -0.431   -0.431
## 74    0.779    0.779
## 75    0.397    0.397
## 76    0.352    0.352
## 77    0.242    0.242
## 82    0.409    0.409
## 83    0.283    0.283
## 84    0.206    0.206
## 90    0.195    0.195
## 91    0.774    0.774
## 92    1.911    1.911
## 93    0.233    0.233
```

```r
# Adjust for the nested nature of the data (institutions within countries)
# Fit model and generate model summary
design <- svydesign(ids = ~Country, nest=TRUE, data=sem_attendance_data)
fit.adj18A <- lavaan.survey(lavaan.fit = mod.18A.fit, survey.design = design)
summary(fit.adj18A, rsq = TRUE)
```

```
## lavaan (0.5-23.1097) converged normally after  29 iterations
##
##   Number of observations                           458
##
##   Estimator                                         ML      Robust
##   Minimum Function Test Statistic               91.797      21.065
##   Degrees of freedom                                10          10
```

```
##   P-value (Chi-square)                          0.000       0.021
##   Scaling correction factor                                 4.358
##     for the Satorra-Bentler correction
##
## Parameter Estimates:
##
##   Information                                 Expected
##   Standard Errors                           Robust.sem
##
## Regressions:
##                      Estimate  Std.Err  z-value  P(>|z|)
##   Attendance2 ~
##     Sp.Richness2       -0.184    0.063   -2.914    0.004
##     Total.Animals2      0.587    0.041   14.248    0.000
##     Mam.Sp.Rchnss2      0.102    0.044    2.319    0.020
##     Mn.Sp.BdyMsXA2      0.340    0.029   11.722    0.000
##     Mean.Rap.Crck2      0.125    0.031    4.041    0.000
##     X10km_Pop2          0.444    0.034   13.006    0.000
##     GDP.Millions2       0.163    0.027    6.084    0.000
##   Total.Animals2 ~
##     Zoo.Area.ha2        0.309    0.045    6.917    0.000
##     Sp.Richness2        0.759    0.050   15.041    0.000
##     Mn.Sp.BdyMsXA2     -0.157    0.036   -4.347    0.000
##     GDP.Millions2      -0.136    0.069   -1.985    0.047
##   Sp.Richness2 ~
##     Mam.Sp.Rchnss2      0.790    0.067   11.745    0.000
##     Mn.Sp.BdyMsXA2     -0.429    0.043  -10.072    0.000
##     Zoo.Area.ha2        0.096    0.040    2.380    0.017
##   Mean.Raup.Crick2 ~
##     Total.Animals2      0.185    0.050    3.677    0.000
##     Mn.Sp.BdyMsXA2     -0.423    0.075   -5.639    0.000
##
## Covariances:
##                              Estimate  Std.Err  z-value  P(>|z|)
##   Mam.Sp.Richness2 ~~
##     Mn.Sp.BdyMsXA2              0.227    0.078    2.921    0.003
##     X10km_Pop2                  0.284    0.066    4.278    0.000
##     GDP.Millions2              -0.060    0.064   -0.937    0.349
##     Zoo.Area.ha2                0.381    0.064    5.985    0.000
##   Mean.Sp.BodyMassXAbund2 ~~
##     X10km_Pop2                  0.021    0.064    0.329    0.742
##     GDP.Millions2              -0.079    0.038   -2.055    0.040
##     Zoo.Area.ha2                0.522    0.101    5.177    0.000
##   X10km_Pop2 ~~
##     GDP.Millions2              -0.028    0.056   -0.505    0.613
##     Zoo.Area.ha2               -0.010    0.078   -0.124    0.901
##   GDP.Millions2 ~~
##     Zoo.Area.ha2               -0.027    0.041   -0.673    0.501
##
## Intercepts:
##                    Estimate  Std.Err  z-value  P(>|z|)
##    .Attendance2      -0.000    0.029   -0.000    1.000
##    .Total.Animals2   -0.000    0.067   -0.000    1.000
##    .Sp.Richness2      0.000    0.075    0.000    1.000
```

```
##    .Mean.Rap.Crck2    0.000    0.097    0.000    1.000
##     Mam.Sp.Rchnss2   -0.000    0.079   -0.000    1.000
##     Mn.Sp.BdyMsXA2    0.000    0.071    0.000    1.000
##     X10km_Pop2        0.000    0.105    0.000    1.000
##     GDP.Millions2    -0.000    0.345   -0.000    1.000
##     Zoo.Area.ha2      0.000    0.064    0.000    1.000
##
## Variances:
##                    Estimate  Std.Err  z-value  P(>|z|)
##    .Attendance2       0.279    0.028   10.018    0.000
##    .Total.Animals2    0.219    0.037    5.986    0.000
##    .Sp.Richness2      0.322    0.032    9.984    0.000
##    .Mean.Rap.Crck2    0.764    0.102    7.516    0.000
##     Mam.Sp.Rchnss2    0.998    0.115    8.669    0.000
##     Mn.Sp.BdyMsXA2    0.998    0.160    6.219    0.000
##     X10km_Pop2        0.998    0.174    5.745    0.000
##     GDP.Millions2     0.998    0.246    4.061    0.000
##     Zoo.Area.ha2      0.998    0.112    8.886    0.000
##
## R-Square:
##                    Estimate
##     Attendance2       0.693
##     Total.Animals2    0.783
##     Sp.Richness2      0.678
##     Mean.Rap.Crck2    0.235
```

```
# Generate fit indices
fitMeasures(fit.adj18A, c("agfi", "rmr", "srmr", "rmsea", "cfi", "nnfi", "tli"))
```

```
##  agfi   rmr  srmr rmsea   cfi  nnfi   tli
## 0.787 0.053 0.048 0.134 0.959 0.893 0.893
```

```
# Generate modification indices
mi18adjA <- modindices(fit.adj18A)
print(mi18adjA[mi18adjA$mi > 3.0,])
```

```
##                         lhs op              rhs     mi mi.scaled    epc
## 52           Total.Animals2  ~      Attendance2  8.010     1.838  0.120
## 55           Total.Animals2  ~       X10km_Pop2 13.828     3.173  0.084
## 56             Sp.Richness2  ~      Attendance2 10.494     2.408  0.172
## 57             Sp.Richness2  ~   Total.Animals2  4.203     0.964 -0.138
## 59             Sp.Richness2  ~       X10km_Pop2 20.019     4.594  0.125
## 60             Sp.Richness2  ~    GDP.Millions2  6.105     1.401  0.066
## 61         Mean.Raup.Crick2  ~      Attendance2  7.558     1.734  0.238
## 62         Mean.Raup.Crick2  ~     Sp.Richness2 12.210     2.802 -0.267
## 63         Mean.Raup.Crick2  ~ Mam.Sp.Richness2  8.560     1.964 -0.171
## 64         Mean.Raup.Crick2  ~       X10km_Pop2  7.788     1.787  0.116
## 66         Mean.Raup.Crick2  ~     Zoo.Area.ha2 16.351     3.752  0.225
## 67         Mam.Sp.Richness2  ~      Attendance2 13.872     3.183 -0.570
## 68         Mam.Sp.Richness2  ~   Total.Animals2  4.458     1.023 -0.256
## 69         Mam.Sp.Richness2  ~     Sp.Richness2 16.544     3.796 -0.935
## 70         Mam.Sp.Richness2  ~ Mean.Raup.Crick2 29.845     6.849 -0.300
## 75  Mean.Sp.BodyMassXAbund2  ~      Attendance2  4.725     1.084 -0.374
## 78  Mean.Sp.BodyMassXAbund2  ~ Mean.Raup.Crick2 17.351     3.982 -0.431
## 83               X10km_Pop2  ~      Attendance2 41.275     9.472  0.816
## 84               X10km_Pop2  ~   Total.Animals2 31.926     7.326  0.394
```

```
## 85              X10km_Pop2  ~     Sp.Richness2 20.265     4.650  0.352
## 86              X10km_Pop2  ~ Mean.Raup.Crick2 23.067     5.293  0.241
## 91            GDP.Millions2  ~      Attendance2  3.143     0.721  0.428
## 92            GDP.Millions2  ~   Total.Animals2  6.783     1.556  0.281
## 93            GDP.Millions2  ~     Sp.Richness2  6.352     1.458  0.206
## 99              Zoo.Area.ha2  ~      Attendance2  6.903     1.584  0.204
## 100             Zoo.Area.ha2  ~   Total.Animals2  6.285     1.442  0.769
## 101             Zoo.Area.ha2  ~     Sp.Richness2 14.271     3.275  1.911
## 102             Zoo.Area.ha2  ~ Mean.Raup.Crick2 26.878     6.168  0.233
##     sepc.lv sepc.all sepc.nox
## 52    0.120    0.113    0.113
## 55    0.084    0.083    0.084
## 56    0.172    0.165    0.165
## 57   -0.138   -0.139   -0.139
## 59    0.125    0.125    0.125
## 60    0.066    0.066    0.066
## 61    0.238    0.228    0.228
## 62   -0.267   -0.266   -0.266
## 63   -0.171   -0.171   -0.171
## 64    0.116    0.116    0.116
## 66    0.225    0.225    0.226
## 67   -0.570   -0.544   -0.544
## 68   -0.256   -0.258   -0.258
## 69   -0.935   -0.935   -0.935
## 70   -0.300   -0.300   -0.300
## 75   -0.374   -0.357   -0.357
## 78   -0.431   -0.431   -0.431
## 83    0.816    0.779    0.779
## 84    0.394    0.397    0.397
## 85    0.352    0.352    0.352
## 86    0.241    0.242    0.242
## 91    0.428    0.409    0.409
## 92    0.281    0.283    0.283
## 93    0.206    0.206    0.206
## 99    0.204    0.195    0.195
## 100   0.769    0.774    0.774
## 101   1.911    1.911    1.911
## 102   0.233    0.233    0.233
```

Model 19

Based on the modification indices generated from the nested seventeenth model, we can see that **Mean.Raup.Crick2** ~ **Zoo.Area.ha2** has an mi value of **16.351**. This is one of the highest mi values and far exceeds the standard cut-off level for the chi-square test criterion of 3.84 (Burnham and Anderson, 2002). Although not the highest mi value, this is the most intuitive and still exceeds the standard cut-off level for the chi-square test criterion of 3.84 (Burnham and Anderson, 2002). This is considered intuituive as it is logical to assume that the size of an institution will influence how many species it can maintain. As a result, we add this relationship to our model. Once again, the model summary, fit indices and modification indices were all generated for the model, adjusting for the nested nature of data.

```
# Attendance SEM (Species Abundance)

# Model 19
```

```r
# Addition of Mean.Raup.Crick2 ~ Zoo.Area.ha2, mi = 16.352

mod.19A <- 'Attendance2 ~ Sp.Richness2 + Total.Animals2
+ Mam.Sp.Richness2 + Mean.Sp.BodyMassXAbund2 + Mean.Raup.Crick2
+ X10km_Pop2 + GDP.Millions2

Total.Animals2 ~ Zoo.Area.ha2 + Sp.Richness2 + Mean.Sp.BodyMassXAbund2 + GDP.Millions2
Sp.Richness2 ~ Mam.Sp.Richness2 + Mean.Sp.BodyMassXAbund2 + Zoo.Area.ha2
Mean.Raup.Crick2 ~ Total.Animals2 + Mean.Sp.BodyMassXAbund2 + Zoo.Area.ha2'

# Fit model and generate model summary
mod.19A.fit <- sem(mod.19A, data = sem_attendance_data, fixed.x=FALSE)
summary(mod.19A.fit, rsq = TRUE)
```

```
## lavaan (0.5-23.1097) converged normally after  31 iterations
##
##   Number of observations                           458
##
##   Estimator                                         ML
##   Minimum Function Test Statistic               75.066
##   Degrees of freedom                                 9
##   P-value (Chi-square)                           0.000
##
## Parameter Estimates:
##
##   Information                                 Expected
##   Standard Errors                             Standard
##
## Regressions:
##                    Estimate  Std.Err  z-value  P(>|z|)
##   Attendance2 ~
##     Sp.Richness2     -0.184    0.058   -3.191    0.001
##     Total.Animals2    0.587    0.047   12.419    0.000
##     Mam.Sp.Rchnss2    0.102    0.044    2.303    0.021
##     Mn.Sp.BdyMsXA2    0.340    0.033   10.341    0.000
##     Mean.Rap.Crck2    0.125    0.028    4.409    0.000
##     X10km_Pop2        0.444    0.026   17.177    0.000
##     GDP.Millions2     0.163    0.026    6.377    0.000
##   Total.Animals2 ~
##     Zoo.Area.ha2      0.309    0.027   11.332    0.000
##     Sp.Richness2      0.759    0.024   32.013    0.000
##     Mn.Sp.BdyMsXA2   -0.157    0.027   -5.724    0.000
##     GDP.Millions2    -0.136    0.022   -6.197    0.000
##   Sp.Richness2 ~
##     Mam.Sp.Rchnss2    0.790    0.029   27.503    0.000
##     Mn.Sp.BdyMsXA2   -0.429    0.031  -13.754    0.000
##     Zoo.Area.ha2      0.096    0.033    2.911    0.004
##   Mean.Raup.Crick2 ~
##     Total.Animals2    0.085    0.047    1.816    0.069
##     Mn.Sp.BdyMsXA2   -0.555    0.052  -10.773    0.000
##     Zoo.Area.ha2      0.226    0.055    4.138    0.000
##
## Covariances:
##                            Estimate  Std.Err  z-value  P(>|z|)
```

```
##   Mam.Sp.Richness2 ~~
##     Mn.Sp.BdyMsXA2            0.227    0.048    4.750    0.000
##     X10km_Pop2                0.284    0.048    5.852    0.000
##     GDP.Millions2            -0.060    0.047   -1.277    0.202
##     Zoo.Area.ha2              0.381    0.050    7.634    0.000
##   Mean.Sp.BodyMassXAbund2 ~~
##     X10km_Pop2                0.021    0.047    0.455    0.649
##     GDP.Millions2            -0.079    0.047   -1.682    0.093
##     Zoo.Area.ha2              0.522    0.053    9.923    0.000
##   X10km_Pop2 ~~
##     GDP.Millions2            -0.028    0.047   -0.604    0.546
##     Zoo.Area.ha2             -0.010    0.047   -0.208    0.835
##   GDP.Millions2 ~~
##     Zoo.Area.ha2             -0.027    0.047   -0.588    0.557
##
## Variances:
##                    Estimate  Std.Err  z-value  P(>|z|)
##    .Attendance2       0.279    0.018   15.133    0.000
##    .Total.Animals2    0.219    0.014   15.133    0.000
##    .Sp.Richness2      0.322    0.021   15.133    0.000
##    .Mean.Rap.Crck2    0.736    0.049   15.133    0.000
##     Mam.Sp.Rchnss2    0.998    0.066   15.133    0.000
##     Mn.Sp.BdyMsXA2    0.998    0.066   15.133    0.000
##     X10km_Pop2        0.998    0.066   15.133    0.000
##     GDP.Millions2     0.998    0.066   15.133    0.000
##     Zoo.Area.ha2      0.998    0.066   15.133    0.000
##
## R-Square:
##                    Estimate
##     Attendance2       0.692
##     Total.Animals2    0.783
##     Sp.Richness2      0.678
##     Mean.Rap.Crck2    0.262
```

```
# Generate fit indices
fitMeasures(mod.19A.fit, c("agfi", "rmr", "srmr", "rmsea", "cfi", "nnfi", "tli"))
```

```
##  agfi   rmr  srmr rmsea   cfi  nnfi   tli
## 0.830 0.050 0.050 0.127 0.967 0.904 0.904
```

```
# Generate modification indices
mi19A <- modindices(mod.19A.fit)
print(mi19A[mi19A$mi > 3.0,])
```

```
##                 lhs op              rhs     mi    epc sepc.lv sepc.all
## 41   Total.Animals2 ~~ Mean.Raup.Crick2  6.564  0.057   0.057    0.057
## 44   Total.Animals2  ~      Attendance2  9.547  0.131   0.131    0.125
## 45   Total.Animals2  ~ Mean.Raup.Crick2  6.564  0.078   0.078    0.077
## 47   Total.Animals2  ~       X10km_Pop2 13.828  0.084   0.084    0.083
## 48     Sp.Richness2  ~      Attendance2 12.187  0.187   0.187    0.178
## 49     Sp.Richness2  ~   Total.Animals2  4.203 -0.138  -0.138   -0.139
## 51     Sp.Richness2  ~       X10km_Pop2 20.019  0.125   0.125    0.125
## 52     Sp.Richness2  ~    GDP.Millions2  6.105  0.066   0.066    0.066
## 53 Mean.Raup.Crick2  ~      Attendance2  9.652  0.259   0.259    0.248
## 54 Mean.Raup.Crick2  ~     Sp.Richness2  7.708 -0.212  -0.212   -0.212
## 55 Mean.Raup.Crick2  ~ Mam.Sp.Richness2  7.036 -0.152  -0.152   -0.152
```

```
## 56 Mean.Raup.Crick2  ~       X10km_Pop2 14.572  0.157   0.157    0.157
## 58 Mam.Sp.Richness2  ~      Attendance2 10.832 -0.518  -0.518   -0.494
## 59 Mam.Sp.Richness2  ~   Total.Animals2  4.458 -0.256  -0.256   -0.258
## 60 Mam.Sp.Richness2  ~     Sp.Richness2 16.544 -0.935  -0.935   -0.935
## 61 Mam.Sp.Richness2  ~ Mean.Raup.Crick2 18.880 -0.262  -0.262   -0.262
## 74       X10km_Pop2  ~      Attendance2 43.225  0.855   0.855    0.815
## 75       X10km_Pop2  ~   Total.Animals2 31.926  0.394   0.394    0.397
## 76       X10km_Pop2  ~     Sp.Richness2 20.265  0.352   0.352    0.352
## 77       X10km_Pop2  ~ Mean.Raup.Crick2 24.261  0.254   0.254    0.254
## 83    GDP.Millions2  ~   Total.Animals2  6.783  0.281   0.281    0.283
## 84    GDP.Millions2  ~     Sp.Richness2  6.352  0.206   0.206    0.206
## 90     Zoo.Area.ha2  ~      Attendance2  3.461  0.151   0.151    0.144
## 91     Zoo.Area.ha2  ~   Total.Animals2  6.284  0.769   0.769    0.774
## 92     Zoo.Area.ha2  ~     Sp.Richness2 14.269  1.911   1.911    1.911
## 93     Zoo.Area.ha2  ~ Mean.Raup.Crick2 21.206  0.730   0.730    0.730
##    sepc.nox
## 41    0.057
## 44    0.125
## 45    0.077
## 47    0.084
## 48    0.178
## 49   -0.139
## 51    0.125
## 52    0.066
## 53    0.248
## 54   -0.212
## 55   -0.152
## 56    0.157
## 58   -0.494
## 59   -0.258
## 60   -0.935
## 61   -0.262
## 74    0.815
## 75    0.397
## 76    0.352
## 77    0.254
## 83    0.283
## 84    0.206
## 90    0.144
## 91    0.774
## 92    1.911
## 93    0.730
```

```r
# Adjust for the nested nature of the data (institutions within countries)
# Fit model and generate model summary
design <- svydesign(ids = ~Country, nest=TRUE, data=sem_attendance_data)
fit.adj19A <- lavaan.survey(lavaan.fit = mod.19A.fit, survey.design = design)
summary(fit.adj19A, rsq = TRUE)
```

```
## lavaan (0.5-23.1097) converged normally after  31 iterations
##
##   Number of observations                           458
##
##   Estimator                                         ML      Robust
##   Minimum Function Test Statistic               75.066      17.209
```

```
##   Degrees of freedom                                 9           9
##   P-value (Chi-square)                           0.000       0.046
##   Scaling correction factor                                  4.362
##     for the Satorra-Bentler correction
##
## Parameter Estimates:
##
##   Information                                 Expected
##   Standard Errors                           Robust.sem
##
## Regressions:
##                      Estimate  Std.Err  z-value  P(>|z|)
##   Attendance2 ~
##     Sp.Richness2       -0.184    0.064   -2.875    0.004
##     Total.Animals2      0.587    0.042   13.824    0.000
##     Mam.Sp.Rchnss2      0.102    0.044    2.315    0.021
##     Mn.Sp.BdyMsXA2      0.340    0.029   11.557    0.000
##     Mean.Rap.Crck2      0.125    0.031    3.966    0.000
##     X10km_Pop2          0.444    0.034   13.023    0.000
##     GDP.Millions2       0.163    0.027    6.012    0.000
##   Total.Animals2 ~
##     Zoo.Area.ha2        0.309    0.045    6.917    0.000
##     Sp.Richness2        0.759    0.050   15.041    0.000
##     Mn.Sp.BdyMsXA2     -0.157    0.036   -4.347    0.000
##     GDP.Millions2      -0.136    0.069   -1.985    0.047
##   Sp.Richness2 ~
##     Mam.Sp.Rchnss2      0.790    0.067   11.745    0.000
##     Mn.Sp.BdyMsXA2     -0.429    0.043  -10.072    0.000
##     Zoo.Area.ha2        0.096    0.040    2.380    0.017
##   Mean.Raup.Crick2 ~
##     Total.Animals2      0.085    0.066    1.280    0.201
##     Mn.Sp.BdyMsXA2     -0.555    0.080   -6.955    0.000
##     Zoo.Area.ha2        0.226    0.074    3.046    0.002
##
## Covariances:
##                              Estimate  Std.Err  z-value  P(>|z|)
##   Mam.Sp.Richness2 ~~
##     Mn.Sp.BdyMsXA2              0.227    0.078    2.921    0.003
##     X10km_Pop2                  0.284    0.066    4.278    0.000
##     GDP.Millions2              -0.060    0.064   -0.937    0.349
##     Zoo.Area.ha2                0.381    0.064    5.985    0.000
##   Mean.Sp.BodyMassXAbund2 ~~
##     X10km_Pop2                  0.021    0.064    0.329    0.742
##     GDP.Millions2              -0.079    0.038   -2.055    0.040
##     Zoo.Area.ha2                0.522    0.101    5.177    0.000
##   X10km_Pop2 ~~
##     GDP.Millions2              -0.028    0.056   -0.505    0.613
##     Zoo.Area.ha2               -0.010    0.078   -0.124    0.901
##   GDP.Millions2 ~~
##     Zoo.Area.ha2               -0.027    0.041   -0.673    0.501
##
## Intercepts:
##                    Estimate  Std.Err  z-value  P(>|z|)
##    .Attendance2      -0.000    0.029   -0.000    1.000
```

```
##    .Total.Animals2   -0.000    0.067   -0.000    1.000
##    .Sp.Richness2      0.000    0.075    0.000    1.000
##    .Mean.Rap.Crck2    0.000    0.091    0.000    1.000
##     Mam.Sp.Rchnss2   -0.000    0.079   -0.000    1.000
##     Mn.Sp.BdyMsXA2    0.000    0.071    0.000    1.000
##     X10km_Pop2        0.000    0.105    0.000    1.000
##     GDP.Millions2    -0.000    0.345   -0.000    1.000
##     Zoo.Area.ha2      0.000    0.064    0.000    1.000
##
## Variances:
##                    Estimate  Std.Err  z-value  P(>|z|)
##    .Attendance2       0.279    0.028   10.018    0.000
##    .Total.Animals2    0.219    0.037    5.986    0.000
##    .Sp.Richness2      0.322    0.032    9.984    0.000
##    .Mean.Rap.Crck2    0.736    0.090    8.216    0.000
##     Mam.Sp.Rchnss2    0.998    0.115    8.669    0.000
##     Mn.Sp.BdyMsXA2    0.998    0.160    6.219    0.000
##     X10km_Pop2        0.998    0.174    5.745    0.000
##     GDP.Millions2     0.998    0.246    4.061    0.000
##     Zoo.Area.ha2      0.998    0.112    8.886    0.000
##
## R-Square:
##                    Estimate
##     Attendance2       0.692
##     Total.Animals2    0.783
##     Sp.Richness2      0.678
##     Mean.Rap.Crck2    0.262
```

```
# Generate fit indices
fitMeasures(fit.adj19A, c("agfi", "rmr", "srmr", "rmsea", "cfi", "nnfi", "tli"))
```

```
##  agfi   rmr  srmr rmsea   cfi  nnfi   tli
## 0.796 0.050 0.046 0.127 0.967 0.904 0.904
```

```
# Generate modification indices
mi19adjA <- modindices(fit.adj19A)
print(mi19adjA[mi19adjA$mi > 3.0,])
```

```
##                  lhs op              rhs     mi mi.scaled    epc sepc.lv
## 50    Total.Animals2 ~~ Mean.Raup.Crick2  6.564     1.505  0.057   0.057
## 53    Total.Animals2  ~      Attendance2  9.547     2.189  0.131   0.131
## 54    Total.Animals2  ~ Mean.Raup.Crick2  6.564     1.505  0.078   0.078
## 56    Total.Animals2  ~       X10km_Pop2 13.828     3.170  0.084   0.084
## 57      Sp.Richness2  ~      Attendance2 12.187     2.794  0.187   0.187
## 58      Sp.Richness2  ~   Total.Animals2  4.203     0.964 -0.138  -0.138
## 60      Sp.Richness2  ~       X10km_Pop2 20.019     4.589  0.125   0.125
## 61      Sp.Richness2  ~    GDP.Millions2  6.105     1.400  0.066   0.066
## 62  Mean.Raup.Crick2  ~      Attendance2  9.652     2.213  0.259   0.259
## 63  Mean.Raup.Crick2  ~     Sp.Richness2  7.708     1.767 -0.212  -0.212
## 64  Mean.Raup.Crick2  ~ Mam.Sp.Richness2  7.036     1.613 -0.152  -0.152
## 65  Mean.Raup.Crick2  ~       X10km_Pop2 14.572     3.341  0.157   0.157
## 67  Mam.Sp.Richness2  ~      Attendance2 10.832     2.483 -0.518  -0.518
## 68  Mam.Sp.Richness2  ~   Total.Animals2  4.458     1.022 -0.256  -0.256
## 69  Mam.Sp.Richness2  ~     Sp.Richness2 16.544     3.793 -0.935  -0.935
## 70  Mam.Sp.Richness2  ~ Mean.Raup.Crick2 18.880     4.328 -0.262  -0.262
## 83        X10km_Pop2  ~      Attendance2 43.225     9.909  0.855   0.855
```

```
## 84        X10km_Pop2  ~   Total.Animals2 31.926     7.319  0.394   0.394
## 85        X10km_Pop2  ~     Sp.Richness2 20.265     4.646  0.352   0.352
## 86        X10km_Pop2  ~ Mean.Raup.Crick2 24.261     5.562  0.254   0.254
## 92     GDP.Millions2  ~   Total.Animals2  6.783     1.555  0.281   0.281
## 93     GDP.Millions2  ~     Sp.Richness2  6.352     1.456  0.206   0.206
## 99      Zoo.Area.ha2  ~      Attendance2  3.461     0.793  0.151   0.151
## 100     Zoo.Area.ha2  ~   Total.Animals2  6.284     1.441  0.769   0.769
## 101     Zoo.Area.ha2  ~     Sp.Richness2 14.269     3.271  1.911   1.911
## 102     Zoo.Area.ha2  ~ Mean.Raup.Crick2 21.206     4.861  0.730   0.730
##     sepc.all sepc.nox
## 50     0.057    0.057
## 53     0.125    0.125
## 54     0.077    0.077
## 56     0.083    0.084
## 57     0.178    0.178
## 58    -0.139   -0.139
## 60     0.125    0.125
## 61     0.066    0.066
## 62     0.248    0.248
## 63    -0.212   -0.212
## 64    -0.152   -0.152
## 65     0.157    0.157
## 67    -0.494   -0.494
## 68    -0.258   -0.258
## 69    -0.935   -0.935
## 70    -0.262   -0.262
## 83     0.815    0.815
## 84     0.397    0.397
## 85     0.352    0.352
## 86     0.254    0.254
## 92     0.283    0.283
## 93     0.206    0.206
## 99     0.144    0.144
## 100    0.774    0.774
## 101    1.911    1.911
## 102    0.730    0.730
```

Model 20

Based on the results generated from the nested nineteenth model, the highest p-value relationship to be considered for removal is **Mean.Raup.Crick2** ~ **Total.Animals2** with a p-value of **0.201**. As a result, we remove this relationship from our model. Once again, the model summary, fit indices and modification indices were all generated for the model, adjusting for the nested nature of data.

```
# Attendance SEM (Species Abundance)

# Model 20
# Removal of Mean.Raup.Crick2 ~ Total.Animals2, p = 0.201

mod.20A <- 'Attendance2 ~ Sp.Richness2 + Total.Animals2
+ Mam.Sp.Richness2 + Mean.Sp.BodyMassXAbund2 + Mean.Raup.Crick2
+ X10km_Pop2 + GDP.Millions2
```

```r
Total.Animals2 ~ Zoo.Area.ha2 + Sp.Richness2 + Mean.Sp.BodyMassXAbund2 + GDP.Millions2
Sp.Richness2 ~ Mam.Sp.Richness2 + Mean.Sp.BodyMassXAbund2 + Zoo.Area.ha2
Mean.Raup.Crick2 ~ Mean.Sp.BodyMassXAbund2 + Zoo.Area.ha2'

# Fit model and generate model summary
mod.20A.fit <- sem(mod.20A, data = sem_attendance_data, fixed.x=FALSE)
summary(mod.20A.fit, rsq = TRUE)
```

```
## lavaan (0.5-23.1097) converged normally after  31 iterations
##
##   Number of observations                           458
##
##   Estimator                                         ML
##   Minimum Function Test Statistic               78.292
##   Degrees of freedom                                10
##   P-value (Chi-square)                           0.000
##
## Parameter Estimates:
##
##   Information                                 Expected
##   Standard Errors                             Standard
##
## Regressions:
##                    Estimate  Std.Err  z-value  P(>|z|)
##   Attendance2 ~
##     Sp.Richness2      -0.184    0.058   -3.190    0.001
##     Total.Animals2     0.587    0.047   12.469    0.000
##     Mam.Sp.Rchnss2     0.102    0.044    2.303    0.021
##     Mn.Sp.BdyMsXA2     0.340    0.033   10.320    0.000
##     Mean.Rap.Crck2     0.125    0.028    4.460    0.000
##     X10km_Pop2         0.444    0.026   17.175    0.000
##     GDP.Millions2      0.163    0.026    6.375    0.000
##   Total.Animals2 ~
##     Zoo.Area.ha2       0.309    0.027   11.332    0.000
##     Sp.Richness2       0.759    0.024   32.013    0.000
##     Mn.Sp.BdyMsXA2    -0.157    0.027   -5.724    0.000
##     GDP.Millions2     -0.136    0.022   -6.197    0.000
##   Sp.Richness2 ~
##     Mam.Sp.Rchnss2     0.790    0.029   27.503    0.000
##     Mn.Sp.BdyMsXA2    -0.429    0.031  -13.754    0.000
##     Zoo.Area.ha2       0.096    0.033    2.911    0.004
##   Mean.Raup.Crick2 ~
##     Mn.Sp.BdyMsXA2    -0.593    0.047  -12.548    0.000
##     Zoo.Area.ha2       0.277    0.047    5.859    0.000
##
## Covariances:
##                            Estimate  Std.Err  z-value  P(>|z|)
##   Mam.Sp.Richness2 ~~
##     Mn.Sp.BdyMsXA2            0.227    0.048    4.750    0.000
##     X10km_Pop2                0.284    0.048    5.852    0.000
##     GDP.Millions2            -0.060    0.047   -1.277    0.202
##     Zoo.Area.ha2              0.381    0.050    7.634    0.000
##   Mean.Sp.BodyMassXAbund2 ~~
##     X10km_Pop2                0.021    0.047    0.455    0.649
```

```
##     GDP.Millions2              -0.079    0.047   -1.682    0.093
##     Zoo.Area.ha2                0.522    0.053    9.923    0.000
##   X10km_Pop2 ~~
##     GDP.Millions2              -0.028    0.047   -0.604    0.546
##     Zoo.Area.ha2               -0.010    0.047   -0.208    0.835
##   GDP.Millions2 ~~
##     Zoo.Area.ha2               -0.027    0.047   -0.588    0.557
##
## Variances:
##                    Estimate  Std.Err  z-value  P(>|z|)
##    .Attendance2       0.279    0.018   15.133    0.000
##    .Total.Animals2    0.219    0.014   15.133    0.000
##    .Sp.Richness2      0.322    0.021   15.133    0.000
##    .Mean.Rap.Crck2    0.742    0.049   15.133    0.000
##     Mam.Sp.Rchnss2    0.998    0.066   15.133    0.000
##     Mn.Sp.BdyMsXA2    0.998    0.066   15.133    0.000
##     X10km_Pop2        0.998    0.066   15.133    0.000
##     GDP.Millions2     0.998    0.066   15.133    0.000
##     Zoo.Area.ha2      0.998    0.066   15.133    0.000
##
## R-Square:
##                    Estimate
##     Attendance2       0.689
##     Total.Animals2    0.783
##     Sp.Richness2      0.678
##     Mean.Rap.Crck2    0.257
```

```
# Generate fit indices
fitMeasures(mod.20A.fit, c("agfi", "rmr", "srmr", "rmsea", "cfi", "nnfi", "tli"))
```

```
##  agfi   rmr  srmr rmsea   cfi  nnfi   tli
## 0.835 0.053 0.053 0.122 0.966 0.910 0.910
```

```
# Generate modification indices
mi20A <- modindices(mod.20A.fit)
print(mi20A[mi20A$mi > 3.0,])
```

```
##                        lhs op               rhs     mi    epc sepc.lv
## 40          Total.Animals2 ~~ Mean.Raup.Crick2  9.778  0.059   0.059
## 43          Total.Animals2  ~      Attendance2 10.760  0.139   0.139
## 44          Total.Animals2  ~ Mean.Raup.Crick2  9.778  0.079   0.079
## 46          Total.Animals2  ~       X10km_Pop2 13.828  0.084   0.084
## 47            Sp.Richness2  ~      Attendance2 13.524  0.196   0.196
## 48            Sp.Richness2  ~   Total.Animals2  4.203 -0.138  -0.138
## 50            Sp.Richness2  ~       X10km_Pop2 20.019  0.125   0.125
## 51            Sp.Richness2  ~    GDP.Millions2  6.105  0.066   0.066
## 52        Mean.Raup.Crick2  ~      Attendance2 13.268  0.214   0.214
## 53        Mean.Raup.Crick2  ~   Total.Animals2  3.157  0.083   0.083
## 56        Mean.Raup.Crick2  ~       X10km_Pop2 19.717  0.179   0.179
## 58        Mam.Sp.Richness2  ~      Attendance2  7.724 -0.429  -0.429
## 59        Mam.Sp.Richness2  ~   Total.Animals2  4.458 -0.256  -0.256
## 60        Mam.Sp.Richness2  ~     Sp.Richness2 16.544 -0.935  -0.935
## 61        Mam.Sp.Richness2  ~ Mean.Raup.Crick2  5.381 -0.110  -0.110
## 69 Mean.Sp.BodyMassXAbund2  ~ Mean.Raup.Crick2  3.013 -0.947  -0.947
## 74              X10km_Pop2  ~      Attendance2 44.801  0.886   0.886
## 75              X10km_Pop2  ~   Total.Animals2 31.926  0.394   0.394
```

```
## 76             X10km_Pop2  ~     Sp.Richness2 20.265  0.352   0.352
## 77             X10km_Pop2  ~ Mean.Raup.Crick2 24.186  0.253   0.253
## 83           GDP.Millions2  ~   Total.Animals2  6.783  0.281   0.281
## 84           GDP.Millions2  ~     Sp.Richness2  6.352  0.206   0.206
## 90            Zoo.Area.ha2  ~      Attendance2  3.200  0.146   0.146
## 91            Zoo.Area.ha2  ~   Total.Animals2  6.284  0.769   0.769
## 92            Zoo.Area.ha2  ~     Sp.Richness2 14.270  1.911   1.911
## 93            Zoo.Area.ha2  ~ Mean.Raup.Crick2  7.024  0.339   0.339
##    sepc.all sepc.nox
## 40    0.059    0.059
## 43    0.131    0.131
## 44    0.079    0.079
## 46    0.083    0.084
## 47    0.186    0.186
## 48   -0.139   -0.139
## 50    0.125    0.125
## 51    0.066    0.066
## 52    0.203    0.203
## 53    0.084    0.084
## 56    0.179    0.179
## 58   -0.407   -0.407
## 59   -0.258   -0.258
## 60   -0.935   -0.935
## 61   -0.110   -0.110
## 69   -0.947   -0.947
## 74    0.841    0.841
## 75    0.397    0.397
## 76    0.352    0.352
## 77    0.253    0.253
## 83    0.283    0.283
## 84    0.206    0.206
## 90    0.138    0.138
## 91    0.774    0.774
## 92    1.911    1.911
## 93    0.339    0.339
```

```
# Adjust for the nested nature of the data (institutions within countries)
# Fit model and generate model summary
design <- svydesign(ids = ~Country, nest=TRUE, data=sem_attendance_data)
fit.adj20A <- lavaan.survey(lavaan.fit = mod.20A.fit, survey.design = design)
summary(fit.adj20A, rsq = TRUE)
```

```
## lavaan (0.5-23.1097) converged normally after  31 iterations
##
##   Number of observations                           458
##
##   Estimator                                         ML      Robust
##   Minimum Function Test Statistic               78.292      19.530
##   Degrees of freedom                                10          10
##   P-value (Chi-square)                           0.000       0.034
##   Scaling correction factor                                  4.009
##     for the Satorra-Bentler correction
##
## Parameter Estimates:
##
```

```
##   Information                                 Expected
##   Standard Errors                           Robust.sem
##
## Regressions:
##                      Estimate  Std.Err  z-value  P(>|z|)
##   Attendance2 ~
##     Sp.Richness2       -0.184    0.064   -2.862    0.004
##     Total.Animals2      0.587    0.041   14.148    0.000
##     Mam.Sp.Rchnss2      0.102    0.044    2.313    0.021
##     Mn.Sp.BdyMsXA2      0.340    0.030   11.436    0.000
##     Mean.Rap.Crck2      0.125    0.031    4.060    0.000
##     X10km_Pop2          0.444    0.034   13.018    0.000
##     GDP.Millions2       0.163    0.027    5.997    0.000
##   Total.Animals2 ~
##     Zoo.Area.ha2        0.309    0.045    6.917    0.000
##     Sp.Richness2        0.759    0.050   15.041    0.000
##     Mn.Sp.BdyMsXA2     -0.157    0.036   -4.347    0.000
##     GDP.Millions2      -0.136    0.069   -1.985    0.047
##   Sp.Richness2 ~
##     Mam.Sp.Rchnss2      0.790    0.067   11.745    0.000
##     Mn.Sp.BdyMsXA2     -0.429    0.043  -10.072    0.000
##     Zoo.Area.ha2        0.096    0.040    2.380    0.017
##   Mean.Raup.Crick2 ~
##     Mn.Sp.BdyMsXA2     -0.593    0.077   -7.676    0.000
##     Zoo.Area.ha2        0.277    0.051    5.419    0.000
##
## Covariances:
##                              Estimate  Std.Err  z-value  P(>|z|)
##   Mam.Sp.Richness2 ~~
##     Mn.Sp.BdyMsXA2              0.227    0.078    2.921    0.003
##     X10km_Pop2                  0.284    0.066    4.278    0.000
##     GDP.Millions2              -0.060    0.064   -0.937    0.349
##     Zoo.Area.ha2                0.381    0.064    5.985    0.000
##   Mean.Sp.BodyMassXAbund2 ~~
##     X10km_Pop2                  0.021    0.064    0.329    0.742
##     GDP.Millions2              -0.079    0.038   -2.055    0.040
##     Zoo.Area.ha2                0.522    0.101    5.177    0.000
##   X10km_Pop2 ~~
##     GDP.Millions2              -0.028    0.056   -0.505    0.613
##     Zoo.Area.ha2               -0.010    0.078   -0.124    0.901
##   GDP.Millions2 ~~
##     Zoo.Area.ha2               -0.027    0.041   -0.673    0.501
##
## Intercepts:
##                    Estimate  Std.Err  z-value  P(>|z|)
##    .Attendance2      -0.000    0.029   -0.000    1.000
##    .Total.Animals2   -0.000    0.067   -0.000    1.000
##    .Sp.Richness2      0.000    0.075    0.000    1.000
##    .Mean.Rap.Crck2    0.000    0.090    0.000    1.000
##     Mam.Sp.Rchnss2   -0.000    0.079   -0.000    1.000
##     Mn.Sp.BdyMsXA2    0.000    0.071    0.000    1.000
##     X10km_Pop2        0.000    0.105    0.000    1.000
##     GDP.Millions2    -0.000    0.345   -0.000    1.000
##     Zoo.Area.ha2      0.000    0.064    0.000    1.000
```

```
##
## Variances:
##                    Estimate  Std.Err  z-value  P(>|z|)
##    .Attendance2       0.279    0.028   10.018    0.000
##    .Total.Animals2    0.219    0.037    5.986    0.000
##    .Sp.Richness2      0.322    0.032    9.984    0.000
##    .Mean.Rap.Crck2    0.742    0.084    8.815    0.000
##     Mam.Sp.Rchnss2    0.998    0.115    8.669    0.000
##     Mn.Sp.BdyMsXA2    0.998    0.160    6.219    0.000
##     X10km_Pop2        0.998    0.174    5.745    0.000
##     GDP.Millions2     0.998    0.246    4.061    0.000
##     Zoo.Area.ha2      0.998    0.112    8.886    0.000
##
## R-Square:
##                    Estimate
##     Attendance2       0.689
##     Total.Animals2    0.783
##     Sp.Richness2      0.678
##     Mean.Rap.Crck2    0.257
```

```
# Generate fit indices
fitMeasures(fit.adj20A, c("agfi", "rmr", "srmr", "rmsea", "cfi", "nnfi", "tli"))
```

```
##  agfi   rmr  srmr rmsea   cfi  nnfi   tli
## 0.802 0.053 0.048 0.122 0.966 0.910 0.910
```

```
# Generate modification indices
mi20adjA <- modindices(fit.adj20A)
print(mi20adjA[mi20adjA$mi > 3.0,])
```

```
##                          lhs op              rhs     mi mi.scaled    epc
## 49           Total.Animals2 ~~ Mean.Raup.Crick2  9.778     2.439  0.059
## 52           Total.Animals2  ~      Attendance2 10.760     2.684  0.139
## 53           Total.Animals2  ~ Mean.Raup.Crick2  9.778     2.439  0.079
## 55           Total.Animals2  ~       X10km_Pop2 13.828     3.450  0.084
## 56             Sp.Richness2  ~      Attendance2 13.524     3.374  0.196
## 57             Sp.Richness2  ~   Total.Animals2  4.203     1.048 -0.138
## 59             Sp.Richness2  ~       X10km_Pop2 20.019     4.994  0.125
## 60             Sp.Richness2  ~    GDP.Millions2  6.105     1.523  0.066
## 61         Mean.Raup.Crick2  ~      Attendance2 13.268     3.310  0.214
## 62         Mean.Raup.Crick2  ~   Total.Animals2  3.157     0.787  0.083
## 65         Mean.Raup.Crick2  ~       X10km_Pop2 19.717     4.918  0.179
## 67         Mam.Sp.Richness2  ~      Attendance2  7.724     1.927 -0.429
## 68         Mam.Sp.Richness2  ~   Total.Animals2  4.458     1.112 -0.256
## 69         Mam.Sp.Richness2  ~     Sp.Richness2 16.544     4.127 -0.935
## 70         Mam.Sp.Richness2  ~ Mean.Raup.Crick2  5.381     1.342 -0.110
## 78  Mean.Sp.BodyMassXAbund2  ~ Mean.Raup.Crick2  3.013     0.752 -0.947
## 83               X10km_Pop2  ~      Attendance2 44.801    11.176  0.886
## 84               X10km_Pop2  ~   Total.Animals2 31.926     7.964  0.394
## 85               X10km_Pop2  ~     Sp.Richness2 20.265     5.055  0.352
## 86               X10km_Pop2  ~ Mean.Raup.Crick2 24.186     6.033  0.253
## 92            GDP.Millions2  ~   Total.Animals2  6.783     1.692  0.281
## 93            GDP.Millions2  ~     Sp.Richness2  6.352     1.585  0.206
## 99             Zoo.Area.ha2  ~      Attendance2  3.200     0.798  0.146
## 100            Zoo.Area.ha2  ~   Total.Animals2  6.284     1.568  0.769
## 101            Zoo.Area.ha2  ~     Sp.Richness2 14.270     3.560  1.911
```

```
## 102           Zoo.Area.ha2  ~ Mean.Raup.Crick2  7.024     1.752  0.339
##     sepc.lv sepc.all sepc.nox
## 49    0.059    0.059    0.059
## 52    0.139    0.131    0.131
## 53    0.079    0.079    0.079
## 55    0.084    0.083    0.084
## 56    0.196    0.186    0.186
## 57   -0.138   -0.139   -0.139
## 59    0.125    0.125    0.125
## 60    0.066    0.066    0.066
## 61    0.214    0.203    0.203
## 62    0.083    0.084    0.084
## 65    0.179    0.179    0.179
## 67   -0.429   -0.407   -0.407
## 68   -0.256   -0.258   -0.258
## 69   -0.935   -0.935   -0.935
## 70   -0.110   -0.110   -0.110
## 78   -0.947   -0.947   -0.947
## 83    0.886    0.841    0.841
## 84    0.394    0.397    0.397
## 85    0.352    0.352    0.352
## 86    0.253    0.253    0.253
## 92    0.281    0.283    0.283
## 93    0.206    0.206    0.206
## 99    0.146    0.138    0.138
## 100   0.769    0.774    0.774
## 101   1.911    1.911    1.911
## 102   0.339    0.339    0.339
```

Model Comparisons 6

At this stage we compare the models generated using AICc values. Overall model selection from the pool of competing models is achieved using AICc values, with a threshold of more than 2 AICc units lower than nearest competing model being considered sufficient for model selection.

```r
# Model Comparisons using AICc

# Comparing models with and without adjustment for nested nature of data
# library(AICcmodavg)
# source("lavaan.modavg.R")

aictab.lavaan(list(mod.1A.fit, mod.2A.fit, mod.3A.fit, mod.4A.fit, mod.5A.fit, mod.6A.fit, mod.7A.fit,
                   mod.8A.fit, mod.9A.fit, mod.10A.fit, mod.11A.fit, mod.12A.fit, mod.13A.fit, mod.14A.
                   mod.15A.fit, mod.16A.fit, mod.17A.fit, mod.18A.fit, mod.19A.fit, mod.20A.fit,fit.adj
                   fit.adj3A, fit.adj4A, fit.adj5A, fit.adj6A, fit.adj7A, fit.adj8A, fit.adj9A, fit.adj
                   fit.adj11A, fit.adj12A, fit.adj13A, fit.adj14A, fit.adj15A, fit.adj16A, fit.adj17A,
                   fit.adj18A, fit.adj19A, fit.adj20A),
              c("mod.1A", "mod.2A", "mod.3A", "mod.4A", "mod.5A", "mod.6A", "mod.7A", "mod.8A",
                "mod.9A", "mod.10A", "mod.11A", "mod.12A", "mod.13A", "mod.14A", "mod.15A", "mod.16A",
                "mod.17A", "mod.18A", "mod.19A", "mod.20A", "mod.1A.nested", "mod.2A.nested", "mod.3A.n
```

```
##
## Model selection based on AICc:
##
```

```
##                  K     AICc Delta_AICc AICcWt Cum.Wt       LL
## mod.19A        36  9556.71       0.00   0.63   0.63 -4741.66
## mod.20A        35  9557.78       1.07   0.37   1.00 -4743.27
## mod.16A        35  9567.54      10.83   0.00   1.00 -4748.15
## mod.18A        35  9571.28      14.57   0.00   1.00 -4750.02
## mod.19A.nested 45  9574.71      18.00   0.00   1.00 -4741.66
## mod.20A.nested 44  9575.78      19.07   0.00   1.00 -4743.27
## mod.17A        34  9577.53      20.83   0.00   1.00 -4754.22
## mod.16A.nested 44  9585.54      28.83   0.00   1.00 -4748.15
## mod.18A.nested 44  9589.28      32.57   0.00   1.00 -4750.02
## mod.17A.nested 43  9595.53      38.83   0.00   1.00 -4754.22
## mod.15A        34  9728.23     171.53   0.00   1.00 -4829.57
## mod.15A.nested 43  9746.23     189.53   0.00   1.00 -4829.57
## mod.14A        42 10222.15     665.44   0.00   1.00 -5068.38
## mod.14A.nested 52 10242.15     685.44   0.00   1.00 -5068.38
## mod.13A        50 10823.47    1266.76   0.00   1.00 -5360.95
## mod.13A.nested 61 10845.47    1288.76   0.00   1.00 -5360.95
## mod.12A.nested 71 11759.11    2202.41   0.00   1.00 -5807.69
## mod.11A.nested 82 12428.03    2871.32   0.00   1.00 -6131.05
## mod.10A.nested 83 12425.44    2868.73   0.00   1.00 -6128.66
## mod.9A.nested  84 12417.36    2860.66   0.00   1.00 -6123.52
## mod.8A.nested  96 13700.23    4143.53   0.00   1.00 -6752.85
## mod.7A.nested  94 14397.64    4840.94   0.00   1.00 -7102.93
## mod.6A.nested  93 14432.28    4875.57   0.00   1.00 -7121.38
## mod.5A.nested  92 14455.87    4899.16   0.00   1.00 -7134.31
## mod.4A.nested  91 14492.11    4935.40   0.00   1.00 -7153.55
## mod.3A.nested  90 14557.06    5000.35   0.00   1.00 -7187.14
## mod.2A.nested  89 14659.00    5102.30   0.00   1.00 -7239.23
## mod.1A.nested  88 15315.69    5758.98   0.00   1.00 -7568.68
## mod.12A        59 11735.11    2178.41   0.00   1.00 -5807.69
## mod.11A        69 12402.03    2845.32   0.00   1.00 -6131.05
## mod.10A        70 12399.44    2842.73   0.00   1.00 -6128.66
## mod.9A         71 12391.36    2834.66   0.00   1.00 -6123.52
## mod.8A         82 13672.23    4115.53   0.00   1.00 -6752.85
## mod.7A         79 14367.64    4810.94   0.00   1.00 -7102.93
## mod.6A         78 14402.28    4845.57   0.00   1.00 -7121.38
## mod.5A         77 14425.87    4869.16   0.00   1.00 -7134.31
## mod.4A         76 14462.11    4905.40   0.00   1.00 -7153.55
## mod.3A         75 14527.06    4970.35   0.00   1.00 -7187.14
## mod.2A         74 14629.00    5072.30   0.00   1.00 -7239.23
## mod.1A         73 15285.69    5728.98   0.00   1.00 -7568.68
```

Based on these results we can see that model 20 is the superior model (lowest AICc values), for both models with and without adjustment for nested nature of data. Although model 19 has the lowest AIC, it isn't by 2 units compared to 20, so the addition of the pathway in model 19 is not justified.

Final Attendance Model (Species Abundance)

Contents
Based on the results, we believe model 20 is an accurate representation of the system. We see no conceptually appealling addition and all pathways are significant. Therefore we present the nested version of model 20 as our final Attendance Model based on species abundance data.

```
# Chosen Attendance SEM (Abundance)
# Model 20

mod.20A.final <- 'Attendance2 ~ Sp.Richness2 + Total.Animals2
+ Mam.Sp.Richness2 + Mean.Sp.BodyMassXAbund2 + Mean.Raup.Crick2
+ X10km_Pop2 + GDP.Millions2

Total.Animals2 ~ Zoo.Area.ha2 + Sp.Richness2 + Mean.Sp.BodyMassXAbund2 + GDP.Millions2
Sp.Richness2 ~ Mam.Sp.Richness2 + Mean.Sp.BodyMassXAbund2 + Zoo.Area.ha2
Mean.Raup.Crick2 ~ Mean.Sp.BodyMassXAbund2 + Zoo.Area.ha2'

# Fit model and generate model summary
mod.20A.final.fit <- sem(mod.20A.final, data = sem_attendance_data, fixed.x=FALSE)
summary(mod.20A.final.fit, rsq = TRUE)
```

```
## lavaan (0.5-23.1097) converged normally after  31 iterations
##
##   Number of observations                           458
##
##   Estimator                                         ML
##   Minimum Function Test Statistic               78.292
##   Degrees of freedom                                10
##   P-value (Chi-square)                           0.000
##
## Parameter Estimates:
##
##   Information                                 Expected
##   Standard Errors                             Standard
##
## Regressions:
##                        Estimate  Std.Err  z-value  P(>|z|)
##   Attendance2 ~
##     Sp.Richness2        -0.184    0.058   -3.190    0.001
##     Total.Animals2       0.587    0.047   12.469    0.000
##     Mam.Sp.Rchnss2       0.102    0.044    2.303    0.021
##     Mn.Sp.BdyMsXA2       0.340    0.033   10.320    0.000
##     Mean.Rap.Crck2       0.125    0.028    4.460    0.000
##     X10km_Pop2           0.444    0.026   17.175    0.000
##     GDP.Millions2        0.163    0.026    6.375    0.000
##   Total.Animals2 ~
##     Zoo.Area.ha2         0.309    0.027   11.332    0.000
##     Sp.Richness2         0.759    0.024   32.013    0.000
##     Mn.Sp.BdyMsXA2      -0.157    0.027   -5.724    0.000
##     GDP.Millions2       -0.136    0.022   -6.197    0.000
##   Sp.Richness2 ~
##     Mam.Sp.Rchnss2       0.790    0.029   27.503    0.000
##     Mn.Sp.BdyMsXA2      -0.429    0.031  -13.754    0.000
##     Zoo.Area.ha2         0.096    0.033    2.911    0.004
##   Mean.Raup.Crick2 ~
##     Mn.Sp.BdyMsXA2      -0.593    0.047  -12.548    0.000
##     Zoo.Area.ha2         0.277    0.047    5.859    0.000
##
## Covariances:
##                                Estimate  Std.Err  z-value  P(>|z|)
```

```
##   Mam.Sp.Richness2 ~~
##     Mn.Sp.BdyMsXA2           0.227    0.048    4.750    0.000
##     X10km_Pop2               0.284    0.048    5.852    0.000
##     GDP.Millions2           -0.060    0.047   -1.277    0.202
##     Zoo.Area.ha2             0.381    0.050    7.634    0.000
##   Mean.Sp.BodyMassXAbund2 ~~
##     X10km_Pop2               0.021    0.047    0.455    0.649
##     GDP.Millions2           -0.079    0.047   -1.682    0.093
##     Zoo.Area.ha2             0.522    0.053    9.923    0.000
##   X10km_Pop2 ~~
##     GDP.Millions2           -0.028    0.047   -0.604    0.546
##     Zoo.Area.ha2            -0.010    0.047   -0.208    0.835
##   GDP.Millions2 ~~
##     Zoo.Area.ha2            -0.027    0.047   -0.588    0.557
##
## Variances:
##                    Estimate  Std.Err  z-value  P(>|z|)
##    .Attendance2       0.279    0.018   15.133    0.000
##    .Total.Animals2    0.219    0.014   15.133    0.000
##    .Sp.Richness2      0.322    0.021   15.133    0.000
##    .Mean.Rap.Crck2    0.742    0.049   15.133    0.000
##     Mam.Sp.Rchnss2    0.998    0.066   15.133    0.000
##     Mn.Sp.BdyMsXA2    0.998    0.066   15.133    0.000
##     X10km_Pop2        0.998    0.066   15.133    0.000
##     GDP.Millions2     0.998    0.066   15.133    0.000
##     Zoo.Area.ha2      0.998    0.066   15.133    0.000
##
## R-Square:
##                    Estimate
##     Attendance2       0.689
##     Total.Animals2    0.783
##     Sp.Richness2      0.678
##     Mean.Rap.Crck2    0.257
```

```
# Adjust for the nested nature of the data (institutions within countries)
# Fit model and generate model summary
design <- svydesign(ids = ~Country, nest=TRUE, data=sem_attendance_data)
fit.adj20A.final <- lavaan.survey(lavaan.fit = mod.20A.final.fit, survey.design = design)
summary(fit.adj20A.final, rsq = TRUE)
```

```
## lavaan (0.5-23.1097) converged normally after  31 iterations
##
##   Number of observations                           458
##
##   Estimator                                         ML      Robust
##   Minimum Function Test Statistic               78.292      19.530
##   Degrees of freedom                                10          10
##   P-value (Chi-square)                           0.000       0.034
##   Scaling correction factor                                  4.009
##     for the Satorra-Bentler correction
##
## Parameter Estimates:
##
##   Information                                 Expected
##   Standard Errors                           Robust.sem
```

```
##
## Regressions:
##                    Estimate  Std.Err  z-value  P(>|z|)
##   Attendance2 ~
##     Sp.Richness2      -0.184    0.064   -2.862    0.004
##     Total.Animals2     0.587    0.041   14.148    0.000
##     Mam.Sp.Rchnss2     0.102    0.044    2.313    0.021
##     Mn.Sp.BdyMsXA2     0.340    0.030   11.436    0.000
##     Mean.Rap.Crck2     0.125    0.031    4.060    0.000
##     X10km_Pop2         0.444    0.034   13.018    0.000
##     GDP.Millions2      0.163    0.027    5.997    0.000
##   Total.Animals2 ~
##     Zoo.Area.ha2       0.309    0.045    6.917    0.000
##     Sp.Richness2       0.759    0.050   15.041    0.000
##     Mn.Sp.BdyMsXA2    -0.157    0.036   -4.347    0.000
##     GDP.Millions2     -0.136    0.069   -1.985    0.047
##   Sp.Richness2 ~
##     Mam.Sp.Rchnss2     0.790    0.067   11.745    0.000
##     Mn.Sp.BdyMsXA2    -0.429    0.043  -10.072    0.000
##     Zoo.Area.ha2       0.096    0.040    2.380    0.017
##   Mean.Raup.Crick2 ~
##     Mn.Sp.BdyMsXA2    -0.593    0.077   -7.676    0.000
##     Zoo.Area.ha2       0.277    0.051    5.419    0.000
##
## Covariances:
##                            Estimate  Std.Err  z-value  P(>|z|)
##   Mam.Sp.Richness2 ~~
##     Mn.Sp.BdyMsXA2             0.227    0.078    2.921    0.003
##     X10km_Pop2                 0.284    0.066    4.278    0.000
##     GDP.Millions2             -0.060    0.064   -0.937    0.349
##     Zoo.Area.ha2               0.381    0.064    5.985    0.000
##   Mean.Sp.BodyMassXAbund2 ~~
##     X10km_Pop2                 0.021    0.064    0.329    0.742
##     GDP.Millions2             -0.079    0.038   -2.055    0.040
##     Zoo.Area.ha2               0.522    0.101    5.177    0.000
##   X10km_Pop2 ~~
##     GDP.Millions2             -0.028    0.056   -0.505    0.613
##     Zoo.Area.ha2              -0.010    0.078   -0.124    0.901
##   GDP.Millions2 ~~
##     Zoo.Area.ha2              -0.027    0.041   -0.673    0.501
##
## Intercepts:
##                  Estimate  Std.Err  z-value  P(>|z|)
##    .Attendance2    -0.000    0.029   -0.000    1.000
##    .Total.Animals2 -0.000    0.067   -0.000    1.000
##    .Sp.Richness2    0.000    0.075    0.000    1.000
##    .Mean.Rap.Crck2  0.000    0.090    0.000    1.000
##     Mam.Sp.Rchnss2 -0.000    0.079   -0.000    1.000
##     Mn.Sp.BdyMsXA2  0.000    0.071    0.000    1.000
##     X10km_Pop2      0.000    0.105    0.000    1.000
##     GDP.Millions2  -0.000    0.345   -0.000    1.000
##     Zoo.Area.ha2    0.000    0.064    0.000    1.000
##
## Variances:
```

```
##                     Estimate  Std.Err  z-value  P(>|z|)
##    .Attendance2        0.279    0.028   10.018    0.000
##    .Total.Animals2     0.219    0.037    5.986    0.000
##    .Sp.Richness2       0.322    0.032    9.984    0.000
##    .Mean.Rap.Crck2     0.742    0.084    8.815    0.000
##     Mam.Sp.Rchnss2     0.998    0.115    8.669    0.000
##     Mn.Sp.BdyMsXA2     0.998    0.160    6.219    0.000
##     X10km_Pop2         0.998    0.174    5.745    0.000
##     GDP.Millions2      0.998    0.246    4.061    0.000
##     Zoo.Area.ha2       0.998    0.112    8.886    0.000
##
## R-Square:
##                     Estimate
##     Attendance2        0.689
##     Total.Animals2     0.783
##     Sp.Richness2       0.678
##     Mean.Rap.Crck2     0.257
```

```
# Generate fit indices
fitMeasures(fit.adj20A.final, c("agfi", "rmr", "srmr", "rmsea", "cfi", "nnfi", "tli"))
```

```
##  agfi   rmr  srmr rmsea   cfi  nnfi   tli
## 0.802 0.053 0.048 0.122 0.966 0.910 0.910
```

Tests of Mediation

It is clear from Model 20 that several pathways are mediated in the system. It is necessary to evaluate whether this mediation is appropriate by comparing models with complete, partial and no mediation. Here we show tests of mediation for three of these relationships.

Attendance2 ~ Mean.Sp.BodyMassXAbund2

Attendance2 ~ Mean.Sp.BodyMassXAbund2 is mediated by **Total.Animals2 ~ Mean.Sp.BodyMassXAbund2**. Below we compare models with complete (full) mediation, partial mediation (i.e. model 20) and no mediation. Results from this output suggest that partial mediation (model 20) is the superior model.

```
# Complete Mediation Model

FULL1A <- 'Attendance2 ~ Sp.Richness2 + Total.Animals2
+ Mam.Sp.Richness2 + Mean.Raup.Crick2
+ X10km_Pop2 + GDP.Millions2

Total.Animals2 ~ Zoo.Area.ha2 + Sp.Richness2 + Mean.Sp.BodyMassXAbund2 + GDP.Millions2
Sp.Richness2 ~ Mam.Sp.Richness2 + Mean.Sp.BodyMassXAbund2 + Zoo.Area.ha2
Mean.Raup.Crick2 ~ Mean.Sp.BodyMassXAbund2 + Zoo.Area.ha2'

FULL1A.fit <- sem(FULL1A, data = sem_attendance_data, fixed.x=FALSE)
design <- svydesign(ids = ~Country, nest=TRUE, data=sem_attendance_data)
FULL1A.fitadj <- lavaan.survey(lavaan.fit = FULL1A.fit, survey.design = design)

# Partial Mediation Model (MOdel 20)

PARTIAL1A.MOD20 <- 'Attendance2 ~ Sp.Richness2 + Total.Animals2
+ Mam.Sp.Richness2 + Mean.Sp.BodyMassXAbund2 + Mean.Raup.Crick2
```

```
+ X10km_Pop2 + GDP.Millions2

Total.Animals2 ~ Zoo.Area.ha2 + Sp.Richness2 + Mean.Sp.BodyMassXAbund2 + GDP.Millions2
Sp.Richness2 ~ Mam.Sp.Richness2 + Mean.Sp.BodyMassXAbund2 + Zoo.Area.ha2
Mean.Raup.Crick2 ~ Mean.Sp.BodyMassXAbund2 + Zoo.Area.ha2'

PARTIAL1A.MOD20.fit <- sem(PARTIAL1A.MOD20, data = sem_attendance_data, fixed.x=FALSE)
design <- svydesign(ids = ~Country, nest=TRUE, data=sem_attendance_data)
PARTIAL1A.MOD20.fitadj <- lavaan.survey(lavaan.fit = PARTIAL1A.MOD20.fit, survey.design = design)

# No Mediation Model

NONE1A <- 'Attendance2 ~ Sp.Richness2 + Total.Animals2
+ Mam.Sp.Richness2 + Mean.Sp.BodyMassXAbund2 + Mean.Raup.Crick2
+ X10km_Pop2 + GDP.Millions2

Total.Animals2 ~ Zoo.Area.ha2 + Sp.Richness2 + GDP.Millions2
Sp.Richness2 ~ Mam.Sp.Richness2 + Mean.Sp.BodyMassXAbund2 + Zoo.Area.ha2
Mean.Raup.Crick2 ~ Mean.Sp.BodyMassXAbund2 + Zoo.Area.ha2'

NONE1A.fit <- sem(NONE1A, data = sem_attendance_data, fixed.x=FALSE)
design <- svydesign(ids = ~Country, nest=TRUE, data=sem_attendance_data)
NONE1A.fitadj <- lavaan.survey(lavaan.fit = NONE1A.fit, survey.design = design)

aictab.lavaan(list(FULL1A.fit, PARTIAL1A.MOD20.fit, NONE1A.fit,
                   FULL1A.fitadj, PARTIAL1A.MOD20.fitadj, NONE1A.fitadj),
              c("full mediation", "partial mediation", "no mediation", "full mediation (nested)", "parti
```

```
##
## Model selection based on AICc:
##
##                              K    AICc Delta_AICc AICcWt Cum.Wt       LL
## partial mediation          35 9557.78       0.00      1      1 -4743.27
## partial mediation (nested) 44 9575.78      18.00      0      1 -4743.27
## no mediation               34 9587.39      29.61      0      1 -4759.15
## no mediation (nested)      43 9605.39      47.61      0      1 -4759.15
## full mediation             34 9651.99      94.21      0      1 -4791.45
## full mediation (nested)    43 9669.99     112.21      0      1 -4791.45
```

Attendance2 ~ Mean.Sp.BodyMassXAbund2

Attendance2 ~ Mean.Sp.BodyMassXAbund2 is also mediated by **Mean.Raup.Crick2 ~ Mean.Sp.BodyMassXAbund2**. Below we compare models with complete (full) mediation, partial mediation (i.e. model 20) and no mediation. Results from this output suggest that partial mediation (model 20) is the superior model.

```
# Complete Mediation Model

FULL2A <- 'Attendance2 ~ Sp.Richness2 + Total.Animals2
+ Mam.Sp.Richness2 + Mean.Raup.Crick2
+ X10km_Pop2 + GDP.Millions2

Total.Animals2 ~ Zoo.Area.ha2 + Sp.Richness2 + Mean.Sp.BodyMassXAbund2 + GDP.Millions2
Sp.Richness2 ~ Mam.Sp.Richness2 + Mean.Sp.BodyMassXAbund2 + Zoo.Area.ha2
```

```
Mean.Raup.Crick2 ~ Mean.Sp.BodyMassXAbund2 + Zoo.Area.ha2'

FULL2A.fit <- sem(FULL2A, data = sem_attendance_data, fixed.x=FALSE)
design <- svydesign(ids = ~Country, nest=TRUE, data=sem_attendance_data)
FULL2A.fitadj <- lavaan.survey(lavaan.fit = FULL2A.fit, survey.design = design)

# Partial Mediation Model (MOdel 20)

PARTIAL2A.MOD20 <- 'Attendance2 ~ Sp.Richness2 + Total.Animals2
+ Mam.Sp.Richness2 + Mean.Sp.BodyMassXAbund2 + Mean.Raup.Crick2
+ X10km_Pop2 + GDP.Millions2

Total.Animals2 ~ Zoo.Area.ha2 + Sp.Richness2 + Mean.Sp.BodyMassXAbund2 + GDP.Millions2
Sp.Richness2 ~ Mam.Sp.Richness2 + Mean.Sp.BodyMassXAbund2 + Zoo.Area.ha2
Mean.Raup.Crick2 ~ Mean.Sp.BodyMassXAbund2 + Zoo.Area.ha2'

PARTIAL2A.MOD20.fit <- sem(PARTIAL2A.MOD20, data = sem_attendance_data, fixed.x=FALSE)
design <- svydesign(ids = ~Country, nest=TRUE, data=sem_attendance_data)
PARTIAL2A.MOD20.fitadj <- lavaan.survey(lavaan.fit = PARTIAL2A.MOD20.fit, survey.design = design)

# No Mediation Model

NONE2A <- 'Attendance2 ~ Sp.Richness2 + Total.Animals2
+ Mam.Sp.Richness2 + Mean.Sp.BodyMassXAbund2 + Mean.Raup.Crick2
+ X10km_Pop2 + GDP.Millions2

Total.Animals2 ~ Zoo.Area.ha2 + Sp.Richness2 + Mean.Sp.BodyMassXAbund2 + GDP.Millions2
Sp.Richness2 ~ Mam.Sp.Richness2 + Mean.Sp.BodyMassXAbund2 + Zoo.Area.ha2
Mean.Raup.Crick2 ~ Zoo.Area.ha2'

NONE2A.fit <- sem(NONE2A, data = sem_attendance_data, fixed.x=FALSE)
design <- svydesign(ids = ~Country, nest=TRUE, data=sem_attendance_data)
NONE2A.fitadj <- lavaan.survey(lavaan.fit = NONE2A.fit, survey.design = design)

aictab.lavaan(list(FULL2A.fit, PARTIAL2A.MOD20.fit, NONE2A.fit,
                   FULL2A.fitadj, PARTIAL2A.MOD20.fitadj, NONE2A.fitadj),
              c("full mediation", "partial mediation", "no mediation", "full mediation (nested)", "parti
```

```
##
## Model selection based on AICc:
##
##                              K    AICc Delta_AICc AICcWt Cum.Wt       LL
## partial mediation           35 9557.78       0.00      1      1 -4743.27
## partial mediation (nested) 44 9575.78      18.00      0      1 -4743.27
## full mediation              34 9651.99      94.21      0      1 -4791.45
## full mediation (nested)    43 9669.99     112.21      0      1 -4791.45
## no mediation                34 9690.97     133.19      0      1 -4810.94
## no mediation (nested)      43 9708.97     151.19      0      1 -4810.94
```

Attendance2 ~ GDP.Millions2

Attendance2 ~ GDP.Millions2 is also mediated by **Total.Animals2 ~ GDP.Millions2**. Below we compare models with complete (full) mediation, partial mediation (i.e. model 20) and no mediation. Results

from this output suggest that partial mediation (model 20) is the superior model.

```
# Complete Mediation Model

FULL3A <- 'Attendance2 ~ Sp.Richness2 + Total.Animals2
+ Mam.Sp.Richness2 + Mean.Sp.BodyMassXAbund2 + Mean.Raup.Crick2
+ X10km_Pop2

Total.Animals2 ~ Zoo.Area.ha2 + Sp.Richness2 + Mean.Sp.BodyMassXAbund2 + GDP.Millions2
Sp.Richness2 ~ Mam.Sp.Richness2 + Mean.Sp.BodyMassXAbund2 + Zoo.Area.ha2
Mean.Raup.Crick2 ~ Mean.Sp.BodyMassXAbund2 + Zoo.Area.ha2'

FULL3A.fit <- sem(FULL3A, data = sem_attendance_data, fixed.x=FALSE)
design <- svydesign(ids = ~Country, nest=TRUE, data=sem_attendance_data)
FULL3A.fitadj <- lavaan.survey(lavaan.fit = FULL3A.fit, survey.design = design)

# Partial Mediation Model (MOdel 20)

PARTIAL3A.MOD20 <- 'Attendance2 ~ Sp.Richness2 + Total.Animals2
+ Mam.Sp.Richness2 + Mean.Sp.BodyMassXAbund2 + Mean.Raup.Crick2
+ X10km_Pop2 + GDP.Millions2

Total.Animals2 ~ Zoo.Area.ha2 + Sp.Richness2 + Mean.Sp.BodyMassXAbund2 + GDP.Millions2
Sp.Richness2 ~ Mam.Sp.Richness2 + Mean.Sp.BodyMassXAbund2 + Zoo.Area.ha2
Mean.Raup.Crick2 ~ Mean.Sp.BodyMassXAbund2 + Zoo.Area.ha2'

PARTIAL3A.MOD20.fit <- sem(PARTIAL3A.MOD20, data = sem_attendance_data, fixed.x=FALSE)
design <- svydesign(ids = ~Country, nest=TRUE, data=sem_attendance_data)
PARTIAL3A.MOD20.fitadj <- lavaan.survey(lavaan.fit = PARTIAL3A.MOD20.fit, survey.design = design)

# No Mediation Model

NONE3A <- 'Attendance2 ~ Sp.Richness2 + Total.Animals2
+ Mam.Sp.Richness2 + Mean.Sp.BodyMassXAbund2 + Mean.Raup.Crick2
+ X10km_Pop2 + GDP.Millions2

Total.Animals2 ~ Zoo.Area.ha2 + Sp.Richness2 + Mean.Sp.BodyMassXAbund2
Sp.Richness2 ~ Mam.Sp.Richness2 + Mean.Sp.BodyMassXAbund2 + Zoo.Area.ha2
Mean.Raup.Crick2 ~ Mean.Sp.BodyMassXAbund2 + Zoo.Area.ha2'

NONE3A.fit <- sem(NONE3A, data = sem_attendance_data, fixed.x=FALSE)
design <- svydesign(ids = ~Country, nest=TRUE, data=sem_attendance_data)
NONE3A.fitadj <- lavaan.survey(lavaan.fit = NONE3A.fit, survey.design = design)

aictab.lavaan(list(FULL3A.fit, PARTIAL3A.MOD20.fit, NONE3A.fit,
                   FULL3A.fitadj, PARTIAL3A.MOD20.fitadj, NONE3A.fitadj),
              c("full mediation", "partial mediation", "no mediation", "full mediation (nested)", "part
```

```
##
## Model selection based on AICc:
##
##                              K    AICc Delta_AICc AICcWt Cum.Wt       LL
## partial mediation           35 9557.78       0.00      1      1 -4743.27
## partial mediation (nested) 44 9575.78      18.00      0      1 -4743.27
## no mediation                34 9592.54      34.76      0      1 -4761.72
```

```
## full mediation             34 9594.10      36.33      0      1 -4762.51
## no mediation (nested)      43 9610.54      52.76      0      1 -4761.72
## full mediation (nested)    43 9612.10      54.33      0      1 -4762.51
```

Validation

Model validation is a process to rovide more evidence for the selected model (Fan *et al.*, 2016), yet it is often overlooked or not reported in SEM studies. It is achieved by testing the model with two or more random datasets from the same sample to ensure paramater estimates are similar when a model is based on different datasets from the same population. Here we validate our model using four random subsets (n = 200 each time) of the existing dataset.

Validation Set 1

Parameter estimates relatively constant (some p-value differences).

```r
# Chosen Attendance SEM (Abundance)
# Model 20

mod.20A.final <- 'Attendance2 ~ Sp.Richness2 + Total.Animals2
+ Mam.Sp.Richness2 + Mean.Sp.BodyMassXAbund2 + Mean.Raup.Crick2
+ X10km_Pop2 + GDP.Millions2

Total.Animals2 ~ Zoo.Area.ha2 + Sp.Richness2 + Mean.Sp.BodyMassXAbund2 + GDP.Millions2
Sp.Richness2 ~ Mam.Sp.Richness2 + Mean.Sp.BodyMassXAbund2 + Zoo.Area.ha2
Mean.Raup.Crick2 ~ Mean.Sp.BodyMassXAbund2 + Zoo.Area.ha2'

# Fit model and generate model summary
mod.20A.final.fit <- sem(mod.20A.final, data = validationset1, fixed.x=FALSE)
summary(mod.20A.final.fit, rsq = TRUE)
```

```
## lavaan (0.5-23.1097) converged normally after  29 iterations
##
##   Number of observations                           200
##
##   Estimator                                         ML
##   Minimum Function Test Statistic               49.560
##   Degrees of freedom                                10
##   P-value (Chi-square)                           0.000
##
## Parameter Estimates:
##
##   Information                                 Expected
##   Standard Errors                             Standard
##
## Regressions:
##                        Estimate  Std.Err  z-value  P(>|z|)
##   Attendance2 ~
##     Sp.Richness2         -0.182    0.086   -2.125    0.034
##     Total.Animals2        0.557    0.064    8.758    0.000
##     Mam.Sp.Rchnss2        0.170    0.071    2.397    0.017
##     Mn.Sp.BdyMsXA2        0.356    0.049    7.209    0.000
##     Mean.Rap.Crck2        0.117    0.042    2.803    0.005
```

```
##     X10km_Pop2          0.382    0.037   10.307    0.000
##     GDP.Millions2       0.163    0.038    4.237    0.000
##   Total.Animals2 ~
##     Zoo.Area.ha2        0.426    0.047    9.110    0.000
##     Sp.Richness2        0.716    0.040   18.012    0.000
##     Mn.Sp.BdyMsXA2     -0.196    0.046   -4.260    0.000
##     GDP.Millions2      -0.102    0.036   -2.836    0.005
##   Sp.Richness2 ~
##     Mam.Sp.Rchnss2      0.798    0.039   20.300    0.000
##     Mn.Sp.BdyMsXA2     -0.326    0.044   -7.379    0.000
##     Zoo.Area.ha2        0.001    0.049    0.028    0.978
##   Mean.Raup.Crick2 ~
##     Mn.Sp.BdyMsXA2     -0.684    0.073   -9.354    0.000
##     Zoo.Area.ha2        0.395    0.076    5.213    0.000
##
## Covariances:
##                              Estimate  Std.Err  z-value  P(>|z|)
##   Mam.Sp.Richness2 ~~
##     Mn.Sp.BdyMsXA2              0.125    0.067    1.855    0.064
##     X10km_Pop2                  0.285    0.074    3.849    0.000
##     GDP.Millions2              -0.099    0.067   -1.483    0.138
##     Zoo.Area.ha2                0.332    0.069    4.849    0.000
##   Mean.Sp.BodyMassXAbund2 ~~
##     X10km_Pop2                 -0.085    0.071   -1.190    0.234
##     GDP.Millions2              -0.048    0.067   -0.723    0.470
##     Zoo.Area.ha2                0.506    0.074    6.877    0.000
##   X10km_Pop2 ~~
##     GDP.Millions2               0.038    0.071    0.540    0.589
##     Zoo.Area.ha2               -0.052    0.069   -0.761    0.447
##   GDP.Millions2 ~~
##     Zoo.Area.ha2               -0.077    0.065   -1.191    0.234
##
## Variances:
##                    Estimate  Std.Err  z-value  P(>|z|)
##    .Attendance2       0.264    0.026   10.000    0.000
##    .Total.Animals2    0.241    0.024   10.000    0.000
##    .Sp.Richness2      0.251    0.025   10.000    0.000
##    .Mean.Rap.Crck2    0.696    0.070   10.000    0.000
##     Mam.Sp.Rchnss2    0.944    0.094   10.000    0.000
##     Mn.Sp.BdyMsXA2    0.942    0.094   10.000    0.000
##     X10km_Pop2        1.075    0.107   10.000    0.000
##     GDP.Millions2     0.943    0.094   10.000    0.000
##     Zoo.Area.ha2      0.879    0.088   10.000    0.000
##
## R-Square:
##                    Estimate
##     Attendance2       0.695
##     Total.Animals2    0.746
##     Sp.Richness2      0.717
##     Mean.Rap.Crck2    0.304
```

```
# Adjust for the nested nature of the data (institutions within countries)
# Fit model and generate model summary
design <- svydesign(ids = ~Country, nest=TRUE, data=validationset1)
```

```r
fit.adj20A.final <- lavaan.survey(lavaan.fit = mod.20A.final.fit, survey.design = design)
summary(fit.adj20A.final, rsq = TRUE)
```

```
## lavaan (0.5-23.1097) converged normally after  31 iterations
##
##   Number of observations                           200
##
##   Estimator                                         ML      Robust
##   Minimum Function Test Statistic               49.560      23.913
##   Degrees of freedom                                10          10
##   P-value (Chi-square)                           0.000       0.008
##   Scaling correction factor                                  2.072
##     for the Satorra-Bentler correction
##
## Parameter Estimates:
##
##   Information                                 Expected
##   Standard Errors                           Robust.sem
##
## Regressions:
##                      Estimate  Std.Err  z-value  P(>|z|)
##   Attendance2 ~
##     Sp.Richness2       -0.182    0.097   -1.867    0.062
##     Total.Animals2      0.557    0.071    7.848    0.000
##     Mam.Sp.Rchnss2      0.170    0.091    1.866    0.062
##     Mn.Sp.BdyMsXA2      0.356    0.052    6.879    0.000
##     Mean.Rap.Crck2      0.117    0.044    2.653    0.008
##     X10km_Pop2          0.382    0.050    7.623    0.000
##     GDP.Millions2       0.163    0.048    3.418    0.001
##   Total.Animals2 ~
##     Zoo.Area.ha2        0.426    0.083    5.107    0.000
##     Sp.Richness2        0.716    0.079    9.099    0.000
##     Mn.Sp.BdyMsXA2     -0.196    0.049   -4.022    0.000
##     GDP.Millions2      -0.102    0.075   -1.352    0.176
##   Sp.Richness2 ~
##     Mam.Sp.Rchnss2      0.798    0.072   11.098    0.000
##     Mn.Sp.BdyMsXA2     -0.326    0.054   -5.976    0.000
##     Zoo.Area.ha2        0.001    0.060    0.023    0.982
##   Mean.Raup.Crick2 ~
##     Mn.Sp.BdyMsXA2     -0.684    0.067  -10.171    0.000
##     Zoo.Area.ha2        0.395    0.103    3.851    0.000
##
## Covariances:
##                              Estimate  Std.Err  z-value  P(>|z|)
##   Mam.Sp.Richness2 ~~
##     Mn.Sp.BdyMsXA2              0.125    0.073    1.702    0.089
##     X10km_Pop2                  0.285    0.092    3.112    0.002
##     GDP.Millions2              -0.099    0.083   -1.197    0.231
##     Zoo.Area.ha2                0.332    0.086    3.867    0.000
##   Mean.Sp.BodyMassXAbund2 ~~
##     X10km_Pop2                 -0.085    0.095   -0.895    0.371
##     GDP.Millions2              -0.048    0.048   -1.006    0.314
##     Zoo.Area.ha2                0.506    0.115    4.391    0.000
##   X10km_Pop2 ~~
```

```
##     GDP.Millions2               0.038    0.078    0.491    0.624
##     Zoo.Area.ha2               -0.052    0.089   -0.588    0.556
##   GDP.Millions2 ~~
##     Zoo.Area.ha2               -0.077    0.058   -1.334    0.182
##
## Intercepts:
##                    Estimate  Std.Err  z-value  P(>|z|)
##    .Attendance2       0.017    0.041    0.426    0.670
##    .Total.Animals2    0.035    0.073    0.481    0.631
##    .Sp.Richness2      0.013    0.071    0.188    0.851
##    .Mean.Rap.Crck2    0.005    0.096    0.047    0.963
##     Mam.Sp.Rchnss2    0.050    0.079    0.638    0.524
##     Mn.Sp.BdyMsXA2   -0.014    0.078   -0.184    0.854
##     X10km_Pop2        0.035    0.107    0.333    0.739
##     GDP.Millions2     0.059    0.360    0.163    0.870
##     Zoo.Area.ha2      0.014    0.080    0.177    0.859
##
## Variances:
##                    Estimate  Std.Err  z-value  P(>|z|)
##    .Attendance2       0.264    0.037    7.135    0.000
##    .Total.Animals2    0.241    0.031    7.662    0.000
##    .Sp.Richness2      0.251    0.027    9.396    0.000
##    .Mean.Rap.Crck2    0.696    0.058   12.018    0.000
##     Mam.Sp.Rchnss2    0.944    0.159    5.919    0.000
##     Mn.Sp.BdyMsXA2    0.942    0.156    6.049    0.000
##     X10km_Pop2        1.075    0.246    4.366    0.000
##     GDP.Millions2     0.943    0.243    3.880    0.000
##     Zoo.Area.ha2      0.879    0.107    8.254    0.000
##
## R-Square:
##                    Estimate
##     Attendance2       0.695
##     Total.Animals2    0.746
##     Sp.Richness2      0.717
##     Mean.Rap.Crck2    0.304
```

```
# Generate fit indices
fitMeasures(fit.adj20A.final, c("agfi", "rmr", "srmr", "rmsea", "cfi", "nnfi", "tli"))
```

```
##  agfi   rmr  srmr rmsea   cfi  nnfi   tli
## 0.743 0.054 0.050 0.141 0.955 0.883 0.883
```

Validation Set 2

Parameter estimates relatively constant (some p-value differences).

```
# Chosen Attendance SEM (Abundance)
# Model 20

mod.20A.final <- 'Attendance2 ~ Sp.Richness2 + Total.Animals2
+ Mam.Sp.Richness2 + Mean.Sp.BodyMassXAbund2 + Mean.Raup.Crick2
+ X10km_Pop2 + GDP.Millions2

Total.Animals2 ~ Zoo.Area.ha2 + Sp.Richness2 + Mean.Sp.BodyMassXAbund2 + GDP.Millions2
```

```
Sp.Richness2 ~ Mam.Sp.Richness2 + Mean.Sp.BodyMassXAbund2 + Zoo.Area.ha2
Mean.Raup.Crick2 ~ Mean.Sp.BodyMassXAbund2 + Zoo.Area.ha2'

# Fit model and generate model summary
mod.20A.final.fit <- sem(mod.20A.final, data = validationset2, fixed.x=FALSE)
summary(mod.20A.final.fit, rsq = TRUE)
```

```
## lavaan (0.5-23.1097) converged normally after  30 iterations
##
##   Number of observations                           200
##
##   Estimator                                         ML
##   Minimum Function Test Statistic               59.562
##   Degrees of freedom                                10
##   P-value (Chi-square)                           0.000
##
## Parameter Estimates:
##
##   Information                                 Expected
##   Standard Errors                             Standard
##
## Regressions:
##                    Estimate  Std.Err  z-value  P(>|z|)
##   Attendance2 ~
##     Sp.Richness2     -0.135    0.092   -1.469    0.142
##     Total.Animals2    0.556    0.081    6.903    0.000
##     Mam.Sp.Rchnss2    0.041    0.066    0.611    0.541
##     Mn.Sp.BdyMsXA2    0.337    0.049    6.940    0.000
##     Mean.Rap.Crck2    0.160    0.041    3.892    0.000
##     X10km_Pop2        0.487    0.040   12.249    0.000
##     GDP.Millions2     0.151    0.038    3.973    0.000
##   Total.Animals2 ~
##     Zoo.Area.ha2      0.245    0.036    6.717    0.000
##     Sp.Richness2      0.762    0.032   23.816    0.000
##     Mn.Sp.BdyMsXA2   -0.129    0.035   -3.669    0.000
##     GDP.Millions2    -0.143    0.029   -4.919    0.000
##   Sp.Richness2 ~
##     Mam.Sp.Rchnss2    0.793    0.045   17.651    0.000
##     Mn.Sp.BdyMsXA2   -0.457    0.046  -10.025    0.000
##     Zoo.Area.ha2      0.159    0.049    3.234    0.001
##   Mean.Raup.Crick2 ~
##     Mn.Sp.BdyMsXA2   -0.497    0.070   -7.135    0.000
##     Zoo.Area.ha2      0.234    0.071    3.286    0.001
##
## Covariances:
##                            Estimate  Std.Err  z-value  P(>|z|)
##   Mam.Sp.Richness2 ~~
##     Mn.Sp.BdyMsXA2            0.277    0.075    3.704    0.000
##     X10km_Pop2                0.301    0.074    4.078    0.000
##     GDP.Millions2            -0.014    0.074   -0.191    0.848
##     Zoo.Area.ha2              0.409    0.076    5.354    0.000
##   Mean.Sp.BodyMassXAbund2 ~~
##     X10km_Pop2                0.175    0.072    2.419    0.016
##     GDP.Millions2            -0.137    0.075   -1.832    0.067
```

```
##     Zoo.Area.ha2                0.453    0.078    5.799    0.000
##   X10km_Pop2 ~~
##     GDP.Millions2              -0.086    0.073   -1.182    0.237
##     Zoo.Area.ha2                0.048    0.070    0.689    0.491
##   GDP.Millions2 ~~
##     Zoo.Area.ha2                0.005    0.073    0.072    0.943
##
## Variances:
##                    Estimate  Std.Err  z-value  P(>|z|)
##    .Attendance2       0.278    0.028   10.000    0.000
##    .Total.Animals2    0.176    0.018   10.000    0.000
##    .Sp.Richness2      0.337    0.034   10.000    0.000
##    .Mean.Rap.Crck2    0.796    0.080   10.000    0.000
##     Mam.Sp.Rchnss2    1.013    0.101   10.000    0.000
##     Mn.Sp.BdyMsXA2    1.029    0.103   10.000    0.000
##     X10km_Pop2        0.985    0.098   10.000    0.000
##     GDP.Millions2     1.074    0.107   10.000    0.000
##     Zoo.Area.ha2      0.987    0.099   10.000    0.000
##
## R-Square:
##                    Estimate
##     Attendance2       0.692
##     Total.Animals2    0.819
##     Sp.Richness2      0.679
##     Mean.Rap.Crck2    0.203
```

```r
# Adjust for the nested nature of the data (institutions within countries)
# Fit model and generate model summary
design <- svydesign(ids = ~Country, nest=TRUE, data=validationset2)
fit.adj20A.final <- lavaan.survey(lavaan.fit = mod.20A.final.fit, survey.design = design)
summary(fit.adj20A.final, rsq = TRUE)
```

```
## lavaan (0.5-23.1097) converged normally after  31 iterations
##
##   Number of observations                           200
##
##   Estimator                                         ML      Robust
##   Minimum Function Test Statistic               59.562      23.928
##   Degrees of freedom                                10          10
##   P-value (Chi-square)                           0.000       0.008
##   Scaling correction factor                                  2.489
##     for the Satorra-Bentler correction
##
## Parameter Estimates:
##
##   Information                                 Expected
##   Standard Errors                           Robust.sem
##
## Regressions:
##                      Estimate  Std.Err  z-value  P(>|z|)
##   Attendance2 ~
##     Sp.Richness2       -0.135    0.082   -1.657    0.098
##     Total.Animals2      0.556    0.058    9.569    0.000
##     Mam.Sp.Rchnss2      0.041    0.068    0.599    0.549
##     Mn.Sp.BdyMsXA2      0.337    0.036    9.283    0.000
```

```
##     Mean.Rap.Crck2      0.160    0.027    5.925    0.000
##     X10km_Pop2          0.487    0.052    9.402    0.000
##     GDP.Millions2       0.151    0.032    4.681    0.000
##   Total.Animals2 ~
##     Zoo.Area.ha2        0.245    0.055    4.483    0.000
##     Sp.Richness2        0.762    0.058   13.083    0.000
##     Mn.Sp.BdyMsXA2     -0.129    0.048   -2.677    0.007
##     GDP.Millions2      -0.143    0.065   -2.196    0.028
##   Sp.Richness2 ~
##     Mam.Sp.Rchnss2      0.793    0.065   12.123    0.000
##     Mn.Sp.BdyMsXA2     -0.457    0.067   -6.812    0.000
##     Zoo.Area.ha2        0.159    0.060    2.654    0.008
##   Mean.Raup.Crick2 ~
##     Mn.Sp.BdyMsXA2     -0.497    0.108   -4.620    0.000
##     Zoo.Area.ha2        0.234    0.092    2.533    0.011
##
## Covariances:
##                            Estimate  Std.Err  z-value  P(>|z|)
##   Mam.Sp.Richness2 ~~
##     Mn.Sp.BdyMsXA2            0.277    0.110    2.518    0.012
##     X10km_Pop2                0.301    0.091    3.310    0.001
##     GDP.Millions2            -0.014    0.077   -0.183    0.855
##     Zoo.Area.ha2              0.409    0.079    5.182    0.000
##   Mean.Sp.BodyMassXAbund2 ~~
##     X10km_Pop2                0.175    0.123    1.421    0.155
##     GDP.Millions2            -0.137    0.053   -2.586    0.010
##     Zoo.Area.ha2              0.453    0.096    4.739    0.000
##   X10km_Pop2 ~~
##     GDP.Millions2            -0.086    0.073   -1.189    0.235
##     Zoo.Area.ha2              0.048    0.076    0.636    0.525
##   GDP.Millions2 ~~
##     Zoo.Area.ha2              0.005    0.049    0.106    0.916
##
## Intercepts:
##                    Estimate  Std.Err  z-value  P(>|z|)
##    .Attendance2      -0.031    0.042   -0.739    0.460
##    .Total.Animals2   -0.029    0.066   -0.448    0.654
##    .Sp.Richness2     -0.001    0.071   -0.020    0.984
##    .Mean.Rap.Crck2    0.015    0.100    0.148    0.882
##     Mam.Sp.Rchnss2   -0.018    0.101   -0.175    0.861
##     Mn.Sp.BdyMsXA2   -0.015    0.094   -0.162    0.872
##     X10km_Pop2       -0.013    0.129   -0.104    0.917
##     GDP.Millions2    -0.044    0.340   -0.131    0.896
##     Zoo.Area.ha2     -0.028    0.083   -0.331    0.741
##
## Variances:
##                    Estimate  Std.Err  z-value  P(>|z|)
##    .Attendance2       0.278    0.033    8.526    0.000
##    .Total.Animals2    0.176    0.042    4.212    0.000
##    .Sp.Richness2      0.337    0.039    8.536    0.000
##    .Mean.Rap.Crck2    0.796    0.108    7.342    0.000
##     Mam.Sp.Rchnss2    1.013    0.121    8.400    0.000
##     Mn.Sp.BdyMsXA2    1.029    0.159    6.453    0.000
##     X10km_Pop2        0.985    0.160    6.171    0.000
```

```
##     GDP.Millions2     1.074    0.270    3.975    0.000
##     Zoo.Area.ha2      0.987    0.126    7.849    0.000
##
## R-Square:
##                    Estimate
##     Attendance2       0.692
##     Total.Animals2    0.819
##     Sp.Richness2      0.679
##     Mean.Rap.Crck2    0.203
```

```
# Generate fit indices
fitMeasures(fit.adj20A.final, c("agfi", "rmr", "srmr", "rmsea", "cfi", "nnfi", "tli"))
```

```
##  agfi   rmr  srmr rmsea   cfi  nnfi   tli
## 0.665 0.063 0.057 0.157 0.945 0.858 0.858
```

Validation Set 3

Parameter estimates relatively constant (some p-value differences).

```
# Chosen Attendance SEM (Abundance)
# Model 20

mod.20A.final <- 'Attendance2 ~ Sp.Richness2 + Total.Animals2
+ Mam.Sp.Richness2 + Mean.Sp.BodyMassXAbund2 + Mean.Raup.Crick2
+ X10km_Pop2 + GDP.Millions2

Total.Animals2 ~ Zoo.Area.ha2 + Sp.Richness2 + Mean.Sp.BodyMassXAbund2 + GDP.Millions2
Sp.Richness2 ~ Mam.Sp.Richness2 + Mean.Sp.BodyMassXAbund2 + Zoo.Area.ha2
Mean.Raup.Crick2 ~ Mean.Sp.BodyMassXAbund2 + Zoo.Area.ha2'

# Fit model and generate model summary
mod.20A.final.fit <- sem(mod.20A.final, data = validationset3, fixed.x=FALSE)
summary(mod.20A.final.fit, rsq = TRUE)
```

```
## lavaan (0.5-23.1097) converged normally after  32 iterations
##
##   Number of observations                           200
##
##   Estimator                                         ML
##   Minimum Function Test Statistic               41.351
##   Degrees of freedom                                10
##   P-value (Chi-square)                           0.000
##
## Parameter Estimates:
##
##   Information                                 Expected
##   Standard Errors                             Standard
##
## Regressions:
##                     Estimate  Std.Err  z-value  P(>|z|)
##   Attendance2 ~
##     Sp.Richness2      -0.144    0.090   -1.599    0.110
##     Total.Animals2     0.589    0.070    8.459    0.000
##     Mam.Sp.Rchnss2     0.055    0.069    0.803    0.422
```

```
##     Mn.Sp.BdyMsXA2      0.304    0.052    5.834    0.000
##     Mean.Rap.Crck2      0.118    0.041    2.883    0.004
##     X10km_Pop2          0.432    0.039   10.941    0.000
##     GDP.Millions2       0.168    0.037    4.554    0.000
##   Total.Animals2 ~
##     Zoo.Area.ha2        0.301    0.041    7.273    0.000
##     Sp.Richness2        0.769    0.039   19.742    0.000
##     Mn.Sp.BdyMsXA2     -0.123    0.042   -2.954    0.003
##     GDP.Millions2      -0.134    0.033   -4.107    0.000
##   Sp.Richness2 ~
##     Mam.Sp.Rchnss2      0.764    0.042   18.189    0.000
##     Mn.Sp.BdyMsXA2     -0.473    0.043  -10.903    0.000
##     Zoo.Area.ha2        0.099    0.046    2.155    0.031
##   Mean.Raup.Crick2 ~
##     Mn.Sp.BdyMsXA2     -0.564    0.072   -7.852    0.000
##     Zoo.Area.ha2        0.262    0.071    3.671    0.000
##
## Covariances:
##                           Estimate  Std.Err  z-value  P(>|z|)
##   Mam.Sp.Richness2 ~~
##     Mn.Sp.BdyMsXA2            0.305    0.074    4.144    0.000
##     X10km_Pop2                0.278    0.071    3.889    0.000
##     GDP.Millions2            -0.049    0.072   -0.677    0.499
##     Zoo.Area.ha2              0.441    0.077    5.703    0.000
##   Mean.Sp.BodyMassXAbund2 ~~
##     X10km_Pop2               -0.014    0.069   -0.206    0.837
##     GDP.Millions2            -0.081    0.073   -1.108    0.268
##     Zoo.Area.ha2              0.516    0.080    6.426    0.000
##   X10km_Pop2 ~~
##     GDP.Millions2             0.016    0.071    0.222    0.825
##     Zoo.Area.ha2             -0.016    0.070   -0.234    0.815
##   GDP.Millions2 ~~
##     Zoo.Area.ha2             -0.048    0.073   -0.651    0.515
##
## Variances:
##                    Estimate  Std.Err  z-value  P(>|z|)
##    .Attendance2       0.269    0.027   10.000    0.000
##    .Total.Animals2    0.223    0.022   10.000    0.000
##    .Sp.Richness2      0.277    0.028   10.000    0.000
##    .Mean.Rap.Crck2    0.768    0.077   10.000    0.000
##     Mam.Sp.Rchnss2    0.986    0.099   10.000    0.000
##     Mn.Sp.BdyMsXA2    1.005    0.101   10.000    0.000
##     X10km_Pop2        0.958    0.096   10.000    0.000
##     GDP.Millions2     1.058    0.106   10.000    0.000
##     Zoo.Area.ha2      1.016    0.102   10.000    0.000
##
## R-Square:
##                    Estimate
##     Attendance2       0.681
##     Total.Animals2    0.768
##     Sp.Richness2      0.687
##     Mean.Rap.Crck2    0.236
```

```r
# Adjust for the nested nature of the data (institutions within countries)
# Fit model and generate model summary
design <- svydesign(ids = ~Country, nest=TRUE, data=validationset3)
fit.adj20A.final <- lavaan.survey(lavaan.fit = mod.20A.final.fit, survey.design = design)
summary(fit.adj20A.final, rsq = TRUE)
```

```
## lavaan (0.5-23.1097) converged normally after  31 iterations
##
##   Number of observations                           200
##
##   Estimator                                         ML      Robust
##   Minimum Function Test Statistic               41.351      14.272
##   Degrees of freedom                                10          10
##   P-value (Chi-square)                           0.000       0.161
##   Scaling correction factor                                  2.897
##     for the Satorra-Bentler correction
##
## Parameter Estimates:
##
##   Information                                 Expected
##   Standard Errors                           Robust.sem
##
## Regressions:
##                    Estimate  Std.Err  z-value  P(>|z|)
##   Attendance2 ~
##     Sp.Richness2     -0.144    0.076   -1.902    0.057
##     Total.Animals2    0.589    0.050   11.742    0.000
##     Mam.Sp.Rchnss2    0.055    0.077    0.713    0.476
##     Mn.Sp.BdyMsXA2    0.304    0.046    6.567    0.000
##     Mean.Rap.Crck2    0.118    0.039    3.002    0.003
##     X10km_Pop2        0.432    0.065    6.614    0.000
##     GDP.Millions2     0.168    0.032    5.288    0.000
##   Total.Animals2 ~
##     Zoo.Area.ha2      0.301    0.064    4.673    0.000
##     Sp.Richness2      0.769    0.099    7.750    0.000
##     Mn.Sp.BdyMsXA2   -0.123    0.050   -2.466    0.014
##     GDP.Millions2    -0.134    0.073   -1.843    0.065
##   Sp.Richness2 ~
##     Mam.Sp.Rchnss2    0.764    0.068   11.295    0.000
##     Mn.Sp.BdyMsXA2   -0.473    0.066   -7.144    0.000
##     Zoo.Area.ha2      0.099    0.050    1.975    0.048
##   Mean.Raup.Crick2 ~
##     Mn.Sp.BdyMsXA2   -0.564    0.099   -5.694    0.000
##     Zoo.Area.ha2      0.262    0.075    3.491    0.000
##
## Covariances:
##                            Estimate  Std.Err  z-value  P(>|z|)
##   Mam.Sp.Richness2 ~~
##     Mn.Sp.BdyMsXA2             0.305    0.090    3.372    0.001
##     X10km_Pop2                 0.278    0.080    3.484    0.000
##     GDP.Millions2             -0.049    0.072   -0.675    0.499
##     Zoo.Area.ha2               0.441    0.078    5.680    0.000
##   Mean.Sp.BodyMassXAbund2 ~~
##     X10km_Pop2                -0.014    0.094   -0.153    0.879
```

```
##     GDP.Millions2             -0.081    0.079   -1.029    0.304
##     Zoo.Area.ha2               0.516    0.094    5.504    0.000
##   X10km_Pop2 ~~
##     GDP.Millions2              0.016    0.071    0.224    0.823
##     Zoo.Area.ha2              -0.016    0.116   -0.141    0.888
##   GDP.Millions2 ~~
##     Zoo.Area.ha2              -0.048    0.062   -0.773    0.439
##
## Intercepts:
##                    Estimate  Std.Err  z-value  P(>|z|)
##    .Attendance2      -0.002    0.038   -0.048    0.962
##    .Total.Animals2    0.007    0.080    0.085    0.932
##    .Sp.Richness2      0.001    0.076    0.008    0.993
##    .Mean.Rap.Crck2    0.088    0.109    0.811    0.417
##     Mam.Sp.Rchnss2   -0.032    0.086   -0.373    0.709
##     Mn.Sp.BdyMsXA2   -0.024    0.102   -0.232    0.816
##     X10km_Pop2        0.028    0.103    0.268    0.789
##     GDP.Millions2     0.013    0.363    0.036    0.971
##     Zoo.Area.ha2     -0.076    0.079   -0.969    0.333
##
## Variances:
##                    Estimate  Std.Err  z-value  P(>|z|)
##    .Attendance2       0.269    0.038    7.071    0.000
##    .Total.Animals2    0.223    0.059    3.748    0.000
##    .Sp.Richness2      0.277    0.030    9.181    0.000
##    .Mean.Rap.Crck2    0.768    0.115    6.701    0.000
##     Mam.Sp.Rchnss2    0.986    0.103    9.607    0.000
##     Mn.Sp.BdyMsXA2    1.005    0.156    6.454    0.000
##     X10km_Pop2        0.958    0.202    4.733    0.000
##     GDP.Millions2     1.058    0.254    4.172    0.000
##     Zoo.Area.ha2      1.016    0.150    6.798    0.000
##
## R-Square:
##                    Estimate
##     Attendance2       0.681
##     Total.Animals2    0.768
##     Sp.Richness2      0.687
##     Mean.Rap.Crck2    0.236
```

```
# Generate fit indices
fitMeasures(fit.adj20A.final, c("agfi", "rmr", "srmr", "rmsea", "cfi", "nnfi", "tli"))
```

```
##  agfi   rmr  srmr rmsea   cfi  nnfi   tli
## 0.757 0.052 0.050 0.125 0.963 0.903 0.903
```

Validation Set 4

Parameter estimates relatively constant (some p-value differences).

```
# Chosen Attendance SEM (Abundance)
# Model 20

mod.20A.final <- 'Attendance2 ~ Sp.Richness2 + Total.Animals2
+ Mam.Sp.Richness2 + Mean.Sp.BodyMassXAbund2 + Mean.Raup.Crick2
```

```r
+ X10km_Pop2 + GDP.Millions2

Total.Animals2 ~ Zoo.Area.ha2 + Sp.Richness2 + Mean.Sp.BodyMassXAbund2 + GDP.Millions2
Sp.Richness2 ~ Mam.Sp.Richness2 + Mean.Sp.BodyMassXAbund2 + Zoo.Area.ha2
Mean.Raup.Crick2 ~ Mean.Sp.BodyMassXAbund2 + Zoo.Area.ha2'

# Fit model and generate model summary
mod.20A.final.fit <- sem(mod.20A.final, data = validationset4, fixed.x=FALSE)
summary(mod.20A.final.fit, rsq = TRUE)
```

```
## lavaan (0.5-23.1097) converged normally after  31 iterations
##
##   Number of observations                           200
##
##   Estimator                                         ML
##   Minimum Function Test Statistic               35.557
##   Degrees of freedom                                10
##   P-value (Chi-square)                           0.000
##
## Parameter Estimates:
##
##   Information                                 Expected
##   Standard Errors                             Standard
##
## Regressions:
##                      Estimate  Std.Err  z-value  P(>|z|)
##   Attendance2 ~
##     Sp.Richness2       -0.247    0.089   -2.776    0.005
##     Total.Animals2      0.627    0.073    8.620    0.000
##     Mam.Sp.Rchnss2      0.149    0.071    2.087    0.037
##     Mn.Sp.BdyMsXA2      0.380    0.051    7.512    0.000
##     Mean.Rap.Crck2      0.101    0.043    2.361    0.018
##     X10km_Pop2          0.428    0.037   11.417    0.000
##     GDP.Millions2       0.173    0.040    4.284    0.000
##   Total.Animals2 ~
##     Zoo.Area.ha2        0.322    0.043    7.547    0.000
##     Sp.Richness2        0.754    0.034   22.128    0.000
##     Mn.Sp.BdyMsXA2     -0.190    0.044   -4.356    0.000
##     GDP.Millions2      -0.129    0.034   -3.797    0.000
##   Sp.Richness2 ~
##     Mam.Sp.Rchnss2      0.852    0.044   19.544    0.000
##     Mn.Sp.BdyMsXA2     -0.470    0.048   -9.706    0.000
##     Zoo.Area.ha2        0.120    0.051    2.346    0.019
##   Mean.Raup.Crick2 ~
##     Mn.Sp.BdyMsXA2     -0.639    0.073   -8.732    0.000
##     Zoo.Area.ha2        0.339    0.075    4.553    0.000
##
## Covariances:
##                              Estimate  Std.Err  z-value  P(>|z|)
##   Mam.Sp.Richness2 ~~
##     Mn.Sp.BdyMsXA2              0.148    0.071    2.092    0.036
##     X10km_Pop2                  0.258    0.076    3.403    0.001
##     GDP.Millions2              -0.057    0.068   -0.829    0.407
##     Zoo.Area.ha2                0.292    0.072    4.070    0.000
```

```
##   Mean.Sp.BodyMassXAbund2 ~~
##     X10km_Pop2                0.006    0.077    0.084    0.933
##     GDP.Millions2            -0.126    0.072   -1.759    0.079
##     Zoo.Area.ha2              0.570    0.082    6.944    0.000
##   X10km_Pop2 ~~
##     GDP.Millions2            -0.119    0.075   -1.583    0.113
##     Zoo.Area.ha2             -0.025    0.075   -0.331    0.741
##   GDP.Millions2 ~~
##     Zoo.Area.ha2             -0.020    0.070   -0.287    0.774
##
## Variances:
##                    Estimate  Std.Err  z-value  P(>|z|)
##    .Attendance2       0.296    0.030   10.000    0.000
##    .Total.Animals2    0.222    0.022   10.000    0.000
##    .Sp.Richness2      0.329    0.033   10.000    0.000
##    .Mean.Rap.Crck2    0.752    0.075   10.000    0.000
##     Mam.Sp.Rchnss2    0.952    0.095   10.000    0.000
##     Mn.Sp.BdyMsXA2    1.029    0.103   10.000    0.000
##     X10km_Pop2        1.140    0.114   10.000    0.000
##     GDP.Millions2     0.980    0.098   10.000    0.000
##     Zoo.Area.ha2      0.992    0.099   10.000    0.000
##
## R-Square:
##                    Estimate
##     Attendance2       0.691
##     Total.Animals2    0.795
##     Sp.Richness2      0.711
##     Mean.Rap.Crck2    0.277
```

```
# Adjust for the nested nature of the data (institutions within countries)
# Fit model and generate model summary
design <- svydesign(ids = ~Country, nest=TRUE, data=validationset4)
fit.adj20A.final <- lavaan.survey(lavaan.fit = mod.20A.final.fit, survey.design = design)
summary(fit.adj20A.final, rsq = TRUE)
```

```
## lavaan (0.5-23.1097) converged normally after  32 iterations
##
##   Number of observations                           200
##
##   Estimator                                         ML      Robust
##   Minimum Function Test Statistic               35.557      16.944
##   Degrees of freedom                                10          10
##   P-value (Chi-square)                           0.000       0.076
##   Scaling correction factor                                  2.098
##     for the Satorra-Bentler correction
##
## Parameter Estimates:
##
##   Information                                 Expected
##   Standard Errors                           Robust.sem
##
## Regressions:
##                      Estimate  Std.Err  z-value  P(>|z|)
##   Attendance2 ~
##     Sp.Richness2       -0.247    0.093   -2.648    0.008
```

```
##     Total.Animals2      0.627    0.054   11.549    0.000
##     Mam.Sp.Rchnss2      0.149    0.082    1.820    0.069
##     Mn.Sp.BdyMsXA2      0.380    0.050    7.631    0.000
##     Mean.Rap.Crck2      0.101    0.042    2.384    0.017
##     X10km_Pop2          0.428    0.044    9.789    0.000
##     GDP.Millions2       0.173    0.043    4.042    0.000
##   Total.Animals2 ~
##     Zoo.Area.ha2        0.322    0.046    6.970    0.000
##     Sp.Richness2        0.754    0.040   18.897    0.000
##     Mn.Sp.BdyMsXA2     -0.190    0.038   -5.032    0.000
##     GDP.Millions2      -0.129    0.074   -1.738    0.082
##   Sp.Richness2 ~
##     Mam.Sp.Rchnss2      0.852    0.097    8.783    0.000
##     Mn.Sp.BdyMsXA2     -0.470    0.044  -10.568    0.000
##     Zoo.Area.ha2        0.120    0.062    1.946    0.052
##   Mean.Raup.Crick2 ~
##     Mn.Sp.BdyMsXA2     -0.639    0.096   -6.629    0.000
##     Zoo.Area.ha2        0.339    0.067    5.058    0.000
##
## Covariances:
##                             Estimate  Std.Err  z-value  P(>|z|)
##   Mam.Sp.Richness2 ~~
##     Mn.Sp.BdyMsXA2             0.148    0.109    1.362    0.173
##     X10km_Pop2                 0.258    0.095    2.716    0.007
##     GDP.Millions2             -0.057    0.093   -0.612    0.540
##     Zoo.Area.ha2               0.292    0.087    3.343    0.001
##   Mean.Sp.BodyMassXAbund2 ~~
##     X10km_Pop2                 0.006    0.105    0.061    0.952
##     GDP.Millions2             -0.126    0.050   -2.527    0.012
##     Zoo.Area.ha2               0.570    0.136    4.203    0.000
##   X10km_Pop2 ~~
##     GDP.Millions2             -0.119    0.071   -1.673    0.094
##     Zoo.Area.ha2              -0.025    0.083   -0.301    0.763
##   GDP.Millions2 ~~
##     Zoo.Area.ha2              -0.020    0.074   -0.269    0.788
##
## Intercepts:
##                    Estimate  Std.Err  z-value  P(>|z|)
##    .Attendance2      -0.024    0.035   -0.690    0.490
##    .Total.Animals2    0.004    0.068    0.057    0.955
##    .Sp.Richness2      0.024    0.071    0.335    0.738
##    .Mean.Rap.Crck2   -0.038    0.097   -0.390    0.696
##     Mam.Sp.Rchnss2    0.013    0.099    0.130    0.896
##     Mn.Sp.BdyMsXA2    0.018    0.096    0.187    0.852
##     X10km_Pop2       -0.008    0.133   -0.059    0.953
##     GDP.Millions2    -0.027    0.338   -0.079    0.937
##     Zoo.Area.ha2      0.067    0.083    0.813    0.417
##
## Variances:
##                    Estimate  Std.Err  z-value  P(>|z|)
##    .Attendance2       0.296    0.039    7.533    0.000
##    .Total.Animals2    0.222    0.032    7.008    0.000
##    .Sp.Richness2      0.329    0.042    7.819    0.000
##    .Mean.Rap.Crck2    0.752    0.089    8.428    0.000
```

```
##     Mam.Sp.Rchnss2    0.952    0.192    4.963    0.000
##     Mn.Sp.BdyMsXA2    1.029    0.201    5.117    0.000
##     X10km_Pop2        1.140    0.196    5.819    0.000
##     GDP.Millions2     0.980    0.264    3.708    0.000
##     Zoo.Area.ha2      0.992    0.107    9.300    0.000
##
## R-Square:
##                    Estimate
##     Attendance2       0.691
##     Total.Animals2    0.795
##     Sp.Richness2      0.711
##     Mean.Rap.Crck2    0.277
```

```
# Generate fit indices
fitMeasures(fit.adj20A.final, c("agfi", "rmr", "srmr", "rmsea", "cfi", "nnfi", "tli"))
```

```
##  agfi   rmr  srmr rmsea   cfi  nnfi   tli
## 0.805 0.056 0.048 0.113 0.971 0.925 0.925
```

Across all the validation models while we see some differences regarding p-values, as would be expected we see qualitatively similar parameter estimates across the four validation sets.

In Situ SEM

Two distinct SEM frameworks were tested, the Attendance model and the In Situ model. The Attendance model tested the relationship between visitor attendance and all the various specified variables for 458 institutions globally. This model did not include any in situ contribution data. The In Situ model tested the relationship between visitor attendance, in situ contributions and all the various specified variables for a subset of 119 institutions in North America for which in situ contribution data was available. The results of the Attendance model were used to guide the development of the Attendance linked pathways in the In Situ model as the larger sample size of the Attendance model had higher power

Similar to the Attendance Model, two In Situ Models were developed, one based on species presence-absence per institution and the the other which also conisdered species' population sizes per institution. This was important for calculating mean species body mass per institution, the proportion of threatened species per institution etc. First we develop the model based on species presence-absence.

Species Presence - Absence

Model 1 (a priori meta-model)

We now define our starting model for the SEM based on the a priori meta-model which is constructed based on evidence from the litrature and combined with proposed causal hypotheses. This model includes all previously identified evidence-based relationships and the numerous proposed causal pathways to visitor attendance. The development of this model can be found in the Supplementary information. Here we use the same proposed predictors of attendance for the in situ contributions. We use the species presence-absence Attendance Model to guide model development and identify relationships to visitor attendance, due to the larger sample size and therefore higher explanatory power in that model.

```
# In Situ SEM (Presence-Absence)

# Model 1
# Based on a priori meta-model and results from the species presence-absence Attendance Model
```

```
mod.IS1 <- 'Attendance2 ~ Zoo.Area.ha2 + Sp.Richness2 + Total.Animals2
+ Mam.Sp.Richness2 + Prop.Mam.Sp2 + Prop.Threat.Sp2
+ Mean.Sp.BodyMass2 + Brillouin.Index2 + Mean.Raup.Crick2
+ X50km_Pop2 + X10km_Pop2

Total.Animals2 ~ Zoo.Area.ha2 + Sp.Richness2
Sp.Richness2 ~ Mam.Sp.Richness2 + Mean.Sp.BodyMass2
Mean.Raup.Crick2 ~ Sp.Richness2 + Total.Animals2 + Mean.Sp.BodyMass2
Brillouin.Index2 ~ Sp.Richness2 + Total.Animals2

insitu2 ~ Attendance2 + Zoo.Area.ha2 + Sp.Richness2 + Total.Animals2
+ Mam.Sp.Richness2 + Prop.Mam.Sp2 + Prop.Threat.Sp2
+ Mean.Sp.BodyMass2 + Brillouin.Index2 + Mean.Raup.Crick2
+ X50km_Pop2 + X10km_Pop2'
```

We then fit the model, generate the model summary and generate a selection of absolute fit indices (e.g. Standardized Root Mean Square Residual) and incremental fit indices (e.g. Comparative Fit Index), to account for the differential sensitivity of fit indices to data distribution, model size and sample size (Hu & Bentler, 1999). We then generate modification indices to identify suspected pathways for inclusion, with a standard cut-off level for the chi-square test criterion of 3.84 (Burnham and Anderson, 2002). As the in situ dataset contains only AZA institutions, there was no need to nest the data or include GDP in the model (institutions within countries).

```
# Fit model and generate model summary
mod.IS1.fit <- sem(mod.IS1, data = sem_in_situ_data, fixed.x=FALSE)
summary(mod.IS1.fit, rsq = TRUE)
```

```
## lavaan (0.5-23.1097) converged normally after  56 iterations
##
##   Number of observations                           119
##
##   Estimator                                         ML
##   Minimum Function Test Statistic              153.717
##   Degrees of freedom                                25
##   P-value (Chi-square)                           0.000
##
## Parameter Estimates:
##
##   Information                                 Expected
##   Standard Errors                             Standard
##
## Regressions:
##                     Estimate  Std.Err  z-value  P(>|z|)
##   Attendance2 ~
##     Zoo.Area.ha2        0.016    0.070    0.223    0.824
##     Sp.Richness2       -0.371    0.177   -2.095    0.036
##     Total.Animals2      0.677    0.135    5.001    0.000
##     Mam.Sp.Rchnss2      0.013    0.099    0.129    0.898
##     Prop.Mam.Sp2        0.050    0.062    0.801    0.423
##     Prop.Thret.Sp2     -0.045    0.053   -0.854    0.393
##     Men.Sp.BdyMss2      0.289    0.077    3.742    0.000
##     Brilloun.Indx2      0.276    0.088    3.149    0.002
##     Mean.Rap.Crck2      0.136    0.059    2.300    0.021
##     X50km_Pop2          0.167    0.075    2.235    0.025
```

```
##     X10km_Pop2          0.302    0.089    3.391    0.001
##   Total.Animals2 ~
##     Zoo.Area.ha2        0.142    0.049    2.903    0.004
##     Sp.Richness2        0.809    0.049   16.493    0.000
##   Sp.Richness2 ~
##     Mam.Sp.Rchnss2      0.877    0.055   15.817    0.000
##     Men.Sp.BdyMss2     -0.168    0.055   -3.028    0.002
##   Mean.Raup.Crick2 ~
##     Sp.Richness2       -0.035    0.140   -0.252    0.801
##     Total.Animals2      0.458    0.141    3.252    0.001
##     Men.Sp.BdyMss2     -0.537    0.073   -7.343    0.000
##   Brillouin.Index2 ~
##     Sp.Richness2        1.573    0.094   16.667    0.000
##     Total.Animals2     -1.081    0.094  -11.496    0.000
##   insitu2 ~
##     Attendance2         0.604    0.125    4.826    0.000
##     Zoo.Area.ha2        0.217    0.095    2.281    0.023
##     Sp.Richness2        0.248    0.246    1.006    0.315
##     Total.Animals2     -0.418    0.203   -2.058    0.040
##     Mam.Sp.Rchnss2      0.171    0.135    1.266    0.205
##     Prop.Mam.Sp2       -0.016    0.085   -0.191    0.849
##     Prop.Thret.Sp2      0.203    0.072    2.808    0.005
##     Men.Sp.BdyMss2     -0.086    0.111   -0.769    0.442
##     Brilloun.Indx2     -0.160    0.124   -1.285    0.199
##     Mean.Rap.Crck2      0.148    0.082    1.800    0.072
##     X50km_Pop2         -0.021    0.104   -0.205    0.838
##     X10km_Pop2          0.037    0.127    0.291    0.771
##
## Covariances:
##                    Estimate  Std.Err  z-value  P(>|z|)
##   Zoo.Area.ha2 ~~
##     Mam.Sp.Rchnss2        0.536    0.103    5.185    0.000
##     Prop.Mam.Sp2          0.287    0.095    3.030    0.002
##     Prop.Thret.Sp2        0.189    0.093    2.045    0.041
##     Men.Sp.BdyMss2        0.560    0.104    5.363    0.000
##     X50km_Pop2           -0.014    0.091   -0.155    0.876
##     X10km_Pop2           -0.099    0.091   -1.087    0.277
##   Mam.Sp.Richness2 ~~
##     Prop.Mam.Sp2          0.094    0.091    1.024    0.306
##     Prop.Thret.Sp2        0.322    0.096    3.368    0.001
##     Men.Sp.BdyMss2        0.373    0.097    3.845    0.000
##     X50km_Pop2            0.363    0.097    3.752    0.000
##     X10km_Pop2            0.421    0.099    4.262    0.000
##   Prop.Mam.Sp2 ~~
##     Prop.Thret.Sp2        0.041    0.091    0.451    0.652
##     Men.Sp.BdyMss2        0.545    0.104    5.256    0.000
##     X50km_Pop2           -0.228    0.093   -2.446    0.014
##     X10km_Pop2           -0.356    0.097   -3.687    0.000
##   Prop.Threat.Sp2 ~~
##     Men.Sp.BdyMss2        0.277    0.094    2.933    0.003
##     X50km_Pop2            0.044    0.091    0.489    0.625
##     X10km_Pop2            0.287    0.095    3.030    0.002
##   Mean.Sp.BodyMass2 ~~
##     X50km_Pop2           -0.094    0.091   -1.027    0.305
```

```
##     X10km_Pop2           -0.023    0.091   -0.248    0.804
##   X50km_Pop2 ~~
##     X10km_Pop2            0.738    0.113    6.511    0.000
##
## Variances:
##                    Estimate  Std.Err  z-value  P(>|z|)
##    .Attendance2       0.250    0.032    7.714    0.000
##    .Total.Animals2    0.243    0.032    7.714    0.000
##    .Sp.Richness2      0.311    0.040    7.714    0.000
##    .Mean.Rap.Crck2    0.602    0.078    7.714    0.000
##    .Brilloun.Indx2    0.274    0.036    7.714    0.000
##    .insitu2           0.466    0.060    7.714    0.000
##     Zoo.Area.ha2      0.992    0.129    7.714    0.000
##     Mam.Sp.Rchnss2    0.992    0.129    7.714    0.000
##     Prop.Mam.Sp2      0.992    0.129    7.714    0.000
##     Prop.Thret.Sp2    0.992    0.129    7.714    0.000
##     Men.Sp.BdyMss2    0.992    0.129    7.714    0.000
##     X50km_Pop2        0.992    0.129    7.714    0.000
##     X10km_Pop2        0.992    0.129    7.714    0.000
##
## R-Square:
##                    Estimate
##     Attendance2       0.733
##     Total.Animals2    0.757
##     Sp.Richness2      0.686
##     Mean.Rap.Crck2    0.382
##     Brilloun.Indx2    0.722
##     insitu2           0.532
```

```r
# Generate fit indices
fitMeasures(mod.IS1.fit, c("agfi", "rmr", "srmr", "rmsea", "cfi", "nnfi", "tli"))
```

```
##  agfi   rmr  srmr rmsea   cfi  nnfi   tli
## 0.534 0.084 0.085 0.208 0.852 0.662 0.662
```

```r
# Generate modification indices
miIS1 <- modindices(mod.IS1.fit)
print(miIS1[miIS1$mi > 3.0,])
```

```
##                  lhs op               rhs     mi    epc sepc.lv sepc.all
## 73     Total.Animals2 ~~ Mean.Raup.Crick2  3.924 -0.320  -0.320   -0.325
## 91     Total.Animals2  ~       X50km_Pop2  8.553  0.143   0.143    0.142
## 93       Sp.Richness2  ~      Attendance2  5.163  0.277   0.277    0.269
## 97       Sp.Richness2  ~          insitu2  6.452  0.378   0.378    0.379
## 99       Sp.Richness2  ~     Prop.Mam.Sp2 54.821 -0.460  -0.460   -0.460
## 101      Sp.Richness2  ~       X50km_Pop2  3.520  0.108   0.108    0.108
## 102      Sp.Richness2  ~       X10km_Pop2 17.968  0.246   0.246    0.246
## 106  Mean.Raup.Crick2  ~     Zoo.Area.ha2  3.923  0.188   0.188    0.189
## 108  Mean.Raup.Crick2  ~     Prop.Mam.Sp2 13.142 -0.312  -0.312   -0.315
## 109  Mean.Raup.Crick2  ~  Prop.Threat.Sp2 13.952 -0.284  -0.284   -0.286
## 134  Mam.Sp.Richness2  ~      Attendance2  3.962 -0.455  -0.455   -0.442
## 136  Mam.Sp.Richness2  ~      Sp.Richness2  3.009 -0.282  -0.282   -0.282
## 146      Prop.Mam.Sp2  ~      Attendance2 29.459 -1.005  -1.005   -0.978
## 147      Prop.Mam.Sp2  ~   Total.Animals2 19.669 -0.448  -0.448   -0.450
## 148      Prop.Mam.Sp2  ~     Sp.Richness2 35.872 -0.725  -0.725   -0.725
## 149      Prop.Mam.Sp2  ~ Mean.Raup.Crick2 29.999 -0.446  -0.446   -0.442
```

```
## 150       Prop.Mam.Sp2  ~ Brillouin.Index2  6.525 -0.205  -0.205   -0.204
## 151       Prop.Mam.Sp2  ~          insitu2 33.521 -2.036  -2.036   -2.040
## 161    Prop.Threat.Sp2  ~ Mean.Raup.Crick2  8.269 -0.276  -0.276   -0.274
## 163    Prop.Threat.Sp2  ~          insitu2  8.686 -1.222  -1.222   -1.224
## 170 Mean.Sp.BodyMass2  ~      Attendance2  6.831  0.506   0.506    0.492
## 171 Mean.Sp.BodyMass2  ~    Total.Animals2  6.134  0.259   0.259    0.260
## 172 Mean.Sp.BodyMass2  ~     Sp.Richness2 25.921  0.800   0.800    0.800
## 173 Mean.Sp.BodyMass2  ~ Mean.Raup.Crick2 15.539  0.396   0.396    0.392
## 175 Mean.Sp.BodyMass2  ~          insitu2 18.023  1.861   1.861    1.864
## 185        X50km_Pop2  ~ Mean.Raup.Crick2  3.862  0.133   0.133    0.132
## 186        X50km_Pop2  ~ Brillouin.Index2  5.214 -0.153  -0.153   -0.152
## 187        X50km_Pop2  ~          insitu2  3.202  0.525   0.525    0.526
##     sepc.nox
## 73    -0.325
## 91     0.143
## 93     0.269
## 97     0.379
## 99    -0.462
## 101    0.108
## 102    0.247
## 106    0.190
## 108   -0.316
## 109   -0.288
## 134   -0.442
## 136   -0.282
## 146   -0.978
## 147   -0.450
## 148   -0.725
## 149   -0.442
## 150   -0.204
## 151   -2.040
## 161   -0.274
## 163   -1.224
## 170    0.492
## 171    0.260
## 172    0.800
## 173    0.392
## 175    1.864
## 185    0.132
## 186   -0.152
## 187    0.526
```

Model 2

None of the modification indices from the first model are conceptually appealling, so now we use p-values to identify potentially unsupported pathways, with a threshold of 0.05. Highest p-values are considered first for removal. Based on the results generated from the first model, the highest p-value relationship to be considered for removal is **insitu2 ~ Prop.Mam.Sp2** with a p-value of **0.849**. As **Attendance2 ~ Prop.Mam.Sp2** was unsupported in the previous models and has a p-value of **0.423** here, we also remove **Attendance2 ~ Prop.Mam.Sp2** here. Therefore we decide to remove these pathways. The model summary, fit indices and modification indices were all generated for the model.

```
# In Situ SEM (Presence-Absence)

# Model 2
# Removal of insitu2 ~ Prop.Mam.Sp2, p = 0.849
# Removal of Attendance2 ~ Prop.Mam.Sp2, p = 0.423

mod.IS2 <- 'Attendance2 ~ Zoo.Area.ha2 + Sp.Richness2 + Total.Animals2
+ Mam.Sp.Richness2 + Prop.Threat.Sp2
+ Mean.Sp.BodyMass2 + Brillouin.Index2 + Mean.Raup.Crick2
+ X50km_Pop2 + X10km_Pop2

Total.Animals2 ~ Zoo.Area.ha2 + Sp.Richness2
Sp.Richness2 ~ Mam.Sp.Richness2 + Mean.Sp.BodyMass2
Mean.Raup.Crick2 ~ Sp.Richness2 + Total.Animals2 + Mean.Sp.BodyMass2
Brillouin.Index2 ~ Sp.Richness2 + Total.Animals2

insitu2 ~ Attendance2 + Zoo.Area.ha2 + Sp.Richness2 + Total.Animals2
+ Mam.Sp.Richness2 + Prop.Threat.Sp2
+ Mean.Sp.BodyMass2 + Brillouin.Index2 + Mean.Raup.Crick2
+ X50km_Pop2 + X10km_Pop2'

# Fit model and generate model summary
mod.IS2.fit <- sem(mod.IS2, data = sem_in_situ_data, fixed.x=FALSE)
summary(mod.IS2.fit, rsq = TRUE)
```

```
## lavaan (0.5-23.1097) converged normally after  52 iterations
##
##   Number of observations                           119
##
##   Estimator                                         ML
##   Minimum Function Test Statistic               64.403
##   Degrees of freedom                                21
##   P-value (Chi-square)                           0.000
##
## Parameter Estimates:
##
##   Information                                 Expected
##   Standard Errors                             Standard
##
## Regressions:
##                    Estimate  Std.Err  z-value  P(>|z|)
##   Attendance2 ~
##     Zoo.Area.ha2      0.016    0.069    0.233    0.816
##     Sp.Richness2     -0.408    0.178   -2.296    0.022
##     Total.Animals2    0.677    0.136    4.993    0.000
##     Mam.Sp.Rchnss2    0.048    0.099    0.483    0.629
##     Prop.Thret.Sp2   -0.051    0.053   -0.973    0.331
##     Men.Sp.BdyMss2    0.302    0.068    4.463    0.000
##     Brilloun.Indx2    0.276    0.088    3.147    0.002
##     Mean.Rap.Crck2    0.116    0.059    1.959    0.050
##     X50km_Pop2        0.175    0.074    2.373    0.018
##     X10km_Pop2        0.289    0.081    3.549    0.000
##   Total.Animals2 ~
##     Zoo.Area.ha2      0.142    0.049    2.903    0.004
```

```
##     Sp.Richness2        0.809    0.049   16.493    0.000
##   Sp.Richness2 ~
##     Mam.Sp.Rchnss2      0.877    0.055   15.817    0.000
##     Men.Sp.BdyMss2     -0.168    0.055   -3.028    0.002
##   Mean.Raup.Crick2 ~
##     Sp.Richness2       -0.035    0.140   -0.252    0.801
##     Total.Animals2      0.458    0.141    3.252    0.001
##     Men.Sp.BdyMss2     -0.537    0.073   -7.343    0.000
##   Brillouin.Index2 ~
##     Sp.Richness2        1.573    0.094   16.667    0.000
##     Total.Animals2     -1.081    0.094  -11.496    0.000
##   insitu2 ~
##     Attendance2         0.603    0.125    4.825    0.000
##     Zoo.Area.ha2        0.217    0.094    2.309    0.021
##     Sp.Richness2        0.259    0.247    1.048    0.294
##     Total.Animals2     -0.418    0.203   -2.055    0.040
##     Mam.Sp.Rchnss2      0.160    0.135    1.184    0.236
##     Prop.Thret.Sp2      0.205    0.072    2.835    0.005
##     Men.Sp.BdyMss2     -0.090    0.100   -0.900    0.368
##     Brilloun.Indx2     -0.160    0.124   -1.283    0.199
##     Mean.Rap.Crck2      0.155    0.082    1.891    0.059
##     X50km_Pop2         -0.024    0.103   -0.231    0.818
##     X10km_Pop2          0.041    0.117    0.354    0.724
##
## Covariances:
##                        Estimate  Std.Err  z-value  P(>|z|)
##   Zoo.Area.ha2 ~~
##     Mam.Sp.Rchnss2        0.536    0.103    5.185    0.000
##     Prop.Thret.Sp2        0.189    0.093    2.045    0.041
##     Men.Sp.BdyMss2        0.560    0.104    5.363    0.000
##     X50km_Pop2           -0.014    0.091   -0.155    0.876
##     X10km_Pop2           -0.099    0.091   -1.087    0.277
##   Mam.Sp.Richness2 ~~
##     Prop.Thret.Sp2        0.322    0.096    3.368    0.001
##     Men.Sp.BdyMss2        0.373    0.097    3.845    0.000
##     X50km_Pop2            0.363    0.097    3.752    0.000
##     X10km_Pop2            0.421    0.099    4.262    0.000
##   Prop.Threat.Sp2 ~~
##     Men.Sp.BdyMss2        0.277    0.094    2.933    0.003
##     X50km_Pop2            0.044    0.091    0.489    0.625
##     X10km_Pop2            0.287    0.095    3.030    0.002
##   Mean.Sp.BodyMass2 ~~
##     X50km_Pop2           -0.094    0.091   -1.027    0.305
##     X10km_Pop2           -0.023    0.091   -0.248    0.804
##   X50km_Pop2 ~~
##     X10km_Pop2            0.738    0.113    6.511    0.000
##
## Variances:
##                    Estimate  Std.Err  z-value  P(>|z|)
##    .Attendance2       0.251    0.033    7.714    0.000
##    .Total.Animals2    0.243    0.032    7.714    0.000
##    .Sp.Richness2      0.311    0.040    7.714    0.000
##    .Mean.Rap.Crck2    0.602    0.078    7.714    0.000
##    .Brilloun.Indx2    0.274    0.036    7.714    0.000
```

```
##    .insitu2           0.466    0.060    7.714    0.000
##     Zoo.Area.ha2      0.992    0.129    7.714    0.000
##     Mam.Sp.Rchnss2    0.992    0.129    7.714    0.000
##     Prop.Thret.Sp2    0.992    0.129    7.714    0.000
##     Men.Sp.BdyMss2    0.992    0.129    7.714    0.000
##     X50km_Pop2        0.992    0.129    7.714    0.000
##     X10km_Pop2        0.992    0.129    7.714    0.000
##
## R-Square:
##                    Estimate
##     Attendance2       0.730
##     Total.Animals2    0.757
##     Sp.Richness2      0.686
##     Mean.Rap.Crck2    0.382
##     Brilloun.Indx2    0.722
##     insitu2           0.531
```

```
# Generate fit indices
fitMeasures(mod.IS2.fit, c("agfi", "rmr", "srmr", "rmsea", "cfi", "nnfi", "tli"))
```

```
##  agfi   rmr  srmr rmsea   cfi  nnfi   tli
## 0.716 0.059 0.060 0.132 0.945 0.866 0.866
```

```
# Generate modification indices
miIS2 <- modindices(mod.IS2.fit)
print(miIS2[miIS2$mi > 3.0,])
```

```
##                  lhs op              rhs     mi    epc sepc.lv sepc.all
## 64    Total.Animals2 ~~ Mean.Raup.Crick2  3.923 -0.320  -0.320   -0.325
## 81    Total.Animals2  ~       X50km_Pop2  8.553  0.143   0.143    0.142
## 83      Sp.Richness2  ~      Attendance2  8.258  0.348   0.348    0.337
## 87      Sp.Richness2  ~          insitu2  7.581  0.410   0.410    0.411
## 90      Sp.Richness2  ~       X50km_Pop2  3.520  0.108   0.108    0.108
## 91      Sp.Richness2  ~       X10km_Pop2 17.968  0.246   0.246    0.246
## 95  Mean.Raup.Crick2  ~     Zoo.Area.ha2  3.923  0.188   0.188    0.189
## 97  Mean.Raup.Crick2  ~   Prop.Threat.Sp2 13.952 -0.284  -0.284   -0.286
## 109     Zoo.Area.ha2  ~      Attendance2  6.245  0.513   0.513    0.497
## 112     Zoo.Area.ha2  ~ Mean.Raup.Crick2  5.938  0.190   0.190    0.188
## 114     Zoo.Area.ha2  ~          insitu2  6.176  0.858   0.858    0.859
## 120 Mam.Sp.Richness2  ~      Attendance2  7.702 -0.679  -0.679   -0.657
## 121 Mam.Sp.Richness2  ~   Total.Animals2  5.031 -0.288  -0.288   -0.289
## 122 Mam.Sp.Richness2  ~     Sp.Richness2  7.706 -0.458  -0.458   -0.458
## 123 Mam.Sp.Richness2  ~ Mean.Raup.Crick2  4.113 -0.194  -0.194   -0.192
## 125 Mam.Sp.Richness2  ~          insitu2  4.428 -0.897  -0.897   -0.898
## 134  Prop.Threat.Sp2  ~ Mean.Raup.Crick2  9.199 -0.291  -0.291   -0.289
## 136  Prop.Threat.Sp2  ~          insitu2  9.670 -1.332  -1.332   -1.334
## 157       X50km_Pop2  ~ Brillouin.Index2  7.650 -0.188  -0.188   -0.187
## 164       X10km_Pop2  ~      Attendance2  3.703  0.290   0.290    0.281
## 165       X10km_Pop2  ~   Total.Animals2  3.809  0.151   0.151    0.151
## 166       X10km_Pop2  ~     Sp.Richness2 17.881  0.392   0.392    0.392
## 168       X10km_Pop2  ~ Brillouin.Index2  7.770  0.171   0.171    0.170
##     sepc.nox
## 64    -0.325
## 81     0.143
## 83     0.337
## 87     0.411
```

```
## 90     0.108
## 91     0.247
## 95     0.190
## 97    -0.288
## 109    0.497
## 112    0.188
## 114    0.859
## 120   -0.657
## 121   -0.289
## 122   -0.458
## 123   -0.192
## 125   -0.898
## 134   -0.289
## 136   -1.334
## 157   -0.187
## 164    0.281
## 165    0.151
## 166    0.392
## 168    0.170
```

Model 3

Based on the results generated from the second model, the highest p-value relationship to be considered for removal is **insitu2 ~ X50km_Pop2** with a p-value of **0.818**. As **Attendance2 ~ X50km_Pop22** was unsupported in the previous models, we also remove **Attendance2 ~ X50km_Pop22** here. Therefore we decide to remove these pathways. The model summary, fit indices and modification indices were all generated for the model.

```
# In Situ SEM (Presence-Absence)

# Model 3
# Removal of insitu2 ~ X50km_Pop2, p = 0.818
# Removal of Attendance2 ~ X50km_Pop22

mod.IS3 <- 'Attendance2 ~ Zoo.Area.ha2 + Sp.Richness2 + Total.Animals2
+ Mam.Sp.Richness2 + Prop.Threat.Sp2
+ Mean.Sp.BodyMass2 + Brillouin.Index2 + Mean.Raup.Crick2
+ X10km_Pop2

Total.Animals2 ~ Zoo.Area.ha2 + Sp.Richness2
Sp.Richness2 ~ Mam.Sp.Richness2 + Mean.Sp.BodyMass2
Mean.Raup.Crick2 ~ Sp.Richness2 + Total.Animals2 + Mean.Sp.BodyMass2
Brillouin.Index2 ~ Sp.Richness2 + Total.Animals2

insitu2 ~ Attendance2 + Zoo.Area.ha2 + Sp.Richness2 + Total.Animals2
+ Mam.Sp.Richness2 + Prop.Threat.Sp2
+ Mean.Sp.BodyMass2 + Brillouin.Index2 + Mean.Raup.Crick2
+ X10km_Pop2'

# Fit model and generate model summary
mod.IS3.fit <- sem(mod.IS3, data = sem_in_situ_data, fixed.x=FALSE)
summary(mod.IS3.fit, rsq = TRUE)
```

```
## lavaan (0.5-23.1097) converged normally after  44 iterations
```

```
##
##   Number of observations                           119
##
##   Estimator                                         ML
##   Minimum Function Test Statistic               52.494
##   Degrees of freedom                                17
##   P-value (Chi-square)                           0.000
##
## Parameter Estimates:
##
##   Information                                 Expected
##   Standard Errors                             Standard
##
## Regressions:
##                      Estimate  Std.Err  z-value  P(>|z|)
##   Attendance2 ~
##     Zoo.Area.ha2        0.027    0.070    0.394    0.694
##     Sp.Richness2       -0.477    0.181   -2.631    0.009
##     Total.Animals2      0.721    0.138    5.209    0.000
##     Mam.Sp.Rchnss2      0.086    0.101    0.854    0.393
##     Prop.Thret.Sp2     -0.083    0.052   -1.606    0.108
##     Men.Sp.BdyMss2      0.287    0.068    4.193    0.000
##     Brilloun.Indx2      0.273    0.090    3.043    0.002
##     Mean.Rap.Crck2      0.130    0.060    2.147    0.032
##     X10km_Pop2          0.429    0.059    7.263    0.000
##   Total.Animals2 ~
##     Zoo.Area.ha2        0.142    0.049    2.903    0.004
##     Sp.Richness2        0.809    0.049   16.493    0.000
##   Sp.Richness2 ~
##     Mam.Sp.Rchnss2      0.877    0.055   15.817    0.000
##     Men.Sp.BdyMss2     -0.168    0.055   -3.028    0.002
##   Mean.Raup.Crick2 ~
##     Sp.Richness2       -0.035    0.140   -0.252    0.801
##     Total.Animals2      0.458    0.141    3.252    0.001
##     Men.Sp.BdyMss2     -0.537    0.073   -7.343    0.000
##   Brillouin.Index2 ~
##     Sp.Richness2        1.573    0.094   16.667    0.000
##     Total.Animals2     -1.081    0.094  -11.496    0.000
##   insitu2 ~
##     Attendance2         0.597    0.122    4.881    0.000
##     Zoo.Area.ha2        0.216    0.093    2.315    0.021
##     Sp.Richness2        0.266    0.249    1.068    0.285
##     Total.Animals2     -0.420    0.205   -2.049    0.040
##     Mam.Sp.Rchnss2      0.155    0.135    1.149    0.251
##     Prop.Thret.Sp2      0.209    0.070    2.980    0.003
##     Men.Sp.BdyMss2     -0.086    0.098   -0.879    0.379
##     Brilloun.Indx2     -0.158    0.124   -1.270    0.204
##     Mean.Rap.Crck2      0.154    0.082    1.871    0.061
##     X10km_Pop2          0.025    0.095    0.261    0.794
##
## Covariances:
##                        Estimate  Std.Err  z-value  P(>|z|)
##   Zoo.Area.ha2 ~~
##     Mam.Sp.Rchnss2        0.536    0.103    5.185    0.000
```

```
##     Prop.Thret.Sp2        0.189    0.093    2.045    0.041
##     Men.Sp.BdyMss2        0.560    0.104    5.363    0.000
##     X10km_Pop2           -0.099    0.091   -1.087    0.277
##   Mam.Sp.Richness2 ~~
##     Prop.Thret.Sp2        0.322    0.096    3.368    0.001
##     Men.Sp.BdyMss2        0.373    0.097    3.845    0.000
##     X10km_Pop2            0.421    0.099    4.262    0.000
##   Prop.Threat.Sp2 ~~
##     Men.Sp.BdyMss2        0.277    0.094    2.933    0.003
##     X10km_Pop2            0.287    0.095    3.030    0.002
##   Mean.Sp.BodyMass2 ~~
##     X10km_Pop2           -0.023    0.091   -0.248    0.804
##
## Variances:
##                    Estimate  Std.Err  z-value  P(>|z|)
##    .Attendance2       0.262    0.034    7.714    0.000
##    .Total.Animals2    0.243    0.032    7.714    0.000
##    .Sp.Richness2      0.311    0.040    7.714    0.000
##    .Mean.Rap.Crck2    0.602    0.078    7.714    0.000
##    .Brilloun.Indx2    0.274    0.036    7.714    0.000
##    .insitu2           0.466    0.060    7.714    0.000
##     Zoo.Area.ha2      0.992    0.129    7.714    0.000
##     Mam.Sp.Rchnss2    0.992    0.129    7.714    0.000
##     Prop.Thret.Sp2    0.992    0.129    7.714    0.000
##     Men.Sp.BdyMss2    0.992    0.129    7.714    0.000
##     X10km_Pop2        0.992    0.129    7.714    0.000
##
## R-Square:
##                    Estimate
##     Attendance2       0.720
##     Total.Animals2    0.757
##     Sp.Richness2      0.686
##     Mean.Rap.Crck2    0.382
##     Brilloun.Indx2    0.722
##     insitu2           0.532
```

```
# Generate fit indices
fitMeasures(mod.IS3.fit, c("agfi", "rmr", "srmr", "rmsea", "cfi", "nnfi", "tli"))
```

```
##  agfi   rmr  srmr rmsea   cfi  nnfi   tli
## 0.736 0.054 0.054 0.132 0.954 0.879 0.879
```

```
# Generate modification indices
miIS3 <- modindices(mod.IS3.fit)
print(miIS3[miIS3$mi > 3.0,])
```

```
##                   lhs op               rhs     mi    epc sepc.lv sepc.all
## 56    Total.Animals2 ~~ Mean.Raup.Crick2  3.923 -0.320  -0.320   -0.325
## 74      Sp.Richness2  ~      Attendance2 11.103  0.404   0.404    0.393
## 78      Sp.Richness2  ~          insitu2  9.070  0.448   0.448    0.450
## 81      Sp.Richness2  ~       X10km_Pop2 17.968  0.246   0.246    0.246
## 85  Mean.Raup.Crick2  ~     Zoo.Area.ha2  3.923  0.188   0.188    0.189
## 87  Mean.Raup.Crick2  ~  Prop.Threat.Sp2 13.952 -0.284  -0.284   -0.286
## 97      Zoo.Area.ha2  ~      Attendance2  7.517  0.571   0.571    0.555
## 100     Zoo.Area.ha2  ~ Mean.Raup.Crick2  6.852  0.206   0.206    0.204
## 102     Zoo.Area.ha2  ~          insitu2  7.004  0.903   0.903    0.905
```

```
## 107 Mam.Sp.Richness2  ~      Attendance2  6.463 -0.604  -0.604   -0.587
## 108 Mam.Sp.Richness2  ~   Total.Animals2  4.646 -0.279  -0.279   -0.280
## 109 Mam.Sp.Richness2  ~     Sp.Richness2  9.050 -0.500  -0.500   -0.500
## 110 Mam.Sp.Richness2  ~ Mean.Raup.Crick2  3.726 -0.185  -0.185   -0.184
## 112 Mam.Sp.Richness2  ~          insitu2  3.608 -0.787  -0.787   -0.789
## 120  Prop.Threat.Sp2  ~ Mean.Raup.Crick2 11.857 -0.344  -0.344   -0.341
## 122  Prop.Threat.Sp2  ~          insitu2 11.645 -1.472  -1.472   -1.475
## 137       X10km_Pop2  ~      Attendance2 10.544  0.671   0.671    0.652
## 138       X10km_Pop2  ~   Total.Animals2 13.199  0.396   0.396    0.397
## 139       X10km_Pop2  ~     Sp.Richness2 18.045  0.554   0.554    0.554
## 140       X10km_Pop2  ~ Mean.Raup.Crick2  5.397  0.204   0.204    0.202
## 142       X10km_Pop2  ~          insitu2  5.091  0.856   0.856    0.858
##     sepc.nox
## 56    -0.325
## 74     0.393
## 78     0.450
## 81     0.247
## 85     0.190
## 87    -0.288
## 97     0.555
## 100    0.204
## 102    0.905
## 107   -0.587
## 108   -0.280
## 109   -0.500
## 110   -0.184
## 112   -0.789
## 120   -0.341
## 122   -1.475
## 137    0.652
## 138    0.397
## 139    0.554
## 140    0.202
## 142    0.858
```

Model 4

Based on the results generated from the third model, the highest p-value relationship to be considered for removal is **Mean.Raup.Crick2** ~ **Sp.Richness2** with a p-value of **0.801**. Therefore we decide to remove this pathway. Once again, the model summary, fit indices and modification indices were all generated for the model.

```
# In Situ SEM (Presence-Absence)

# Model 4
# Removal of Mean.Raup.Crick2 ~ Sp.Richness2, p = 0.801

mod.IS4 <- 'Attendance2 ~ Zoo.Area.ha2 + Sp.Richness2 + Total.Animals2
+ Mam.Sp.Richness2 + Prop.Threat.Sp2
+ Mean.Sp.BodyMass2 + Brillouin.Index2 + Mean.Raup.Crick2
+ X10km_Pop2

Total.Animals2 ~ Zoo.Area.ha2 + Sp.Richness2
```

```
Sp.Richness2 ~ Mam.Sp.Richness2 + Mean.Sp.BodyMass2
Mean.Raup.Crick2 ~ Total.Animals2 + Mean.Sp.BodyMass2
Brillouin.Index2 ~ Sp.Richness2 + Total.Animals2

insitu2 ~ Attendance2 + Zoo.Area.ha2 + Sp.Richness2 + Total.Animals2
+ Mam.Sp.Richness2 + Prop.Threat.Sp2
+ Mean.Sp.BodyMass2 + Brillouin.Index2 + Mean.Raup.Crick2
+ X10km_Pop2'

# Fit model and generate model summary
mod.IS4.fit <- sem(mod.IS4, data = sem_in_situ_data, fixed.x=FALSE)
summary(mod.IS4.fit, rsq = TRUE)
```

```
## lavaan (0.5-23.1097) converged normally after  42 iterations
##
##   Number of observations                           119
##
##   Estimator                                         ML
##   Minimum Function Test Statistic               52.558
##   Degrees of freedom                                18
##   P-value (Chi-square)                           0.000
##
## Parameter Estimates:
##
##   Information                                 Expected
##   Standard Errors                             Standard
##
## Regressions:
##                    Estimate  Std.Err  z-value  P(>|z|)
##   Attendance2 ~
##     Zoo.Area.ha2      0.027    0.070    0.394    0.694
##     Sp.Richness2     -0.477    0.181   -2.632    0.009
##     Total.Animals2    0.721    0.138    5.223    0.000
##     Mam.Sp.Rchnss2    0.086    0.101    0.854    0.393
##     Prop.Thret.Sp2   -0.083    0.052   -1.606    0.108
##     Men.Sp.BdyMss2    0.287    0.068    4.192    0.000
##     Brilloun.Indx2    0.273    0.090    3.043    0.002
##     Mean.Rap.Crck2    0.130    0.060    2.148    0.032
##     X10km_Pop2        0.429    0.059    7.263    0.000
##   Total.Animals2 ~
##     Zoo.Area.ha2      0.142    0.049    2.903    0.004
##     Sp.Richness2      0.809    0.049   16.493    0.000
##   Sp.Richness2 ~
##     Mam.Sp.Rchnss2    0.877    0.055   15.817    0.000
##     Men.Sp.BdyMss2   -0.168    0.055   -3.028    0.002
##   Mean.Raup.Crick2 ~
##     Total.Animals2    0.427    0.073    5.874    0.000
##     Men.Sp.BdyMss2   -0.537    0.073   -7.354    0.000
##   Brillouin.Index2 ~
##     Sp.Richness2      1.573    0.094   16.667    0.000
##     Total.Animals2   -1.080    0.094  -11.496    0.000
##   insitu2 ~
##     Attendance2       0.597    0.122    4.881    0.000
##     Zoo.Area.ha2      0.216    0.093    2.315    0.021
```

```
##     Sp.Richness2        0.266    0.249    1.069    0.285
##     Total.Animals2     -0.420    0.204   -2.053    0.040
##     Mam.Sp.Rchnss2      0.155    0.135    1.149    0.251
##     Prop.Thret.Sp2      0.209    0.070    2.980    0.003
##     Men.Sp.BdyMss2     -0.086    0.098   -0.879    0.379
##     Brilloun.Indx2     -0.158    0.124   -1.270    0.204
##     Mean.Rap.Crck2      0.154    0.082    1.871    0.061
##     X10km_Pop2          0.025    0.095    0.261    0.794
##
## Covariances:
##                        Estimate  Std.Err  z-value  P(>|z|)
##   Zoo.Area.ha2 ~~
##     Mam.Sp.Rchnss2        0.536    0.103    5.185    0.000
##     Prop.Thret.Sp2        0.189    0.093    2.045    0.041
##     Men.Sp.BdyMss2        0.560    0.104    5.363    0.000
##     X10km_Pop2           -0.099    0.091   -1.087    0.277
##   Mam.Sp.Richness2 ~~
##     Prop.Thret.Sp2        0.322    0.096    3.368    0.001
##     Men.Sp.BdyMss2        0.373    0.097    3.845    0.000
##     X10km_Pop2            0.421    0.099    4.262    0.000
##   Prop.Threat.Sp2 ~~
##     Men.Sp.BdyMss2        0.277    0.094    2.933    0.003
##     X10km_Pop2            0.287    0.095    3.030    0.002
##   Mean.Sp.BodyMass2 ~~
##     X10km_Pop2           -0.023    0.091   -0.248    0.804
##
## Variances:
##                    Estimate  Std.Err  z-value  P(>|z|)
##    .Attendance2       0.262    0.034    7.714    0.000
##    .Total.Animals2    0.243    0.032    7.714    0.000
##    .Sp.Richness2      0.311    0.040    7.714    0.000
##    .Mean.Rap.Crck2    0.602    0.078    7.714    0.000
##    .Brilloun.Indx2    0.274    0.036    7.714    0.000
##    .insitu2           0.466    0.060    7.714    0.000
##     Zoo.Area.ha2      0.992    0.129    7.714    0.000
##     Mam.Sp.Rchnss2    0.992    0.129    7.714    0.000
##     Prop.Thret.Sp2    0.992    0.129    7.714    0.000
##     Men.Sp.BdyMss2    0.992    0.129    7.714    0.000
##     X10km_Pop2        0.992    0.129    7.714    0.000
##
## R-Square:
##                    Estimate
##     Attendance2       0.721
##     Total.Animals2    0.757
##     Sp.Richness2      0.686
##     Mean.Rap.Crck2    0.382
##     Brilloun.Indx2    0.722
##     insitu2           0.532
```

```r
# Generate fit indices
fitMeasures(mod.IS4.fit, c("agfi", "rmr", "srmr", "rmsea", "cfi", "nnfi", "tli"))
```

```
##  agfi   rmr  srmr rmsea   cfi  nnfi   tli
## 0.751 0.054 0.054 0.127 0.955 0.888 0.888
```

```r
# Generate modification indices
miIS4 <- modindices(mod.IS4.fit)
print(miIS4[miIS4$mi > 3.0,])
```

```
##                   lhs op               rhs     mi    epc sepc.lv sepc.all
## 73       Sp.Richness2  ~       Attendance2 10.931  0.400   0.400    0.389
## 77       Sp.Richness2  ~           insitu2  8.568  0.430   0.430    0.431
## 80       Sp.Richness2  ~        X10km_Pop2 17.968  0.246   0.246    0.246
## 85   Mean.Raup.Crick2  ~       Zoo.Area.ha2  3.986  0.189   0.189    0.191
## 87   Mean.Raup.Crick2  ~  Prop.Threat.Sp2 13.894 -0.281  -0.281   -0.284
## 97       Zoo.Area.ha2  ~       Attendance2  7.579  0.573   0.573    0.557
## 100      Zoo.Area.ha2  ~ Mean.Raup.Crick2  6.889  0.204   0.204    0.202
## 102      Zoo.Area.ha2  ~           insitu2  7.043  0.894   0.894    0.897
## 107 Mam.Sp.Richness2  ~       Attendance2  6.557 -0.604  -0.604   -0.587
## 108 Mam.Sp.Richness2  ~    Total.Animals2  4.646 -0.279  -0.279   -0.280
## 109 Mam.Sp.Richness2  ~      Sp.Richness2  9.049 -0.500  -0.500   -0.500
## 110 Mam.Sp.Richness2  ~ Mean.Raup.Crick2  3.537 -0.167  -0.167   -0.165
## 112 Mam.Sp.Richness2  ~           insitu2  3.434 -0.709  -0.709   -0.711
## 120  Prop.Threat.Sp2  ~ Mean.Raup.Crick2 11.924 -0.346  -0.346   -0.343
## 122  Prop.Threat.Sp2  ~           insitu2 11.720 -1.481  -1.481   -1.485
## 137        X10km_Pop2  ~       Attendance2 10.596  0.674   0.674    0.655
## 138        X10km_Pop2  ~    Total.Animals2 13.199  0.396   0.396    0.397
## 139        X10km_Pop2  ~      Sp.Richness2 18.045  0.554   0.554    0.554
## 140        X10km_Pop2  ~ Mean.Raup.Crick2  5.427  0.205   0.205    0.203
## 142        X10km_Pop2  ~           insitu2  5.124  0.861   0.861    0.864
##     sepc.nox
## 73     0.389
## 77     0.431
## 80     0.247
## 85     0.192
## 87    -0.285
## 97     0.557
## 100    0.202
## 102    0.897
## 107   -0.587
## 108   -0.280
## 109   -0.500
## 110   -0.165
## 112   -0.711
## 120   -0.343
## 122   -1.485
## 137    0.655
## 138    0.397
## 139    0.554
## 140    0.203
## 142    0.864
```

Model 5

Based on the results generated from the fourth model, the highest p-value relationship to be considered for removal is **insitu2 ~ X10km_Pop2** with a p-value of **0.794**. Therefore we decide to remove this pathway. Once again, the model summary, fit indices and modification indices were all generated for the model.

```r
# In Situ SEM (Presence-Absence)

# Model 5
# Removal of insitu2 ~ X10km_Pop2, p = 0.794

mod.IS5 <- 'Attendance2 ~ Zoo.Area.ha2 + Sp.Richness2 + Total.Animals2
+ Mam.Sp.Richness2 + Prop.Threat.Sp2
+ Mean.Sp.BodyMass2 + Brillouin.Index2 + Mean.Raup.Crick2
+ X10km_Pop2

Total.Animals2 ~ Zoo.Area.ha2 + Sp.Richness2
Sp.Richness2 ~ Mam.Sp.Richness2 + Mean.Sp.BodyMass2
Mean.Raup.Crick2 ~ Total.Animals2 + Mean.Sp.BodyMass2
Brillouin.Index2 ~ Sp.Richness2 + Total.Animals2

insitu2 ~ Attendance2 + Zoo.Area.ha2 + Sp.Richness2 + Total.Animals2
+ Mam.Sp.Richness2 + Prop.Threat.Sp2
+ Mean.Sp.BodyMass2 + Brillouin.Index2 + Mean.Raup.Crick2'

# Fit model and generate model summary
mod.IS5.fit <- sem(mod.IS5, data = sem_in_situ_data, fixed.x=FALSE)
summary(mod.IS5.fit, rsq = TRUE)
```

```
## lavaan (0.5-23.1097) converged normally after  43 iterations
##
##   Number of observations                           119
##
##   Estimator                                         ML
##   Minimum Function Test Statistic               52.618
##   Degrees of freedom                                19
##   P-value (Chi-square)                           0.000
##
## Parameter Estimates:
##
##   Information                                 Expected
##   Standard Errors                             Standard
##
## Regressions:
##                     Estimate  Std.Err  z-value  P(>|z|)
##   Attendance2 ~
##     Zoo.Area.ha2       0.027    0.070    0.394    0.694
##     Sp.Richness2      -0.477    0.181   -2.632    0.009
##     Total.Animals2     0.721    0.138    5.223    0.000
##     Mam.Sp.Rchnss2     0.086    0.101    0.854    0.393
##     Prop.Thret.Sp2    -0.083    0.052   -1.606    0.108
##     Men.Sp.BdyMss2     0.287    0.068    4.192    0.000
##     Brilloun.Indx2     0.273    0.090    3.043    0.002
##     Mean.Rap.Crck2     0.130    0.060    2.148    0.032
##     X10km_Pop2         0.429    0.059    7.263    0.000
##   Total.Animals2 ~
##     Zoo.Area.ha2       0.142    0.049    2.903    0.004
##     Sp.Richness2       0.809    0.049   16.493    0.000
##   Sp.Richness2 ~
##     Mam.Sp.Rchnss2     0.877    0.055   15.817    0.000
```

```
##     Men.Sp.BdyMss2     -0.168    0.055   -3.028    0.002
##   Mean.Raup.Crick2 ~
##     Total.Animals2      0.427    0.073    5.874    0.000
##     Men.Sp.BdyMss2     -0.537    0.073   -7.354    0.000
##   Brillouin.Index2 ~
##     Sp.Richness2        1.573    0.094   16.667    0.000
##     Total.Animals2     -1.081    0.094  -11.496    0.000
##   insitu2 ~
##     Attendance2         0.613    0.102    6.013    0.000
##     Zoo.Area.ha2        0.207    0.088    2.353    0.019
##     Sp.Richness2        0.282    0.247    1.141    0.254
##     Total.Animals2     -0.429    0.199   -2.159    0.031
##     Mam.Sp.Rchnss2      0.156    0.130    1.195    0.232
##     Prop.Thret.Sp2      0.214    0.068    3.154    0.002
##     Men.Sp.BdyMss2     -0.089    0.095   -0.937    0.349
##     Brilloun.Indx2     -0.163    0.123   -1.324    0.186
##     Mean.Rap.Crck2      0.153    0.082    1.876    0.061
##
## Covariances:
##                        Estimate  Std.Err  z-value  P(>|z|)
##   Zoo.Area.ha2 ~~
##     Mam.Sp.Rchnss2        0.536    0.103    5.185    0.000
##     Prop.Thret.Sp2        0.189    0.093    2.045    0.041
##     Men.Sp.BdyMss2        0.560    0.104    5.363    0.000
##     X10km_Pop2           -0.099    0.091   -1.087    0.277
##   Mam.Sp.Richness2 ~~
##     Prop.Thret.Sp2        0.322    0.096    3.368    0.001
##     Men.Sp.BdyMss2        0.373    0.097    3.845    0.000
##     X10km_Pop2            0.421    0.099    4.262    0.000
##   Prop.Threat.Sp2 ~~
##     Men.Sp.BdyMss2        0.277    0.094    2.933    0.003
##     X10km_Pop2            0.287    0.095    3.030    0.002
##   Mean.Sp.BodyMass2 ~~
##     X10km_Pop2           -0.023    0.091   -0.248    0.804
##
## Variances:
##                    Estimate  Std.Err  z-value  P(>|z|)
##    .Attendance2       0.262    0.034    7.714    0.000
##    .Total.Animals2    0.243    0.032    7.714    0.000
##    .Sp.Richness2      0.311    0.040    7.714    0.000
##    .Mean.Rap.Crck2    0.602    0.078    7.714    0.000
##    .Brilloun.Indx2    0.274    0.036    7.714    0.000
##    .insitu2           0.467    0.060    7.714    0.000
##     Zoo.Area.ha2      0.992    0.129    7.714    0.000
##     Mam.Sp.Rchnss2    0.992    0.129    7.714    0.000
##     Prop.Thret.Sp2    0.992    0.129    7.714    0.000
##     Men.Sp.BdyMss2    0.992    0.129    7.714    0.000
##     X10km_Pop2        0.992    0.129    7.714    0.000
##
## R-Square:
##                    Estimate
##     Attendance2       0.721
##     Total.Animals2    0.757
##     Sp.Richness2      0.686
```

```
##     Mean.Rap.Crck2    0.382
##     Brilloun.Indx2    0.722
##     insitu2           0.532
```

```r
# Generate fit indices
fitMeasures(mod.IS5.fit, c("agfi", "rmr", "srmr", "rmsea", "cfi", "nnfi", "tli"))
```

```
##  agfi   rmr  srmr rmsea   cfi  nnfi   tli
## 0.763 0.054 0.054 0.122 0.957 0.897 0.897
```

```r
# Generate modification indices
miIS5 <- modindices(mod.IS5.fit)
print(miIS5[miIS5$mi > 3.0,])
```

```
##                   lhs op             rhs     mi    epc sepc.lv sepc.all
## 72      Sp.Richness2  ~      Attendance2 10.931  0.400   0.400    0.389
## 76      Sp.Richness2  ~          insitu2  7.929  0.424   0.424    0.425
## 79      Sp.Richness2  ~      X10km_Pop2 17.968  0.246   0.246    0.246
## 84  Mean.Raup.Crick2  ~     Zoo.Area.ha2  3.986  0.189   0.189    0.191
## 86  Mean.Raup.Crick2  ~  Prop.Threat.Sp2 13.894 -0.281  -0.281   -0.284
## 97      Zoo.Area.ha2  ~      Attendance2  7.579  0.573   0.573    0.557
## 100     Zoo.Area.ha2  ~ Mean.Raup.Crick2  6.889  0.204   0.204    0.202
## 102     Zoo.Area.ha2  ~          insitu2  3.720  0.424   0.424    0.425
## 107 Mam.Sp.Richness2  ~      Attendance2  6.557 -0.604  -0.604   -0.587
## 108 Mam.Sp.Richness2  ~   Total.Animals2  4.646 -0.279  -0.279   -0.280
## 109 Mam.Sp.Richness2  ~     Sp.Richness2  9.049 -0.500  -0.500   -0.500
## 110 Mam.Sp.Richness2  ~ Mean.Raup.Crick2  3.537 -0.167  -0.167   -0.165
## 120  Prop.Threat.Sp2  ~ Mean.Raup.Crick2 11.924 -0.346  -0.346   -0.343
## 122  Prop.Threat.Sp2  ~          insitu2  9.071 -1.088  -1.088   -1.091
## 137       X10km_Pop2  ~      Attendance2 10.596  0.674   0.674    0.655
## 138       X10km_Pop2  ~   Total.Animals2 13.199  0.396   0.396    0.397
## 139       X10km_Pop2  ~     Sp.Richness2 18.045  0.554   0.554    0.554
## 140       X10km_Pop2  ~ Mean.Raup.Crick2  5.427  0.205   0.205    0.203
##     sepc.nox
## 72     0.389
## 76     0.425
## 79     0.247
## 84     0.192
## 86    -0.285
## 97     0.557
## 100    0.202
## 102    0.425
## 107   -0.587
## 108   -0.280
## 109   -0.500
## 110   -0.165
## 120   -0.343
## 122   -1.091
## 137    0.655
## 138    0.397
## 139    0.554
## 140    0.203
```

Model 6

Based on the results generated from the fifth model, the highest p-value relationship to be considered for removal is **Attendance2** ~ **Zoo.Area.ha2** with a p-value of **0.694**. Therefore we decide to remove this pathway. Once again, the model summary, fit indices and modification indices were all generated for the model.

```
# In Situ SEM (Presence-Absence)

# Model 6
# Removal of Attendance2 ~ Zoo.Area.ha2, p = 0.694

mod.IS6 <- 'Attendance2 ~ Sp.Richness2 + Total.Animals2
+ Mam.Sp.Richness2 + Prop.Threat.Sp2
+ Mean.Sp.BodyMass2 + Brillouin.Index2 + Mean.Raup.Crick2
+ X10km_Pop2

Total.Animals2 ~ Zoo.Area.ha2 + Sp.Richness2
Sp.Richness2 ~ Mam.Sp.Richness2 + Mean.Sp.BodyMass2
Mean.Raup.Crick2 ~ Total.Animals2 + Mean.Sp.BodyMass2
Brillouin.Index2 ~ Sp.Richness2 + Total.Animals2

insitu2 ~ Attendance2 + Zoo.Area.ha2 + Sp.Richness2 + Total.Animals2
+ Mam.Sp.Richness2 + Prop.Threat.Sp2
+ Mean.Sp.BodyMass2 + Brillouin.Index2 + Mean.Raup.Crick2'

# Fit model and generate model summary
mod.IS6.fit <- sem(mod.IS6, data = sem_in_situ_data, fixed.x=FALSE)
summary(mod.IS6.fit, rsq = TRUE)
```

```
## lavaan (0.5-23.1097) converged normally after  43 iterations
##
##   Number of observations                           119
##
##   Estimator                                         ML
##   Minimum Function Test Statistic               52.755
##   Degrees of freedom                                20
##   P-value (Chi-square)                           0.000
##
## Parameter Estimates:
##
##   Information                                 Expected
##   Standard Errors                             Standard
##
## Regressions:
##                      Estimate  Std.Err  z-value  P(>|z|)
##   Attendance2 ~
##     Sp.Richness2       -0.491    0.181   -2.715    0.007
##     Total.Animals2      0.735    0.137    5.367    0.000
##     Mam.Sp.Rchnss2      0.098    0.094    1.039    0.299
##     Prop.Thret.Sp2     -0.082    0.052   -1.579    0.114
##     Men.Sp.BdyMss2      0.300    0.064    4.655    0.000
##     Brilloun.Indx2      0.278    0.090    3.105    0.002
##     Mean.Rap.Crck2      0.135    0.060    2.232    0.026
##     X10km_Pop2          0.419    0.055    7.641    0.000
```

```
##   Total.Animals2 ~
##     Zoo.Area.ha2        0.142    0.049    2.903    0.004
##     Sp.Richness2        0.809    0.049   16.493    0.000
##   Sp.Richness2 ~
##     Mam.Sp.Rchnss2      0.877    0.055   15.817    0.000
##     Men.Sp.BdyMss2     -0.168    0.055   -3.028    0.002
##   Mean.Raup.Crick2 ~
##     Total.Animals2      0.427    0.073    5.874    0.000
##     Men.Sp.BdyMss2     -0.537    0.073   -7.354    0.000
##   Brillouin.Index2 ~
##     Sp.Richness2        1.573    0.094   16.667    0.000
##     Total.Animals2     -1.081    0.094  -11.496    0.000
##   insitu2 ~
##     Attendance2         0.613    0.103    5.975    0.000
##     Zoo.Area.ha2        0.207    0.088    2.340    0.019
##     Sp.Richness2        0.282    0.247    1.139    0.255
##     Total.Animals2     -0.429    0.199   -2.151    0.031
##     Mam.Sp.Rchnss2      0.156    0.131    1.193    0.233
##     Prop.Thret.Sp2      0.214    0.068    3.154    0.002
##     Men.Sp.BdyMss2     -0.089    0.095   -0.933    0.351
##     Brilloun.Indx2     -0.163    0.123   -1.322    0.186
##     Mean.Rap.Crck2      0.153    0.082    1.874    0.061
##
## Covariances:
##                        Estimate  Std.Err  z-value  P(>|z|)
##   Mam.Sp.Richness2 ~~
##     Prop.Thret.Sp2        0.322    0.096    3.368    0.001
##     Men.Sp.BdyMss2        0.373    0.097    3.845    0.000
##     X10km_Pop2            0.421    0.099    4.262    0.000
##     Zoo.Area.ha2          0.536    0.103    5.185    0.000
##   Prop.Threat.Sp2 ~~
##     Men.Sp.BdyMss2        0.277    0.094    2.933    0.003
##     X10km_Pop2            0.287    0.095    3.030    0.002
##     Zoo.Area.ha2          0.189    0.093    2.045    0.041
##   Mean.Sp.BodyMass2 ~~
##     X10km_Pop2           -0.023    0.091   -0.248    0.804
##     Zoo.Area.ha2          0.560    0.104    5.363    0.000
##   X10km_Pop2 ~~
##     Zoo.Area.ha2         -0.099    0.091   -1.087    0.277
##
## Variances:
##                    Estimate  Std.Err  z-value  P(>|z|)
##    .Attendance2       0.262    0.034    7.714    0.000
##    .Total.Animals2    0.243    0.032    7.714    0.000
##    .Sp.Richness2      0.311    0.040    7.714    0.000
##    .Mean.Rap.Crck2    0.602    0.078    7.714    0.000
##    .Brilloun.Indx2    0.274    0.036    7.714    0.000
##    .insitu2           0.467    0.060    7.714    0.000
##     Mam.Sp.Rchnss2    0.992    0.129    7.714    0.000
##     Prop.Thret.Sp2    0.992    0.129    7.714    0.000
##     Men.Sp.BdyMss2    0.992    0.129    7.714    0.000
##     X10km_Pop2        0.992    0.129    7.714    0.000
##     Zoo.Area.ha2      0.992    0.129    7.714    0.000
##
```

```
## R-Square:
##                    Estimate
##     Attendance2       0.721
##     Total.Animals2    0.757
##     Sp.Richness2      0.686
##     Mean.Rap.Crck2    0.382
##     Brilloun.Indx2    0.722
##     insitu2           0.531
```

```
# Generate fit indices
fitMeasures(mod.IS6.fit, c("agfi", "rmr", "srmr", "rmsea", "cfi", "nnfi", "tli"))
```

```
##  agfi   rmr  srmr rmsea   cfi  nnfi   tli
## 0.775 0.054 0.054 0.117 0.958 0.905 0.905
```

```
# Generate modification indices
miIS6 <- modindices(mod.IS6.fit)
print(miIS6[miIS6$mi > 3.0,])
```

```
##                   lhs op             rhs     mi    epc sepc.lv sepc.all
## 72      Sp.Richness2  ~      Attendance2 10.639  0.395   0.395    0.384
## 76      Sp.Richness2  ~          insitu2  7.917  0.428   0.428    0.429
## 78      Sp.Richness2  ~       X10km_Pop2 17.968  0.246   0.246    0.246
## 85  Mean.Raup.Crick2  ~  Prop.Threat.Sp2 13.894 -0.281  -0.281   -0.284
## 87  Mean.Raup.Crick2  ~      Zoo.Area.ha2  3.986  0.189   0.189    0.191
## 97  Mam.Sp.Richness2  ~      Attendance2  4.200 -0.335  -0.335   -0.326
## 98  Mam.Sp.Richness2  ~    Total.Animals2  4.646 -0.279  -0.279   -0.280
## 99  Mam.Sp.Richness2  ~      Sp.Richness2  9.049 -0.500  -0.500   -0.500
## 100 Mam.Sp.Richness2  ~ Mean.Raup.Crick2  3.537 -0.167  -0.167   -0.165
## 110  Prop.Threat.Sp2  ~ Mean.Raup.Crick2 11.924 -0.346  -0.346   -0.343
## 112  Prop.Threat.Sp2  ~          insitu2  9.021 -1.069  -1.069   -1.072
## 127       X10km_Pop2  ~      Attendance2  9.166  0.544   0.544    0.529
## 128       X10km_Pop2  ~    Total.Animals2 13.199  0.396   0.396    0.397
## 129       X10km_Pop2  ~      Sp.Richness2 18.045  0.554   0.554    0.554
## 130       X10km_Pop2  ~ Mean.Raup.Crick2  5.427  0.205   0.205    0.203
## 137     Zoo.Area.ha2  ~      Attendance2  3.043  0.186   0.186    0.181
## 140     Zoo.Area.ha2  ~ Mean.Raup.Crick2  6.890  0.204   0.204    0.202
##     sepc.nox
## 72     0.384
## 76     0.429
## 78     0.247
## 85    -0.285
## 87     0.192
## 97    -0.326
## 98    -0.280
## 99    -0.500
## 100   -0.165
## 110   -0.343
## 112   -1.072
## 127    0.529
## 128    0.397
## 129    0.554
## 130    0.203
## 137    0.181
## 140    0.202
```

Model 7

Based on the results generated from the sixth model, the highest p-value relationship to be considered for removal is **insitu2 ~ Men.Sp.BdyMss2** with a p-value of **0.351**. Therefore we decide to remove this pathway. Once again, the model summary, fit indices and modification indices were all generated for the model.

```
# In Situ SEM (Presence-Absence)

# Model 7
# Removal of insitu2 ~ Men.Sp.BdyMss2, p = 0.351

mod.IS7 <- 'Attendance2 ~ Sp.Richness2 + Total.Animals2
+ Mam.Sp.Richness2 + Prop.Threat.Sp2
+ Mean.Sp.BodyMass2 + Brillouin.Index2 + Mean.Raup.Crick2
+ X10km_Pop2

Total.Animals2 ~ Zoo.Area.ha2 + Sp.Richness2
Sp.Richness2 ~ Mam.Sp.Richness2 + Mean.Sp.BodyMass2
Mean.Raup.Crick2 ~ Total.Animals2 + Mean.Sp.BodyMass2
Brillouin.Index2 ~ Sp.Richness2 + Total.Animals2

insitu2 ~ Attendance2 + Zoo.Area.ha2 + Sp.Richness2 + Total.Animals2
+ Mam.Sp.Richness2 + Prop.Threat.Sp2
+ Brillouin.Index2 + Mean.Raup.Crick2'

# Fit model and generate model summary
mod.IS7.fit <- sem(mod.IS7, data = sem_in_situ_data, fixed.x=FALSE)
summary(mod.IS7.fit, rsq = TRUE)
```

```
## lavaan (0.5-23.1097) converged normally after  41 iterations
##
##   Number of observations                           119
##
##   Estimator                                         ML
##   Minimum Function Test Statistic               53.553
##   Degrees of freedom                                21
##   P-value (Chi-square)                           0.000
##
## Parameter Estimates:
##
##   Information                                 Expected
##   Standard Errors                             Standard
##
## Regressions:
##                    Estimate  Std.Err  z-value  P(>|z|)
##   Attendance2 ~
##     Sp.Richness2     -0.491    0.181   -2.715    0.007
##     Total.Animals2    0.735    0.137    5.367    0.000
##     Mam.Sp.Rchnss2    0.098    0.094    1.039    0.299
##     Prop.Thret.Sp2   -0.082    0.052   -1.579    0.114
##     Men.Sp.BdyMss2    0.300    0.064    4.655    0.000
##     Brilloun.Indx2    0.278    0.090    3.105    0.002
##     Mean.Rap.Crck2    0.135    0.060    2.232    0.026
##     X10km_Pop2        0.419    0.055    7.641    0.000
```

```
##   Total.Animals2 ~
##     Zoo.Area.ha2        0.142    0.049    2.903    0.004
##     Sp.Richness2        0.809    0.049   16.493    0.000
##   Sp.Richness2 ~
##     Mam.Sp.Rchnss2      0.877    0.055   15.817    0.000
##     Men.Sp.BdyMss2     -0.168    0.055   -3.028    0.002
##   Mean.Raup.Crick2 ~
##     Total.Animals2      0.427    0.073    5.874    0.000
##     Men.Sp.BdyMss2     -0.537    0.073   -7.354    0.000
##   Brillouin.Index2 ~
##     Sp.Richness2        1.573    0.094   16.667    0.000
##     Total.Animals2     -1.081    0.094  -11.496    0.000
##   insitu2 ~
##     Attendance2         0.581    0.098    5.908    0.000
##     Zoo.Area.ha2        0.164    0.080    2.043    0.041
##     Sp.Richness2        0.257    0.248    1.038    0.299
##     Total.Animals2     -0.395    0.200   -1.978    0.048
##     Mam.Sp.Rchnss2      0.149    0.131    1.139    0.255
##     Prop.Thret.Sp2      0.213    0.067    3.178    0.001
##     Brilloun.Indx2     -0.143    0.123   -1.161    0.246
##     Mean.Rap.Crck2      0.196    0.071    2.746    0.006
##
## Covariances:
##                        Estimate  Std.Err  z-value  P(>|z|)
##   Mam.Sp.Richness2 ~~
##     Prop.Thret.Sp2        0.322    0.096    3.368    0.001
##     Men.Sp.BdyMss2        0.373    0.097    3.845    0.000
##     X10km_Pop2            0.421    0.099    4.262    0.000
##     Zoo.Area.ha2          0.536    0.103    5.185    0.000
##   Prop.Threat.Sp2 ~~
##     Men.Sp.BdyMss2        0.277    0.094    2.933    0.003
##     X10km_Pop2            0.287    0.095    3.030    0.002
##     Zoo.Area.ha2          0.189    0.093    2.045    0.041
##   Mean.Sp.BodyMass2 ~~
##     X10km_Pop2           -0.023    0.091   -0.248    0.804
##     Zoo.Area.ha2          0.560    0.104    5.363    0.000
##   X10km_Pop2 ~~
##     Zoo.Area.ha2         -0.099    0.091   -1.087    0.277
##
## Variances:
##                    Estimate  Std.Err  z-value  P(>|z|)
##    .Attendance2       0.262    0.034    7.714    0.000
##    .Total.Animals2    0.243    0.032    7.714    0.000
##    .Sp.Richness2      0.311    0.040    7.714    0.000
##    .Mean.Rap.Crck2    0.602    0.078    7.714    0.000
##    .Brilloun.Indx2    0.274    0.036    7.714    0.000
##    .insitu2           0.470    0.061    7.714    0.000
##     Mam.Sp.Rchnss2    0.992    0.129    7.714    0.000
##     Prop.Thret.Sp2    0.992    0.129    7.714    0.000
##     Men.Sp.BdyMss2    0.992    0.129    7.714    0.000
##     X10km_Pop2        0.992    0.129    7.714    0.000
##     Zoo.Area.ha2      0.992    0.129    7.714    0.000
##
## R-Square:
```

```
##                     Estimate
##     Attendance2        0.721
##     Total.Animals2     0.757
##     Sp.Richness2       0.686
##     Mean.Rap.Crck2     0.382
##     Brilloun.Indx2     0.722
##     insitu2            0.530
```

```r
# Generate fit indices
fitMeasures(mod.IS7.fit, c("agfi", "rmr", "srmr", "rmsea", "cfi", "nnfi", "tli"))
```

```
##  agfi   rmr  srmr rmsea   cfi  nnfi   tli
## 0.783 0.054 0.055 0.114 0.958 0.910 0.910
```

```r
# Generate modification indices
miIS7 <- modindices(mod.IS7.fit)
print(miIS7[miIS7$mi > 3.0,])
```

```
##                  lhs op              rhs     mi    epc sepc.lv sepc.all
## 71      Sp.Richness2  ~      Attendance2 10.639  0.395   0.395    0.384
## 75      Sp.Richness2  ~          insitu2  5.059  0.314   0.314    0.316
## 77      Sp.Richness2  ~       X10km_Pop2 17.968  0.246   0.246    0.246
## 82  Mean.Raup.Crick2  ~          insitu2  3.277 -0.258  -0.258   -0.262
## 84  Mean.Raup.Crick2  ~   Prop.Threat.Sp2 13.894 -0.281  -0.281   -0.284
## 86  Mean.Raup.Crick2  ~      Zoo.Area.ha2  3.986  0.189   0.189    0.191
## 97  Mam.Sp.Richness2  ~      Attendance2  4.200 -0.335  -0.335   -0.326
## 98  Mam.Sp.Richness2  ~    Total.Animals2  4.646 -0.279  -0.279   -0.280
## 99  Mam.Sp.Richness2  ~      Sp.Richness2  9.050 -0.500  -0.500   -0.500
## 100 Mam.Sp.Richness2  ~ Mean.Raup.Crick2  3.537 -0.167  -0.167   -0.165
## 110  Prop.Threat.Sp2  ~ Mean.Raup.Crick2 11.924 -0.346  -0.346   -0.343
## 112  Prop.Threat.Sp2  ~          insitu2  6.945 -0.798  -0.798   -0.802
## 127       X10km_Pop2  ~      Attendance2  9.166  0.544   0.544    0.529
## 128       X10km_Pop2  ~    Total.Animals2 13.199  0.396   0.396    0.397
## 129       X10km_Pop2  ~      Sp.Richness2 18.045  0.554   0.554    0.554
## 130       X10km_Pop2  ~ Mean.Raup.Crick2  5.427  0.205   0.205    0.203
## 137     Zoo.Area.ha2  ~      Attendance2  3.043  0.186   0.186    0.181
## 140     Zoo.Area.ha2  ~ Mean.Raup.Crick2  6.890  0.204   0.204    0.202
## 142     Zoo.Area.ha2  ~          insitu2  3.637  0.234   0.234    0.235
##     sepc.nox
## 71     0.384
## 75     0.316
## 77     0.247
## 82    -0.262
## 84    -0.285
## 86     0.192
## 97    -0.326
## 98    -0.280
## 99    -0.500
## 100   -0.165
## 110   -0.343
## 112   -0.802
## 127    0.529
## 128    0.397
## 129    0.554
## 130    0.203
## 137    0.181
```

```
## 140    0.202
## 142    0.235
```

Model 8

Based on the results generated from the seventh model, the highest p-value relationship to be considered for removal is **Attendance2** ~ **Mam.Sp.Rchnss2** with a p-value of **0.299**. Therefore we decide to remove this pathway. Once again, the model summary, fit indices and modification indices were all generated for the model.

```
# In Situ SEM (Presence-Absence)

# Model 8
# Removal of Attendance2 ~ Mam.Sp.Rchnss2, p = 0.299

mod.IS8 <- 'Attendance2 ~ Sp.Richness2 + Total.Animals2
+ Prop.Threat.Sp2 + Mean.Sp.BodyMass2 + Brillouin.Index2
+ Mean.Raup.Crick2 + X10km_Pop2

Total.Animals2 ~ Zoo.Area.ha2 + Sp.Richness2
Sp.Richness2 ~ Mam.Sp.Richness2 + Mean.Sp.BodyMass2
Mean.Raup.Crick2 ~ Total.Animals2 + Mean.Sp.BodyMass2
Brillouin.Index2 ~ Sp.Richness2 + Total.Animals2

insitu2 ~ Attendance2 + Zoo.Area.ha2 + Sp.Richness2 + Total.Animals2
+ Mam.Sp.Richness2 + Prop.Threat.Sp2
+ Brillouin.Index2 + Mean.Raup.Crick2'

# Fit model and generate model summary
mod.IS8.fit <- sem(mod.IS8, data = sem_in_situ_data, fixed.x=FALSE)
summary(mod.IS8.fit, rsq = TRUE)
```

```
## lavaan (0.5-23.1097) converged normally after  40 iterations
##
##   Number of observations                           119
##
##   Estimator                                         ML
##   Minimum Function Test Statistic               54.684
##   Degrees of freedom                                22
##   P-value (Chi-square)                           0.000
##
## Parameter Estimates:
##
##   Information                                 Expected
##   Standard Errors                             Standard
##
## Regressions:
##                    Estimate  Std.Err  z-value  P(>|z|)
##   Attendance2 ~
##     Sp.Richness2     -0.426    0.171   -2.485    0.013
##     Total.Animals2    0.751    0.137    5.465    0.000
##     Prop.Thret.Sp2   -0.077    0.052   -1.470    0.141
##     Men.Sp.BdyMss2    0.320    0.060    5.309    0.000
##     Brilloun.Indx2    0.277    0.090    3.070    0.002
```

```
##     Mean.Rap.Crck2      0.131    0.061    2.162    0.031
##     X10km_Pop2          0.416    0.053    7.825    0.000
##   Total.Animals2 ~
##     Zoo.Area.ha2        0.142    0.049    2.903    0.004
##     Sp.Richness2        0.809    0.049   16.493    0.000
##   Sp.Richness2 ~
##     Mam.Sp.Rchnss2      0.877    0.055   15.817    0.000
##     Men.Sp.BdyMss2     -0.168    0.055   -3.028    0.002
##   Mean.Raup.Crick2 ~
##     Total.Animals2      0.427    0.073    5.874    0.000
##     Men.Sp.BdyMss2     -0.537    0.073   -7.354    0.000
##   Brillouin.Index2 ~
##     Sp.Richness2        1.573    0.094   16.667    0.000
##     Total.Animals2     -1.081    0.094  -11.496    0.000
##   insitu2 ~
##     Attendance2         0.581    0.098    5.952    0.000
##     Zoo.Area.ha2        0.164    0.080    2.045    0.041
##     Sp.Richness2        0.257    0.247    1.043    0.297
##     Total.Animals2     -0.395    0.200   -1.973    0.049
##     Mam.Sp.Rchnss2      0.149    0.128    1.164    0.244
##     Prop.Thret.Sp2      0.213    0.067    3.176    0.001
##     Brilloun.Indx2     -0.143    0.123   -1.162    0.245
##     Mean.Rap.Crck2      0.196    0.071    2.747    0.006
##
## Covariances:
##                       Estimate  Std.Err  z-value  P(>|z|)
##   Prop.Threat.Sp2 ~~
##     Men.Sp.BdyMss2        0.277    0.094    2.933    0.003
##     X10km_Pop2            0.287    0.095    3.030    0.002
##     Zoo.Area.ha2          0.189    0.093    2.045    0.041
##     Mam.Sp.Rchnss2        0.322    0.096    3.368    0.001
##   Mean.Sp.BodyMass2 ~~
##     X10km_Pop2           -0.023    0.091   -0.248    0.804
##     Zoo.Area.ha2          0.560    0.104    5.363    0.000
##     Mam.Sp.Rchnss2        0.373    0.097    3.845    0.000
##   X10km_Pop2 ~~
##     Zoo.Area.ha2         -0.099    0.091   -1.087    0.277
##     Mam.Sp.Rchnss2        0.421    0.099    4.262    0.000
##   Zoo.Area.ha2 ~~
##     Mam.Sp.Rchnss2        0.536    0.103    5.185    0.000
##
## Variances:
##                   Estimate  Std.Err  z-value  P(>|z|)
##    .Attendance2       0.265    0.034    7.714    0.000
##    .Total.Animals2    0.243    0.032    7.714    0.000
##    .Sp.Richness2      0.311    0.040    7.714    0.000
##    .Mean.Rap.Crck2    0.602    0.078    7.714    0.000
##    .Brilloun.Indx2    0.274    0.036    7.714    0.000
##    .insitu2           0.470    0.061    7.714    0.000
##     Prop.Thret.Sp2    0.992    0.129    7.714    0.000
##     Men.Sp.BdyMss2    0.992    0.129    7.714    0.000
##     X10km_Pop2        0.992    0.129    7.714    0.000
##     Zoo.Area.ha2      0.992    0.129    7.714    0.000
##     Mam.Sp.Rchnss2    0.992    0.129    7.714    0.000
```

```
##
## R-Square:
##                    Estimate
##     Attendance2       0.715
##     Total.Animals2    0.757
##     Sp.Richness2      0.686
##     Mean.Rap.Crck2    0.382
##     Brilloun.Indx2    0.722
##     insitu2           0.525
```

```r
# Generate fit indices
fitMeasures(mod.IS8.fit, c("agfi", "rmr", "srmr", "rmsea", "cfi", "nnfi", "tli"))
```

```
##  agfi   rmr  srmr rmsea   cfi  nnfi   tli
## 0.788 0.055 0.056 0.112 0.958 0.914 0.914
```

```r
# Generate modification indices
miIS8 <- modindices(mod.IS8.fit)
print(miIS8[miIS8$mi > 3.0,])
```

```
##                   lhs op              rhs     mi    epc sepc.lv sepc.all
## 77       Sp.Richness2  ~       X10km_Pop2 17.968  0.246   0.246    0.246
## 82   Mean.Raup.Crick2  ~          insitu2  3.340 -0.268  -0.268   -0.270
## 83   Mean.Raup.Crick2  ~  Prop.Threat.Sp2 13.894 -0.281  -0.281   -0.284
## 85   Mean.Raup.Crick2  ~      Zoo.Area.ha2  3.986  0.189   0.189    0.191
## 100   Prop.Threat.Sp2  ~ Mean.Raup.Crick2 11.924 -0.346  -0.346   -0.343
## 102   Prop.Threat.Sp2  ~          insitu2  7.206 -0.794  -0.794   -0.793
## 117        X10km_Pop2  ~      Attendance2  7.826  0.453   0.453    0.439
## 118        X10km_Pop2  ~    Total.Animals2 13.199  0.396   0.396    0.397
## 119        X10km_Pop2  ~      Sp.Richness2 18.045  0.554   0.554    0.554
## 120        X10km_Pop2  ~ Mean.Raup.Crick2  5.427  0.205   0.205    0.203
## 130      Zoo.Area.ha2  ~ Mean.Raup.Crick2  6.890  0.204   0.204    0.202
## 132      Zoo.Area.ha2  ~          insitu2  3.499  0.227   0.227    0.227
## 138 Mam.Sp.Richness2  ~    Total.Animals2  4.646 -0.279  -0.279   -0.280
## 139 Mam.Sp.Richness2  ~      Sp.Richness2  9.050 -0.500  -0.500   -0.500
## 140 Mam.Sp.Richness2  ~ Mean.Raup.Crick2  3.537 -0.167  -0.167   -0.165
##     sepc.nox
## 77     0.247
## 82    -0.270
## 83    -0.285
## 85     0.192
## 100   -0.343
## 102   -0.793
## 117    0.439
## 118    0.397
## 119    0.554
## 120    0.203
## 130    0.202
## 132    0.227
## 138   -0.280
## 139   -0.500
## 140   -0.165
```

Model 9

Based on the results generated from the eight model, the highest p-value relationship to be considered for removal is **insitu2 ~ Sp.Richness2** with a p-value of **0.297**. Therefore we decide to remove this pathway. Once again, the model summary, fit indices and modification indices were all generated for the model.

```
# In Situ SEM (Presence-Absence)

# Model 9
# Removal of insitu2 ~ Sp.Richness2, p = 0.297

mod.IS9 <- 'Attendance2 ~ Sp.Richness2 + Total.Animals2
+ Prop.Threat.Sp2 + Mean.Sp.BodyMass2 + Brillouin.Index2
+ Mean.Raup.Crick2 + X10km_Pop2

Total.Animals2 ~ Zoo.Area.ha2 + Sp.Richness2
Sp.Richness2 ~ Mam.Sp.Richness2 + Mean.Sp.BodyMass2
Mean.Raup.Crick2 ~ Total.Animals2 + Mean.Sp.BodyMass2
Brillouin.Index2 ~ Sp.Richness2 + Total.Animals2

insitu2 ~ Attendance2 + Zoo.Area.ha2 + Total.Animals2
+ Mam.Sp.Richness2 + Prop.Threat.Sp2
+ Brillouin.Index2 + Mean.Raup.Crick2'

# Fit model and generate model summary
mod.IS9.fit <- sem(mod.IS9, data = sem_in_situ_data, fixed.x=FALSE)
summary(mod.IS9.fit, rsq = TRUE)
```

```
## lavaan (0.5-23.1097) converged normally after  36 iterations
##
##   Number of observations                           119
##
##   Estimator                                         ML
##   Minimum Function Test Statistic               55.739
##   Degrees of freedom                                23
##   P-value (Chi-square)                           0.000
##
## Parameter Estimates:
##
##   Information                                 Expected
##   Standard Errors                             Standard
##
## Regressions:
##                    Estimate  Std.Err  z-value  P(>|z|)
##   Attendance2 ~
##     Sp.Richness2     -0.426    0.171   -2.485    0.013
##     Total.Animals2    0.751    0.137    5.465    0.000
##     Prop.Thret.Sp2   -0.077    0.052   -1.470    0.141
##     Men.Sp.BdyMss2    0.320    0.060    5.309    0.000
##     Brilloun.Indx2    0.277    0.090    3.070    0.002
##     Mean.Rap.Crck2    0.131    0.061    2.162    0.031
##     X10km_Pop2        0.416    0.053    7.825    0.000
##   Total.Animals2 ~
##     Zoo.Area.ha2      0.142    0.049    2.903    0.004
##     Sp.Richness2      0.809    0.049   16.493    0.000
```

```
##   Sp.Richness2 ~
##     Mam.Sp.Rchnss2      0.877    0.055   15.817    0.000
##     Men.Sp.BdyMss2     -0.168    0.055   -3.028    0.002
##   Mean.Raup.Crick2 ~
##     Total.Animals2      0.427    0.073    5.874    0.000
##     Men.Sp.BdyMss2     -0.537    0.073   -7.354    0.000
##   Brillouin.Index2 ~
##     Sp.Richness2        1.573    0.094   16.667    0.000
##     Total.Animals2     -1.081    0.094  -11.496    0.000
##   insitu2 ~
##     Attendance2         0.572    0.096    5.954    0.000
##     Zoo.Area.ha2        0.140    0.080    1.765    0.078
##     Total.Animals2     -0.225    0.112   -2.012    0.044
##     Mam.Sp.Rchnss2      0.203    0.118    1.716    0.086
##     Prop.Thret.Sp2      0.208    0.067    3.086    0.002
##     Brilloun.Indx2     -0.040    0.076   -0.534    0.593
##     Mean.Rap.Crck2      0.195    0.071    2.734    0.006
##
## Covariances:
##                        Estimate  Std.Err  z-value  P(>|z|)
##   Prop.Threat.Sp2 ~~
##     Men.Sp.BdyMss2        0.277    0.094    2.933    0.003
##     X10km_Pop2            0.287    0.095    3.030    0.002
##     Zoo.Area.ha2          0.189    0.093    2.045    0.041
##     Mam.Sp.Rchnss2        0.322    0.096    3.368    0.001
##   Mean.Sp.BodyMass2 ~~
##     X10km_Pop2           -0.023    0.091   -0.248    0.804
##     Zoo.Area.ha2          0.560    0.104    5.363    0.000
##     Mam.Sp.Rchnss2        0.373    0.097    3.845    0.000
##   X10km_Pop2 ~~
##     Zoo.Area.ha2         -0.099    0.091   -1.087    0.277
##     Mam.Sp.Rchnss2        0.421    0.099    4.262    0.000
##   Zoo.Area.ha2 ~~
##     Mam.Sp.Rchnss2        0.536    0.103    5.185    0.000
##
## Variances:
##                    Estimate  Std.Err  z-value  P(>|z|)
##    .Attendance2       0.265    0.034    7.714    0.000
##    .Total.Animals2    0.243    0.032    7.714    0.000
##    .Sp.Richness2      0.311    0.040    7.714    0.000
##    .Mean.Rap.Crck2    0.602    0.078    7.714    0.000
##    .Brilloun.Indx2    0.274    0.036    7.714    0.000
##    .insitu2           0.474    0.061    7.714    0.000
##     Prop.Thret.Sp2    0.992    0.129    7.714    0.000
##     Men.Sp.BdyMss2    0.992    0.129    7.714    0.000
##     X10km_Pop2        0.992    0.129    7.714    0.000
##     Zoo.Area.ha2      0.992    0.129    7.714    0.000
##     Mam.Sp.Rchnss2    0.992    0.129    7.714    0.000
##
## R-Square:
##                    Estimate
##     Attendance2       0.715
##     Total.Animals2    0.757
##     Sp.Richness2      0.686
```

```
##     Mean.Rap.Crck2    0.382
##     Brilloun.Indx2    0.722
##     insitu2           0.522
```

```r
# Generate fit indices
fitMeasures(mod.IS9.fit, c("agfi", "rmr", "srmr", "rmsea", "cfi", "nnfi", "tli"))
```

```
##  agfi   rmr  srmr rmsea   cfi  nnfi   tli
## 0.794 0.055 0.056 0.109 0.958 0.917 0.917
```

```r
# Generate modification indices
miIS9 <- modindices(mod.IS9.fit)
print(miIS9[miIS9$mi > 3.0,])
```

```
##                    lhs op              rhs     mi    epc sepc.lv sepc.all
## 76      Sp.Richness2  ~       X10km_Pop2 17.968  0.246   0.246    0.246
## 81  Mean.Raup.Crick2  ~          insitu2  3.214 -0.262  -0.262   -0.264
## 82  Mean.Raup.Crick2  ~  Prop.Threat.Sp2 13.894 -0.281  -0.281   -0.284
## 84  Mean.Raup.Crick2  ~      Zoo.Area.ha2  3.986  0.189   0.189    0.191
## 100  Prop.Threat.Sp2  ~ Mean.Raup.Crick2 11.924 -0.346  -0.346   -0.343
## 102  Prop.Threat.Sp2  ~          insitu2  6.078 -0.711  -0.711   -0.711
## 117       X10km_Pop2  ~      Attendance2  7.826  0.453   0.453    0.439
## 118       X10km_Pop2  ~   Total.Animals2 13.199  0.396   0.396    0.397
## 119       X10km_Pop2  ~     Sp.Richness2 18.045  0.554   0.554    0.554
## 120       X10km_Pop2  ~ Mean.Raup.Crick2  5.427  0.205   0.205    0.203
## 130     Zoo.Area.ha2  ~ Mean.Raup.Crick2  6.889  0.204   0.204    0.202
## 132     Zoo.Area.ha2  ~          insitu2  4.384  0.254   0.254    0.254
## 138 Mam.Sp.Richness2  ~   Total.Animals2  4.646 -0.279  -0.279   -0.280
## 139 Mam.Sp.Richness2  ~     Sp.Richness2  9.049 -0.500  -0.500   -0.500
## 140 Mam.Sp.Richness2  ~ Mean.Raup.Crick2  3.537 -0.167  -0.167   -0.165
##     sepc.nox
## 76     0.247
## 81    -0.264
## 82    -0.285
## 84     0.192
## 100   -0.343
## 102   -0.711
## 117    0.439
## 118    0.397
## 119    0.554
## 120    0.203
## 130    0.202
## 132    0.254
## 138   -0.280
## 139   -0.500
## 140   -0.165
```

Model 10

Based on the results generated from the ninth model, the highest p-value relationship to be considered for removal is **insitu2 ~ Brilloun.Indx2** with a p-value of **0.593**. Therefore we decide to remove this pathway. Once again, the model summary, fit indices and modification indices were all generated for the model.

```r
# In Situ SEM (Presence-Absence)
```

```r
# Model 10
# Removal of insitu2 ~ Brilloun.Indx2, p = 0.593

mod.IS10 <- 'Attendance2 ~ Sp.Richness2 + Total.Animals2
+ Prop.Threat.Sp2 + Mean.Sp.BodyMass2 + Brillouin.Index2
+ Mean.Raup.Crick2 + X10km_Pop2

Total.Animals2 ~ Zoo.Area.ha2 + Sp.Richness2
Sp.Richness2 ~ Mam.Sp.Richness2 + Mean.Sp.BodyMass2
Mean.Raup.Crick2 ~ Total.Animals2 + Mean.Sp.BodyMass2
Brillouin.Index2 ~ Sp.Richness2 + Total.Animals2

insitu2 ~ Attendance2 + Zoo.Area.ha2 + Total.Animals2
+ Mam.Sp.Richness2 + Prop.Threat.Sp2
+ Mean.Raup.Crick2'

# Fit model and generate model summary
mod.IS10.fit <- sem(mod.IS10, data = sem_in_situ_data, fixed.x=FALSE)
summary(mod.IS10.fit, rsq = TRUE)
```

```
## lavaan (0.5-23.1097) converged normally after  35 iterations
##
##   Number of observations                           119
##
##   Estimator                                         ML
##   Minimum Function Test Statistic               56.035
##   Degrees of freedom                                24
##   P-value (Chi-square)                           0.000
##
## Parameter Estimates:
##
##   Information                                 Expected
##   Standard Errors                             Standard
##
## Regressions:
##                    Estimate  Std.Err  z-value  P(>|z|)
##   Attendance2 ~
##     Sp.Richness2     -0.426    0.171   -2.485    0.013
##     Total.Animals2    0.751    0.137    5.465    0.000
##     Prop.Thret.Sp2   -0.077    0.052   -1.470    0.141
##     Men.Sp.BdyMss2    0.320    0.060    5.309    0.000
##     Brilloun.Indx2    0.277    0.090    3.070    0.002
##     Mean.Rap.Crck2    0.131    0.061    2.162    0.031
##     X10km_Pop2        0.416    0.053    7.825    0.000
##   Total.Animals2 ~
##     Zoo.Area.ha2      0.142    0.049    2.903    0.004
##     Sp.Richness2      0.809    0.049   16.493    0.000
##   Sp.Richness2 ~
##     Mam.Sp.Rchnss2    0.877    0.055   15.817    0.000
##     Men.Sp.BdyMss2   -0.168    0.055   -3.028    0.002
##   Mean.Raup.Crick2 ~
##     Total.Animals2    0.427    0.073    5.874    0.000
##     Men.Sp.BdyMss2   -0.537    0.073   -7.354    0.000
##   Brillouin.Index2 ~
```

```
##     Sp.Richness2        1.573    0.094   16.667    0.000
##     Total.Animals2     -1.081    0.094  -11.496    0.000
##   insitu2 ~
##     Attendance2         0.563    0.096    5.886    0.000
##     Zoo.Area.ha2        0.146    0.079    1.853    0.064
##     Total.Animals2     -0.210    0.110   -1.902    0.057
##     Mam.Sp.Rchnss2      0.177    0.106    1.665    0.096
##     Prop.Thret.Sp2      0.206    0.068    3.048    0.002
##     Mean.Rap.Crck2      0.190    0.071    2.664    0.008
##
## Covariances:
##                        Estimate  Std.Err  z-value  P(>|z|)
##   Prop.Threat.Sp2 ~~
##     Men.Sp.BdyMss2        0.277    0.094    2.933    0.003
##     X10km_Pop2            0.287    0.095    3.030    0.002
##     Zoo.Area.ha2          0.189    0.093    2.045    0.041
##     Mam.Sp.Rchnss2        0.322    0.096    3.368    0.001
##   Mean.Sp.BodyMass2 ~~
##     X10km_Pop2           -0.023    0.091   -0.248    0.804
##     Zoo.Area.ha2          0.560    0.104    5.363    0.000
##     Mam.Sp.Rchnss2        0.373    0.097    3.845    0.000
##   X10km_Pop2 ~~
##     Zoo.Area.ha2         -0.099    0.091   -1.087    0.277
##     Mam.Sp.Rchnss2        0.421    0.099    4.262    0.000
##   Zoo.Area.ha2 ~~
##     Mam.Sp.Rchnss2        0.536    0.103    5.185    0.000
##
## Variances:
##                    Estimate  Std.Err  z-value  P(>|z|)
##    .Attendance2       0.265    0.034    7.714    0.000
##    .Total.Animals2    0.243    0.032    7.714    0.000
##    .Sp.Richness2      0.311    0.040    7.714    0.000
##    .Mean.Rap.Crck2    0.602    0.078    7.714    0.000
##    .Brilloun.Indx2    0.274    0.036    7.714    0.000
##    .insitu2           0.475    0.062    7.714    0.000
##     Prop.Thret.Sp2    0.992    0.129    7.714    0.000
##     Men.Sp.BdyMss2    0.992    0.129    7.714    0.000
##     X10km_Pop2        0.992    0.129    7.714    0.000
##     Zoo.Area.ha2      0.992    0.129    7.714    0.000
##     Mam.Sp.Rchnss2    0.992    0.129    7.714    0.000
##
## R-Square:
##                    Estimate
##     Attendance2       0.715
##     Total.Animals2    0.757
##     Sp.Richness2      0.686
##     Mean.Rap.Crck2    0.382
##     Brilloun.Indx2    0.722
##     insitu2           0.519
```

```r
# Generate fit indices
fitMeasures(mod.IS10.fit, c("agfi", "rmr", "srmr", "rmsea", "cfi", "nnfi", "tli"))
```

```
##  agfi   rmr  srmr rmsea   cfi  nnfi   tli
## 0.802 0.055 0.055 0.106 0.959 0.922 0.922
```

```r
# Generate modification indices
miIS10 <- modindices(mod.IS10.fit)
print(miIS10[miIS10$mi > 3.0,])
```

```
##                  lhs op              rhs     mi    epc sepc.lv sepc.all
## 75      Sp.Richness2  ~       X10km_Pop2 17.968  0.246   0.246    0.246
## 81  Mean.Raup.Crick2  ~  Prop.Threat.Sp2 13.894 -0.281  -0.281   -0.284
## 83  Mean.Raup.Crick2  ~     Zoo.Area.ha2  3.986  0.189   0.189    0.191
## 100  Prop.Threat.Sp2  ~ Mean.Raup.Crick2 11.924 -0.346  -0.346   -0.343
## 102  Prop.Threat.Sp2  ~          insitu2  5.722 -0.686  -0.686   -0.685
## 117       X10km_Pop2  ~      Attendance2  7.826  0.453   0.453    0.439
## 118       X10km_Pop2  ~   Total.Animals2 13.199  0.396   0.396    0.397
## 119       X10km_Pop2  ~     Sp.Richness2 18.045  0.554   0.554    0.554
## 120       X10km_Pop2  ~ Mean.Raup.Crick2  5.427  0.205   0.205    0.203
## 130     Zoo.Area.ha2  ~ Mean.Raup.Crick2  6.889  0.204   0.204    0.202
## 132     Zoo.Area.ha2  ~          insitu2  4.372  0.254   0.254    0.253
## 138 Mam.Sp.Richness2  ~   Total.Animals2  4.646 -0.279  -0.279   -0.280
## 139 Mam.Sp.Richness2  ~     Sp.Richness2  9.049 -0.500  -0.500   -0.500
## 140 Mam.Sp.Richness2  ~ Mean.Raup.Crick2  3.537 -0.167  -0.167   -0.165
##     sepc.nox
## 75     0.247
## 81    -0.285
## 83     0.192
## 100   -0.343
## 102   -0.685
## 117    0.439
## 118    0.397
## 119    0.554
## 120    0.203
## 130    0.202
## 132    0.253
## 138   -0.280
## 139   -0.500
## 140   -0.165
```

Model 11

Based on the results generated from the tenth model, the highest p-value relationship to be considered for removal is **Attendance2** ~ **Prop.Thret.Sp2** with a p-value of **0.141**. Therefore we decide to remove this pathway. Once again, the model summary, fit indices and modification indices were all generated for the model.

```r
# In Situ SEM (Presence-Absence)

# Model 11
# Removal of Attendance2 ~ Prop.Thret.Sp2, p = 0.141

mod.IS11 <- 'Attendance2 ~ Sp.Richness2 + Total.Animals2
+ Mean.Sp.BodyMass2 + Brillouin.Index2
+ Mean.Raup.Crick2 + X10km_Pop2

Total.Animals2 ~ Zoo.Area.ha2 + Sp.Richness2
Sp.Richness2 ~ Mam.Sp.Richness2 + Mean.Sp.BodyMass2
```

```
Mean.Raup.Crick2 ~ Total.Animals2 + Mean.Sp.BodyMass2
Brillouin.Index2 ~ Sp.Richness2 + Total.Animals2

insitu2 ~ Attendance2 + Zoo.Area.ha2 + Total.Animals2
+ Mam.Sp.Richness2 + Prop.Threat.Sp2
+ Mean.Raup.Crick2'

# Fit model and generate model summary
mod.IS11.fit <- sem(mod.IS11, data = sem_in_situ_data, fixed.x=FALSE)
summary(mod.IS11.fit, rsq = TRUE)
```

```
## lavaan (0.5-23.1097) converged normally after  38 iterations
##
##   Number of observations                           119
##
##   Estimator                                         ML
##   Minimum Function Test Statistic               57.921
##   Degrees of freedom                                25
##   P-value (Chi-square)                           0.000
##
## Parameter Estimates:
##
##   Information                                 Expected
##   Standard Errors                             Standard
##
## Regressions:
##                      Estimate  Std.Err  z-value  P(>|z|)
##   Attendance2 ~
##     Sp.Richness2       -0.403    0.173   -2.334    0.020
##     Total.Animals2      0.715    0.139    5.163    0.000
##     Men.Sp.BdyMss2      0.315    0.059    5.338    0.000
##     Brilloun.Indx2      0.263    0.091    2.896    0.004
##     Mean.Rap.Crck2      0.162    0.061    2.641    0.008
##     X10km_Pop2          0.398    0.052    7.678    0.000
##   Total.Animals2 ~
##     Zoo.Area.ha2        0.142    0.049    2.903    0.004
##     Sp.Richness2        0.809    0.049   16.493    0.000
##   Sp.Richness2 ~
##     Mam.Sp.Rchnss2      0.877    0.055   15.817    0.000
##     Men.Sp.BdyMss2     -0.168    0.055   -3.028    0.002
##   Mean.Raup.Crick2 ~
##     Total.Animals2      0.427    0.073    5.874    0.000
##     Men.Sp.BdyMss2     -0.537    0.073   -7.354    0.000
##   Brillouin.Index2 ~
##     Sp.Richness2        1.573    0.094   16.667    0.000
##     Total.Animals2     -1.081    0.094  -11.496    0.000
##   insitu2 ~
##     Attendance2         0.563    0.097    5.832    0.000
##     Zoo.Area.ha2        0.146    0.079    1.854    0.064
##     Total.Animals2     -0.210    0.110   -1.913    0.056
##     Mam.Sp.Rchnss2      0.177    0.106    1.668    0.095
##     Prop.Thret.Sp2      0.206    0.068    3.014    0.003
##     Mean.Rap.Crck2      0.190    0.072    2.661    0.008
##
```

```
## Covariances:
##                       Estimate  Std.Err  z-value  P(>|z|)
##   Mean.Sp.BodyMass2 ~~
##     X10km_Pop2          -0.023    0.091   -0.248    0.804
##     Zoo.Area.ha2         0.560    0.104    5.363    0.000
##     Mam.Sp.Rchnss2       0.373    0.097    3.845    0.000
##     Prop.Thret.Sp2       0.277    0.094    2.933    0.003
##   X10km_Pop2 ~~
##     Zoo.Area.ha2        -0.099    0.091   -1.087    0.277
##     Mam.Sp.Rchnss2       0.421    0.099    4.262    0.000
##     Prop.Thret.Sp2       0.287    0.095    3.030    0.002
##   Zoo.Area.ha2 ~~
##     Mam.Sp.Rchnss2       0.536    0.103    5.185    0.000
##     Prop.Thret.Sp2       0.189    0.093    2.045    0.041
##   Mam.Sp.Richness2 ~~
##     Prop.Thret.Sp2       0.322    0.096    3.368    0.001
##
## Variances:
##                   Estimate  Std.Err  z-value  P(>|z|)
##    .Attendance2       0.269    0.035    7.714    0.000
##    .Total.Animals2    0.243    0.032    7.714    0.000
##    .Sp.Richness2      0.311    0.040    7.714    0.000
##    .Mean.Rap.Crck2    0.602    0.078    7.714    0.000
##    .Brilloun.Indx2    0.274    0.036    7.714    0.000
##    .insitu2           0.475    0.062    7.714    0.000
##     Men.Sp.BdyMss2    0.992    0.129    7.714    0.000
##     X10km_Pop2        0.992    0.129    7.714    0.000
##     Zoo.Area.ha2      0.992    0.129    7.714    0.000
##     Mam.Sp.Rchnss2    0.992    0.129    7.714    0.000
##     Prop.Thret.Sp2    0.992    0.129    7.714    0.000
##
## R-Square:
##                   Estimate
##     Attendance2       0.712
##     Total.Animals2    0.757
##     Sp.Richness2      0.686
##     Mean.Rap.Crck2    0.382
##     Brilloun.Indx2    0.722
##     insitu2           0.529
```

```r
# Generate fit indices
fitMeasures(mod.IS11.fit, c("agfi", "rmr", "srmr", "rmsea", "cfi", "nnfi", "tli"))
```

```
##  agfi   rmr  srmr rmsea   cfi  nnfi   tli
## 0.804 0.056 0.056 0.105 0.957 0.923 0.923
```

```r
# Generate modification indices
miIS11 <- modindices(mod.IS11.fit)
print(miIS11[miIS11$mi > 3.0,])
```

```
##                   lhs op            rhs     mi    epc sepc.lv sepc.all
## 74      Sp.Richness2  ~     X10km_Pop2 17.968  0.246   0.246    0.246
## 80  Mean.Raup.Crick2  ~        insitu2  3.644 -0.269  -0.269   -0.274
## 82  Mean.Raup.Crick2  ~   Zoo.Area.ha2  3.986  0.189   0.189    0.191
## 84  Mean.Raup.Crick2  ~ Prop.Threat.Sp2 13.894 -0.281  -0.281   -0.284
## 107       X10km_Pop2  ~    Attendance2  9.223  0.475   0.475    0.461
```

```
## 108       X10km_Pop2  ~   Total.Animals2 13.199  0.396   0.396    0.397
## 109       X10km_Pop2  ~     Sp.Richness2 18.045  0.554   0.554    0.554
## 110       X10km_Pop2  ~ Mean.Raup.Crick2  5.427  0.205   0.205    0.203
## 120     Zoo.Area.ha2  ~ Mean.Raup.Crick2  6.890  0.204   0.204    0.202
## 122     Zoo.Area.ha2  ~          insitu2  4.311  0.249   0.249    0.251
## 128 Mam.Sp.Richness2  ~   Total.Animals2  4.646 -0.279  -0.279   -0.280
## 129 Mam.Sp.Richness2  ~     Sp.Richness2  9.049 -0.500  -0.500   -0.500
## 130 Mam.Sp.Richness2  ~ Mean.Raup.Crick2  3.537 -0.167  -0.167   -0.165
## 140  Prop.Threat.Sp2  ~ Mean.Raup.Crick2 11.924 -0.346  -0.346   -0.343
## 142  Prop.Threat.Sp2  ~          insitu2  7.186 -0.533  -0.533   -0.538
##     sepc.nox
## 74     0.247
## 80    -0.274
## 82     0.192
## 84    -0.285
## 107    0.461
## 108    0.397
## 109    0.554
## 110    0.203
## 120    0.202
## 122    0.251
## 128   -0.280
## 129   -0.500
## 130   -0.165
## 140   -0.343
## 142   -0.538
```

Model 12

Based on the results generated from the eleventh model, the highest p-value relationship to be considered for removal is **insitu2** ~ **Mam.Sp.Rchnss2** with a p-value of **0.095**. Therefore we decide to remove this pathway. Once again, the model summary, fit indices and modification indices were all generated for the model.

```
# In Situ SEM (Presence-Absence)

# Model 12
# Removal of insitu2 ~ Mam.Sp.Rchnss2, p = 0.095

mod.IS12 <- 'Attendance2 ~ Sp.Richness2 + Total.Animals2
+ Mean.Sp.BodyMass2 + Brillouin.Index2
+ Mean.Raup.Crick2 + X10km_Pop2

Total.Animals2 ~ Zoo.Area.ha2 + Sp.Richness2
Sp.Richness2 ~ Mam.Sp.Richness2 + Mean.Sp.BodyMass2
Mean.Raup.Crick2 ~ Total.Animals2 + Mean.Sp.BodyMass2
Brillouin.Index2 ~ Sp.Richness2 + Total.Animals2

insitu2 ~ Attendance2 + Zoo.Area.ha2 + Total.Animals2
+ Prop.Threat.Sp2 + Mean.Raup.Crick2'

# Fit model and generate model summary
mod.IS12.fit <- sem(mod.IS12, data = sem_in_situ_data, fixed.x=FALSE)
```

```r
summary(mod.IS12.fit, rsq = TRUE)
```

```
## lavaan (0.5-23.1097) converged normally after  35 iterations
##
##   Number of observations                           119
##
##   Estimator                                         ML
##   Minimum Function Test Statistic               60.519
##   Degrees of freedom                                26
##   P-value (Chi-square)                           0.000
##
## Parameter Estimates:
##
##   Information                                 Expected
##   Standard Errors                             Standard
##
## Regressions:
##                      Estimate  Std.Err  z-value  P(>|z|)
##   Attendance2 ~
##     Sp.Richness2       -0.403    0.173   -2.334    0.020
##     Total.Animals2      0.715    0.139    5.163    0.000
##     Men.Sp.BdyMss2      0.315    0.059    5.338    0.000
##     Brilloun.Indx2      0.263    0.091    2.896    0.004
##     Mean.Rap.Crck2      0.162    0.061    2.641    0.008
##     X10km_Pop2          0.398    0.052    7.678    0.000
##   Total.Animals2 ~
##     Zoo.Area.ha2        0.142    0.049    2.903    0.004
##     Sp.Richness2        0.809    0.049   16.493    0.000
##   Sp.Richness2 ~
##     Mam.Sp.Rchnss2      0.877    0.055   15.817    0.000
##     Men.Sp.BdyMss2     -0.168    0.055   -3.028    0.002
##   Mean.Raup.Crick2 ~
##     Total.Animals2      0.427    0.073    5.874    0.000
##     Men.Sp.BdyMss2     -0.537    0.073   -7.354    0.000
##   Brillouin.Index2 ~
##     Sp.Richness2        1.573    0.094   16.667    0.000
##     Total.Animals2     -1.081    0.094  -11.496    0.000
##   insitu2 ~
##     Attendance2         0.595    0.094    6.323    0.000
##     Zoo.Area.ha2        0.187    0.075    2.478    0.013
##     Total.Animals2     -0.113    0.099   -1.138    0.255
##     Prop.Thret.Sp2      0.217    0.068    3.191    0.001
##     Mean.Rap.Crck2      0.168    0.072    2.332    0.020
##
## Covariances:
##                        Estimate  Std.Err  z-value  P(>|z|)
##   Mean.Sp.BodyMass2 ~~
##     X10km_Pop2           -0.023    0.091   -0.248    0.804
##     Zoo.Area.ha2          0.560    0.104    5.363    0.000
##     Mam.Sp.Rchnss2        0.373    0.097    3.845    0.000
##     Prop.Thret.Sp2        0.277    0.094    2.933    0.003
##   X10km_Pop2 ~~
##     Zoo.Area.ha2         -0.099    0.091   -1.087    0.277
##     Mam.Sp.Rchnss2        0.421    0.099    4.262    0.000
```

```
##     Prop.Thret.Sp2        0.287    0.095    3.030    0.002
##   Zoo.Area.ha2 ~~
##     Mam.Sp.Rchnss2        0.536    0.103    5.185    0.000
##     Prop.Thret.Sp2        0.189    0.093    2.045    0.041
##   Mam.Sp.Richness2 ~~
##     Prop.Thret.Sp2        0.322    0.096    3.368    0.001
##
## Variances:
##                       Estimate  Std.Err  z-value  P(>|z|)
##    .Attendance2          0.269    0.035    7.714    0.000
##    .Total.Animals2       0.243    0.032    7.714    0.000
##    .Sp.Richness2         0.311    0.040    7.714    0.000
##    .Mean.Rap.Crck2       0.602    0.078    7.714    0.000
##    .Brilloun.Indx2       0.274    0.036    7.714    0.000
##    .insitu2              0.486    0.063    7.714    0.000
##     Men.Sp.BdyMss2       0.992    0.129    7.714    0.000
##     X10km_Pop2           0.992    0.129    7.714    0.000
##     Zoo.Area.ha2         0.992    0.129    7.714    0.000
##     Mam.Sp.Rchnss2       0.992    0.129    7.714    0.000
##     Prop.Thret.Sp2       0.992    0.129    7.714    0.000
##
## R-Square:
##                       Estimate
##     Attendance2          0.712
##     Total.Animals2       0.757
##     Sp.Richness2         0.686
##     Mean.Rap.Crck2       0.382
##     Brilloun.Indx2       0.722
##     insitu2              0.513
```

```
# Generate fit indices
fitMeasures(mod.IS12.fit, c("agfi", "rmr", "srmr", "rmsea", "cfi", "nnfi", "tli"))
```

```
##  agfi   rmr  srmr rmsea   cfi  nnfi   tli
## 0.803 0.057 0.058 0.106 0.955 0.923 0.923
```

```
# Generate modification indices
miIS12 <- modindices(mod.IS12.fit)
print(miIS12[miIS12$mi > 3.0,])
```

```
##                  lhs op              rhs     mi    epc sepc.lv sepc.all
## 73      Sp.Richness2  ~       X10km_Pop2 17.968  0.246   0.246    0.246
## 81   Mean.Raup.Crick2  ~     Zoo.Area.ha2  3.986  0.189   0.189    0.191
## 83   Mean.Raup.Crick2  ~   Prop.Threat.Sp2 13.894 -0.281  -0.281   -0.284
## 107        X10km_Pop2  ~      Attendance2  9.223  0.475   0.475    0.461
## 108        X10km_Pop2  ~    Total.Animals2 13.199  0.396   0.396    0.397
## 109        X10km_Pop2  ~     Sp.Richness2 18.045  0.554   0.554    0.554
## 110        X10km_Pop2  ~ Mean.Raup.Crick2  5.427  0.205   0.205    0.203
## 120      Zoo.Area.ha2  ~ Mean.Raup.Crick2  6.889  0.204   0.204    0.202
## 128 Mam.Sp.Richness2  ~    Total.Animals2  4.646 -0.279  -0.279   -0.280
## 129 Mam.Sp.Richness2  ~     Sp.Richness2  9.049 -0.500  -0.500   -0.500
## 130 Mam.Sp.Richness2  ~ Mean.Raup.Crick2  3.537 -0.167  -0.167   -0.165
## 140  Prop.Threat.Sp2  ~ Mean.Raup.Crick2 11.924 -0.346  -0.346   -0.343
## 142  Prop.Threat.Sp2  ~          insitu2  7.160 -0.483  -0.483   -0.484
##     sepc.nox
## 73     0.247
```

```
## 81     0.192
## 83    -0.285
## 107    0.461
## 108    0.397
## 109    0.554
## 110    0.203
## 120    0.202
## 128   -0.280
## 129   -0.500
## 130   -0.165
## 140   -0.343
## 142   -0.484
```

Model 13

Based on the results generated from the twelfth model, the highest p-value relationship to be considered for removal is **insitu2** ~ **Total.Animals2** with a p-value of **0.255**. Therefore we decide to remove this pathway. Once again, the model summary, fit indices and modification indices were all generated for the model.

```
# In Situ SEM (Presence-Absence)

# Model 13
# Removal of insitu2 ~ Total.Animals2, p = 0.255

mod.IS13 <- 'Attendance2 ~ Sp.Richness2 + Total.Animals2
+ Mean.Sp.BodyMass2 + Brillouin.Index2
+ Mean.Raup.Crick2 + X10km_Pop2

Total.Animals2 ~ Zoo.Area.ha2 + Sp.Richness2
Sp.Richness2 ~ Mam.Sp.Richness2 + Mean.Sp.BodyMass2
Mean.Raup.Crick2 ~ Total.Animals2 + Mean.Sp.BodyMass2
Brillouin.Index2 ~ Sp.Richness2 + Total.Animals2

insitu2 ~ Attendance2 + Zoo.Area.ha2
+ Prop.Threat.Sp2 + Mean.Raup.Crick2'

# Fit model and generate model summary
mod.IS13.fit <- sem(mod.IS13, data = sem_in_situ_data, fixed.x=FALSE)
summary(mod.IS13.fit, rsq = TRUE)
```

```
## lavaan (0.5-23.1097) converged normally after  32 iterations
##
##   Number of observations                           119
##
##   Estimator                                         ML
##   Minimum Function Test Statistic               61.627
##   Degrees of freedom                                27
##   P-value (Chi-square)                           0.000
##
## Parameter Estimates:
##
##   Information                                 Expected
##   Standard Errors                             Standard
##
```

```
## Regressions:
##                    Estimate  Std.Err  z-value  P(>|z|)
##   Attendance2 ~
##     Sp.Richness2      -0.403    0.173   -2.334    0.020
##     Total.Animals2     0.715    0.139    5.163    0.000
##     Men.Sp.BdyMss2     0.315    0.059    5.338    0.000
##     Brilloun.Indx2     0.263    0.091    2.896    0.004
##     Mean.Rap.Crck2     0.162    0.061    2.641    0.008
##     X10km_Pop2         0.398    0.052    7.678    0.000
##   Total.Animals2 ~
##     Zoo.Area.ha2       0.142    0.049    2.903    0.004
##     Sp.Richness2       0.809    0.049   16.493    0.000
##   Sp.Richness2 ~
##     Mam.Sp.Rchnss2     0.877    0.055   15.817    0.000
##     Men.Sp.BdyMss2    -0.168    0.055   -3.028    0.002
##   Mean.Raup.Crick2 ~
##     Total.Animals2     0.427    0.073    5.874    0.000
##     Men.Sp.BdyMss2    -0.537    0.073   -7.354    0.000
##   Brillouin.Index2 ~
##     Sp.Richness2       1.573    0.094   16.667    0.000
##     Total.Animals2    -1.081    0.094  -11.496    0.000
##   insitu2 ~
##     Attendance2        0.529    0.076    6.953    0.000
##     Zoo.Area.ha2       0.166    0.070    2.374    0.018
##     Prop.Thret.Sp2     0.199    0.068    2.907    0.004
##     Mean.Rap.Crck2     0.140    0.068    2.046    0.041
##
## Covariances:
##                        Estimate  Std.Err  z-value  P(>|z|)
##   Mean.Sp.BodyMass2 ~~
##     X10km_Pop2           -0.023    0.091   -0.248    0.804
##     Zoo.Area.ha2          0.560    0.104    5.363    0.000
##     Mam.Sp.Rchnss2        0.373    0.097    3.845    0.000
##     Prop.Thret.Sp2        0.277    0.094    2.933    0.003
##   X10km_Pop2 ~~
##     Zoo.Area.ha2         -0.099    0.091   -1.087    0.277
##     Mam.Sp.Rchnss2        0.421    0.099    4.262    0.000
##     Prop.Thret.Sp2        0.287    0.095    3.030    0.002
##   Zoo.Area.ha2 ~~
##     Mam.Sp.Rchnss2        0.536    0.103    5.185    0.000
##     Prop.Thret.Sp2        0.189    0.093    2.045    0.041
##   Mam.Sp.Richness2 ~~
##     Prop.Thret.Sp2        0.322    0.096    3.368    0.001
##
## Variances:
##                  Estimate  Std.Err  z-value  P(>|z|)
##    .Attendance2     0.269    0.035    7.714    0.000
##    .Total.Animals2  0.243    0.032    7.714    0.000
##    .Sp.Richness2    0.311    0.040    7.714    0.000
##    .Mean.Rap.Crck2  0.602    0.078    7.714    0.000
##    .Brilloun.Indx2  0.274    0.036    7.714    0.000
##    .insitu2         0.490    0.064    7.714    0.000
##     Men.Sp.BdyMss2  0.992    0.129    7.714    0.000
##     X10km_Pop2      0.992    0.129    7.714    0.000
```

```
##     Zoo.Area.ha2      0.992    0.129    7.714    0.000
##     Mam.Sp.Rchnss2    0.992    0.129    7.714    0.000
##     Prop.Thret.Sp2    0.992    0.129    7.714    0.000
##
## R-Square:
##                    Estimate
##     Attendance2       0.712
##     Total.Animals2    0.757
##     Sp.Richness2      0.686
##     Mean.Rap.Crck2    0.382
##     Brilloun.Indx2    0.722
##     insitu2           0.504
```

```r
# Generate fit indices
fitMeasures(mod.IS13.fit, c("agfi", "rmr", "srmr", "rmsea", "cfi", "nnfi", "tli"))
```

```
##  agfi   rmr  srmr rmsea   cfi  nnfi   tli
## 0.809 0.056 0.056 0.104 0.955 0.925 0.925
```

```r
# Generate modification indices
miIS13 <- modindices(mod.IS13.fit)
print(miIS13[miIS13$mi > 3.0,])
```

```
##                  lhs op              rhs     mi    epc sepc.lv sepc.all
## 72      Sp.Richness2  ~       X10km_Pop2 17.968  0.246   0.246    0.246
## 80  Mean.Raup.Crick2  ~     Zoo.Area.ha2  3.986  0.189   0.189    0.191
## 82  Mean.Raup.Crick2  ~   Prop.Threat.Sp2 13.894 -0.281  -0.281   -0.284
## 107       X10km_Pop2  ~      Attendance2  9.223  0.475   0.475    0.461
## 108       X10km_Pop2  ~   Total.Animals2 13.199  0.396   0.396    0.397
## 109       X10km_Pop2  ~     Sp.Richness2 18.045  0.554   0.554    0.554
## 110       X10km_Pop2  ~ Mean.Raup.Crick2  5.427  0.205   0.205    0.203
## 120     Zoo.Area.ha2  ~ Mean.Raup.Crick2  6.890  0.204   0.204    0.202
## 128 Mam.Sp.Richness2  ~   Total.Animals2  4.646 -0.279  -0.279   -0.280
## 129 Mam.Sp.Richness2  ~     Sp.Richness2  9.050 -0.500  -0.500   -0.500
## 130 Mam.Sp.Richness2  ~ Mean.Raup.Crick2  3.537 -0.167  -0.167   -0.165
## 140  Prop.Threat.Sp2  ~ Mean.Raup.Crick2 11.924 -0.346  -0.346   -0.343
## 142  Prop.Threat.Sp2  ~          insitu2  5.585 -0.442  -0.442   -0.441
##     sepc.nox
## 72     0.247
## 80     0.192
## 82    -0.285
## 107    0.461
## 108    0.397
## 109    0.554
## 110    0.203
## 120    0.202
## 128   -0.280
## 129   -0.500
## 130   -0.165
## 140   -0.343
## 142   -0.441
```

Model Comparisons 7

At this stage we compare the models generated using AICc values. Overall model selection from the pool of competing models is achieved using AICc values, with a threshold of more than 2 AICc units lower than nearest competing model being considered sufficient for model selection.

```r
# Model Comparisons using AICc

# Comparing models with and without adjustment for nested nature of data
# library(AICcmodavg)
# source("lavaan.modavg.R")

aictab.lavaan(list(mod.IS1.fit, mod.IS2.fit, mod.IS3.fit, mod.IS4.fit, mod.IS5.fit, mod.IS6.fit,
                   mod.IS7.fit, mod.IS8.fit, mod.IS9.fit, mod.IS10.fit, mod.IS11.fit, mod.IS12.fit, mod
              c("mod.1", "mod.2", "mod.3", "mod.4", "mod.5", "mod.6", "mod.7", "mod.8",
                "mod.9", "mod.10", "mod.11", "mod.12", "mod.13"))
```

```
##
## Model selection based on AICc:
##
##          K    AICc Delta_AICc AICcWt Cum.Wt       LL
## mod.13 39 2872.16       0.00   0.48   0.48 -1393.66
## mod.12 40 2873.88       1.73   0.20   0.68 -1393.10
## mod.11 41 2874.18       2.03   0.17   0.86 -1391.80
## mod.10 42 2875.25       3.09   0.10   0.96 -1390.86
## mod.9  43 2877.97       5.81   0.03   0.99 -1390.71
## mod.8  44 2879.99       7.84   0.01   1.00 -1390.19
## mod.7  45 2882.01       9.85   0.00   1.00 -1389.62
## mod.6  46 2884.42      12.27   0.00   1.00 -1389.22
## mod.5  47 2887.57      15.41   0.00   1.00 -1389.15
## mod.4  48 2890.86      18.71   0.00   1.00 -1389.12
## mod.3  49 2894.23      22.07   0.00   1.00 -1389.09
## mod.2  57 3133.59     261.43   0.00   1.00 -1499.23
## mod.1  66 3419.69     547.53   0.00   1.00 -1631.57
```

Based on these results we can see that model 13 is the superior model (lowest AICc value).

Model 14

Due to the fact that **Brillouin.Index2** was deemed unnecessary in the Attendance Model and has proven unnecessary for the In Situ portion of this model, we choose to remove this variable entirely once again. The model summary, fit indices and modification indices were all generated for the model.

```r
# In Situ SEM (Presence-Absence)

# Model 14
# Removal of Brillouin.Index2

mod.IS14 <- 'Attendance2 ~ Sp.Richness2 + Total.Animals2
+ Mean.Sp.BodyMass2 + Mean.Raup.Crick2 + X10km_Pop2

Total.Animals2 ~ Zoo.Area.ha2 + Sp.Richness2
Sp.Richness2 ~ Mam.Sp.Richness2 + Mean.Sp.BodyMass2
Mean.Raup.Crick2 ~ Total.Animals2 + Mean.Sp.BodyMass2
```

```r
insitu2 ~ Attendance2 + Zoo.Area.ha2
+ Prop.Threat.Sp2 + Mean.Raup.Crick2'

# Fit model and generate model summary
mod.IS14.fit <- sem(mod.IS14, data = sem_in_situ_data, fixed.x=FALSE)
summary(mod.IS14.fit, rsq = TRUE)
```

```
## lavaan (0.5-23.1097) converged normally after  30 iterations
##
##   Number of observations                           119
##
##   Estimator                                         ML
##   Minimum Function Test Statistic               53.695
##   Degrees of freedom                                20
##   P-value (Chi-square)                           0.000
##
## Parameter Estimates:
##
##   Information                                 Expected
##   Standard Errors                             Standard
##
## Regressions:
##                    Estimate  Std.Err  z-value  P(>|z|)
##   Attendance2 ~
##     Sp.Richness2      0.019    0.100    0.185    0.853
##     Total.Animals2    0.431    0.101    4.262    0.000
##     Men.Sp.BdyMss2    0.309    0.061    5.062    0.000
##     Mean.Rap.Crck2    0.165    0.063    2.608    0.009
##     X10km_Pop2        0.384    0.054    7.171    0.000
##   Total.Animals2 ~
##     Zoo.Area.ha2      0.142    0.049    2.903    0.004
##     Sp.Richness2      0.809    0.049   16.493    0.000
##   Sp.Richness2 ~
##     Mam.Sp.Rchnss2    0.877    0.055   15.817    0.000
##     Men.Sp.BdyMss2   -0.168    0.055   -3.028    0.002
##   Mean.Raup.Crick2 ~
##     Total.Animals2    0.427    0.073    5.874    0.000
##     Men.Sp.BdyMss2   -0.537    0.073   -7.354    0.000
##   insitu2 ~
##     Attendance2       0.529    0.077    6.909    0.000
##     Zoo.Area.ha2      0.166    0.070    2.371    0.018
##     Prop.Thret.Sp2    0.199    0.068    2.909    0.004
##     Mean.Rap.Crck2    0.140    0.069    2.040    0.041
##
## Covariances:
##                      Estimate  Std.Err  z-value  P(>|z|)
##   Mean.Sp.BodyMass2 ~~
##     X10km_Pop2         -0.023    0.091   -0.248    0.804
##     Zoo.Area.ha2        0.560    0.104    5.363    0.000
##     Mam.Sp.Rchnss2      0.373    0.097    3.845    0.000
##     Prop.Thret.Sp2      0.277    0.094    2.933    0.003
##   X10km_Pop2 ~~
##     Zoo.Area.ha2       -0.099    0.091   -1.087    0.277
##     Mam.Sp.Rchnss2      0.421    0.099    4.262    0.000
```

```
##     Prop.Thret.Sp2        0.287    0.095    3.030    0.002
##   Zoo.Area.ha2 ~~
##     Mam.Sp.Rchnss2        0.536    0.103    5.185    0.000
##     Prop.Thret.Sp2        0.189    0.093    2.045    0.041
##   Mam.Sp.Richness2 ~~
##     Prop.Thret.Sp2        0.322    0.096    3.368    0.001
##
## Variances:
##                    Estimate  Std.Err  z-value  P(>|z|)
##    .Attendance2       0.288    0.037    7.714    0.000
##    .Total.Animals2    0.243    0.032    7.714    0.000
##    .Sp.Richness2      0.311    0.040    7.714    0.000
##    .Mean.Rap.Crck2    0.602    0.078    7.714    0.000
##    .insitu2           0.490    0.064    7.714    0.000
##     Men.Sp.BdyMss2    0.992    0.129    7.714    0.000
##     X10km_Pop2        0.992    0.129    7.714    0.000
##     Zoo.Area.ha2      0.992    0.129    7.714    0.000
##     Mam.Sp.Rchnss2    0.992    0.129    7.714    0.000
##     Prop.Thret.Sp2    0.992    0.129    7.714    0.000
##
## R-Square:
##                    Estimate
##     Attendance2       0.690
##     Total.Animals2    0.757
##     Sp.Richness2      0.686
##     Mean.Rap.Crck2    0.382
##     insitu2           0.503
```

```
# Generate fit indices
fitMeasures(mod.IS14.fit, c("agfi", "rmr", "srmr", "rmsea", "cfi", "nnfi", "tli"))
```

```
##  agfi   rmr  srmr rmsea   cfi  nnfi   tli
## 0.795 0.060 0.061 0.119 0.945 0.904 0.904
```

```
# Generate modification indices
miIS14 <- modindices(mod.IS14.fit)
print(miIS14[miIS14$mi > 3.0,])
```

```
##                  lhs op              rhs     mi    epc sepc.lv sepc.all
## 61      Sp.Richness2  ~       X10km_Pop2 17.968  0.246   0.246    0.246
## 68  Mean.Raup.Crick2  ~     Zoo.Area.ha2  3.986  0.189   0.189    0.191
## 70  Mean.Raup.Crick2  ~  Prop.Threat.Sp2 13.894 -0.281  -0.281   -0.284
## 85        X10km_Pop2  ~      Attendance2 11.383  0.541   0.541    0.523
## 86        X10km_Pop2  ~   Total.Animals2 13.199  0.396   0.396    0.397
## 87        X10km_Pop2  ~     Sp.Richness2 18.045  0.554   0.554    0.554
## 88        X10km_Pop2  ~ Mean.Raup.Crick2  5.427  0.205   0.205    0.203
## 97      Zoo.Area.ha2  ~ Mean.Raup.Crick2  6.889  0.204   0.204    0.202
## 104 Mam.Sp.Richness2  ~   Total.Animals2  4.646 -0.279  -0.279   -0.280
## 105 Mam.Sp.Richness2  ~     Sp.Richness2  9.050 -0.500  -0.500   -0.500
## 106 Mam.Sp.Richness2  ~ Mean.Raup.Crick2  3.537 -0.167  -0.167   -0.165
## 115  Prop.Threat.Sp2  ~ Mean.Raup.Crick2 11.924 -0.346  -0.346   -0.343
## 116  Prop.Threat.Sp2  ~          insitu2  5.528 -0.437  -0.437   -0.436
##     sepc.nox
## 61     0.247
## 68     0.192
## 70    -0.285
```

```
## 85     0.523
## 86     0.397
## 87     0.554
## 88     0.203
## 97     0.202
## 104   -0.280
## 105   -0.500
## 106   -0.165
## 115   -0.343
## 116   -0.436
```

Model Comparisons 8

Once again we compare the models generated using AICc values in order to ensure the removal of the **Brillouin.Index2** was statistically justified. Overall model selection from the pool of competing models is achieved using AICc values, with a threshold of more than 2 AICc units lower than nearest competing model being considered sufficient for model selection.

```
# Model Comparisons using AICc

# Comparing models with and without adjustment for nested nature of data
# library(AICcmodavg)
# source("lavaan.modavg.R")

aictab.lavaan(list(mod.IS1.fit, mod.IS2.fit, mod.IS3.fit, mod.IS4.fit, mod.IS5.fit, mod.IS6.fit,
                   mod.IS7.fit, mod.IS8.fit, mod.IS9.fit, mod.IS10.fit, mod.IS11.fit, mod.IS12.fit, mod
              c("mod.1", "mod.2", "mod.3", "mod.4", "mod.5", "mod.6", "mod.7", "mod.8",
                "mod.9", "mod.10", "mod.11", "mod.12", "mod.13", "mod.14"))
```

```
##
## Model selection based on AICc:
##
##         K    AICc Delta_AICc AICcWt Cum.Wt       LL
## mod.14 35 2686.40       0.00      1      1 -1305.87
## mod.13 39 2872.16     185.75      0      1 -1393.66
## mod.12 40 2873.88     187.48      0      1 -1393.10
## mod.11 41 2874.18     187.78      0      1 -1391.80
## mod.10 42 2875.25     188.84      0      1 -1390.86
## mod.9  43 2877.97     191.56      0      1 -1390.71
## mod.8  44 2879.99     193.59      0      1 -1390.19
## mod.7  45 2882.01     195.60      0      1 -1389.62
## mod.6  46 2884.42     198.02      0      1 -1389.22
## mod.5  47 2887.57     201.16      0      1 -1389.15
## mod.4  48 2890.86     204.46      0      1 -1389.12
## mod.3  49 2894.23     207.82      0      1 -1389.09
## mod.2  57 3133.59     447.19      0      1 -1499.23
## mod.1  66 3419.69     733.28      0      1 -1631.57
```

Based on these results we can see that model 14 is the superior model (lowest AICc value) and that **Brillouin.Index2** was not supported.

Model 15

Based on the results generated from the fourteenth model, the highest p-value relationship to be considered for removal is **Attendance2 ~ Sp.Richness2** with a p-value of **0.853**. Therefore we decide to remove this pathway. Once again, the model summary, fit indices and modification indices were all generated for the model.

```
# In Situ SEM (Presence-Absence)

# Model 15
# Removal of Attendance2 ~ Sp.Richness2, p = 0.853

mod.IS15 <- 'Attendance2 ~ Total.Animals2
+ Mean.Sp.BodyMass2 + Mean.Raup.Crick2 + X10km_Pop2

Total.Animals2 ~ Zoo.Area.ha2 + Sp.Richness2
Sp.Richness2 ~ Mam.Sp.Richness2 + Mean.Sp.BodyMass2
Mean.Raup.Crick2 ~ Total.Animals2 + Mean.Sp.BodyMass2

insitu2 ~ Attendance2 + Zoo.Area.ha2
+ Prop.Threat.Sp2 + Mean.Raup.Crick2'

# Fit model and generate model summary
mod.IS15.fit <- sem(mod.IS15, data = sem_in_situ_data, fixed.x=FALSE)
summary(mod.IS15.fit, rsq = TRUE)
```

```
## lavaan (0.5-23.1097) converged normally after  26 iterations
##
##   Number of observations                           119
##
##   Estimator                                         ML
##   Minimum Function Test Statistic               53.728
##   Degrees of freedom                                21
##   P-value (Chi-square)                           0.000
##
## Parameter Estimates:
##
##   Information                                 Expected
##   Standard Errors                             Standard
##
## Regressions:
##                    Estimate  Std.Err  z-value  P(>|z|)
##   Attendance2 ~
##     Total.Animals2    0.445    0.059    7.507    0.000
##     Men.Sp.BdyMss2    0.310    0.061    5.073    0.000
##     Mean.Rap.Crck2    0.165    0.063    2.609    0.009
##     X10km_Pop2        0.388    0.052    7.492    0.000
##   Total.Animals2 ~
##     Zoo.Area.ha2      0.142    0.049    2.903    0.004
##     Sp.Richness2      0.809    0.049   16.493    0.000
##   Sp.Richness2 ~
##     Mam.Sp.Rchnss2    0.877    0.055   15.817    0.000
##     Men.Sp.BdyMss2   -0.168    0.055   -3.028    0.002
##   Mean.Raup.Crick2 ~
##     Total.Animals2    0.427    0.073    5.874    0.000
```

```
##     Men.Sp.BdyMss2     -0.537    0.073   -7.354    0.000
##   insitu2 ~
##     Attendance2         0.529    0.076    6.913    0.000
##     Zoo.Area.ha2        0.166    0.070    2.372    0.018
##     Prop.Thret.Sp2      0.199    0.068    2.909    0.004
##     Mean.Rap.Crck2      0.140    0.069    2.041    0.041
##
## Covariances:
##                        Estimate  Std.Err  z-value  P(>|z|)
##   Mean.Sp.BodyMass2 ~~
##     X10km_Pop2           -0.023    0.091   -0.248    0.804
##     Zoo.Area.ha2          0.560    0.104    5.363    0.000
##     Mam.Sp.Rchnss2        0.373    0.097    3.845    0.000
##     Prop.Thret.Sp2        0.277    0.094    2.933    0.003
##   X10km_Pop2 ~~
##     Zoo.Area.ha2         -0.099    0.091   -1.087    0.277
##     Mam.Sp.Rchnss2        0.421    0.099    4.262    0.000
##     Prop.Thret.Sp2        0.287    0.095    3.030    0.002
##   Zoo.Area.ha2 ~~
##     Mam.Sp.Rchnss2        0.536    0.103    5.185    0.000
##     Prop.Thret.Sp2        0.189    0.093    2.045    0.041
##   Mam.Sp.Richness2 ~~
##     Prop.Thret.Sp2        0.322    0.096    3.368    0.001
##
## Variances:
##                    Estimate  Std.Err  z-value  P(>|z|)
##    .Attendance2       0.288    0.037    7.714    0.000
##    .Total.Animals2    0.243    0.032    7.714    0.000
##    .Sp.Richness2      0.311    0.040    7.714    0.000
##    .Mean.Rap.Crck2    0.602    0.078    7.714    0.000
##    .insitu2           0.490    0.064    7.714    0.000
##     Men.Sp.BdyMss2    0.992    0.129    7.714    0.000
##     X10km_Pop2        0.992    0.129    7.714    0.000
##     Zoo.Area.ha2      0.992    0.129    7.714    0.000
##     Mam.Sp.Rchnss2    0.992    0.129    7.714    0.000
##     Prop.Thret.Sp2    0.992    0.129    7.714    0.000
##
## R-Square:
##                    Estimate
##     Attendance2       0.690
##     Total.Animals2    0.757
##     Sp.Richness2      0.686
##     Mean.Rap.Crck2    0.382
##     insitu2           0.503
```

```
# Generate fit indices
fitMeasures(mod.IS15.fit, c("agfi", "rmr", "srmr", "rmsea", "cfi", "nnfi", "tli"))
```

```
##  agfi   rmr  srmr rmsea   cfi  nnfi   tli
## 0.805 0.061 0.061 0.114 0.947 0.911 0.911
```

```
# Generate modification indices
miIS15 <- modindices(mod.IS15.fit)
print(miIS15[miIS15$mi > 3.0,])
```

```
##                  lhs op             rhs     mi    epc sepc.lv sepc.all
```

```
## 61      Sp.Richness2  ~       X10km_Pop2 17.968  0.246   0.246    0.246
## 68  Mean.Raup.Crick2  ~      Zoo.Area.ha2  3.986  0.189   0.189    0.191
## 70  Mean.Raup.Crick2  ~  Prop.Threat.Sp2 13.894 -0.281  -0.281   -0.284
## 85        X10km_Pop2  ~      Attendance2 10.139  0.492   0.492    0.476
## 86        X10km_Pop2  ~    Total.Animals2 13.199  0.396   0.396    0.397
## 87        X10km_Pop2  ~     Sp.Richness2 18.045  0.554   0.554    0.554
## 88        X10km_Pop2  ~ Mean.Raup.Crick2  5.427  0.205   0.205    0.203
## 97      Zoo.Area.ha2  ~ Mean.Raup.Crick2  6.889  0.204   0.204    0.202
## 104 Mam.Sp.Richness2  ~    Total.Animals2  4.646 -0.279  -0.279   -0.280
## 105 Mam.Sp.Richness2  ~     Sp.Richness2  9.049 -0.500  -0.500   -0.500
## 106 Mam.Sp.Richness2  ~ Mean.Raup.Crick2  3.537 -0.167  -0.167   -0.165
## 115  Prop.Threat.Sp2  ~ Mean.Raup.Crick2 11.924 -0.346  -0.346   -0.343
## 116  Prop.Threat.Sp2  ~          insitu2  5.511 -0.436  -0.436   -0.435
##     sepc.nox
## 61     0.247
## 68     0.192
## 70    -0.285
## 85     0.476
## 86     0.397
## 87     0.554
## 88     0.203
## 97     0.202
## 104   -0.280
## 105   -0.500
## 106   -0.165
## 115   -0.343
## 116   -0.435
```

Model 16

Based on the modification indices generated from the fifteenth model, we can see that **Mean.Raup.Crick2 ~ Prop.Threat.Sp2** has one of the highest mi value of **13.894**. This far exceeds the standard cut-off level for the chi-square test criterion of 3.84 (Burnham and Anderson, 2002). This is also an intuitive relationships, as threatened species by their very nature are not common, therefore an institution with a greater proportion of threatened species will be statistically more likely to be dissimilar to other collections. As a result, we add this relationship to our model. Once again, the model summary, fit indices and modification indices were all generated for the model.

```
# In Situ SEM (Presence-Absence)

# Model 16
# Addition of Mean.Raup.Crick2 ~ Prop.Threat.Sp2, mi = 13.894

mod.IS16 <- 'Attendance2 ~ Total.Animals2
+ Mean.Sp.BodyMass2 + Mean.Raup.Crick2 + X10km_Pop2

Total.Animals2 ~ Zoo.Area.ha2 + Sp.Richness2
Sp.Richness2 ~ Mam.Sp.Richness2 + Mean.Sp.BodyMass2
Mean.Raup.Crick2 ~ Total.Animals2 + Mean.Sp.BodyMass2 + Prop.Threat.Sp2

insitu2 ~ Attendance2 + Zoo.Area.ha2
+ Prop.Threat.Sp2 + Mean.Raup.Crick2'
```

```r
# Fit model and generate model summary
mod.IS16.fit <- sem(mod.IS16, data = sem_in_situ_data, fixed.x=FALSE)
summary(mod.IS16.fit, rsq = TRUE)
```

```
## lavaan (0.5-23.1097) converged normally after  25 iterations
##
##   Number of observations                           119
##
##   Estimator                                         ML
##   Minimum Function Test Statistic               38.629
##   Degrees of freedom                                20
##   P-value (Chi-square)                           0.007
##
## Parameter Estimates:
##
##   Information                                 Expected
##   Standard Errors                             Standard
##
## Regressions:
##                    Estimate  Std.Err  z-value  P(>|z|)
##   Attendance2 ~
##     Total.Animals2    0.445    0.060    7.361    0.000
##     Men.Sp.BdyMss2    0.310    0.061    5.047    0.000
##     Mean.Rap.Crck2    0.165    0.064    2.600    0.009
##     X10km_Pop2        0.388    0.052    7.458    0.000
##   Total.Animals2 ~
##     Zoo.Area.ha2      0.142    0.049    2.903    0.004
##     Sp.Richness2      0.809    0.049   16.493    0.000
##   Sp.Richness2 ~
##     Mam.Sp.Rchnss2    0.877    0.055   15.817    0.000
##     Men.Sp.BdyMss2   -0.168    0.055   -3.028    0.002
##   Mean.Raup.Crick2 ~
##     Total.Animals2    0.489    0.069    7.057    0.000
##     Men.Sp.BdyMss2   -0.468    0.071   -6.614    0.000
##     Prop.Thret.Sp2   -0.287    0.071   -4.053    0.000
##   insitu2 ~
##     Attendance2       0.529    0.077    6.857    0.000
##     Zoo.Area.ha2      0.166    0.070    2.383    0.017
##     Prop.Thret.Sp2    0.199    0.072    2.748    0.006
##     Mean.Rap.Crck2    0.140    0.072    1.933    0.053
##
## Covariances:
##                      Estimate  Std.Err  z-value  P(>|z|)
##   Mean.Sp.BodyMass2 ~~
##     X10km_Pop2         -0.023    0.091   -0.248    0.804
##     Zoo.Area.ha2        0.560    0.104    5.363    0.000
##     Mam.Sp.Rchnss2      0.373    0.097    3.845    0.000
##     Prop.Thret.Sp2      0.277    0.094    2.933    0.003
##   X10km_Pop2 ~~
##     Zoo.Area.ha2       -0.099    0.091   -1.087    0.277
##     Mam.Sp.Rchnss2      0.421    0.099    4.262    0.000
##     Prop.Thret.Sp2      0.287    0.095    3.030    0.002
##   Zoo.Area.ha2 ~~
##     Mam.Sp.Rchnss2      0.536    0.103    5.185    0.000
```

```
##     Prop.Thret.Sp2        0.189    0.093    2.045    0.041
##   Mam.Sp.Richness2 ~~
##     Prop.Thret.Sp2        0.322    0.096    3.368    0.001
##
## Variances:
##                    Estimate  Std.Err  z-value  P(>|z|)
##    .Attendance2       0.288    0.037    7.714    0.000
##    .Total.Animals2    0.243    0.032    7.714    0.000
##    .Sp.Richness2      0.311    0.040    7.714    0.000
##    .Mean.Rap.Crck2    0.530    0.069    7.714    0.000
##    .insitu2           0.490    0.064    7.714    0.000
##     Men.Sp.BdyMss2    0.992    0.129    7.714    0.000
##     X10km_Pop2        0.992    0.129    7.714    0.000
##     Zoo.Area.ha2      0.992    0.129    7.714    0.000
##     Mam.Sp.Rchnss2    0.992    0.129    7.714    0.000
##     Prop.Thret.Sp2    0.992    0.129    7.714    0.000
##
## R-Square:
##                    Estimate
##     Attendance2       0.688
##     Total.Animals2    0.757
##     Sp.Richness2      0.686
##     Mean.Rap.Crck2    0.462
##     insitu2           0.490
```

```
# Generate fit indices
fitMeasures(mod.IS16.fit, c("agfi", "rmr", "srmr", "rmsea", "cfi", "nnfi", "tli"))
```

```
##  agfi   rmr  srmr rmsea   cfi  nnfi   tli
## 0.849 0.053 0.053 0.088 0.970 0.947 0.947
```

```
# Generate modification indices
miIS16 <- modindices(mod.IS16.fit)
print(miIS16[miIS16$mi > 3.0,])
```

```
##                  lhs op              rhs     mi    epc sepc.lv sepc.all
## 62      Sp.Richness2  ~       X10km_Pop2 17.968  0.246   0.246    0.246
## 69  Mean.Raup.Crick2  ~     Zoo.Area.ha2  3.688  0.171   0.171    0.171
## 85        X10km_Pop2  ~      Attendance2 10.096  0.490   0.490    0.472
## 86        X10km_Pop2  ~   Total.Animals2 13.199  0.396   0.396    0.397
## 87        X10km_Pop2  ~     Sp.Richness2 18.045  0.554   0.554    0.554
## 88        X10km_Pop2  ~ Mean.Raup.Crick2  5.823  0.220   0.220    0.219
## 97      Zoo.Area.ha2  ~ Mean.Raup.Crick2  7.046  0.216   0.216    0.215
## 104 Mam.Sp.Richness2  ~   Total.Animals2  4.646 -0.279  -0.279   -0.280
## 105 Mam.Sp.Richness2  ~     Sp.Richness2  9.049 -0.500  -0.500   -0.500
## 106 Mam.Sp.Richness2  ~ Mean.Raup.Crick2  6.029 -0.228  -0.228   -0.227
##     sepc.nox
## 62     0.247
## 69     0.172
## 85     0.472
## 86     0.397
## 87     0.554
## 88     0.219
## 97     0.215
## 104   -0.280
## 105   -0.500
```

```
## 106   -0.227
```

Model 17

Based on the results generated from the fourteenth model, the highest p-value relationship to be considered for removal is **insitu2** ~ **Mean.Rap.Crck2** with a p-value of **0.053**. Therefore we decide to remove this pathway. Once again, the model summary, fit indices and modification indices were all generated for the model.

```
# In Situ SEM (Presence-Absence)

# Model 17
# Removal of insitu2 ~ Mean.Rap.Crck2, p = 0.053

mod.IS17 <- 'Attendance2 ~ Total.Animals2
+ Mean.Sp.BodyMass2 + Mean.Raup.Crick2 + X10km_Pop2

Total.Animals2 ~ Zoo.Area.ha2 + Sp.Richness2
Sp.Richness2 ~ Mam.Sp.Richness2 + Mean.Sp.BodyMass2
Mean.Raup.Crick2 ~ Total.Animals2 + Mean.Sp.BodyMass2 + Prop.Threat.Sp2

insitu2 ~ Attendance2 + Zoo.Area.ha2
+ Prop.Threat.Sp2'

# Fit model and generate model summary
mod.IS17.fit <- sem(mod.IS17, data = sem_in_situ_data, fixed.x=FALSE)
summary(mod.IS17.fit, rsq = TRUE)
```

```
## lavaan (0.5-23.1097) converged normally after  25 iterations
##
##   Number of observations                           119
##
##   Estimator                                         ML
##   Minimum Function Test Statistic               42.418
##   Degrees of freedom                                21
##   P-value (Chi-square)                           0.004
##
## Parameter Estimates:
##
##   Information                                 Expected
##   Standard Errors                             Standard
##
## Regressions:
##                      Estimate  Std.Err  z-value  P(>|z|)
##   Attendance2 ~
##     Total.Animals2      0.445    0.060    7.361    0.000
##     Men.Sp.BdyMss2      0.310    0.061    5.047    0.000
##     Mean.Rap.Crck2      0.165    0.064    2.600    0.009
##     X10km_Pop2          0.388    0.052    7.458    0.000
##   Total.Animals2 ~
##     Zoo.Area.ha2        0.142    0.049    2.903    0.004
##     Sp.Richness2        0.809    0.049   16.493    0.000
##   Sp.Richness2 ~
##     Mam.Sp.Rchnss2      0.877    0.055   15.817    0.000
```

```
##     Men.Sp.BdyMss2    -0.168    0.055   -3.028    0.002
##   Mean.Raup.Crick2 ~
##     Total.Animals2     0.489    0.069    7.057    0.000
##     Men.Sp.BdyMss2    -0.468    0.071   -6.614    0.000
##     Prop.Thret.Sp2    -0.287    0.071   -4.053    0.000
##   insitu2 ~
##     Attendance2        0.576    0.074    7.830    0.000
##     Zoo.Area.ha2       0.156    0.070    2.235    0.025
##     Prop.Thret.Sp2     0.149    0.068    2.186    0.029
##
## Covariances:
##                        Estimate  Std.Err  z-value  P(>|z|)
##   Mean.Sp.BodyMass2 ~~
##     X10km_Pop2           -0.023    0.091   -0.248    0.804
##     Zoo.Area.ha2          0.560    0.104    5.363    0.000
##     Mam.Sp.Rchnss2        0.373    0.097    3.845    0.000
##     Prop.Thret.Sp2        0.277    0.094    2.933    0.003
##   X10km_Pop2 ~~
##     Zoo.Area.ha2         -0.099    0.091   -1.087    0.277
##     Mam.Sp.Rchnss2        0.421    0.099    4.262    0.000
##     Prop.Thret.Sp2        0.287    0.095    3.030    0.002
##   Zoo.Area.ha2 ~~
##     Mam.Sp.Rchnss2        0.536    0.103    5.185    0.000
##     Prop.Thret.Sp2        0.189    0.093    2.045    0.041
##   Mam.Sp.Richness2 ~~
##     Prop.Thret.Sp2        0.322    0.096    3.368    0.001
##
## Variances:
##                    Estimate  Std.Err  z-value  P(>|z|)
##    .Attendance2       0.288    0.037    7.714    0.000
##    .Total.Animals2    0.243    0.032    7.714    0.000
##    .Sp.Richness2      0.311    0.040    7.714    0.000
##    .Mean.Rap.Crck2    0.530    0.069    7.714    0.000
##    .insitu2           0.506    0.066    7.714    0.000
##     Men.Sp.BdyMss2    0.992    0.129    7.714    0.000
##     X10km_Pop2        0.992    0.129    7.714    0.000
##     Zoo.Area.ha2      0.992    0.129    7.714    0.000
##     Mam.Sp.Rchnss2    0.992    0.129    7.714    0.000
##     Prop.Thret.Sp2    0.992    0.129    7.714    0.000
##
## R-Square:
##                    Estimate
##     Attendance2       0.688
##     Total.Animals2    0.757
##     Sp.Richness2      0.686
##     Mean.Rap.Crck2    0.462
##     insitu2           0.476
```

```r
# Generate fit indices
fitMeasures(mod.IS17.fit, c("agfi", "rmr", "srmr", "rmsea", "cfi", "nnfi", "tli"))
```

```
##  agfi   rmr  srmr rmsea   cfi  nnfi   tli
## 0.842 0.058 0.059 0.093 0.965 0.942 0.942
```

```r
# Generate modification indices
miIS17 <- modindices(mod.IS17.fit)
print(miIS17[miIS17$mi > 3.0,])
```

```
##                     lhs op               rhs     mi    epc sepc.lv sepc.all
## 44   Mean.Raup.Crick2 ~~          insitu2  3.069  0.084   0.084    0.086
## 61       Sp.Richness2  ~       X10km_Pop2 17.968  0.246   0.246    0.246
## 66   Mean.Raup.Crick2  ~          insitu2  3.318  0.164   0.164    0.162
## 68   Mean.Raup.Crick2  ~      Zoo.Area.ha2  3.688  0.171   0.171    0.171
## 72            insitu2  ~ Mean.Raup.Crick2  3.843  0.144   0.144    0.146
## 80  Mean.Sp.BodyMass2  ~          insitu2  3.180 -0.177  -0.177   -0.175
## 85         X10km_Pop2  ~      Attendance2 10.096  0.490   0.490    0.472
## 86         X10km_Pop2  ~   Total.Animals2 13.199  0.396   0.396    0.397
## 87         X10km_Pop2  ~     Sp.Richness2 18.045  0.554   0.554    0.554
## 88         X10km_Pop2  ~ Mean.Raup.Crick2  5.823  0.220   0.220    0.219
## 97       Zoo.Area.ha2  ~ Mean.Raup.Crick2  7.046  0.216   0.216    0.215
## 104  Mam.Sp.Richness2  ~   Total.Animals2  4.646 -0.279  -0.279   -0.280
## 105  Mam.Sp.Richness2  ~     Sp.Richness2  9.050 -0.500  -0.500   -0.500
## 106  Mam.Sp.Richness2  ~ Mean.Raup.Crick2  6.029 -0.228  -0.228   -0.227
##     sepc.nox
## 44     0.086
## 61     0.247
## 66     0.162
## 68     0.172
## 72     0.146
## 80    -0.175
## 85     0.472
## 86     0.397
## 87     0.554
## 88     0.219
## 97     0.215
## 104   -0.280
## 105   -0.500
## 106   -0.227
```

Model Comparisons 9

Once again we compare the models generated using AICc values. Overall model selection from the pool of competing models is achieved using AICc values, with a threshold of more than 2 AICc units lower than nearest competing model being considered sufficient for model selection.

```r
# Model Comparisons using AICc

# Comparing models with and without adjustment for nested nature of data
# library(AICcmodavg)
# source("lavaan.modavg.R")

aictab.lavaan(list(mod.IS1.fit, mod.IS2.fit, mod.IS3.fit, mod.IS4.fit, mod.IS5.fit, mod.IS6.fit,
                   mod.IS7.fit, mod.IS8.fit, mod.IS9.fit, mod.IS10.fit, mod.IS11.fit, mod.IS12.fit, mod
              c("mod.1", "mod.2", "mod.3", "mod.4", "mod.5", "mod.6", "mod.7", "mod.8",
                "mod.9", "mod.10", "mod.11", "mod.12", "mod.13", "mod.14", "mod.15", "mod.16", "mod.17"
```

```
##
```

```
## Model selection based on AICc:
##
##          K    AICc Delta_AICc AICcWt Cum.Wt       LL
## mod.16 35 2671.34       0.00   0.64   0.64 -1298.34
## mod.17 34 2672.51       1.17   0.36   1.00 -1300.23
## mod.15 34 2683.82      12.48   0.00   1.00 -1305.89
## mod.14 35 2686.40      15.07   0.00   1.00 -1305.87
## mod.13 39 2872.16     200.82   0.00   1.00 -1393.66
## mod.12 40 2873.88     202.55   0.00   1.00 -1393.10
## mod.11 41 2874.18     202.84   0.00   1.00 -1391.80
## mod.10 42 2875.25     203.91   0.00   1.00 -1390.86
## mod.9  43 2877.97     206.63   0.00   1.00 -1390.71
## mod.8  44 2879.99     208.65   0.00   1.00 -1390.19
## mod.7  45 2882.01     210.67   0.00   1.00 -1389.62
## mod.6  46 2884.42     213.08   0.00   1.00 -1389.22
## mod.5  47 2887.57     216.23   0.00   1.00 -1389.15
## mod.4  48 2890.86     219.52   0.00   1.00 -1389.12
## mod.3  49 2894.23     222.89   0.00   1.00 -1389.09
## mod.2  57 3133.59     462.25   0.00   1.00 -1499.23
## mod.1  66 3419.69     748.35   0.00   1.00 -1631.57
```

Based on these results we can see that model 17 is the superior model (lowest AICc value), as the addition of **insitu2** ~ **Mean.Rap.Crck2** does not lower the AIC value by more than 2 units.

Final In Situ Model (Species Presence - Absence)

Contents

Based on the results, we believe model 17 is an accurate representation of the system. No further addition seems conceptually appealling or statistically justified. In addition all pathways appear significant. Therefore we present model 17 as our final In Situ Model based on species presence-absence data.

```
# Chosen In Situ SEM (Presence-Absence)
# Model 17

mod.ISPAFinal <- 'Attendance2 ~ Total.Animals2
+ Mean.Sp.BodyMass2 + Mean.Raup.Crick2 + X10km_Pop2

Total.Animals2 ~ Zoo.Area.ha2 + Sp.Richness2
Sp.Richness2 ~ Mam.Sp.Richness2 + Mean.Sp.BodyMass2
Mean.Raup.Crick2 ~ Total.Animals2 + Mean.Sp.BodyMass2 + Prop.Threat.Sp2

insitu2 ~ Attendance2 + Zoo.Area.ha2
+ Prop.Threat.Sp2'

# Fit model and generate model summary
mod.ISPAFinal.fit <- sem(mod.ISPAFinal, data = sem_in_situ_data, fixed.x=FALSE)
summary(mod.ISPAFinal.fit, rsq = TRUE)
```

```
## lavaan (0.5-23.1097) converged normally after  25 iterations
##
##   Number of observations                           119
##
##   Estimator                                         ML
##   Minimum Function Test Statistic               42.418
```

```
##   Degrees of freedom                                21
##   P-value (Chi-square)                           0.004
##
## Parameter Estimates:
##
##   Information                                 Expected
##   Standard Errors                             Standard
##
## Regressions:
##                      Estimate  Std.Err  z-value  P(>|z|)
##   Attendance2 ~
##     Total.Animals2      0.445    0.060    7.361    0.000
##     Men.Sp.BdyMss2      0.310    0.061    5.047    0.000
##     Mean.Rap.Crck2      0.165    0.064    2.600    0.009
##     X10km_Pop2          0.388    0.052    7.458    0.000
##   Total.Animals2 ~
##     Zoo.Area.ha2        0.142    0.049    2.903    0.004
##     Sp.Richness2        0.809    0.049   16.493    0.000
##   Sp.Richness2 ~
##     Mam.Sp.Rchnss2      0.877    0.055   15.817    0.000
##     Men.Sp.BdyMss2     -0.168    0.055   -3.028    0.002
##   Mean.Raup.Crick2 ~
##     Total.Animals2      0.489    0.069    7.057    0.000
##     Men.Sp.BdyMss2     -0.468    0.071   -6.614    0.000
##     Prop.Thret.Sp2     -0.287    0.071   -4.053    0.000
##   insitu2 ~
##     Attendance2         0.576    0.074    7.830    0.000
##     Zoo.Area.ha2        0.156    0.070    2.235    0.025
##     Prop.Thret.Sp2      0.149    0.068    2.186    0.029
##
## Covariances:
##                        Estimate  Std.Err  z-value  P(>|z|)
##   Mean.Sp.BodyMass2 ~~
##     X10km_Pop2           -0.023    0.091   -0.248    0.804
##     Zoo.Area.ha2          0.560    0.104    5.363    0.000
##     Mam.Sp.Rchnss2        0.373    0.097    3.845    0.000
##     Prop.Thret.Sp2        0.277    0.094    2.933    0.003
##   X10km_Pop2 ~~
##     Zoo.Area.ha2         -0.099    0.091   -1.087    0.277
##     Mam.Sp.Rchnss2        0.421    0.099    4.262    0.000
##     Prop.Thret.Sp2        0.287    0.095    3.030    0.002
##   Zoo.Area.ha2 ~~
##     Mam.Sp.Rchnss2        0.536    0.103    5.185    0.000
##     Prop.Thret.Sp2        0.189    0.093    2.045    0.041
##   Mam.Sp.Richness2 ~~
##     Prop.Thret.Sp2        0.322    0.096    3.368    0.001
##
## Variances:
##                    Estimate  Std.Err  z-value  P(>|z|)
##    .Attendance2       0.288    0.037    7.714    0.000
##    .Total.Animals2    0.243    0.032    7.714    0.000
##    .Sp.Richness2      0.311    0.040    7.714    0.000
##    .Mean.Rap.Crck2    0.530    0.069    7.714    0.000
##    .insitu2           0.506    0.066    7.714    0.000
```

```
##     Men.Sp.BdyMss2    0.992    0.129    7.714    0.000
##     X10km_Pop2        0.992    0.129    7.714    0.000
##     Zoo.Area.ha2      0.992    0.129    7.714    0.000
##     Mam.Sp.Rchnss2    0.992    0.129    7.714    0.000
##     Prop.Thret.Sp2    0.992    0.129    7.714    0.000
##
## R-Square:
##                    Estimate
##     Attendance2       0.688
##     Total.Animals2    0.757
##     Sp.Richness2      0.686
##     Mean.Rap.Crck2    0.462
##     insitu2           0.476
```

```
# Generate fit indices
fitMeasures(mod.ISPAFinal.fit, c("agfi", "rmr", "srmr", "rmsea", "cfi", "nnfi", "tli"))
```

```
##  agfi   rmr  srmr rmsea   cfi  nnfi   tli
## 0.842 0.058 0.059 0.093 0.965 0.942 0.942
```

```
# Generate modification indices
miISPAFinal <- modindices(mod.ISPAFinal.fit)
print(miISPAFinal[miISPAFinal$mi > 3.0,])
```

```
##                   lhs op               rhs     mi    epc sepc.lv sepc.all
## 44   Mean.Raup.Crick2 ~~          insitu2  3.069  0.084   0.084    0.086
## 61       Sp.Richness2  ~       X10km_Pop2 17.968  0.246   0.246    0.246
## 66   Mean.Raup.Crick2  ~          insitu2  3.318  0.164   0.164    0.162
## 68   Mean.Raup.Crick2  ~      Zoo.Area.ha2  3.688  0.171   0.171    0.171
## 72            insitu2  ~ Mean.Raup.Crick2  3.843  0.144   0.144    0.146
## 80  Mean.Sp.BodyMass2  ~          insitu2  3.180 -0.177  -0.177   -0.175
## 85         X10km_Pop2  ~      Attendance2 10.096  0.490   0.490    0.472
## 86         X10km_Pop2  ~    Total.Animals2 13.199  0.396   0.396    0.397
## 87         X10km_Pop2  ~      Sp.Richness2 18.045  0.554   0.554    0.554
## 88         X10km_Pop2  ~ Mean.Raup.Crick2  5.823  0.220   0.220    0.219
## 97       Zoo.Area.ha2  ~ Mean.Raup.Crick2  7.046  0.216   0.216    0.215
## 104  Mam.Sp.Richness2  ~   Total.Animals2  4.646 -0.279  -0.279   -0.280
## 105  Mam.Sp.Richness2  ~     Sp.Richness2  9.050 -0.500  -0.500   -0.500
## 106  Mam.Sp.Richness2  ~ Mean.Raup.Crick2  6.029 -0.228  -0.228   -0.227
##     sepc.nox
## 44     0.086
## 61     0.247
## 66     0.162
## 68     0.172
## 72     0.146
## 80    -0.175
## 85     0.472
## 86     0.397
## 87     0.554
## 88     0.219
## 97     0.215
## 104   -0.280
## 105   -0.500
## 106   -0.227
```

Tests of Mediation

It is clear from Model 17 that several pathways are mediated in the system. It is necessary to evaluate whether this mediation is appropriate by comparing models with complete, partial and no mediation. Here we show tests of mediation for three of these relationships.

insitu2 ~ Mean.Sp.BodyMass2

insitu2 ~ **Mean.Sp.BodyMass2** is completely mediated by **Attendance2** ~ **Mean.Sp.BodyMass2**. Below we compare models with complete (full, i.e. Model 17) mediation, partial mediation and no mediation. Results from this output suggest that complete mediation (model 17) is the superior model.

```
# Complete Mediation Model (Model 17)

FULL1A.MOD17 <- 'Attendance2 ~ Total.Animals2
+ Mean.Sp.BodyMass2 + Mean.Raup.Crick2 + X10km_Pop2

Total.Animals2 ~ Zoo.Area.ha2 + Sp.Richness2
Sp.Richness2 ~ Mam.Sp.Richness2 + Mean.Sp.BodyMass2
Mean.Raup.Crick2 ~ Total.Animals2 + Mean.Sp.BodyMass2 + Prop.Threat.Sp2

insitu2 ~ Attendance2 + Zoo.Area.ha2
+ Prop.Threat.Sp2'

FULL1A.MOD17.fit <- sem(FULL1A.MOD17, data = sem_in_situ_data, fixed.x=FALSE)

# Partial Mediation Model

PARTIAL1A <- 'Attendance2 ~ Total.Animals2
+ Mean.Sp.BodyMass2 + Mean.Raup.Crick2 + X10km_Pop2

Total.Animals2 ~ Zoo.Area.ha2 + Sp.Richness2
Sp.Richness2 ~ Mam.Sp.Richness2 + Mean.Sp.BodyMass2
Mean.Raup.Crick2 ~ Total.Animals2 + Mean.Sp.BodyMass2 + Prop.Threat.Sp2

insitu2 ~ Attendance2 + Zoo.Area.ha2
+ Prop.Threat.Sp2 + Mean.Sp.BodyMass2'

PARTIAL1A.fit <- sem(PARTIAL1A, data = sem_in_situ_data, fixed.x=FALSE)

# No Mediation Model

NONE1A <- 'Attendance2 ~ Total.Animals2
+ Mean.Raup.Crick2 + X10km_Pop2

Total.Animals2 ~ Zoo.Area.ha2 + Sp.Richness2
Sp.Richness2 ~ Mam.Sp.Richness2 + Mean.Sp.BodyMass2
Mean.Raup.Crick2 ~ Total.Animals2 + Mean.Sp.BodyMass2 + Prop.Threat.Sp2

insitu2 ~ Attendance2 + Zoo.Area.ha2
+ Prop.Threat.Sp2 + Mean.Sp.BodyMass2'

NONE1A.fit <- sem(NONE1A, data = sem_in_situ_data, fixed.x=FALSE)
```

```r
aictab.lavaan(list(FULL1A.MOD17.fit, PARTIAL1A.fit, NONE1A.fit),
              c("full mediation", "partial mediation", "no mediation"))
```

```
##
## Model selection based on AICc:
##
##                      K    AICc Delta_AICc AICcWt Cum.Wt       LL
## full mediation    34 2672.51       0.00   0.54   0.54 -1300.23
## partial mediation 35 2672.84       0.33   0.46   1.00 -1299.09
## no mediation      34 2693.38      20.87   0.00   1.00 -1310.67
```

insitu2 ~ Prop.Threat.Sp2

insitu2 ~ **Prop.Threat.Sp2** is not mediated. Therefore we want to test if partial mediation is appropriate by including by **Attendance2** ~ **Prop.Threat.Sp2**. Below we compare models with complete (full) mediation, partial mediation and no mediation (Model 17). Results from this output suggest that no mediation (model 17) is the superior model.

```r
# Complete Mediation Model

FULL2A <- 'Attendance2 ~ Total.Animals2
+ Mean.Sp.BodyMass2 + Mean.Raup.Crick2 + X10km_Pop2 + Prop.Threat.Sp2

Total.Animals2 ~ Zoo.Area.ha2 + Sp.Richness2
Sp.Richness2 ~ Mam.Sp.Richness2 + Mean.Sp.BodyMass2
Mean.Raup.Crick2 ~ Total.Animals2 + Mean.Sp.BodyMass2 + Prop.Threat.Sp2

insitu2 ~ Attendance2 + Zoo.Area.ha2'

FULL2A.fit <- sem(FULL2A, data = sem_in_situ_data, fixed.x=FALSE)

# Partial Mediation Model

PARTIAL2A <- 'Attendance2 ~ Total.Animals2
+ Mean.Sp.BodyMass2 + Mean.Raup.Crick2 + X10km_Pop2 + Prop.Threat.Sp2

Total.Animals2 ~ Zoo.Area.ha2 + Sp.Richness2
Sp.Richness2 ~ Mam.Sp.Richness2 + Mean.Sp.BodyMass2
Mean.Raup.Crick2 ~ Total.Animals2 + Mean.Sp.BodyMass2 + Prop.Threat.Sp2

insitu2 ~ Attendance2 + Zoo.Area.ha2
+ Prop.Threat.Sp2'

PARTIAL2A.fit <- sem(PARTIAL2A, data = sem_in_situ_data, fixed.x=FALSE)

# No Mediation Model (Model 17)

NONE2A.MOD17 <- 'Attendance2 ~ Total.Animals2
+ Mean.Sp.BodyMass2 + Mean.Raup.Crick2 + X10km_Pop2

Total.Animals2 ~ Zoo.Area.ha2 + Sp.Richness2
Sp.Richness2 ~ Mam.Sp.Richness2 + Mean.Sp.BodyMass2
Mean.Raup.Crick2 ~ Total.Animals2 + Mean.Sp.BodyMass2 + Prop.Threat.Sp2
```

```
insitu2 ~ Attendance2 + Zoo.Area.ha2
+ Prop.Threat.Sp2'

NONE2A.MOD17.fit <- sem(NONE2A.MOD17, data = sem_in_situ_data, fixed.x=FALSE)

aictab.lavaan(list(FULL2A.fit, PARTIAL2A.fit, NONE2A.MOD17.fit),
              c("full mediation", "partial mediation", "no mediation"))
```

```
##
## Model selection based on AICc:
##
##                    K    AICc Delta_AICc AICcWt Cum.Wt       LL
## no mediation      34 2672.51       0.00   0.62   0.62 -1300.23
## partial mediation 35 2674.09       1.59   0.28   0.90 -1299.72
## full mediation    34 2676.23       3.72   0.10   1.00 -1302.10
```

Similar to the Attendance Model, two In Situ Models were developed, one based on species presence-absence per institution and the the other which also conisdered species' population sizes per institution. This was important for calculating mean species body mass per institution, the proportion of threatened species per institution etc. Here we develop the model based on species abundance.

Species Abundance

Model 1 (a priori meta-model)

Contents

We now define our starting model for the SEM based on the a priori meta-model which is constructed based on evidence from the litrature and combined with proposed causal hypotheses. This model includes all previously identified evidence-based relationships and the numerous proposed causal pathways to visitor attendance. The development of this model can be found in the Supplementary information. Here we use the same proposed predictors of attendance for the in situ contributions. We use the species abundance Attendance Model to guide model development and identify relationships to visitor attendance, due to the larger sample size and therefore higher explanatory power in that model.

```
# In Situ SEM (Abundance)

# Model 1
# Based on a priori meta-model and results from the species abundance Attendance Model

mod.IS1A <- 'Attendance2 ~ Zoo.Area.ha2 + Sp.Richness2 + Total.Animals2
+ Mam.Sp.Richness2 + Prop.Mam.Abdun2 + Prop.Threat.Abund2
+ Mean.Sp.BodyMassXAbund2 + Brillouin.Index2 + Mean.Raup.Crick2
+ X50km_Pop2 + X10km_Pop2

Total.Animals2 ~ Zoo.Area.ha2 + Sp.Richness2 + Mean.Sp.BodyMassXAbund2
Sp.Richness2 ~ Mam.Sp.Richness2 + Mean.Sp.BodyMassXAbund2 + Zoo.Area.ha2
Mean.Raup.Crick2 ~ Mean.Sp.BodyMassXAbund2 + Zoo.Area.ha2
Brillouin.Index2 ~ Sp.Richness2 + Total.Animals2

insitu2 ~ Attendance2 + Zoo.Area.ha2 + Sp.Richness2 + Total.Animals2
+ Mam.Sp.Richness2 + Prop.Mam.Abdun2 + Prop.Threat.Abund2
```

```
+ Mean.Sp.BodyMassXAbund2 + Brillouin.Index2 + Mean.Raup.Crick2
+ X50km_Pop2 + X10km_Pop2'
```

We then fit the model, generate the model summary and generate a selection of absolute fit indices (e.g. Standardized Root Mean Square Residual) and incremental fit indices (e.g. Comparative Fit Index), to account for the differential sensitivity of fit indices to data distribution, model size and sample size (Hu & Bentler, 1999). We then generate modification indices to identify suspected pathways for inclusion, with a standard cut-off level for the chi-square test criterion of 3.84 (Burnham and Anderson, 2002). As the in situ dataset contains only AZA institutions, there was no need to nest the data or include GDP in the model (institutions within countries).

```
# Fit model and generate model summary
mod.IS1A.fit <- sem(mod.IS1A , data = sem_in_situ_data, fixed.x=FALSE)
summary(mod.IS1A.fit, rsq = TRUE)
```

```
## lavaan (0.5-23.1097) converged normally after  51 iterations
##
##   Number of observations                           119
##
##   Estimator                                         ML
##   Minimum Function Test Statistic              101.985
##   Degrees of freedom                                24
##   P-value (Chi-square)                           0.000
##
## Parameter Estimates:
##
##   Information                                 Expected
##   Standard Errors                             Standard
##
## Regressions:
##                    Estimate  Std.Err  z-value  P(>|z|)
##   Attendance2 ~
##     Zoo.Area.ha2      0.029    0.079    0.373    0.709
##     Sp.Richness2     -0.413    0.184   -2.238    0.025
##     Total.Animals2    0.742    0.144    5.150    0.000
##     Mam.Sp.Rchnss2    0.057    0.102    0.558    0.577
##     Prop.Mam.Abdn2   -0.015    0.057   -0.261    0.794
##     Prp.Thrt.Abnd2   -0.068    0.053   -1.282    0.200
##     Mn.Sp.BdyMsXA2    0.250    0.082    3.056    0.002
##     Brilloun.Indx2    0.247    0.090    2.737    0.006
##     Mean.Rap.Crck2    0.087    0.058    1.488    0.137
##     X50km_Pop2        0.148    0.075    1.966    0.049
##     X10km_Pop2        0.335    0.085    3.963    0.000
##   Total.Animals2 ~
##     Zoo.Area.ha2      0.303    0.053    5.687    0.000
##     Sp.Richness2      0.748    0.045   16.568    0.000
##     Mn.Sp.BdyMsXA2   -0.262    0.050   -5.242    0.000
##   Sp.Richness2 ~
##     Mam.Sp.Rchnss2    0.867    0.059   14.592    0.000
##     Mn.Sp.BdyMsXA2   -0.208    0.059   -3.518    0.000
##     Zoo.Area.ha2     -0.013    0.068   -0.192    0.848
##   Mean.Raup.Crick2 ~
##     Mn.Sp.BdyMsXA2   -0.687    0.088   -7.810    0.000
##     Zoo.Area.ha2      0.350    0.088    3.973    0.000
##   Brillouin.Index2 ~
```

```
##     Sp.Richness2        1.573    0.094   16.749    0.000
##     Total.Animals2     -1.081    0.094  -11.507    0.000
##   insitu2 ~
##     Attendance2         0.590    0.121    4.873    0.000
##     Zoo.Area.ha2        0.204    0.105    1.951    0.051
##     Sp.Richness2        0.189    0.249    0.760    0.448
##     Total.Animals2     -0.331    0.210   -1.574    0.115
##     Mam.Sp.Rchnss2      0.196    0.135    1.447    0.148
##     Prop.Mam.Abdn2     -0.059    0.076   -0.774    0.439
##     Prp.Thrt.Abnd2      0.197    0.070    2.801    0.005
##     Mn.Sp.BdyMsXA2     -0.045    0.112   -0.400    0.689
##     Brilloun.Indx2     -0.196    0.123   -1.592    0.111
##     Mean.Rap.Crck2      0.102    0.078    1.314    0.189
##     X50km_Pop2         -0.024    0.101   -0.236    0.814
##     X10km_Pop2          0.025    0.119    0.207    0.836
##
## Covariances:
##                            Estimate  Std.Err  z-value  P(>|z|)
##   Zoo.Area.ha2 ~~
##     Mam.Sp.Rchnss2            0.536    0.103    5.185    0.000
##     Prop.Mam.Abdn2            0.223    0.093    2.396    0.017
##     Prp.Thrt.Abnd2            0.071    0.091    0.776    0.438
##     Mn.Sp.BdyMsXA2            0.527    0.103    5.120    0.000
##     X50km_Pop2               -0.014    0.091   -0.155    0.876
##     X10km_Pop2               -0.099    0.091   -1.087    0.277
##   Mam.Sp.Richness2 ~~
##     Prop.Mam.Abdn2           -0.025    0.091   -0.273    0.785
##     Prp.Thrt.Abnd2            0.203    0.093    2.190    0.028
##     Mn.Sp.BdyMsXA2            0.219    0.093    2.357    0.018
##     X50km_Pop2                0.363    0.097    3.752    0.000
##     X10km_Pop2                0.421    0.099    4.262    0.000
##   Prop.Mam.Abdun2 ~~
##     Prp.Thrt.Abnd2           -0.195    0.093   -2.102    0.036
##     Mn.Sp.BdyMsXA2            0.453    0.100    4.531    0.000
##     X50km_Pop2               -0.290    0.095   -3.062    0.002
##     X10km_Pop2               -0.373    0.097   -3.839    0.000
##   Prop.Threat.Abund2 ~~
##     Mn.Sp.BdyMsXA2            0.092    0.091    1.012    0.312
##     X50km_Pop2                0.035    0.091    0.382    0.703
##     X10km_Pop2                0.278    0.094    2.948    0.003
##   Mean.Sp.BodyMassXAbund2 ~~
##     X50km_Pop2               -0.235    0.093   -2.516    0.012
##     X10km_Pop2               -0.209    0.093   -2.245    0.025
##   X50km_Pop2 ~~
##     X10km_Pop2                0.738    0.113    6.511    0.000
##
## Variances:
##                    Estimate  Std.Err  z-value  P(>|z|)
##    .Attendance2       0.265    0.034    7.714    0.000
##    .Total.Animals2    0.198    0.026    7.714    0.000
##    .Sp.Richness2      0.292    0.038    7.714    0.000
##    .Mean.Rap.Crck2    0.655    0.085    7.714    0.000
##    .Brilloun.Indx2    0.274    0.036    7.714    0.000
##    .insitu2           0.462    0.060    7.714    0.000
```

```
##     Zoo.Area.ha2      0.992    0.129    7.714    0.000
##     Mam.Sp.Rchnss2    0.992    0.129    7.714    0.000
##     Prop.Mam.Abdn2    0.992    0.129    7.714    0.000
##     Prp.Thrt.Abnd2    0.992    0.129    7.714    0.000
##     Mn.Sp.BdyMsXA2    0.992    0.129    7.714    0.000
##     X50km_Pop2        0.992    0.129    7.714    0.000
##     X10km_Pop2        0.992    0.129    7.714    0.000
##
## R-Square:
##                    Estimate
##     Attendance2       0.707
##     Total.Animals2    0.801
##     Sp.Richness2      0.705
##     Mean.Rap.Crck2    0.339
##     Brilloun.Indx2    0.724
##     insitu2           0.529
```

```
# Generate fit indices
fitMeasures(mod.IS1A.fit, c("agfi", "rmr", "srmr", "rmsea", "cfi", "nnfi", "tli"))
```

```
##  agfi   rmr  srmr rmsea   cfi  nnfi   tli
## 0.617 0.071 0.072 0.165 0.906 0.778 0.778
```

```
# Generate modification indices
miIS1A <- modindices(mod.IS1A.fit)
print(miIS1A[miIS1A$mi > 3.0,])
```

```
##                      lhs op                rhs     mi    epc sepc.lv
## 73          Total.Animals2 ~~       Sp.Richness2  3.234 -0.049  -0.049
## 77            Sp.Richness2 ~~   Mean.Raup.Crick2  5.959  0.098   0.098
## 84          Total.Animals2  ~        Attendance2  5.124  0.223   0.223
## 88          Total.Animals2  ~   Mam.Sp.Richness2  3.234  0.147   0.147
## 90          Total.Animals2  ~ Prop.Threat.Abund2  7.697 -0.116  -0.116
## 91          Total.Animals2  ~         X50km_Pop2  5.878  0.110   0.110
## 93            Sp.Richness2  ~        Attendance2  7.396  0.319   0.319
## 94            Sp.Richness2  ~     Total.Animals2  3.234 -0.250  -0.250
## 95            Sp.Richness2  ~   Mean.Raup.Crick2  5.959  0.149   0.149
## 97            Sp.Richness2  ~            insitu2 18.233  0.617   0.617
## 98            Sp.Richness2  ~     Prop.Mam.Abdun2 30.555 -0.313  -0.313
## 101           Sp.Richness2  ~         X10km_Pop2 15.061  0.240   0.240
## 102       Mean.Raup.Crick2  ~        Attendance2  3.863  0.206   0.206
## 103       Mean.Raup.Crick2  ~     Total.Animals2  3.138  0.163   0.163
## 104       Mean.Raup.Crick2  ~       Sp.Richness2  3.946  0.163   0.163
## 108       Mean.Raup.Crick2  ~     Prop.Mam.Abdun2  6.145 -0.208  -0.208
## 110       Mean.Raup.Crick2  ~         X50km_Pop2  3.997  0.155   0.155
## 118      Brillouin.Index2  ~ Prop.Threat.Abund2  7.234  0.131   0.131
## 124           Zoo.Area.ha2  ~       Sp.Richness2 13.757  1.240   1.240
## 126           Zoo.Area.ha2  ~   Brillouin.Index2  6.857  0.300   0.300
## 136       Mam.Sp.Richness2  ~       Sp.Richness2 11.364 -0.687  -0.687
## 138       Mam.Sp.Richness2  ~   Brillouin.Index2  7.180 -0.281  -0.281
## 146        Prop.Mam.Abdun2  ~        Attendance2 10.517 -0.780  -0.780
## 147        Prop.Mam.Abdun2  ~     Total.Animals2 13.583 -0.462  -0.462
## 148        Prop.Mam.Abdun2  ~       Sp.Richness2 21.679 -0.650  -0.650
## 149        Prop.Mam.Abdun2  ~   Mean.Raup.Crick2  6.344 -0.235  -0.235
## 151        Prop.Mam.Abdun2  ~            insitu2  6.225 -1.291  -1.291
## 159     Prop.Threat.Abund2  ~     Total.Animals2  8.211 -0.390  -0.390
```

```
## 162      Prop.Threat.Abund2  ~   Brillouin.Index2 10.254  0.319   0.319
## 163      Prop.Threat.Abund2  ~            insitu2  6.853 -1.469  -1.469
## 170 Mean.Sp.BodyMassXAbund2  ~        Attendance2 12.008  1.334   1.334
## 171 Mean.Sp.BodyMassXAbund2  ~     Total.Animals2 22.348  1.168   1.168
## 172 Mean.Sp.BodyMassXAbund2  ~       Sp.Richness2 20.452  1.246   1.246
## 173 Mean.Sp.BodyMassXAbund2  ~   Mean.Raup.Crick2 10.504  0.587   0.587
## 175 Mean.Sp.BodyMassXAbund2  ~            insitu2 13.991  3.607   3.607
## 185              X50km_Pop2  ~   Mean.Raup.Crick2  3.366  0.130   0.130
## 187              X50km_Pop2  ~            insitu2  4.447  0.829   0.829
## 195              X10km_Pop2  ~     Total.Animals2  4.544  0.181   0.181
## 196              X10km_Pop2  ~       Sp.Richness2  8.735  0.279   0.279
##     sepc.all sepc.nox
## 73    -0.050   -0.050
## 77     0.099    0.099
## 84     0.213    0.213
## 88     0.147    0.147
## 90    -0.116   -0.116
## 91     0.110    0.111
## 93     0.305    0.305
## 94    -0.250   -0.250
## 95     0.149    0.149
## 97     0.614    0.614
## 98    -0.313   -0.314
## 101    0.240    0.241
## 102    0.197    0.197
## 103    0.163    0.163
## 104    0.163    0.163
## 108   -0.208   -0.209
## 110    0.155    0.155
## 118    0.131    0.132
## 124    1.240    1.240
## 126    0.300    0.300
## 136   -0.687   -0.687
## 138   -0.281   -0.281
## 146   -0.745   -0.745
## 147   -0.462   -0.462
## 148   -0.650   -0.650
## 149   -0.235   -0.235
## 151   -1.285   -1.285
## 159   -0.390   -0.390
## 162    0.319    0.319
## 163   -1.462   -1.462
## 170    1.273    1.273
## 171    1.168    1.168
## 172    1.246    1.246
## 173    0.587    0.587
## 175    3.590    3.590
## 185    0.130    0.130
## 187    0.825    0.825
## 195    0.181    0.181
## 196    0.279    0.279
```

Model 2

None of the modification indices from the first model are conceptually appealling, so now we use p-values to identify potentially unsupported pathways, with a threshold of 0.05. Highest p-values are considered first for removal. Based on the results generated from the first model, the highest p-value relationship to be considered for removal is **Sp.Richness2** ~ **Zoo.Area.ha2** with a p-value of **0.848**. Therefore we decide to remove this pathway. The model summary, fit indices and modification indices were all generated for the model.

```
# In Situ SEM (Abundance)

# Model 2
# Removal of Sp.Richness2 ~ Zoo.Area.ha2, p = 0.848

mod.IS2A <- 'Attendance2 ~ Zoo.Area.ha2 + Sp.Richness2 + Total.Animals2
+ Mam.Sp.Richness2 + Prop.Mam.Abdun2 + Prop.Threat.Abund2
+ Mean.Sp.BodyMassXAbund2 + Brillouin.Index2 + Mean.Raup.Crick2
+ X50km_Pop2 + X10km_Pop2

Total.Animals2 ~ Zoo.Area.ha2 + Sp.Richness2 + Mean.Sp.BodyMassXAbund2
Sp.Richness2 ~ Mam.Sp.Richness2 + Mean.Sp.BodyMassXAbund2
Mean.Raup.Crick2 ~ Mean.Sp.BodyMassXAbund2 + Zoo.Area.ha2
Brillouin.Index2 ~ Sp.Richness2 + Total.Animals2

insitu2 ~ Attendance2 + Zoo.Area.ha2 + Sp.Richness2 + Total.Animals2
+ Mam.Sp.Richness2 + Prop.Mam.Abdun2 + Prop.Threat.Abund2
+ Mean.Sp.BodyMassXAbund2 + Brillouin.Index2 + Mean.Raup.Crick2
+ X50km_Pop2 + X10km_Pop2'

# Fit model and generate model summary
mod.IS2A.fit <- sem(mod.IS2A , data = sem_in_situ_data, fixed.x=FALSE)
summary(mod.IS2A.fit, rsq = TRUE)
```

```
## lavaan (0.5-23.1097) converged normally after  55 iterations
##
##   Number of observations                           119
##
##   Estimator                                         ML
##   Minimum Function Test Statistic              102.021
##   Degrees of freedom                                25
##   P-value (Chi-square)                           0.000
##
## Parameter Estimates:
##
##   Information                                 Expected
##   Standard Errors                             Standard
##
## Regressions:
##                      Estimate  Std.Err  z-value  P(>|z|)
##   Attendance2 ~
##     Zoo.Area.ha2        0.029    0.079    0.373    0.709
##     Sp.Richness2       -0.413    0.184   -2.238    0.025
##     Total.Animals2      0.742    0.144    5.150    0.000
##     Mam.Sp.Rchnss2      0.057    0.102    0.561    0.575
##     Prop.Mam.Abdn2     -0.015    0.057   -0.261    0.794
```

```
##     Prp.Thrt.Abnd2     -0.068    0.053   -1.282    0.200
##     Mn.Sp.BdyMsXA2      0.250    0.082    3.052    0.002
##     Brilloun.Indx2      0.247    0.090    2.737    0.006
##     Mean.Rap.Crck2      0.087    0.058    1.488    0.137
##     X50km_Pop2          0.148    0.075    1.966    0.049
##     X10km_Pop2          0.335    0.085    3.963    0.000
##   Total.Animals2 ~
##     Zoo.Area.ha2        0.303    0.054    5.662    0.000
##     Sp.Richness2        0.748    0.045   16.498    0.000
##     Mn.Sp.BdyMsXA2     -0.262    0.050   -5.234    0.000
##   Sp.Richness2 ~
##     Mam.Sp.Rchnss2      0.861    0.051   16.866    0.000
##     Mn.Sp.BdyMsXA2     -0.213    0.051   -4.179    0.000
##   Mean.Raup.Crick2 ~
##     Mn.Sp.BdyMsXA2     -0.687    0.088   -7.810    0.000
##     Zoo.Area.ha2        0.350    0.088    3.973    0.000
##   Brillouin.Index2 ~
##     Sp.Richness2        1.573    0.094   16.708    0.000
##     Total.Animals2     -1.081    0.094  -11.496    0.000
##   insitu2 ~
##     Attendance2         0.590    0.121    4.873    0.000
##     Zoo.Area.ha2        0.204    0.105    1.951    0.051
##     Sp.Richness2        0.189    0.249    0.760    0.447
##     Total.Animals2     -0.331    0.210   -1.574    0.115
##     Mam.Sp.Rchnss2      0.196    0.135    1.452    0.146
##     Prop.Mam.Abdn2     -0.059    0.076   -0.774    0.439
##     Prp.Thrt.Abnd2      0.197    0.070    2.801    0.005
##     Mn.Sp.BdyMsXA2     -0.045    0.113   -0.399    0.690
##     Brilloun.Indx2     -0.196    0.123   -1.592    0.111
##     Mean.Rap.Crck2      0.102    0.078    1.314    0.189
##     X50km_Pop2         -0.024    0.101   -0.236    0.814
##     X10km_Pop2          0.025    0.119    0.207    0.836
##
## Covariances:
##                            Estimate  Std.Err  z-value  P(>|z|)
##   Zoo.Area.ha2 ~~
##     Mam.Sp.Rchnss2            0.536    0.103    5.185    0.000
##     Prop.Mam.Abdn2            0.223    0.093    2.396    0.017
##     Prp.Thrt.Abnd2            0.071    0.091    0.776    0.438
##     Mn.Sp.BdyMsXA2            0.527    0.103    5.120    0.000
##     X50km_Pop2               -0.014    0.091   -0.155    0.876
##     X10km_Pop2               -0.099    0.091   -1.087    0.277
##   Mam.Sp.Richness2 ~~
##     Prop.Mam.Abdn2           -0.025    0.091   -0.273    0.785
##     Prp.Thrt.Abnd2            0.203    0.093    2.190    0.028
##     Mn.Sp.BdyMsXA2            0.219    0.093    2.357    0.018
##     X50km_Pop2                0.363    0.097    3.752    0.000
##     X10km_Pop2                0.421    0.099    4.262    0.000
##   Prop.Mam.Abdun2 ~~
##     Prp.Thrt.Abnd2           -0.195    0.093   -2.102    0.036
##     Mn.Sp.BdyMsXA2            0.453    0.100    4.531    0.000
##     X50km_Pop2               -0.290    0.095   -3.062    0.002
##     X10km_Pop2               -0.373    0.097   -3.839    0.000
##   Prop.Threat.Abund2 ~~
```

```
##     Mn.Sp.BdyMsXA2              0.092    0.091    1.012    0.312
##     X50km_Pop2                  0.035    0.091    0.382    0.703
##     X10km_Pop2                  0.278    0.094    2.948    0.003
##   Mean.Sp.BodyMassXAbund2 ~~
##     X50km_Pop2                 -0.235    0.093   -2.516    0.012
##     X10km_Pop2                 -0.209    0.093   -2.245    0.025
##   X50km_Pop2 ~~
##     X10km_Pop2                  0.738    0.113    6.511    0.000
##
## Variances:
##                    Estimate  Std.Err  z-value  P(>|z|)
##    .Attendance2       0.265    0.034    7.714    0.000
##    .Total.Animals2    0.198    0.026    7.714    0.000
##    .Sp.Richness2      0.292    0.038    7.714    0.000
##    .Mean.Rap.Crck2    0.655    0.085    7.714    0.000
##    .Brilloun.Indx2    0.274    0.036    7.714    0.000
##    .insitu2           0.462    0.060    7.714    0.000
##     Zoo.Area.ha2      0.992    0.129    7.714    0.000
##     Mam.Sp.Rchnss2    0.992    0.129    7.714    0.000
##     Prop.Mam.Abdn2    0.992    0.129    7.714    0.000
##     Prp.Thrt.Abnd2    0.992    0.129    7.714    0.000
##     Mn.Sp.BdyMsXA2    0.992    0.129    7.714    0.000
##     X50km_Pop2        0.992    0.129    7.714    0.000
##     X10km_Pop2        0.992    0.129    7.714    0.000
##
## R-Square:
##                    Estimate
##     Attendance2       0.707
##     Total.Animals2    0.801
##     Sp.Richness2      0.705
##     Mean.Rap.Crck2    0.339
##     Brilloun.Indx2    0.723
##     insitu2           0.529
```

```
# Generate fit indices
fitMeasures(mod.IS2A.fit, c("agfi", "rmr", "srmr", "rmsea", "cfi", "nnfi", "tli"))
```

```
##  agfi   rmr  srmr rmsea   cfi  nnfi   tli
## 0.632 0.071 0.072 0.161 0.907 0.789 0.789
```

```
# Generate modification indices
miIS2A <- modindices(mod.IS2A.fit)
print(miIS2A[miIS2A$mi > 3.0,])
```

```
##                   lhs op                rhs     mi    epc sepc.lv
## 72         Total.Animals2 ~~      Sp.Richness2  3.206 -0.049  -0.049
## 76           Sp.Richness2 ~~  Mean.Raup.Crick2  5.986  0.098   0.098
## 83         Total.Animals2  ~       Attendance2  5.117  0.223   0.223
## 87         Total.Animals2  ~  Mam.Sp.Richness2  3.206  0.145   0.145
## 89         Total.Animals2  ~ Prop.Threat.Abund2  7.695 -0.116  -0.116
## 90         Total.Animals2  ~        X50km_Pop2  5.874  0.110   0.110
## 92           Sp.Richness2  ~       Attendance2  7.188  0.313   0.313
## 94           Sp.Richness2  ~  Mean.Raup.Crick2  5.188  0.133   0.133
## 96           Sp.Richness2  ~           insitu2 14.693  0.507   0.507
## 98           Sp.Richness2  ~    Prop.Mam.Abdun2 30.563 -0.313  -0.313
## 101          Sp.Richness2  ~        X10km_Pop2 13.975  0.219   0.219
```

```
## 102        Mean.Raup.Crick2  ~        Attendance2  3.876  0.207   0.207
## 103        Mean.Raup.Crick2  ~     Total.Animals2  3.156  0.164   0.164
## 104        Mean.Raup.Crick2  ~       Sp.Richness2  3.980  0.165   0.165
## 108        Mean.Raup.Crick2  ~    Prop.Mam.Abdun2  6.144 -0.208  -0.208
## 110        Mean.Raup.Crick2  ~         X50km_Pop2  3.997  0.155   0.155
## 118        Brillouin.Index2  ~ Prop.Threat.Abund2  7.232  0.131   0.131
## 126            Zoo.Area.ha2  ~   Brillouin.Index2  4.076  0.188   0.188
## 136        Mam.Sp.Richness2  ~       Sp.Richness2  6.754 -0.427  -0.427
## 138        Mam.Sp.Richness2  ~   Brillouin.Index2  6.322 -0.253  -0.253
## 146         Prop.Mam.Abdun2  ~        Attendance2 10.515 -0.780  -0.780
## 147         Prop.Mam.Abdun2  ~     Total.Animals2 13.581 -0.462  -0.462
## 148         Prop.Mam.Abdun2  ~       Sp.Richness2 21.672 -0.649  -0.649
## 149         Prop.Mam.Abdun2  ~   Mean.Raup.Crick2  6.344 -0.235  -0.235
## 151         Prop.Mam.Abdun2  ~            insitu2  6.225 -1.291  -1.291
## 159      Prop.Threat.Abund2  ~     Total.Animals2  8.210 -0.390  -0.390
## 162      Prop.Threat.Abund2  ~   Brillouin.Index2 10.253  0.319   0.319
## 163      Prop.Threat.Abund2  ~            insitu2  6.853 -1.469  -1.469
## 170 Mean.Sp.BodyMassXAbund2  ~        Attendance2 10.498  1.138   1.138
## 171 Mean.Sp.BodyMassXAbund2  ~     Total.Animals2 16.524  0.842   0.842
## 172 Mean.Sp.BodyMassXAbund2  ~       Sp.Richness2 11.403  0.667   0.667
## 173 Mean.Sp.BodyMassXAbund2  ~   Mean.Raup.Crick2 10.504  0.587   0.587
## 175 Mean.Sp.BodyMassXAbund2  ~            insitu2 13.900  3.592   3.592
## 185              X50km_Pop2  ~   Mean.Raup.Crick2  3.366  0.130   0.130
## 187              X50km_Pop2  ~            insitu2  4.447  0.829   0.829
## 195              X10km_Pop2  ~     Total.Animals2  4.544  0.181   0.181
## 196              X10km_Pop2  ~       Sp.Richness2  8.733  0.279   0.279
##     sepc.all sepc.nox
## 72    -0.050   -0.050
## 76     0.099    0.099
## 83     0.213    0.213
## 87     0.145    0.146
## 89    -0.115   -0.116
## 90     0.110    0.110
## 92     0.299    0.299
## 94     0.133    0.133
## 96     0.505    0.505
## 98    -0.313   -0.314
## 101    0.219    0.220
## 102    0.198    0.198
## 103    0.164    0.164
## 104    0.165    0.165
## 108   -0.208   -0.209
## 110    0.155    0.155
## 118    0.132    0.132
## 126    0.188    0.188
## 136   -0.427   -0.427
## 138   -0.253   -0.253
## 146   -0.745   -0.745
## 147   -0.463   -0.463
## 148   -0.649   -0.649
## 149   -0.235   -0.235
## 151   -1.285   -1.285
## 159   -0.390   -0.390
## 162    0.318    0.318
```

```
## 163   -1.462   -1.462
## 170    1.086    1.086
## 171    0.843    0.843
## 172    0.667    0.667
## 173    0.587    0.587
## 175    3.574    3.574
## 185    0.130    0.130
## 187    0.825    0.825
## 195    0.182    0.182
## 196    0.279    0.279
```

Model 3

Based on the results generated from the second model, the highest p-value relationship to be considered for removal is **insitu2 ~ X10km_Pop2** with a p-value of **0.836**. Therefore we decide to remove this pathway. The model summary, fit indices and modification indices were all generated for the model.

```
# In Situ SEM (Abundance)

# Model 3
# Removal of insitu2 ~ X10km_Pop2, p = 0.836

mod.IS3A <- 'Attendance2 ~ Zoo.Area.ha2 + Sp.Richness2 + Total.Animals2
+ Mam.Sp.Richness2 + Prop.Mam.Abdun2 + Prop.Threat.Abund2
+ Mean.Sp.BodyMassXAbund2 + Brillouin.Index2 + Mean.Raup.Crick2
+ X50km_Pop2 + X10km_Pop2

Total.Animals2 ~ Zoo.Area.ha2 + Sp.Richness2 + Mean.Sp.BodyMassXAbund2
Sp.Richness2 ~ Mam.Sp.Richness2 + Mean.Sp.BodyMassXAbund2
Mean.Raup.Crick2 ~ Mean.Sp.BodyMassXAbund2 + Zoo.Area.ha2
Brillouin.Index2 ~ Sp.Richness2 + Total.Animals2

insitu2 ~ Attendance2 + Zoo.Area.ha2 + Sp.Richness2 + Total.Animals2
+ Mam.Sp.Richness2 + Prop.Mam.Abdun2 + Prop.Threat.Abund2
+ Mean.Sp.BodyMassXAbund2 + Brillouin.Index2 + Mean.Raup.Crick2
+ X50km_Pop2'

# Fit model and generate model summary
mod.IS3A.fit <- sem(mod.IS3A , data = sem_in_situ_data, fixed.x=FALSE)
summary(mod.IS3A.fit, rsq = TRUE)
```

```
## lavaan (0.5-23.1097) converged normally after  52 iterations
##
##   Number of observations                           119
##
##   Estimator                                         ML
##   Minimum Function Test Statistic              102.059
##   Degrees of freedom                                26
##   P-value (Chi-square)                           0.000
##
## Parameter Estimates:
##
##   Information                                 Expected
##   Standard Errors                             Standard
```

```
##
## Regressions:
##                    Estimate  Std.Err  z-value  P(>|z|)
##   Attendance2 ~
##     Zoo.Area.ha2       0.029    0.079    0.373    0.709
##     Sp.Richness2      -0.413    0.184   -2.238    0.025
##     Total.Animals2     0.742    0.144    5.150    0.000
##     Mam.Sp.Rchnss2     0.057    0.102    0.561    0.575
##     Prop.Mam.Abdn2    -0.015    0.057   -0.261    0.794
##     Prp.Thrt.Abnd2    -0.068    0.053   -1.282    0.200
##     Mn.Sp.BdyMsXA2     0.250    0.082    3.052    0.002
##     Brilloun.Indx2     0.247    0.090    2.737    0.006
##     Mean.Rap.Crck2     0.087    0.058    1.488    0.137
##     X50km_Pop2         0.148    0.075    1.966    0.049
##     X10km_Pop2         0.335    0.085    3.963    0.000
##   Total.Animals2 ~
##     Zoo.Area.ha2       0.303    0.054    5.662    0.000
##     Sp.Richness2       0.748    0.045   16.498    0.000
##     Mn.Sp.BdyMsXA2    -0.262    0.050   -5.234    0.000
##   Sp.Richness2 ~
##     Mam.Sp.Rchnss2     0.861    0.051   16.866    0.000
##     Mn.Sp.BdyMsXA2    -0.213    0.051   -4.179    0.000
##   Mean.Raup.Crick2 ~
##     Mn.Sp.BdyMsXA2    -0.687    0.088   -7.810    0.000
##     Zoo.Area.ha2       0.350    0.088    3.973    0.000
##   Brillouin.Index2 ~
##     Sp.Richness2       1.573    0.094   16.708    0.000
##     Total.Animals2    -1.081    0.094  -11.496    0.000
##   insitu2 ~
##     Attendance2        0.598    0.114    5.250    0.000
##     Zoo.Area.ha2       0.198    0.101    1.969    0.049
##     Sp.Richness2       0.203    0.248    0.817    0.414
##     Total.Animals2    -0.337    0.208   -1.616    0.106
##     Mam.Sp.Rchnss2     0.194    0.132    1.471    0.141
##     Prop.Mam.Abdn2    -0.058    0.075   -0.779    0.436
##     Prp.Thrt.Abnd2     0.202    0.067    3.041    0.002
##     Mn.Sp.BdyMsXA2    -0.046    0.112   -0.411    0.681
##     Brilloun.Indx2    -0.199    0.122   -1.629    0.103
##     Mean.Rap.Crck2     0.099    0.078    1.278    0.201
##     X50km_Pop2        -0.012    0.084   -0.142    0.887
##
## Covariances:
##                            Estimate  Std.Err  z-value  P(>|z|)
##   Zoo.Area.ha2 ~~
##     Mam.Sp.Rchnss2             0.536    0.103    5.185    0.000
##     Prop.Mam.Abdn2             0.223    0.093    2.396    0.017
##     Prp.Thrt.Abnd2             0.071    0.091    0.776    0.438
##     Mn.Sp.BdyMsXA2             0.527    0.103    5.120    0.000
##     X50km_Pop2                -0.014    0.091   -0.155    0.876
##     X10km_Pop2                -0.099    0.091   -1.087    0.277
##   Mam.Sp.Richness2 ~~
##     Prop.Mam.Abdn2            -0.025    0.091   -0.273    0.785
##     Prp.Thrt.Abnd2             0.203    0.093    2.190    0.028
##     Mn.Sp.BdyMsXA2             0.219    0.093    2.357    0.018
```

```
##     X50km_Pop2                 0.363    0.097    3.752    0.000
##     X10km_Pop2                 0.421    0.099    4.262    0.000
##   Prop.Mam.Abdun2 ~~
##     Prp.Thrt.Abnd2            -0.195    0.093   -2.102    0.036
##     Mn.Sp.BdyMsXA2             0.453    0.100    4.531    0.000
##     X50km_Pop2                -0.290    0.095   -3.062    0.002
##     X10km_Pop2                -0.373    0.097   -3.839    0.000
##   Prop.Threat.Abund2 ~~
##     Mn.Sp.BdyMsXA2             0.092    0.091    1.012    0.312
##     X50km_Pop2                 0.035    0.091    0.382    0.703
##     X10km_Pop2                 0.278    0.094    2.948    0.003
##   Mean.Sp.BodyMassXAbund2 ~~
##     X50km_Pop2                -0.235    0.093   -2.516    0.012
##     X10km_Pop2                -0.209    0.093   -2.245    0.025
##   X50km_Pop2 ~~
##     X10km_Pop2                 0.738    0.113    6.511    0.000
##
## Variances:
##                    Estimate  Std.Err  z-value  P(>|z|)
##    .Attendance2       0.265    0.034    7.714    0.000
##    .Total.Animals2    0.198    0.026    7.714    0.000
##    .Sp.Richness2      0.292    0.038    7.714    0.000
##    .Mean.Rap.Crck2    0.655    0.085    7.714    0.000
##    .Brilloun.Indx2    0.274    0.036    7.714    0.000
##    .insitu2           0.463    0.060    7.714    0.000
##     Zoo.Area.ha2      0.992    0.129    7.714    0.000
##     Mam.Sp.Rchnss2    0.992    0.129    7.714    0.000
##     Prop.Mam.Abdn2    0.992    0.129    7.714    0.000
##     Prp.Thrt.Abnd2    0.992    0.129    7.714    0.000
##     Mn.Sp.BdyMsXA2    0.992    0.129    7.714    0.000
##     X50km_Pop2        0.992    0.129    7.714    0.000
##     X10km_Pop2        0.992    0.129    7.714    0.000
##
## R-Square:
##                    Estimate
##     Attendance2       0.707
##     Total.Animals2    0.801
##     Sp.Richness2      0.705
##     Mean.Rap.Crck2    0.339
##     Brilloun.Indx2    0.723
##     insitu2           0.528
```

```
# Generate fit indices
fitMeasures(mod.IS3A.fit, c("agfi", "rmr", "srmr", "rmsea", "cfi", "nnfi", "tli"))
```

```
##  agfi   rmr  srmr rmsea   cfi  nnfi   tli
## 0.646 0.071 0.072 0.157 0.909 0.800 0.800
```

```
# Generate modification indices
miIS3A <- modindices(mod.IS3A.fit)
print(miIS3A[miIS3A$mi > 3.0,])
```

```
##                        lhs op               rhs    mi    epc sepc.lv
## 71          Total.Animals2 ~~      Sp.Richness2 3.206 -0.049  -0.049
## 75            Sp.Richness2 ~~  Mean.Raup.Crick2 5.986  0.098   0.098
## 82          Total.Animals2  ~       Attendance2 5.117  0.223   0.223
```

```
## 86           Total.Animals2  ~   Mam.Sp.Richness2  3.206  0.145   0.145
## 88           Total.Animals2  ~ Prop.Threat.Abund2  7.695 -0.116  -0.116
## 89           Total.Animals2  ~         X50km_Pop2  5.874  0.110   0.110
## 91             Sp.Richness2  ~        Attendance2  7.188  0.313   0.313
## 93             Sp.Richness2  ~   Mean.Raup.Crick2  5.188  0.133   0.133
## 95             Sp.Richness2  ~            insitu2 13.944  0.501   0.501
## 97             Sp.Richness2  ~    Prop.Mam.Abdun2 30.563 -0.313  -0.313
## 100            Sp.Richness2  ~         X10km_Pop2 13.975  0.219   0.219
## 101        Mean.Raup.Crick2  ~        Attendance2  3.876  0.207   0.207
## 102        Mean.Raup.Crick2  ~     Total.Animals2  3.156  0.164   0.164
## 103        Mean.Raup.Crick2  ~       Sp.Richness2  3.980  0.165   0.165
## 107        Mean.Raup.Crick2  ~    Prop.Mam.Abdun2  6.144 -0.208  -0.208
## 109        Mean.Raup.Crick2  ~         X50km_Pop2  3.997  0.155   0.155
## 117        Brillouin.Index2  ~ Prop.Threat.Abund2  7.232  0.131   0.131
## 126            Zoo.Area.ha2  ~   Brillouin.Index2  4.076  0.188   0.188
## 136        Mam.Sp.Richness2  ~       Sp.Richness2  6.754 -0.427  -0.427
## 138        Mam.Sp.Richness2  ~   Brillouin.Index2  6.322 -0.253  -0.253
## 146         Prop.Mam.Abdun2  ~        Attendance2 10.515 -0.780  -0.780
## 147         Prop.Mam.Abdun2  ~     Total.Animals2 13.581 -0.462  -0.462
## 148         Prop.Mam.Abdun2  ~       Sp.Richness2 21.672 -0.649  -0.649
## 149         Prop.Mam.Abdun2  ~   Mean.Raup.Crick2  6.344 -0.235  -0.235
## 151         Prop.Mam.Abdun2  ~            insitu2  4.079 -0.842  -0.842
## 159      Prop.Threat.Abund2  ~     Total.Animals2  8.210 -0.390  -0.390
## 162      Prop.Threat.Abund2  ~   Brillouin.Index2 10.253  0.319   0.319
## 163      Prop.Threat.Abund2  ~            insitu2  3.072 -0.588  -0.588
## 170 Mean.Sp.BodyMassXAbund2  ~        Attendance2 10.498  1.138   1.138
## 171 Mean.Sp.BodyMassXAbund2  ~     Total.Animals2 16.524  0.842   0.842
## 172 Mean.Sp.BodyMassXAbund2  ~       Sp.Richness2 11.403  0.667   0.667
## 173 Mean.Sp.BodyMassXAbund2  ~   Mean.Raup.Crick2 10.504  0.587   0.587
## 175 Mean.Sp.BodyMassXAbund2  ~            insitu2 11.728  2.985   2.985
## 185              X50km_Pop2  ~   Mean.Raup.Crick2  3.366  0.130   0.130
## 195              X10km_Pop2  ~     Total.Animals2  4.544  0.181   0.181
## 196              X10km_Pop2  ~       Sp.Richness2  8.733  0.279   0.279
##     sepc.all sepc.nox
## 71    -0.050   -0.050
## 75     0.099    0.099
## 82     0.213    0.213
## 86     0.145    0.146
## 88    -0.115   -0.116
## 89     0.110    0.110
## 91     0.299    0.299
## 93     0.133    0.133
## 95     0.498    0.498
## 97    -0.313   -0.314
## 100    0.219    0.220
## 101    0.198    0.198
## 102    0.164    0.164
## 103    0.165    0.165
## 107   -0.208   -0.209
## 109    0.155    0.155
## 117    0.132    0.132
## 126    0.188    0.188
## 136   -0.427   -0.427
## 138   -0.253   -0.253
```

```
## 146   -0.745   -0.745
## 147   -0.463   -0.463
## 148   -0.649   -0.649
## 149   -0.235   -0.235
## 151   -0.838   -0.838
## 159   -0.390   -0.390
## 162    0.318    0.318
## 163   -0.584   -0.584
## 170    1.086    1.086
## 171    0.843    0.843
## 172    0.667    0.667
## 173    0.587    0.587
## 175    2.969    2.969
## 185    0.130    0.130
## 195    0.182    0.182
## 196    0.279    0.279
```

Model 4

Based on the results generated from the third model, the highest p-value relationship to be considered for removal is **insitu2 ~ X50km_Pop2** with a p-value of **0.887**. As **Attendance2 ~ X50km_Pop22** was unsupported in the previous models, we also remove **Attendance2 ~ X50km_Pop22** here. Therefore we decide to remove these pathways. The model summary, fit indices and modification indices were all generated for the model.

```
# In Situ SEM (Abundance)

# Model 4
# Removal of insitu2 ~ X50km_Pop2, p = 0.887
# Removal of Attendance2 ~ X50km_Pop22

mod.IS4A <- 'Attendance2 ~ Zoo.Area.ha2 + Sp.Richness2 + Total.Animals2
+ Mam.Sp.Richness2 + Prop.Mam.Abdun2 + Prop.Threat.Abund2
+ Mean.Sp.BodyMassXAbund2 + Brillouin.Index2 + Mean.Raup.Crick2
+ X10km_Pop2

Total.Animals2 ~ Zoo.Area.ha2 + Sp.Richness2 + Mean.Sp.BodyMassXAbund2
Sp.Richness2 ~ Mam.Sp.Richness2 + Mean.Sp.BodyMassXAbund2
Mean.Raup.Crick2 ~ Mean.Sp.BodyMassXAbund2 + Zoo.Area.ha2
Brillouin.Index2 ~ Sp.Richness2 + Total.Animals2

insitu2 ~ Attendance2 + Zoo.Area.ha2 + Sp.Richness2 + Total.Animals2
+ Mam.Sp.Richness2 + Prop.Mam.Abdun2 + Prop.Threat.Abund2
+ Mean.Sp.BodyMassXAbund2 + Brillouin.Index2 + Mean.Raup.Crick2'

# Fit model and generate model summary
mod.IS4A.fit <- sem(mod.IS4A , data = sem_in_situ_data, fixed.x=FALSE)
summary(mod.IS4A.fit, rsq = TRUE)
```

```
## lavaan (0.5-23.1097) converged normally after  45 iterations
##
##   Number of observations                           119
##
##   Estimator                                         ML
```

```
##   Minimum Function Test Statistic               91.859
##   Degrees of freedom                                22
##   P-value (Chi-square)                           0.000
##
## Parameter Estimates:
##
##   Information                                 Expected
##   Standard Errors                             Standard
##
## Regressions:
##                      Estimate  Std.Err  z-value  P(>|z|)
##   Attendance2 ~
##     Zoo.Area.ha2        0.038    0.080    0.471    0.637
##     Sp.Richness2       -0.479    0.187   -2.556    0.011
##     Total.Animals2      0.766    0.146    5.238    0.000
##     Mam.Sp.Rchnss2      0.093    0.103    0.901    0.367
##     Prop.Mam.Abdn2     -0.023    0.058   -0.392    0.695
##     Prp.Thrt.Abnd2     -0.093    0.052   -1.800    0.072
##     Mn.Sp.BdyMsXA2      0.246    0.083    2.973    0.003
##     Brilloun.Indx2      0.255    0.091    2.786    0.005
##     Mean.Rap.Crck2      0.110    0.059    1.864    0.062
##     X10km_Pop2          0.453    0.063    7.157    0.000
##   Total.Animals2 ~
##     Zoo.Area.ha2        0.303    0.054    5.662    0.000
##     Sp.Richness2        0.748    0.045   16.498    0.000
##     Mn.Sp.BdyMsXA2     -0.262    0.050   -5.234    0.000
##   Sp.Richness2 ~
##     Mam.Sp.Rchnss2      0.861    0.051   16.866    0.000
##     Mn.Sp.BdyMsXA2     -0.213    0.051   -4.179    0.000
##   Mean.Raup.Crick2 ~
##     Mn.Sp.BdyMsXA2     -0.687    0.088   -7.810    0.000
##     Zoo.Area.ha2        0.350    0.088    3.973    0.000
##   Brillouin.Index2 ~
##     Sp.Richness2        1.573    0.094   16.708    0.000
##     Total.Animals2     -1.081    0.094  -11.496    0.000
##   insitu2 ~
##     Attendance2         0.590    0.100    5.914    0.000
##     Zoo.Area.ha2        0.200    0.101    1.991    0.046
##     Sp.Richness2        0.202    0.248    0.813    0.416
##     Total.Animals2     -0.334    0.205   -1.628    0.103
##     Mam.Sp.Rchnss2      0.191    0.131    1.461    0.144
##     Prop.Mam.Abdn2     -0.058    0.075   -0.774    0.439
##     Prp.Thrt.Abnd2      0.202    0.066    3.049    0.002
##     Mn.Sp.BdyMsXA2     -0.044    0.110   -0.403    0.687
##     Brilloun.Indx2     -0.198    0.122   -1.621    0.105
##     Mean.Rap.Crck2      0.098    0.078    1.263    0.207
##
## Covariances:
##                              Estimate  Std.Err  z-value  P(>|z|)
##   Zoo.Area.ha2 ~~
##     Mam.Sp.Rchnss2              0.536    0.103    5.185    0.000
##     Prop.Mam.Abdn2              0.223    0.093    2.396    0.017
##     Prp.Thrt.Abnd2              0.071    0.091    0.776    0.438
##     Mn.Sp.BdyMsXA2              0.527    0.103    5.120    0.000
```

```
##     X10km_Pop2             -0.099    0.091   -1.087    0.277
##   Mam.Sp.Richness2 ~~
##     Prop.Mam.Abdn2         -0.025    0.091   -0.273    0.785
##     Prp.Thrt.Abnd2          0.203    0.093    2.190    0.028
##     Mn.Sp.BdyMsXA2          0.219    0.093    2.357    0.018
##     X10km_Pop2              0.421    0.099    4.262    0.000
##   Prop.Mam.Abdun2 ~~
##     Prp.Thrt.Abnd2         -0.195    0.093   -2.102    0.036
##     Mn.Sp.BdyMsXA2          0.453    0.100    4.531    0.000
##     X10km_Pop2             -0.373    0.097   -3.839    0.000
##   Prop.Threat.Abund2 ~~
##     Mn.Sp.BdyMsXA2          0.092    0.091    1.012    0.312
##     X10km_Pop2              0.278    0.094    2.948    0.003
##   Mean.Sp.BodyMassXAbund2 ~~
##     X10km_Pop2             -0.209    0.093   -2.245    0.025
##
## Variances:
##                    Estimate  Std.Err  z-value  P(>|z|)
##    .Attendance2       0.273    0.035    7.714    0.000
##    .Total.Animals2    0.198    0.026    7.714    0.000
##    .Sp.Richness2      0.292    0.038    7.714    0.000
##    .Mean.Rap.Crck2    0.655    0.085    7.714    0.000
##    .Brilloun.Indx2    0.274    0.036    7.714    0.000
##    .insitu2           0.463    0.060    7.714    0.000
##     Zoo.Area.ha2      0.992    0.129    7.714    0.000
##     Mam.Sp.Rchnss2    0.992    0.129    7.714    0.000
##     Prop.Mam.Abdn2    0.992    0.129    7.714    0.000
##     Prp.Thrt.Abnd2    0.992    0.129    7.714    0.000
##     Mn.Sp.BdyMsXA2    0.992    0.129    7.714    0.000
##     X10km_Pop2        0.992    0.129    7.714    0.000
##
## R-Square:
##                    Estimate
##     Attendance2       0.698
##     Total.Animals2    0.801
##     Sp.Richness2      0.705
##     Mean.Rap.Crck2    0.339
##     Brilloun.Indx2    0.723
##     insitu2           0.531
```

```
# Generate fit indices
fitMeasures(mod.IS4A.fit, c("agfi", "rmr", "srmr", "rmsea", "cfi", "nnfi", "tli"))
```

```
##  agfi   rmr  srmr rmsea   cfi  nnfi   tli
## 0.646 0.073 0.073 0.163 0.915 0.804 0.804
```

```
# Generate modification indices
miIS4A <- modindices(mod.IS4A.fit)
print(miIS4A[miIS4A$mi > 3.0,])
```

```
##                  lhs op                rhs    mi    epc sepc.lv
## 62         Total.Animals2 ~~       Sp.Richness2 3.206 -0.049  -0.049
## 66           Sp.Richness2 ~~   Mean.Raup.Crick2 5.986  0.098   0.098
## 73         Total.Animals2  ~        Attendance2 4.224  0.200   0.200
## 77         Total.Animals2  ~   Mam.Sp.Richness2 3.206  0.145   0.145
## 79         Total.Animals2  ~ Prop.Threat.Abund2 7.695 -0.116  -0.116
```

```
## 81             Sp.Richness2  ~        Attendance2 10.568  0.378   0.378
## 83             Sp.Richness2  ~   Mean.Raup.Crick2  5.187  0.133   0.133
## 85             Sp.Richness2  ~            insitu2 16.450  0.534   0.534
## 87             Sp.Richness2  ~    Prop.Mam.Abdun2 30.563 -0.313  -0.313
## 89             Sp.Richness2  ~        X10km_Pop2 13.975  0.219   0.219
## 91         Mean.Raup.Crick2  ~     Total.Animals2  3.156  0.164   0.164
## 92         Mean.Raup.Crick2  ~       Sp.Richness2  3.980  0.165   0.165
## 96         Mean.Raup.Crick2  ~    Prop.Mam.Abdun2  6.144 -0.208  -0.208
## 105        Brillouin.Index2  ~ Prop.Threat.Abund2  7.232  0.131   0.131
## 113             Zoo.Area.ha2  ~   Brillouin.Index2  3.423  0.175   0.175
## 122        Mam.Sp.Richness2  ~       Sp.Richness2  7.427 -0.448  -0.448
## 124        Mam.Sp.Richness2  ~   Brillouin.Index2  6.750 -0.262  -0.262
## 131          Prop.Mam.Abdun2  ~        Attendance2  9.482 -0.743  -0.743
## 132          Prop.Mam.Abdun2  ~     Total.Animals2 13.552 -0.462  -0.462
## 133          Prop.Mam.Abdun2  ~       Sp.Richness2 21.450 -0.646  -0.646
## 134          Prop.Mam.Abdun2  ~   Mean.Raup.Crick2  6.479 -0.237  -0.237
## 143       Prop.Threat.Abund2  ~     Total.Animals2  8.349 -0.408  -0.408
## 146       Prop.Threat.Abund2  ~   Brillouin.Index2 13.296  0.376   0.376
## 147       Prop.Threat.Abund2  ~            insitu2  7.287 -1.233  -1.233
## 153 Mean.Sp.BodyMassXAbund2  ~        Attendance2  9.756  1.147   1.147
## 154 Mean.Sp.BodyMassXAbund2  ~     Total.Animals2 18.717  0.934   0.934
## 155 Mean.Sp.BodyMassXAbund2  ~       Sp.Richness2 15.249  0.796   0.796
## 156 Mean.Sp.BodyMassXAbund2  ~   Mean.Raup.Crick2  7.777  0.536   0.536
## 158 Mean.Sp.BodyMassXAbund2  ~            insitu2  6.758  2.181   2.181
## 164               X10km_Pop2  ~        Attendance2  4.104  0.449   0.449
## 165               X10km_Pop2  ~     Total.Animals2  6.848  0.302   0.302
## 166               X10km_Pop2  ~       Sp.Richness2  6.043  0.315   0.315
##     sepc.all sepc.nox
## 62    -0.050   -0.050
## 66     0.099    0.099
## 73     0.190    0.190
## 77     0.145    0.146
## 79    -0.115   -0.116
## 81     0.362    0.362
## 83     0.133    0.133
## 85     0.533    0.533
## 87    -0.313   -0.314
## 89     0.219    0.220
## 91     0.164    0.164
## 92     0.165    0.165
## 96    -0.208   -0.209
## 105    0.132    0.132
## 113    0.175    0.175
## 122   -0.448   -0.448
## 124   -0.262   -0.262
## 131   -0.710   -0.710
## 132   -0.463   -0.463
## 133   -0.646   -0.646
## 134   -0.237   -0.237
## 143   -0.408   -0.408
## 146    0.375    0.375
## 147   -1.229   -1.229
## 153    1.096    1.096
## 154    0.935    0.935
```

```
## 155    0.796    0.796
## 156    0.536    0.536
## 158    2.174    2.174
## 164    0.429    0.429
## 165    0.302    0.302
## 166    0.315    0.315
```

Model 5

Based on the results generated from the fourth model, the highest p-value relationship to be considered for removal is **Attendance2** ~ **Prop.Mam.Abdn2** with a p-value of **0.695**. Therefore we decide to remove this pathway. The model summary, fit indices and modification indices were all generated for the model.

```
# In Situ SEM (Abundance)

# Model 5
# Removal of Attendance2 ~ Prop.Mam.Abdn2, p = 0.695

mod.IS5A <- 'Attendance2 ~ Zoo.Area.ha2 + Sp.Richness2 + Total.Animals2
+ Mam.Sp.Richness2 + Prop.Threat.Abund2
+ Mean.Sp.BodyMassXAbund2 + Brillouin.Index2 + Mean.Raup.Crick2
+ X10km_Pop2

Total.Animals2 ~ Zoo.Area.ha2 + Sp.Richness2 + Mean.Sp.BodyMassXAbund2
Sp.Richness2 ~ Mam.Sp.Richness2 + Mean.Sp.BodyMassXAbund2
Mean.Raup.Crick2 ~ Mean.Sp.BodyMassXAbund2 + Zoo.Area.ha2
Brillouin.Index2 ~ Sp.Richness2 + Total.Animals2

insitu2 ~ Attendance2 + Zoo.Area.ha2 + Sp.Richness2 + Total.Animals2
+ Mam.Sp.Richness2 + Prop.Mam.Abdun2 + Prop.Threat.Abund2
+ Mean.Sp.BodyMassXAbund2 + Brillouin.Index2 + Mean.Raup.Crick2'

# Fit model and generate model summary
mod.IS5A.fit <- sem(mod.IS5A , data = sem_in_situ_data, fixed.x=FALSE)
summary(mod.IS5A.fit, rsq = TRUE)
```

```
## lavaan (0.5-23.1097) converged normally after  45 iterations
##
##   Number of observations                           119
##
##   Estimator                                         ML
##   Minimum Function Test Statistic               91.971
##   Degrees of freedom                                23
##   P-value (Chi-square)                           0.000
##
## Parameter Estimates:
##
##   Information                                 Expected
##   Standard Errors                             Standard
##
## Regressions:
##                    Estimate  Std.Err  z-value  P(>|z|)
##   Attendance2 ~
##     Zoo.Area.ha2        0.035    0.080    0.434    0.664
```

```
##     Sp.Richness2       -0.458    0.187   -2.443    0.015
##     Total.Animals2      0.767    0.146    5.241    0.000
##     Mam.Sp.Rchnss2      0.079    0.103    0.767    0.443
##     Prp.Thrt.Abnd2     -0.087    0.051   -1.720    0.085
##     Mn.Sp.BdyMsXA2      0.243    0.079    3.080    0.002
##     Brilloun.Indx2      0.249    0.092    2.719    0.007
##     Mean.Rap.Crck2      0.114    0.059    1.922    0.055
##     X10km_Pop2          0.454    0.061    7.390    0.000
##   Total.Animals2 ~
##     Zoo.Area.ha2        0.303    0.054    5.662    0.000
##     Sp.Richness2        0.748    0.045   16.498    0.000
##     Mn.Sp.BdyMsXA2     -0.262    0.050   -5.234    0.000
##   Sp.Richness2 ~
##     Mam.Sp.Rchnss2      0.861    0.051   16.866    0.000
##     Mn.Sp.BdyMsXA2     -0.213    0.051   -4.179    0.000
##   Mean.Raup.Crick2 ~
##     Mn.Sp.BdyMsXA2     -0.687    0.088   -7.810    0.000
##     Zoo.Area.ha2        0.350    0.088    3.973    0.000
##   Brillouin.Index2 ~
##     Sp.Richness2        1.573    0.094   16.708    0.000
##     Total.Animals2     -1.081    0.094  -11.496    0.000
##   insitu2 ~
##     Attendance2         0.590    0.100    5.920    0.000
##     Zoo.Area.ha2        0.200    0.101    1.990    0.047
##     Sp.Richness2        0.202    0.248    0.814    0.415
##     Total.Animals2     -0.334    0.205   -1.628    0.103
##     Mam.Sp.Rchnss2      0.191    0.130    1.466    0.143
##     Prop.Mam.Abdn2     -0.058    0.074   -0.777    0.437
##     Prp.Thrt.Abnd2      0.202    0.066    3.050    0.002
##     Mn.Sp.BdyMsXA2     -0.044    0.109   -0.403    0.687
##     Brilloun.Indx2     -0.198    0.122   -1.623    0.105
##     Mean.Rap.Crck2      0.098    0.078    1.262    0.207
##
## Covariances:
##                            Estimate  Std.Err  z-value  P(>|z|)
##   Zoo.Area.ha2 ~~
##     Mam.Sp.Rchnss2             0.536    0.103    5.185    0.000
##     Prp.Thrt.Abnd2             0.071    0.091    0.776    0.438
##     Mn.Sp.BdyMsXA2             0.527    0.103    5.120    0.000
##     X10km_Pop2                -0.099    0.091   -1.087    0.277
##     Prop.Mam.Abdn2             0.223    0.093    2.396    0.017
##   Mam.Sp.Richness2 ~~
##     Prp.Thrt.Abnd2             0.203    0.093    2.190    0.028
##     Mn.Sp.BdyMsXA2             0.219    0.093    2.357    0.018
##     X10km_Pop2                 0.421    0.099    4.262    0.000
##     Prop.Mam.Abdn2            -0.025    0.091   -0.273    0.785
##   Prop.Threat.Abund2 ~~
##     Mn.Sp.BdyMsXA2             0.092    0.091    1.012    0.312
##     X10km_Pop2                 0.278    0.094    2.948    0.003
##     Prop.Mam.Abdn2            -0.195    0.093   -2.102    0.036
##   Mean.Sp.BodyMassXAbund2 ~~
##     X10km_Pop2                -0.209    0.093   -2.245    0.025
##     Prop.Mam.Abdn2             0.453    0.100    4.531    0.000
##   X10km_Pop2 ~~
```

```
##     Prop.Mam.Abdn2             -0.373    0.097   -3.839    0.000
##
## Variances:
##                    Estimate  Std.Err  z-value  P(>|z|)
##    .Attendance2       0.273    0.035    7.714    0.000
##    .Total.Animals2    0.198    0.026    7.714    0.000
##    .Sp.Richness2      0.292    0.038    7.714    0.000
##    .Mean.Rap.Crck2    0.655    0.085    7.714    0.000
##    .Brilloun.Indx2    0.274    0.036    7.714    0.000
##    .insitu2           0.463    0.060    7.714    0.000
##     Zoo.Area.ha2      0.992    0.129    7.714    0.000
##     Mam.Sp.Rchnss2    0.992    0.129    7.714    0.000
##     Prp.Thrt.Abnd2    0.992    0.129    7.714    0.000
##     Mn.Sp.BdyMsXA2    0.992    0.129    7.714    0.000
##     X10km_Pop2        0.992    0.129    7.714    0.000
##     Prop.Mam.Abdn2    0.992    0.129    7.714    0.000
##
## R-Square:
##                    Estimate
##     Attendance2       0.698
##     Total.Animals2    0.801
##     Sp.Richness2      0.705
##     Mean.Rap.Crck2    0.339
##     Brilloun.Indx2    0.723
##     insitu2           0.530
```

```
# Generate fit indices
fitMeasures(mod.IS5A.fit, c("agfi", "rmr", "srmr", "rmsea", "cfi", "nnfi", "tli"))
```

```
##  agfi   rmr  srmr rmsea   cfi  nnfi   tli
## 0.661 0.073 0.073 0.159 0.916 0.815 0.815
```

```
# Generate modification indices
miIS5A <- modindices(mod.IS5A.fit)
print(miIS5A[miIS5A$mi > 3.0,])
```

```
##                       lhs op                 rhs     mi    epc sepc.lv
## 61          Total.Animals2 ~~       Sp.Richness2  3.206 -0.049  -0.049
## 65            Sp.Richness2 ~~   Mean.Raup.Crick2  5.986  0.098   0.098
## 73          Total.Animals2  ~        Attendance2  4.017  0.198   0.198
## 77          Total.Animals2  ~   Mam.Sp.Richness2  3.206  0.145   0.145
## 78          Total.Animals2  ~ Prop.Threat.Abund2  7.695 -0.116  -0.116
## 81            Sp.Richness2  ~        Attendance2  9.210  0.355   0.355
## 83            Sp.Richness2  ~   Mean.Raup.Crick2  5.188  0.133   0.133
## 85            Sp.Richness2  ~            insitu2 15.327  0.518   0.518
## 88            Sp.Richness2  ~         X10km_Pop2 13.975  0.219   0.219
## 89            Sp.Richness2  ~     Prop.Mam.Abdun2 30.563 -0.313  -0.313
## 91        Mean.Raup.Crick2  ~      Total.Animals2  3.156  0.164   0.164
## 92        Mean.Raup.Crick2  ~        Sp.Richness2  3.980  0.165   0.165
## 98        Mean.Raup.Crick2  ~     Prop.Mam.Abdun2  6.144 -0.208  -0.208
## 104       Brillouin.Index2  ~ Prop.Threat.Abund2  7.232  0.131   0.131
## 113           Zoo.Area.ha2  ~   Brillouin.Index2  3.423  0.175   0.175
## 122       Mam.Sp.Richness2  ~       Sp.Richness2  7.427 -0.448  -0.448
## 124       Mam.Sp.Richness2  ~   Brillouin.Index2  6.750 -0.262  -0.262
## 131     Prop.Threat.Abund2  ~        Attendance2  3.141 -0.450  -0.450
## 132     Prop.Threat.Abund2  ~      Total.Animals2  8.349 -0.408  -0.408
```

```
## 135      Prop.Threat.Abund2  ~   Brillouin.Index2 13.296  0.376   0.376
## 136      Prop.Threat.Abund2  ~            insitu2  7.424 -1.176  -1.176
## 142 Mean.Sp.BodyMassXAbund2  ~       Attendance2  5.845  0.596   0.596
## 143 Mean.Sp.BodyMassXAbund2  ~     Total.Animals2 18.717  0.934   0.934
## 144 Mean.Sp.BodyMassXAbund2  ~       Sp.Richness2 15.249  0.796   0.796
## 145 Mean.Sp.BodyMassXAbund2  ~   Mean.Raup.Crick2  7.777  0.536   0.536
## 147 Mean.Sp.BodyMassXAbund2  ~            insitu2  3.289  0.851   0.851
## 153              X10km_Pop2  ~       Attendance2  3.316  0.367   0.367
## 154              X10km_Pop2  ~     Total.Animals2  6.848  0.302   0.302
## 155              X10km_Pop2  ~       Sp.Richness2  6.043  0.315   0.315
## 164          Prop.Mam.Abdun2  ~       Attendance2  3.635 -0.235  -0.235
## 165          Prop.Mam.Abdun2  ~     Total.Animals2 13.552 -0.462  -0.462
## 166          Prop.Mam.Abdun2  ~       Sp.Richness2 21.450 -0.646  -0.646
## 167          Prop.Mam.Abdun2  ~   Mean.Raup.Crick2  6.479 -0.237  -0.237
##     sepc.all sepc.nox
## 61    -0.050   -0.050
## 65     0.099    0.099
## 73     0.189    0.189
## 77     0.145    0.146
## 78    -0.115   -0.116
## 81     0.340    0.340
## 83     0.133    0.133
## 85     0.516    0.516
## 88     0.219    0.220
## 89    -0.313   -0.314
## 91     0.164    0.164
## 92     0.165    0.165
## 98    -0.208   -0.209
## 104    0.132    0.132
## 113    0.175    0.175
## 122   -0.448   -0.448
## 124   -0.262   -0.262
## 131   -0.430   -0.430
## 132   -0.408   -0.408
## 135    0.375    0.375
## 136   -1.172   -1.172
## 142    0.570    0.570
## 143    0.935    0.935
## 144    0.796    0.796
## 145    0.536    0.536
## 147    0.848    0.848
## 153    0.351    0.351
## 154    0.302    0.302
## 155    0.315    0.315
## 164   -0.224   -0.224
## 165   -0.463   -0.463
## 166   -0.646   -0.646
## 167   -0.237   -0.237
```

Model 6

Based on the results generated from the fifth model, the highest p-value relationship to be considered for removal is **insitu2** ~ **Mn.Sp.BdyMsXA2** with a p-value of **0.687**. Therefore we decide to remove this

pathway. The model summary, fit indices and modification indices were all generated for the model.

```
# In Situ SEM (Abundance)

# Model 6
# Removal of insitu2 ~ Mn.Sp.BdyMsXA2, p = 0.687

mod.IS6A <- 'Attendance2 ~ Zoo.Area.ha2 + Sp.Richness2 + Total.Animals2
+ Mam.Sp.Richness2 + Prop.Threat.Abund2
+ Mean.Sp.BodyMassXAbund2 + Brillouin.Index2 + Mean.Raup.Crick2
+ X10km_Pop2

Total.Animals2 ~ Zoo.Area.ha2 + Sp.Richness2 + Mean.Sp.BodyMassXAbund2
Sp.Richness2 ~ Mam.Sp.Richness2 + Mean.Sp.BodyMassXAbund2
Mean.Raup.Crick2 ~ Mean.Sp.BodyMassXAbund2 + Zoo.Area.ha2
Brillouin.Index2 ~ Sp.Richness2 + Total.Animals2

insitu2 ~ Attendance2 + Zoo.Area.ha2 + Sp.Richness2 + Total.Animals2
+ Mam.Sp.Richness2 + Prop.Mam.Abdun2 + Prop.Threat.Abund2
+ Brillouin.Index2 + Mean.Raup.Crick2'

# Fit model and generate model summary
mod.IS6A.fit <- sem(mod.IS6A , data = sem_in_situ_data, fixed.x=FALSE)
summary(mod.IS6A.fit, rsq = TRUE)
```

```
## lavaan (0.5-23.1097) converged normally after  45 iterations
##
##   Number of observations                           119
##
##   Estimator                                         ML
##   Minimum Function Test Statistic               92.152
##   Degrees of freedom                                24
##   P-value (Chi-square)                           0.000
##
## Parameter Estimates:
##
##   Information                                 Expected
##   Standard Errors                             Standard
##
## Regressions:
##                    Estimate  Std.Err  z-value  P(>|z|)
##   Attendance2 ~
##     Zoo.Area.ha2      0.035    0.080    0.434    0.664
##     Sp.Richness2     -0.458    0.187   -2.443    0.015
##     Total.Animals2    0.767    0.146    5.241    0.000
##     Mam.Sp.Rchnss2    0.079    0.103    0.767    0.443
##     Prp.Thrt.Abnd2   -0.087    0.051   -1.720    0.085
##     Mn.Sp.BdyMsXA2    0.243    0.079    3.080    0.002
##     Brilloun.Indx2    0.249    0.092    2.719    0.007
##     Mean.Rap.Crck2    0.114    0.059    1.922    0.055
##     X10km_Pop2        0.454    0.061    7.390    0.000
##   Total.Animals2 ~
##     Zoo.Area.ha2      0.303    0.054    5.662    0.000
##     Sp.Richness2      0.748    0.045   16.498    0.000
##     Mn.Sp.BdyMsXA2   -0.262    0.050   -5.234    0.000
```

```
##   Sp.Richness2 ~
##     Mam.Sp.Rchnss2      0.861    0.051   16.866    0.000
##     Mn.Sp.BdyMsXA2     -0.213    0.051   -4.179    0.000
##   Mean.Raup.Crick2 ~
##     Mn.Sp.BdyMsXA2     -0.687    0.088   -7.810    0.000
##     Zoo.Area.ha2        0.350    0.088    3.973    0.000
##   Brillouin.Index2 ~
##     Sp.Richness2        1.573    0.094   16.708    0.000
##     Total.Animals2     -1.081    0.094  -11.496    0.000
##   insitu2 ~
##     Attendance2         0.579    0.098    5.917    0.000
##     Zoo.Area.ha2        0.176    0.082    2.136    0.033
##     Sp.Richness2        0.190    0.248    0.769    0.442
##     Total.Animals2     -0.305    0.196   -1.557    0.119
##     Mam.Sp.Rchnss2      0.187    0.130    1.443    0.149
##     Prop.Mam.Abdn2     -0.062    0.070   -0.894    0.371
##     Prp.Thrt.Abnd2      0.203    0.066    3.092    0.002
##     Brilloun.Indx2     -0.197    0.122   -1.619    0.106
##     Mean.Rap.Crck2      0.114    0.067    1.701    0.089
##
## Covariances:
##                            Estimate  Std.Err  z-value  P(>|z|)
##   Zoo.Area.ha2 ~~
##     Mam.Sp.Rchnss2            0.536    0.103    5.185    0.000
##     Prp.Thrt.Abnd2            0.071    0.091    0.776    0.438
##     Mn.Sp.BdyMsXA2            0.527    0.103    5.120    0.000
##     X10km_Pop2               -0.099    0.091   -1.087    0.277
##     Prop.Mam.Abdn2            0.223    0.093    2.396    0.017
##   Mam.Sp.Richness2 ~~
##     Prp.Thrt.Abnd2            0.203    0.093    2.190    0.028
##     Mn.Sp.BdyMsXA2            0.219    0.093    2.357    0.018
##     X10km_Pop2                0.421    0.099    4.262    0.000
##     Prop.Mam.Abdn2           -0.025    0.091   -0.273    0.785
##   Prop.Threat.Abund2 ~~
##     Mn.Sp.BdyMsXA2            0.092    0.091    1.012    0.312
##     X10km_Pop2                0.278    0.094    2.948    0.003
##     Prop.Mam.Abdn2           -0.195    0.093   -2.102    0.036
##   Mean.Sp.BodyMassXAbund2 ~~
##     X10km_Pop2               -0.209    0.093   -2.245    0.025
##     Prop.Mam.Abdn2            0.453    0.100    4.531    0.000
##   X10km_Pop2 ~~
##     Prop.Mam.Abdn2           -0.373    0.097   -3.839    0.000
##
## Variances:
##                    Estimate  Std.Err  z-value  P(>|z|)
##    .Attendance2       0.273    0.035    7.714    0.000
##    .Total.Animals2    0.198    0.026    7.714    0.000
##    .Sp.Richness2      0.292    0.038    7.714    0.000
##    .Mean.Rap.Crck2    0.655    0.085    7.714    0.000
##    .Brilloun.Indx2    0.274    0.036    7.714    0.000
##    .insitu2           0.463    0.060    7.714    0.000
##     Zoo.Area.ha2      0.992    0.129    7.714    0.000
##     Mam.Sp.Rchnss2    0.992    0.129    7.714    0.000
##     Prp.Thrt.Abnd2    0.992    0.129    7.714    0.000
```

```
##     Mn.Sp.BdyMsXA2    0.992    0.129    7.714    0.000
##     X10km_Pop2        0.992    0.129    7.714    0.000
##     Prop.Mam.Abdn2    0.992    0.129    7.714    0.000
##
## R-Square:
##                    Estimate
##     Attendance2       0.698
##     Total.Animals2    0.801
##     Sp.Richness2      0.705
##     Mean.Rap.Crck2    0.339
##     Brilloun.Indx2    0.723
##     insitu2           0.528
```

```
# Generate fit indices
fitMeasures(mod.IS6A.fit, c("agfi", "rmr", "srmr", "rmsea", "cfi", "nnfi", "tli"))
```

```
##  agfi   rmr  srmr rmsea   cfi  nnfi   tli
## 0.674 0.073 0.074 0.154 0.917 0.824 0.824
```

```
# Generate modification indices
miIS6A <- modindices(mod.IS6A.fit)
print(miIS6A[miIS6A$mi > 3.0,])
```

```
##                           lhs op                rhs     mi    epc sepc.lv
## 60             Total.Animals2 ~~       Sp.Richness2  3.206 -0.049  -0.049
## 64               Sp.Richness2 ~~   Mean.Raup.Crick2  5.986  0.098   0.098
## 72             Total.Animals2  ~        Attendance2  4.017  0.198   0.198
## 76             Total.Animals2  ~   Mam.Sp.Richness2  3.206  0.145   0.145
## 77             Total.Animals2  ~ Prop.Threat.Abund2  7.695 -0.116  -0.116
## 80               Sp.Richness2  ~        Attendance2  9.210  0.355   0.355
## 82               Sp.Richness2  ~   Mean.Raup.Crick2  5.188  0.133   0.133
## 84               Sp.Richness2  ~            insitu2 12.224  0.426   0.426
## 87               Sp.Richness2  ~         X10km_Pop2 13.975  0.219   0.219
## 88               Sp.Richness2  ~     Prop.Mam.Abdun2 30.563 -0.313  -0.313
## 90           Mean.Raup.Crick2  ~     Total.Animals2  3.156  0.164   0.164
## 91           Mean.Raup.Crick2  ~       Sp.Richness2  3.980  0.165   0.165
## 97           Mean.Raup.Crick2  ~     Prop.Mam.Abdun2  6.144 -0.208  -0.208
## 103          Brillouin.Index2  ~ Prop.Threat.Abund2  7.232  0.131   0.131
## 113              Zoo.Area.ha2  ~   Brillouin.Index2  3.423  0.175   0.175
## 122          Mam.Sp.Richness2  ~       Sp.Richness2  7.427 -0.448  -0.448
## 124          Mam.Sp.Richness2  ~   Brillouin.Index2  6.750 -0.262  -0.262
## 131        Prop.Threat.Abund2  ~        Attendance2  3.141 -0.450  -0.450
## 132        Prop.Threat.Abund2  ~     Total.Animals2  8.349 -0.408  -0.408
## 135        Prop.Threat.Abund2  ~   Brillouin.Index2 13.296  0.376   0.376
## 136        Prop.Threat.Abund2  ~            insitu2  6.179 -0.947  -0.947
## 142 Mean.Sp.BodyMassXAbund2  ~        Attendance2  5.845  0.596   0.596
## 143 Mean.Sp.BodyMassXAbund2  ~     Total.Animals2 18.717  0.934   0.934
## 144 Mean.Sp.BodyMassXAbund2  ~       Sp.Richness2 15.249  0.796   0.796
## 145 Mean.Sp.BodyMassXAbund2  ~   Mean.Raup.Crick2  7.777  0.536   0.536
## 153                X10km_Pop2  ~        Attendance2  3.316  0.367   0.367
## 154                X10km_Pop2  ~     Total.Animals2  6.848  0.302   0.302
## 155                X10km_Pop2  ~       Sp.Richness2  6.043  0.315   0.315
## 164           Prop.Mam.Abdun2  ~        Attendance2  3.635 -0.235  -0.235
## 165           Prop.Mam.Abdun2  ~     Total.Animals2 13.552 -0.462  -0.462
## 166           Prop.Mam.Abdun2  ~       Sp.Richness2 21.450 -0.646  -0.646
## 167           Prop.Mam.Abdun2  ~   Mean.Raup.Crick2  6.479 -0.237  -0.237
```

```
##     sepc.all sepc.nox
## 60    -0.050   -0.050
## 64     0.099    0.099
## 72     0.189    0.189
## 76     0.145    0.146
## 77    -0.115   -0.116
## 80     0.340    0.340
## 82     0.133    0.133
## 84     0.424    0.424
## 87     0.219    0.220
## 88    -0.313   -0.314
## 90     0.164    0.164
## 91     0.165    0.165
## 97    -0.208   -0.209
## 103    0.132    0.132
## 113    0.175    0.175
## 122   -0.448   -0.448
## 124   -0.262   -0.262
## 131   -0.430   -0.430
## 132   -0.408   -0.408
## 135    0.375    0.375
## 136   -0.942   -0.942
## 142    0.570    0.570
## 143    0.935    0.935
## 144    0.796    0.796
## 145    0.536    0.536
## 153    0.351    0.351
## 154    0.302    0.302
## 155    0.315    0.315
## 164   -0.224   -0.224
## 165   -0.463   -0.463
## 166   -0.646   -0.646
## 167   -0.237   -0.237
```

Model 7

Based on the results generated from the sixth model, the highest p-value relationship to be considered for removal is **Attendance2** ~ **Zoo.Area.ha2** with a p-value of **0.664**. Therefore we decide to remove this pathway. The model summary, fit indices and modification indices were all generated for the model.

```
# In Situ SEM (Abundance)

# Model 7
# Removal of Attendance2 ~ Zoo.Area.ha2, p = 0.664

mod.IS7A <- 'Attendance2 ~ Sp.Richness2 + Total.Animals2
+ Mam.Sp.Richness2 + Prop.Threat.Abund2
+ Mean.Sp.BodyMassXAbund2 + Brillouin.Index2 + Mean.Raup.Crick2
+ X10km_Pop2

Total.Animals2 ~ Zoo.Area.ha2 + Sp.Richness2 + Mean.Sp.BodyMassXAbund2
Sp.Richness2 ~ Mam.Sp.Richness2 + Mean.Sp.BodyMassXAbund2
Mean.Raup.Crick2 ~ Mean.Sp.BodyMassXAbund2 + Zoo.Area.ha2
```

```
Brillouin.Index2 ~ Sp.Richness2 + Total.Animals2

insitu2 ~ Attendance2 + Zoo.Area.ha2 + Sp.Richness2 + Total.Animals2
+ Mam.Sp.Richness2 + Prop.Mam.Abdun2 + Prop.Threat.Abund2
+ Brillouin.Index2 + Mean.Raup.Crick2'

# Fit model and generate model summary
mod.IS7A.fit <- sem(mod.IS7A , data = sem_in_situ_data, fixed.x=FALSE)
summary(mod.IS7A.fit, rsq = TRUE)
```

```
## lavaan (0.5-23.1097) converged normally after  49 iterations
##
##   Number of observations                           119
##
##   Estimator                                         ML
##   Minimum Function Test Statistic               92.338
##   Degrees of freedom                                25
##   P-value (Chi-square)                           0.000
##
## Parameter Estimates:
##
##   Information                                 Expected
##   Standard Errors                             Standard
##
## Regressions:
##                      Estimate  Std.Err  z-value  P(>|z|)
##   Attendance2 ~
##     Sp.Richness2       -0.473    0.184   -2.561    0.010
##     Total.Animals2      0.790    0.140    5.661    0.000
##     Mam.Sp.Rchnss2      0.091    0.097    0.932    0.351
##     Prp.Thrt.Abnd2     -0.085    0.051   -1.663    0.096
##     Mn.Sp.BdyMsXA2      0.261    0.066    3.962    0.000
##     Brilloun.Indx2      0.251    0.092    2.743    0.006
##     Mean.Rap.Crck2      0.120    0.057    2.090    0.037
##     X10km_Pop2          0.444    0.059    7.498    0.000
##   Total.Animals2 ~
##     Zoo.Area.ha2        0.303    0.054    5.662    0.000
##     Sp.Richness2        0.748    0.045   16.498    0.000
##     Mn.Sp.BdyMsXA2     -0.262    0.050   -5.234    0.000
##   Sp.Richness2 ~
##     Mam.Sp.Rchnss2      0.861    0.051   16.866    0.000
##     Mn.Sp.BdyMsXA2     -0.213    0.051   -4.179    0.000
##   Mean.Raup.Crick2 ~
##     Mn.Sp.BdyMsXA2     -0.687    0.088   -7.810    0.000
##     Zoo.Area.ha2        0.350    0.088    3.973    0.000
##   Brillouin.Index2 ~
##     Sp.Richness2        1.573    0.094   16.708    0.000
##     Total.Animals2     -1.081    0.094  -11.496    0.000
##   insitu2 ~
##     Attendance2         0.579    0.098    5.902    0.000
##     Zoo.Area.ha2        0.176    0.082    2.134    0.033
##     Sp.Richness2        0.190    0.248    0.769    0.442
##     Total.Animals2     -0.305    0.196   -1.553    0.120
##     Mam.Sp.Rchnss2      0.187    0.130    1.440    0.150
```

```
##     Prop.Mam.Abdn2     -0.062    0.070   -0.895    0.371
##     Prp.Thrt.Abnd2      0.203    0.066    3.092    0.002
##     Brilloun.Indx2     -0.197    0.122   -1.618    0.106
##     Mean.Rap.Crck2      0.114    0.067    1.701    0.089
##
## Covariances:
##                            Estimate  Std.Err  z-value  P(>|z|)
##   Mam.Sp.Richness2 ~~
##     Prp.Thrt.Abnd2            0.203    0.093    2.190    0.028
##     Mn.Sp.BdyMsXA2            0.219    0.093    2.357    0.018
##     X10km_Pop2                0.421    0.099    4.262    0.000
##     Zoo.Area.ha2              0.536    0.103    5.185    0.000
##     Prop.Mam.Abdn2           -0.025    0.091   -0.273    0.785
##   Prop.Threat.Abund2 ~~
##     Mn.Sp.BdyMsXA2            0.092    0.091    1.012    0.312
##     X10km_Pop2                0.278    0.094    2.948    0.003
##     Zoo.Area.ha2              0.071    0.091    0.776    0.438
##     Prop.Mam.Abdn2           -0.195    0.093   -2.102    0.036
##   Mean.Sp.BodyMassXAbund2 ~~
##     X10km_Pop2               -0.209    0.093   -2.245    0.025
##     Zoo.Area.ha2              0.527    0.103    5.120    0.000
##     Prop.Mam.Abdn2            0.453    0.100    4.531    0.000
##   X10km_Pop2 ~~
##     Zoo.Area.ha2             -0.099    0.091   -1.087    0.277
##     Prop.Mam.Abdn2           -0.373    0.097   -3.839    0.000
##   Zoo.Area.ha2 ~~
##     Prop.Mam.Abdn2            0.223    0.093    2.396    0.017
##
## Variances:
##                    Estimate  Std.Err  z-value  P(>|z|)
##    .Attendance2       0.274    0.035    7.714    0.000
##    .Total.Animals2    0.198    0.026    7.714    0.000
##    .Sp.Richness2      0.292    0.038    7.714    0.000
##    .Mean.Rap.Crck2    0.655    0.085    7.714    0.000
##    .Brilloun.Indx2    0.274    0.036    7.714    0.000
##    .insitu2           0.463    0.060    7.714    0.000
##     Mam.Sp.Rchnss2    0.992    0.129    7.714    0.000
##     Prp.Thrt.Abnd2    0.992    0.129    7.714    0.000
##     Mn.Sp.BdyMsXA2    0.992    0.129    7.714    0.000
##     X10km_Pop2        0.992    0.129    7.714    0.000
##     Zoo.Area.ha2      0.992    0.129    7.714    0.000
##     Prop.Mam.Abdn2    0.992    0.129    7.714    0.000
##
## R-Square:
##                    Estimate
##     Attendance2       0.697
##     Total.Animals2    0.801
##     Sp.Richness2      0.705
##     Mean.Rap.Crck2    0.339
##     Brilloun.Indx2    0.723
##     insitu2           0.527
```

```r
# Generate fit indices
fitMeasures(mod.IS7A.fit, c("agfi", "rmr", "srmr", "rmsea", "cfi", "nnfi", "tli"))
```

```
##  agfi   rmr  srmr rmsea   cfi  nnfi   tli
## 0.687 0.073 0.074 0.150 0.918 0.833 0.833
```

```
# Generate modification indices
miIS7A <- modindices(mod.IS7A.fit)
print(miIS7A[miIS7A$mi > 3.0,])
```

```
##                          lhs op                rhs     mi    epc sepc.lv
## 59            Total.Animals2 ~~       Sp.Richness2  3.206 -0.049  -0.049
## 63              Sp.Richness2 ~~   Mean.Raup.Crick2  5.986  0.098   0.098
## 76            Total.Animals2  ~   Mam.Sp.Richness2  3.206  0.145   0.145
## 77            Total.Animals2  ~ Prop.Threat.Abund2  7.695 -0.116  -0.116
## 80              Sp.Richness2  ~        Attendance2  8.991  0.354   0.354
## 82              Sp.Richness2  ~   Mean.Raup.Crick2  5.188  0.133   0.133
## 84              Sp.Richness2  ~            insitu2 12.203  0.430   0.430
## 86              Sp.Richness2  ~        X10km_Pop2 13.975  0.219   0.219
## 88              Sp.Richness2  ~    Prop.Mam.Abdun2 30.563 -0.313  -0.313
## 90          Mean.Raup.Crick2  ~     Total.Animals2  3.156  0.164   0.164
## 91          Mean.Raup.Crick2  ~       Sp.Richness2  3.980  0.165   0.165
## 97          Mean.Raup.Crick2  ~    Prop.Mam.Abdun2  6.144 -0.208  -0.208
## 102         Brillouin.Index2  ~ Prop.Threat.Abund2  7.232  0.131   0.131
## 111         Mam.Sp.Richness2  ~       Sp.Richness2  7.427 -0.448  -0.448
## 113         Mam.Sp.Richness2  ~   Brillouin.Index2  6.750 -0.262  -0.262
## 120       Prop.Threat.Abund2  ~        Attendance2  3.271 -0.447  -0.447
## 121       Prop.Threat.Abund2  ~     Total.Animals2  8.349 -0.408  -0.408
## 124       Prop.Threat.Abund2  ~   Brillouin.Index2 13.296  0.376   0.376
## 125       Prop.Threat.Abund2  ~            insitu2  6.343 -0.944  -0.944
## 131 Mean.Sp.BodyMassXAbund2  ~        Attendance2  3.378  0.382   0.382
## 132 Mean.Sp.BodyMassXAbund2  ~     Total.Animals2 18.717  0.934   0.934
## 133 Mean.Sp.BodyMassXAbund2  ~       Sp.Richness2 15.249  0.796   0.796
## 134 Mean.Sp.BodyMassXAbund2  ~   Mean.Raup.Crick2  7.777  0.536   0.536
## 142               X10km_Pop2  ~        Attendance2  3.517  0.344   0.344
## 143               X10km_Pop2  ~     Total.Animals2  6.848  0.302   0.302
## 144               X10km_Pop2  ~       Sp.Richness2  6.043  0.315   0.315
## 157              Zoo.Area.ha2  ~   Brillouin.Index2  3.423  0.175   0.175
## 164          Prop.Mam.Abdun2  ~        Attendance2  3.572 -0.231  -0.231
## 165          Prop.Mam.Abdun2  ~     Total.Animals2 13.552 -0.462  -0.462
## 166          Prop.Mam.Abdun2  ~       Sp.Richness2 21.450 -0.646  -0.646
## 167          Prop.Mam.Abdun2  ~   Mean.Raup.Crick2  6.479 -0.237  -0.237
##     sepc.all sepc.nox
## 59    -0.050   -0.050
## 63     0.099    0.099
## 76     0.145    0.146
## 77    -0.115   -0.116
## 80     0.338    0.338
## 82     0.133    0.133
## 84     0.427    0.427
## 86     0.219    0.220
## 88    -0.313   -0.314
## 90     0.164    0.164
## 91     0.165    0.165
## 97    -0.208   -0.209
## 102    0.132    0.132
## 111   -0.448   -0.448
## 113   -0.262   -0.262
```

```
## 120   -0.426   -0.426
## 121   -0.408   -0.408
## 124    0.375    0.375
## 125   -0.937   -0.937
## 131    0.365    0.365
## 132    0.935    0.935
## 133    0.796    0.796
## 134    0.536    0.536
## 142    0.328    0.328
## 143    0.302    0.302
## 144    0.315    0.315
## 157    0.175    0.175
## 164   -0.220   -0.220
## 165   -0.463   -0.463
## 166   -0.646   -0.646
## 167   -0.237   -0.237
```

Model 8

Based on the results generated from the seventh model, the highest p-value relationship to be considered for removal is **insitu2** ~ **Sp.Richness2** with a p-value of **0.442**. Therefore we decide to remove this pathway. The model summary, fit indices and modification indices were all generated for the model.

```
# In Situ SEM (Abundance)

# Model 8
# Removal of insitu2 ~ Sp.Richness2, p = 0.442

mod.IS8A <- 'Attendance2 ~ Sp.Richness2 + Total.Animals2
+ Mam.Sp.Richness2 + Prop.Threat.Abund2
+ Mean.Sp.BodyMassXAbund2 + Brillouin.Index2 + Mean.Raup.Crick2
+ X10km_Pop2

Total.Animals2 ~ Zoo.Area.ha2 + Sp.Richness2 + Mean.Sp.BodyMassXAbund2
Sp.Richness2 ~ Mam.Sp.Richness2 + Mean.Sp.BodyMassXAbund2
Mean.Raup.Crick2 ~ Mean.Sp.BodyMassXAbund2 + Zoo.Area.ha2
Brillouin.Index2 ~ Sp.Richness2 + Total.Animals2

insitu2 ~ Attendance2 + Zoo.Area.ha2 + Total.Animals2
+ Mam.Sp.Richness2 + Prop.Mam.Abdun2 + Prop.Threat.Abund2
+ Brillouin.Index2 + Mean.Raup.Crick2'

# Fit model and generate model summary
mod.IS8A.fit <- sem(mod.IS8A , data = sem_in_situ_data, fixed.x=FALSE)
summary(mod.IS8A.fit, rsq = TRUE)
```

```
## lavaan (0.5-23.1097) converged normally after  42 iterations
##
##   Number of observations                           119
##
##   Estimator                                         ML
##   Minimum Function Test Statistic               92.869
##   Degrees of freedom                                26
##   P-value (Chi-square)                           0.000
```

```
##
## Parameter Estimates:
##
##   Information                                 Expected
##   Standard Errors                             Standard
##
## Regressions:
##                      Estimate  Std.Err  z-value  P(>|z|)
##   Attendance2 ~
##     Sp.Richness2       -0.473    0.184   -2.561    0.010
##     Total.Animals2      0.790    0.140    5.661    0.000
##     Mam.Sp.Rchnss2      0.091    0.097    0.932    0.351
##     Prp.Thrt.Abnd2     -0.085    0.051   -1.663    0.096
##     Mn.Sp.BdyMsXA2      0.261    0.066    3.962    0.000
##     Brilloun.Indx2      0.251    0.092    2.743    0.006
##     Mean.Rap.Crck2      0.120    0.057    2.090    0.037
##     X10km_Pop2          0.444    0.059    7.498    0.000
##   Total.Animals2 ~
##     Zoo.Area.ha2        0.303    0.054    5.662    0.000
##     Sp.Richness2        0.748    0.045   16.498    0.000
##     Mn.Sp.BdyMsXA2     -0.262    0.050   -5.234    0.000
##   Sp.Richness2 ~
##     Mam.Sp.Rchnss2      0.861    0.051   16.866    0.000
##     Mn.Sp.BdyMsXA2     -0.213    0.051   -4.179    0.000
##   Mean.Raup.Crick2 ~
##     Mn.Sp.BdyMsXA2     -0.687    0.088   -7.810    0.000
##     Zoo.Area.ha2        0.350    0.088    3.973    0.000
##   Brillouin.Index2 ~
##     Sp.Richness2        1.573    0.094   16.708    0.000
##     Total.Animals2     -1.081    0.094  -11.496    0.000
##   insitu2 ~
##     Attendance2         0.573    0.097    5.921    0.000
##     Zoo.Area.ha2        0.165    0.081    2.035    0.042
##     Total.Animals2     -0.194    0.103   -1.886    0.059
##     Mam.Sp.Rchnss2      0.236    0.118    1.994    0.046
##     Prop.Mam.Abdn2     -0.083    0.070   -1.182    0.237
##     Prp.Thrt.Abnd2      0.195    0.066    2.963    0.003
##     Brilloun.Indx2     -0.122    0.077   -1.590    0.112
##     Mean.Rap.Crck2      0.111    0.067    1.650    0.099
##
## Covariances:
##                              Estimate  Std.Err  z-value  P(>|z|)
##   Mam.Sp.Richness2 ~~
##     Prp.Thrt.Abnd2              0.203    0.093    2.190    0.028
##     Mn.Sp.BdyMsXA2              0.219    0.093    2.357    0.018
##     X10km_Pop2                  0.421    0.099    4.262    0.000
##     Zoo.Area.ha2                0.536    0.103    5.185    0.000
##     Prop.Mam.Abdn2             -0.025    0.091   -0.273    0.785
##   Prop.Threat.Abund2 ~~
##     Mn.Sp.BdyMsXA2              0.092    0.091    1.012    0.312
##     X10km_Pop2                  0.278    0.094    2.948    0.003
##     Zoo.Area.ha2                0.071    0.091    0.776    0.438
##     Prop.Mam.Abdn2             -0.195    0.093   -2.102    0.036
##   Mean.Sp.BodyMassXAbund2 ~~
```

```
##     X10km_Pop2                -0.209    0.093   -2.245    0.025
##     Zoo.Area.ha2               0.527    0.103    5.120    0.000
##     Prop.Mam.Abdn2             0.453    0.100    4.531    0.000
##   X10km_Pop2 ~~
##     Zoo.Area.ha2              -0.099    0.091   -1.087    0.277
##     Prop.Mam.Abdn2            -0.373    0.097   -3.839    0.000
##   Zoo.Area.ha2 ~~
##     Prop.Mam.Abdn2             0.223    0.093    2.396    0.017
##
## Variances:
##                    Estimate  Std.Err  z-value  P(>|z|)
##    .Attendance2       0.274    0.035    7.714    0.000
##    .Total.Animals2    0.198    0.026    7.714    0.000
##    .Sp.Richness2      0.292    0.038    7.714    0.000
##    .Mean.Rap.Crck2    0.655    0.085    7.714    0.000
##    .Brilloun.Indx2    0.274    0.036    7.714    0.000
##    .insitu2           0.465    0.060    7.714    0.000
##     Mam.Sp.Rchnss2    0.992    0.129    7.714    0.000
##     Prp.Thrt.Abnd2    0.992    0.129    7.714    0.000
##     Mn.Sp.BdyMsXA2    0.992    0.129    7.714    0.000
##     X10km_Pop2        0.992    0.129    7.714    0.000
##     Zoo.Area.ha2      0.992    0.129    7.714    0.000
##     Prop.Mam.Abdn2    0.992    0.129    7.714    0.000
##
## R-Square:
##                    Estimate
##     Attendance2       0.697
##     Total.Animals2    0.801
##     Sp.Richness2      0.705
##     Mean.Rap.Crck2    0.339
##     Brilloun.Indx2    0.723
##     insitu2           0.526
```

```
# Generate fit indices
fitMeasures(mod.IS8A.fit, c("agfi", "rmr", "srmr", "rmsea", "cfi", "nnfi", "tli"))
```

```
##  agfi   rmr  srmr rmsea   cfi  nnfi   tli
## 0.697 0.073 0.074 0.147 0.919 0.841 0.841
```

```
# Generate modification indices
miIS8A <- modindices(mod.IS8A.fit)
print(miIS8A[miIS8A$mi > 3.0,])
```

```
##                   lhs op               rhs     mi    epc sepc.lv
## 58         Total.Animals2 ~~      Sp.Richness2  3.206 -0.049  -0.049
## 62           Sp.Richness2 ~~   Mean.Raup.Crick2  5.986  0.098   0.098
## 75         Total.Animals2  ~   Mam.Sp.Richness2  3.205  0.145   0.145
## 76         Total.Animals2  ~ Prop.Threat.Abund2  7.695 -0.116  -0.116
## 79           Sp.Richness2  ~        Attendance2  8.991  0.354   0.354
## 81           Sp.Richness2  ~   Mean.Raup.Crick2  5.188  0.133   0.133
## 83           Sp.Richness2  ~            insitu2 10.848  0.313   0.313
## 85           Sp.Richness2  ~         X10km_Pop2 13.975  0.219   0.219
## 87           Sp.Richness2  ~     Prop.Mam.Abdun2 30.563 -0.313  -0.313
## 89       Mean.Raup.Crick2  ~      Total.Animals2  3.156  0.164   0.164
## 90       Mean.Raup.Crick2  ~        Sp.Richness2  3.980  0.165   0.165
## 96       Mean.Raup.Crick2  ~     Prop.Mam.Abdun2  6.144 -0.208  -0.208
```

```
## 101        Brillouin.Index2  ~ Prop.Threat.Abund2  7.232  0.131   0.131
## 111        Mam.Sp.Richness2  ~       Sp.Richness2  7.427 -0.448  -0.448
## 113        Mam.Sp.Richness2  ~   Brillouin.Index2  6.750 -0.262  -0.262
## 120      Prop.Threat.Abund2  ~        Attendance2  3.271 -0.447  -0.447
## 121      Prop.Threat.Abund2  ~     Total.Animals2  8.349 -0.408  -0.408
## 124      Prop.Threat.Abund2  ~   Brillouin.Index2 13.296  0.376   0.376
## 125      Prop.Threat.Abund2  ~            insitu2  4.582 -0.785  -0.785
## 131 Mean.Sp.BodyMassXAbund2  ~        Attendance2  3.378  0.382   0.382
## 132 Mean.Sp.BodyMassXAbund2  ~     Total.Animals2 18.717  0.934   0.934
## 133 Mean.Sp.BodyMassXAbund2  ~       Sp.Richness2 15.249  0.796   0.796
## 134 Mean.Sp.BodyMassXAbund2  ~   Mean.Raup.Crick2  7.776  0.536   0.536
## 142              X10km_Pop2  ~        Attendance2  3.517  0.344   0.344
## 143              X10km_Pop2  ~     Total.Animals2  6.848  0.302   0.302
## 144              X10km_Pop2  ~       Sp.Richness2  6.043  0.315   0.315
## 157            Zoo.Area.ha2  ~   Brillouin.Index2  3.423  0.175   0.175
## 164          Prop.Mam.Abdun2  ~        Attendance2  3.572 -0.231  -0.231
## 165          Prop.Mam.Abdun2  ~     Total.Animals2 13.552 -0.462  -0.462
## 166          Prop.Mam.Abdun2  ~       Sp.Richness2 21.450 -0.646  -0.646
## 167          Prop.Mam.Abdun2  ~   Mean.Raup.Crick2  6.479 -0.237  -0.237
##     sepc.all sepc.nox
## 58    -0.050   -0.050
## 62     0.099    0.099
## 75     0.145    0.146
## 76    -0.115   -0.116
## 79     0.338    0.338
## 81     0.133    0.133
## 83     0.311    0.311
## 85     0.219    0.220
## 87    -0.313   -0.314
## 89     0.164    0.164
## 90     0.165    0.165
## 96    -0.208   -0.209
## 101    0.132    0.132
## 111   -0.448   -0.448
## 113   -0.262   -0.262
## 120   -0.426   -0.426
## 121   -0.408   -0.408
## 124    0.375    0.375
## 125   -0.781   -0.781
## 131    0.365    0.365
## 132    0.935    0.935
## 133    0.796    0.796
## 134    0.536    0.536
## 142    0.328    0.328
## 143    0.302    0.302
## 144    0.315    0.315
## 157    0.175    0.175
## 164   -0.220   -0.220
## 165   -0.463   -0.463
## 166   -0.646   -0.646
## 167   -0.237   -0.237
```

Model 9

Based on the results generated from the eight model, the highest p-value relationship to be considered for removal is **Attendance2** ~ **Mam.Sp.Rchnss2** with a p-value of **0.351**. Therefore we decide to remove this pathway. The model summary, fit indices and modification indices were all generated for the model.

```
# In Situ SEM (Abundance)

# Model 9
# Removal of Attendance2 ~ Mam.Sp.Rchnss2, p = 0.351

mod.IS9A <- 'Attendance2 ~ Sp.Richness2 + Total.Animals2
+ Prop.Threat.Abund2 + Mean.Sp.BodyMassXAbund2 + Brillouin.Index2
+ Mean.Raup.Crick2 + X10km_Pop2

Total.Animals2 ~ Zoo.Area.ha2 + Sp.Richness2 + Mean.Sp.BodyMassXAbund2
Sp.Richness2 ~ Mam.Sp.Richness2 + Mean.Sp.BodyMassXAbund2
Mean.Raup.Crick2 ~ Mean.Sp.BodyMassXAbund2 + Zoo.Area.ha2
Brillouin.Index2 ~ Sp.Richness2 + Total.Animals2

insitu2 ~ Attendance2 + Zoo.Area.ha2 + Total.Animals2
+ Mam.Sp.Richness2 + Prop.Mam.Abdun2 + Prop.Threat.Abund2
+ Brillouin.Index2 + Mean.Raup.Crick2'

# Fit model and generate model summary
mod.IS9A.fit <- sem(mod.IS9A , data = sem_in_situ_data, fixed.x=FALSE)
summary(mod.IS9A.fit, rsq = TRUE)
```

```
## lavaan (0.5-23.1097) converged normally after  40 iterations
##
##   Number of observations                           119
##
##   Estimator                                         ML
##   Minimum Function Test Statistic               93.755
##   Degrees of freedom                                27
##   P-value (Chi-square)                           0.000
##
## Parameter Estimates:
##
##   Information                                 Expected
##   Standard Errors                             Standard
##
## Regressions:
##                    Estimate  Std.Err  z-value  P(>|z|)
##   Attendance2 ~
##     Sp.Richness2     -0.412    0.176   -2.343    0.019
##     Total.Animals2    0.813    0.139    5.840    0.000
##     Prp.Thrt.Abnd2   -0.079    0.051   -1.550    0.121
##     Mn.Sp.BdyMsXA2    0.283    0.058    4.860    0.000
##     Brilloun.Indx2    0.243    0.092    2.647    0.008
##     Mean.Rap.Crck2    0.115    0.057    2.013    0.044
##     X10km_Pop2        0.444    0.057    7.821    0.000
##   Total.Animals2 ~
##     Zoo.Area.ha2      0.303    0.054    5.662    0.000
##     Sp.Richness2      0.748    0.045   16.498    0.000
```

```
##     Mn.Sp.BdyMsXA2     -0.262    0.050   -5.234    0.000
##   Sp.Richness2 ~
##     Mam.Sp.Rchnss2      0.861    0.051   16.866    0.000
##     Mn.Sp.BdyMsXA2     -0.213    0.051   -4.179    0.000
##   Mean.Raup.Crick2 ~
##     Mn.Sp.BdyMsXA2     -0.687    0.088   -7.810    0.000
##     Zoo.Area.ha2        0.350    0.088    3.973    0.000
##   Brillouin.Index2 ~
##     Sp.Richness2        1.573    0.094   16.708    0.000
##     Total.Animals2     -1.081    0.094  -11.496    0.000
##   insitu2 ~
##     Attendance2         0.573    0.097    5.934    0.000
##     Zoo.Area.ha2        0.165    0.081    2.035    0.042
##     Total.Animals2     -0.194    0.105   -1.851    0.064
##     Mam.Sp.Rchnss2      0.236    0.117    2.021    0.043
##     Prop.Mam.Abdn2     -0.083    0.070   -1.183    0.237
##     Prp.Thrt.Abnd2      0.195    0.066    2.963    0.003
##     Brilloun.Indx2     -0.122    0.077   -1.587    0.113
##     Mean.Rap.Crck2      0.111    0.067    1.652    0.099
##
## Covariances:
##                           Estimate  Std.Err  z-value  P(>|z|)
##   Prop.Threat.Abund2 ~~
##     Mn.Sp.BdyMsXA2           0.092    0.091    1.012    0.312
##     X10km_Pop2               0.278    0.094    2.948    0.003
##     Zoo.Area.ha2             0.071    0.091    0.776    0.438
##     Mam.Sp.Rchnss2           0.203    0.093    2.190    0.028
##     Prop.Mam.Abdn2          -0.195    0.093   -2.102    0.036
##   Mean.Sp.BodyMassXAbund2 ~~
##     X10km_Pop2              -0.209    0.093   -2.245    0.025
##     Zoo.Area.ha2             0.527    0.103    5.120    0.000
##     Mam.Sp.Rchnss2           0.219    0.093    2.357    0.018
##     Prop.Mam.Abdn2           0.453    0.100    4.531    0.000
##   X10km_Pop2 ~~
##     Zoo.Area.ha2            -0.099    0.091   -1.087    0.277
##     Mam.Sp.Rchnss2           0.421    0.099    4.262    0.000
##     Prop.Mam.Abdn2          -0.373    0.097   -3.839    0.000
##   Zoo.Area.ha2 ~~
##     Mam.Sp.Rchnss2           0.536    0.103    5.185    0.000
##     Prop.Mam.Abdn2           0.223    0.093    2.396    0.017
##   Mam.Sp.Richness2 ~~
##     Prop.Mam.Abdn2          -0.025    0.091   -0.273    0.785
##
## Variances:
##                    Estimate  Std.Err  z-value  P(>|z|)
##    .Attendance2       0.276    0.036    7.714    0.000
##    .Total.Animals2    0.198    0.026    7.714    0.000
##    .Sp.Richness2      0.292    0.038    7.714    0.000
##    .Mean.Rap.Crck2    0.655    0.085    7.714    0.000
##    .Brilloun.Indx2    0.274    0.036    7.714    0.000
##    .insitu2           0.465    0.060    7.714    0.000
##     Prp.Thrt.Abnd2    0.992    0.129    7.714    0.000
##     Mn.Sp.BdyMsXA2    0.992    0.129    7.714    0.000
##     X10km_Pop2        0.992    0.129    7.714    0.000
```

```
##     Zoo.Area.ha2      0.992    0.129    7.714    0.000
##     Mam.Sp.Rchnss2    0.992    0.129    7.714    0.000
##     Prop.Mam.Abdn2    0.992    0.129    7.714    0.000
##
## R-Square:
##                    Estimate
##     Attendance2       0.692
##     Total.Animals2    0.801
##     Sp.Richness2      0.705
##     Mean.Rap.Crck2    0.339
##     Brilloun.Indx2    0.723
##     insitu2           0.520
```

```
# Generate fit indices
fitMeasures(mod.IS9A.fit, c("agfi", "rmr", "srmr", "rmsea", "cfi", "nnfi", "tli"))
```

```
##  agfi   rmr  srmr rmsea   cfi  nnfi   tli
## 0.706 0.074 0.075 0.144 0.919 0.847 0.847
```

```
# Generate modification indices
miIS9A <- modindices(mod.IS9A.fit)
print(miIS9A[miIS9A$mi > 3.0,])
```

```
##                          lhs op                rhs     mi    epc sepc.lv
## 57            Total.Animals2 ~~       Sp.Richness2  3.206 -0.049  -0.049
## 61              Sp.Richness2 ~~    Mean.Raup.Crick2  5.986  0.098   0.098
## 75            Total.Animals2  ~ Prop.Threat.Abund2  7.695 -0.116  -0.116
## 77            Total.Animals2  ~   Mam.Sp.Richness2  3.206  0.145   0.145
## 81              Sp.Richness2  ~   Mean.Raup.Crick2  5.188  0.133   0.133
## 83              Sp.Richness2  ~            insitu2  6.597  0.221   0.221
## 85              Sp.Richness2  ~         X10km_Pop2 13.975  0.219   0.219
## 87              Sp.Richness2  ~     Prop.Mam.Abdun2 30.563 -0.313  -0.313
## 89          Mean.Raup.Crick2  ~      Total.Animals2  3.156  0.164   0.164
## 90          Mean.Raup.Crick2  ~       Sp.Richness2  3.980  0.165   0.165
## 96          Mean.Raup.Crick2  ~     Prop.Mam.Abdun2  6.144 -0.208  -0.208
## 100         Brillouin.Index2  ~ Prop.Threat.Abund2  7.232  0.131   0.131
## 109       Prop.Threat.Abund2  ~        Attendance2  3.327 -0.417  -0.417
## 110       Prop.Threat.Abund2  ~      Total.Animals2  8.349 -0.408  -0.408
## 113       Prop.Threat.Abund2  ~   Brillouin.Index2 13.296  0.376   0.376
## 114       Prop.Threat.Abund2  ~            insitu2  5.194 -0.837  -0.837
## 121 Mean.Sp.BodyMassXAbund2  ~      Total.Animals2 18.717  0.934   0.934
## 122 Mean.Sp.BodyMassXAbund2  ~       Sp.Richness2 15.249  0.796   0.796
## 123 Mean.Sp.BodyMassXAbund2  ~   Mean.Raup.Crick2  7.777  0.536   0.536
## 132               X10km_Pop2  ~      Total.Animals2  6.848  0.302   0.302
## 133               X10km_Pop2  ~       Sp.Richness2  6.043  0.315   0.315
## 146             Zoo.Area.ha2  ~   Brillouin.Index2  3.423  0.175   0.175
## 155         Mam.Sp.Richness2  ~       Sp.Richness2  7.427 -0.448  -0.448
## 157         Mam.Sp.Richness2  ~   Brillouin.Index2  6.750 -0.262  -0.262
## 164          Prop.Mam.Abdun2  ~        Attendance2  3.383 -0.219  -0.219
## 165          Prop.Mam.Abdun2  ~      Total.Animals2 13.552 -0.462  -0.462
## 166          Prop.Mam.Abdun2  ~       Sp.Richness2 21.450 -0.646  -0.646
## 167          Prop.Mam.Abdun2  ~   Mean.Raup.Crick2  6.479 -0.237  -0.237
##     sepc.all sepc.nox
## 57    -0.050   -0.050
## 61     0.099    0.099
## 75    -0.115   -0.116
```

```
## 77      0.145    0.146
## 81      0.133    0.133
## 83      0.219    0.219
## 85      0.219    0.220
## 87     -0.313   -0.314
## 89      0.164    0.164
## 90      0.165    0.165
## 96     -0.208   -0.209
## 100     0.132    0.132
## 109    -0.396   -0.396
## 110    -0.408   -0.408
## 113     0.375    0.375
## 114    -0.828   -0.828
## 121     0.935    0.935
## 122     0.796    0.796
## 123     0.536    0.536
## 132     0.302    0.302
## 133     0.315    0.315
## 146     0.175    0.175
## 155    -0.448   -0.448
## 157    -0.262   -0.262
## 164    -0.208   -0.208
## 165    -0.463   -0.463
## 166    -0.646   -0.646
## 167    -0.237   -0.237
```

Model 10

Based on the results generated from the ninth model, the highest p-value relationship to be considered for removal is **insitu2** ~ **Prop.Mam.Abdn2** with a p-value of **0.237**. Therefore we decide to remove this pathway. The model summary, fit indices and modification indices were all generated for the model.

```
# In Situ SEM (Abundance)

# Model 10
# Removal of insitu2 ~ Prop.Mam.Abdn2, p = 0.237

mod.IS10A <- 'Attendance2 ~ Sp.Richness2 + Total.Animals2
+ Prop.Threat.Abund2 + Mean.Sp.BodyMassXAbund2 + Brillouin.Index2
+ Mean.Raup.Crick2 + X10km_Pop2

Total.Animals2 ~ Zoo.Area.ha2 + Sp.Richness2 + Mean.Sp.BodyMassXAbund2
Sp.Richness2 ~ Mam.Sp.Richness2 + Mean.Sp.BodyMassXAbund2
Mean.Raup.Crick2 ~ Mean.Sp.BodyMassXAbund2 + Zoo.Area.ha2
Brillouin.Index2 ~ Sp.Richness2 + Total.Animals2

insitu2 ~ Attendance2 + Zoo.Area.ha2 + Total.Animals2
+ Mam.Sp.Richness2 + Prop.Threat.Abund2
+ Brillouin.Index2 + Mean.Raup.Crick2'

# Fit model and generate model summary
mod.IS10A.fit <- sem(mod.IS10A , data = sem_in_situ_data, fixed.x=FALSE)
summary(mod.IS10A.fit, rsq = TRUE)
```

```
## lavaan (0.5-23.1097) converged normally after  38 iterations
##
##   Number of observations                           119
##
##   Estimator                                         ML
##   Minimum Function Test Statistic               56.305
##   Degrees of freedom                                22
##   P-value (Chi-square)                           0.000
##
## Parameter Estimates:
##
##   Information                                 Expected
##   Standard Errors                             Standard
##
## Regressions:
##                      Estimate  Std.Err  z-value  P(>|z|)
##   Attendance2 ~
##     Sp.Richness2       -0.412    0.176   -2.343    0.019
##     Total.Animals2      0.813    0.139    5.840    0.000
##     Prp.Thrt.Abnd2     -0.079    0.051   -1.550    0.121
##     Mn.Sp.BdyMsXA2      0.283    0.058    4.860    0.000
##     Brilloun.Indx2      0.243    0.092    2.647    0.008
##     Mean.Rap.Crck2      0.115    0.057    2.013    0.044
##     X10km_Pop2          0.444    0.057    7.821    0.000
##   Total.Animals2 ~
##     Zoo.Area.ha2        0.303    0.054    5.662    0.000
##     Sp.Richness2        0.748    0.045   16.498    0.000
##     Mn.Sp.BdyMsXA2     -0.262    0.050   -5.234    0.000
##   Sp.Richness2 ~
##     Mam.Sp.Rchnss2      0.861    0.051   16.866    0.000
##     Mn.Sp.BdyMsXA2     -0.213    0.051   -4.179    0.000
##   Mean.Raup.Crick2 ~
##     Mn.Sp.BdyMsXA2     -0.687    0.088   -7.810    0.000
##     Zoo.Area.ha2        0.350    0.088    3.973    0.000
##   Brillouin.Index2 ~
##     Sp.Richness2        1.573    0.094   16.708    0.000
##     Total.Animals2     -1.081    0.094  -11.496    0.000
##   insitu2 ~
##     Attendance2         0.572    0.097    5.912    0.000
##     Zoo.Area.ha2        0.139    0.078    1.789    0.074
##     Total.Animals2     -0.137    0.105   -1.304    0.192
##     Mam.Sp.Rchnss2      0.200    0.117    1.705    0.088
##     Prp.Thrt.Abnd2      0.216    0.065    3.344    0.001
##     Brilloun.Indx2     -0.114    0.077   -1.481    0.139
##     Mean.Rap.Crck2      0.128    0.066    1.936    0.053
##
## Covariances:
##                              Estimate  Std.Err  z-value  P(>|z|)
##   Prop.Threat.Abund2 ~~
##     Mn.Sp.BdyMsXA2              0.092    0.091    1.012    0.312
##     X10km_Pop2                  0.278    0.094    2.948    0.003
##     Zoo.Area.ha2                0.071    0.091    0.776    0.438
##     Mam.Sp.Rchnss2              0.203    0.093    2.190    0.028
##   Mean.Sp.BodyMassXAbund2 ~~
```

```
##     X10km_Pop2                -0.209    0.093   -2.245    0.025
##     Zoo.Area.ha2               0.527    0.103    5.120    0.000
##     Mam.Sp.Rchnss2             0.219    0.093    2.357    0.018
##   X10km_Pop2 ~~
##     Zoo.Area.ha2              -0.099    0.091   -1.087    0.277
##     Mam.Sp.Rchnss2             0.421    0.099    4.262    0.000
##   Zoo.Area.ha2 ~~
##     Mam.Sp.Rchnss2             0.536    0.103    5.185    0.000
##
## Variances:
##                    Estimate  Std.Err  z-value  P(>|z|)
##    .Attendance2       0.276    0.036    7.714    0.000
##    .Total.Animals2    0.198    0.026    7.714    0.000
##    .Sp.Richness2      0.292    0.038    7.714    0.000
##    .Mean.Rap.Crck2    0.655    0.085    7.714    0.000
##    .Brilloun.Indx2    0.274    0.036    7.714    0.000
##    .insitu2           0.469    0.061    7.714    0.000
##     Prp.Thrt.Abnd2    0.992    0.129    7.714    0.000
##     Mn.Sp.BdyMsXA2    0.992    0.129    7.714    0.000
##     X10km_Pop2        0.992    0.129    7.714    0.000
##     Zoo.Area.ha2      0.992    0.129    7.714    0.000
##     Mam.Sp.Rchnss2    0.992    0.129    7.714    0.000
##
## R-Square:
##                    Estimate
##     Attendance2       0.692
##     Total.Animals2    0.801
##     Sp.Richness2      0.705
##     Mean.Rap.Crck2    0.339
##     Brilloun.Indx2    0.723
##     insitu2           0.516
```

```
# Generate fit indices
fitMeasures(mod.IS10A.fit, c("agfi", "rmr", "srmr", "rmsea", "cfi", "nnfi", "tli"))
```

```
##  agfi   rmr  srmr rmsea   cfi  nnfi   tli
## 0.780 0.064 0.065 0.114 0.957 0.911 0.911
```

```
# Generate modification indices
miIS10A <- modindices(mod.IS10A.fit)
print(miIS10A[miIS10A$mi > 3.0,])
```

```
##                     lhs op               rhs     mi    epc sepc.lv
## 50          Total.Animals2 ~~       Sp.Richness2  3.206 -0.049  -0.049
## 54            Sp.Richness2 ~~   Mean.Raup.Crick2  5.986  0.098   0.098
## 67          Total.Animals2  ~ Prop.Threat.Abund2  7.695 -0.116  -0.116
## 69          Total.Animals2  ~   Mam.Sp.Richness2  3.206  0.145   0.145
## 72            Sp.Richness2  ~   Mean.Raup.Crick2  5.188  0.133   0.133
## 74            Sp.Richness2  ~            insitu2  4.266  0.178   0.178
## 76            Sp.Richness2  ~         X10km_Pop2 13.975  0.219   0.219
## 79        Mean.Raup.Crick2  ~     Total.Animals2  3.156  0.164   0.164
## 80        Mean.Raup.Crick2  ~       Sp.Richness2  3.980  0.165   0.165
## 89       Brillouin.Index2  ~ Prop.Threat.Abund2  7.232  0.131   0.131
## 98      Prop.Threat.Abund2  ~     Total.Animals2  4.969 -0.320  -0.320
## 101     Prop.Threat.Abund2  ~   Brillouin.Index2 15.493  0.414   0.414
## 102     Prop.Threat.Abund2  ~            insitu2  5.044 -0.776  -0.776
```

```
## 108 Mean.Sp.BodyMassXAbund2  ~     Total.Animals2  5.683  0.764   0.764
## 117              X10km_Pop2  ~        Attendance2  6.213  0.431   0.431
## 118              X10km_Pop2  ~     Total.Animals2 13.102  0.430   0.430
## 119              X10km_Pop2  ~       Sp.Richness2 13.645  0.488   0.488
## 131            Zoo.Area.ha2  ~   Brillouin.Index2  3.631  0.180   0.180
## 139        Mam.Sp.Richness2  ~       Sp.Richness2  9.069 -0.496  -0.496
## 141        Mam.Sp.Richness2  ~   Brillouin.Index2  7.112 -0.269  -0.269
##     sepc.all sepc.nox
## 50    -0.050   -0.050
## 54     0.099    0.099
## 67    -0.115   -0.116
## 69     0.145    0.146
## 72     0.133    0.133
## 74     0.176    0.176
## 76     0.219    0.220
## 79     0.164    0.164
## 80     0.165    0.165
## 89     0.132    0.132
## 98    -0.321   -0.321
## 101    0.413    0.413
## 102   -0.767   -0.767
## 108    0.765    0.765
## 117    0.410    0.410
## 118    0.431    0.431
## 119    0.488    0.488
## 131    0.180    0.180
## 139   -0.496   -0.496
## 141   -0.269   -0.269
```

Model 11

Based on the results generated from the tenth model, the highest p-value relationship to be considered for removal is **insitu2** ~ **Total.Animals2** with a p-value of **0.192**. Therefore we decide to remove this pathway. The model summary, fit indices and modification indices were all generated for the model.

```
# In Situ SEM (Abundance)

# Model 11
# Removal of insitu2 ~ Total.Animals2, p = 0.192

mod.IS11A <- 'Attendance2 ~ Sp.Richness2 + Total.Animals2
+ Prop.Threat.Abund2 + Mean.Sp.BodyMassXAbund2 + Brillouin.Index2
+ Mean.Raup.Crick2 + X10km_Pop2

Total.Animals2 ~ Zoo.Area.ha2 + Sp.Richness2 + Mean.Sp.BodyMassXAbund2
Sp.Richness2 ~ Mam.Sp.Richness2 + Mean.Sp.BodyMassXAbund2
Mean.Raup.Crick2 ~ Mean.Sp.BodyMassXAbund2 + Zoo.Area.ha2
Brillouin.Index2 ~ Sp.Richness2 + Total.Animals2

insitu2 ~ Attendance2 + Zoo.Area.ha2
+ Mam.Sp.Richness2 + Prop.Threat.Abund2
+ Brillouin.Index2 + Mean.Raup.Crick2'
```

```r
# Fit model and generate model summary
mod.IS11A.fit <- sem(mod.IS11A , data = sem_in_situ_data, fixed.x=FALSE)
summary(mod.IS11A.fit, rsq = TRUE)
```

```
## lavaan (0.5-23.1097) converged normally after  36 iterations
##
##   Number of observations                           119
##
##   Estimator                                         ML
##   Minimum Function Test Statistic               57.541
##   Degrees of freedom                                23
##   P-value (Chi-square)                           0.000
##
## Parameter Estimates:
##
##   Information                                 Expected
##   Standard Errors                             Standard
##
## Regressions:
##                    Estimate  Std.Err  z-value  P(>|z|)
##   Attendance2 ~
##     Sp.Richness2     -0.412    0.176   -2.343    0.019
##     Total.Animals2    0.813    0.139    5.840    0.000
##     Prp.Thrt.Abnd2   -0.079    0.051   -1.550    0.121
##     Mn.Sp.BdyMsXA2    0.283    0.058    4.860    0.000
##     Brilloun.Indx2    0.243    0.092    2.647    0.008
##     Mean.Rap.Crck2    0.115    0.057    2.013    0.044
##     X10km_Pop2        0.444    0.057    7.821    0.000
##   Total.Animals2 ~
##     Zoo.Area.ha2      0.303    0.054    5.662    0.000
##     Sp.Richness2      0.748    0.045   16.498    0.000
##     Mn.Sp.BdyMsXA2   -0.262    0.050   -5.234    0.000
##   Sp.Richness2 ~
##     Mam.Sp.Rchnss2    0.861    0.051   16.866    0.000
##     Mn.Sp.BdyMsXA2   -0.213    0.051   -4.179    0.000
##   Mean.Raup.Crick2 ~
##     Mn.Sp.BdyMsXA2   -0.687    0.088   -7.810    0.000
##     Zoo.Area.ha2      0.350    0.088    3.973    0.000
##   Brillouin.Index2 ~
##     Sp.Richness2      1.573    0.094   16.708    0.000
##     Total.Animals2   -1.081    0.094  -11.496    0.000
##   insitu2 ~
##     Attendance2       0.519    0.088    5.885    0.000
##     Zoo.Area.ha2      0.136    0.078    1.748    0.080
##     Mam.Sp.Rchnss2    0.127    0.105    1.218    0.223
##     Prp.Thrt.Abnd2    0.222    0.065    3.418    0.001
##     Brilloun.Indx2   -0.099    0.077   -1.287    0.198
##     Mean.Rap.Crck2    0.100    0.065    1.541    0.123
##
## Covariances:
##                            Estimate  Std.Err  z-value  P(>|z|)
##   Prop.Threat.Abund2 ~~
##     Mn.Sp.BdyMsXA2            0.092    0.091    1.012    0.312
##     X10km_Pop2                0.278    0.094    2.948    0.003
```

```
##     Zoo.Area.ha2              0.071    0.091    0.776    0.438
##     Mam.Sp.Rchnss2            0.203    0.093    2.190    0.028
##   Mean.Sp.BodyMassXAbund2 ~~
##     X10km_Pop2               -0.209    0.093   -2.245    0.025
##     Zoo.Area.ha2              0.527    0.103    5.120    0.000
##     Mam.Sp.Rchnss2            0.219    0.093    2.357    0.018
##   X10km_Pop2 ~~
##     Zoo.Area.ha2             -0.099    0.091   -1.087    0.277
##     Mam.Sp.Rchnss2            0.421    0.099    4.262    0.000
##   Zoo.Area.ha2 ~~
##     Mam.Sp.Rchnss2            0.536    0.103    5.185    0.000
##
## Variances:
##                    Estimate  Std.Err  z-value  P(>|z|)
##    .Attendance2       0.276    0.036    7.714    0.000
##    .Total.Animals2    0.198    0.026    7.714    0.000
##    .Sp.Richness2      0.292    0.038    7.714    0.000
##    .Mean.Rap.Crck2    0.655    0.085    7.714    0.000
##    .Brilloun.Indx2    0.274    0.036    7.714    0.000
##    .insitu2           0.474    0.061    7.714    0.000
##     Prp.Thrt.Abnd2    0.992    0.129    7.714    0.000
##     Mn.Sp.BdyMsXA2    0.992    0.129    7.714    0.000
##     X10km_Pop2        0.992    0.129    7.714    0.000
##     Zoo.Area.ha2      0.992    0.129    7.714    0.000
##     Mam.Sp.Rchnss2    0.992    0.129    7.714    0.000
##
## R-Square:
##                    Estimate
##     Attendance2       0.692
##     Total.Animals2    0.801
##     Sp.Richness2      0.705
##     Mean.Rap.Crck2    0.339
##     Brilloun.Indx2    0.723
##     insitu2           0.509
```

```r
# Generate fit indices
fitMeasures(mod.IS11A.fit, c("agfi", "rmr", "srmr", "rmsea", "cfi", "nnfi", "tli"))
```

```
##  agfi   rmr  srmr rmsea   cfi  nnfi   tli
## 0.786 0.064 0.064 0.112 0.956 0.915 0.915
```

```r
# Generate modification indices
miIS11A <- modindices(mod.IS11A.fit)
print(miIS11A[miIS11A$mi > 3.0,])
```

```
##                         lhs op                rhs     mi    epc sepc.lv
## 49           Total.Animals2 ~~       Sp.Richness2  3.206 -0.049  -0.049
## 53             Sp.Richness2 ~~   Mean.Raup.Crick2  5.986  0.098   0.098
## 66           Total.Animals2  ~ Prop.Threat.Abund2  7.695 -0.116  -0.116
## 68           Total.Animals2  ~   Mam.Sp.Richness2  3.205  0.145   0.145
## 71             Sp.Richness2  ~   Mean.Raup.Crick2  5.188  0.133   0.133
## 75             Sp.Richness2  ~         X10km_Pop2 13.975  0.219   0.219
## 78         Mean.Raup.Crick2  ~     Total.Animals2  3.156  0.164   0.164
## 79         Mean.Raup.Crick2  ~       Sp.Richness2  3.980  0.165   0.165
## 88         Brillouin.Index2  ~ Prop.Threat.Abund2  7.232  0.131   0.131
## 98       Prop.Threat.Abund2  ~     Total.Animals2  4.969 -0.320  -0.320
```

```
## 101      Prop.Threat.Abund2  ~   Brillouin.Index2 15.493  0.414   0.414
## 102      Prop.Threat.Abund2  ~           insitu2  5.907 -0.769  -0.769
## 108 Mean.Sp.BodyMassXAbund2  ~     Total.Animals2  5.683  0.764   0.764
## 117              X10km_Pop2  ~       Attendance2  6.213  0.431   0.431
## 118              X10km_Pop2  ~     Total.Animals2 13.102  0.430   0.430
## 119              X10km_Pop2  ~       Sp.Richness2 13.645  0.488   0.488
## 131             Zoo.Area.ha2  ~   Brillouin.Index2  3.631  0.180   0.180
## 139         Mam.Sp.Richness2  ~       Sp.Richness2  9.069 -0.496  -0.496
## 141         Mam.Sp.Richness2  ~   Brillouin.Index2  7.112 -0.269  -0.269
##     sepc.all sepc.nox
## 49    -0.050   -0.050
## 53     0.099    0.099
## 66    -0.115   -0.116
## 68     0.145    0.146
## 71     0.133    0.133
## 75     0.219    0.220
## 78     0.164    0.164
## 79     0.165    0.165
## 88     0.132    0.132
## 98    -0.321   -0.321
## 101    0.413    0.413
## 102   -0.759   -0.759
## 108    0.765    0.765
## 117    0.410    0.410
## 118    0.431    0.431
## 119    0.488    0.488
## 131    0.180    0.180
## 139   -0.496   -0.496
## 141   -0.269   -0.269
```

Model 12

Based on the results generated from the eleventh model, the highest p-value relationship to be considered for removal is **insitu2** ~ **Mam.Sp.Rchnss2** with a p-value of **0.223**. Therefore we decide to remove this pathway. The model summary, fit indices and modification indices were all generated for the model.

```
# In Situ SEM (Abundance)

# Model 12
# Removal of insitu2 ~ Mam.Sp.Rchnss2, p = 0.223

mod.IS12A <- 'Attendance2 ~ Sp.Richness2 + Total.Animals2
+ Prop.Threat.Abund2 + Mean.Sp.BodyMassXAbund2 + Brillouin.Index2
+ Mean.Raup.Crick2 + X10km_Pop2

Total.Animals2 ~ Zoo.Area.ha2 + Sp.Richness2 + Mean.Sp.BodyMassXAbund2
Sp.Richness2 ~ Mam.Sp.Richness2 + Mean.Sp.BodyMassXAbund2
Mean.Raup.Crick2 ~ Mean.Sp.BodyMassXAbund2 + Zoo.Area.ha2
Brillouin.Index2 ~ Sp.Richness2 + Total.Animals2

insitu2 ~ Attendance2 + Zoo.Area.ha2
+ Prop.Threat.Abund2
+ Brillouin.Index2 + Mean.Raup.Crick2'
```

```r
# Fit model and generate model summary
mod.IS12A.fit <- sem(mod.IS12A , data = sem_in_situ_data, fixed.x=FALSE)
summary(mod.IS12A.fit, rsq = TRUE)
```

```
## lavaan (0.5-23.1097) converged normally after  36 iterations
##
##   Number of observations                           119
##
##   Estimator                                         ML
##   Minimum Function Test Statistic               59.166
##   Degrees of freedom                                24
##   P-value (Chi-square)                           0.000
##
## Parameter Estimates:
##
##   Information                                 Expected
##   Standard Errors                             Standard
##
## Regressions:
##                         Estimate  Std.Err  z-value  P(>|z|)
##   Attendance2 ~
##     Sp.Richness2          -0.412    0.176   -2.343    0.019
##     Total.Animals2         0.813    0.139    5.840    0.000
##     Prp.Thrt.Abnd2        -0.079    0.051   -1.550    0.121
##     Mn.Sp.BdyMsXA2         0.283    0.058    4.860    0.000
##     Brilloun.Indx2         0.243    0.092    2.647    0.008
##     Mean.Rap.Crck2         0.115    0.057    2.013    0.044
##     X10km_Pop2             0.444    0.057    7.821    0.000
##   Total.Animals2 ~
##     Zoo.Area.ha2           0.303    0.054    5.662    0.000
##     Sp.Richness2           0.748    0.045   16.498    0.000
##     Mn.Sp.BdyMsXA2        -0.262    0.050   -5.234    0.000
##   Sp.Richness2 ~
##     Mam.Sp.Rchnss2         0.861    0.051   16.866    0.000
##     Mn.Sp.BdyMsXA2        -0.213    0.051   -4.179    0.000
##   Mean.Raup.Crick2 ~
##     Mn.Sp.BdyMsXA2        -0.687    0.088   -7.810    0.000
##     Zoo.Area.ha2           0.350    0.088    3.973    0.000
##   Brillouin.Index2 ~
##     Sp.Richness2           1.573    0.094   16.708    0.000
##     Total.Animals2        -1.081    0.094  -11.496    0.000
##   insitu2 ~
##     Attendance2            0.577    0.078    7.411    0.000
##     Zoo.Area.ha2           0.180    0.068    2.640    0.008
##     Prp.Thrt.Abnd2         0.225    0.065    3.475    0.001
##     Brilloun.Indx2        -0.068    0.069   -0.980    0.327
##     Mean.Rap.Crck2         0.094    0.065    1.445    0.148
##
## Covariances:
##                                 Estimate  Std.Err  z-value  P(>|z|)
##   Prop.Threat.Abund2 ~~
##     Mn.Sp.BdyMsXA2                 0.092    0.091    1.012    0.312
##     X10km_Pop2                     0.278    0.094    2.948    0.003
```

```
##     Zoo.Area.ha2               0.071    0.091    0.776    0.438
##     Mam.Sp.Rchnss2             0.203    0.093    2.190    0.028
##   Mean.Sp.BodyMassXAbund2 ~~
##     X10km_Pop2                -0.209    0.093   -2.245    0.025
##     Zoo.Area.ha2               0.527    0.103    5.120    0.000
##     Mam.Sp.Rchnss2             0.219    0.093    2.357    0.018
##   X10km_Pop2 ~~
##     Zoo.Area.ha2              -0.099    0.091   -1.087    0.277
##     Mam.Sp.Rchnss2             0.421    0.099    4.262    0.000
##   Zoo.Area.ha2 ~~
##     Mam.Sp.Rchnss2             0.536    0.103    5.185    0.000
##
## Variances:
##                    Estimate  Std.Err  z-value  P(>|z|)
##    .Attendance2       0.276    0.036    7.714    0.000
##    .Total.Animals2    0.198    0.026    7.714    0.000
##    .Sp.Richness2      0.292    0.038    7.714    0.000
##    .Mean.Rap.Crck2    0.655    0.085    7.714    0.000
##    .Brilloun.Indx2    0.274    0.036    7.714    0.000
##    .insitu2           0.481    0.062    7.714    0.000
##     Prp.Thrt.Abnd2    0.992    0.129    7.714    0.000
##     Mn.Sp.BdyMsXA2    0.992    0.129    7.714    0.000
##     X10km_Pop2        0.992    0.129    7.714    0.000
##     Zoo.Area.ha2      0.992    0.129    7.714    0.000
##     Mam.Sp.Rchnss2    0.992    0.129    7.714    0.000
##
## R-Square:
##                    Estimate
##     Attendance2       0.692
##     Total.Animals2    0.801
##     Sp.Richness2      0.705
##     Mean.Rap.Crck2    0.339
##     Brilloun.Indx2    0.723
##     insitu2           0.500
```

```
# Generate fit indices
fitMeasures(mod.IS12A.fit, c("agfi", "rmr", "srmr", "rmsea", "cfi", "nnfi", "tli"))
```

```
##  agfi   rmr  srmr rmsea   cfi  nnfi   tli
## 0.789 0.066 0.066 0.111 0.956 0.917 0.917
```

```
# Generate modification indices
miIS12A <- modindices(mod.IS12A.fit)
print(miIS12A[miIS12A$mi > 3.0,])
```

```
##                         lhs op                rhs     mi    epc sepc.lv
## 48           Total.Animals2 ~~       Sp.Richness2  3.206 -0.049  -0.049
## 52             Sp.Richness2 ~~   Mean.Raup.Crick2  5.986  0.098   0.098
## 65           Total.Animals2  ~ Prop.Threat.Abund2  7.695 -0.116  -0.116
## 67           Total.Animals2  ~   Mam.Sp.Richness2  3.206  0.145   0.145
## 70             Sp.Richness2  ~   Mean.Raup.Crick2  5.188  0.133   0.133
## 74             Sp.Richness2  ~         X10km_Pop2 13.975  0.219   0.219
## 77         Mean.Raup.Crick2  ~     Total.Animals2  3.156  0.164   0.164
## 78         Mean.Raup.Crick2  ~       Sp.Richness2  3.980  0.165   0.165
## 87         Brillouin.Index2  ~ Prop.Threat.Abund2  7.232  0.131   0.131
## 98       Prop.Threat.Abund2  ~     Total.Animals2  4.969 -0.320  -0.320
```

```
## 101      Prop.Threat.Abund2  ~   Brillouin.Index2 15.493  0.414   0.414
## 102      Prop.Threat.Abund2  ~           insitu2  5.028 -0.670  -0.670
## 108 Mean.Sp.BodyMassXAbund2  ~     Total.Animals2  5.683  0.764   0.764
## 117             X10km_Pop2  ~        Attendance2  6.213  0.431   0.431
## 118             X10km_Pop2  ~     Total.Animals2 13.102  0.430   0.430
## 119             X10km_Pop2  ~       Sp.Richness2 13.646  0.488   0.488
## 131            Zoo.Area.ha2  ~   Brillouin.Index2  3.631  0.180   0.180
## 139        Mam.Sp.Richness2  ~       Sp.Richness2  9.068 -0.496  -0.496
## 141        Mam.Sp.Richness2  ~   Brillouin.Index2  7.112 -0.269  -0.269
##     sepc.all sepc.nox
## 48    -0.050   -0.050
## 52     0.099    0.099
## 65    -0.115   -0.116
## 67     0.145    0.146
## 70     0.133    0.133
## 74     0.219    0.220
## 77     0.164    0.164
## 78     0.165    0.165
## 87     0.132    0.132
## 98    -0.321   -0.321
## 101    0.413    0.413
## 102   -0.659   -0.659
## 108    0.765    0.765
## 117    0.410    0.410
## 118    0.431    0.431
## 119    0.488    0.488
## 131    0.180    0.180
## 139   -0.496   -0.496
## 141   -0.269   -0.269
```

Model 13

Based on the results generated from the twelfth model, the highest p-value relationship to be considered for removal is **insitu2** ~ **Brilloun.Indx2** with a p-value of **0.327**. Therefore we decide to remove this pathway. The model summary, fit indices and modification indices were all generated for the model.

```
# In Situ SEM (Abundance)

# Model 13
# Removal of insitu2 ~ Brilloun.Indx2, p = 0.327

mod.IS13A <- 'Attendance2 ~ Sp.Richness2 + Total.Animals2
+ Prop.Threat.Abund2 + Mean.Sp.BodyMassXAbund2 + Brillouin.Index2
+ Mean.Raup.Crick2 + X10km_Pop2

Total.Animals2 ~ Zoo.Area.ha2 + Sp.Richness2 + Mean.Sp.BodyMassXAbund2
Sp.Richness2 ~ Mam.Sp.Richness2 + Mean.Sp.BodyMassXAbund2
Mean.Raup.Crick2 ~ Mean.Sp.BodyMassXAbund2 + Zoo.Area.ha2
Brillouin.Index2 ~ Sp.Richness2 + Total.Animals2

insitu2 ~ Attendance2 + Zoo.Area.ha2
+ Prop.Threat.Abund2 + Mean.Raup.Crick2'
```

```r
# Fit model and generate model summary
mod.IS13A.fit <- sem(mod.IS13A , data = sem_in_situ_data, fixed.x=FALSE)
summary(mod.IS13A.fit, rsq = TRUE)
```

```
## lavaan (0.5-23.1097) converged normally after  41 iterations
##
##   Number of observations                           119
##
##   Estimator                                         ML
##   Minimum Function Test Statistic               59.980
##   Degrees of freedom                                25
##   P-value (Chi-square)                           0.000
##
## Parameter Estimates:
##
##   Information                                 Expected
##   Standard Errors                             Standard
##
## Regressions:
##                    Estimate  Std.Err  z-value  P(>|z|)
##   Attendance2 ~
##     Sp.Richness2     -0.412    0.176   -2.343    0.019
##     Total.Animals2    0.813    0.139    5.840    0.000
##     Prp.Thrt.Abnd2   -0.079    0.051   -1.550    0.121
##     Mn.Sp.BdyMsXA2    0.283    0.058    4.860    0.000
##     Brilloun.Indx2    0.243    0.092    2.647    0.008
##     Mean.Rap.Crck2    0.115    0.057    2.013    0.044
##     X10km_Pop2        0.444    0.057    7.821    0.000
##   Total.Animals2 ~
##     Zoo.Area.ha2      0.303    0.054    5.662    0.000
##     Sp.Richness2      0.748    0.045   16.498    0.000
##     Mn.Sp.BdyMsXA2   -0.262    0.050   -5.234    0.000
##   Sp.Richness2 ~
##     Mam.Sp.Rchnss2    0.861    0.051   16.866    0.000
##     Mn.Sp.BdyMsXA2   -0.213    0.051   -4.179    0.000
##   Mean.Raup.Crick2 ~
##     Mn.Sp.BdyMsXA2   -0.687    0.088   -7.810    0.000
##     Zoo.Area.ha2      0.350    0.088    3.973    0.000
##   Brillouin.Index2 ~
##     Sp.Richness2      1.573    0.094   16.708    0.000
##     Total.Animals2   -1.081    0.094  -11.496    0.000
##   insitu2 ~
##     Attendance2       0.557    0.073    7.603    0.000
##     Zoo.Area.ha2      0.179    0.068    2.616    0.009
##     Prp.Thrt.Abnd2    0.199    0.065    3.071    0.002
##     Mean.Rap.Crck2    0.091    0.065    1.398    0.162
##
## Covariances:
##                            Estimate  Std.Err  z-value  P(>|z|)
##   Prop.Threat.Abund2 ~~
##     Mn.Sp.BdyMsXA2            0.092    0.091    1.012    0.312
##     X10km_Pop2                0.278    0.094    2.948    0.003
##     Zoo.Area.ha2              0.071    0.091    0.776    0.438
##     Mam.Sp.Rchnss2            0.203    0.093    2.190    0.028
```

```
##   Mean.Sp.BodyMassXAbund2 ~~
##     X10km_Pop2               -0.209    0.093   -2.245    0.025
##     Zoo.Area.ha2              0.527    0.103    5.120    0.000
##     Mam.Sp.Rchnss2            0.219    0.093    2.357    0.018
##   X10km_Pop2 ~~
##     Zoo.Area.ha2             -0.099    0.091   -1.087    0.277
##     Mam.Sp.Rchnss2            0.421    0.099    4.262    0.000
##   Zoo.Area.ha2 ~~
##     Mam.Sp.Rchnss2            0.536    0.103    5.185    0.000
##
## Variances:
##                    Estimate  Std.Err  z-value  P(>|z|)
##    .Attendance2       0.276    0.036    7.714    0.000
##    .Total.Animals2    0.198    0.026    7.714    0.000
##    .Sp.Richness2      0.292    0.038    7.714    0.000
##    .Mean.Rap.Crck2    0.655    0.085    7.714    0.000
##    .Brilloun.Indx2    0.274    0.036    7.714    0.000
##    .insitu2           0.484    0.063    7.714    0.000
##     Prp.Thrt.Abnd2    0.992    0.129    7.714    0.000
##     Mn.Sp.BdyMsXA2    0.992    0.129    7.714    0.000
##     X10km_Pop2        0.992    0.129    7.714    0.000
##     Zoo.Area.ha2      0.992    0.129    7.714    0.000
##     Mam.Sp.Rchnss2    0.992    0.129    7.714    0.000
##
## R-Square:
##                    Estimate
##     Attendance2       0.692
##     Total.Animals2    0.801
##     Sp.Richness2      0.705
##     Mean.Rap.Crck2    0.339
##     Brilloun.Indx2    0.723
##     insitu2           0.491
```

```
# Generate fit indices
fitMeasures(mod.IS13A.fit, c("agfi", "rmr", "srmr", "rmsea", "cfi", "nnfi", "tli"))
```

```
##  agfi   rmr  srmr rmsea   cfi  nnfi   tli
## 0.794 0.064 0.065 0.108 0.956 0.921 0.921
```

```
# Generate modification indices
miIS13A <- modindices(mod.IS13A.fit)
print(miIS13A[miIS13A$mi > 3.0,])
```

```
##                         lhs op                rhs     mi    epc sepc.lv
## 47           Total.Animals2 ~~       Sp.Richness2  3.206 -0.049  -0.049
## 51             Sp.Richness2 ~~    Mean.Raup.Crick2  5.986  0.098   0.098
## 64           Total.Animals2  ~ Prop.Threat.Abund2  7.695 -0.116  -0.116
## 66           Total.Animals2  ~    Mam.Sp.Richness2  3.206  0.145   0.145
## 69             Sp.Richness2  ~    Mean.Raup.Crick2  5.188  0.133   0.133
## 73             Sp.Richness2  ~         X10km_Pop2 13.975  0.219   0.219
## 76         Mean.Raup.Crick2  ~     Total.Animals2  3.156  0.164   0.164
## 77         Mean.Raup.Crick2  ~       Sp.Richness2  3.980  0.165   0.165
## 86         Brillouin.Index2  ~ Prop.Threat.Abund2  7.232  0.131   0.131
## 98       Prop.Threat.Abund2  ~     Total.Animals2  4.969 -0.320  -0.320
## 101      Prop.Threat.Abund2  ~   Brillouin.Index2 15.493  0.414   0.414
## 108 Mean.Sp.BodyMassXAbund2  ~     Total.Animals2  5.683  0.764   0.764
```

```
## 117              X10km_Pop2  ~        Attendance2  6.213  0.431   0.431
## 118              X10km_Pop2  ~     Total.Animals2 13.102  0.430   0.430
## 119              X10km_Pop2  ~       Sp.Richness2 13.645  0.488   0.488
## 131             Zoo.Area.ha2  ~   Brillouin.Index2  3.631  0.180   0.180
## 139         Mam.Sp.Richness2  ~       Sp.Richness2  9.068 -0.496  -0.496
## 141         Mam.Sp.Richness2  ~   Brillouin.Index2  7.112 -0.269  -0.269
##     sepc.all sepc.nox
## 47    -0.050   -0.050
## 51     0.099    0.099
## 64    -0.115   -0.116
## 66     0.145    0.146
## 69     0.133    0.133
## 73     0.219    0.220
## 76     0.164    0.164
## 77     0.165    0.165
## 86     0.132    0.132
## 98    -0.321   -0.321
## 101    0.413    0.413
## 108    0.765    0.765
## 117    0.410    0.410
## 118    0.431    0.431
## 119    0.488    0.488
## 131    0.180    0.180
## 139   -0.496   -0.496
## 141   -0.269   -0.269
```

Model 14

Based on the results generated from the thirteenth model, the highest p-value relationship to be considered for removal is **insitu2** ~ **Mean.Rap.Crck2** with a p-value of **0.162**. Therefore we decide to remove this pathway. The model summary, fit indices and modification indices were all generated for the model.

```
# In Situ SEM (Abundance)

# Model 14
# Removal of insitu2 ~ Mean.Rap.Crck2, p = 0.162

mod.IS14A <- 'Attendance2 ~ Sp.Richness2 + Total.Animals2
+ Prop.Threat.Abund2 + Mean.Sp.BodyMassXAbund2 + Brillouin.Index2
+ Mean.Raup.Crick2 + X10km_Pop2

Total.Animals2 ~ Zoo.Area.ha2 + Sp.Richness2 + Mean.Sp.BodyMassXAbund2
Sp.Richness2 ~ Mam.Sp.Richness2 + Mean.Sp.BodyMassXAbund2
Mean.Raup.Crick2 ~ Mean.Sp.BodyMassXAbund2 + Zoo.Area.ha2
Brillouin.Index2 ~ Sp.Richness2 + Total.Animals2

insitu2 ~ Attendance2 + Zoo.Area.ha2
+ Prop.Threat.Abund2'

# Fit model and generate model summary
mod.IS14A.fit <- sem(mod.IS14A , data = sem_in_situ_data, fixed.x=FALSE)
summary(mod.IS14A.fit, rsq = TRUE)
```

```
## lavaan (0.5-23.1097) converged normally after  35 iterations
```

```
##
##   Number of observations                           119
##
##   Estimator                                         ML
##   Minimum Function Test Statistic              61.827
##   Degrees of freedom                                26
##   P-value (Chi-square)                          0.000
##
## Parameter Estimates:
##
##   Information                                 Expected
##   Standard Errors                             Standard
##
## Regressions:
##                      Estimate  Std.Err  z-value  P(>|z|)
##   Attendance2 ~
##     Sp.Richness2       -0.412    0.176   -2.343    0.019
##     Total.Animals2      0.813    0.139    5.840    0.000
##     Prp.Thrt.Abnd2     -0.079    0.051   -1.550    0.121
##     Mn.Sp.BdyMsXA2      0.283    0.058    4.860    0.000
##     Brilloun.Indx2      0.243    0.092    2.647    0.008
##     Mean.Rap.Crck2      0.115    0.057    2.013    0.044
##     X10km_Pop2          0.444    0.057    7.821    0.000
##   Total.Animals2 ~
##     Zoo.Area.ha2        0.303    0.054    5.662    0.000
##     Sp.Richness2        0.748    0.045   16.498    0.000
##     Mn.Sp.BdyMsXA2     -0.262    0.050   -5.234    0.000
##   Sp.Richness2 ~
##     Mam.Sp.Rchnss2      0.861    0.051   16.866    0.000
##     Mn.Sp.BdyMsXA2     -0.213    0.051   -4.179    0.000
##   Mean.Raup.Crick2 ~
##     Mn.Sp.BdyMsXA2     -0.687    0.088   -7.810    0.000
##     Zoo.Area.ha2        0.350    0.088    3.973    0.000
##   Brillouin.Index2 ~
##     Sp.Richness2        1.573    0.094   16.708    0.000
##     Total.Animals2     -1.081    0.094  -11.496    0.000
##   insitu2 ~
##     Attendance2         0.583    0.073    8.025    0.000
##     Zoo.Area.ha2        0.169    0.069    2.456    0.014
##     Prp.Thrt.Abnd2      0.189    0.065    2.908    0.004
##
## Covariances:
##                              Estimate  Std.Err  z-value  P(>|z|)
##   Prop.Threat.Abund2 ~~
##     Mn.Sp.BdyMsXA2              0.092    0.091    1.012    0.312
##     X10km_Pop2                  0.278    0.094    2.948    0.003
##     Zoo.Area.ha2                0.071    0.091    0.776    0.438
##     Mam.Sp.Rchnss2              0.203    0.093    2.190    0.028
##   Mean.Sp.BodyMassXAbund2 ~~
##     X10km_Pop2                 -0.209    0.093   -2.245    0.025
##     Zoo.Area.ha2                0.527    0.103    5.120    0.000
##     Mam.Sp.Rchnss2              0.219    0.093    2.357    0.018
##   X10km_Pop2 ~~
##     Zoo.Area.ha2               -0.099    0.091   -1.087    0.277
```

```
##     Mam.Sp.Rchnss2              0.421    0.099    4.262    0.000
##   Zoo.Area.ha2 ~~
##     Mam.Sp.Rchnss2              0.536    0.103    5.185    0.000
##
## Variances:
##                    Estimate  Std.Err  z-value  P(>|z|)
##    .Attendance2       0.276    0.036    7.714    0.000
##    .Total.Animals2    0.198    0.026    7.714    0.000
##    .Sp.Richness2      0.292    0.038    7.714    0.000
##    .Mean.Rap.Crck2    0.655    0.085    7.714    0.000
##    .Brilloun.Indx2    0.274    0.036    7.714    0.000
##    .insitu2           0.492    0.064    7.714    0.000
##     Prp.Thrt.Abnd2    0.992    0.129    7.714    0.000
##     Mn.Sp.BdyMsXA2    0.992    0.129    7.714    0.000
##     X10km_Pop2        0.992    0.129    7.714    0.000
##     Zoo.Area.ha2      0.992    0.129    7.714    0.000
##     Mam.Sp.Rchnss2    0.992    0.129    7.714    0.000
##
## R-Square:
##                    Estimate
##     Attendance2       0.692
##     Total.Animals2    0.801
##     Sp.Richness2      0.705
##     Mean.Rap.Crck2    0.339
##     Brilloun.Indx2    0.723
##     insitu2           0.485
```

```
# Generate fit indices
fitMeasures(mod.IS14A.fit, c("agfi", "rmr", "srmr", "rmsea", "cfi", "nnfi", "tli"))
```

```
##  agfi   rmr  srmr rmsea   cfi  nnfi   tli
## 0.799 0.066 0.066 0.108 0.955 0.922 0.922
```

```
# Generate modification indices
miIS14A <- modindices(mod.IS14A.fit)
print(miIS14A[miIS14A$mi > 3.0,])
```

```
##                        lhs op               rhs     mi    epc sepc.lv
## 46          Total.Animals2 ~~      Sp.Richness2  3.206 -0.049  -0.049
## 50            Sp.Richness2 ~~   Mean.Raup.Crick2  5.986  0.098   0.098
## 63          Total.Animals2  ~ Prop.Threat.Abund2  7.695 -0.116  -0.116
## 65          Total.Animals2  ~   Mam.Sp.Richness2  3.206  0.145   0.145
## 68            Sp.Richness2  ~   Mean.Raup.Crick2  5.188  0.133   0.133
## 72            Sp.Richness2  ~         X10km_Pop2 13.975  0.219   0.219
## 75        Mean.Raup.Crick2  ~     Total.Animals2  3.156  0.164   0.164
## 76        Mean.Raup.Crick2  ~       Sp.Richness2  3.980  0.165   0.165
## 85        Brillouin.Index2  ~ Prop.Threat.Abund2  7.232  0.131   0.131
## 98      Prop.Threat.Abund2  ~     Total.Animals2  4.969 -0.320  -0.320
## 101     Prop.Threat.Abund2  ~   Brillouin.Index2 15.493  0.414   0.414
## 108 Mean.Sp.BodyMassXAbund2  ~     Total.Animals2  5.683  0.764   0.764
## 117             X10km_Pop2  ~        Attendance2  6.213  0.431   0.431
## 118             X10km_Pop2  ~     Total.Animals2 13.102  0.430   0.430
## 119             X10km_Pop2  ~       Sp.Richness2 13.645  0.488   0.488
## 131           Zoo.Area.ha2  ~   Brillouin.Index2  3.631  0.180   0.180
## 139       Mam.Sp.Richness2  ~       Sp.Richness2  9.068 -0.496  -0.496
## 141       Mam.Sp.Richness2  ~   Brillouin.Index2  7.112 -0.269  -0.269
```

```
##     sepc.all sepc.nox
## 46    -0.050   -0.050
## 50     0.099    0.099
## 63    -0.115   -0.116
## 65     0.145    0.146
## 68     0.133    0.133
## 72     0.219    0.220
## 75     0.164    0.164
## 76     0.165    0.165
## 85     0.132    0.132
## 98    -0.321   -0.321
## 101    0.413    0.413
## 108    0.765    0.765
## 117    0.410    0.410
## 118    0.431    0.431
## 119    0.488    0.488
## 131    0.180    0.180
## 139   -0.496   -0.496
## 141   -0.269   -0.269
```

Model 15

Based on the results generated from the fourteenth model, the highest p-value relationship to be considered for removal is **Attendance2** ~ **Prp.Thrt.Abnd2** with a p-value of **0.121**. Therefore we decide to remove this pathway. The model summary, fit indices and modification indices were all generated for the model.

```
# In Situ SEM (Abundance)

# Model 15
# Removal of Attendance2 ~ Prp.Thrt.Abnd2, p = 0.121

mod.IS15A <- 'Attendance2 ~ Sp.Richness2 + Total.Animals2
+ Mean.Sp.BodyMassXAbund2 + Brillouin.Index2
+ Mean.Raup.Crick2 + X10km_Pop2

Total.Animals2 ~ Zoo.Area.ha2 + Sp.Richness2 + Mean.Sp.BodyMassXAbund2
Sp.Richness2 ~ Mam.Sp.Richness2 + Mean.Sp.BodyMassXAbund2
Mean.Raup.Crick2 ~ Mean.Sp.BodyMassXAbund2 + Zoo.Area.ha2
Brillouin.Index2 ~ Sp.Richness2 + Total.Animals2

insitu2 ~ Attendance2 + Zoo.Area.ha2
+ Prop.Threat.Abund2'

# Fit model and generate model summary
mod.IS15A.fit <- sem(mod.IS15A , data = sem_in_situ_data, fixed.x=FALSE)
summary(mod.IS15A.fit, rsq = TRUE)
```

```
## lavaan (0.5-23.1097) converged normally after  33 iterations
##
##   Number of observations                           119
##
##   Estimator                                         ML
##   Minimum Function Test Statistic               63.854
##   Degrees of freedom                                27
```

```
##   P-value (Chi-square)                           0.000
##
## Parameter Estimates:
##
##   Information                                 Expected
##   Standard Errors                             Standard
##
## Regressions:
##                      Estimate  Std.Err  z-value  P(>|z|)
##   Attendance2 ~
##     Sp.Richness2       -0.390    0.177   -2.199    0.028
##     Total.Animals2      0.809    0.140    5.763    0.000
##     Mn.Sp.BdyMsXA2      0.280    0.058    4.808    0.000
##     Brilloun.Indx2      0.204    0.093    2.201    0.028
##     Mean.Rap.Crck2      0.122    0.058    2.117    0.034
##     X10km_Pop2          0.421    0.055    7.650    0.000
##   Total.Animals2 ~
##     Zoo.Area.ha2        0.303    0.054    5.662    0.000
##     Sp.Richness2        0.748    0.045   16.498    0.000
##     Mn.Sp.BdyMsXA2     -0.262    0.050   -5.234    0.000
##   Sp.Richness2 ~
##     Mam.Sp.Rchnss2      0.861    0.051   16.866    0.000
##     Mn.Sp.BdyMsXA2     -0.213    0.051   -4.179    0.000
##   Mean.Raup.Crick2 ~
##     Mn.Sp.BdyMsXA2     -0.687    0.088   -7.810    0.000
##     Zoo.Area.ha2        0.350    0.088    3.973    0.000
##   Brillouin.Index2 ~
##     Sp.Richness2        1.573    0.094   16.708    0.000
##     Total.Animals2     -1.081    0.094  -11.496    0.000
##   insitu2 ~
##     Attendance2         0.583    0.074    7.922    0.000
##     Zoo.Area.ha2        0.169    0.069    2.449    0.014
##     Prp.Thrt.Abnd2      0.189    0.066    2.873    0.004
##
## Covariances:
##                              Estimate  Std.Err  z-value  P(>|z|)
##   Mean.Sp.BodyMassXAbund2 ~~
##     X10km_Pop2                 -0.209    0.093   -2.245    0.025
##     Zoo.Area.ha2                0.527    0.103    5.120    0.000
##     Mam.Sp.Rchnss2              0.219    0.093    2.357    0.018
##     Prp.Thrt.Abnd2              0.092    0.091    1.012    0.312
##   X10km_Pop2 ~~
##     Zoo.Area.ha2               -0.099    0.091   -1.087    0.277
##     Mam.Sp.Rchnss2              0.421    0.099    4.262    0.000
##     Prp.Thrt.Abnd2              0.278    0.094    2.948    0.003
##   Zoo.Area.ha2 ~~
##     Mam.Sp.Rchnss2              0.536    0.103    5.185    0.000
##     Prp.Thrt.Abnd2              0.071    0.091    0.776    0.438
##   Mam.Sp.Richness2 ~~
##     Prp.Thrt.Abnd2              0.203    0.093    2.190    0.028
##
## Variances:
##                    Estimate  Std.Err  z-value  P(>|z|)
##    .Attendance2       0.280    0.036    7.714    0.000
```

```
##    .Total.Animals2    0.198    0.026    7.714    0.000
##    .Sp.Richness2      0.292    0.038    7.714    0.000
##    .Mean.Rap.Crck2    0.655    0.085    7.714    0.000
##    .Brilloun.Indx2    0.274    0.036    7.714    0.000
##    .insitu2           0.492    0.064    7.714    0.000
##     Mn.Sp.BdyMsXA2    0.992    0.129    7.714    0.000
##     X10km_Pop2        0.992    0.129    7.714    0.000
##     Zoo.Area.ha2      0.992    0.129    7.714    0.000
##     Mam.Sp.Rchnss2    0.992    0.129    7.714    0.000
##     Prp.Thrt.Abnd2    0.992    0.129    7.714    0.000
##
## R-Square:
##                    Estimate
##     Attendance2       0.689
##     Total.Animals2    0.801
##     Sp.Richness2      0.705
##     Mean.Rap.Crck2    0.339
##     Brilloun.Indx2    0.723
##     insitu2           0.495
```

```
# Generate fit indices
fitMeasures(mod.IS15A.fit, c("agfi", "rmr", "srmr", "rmsea", "cfi", "nnfi", "tli"))
```

```
##  agfi   rmr  srmr rmsea   cfi  nnfi   tli
## 0.802 0.066 0.067 0.107 0.953 0.922 0.922
```

```
# Generate modification indices
miIS15A <- modindices(mod.IS15A.fit)
print(miIS15A[miIS15A$mi > 3.0,])
```

```
##                          lhs op                rhs     mi    epc sepc.lv
## 45            Total.Animals2 ~~       Sp.Richness2  3.206 -0.049  -0.049
## 49              Sp.Richness2 ~~    Mean.Raup.Crick2  5.986  0.098   0.098
## 64            Total.Animals2  ~    Mam.Sp.Richness2  3.206  0.145   0.145
## 65            Total.Animals2  ~ Prop.Threat.Abund2  7.695 -0.116  -0.116
## 68              Sp.Richness2  ~    Mean.Raup.Crick2  5.188  0.133   0.133
## 71              Sp.Richness2  ~          X10km_Pop2 13.975  0.219   0.219
## 75          Mean.Raup.Crick2  ~      Total.Animals2  3.156  0.164   0.164
## 76          Mean.Raup.Crick2  ~        Sp.Richness2  3.980  0.165   0.165
## 89          Brillouin.Index2  ~ Prop.Threat.Abund2  7.232  0.131   0.131
## 98  Mean.Sp.BodyMassXAbund2  ~      Total.Animals2  5.683  0.764   0.764
## 107               X10km_Pop2  ~         Attendance2  7.761  0.455   0.455
## 108               X10km_Pop2  ~      Total.Animals2 13.102  0.430   0.430
## 109               X10km_Pop2  ~        Sp.Richness2 13.645  0.488   0.488
## 121             Zoo.Area.ha2  ~    Brillouin.Index2  3.631  0.180   0.180
## 129         Mam.Sp.Richness2  ~        Sp.Richness2  9.068 -0.496  -0.496
## 131         Mam.Sp.Richness2  ~    Brillouin.Index2  7.112 -0.269  -0.269
## 137       Prop.Threat.Abund2  ~         Attendance2  3.970 -0.269  -0.269
## 138       Prop.Threat.Abund2  ~      Total.Animals2  4.969 -0.320  -0.320
## 141       Prop.Threat.Abund2  ~    Brillouin.Index2 15.493  0.414   0.414
## 142       Prop.Threat.Abund2  ~             insitu2  3.301 -0.379  -0.379
##     sepc.all sepc.nox
## 45    -0.050   -0.050
## 49     0.099    0.099
## 64     0.145    0.146
## 65    -0.115   -0.116
```

```
## 68      0.133    0.133
## 71      0.219    0.220
## 75      0.164    0.164
## 76      0.165    0.165
## 89      0.132    0.132
## 98      0.765    0.765
## 107     0.434    0.434
## 108     0.431    0.431
## 109     0.488    0.488
## 121     0.180    0.180
## 129    -0.496   -0.496
## 131    -0.269   -0.269
## 137    -0.256   -0.256
## 138    -0.321   -0.321
## 141     0.413    0.413
## 142    -0.375   -0.375
```

Model Comparisons 10

Once again we compare the models generated using AICc values. Overall model selection from the pool of competing models is achieved using AICc values, with a threshold of more than 2 AICc units lower than nearest competing model being considered sufficient for model selection.

```
# Model Comparisons using AICc

# Comparing models with and without adjustment for nested nature of data
# library(AICcmodavg)
# source("lavaan.modavg.R")

aictab.lavaan(list(mod.IS1A.fit, mod.IS2A.fit, mod.IS3A.fit, mod.IS4A.fit, mod.IS5A.fit,
                   mod.IS6A.fit, mod.IS7A.fit, mod.IS8A.fit, mod.IS9A.fit, mod.IS10A.fit,
                   mod.IS11A.fit, mod.IS12A.fit, mod.IS13A.fit, mod.IS14A.fit, mod.IS15A.fit),
              c("mod.1", "mod.2", "mod.3", "mod.4", "mod.5", "mod.6", "mod.7", "mod.8",
                "mod.9", "mod.10", "mod.11", "mod.12", "mod.13", "mod.14", "mod.15"))
```

```
##
## Model selection based on AICc:
##
##         K    AICc Delta_AICc AICcWt Cum.Wt       LL
## mod.15 39 2868.82       0.00   0.44   0.44 -1391.99
## mod.14 40 2869.63       0.81   0.29   0.73 -1390.98
## mod.13 41 2870.68       1.86   0.17   0.90 -1390.05
## mod.12 42 2872.82       4.00   0.06   0.96 -1389.65
## mod.11 43 2874.21       5.39   0.03   0.99 -1388.83
## mod.10 44 2876.05       7.23   0.01   1.00 -1388.22
## mod.9  51 3181.51     312.69   0.00   1.00 -1533.37
## mod.8  52 3183.83     315.01   0.00   1.00 -1532.93
## mod.7  53 3186.59     317.77   0.00   1.00 -1532.66
## mod.6  54 3189.76     320.93   0.00   1.00 -1532.57
## mod.5  55 3193.00     324.18   0.00   1.00 -1532.48
## mod.4  56 3196.40     327.58   0.00   1.00 -1532.42
## mod.3  65 3440.04     571.22   0.00   1.00 -1643.62
## mod.2  66 3443.75     574.93   0.00   1.00 -1643.60
```

```
## mod.1  67 3447.56     578.74   0.00   1.00 -1643.58
```

Based on these results we can see that model 15 is the superior model (lowest AICc value).

Model 16

Due to the fact that **Brillouin.Index2** was deemed unnecessary in the Attendance Model and has proven unnecessary for the In Situ portion of this model, we choose to remove this variable entirely once again. The model summary, fit indices and modification indices were all generated for the model.

```
# In Situ SEM (Abundance)

# Model 16
# Removal of Brillouin.Index2

mod.IS16A <- 'Attendance2 ~ Sp.Richness2 + Total.Animals2
+ Mean.Sp.BodyMassXAbund2 + Mean.Raup.Crick2 + X10km_Pop2

Total.Animals2 ~ Zoo.Area.ha2 + Sp.Richness2 + Mean.Sp.BodyMassXAbund2
Sp.Richness2 ~ Mam.Sp.Richness2 + Mean.Sp.BodyMassXAbund2
Mean.Raup.Crick2 ~ Mean.Sp.BodyMassXAbund2 + Zoo.Area.ha2

insitu2 ~ Attendance2 + Zoo.Area.ha2
+ Prop.Threat.Abund2'

# Fit model and generate model summary
mod.IS16A.fit <- sem(mod.IS16A , data = sem_in_situ_data, fixed.x=FALSE)
summary(mod.IS16A.fit, rsq = TRUE)
```

```
## lavaan (0.5-23.1097) converged normally after  26 iterations
##
##   Number of observations                           119
##
##   Estimator                                         ML
##   Minimum Function Test Statistic               46.140
##   Degrees of freedom                                20
##   P-value (Chi-square)                           0.001
##
## Parameter Estimates:
##
##   Information                                 Expected
##   Standard Errors                             Standard
##
## Regressions:
##                    Estimate  Std.Err  z-value  P(>|z|)
##   Attendance2 ~
##     Sp.Richness2     -0.069    0.103   -0.667    0.505
##     Total.Animals2    0.589    0.100    5.873    0.000
##     Mn.Sp.BdyMsXA2    0.292    0.059    4.923    0.000
##     Mean.Rap.Crck2    0.134    0.059    2.276    0.023
##     X10km_Pop2        0.415    0.056    7.389    0.000
##   Total.Animals2 ~
##     Zoo.Area.ha2      0.303    0.054    5.662    0.000
##     Sp.Richness2      0.748    0.045   16.498    0.000
```

```
##     Mn.Sp.BdyMsXA2     -0.262    0.050   -5.234    0.000
##   Sp.Richness2 ~
##     Mam.Sp.Rchnss2      0.861    0.051   16.866    0.000
##     Mn.Sp.BdyMsXA2     -0.213    0.051   -4.179    0.000
##   Mean.Raup.Crick2 ~
##     Mn.Sp.BdyMsXA2     -0.687    0.088   -7.810    0.000
##     Zoo.Area.ha2        0.350    0.088    3.973    0.000
##   insitu2 ~
##     Attendance2         0.583    0.074    7.895    0.000
##     Zoo.Area.ha2        0.169    0.069    2.442    0.015
##     Prp.Thrt.Abnd2      0.189    0.066    2.873    0.004
##
## Covariances:
##                               Estimate  Std.Err  z-value  P(>|z|)
##   Mean.Sp.BodyMassXAbund2 ~~
##     X10km_Pop2                  -0.209    0.093   -2.245    0.025
##     Zoo.Area.ha2                 0.527    0.103    5.120    0.000
##     Mam.Sp.Rchnss2               0.219    0.093    2.357    0.018
##     Prp.Thrt.Abnd2               0.092    0.091    1.012    0.312
##   X10km_Pop2 ~~
##     Zoo.Area.ha2                -0.099    0.091   -1.087    0.277
##     Mam.Sp.Rchnss2               0.421    0.099    4.262    0.000
##     Prp.Thrt.Abnd2               0.278    0.094    2.948    0.003
##   Zoo.Area.ha2 ~~
##     Mam.Sp.Rchnss2               0.536    0.103    5.185    0.000
##     Prp.Thrt.Abnd2               0.071    0.091    0.776    0.438
##   Mam.Sp.Richness2 ~~
##     Prp.Thrt.Abnd2               0.203    0.093    2.190    0.028
##
## Variances:
##                    Estimate  Std.Err  z-value  P(>|z|)
##    .Attendance2       0.292    0.038    7.714    0.000
##    .Total.Animals2    0.198    0.026    7.714    0.000
##    .Sp.Richness2      0.292    0.038    7.714    0.000
##    .Mean.Rap.Crck2    0.655    0.085    7.714    0.000
##    .insitu2           0.492    0.064    7.714    0.000
##     Mn.Sp.BdyMsXA2    0.992    0.129    7.714    0.000
##     X10km_Pop2        0.992    0.129    7.714    0.000
##     Zoo.Area.ha2      0.992    0.129    7.714    0.000
##     Mam.Sp.Rchnss2    0.992    0.129    7.714    0.000
##     Prp.Thrt.Abnd2    0.992    0.129    7.714    0.000
##
## R-Square:
##                    Estimate
##     Attendance2       0.676
##     Total.Animals2    0.801
##     Sp.Richness2      0.705
##     Mean.Rap.Crck2    0.339
##     insitu2           0.496
```

```r
# Generate fit indices
fitMeasures(mod.IS16A.fit, c("agfi", "rmr", "srmr", "rmsea", "cfi", "nnfi", "tli"))
```

```
##  agfi   rmr  srmr rmsea   cfi  nnfi   tli
## 0.820 0.057 0.058 0.105 0.958 0.927 0.927
```

```r
# Generate modification indices
miIS16A <- modindices(mod.IS16A.fit)
print(miIS16A[miIS16A$mi > 3.0,])
```

```
##                          lhs op                rhs     mi    epc sepc.lv
## 40             Total.Animals2 ~~       Sp.Richness2  3.206 -0.049  -0.049
## 43               Sp.Richness2 ~~   Mean.Raup.Crick2  5.986  0.098   0.098
## 54             Total.Animals2  ~   Mam.Sp.Richness2  3.206  0.145   0.145
## 55             Total.Animals2  ~ Prop.Threat.Abund2  7.695 -0.116  -0.116
## 58               Sp.Richness2  ~   Mean.Raup.Crick2  5.188  0.133   0.133
## 60               Sp.Richness2  ~        X10km_Pop2 13.975  0.219   0.219
## 64           Mean.Raup.Crick2  ~     Total.Animals2  3.156  0.164   0.164
## 65           Mean.Raup.Crick2  ~       Sp.Richness2  3.980  0.165   0.165
## 77  Mean.Sp.BodyMassXAbund2  ~     Total.Animals2  5.683  0.764   0.764
## 85                 X10km_Pop2  ~       Attendance2  8.773  0.493   0.493
## 86                 X10km_Pop2  ~     Total.Animals2 13.102  0.430   0.430
## 87                 X10km_Pop2  ~       Sp.Richness2 13.646  0.488   0.488
## 105          Mam.Sp.Richness2  ~       Sp.Richness2  9.068 -0.496  -0.496
## 112        Prop.Threat.Abund2  ~       Attendance2  3.951 -0.267  -0.267
## 113        Prop.Threat.Abund2  ~     Total.Animals2  4.969 -0.320  -0.320
## 116        Prop.Threat.Abund2  ~           insitu2  3.284 -0.377  -0.377
##     sepc.all sepc.nox
## 40    -0.050   -0.050
## 43     0.099    0.099
## 54     0.145    0.146
## 55    -0.115   -0.116
## 58     0.133    0.133
## 60     0.219    0.220
## 64     0.164    0.164
## 65     0.165    0.165
## 77     0.765    0.765
## 85     0.470    0.470
## 86     0.431    0.431
## 87     0.488    0.488
## 105   -0.496   -0.496
## 112   -0.255   -0.255
## 113   -0.321   -0.321
## 116   -0.374   -0.374
```

Model Comparisons 10

Once again we compare the models generated using AICc values in order to ensure the removal of the **Brillouin.Index2** was statistically justified. Overall model selection from the pool of competing models is achieved using AICc values, with a threshold of more than 2 AICc units lower than nearest competing model being considered sufficient for model selection.

```r
# Model Comparisons using AICc

# Comparing models with and without adjustment for nested nature of data
# library(AICcmodavg)
# source("lavaan.modavg.R")

aictab.lavaan(list(mod.IS1A.fit, mod.IS2A.fit, mod.IS3A.fit, mod.IS4A.fit, mod.IS5A.fit,
```

```r
                    mod.IS6A.fit, mod.IS7A.fit, mod.IS8A.fit, mod.IS9A.fit, mod.IS10A.fit,
                    mod.IS11A.fit, mod.IS12A.fit, mod.IS13A.fit, mod.IS14A.fit, mod.IS15A.fit, mod.IS16A
               c("mod.1", "mod.2", "mod.3", "mod.4", "mod.5", "mod.6", "mod.7", "mod.8",
                 "mod.9", "mod.10", "mod.11", "mod.12", "mod.13", "mod.14", "mod.15", "mod.16"))
```

```
##
## Model selection based on AICc:
##
##          K    AICc Delta_AICc AICcWt Cum.Wt       LL
## mod.16 35 2679.70       0.00      1      1 -1302.52
## mod.15 39 2868.82     189.12      0      1 -1391.99
## mod.14 40 2869.63     189.93      0      1 -1390.98
## mod.13 41 2870.68     190.97      0      1 -1390.05
## mod.12 42 2872.82     193.11      0      1 -1389.65
## mod.11 43 2874.21     194.51      0      1 -1388.83
## mod.10 44 2876.05     196.35      0      1 -1388.22
## mod.9  51 3181.51     501.80      0      1 -1533.37
## mod.8  52 3183.83     504.13      0      1 -1532.93
## mod.7  53 3186.59     506.88      0      1 -1532.66
## mod.6  54 3189.76     510.05      0      1 -1532.57
## mod.5  55 3193.00     513.30      0      1 -1532.48
## mod.4  56 3196.40     516.69      0      1 -1532.42
## mod.3  65 3440.04     760.33      0      1 -1643.62
## mod.2  66 3443.75     764.05      0      1 -1643.60
## mod.1  67 3447.56     767.85      0      1 -1643.58
```

Based on these results we can see that model 16 is the superior model (lowest AICc value) and that **Brillouin.Index2** was not supported.

Model 17

Based on the results generated from the sixteenth model, the highest p-value relationship to be considered for removal is **Attendance2** ~ **Sp.Richness2** with a p-value of **0.505**. Therefore we decide to remove this pathway. The model summary, fit indices and modification indices were all generated for the model.

```r
# In Situ SEM (Abundance)

# Model 17
# Removal of Attendance2 ~ Sp.Richness2, p = 0.505

mod.IS17A <- 'Attendance2 ~ Total.Animals2
+ Mean.Sp.BodyMassXAbund2 + Mean.Raup.Crick2 + X10km_Pop2

Total.Animals2 ~ Zoo.Area.ha2 + Sp.Richness2 + Mean.Sp.BodyMassXAbund2
Sp.Richness2 ~ Mam.Sp.Richness2 + Mean.Sp.BodyMassXAbund2
Mean.Raup.Crick2 ~ Mean.Sp.BodyMassXAbund2 + Zoo.Area.ha2

insitu2 ~ Attendance2 + Zoo.Area.ha2
+ Prop.Threat.Abund2'

# Fit model and generate model summary
mod.IS17A.fit <- sem(mod.IS17A , data = sem_in_situ_data, fixed.x=FALSE)
summary(mod.IS17A.fit, rsq = TRUE)
```

```
## lavaan (0.5-23.1097) converged normally after  27 iterations
##
##   Number of observations                           119
##
##   Estimator                                         ML
##   Minimum Function Test Statistic               46.566
##   Degrees of freedom                                21
##   P-value (Chi-square)                           0.001
##
## Parameter Estimates:
##
##   Information                                 Expected
##   Standard Errors                             Standard
##
## Regressions:
##                      Estimate  Std.Err  z-value  P(>|z|)
##   Attendance2 ~
##     Total.Animals2      0.537    0.054    9.954    0.000
##     Mn.Sp.BdyMsXA2      0.283    0.059    4.829    0.000
##     Mean.Rap.Crck2      0.130    0.059    2.215    0.027
##     X10km_Pop2          0.400    0.054    7.447    0.000
##   Total.Animals2 ~
##     Zoo.Area.ha2        0.303    0.054    5.662    0.000
##     Sp.Richness2        0.748    0.045   16.498    0.000
##     Mn.Sp.BdyMsXA2     -0.262    0.050   -5.234    0.000
##   Sp.Richness2 ~
##     Mam.Sp.Rchnss2      0.861    0.051   16.866    0.000
##     Mn.Sp.BdyMsXA2     -0.213    0.051   -4.179    0.000
##   Mean.Raup.Crick2 ~
##     Mn.Sp.BdyMsXA2     -0.687    0.088   -7.810    0.000
##     Zoo.Area.ha2        0.350    0.088    3.973    0.000
##   insitu2 ~
##     Attendance2         0.583    0.074    7.903    0.000
##     Zoo.Area.ha2        0.169    0.069    2.443    0.015
##     Prp.Thrt.Abnd2      0.189    0.066    2.873    0.004
##
## Covariances:
##                              Estimate  Std.Err  z-value  P(>|z|)
##   Mean.Sp.BodyMassXAbund2 ~~
##     X10km_Pop2                 -0.209    0.093   -2.245    0.025
##     Zoo.Area.ha2                0.527    0.103    5.120    0.000
##     Mam.Sp.Rchnss2              0.219    0.093    2.357    0.018
##     Prp.Thrt.Abnd2              0.092    0.091    1.012    0.312
##   X10km_Pop2 ~~
##     Zoo.Area.ha2               -0.099    0.091   -1.087    0.277
##     Mam.Sp.Rchnss2              0.421    0.099    4.262    0.000
##     Prp.Thrt.Abnd2              0.278    0.094    2.948    0.003
##   Zoo.Area.ha2 ~~
##     Mam.Sp.Rchnss2              0.536    0.103    5.185    0.000
##     Prp.Thrt.Abnd2              0.071    0.091    0.776    0.438
##   Mam.Sp.Richness2 ~~
##     Prp.Thrt.Abnd2              0.203    0.093    2.190    0.028
##
## Variances:
```

```
##                    Estimate  Std.Err  z-value  P(>|z|)
##    .Attendance2       0.293    0.038    7.714    0.000
##    .Total.Animals2    0.198    0.026    7.714    0.000
##    .Sp.Richness2      0.292    0.038    7.714    0.000
##    .Mean.Rap.Crck2    0.655    0.085    7.714    0.000
##    .insitu2           0.492    0.064    7.714    0.000
##     Mn.Sp.BdyMsXA2    0.992    0.129    7.714    0.000
##     X10km_Pop2        0.992    0.129    7.714    0.000
##     Zoo.Area.ha2      0.992    0.129    7.714    0.000
##     Mam.Sp.Rchnss2    0.992    0.129    7.714    0.000
##     Prp.Thrt.Abnd2    0.992    0.129    7.714    0.000
##
## R-Square:
##                    Estimate
##     Attendance2       0.675
##     Total.Animals2    0.801
##     Sp.Richness2      0.705
##     Mean.Rap.Crck2    0.339
##     insitu2           0.496
```

```r
# Generate fit indices
fitMeasures(mod.IS17A.fit, c("agfi", "rmr", "srmr", "rmsea", "cfi", "nnfi", "tli"))
```

```
##  agfi   rmr  srmr rmsea   cfi  nnfi   tli
## 0.826 0.056 0.057 0.101 0.959 0.932 0.932
```

```r
# Generate modification indices
miIS17A <- modindices(mod.IS17A.fit)
print(miIS17A[miIS17A$mi > 3.0,])
```

```
##                          lhs op                rhs     mi    epc sepc.lv
## 39            Total.Animals2 ~~       Sp.Richness2  3.206 -0.049  -0.049
## 42              Sp.Richness2 ~~     Mean.Raup.Crick2  5.986  0.098   0.098
## 54            Total.Animals2  ~     Mam.Sp.Richness2  3.206  0.145   0.145
## 55            Total.Animals2  ~ Prop.Threat.Abund2  7.695 -0.116  -0.116
## 58              Sp.Richness2  ~     Mean.Raup.Crick2  5.188  0.133   0.133
## 60              Sp.Richness2  ~          X10km_Pop2 13.975  0.219   0.219
## 64          Mean.Raup.Crick2  ~     Total.Animals2  3.156  0.164   0.164
## 65          Mean.Raup.Crick2  ~       Sp.Richness2  3.980  0.165   0.165
## 77  Mean.Sp.BodyMassXAbund2  ~     Total.Animals2  5.683  0.764   0.764
## 85                X10km_Pop2  ~        Attendance2  9.782  0.501   0.501
## 86                X10km_Pop2  ~     Total.Animals2 13.102  0.430   0.430
## 87                X10km_Pop2  ~       Sp.Richness2 13.645  0.488   0.488
## 105         Mam.Sp.Richness2  ~       Sp.Richness2  9.068 -0.496  -0.496
## 112       Prop.Threat.Abund2  ~        Attendance2  3.992 -0.270  -0.270
## 113       Prop.Threat.Abund2  ~     Total.Animals2  4.969 -0.320  -0.320
## 116       Prop.Threat.Abund2  ~            insitu2  3.312 -0.380  -0.380
##     sepc.all sepc.nox
## 39    -0.050   -0.050
## 42     0.099    0.099
## 54     0.145    0.146
## 55    -0.115   -0.116
## 58     0.133    0.133
## 60     0.219    0.220
## 64     0.164    0.164
## 65     0.165    0.165
```

```
## 77      0.765    0.765
## 85      0.477    0.477
## 86      0.431    0.431
## 87      0.488    0.488
## 105   -0.496   -0.496
## 112   -0.257   -0.257
## 113   -0.321   -0.321
## 116   -0.377   -0.377
```

Final In Situ Model (Species Abundance)

Contents
Based on the results, we believe model 17 is an accurate representation of the system. No further addition seems conceptually appealling or statistically justified. In addition all pathways appear significant. Therefore we present model 17 as our final In Situ Model based on species abundance data.

```
# Chosen In Situ SEM (Abundance)
# Model 17

mod.ISAFinal <- 'Attendance2 ~ Total.Animals2
+ Mean.Sp.BodyMassXAbund2 + Mean.Raup.Crick2 + X10km_Pop2

Total.Animals2 ~ Zoo.Area.ha2 + Sp.Richness2 + Mean.Sp.BodyMassXAbund2
Sp.Richness2 ~ Mam.Sp.Richness2 + Mean.Sp.BodyMassXAbund2
Mean.Raup.Crick2 ~ Mean.Sp.BodyMassXAbund2 + Zoo.Area.ha2

insitu2 ~ Attendance2 + Zoo.Area.ha2
+ Prop.Threat.Abund2'

# Fit model and generate model summary
mod.ISAFinal.fit <- sem(mod.ISAFinal, data = sem_in_situ_data, fixed.x=FALSE)
summary(mod.ISAFinal.fit, rsq = TRUE)
```

```
## lavaan (0.5-23.1097) converged normally after  27 iterations
##
##   Number of observations                           119
##
##   Estimator                                         ML
##   Minimum Function Test Statistic               46.566
##   Degrees of freedom                                21
##   P-value (Chi-square)                           0.001
##
## Parameter Estimates:
##
##   Information                                 Expected
##   Standard Errors                             Standard
##
## Regressions:
##                    Estimate  Std.Err  z-value  P(>|z|)
##   Attendance2 ~
##     Total.Animals2    0.537    0.054    9.954    0.000
##     Mn.Sp.BdyMsXA2    0.283    0.059    4.829    0.000
##     Mean.Rap.Crck2    0.130    0.059    2.215    0.027
```

```
##     X10km_Pop2          0.400    0.054    7.447    0.000
##   Total.Animals2 ~
##     Zoo.Area.ha2        0.303    0.054    5.662    0.000
##     Sp.Richness2        0.748    0.045   16.498    0.000
##     Mn.Sp.BdyMsXA2     -0.262    0.050   -5.234    0.000
##   Sp.Richness2 ~
##     Mam.Sp.Rchnss2      0.861    0.051   16.866    0.000
##     Mn.Sp.BdyMsXA2     -0.213    0.051   -4.179    0.000
##   Mean.Raup.Crick2 ~
##     Mn.Sp.BdyMsXA2     -0.687    0.088   -7.810    0.000
##     Zoo.Area.ha2        0.350    0.088    3.973    0.000
##   insitu2 ~
##     Attendance2         0.583    0.074    7.903    0.000
##     Zoo.Area.ha2        0.169    0.069    2.443    0.015
##     Prp.Thrt.Abnd2      0.189    0.066    2.873    0.004
##
## Covariances:
##                             Estimate  Std.Err  z-value  P(>|z|)
##   Mean.Sp.BodyMassXAbund2 ~~
##     X10km_Pop2                -0.209    0.093   -2.245    0.025
##     Zoo.Area.ha2               0.527    0.103    5.120    0.000
##     Mam.Sp.Rchnss2             0.219    0.093    2.357    0.018
##     Prp.Thrt.Abnd2             0.092    0.091    1.012    0.312
##   X10km_Pop2 ~~
##     Zoo.Area.ha2              -0.099    0.091   -1.087    0.277
##     Mam.Sp.Rchnss2             0.421    0.099    4.262    0.000
##     Prp.Thrt.Abnd2             0.278    0.094    2.948    0.003
##   Zoo.Area.ha2 ~~
##     Mam.Sp.Rchnss2             0.536    0.103    5.185    0.000
##     Prp.Thrt.Abnd2             0.071    0.091    0.776    0.438
##   Mam.Sp.Richness2 ~~
##     Prp.Thrt.Abnd2             0.203    0.093    2.190    0.028
##
## Variances:
##                    Estimate  Std.Err  z-value  P(>|z|)
##    .Attendance2       0.293    0.038    7.714    0.000
##    .Total.Animals2    0.198    0.026    7.714    0.000
##    .Sp.Richness2      0.292    0.038    7.714    0.000
##    .Mean.Rap.Crck2    0.655    0.085    7.714    0.000
##    .insitu2           0.492    0.064    7.714    0.000
##     Mn.Sp.BdyMsXA2    0.992    0.129    7.714    0.000
##     X10km_Pop2        0.992    0.129    7.714    0.000
##     Zoo.Area.ha2      0.992    0.129    7.714    0.000
##     Mam.Sp.Rchnss2    0.992    0.129    7.714    0.000
##     Prp.Thrt.Abnd2    0.992    0.129    7.714    0.000
##
## R-Square:
##                    Estimate
##     Attendance2       0.675
##     Total.Animals2    0.801
##     Sp.Richness2      0.705
##     Mean.Rap.Crck2    0.339
##     insitu2           0.496
```

```r
# Generate fit indices
fitMeasures(mod.ISAFinal.fit, c("agfi", "rmr", "srmr", "rmsea", "cfi", "nnfi", "tli"))
```

```
##  agfi   rmr  srmr rmsea   cfi  nnfi   tli
## 0.826 0.056 0.057 0.101 0.959 0.932 0.932
```

```r
# Generate modification indices
miISAFinal <- modindices(mod.ISAFinal.fit)
print(miISAFinal[miISAFinal$mi > 3.0,])
```

```
##                          lhs op               rhs     mi    epc sepc.lv
## 39            Total.Animals2 ~~      Sp.Richness2  3.206 -0.049  -0.049
## 42              Sp.Richness2 ~~  Mean.Raup.Crick2  5.986  0.098   0.098
## 54            Total.Animals2  ~  Mam.Sp.Richness2  3.206  0.145   0.145
## 55            Total.Animals2  ~ Prop.Threat.Abund2  7.695 -0.116  -0.116
## 58              Sp.Richness2  ~  Mean.Raup.Crick2  5.188  0.133   0.133
## 60              Sp.Richness2  ~        X10km_Pop2 13.975  0.219   0.219
## 64          Mean.Raup.Crick2  ~    Total.Animals2  3.156  0.164   0.164
## 65          Mean.Raup.Crick2  ~      Sp.Richness2  3.980  0.165   0.165
## 77  Mean.Sp.BodyMassXAbund2  ~    Total.Animals2  5.683  0.764   0.764
## 85                X10km_Pop2  ~       Attendance2  9.782  0.501   0.501
## 86                X10km_Pop2  ~    Total.Animals2 13.102  0.430   0.430
## 87                X10km_Pop2  ~      Sp.Richness2 13.645  0.488   0.488
## 105         Mam.Sp.Richness2  ~      Sp.Richness2  9.068 -0.496  -0.496
## 112       Prop.Threat.Abund2  ~       Attendance2  3.992 -0.270  -0.270
## 113       Prop.Threat.Abund2  ~    Total.Animals2  4.969 -0.320  -0.320
## 116       Prop.Threat.Abund2  ~           insitu2  3.312 -0.380  -0.380
##     sepc.all sepc.nox
## 39    -0.050   -0.050
## 42     0.099    0.099
## 54     0.145    0.146
## 55    -0.115   -0.116
## 58     0.133    0.133
## 60     0.219    0.220
## 64     0.164    0.164
## 65     0.165    0.165
## 77     0.765    0.765
## 85     0.477    0.477
## 86     0.431    0.431
## 87     0.488    0.488
## 105   -0.496   -0.496
## 112   -0.257   -0.257
## 113   -0.321   -0.321
## 116   -0.377   -0.377
```

Tests of Mediation

It is clear from Model 17 that several pathways are mediated in the system. It is necessary to evaluate whether this mediation is appropriate by comparing models with complete, partial and no mediation. Here we show tests of mediation for three of these relationships.

insitu2 ~ Mean.Sp.BodyMassXAbund2

insitu2 ~ Mean.Sp.BodyMassXAbund2 is completely mediated by **Attendance2 ~ Mean.Sp.BodyMass2**. Below we compare models with complete (full, i.e. Model 17) mediation, partial mediation and no mediation. Results from this output suggest that complete mediation (model 17) is the superior model.

```
# Complete Mediation Model (Model 17)

FULL1A.MOD17 <- 'Attendance2 ~ Total.Animals2
+ Mean.Sp.BodyMassXAbund2 + Mean.Raup.Crick2 + X10km_Pop2

Total.Animals2 ~ Zoo.Area.ha2 + Sp.Richness2 + Mean.Sp.BodyMassXAbund2
Sp.Richness2 ~ Mam.Sp.Richness2 + Mean.Sp.BodyMassXAbund2
Mean.Raup.Crick2 ~ Mean.Sp.BodyMassXAbund2 + Zoo.Area.ha2

insitu2 ~ Attendance2 + Zoo.Area.ha2
+ Prop.Threat.Abund2'

FULL1A.MOD17.fit <- sem(FULL1A.MOD17, data = sem_in_situ_data, fixed.x=FALSE)

# Partial Mediation Model

PARTIAL1A <- 'Attendance2 ~ Total.Animals2
+ Mean.Sp.BodyMassXAbund2 + Mean.Raup.Crick2 + X10km_Pop2

Total.Animals2 ~ Zoo.Area.ha2 + Sp.Richness2 + Mean.Sp.BodyMassXAbund2
Sp.Richness2 ~ Mam.Sp.Richness2 + Mean.Sp.BodyMassXAbund2
Mean.Raup.Crick2 ~ Mean.Sp.BodyMassXAbund2 + Zoo.Area.ha2

insitu2 ~ Attendance2 + Zoo.Area.ha2
+ Prop.Threat.Abund2 + Mean.Sp.BodyMassXAbund2'

PARTIAL1A.fit <- sem(PARTIAL1A, data = sem_in_situ_data, fixed.x=FALSE)

# No Mediation Model

NONE1A <- 'Attendance2 ~ Total.Animals2
+ Mean.Raup.Crick2 + X10km_Pop2

Total.Animals2 ~ Zoo.Area.ha2 + Sp.Richness2 + Mean.Sp.BodyMassXAbund2
Sp.Richness2 ~ Mam.Sp.Richness2 + Mean.Sp.BodyMassXAbund2
Mean.Raup.Crick2 ~ Mean.Sp.BodyMassXAbund2 + Zoo.Area.ha2

insitu2 ~ Attendance2 + Zoo.Area.ha2
+ Prop.Threat.Abund2 + Mean.Sp.BodyMassXAbund2'

NONE1A.fit <- sem(NONE1A, data = sem_in_situ_data, fixed.x=FALSE)

aictab.lavaan(list(FULL1A.MOD17.fit, PARTIAL1A.fit, NONE1A.fit),
              c("full mediation", "partial mediation", "no mediation"))
```

```
##
## Model selection based on AICc:
##
##                      K    AICc Delta_AICc AICcWt Cum.Wt       LL
```

```
## full mediation    34 2677.51       0.00   0.71   0.71 -1302.74
## partial mediation 35 2679.28       1.77   0.29   1.00 -1302.31
## no mediation      34 2697.79      20.28   0.00   1.00 -1312.88
```

insitu2 ~ Prop.Threat.Abund2

insitu2 ~ Prop.Threat.Abund2 is not mediated. Therefore we want to test if partial mediation is appropriate by including by **Attendance2 ~ Prop.Threat.Abund2**. Below we compare models with complete (full) mediation, partial mediation and no mediation (Model 17). Results from this output suggest that no mediation (model 17) is the superior model.

```
# Complete Mediation Model

FULL2A <- 'Attendance2 ~ Total.Animals2
+ Mean.Sp.BodyMassXAbund2 + Mean.Raup.Crick2 + X10km_Pop2 + Prop.Threat.Abund2

Total.Animals2 ~ Zoo.Area.ha2 + Sp.Richness2 + Mean.Sp.BodyMassXAbund2
Sp.Richness2 ~ Mam.Sp.Richness2 + Mean.Sp.BodyMassXAbund2
Mean.Raup.Crick2 ~ Mean.Sp.BodyMassXAbund2 + Zoo.Area.ha2

insitu2 ~ Attendance2 + Zoo.Area.ha2'

FULL2A.fit <- sem(FULL2A, data = sem_in_situ_data, fixed.x=FALSE)

# Partial Mediation Model

PARTIAL2A <- 'Attendance2 ~ Total.Animals2
+ Mean.Sp.BodyMassXAbund2 + Mean.Raup.Crick2 + X10km_Pop2 + Prop.Threat.Abund2

Total.Animals2 ~ Zoo.Area.ha2 + Sp.Richness2 + Mean.Sp.BodyMassXAbund2
Sp.Richness2 ~ Mam.Sp.Richness2 + Mean.Sp.BodyMassXAbund2
Mean.Raup.Crick2 ~ Mean.Sp.BodyMassXAbund2 + Zoo.Area.ha2

insitu2 ~ Attendance2 + Zoo.Area.ha2
+ Prop.Threat.Abund2'

PARTIAL2A.fit <- sem(PARTIAL2A, data = sem_in_situ_data, fixed.x=FALSE)

# No Mediation Model (Model 17)

NONE2A.MOD17 <- 'Attendance2 ~ Total.Animals2
+ Mean.Sp.BodyMassXAbund2 + Mean.Raup.Crick2 + X10km_Pop2

Total.Animals2 ~ Zoo.Area.ha2 + Sp.Richness2 + Mean.Sp.BodyMassXAbund2
Sp.Richness2 ~ Mam.Sp.Richness2 + Mean.Sp.BodyMassXAbund2
Mean.Raup.Crick2 ~ Mean.Sp.BodyMassXAbund2 + Zoo.Area.ha2

insitu2 ~ Attendance2 + Zoo.Area.ha2
+ Prop.Threat.Abund2'

NONE2A.MOD17.fit <- sem(NONE2A.MOD17, data = sem_in_situ_data, fixed.x=FALSE)

aictab.lavaan(list(FULL2A.fit, PARTIAL2A.fit, NONE2A.MOD17.fit),
              c("full mediation", "partial mediation", "no mediation"))
```

```
##
## Model selection based on AICc:
##
##                     K    AICc Delta_AICc AICcWt Cum.Wt       LL
## no mediation       34 2677.51       0.00   0.71   0.71 -1302.74
## partial mediation 35 2679.38       1.87   0.28   0.98 -1302.36
## full mediation     34 2684.97       7.46   0.02   1.00 -1306.46
```

Calculating Total Effects

Contents
Total effects of insitutional variables and socio-economic factors on visitor Attendance and In situ contributions is calculated using the direct and indirect effects from the final model. An example for the Attendance and In Situ models for the abundance adjusted models (Table 2) is given below.

```
## Total effects
## Yvonne Buckley
## 03/10/19

## Attendance model
att_totanimal <- coef(mod.20A.final.fit)[labels(coef(mod.20A.final.fit)) == "Attendance2~Total.Animals2
att_10k <- coef(mod.20A.final.fit)[labels(coef(mod.20A.final.fit)) == "Attendance2~X10km_Pop2"]
att_body <- coef(mod.20A.final.fit)[labels(coef(mod.20A.final.fit)) == "Attendance2~Mean.Sp.BodyMassXAb
att_GDP <- coef(mod.20A.final.fit)[labels(coef(mod.20A.final.fit)) == "Attendance2~GDP.Millions2"]
att_diss <- coef(mod.20A.final.fit)[labels(coef(mod.20A.final.fit)) == "Attendance2~Mean.Raup.Crick2"]
att_mamm <- coef(mod.20A.final.fit)[labels(coef(mod.20A.final.fit)) == "Attendance2~Mam.Sp.Richness2"]
att_spr <- coef(mod.20A.final.fit)[labels(coef(mod.20A.final.fit)) == "Attendance2~Sp.Richness2"]

totan_spr <- coef(mod.20A.final.fit)[labels(coef(mod.20A.final.fit)) == "Total.Animals2~Sp.Richness2"]
totan_area <- coef(mod.20A.final.fit)[labels(coef(mod.20A.final.fit)) == "Total.Animals2~Zoo.Area.ha2"]
totan_GDP <- coef(mod.20A.final.fit)[labels(coef(mod.20A.final.fit)) == "Total.Animals2~GDP.Millions2"]
totan_body <- coef(mod.20A.final.fit)[labels(coef(mod.20A.final.fit)) == "Total.Animals2~Mean.Sp.BodyMa

spr_mamm <- coef(mod.20A.final.fit)[labels(coef(mod.20A.final.fit)) == "Sp.Richness2~Mam.Sp.Richness2"]
spr_area <- coef(mod.20A.final.fit)[labels(coef(mod.20A.final.fit)) == "Sp.Richness2~Zoo.Area.ha2"]
spr_body <- coef(mod.20A.final.fit)[labels(coef(mod.20A.final.fit)) == "Sp.Richness2~Mean.Sp.BodyMassXA

diss_area <- coef(mod.20A.final.fit)[labels(coef(mod.20A.final.fit)) == "Mean.Raup.Crick2~Zoo.Area.ha2"
diss_body <- coef(mod.20A.final.fit)[labels(coef(mod.20A.final.fit)) == "Mean.Raup.Crick2~Mean.Sp.BodyM

##In situ model
is_att <- coef(mod.ISAFinal.fit)[labels(coef(mod.ISAFinal.fit)) == "insitu2~Attendance2"]
is_tsp <- coef(mod.ISAFinal.fit)[labels(coef(mod.ISAFinal.fit)) == "insitu2~Prop.Threat.Abund2"]
is_area <- coef(mod.ISAFinal.fit)[labels(coef(mod.ISAFinal.fit)) == "insitu2~Zoo.Area.ha2"]

## ATTENDANCE Total Effects
##Total Animals
TEatt_totanimal <- att_totanimal
print(paste("Attendance ~ Total Animals", TEatt_totanimal, sep = "  "))
```

```
## [1] "Attendance ~ Total Animals  0.627018604309392"
```

```
## 10km population density
TEatt_10k <- att_10k
```

```r
print(paste("Attendance ~ 10km Population", TEatt_10k, sep = "  "))
```

```
## [1] "Attendance ~ 10km Population  0.427537393329003"
```

```r
## Body Mass
TEatt_body <- att_body + diss_body * att_diss + spr_body * att_spr + spr_body * totan_spr *att_totanimal
  totan_body * att_totanimal
print(paste("Attendance ~ Body Mass", TEatt_body, sep = "  "))
```

```
## [1] "Attendance ~ Body Mass  0.0902769048928655"
```

```r
## GDP
TEatt_GDP <- att_GDP + totan_GDP * att_totanimal
print(paste("Attendance ~ GDP", TEatt_GDP, sep = "  "))
```

```
## [1] "Attendance ~ GDP  0.0915382895191026"
```

```r
## Dissimilarity
TEatt_diss <- att_diss
print(paste("Attendance ~ Dissimilarity", TEatt_diss, sep = "  "))
```

```
## [1] "Attendance ~ Dissimilarity  0.101144877144399"
```

```r
## Mammal Species Richness
TEatt_mamm <- att_mamm +spr_mamm * totan_spr * att_totanimal + spr_mamm * att_spr
print(paste("Attendance ~ Mammal Species Richness", TEatt_mamm, sep = "  "))
```

```
## [1] "Attendance ~ Mammal Species Richness  0.341358881401393"
```

```r
## Species Richness
TEatt_spr <- att_spr + totan_spr * att_totanimal
print(paste("Attendance ~ Species Richness", TEatt_spr, sep = "  "))
```

```
## [1] "Attendance ~ Species Richness  0.225736512315213"
```

```r
## TOTAL ANIMALS Total Effects
## Species richness
TEta_spr <- totan_spr
print(paste("Total Animals ~ Species Richness", TEta_spr, sep = "  "))
```

```
## [1] "Total Animals ~ Species Richness  0.754452156109668"
```

```r
## Institution Area
TEta_area <- totan_area + spr_area * totan_spr
print(paste("Total Animals ~ Area", TEta_area, sep = "  "))
```

```
## [1] "Total Animals ~ Area  0.412574382288663"
```

```r
## GDP
TEta_GDP <- totan_GDP
print(paste("Total Animals ~ GDP", TEta_GDP, sep = "  "))
```

```
## [1] "Total Animals ~ GDP  -0.129245340273416"
```

```r
## Body Mass
TEta_body <- totan_body + spr_body *totan_spr
print(paste("Total Animals ~ Body Mass", TEta_body, sep = "  "))
```

```
## [1] "Total Animals ~ Body Mass  -0.544683135149638"
```

```r
## SPECIES RICHNESS Total Effects
## Mammal Species Richness
TEsr_mamm <- spr_mamm
print(paste("Species Richness ~ Mammal Species Richness", TEsr_mamm, sep = "  "))
```

```
## [1] "Species Richness ~ Mammal Species Richness  0.852022091698783"
```

```r
## Institution Area
TEsr_area <- spr_area
print(paste("Species Richness ~ Area", TEsr_area, sep = "  "))
```

```
## [1] "Species Richness ~ Area  0.11991484776747"
```

```r
## Body Mass
TEsr_body <- spr_body
print(paste("Species Richness ~ Body Mass", TEsr_body, sep = "  "))
```

```
## [1] "Species Richness ~ Body Mass  -0.469976902634104"
```

```r
## DISSIMILARITY Total Effects
##Institution Area
TEds_area <- diss_area
print(paste("Dissimilarity ~ Area", TEds_area, sep = "  "))
```

```
## [1] "Dissimilarity ~ Area  0.339212718118449"
```

```r
## Body Mass
TEds_body <- diss_body
print(paste("Dissimilarity ~ Body Mass", TEds_body, sep = "  "))
```

```
## [1] "Dissimilarity ~ Body Mass  -0.63877310140421"
```

```r
## IN SITU Total Effects
## Attendance
TEis_att <- is_att
print(paste("In Situ ~ Attendance", TEis_att, sep = "  "))
```

```
## [1] "In Situ ~ Attendance  0.583455412761181"
```

```r
## Proportion Threatened Species
TEis_ts <- is_tsp
print(paste("In Situ ~ Threatened Species Proportion", TEis_ts, sep = "  "))
```

```
## [1] "In Situ ~ Threatened Species Proportion  0.189452594520929"
```

```r
## Institution Area
TEis_area <- is_area + diss_area * att_diss * is_att + spr_area * totan_spr * att_totanimal * is_att +
  totan_area * att_totanimal * is_att
print(paste("In Situ ~ Area", TEis_area, sep = "  "))
```

```
## [1] "In Situ ~ Area  0.339566067381254"
```

References:

Burnham, K. P., Anderson, D. R. & Burnham, K. P. Model selection and multimodel inference: a practical information-theoretic approach. (Springer, 2002).

Fan, Y. et al. Applications of structural equation modeling (SEM) in ecological studies: an updated review. Ecol. Process. 5, 19 (2016).

Grace, J. B., Scheiner, S. M. & Schoolmaster, D. R. J. Structural equation modeling: building and evaluating causal models. in Ecological Statistics: Contemporary theory and application (eds. Fox, G. A., Negrete-Yankelevich, S. & Sosa, V. J.) 168-199 (Oxford University Press, 2015).

Grace, J. B. et al. Integrative modelling reveals mechanisms linking productivity and plant species richness. Nature 529, 390-393 (2016).

Hu, L. & Bentler, P. M. Cutoff criteria for fit indexes in covariance structure analysis: Conventional criteria versus new alternatives. Struct. Equ. Model. A Multidiscip. J. 6, 1-55 (1999).

Martin et al., 2014: Mammal and bird species held in zoos are less endemic and less threatened than their close relatives not held in zoos Frynta et al., 2013: Mammalian Collection on Noah's Ark: The Effects of Beauty, Brain and Body Size
